# Systematic Review of Methods in Low-Consensus Fields: Supporting Commensuration through `Construct-Centered Methods Aggregation’ in the Case of Climate Change Vulnerability Research

**DOI:** 10.1371/journal.pone.0149071

**Published:** 2016-02-22

**Authors:** Aogán Delaney, Peter A. Tamás, Todd A. Crane, Sabrina Chesterman

**Affiliations:** 1 Research Methodology Group, Wageningen University, Wageningen, Netherlands; 2 International Livestock Research Institute, Nairobi, Kenya; 3 London School of Hygiene and Tropical Medicine, London, United Kingdom; Iran University of Medical Sciences, ISLAMIC REPUBLIC OF IRAN

## Abstract

There is increasing interest in using systematic review to synthesize evidence on the social and environmental effects of and adaptations to climate change. Use of systematic review for evidence in this field is complicated by the heterogeneity of methods used and by uneven reporting. In order to facilitate synthesis of results and design of subsequent research a method, *construct-centered methods aggregation*, was designed to 1) provide a transparent, valid and reliable description of research methods, 2) support comparability of primary studies and 3) contribute to a shared empirical basis for improving research practice. Rather than taking research reports at face value, research designs are reviewed through inductive analysis. This involves bottom-up identification of constructs, definitions and operationalizations; assessment of concepts’ commensurability through comparison of definitions; identification of theoretical frameworks through patterns of construct use; and integration of transparently reported and valid operationalizations into ideal-type research frameworks. Through the integration of reliable bottom-up inductive coding from operationalizations and top-down coding driven from stated theory with expert interpretation, construct-centered methods aggregation enabled both resolution of heterogeneity within identically named constructs and merging of differently labeled but identical constructs. These two processes allowed transparent, rigorous and contextually sensitive synthesis of the research presented in an uneven set of reports undertaken in a heterogenous field. If adopted more broadly, construct-centered methods aggregation may contribute to the emergence of a valid, empirically-grounded description of methods used in primary research. These descriptions may function as a set of expectations that improves the transparency of reporting and as an evolving comprehensive framework that supports both interpretation of existing and design of future research.

## Introduction

The number of studies on vulnerability to and subsequent adaptation options for climate change (climate vulnerability/adaptation studies) have increased dramatically over the past two decades. Much of this research takes the form of geographically and contextually specific case studies undertaken by independent research teams. This has led to the development and use of a diversity of theories and methods. There is an increasing interest in synthesizing across these disparate research projects. Systematic review initially emerged in the field of medical trials precisely to support this sort of synthesis and its application has become increasingly popular in other fields, including climate change impact studies.

In fields like climate change research that lack the degree of standardization of clinical medical research, attempts to use systematic review to synthesize the results of empirical studies can be frustrated by a lack of equivalence between those studies. The use of heterogeneous methods, for example, immediately complicates quality assessment and comparability. As such, systematic review has required substantial adaptation as it has been taken up in disciplines outside medicine. One of the adaptations made has been to shift the objective of review. Rather than attempt to compare results, review is used to compare methods or theory. Ongoing transparent, comprehensive and rigorous (i.e. systematic) review of methods and theory used in research may support the emergence of consensus around which methods to use. Subsequent dissemination of these methods, then, improves the comparability of results. Several iterations of this cycle may yield a body of empirical findings that supports evidence review. The identification of compatible, if not common, methods by which a given object is measured is called commensuration [1]. Commensuration is typically an unevenly transparent socially mediated process. This paper is an examination of the relevance of systematic review to commensuration undertaken in the context of the case provided by a specific commission within climate vulnerability/adaptation studies.

In 2014, the Climate Change, Agriculture and Food Security program (CCAFS) commissioned a review to identify methods for researching local level vulnerability to climate change in smallholder agro-pastoral systems. The purpose of this study was to generate empirical foundations for the design of long-term and broadly applicable monitoring and evaluation protocols for their interventions aimed at reducing vulnerability and improving adaptive capacity. Adopting the language of Plummer [2], the goal of the CCAFS study was to increase the consistency among local vulnerability assessment tools, while still permitting contextual sensitivity that would facilitate comparison that better supported transferability. While the primary results have been published elsewhere [3] and are available here S1 File with full supporting information S2 File — undertaken to help answer the programmatically useful question ‘how should the topic be researched’ — also afforded an opportunity to develop methods for systematic review. The present paper reports only on our pursuit of our methodological objectives:

1. what is the relevance of systematic review to commensuration within climate vulnerability/adaptation studies, and
2. what challenges did we encounter and what are their implications.

Following the call for systematic reviewers to “produce critical reflexive accounts of their experiences of using [new] methods” so that others can “benefit from their learning and for the methods to be improved and become more sophisticated” [4], we pursued our objectives through an evidence-based contribution to the development of systematic review methods to support commensuration within vulnerability/adaptation studies. The principles outlined in this article may be relevant in fields characterized by lack of standardized concepts and methods, especially ones that integrate both biophysical and social data.

We open with an overview of the processes by which measurements of research objects are made comparable (commensuration), followed by detailing an argument for systematic review in commensuration. The article then describes how systematic review has been used to describe research methods used in studies relevant to climate change. It goes on to describe relevant aspects of the methods we used and the issues encountered in our review of studies that operationalized the construct ‘local vulnerability to the effects of climate change’. The paper concludes with a discussion of the implications of our findings for the use of systematic review in commensuration generally and the reporting of empirical research within climate vulnerability/adaptation studies specifically.

### Commensuration

One challenge facing climate vulnerability/adaptation researchers is that the results of locally adequate studies may not be mutually compatible. This lack of compatibility frustrates both comparison, which is useful for guiding programmatic decisions, and aggregation, which is useful for national and regional overviews on the state of vulnerability to climate change. The mechanisms by which indicators are constructed such that different entities become comparable is an often overlooked social process of negotiation that involves technical considerations along with actors’ value judgments, opinions, practical concerns, political interests and power strategies. This has been documented both in the domain of climate impact studies (e.g. [5–7]) and in other fields (e.g. [1, 8–13]).

In negotiation over how to measure, some information is discarded [8, 10, 12, 14] and the remaining information is re-arranged in a manner that reduces complexity so that actors and observers have the capability to act [15]. Commensuration includes and excludes what is seen of the world through research in ways that make information look more convincing. This occurs through practices of reduction and representation that absorb contingency and uncertainty. When completed, the differences that are visible are matters of quantity, not qualitative difference, and, as such, information becomes more portable [1, 15].

Broadly speaking, there are two ways to think about commensuration. First, and most simply, it can be understood as the standardization of measurement. Second, commensuration refers to the specification of the frame within which measurement of a given object is negotiated. Both of these are discussed, with examples, below.

Standards are explicitly formulated and decided rules [16]. Standardization involves construction of uniformity across activity sites through the generation of accepted rules whose application is often overseen by some external body [13]. Standardized operationalization of constructs is most useful in well-established contexts because the instrument has known precision across the full diversity of circumstances in which it will be used. Standardization is not well suited in research on complex, contextually specific dynamic topics such as resilience (e.g. [17]). This is because the circumstances in which the instrument would be used are not predictable enough to support the claim that any instrument would be reliably and adequately sensitive across the full diversity of circumstances and contexts in which it would be applied.

Rather than delimit operationalization, it is possible for commensuration to focus on the frames within which operationalization is undertaken. Commensuration as frame is exemplified in the review of Milne [12] on competing specifications of carbon accounting and consistent with the observation by Liquete [18] that a single classification scheme would be unworkable. While such a framework may be helpful in bounding and structuring discussion, a frame-based approach to commensuration does not provide a final or definite standard for methodological choices in how to conduct research.

Bringing discussion of standardization and framing together, Brunsson [16] argues that although standards are most often associated with stability, standardization is dynamic. The language of framing, introduced above, provides a means by which commensuration remains relevant even when rejecting the direct specification of research protocols. If commensuration is recognized on the model of framing, individual researchers will each exercise discretion in their operationalizations. One facet of the frame in which scholars will justify their individual operationalizations is an accurate, adequate and common understanding of how their peers have operationalized the same construct in, perhaps, different circumstances. In setting standards, the options are lowest common denominator, accepting the views of the powerful, negotiated order among stakeholders and confirmation of past practice [13]. Of these, we advocate, and find systematic review relevant to, transparent on-going negotiation.

Systematic review of methods becomes relevant when researchers design their studies because description of the methods used constitutes an aspect of the frame within which subsequent researchers select research methods. What distinguishes systematic review of methods from traditional narrative review for use in commensuration are its commitments to complete identification of relevant material, comprehensive treatment of data and transparent description of methods [19]. Rephrasing, and this time extending slightly the argument of Plummer [2], systematic review of methods may provide the best available frame for the commensuration of primary research on complex objects in climate change impact studies.

### Systematic review of methods in climate change impact studies

Systematic review is gaining in popularity and has been capably introduced to the field of climate studies not least by Plummer [2]. As such, only a brief description of the method is given here. Systematic review is a formal research methodology which originated in the health sciences with the task of surveying all available randomized controlled trial evidence on a given research question [20, 21]. Reviewers were concerned with deriving global conclusions from disparate trial studies and so based the methodology on rigorous, replicable, and transparent steps leading it to be considered as a research method rather than simply a literature review. The review method has been adapted in recent years. It is now used in fields other than the health sciences, it can analyze additional types of evidence beyond randomized controlled trials, and in some cases it is used to analyze methods and theory as opposed to evidence [2, 22–25]. Nonetheless, with each adaptation, constituent methods adhere to the systematic review principals of rigor, transparency, reliability, and comprehensiveness. These constituent methods usually consist of four steps: a recordable search strategy; screening search results according to defined inclusion protocols; transparent and reproducible data extraction from subject literature; and secondary analysis of extracted data [19, 26].

In order to describe how systematic review is used in the context of studies on climate change the authors undertook a limited review of reviews in the field. The authors searched for (systematic review AND (climate change OR (environmen* AND polic*) OR adaptive capacity OR (vulnerability AND climate)) NOT medic*) in Web of Science, OVID, Proquest and Scopus in February and March of 2015. After eliminating duplicates, returns (n = 425) were classified as shown in Table 1 based on review of titles and abstracts. Non-relevant returns (n = 292), most of which were entirely medical, are not reflected in Table 1. Based on examination of titles and abstracts, systematic review appears to be used within climate change studies to answer a number of different kinds of questions (Table 1) (Full details of this search and analysis, along with primary records, are available from the corresponding author.)

**Table 1:** Uses of systematic review in climate change studies.

*What are people doing (e.g. to pre-adapt)?*	*10*
*What adaptations do researchers suggest?*	*1*
*What knowledge claims do reviews actually support?*	*2*
*What is known about a given topic?*	*61*
What methods are being used for primary research?	16
What methods should be used for review?	12
What theory is being used?	5
*What interventions are effective?*	*14*

Examination of the articles whose titles and/or abstracts indicated an interest in methods (detailed in S3 File) found research questions related to:

1. description of the use of results (n = 3)
2. description of the methods used in primary research (n = 5)
3. assessment of methods used in primary research (n = 4)
4. description of the links between theory and practice (n = 3)
5. description of theory (n = 4)

Limiting our interest to those articles that described the methods used in primary research and/or described theory identified seven articles. These articles were analyzed to answer the following questions:

1. What objectives are stated relevant to methods or theory?
2. What are the steps used to describe theory?
3. What are the steps used to describe methods?
4. What recommendations do they make for review and/or reporting of primary research?
5. What issues did they encounter?

Summary results of this review are presented in Table 2 and discussed below. Full citations for the articles can be found in the bibliography.

**Table 2:** Results of review of systematic reviews.

	Delaney ʼ14	Driscol	Halfors	Castleden	Liquete	Plummer ʼ12	Schultz	Plummer ʼ13
Research objectives relevant to methods or theory								
*describe methods/indicators/tools/approach used*	**x**	**x**			**x**	**x**	**x**	
*assess adequacy of methods used*	**x**				**x**	**x**	**x**	
*describe/assess constructs/terms/principles*	**x**		**x**	**x**				**x**
Operationalization: theory								
*identified theory deductively*	**x**		**x**	**x**	**x**		**x**	
*identified theory inductively*	**x**		**x**		**x**	**x**		**x**
*synthesized theory aggregatively*	**x**		**x**			**x**		**x**
*synthesized theory interpretive*	**x**		**x**		**x**			
*described theory as identified*	**x**		**x**		**x**	**x**	**x**	**x**
*described theory as as synthesized*	**x**					**x**		**x**
*compared theory as identified and synthesized*	**x**							
*identified constructs—inductively*	**x**		**x**			**x**		**x**
*identified constructs—deductively*	**x**	**x**	**x**	**x**	**x**	**x**	**x**	**x**
*synthesized constructs aggregatively*	**x**		**x**			**x**		**x**
*synthesized constructs interpretively*	**x**		**x**			**x**		**x**
*described constructs as identified*	**x**		**x**	**x**	**x**	**x**	**x**	**x**
*described constructs as synthesized*	**x**		**x**			**x**		**x**
*described relationships between constructs*			**x**					
*compared constructs as identified and synthesized*	**x**							
Operationalization: methods								
*assessed operationalizations for transparency*	**x**							
*assessed operationalizations (op’s) for validity*	**x**	**x**			**x**	**x**	**x**	
*assessed op’s for reach*	**x**							
*described op’s as identified*	**x**	**x**			**x**	**x**	**x**	
*described op’s as synthesized*	**x**					**x**		
*compared op’s as identified & as synthesized*	**x**					**x**		
*synthesized op’s aggregatively*	**x**							
Recommendations								
*specify clear commonly defined key constructs*			**x**	**x**				
*study commensurability/interchangeability of methods*	**x**	**x**			**x**			
*study validity of indirect measures*	**x**	**x**					**x**	
*prescribed standards for research practice*		**x**			**x**	**x**	**x**	
*prescribed standards for reporting research*	**x**							
*prescribed theory definition/classification*			**x**	**x**	**x**			
Issues								
*bias in prevalence of method use*		**x**			**x**			
*novel contributions to lit are under-represented*	**x**							
*database searches are not discriminating*	**x**	**x**	**x**	**x**	**x**	**x**		**x**
*resources arbitrarily constrained study*	**x**	**x**			**x**			
*found article as sole research unit to be inadequate*	**x**	**x**	**x**				**x**	
*instability of language use*	**x**		**x**	**x**	**x**	**x**		**x**
*uneven reporting*	**x**				**x**	**x**		
Rationale for study								
*increase comparability*	**x**		**x**	**x**	**x**	**x**		

As noted above, systematic review has been adapted from its roots in the medical sciences, a field where standardization of methods and reporting has allowed efficient and powerful reviews of evidence from disparate studies. The use of systematic reviews in climate impact studies is growing and, as indicated in the italicized rows of Table 1, the majority of this growth relates to reviews of evidence. Although only a small proportion of the systematic reviews in the field of climate impact studies declare an explicit interest in methods, those that do consistently find methodological heterogeneity in the primary research they examine and these reviews often state as a primary purpose supporting the comparability of the results of primary research. For example, Plummer [2] decided on systematic review when confronted with the “myriad of ways in which water vulnerability is assessed.” In the case of Plummer’s study, ‘myriad’ meant that for the 55 studies examined, they found 50 distinct instruments.

Further complicating the comparability required for aggregation of evidence, most of the reviews documented in Table 2 found that authors’ use of language was not stable. For example, in our own review we found that even when constructs share a name and a definition, their operationalizations may diverge considerably. In such cases comparability is not secured when a reviewer identifies identical use of language nor is it secured if the reviewer finds identical definitions. Comparability is only established by painstaking review of the operationalization of a construct.

Taken together, reviews of the methods used in primary research in the field of climate impact studies suggest that those who wish to aggregate evidence should first test whether the primary research of interest to them is undertaken using a myriad of inconsistently described methods. Taking the next step, proper description of the possible heterogeneity of methods used requires reviewers to correct authors’ inconsistent use of language.

The reviews presented in Table 2 used inductive and deductive approaches to describe the methods used in primary research. Deductive approaches are desirable in that their reliance on clearly identifiable features in the text (e.g. constructs as named by authors) produces reliable and efficient descriptions of texts. Authors’ possible inconsistency in language use, however, makes it possible that these reliable descriptions are not valid. Inductive approaches to describe methods start with open coding. While this method is robust in its sensitivity to the characteristics of the individual texts reviewed, they may lack both the reliability and transparency that are thought traditionally to be hallmarks of systematic review.

As indicated in the rows of Table 2 that identify induction and its synthetic products, the method we tested in our review was designed to correct for instability in authors’ use of language while remaining rigorous, replicable and transparent. The review method we use is a form of inductive and aggregative methods-synthesis, which we will call ‘construct-centered methods aggregation’.

## Methods

In this section, only those methods which are relevant for coding and analysis of constructs are reported. This corresponds to two of research questions posed in the commission from CCAFS, namely:

1. How is vulnerability defined?
2. How is vulnerability operationalized?

These questions examine how research is done rather than the results reported. To grasp this operationally, we used the term *theoretical framework* which we then decomposed into three components: constructs; construct definitions; and relationships [27, 28]. We defined a *construct* as a conceptual representation of a phenomena, which can be divided into *sub-constructs* or abstracted to *higher-order constructs* or to *theory*. A *construct definition* was a bounded delineation of what phenomena the construct represents (and what it does not represent). *Construct relationships* were elements which link constructs together in such a way as to give structure to a framework. Further, while a theoretical framework structures research, it is implemented through a methodology. We used *operationalization* to represent the act of generating data to empirically represent or measure a construct, including both the intermediate steps of conceptual decomposition and the final act of measurement.

Of these, *construct* is the most important and functions in our method as the organizing unit of analysis. In our review we identified author-reported constructs, construct definitions, relationships, and operationalizations inductively. We then stratified reviewed articles deductively, in order to improve reliability and feasibility, using a pre-defined taxonomy of theoretical frameworks. After sorting into this deductively applied taxonomy, author-reported constructs, theoretical frameworks and operationalizations were analyzed for commensurability, sorted as required, and synthesized into an analyst-generated set of frameworks.

**Fig 1:**
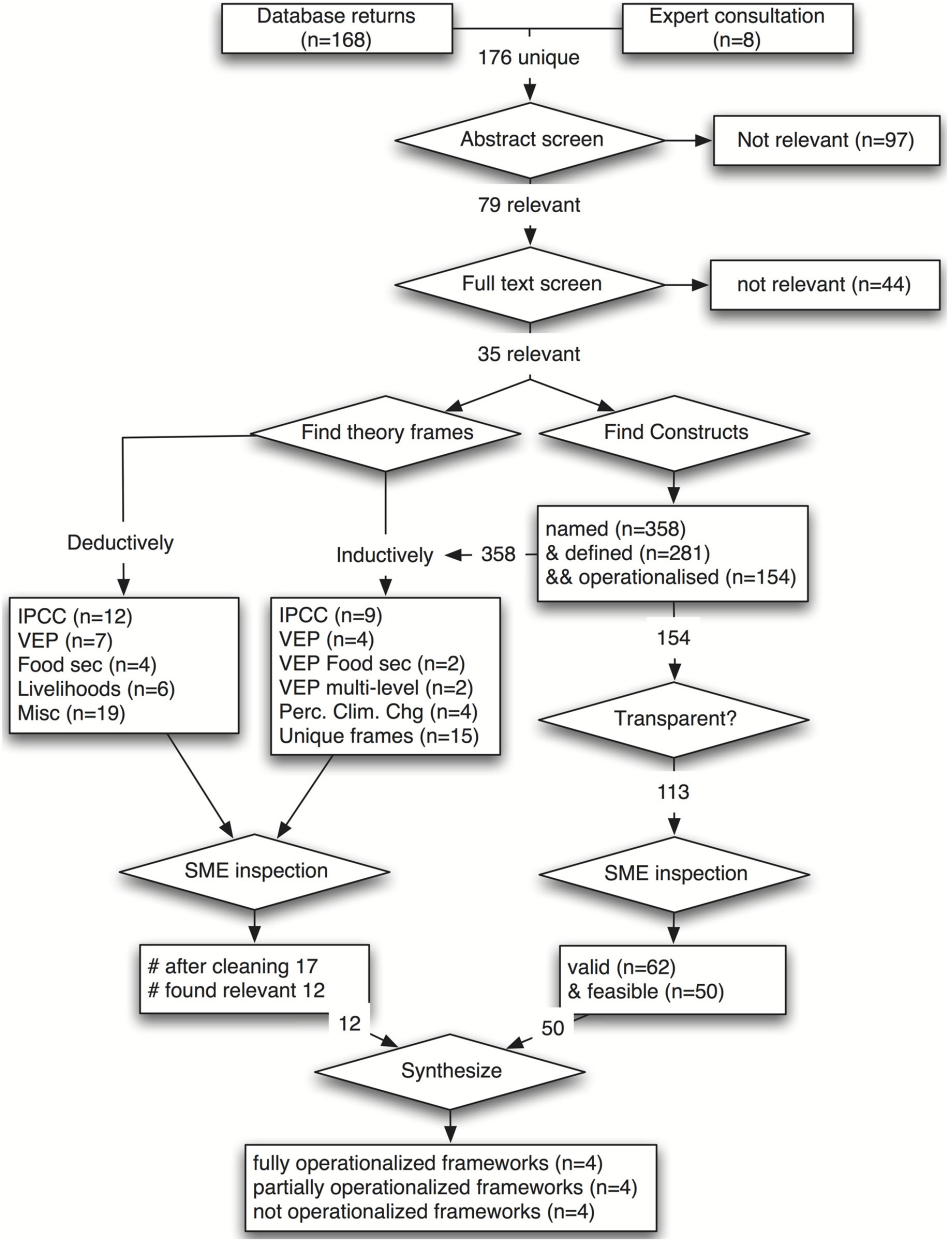
Review Process.

Data extraction for each article followed the steps summarized below. These steps were executed using a data extraction form (S1 Table), using coding functions in the qualitative data analysis software, NVivo, and with a coding framework for categorization of theoretical frameworks (S2 Table). A flow chart of the review process can be found as Fig 1.

1. Read the abstract, introduction, and theoretical framework sections until a research question (RQ) is identified. Apply a code ‘Research Question’.
2. Within the RQ, identify and code each construct.
3. Re-read the theoretical framework and then identify and code all additional constructs that relate in some way to those in the RQ.
4. Add all identified constructs to the article-specific construct table.
5. For each construct, return to the article and identify a definition for that construct. Code, then copy and paste definitions into the construct table, filling in ‘Not defined’ for those constructs without definitions.
6. Return to read the abstract, introduction, and theoretical framework sections. Wherever a relationship is specified or hypothesised between constructs, apply codes denoting vertical, horizontal, or associated/causal relationships as appropriate.
7. Read the abstract, introduction, and theoretical framework sections and locate a segment of text that articulates the theoretical framework used by the article, according to the theory-coding-framework. Apply one or more of the a priori author-reported framework codes as appropriate.
8. Create a visual representation of the theoretical framework using symbols to denote each construct used in the article.
9. Consult the construct table and for each well-defined construct, return to the article and determine if and where that construct is operationalized, applying codes and pasting text into the table as appropriate.
10. For each defined construct which is not directly operationalized, consult the construct-relationship codes and determine if it has been operationalized indirectly. Repeat until no more indirect operationalizations are identified, and code remaining constructs as not operationalized.

Once all articles under review had been coded, we proceeded by synthesizing the article-specific, author-reported emic constructs into a global set of analyst-generated etic constructs. We use the terms emic and etic to distinguish between knowledge as used and understood by participants of research compared to how that knowledge is then understood and classified by researchers. In our case, we use emic to refer to objects (theories, constructs, etc) using the terminology, definitions, and categorizations of the authors of our subject literature, while etic refers to our use of objects as termed, defined and categorized by us as analysts. This was done by refining the initial theory-coding framework and by generating ideal-type representations of etic frameworks. This second stage was executed according to the following summarized steps:

1. For each cluster created through theory coding, perform a *within-comparative-analysis* on visual summaries to determine internal uniformity. Where clusters are considered not-uniform, apply ‘Bridging Framework’ codes to the summaries to subdivide a cluster into two or more domains.
2. Following *within-analysis*, select a representative example from the most recent set of clusters, and perform a *cross-comparative analysis* to determine if each cluster is mutually distinct. Where two representatives are considered to be of a kind, apply Bridging Framework codes to merge clusters.
3. Within each framework cluster, compare visuals and select as ‘framework-defining constructs’ those constructs with suspected equivalences across all or all but one article in the cluster (for clusters containing only one article, choose six constructs at the highest level of generality) and code then as ‘key emic constructs’.
4. Perform a *within analysis* of each key emic construct through comparing definitions from each article reporting an identically-named construct to determine whether identically-named article-specific constructs do represent the same phenomena. Where construct definitions differ, apply the code ‘Bridging Constructs—unrecognised divergence’ to create two (or more) domains. Where under-reporting of construct definitions obstructs within-analysis, apply the code ‘Bridging constructs—poor definition’ to the poorly defined construct.
5. Select representative definitions for each uniform and/or singular construct grouping and conduct a *cross-analysis* to determine if all construct groupings do indeed refer to distinct constructs. Where distinctly-named constructs have equivalent definitions, apply the code ‘Bridging constructs—duplicate correction’ in order to merge them.
6. If any constructs have been merged or sub-divided, return to the visual summaries and re-perform a *within* and *cross* analysis of theoretical framework clusters following steps 1 and 2.
7. Compile a report of the most recent set of theoretical frameworks, listing their constructs, and of the most recent set of constructs, listing all definitions. This report is to be reviewed by a member of the team with expertise in the subject-matter. If changes are recommended, the team is to verify changes, making additional splits and mergers where necessary, using the review methods described thus far. This process is to be repeated until agreement is reached between the lead reviewer and the expert.
8. The resulting set of frameworks and constructs constitutes the review’s set of etic, analyst-generated frameworks and constructs.

To move from author-reported to analyst-generated theoretical frameworks and constructs we used domain analysis [29] and the constant comparative method [30] at both conceptual levels. Each cluster of author-reported theoretical frameworks was analyzed for internal uniformity based on presence of suspected equivalent constructs. Representative examples from each uniform cluster were subsequently compared. Once we had made a selection of key constructs from each framework, construct definitions were analyzed for internal uniformity within groups of commonly-named article-specific constructs. We then compared representative definitions of all constructs that were key to defining a framework. Following these steps, the entire set of frameworks was examined to see if changes in constructs had implications for the categorization of frameworks.

At both levels this analysis resulted in splits and mergers of the initial set of frameworks and constructs coded in Stage 1. Where this was done (or where it was frustrated due for example to absent construct definitions) the process was recorded using codes. This enabled us to track the process, and was used in later stages to match article-specific operationalizations to analyst-generated constructs. Once a stable categorization of frameworks and constructs had been reached, this was compiled into a report that was examined by a team member who had expertise in the subject matter. This member critically reviewed the report and sought to identify instances where the categorizations created through the structural review were not meaningful from the perspective of the field. Where changes were recommended, the lead reviewer attempted to refute them on an evidentiary basis. This interchange, based on the principles of refutational synthesis [31], continued until consensus was reached between the two team-members, signifying agreement reached between two modes of enquiry—empirical and expert-guided. The set of frameworks and constructs agreed on was then used in the remainder of the review as, analyst-generated etic representations.

After these two stages, we then conducted transparency, validity, and feasibility tests, modeled on Kampen and Tamás [32] and da Silva et. al. [33], on all articles-specific operationalizations. We subsequently integrated those remaining transparent, valid, and feasible operationalizations into the ideal-type etic theoretical frameworks. However, these steps of the methodology are not of direct relevance to the purpose of this article and will not be recounted. Readers interested in these extra steps are directed to the technical report produced for this project [3].

### Data collection

We executed the above methodology on 35 research articles on local-level climate vulnerability that had been selected through the initial phases of systematic review. Details of these articles, and the search and screening process through which they were selected have been published previously [3]. We identified 358 article-specific constructs, 281 of which were defined and 155 of which were directly operationalized. We coded articles for the theoretical framework based on authors’ own declaration of which framework they were using and clustered articles on that basis. Subsequent inductive examination of articles was able to identify and correct for instances where authors’ declarations were inconsistent with one another.

We were able to identify 114 framework-defining constructs and through analysis found that authors’ use of terms did not reliably predict common definition and that in some cases authors did not provide definitions sufficient to interpret and compare their constructs. Constructs that had the same name across papers were treated as suspected equivalents (n = 14). These 14 were inspected for within-uniformity through comparing article-specific definitions. This set of provisional constructs was then cross-compared with one another and with the remaining 100. Some of these were found to have equivalents that did not share the same name. To correct for inconsistencies in authors’ use of language we merged constructs which were found to equivalent following inspection of definitions. Unfortunately, we were not able to use this method on all constructs, as definitions were not consistently provided in reports.

We selected and reported framework-defining constructs using the corrections produced in the previous step. The subject matter expert inspected the corrections suggested through inductive study of the reports. Delaney et. al. [3] found that it was effective to work back and forth between expert opinion and inductively-generated evidence by a methodologically competent but subject-matter ignorant lead investigator in reaching consensus on both specification of frameworks and constructs.

## Results

Comparison of frameworks and constructs as defined by authors and as defined through systematic examination produces inconsistent results. This inconsistency calls into question the validity of assuming that authors’ use of the same words indicates common definitions/operationalizations and vice versa. For example, the common construct ‘Adaptive Capacity’, fully and transparently operationalized in only four articles, revealed four different paths of operationalization. This heterogeneity was found despite authors’ presenting definitions that suggest conceptual equivalence (see Table 3).

**Table 3:** Operationalization of ‘Adaptive Capacity’ [27].

Subconstruct (level 1)	Subconstruct (level 2)	Indicators	Articles
Livelihood Assets	Social Capital	Community organization membership	Piya et al
		Access to credit	
	Physical Capital	Irrigation	Antwi-Agyei et al
		Communication devices	
		Type of house	Piya et al
		Communication devices	
		Distance to road	
		Irrigation	
	Natural Capital	Farm size	Antwi-Agyei et al
		Tenure system	
		% of productive land	Piya et al
		Livestock	
	Financial Capital	Credit	Antwi-Agyei et al
		Livestock	
		Remittances	
		Household income	Piya et al
		Livelihood diversification	
		Household savings	
		Livestock	
		Membership of community orgs	
	Human Capital	Education Level	Antwi-Agyei et al
		Health Status	
		Education	Piya et al
		Dependency	
		Trainings	
Socio-demographic profile; livelihood strategies; social network	Socio-demographic profile	Dependency ratio	Hahn et al
		Female headed households	
		Uneducated headed households	
		Households with orphans	
	Livelihood strategies	Households working elsewhere	Hahn et al
		Livelihood diversification	
		Agriculture dependent household	
	Social network	Receive/give ratio	Hahn et al
		Borrow/lend ratio	
		Independent of local government	
Direct Operationalizations		Number of cultivated production zones	Sietz et al
		Crop area	
		Livestock units	
		Potato productivity	
		Quinoa productivity	
		Education level of household head	
		Local off-farm income and remittances	

A systematic approach to review that uses inductive methods, therefore, is valuable in flagging heterogeneity in nominally commensurable constructs and operationalizations, and in demonstrating where verifiably commensurable constructs, and their operationalizations, can be interchanged. Inductive examination of reports of primary research may be particularly useful in domains where the social dynamics that inform commensuration reward authors for indicating compliance through use of accepted terms (such as ‘adaptive capacity’) but do not check if the associated operationalizations and definitions follow suit.

For instance, initial scanning of the articles resulted in the identification of four recurrent frameworks for the study of vulnerability—Inter-governmental Panel on Climate Change (IPCC) Vulnerability Definition; Vulnerability as expected poverty (VEP); Food Security; and Livelihoods—which were partially confirmed by consultation with experts during literature gathering. We created a coding framework from this set of recurrent approaches using authors’ own declarations as indicators. Nevertheless, even such a ‘deductive-light’ approach, where we used an *a priori* framework operationalized through authors’ self-representation, ultimately had little verifiable relationship to the actual patterns of theory use. Nineteen of the thirty-five articles were coded with the miscellaneous ‘Other framework’, while analysis based on construct presence effectively eliminated of two of the *a priori* theory codes (‘Food Security’ and ‘Livelihoods’) which were found to be concepts used across multiple frameworks, rather than theoretical frameworks in their own right.

If authors represent their research in a manner that is compliant with social expectations, then the method we have followed here is likely to be adequate. Nonetheless, the conclusions of systematic reviews of published records are, by definition, limited to the contents of those publications themselves unless evidence is provided that those reports are valid indicators of research practice and, as such, support inference beyond the text examined. Observations on the political and legitimation functions of citation practices [34], for example, cast serious doubt on the assumption that research reports accurately reflect research practices. In summary, we consider the value of our inductive approach to be demonstrated. Deductive study of our articles, in which authors’ labels are accepted at face value, would have held us hostage to the vagaries of authors’ terminological choices. This same instability, however, makes us uncertain whether authors’ descriptions, even when corrected, adequately represent their practice.

It is useful to combine empirically-guided and expert-guided analysis and synthesis in systematic review of articles for theory, but this approach may benefit from having more than one content expert on the team. A purely expert-guided review makes the analysis vulnerable to some combination of expert biases in interpretation, authors’ inconsistent use of language and uneven depth of reporting. A purely empirically-guided approach may be unworkable as, if our articles were at all indicative, authors’ theoretical sections often include far more constructs than they define, operationalize or use in the studies they report and articles appear to be accounts whose necessary partiality is in part shaped by social norms known to experts. In response to these two constraints, we accepted authors’ reported framework as an initial hypothesis. We then successfully structured an analysis that combined bottom-up identification of the components of a framework with expert correction of those identifications based on the technique of refutational synthesis. For example, we initially distinguished between three uses of the term ‘adaptive capacity’ (AdCap). Quoting from our technical report [3], subsequently the subject-matter expert rejected this split, acknowledging that although definitions differed, the degree difference was not sufficient to warrant treatment as distinct constructs:

AdCap B and C emphasize household level adaptation rather than the vague “system” of A and they also point more toward AdCap as a practice than a quality. However, they are not fundamentally incompatible with AdCap A.

Similarly, with the case of the Vulnerability as Expected Poverty framework and its variants, the lead researcher originally classified these as distinct. However, neither a split nor a merger could be unambiguously confirmed nor disproved based solely on the empirical presence of constructs. As it was not possible to draw a conclusion based on the evidence, we accepted the subject matter expert’s recommendation that the variations were better classified as a single framework exhibiting a core approach. One legitimate concern with use of an expert is that experts’ interpretations are informed by their personal backgrounds. Their clarity of vision, in short, may be one of several equally plausible, but mutually incompatible views. Forcing experts to exercise judgment at the level of inductively identified components of frameworks may stop them from relying on heuristic assessments based on the labels used by authors. However, the same biases may operate at the level of constructs and operationalizations. Though we had neither the time nor the resources required to do so, we strongly encourage systematic reviewers to work with a range of experts.

It is appropriate to describe articles in terms of constructs, construct definitions, sub-constructs, relationships between (sub)constructs, and operationalizations. Not all articles reported at the same level of resolution. That is, some articles reported in fine detail and contained additional elements beyond those anticipated in the *theory of theory*, while others who reported from a broader perspective were found to collapse two or more of these elements into single objects. Articles that reported their theory at lower or higher levels of resolution than that in our coding framework frustrated its application. There were four circumstances in which inadequate reporting was particularly relevant. First, we were unable to compare a number of constructs (37 out of 114) because authors failed to report definitions. Second, we found that authors did not consistently and explicitly differentiate between conceptual and operational definitions (what a construct is supposed to represent as opposed to how it is measured). Third, we could not code relevant constructs reliably because authors tended to mention far more constructs in their theoretical discussions than were found in their actual research. Finally, authors did not distinguish in consistent levels of detail between sub-constructs, indicators and measures during operationalization of a construct.

As clarity on each of these points is required in order to determine the commensurability of research, we concluded that our coding framework (introduced above and in part presented in S2 Table) constituted an appropriate minimum standard for reporting. When confronted with articles that reported in greater detail, our representations destroyed some information. We found that, while many articles failed to provide the level of resolution we expected, very few exceeded our expectations. In terms of coding reliability, the most straightforward article to code was that of Baca [35], which explicitly stated in their research question any constructs they would use to address their research question and then defined ‘vulnerability’ and its sub-constructs. Similarly, in terms of distinguishing steps of operationalization, the only report to have, commendably, exceeded our level of resolution was that of Hahn [36]. We could have reduced our expectations, but we chose not to because adjusting the resolution of our method would result in more data loss from the better quality articles and make invisible the uneven reporting we found in most cases.

Construct-centered methods aggregation, which examines theoretical frameworks through their constructs, is able to identify moments of incoherence that are not visible through approaches that focus only on frameworks or on constructs in isolation. Inductive examination of definitions and operationalizations showed that authors variably conceived of “vulnerability” as an internal state of being, or as the outcome of a set of external drivers, differences which to a certain but limited extent correspond to difference in theoretical frameworks used. For example, the econometric articles of the VEP approach characterized vulnerability as when one has large probability of becoming poor due to environmental shock, and sought to identify socio-economic independent variables statistically associated with higher probability levels. In contrast, many of the indicator-based approaches compiled indices which were used to create a value for a household or area and then identify the most important factors that contributed to vulnerability in the study site. In examining these differences, we found that there was no consistency in terms of how authors address vulnerability *outcomes*, (e.g. the problems faced when vulnerable, as opposed to the probability of facing problems), nor in terms of *drivers* and underlying causal mechanisms (e.g. determining factors contributing to particular outcomes). At the more explicit level of research objectives, measuring vulnerability as an outcome (how vulnerable is this community?) is very different than analyzing the factors that produce vulnerability (what is causing this community to be vulnerable?). Worryingly though, this difference is rarely articulated explicitly and only became apparent to us when we attempted to systematically categorize constructs posited as drivers of vulnerability, but failed to do so reliably when faced with unstated underlying theoretical differences. Had we adopted a less rigorous approach to our study or one which examined constructs and frameworks in isolation from one another, we may not have identified this fundamental conceptual fissure in the literature, resulting in reproduction of their conflation. Instead, the rigor of the method applied enabled us to identify theoretical incommensurabilities in literature whose deceptive coherence might otherwise encourage unwarranted comparison of results. This was important because the initial ambition of the research was to use methods identified through empirical examination to support a continental development program to expand future work on vulnerability assessment.

### Challenges Encountered

The review method we used, construct-centered methods aggregation, has a built in bias that favors evaluations that use well established and deductive methods over inductive or exploratory research, and over innovative approaches or those using complex methods. Inductive studies necessarily use constructs as tools to *organize* rather than *generate* empirical data. Their construct definitions are more valued for their flexibility and capacity for development rather than clarity of boundedness. Transparency protocols in the review required strong construct definitions (in order to be able to test for validity, a quality criterion which in any case has less traction in inductive research, particularly qualitative). In pilot papers, much more space is generally given to the description of the theoretical framework than to explaining precisely how data was gathered and analyzed. Our method, therefore, discriminates especially against complex frameworks, as is often found in those using mixed methods, simply because the space required for adequate description is greater. This is particularly important in climate vulnerability research as this requires analysis of interactions between and within biophysical and socio-economic phenomena.

The bias in our design in favor of deductive studies also resulted in inductive studies being coded as having a frame that is ‘not defined’. The term ‘not defined’ carries negative connotations which are easy to interpret as indicating inferiority. As qualitative methods were disproportionately used in inductive studies, the normative interpretation of ‘not defined’ as inferior risks reinforcing the notion that qualitative inquiry is necessarily inferior. This interpretation is incorrect. The codes ‘defined’ and ‘not defined’ are project-specific instrumental valuations, not objective quality appraisals. In the context of our review, this bias is defensible. The goal of the review was to recommend quality methods of operationalizing ‘vulnerability’ suitable for monitoring and evaluation. As such, deductive study was appropriate. However, with a view to commensurability of research more generally, the bias in our design in favor of deductive studies should be corrected. What is needed is a framework where equivalent quality criteria for deductive as well as inductive research can be invoked as appropriate. With this in mind, we conclude that parts of the construct-centered methods aggregation we used are not suitable for reviews of inductive research.

Results of our construct-centered methods aggregation are sensitive to arbitrary decisions in the design, as also encountered by others (i.e. [2, 18, 37]). Systematic review requires consistent and even treatment of all data. For each framework where we had only one example study, we decided to examine the six most important constructs rather than selecting constructs based on prevalence as was done for frameworks with multiple exemplars. This choice produced two problems. First, in some cases the exemplar article did not fully specify six constructs. In clusters of more than one article, individual failure was compensated for by other articles’ coverage. This same complementarity did not take place in single-article clusters. As such, frameworks that were represented in only one article appear less well specified than those represented multiple times, independent of reporting adequacy. Second, the number ‘six’ is truly arbitrary and this limit did a disservice to some articles. For example, the current and future vulnerability framework, used by Ford [38] requires three constructs to fully report on two conceptual levels, or seven, to fully report on three conceptual levels. With six constructs, the third conceptual level in not fully reported. The method we used should be amended such that it is more sensitive to the requirements of the frameworks reviewed and sampling should be amended such that there are, ideally, at least three articles within each cluster.

Our construct-centered methods aggregation assumed transitivity and this assumption was not always merited. In synthesizing equivalent or commensurable constructs, we assumed that if construct A is equivalent to construct B, and B is equivalent to C, then A must also be equal to C. In general this assumption was plausible. However, among the VEP papers, some constructs were reported with different levels of resolution and conceptual development. We encountered detailed constructs which were found to be distinct, but also less-detailed constructs which could be collapsed with both. This was the case with a series of constructs (‘household consumption’; ‘minimum consumption (income) level’; ‘poverty’; ‘poverty line’; and ‘welfare indicator’) which each provided overlapping elements of an incoherent whole. In the review, these article-specific constructs were merged as one etic construct, in effect collapsing levels to synthesize a broader construct from its uneven parts. While we wished to avoid interpretive as opposed to aggregative forms of synthesis (see Dixon-Woods [21] for distinction between interpretive and aggregative types of syntheses; see Dubois [39] for an example of interpretive synthesis of constructs), it was unavoidable as this set of constructs was neither entirely distinct, nor could they be reliably categorized. As the goal of our review was to generate a set of analyst-defined, etic constructs and frameworks, we encountered a limitation in the extent to which purely aggregative syntheses can be applied to data in which partialities of reporting are inconsistently and socially informed.

As argued earlier, the level of resolution we expected is appropriate because it supported our objectives and set expectations of reports that were exceeded only rarely. There is one exception to this. We initially coded for both constructs and for relationships between constructs. However, we did not examine the implications of construct relationships for the structure a theory. Consequently in our synthesis we only used relationships specifying taxonomic hierarchy. If a review of theory wished to identify causal models, as had been originally intended, direction and conditionality of construct-relationships would be important. That said, one of the reasons we dropped detailed examination of relationships between constructs was inconsistent and inadequate reporting of those relationships, along with unstated differences in underlying conceptions of causality. Looking at the question of resolution, then, we find that our approach requires extension for the generation of causal models, but that the realization of this goal will likely be frustrated by other factors.

## Discussion

Some of the limitations we encountered in our study suggest areas for future methodological development. The first of these concerns the bias in our study in favor of established deductive methods. Extension of our method to better accommodate inductive research will require theoretical development as to what constitutes equivalence between constructs that are not fixed and defined in such a way as to allow variability, how to account for constructs which might be at different stages of conceptual development and operationalization of a conception of quality that is compatible with inductive research methods. While there is agreement that the ‘validity’ criterion familiar in deductive research is unworkable in inductive inquiry, there is no agreement on what should take its place [4, 21, 40–44]. One contribution we might make to this discussion is that authors tended to reference far more constructs in their theoretical discussion than they eventually required for the research they reported. In the papers we reviewed it was not possible for us to entirely reliably distinguish between those constructs that were essential, those that supported and those that were superfluous to their actual study. If journal editors made clear their expectations of reporting, then authors might be more careful in the introduction and then proper specification of only those constructs they require. The specification of a standard for reporting by journal editors may, thus, encourage a transparency that supports first interpretation and, through that, comparison of results across studies.

A second area for development concerns the integration of expert- and empirical- analysis. We successfully used a form of refutational synthesis between these two forms of knowledge. Although we find strong reasons to encourage the use of systematic approaches in the review of theory and methods, on reflection we find that expert-empirical dialogue is advised as this acknowledges that theoretical frameworks and the accounts given of their operationalization are socially constructed culturally-specific objects. As such, a purely technical investigation can only go so far without the culturally-informed input required to make sound inferences. In our own study, resource and time limitations forced us to work with one form of dialogue with one expert and as such we were unable to identify any patterns in how expert competence and inclination biased decision. Wider expert consultation would improve analysis of the frameworks. We encourage experimentation with different methods to elicit and synthesize insights between systematic reviewers and subject matter experts. Methods such as refutational synthesis, focus groups, dialogical methods, or Delphi method may be useful in reviewing articles from a literature in which authors unevenly rely on expert readers to make appropriate inferences.

As has been found by others (e.g. Hallfors [45], Castleden [17] and Schultz [46]) the adequacy of reports as a unit of analysis was a recurrent theme encountered throughout our entire review process. Our review initially assumed that reports provided a valid indication of underlying research, but this assumption has not been well supported by the findings. We have, above, suggested that a number of issues we encountered could be dealt with through using a minimum of three exemplar reports in order to identify the constituent elements of a framework. However, we also recognize that this may neither be possible nor adequate. A second option is for journals to increase the length allowances for articles. A third may be for articles to be recognized as public and accessible summaries of immediately available and painstakingly detailed technical reports which, taken together, constitute an article. Fourth, review may choose to recognize as legitimate requesting authors to fill in missing cells in a standardized data extraction form, interviews with researchers, or even field studies whose purpose is to test the validity of reports of research.

These extensions treat the article as a socially shaped partial index of a particular research project and, using it as a starting point, identifies and draws on valid supplemental sources of data until either an adequate picture of how the research has been conducted is obtained or those supplemental sources are exhausted. Each of these extensions will bring problems, relating both to the methods themselves (e.g. getting researchers to open up in an interview and express uncertainty over their work) and to their consistency with the principles of systematic review (e.g. the influence of the reviewer in generating data and the variance caused by author non-response would destroy any vestige of reliability and consistency). Nevertheless, reliance on refereed publication appears constraining sufficiently to justify exploration of alternatives, especially in fields that have complex and diverse conceptual, methodological and reporting practices, such as climate impact studies. This may be especially challenging for more operational outcome driven programs that straddle scientific research and development. These programs often operate on annual budget cycles which means that the turn-around time for peer reviewed consensus and rigor required for a comprehensive systematic review are not feasible to guide annual or even bi-annual planning.

Until such time as every published report of research is backed up by an immediately accessible fully specified technical report, review that wishes to make inferences from published report to practice must be sensitive to the peculiarities of the form of the report included in review. Thus, this research has found grounds to expand coding standards to permit variations across report format and/or draw on subject matter experts to assist in their interpretation. Theses, conference papers, working papers and journal articles comprise a subset of formats used to report research, with monographs, power points, lectures, open datasets and archives, private research diaries, and book chapters being examples of other formats (and even within the format of journal articles, practices and functions vary widely from journal to journal). A single research project will usually be reported in a variety of these media, each of which highlights and obscures aspects of research that others do not. Although a fundamental principle of systematic review is the consistent treatment of all sources, it does not make sense to treat all reports equally when we know that their form and content systematically vary from the ideal of a fully adequate technical report.

## Conclusion

Research on climate change vulnerability and adaptive capacity is expanding and gaining political significance. This growing relevance increases pressure to conduct systematic reviews of evidence that draw valid conclusions across individual studies. Review of this body of evidence is confounded by a lack of conceptual and methodological coherence as well as by inconsistent reporting. Systematic review, if properly adapted for the study of methods, is uniquely suited to identify the fault lines of incoherence and to render rigorous and transparent description of the varied ways in which concepts and methods are used.

The construct-centered methods aggregation approach to systematic review reported in this article integrates inductive and deductive analytical methods. Its combination of author-reported and expert-guided analysis is designed to accommodate heterogeneity of theory and of research and reporting practices of the sort found in studies of climate change vulnerability and adaptive capacity. This method enables constructive engagement within fields of research that are not yet conceptually and methodologically coherent while maintaining the transparency and rigor that are fundamental strengths of systematic review.

Empirically demonstrating variability in concepts and methods contributes to commensuration by providing a transparent and empirically grounded framework within which researchers may discuss how, when, or even whether, to move toward greater coherence. More importantly, it does so without presuming to impose an orthodox conceptual or methodological standard that would constrain scientific innovation. A stronger facilitated connection between climate change vulnerability researchers (especially focused on qualitative approaches) to engage with systematic review and methodology experts at the inception or planning of research could significantly alter the trajectory of the planned research towards more rigorous approaches feasible within the time, budget and context of the individual research project’s aims. In the long-term, improved transparency in reporting, effected first through reviews conducted on the model we suggest and eventually, we hope, through the improved reporting motivated in part by researchers’ desire to have their reports included in reviews, will provide an increasingly secure framework in which researchers may both interpret existing reports of research and consider the relative merits of the options they have for undertaking future research.

## Supporting Information

S1 File Technical report. Technical report.
(PDF)

S2 File Source articles and bibliography. Source articles and bibliography.
(ZIP)

S3 File Research questions posed in systematic review. Research questions posed in systematic review.
(PDF)

S1 Table Example of data extraction form, as used for Hahn [29]. (PDF)

S2 Table Framework for coding of author reported frameworks. Framework for coding of author reported frameworks.
(PDF)

## References

[1] Espeland WN, Stevens ML. Commensuration as a social process. Annu Rev Sociol. 1998;24: 313–343. 10.1146/annurev.soc.24.1.313

[2] Plummer R, de Loe R, Armitage D. A Systematic Review of Water Vulnerability Assessment Tools. Water Resour Manag. 2012;26: 4327–4346. 10.1007/s11269-012-0147-5

[3] Delaney A, Chesterman S, Crane TA, Tamás PA, Ericksen P. A systematic review of local vulnerability to climate change: In search of transparency, coherence and compatability. CCAFS Work Pap. 2014;97 Available: http://hdl.handle.net/10568/56692.

[4] Dixon-Woods M, Cavers D, Agarwal S, Annandale E, Arthur A, Harvey J, et al Conducting a critical interpretive synthesis of the literature on access to healthcare by vulnerable groups. BMC Med Res Methodol. 2006;6: 35 10.1186/1471-2288-6-35 16872487 PMC1559637

[5] Lakerveld RP, Lele S, Crane TA, Fortuin KPJ, Springate-Baginski O. The social distribution of provisioning forest ecosystem services: Evidence and insights from Odisha, India. Ecosyst Serv. 2015;14: 56–66. 10.1016/j.ecoser.2015.04.001

[6] Pronk M, Maat H, Crane TA. Vulnerability Assessments as a Political Creation: Tsunami Management in Portugal. Disasters. Submitted. 10.1111/disa.12223 27982460

[7] Weiler V, Udo HMJ, Viets T, Crane TA, De Boer IJM. Handling multi-functionality of livestock in a life cycle assessment: the case of smallholder dairying in Kenya. Curr Opin Environ Sustain. 2014;8: 29–38. 10.1016/j.cosust.2014.07.009

[8] Beauchamp A, Backholer K, Magliano D, Peeters A. The effect of obesity prevention interventions according to socioeconomic position: a systematic review. Obes Rev. 2014;15: 541–554. 10.1111/obr.12161 24629126

[9] Edwards PN. “A vast machine”: Standards as social technology. Science. 2004;304: 827–828. 10.1126/science.1099290 15131292

[10] Lepori B, Bonaccorsi A. The socio-political construction of a European census of higher education institutions: design, methodology and comparability issues. Minerva. 2013;51: 271–293. 10.1007/s11024-013-9235-9

[11] Lovell H. Climate change, markets and standards: the case of financial accounting. Econ Soc. 2014;43: 260–284. 10.1080/03085147.2013.812830

[12] Ascui F, Lovell H. As frames collide: making sense of carbon accounting. Account Audit Account J. 2011;24: 978–999. 10.1108/09513571111184724

[13] Timmermans S, Epstein S. A World of Standards but not a Standard World: Toward a Sociology of Standards and Standardization*. Annu Rev Sociol. 2010;36: 69–89. 10.1146/annurev.soc.012809.102629

[14] Merry SE, Coutin SB. Technologies of truth in the anthropology of conflict: AES/APLA Presidential Address, 2013. Am Ethnol. 2014;41: 1–16. 10.1111/amet.12055

[15] Alastalo M, Pösö T. Number of Children Placed Outside the Home as an Indicator—Social and Moral Implications of Commensuration. Soc Policy Adm. 2014;48: 721–738. 10.1111/spol.12073

[16] Brunsson N, Rasche A, Seidl D. The Dynamics of Standardization: Three Perspectives on Standards in Organization Studies. Organ Stud. 2012;33: 613–632. 10.1177/0170840612450120

[17] Castleden M, McKee M, Murray V, Leonardi G. Resilience thinking in health protection. J Public Health. 2011; 369–377. 10.1093/pubmed/fdr027 21471159

[18] Liquete C, Piroddi C, Drakou EG, Gurney L, Katsanevakis S, Charef A, et al Current Status and Future Prospects for the Assessment of Marine and Coastal Ecosystem Services: A Systematic Review. PLoS ONE. 2013;8: e67737 10.1371/journal.pone.0067737 23844080 PMC3701056

[19] Magarey JM. Elements of a systematic review. Int J Nurs Pract. 2001;7: 376–382. 10.1046/j.1440-172X.2001.00295.x 11785440

[20] Thomas, James, and Harden Angela. Methods for the Thematic Synthesis of Qualitative Research in Systematic Reviews. BMC Medical Research Methodology. 2008;8 (1): 45 10.1186/1471-2288-8-45 18616818 PMC2478656

[21] Dixon-Woods, Mary, Bonas Sheila, Booth Andrew, Jones David R., Miller Tina, Sutton Alex J., Shaw Rachel L., Smith Jonathan A., and Young Bridget. How Can Systematic Reviews Incorporate Qualitative Research? A Critical Perspective. Qualitative Research. 2006;6 (1): 27–44. 10.1177/1468794106058867

[22] Bing-Jonsson, Pia Cecilie, Ida Torunn Bjørk, Dag Hofoss, Marit Kirkevold, and Christina Foss. Instruments Measuring Nursing Staff Competence in Community Health Care: A Systematic Literature Review. Home Health Care Management and Practice. 2013;25 (6): 282–94. 10.1177/1084822313494784

[23] Dubois, Carl-Ardy, Danielle D’Amour, Marie-Pascale Pomey, Francine Girard, and Isabelle Brault. Conceptualizing Performance of Nursing Care as a Prerequisite for Better Measurement: A Systematic and Interpretive Review. BMC Nursing. 2013;12: 7 10.1186/1472-6955-12-7 23496961 PMC3600011

[24] Le Reste, Yves Jean, Patrice Nabbe, Benedicte Manceau, Charilaos Lygidakis, Christa Doerr, Heidrun Lingner, Slawomir Czachowski, et al The European General Practice Research Network Presents a Comprehensive Definition of Multimorbidity in Family Medicine and Long Term Care, Following a Systematic Review of Relevant Literature. Journal of American Medical Directors Association. 2013;14: 319–25. 10.1016/j.jamda.2013.01.001 23411065

[25] van der Lee, Johanna, Lidwine Mokkink, Martha Grootenhuis, Hugo Heymans, and Martin Offringa. Definitions and Measurement of Chronic Health Conditions in Childhood: A Systematic Review. JAMA?: The Journal of the American Medical Association. 2007;297 (24): 2741–51. 10.1001/jama.297.24.2741 17595275

[26] Wells, Kathleen, and Littell Julia H.. Study Quality Assessment in Systematic Reviews of Research on Intervention Effects. Research on Social Work Practice. 2009;19 (1): 52–62. 10.1177/1049731508317278

[27] Carroll C, Booth A, Leaviss J, Rick J. Best fit? framework synthesis: refining the method. BMC Med Res Methodol. 2013;13: 37. 23497061 10.1186/1471-2288-13-37 PMC3618126

[28] Morse JM. Constructing Qualitatively Derived Theory: Concept Construction and Concept Typologies. Qual Health Res. 2004;14: 1387–1395. 10.1177/1049732304269676 15538006

[29] Borgatti SP. Cultural Domain Analysis. J Quant Anthropol. 1994;4: 261–278.

[30] Glaser BG. The constant comparative method of qualitative analysis. Soc Probl. 1965;12: 436–445.

[31] Noblit GW, Hare RD. Meta-ethnography: Synthesizing qualitative studies. Newbury Park, California: Sage; 1988.

[32] Kampen JK, Tamás PA. Should I take this seriously? A simple checklist for calling bullshit on policy supporting research. Qual Quant. 2014;48: 1213–1223. 10.1007/s11135-013-9830-8

[33] da Silva S, Tamás PA, Kampen JK. Is research on Latin American social movements interpretable? Using systematic review to test for transparency and structure prior to quality assessment. Lat Am Res Rev. Forthcoming;

[34] Jensen, Mads Dagnis, and Kristensen Peter Marcus. The Elephant in the Room: Mapping the Latent Communication Pattern in European Union Studies. Journal of European Public Policy. 2013;20 (1): 1–20. 10.1080/13501763.2012.699656

[35] Baca M, Läderach P, Haggar J, Schroth G, Ovalle O. An Integrated Framework for Assessing Vulnerability to Climate Change and Developing Adaptation Strategies for Coffee Growing Families in Mesoamerica. PLoS ONE. 2014;9: e88463 10.1371/journal.pone.0088463 24586328 PMC3935832

[36] Hahn MB, Riederer A, Foster S. The Livelihood Vulnerability Index: A pragmatic approach to assessing risks from climate variability and change? A case study in Mozambique. Glob Environ Change. 2009;19: 74–88. 10.1016/j.gloenvcha.2008.11.002

[37] Driscoll DA, Banks SC, Barton PS, Ikin K, Lentini P, Lindenmayer DB, et al The Trajectory of Dispersal Research in Conservation Biology. Systematic Review. PLoS ONE. 2014;9: e95053 10.1371/journal.pone.0095053 24743447 PMC3990620

[38] Ford J, Smit B. A Framework for Assessing the Vulnerability of Communities in the Canadian Arctic to Risks Associated with Climate Change. Arctic. 2004;57: 389–400. 10.14430/arctic516

[39] Dubois C-A, Amour D D’, Pomey M-P, Girard F, Brault I. Conceptualizing performance of nursing care as a prerequisite for better measurement: a systematic and interpretive review. BMC Nurs. 2013;12: 7 10.1186/1472-6955-12-7 23496961 PMC3600011

[40] Delaney A, Tamás PA, Tobi H. Which standards from which disciplines? A test of Systematic Review for designing quality interdisciplinary evaluations. J Dev Eff. Forthcoming;

[41] Campbell R, Pound P, Pope C, Britten N, Pill R, Morgan M, et al Evaluating meta-ethnography: a synthesis of qualitative research on lay experiences of diabetes and diabetes care. Soc Sci Med. 2003;56: 671–684. 10.1016/S0277-9536(02)00064-3 12560003

[42] Carroll C, Booth A, Lloyd-Jones M. Should We Exclude Inadequately Reported Studies From Qualitative Systematic Reviews? An Evaluation of Sensitivity Analyses in Two Case Study Reviews. Qual Health Res. 2012;22: 1425–1434. 10.1177/1049732312452937 22865107

[43] Edwards AG, Russell IT, Stott NC. Signal versus noise in the evidence base for medicine: an alternative to hierarchies of evidence? Fam Pract. 1998;15: 319–322. 10.1093/fampra/15.4.319 9792346

[44] Lemmer B, Grellier R, Steven J. Systematic Review of Nonrandom and Qualitative Research Literature: Exploring and Uncovering an Evidence Base for Health Visiting and Decision Making. Qual Health Res. 1999;9: 315–328. 10.1177/104973299129121884

[45] Hallfors MH, Vaara EM, Hyvarinen M, Oksanen M, Schulman LE, Siipi H, et al Coming to Terms with the Concept of Moving Species Threatened by Climate Change—A Systematic Review of the Terminology and Definitions. PLoS ONE. 2014;9: e102979 10.1371/journal.pone.0102979 25055023 PMC4108403

[46] Schultz CA. The U.S. Forest Service’s analysis of cumulative effects to wildlife: A study of legal standards, current practice, and ongoing challenges on a National Forest. Environ Impact Assess Rev. 2012;32: 74–81. 10.1016/j.eiar.2011.03.003

