# Supplementary material for: Systematic Review of Methods in Low-Consensus Fields: Supporting Commensuration through `Construct-Centered Methods Aggregation’ in the Case of Climate Change Vulnerability Research
Source: PLoS One. 2016 Feb 22;11(2):e0149071. doi: 10.1371/journal.pone.0149071 (PMC4762661; doi:10.1371/journal.pone.0149071)
Supplement: S1 File — Technical report. (PDF) [file pone.0149071.s001.pdf]

A systematic review of local vulnerability to climate change: In search of transparency, coherence and comparability

Technical Report supporting Working Paper 97
CGIAR research program on Climate Change Agriculture and Food Security (CCAFS)

Aogán Delaney
Peter A. Tamás

Wageningen University

December 22, 2014

TABLE OF CONTENTS

INTRODUCTION 4

BACKGROUND 4

METHODS 5

SELECTION OF LITERATURE 5

Selection of literature: First review 5

Selection of literature: Second review 7

IDENTIFICATION OF CONSTRUCTS, FRAMEWORKS AND OPERATIONALIZATIONS 12

SYNTHESIS OF FRAMEWORKS AND CONSTRUCTS 17

TRANSPARENCY ASSESSMENT OF OPERATIONALIZED CONSTRUCTS. 27

VALIDITY AND FEASIBILITY ASSESSMENT OF OPERATIONALIZED CONSTRUCTS 31

INTEGRATION OF CANDIDATE OPERATIONALIZATIONS INTO IDEAL-TYPE FRAMEWORKS 32

CONVERSION TO DELIVERABLES 35

APPENDIX A: RESULTS OF RELEVANCE AND QUALITY SCREENING IN FIRST REVIEW 36

APPENDIX B: EMAIL SENT TO VULNERABILITY EXPERTS 45

APPENDIX C: ARTICLE-SPECIFIC CONSTRUCT TABLES 52

APPENDIX D: SUMMARY OF ARTICLE-SPECIFIC CONSTRUCTS IDENTIFIED 168

APPENDIX E: SELECTION OF FRAMEWORK-DEFINING EMIC CONSTRUCTS 176

APPENDIX F: RECORD OF WITHIN-COMPARISON OF EMIC CONSTRUCTS (STEP 2.12) 183

APPENDIX G: CONSTRUCT MERGERS MADE IN STEP 2.13 211

APPENDIX H: IDENTIFICATION OF FRAMEWORK-DEFINING CONSTRUCTS (STEP 2.14) 215

APPENDIX I: REVIEW OF FRAMEWORKS BY TEAM MEMBER WITH EXPERTISE IN FIELD 217

APPENDIX J: SCRUTINY BY FIRST REVIEWER OF CONSTRUCT SPLITS SUGGESTED BY EXPERT REVIEWER 224

APPENDIX K: EMIC-ETIC CONSTRUCT MAP 228

APPENDIX L: RECORDS OF OPERATIONALIZATIONS ASSESSED AS NOT TRANSPARENT OR PARTIALLY TRANSPARENT 234

APPENDIX M: VALIDITY ASSESSMENTS 284

APPENDIX N: REPORT OF SELECTED OPERATIONALIZATIONS OF RETAINED FRAMEWORKS 577

IPCC 577

VULNERABILITY AS EXPECTED POVERTY (WITH EXTENSIONS) 579

PERCEPTIONS OF CLIMATE CHANGE 586

ASSET VULNERABILITY 590

NESTED VULNERABILITY 594

CURRENT AND FUTURE VULNERABILITY 597

LIVELIHOOD VULNERABILITY INDEX 600

INTENSIFYING VULNERABILITY TO FOOD INSECURITY 618

NKONDZE ET AL (2013) 622

Patterns of smallholder vulnerability 623
Livelihood trajectories and resilience and vulnerability 628
Determinants of Resilience 630

APPENDIX O: RESULTS – FRAMEWORK SUMMARIES, CONSTRUCTS, & OPERATIONALIZATIONS .. 637

Asset vulnerability 637
Current and future vulnerability 641
Determinants of Resilience 644
Intensifying vulnerability to food insecurity 652
IPCC 657
Livelihood trajectories and resilience and vulnerability 659
Livelihood vulnerability index 662
Nested vulnerability 680
Nkondze et al 684
Patterns of smallholder vulnerability 685
Perceptions of climate change 691
Vulnerability as expected poverty, with extensions 695

APPENDIX P: QUESTIONNAIRE – CANDIDATE OPERATIONALIZATIONS 704

Adaptation strategy 705
Adaptive capacity 709
Community level 727
Covariate shocks 730
Exposure 733
Household level 742
Idiosyncratic shocks 745
Poverty 748
Sensitivity 752
Vulnerability (IPCC) 768
Vulnerability as Expected Poverty 779

APPENDIX Q: ORDER OF MEASURES AND CONSTRUCTS 782

REFERENCES 789

Introduction

This document reports on a systematic review which was done as part of a project called "Coping with Climate Change: What is vulnerability and how should it be researched?". The purpose of this report is to provide an archival record of the systematic review method that was used in the research.

Background

This research was commissioned by the International Livestock Research Institute (ILRI) and feeds into their CCAFS programme. The CCAFS programme is to be implemented and will seek to improve the conditions of those who are considered vulnerable to the effects of climate change. In order to draw conclusions about the impacts of their programme, a well-designed evaluation study will be required. This systematic review was commissioned as an input into the design of such a study. The review looked at studies of 'vulnerability' in order to generate an operationalizable research framework based on the best quality methods in the source literature.

The review had as a research question (RQ):

- How is vulnerability defined
- How is vulnerability operationalized
- Which operationalizations are empirically valid
- Which definitions do sound operationalizations support

As can be seen from the RQs, this review is concerned with reviewing methods rather than data or conclusions. It takes as its central focus an object of research, in this case *vulnerability*. An object of research is researched through a theoretical framework and a methodology. A 'theoretical framework' can be deconstructed into three components: constructs[1]; construct definitions; and relationships (Carroll, Booth, et al. 2013; Morse 2004). Of these three components, we use a definition of a *construct* as a conceptual representation of a phenomenon. A construct can itself be deconstructed into a set of *sub-constructs* or can be abstracted as higher-order construct, or possibly as a theory (Morse 2004). We take a *construct definition* as a delineation of what phenomena the construct represents, a delineation which must be bounded such that a reader can determine what does and what does not count as an example of that construct (Morse 2004). Note that constructs can be defined either conceptually (what real world phenomena does the construct represent) or operationally (what data will be used to empirically represent the construct (Morse 2004). However, this level of resolution is not carried explicitly forward in the analysis as to do so would risk that our theoretical framework would not fit our subject articles which may not adhere to such a level of resolution. We use *construct relationships* as elements of a theoretical framework which are used to link constructs together in such a way as to shape the framework.

Of these three sub-components of a theoretical framework, analytically the most important for our purposes is that of the *construct*, as it functions as an organizing unit of analysis, which form the basis of conceptual frameworks and which are themselves operationalized and thus form the link between conceptual frameworks and research methods.

Another important component of our RQ is that of *operationalization*. An 'operationalization' of a construct is used to describe any step in which a researcher moves a theoretical concept towards an actual act of gathering data to measure or represent that concept. The term operationalization in this paper is used to describe both intermediary steps of conceptual deconstruction, and the final instrumentation. Finally, we consider an

[1] For general purposes, throughout this document the terms 'construct', 'concept' and 'construction' are used interchangeably, although the use of the term 'construct' is preferred.

operationalization to be *valid* if the empirical data used to represent the construct also represents the phenomenon represented by the construct (Kampen and Tamás 2014).

Methods

The methods used to conduct this review can be organised into six broad stages:

1. Selection of literature
2. Identification of constructs, frameworks and operationalizations
3. Synthesis of frameworks and constructs
4. Transparency assessment of operationalized constructs.
5. Validity and feasibility assessment of operationalized constructs
6. Integration of candidate operationalizations into ideal-type frameworks

Selection of literature

This review was commissioned by ILRI for the CCAFS programme. A similar systematic review, but with different research questions, was also commissioned one year previously. The literature selection for the first review was also brought forward and added to in the second review. Therefore this section reports on two distinct stages of selection of literature.

Selection of literature: First review

A search was carried out across 15 scientific databases (AJOL; AGRICOLA; AGRIS; Ingenta Connect; JSTOR; Mendeley; Scholar (Google); Science Direct; Scopus; SSRN (social science research network); Springer ; ink; Web of Knowledge; Web of science; Scopus; Ebscohost). A separate search string was composed for each database reflecting the particular characteristics of that database. Search strings were based on a common set of terms which were derived from the central research question of that review[2], and then adapted to the specific databases. The common set of terms is listed as follows:

- Poverty and vulnerability to climate risk
- Rural livelihoods and vulnerability
- Food insecurity and climate risk
- Climate variability and household vulnerability [and community]
- Causes of vulnerability
- Agriculture and climate change and vulnerability outcome
- Agriculture and food security and climate change
- Vulnerability and household agriculture
- Food insecurity and household poverty
- Climate hazards and vulnerability
- Searched using vulnerability and secondly with assessment:
- Climate risk and vulnerability [assessment]
- Climate change and vulnerability [assessment]
- Food insecurity and vulnerability [assessment]
- Poverty and vulnerability [assessment]
- Climate and floods and vulnerability assessment]
- Households and vulnerability [assessment]
- Climate and drought and vulnerability [assessment]
- Vulnerability status and climate impact

[2] The Research Question of the first review is as follows:

1. What determinants of vulnerability are common across the studies?
2. What are the causal mechanisms that link determinants and vulnerability outcomes?
3. What are the methodological approaches that give most robust and reliable results in understanding determinants and mechanisms of vulnerability?

- Gender and climate change and vulnerability
- Household level vulnerability to climate change
- Poverty and vulnerability
- Climate risk assessments [and households / communities]
- Climate change vulnerability and hazard exposure
- Climate change risks and household characteristics
- Sensitivity and climate change risk and vulnerability status
- Droughts and household food security and vulnerability
- Floods and household food security and vulnerability
- Climate risk [and hazard] and food security
- Vulnerability determinants and climate change
- Institutions and vulnerability outcomes
- Determinants of [household] vulnerability
- Local level vulnerability assessment climate change
- Household vulnerability and climate change case studies

This search of databases returned 168 papers. Initial screening for relevance was conducted on titles and abstracts of these articles. This screening was based on the PICOTT framework and the eligibility criteria derived from this framework are summarized in the list below:

- Rural livelihoods and households
- Sub-national unit of analysis
- Poverty
- Food insecurity
- Agriculture
- Climate change
- Climate risk
- Climate variability (includes drought and floods)
- Multiple stressors including a climate-related risk

Initial screening reduced the pool of articles to 71. These 71 articles were brought forward for a full text review. This second stage of relevance and quality screening was based on the following criteria:

- Located in the global tropics
- Local level focus of assessment
- Clear research question
- Well-articulated sampling process and data collection methods
- Methodology that used empirical data (primary or secondary)
- Description of data analysis
- Analysis section went beyond simple description of determinants and attempted to unpack the causality of vulnerability
- Findings and analysis were focused on vulnerability outcomes and determinants specifically, in line with our key research question and aims, rather than topical areas such as adaptive capacity, resilience or coping mechanisms
- Draws conclusions about vulnerability determinants

The results of this second stage of screening are summarized in the table in Appendix A. 29 papers were considered to be relevant and of sufficient quality to be included in the study. These papers then constituted the subject literature for the first review. Further methods of the first review will not be detailed here as the primary focus of this report is the methods used to conduct the second review. 28 of these 29 articles were subsequently sent to the research team working on the second review and constituted the initial pool of articles.

Selection of literature: Second review

28 articles were given by the team working on the first review to the team working on the second review. As this set of literature was gathered initially for related by distinct purposes, the team conducted a second stage of literature gathering based on consultation of experts in the field (as do (Sandoval et al. 2012)). Expert selection was to be guided by the principles of purposeful and theoretical sampling, whereby the goal was to capture at least one article from all relevant approaches. That is, this review would be more analogous to grounded theory than to and RCT study, with the implication that sampling is guided by 'theoretical sampling'. What was required theoretically was one example, preferably the best example, of each framework used for the study of vulnerability. It was therefore first necessary to identify and map what approaches are present in the initial pool of 28 articles.

Initial analysis began with reading an article and drawing diagrams of the theoretical framework used in that paper. I proceeded to do this with all 28 articles[3], using some of the techniques of the constant comparative method (Glaser 1965). It is important to note that this was an unstructured exercise, so the method wasn't followed strictly.

At some point, four significant categories of difference were identified as emergent. These were:

	Category	Definition[4]	Values
1.	Data type	What type of data is used to conduct analysis and draw conclusions?	metric;<br>indicator;<br>interpretive;<br>other
2.	Dimensions of vulnerability	How is vulnerability defined and what aspects of the concept is the article interested in? This is categorised into 6 prevalent approaches based on recognised frameworks or adaptations of those frameworks.	IPCC;<br>VEP;<br>Food Insecurity;<br>Sustainable Livelihoods;<br>Resilience;<br>Other
3.	Determinants of vulnerability	What independent variables is the paper interested in investigating. What do they see as the important factors that influence vulnerability	n/a[5]
4.	Scale	This variable is concerned with the level at which data is collected and conclusions are derived. At lowest level, household data is used to conclude about those households. At the next step up, household data from one or more localities is used to draw	household;<br>household-local;<br>local-regional;<br>continental/global

[3] In the following order: (Günther and Harttgen 2009); (Bogale, Taeb, and Endo 2006); (Mubaya et al. 2012); (Chhihn and Poch 2012); (Tesso, Emana, and Ketema 2012); (Dhamija and Bhide 2011); (Antwi-Agyei et al. 2013); (Piya, Maharjan, and Joshi 2012); (Deressa, Hassan, and Ringler 2009); (Misselhorn 2005); (Hahn, Riederer, and Foster 2009); (Eakin, Winkels, and Sendzimir 2009); (Échevin 2011); (Sarris and Karfakis 2010); (Acosta et al. 2013); (Dasgupta and Baschieri 2010); (Sallu, Twyman, and Stringer 2010); (Luers et al. 2003); (Westerhoff and Smit 2009); (Nkondze, Masuku, and Manyatsi 2013); (Sietz, Choque, and Lüdeke 2012); (Capaldo et al. 2010); (Mutsvangwa 2011); (Mengistu 2011); (Gandure, Walker, and Botha 2013); (Jamir et al. 2013); (Calvo and Dercon 2013); (Notenbaert et al. 2013); (Khan and Salman 2012)

[4] Definitions are working definitions as it is a bottom-up emergent coding exercise

[5] It did not prove to be feasible to categorise determinants due to the wide variety.

		conclusions about households and about the specific characteristics of the localit(y/ies)	

Initially these were loose categories, with open-ended values. As comparison continued, I came to settle on a discrete set of values for each category with the exception of 'determinants'. The wide variety of determinants used in the articles meant that it was not really possible to create a small number of discrete values, while a large number would defeat the purpose of clustering. This category was mostly dropped in the clustering exercise that followed.

When diagrams had been drawn for all 28 articles, the notes were revised in order to give values on each of these three remaining categories of difference.

Next, I turned towards creating clusters based on these 3 remaining categories. The most significant difference was that between metric and indicator data (the majority of articles) on the one hand and interpretive data on the other. This category functions like a decision tree then. Within the 'more quantitative[6]' articles, I constructed a table tabulating 'dimensions' with 'scale'. This proved reasonably nice for clustering, but with the caveat that 'scale' seemed to be partly correlated with 'data-type'. As it turned out, almost all the quantitative studies dealt with the household or household-local level, making this category semi-redundant. This was to be partly expected as the search strategy from the first review specifically targeted studies at a local level. However, an exclusive focus on the local was not apparent on initial exploratory reading (hence the creation of additional values), and furthermore, the distinction between household and local/community scales was not uniform.

With this table, 11 clusters were created in a structured fashion.

Two additional 'residual' clusters were created: first those articles using interpretive data. Second, those quantitative articles that use frameworks that don't fit into the prevalent frameworks. This cluster set is represented in the table that follows:

Quantitative approaches.					
	IPCC and adaptations	VEP and adaptations	Food InSecurity	Sustainable livelihoods (SLA) and adaptations	Resilience
Household	Luers et al 2003 ; Tesso et al 2012; Westerhoff & Smit 2009	Bogale et al 2006; Sarris & Karfakis 2006	Capaldo et al 2010; Sietz et al 2012	sallu et al 2010	sallu et al 2010 ; Tesso et al 2012
Household – Local	Antwi-Agyei et al 2013; Hahn et al 2009; Jamir et al 2013; Piya et al 2012;	Chhinh & poch 2012; Deressa et al 2009; Echevin 2011; (gunther &	Misselhorn 2005; Mutsvangwa 2011	Antwi-Agyei et al 2013; Eakin_etal_2008 ; Hahn et al 2009; Piya et al 2012;	Antwi-Agyei et al 2013

[6] Quantitative-qualitative is an insufficient distinction. Instead an imperfect 'quantitative-interpretive' distinction is used. This distinguishes between on the one hand, approaches that seek to generate quantitative or structured categorical conclusions, eventhough many of them collect qualitative data in the process; and those whose goal is to build theory on vulnerability based on interpretive research with people exeriencing or at risk of vulnerability.

		herttgen 2009); Mutsvangwa 2011; Nkondze et al 2013			
Continental / Global				Eakin_etal_2008	

NOTE ON FRAMEWORKS:
There are 5 principle theoretical frameworks through which dimensions of vulnerability are viewed:

- those derived from the IPCC (2001; 2007) or Füssel et al (2007), based on three factors – exposure to change, sensitivity to change; adaptive capacity.
- those derived from Vulnerability to Expected Poverty (VEP) as developed by Chaudhuri et al (2002); Christiaensen & Subbarao (2005);
- Food insecurity;
- frameworks adapted from the Sustainable Livelihoods Approach (SLA) as developed by DFID (1999); Ellis (2000); Scoones (1998);
- and those that look at Resilience.

Interpretive approaches

Gandure et al 20131

- Perceptions of climate changes; risks; and adaptation strategies

Mengistu 2011

- Perceptions of climate change; vulnerable groups; and coping strategies at village level

(Mubaya et al 2012):

- Perceptions of threats to livelihoods at household – local scale.

Notenbaert 2013

- Looks at Adaptive Capacity from IPCC framework through interpretive lens at household and village level.

Miscellaneous approaches

Calvo & Dercon 2012

- Looks at vulnerability as uncertainty regarding future poverty (distinct from VEP) at household and aggregate (village) level.

Dasgupta and baschier 2010

- Vulnerability is viewed in terms of “asset vulnerability index”. This could be seen as an adaptation of VEP but seems sufficiently extended to warrant not being grouped with the others.

dhamija & bhide 2001

- Looks at determinants of both the incidence and transitory-ness of poverty, but does not relate this to climate change. (Not relevant?)

Khan & Salman 2012

- Examines vulnerability through a framework they develop called the ‘Human Vulnerability Index’, (indices; population density, lack of knowledge, lack of decent housing, lack of decent standard of living, livestock household and farm households) which is used to look at variability in district-level vulnerability across a region.

An email was composed and sent to a number of hand-chosen experts. The text of the email body and the accompanying attachment can be seen in Appendix B. Respondents were asked to look at our categorization of framework approaches to *Vulnerability* and asked two questions. First they were asked if there were additional models/frameworks that should be included in the review, and if so asked to suggest an article as an example

of that framework. Secondly they were asked if there were articles which are stronger representatives of the seven frameworks we had identified.

3 replies containing a total of 8 suggested articles were received prior to our deadline, with one further response coming after we had moved on to the next stage of the review. This brought our pool of articles up to 36. During analysis, one article (Dhamija and Bhide 2011) which was received from the first review was suspected by one member of the team to be irrelevant. Another member of the team examined this article and a decision was made to exclude it[7]. Thus the total number of articles in the review was 35. Details of this set are indexed in the table below.

Reference	Title	Included through
(Antwi-Agyei et al. 2013)	Characterising the nature of household vulnerability to climate variability: empirical evidence from two regions of Ghana	Literature search in first review
(Baca et al. 2014)	An Integrated Framework for Assessing Vulnerability to Climate Change and Developing Adaptation Strategies for Coffee Growing Families in Mesoamerica	Recommended by respondents
(Berkes and Ross 2013)	Community Resilience: Toward an Integrated Approach	Recommended by respondents
(Bogale, Taeb, and Endo 2006)	Land ownership and conflicts over the use of resources: Implication for household vulnerability in eastern Ethiopia	Literature search in first review
(Calvo and Dercon 2013)	Vulnerability to individual and aggregate poverty	Literature search in first review
(Capaldo et al. 2010)	A model of vulnerability to food insecurity	Literature search in first review
(CARE 2009)	Climate Vulnerability and Capacity Analysis: Handbook	Recommended by respondents
(Chhihn and Poch 2012)	Climate Change Impacts on Agriculture and Vulnerability as Expected Poverty of Kampong Speu Province, Cambodia	Literature search in first review
(Dasgupta and Baschieri 2010)	Vulnerability to Climate Change in rural Ghana: Mainstreaming climate change in poverty-reduction strategies	Literature search in first review
(Deressa, Hassan, and Ringler 2009)	Assessing Household Vulnerability To Climate Change: The Case Of Farmers In The Nile Basin Of Ethiopia	Literature search in first review
(Eakin, Winkels, and Sendzimir 2009)	Nested vulnerability: exploring cross-scale linkages and vulnerability teleconnections in Mexican and Vietnamese coffee systems	Literature search in first review
(Eakin et al. 2012)	Livelihoods and landscapes at the threshold of change: disaster and resilience in a Chiapas coffee community	Recommended by respondents
(Échevin 2011)	Characterizing poverty and vulnerability in rural Haiti: a multilevel decomposition approach	Literature search in first review
(Ford and Smit 2004)	A Framework for Assessing the Vulnerability of Communities in the Canadian Arctic to Risks Associated with Climate Change	Recommended by respondents
(Füssel and Klein 2006)	Climate change vulnerability Assessments: An evolution of conceptual thinking	Recommended by respondents
(Gandure, Walker, and	Farmers' perceptions of adaptation to climate	Literature search in

[7] This decision was based on the article focussing on the determinants of transitions in and out of poverty, but these determinants could not be linked to climate change.

Botha 2013)	change and water stress in a South African rural community	first review
(Günther and Harttgen 2009)	Estimating Households Vulnerability to Idiosyncratic and Covariate Shocks: A Novel Method Applied in Madagascar	Literature search in first review
(Hahn, Riederer, and Foster 2009)	The Livelihood Vulnerability Index: A pragmatic approach to assessing risks from climate variability and change—A case study in Mozambique	Literature search in first review
(Ionesco et al. 2009)	Towards a Formal Framework of Vulnerability to Climate Change	Recommended by respondents
(Jamir et al. 2013)	Farmers' vulnerability to climate variability in Dimapur district of Nagaland, India	Literature search in first review
(Khan and Salman 2012)	A simple human vulnerability index to climate change hazards for Pakistan	Literature search in first review
(Luers et al. 2003)	A method for quantifying vulnerability, applied to the agricultural system of the Yaqui Valley, Mexico	Literature search in first review
(Marshall 2010)	Understanding social resilience to climate variability in primary enterprises and industries	Recommended by respondents
(Mengistu 2011)	Farmers' perception and knowledge of climate change and their coping strategies to the related hazards: Case study from Adiha, central Tigray, Ethiopia	Literature search in first review
(Misselhorn 2005)	What drives food insecurity in southern Africa? a meta-analysis of household economy studies	Literature search in first review
(Mubaya et al. 2012)	Climate variability and change or multiple stressors? Farmer perceptions regarding threats to livelihoods in Zimbabwe and Zambia	Literature search in first review
(Mutsvangwa 2011)	Climate Change and Vulnerability to Food Insecurity among Smallholder Farmers: A Case Study of Gweru and Lupane Districts in Zimbabwe	Literature search in first review
(Nkondze, Masuku, and Manyatsi 2013)	Factors Affecting Households Vulnerability to Climate Change in Swaziland: A Case of Mpolonjeni Area Development Programme (ADP)	Literature search in first review
(Notenbaert et al. 2013)	Derivation of a household-level vulnerability index for empirically testing measures of adaptive capacity and vulnerability	Literature search in first review
(Piya, Maharjan, and Joshi 2012)	Vulnerability of rural households to climate change and extremes: Analysis of Chepang households in the Mid-Hills of Nepal	Literature search in first review
(Sallu, Twyman, and Stringer 2010)	Resilient or Vulnerable Livelihoods? Assessing Livelihood Dynamics and Trajectories in Rural Botswana	Literature search in first review
(Sarris and Karfakis 2010)	Vulnerability to Covariate and Idiosyncratic Shocks and Safety Net Targeting of Rural Households with an Application to Rural Tanzania	Literature search in first review
(Sietz, Choque, and Lüdeke 2012)	Typical patterns of smallholder vulnerability to weather extremes with regard to food security in the Peruvian Altiplano	Literature search in first review
(Tesso, Emana, and Ketema 2012)	Analysis of vulnerability and resilience to climate change induced shocks in North Shewa, Ethiopia	Literature search in first review
(Westerhoff and Smit 2009)	The rains are disappointing us: dynamic vulnerability and adaptation to multiple stressors in the Afram Plains, Ghana	Literature search in first review

Identification of constructs, frameworks and operationalizations

The nest stage was data extraction, specifically the identification of theoretical frameworks, constructs, and operationalizations used in the papers. The 35 articles were imported into NVivo and a coding protocol was designed that would allow the 35 articles to be coded evenly and transparently, and to extract data in a standardised format.

A set of instructions for this step was drawn up and pilot tested by two members of the research team (Aogán Delaney and Peter Tamás) on two articles (Mengistu 2011; Notenbaert et al. 2013). This inter-rater test was not designed as a 'hard' test with pre-defined divergence thresholds. Instead we used it as a means to spot differences in interpretation of the instructions which we then discussed in order to reach agreement on interpretation and to clarify ambiguities. Additionally a number of mechanical inefficiencies were spotted during the inter-rater test and the instructions were revised accordingly. What follows in this report recounts the execution of the revised instructions.

The revised instructions are reproduced below, with footnotes used to clarify interpretations, followed by a description of the methodological contribution of the step.

1.1 Read the abstract, introduction, and theoretical framework sections until a Research Question[8] is identified. Apply the node 'Research question' to the segment of text.

1.2 Under the node 'Article-specific constructs' create a sub-node of the form '[author] ([year])'. Within the research question, identify all constructs and for each, create a new node under the node '[author] ([year])' and apply it to the text where the construct appears. Re-read the theoretical framework and identify all additional constructs that in some way relate to those initially identified in the research question. Using the same technique, create new sub-nodes for each new construct identified.

1.3 Create a new word document with the title 'article-specific constructs'. For each article, paste a table of the form below into the document. Once coding for constructs is complete, for each article make a list in the first column of all concepts/constructs (output 1.a) that have been identified and for which nodes have been created.

1.4 For each construct, return to the paper and identify a definition for that construct. As a sub-node of the construct node, create a node called 'definition' and apply to that segment of text defining the construct. Where a definition for a construct is not given in the paper, under the construct node create and apply the sub-node 'construct not defined' to the part of the text where the construct was introduced[9]. In cases where no definition is given in the article, but where it is stated that further information is available from the authors, or where it is stated that constructs or models are adapted from another publication, create and apply the sub-node 'definition' to that segment of text where it is specified that further information is available. In the article-specific table, for each well-defined construct, fill in 'yes' in the appropriate cell and add a definition (or further information to be retrieved) in the adjoining cell. For all those constructs without a definition, fill in 'no' in the appropriate cell (output 1.a).

1.5 Return to the article and begin to read the abstract, introduction, theoretical framework, and methods sections. Whenever a relationship between constructs is specified or hypothesised, create a node relationship[10] between the appropriate construct nodes, using the most appropriate relationship type out

[8] In some cases, the paper is not concerned with executing research based on a framework, but rather seeks to generate a framework. In such cases, the RQ that would be operationalized through the resulting framework is what should be coded. And 'analytically relevant' constructs are to be derived from that.

[9] The coder may omit creating and applying codes for constructs not defined. As long as the assessments are recorded in the article-specific construct table, then silence in NVivo can suffice.

[10] For certain papers, it may be that the number of constructs makes this strategy too cumbursome. The reviewer may decide to use a different strategy to achieve the same result (although this will also entail an adaptation of 1.7): "Return to the article and begin to read the abstract, introduction, theoretical framework, and methods sections. Whenever a relationship between constructs is specified, apply the codes 'horizontally grouped', 'vertically composed', and/or 'associated or causal relationship'. Every time a piece of text is coded create a new annotation with the format: "horizontal: [list of all construct in this relationship]", "vertical: [list of

of associated-causal; vertical; or horizontal. Where a construct is deconstructed into two or more sub-constructs, create a new node relationship between the parent construct and each sub-construct. When two or more constructs form a horizontal group, create a separate node relationship linking each construct with all other constructs in the group (output 1.b)

1.6 Create a new node called "Emic Research Frameworks" and five sub-nodes called "IPCC", "VEP", "Food Insecurity", "Livelihoods Approach", and "Other framework". Return to the article and begin to read the abstract, introduction, theoretical framework, and methods sections. Locate a segment of text where it best articulates the theoretical framework used in the article and apply the most appropriate sub-node or sub-nodes.

1.7 Using the 'create model' function in NVivo, create a new model with the title 'Graphic Summary [author year]'[11]. Using the 'add project items' function, add all construct nodes under the appropriate sub-node under 'emic constructs', ensuring that 'automatically select descendant nodes' is unchecked; when prompted about selected associated data, ensure 'relationships' is checked. Again using 'add project items' add the appropriate Emic research framework node as an item, and the source as an item, ensuring all associated data is unchecked. Situate items to create a graphic representation of the theoretical framework used in the paper. Export this model as a picture (Output 1.c).

1.8 Consult the list of well-defined constructs. For each construct on that list, return to the paper and identify if and where that construct is operationalized. In cases where an operationalization can be located, create as a sub-node of the construct node the node ' 'operationalized' and apply to that segment of text defining how the construct is operationalized, fill in 'yes' in the appropriate cell in the article specific construct table, and copy the relevant text to the adjoining cell. Where a description of operationalization for a construct is not given in the paper, proceed to the next construct. When you have reached the end of the list, consult the list of node-relationships. Return to your list of constructs and for each construct not coded by 'operationalized', check to see if it has been operationalized through a sub- or higher order- or determining construct. Where the construct has been operationalized through another construct create the node 'operationalized through other' as a sub-node of the construct code, and apply to the segment of text coded at the node relationship, add the name of the mediating construct to the appropriate cell in the article-specific construct table and paste the coded text into the adjoining cell. Continue through until the end of the list of constructs. If a node of the form 'operationalized through other' has been applied during the course of the list, return and repeat. When the list is run-through without identifying any more operationalizations, for each remaining construct, locate any appearance in the article, create and apply the sub-node 'not operationalized' and fill in 'not operationalized' in the appropriate cell in the article-specific construct table (output 1.a)

1.9 Repeat Steps 1.1 – 1.8 for each article.

This coding framework was designed based on the first two research sub-questions of the review ("how is vulnerability defined" and "how is vulnerability operationalised"). At the conceptual level, a 'theoretical framework' can be deconstructed into three components: constructs[12]; construct definitions; and

all higher order constructs]| [list of all lower order constructs]", or "associated/causal: [list of all construct in this relationship]"."

[11] If the coder has coded for relationships using annotations rather than node relationships, then the instructions for this step are: "Using the 'create model' function in NVivo, for each construct on the list create a box and label with the name of the construct. Situate boxes into groups based on the relationships between models to create a graphic representation of the theoretical framework used in the paper. Using the 'add project items' function, add the current paper, and the appropriate Emic research framework codes, as items in the model. Export this model as a picture"

[12] For general purposes, throughout this document the terms 'construct', 'concept' and 'construction' are used interchangeably, although the use of the term 'construct' is preferred.

relationships; following Carroll, Booth et al (2013) and Morse (2004). Within these three components, 'relationships' can be further deconstructed into three classifications: horizontal, vertical; and associative/causal. These 6 components make the basis of the theory-coding framework.

However, consistent with previous systematic review studies (Carroll, Booth, et al. 2013; Carroll, Rick, et al. 2013), and methodologists (Morse 2004), of these 6 components, the 'construct' is the most significant in terms of the foundations of a theoretical framework. Therefore the coding framework begins by identifying constructs. In order to code only analytically-relevant constructs (and not each and every construct mentioned in discussing theoretical approaches), the coder first identifies a Research Question, and from there, constructs contained in the Research Question, or constructs that relate to those constructs, are identified.

The design is careful to maintain the article-specificity of constructs identified in this way. Rather than create a global set of nodes which can be applied to recurring constructs, the coding framework instead creates a new node for each construct in each article. As such, these constructs are referred to as *emic constructs*. In later stages of the methodology, a set of *etic* or analyst-defined constructs are generated from this set of *emic* constructs.

For each article, a table was created of article-specific constructs, their definitions, if provided, and if applicable the operationalization of these constructs. A standard template was created for these tables to ensure even treatment across articles. The completed tables are included in Appendix C.

Relations between these constructs were then identified and coded. Construct relations are important due to their role in the composition of theoretical frameworks. They are used later in this review as a means of integrating operationalizations of sub-constructs into theoretical frameworks that are defined at a higher level of abstraction. Coding them at an early stage is a means of increasing the transparency around the selection of operationalizations from different papers.

The following step involved coding articles for their theoretical framework. Unlike other codes performed in this stage, this code was not used in a decisive way. A set of 5 framework nodes were created, based on the types of theoretical frameworks identified in the initial exploration of the literature prior to consulting experts for their views. Although it would be preferable to identify theoretical frameworks in an entirely bottom-up manner, this seemed impossible in this context. It was observed prior to commencing the review (and confirmed during the review) that there was a high level of inconsistency with use of terminology in this field. Therefore, using author-reported constructs as a basis of identifying theoretical frameworks through emergent clusters of constructs was considered to be too messy a strategy. Instead, we used 4 *a priori* identified nodes to code for author-identified theoretical frameworks. The four framework categories (plus a miscellaneous category) and instructions on how to recognise them, are given in the table below. These categories were not taken as a final word however, but rather as an instrument to organise the literature. In later stages, the coding and the categories themselves, were subject to interrogation and amended.

Code	Refers to	Possible indicators
IPCC	The framework used by the Intergovernmental Panel on Climate Change (IPCC), which views 'vulnerability' as composed of three elements: 'exposure to climate change-induced stress'; 'sensitivity to climate change-induced stress'; and 'adaptive capacity'	- The theoretical framework contains the three elements of 'exposure', 'sensitivity', and 'adaptive capacity'.<br>- The authors report that they build their framework with reference to any publications from the IPCC
VEP	This framework, called 'Vulnerability as Expected Poverty" conceives of 'vulnerability' as the	- The theoretical framework contains the construct 'poverty', 'probability', and 'risk'.<br>- The authors report that they use a framework based on the

	probability that research units will be below a given poverty threshold given certain risk factors associated with climate change.	"Vulnerability as Expected Poverty" approach.<br>- The authors report that they build their framework with reference to any of the following publications:<br>- Chaudhuri, S. 2003. Assessing vulnerability to poverty: concepts, empirical methods and illustrative example http://info.worldbank.org/etools/docs/library/97185/Keny_0304/Ke_0304/vulnerabilityassessment.pdf.<br>- Chaudhuri S., Jalan, J. and Suryahadi, A. (2002) Assessing household vulnerability to poverty from cross-sectional data: a methodology and estimates from Indonesia. Discussion Paper 0102-02, Department of Economics, Columbia University<br>- Christiaensen, L., and Subbarao, K. (2005) Towards an understanding of vulnerability in rural Kenya. *Journal of African Economies*, 14(4), 520-558.
Food Insecurity	This code refers to frameworks where vulnerability is conceived in terms of food security/insecurity. There are usually four subconstructs under food security: 'availability of food'; 'access to food'; 'stability of access'; 'utilization of accessible food'.	- The theoretical framework contains a conception of 'food security' or 'food insecurity', which may be subdivided into four subconstructs similar to: 'availability'; 'access'; 'stability'; 'utilization'.<br>- The authors report that they use a framework based on a "Food security" or "food insecurity" approach.<br>- The authors report that they use a framework which is built on any of the following references:<br>- Løvendal C.R and M. Knowles, 2005. "Tomorrow's hunger: a framework for analyzing vulnerability to food insecurity". FAO-ESA Working Paper No. 05-07. FAO, Agricultural and Development Economics Division, Rome.<br>- FAO (2000) Guidelines for national FIVIMS. Background and principles. www.fao.org/docrep/003/X8346E/X8346E00.HTM
Livelihoods Approach	This code refers to a series of similar frameworks which contain conceptions of 'livelihood capabilities', 'livelihood strategies', and 'livelihood assets'. The later is usually composed of natural, social, financial, physical, and human capital.	- The theoretical framework contains a combination of some of the following constructs: 'livelihood capabilities', 'livelihood strategies', 'livelihood assets', 'natural capital', 'social capital', 'financial capital', 'physical capital', or 'human capital'.<br>- The authors report that they use a framework based on a "Livelihoods" or "Sustainable livelihoods" approach.<br>- The authors report that they use a framework which is built on any of the following references:<br>- Fraser, E.D.G, A. Dougill, K. Hubacek, C. Quinn, J. Sendzimir, and M. Termansen. 2010. Assessing vulnerability to climate change in dryland livelihood systems: conceptual challenges and interdisciplinary solutions. *Ecology and Society*.<br>- Chambers, R., and G. Conway. 1992. Sustainable rural livelihoods: practical concepts for the 21st century. IDS Discussion Paper 296. Institute of Development Studies, Brighton, UK.<br>- Scoones, I. 1998. Sustainable rural livelihoods: a framework for analysis. IDS Working Paper 72. Institute of Development Studies, Brighton, UK<br>- DFID. (1999). *Sustainable Livelihoods Guidance Sheets.* London, UK: Department for International Development.
Other framework	This denotes that a framework is used which doesn't not fall into any of the other frameworks specified.	

Subsequently, graphical representations were created for each theoretical framework in each paper. These were created as indicated in the instructions and had the purpose of serving as easily digestible summaries of the frameworks which were consulted upon throughout the review process. Finally, operationalizations (direct and indirect) were identified, coded and added to the construct tables which are included in Appendix C.

This set of steps was executed on all 35 articles. The article-specific construct tables produced through this can be found in Appendix C. In total, 358 article-specific constructs were identified, of which 281 were defined (in some cases through reference to other works), of which 154 were directly operationalised. A summary table outlining these figures is in Appendix D.

In terms of framework codes, the following table indicates which codes were applied to which articles. From the table it can be seen that 12 articles were coded as IPCC; 7 as 'Vulnerability as Expected Poverty' (VEP); 4 as Food Insecurity; 6 as Livelihoods Approach; and 19 as 'Other Framework'.

It should be noted that one of the articles (Hahn, Riederer, and Foster 2009), presented two distinct frameworks. Therefore two different graphic summaries were produced (Hahn et al A; Hahn et al B), and the coding applied to these two graphics differs.

The fact that 19 were coded using the miscellaneous 'Other Framework' code suggests that the initial categorisation was appropriate for less than half of the articles under review. It should be noted in particular that this coding framework relied on authors' own declarations of frameworks or approaches and as such, the outcome of the coding exercise would suggest the need for a systematic approach to detecting frameworks and approaches in this field. A more structured approach to framework categorisation was done next.

Article	Framework codes
(Antwi-Agyei et al. 2013)	IPCC; Livelihoods Approach
(Baca et al. 2014)	IPCC;
(Berkes and Ross 2013)	Other Framework
(Bogale, Taeb, and Endo 2006)	Other Framework
(Calvo and Dercon 2013)	Vulnerability as Expected Poverty
(Capaldo et al. 2010)	Food insecurity
(CARE 2009)	IPCC;
(Chhihn and Poch 2012)	Vulnerability as Expected Poverty
(Dasgupta and Baschieri 2010)	Other Framework
(Deressa, Hassan, and Ringler 2009)	Vulnerability as Expected Poverty
(Eakin, Winkels, and Sendzimir 2009)	Livelihoods Approach; Other Framework
(Eakin et al. 2012)	Other Framework
(Échevin 2011)	Vulnerability as Expected Poverty; Other Framework
(Ford and Smit 2004)	Other Framework
(Füssel and Klein 2006)	IPCC;
(Gandure, Walker, and Botha 2013)	Other Framework
(Günther and Harttgen 2009)	Vulnerability as Expected Poverty; Other Framework
(Hahn, Riederer, and Foster 2009)	IPCC; Livelihoods Approach; Other Framework
(Ionesco et al. 2009)	IPCC; Other Framework
(Jamir et al. 2013)	IPCC;
(Khan and Salman 2012)	Other Framework
(Luers et al. 2003)	IPCC;
(Marshall 2010)	Other Framework
(Mengistu 2011)	Other Framework
(Misselhorn 2005)	Food insecurity; Livelihoods Approach
(Mubaya et al. 2012)	Other Framework
(Mutsvangwa 2011)	Vulnerability as Expected Poverty; Food insecurity

(Nkondze, Masuku, and Manyatsi 2013)	Other Framework
(Notenbaert et al. 2013)	IPCC;
(Piya, Maharjan, and Joshi 2012)	IPCC; Livelihoods Approach
(Sallu, Twyman, and Stringer 2010)	Livelihoods Approach
(Sarris and Karfakis 2010)	Vulnerability as Expected Poverty
(Sietz, Choque, and Lüdeke 2012)	IPCC; Food insecurity; Other Framework
(Tesso, Emana, and Ketema 2012)	IPCC; Other Framework
(Westerhoff and Smit 2009)	Other Framework

Synthesis of frameworks and constructs

The second stage of analysis involved synthesizing the article-specific, author-reported constructs into a global set of analyst-generated constructs, the refinement of the initial categorization of frameworks, and the generation of a set of ideal-type representations of these frameworks.

Listed below are the first seven steps of this stage, followed by a description of the method.

2.1 Import into NVivo all Images created as exports of graphic summary models created in Stage 1 (Output 1.c). For each graphic summary, identify which nodes under 'Emic Frameworks' have been included as project items in the model, and apply that node(s) to the graphic summary.

2.2 Create a new word document and paste the template of the table 'Emic-Ideal framework map' that is included below. For each node created under 'Emic frameworks', list it in a separate row in the table (output 2.a).

2.3 Create a new node called 'Bridging Frameworks Emic-Ideal'. Retrieve all graphic summaries coded with the 'other framework' node. Compare the graphics to see if any clusters of frameworks can be identified. For each cluster identified, create as a sub-node under 'Bridging Frameworks Emic-Ideal' a node of any name and apply to the relevant graphic summaries. Create a new node under 'Bridging Frameworks Emic-Ideal' called 'Residual' and apply this to all graphic summaries for which a cluster was not identified. In the centre and right column in the Emic-Ideal table, on the row corresponding to 'other frameworks', using 'split cells' subdivide creating a row for each node newly created under 'Bridging Frameworks Emic-Ideal'. Add the name of each node into the rows in the centre column.

2.4 Run a cluster analysis, clustering graphic summaries by nodes under 'Emic frameworks'.

2.5 For each cluster of identified stated frameworks (excluding those coded as 'residual'), beginning with those clusters created from a single framework node, retrieve all graphic summaries in that cluster. Following the principles of constant comparative analysis and cultural domain analysis, and with knowledge from the field, make a subjective judgement as to whether that group of frameworks are 'of a kind' and should constitute an ideal type framework, or whether more subdivision is necessary. Where more subdivision is considered necessary, create and apply an additional set of sub-nodes under "Bridging Frameworks Emic-Ideal' of the form "[e.g. IPCC]-A", "[e.g. IPCC]-B". When no more subdivision is necessary, or if no subdivision is considered necessary to begin with, proceed to the next cluster. When all clusters formed through singular framework nodes have been scrutinised, move on to those clusters formed through combinations of framework nodes. Where clusters formed through combinations of emic frameworks are considered to be of a kind or need subdivision, create nodes under 'Bridging frameworks Emic-Ideal' as appropriate. When all frameworks have been scrutinised, add the names of the new nodes to the appropriate cells in the centre column of the "emic-ideal" table, splitting cells as in the previous step.

2.6 For each category of framework (including residuals), retrieve one graphic summary as a representative example of that framework. Compare across frameworks and make a subjective judgement as to whether each framework is sufficiently distinct to be considered separate frameworks. Where it is considered that two (or more) examples are of the one framework, create a

node under ‘Bridging Frameworks Emic-Ideal’ called ‘merged: [name of framework 1]-[name of framework 2]”. Retrieve all graphic summaries represented by these two (or more) examples and code with this new node. Continue this analysis until all representative examples constitute distinct frameworks. Add the names of any new nodes created to the emic-ideal table as in previous steps.

2.7 For a framework where no subdivision or merging was considered necessary, create a sub-node under “Bridging Frameworks Emic-Ideal” using a name of the form “unchanged-[name of framework]” and apply to all relevant graphic summaries.

These steps involved inspecting the framework clusters that were created in the first stage of analysis. Inspection here had the aim of generating uniform and discreet categories of frameworks. This was done through two steps of inspection. First, within each cluster, the graphic summaries of the article-specific frameworks were compared in order to tell if they were ‘of a kind’. Where clusters were not assessed to be ‘of a kind’, codes were to be applied to enable the cluster to be split into two ‘domains’ (Borgatti 1994). Once *with-in* examination was complete, representative examples of each cluster were compared in order to assess whether clusters were distinct. Where clusters were judged to be similar, codes were to be applied to enable a merger.

In step 2.3, the 19 ‘residual clusters’ that is, clusters each comprising one framework which had been coded as ‘Other framework’, were examined to see if any non-trivial clusters could be detected. Only one cluster could be spotted: an extension of the VEP framework which was used by two articles (Échevin 2011; Günther and Harttgen 2009).

Excluding frameworks coded as ‘other’, there were 7 clusters to be inspected for uniformity (Step 2.5). Three clusters contained only one cluster each and so were deemed uniform by default. Three clusters were judged to be non-trivially uniform. And one cluster was subdivided. These assessments are recorded in the table below:

Table: Summary of *within* inspection of framework clusters

Categories based on framework coding in stage 1 (excluding those coded as other)	Articles	Assessment based on *within* analysis
IPCC	(Baca et al. 2014); (CARE 2009); (Füssel and Klein 2006); (Jamir et al. 2013); (Luers et al. 2003); (Notenbaert et al. 2013)	No subdivision of IPCC
VEP	(Calvo and Dercon 2013); (Chhihn and Poch 2012); (Deressa, Hassan, and Ringler 2009); (Sarris and Karfakis 2010)	No subdivision of VEP.
Food Insecurity	(Capaldo et al. 2010)	No subdivision by default
Livelihoods Approach	(Hahn, Riederer, and Foster 2009) A; (Sallu, Twyman, and Stringer 2010)	Subdivide Livelihoods framework:<br>Livelihoods A - (Hahn, Riederer, and Foster 2009) A<br>Livelihoods B - (Sallu, Twyman, and Stringer 2010)
IPCC & livelihoods Approach	(Antwi-Agyei et al. 2013); (Piya, Maharjan, and Joshi 2012)	No subdivision
Livelihoods Approach and Food Insecurity	(Misselhorn 2005)	No subdivision by default
VEP & Food Insecurity	(Mutsvangwa 2011)	No subdivision by default

The resulting 8 clusters were then brought forward for *across* analysis as per step 2.6, along with the VEP extension cluster and the 17 remaining 'residual clusters'. Of the 26 clusters to be compared, only 4 were non-trivial clusters. Therefore only four representative frameworks needed to be chosen for the comparison, whereas 22 representatives were selected by default. This step resulted in judgments for three mergers to be made. This step is summarized in the table below.

**Table: Record of framework *across* comparisons (step 2.6)**			
**Framework category**	**Includes**	**Representative selected for *across* comparison**	**To be merged?**
Food security	(Capaldo et al. 2010)	default	With VE Food security
Food Security - Livelihoods	(Misselhorn 2005)	default	No
VE Food Security	(Mutsvangwa 2011)	default	With Food security
IPCC	(Baca et al. 2014); (CARE 2009); (Füssel and Klein 2006); (Jamir et al. 2013); (Luers et al. 2003); (Notenbaert et al. 2013)	(Jamir et al. 2013)	Residual 7; livelihoods integrated into IPCC;
livelihoods integrated into IPCC	(Antwi-Agyei et al. 2013); (Piya, Maharjan, and Joshi 2012);	(Piya, Maharjan, and Joshi 2012)	IPCC; Residual 7
Livelihoods A	(Hahn, Riederer, and Foster 2009) A	default	No
Livelihoods B	(Sallu, Twyman, and Stringer 2010)	default	No
VEP	(Calvo and Dercon 2013); (Chhihn and Poch 2012); (Deressa, Hassan, and Ringler 2009); (Sarris and Karfakis 2010)	(Deressa, Hassan, and Ringler 2009)	No
oth-VEP Extensions	(Échevin 2011); (Günther and Harttgen 2009)	(Günther and Harttgen 2009)	No
Residual 1	(Berkes and Ross 2013)	default	No
Residual 2	(Bogale, Taeb, and Endo 2006)	default	No
Residual 3	(Dasgupta and Baschieri 2010)	default	No
Residual 4	(Eakin et al. 2012)	default	No
Residual 5	(Ford and Smit 2004)	default	No
Residual 6	(Gandure, Walker, and Botha 2013)	default	Mengitsu; Mubaya et al.; Westerhoff & Smit
Residual 7	(Hahn, Riederer, and Foster 2009) B	default	IPCC; livelihoods integrated into IPCC
Residual 8	(Ionesco et al. 2009)	default	No
Residual 9	(Khan and Salman 2012)	default	No
Residual 10	(Marshall 2010)	default	No
Residual 11	(Mengistu 2011)	default	Residual 6; Residual 12; Residual 16
Residual 12	(Mubaya et al. 2012)	default	Residual 6; Residual 11; Residual 16
Residual 13	(Nkondze, Masuku, and Manyatsi 2013)	default	No

Residual 14	(Sietz, Choque, and Lüdeke 2012)	default	No
Residual 15	(Tesso, Emana, and Ketema 2012)	default	No
Residual 16	(Westerhoff and Smit 2009)	default	Residual 6; Residual 11; Residual 12
Residual 17	(Eakin, Winkels, and Sendzimir 2009)	default	No

There are three results of note arising from steps 2.1 to 2.7. First is the elimination of 'Livelihoods Approach' and 'Food Insecurity' as meaningful categories. This resulted from the observed trend that it makes less sense to speak of these as theoretical frameworks or research approaches in and of themselves than as substantive topics or concepts that are examined through a given framework. In the case of 'food insecurity', this was more often than not used as an extension to the VEP approach, such that food security was a measure of poverty. A second result of note is the still large number of articles that resist categorization. And thirdly, one further cluster was notices among the residual clusters – that of 'farmer perceptions'. This category was discovered through a repeated examination and comparison of frameworks and would not have been made as the authors do not declare to be following an established approach. Rather, grouping these together as an approach is a product of analysis.

Following the identification of frameworks, the next steps involved the identification of key constructs that make up that framework. The instructions are in the box below:

2.8 Create a new node called 'Key Emic constructs'. For each of the identified frameworks (excluding those coded as 'residual', or frameworks for which only one graphic summary exists), retrieve all graphic summaries coded under the relevant 'Bridging Frameworks Emic-Ideal' node. Identify those constructs that appear to have equivalences across all papers using that framework, create them (unless already present) as sub-nodes under 'Key Emic constructs', and apply them to the graphic summaries. Create a new document and paste the table "Framework defining constructs". List in the appropriate row in the second column those candidate equivalent constructs. For each residual framework or framework category with only one graphic summary, choose six constructs at the highest level of generality and create and apply nodes as above and paste construct names into the "Framework defining constructs" table.

2.9 For each framework category for which there are more than two graphic summaries in that category, consult the theoretical framework section of the relevant articles and identify any additional candidate equivalent constructs that appear in all but one of the relevant papers. Create these constructs (unless already present) as sub-nodes under 'Key Emic constructs', apply them to the graphic summaries, and add them to the third column in the "Framework defining constructs" table, listing in the adjoining cell those articles which fail to include it. For framework categories with more than three articles, identify all candidate equivalent constructs common in all but two[13] of the relevant papers, add to the table with a reference to omitting articles, and then identify constructs in all but three and repeat.

2.10Create a new word document. Paste the tables 'Report of uniform and discreet frameworks' and 'report of uniform and discreet constructs' into the word document. For each distinct and uniform

[13] Those constructs appearing in all but 2 and all but 3 articles are not to be coded. In this step they are merely identified and recorded in the table and maybe be returned to in future stages.

framework identified through the previous steps, fill in details of the name of the framework; a short description of the framework; the main constructs defining the framework[14]; and references to the articles in which they appear. Save this document as “Report of frameworks and constructs” (output 2.b).

2.11 Create a new word document and paste the template of the table ‘Emic-Etic construct map’ that is included below. For each node created under ‘Key Emic constructs’ list it in a separate row in the table (output 2.c).

2.12 Create a new node called “Bridging constructs GS-IT”. Create three sub-nodes called “Unrecognised divergence”, “Duplicate correction” and “Variance – poor definition”. For each construct in the emic-etic table, identify which graphic summaries have been coded with this node and retrieve construct definitions from each of the relevant article-specific construct tables (Output 1.a). Where definitions cannot be retrieved, ignore this unless a divergence is identified. For each construct compare available definitions[15] from each article in which it occurs, and make an informed judgement about whether they constitute the same construct. If not, create two (or more if more than two domains are identified) new sub-nodes under the node “Unrecognised divergence”, naming them “[name of construct] A” and “[name of construct] B” and if there are articles in which the construct is not defined, create a sub-node under ‘Variance – poor definition’ called ‘[name of construct]” and code the graphic summaries appropriately. Add the names of these nodes into the appropriate rows in the middle column of the Emic-Etic table. When satisfied that each construct appearing under the node in question does indeed refer to only one construct, move onto the next construct and repeat for all constructs identified under ‘Key Emic constructs’

2.13 When all constructs have been individually scrutinised for uniformity, compare representative definitions of each construct to ensure that they do indeed refer to distinct constructs. If not, create a new node under “Duplicate Correction” called “merged: [names of constructs]”, and apply this code to the graphic summaries. In the Emic-Etic table, move the relevant Emic constructs so that their rows adjoin, merge the two (or more) cells in the centre column, and enter ‘merged: [names of constructs]’ in the new cell.

2.14 If any constructs have been sub-divided or merged, retrieve the relevant graphic summaries and scrutinise the frameworks for uniformity and discretion following the procedures in 2.4 and 2.5. Update[16] the table ‘Report of uniform and discreet frameworks’.

2.15 Retrieve the document ‘Report of frameworks and constructs”[17]. For each construct listed in the centre column of the ‘Emic-etic map’, list it in the first column of the table ‘Report of uniform and discreet constructs”[18]. Consult the list of article specific tables of emic constructs (Output 1.a) and

[14] Note on implementation: When I encountered this step I chose to defer creating lists of framework-defining constructs until after constructs have been scrutinised. Thus in step 2.14, rather than updating the table, I will be creating the lists for the first time.

[15] Ideally, papers will provide conceptual definitions about what it is in the world that they wish to represent by using the construct. In cases where this ideal is not met, for example when their definitions resemble operational definitions (i.e. what empirical phenomena do they use to represent this construct), the coder shall try on the basis of available construct definitions and if necessary discussions in theoretical frameworks, to answer the question ‘do these two constructs strive to represent the same phenomena’? However, if this is not possible, those constructs that are defined operationally will be treated as undefined constructs in this and the following step.

[16] I didnt create constructs when first creating this table, so rather than update them, I create them from scratch.

[17] To aid the transparancy and structure of the review process, I am adding some columns in which closed-ended responses are sought from the expert coder.

[18] For the ease of readability when handing over to the expert, I will create names for these constructs, rather than using the node-name, which is probably uninterpretable to anybody not closely following the analysis.

retrieve definitions for each appearance of each construct. List definitions and references to source articles in the centre and right columns in the 'report of uniform and discreet constructs' table (Output 2.b).

It would be impractical and not useful to examine and compare all 358 emic constructs in the articles. Instead, a selection of framework-defining constructs was first made. That is, a set of constructs that are common across a given framework. The problem is that, in dealing with emic constructs, it is challenging to transparently identify constructs in different papers which are suspected to be equivalent. This becomes a chicken-and-egg situation. The solution is to make an imperfect selection of suspected equivalent constructs, which are later examined, after which the set of framework-defining constructs are updated.

Step 2.8 and 2.9 are an attempt to select important constructs in a structured by imperfect way. The 'framework-defining table' can be viewed in Appendix E and illustrates which constructs were selected for which frameworks. Where constructs in different papers had the same names they were provisionally presumed to be equivalent. In other cases, constructs of different names were suspected to be equivalent based on factors such as similarity of names, positions in graphic summaries, or similarity of employment.

In step 2.10 a start was made in generating the report of frameworks and constructs, but as stated in the footnote, part of this step was deferred until later – that is, I did not create lists of constructs immediately, because I felt that listing constructs while still dealing with author-reported constructs would add confusion and, moreover, the list of constructs would be subject to change in the following steps.

In Step 2.11, all emic constructs identified in steps 2.8 and 2.9 were listed in a skeleton of a map between author-reported emic constructs and (eventual) analyst-generated etic constructs. In doing so, all constructs with the same name were treated as one. This provided us with a total of 114 emic constructs. The purpose of this map is to allow a record of the move from emic constructs to etic constructs, and a record of how such movement was made. The map was only to be completed at Step 2.18, so it will be returned to the description of the steps that follow.

These 114 constructs (suspected of being important), were then brought forward for scrutiny. As with the scrutiny of framework clusters, scrutiny of constructs was done first *within* a set of constructs of the same name to test for uniformity, and secondly, *across* constructs to test for distinction. Step 2.12 involved *within* analysis.

Analysis was done on the basis of construct definitions. In some cases, in the first stage of analysis where construct definitions were identified, constructs were defined by authors through reference to other works. In this review, we recorded these references. However we adopted as a reliable threshold that we would only follow references in cases where a page number was provided in the reference. As it happened, in no case was this threshold reached. Therefore, in no case did we chase references. Nevertheless, comparison was made on the basis of available information. For example if two articles each had a construct of the same name which they declare is based on the works of the same reference, then it may be concluded that they are the same construct.

This analysis is recorded in the table in Appendix F. 100 of the 114 constructs appeared in only one article, with the implication that they were each uniform by default. Therefore only 14 sets of construct definitions were inspected for uniformity. Of these, 10 were judged to be uniform, and 4 were split. Those which were split were 'Adaptive capacity'; 'Livelihood vulnerability'; 'Sensitivity'; and 'Vulnerability'.

After *within* analysis, *across* analysis was performed. As per the instructions for Step 2.13, a representative definition was selected for each construct (in many cases this selection was made by default). Records of the selection of representatives are in the table in Appendix F. This analysis was done through a cross-tabs comparison in Excel. In total, 26 representative definitions were assessed to be equivalent to one or more others. Out of these 26 definitions, 7 merged constructs were created. Details of these mergers are listed in Appendix G.

Notable among the mergers made was the merger of 'adaptation to long term climate change' and 'Farmer perceptions'. This lent support to the earlier decision to merge the articles in which they appear into one new framework (Farmer Perceptions).

The next step, 2.14, involved selecting from this new set of constructs, constructs to list in the report of frameworks and constructs. The rationale of creating such a report is to create a formalized record of the theoretical frameworks and their key constructs uncovered by this review. Significantly however, it also allows the classification generated by the lead reviewer (Aogán Delaney) to be inspected by the member of the review team with most expertise in the field (Todd Crane).

As a method of selecting constructs for inclusion in the Report, first for each framework all graphic summaries were consulted. The summary with the least amount of constructs was then examined and for each construct the other graphic summaries in the framework cluster were examined to see if the constructs were appearing in all or all bar one of the other frameworks (And for a framework with over five papers, this threshold was lowered to all bar 2 – specifically, the IPCC framework). A list was made of such recurrent constructs and then I moved on the next smallest graphic summary in the framework to see if there were any leftover constructs that appear in all bar the first summary. This method was repeated for each framework. The lists used in this method are included in Appendix H for reference.

The Report of frameworks and constructs was then updated following Steps 2.14 and 2.15.

The next set of steps deals with cross-examining this set of frameworks and constructs by another member of the team, and then moving from emic, author-reported constructs and frameworks to etic analyst-generated constructs and frameworks. The instructions for these steps are in the box below:

2.16 The report finalised in step 2.13 is to be reviewed by a member of the research team with expertise in the field. The expert will first verify the classifications of frameworks and constructs produced in this Stage. Secondly, the expert will examine frameworks and judge which frameworks are relevant for the review, marking it "Retained", and which ones are to be excluded from further analysis, marking them "discarded".

2.17 If any revisions are recommended by the reviewer, the lead researcher is to create and apply appropriate codes and update the report following the procedures already outlined above. The updated report is to be again reviewed by a member of the research team with expertise in the field, and if necessary recoding and updating is to be repeated. When no revisions are recommended, this most recent version of the report is to be saved as a pdf (Output 2.b).

2.18 Create a new node called "Etic constructs". Consult the verified report of constructs. For each construct[19], create a node under "Etic constructs". Code all Graphic Summaries with this new code set. In the "Emic-Etic construct map" fill in the names of the nodes created under "Etic constructs" into the

[19] This step is to be done after the following step. First graphic Summaries are to be coded as retained or discarded. Then Etic construct nodes are only to be created for the constructs appearing in the retained frameworks. However, once created, they are to be applied to all graphics, even those which have been discarded (because irrelevant frameworks might still operationalize relevant constructs).

appropriate cells in the right column (Output 2.c).

2.19 Under the 'Residual' node, under the node 'Bridging Frameworks Emic-Ideal', create two sub-nodes called "retained" and "discarded". For each graphic summary[20], check the report and following the guidance on which frameworks are to be retained or discarded, apply the appropriate nodes to the graphic summaries.

2.20 Create a new node called "Ideal type Frameworks". Consult the verified report of frameworks. For each framework, excluding discarded residuals, create a node under "Ideal type Frameworks". Code all Graphic Summaries with this new code set. In the "Emic-Ideal framework map" fill in the names of the nodes created under "Ideal type Frameworks" into the appropriate cells in the right column (Output 2.a).

2.21 For each ideal type framework, consult the verified report of frameworks and create a new Model with the name of the present ideal type, and using 'add project items' add all defining construct nodes under 'etic constructs'. Arrange these items to graphically represent the framework. Repeat for each ideal type framework. This set of models constitutes output 2.d.

2.22 Create a new model. Using 'add project items', add all etic codes. Arrange to graphically create a theoretical meta-framework, using those constructs found in overlapping ideal types as points of merger. This constitutes output 2.e and Project Output 1.

The Report finalized in Step 2.15 was handed over from Aogán Delaney to Todd Crane. Prior to hand-over, the Report was converted into a questionnaire format in order that the feedback be recorded and structured. The purposes of this exchange was to see if the categorization that was create through a structured review process, if that was meaningful to somebody who was familiar with the domain in which such categorization would be applied. It could be described as a refutational analysis. A second purpose was to steer the further course of the review. Todd was asked to indicate among the frameworks uncovered by the review, which ones were relevant for the purposes of the CCAFS project, and which ones were not.

This inspection made a number of suggestions. In terms of frameworks, 4 frameworks were suggested to be collapsed into one ('Vulnerability as Expected Poverty'; 'Vulnerability as Expected food security'; 'Vulnerability as Expected Poverty – multi-level analysis'; 'Asset vulnerability (Residual)'), while it was also suggested to merge a residual article (Mathematical formalisation of vulnerability) into the IPCC category.

15 (included the 4 to be collapsed) frameworks were considered relevant to the purposes of the review, (IPCC; Vulnerability as Expected Poverty; Vulnerability as Expected Food security; Vulnerability as Expected Poverty – multi-level analysis; Perceptions of climate change; Asset vulnerability; Nested Vulnerability; Current and future vulnerability; Livelihood vulnerability index; Mathematical formalisation of vulnerability; Intensifying vulnerability to food insecurity; Nkondze et al (2013); Patterns of smallholder vulnerability; Livelihood trajectories and resilience and vulnerability; Determinants of Resilience) and 5 were considered not relevant (Community Resilience; Choice of property rights regime; Disaster resilience of rural livelihoods; Regional vulnerability; Social Resilience). The feedback on frameworks in questionnaire form can be found in Appendix I.

Of these suggested changes, the first reviewer accepted all decisions regarding retention or discarding of frameworks for the remainder of the review. As regards the suggestion to merge four frameworks into one, the first reviewer strongly suspected that such a categorization would not survive empirical scrutiny. He asked the expert reviewer to look at this suggestion again and on second inspection he concluded that the 'Asset vulnerability' framework was not compatible with the 'Vulnerability as Expected Poverty' frameworks.

[20] It may be that clusters of frameworks are also considered not relevant.

As regards the suggestion to merge the three econometric frameworks, this new classification was examined and appeared feasible based on the presence of a set of core constructs across all articles. Nevertheless, constructs that were specific to the 'extensions' of this framework were not discarded from further review, even though statistically they now appeared trivial when diluted in a larger category. This was a decision that was taken by the reviewer.

The decision to merge the Mathematical formalisation of vulnerability into the IPCC category was also validated.

In terms of constructs, two mergers were suggested: the constructs that had been previously split into 'Adaptive Capacity A', 'Adaptive Capacity B', and 'Adaptive Capacity C' were recommended to be re-merged. Likewise the constructs that had previously been split into 'Sensitivity A' and 'Sensitivity B' were recommended to be merged again.

9 splits were recommended. These are summarized as follows:

- 'vulnerability' of Ionesco et al (2009)to be split from vulnerability IPCC construct
- The constructs of 'exposure' of Jamir et al's (2013), and Sietz et al's (2012)were not sufficiently well-defined to be verifiably of a kind with general concept of 'exposure'
- The constructs of 'Sensitivity' of Jamir et al (2013), and Notenbaert et al (2013)were not sufficiently well-defined to be verifiably placed in the same category as that of 'Sensitivity (A)'.
- The constructs of 'Adaptive capacity' of Jamir et al (2013), and Notenbaert et al (2013) are not sufficiently well defined to be verifiably placed in the same category as 'Adaptive Capacity (A)'.
- The construct of 'Household vulnerability to poverty' of Günther & Harttgen (2009) was not sufficiently well defined to be verifiably placed in the same category as 'Vulnerability (B)'.
- The constructs of 'poverty line' and 'minimum consumption(income) level' of Calvo & Dercon (2013) and Deressa et al (2009), respectively were not sufficiently well-defined to be verifiably placed in the same category as 'poverty'.
- 'Household level' as defined by Échevin (2011) and by Günther & Harttgen (2009) contained to little information to be placed in the same category as one another.
- 'Community level' as defined by Échevin (2011) and by Günther & Harttgen (2009) contained to little information to be placed in the same category as one another.
- The construct 'Adaptation to long-term climate change' of Gandure et al (2013) contained too little information to be placed verifiably in a category with 'Farmer perceptions' of (Mubaya et al. 2012).

The first reviewer then set about examining the review by the expert. The suggested mergers were validated – the discrepancy between the initial decision to split and the subsequent agreement to merge the split can be explained in terms of *acceptable degree of difference*. The comments of the reviewer who is familiar with the field suggested that the threshold of an acceptable degree of difference used by the first reviewer was smaller than is necessary.

In terms of suggested splits, some were validated and some refuted. Reasons for refuting a suggested split include that poor definitions nevertheless contained references to common publications or where constructs had initial been merged not so much on their similarity to one another, but instead on how different they were from others, and that overlapping they formed something distinct from the others. Closer detail on the validation and refutation of suggested splits is contained in Appendix J.

Due to time pressures, the refutation of the refutation was not handed back among the team. Nevertheless, consensus between the reviewers was reached at the level of frameworks. Therefore potential disagreement can be limited to the level of constructs.

In NVivo, codes were applied with respect to the retention or discarding of frameworks (Step 2.19) and then a node-set of etic constructs was created based on the classification finalized in Step 2.17. This set of etic construct nodes was then filled into the Emic-Etic construct map to complete it. The finalized Emic-Etic construct map can be seen in Appendix K. Similarly, NVivo nodes were created to denote analyst-generated

ideal type research frameworks based on the classification finalized in step 2.17, and the Emic-Ideal Framework map was completed (Step 2.20). This map is shown below:

**Emic-Ideal Framework map**		
Emic stated frameworks	Bridging frameworks Emic-Ideal	Etic/Ideal type Frameworks
Food security	Merged [Food Security][VE Food security][oth-VEP Extensions][unchanged]	Vulnerability as expected poverty, with extensions
	Food Security - Livelihoods	Intensifying vulnerability to food insecurity
IPCC	Merged [IPCC][Livelihoods integrated into IPCC][Residual7][Residual8]	IPCC
Livelihoods approach	Livelihoods A	Livelihood vulnerability index
	Livelihoods B	Livelihood trajectories and resilience and vulnerability
	Merged [IPCC][Livelihoods integrated into IPCC][Residual7][Residual8]	IPCC
	Food Security - Livelihoods	Intensifying vulnerability to food insecurity
VEP	Merged [Food Security][VE Food security][oth-VEP Extensions][unchanged]	Vulnerability as expected poverty, with extensions
Other framework	Merged [Food Security][VE Food security][oth-VEP Extensions][unchanged]	Vulnerability as expected poverty, with extensions
	Residual	Asset vulnerability
		Current and future vulnerability
		Determinants of Resilience
		Livelihood trajectories and resilience and vulnerability
		Nested Vulnerability
		Nkondze et al (2013)
		Patterns of smallholder vulnerability
	Merged [IPCC][Livelihoods integrated into IPCC][Residual7][Residual8]	IPCC
	Merged [Residual6][Residual11] [Residual12][Residual16]	Perceptions of climate change

For each framework then, a graphical model was to be created in NVivo using etic constructs. These steps were originally designed in order to aid understanding and digestion of the frameworks. However, for three principal reasons, they did not work out very well. First, the level of attrition in not converting poorly-defined emic constructs into etic constructs means that for some frameworks, the models contain an incomplete set of constructs. Secondly, because of the structured approach in selecting framework-defining constructs, an arbitrary number of 6 constructs were selected for each residual framework. Because of this arbitrary number, most of these models appear erratic. Third, I did not synthesize a set of analyst-generated construct relations, and so the relations between the constructs, if at all present, is implicit.

Each model is incorporated into the final set of the results, which will be introduced later in this report.

Transparency assessment of operationalized constructs.

The third stage of analysis comprised transparency assessments of operationalized constructs. A transparency instrument was designed based on that developed by Da Silva (2014), with five adaptations. First, Da Silva's instrument was developed to appraise an article as a whole, whereas the present study conducts assessment at the level of the operationalization. Therefore only a subset of the 7 items in Da Silva's framework are used (Data collection methods reported; sampling strategies reported; sample sizes reported; data analysis methods reported). Secondly, an additional criterion is added to ask whether the article reports the operational questions or data collection instruments to represent the construct. Third, a criterion is added which asks whether the construct being operationalized has been defined in the paper, using the codes for construct definitions in the first stage of research (output 1.b). Fourth, while Da Silva lists three values for many criteria (e.g. missing; unclear; clear), here only dichotomies are used (e.g. missing or unclear; clear). One exception to this is the value '2ndary data', where allowances are made for less than full reporting when authors use an existing data source. And finally, an additional value is created for when sufficient information is not reported in the article, but where it is stated that more information is available from the authors. This should be seen as a temporary code: further information should be requested from the authors to complete the review and a final value is to be given when that information arrives. In the execution of this review, authors were not contacted due to the timeframe of the project.

The purpose of this Assessment is as a preliminary stage of screening for validity assessments. The logic at work is that a certain amount of information is necessary in order to be able to assess the validity of an operationalization. In this stage, both the necessary information is extracted from the articles, and a screening out of operationalizations is done for those which are not transparently reported.

This assessment instrument sets a high standard of compliance: For an operationalization to be considered transparently reported it must receive a positive evaluation on four out of six of these criteria. Allowances are made for operationalizations in which sampling sizes and strategies are not reported.

Instructions for this step were specified as follows:

3.1 Consult the Graphic summaries. For each GS which has been coded as 'retained', create a new word file called "structured summaries for transparency assessment – [authors]". Consult the corresponding article-specific table of construct (output 1.a). For each directly operationalized construct, create a table in the new word file using the template below. For those Graphic Summaries which have been coded as 'discarded' but which have been coded with one or more Etic construct nodes, create a new word file called "structured summaries of relevant constructs for transparency assessment – [authors]". For each etic construct, consult the article-specific table of constructs and for each construct through which the etic construct(s) is operationalize, paste the table 'Structured summary of operationalization – transparency assessment' into the word file.

3.2 For each operationalization, consult the third column in the article-specific table of construct (output 1.a). If this text segment contains a statement that further information is available, then create two sub-node called 'construct definition info requested' and 'Inconclusive Operationalization' under the appropriate article-specific construct node, and apply these to the relevant segment of text coded as 'operationalized'. For those with a definition provided, fill in 'yes' in the middle column of the 'construct defined' row in the structured summary, and copy the coded text and paste it into the third column.

3.3 For each operationalized construct, return to the article and search for specification of what data collection methods are used in this operationalization[21]. If there is explicit mention of data collection

[21] where a study uses existing data (in whole or in part), then data collection methods, sample strategies, sample sizes, survey questions for the existing data are unlikely to be reproduced in the report. In such cases I

methods for the operationalization, create a sub-node called 'DCM reported' under the appropriate article-specific construct node and, code that segment of the text, fill in 'yes' in the appropriate cell in the structured summary, copy and paste the relevant text into the adjoining cell, and then proceed to the next operationalization. If data collection methods are either not explicitly or are ambiguously specified, and if there is no indication in the paper that more information is available from the authors then create and apply the sub-nodes 'DCM inadequately reported' and 'NON-transparent Operationalization' to the segment of text coded as 'operationalized', fill in 'no' in the appropriate cell in the structured summary, include a rationale in the adjoining cell, and fill in 'NOT Transparent' in the final cell in the table. Exclude this operationalization from further steps in the transparency assessment tool. If data collection methods are either not explicitly or are ambiguously specified, but the article states more information is available from the authors then create and apply the sub-nodes 'DCM info requested' and 'Inconclusive Operationalization' (if this conclusion code has not already been applied) to the segment of text coded as 'operationalized'.

3.4 For each operationalized construct, return to the article and search for specification of indicators and/or questions used in the data collection instrument[22]. If there is explicit mention of at least one indicator or question for the operationalization, create a sub-node called 'OpQ-I reported' under the appropriate article-specific construct node and, code that segment of the text, fill in 'yes' in the appropriate cell in the structured summary, copy and paste the relevant text into the adjoining cell, and then proceed to the next operationalization. If no indicators or questions are specified, and if there is no indication in the paper that more information is available from the authors then create and apply the sub-nodes 'OpQ/I inadequately reported' and 'NON-transparent Operationalization' to the segment of text coded as 'operationalized', fill in 'no' in the appropriate cell in the structured summary, include a rationale in the adjoining cell, and fill in 'NOT Transparent' in the final cell in the table. Exclude this operationalization from further steps in the transparency assessment tool. If no indicators or questions are specified, but the article states more information is available from the authors then create and apply the sub-nodes 'OpQ/I info requested' and 'Inconclusive Operationalization' (if this conclusion code has not already been applied) to the segment of text coded as 'operationalized', and create the annotation: "request info: OpQ/I: [name of construct]".

3.5 For each operationalized construct, return to the article and search for specification of sampling strategies used to select the research units on which data is collected[23] for this operationalization[24]. If there is explicit mention of sampling strategies for the operationalization, create a sub-node called 'Sampling Strategies reported' under the appropriate article-specific construct node and code that segment of the text, fill in 'yes' in the appropriate cell in the structured summary, copy and paste the relevant text into the adjoining cell, and then proceed to the next operationalization. If there is no discussion of sampling strategies, and if there is no indication in the paper that more information is available from the authors then create and apply the sub-nodes 'Sampling strategies inadequately reported' and 'NON-transparent Operationalization' to the segment of text coded as 'operationalized', fill in 'no' in the appropriate cell in the structured summary, include a rationale in the adjoining cell, and fill in 'NOT Transparent' in the final cell in the table. Exclude this operationalization from further steps in the transparency assessment tool. If sampling strategies are not discussed in the article, but the article states that more information is available from the authors then create and apply the sub-

take it that if a paper refers to an existing data source, then it can be assumed for the purposes of coding that data collection methods, operational questions, sample strategies, and sample sizes have been reported. At a later stage, the adequacy of such sources of data will be assessed (by an expert). However, it is still necessary that papers report how they analyzed such data.

[22] For constructs operationalized through existing data, see footnote in step 3.3.

[23] In some cases, an article presents a methodology as the *outcome* of the paper, rather than as a means of research. In such cases, it is unlikely that sample strategies or sample sizes are stipulated. Therefore, for such papers, it is admissible that sample strategies and sizes are not reported.

[24] For constructs operationalized through existing data, see footnote in step 3.3.

nodes ‘Sampling strategies info requested’ and ‘Inconclusive Operationalization’ (if this conclusion code has not already been applied) to the segment of text coded as ‘operationalized’.

3.6 For each operationalized construct, return to the article and search for specification of sample sizes[25] of the research units on which data is collected[26] for this operationalization. If there is explicit mention of sample size for the operationalization, create a sub-node called ‘sample-size reported’ under the appropriate article-specific construct node and code that segment of the text, fill in ‘yes’ in the appropriate cell in the structured summary, copy and paste the relevant text into the adjoining cell, and then proceed to the next operationalization. If sample sizes are either not explicitly or are ambiguously stated, and if there is no indication in the paper that more information is available from the authors then create and apply the sub-nodes ‘Sample size inadequately reported’ and ‘NON-transparent Operationalization’ to the segment of text coded as ‘operationalized’, fill in ‘no’ in the appropriate cell in the structured summary, include a rationale in the adjoining cell, and fill in ‘NOT Transparent’ in the final cell in the table. Exclude this operationalization from further steps in the transparency assessment tool. If are not discussed in the article, but the article states more information is available from the authors then create and apply the sub-nodes ‘Sample size info requested’ and ‘Inconclusive Operationalization’ (if this conclusion code has not already been applied) to the segment of text coded as ‘operationalized’.

3.7 For each operationalized construct, return to the article and search for specification of what data analysis methods are used in this operationalization. If there is explicit mention of data analysis methods for the operationalization, create a sub-node called ‘DAM reported’ under the appropriate article-specific construct node and code that segment of the text, fill in ‘yes’ in the appropriate cell in the structured summary, copy and paste the relevant text into the adjoining cell, and then proceed to the next operationalization. If data analysis methods are either not explicitly or are ambiguously specified, and if there is no indication in the paper that more information is available from the authors then create and apply the sub-nodes ‘DAM inadequately reported’ and ‘NON-transparent Operationalization’ to the segment of text coded as ‘operationalized’ , fill in ‘no’ in the appropriate cell in the structured summary, include a rationale in the adjoining cell, and fill in ‘NOT Transparent’ in the final cell in the table. Exclude this operationalization from further steps in the transparency assessment tool. If data analysis methods are either not explicitly or are ambiguously specified, but the article states more information is available from the authors then create and apply the sub-nodes ‘DAM info requested’ and ‘Inconclusive Operationalization’ (if this conclusion code has not already been applied) to the segment of text coded as ‘operationalized’.

3.8 Consult the article-specific document of structured summaries and consult the node structure for constructs appearing in that article. For each operationalization that has not been labeled as “NOT Transparent” in the final cell of its table, check its node structure to see if it has been coded with ‘Inconclusive Operationalization’. Create a new word document called “Inconclusive operationalizations – authors to contact”. Assemble into this document a list of inconclusive operationalizations appearing in an article, and the incomplete structured summary tables. Authors will not be contacted in this project, but this document forms an important reference point for any follow-up study (output 3.a).

3.9 Create a new parent-level node called ‘Transparent Operationalization’. Consult the article-specific document of structured summaries For each operationalization that has been assessed positively for each of the six criteria, fill in ‘yes’ in the final cell of the structured summary, apply the node ‘Transparent Operationalization’ to the text coded around ‘operationalization’. At the top of the article-specific document of structured summaries create a list of transparently operationalized constructs (output 3.b).

3.10Repeat Steps 3.1 – 3.9 for each article whose graphic summary was coded as ‘retained’.

[25] For constructs operationalized through existing data, see footnote in step 3.3.

[26] For articles which present rather than implement a methodology, se footnote in step 3.5.

3.11 For each article whose framework was coded as ‘discard’, check each coded construct in the record of comparisons of emic constructs (step 2.13) and see if it has been merged with any constructs appearing in a retained framework. For each relevant construct, repeat steps 3.1-3.9.

The template used to create structured summaries is as follows:

**Structured summary of operationalization – transparency assessment**		
**Construct:** [name]		
**Article:**		
Criterion	Assessment	Quoted text or Rationale for negative assessment
Construct defined?	Yes/no	
Data collection methods reported?	Yes/no	
Reporting of indicators/questions used to operationalize construct?	Yes/no	
Sampling strategies reported?	Yes/no	
Sampling sizes reported?	Yes/no	
Data analysis methods reported?	Yes/no	
Conclusion		
Transparency Conclusion:	Yes/no	

This Stage was executed as per the instructions with the exception that authors were not contacted.

As with comparison of constructs, where definitions were partial but contained references to other works, these were assessed to be defined.

This assessment was carried out on 147 defined directly operationalized article-specific constructs. Of these, 113 were assessed to be transparent, and 27 were assessed as not transparent, while 7 were considered partially transparent or were inconclusive until authors are contacted.

Operationalizations that were assessed as transparent were brought forward to the next stage for validity assessment. Structured summaries of those operationalizations which were not assessed to be transparent are included in Appendix L.

Validity and feasibility assessment of operationalized constructs

Da Silva's Transparency instrument was itself constructed around the needs of the quality assessment checklist of Kampen and Tamás (2014). However, as noted by Da Silva, it is not practical to apply the quality assessment checklist unless you have substantial knowledge of the field of research in question (2014). Therefore this Stage of analysis was carried out solely by the team member with the most knowledge of the field (Todd Crane).

A validity assessment was conducted on the basis of the data collected in the transparency assessment. Two criteria for validity were used:

1. The data collection methods correspond to the epistemological type of data required to represent the construct as defined.
2. The data collection methods, instruments, and analysis methods provide a complete and valid understanding of the construct defined.

To be considered valid, both criteria had to be satisfied.

In addition to validity, an assessment of feasibility was also conducted. This involved a subjective judgment about whether the operationalization of each construct was feasible to be executed within the CCAFS program.

This validity assessment instrument was executed on the transparently operationalized, directly operationalized, defined constructs[27]. The complete assessment questionnaire is contained in Appendix M.

[27] Through mechanical error, three of the 147 transparent operationalizations were omitted from the validity assessment.

Integration of candidate operationalizations into ideal-type frameworks

The final stage of analysis involves integrating those article-specific operationalizations into the ideal-type research frameworks. This stage comprised two principal operationalizations. First, the etic constructs in the ideal-type frameworks were to be matched to article-specific operationalizations. Secondly, where more than one candidate operationalization was found for a given etic construct, a selection among them was to be made.

Due to deadlines in the timeframe of the project, selection among candidates was not completed prior to the handover of deliverables and the writing of this report. Therefore, the output was structured in such a way that first-choice candidate can be inserted once selected.

Instructions for the matching of etic constructs to article-specific operationalizations are the box below:

5.1 Create a new word document called ‘Questionnaire – candidate operationalizations’.

5.2 For each retained framework cluster, retrieve the article-specific construct tables (output 1.a) of the relevant articles, and for each of the constructs listed in the ‘main constructs’ cell in the report of frameworks and constructs (excluding constructs for which all immediate sub-constructs in the ‘operationalized through’ cell also appear in the ‘main constructs’ cell and where each of the sub-constructs appears in more than one paper; excluding also constructs which appear as sub-constructs of a higher-order construct but where the sub-constructs appear in only one paper), create a new section and heading in the ‘questionnaire – candidate operationalizations’ document, and paste the ‘Selection of most useful operationalizations’ table. Repeat for each retained framework cluster. For residual frameworks, choose those constructs at the highest levels of operational chains, as represented in the ‘operationalized through’ cells, ensuring that all directly operationalized constructs are represented by some chosen construct. Create sections for these constructs in the ‘questionnaire – candidate operationalizations’ document.

5.3 For each section in the ‘questionnaire – candidate operationalizations’ document, open the relevant etic construct node in NVivo to see all articles in which that construct is (indirectly or directly) operationalized. Copy the table ‘structured summary of candidate operationalizations’ and in the current section of the document paste a table for each article in which the present construct is operationalized. Fill in the cells ‘candidate article’ and ‘construct operationalized’. Repeat for each section.

5.4 For each candidate operationalization, retrieve from the article-specific construct tables, a list of all constructs through which the main construct is indirectly operationalized (if any), placing them either in the ‘Intermediate constructs’ or the ‘Directly operationalized constructs’ cells (if the construct is directly operationalized, leave these cells blank). If for a given candidate operationalization, an intermediate or direct construct also appears in a section of the questionnaire itself as a candidate operationalization, include ‘SEE CANDIDATE SECTION’ next to that construct and exempt it from any further analysis as an intermediate or direct operationalization.

5.5 For each candidate operationalization, consult the article in NVivo, opening either the relevant ‘relationship nodes’ or the nodes under ‘construct relations’. Paste the coded text into the cell adjoining ‘conceptual framework’. If the candidate operationalization consists of one directly operationalized construct, write ‘DIRECT OPERATIONALIZATION’ in this cell.

5.6 For each directly operationalized construct for a given candidate operationalization, insert it in the rows below the cell ‘operationalization of sub-constructs’. Consult the completed validity questionnaire. For operationalizations which have been negatively assessed on any of the validity or feasibility questions, insert ‘not valid/feasible’ in the appropriate Data collection cells and exclude from further analysis. For constructs which are not present in the questionnaire, fill in ‘not transparent/operationalized’ in the appropriate cells’. For those that have been positively assessed, copy the data in the ‘Data collection methods reported?’ and ‘Reporting of indicators/questions used to operationalize construct?’ cells from

the validity questionnaire, and paste them into the corresponding 'data collection' and 'operational questions' cells in the candidate table.

5.7 For each candidate operationalization, retrieve from the article text describing the methods of analysis used to formulate findings at the level of the candidate construct. In NVivo, create a new sub-node called 'analysis of sub-constructs' under the relevant article-specific construct node, and apply to this segment of text. Copy this text and paste into the cell adjoining 'Candidate-level Analysis' in the candidate table. If no such description of analyses can be retrieved, fill in 'not reported' in the cell adjoining 'Candidate-level Analysis'

5.8 When tables for all candidate operationalizations for all relevant constructs in all frameworks are completed, make a copy of the document, calling it 'candidate operationalizations – defaults retained'. In the original document, paste onto the first page of the template of instructions on how to complete the questionnaire, and delete each section for which only one candidate operationalization is offered. Hand the questionnaire document to a team member with expertise in the field. For each construct section, the expert is to select from among the candidate operationalizations three ordered preferences in terms of operationalizations that are useful for the purposes of the project. This selection is to be filled in in the 'selection of most useful' tables at the beginning of each section.

Step 5.2 outlines a complicated but structured process for the selection of constructs to be included as candidate operationalizations. This is based on using the set of etic constructs contained in the Report of Frameworks and constructs (See Appendix I) for each framework, and using the etic constructs as a guide, in each article in which the etic constructs have a corresponding emic construct, following the chain of operationalization as recorded in the article-specific construct tables (See Appendix C). The remainders of the steps 5.3 to 5.7 involve structured processes for gathering existing information necessary to have a full description of indirect operationalizations of a high-order construct. These steps were executed as instructed.

When all necessary data was gathered into the structured form, the next step (5.8) involved orientating this document into a structured questionnaire format to allow a selection to be made where there exists more than one candidate operationalization for a given etic construct. The questionnaire was to provide a transparent record of where selection between candidates was made for the purposes of providing the CCAFS program with those operationalizations most useful to their purposes for which they commissioned this review.

As mentioned previously, the timeframe of the project did not allow the questionnaire to be completed by the vulnerability expert in the team prior to the next steps, as had originally been intended. Therefore the lead reviewer continued the review process marking clearly any segment where the results of a selection were to be inserted.

Apart from candidate operationalizations, it then remained to select operationalizations for all etic constructs used in all retained frameworks. The instructions for doing so are in the box below:

5.9 Create a new word document called 'report of selected operationalizations of retained frameworks'. In this document, create a section for each retained framework.

5.10 Within each section, from the 'main constructs' cell in the report of uniform and discreet frameworks, create a list comprised of the smallest possible number of constructs, which themselves are directly or indirectly operationalized in at least one article in which they appear, as evidenced in the article-specific construct tables, such that all constructs within the 'main constructs' cell can be said to be represented on the list either directly or by a higher order construct as denoted in the articles using this framework, specifically in the annotations of text coded by the node 'vertically composed' or as specified by

relationship nodes. Copy the table ‘record of selection of constructs’ and paste below the list of constructs. Insert each construct from the ‘main constructs’ cell into a new row in the left column of the table. In the center column insert the name of the construct in the newly created list which represents the construct in the adjoining cell. In the right column, paste the coded text which specifies a vertical relationship between the constructs in the left and center columns for that row. For constructs which are directly represented on the list, write ‘directly represented’ in the right-most column.

5.11 For each construct on the list in each section, copy and paste the table “operationalization of constructs”.

5.12 For operationalizations which appeared in the *questionnaire*, retrieve that which was selected as the best example by the expert. Fill in ‘expert selection’ in the cell adjoining ‘selected by’, and give a justification given by the expert for the selection. Retrieve information from equivalent cells in the questionnaire. In addition, retrieve information on sampling strategies, sample sizes, and data analysis methods from the validity assessment report. If sampling strategies and sample sizes have been reported, and if data collection methods for a given sub-construct differ from those of the overall operationalization, paste this information into the appropriate cells. Otherwise paste ‘not reported’, ‘not reported’, or ‘see candidate level analysis’, respectively.

5.13 For constructs which do not appear in the Questionnaire, that is those operationalizations for which only one valid operationalization can be found in the literature, fill in the tables using the methods outlined in steps 5.3 – 5.7 and in 5.11. This report of selected operationalizations of retained frameworks’ constitutes output 5.a

Step 5.10 is comprised of a structured method to select the highest order constructs for each framework such that all key constructs for that framework are represented in the operationalization of those selected frameworks. Steps 5.11 to 5.13 consist of instructions for the gathering of the required existing data for the operationalizations, either through copying and pasting from the *Questionnaire* (in such cases marking clearly where candidate operationalizations are to be inserted once selected), or through repeating the process for those constructs for which only one (or none) examples exist. The results of this, including a record of the selection among etic constructs, can be seen in Appendix N.

Conversion to deliverables

A final stage of analysis involved structuring the resulting synthesis as a deliverable output. Instructions for this operation are in the box below:

6.1 For each framework, create a word document. Paste the 'framework summary' table into each document.
6.2 From the report of constructs and frameworks (output 2.b) retrieve information on the name of the framework, description of that framework, key constructs used in that frameworks, the definitions of those key constructs, and the articles using that framework. Paste this information into the appropriate cells in the table.
6.3 Copy the model of the ideal type framework (output 2.d) and paste it into the cell under 'Ideal type model'
6.4 For each key construct retrieve from the report of selected operationalizations document (output 5.a) the corresponding 'operationalization of constructs' table, and paste these tables into the operationalization of key constructs cell.
6.5 For each framework, paste the table 'Information relating to further development of framework'.
6.6 Under the cell 'Constructs with no adequate operationalizations' list all key constructs in that framework for which no adequate operationalizations could be found in the subject literature.
6.7 For each construct listed in the Questionnaire, select the 2nd and 3rd choice preferences as selected by the expert, copy and for each framework in which that construct is used, paste into the cells below 'Summary of operationalization' in the 'information relating to further development of framework' table. Insert details of the construct name and its preference rank in the corresponding cells.

These instructions were executed as specified with the following changes: First, because of problems with the creation of models (see stage 2 of analysis), for some framework there was no model to insert (Step 6.3). For others, the model that was inserted was considered to be uneven. Therefore explanatory comments were included in these cells.

Second, because selection among candidate operationalizatoins had not yet been made, instructions where included on where to insert such selections one made (6.4, 6.7).

The final set of results created here can be seen in Appendix O. Additionally, the Questionnaire on candidate operationalizations, which was also handed as a deliverable, can be seen in Appendix P.

Appendix A: Results of relevance and quality screening in First Review

Paper #	Title	Lead Author	Publication Date	Quality review	Comments
Paper 1	Characterizing the nature of household vulnerability to climate variability: empirical evidence from two regions of Ghana	Antwi-Agyei	2012	Yes	Application of the sustainable livelihoods framework to direct the approach. Excellent mix of methodologies and analysis to derive final causation and determinants.
Paper 2	Assessment of climate change vulnerabilities in Kangpara Gewog, Trashigang	UNDP	2012	No	Descriptive methodology on a single project. No analytical methods to determine factors contributing toward vulnerability in the site.
Paper 3	Climate change vulnerability assessments in Miombo Woodlands. WWF.	Shumba	2012	No	Descriptive methodology, no determinants or causation laid out.
Paper 4	Assessing vulnerability of selected farming communities in the Philippines based on a behavioral model to agent's adaptation to global environmental change.	Acosta-Michlik	2008	Yes	Cited accompanying paper to justify some of the methodological approaches.
Paper 5	Assessing household vulnerability to climate change. The case of farmers in the Nile Basin of Ethiopia	Deressa	2009	Yes	Statistical analysis of agro-ecological zones and income levels as key factors determining vulnerability.
Paper 6	A Cross-Sectional, Randomized Cluster Sample Survey of Household Vulnerability to Extreme Heat among Slum Dwellers in Ahmedabad, India	Tran	2013	No	Good use of statistical regression and correlation, but outcomes were focused on heat related morbidity and effect of heat, rather than heat as one contributor to household vulnerability.
Paper 7	A method for quantifying vulnerability, applied to the agricultural system of the Yaqui Valley, Mexico	Luers	2003	Yes	Range of methods including statistical regression and spatial analysis. The paper provided a framework for assessing the relative importance of market fluctuations compared to temperature changes in determining vulnerability. Wheat yield was the outcome variable of concern in delineating vulnerability.
Paper 8	A Simple Human Vulnerability Index to Climate Change Hazards for Pakistan	Khan	2012	Yes	Range of methods and statistical approaches utilized. Outcomes showed significant factors at district level vulnerability. Robust regression to test the causation elements the authors identified.

Paper 9	Derivation of a household-level vulnerability index for empirically testing measures of adaptive capacity and vulnerability	Notenbaert	2013	Yes	Regression and correlation analysis of determinants that were used in the household vulnerability index. Good use of literature to explain the causal relationships illustrated by the statistically significant variables.
Paper 10	Who is susceptible and why? An agent-based approach to assessing vulnerability to drought	Kromker	2008	No	Range of modeling and index development, but approach was focused on susceptibility to drought and psychological response. Outcomes for India case study (fits criteria of geographic scope of systematic review) are descriptive rather than outlining key causes of vulnerability.
Paper 11	Climate vulnerability index - measure of climate change vulnerability to communities: a case of rural Lower Himalaya, India	Pandey	2012	No	Description of Composite Vulnerability Index and components between households near to the administrative headquarters and those far. Statistics carried out, but description of significant correlates and invalidated assumptions.
Paper 12	Climate variability and farmer's vulnerability in a flood-prone district of Assam	Chaliha	2011	No	Composite Vulnerability Index was derived and taken to be representative of the agricultural vulnerability of the farmers of the district with respect to floods. Indices calculated were apportioned weights according to the ranks assigned to the sources of vulnerability. This was done by the farmers based on their perceptions during the Participatory Rural Appraisal. Outcome was a weighted biophysical, agricultural, socio-economic vulnerability indices of study villages. No correlation of causation of specific indicators.
Paper 13	Climate variability and change or multiple stressors? Farmer perceptions regarding threats to livelihoods in Zimbabwe and Zambia	Mubaya	2012	Yes	Descriptive statistics and participant ranking of stressors linked to climate variability. Points allocated by participants to each stressor under a specific criterion.
Paper 14	Climate Change Impacts on Agriculture and Vulnerability as Expected Poverty of Kampong Speu Province, Cambodia	Chhinh	2012	Yes	The study aimed to identify the impact of environmental shocks (flash floods, windstorms and drought) and household characteristics on per capital income. Vulnerability indexes to predict future poverty incidence in the communities were produced.
Paper 15	Vulnerability to Weather Disasters: the Choice of Coping Strategies in Rural Uganda	Helgeson	2013	No	Focus was on analysis of coping strategies rather than vulnerability determinants.
Paper 16	Multi-Agent Modelling of Climate Outlooks and Food Security on a Community Garden Scheme in Limpopo,	Bharwani	2005	No	Investigated the effect of a climate scenario and resulting market effects, did not illustrate additional vulnerability factors

	South Africa				
Paper 17	Adaptation to climate change and variability: farmer responses to intra-seasonal precipitation trends in South Africa	Thomas	2007	No	The study analyzed and coded qualitative data for risk factors but focus was made on adaptation and coping rather than vulnerability.
Paper 18	Adapting agriculture to climate change in Kenya: Household strategies and determinants	Bryan	2013	No	Assessed determinants of adaptation versus vulnerability.
Paper 19	Analysis of vulnerability and resilience to climate change induced shocks in North Shewa, Ethiopia	Tesso	2012	Yes	Principal component analysis used to outline vulnerability factors, with relation to agro-ecological zones.
Paper 20	Application of Fuzzy Cognitive Mapping in Livelihood Vulnerability Analysis	Murungweni	2011	No	Used three scenarios to construct fuzzy cognitive maps for livelihood analysis. Results show qualitative patterns where different vulnerability factors emerge.
Paper 21	Can farmers' adaptation to climate change be explained by socio-economic household-level variables?	Below	2012	No	Multi-linear regression model to look at factors. Focused was placed adaptation interventions
Paper 22	Community Vulnerability to Floods and Landslides in Nepal	Samir	2013	No	Assessed the relative importance of socioeconomic factors associated with differential community vulnerability to floods and landslides in Nepal. Results from regression were used by authors to describe patterns and assumptions of vulnerability
Paper 23	Effects of Landscape Segregation on Livelihood Vulnerability: Moving From Extensive Shifting Cultivation to Rotational Agriculture and Natural Forests in Northern Laos	Castella	2013	No	Developed an analytical framework for assessing the Impact of Landscape Segregation on Ecosystem Service Provision and Livelihood Vulnerability. No vulnerability determinants identified and descriptions used.
Paper 24	Food insecurity and vulnerability in Nepal: profiles of seven vulnerable groups.	Lovendal	2004	No	Workshops at national and sub-national level and focus group discussions at community scale. Descriptions of vulnerability made largely from summaries of national workshops.
Paper 25	Farmers' perception and knowledge of climate change and their coping strategies to the related hazards: Case study from Adiha, central Tigray, Ethiopia	Mengistu	2011	Yes	Hazard identification and characterization from the results of focus group discussions. Hazards were ranked by gender.
Paper 26	Farmers' perceptions of adaptation to climatechange and water stress in a South African rural community	Gandure	2013	Yes	Focus group discussions with farmers ranking factors causing changes to their livelihood including climate variability and change. Findings show age disaggregation important in ranking of hazard (e.g.

					unemployment vs. climate change for youth).
Paper 27	Farmers' vulnerability to climate variability in Dimapur district of Nagaland, India	Jamir	2013	Yes	Weights were assigned to the different indicators for obtaining the composite vulnerability index. Normalization of the values for each of the indicators was carried out. IPPC framework used to group indicators under the heads: demographic, biophysical, agricultural and socio-economic sources of vulnerability
Paper 28	Household vulnerability to climate change: Examining perceptions of households of flood risks in Georgetown and Paramaribo	Linnekamp	2011	No	Assessed direct impact of floods on households and where households took preventative action.
Paper 29	Insights into the composition of household vulnerability from multicriteria decision analysis	Eakin	2008	Yes	Development of indices based on survey data structured on livelihood capitals framework. Analytical hierarchy process applied for determining criteria weights. This was followed by compromise programming to rank households in terms of sensitivity and adaptive capacity. Fuzzy classification of households into vulnerability categories.
Paper 30	Institutional Change, Climate Risk, and Rural Vulnerability: Cases from Central Mexico	Eakin	2005	No	Livelihoods approach to explore vulnerability across three communities. Focus was on household risk management strategies.
Paper 31	Land ownership and conflicts over the use of resources: Implication for household vulnerability in eastern Ethiopia	Bogale	2006	No	Study attempts to investigate factors associated with the choice of various property right institutional arrangements for sustainable use of the land resource. Regression analysis focused on land right and property regimes.
Paper 32	Livelihood Security, Vulnerability and Resilience: A Historical Analysis of Chibuene, Southern Mozambique	Ekblom	2012	No	Historical account and more focus on ways to reduce vulnerability
Paper 33	What drives food insecurity in southern Africa? A meta-analysis of household economy studies	Misselhorn	2005	Yes	Meta-analysis of local level Household Economy Approach (HEA), citation counts of direct and indirect drivers of food insecurity as component focus of vulnerability
Paper 34	Vulnerability to individual and aggregate poverty	Calvo	2012	Yes	Axiomatic approach to the measurement of both individual and aggregate vulnerability. Constructed a vulnerability profile, based on (multivariate) correlations of household vulnerability with a set of basic characteristics, such as demographics, assets, and other general household- and village-level characteristics

Paper 35	Vulnerability to climate change in rural Ghana: mainstreaming climate change in poverty-reduction strategies	Dasgupta	2010	Yes	Constructed an index of vulnerability to climate change, at the household level. The regional risk of drought using average annual rainfall data
Paper 36	Vulnerability of smallholder rural households to food insecurity in Eastern Ethiopia	Bogale	2012	Yes	Study adapted the Vulnerability as Expected Poverty (VEP) approach to food insecurity. The study scrutinizes factors that are associated with household level vulnerability to food insecurity by adapting VEP approach.
Paper 37	A model of vulnerability to food insecurity	Capaldo	2003	Yes	Developed a forward-looking model, which identifies the risks that households are exposed to while also estimating the magnitude of the impact of these risks on household food security. The model allows the relative vulnerability to food security given each typology of households to be estimated. Vulnerability factors and correlation were identified.
Paper 38	Estimating Households Vulnerability to Idiosyncratic and Covariate Shocks: A Novel Method Applied in Madagascar	Gunther	2008	Yes	The study analyses whether vulnerability is mainly driven by permanent low consumption prospects i.e. structural or poverty-induced vulnerability or by high consumption volatility i.e. transitory or risk-induced vulnerability. The study shows covariate shocks have higher impacts on rural households.
Paper 39	Dynamics of Chronic Poverty: Variations in Factors Influencing Entry and Exit of Chronic Poor	Dhamija	2008	Yes	Used panel data from three-year blocks to assess the emergence of poverty. A regression analysis showed household size and composition, and caste to be significant in affecting poverty
Paper 40	Characterizing poverty and vulnerability in rural Haiti: a multilevel decomposition approach	Echevin	2011	Yes	Two level modeling and regression analysis of the impact of both observable and unobservable idiosyncratic and covariate shocks on household economic well being. Findings related to climate shocks and interaction with income.
Paper 41	Growth and shocks: evidence from rural Ethiopia	Dercon	2004	No	Econometric approach to test for the impact of uninsured risk. Study measured recent and past shocks which were directly introduced in regressions, and their cumulative impact quantified. (In some regressions shocks had no explicit role to play in the formulation).
Paper 42	Measuring vulnerability to poverty	Kamanou	2002	No	Capture the idea of vulnerability by starting with micro-economic theory of risk & uncertainty. The study took the changes in per capita income and consumption to signal 'shocks' like price changes or low rainfall. The idea was to generate a distribution of possible future outcomes for households based on observed characteristics. Focus was on framework development and

					methodology, no determinants clearly outlined.
Paper 43	Modelling the economic vulnerability of households in the Phang-Nga Province (Thailand) to natural disasters	Willroth	2011	No	Aimed to assess economic vulnerability of households using a questionnaire based survey and remote sensing. This was integrated into a structural equation model (SEM). Focus of analysis was vulnerability to the Tsunami and not at additional determinants of vulnerability.
Paper 44	The impact of conflict on household vulnerability to climate stress: evidence from Turkana and Kitui Districts in Kenya	Eriksen	2005	No	Investigated the impact of conflict and violence on household vulnerability to climate stress. Descriptive analysis of interview outcomes and focus was on adaptation needs.
Paper 45	The rains are disappointing us: dynamic vulnerability and adaptation to multiple sytressors in the Afram Plains, Ghana	Westerho ff	2009	Yes	Application of a generic vulnerability framework to understand community relevant exposure sensitivities. Explored four key vulnerability determinants as outcome of exposure-sensitivity analysis.
Paper 46	Typical patterns of smallholder vulnerability to weather extremes with regard to food security in the Peruvian Altiplano	Sietz	2012	Yes	Pattern analysis where vulnerability-creating mechanisms based on similarities at household level were compared. The cluster analysis examined vulnerability profiles when exposed to weather extremes, with a focus on the food security aspects of vulnerability. The cluster analysis revealed four vulnerability patterns that depict typical combinations of household attributes, including their harvest failure risk, agricultural resources, education level and non-agricultural income.
Paper 47	Factors Affecting Households Vulnerability to Climate Change in Swaziland: A Case of Mpolonjeni Area Development Programme(ADP)	Nkondze	2013	Yes	Developed a household vulnerability index based on survey results
Paper 48	Resilient or Vulnerable Livelihoods? Assessing Livelihood Dynamics and Trajectories in Rural Botswana	Sallu	2010	Yes	Quantified the impact of different livelihood trajectories. The focus was more on resilience factors, however cluster analysis split the households into varying vulnerability levels.

Paper 49	Vulnerability and poverty in Bangladesh	Azam	2009	No	The study estimates the ex ante welfare of households. Estimates were made of both the expected mean and as well as variability of consumption, with the later being determined by idiosyncratic and covariate shocks. Focus places on idiosyncratic shocks and regression didn't pinpoint specific determinants.
Paper 50	Vulnerability to Covariate and Idiosyncratic Shocks and Safety Net Targeting of Rural Households with an Application to Rural Tanzania	Sarris	2010	Yes	Household surveys, secondary data and the estimation of crop income variability were collected. In addition time series data on market prices as well as a time series on regional production and rainfall. Quantitative analysis and regressions outlined key vulnerability factors of households in both surveyed districts.
Paper 51	Vulnerability of rural households to climate change and extremes: Analysis of Chepang households in the Mid-Hills of Nepal	Piya	2012	Yes	Household survey and subsequent Principal Component Analysis for IPCC vulnerability framework. The coefficient of the trends of climate variables (rainfall and temperature) was calculated using ArcGIS and calculated separately for each household. The PCA identified vulnerability determinants under sensitivity, exposure and adaptive capacity categories.
Paper 52	Current vulnerability in the Tri-National de la Sangha landscape, Cameroon	Devissche r	2013	No	Multiple data collection methods applied to understand vulnerability under a dynamic vulnerability framework, but analysis was qualitative descriptions of the survey and community results.
Paper 53	Rural Households: Socio-Economic Characteristics, Community Organizing and Adaptation Abilities	Bruun	2013	No	Used an existing socio-economic survey to identify livelihood changes and impact of climate. Some group of vulnerability was made with specific combinations of vulnerability factors. However the method was a qualitative descriptive review based on expert opinion of the author and local knowledge.
Paper 54	Livelihood Strategies Under the Constraints of Climate Change Vulnerability in Quang Nam	Casse	2013	No	Looked at vulnerability after a disaster (typhoon) and investigated the standard deviation of income levels to determine vulnerability factors and where significant interactions may have occurred. Key factors outlined were poverty, inequality and institutional adaptation. Analysis focused on the impact of the typhoon versus general vulnerability factors
Paper 55	Perceptions of climate change, multiple stressors and livelihoods on marginal African coasts	Bunce	2010	No	Carried out rapid rural appraisals and participatory field work in Tanzania and Mozambique with a small sample to understand stressors to livelihoods. Outlined climate change as a major factor but analysis was descriptive and based on small sample and not focused on additional vulnerability determinants.

Paper 56	Natural Resource Management Impact on Vulnerability in Relation to Climate Change: A Case in a Micro-Scale Vietnamese Context	Platten-Hallermund	2013	No	Small household survey and interview to find out changes. Descriptive analysis of results, methodology for analysis was not clearly laid out.
Paper 57	Poverty, vulnerability and the impact of flooding in the Limpopo Province, South Africa	Khandlhela	2006	No	Multi-dimensional approach to the analysis of vulnerability in the face of floods. Descriptive analysis of impact a specific flood had on communities and most affected assets and factors.
Paper 58	The Livelihood Vulnerability Index: A pragmatic approach to assessing risks from climate variability and change—A case study in Mozambique	Hahn	2009	Yes	Developed an LVI for two communities in Mozambique to quantify the strength of current indicators in response to current exposure to climate extremes. Determined factors that contributed to increased vulnerability.
Paper 59	Exploring vulnerability and adaptation to climate change of communities in the forest zone of Cameroon	Bele	2013	No	Assess local people's vulnerability to climate change in the humid forest zone of Cameroon in order to understand how they are affected and respond and to identify their specific needs for adaptation. Analysis was through descriptions and focus was on climate factors and impact on livelihoods.
Paper 60	Vulnerability Assessment of Weather Disasters in Syangja District, Nepal: A Case Study in Putalibazaar Municipality	Shrestha	2005	No	This assessment includes analysis of current vulnerability as the quantitative integration of physical and socio-economical vulnerability, analysis of existing qualitative adaptive capacity and identification of adaptive measures in reducing the vulnerability. The analysis was descriptive and focused on potential coping mechanisms.
Paper 61	Spatial vulnerability assessments of rural households to climate change in Nigeria: Towards evidence-based adaptation policies	Madu	2012	No	Assessed district level vulnerability comparing urban and rural areas of Nigeria. Performed cluster analysis and identified determinants of each level of vulnerability. Focus was on adaptive actions and policy needs.
Paper 62	Climate Change and Vulnerability to Food Insecurity among Smallholder Farmers: A Case Study of Gweru and Lupane Districts in Zimbabwe	Mtswangwa	2011	Yes	This study assesses the vulnerability of smallholder farmers in two districts of Zimbabwe by assessing the likelihood of individual households being food insecure. The study assesses how households' own production levels interact with household characteristic. Regression carried out to determine significant factors with cereal production.
Paper 63	Measuring Household Food Vulnerability: Case Evidence from Northern Mali	Christiaensen	2000	No	Develop a methodology to analyze and measure household food vulnerability, defined as the probability now of caloric shortfall in the future.

Paper 64	Measuring Vulnerability and Poverty Estimates for Rural India	Gaiha	2008	No	Assessment of the vulnerability of rural households using panel data was made with ex ante and ex post measures of vulnerability calculated using poverty based vulnerability framework and econometric methods. Aggregate idiosyncratic and poverty components were calculated.
Paper 65	The Impact of Drought on Household Vulnerability: The Case of Rural Malawi	Makoka	2008	No	Econometric approach to analyzing household vulnerability. Methods were descriptive and not analytical.
Paper 66	Quantifying Vulnerability to Poverty: A Proposed Measure, with Application to Indonesia	Pritchett	2000	No	Quantified vulnerability to poverty. The outcomes, although quantified by the econometric model didn't show a clear methodological approach to differentiate determinants beyond the two data sets used.
Paper 67	Vulnerability assessment if the climate risks in the lower Songkhram River Basin,Thailand	UNDP	2007	No	No evaluation methodology applied and results were descriptive.
Paper 68	Village vulnerability assessment and climate change adaptation planning (V&A) Mlingotini & Kitonga, Bagamoyo district, Tanzania	Tobey	2011	No	Range of data collection but descriptive analysis of results.
Paper 69	Farmer Vulnerability Amidst Climate Variability: A case study of Dry Zone of Myanmar	Kyi	2012	No	Did not utilize data collection / secondary sources of data or an applied set of methods to describe vulnerability outcomes.
Paper 70	Climate change impacts on livelihood, vulnerability and coping mechanisms. A case study of West-Arsi Zone, Ethiopia.	Senbeta	2009	No	Vulnerability groupings were made with no clear empirical rationale.
Paper 71	Stakeholders' views in reducing rural vulnerability to natural disasters in Southern Mexico: Hazard exposure and coping and adaptive capacity	Saldana-Zorilla	2008	No	Multiple methods of data collection, but analysis was focused on coping responses and methods to reduce vulnerability, rather than the determinants in the surveyed communities.

Appendix B: Email sent to vulnerability experts

Climate vulnerability review project

<[redacted]> Tue, Jul 22, 2014 at 2:55 PM

As part of its work in the Climate Change, Agriculture and Food Security programme (ccafs.org), the International Livestock Research Institute (www.ilri.org) is conducting a systematic review of research on local level vulnerability to climate variability in rural communities. The purpose of our study is to identify best practices for tracking changes in climate vulnerability, the results of which will serve as an important input for the CCAFS programme over the coming years. However, our study will only be as good as the publications we review. We have thus far conducted systematic searches of research databases for empirical articles measuring climate vulnerability (resulting in over 300 articles) and screened them for their precision and transparency in any one or a mix of the following aspects: conceptualization, operationalization, empirical measurement and analysis.

Within our systematic review approach, we would like help from you, as an expert in the field, to make sure that we include a) all significant frameworks for the study of local vulnerability to climate change, and b) to make sure that we review an ideal mix of examples of empirical cases within each of these frameworks. Within each framework, we expect that papers will not present all aspects equally well. As such, we intend to synthesize across papers within each model. To support our synthesis, we need a mix of papers, each of which is excellent in one or several aspects of studying climate vulnerability (conceptualization, operationalization, empirical measurement and analysis).

The attachment to this email contains a preliminary distillation and analysis of materials we have gathered so far. In the interest of cross-checking our work to ensure that we have not overlooked any important papers or models, we would like to ask that you review the attachment with an eye toward the following questions:

1. Are there additional*models* that we have missed? If yes,

a. what article(s) best describe the model

b. what articles are the best examples of its operationalization

2. Are there strong*papers* that should be substituted for, or added to, those we have listed with each of the models we have identified?

We will appreciate any suggestions you contribute to refine and strengthening our list. Please provide a brief justification for any suggested additions or substitutions, and do not be shy about indicating your own work where appropriate. Examples from both academic and grey literature are welcome.

You are undoubtedly very busy over the coming weeks, but we would appreciate receiving your response by September 1, so that we can proceed with our analyses in a timely fashion.

Do not hesitate to contact me with any questions or concerns you may have regarding our project. We look forward to receiving your input.

Best wishes,

[redacted]

Livestock Systems and Environment

International Livestock Research Institute

Nairobi, Kenya

Climate vulnerability framekworks.docx
32K

Attachment:

Seven frameworks for the study of local level climate vulnerability and good examples thereof

Framework: IPCC and adaptations at Household level

Description: This framework looks at vulnerability as conceived by IPCC (2001; 2007) or Fussel (2007), or adaptations of these approaches, and is operationalised on a household level.
The IPCC framework identifies three dimensions of vulnerability:

- Exposure to climate-change induced shocks or hazards
- Sensitivity to climate-change induced shocks or hazards
- Adaptive Capacity – the capacity to adapt to or mitigate the effects of climate change induced shocks or hazards

The framework seeks to identify which determinants have the greatest impact on household vulnerability, as defined above.
Best Example: Luers et al (2003) A method for quantifying vulnerability, applied to the agricultural system of the Yaqui Valley, Mexico. *Global Environmental Change* 13: 255–267
Description of example: This paper presents a methodology which is then applied to a case study of an agricultural system in Mexico. It is an early example of an attempt to create a comprehensive methodology around the IPCC framework. The methodology measures the vulnerability of a variable of concern (in this case wheat yields) to stressors (climate change or market shocks) as a function of exposure and sensitivity indicators. Vulnerability score is however countered by measures for adaptive capacity, which is the major contribution of this paper.
Other examples:
Tesso, Gutu, Bezabih Emana, and Mengistu Ketema
2012 Analysis of Vulnerability and Resilience to Climate Change Induced Shocks in North Shewa, Ethiopia. Agricultural Sciences 3(6): 871–888.

Westerhoff, Lisa, and Barry Smit
2009 The Rains Are Disappointing Us: Dynamic Vulnerability and Adaptation to Multiple Stressors in the Afram Plains, Ghana. Mitigation and Adaptation Strategies for Global Change 14(4): 317–337.

Supporting literature:
IPCC
2001 Climate Change 2001: Impacts, Adaptation, and Vulnerability. Third Assessment Report of the IPCC. UK: University Press, Cambridge.
2007 Climate Change 2007: Impacts, Adaptation, and Vulnerability. Contribution of Working Group II to the Third Assessment Report of the Intergovernmental Panel on Climate Change. Cambridge, UK: Cambridge University Press.

Füssel, Hans-Martin
2007 Vulnerability: A Generally Applicable Conceptual Framework for Climate Change Research. Global Environmental Change 17: 155–167.

Framework: IPCC and adaptations at local level

Description: This framework is defined similarly to that above, but is operationalised at a village level. The framework is used to make comparisons between villages in terms of what factors impact on vulnerability and identify any locality-specific factors.

Best Example: Jamir et al (2013) Farmers' vulnerability to climate variability in Dimapur district of Nagaland, India. *Regional Environmental Change* 13(1): 153-164

Description of example: This paper constructs an indicator-based model of vulnerability, based on the three IPCC dimensions of exposure, sensitivity, and adaptive capacity, to examine farmers' vulnerability to climate-induced stress, in this case to drought. The research uses a combination of household surveys, participatory rural appraisals, and secondary data to examine the contribution of a set of factors categorised as biophysical, agricultural, demographic, and socio-economic, to farmers' vulnerability. Households in five villages in Nagaland in India are surveyed, and these villages are then ranked according to village-level vulnerability scores.

Other examples

Antwi-Agyei, Philip, Andrew J. Dougill, Evan D. G. Fraser, and Lindsay C. Stringer
2013 Characterising the Nature of Household Vulnerability to Climate Variability: Empirical Evidence from Two Regions of Ghana. Environment, Development and Sustainability 15(4): 903–926.

Hahn, Micah B., Anne Riederer, and Stanley Foster
2009 The Livelihood Vulnerability Index: A Pragmatic Approach to Assessing Risks from Climate Variability and change—A Case Study in Mozambique. Global Environmental Change 19: 74–88.

Supporting literature:

IPCC
2001 Climate Change 2001: Impacts, Adaptation, and Vulnerability. Third Assessment Report of the IPCC. UK: University Press, Cambridge.
2007 Climate Change 2007: Impacts, Adaptation, and Vulnerability. Contribution of Working Group II to the Third Assessment Report of the Intergovernmental Panel on Climate Change. Cambridge, UK: Cambridge University Press.

Füssel, Hans-Martin
2007 Vulnerability: A Generally Applicable Conceptual Framework for Climate Change Research. Global Environmental Change 17: 155–167.

Framework: Vulnerability as Expected Poverty (and adaptations) at Household level

Description: This framework looks at Vulnerability as Expected Poverty (VEP), as developed by Chaudhuri et al (2002); Christiaensen & Subbarao (2005), or adaptations of these approaches, and is operationalised on a household level.
Household Vulnerability as Expected Poverty is defined as the probability of household income or consumption falling below a defined poverty line given risks of shocks. A household is considered vulnerable if this probability is below a given threshold (e.g. 0.5).
The framework seeks to identify which determinants have the greatest impact on the probability of falling into, or remaining in, poverty.

Best Example: Sarris & Karfakis (2010) Vulnerability to Covariate and Idiosyncratic Shocks and Safety Net Targeting of Rural Households with an Application to Rural Tanzania. *Conference Paper*. http://erd.eui.eu/media/2010/Sarris.pdf.

Description of example: This research takes the Vulnerability as Expected Poverty framework and defines

poverty in terms of consumption. The effects on vulnerability of idiosyncratic shocks and covariate shocks are estimated. The framework is applied to a data set of household survey data from rural smallholder farms in Tanzania.

Supporting literature

Chaudhuri, S., J. Jalan, and A. Sryahadi

2002 Assessing Household Vulnerability to Poverty from Cross-sectional Data: A Methodology and Estimates from Indonesia. Columbia University, Department of Economics Discussion Paper Series 0102(52).

Christiaensen, Luc J., and Kalanidhi Subbarao

2005 Towards an Understanding of Household Vulnerability in Rural Kenya. JOURNAL OF AFRICAN ECONOMIES 14(4): 520–558.

Framework: Vulnerability as Expected Poverty at household and local level

Description: This is defined similarly to above but is operationalised at more than just household level. It is used to compare across villages, sometimes in different climatic zones.

Best Example: Echevin, Damien (2014) Characterizing Vulnerability to Poverty in Rural Haiti. *Journal of Agricultural Economics* 65(1): 131–150.

Description of example: This article uses the Vulnerability as Expected Poverty model, as developed by Chaudhuri et al (2002) and Christiaensen & Subbarao (2005), with poverty defined both by consumption and by income. This model is operationalised at both a household and community level. Data is based on household surveys which gather quantitative information on socio-economic indicators and qualitative information on perceived shocks and coping strategies, which together provides a cross-section of current poverty levels. This data is then analysed according to VEP econometric models to arrive at probabilities of future levels of poverty, and to identify how these probability levels are impacted by different forms of shocks (idiosyncratic or covariate).

Other examples

Günther, Isabel, and Kenneth Harttgen

2009 Estimating Households Vulnerability to Idiosyncratic and Covariate Shocks: A Novel Method Applied in Madagascar. World Development 37(7): 1222–1234.

Nkondze, Majahodvwa S., Micah B. Masuku, and Absalom Manyatsi

2013 Factors Affecting Households Vulnerability to Climate Change in Swaziland: A Case of Mpolonjeni Area Development Programme (ADP). Journal of Agricultural Science 5(10): p108.

Supporting literature

Chaudhuri, S., J. Jalan, and A. Sryahadi

2002 Assessing Household Vulnerability to Poverty from Cross-sectional Data: A Methodology and Estimates from Indonesia. Columbia University, Department of Economics Discussion Paper Series 0102(52).

Christiaensen, Luc J., and Kalanidhi Subbarao

2005 Towards an Understanding of Household Vulnerability in Rural Kenya. JOURNAL OF AFRICAN ECONOMIES 14(4): 520–558.

Framework: Food insecurity

Description: This framework takes a conception of food security from the FAO (2013) or Lovedal & Knowles (2006) and adapts them to focus on vulnerability, usually through a combination with either the IPCC

framework or the VEP framework.
Food security is defined as having four dimensions:

- Availability or production of food
- Access to food
- Stability of availability or access
- Utilisation of available and accessible food.

Best Example: Capaldo et al (2010) A model of vulnerability to food insecurity. *ESA Working paper*. 10(3). http://bvsan.uni.edu.ni:8080/48/1/model_vulnerability.pdf.
Description of example: This study used a conceptual framework of food insecurity (with four dimensions: availability; access; consumption; utilization, following Lovendal-Knowles 2006) which is combined with an adaptation of the Vulnerability as Expected Poverty econometric model, to create a 'Vulnerability as Expected Food insecurity' framework. This is applied to data collected from households in Nicaragua to determine the impact of a set of socio-economic household variables on expected food insecurity.
Other examples
Sietz, Diana, Sabino Edgar Mamani Choque, and Matthias K. B. Lüdeke
2012 Typical Patterns of Smallholder Vulnerability to Weather Extremes with Regard to Food Security in the Peruvian Altiplano. Regional Environmental Change 12(3): 489–505.
Mutsvangwa, Eness P.
2011 Climate Change and Vulnerability to Food Insecurity among Smallholder Farmers: A Case Study of Gweru and Lupane Districts in Zimbabwe. University of Free State Bloemfontein. http://etd.uovs.ac.za/ETD-db/theses/available/etd-08182011-105132/unrestricted/MutsvangwaEP.pdf.

Supporting literature
FAO, IFAD, and WFP
2013 The State of Food Insecurity in the World 2013: The Multiple Dimensions of Food Security. Rome. http://www.fao.org/docrep/018/i3434e/i3434e.pdf.
Lovendal, Christian Romer, and Marco Knowles
2006 Tomorrow's Hunger: A Framework for Analysing Vulnerability to Food Security. Research Paper, UNU-WIDER, United Nations University (UNU) 2006(119).

Framework: Sustainable livelihoods

Description: This framework takes a livelihoods framework, based on theorists such as Chambers and Conway (1992), Scoones (1998), and Sen (1981) and converts such a framework to deal with the concept of vulnerability to climate-change-induced shocks or risks. In the livelihoods approach household livelihoods are defined as a function of access to five forms of capital (natural, social, financial, physical, human), and strategies of utilising these assets. Such a framework is adapted to study vulnerability usually through using a vulnerability framework such as IPCC (2001), or VEP (Chaudhuri et al 2002), or Fraser et al 2010 in order to categorise or structure data generated through the livelihoods approach.
Best Example: Hahn, M.B., et al., The Livelihood Vulnerability Index: A pragmatic approach to assessing risks from climate variability and change—A case study in Mozambique. Global Environ. Change (2009). doi:10.1016/j.gloenvcha.2008.11.002
Description of example: This study uses a framework derived from classifying the indicators Sustainable Livelihoods Approach according to the three dimensions of the IPCC's concept of vulnerability to climate change variability (exposure, sensitivity, adaptive capacity). Data is collected through household surveys, which is then aggregated up to create village-level vulnerability scores for two villages. Conclusions are drawn as to which sources of vulnerability are most relevant in either village.
Other examples
Sallu, Susannah, Chasca Twyman, and Lindsay C. Stringer
2010 Resilient or Vulnerable Livelihoods? Assessing Livelihood Dynamics and Trajectories in Rural Botswana. Ecology and Society 15(4): 3.
Antwi-Agyei, Philip, Andrew J. Dougill, Evan D. G. Fraser, and Lindsay C. Stringer

2013 Characterising the Nature of Household Vulnerability to Climate Variability: Empirical Evidence from Two Regions of Ghana. Environment, Development and Sustainability 15(4): 903–926.

Supporting literature

Chambers, R., and G. Conway
1992 Sustainable Rural Livelihoods: Practical Concepts for the 21st Century. IDS Discussion Paper 296.

Chaudhuri, S., J. Jalan, and A. Sryahadi
2002 Assessing Household Vulnerability to Poverty from Crosssectional Data: A Methodology and Estimates from Indonesia. Columbia University, Department of Economics Discussion Paper Series 0102(52).

Fraser, Evan D. G., Andrew J. Dougill, Klaus Hubacek, et al.
2010 Assessing Vulnerability to Climate Change in Dryland Livelihood Systems: Conceptual Challenges and Interdisciplinary Solutions. Ecology and Society 16(3): 3.

IPCC
2001 Climate Change 2001: Impacts, Adaptation, and Vulnerability. Third Assessment Report of the IPCC. UK: University Press, Cambridge.

Scoones, Ian
1998 Sustainable Rural Livelihoods: A Framework for Analysis. Brighton, UK: Institute of Development Studies.

Sen, Amartya
1981 Poverty and Famines: An Essay on Entitlement and Deprivation. Oxford, UK: Clarendon Press.

Framework: Resilience

Description: There does not appear to be much consensus on how resilience is conceptualised. Different theories have been cited (eg DFID; Fraser et a 2010) and variously refers to either the capacity to withstand shocks, and/or the recovery after being hit by shocks.

In terms of withstanding shocks, this can be operationalized at household, community, or agro ecological system level and is measured according to the size of the shock – i.e. the greater the shock withstood, the greater the resilience of the community/household/agro system.

In terms of recovery, this refers to how long it takes a household/community/agro ecological system to return to its pre-shock state.

Best Example: Tesso et al (2012) Analysis of vulnerability and resilience to climate change induced shocks in North Shewa, Ethiopia. *Agricultural Sciences* 3 (2012) 871-888.

Description of example: A framework largely derived from the IPCC (2001) is used to survey rural farm households in Ethiopia. A combination of socio-economic and bio-physical indicators are combined and classified into the three categories of exposure, sensitivity and adaptive capacity. This framework is extended to include a 'resilience' component, which is defined in terms of how long it takes a household to return to a pre-shock operating state (building on DFID). The data is used to create a vulnerability index for each agro ecological zone in the study.

Other examples: None found of sufficient quality.

Supporting literature

Department for International Development
2013 Defining Disaster Resilience: A DFID Approach Paper. UK. https://www.gov.uk/government/uploads/system/uploads/attachment_data/file/186874/defining-disaster-resilience-approach-paper.pdf.

Fraser, Evan D. G., Andrew J. Dougill, Klaus Hubacek, et al.
2010 Assessing Vulnerability to Climate Change in Dryland Livelihood Systems: Conceptual Challenges and Interdisciplinary Solutions. Ecology and Society 16(3): 3.

IPCC
2001 Climate Change 2001: Impacts, Adaptation, and Vulnerability. Third Assessment Report of the IPCC. UK: University Press, Cambridge.

Appendix C: Article-specific construct tables

**Article**: (Antwi-Agyei et al. 2013)					
Construct	Defined?	Definition or further info	Directly Operationalized?	Indirectly operationalized through:	Operational text
Access to livelihood capital assets	Yes	"Traditionally, the SLA has been applied by considering the five livelihood capital assets—human, financial, natural, physical and social—as well as their links to an overall vulnerability context, processes, institutions (both formal and informal) and poli- cies that govern people's access to these capital assets (Scoones 1998)." (Antwi-Agyei et al. 2013, 909)	Yes/no/ not operationalized	[name of construct]	
Adaptive capacity	Yes	Adaptive capacity in the context of climate change has been defined by the IPCC (2007, p. 869)as ''the ability of a system to adjust to climate change (including climate variability and extremes) to moderate potential damages, to take advantage of opportunities, or to cope with the consequences.'' Adaptive capacity connotes some positive attributes of a system that enable it to reduce the adverse impacts (vulnerability) associated with climate change (Engle 2011). (Antwi-Agyei et al. 2013, 905)	No	Livelihoods	
Climatic risk	Yes	a specific climatic risk (Vincent 2007),<br>which in the case of this paper, is	Not operationalized		

		drought<br>(Antwi-Agyei et al. 2013, 905)			
Community	Yes	Nevertheless, households are connected to the wider community, which can greatly influence the decision-making process in relation to the use of pro- ductive resources of a particular household; hence, the need to explore vulnerability and adaptation strategies at the household level in relation to the wider socioeconomic and cultural processes occurring at the community level (Thomas et al. 2007).<br>(Antwi-Agyei et al. 2013, 905)	Yes		communities were Aframso, Babaso and Nyamebekyere located in the Ejura Sekyere- dumasi district of Ashanti region, while vulnerable communities were Adaboya, Ayelbia and Vea located in the Bongo district in the Upper East region (Fig. 1; Antwi-Agyei et al.<br>(Antwi-Agyei et al. 2013, 907)
Diversified livelihood activities	Yes	ortant because diversification has been reported as one of the main strategies for reducing household vulnerability to the impacts of climate change and variability (see Ellis 1998; Barrett et al. 2001). Therefore, the number of livelihood activities that a household was engaged in was also assessed. It is assumed that households with more diversified livelihood sources may be less vulnerable to the impacts of climate change compared to households that depend only on agriculture. The livelihood approach argues that agricul- ture-dependent households may be able to reduce their overall vulnerability to climate variability by diversifying the strategies pursued within their	Yes		Therefore, the number of livelihood activities that a household was engaged in was also assessed. It is assumed that households with more diversified livelihood sources may be less vulnerable to the impacts of climate change compared to households that depend only on agriculture. The livelihood approach argues that agricul- ture-dependent households may be able to reduce their overall vulnerability to climate variability by diversifying the strategies pursued within their livelihood portfolios or specialising to take advantage of a niche (see Ellis 1998; Bebbington 1999; Fraser et al. 2005). Hence, the livelihood vulnerability index is estimated to be directly proportional to the number of livelihood activities in which a household engages. A score of 1 was

		livelihood portfolios or specialising to take advantage of a niche (see Ellis 1998; Bebbington 1999; Fraser et al. 2005). Hence, th<br>(Antwi-Agyei et al. 2013, 912)			therefore given to households that had only one livelihood activity, 2 for households having two livelihood activities, 3 for those with three livelihood activities, 4 for those with four livelihood activities, and households with[4 livelihood activities scored 5.<br>(Antwi-Agyei et al. 2013, 912)
Drought	No				
Exposure	Yes	Exposure relates to the extent to which a particular system may be exposed to climatic stresses or variations (IPCC 2007).<br>(Antwi-Agyei et al. 2013, 905)	Yes		In this regard, it is assumed that households within the same agroecological zone may be exposed to the same level of climate anomaly (drought in this case) (Eakin and Bojorquez-Tapia 2008).<br>(Antwi-Agyei et al. 2013, 905)
Financial capital	Yes	Financial capital assets such as savings and remittances play a crucial role in cushioning households against drought-related food shortages. Eliciting information on financial assets was very problematic because of a lack of records on sales and memory lapses. Livestock were considered to offer readily available cash in times of crop failure due to erratic rainfall patterns in the study communities.<br>(Antwi-Agyei et al. 2013, 911)	Yes		Financial capital assets such as savings and remittances play a crucial role in cushioning households against drought-related food shortages. Eliciting information on financial assets was very problematic because of a lack of records on sales and memory lapses. Livestock were considered to offer readily available cash in times of crop failure due to erratic rainfall patterns in the study communities. Indeed, Hesselberg and Yaro (2006) argue that a peasant household's ability to obtain food in northern Ghana, especially in the lean season, largely depends on the availability of disposable livestock and poultry. Households without poultry or livestock scored 1 whilst those with livestock scored 2. In addition, financial assets were assessed by examining the remittances received by the household

					from family members or friends over the past 12 months. In rural agriculture-dependent communities, remittances from family and friends play a crucial role in helping farmers to cope with the livelihood impacts resulting from climate variability. Households that received remittances in the last 12 months scored 2 and those that did not receive any remittances scored 1. Access to credit may also influence adaptation to climate change including access to inputs such as improved cultivars of crops (e.g. Butt et al. 2006; Fosu-Mensah et al. 2012). Hence, it is assumed that households that have no access to credit will be more vulnerable and scored 1 whilst those with access to credit were given a score of 2. (Antwi-Agyei et al. 2013, 911)
Household	Yes	The household was selected as the main unit of analysis because major decisions about adaptation to climate change and livelihood processes are taken at the household level (Thomas et al. 2007). (Antwi-Agyei et al. 2013, 905)	No	Community	
Human Capital	Yes	Human capital assets were represented by two indicators: the educational level of the head of the household (or the most educated person in the household) and the health status of the household (Table 1). (Antwi-Agyei et al. 2013, 910)	Yes		Human capital assets were represented by two indicators: the educational level of the head of the household (or the most educated person in the household) and the health status of the household (Table 1). No formal education was afforded a value of 1; 2 in the case of only primary education; 3 in the case of secondary education; and 4 for households that had tertiary education.

					As there is a link between health and climate change (Haines et al. 2006), it is assumed that households with significant health problems will have lower human capital as they must allocate a substantial part of their scarce resources to treating illnesses (e.g. Allison et al. 2009), thereby reducing their capacity to withstand the impacts of climate variability. To assess health status, households were asked about the number of times they have been to the hospital (or hospitalised) within the last 12 months. House- holds with members that had been to the hospital were scored 1 whilst those with members that had not been to hospital as out patients (and those not needing any medical attention) within this period were scored 2. Also, situations where members of a household required hospital treatment but could not arrange transport and other resources needed were taken into consideration when scoring such a household.<br>(Antwi-Agyei et al. 2013, 910)
Livelihood capital assets	Yes	Traditionally, the SLA has been applied by considering the five livelihood capital assets—human, financial, natural, physical and social—<br>(Antwi-Agyei et al. 2013, 909)	No	Social capital; financial capital; natural capital; physical capital; human capital	
Livelihoods	Yes	Traditionally, the SLA has been applied by considering the five livelihood capital assets—human, financial, natural, physical and social—as well as their links to an	No	Livelihood capital assets	

		overall vulnerability context, processes, institutions (both formal and informal) and poli- cies that govern people's access to these capital assets (Scoones 1998). (Antwi-Agyei et al. 2013, 909)			
Natural capital	Yes	Natural capital assets were assessed by two indicators. The first was the size of the farm holding under cultivation scored 3; those cultivating 16-20 acres scored 4, and households cultivating [20 acres scored 5. T (Antwi-Agyei et al. 2013, 910)	Yes		Natural capital assets were assessed by two indicators. The first was the size of the farm holding under cultivation (this was estimated as the average area of cultivated land over the past 5 years) (Table 1). It is assumed that the larger the farm holding, the greater the opportunity for the household to have more crops and yield, and hence the lower the vulnerability to climate change, though it is noted that labour availability and financial capital both affect the reality of how much land can be cultivated. Households which cultivated less than 5 acres scored 1; those cultivating between 5 and 10 acres scored 2; those cultivating between 11 and 15 acres scored 3; those cultivating 16-20 acres scored 4, and households cultivating [20 acres scored 5. The type of land tenure and level of security it provides may have serious implications for the management of agricultural soils and could indirectly affect crop productivity and environmental sustainability, conse- quently influencing household vulnerability (Butt et al. 2006). Three different tenure arrangements were identified in the study communities. These were ''land inherited'', ''land purchased'' and ''land rented'' by the

					household. A score of 1 was given to households who rented their farmlands; 2 for households who purchased their farmlands; and 3 for those who inherited their farmlands. Households that inherited their farm lands were given the highest score because it is assumed that they will have the most secure land tenure.<br>(Antwi-Agyei et al. 2013, 910)
Physical capital	Yes	Physical assets that were assessed included the presence of irrigation facilities and own- ership of radios, television or mobile phones by a household (Table 1).<br>(Antwi-Agyei et al. 2013, 911)	Yes		Physical assets that were assessed included the presence of irrigation facilities and own- ership of radios, television or mobile phones by a household (Table 1). Irrigation facilities are crucial for rain-fed agriculture-dependent households, as these facilities help farmers to practise dry season farming. It is assumed that households with irrigation facilities will be less vulnerable to changing rainfall patterns. Hence, households without irrigation facilities scored 1, whilst those with these facilities scored 2. The presence of radios, television or mobile phone in a rural household can be an effective tool for communication and accessing information on changing weather patterns (see Naab and Koranteng 2012). Here, households with any of these three assets scored 2, and those without any scored 1. Physical assets such as road networks and the availability of markets and health facilities may enhance the adaptive capacity of a household (see Zhang et al. 2007). These assets were not included in the vulnerability computation because

					field observations suggested that these physical assets did not significantly differ amongst either the resilient or vul- nerable communities.<br>(Antwi-Agyei et al. 2013, 911)
Resilience	Yes	Consideration of resilience in this paper provides the opportunity to explore livelihood dynamics in order to understand the capacity of a particular system to withstand the adverse impacts of climate variability (Marschke and Berkes 2006).<br>(Antwi-Agyei et al. 2013, 905)	No	Livelihoods	
Resilient and vulnerable communitie s	Yes	was based on a definition of ‘‘vulnerable’’ regions and districts as those where relatively minor perturbations in rainfall over the past 40 years had significant impacts on crop yields (Antwi-Agyei et al. 2012). Conversely, ‘‘resilient’’ regions and districts were defined as those where even large droughts were observed to have had only minor impacts on crop yields (Simelton e<br>(Antwi-Agyei et al. 2013, 906)	Yes		communities were Aframso, Babaso and Nyamebekyere located in the Ejura Sekyere- dumasi district of Ashanti region, while vulnerable communities were Adaboya, Ayelbia and Vea located in the Bongo district in the Upper East region (Fig. 1; Antwi-Agyei et al. 2012).<br>(Antwi-Agyei et al. 2013, 907)
Resilient and vulnerable households	No				
Sensitivity	Yes	sensitivity determines the response of a given system to climate change and may be shaped by socioeconomic and ecological conditions of the system (IPCC 2007).<br>(Antwi-Agyei et al. 2013, 905)	No	Livelihoods	

Social capital	Yes	Social capital—including connections to technical support and social resources such as networks, associations and affiliations—was assessed by counting the number of associations or groups to which the members of the household belong (Antwi-Agyei et al. 2013, 909)	Yes		A scoring procedure for social capital followed the methods of Vincent (2007). A score of 1 was given to households that belonged to no identifiable group, 2 for those who were members of one group, 3 for membership of two groups and 4 for membership of more than three groups. (Antwi-Agyei et al. 2013, 910)
Socio-economic, environmental, and community characteristics	No				
Vulnerability	Yes	Nevertheless, the most commonly accepted approach, which is the approach adopted in this paper, comes from the Intergovernmental Panel on Climate Change (IPCC)'s definition of vulnerability (to climate change) where vulnerability is ''the degree to which an environmental or social system is susceptible to, and unable to cope with, adverse effects of climate change, including climate variability and extremes'' (Antwi-Agyei et al. 2013, 904)	No	Access to livelihood capitals; diversified livelihood activities; exposure; sensitivity	

**Article**: (Baca et al. 2014)					
Construct	Defined?	Definition or further info	Directly Operationalized?	Indirectly operationalized through:	Operational text
Adaptation strategies	No		Yes/no/ not operationalized	[name of construct]	

Adaptive capacity		In contrast, adaptive capacity is defined as a system's ability to adjust to climate change in order to reduce or mitigate possible damage [3]. Adaptive capacity is dynamic, and depends partly on the society productive base, such as: natural and artificial assets, social benefits and networks, human capital and institutions, governance, national income, health and technology [2], and how much capability a society has to adapt to the changes so as to maintain, minimize loss of, or maximize gain in welfare.<br>(Baca et al. 2014, 2)	Yes		Indicators of the sensitivity to climate change and adaptive<br>capacity were devised in collaboration with organizations and experts from the region using an expert panel, focus groups, and semi-structured interviews. For the expert panel, semi-structured individual interviews were conducted with 17 key informants of the coffee sector in Nicaragua, including technicians, farmers and researchers. It included questions about the most important factors affecting coffee production. Four focus groups were carried out in Nicaragua and three groups in each of the remaining countries (El Salvador, Guatemala and Mexico). Participants discussed and assessed the significance of climate change over time and identified key indicators for coffee livelihoods. The list of key indicators was structured according to the five community capitals (natural, human, social, physical and financial) of the Livelihoods Approach [1].<br>(Baca et al. 2014, 3)
Exposure		Exposure is the nature and extent of changes that a place's climate is subjected to with regard to variables such as temperature, precipitation, and extreme weather events.<br>(Baca et al. 2014, 2)	Yes		Exposure To quantify exposure to climate change, crop suitability models predicting future changes of climatic suitability of coffee were used for the four countries. The methodology combined current climate data with future climate change predictions. To map current climatic suitability, the historical climate database WorldClim (www.worldclim.org) was used. The variables included a total of 19 bioclimatic variables derived from<br>(Baca et al. 2014, 3)
Sensitivity		Sensitivity is a measure of how systems	Yes		Indicators of the sensitivity to climate

		could be affected by the change in climate (e.g. how much crop yields change or how much human health might be affected).<br>(Baca et al. 2014, 2)			change and adaptive<br>capacity were devised in collaboration with organizations and experts from the region using an expert panel, focus groups, and semi-structured interviews. For the expert panel, semi-structured individual interviews were conducted with 17 key informants of the coffee sector in Nicaragua, including technicians, farmers and researchers. It included questions about the most important factors affecting coffee production. Four focus groups were carried out in Nicaragua and three groups in each of the remaining countries (El Salvador, Guatemala and Mexico). Participants discussed and assessed the significance of climate change over time and identified key indicators for coffee livelihoods. The list of key indicators was structured according to the five community capitals (natural, human, social, physical and financial) of the Livelihoods Approach [1].<br>(Baca et al. 2014, 3)
Vulnerability of coffee farming communities		For our methodology, vulnerability is defined as changes in climate variables that affect agricultural and natural systems over a timeframe. The vulnerability in the livelihoods of small coffee farmers is a function of three factors: exposure, sensitivity and adaptive capacity.<br>(Baca et al. 2014, 2, 3)	No	Exposure; sensitivity; adaptive capacity	

**Article**: (Berkes and Ross 2013)

Construct	Defined?	Definition or further info	Directly Operationalized?	Indirectly operationalized through:	Operational text
Adaptive capacity	Yes/no	Adaptive capacity is the capacity of actors in a system to influence resilience<br>(Folke et al. 2010), and often works through social networks and learning communi- ties (Goldstein 2012).<br>[...]<br>We view adaptive capacity as a latent pro- perty, which can be activated when people exercise their agency. The processes by which this occurs have not been well explored.<br>(Berkes and Ross 2013, 15)	not operationalized		
Agency	No				
Community resilience		Community resilience as a function of the strengths or characteristics that have been identified as important, leading to agency and self-organization. (Berkes and Ross 2013) 14 (Berkes and Ross 2013, 14)	not operationalized		
Self-organizing	No				

**Article**: (Bogale, Taeb, and Endo 2006)					
Construct	Defined?	Definition or further info	Directly Operationalized?	Indirectly operationalized through:	Operational text
Common property	No		Yes/no/ not operationalized	[name of construct]	
Household choice	No				
Private property	No				

Property rights	Yes	Property rights can be defined as bthe capacity to call upon the collective stand behind one's claim to a benefit stream (Bromley, 1991).Q Thus, property rights involve a relationship between the right holder, others, and an institution to back up the claim (Bogale, Taeb, and Endo 2006, 136)	Not operationalized		
Property rights regime	Yes	Property rights over land and other natural resources are often broadly classified as public, com- mon, and private or blegal individualsQ such as com- panies.<br>(Bogale, Taeb, and Endo 2006, 136)	Not operationalized		
Public property	No				
Vulnerability	No				

**Article**: (Calvo and Dercon 2013)					
<u>Construct</u>	<u>Defined?</u>	<u>Definition or further info</u>	<u>Directly Operationalized?</u>	<u>Indirectly operationalized through:</u>	<u>Operational text</u>
Aggregate vulnerability	Yes	Theorem 2 F satisfies SOS, D, SI, SDEO, PTe, N, CRRSe, SCO, SOI and RI if and only if<br>F (z,p,Y) = � 1<br>� �1−E<br>� �<br>� n<br>� i=1<br>xi<br>1 n<br>��� �	No	Individual vulnerability	

		� �, with�<0 or a positive multiple thereof. (See proof in Appendix 3). The requirement�<0 follows from our version of SCO. If we had followed risk equity and enforced a negative reaction to stronger positive correlations in individual outcomes, the condition would read 0 <�< 1, provided we impose an additional axiom securing risk sensitivity (see footnote 12 and the proof in Appendix 3). (Calvo and Dercon 2013, 729)			
Covariant shocks	No				
Idiosyncratic shocks	No				
Individual vulnerability	Yes	Let individual vulnerability (v) depend on the poverty line (z ∈ R++), a vector con- taining outcomes for k possible states of the world �y ∈ Rk s stand for an r-dimensional v = f (z,p, y). Next, define expected outcome E[y] ≡ �k the corresponding probabilities �p ∈ Pk�, where Pk ≡ �p ∈ Rk Vulnerability is thus measured by a function f : R++ × Pk × Rk +: �k +�, and a vector containing s=1 ps = 1�. outcome yc, which is determined by f (z,p, y) ≡ f (z,p, yc1k). Also, define vector x and its elements xs ≡ ys s=1 ps ys and the risk-free	No	poverty line; possible states of the world; probabilities of possible states of the world.	

		equivalent With this notation, (z,p, y) will summarise the information of an individual who z , which rescale outcomes in terms of the poverty line.<br>realises and fears that, with some likelihood, the future may turn out to be a state of affairs, where outcome is painfully low. It will be convenient, though not necessary, to think of our outcomes ys as consumption levels. What we need to remark is that outcomes aremeasured after all smoothing efforts have been deployed<br>(Calvo and Dercon 2013, 724)			
Possible states of the world	Yes	the proba-<br>bility of low outcomes or overall risk exposure (as defined in Rothschild and Stigliz 1970) increases.<br>(Calvo and Dercon 2013, 725)	Yes		It will be convenient, though not necessary, to think of our outcomes ys as consumption levels.<br>(Calvo and Dercon 2013, 724)<br>[...]<br>Consumption values were constructed using the total value of food and non-food consumption, based on purchased items, as well as from the own harvest and from gifts. They were deflated using a local food Laspeyres price deflator using 1994 as the base.<br>(Calvo and Dercon 2013, 732)<br>[...]<br>Further controls are introduced via village fixed effects (a set of dummies) and variables accounting for household composition changes over time. To account for the endogeneity of lagged consump-<br>tion, we used lagged holdings of land and of livestock as identifying instruments.20,21<br>20 Land is not privately owned, but user

					rights are allocated by local authorities, while livestock is both a factor of production for these mixed farmers, and the main liquid asset for accumulation and smoothing. Together they are by far the most important assets in this rural economy. 21 More detailed diagnostics and discussion of the validity of the instruments is available upon request. Note nevertheless that the key purpose is to get a predictionmodel for different values of the shock variables. 22 The Hausman test provided no guidance in our case (as not infrequent in small samples), but the Bre- usch-Pagan test suggested the existence of random e<br>(Calvo and Dercon 2013, 732)
Poverty line	Yes	Our aim is merely to make an ex-ante statement on the vulnerability of the individual to fall below a poverty norm z,<br>(Calvo and Dercon 2013, 724)	Yes		More detailed diagnostics and discussion of the validity of the instruments is available upon request. Note nevertheless that the key purpose is to get a predictionmodel for different values of the shock variables(Calvo and Dercon 2013, 732)
Probabilities of possible states of the world	Yes	the proba-<br>bility of low outcomes or overall risk exposure (as defined in Rothschild and Stigliz 1970) increases.<br>(Calvo and Dercon 2013, 725)	Yes		More detailed diagnostics and discussion of the validity of the instruments is available upon request. Note nevertheless that the key purpose is to get a predictionmodel for different values of the shock variables.<br>(Calvo and Dercon 2013, 732)<br>[...]<br>We can now use this model in each period t − 1 to predict outcomes for possible<br>states of the world. For rainfall, we will be able to use the village-specific distribution as implied by the rainfall patterns of the last 30years. For other sources of risk, we assume for simplicity that these risks are idiosyncratic, and that for each year, the village-specific realisations in the data give

					the probability distribution of this risk.<br>We assume that this village-level distribution is independent of rainfall risk. Alterna- tive distributional assumptions were also explored, with only a limited impact on the findings.<br>(Calvo and Dercon 2013, 733)
Shocks	No				
Vulnerability to poverty	Yes	Remarking that we are interested in vulnerability to poverty will also be useful to<br>preempt any confusion with vulnerability to downfalls in wellbeing. Our reference point is an absolute poverty norm (e.g. as in Chaudhuri 2003; Suryahadi and Sumarto 2003,or Christiaensen and Subbarao 2005), and not the initial individual position.<br>(Calvo and Dercon 2013, 723)	No	Individual vulnerability; aggregate vulnerability[28]	
vulnerability	Yes	In this article, we explore the notion of vulnerability to poverty, closely linked with the magnitude of the threat of poverty, measured ex-ante, before uncertainty has been resolved.<br>[...]<br>To clarify how all these intuitions come together under the concept of vulnerability, this paper proposes an axiomatic approach to themeasurement of both individual and aggregate vulnerability.<br>(Calvo and Dercon 2013, 722)	No	Individual vulnerability; aggregate vulnerability	

[28] The deconstruction of vulnerability to poverty as composed of these two constructs is strongly implied, although never made entirely explicit.

**Article**: (Capaldo et al. 2010)					
Construct	Defined?	Definition or further info	Directly Operationalized?	Indirectly operationalized through:	Operational text
Access to food	Yes	conceptual framework drawn from it by Løvendal and Knowles (2005). (Capaldo et al. 2010, 7)	Not operationalized	[name of construct]	
Chronically food insecure	yes	undernourished (food insecure) while also being vulnerable; these are considered chronically food (Capaldo et al. 2010, 16)	No	Present food security status; Expected future food security status	
Current exposure to risk	YES	conceptual framework drawn from it by Løvendal and Knowles (2005). (Capaldo et al. 2010, 7)	Yes		We use ex-post data on shocks and risk management strategies. These include information on the incidence of a covariate shock (such as drought) and an idiosyncratic shock (illness), as well as the number of government and non-governmental programs from which households received assistance. In this application, we are not able to complement this with information on future risks and risk management strategies. We note that nearly a quarter of households report being affected by drought (Capaldo et al. 2010, 12)
Current socio-economic characteristics	YES	conceptual framework drawn from it by Løvendal and Knowles (2005). (Capaldo et al. 2010, 7)	Yes		We estimate daily per capita kilocalorie consumption as a function of several variables representing the households' demographic and social characteristics, asset holdings, liquidity constraints, access to infrastructure, occurrence of shocks

					and geographic location. Special attention is given to households that are linked to - or earn a significant proportion of their livelihoods from the agricultural sector. Table 1 provides a list of all variables, including their mean value and standard deviation. We have omitted from Table 1 the dummy variables for household location. Table 1: Summary of variables (Capaldo et al. 2010, 11)
Events	Yes	conceptual framework drawn from it by Løvendal and Knowles (2005). (Capaldo et al. 2010, 7)	No	Risks; risk management	
Expected future food security status	Yes	conceptual framework drawn from it by Løvendal and Knowles (2005). (Capaldo et al. 2010, 7)	No	Present food security status; events	
Food availability	Yes	conceptual framework drawn from it by Løvendal and Knowles (2005). (Capaldo et al. 2010, 7)	Not operationalized		
Food consumption	Yes	conceptual framework drawn from it by Løvendal and Knowles (2005). (Capaldo et al. 2010, 7)	Not operationalized		
Food security	Yes	As table 4 shows, only 44.3% of households enjoy stable levels of food security in our sample; that is they are food secure and not vulnerable. On the other hand, 20.3% of the population is undernourished (food insecure) while also being vulnerable; these are considered chronically food insecure. 29.2% of households are currently undernourished but only temporarily (transient food insecure). Most	No	Present food security status; Expected future food security status	

		importantly, about 6% of households in our sample are food secure at present, while being at risk of being undernourished (food insecure) in the future. Therefore, in the case of Nicaragua a targeting error could potentially affect more than one third of the population (29.2%+6.2%=35.4%). Overall, in Nicaragua 26.5% of households are vulnerable to food insecurity, exhibiting an average vulnerability of 77%. (Capaldo et al. 2010, 16)			
Food utilization	Yes	conceptual framework drawn from it by Løvendal and Knowles (2005). (Capaldo et al. 2010, 7)	Not operationalized		
Future food security	Yes	conceptual framework drawn from it by Løvendal and Knowles (2005). (Capaldo et al. 2010, 7)	Not operationalized		
Future nutritional status	Yes	conceptual framework drawn from it by Løvendal and Knowles (2005). (Capaldo et al. 2010, 7)	Not operationalized		
Permanently food secure	Yes	BY DEFAULT	No	Present food security status; Expected future food security status	
Present characteristics	Yes	conceptual framework drawn from it by Løvendal and Knowles (2005). (Capaldo et al. 2010, 7)	No	Current socio-economic characteristics; current exposure to risks	
Present food security status	Yes	conceptual framework drawn from it by Løvendal and Knowles (2005). (Capaldo et al. 2010, 7)	No	Present characteristics	

Risk management	Yes	conceptual framework drawn from it by Løvendal and Knowles (2005). (Capaldo et al. 2010, 7)	Yes		We use ex-post data on shocks and risk management strategies. These include information on the incidence of a covariate shock (such as drought) and an idiosyncratic shock (illness), as well as the number of government and non-governmental programs from which households received assistance. In this application, we are not able to complement this with information on future risks and risk management strategies. We note that nearly a quarter of households report being affected by drought (Capaldo et al. 2010, 12)
Risks	Yes	conceptual framework drawn from it by Løvendal and Knowles (2005). (Capaldo et al. 2010, 7)	Yes		We use ex-post data on shocks and risk management strategies. These include information on the incidence of a covariate shock (such as drought) and an idiosyncratic shock (illness), as well as the number of government and non-governmental programs from which households received assistance. In this application, we are not able to complement this with information on future risks and risk management strategies. We note that nearly a quarter of households report being affected by drought (Capaldo et al. 2010, 12)
Transitory food insecure	Yes	undernourished but only temporarily (transient (Capaldo et al. 2010, 16)	No	Present food security status; Expected future food security status	

Transitory food secure		food secure at present, while being at risk of being undernourished (food insecure)<br>(Capaldo et al. 2010, 16)	No	Present food security status; Expected future food security status	
Vulnerability	Yes	conceptual framework drawn from it by Løvendal and Knowles (2005).<br>(Capaldo et al. 2010, 7)	Not operationalized		
Vulnerability to future food insecurity	Yes	conceptual framework drawn from it by Løvendal and Knowles (2005).<br>(Capaldo et al. 2010, 7)		Expected future food security status	

**Article**: (CARE 2009)					
Construct	Defined?	Definition or further info	Directly Operationalized?	Indirectly operationalized through:	Operational text
Adaptation to climate change	Yes/no	Adjustment in natural or human systems in response to actual or expected climatic stimuli or their effects, which moderates harm or exploits beneficial opportunities.10<br>(CARE 2009, 7)	Not operationalized		
adaptive capacity	Yes	The ability of a system to adjust to climate change (including climate variability and extremes) to moderate potential damages, to take advantage of opportunities, or to cope with the consequences.6<br>(CARE 2009, 5)	Yes		Capacity Development<br>- What institutions (governmental and non-governmental) are involved in research, planning<br>and implementation of adaptation? What are the most important institutions in facilitating or constraining adaptation? - Do local institutions (governmental and non-governmental) have capacity to monitor and

					- -<br>analyze information on current and future climate risks? Are mechanisms in place to disseminate this information?<br>- Do local institutions have capacity to plan and implement adaptation activities? - Are resources allocated for implementation of adaptation-related policies? What is the budget?<br>Where are the resources coming from? What are the existing capacity and resource needs and/or gaps for climate change adaptation? - What new capacities may be needed to address changing circumstances due to climate change?<br>-<br>[...]<br>Addressing<br>Underlying Causes of Vulnerability<br>- What social groups within the community are most vulnerable to climate change? - Are local planning processes participatory?<br>- Do women and other marginalized groups have a voice in local planning processes? - Do local policies provide access to and control over critical livelihoods resources for all? - What are the other factors constraining adaptive capacity of the most vulnerable groups? Do<br>vulnerable communities and groups have any influence over these factors?<br>(CARE 2009, 16)
Climate change	Yes	Any change in climate over time, whether due to natural variability or as a result of human activity.4<br>(CARE 2009, 5)	Not operationalized		
community level	No				
financial capital	No				

Hazard	Yes	A dangerous phenomenon, substance, human activity or condition that may cause loss of life, injury or other health impacts, property damage, loss of livelihoods and services, social and economic disruption, or environmental damage.9<br>(CARE 2009, 6)	yes		Disaster Risk Reduction<br>- What are the most important climate-related hazards the region and/or ecological zone faces?<br>Non-climate related? How are hazards likely to change over time as a result of climate change? - What groups within the community are most vulnerable to disasters? - Do local institutions have access to disaster risk information? - Are local disaster risk management plans being implemented? - Are functional early warning systems in place at the local level?<br>- Does the local government have the capacity to respond to disasters? - Which other institutions are engaged disaster risk management at local level?<br>-<br>(CARE 2009, 16)
human capital	No				
natural capital	No				
physical capital	No				
Resilience	Yes	The ability of a community to resist, absorb, and recover from the effects of hazards in a timely and efficient manner, preserving or restoring its essential basic structures, functions and identity.8<br>(CARE 2009, 6)	Yes		Resilient Livelihoods - Are scaled-down climate projections available? - If so, what are the observed and predicted impacts of climate change for the region and/or ecological zone? Do local institutions have access to information on current and future climate risks? - What livelihood groups or economic sectors are most vulnerable to climate change? - Do local plans or policies support climate-resilient livelihoods?<br>-<br>- Do local government and NGO extension workers understand climate risks and promote adaptation strategies?<br>(CARE 2009, 16)
social cpaital	No				

vulnerability to climate change	Yes	The degree to which a system is susceptible to, or unable to cope with, adverse effects of climate change, including climate variability and extremes. Vulnerability is a function of the character, magnitude, and rate of climate variation to which a system is exposed, its sensitivity, and its adaptive capacity.5<br>(CARE 2009, 5)	No	Adaptive capacity	

**Article**: (Chhihn and Poch 2012)					
Construct	Defined?	Definition or further info	Directly Operationalized?	Indirectly operationalized through:	Operational text
Climate change	No		Yes/no/ not operationalized	[name of construct]	
Current poverty status	Yes	This study adopts the approach to measuring household economic vulnerability posited and elaborated in Chaudhuri's (2003) study of household vulnerability (Chhihn and Poch 2012, 30)	Yes		Unlike Chaudhuri (2003), who analysed households' monthly per capita consumption expenditure, this study analyses households' monthly income to measure the household vulnerability index due to the lack of expenditure data.<br>(Chhihn and Poch 2012, 30)
Environmental shocks	Yes	This study adopts the approach to measuring household economic vulnerability posited and elaborated in Chaudhuri's (2003) study of household vulnerability (Chhihn and Poch 2012, 30)	Yes		The predictors of log per capita income used in the analysis include: droughts in the past 12 years (dummy); windstorms in the past 12 years (dummy); floods in the past 12 years (dummy); household size; level of education; possession of motored vehicle (dummy); access to credit (dummy); presence of disabled persons in the households (dummy); and the dependency of liveli- hood on agriculture (dummy).<br>(Chhihn and Poch 2012, 30)

Farmers	no				
Household characteristics	Yes	This study adopts the approach to measuring household economic vulnerability posited and elaborated in Chaudhuri's (2003) study of household vulnerability (Chhihn and Poch 2012, 30)	Yes		The predictors of log per capita income used in the analysis include: droughts in the past 12 years (dummy); windstorms in the past 12 years (dummy); floods in the past 12 years (dummy); household size; level of education; possession of motored vehicle (dummy); access to credit (dummy); presence of disabled persons in the households (dummy); and the dependency of liveli- hood on agriculture (dummy). (Chhihn and Poch 2012, 30)
Household vulnerability as expected poverty	Yes	Household vulnerability as ex- pected poverty is defined as the probability that households will move into poverty given certain environmental shocks, current poverty status and household characteristics of respondents. (Chhihn and Poch 2012, 30)	No	Environmental shocks; current poverty status; household characteristics; poverty	
Households	no				
Natural hazards	no				
Poverty	Yes	Technically, the household vulnerability index is derived from the difference between the ex- pected log per capita income and the minimum log per capita income threshold, with households having per capita incomes lower than the minimum per capita income defined as vulnerable (poor). The expected log per capita income is estimated using the three-step feasible generalised least squares (FGLS) method. (Chhihn and Poch 2012, 30)	Yes		Technically, the household vulnerability index is derived from the difference between the ex- pected log per capita income and the minimum log per capita income threshold, with households having per capita incomes lower than the minimum per capita income defined as vulnerable (poor). The expected log per capita income is estimated using the three-step feasible generalised least squares (FGLS) method. (Chhihn and Poch 2012, 30)

**Article**: (Dasgupta and Baschieri 2010)					
Construct	Defined?	Definition or further info	Directly Operationalized?	Indirectly operationalized through:	Operational text
Asset vulnerability	Yes	Using Moser's (1998) asset vulnerability framework as guidance, we selected a range of variables to create an index of household vulnerability from GLSS 4. Each variable captures an aspect of vulnerability. (Dasgupta and Baschieri 2010, 807)	no	Labour; human capital; non-labour productive assets; social capital	
Climate shocks	Yes	climate change shock, namely, drought. (Dasgupta and Baschieri 2010, 810)	No	drought	
Communities	No				
Communities at risk of climate shocks	No				
Drought	Yes	We consider the first approach and use deficiency in rainfall as the definition of drought in this study. [...] for some, drought is defined as a deficiency in rainfall, or rainfall which is lower than the expected amount in a certain period (van der	Yes		We consider the first approach and use deficiency in rainfall as the definition of drought in this study. (Dasgupta and Baschieri 2010, 810)

		Ge<br>(Dasgupta and Baschieri 2010, 810)			
Household relations	Yes	Moser (1998) identified household relations as the fourth asset, as these influence the ability of households to adjust to shocks and changes. Their households are the first safety t for vulnerable individuals. The structure, composition and cohesion of each household's members determine those households' ability to mobilise labour and to share both expenditure-reducing and income-generating strategies. However, the ques<br>(Dasgupta and Baschieri 2010, 808)	Not operationalized		
Household vulnerability to climate change	Yes	Using the GLSS 4, we applied the asset vulnerability framework developed by Moser (1996, 1998, 2007). We constructed an index of vulnerability to climate change, at the household level.<br>(Dasgupta and Baschieri 2010, 807)	No	Asset vulnerability	
Human capital	Yes	The second asset Moser (1998) identified is	Yes		We used the level of education of the heads of households and access to health care as proxies for human capital. It can

		human capital. Social services that offer education, health care and economic infrastructure for water, transport and electricity help to determine the ability of households to work and to profit from that work.<br>(Dasgupta and Baschieri 2010, 808)			be argued that more educated households are likely to survive climate shocks better, as they are more likely to find alternative avenues of employment. Similarly, households which have higher levels of female education are more likely to be able to mobilise more members into the workforce in the event of a shock. Education level was treated as binary where the household head either had achieved primary school education or less, or secondary education or higher. A growing amount of literature also suggests human health is likely to be affected by<br>global climate change (Haines and Parry, 1993; Kovats et al., 2003; Epstein, 2005; Haines et al., 2006). Evidence exists that an increase in infectious diseases including malaria and diarrhoeal diseases is likely in the face of climate change.We therefore included a variable to capture a household's ability to deal with increased morbidity in the vulnerability index, assuming that households without access to decent health facilities would be more likely to be affected by climate change shocks. Data of the existence of health facilities in the community were assigned to each household.We considered the existence of a hospital to be ideal, followed by that of a clinic. The third and lowest category was a household with access to neither a hospital nor clinic.<br>(Dasgupta and Baschieri 2010, 808)
Labour	Yes	The first asset Moser identified is labour (Dasgupta and Baschieri 2010, 807)	Yes		The primary type of work in which the head of the household was engaged was included<br>into the vulnerability index. This variable was binary, the categories being either in agricultural work or not. The percentage of total income derived from agriculture was also included, with a high percentage being taken to indicate more vulnerable households.We created this variable by dividing household income from agriculture by the total household income. It is possible that a household that has a high percentage of income from agriculture could be because they are an agriculturally successful household. Nevertheless, we still consider them to be a vulnerable household, as they are more dependent on climate and

					changes in climate—such as drought—have potentially negative implications for their livelihood. We also considered the percentage of income derived from remittances.We assume that households receiving incomefrom peoplewhowork elsewhere are less vulnerable, because household production and remittance income are less likely to be correlated, and the household is therefore less reliant on one source of income (Moser and Felton, 2007). However, it is important to note that heavy dependence on remittances can in some cases be a sign of vulnerability, as an economic shock elsewhere may stop the flow of remittances. For the purpose of this study, we assume that remittances are a sign of security, as in the event of a climate change shock in the local area, the household is more likely to have an ongoing income in the form of remittances. A variable detailing the proportion of the household that is under 15 or over the age of 65 was included, to reflect how many dependents there are in a household who are less likely to be contributing economically. Finally, we considered the percentage of total household expenditure spent on food. Households that spend a large percentage of their money on food may be considered more vulnerable, as food is a necessity (Sagoe, 2006).<br>(Dasgupta and Baschieri 2010, 808)
Non-labour productive assets	Yes	Non-labour productive assets are the third type. Moser (1998) identified land, sewing machines, radios, refrigerators and motor vehicles as important productive assets for rural households, which can either be used or sold in order to buffer short-term climatic shocks.	Yes		In order to measure the different degrees of productive assets between households we used the total number of productive assets owned by the household as a proxy. Among reproducible capital assets the questionnaire included furniture, sewing machines, stoves, refrigerator-freezers, air conditioners, fans, radios, radio-cassette players, record players, three-in-one radio-cassette players, video equipment, washing machines, TVs, cameras, electric irons, bicycles, motorcycles, cars, houses, land, shares, boats, canoes and outboard motors. Each asset was weighted equally.<br>(Dasgupta and Baschieri 2010, 808)

		(Dasgupta and Baschieri 2010, 808)			
Prepared for adverse consequences	No				
Risk of experiencing climate change shock	Yes	We use average annual rainfall data, which serves as a proxy for risk of climate-change-related shock.<br>(Dasgupta and Baschieri 2010, 810)	Yes		We use average annual rainfall data, which serves as a proxy for risk of climate-change-related shock.<br>(Dasgupta and Baschieri 2010, 810)
Social capital	Yes	Social capital isMoser'sfifth asset as it reduces vulnerability and increases opportunities.<br>Moser and Felton (2007: p. 13) defined social capital as 'the rules, norms, obligations, reciprocity and trust embedded in social relations, social structures and societies' institutional arrangements.' Social capital is generally provided through membership of social networks which can be bonded in a formal or informal nature. Social capital can also be enhanced through social learning and adaptive governance (Olsson et al., 2004; Folke et al.,	Yes		However, social capital is often considered difficult to operationalize in a household survey as it can operate at different levels and scales.We used a variable from the community questionnaire to serve as a proxy. This variable iswhether a system of mutual aid forfieldworkexistedamongthe farmers of thehousehold'scommunity.We considered those with a system of mutual aid to be less vulnerable. It could be argued that social capital is not useful in the face of a climate change shock, as that would affect all the households in a community. If one agricultural household is hit, their neighbours are likely to be hit, too. However, we argue that although these households would be hit equally, a household in the community that is involved in another profession or is more educated might be able to offer assistance to more vulnerable households. Moreover, Brons et al. (2004) found in their study of livelihood strategies in Burkina Faso and Mali that food security depends on institutional and social-exchange networks. We therefore included this proxy for social capital. Althoughsystemsof mutual aidamongfarmers reflect only social networks between those involvedin agriculture, they are still useful as aproxyfornetworks withincommunities between households of different professions and characteristics. We consider social capital in its widest sense as social-resource networks, social groups,

		2005; Pelling and High, 2005; Pelling, 2007). Adaptive governance as a dynamic management approach of social-ecological systems has proven itself particularly useful in periods of crisis as it utilises social sources and social learning, drawing on experiences and common understanding and policies of different groups. In the specific context of climate change a number of studies have identified social capital as important in enhancing the community adaptive capacity to climate change (Adger, 2003; van der Geest, 2004; Bryan et al., 2009)<br>[...]<br>We consider social capital in its widest sense as social-resource networks, social groups, trust and reciprocity (Dasgupta and Baschieri 2010, 809)			trust and reciprocity. For this reason, we also include whether there is a road near the community to which its members have access, as it can be argued that roads are one type of proxy for the extent to which communities are able to interact with the outside world and potentially receive assistance (Sachs, 2005). This information was available in the community-level data.We divided this variable into three main categories: (a) Yes, always usable, (b) Yes, sometimes unusable, (c) No road.<br>(Dasgupta and Baschieri 2010, 809)
Welfare of rural households	No				

**Article**: (Deressa, Hassan, and Ringler 2009)

Construct	Defined?	Definition or further info	Directly Operationalized?	Indirectly operationalized through:	Operational text
Climate and non-climate shocks	no		Yes/no/ not operationalized	[name of construct]	
Ethiopean Farmers	no				
Expected Poverty	Yes	This method is based on estimating the probability that a given shock or set of shocks will move household consumption below a given minimum level (such as a consumption poverty line) or force the consumption level to stay below the minimum if it is already below this level (Chaudhuri et al. 2002). (Deressa, Hassan, and Ringler 2009, 3)	No	Minimum consumption (income) level	
Household consumption (income)	no				
Minimum consumption (income) level	Yes	a given minimum level (such as a consumption poverty line) (Deressa, Hassan, and Ringler 2009, 3)	Yes		The choice of minimum levels of income is based on different assumptions such as the international poverty line of 1.25 US per day (World Bank, 2008), average income of the surveyed households and arbitrary values above and below the average income of the surveyed households. (Deressa, Hassan, and Ringler 2009, 11)
Vulnerability	Yes	Thus, vulnerability is seen as expected poverty, while consumption (income) is used as a proxy for well-being.	No	Expected Poverty	

		(Deressa, Hassan, and Ringler 2009, 3)			

Article: (Eakin, Winkels, and Sendzimir 2009)

Construct	Defined?	Definition or further info	Directly Operationalized ?	Indirectly operationalized through:	Operational text
Cross- scalar teleconnection	Yes	‘‘teleconnections’’, a term used in climatology in relation to ‘‘any transmission of a coherent effect beyond the location where the forcing occurred’’ (Chase et al., 2005). For example, one of the teleconnections associated with the El Nin ˜ o-Southern Oscillation effect is severe drought in Northeastern Brazil. Teleconnections are also associated with other climate phenomena such as the North Atlantic Oscillation. The label of ‘‘teleconnection’’ is not explanatory in and of itself, but rather signifies the existence of a correlation in events, and highlights the need to explore the connecting mechanisms and drivers in order to anticipate outcomes. (Eakin, Winkels, and Sendzimir 2009, 400)	No	Nested system	
Exogenous drivers	Yes	exogenous drivers (i.e. the risk and stress factors) (Eakin, Winkels, and Sendzimir 2009, 399)	Yes		The volatility of prices has historically been a source of significant economic uncertainty for producers. (Eakin, Winkels, and Sendzimir 2009, 401)
Geographically distant household vulnerability	Yes	vulnerabilities and responses of farm households in distinct geographic locations (Eakin, Winkels, and Sendzimir 2009, 400)	Yes		The Mexican case study took place in 2003, as farmers were emerging from the most recent coffee crisis. The research took place in two coffee-producing communities in the region of

					Coatepec, in Central Veracruz. In addition to interviews with public officials, coffee association leaders, academics and coffee processors and traders, a household survey collected data on the perceptions and responses of 60 households to the coffee situation (Eakin et al., 2006). The project was part of a broader study exploring the implications of climatic variability and change for coffee farming in Mexico (Gay Garcia et al., 2006). In Vietnam, a study of migrant livelihoods included many coffee farmers, mostly migrants to the Central Highland region (Winkels, 2004). Livelihood surveys and interviews with 81 households originating from the overpopulated Red River Delta in the north provides important insights into both the opportunities andrisk of coffee farming at Vietnam's southern mountain frontier when the first signs of the looming coffee crisis became evident in 2000 and 2001. (Eakin, Winkels, and Sendzimir 2009, 402)
Geographically specific signals of change	Yes	geographically specific signals of change – such as a shift in market opportunities, a drought, a change in public policy or new form of land use in a specific location – (Eakin, Winkels, and Sendzimir 2009, 400)	Yes		The Mexican case study took place in 2003, as farmers were emerging from the most recent coffee crisis. The research took place in two coffee-producing communities in the region of Coatepec, in Central Veracruz. In

					addition to interviews with public officials, coffee association leaders, academics and coffee processors and traders, a household survey collected data on the perceptions and responses of 60 households to the coffee situation (Eakin et al., 2006). The project was part of a broader study exploring the implications of climatic variability and change for coffee farming in Mexico (Gay Garcia et al., 2006). In Vietnam, a study of migrant livelihoods included many<br>coffee farmers, mostly migrants to the Central Highland region (Winkels, 2004). Livelihood surveys and interviews with 81 households originating from the overpopulated Red River Delta in the north provides important insights into both the opportunities andrisk of coffee farming at Vietnam's southern mountain frontier when the first signs of the looming coffee crisis became evident in 2000 and 2001. (Eakin, Winkels, and Sendzimir 2009, 402)
Household responses	Yes	factors internal to the household (i.e. ability to mitigate and cope with stress)<br>(Eakin, Winkels, and Sendzimir 2009, 399)	Yes		The Mexican case study took place in 2003, as farmers were emerging from the most recent coffee crisis. The research took place in two coffee-producing communities in the region of Coatepec, in Central Veracruz. In addition to interviews with public

					officials, coffee association leaders, academics and coffee processors and traders, a household survey collected data on the perceptions and responses of 60 households to the coffee situation (Eakin et al., 2006). The project was part of a broader study exploring the implications of climatic variability and change for coffee farming in Mexico (Gay Garcia et al., 2006). In Vietnam, a study of migrant livelihoods included many coffee farmers, mostly migrants to the Central Highland region (Winkels, 2004). Livelihood surveys and interviews with 81 households originating from the overpopulated Red River Delta in the north provides important insights into both the opportunities andrisk of coffee farming at Vietnam's southern mountain frontier when the first signs of the looming coffee crisis became evident in 2000 and 2001. (Eakin, Winkels, and Sendzimir 2009, 402)
Livelihood vulnerability	Yes	By placing the household as the focus of analysis, livelihood approaches highlight both the exogenous drivers (i.e. the risk and stress factors) and the factors internal to the household (i.e. ability to mitigate and cope with stress) which together influence household security and well-being (Chambers and Conway, 1992; Ellis, 1998). (Eakin, Winkels, and Sendzimir 2009, 399)	No	Exogenous drivers; geographically specific signals of change; geographically distant household vulnerability;	

				household responses; response outcomes	
Nested and teleconnected livelihood vulnerability	Yes	In this article we use the concept of ‘‘nested and tele-<br>connected vulnerabilities’’ to illustrate how the vulnerabilities and responses of farm households in distinct geographic locations are linked through cross-scalar processes, as well as ‘‘teleconnected’’ in space and time. In a nested system, profoundchanges inkeyvariablesthatoperatenormallyonly at one level, e.g., within a defined geographic region or admin- istrative domain, can have non-linear outcomes for processes operating at broader scales of analysis (Gunderson andHolling, 2001).<br>(Eakin, Winkels, and Sendzimir 2009, 400)	No	Livelihood vulnerability; Nested Systems	
Nested system	Yes	In a nested system, profoundchanges inkeyvariablesthatoperatenormallyonly at one level, e.g., within a defined geographic region or admin- istrative domain, can have non-linear outcomes for processes operating at broader scales of analysis (Gunderson andHolling, 2001). Local level processes can episodically influence larger scale phenomena, and such explosive ‘‘upward cascades’’ can be sources of surprise at distant locations.<br>(Eakin, Winkels, and Sendzimir 2009, 400)	Yes		In the following sections, we use the case of the responses<br>of farmers in Vietnam and Mexico to the evolution of the global coffeemarket over the past three decades<br>[...]<br>(Eakin, Winkels, and Sendzimir 2009, 399)<br>The last two decades have witnessed a significant shift in the structure of the international coffee market, moving from a system of government-mediated market quotas to a neoliberalmodel, characterized by the elimination of barriers to trade. This precipitated an abrupt restructuring of the relationships

					between producing nations, traders and con- sumers, as well as between farmers and domestic institutions, all with direct implications for the livelihood security of coffee producers globally (Lewin et al., 2004). In 1989 the Interna- tional Coffee Agreement, which had operated a quota system to regulate coffee exports from the world's largest coffee producers, collapsed. In the absence of export restrictions, large amounts of coffee entered the market, inventories of green coffee in importing nations increased and, in the face of relatively stagnant demand, world coffee prices began a phase of steep decline (Ponte, 2002). Concurrent with the closure of the ICA, the market power of a handful of coffee traders and distributors increased (e.g., Proctor&Gamble, Nestle´ , and Sara Lee), concentrating profit in the coffee roasting and distribu- tion stage of the commodity chain (Lewin et al., 2004). The end of the 1980s and early 1990s alsomarked a period of transition in the domestic policies in many coffee- producing countries, inspired by a global shift in economic and political ideology. The end of the ColdWar and the rise of neoliberalism generated a shift in both the political motiva-

					tions behind economic policy intervention and the participa-tion of coffee-producing countries in global markets.<br>(Eakin, Winkels, and Sendzimir 2009, 401)
Response outcome	Yes	outcomes of these responses in terms of individual or household welfare.<br>(Eakin, Winkels, and Sendzimir 2009, 399)	Yes		The Mexican case study took place in 2003, as farmers were emerging from the most recent coffee crisis. The research took place in two coffee-producing communities in the region of Coatepec, in Central Veracruz. In addition to interviews with public officials, coffee association leaders, academics and coffee processors and traders, a household survey collected data on the perceptions and responses of 60 households to the coffee situation (Eakin et al., 2006). The project was part of a broader study exploring the implications of climatic variability and change for coffee farming in Mexico (Gay Garcia et al., 2006). In Vietnam, a study of migrant livelihoods included many<br>coffee farmers, mostly migrants to the Central Highland region (Winkels, 2004). Livelihood surveys and interviews with 81 households originating from the overpopulated Red River Delta in the north provides important insights into both the opportunities andrisk of coffee farming at Vietnam's southern

					mountain frontier when the first signs of the looming coffee crisis became evident in 2000 and 2001. (Eakin, Winkels, and Sendzimir 2009, 402)

**Article**: (Eakin et al. 2012)					
Construct	Defined ?	Definition or further info	Directly Operationalized?	Indirectly operationalized through:	Operational text
Adaptiveness	Yes	Conceptually, the process of household adaptation could be considered a function of the current state of the household (entitlements, assets, activities) and the biophysical, politi- cal, economic, institutional contexts in which decisions are made (determining the choice set for any household); the exposure and sensitivity of a household to stress and change; the decisions taken; and the outcome of those decisions. Adaptation is a decision process designed to ‘‘maintain capacities to deal with future change’’ and thus can involve actions that enhance adaptive capacities (Nelson et al. 2007). A household's experience of an environmental shock or change—how it copes with the event—may result in a rel- atively dramatic change in livelihood activities with poten- tially negative welfare outcomes (e.g., increased poverty) or, alternatively, may provide opportunities for learning and welfare improvements and thus enhanced adaptive capaci- ties (McSweeney and Coomes 2011)	No	Impacts & responses to Hurricane Stan by coffee farmers	

		(Eakin et al. 2012, 477)			
Disaster	No				
Impacts & responses to Hurricane Stan by coffee farmers	Yes	In this paper, we document household responses to a climatic shock, Stan, to gain insight into how natural resource-dependent communities move to secure their livelihoods following significant loss, the implications of household responses for coffee farming as a ‘‘domain of attraction,’’ as well as to highlight those aspects of household choices and perceptions that may be indicative of resilience at broader scales. (Eakin et al. 2012, 477)	yes		This study is based on 64 household surveys and additional in-depth expert and key-informant interviews, conducted in 2006 and 2007. The surveys, implemented 18 months following Stan, collected information regarding pre- and post-Hurricane Stan activities and income sources, house- hold demographics, land holdings, production attributes, hurricane impacts (to property, production and health and welfare), household assets before and after Stan and access to agricultural and emergency response services. As described later, the survey also captured households’ per- ceptions and attitudes about the disaster and their suscep- tibility to damage. Three of the most affected communities by Hurricane Stan in the municipio of Siltepec, Vega de Guerrero (pop. 410), Vicente Guerrero (pop. 151) and San Bartolo (pop. 185) were purposely selected for study on the basis of prior experience of one of the investigators in the region.1<br>(Eakin et al. 2012, 478)
Resilience	yes	A resilient system is one that maintains continued integrity of fundamental social–ecological services and functions under conditions of variability, surprise and stress (Carpenter et al. 2001; Folke et al. 2002). Learning, self- organization and adaptiveness have been proposed as core components of resilient communities. In this interpretation, adaptiveness refers to the ability of communities to ‘‘col- lectively manage	No	Adaptiveness	

		the resilience of the system'' (Walker et al. 2004) or, in other words, to actively manage how a system responds to change. Resilience is often evaluated with explicit reference to a desired state or (in less nor- mative terms) a ''domain of attraction'' (Gallopin 2006). A given system can have multiple domains of attraction, shifting states once thresholds are crossed. Resilience research seeks to understand the conditions in which thresholds are surpassed and shifts in state occur and strives to relate those conditions to specific human inter- ventions that facilitate or inhibit such shifts in state (Walker and Meyers 2004).<br>(Eakin et al. 2012, 477)			
Resilience of rural livelihoods	Yes	In the next section, we briefly review the related con-<br>cepts of resilience and vulnerability, focusing on an attri- bute central to the definition of both concepts: ''adaptiveness'' and ''adaptive capacity.'<br>(Eakin et al. 2012, 476)	No	Resilience; vulnerability	
Vulnerability	Yes	The concept of vulnerability is closely linked to that of<br>resilience; however, the concepts emerged from different disciplinary traditions and have distinct applications, with implications for the utility of these concepts for different units of analysis (Eakin and Luers 2006; Turner 2010). Vulnerability generally refers to the propensity of some unit of exposure to experience harm. In practice, house- holds are often a convenient unit of analysis for vulnera- bility assessments	No	Adaptiveness	

		that aim to differentiate a population in terms of sensitivity to a particular stressor and capacities to effectively respond (Eakin and Luers 2006). At the household level, vulnerability is often evaluated by assessing exposure (the physical relation of the household to a stressor) and sensitivities to the losses experienced (e.g., what the impact means for the household's function and survival), as well as by the households' ability to cope and adapt, or its ''adaptive capacity,'' prior to and after experiencing loss.<br>(Eakin et al. 2012, 477)			

Article: (Échevin 2011)

Construct	Defined?	Definition or further info	Directly Operationalized?	Indirectly operationalized through:	Operational text
Community level	Yes	an extension of this empirical framework will consist in using two-level (i.e. household and community levels) modelling of the impact of those shocks following Günther and Harttgen (2009)'s approach.<br>(Échevin 2011, 3)	Yes		Table 2 presents summary statistics for variables used in the analysis. Consumption and as<br>income are expressed in Gourdes. The agricultural index is a composite indicator which is a linear combination of categorical variables obtained from a multiple correspondence analysis (cf. Asselin, 2009). Variables considered in the analysis are the number of lands, animals and agricultural materials owned by the household. The community index is a linear combination of community basic infrastructure and access to market variables (roads, access to elementary or secondary schools, health centres, markets, electricity and cell phone). A score of income diversity has also been built from the various income sources earned by the household. As four main income sources are declared by the household, the income diversity variable (ID) is defined 4

					IDi =<br>2 1<br>□ −1 ∑ (s) □ , where k k 2<br>□ □<br>k =1 i<br>□ □<br>i s is the share of the kth income source in total income of<br>household i. This score equals 0 when only one source of income is declared by the household. It averages 0.17 in the studied population.<br>(Échevin 2011, 10)
Covariate shocks	No				
Determinants of poverty and vulnerability	No				
Economic well-being	Yes	In order to fully characterize the determinants of poverty and vulnerability in rural Haiti, a<br>unique survey can be used to assess the impact of idiosyncratic and covariate shocks on econ<br>(Échevin 2011, 3)	No	Household level; community level	
Household vulnerability to poverty	Yes	we can define vulnerability to poverty as the probability of falling into<br>poverty when one's consumption/income falls below a predefined poverty line.<br>(Échevin 2011, 5)	No	Economic well-being	
Household level	Yes	an extension of this empirical framework will consist in using two-level (i.e. household and	Yes		Table 2 presents summary statistics for variables used in the analysis. Consumption and as<br>income are expressed in Gourdes. The agricultural index

		community levels) modelling of the impact of those shocks following Günther and Harttgen (2009)'s approach. (Échevin 2011, 3)			is a composite indicator which is a linear combination of categorical variables obtained from a multiple correspondence analysis (cf. Asselin, 2009). Variables considered in the analysis are the number of lands, animals and agricultural materials owned by the household. The community index is a linear combination of community basic infrastructure and access to market variables (roads, access to elementary or secondary schools, health centres, markets, electricity and cell phone). A score of income diversity has also been built from the various income sources earned by the household. As four main income sources are declared by the household, the income diversity variable (ID) is defined 4<br>IDi =<br>2 1<br>[illegible] −1 ∑ (s) [illegible] , where k k 2<br>[illegible] [illegible]<br>k =1 i<br>[illegible] [illegible]<br>i s is the share of the kth income source in total income of<br>household i. This score equals 0 when only one source of income is declared by the household. It averages 0.17 in the studied population.<br>(Échevin 2011, 10)
Idiosyncratic shocks	No				
Observable covariate shocks	No				
Observable idiosyncratic shocks	No				
Poverty	Yes	In order to fully characterize the determinants of poverty and vulnerability in rural Haiti, a	No	Economic well-being	

		unique survey can be used to assess the impact of idiosyncratic and covariate shocks on economic well-being (Échevin 2011, 3)			
Unobservable covariate shocks	No				
unobservable idiosyncratic shocks	No				
vulnerability	Yes	Following Chaudhuri et al. (2002) or Christiaensen and Subbarao (2005), it will be possible to provide estimates of household vulnerability to poverty considering these various components (Échevin 2011, 3, 4)	No	Household vulnerability to poverty	

**Article**: (Ford and Smit 2004)					
Construct	Defined?	Definition or further info	Directly Operationalized?	Indirectly operationalized through:	Operational text
current adaptive capacity	Yes/no	Adaptive capacity refers to a community's potential or ability to address, plan for, or adapt to exposure (Smit and Pilifosova, 2003). Most communities can cope with normal climatic conditions and a range of deviations around norms. People have learned to modify their behaviour and their environment to manage and take advantage of their local climatic conditions (Jones and Boer, 2003). This ability to cope is referred to in the literature as the "coping range"; it	No	Current vulnerability	

		reflects resource use options and risk management strategies to prepare for, avoid or moderate, and recover from exposure effects (Hewitt and Burton, 1971; Smit et al., 1999; Jones, 2001; Smit and Pilifosova, 2003). Adaptive capacity relates to communities' resilience, resistance, flexibility, and ro- bustness (Smithers and Smit, 1997). It is influenced by economic wealth, social networks, infrastructure, social in- stitutions, social capital, experience with previous risk, the range of technological adaptation available, and equity of access to resources within the community, as well as by other stresses that contribute to the environment in which decisions are made (Adger and Kelly, 1999; Smit and Pilifosova, 2001; Smith et al., 2003).<br>(Ford and Smit 2004, 393)			
Current exposure		Exposure is a property of a community relative to climatic conditions. It reflects both the nature of the climatic conditions and nature of the community itself. Some communities may be exposed to a particular climate event whereas the same event may not affect another community. Climatic characteristics include magnitude, frequency, spatial dispersion, duration, speed of onset, and temporal spacing of climatic risks, relating to tem- peratures, precipitation, and wind. The nature of the com- munity concerns its location relative to the climatic risks<br>(Ford and Smit 2004, 393)	No	Current vulnerability	
Current		The assessment of current vulnerability	yes		experience, and the traditional and local

vulnerability		requires analyzing and documenting communities' experiences with climatic risks (current exposure) and the adaptive options and resource management strategies employed to address these risks (current adaptive capacity).<br>(Ford and Smit 2004, 395)			knowledge of community members (Inuit Qaujimajatuqangit) are cen- tral to assessing current vulnerability. Indigenous populations possess detailed knowledge of their environ- ment built up through personal observation and experience and from shared experience of members of the community (Duerden and Kuhn, 1998; Huntington, 1998; Usher, 2000). Knowledge about the environment and its use can be employed to identify and reconstruct events and condi- tions that represent climatic risks to the community and to provide insights into the resource-use options and risk- management strategies employed to prepare for, avoid or moderate, and recover from the effects of exposure. Such knowledge can be gained through several estab- lished ethnographic techniques, including focus groups, interviews, and participant observation. These techniques have been successfully used in research documenting indigenous observations on climate and environmental change throughout Arctic North America (Ferguson et al., 1998; Huntington, 1998; Krupnik and Jolly, 2002; DSD, 2003). Inuit Qaujimajatuqangit has also been documented to show how communities are adapting to changes and to identify adaptation needs (Fox, 2002; Nickels et al., 2002; DSD, 2003; Government of Nunavut, 2003). Information on risks and adaptation strategies can also be derived from content analysis of government reports, newspaper articles, Hudson Bay Company postal records, Distant Early Warning Site reports, and the insights of experienced land and

					resource use managers (Duerden, 2001). Solomon and Hart (1999) used Hudson Bay Com- pany postal records and ships' logbooks to examine storm frequency and severity in the Beaufort Sea. Fienup-Riordan (1999) used Catholic mission records and letters between government officials to assess the nature and impacts of a storm surge in 1931 in southwestern Alaska.<br>(Ford and Smit 2004, 396)
future adaptive capacity		Future adaptive capacity concerns the degree to which the community can deal with the estimated future exposures<br>(Ford and Smit 2004, 396)	yes		Future adaptive capacity concerns the degree to which the community can deal with the estimated future exposures. By examining past responses to climate variability and extremes and having the commu- nity identify its future adaptation options and constraints, researchers can characterize a community's ability to cope with future changes and collaborate to identify adaptive strategies that will reduce risk.<br>(Ford and Smit 2004, 396)
future climate probabilities	no				
future exposure		Future exposure also includes estimating the future state of the socioeco- nomic conditions, given that exposure is a property of the system relative to risk.<br>(Ford and Smit 2004, 396)	yes		Assessing future exposure involves collaboration with the climate science community to estimate the likelihood of changes in cli- matic attributes identified by the community. For exam- ple, will extreme events or climatic variability continue to increase? Will the unexpected winds that have caused problems to hunters in many Nunavut communities be- come even stronger and less predictable? Will the storm surges that have damaged infrastructure and sea defenses increase in magnitude or frequency? Which areas will

					experience most exposure to erosion? Future exposure also includes estimating the future state of the socioeco- nomic conditions, given that exposure is a property of the system relative to risk. (Ford and Smit 2004, 396)
future social probability	No				
Future vulnerability		Future vulnerability is assessed by analyzing how cli- mate change will alter the nature of the climate-related risks and whether the communities' coping strategies will have the capacity to deal with these risks. Assessing future exposure involves collaboration with the climate science community to estimate the likelihood of changes in cli- matic attributes identified by the community (Ford and Smit 2004, 396)	No	Future exposure; future adaptive capacity	
vulnerability to climate risks		The conceptual model of community vulnerability to climate change outlined here builds on the literature, conceptualizing vulnerability as a function of exposure of the community to climate-change effects and its adaptive capacity to deal with that exposure. (Ford and Smit 2004, 393) [...] A research framework for empirically applying the model of vulnerability proposed above to Arctic commu- nities is illustrated in Figure 3. The first stage assesses current vulnerability by documenting current exposures and current adaptive strategies. The second stage assesses future vulnerability by	No	Current vulnerability; future vulnerability	

		estimating directional changes in exposure and predicting future adaptive capacity on the basis of past behavior.<br>(Ford and Smit 2004, 395)			

**Article**: (Füssel and Klein 2006)					
Construct	Defined?	Definition or further info	Directly Operationalized?	Indirectly operationalized through:	Operational text
Adaptation	Yes	Adaptation: Adjustment in natural or human systems in response to actual or expected climatic stimuli or their effects, which moderates harm or exploits beneficial opportunities. Various types of adaptation can be distinguished, including anticipatory and reactive adaptation, private and public adaptation, and autonomous and planned adaptation. [...]<br>Adaptation to climate change, as defined by the IPCC, comprises a broad range of actions. Alternative definitions have sometimes restricted the use of this term to adjustments in social systems, to deliberate changes, to major structural changes in a system, or to a subset of climatic stimuli (Smit et al., 2000)<br>(Füssel and Klein 2006, 318)	not operationalized	[name of construct]	
Adaptatoin-Facilitation	Yes	Facilitation refers to activities that enhance adaptive capacity, such as scientific research, data collection, awareness raising, capacity building, and the establishment of institutions, information networks, and legal frameworks for action.<br>(Füssel and Klein 2006, 323)	not operationalized		
Adaptation-implmentation	Yes	Implemen- tation refers to activities that actually avoid adverse climate impacts on a sys- tem by reducing its exposure or sensitivity to climatic hazards, or by moderating relevant non-climatic factors (see Section 3.4 for examples).<br>(Füssel and Klein 2006, 323)	not operationalized		

Adaptive capacity	Yes	Adaptive capacity: The ability of a system to adjust to climate change (in- cluding climate variability and extremes) to moderate potential damages, to take advantage of opportunities, or to cope with the consequences.<br>(Füssel and Klein 2006, 319)	not operationalized		
Climate change	Yes	Climate change: A statistically significant variation in either the mean state of the climate or in its variability, persisting for an extended period (typically decades or longer). [...]<br>(Füssel and Klein 2006, 313)	not operationalized		
Climate variability	Yes	Climate variability: Variations in the mean state and other statistics (such as standard deviations, the occurrence of extremes, etc.) of the climate on all temporal and spatial scales beyond that of individual weather events. Vari- ability may be due to natural internal processes within the climate system (internal variability), or to variations in natural or anthropogenic external forcing (external variability).<br>(Füssel and Klein 2006) 316 (Füssel and Klein 2006, 316)	not operationalized		
concentrations	No				
Emissions	No				
Exposure	Yes	Exposure: The nature and degree to which a system is exposed to significant climatic variations.<br>The exposure of a system to climate stimuli depends on the level of global cli- mate change and, due to the spatial heterogeneity of anthropogenic climate change, on the system's location<br>(Füssel and Klein 2006, 313)	not operationalized		
Impacts	Yes	Impacts: Consequences of climate change on natural and human systems. Depending on the consideration of adaptation, one can distinguish between potential and residual impacts. [...]<br>(Füssel and Klein 2006, 314)	not operationalized		
Mitigation	Yes	Mitigation: An anthropogenic intervention to reduce the sources or enhance the sinks of greenhouse gases.<br>(Füssel and Klein 2006, 317)	not operationalized		
Mitigative capacity	Yes	The concept of mitigative capacity has been introduced into the literature only recently (Yohe, 2001). Mitigative	not operationalized		

		capacity is affected by various non-climatic factors. For instance, the effectiveness of a carbon trading scheme in reducing greenhouse gas emissions is partly determined by the presence and effectiveness of appropriate institutional arrangements in the respective region.<br>(Füssel and Klein 2006, 323)			
Mitigation Faciliation	Yes	The mitigative capacity of a region, sector, or other social unit may be enhanced by facilitation measures, such as the establishment of a carbon trading scheme.<br>(Füssel and Klein 2006, 323)	not operationalized		
Mitigation-implementation	Yes	An example for an implementation measure is the replacement of an old power plant by a less carbon-intensive one, which may have become economically viable due to the possibility for trading carbon permits.<br>(Füssel and Klein 2006, 323)	not operationalized		
Non-climatic drivers	Yes	non-climatic drivers (e.g., demographic, economic, sociopolitical, technological, and biophysical drivers). These drivers affect relevant non-climatic factors (e.g., the degree of economic diversification, the level of edu-cation, and the strength of social networks) that, in turn, determine the sensitivity of a system or community to climate change. In the context of climate change vul-nerability assessments, large-scale processes associated with global change, such as economic globalization and urbanization, are particularly important.<br>(Füssel and Klein 2006, 320)	not operationalized		
Non-climatic factors	Yes	Generic determinants of adaptive capacity in social systems comprise such non-climatic factors as economic resources, technology, information and skills, infrastructure, institutions, and equity (Smit and Pilifosova, 2001; Yohe and Tol, 2002).<br>(Füssel and Klein 2006, 320)	not operationalized		
Sensitivity	Yes	Sensitivity: The degree to which a system is affected, either adversely or beneficially, by climate-related stimuli. [...] The effect may be direct [...]or indirect [...]<br>[...]<br>The sensitivity of a system denotes the (generally multi-factorial and dynamic) dose – response relationship	not operationalized		

		between its exposure to climatic stimuli and the re- sulting impacts.<br>(Füssel and Klein 2006, 314)			
Vulnerability	Yes	Vulnerability: The degree to which a system is susceptible to, or unable to cope with, adverse effects of climate change, including climate variability and extremes. Vulnerability is a function of the character, magnitude, and rate of climate variation to which a system is exposed, its sensitivity, and its adaptive capacity.<br>(Füssel and Klein 2006, 306)	not operationalized		

**Article**: (Gandure, Walker, and Botha 2013)					
Construct	Defined?	Definition or further info	Directly Operationalized?	Indirectly operationalized through:	Operational text
Actual meterological observation	Yes	actual meteorological observations, rainfall and temperature data obtained from the South Africa Weather Services were analysed. Rainfall and air temperature are routinely measured at various stations distributed across South Africa, although not all districts have weather stations.<br>(Gandure, Walker, and Botha 2013, 42)	Yes		rainfall and temperature data obtained from the South Africa Weather Services were analysed. Rainfall and air temperature are routinely measured at various stations distributed across South Africa, although not all districts have weather stations. Rainfall data for our analysis was obtained from the station at Thaba Nchu; for temperature; the Bloemfontein station data was used due to lack of such data for Thaba Nchu. Temperature data for Bloemfontein provided a near representation of climate conditions in Thaba Nchu. Trends of the recorded rainfall and temperature data over the last 49 years (1960–2009) were analysed to determine how scientific observations and farmers' experiences interrelate and to understand the factors influencing community experiences.<br>(Gandure, Walker, and Botha 2013, 42)
Adaptation to long term	Yes	Unique in our study, is the use of individual perceptions in	Yes		Open ended questions were used to seek information on actions farmers take to adapt to

climate change		identifying and understanding the processes of adaptation in an area that has undergone significant political and socio-economic reformation resulting from a series of conflicts over land resources.<br>(Gandure, Walker, and Botha 2013, 40)			perceived<br>changes in temperature and rainfall and whether these actions were temporary or permanent. Firstly, farmerswere askedwhether they had changed theirway of life due to climate change. If the answerwas yes, then follow up questions of how they had changed andwhether they felt the changewas temporary or permanent were asked. If the answer was no, the reason(s) for not changing were then probed.<br>(Gandure, Walker, and Botha 2013, 42)
Climatic risk factors	No				
Experience of long term climate change	Yes	The study relied on the experience and knowledge of farmers and community members in<br>Gladstone to characterise their livelihood risks fromclimatic and non-climatic risk factors.<br>(Gandure, Walker, and Botha 2013, 41)	Not operationalized		
Livelihood risks	No				
Non-climatic risk factors	No				
Perception of long term climate change	Yes	Unique in our study, is the use of individual perceptions in identifying and understanding the processes of adaptation in an area that has undergone significant political and socio-economic reformation resulting from a series of conflicts over land resources.<br>(Gandure, Walker, and Botha 2013, 40)	Yes		Farmers' perceptions were sought by means of open ended questions on their observations/ experiences of long-term changes in temperature and/or rainfall. For temperature, farmers' opinions were sought on whether it has become warmer, cooler, more extreme, or no change noted. They could also report any other characteristics noted or say they did not know. Similarly, rainfall could be perceived as wetter, drier, more extreme, no change noted, other characteristics noted or admit to having no knowledge. Additional questions were asked on the manner in which changes occurred and farmers'

					perceptions of these changes.<br>(Gandure, Walker, and Botha 2013, 42)

**Article**: (Günther and Harttgen 2009)					
Construct	Defined?	Definition or further info	Directly Operationalized?	Indirectly operationalized through:	Operational text
Community level	Yes	Multilevel models are designed to analyze the relationship between variables that are measured at different hierarchical levels (for an introduction see, e.g., Bryk & Raudenbush, 1992; Goldstein, 1999; Hox, 2002). We speak of ‘‘hierarchical” or ‘‘multilevel” data structure whenever variables are collected at different hierarchical levels with lower-levels (e.g., house- holds) nested within higher-levels (e.g., communities).<br>(Günther and Harttgen 2009, 1225)	Yes		The community census is the 2001 ILO/Cornell Commune Level census which covers 1,385 of the 1,395 communities in Mada- gascar.<br>The community survey provides information on community characteristics such as social and economic infrastructure as well as data on the occurrence of some limited number of covariate shocks. More precisely, for each community and for the three years preceding the survey (2001, 2000, 1999) it is reported whether the community was exposed to any of 16 covariate shocks (most of these are reported in Tables A.1 and A.2 in Appendix). In many studies, the village has been used as the ‘‘natural” covariate level, but there is no necessity to do so (Genicot & Ray, 2003; Morduch, 2005), and using communities instead, as we do in this analysis, does not seem less useful.<br>(Günther and Harttgen 2009, 1227)
Covariate shocks	Yes	Households in developing countries are	yes		More precisely, for each

		frequently hit by se-<br>vere idiosyncratic and covariate shocks resulting in high income volatility. 1<br>(Günther and Harttgen 2009, 1222)<br>[...]<br>1. Here, and in the following, idiosyncratic shocks refer to household-specific shocks (e.g., injury, birth, death, or job loss of a household member) that are only weakly correlated across households within a community. Covariate shocks refer to shocks that are correlated across households within communities but only weakly correlated across communities (e.g., natural disasters or epidemics).<br>(Günther and Harttgen 2009, 1231)			community and for the three years preceding the survey (2001, 2000, 1999) it is reported whether the community was exposed to any of 16 covariate shocks (most of these are reported in Tables A.1 and A.2 in Appendix).<br>(Günther and Harttgen 2009, 1227)
Household level	Yes	Multilevel models are designed to analyze the relationship between variables that are measured at different hierarchical levels (for an introduction see, e.g., Bryk & Raudenbush, 1992; Goldstein, 1999; Hox, 2002). We speak of ''hierarchical" or ''multilevel" data structure whenever variables are collected at different hierarchical levels with lower-levels (e.g., house- holds) nested within higher-levels (e.g., communities).<br>(Günther and Harttgen 2009, 1225)	Yes		Data on household characteristics are taken from the na-<br>tional representative household survey of 2001 (Enqueˆte Aupre`s Des Me´nages), covering 5,080 households (1,778 ur- ban and 3,302 rural households) in 186 communities.<br>[...]<br>To estimate households' expected mean and variance in con-<br>sumption, we first use the household characteristics in Table 1. In addition, we consider an agricultural asset index (composed of eight productive assets) estimated via principal component analysis (Filmer & Pritchett, 2001). At the community level, we include population density, mean

					educational level, the per- centage of households working in the formal sector and the percentage of households possessing an enterprise within the community. Moreover, we construct an infrastructure index, again based on principal component analysis, using fourteen characteristics reflecting the infrastructure of the community (see Table A.4 in Appendix).<br>(Günther and Harttgen 2009, 1227)
Household vulnerability to poverty	Yes	The suggested approach is an integration of multilevel analysis (e.g., Goldstein, 1999) into Chaudhuri's (2002) method to estimate vulnerabil- ity<br>(Günther and Harttgen 2009, 1223)	No	Risk-induced vulnerability; household level	
Idiosyncratic shocks	Yes	Households in developing countries are frequently hit by se-<br>vere idiosyncratic and covariate shocks resulting in high income volatility. 1<br>(Günther and Harttgen 2009, 1222)<br>[...]<br>1. Here, and in the following, idiosyncratic shocks refer to household-specific shocks (e.g., injury, birth, death, or job loss of a household member) that are only weakly correlated across households within a community. Covariate shocks refer to shocks that are correlated across households within communities but only weakly correlated across communities (e.g., natural disasters or epidemics).	Yes		shocks (most of these are reported in Tables A.1 and A.2 in App<br>(Günther and Harttgen 2009, 1227)

		(Günther and Harttgen 2009, 1231)			
Risk-induced poverty	Yes	Here, and in the following, idiosyncratic shocks refer to household- specific shocks (e.g., injury, birth, death, or job loss of a household member) that are only weakly correlated across households within a community. Covariate shocks refer to shocks that are correlated across households within communities but only weakly correlated across communities (e.g., natural disasters or epidemics). (Günther and Harttgen 2009, 1231)	No	Idiosyncratic shocks; covariate shocks	
Structural poverty	Yes	Moreover, these poverty measures cannot assess whether high poverty rates are a cause of structural poverty (i.e., low endowments) or a cause of poverty risk (i.e., high uninsured income fluctuations), which is important to know from a policy perspective. (Günther and Harttgen 2009, 1222)	Yes		st, we decompose vulnerability estimates into the sources of vulnerability. We first analyze whether vulnerability is mainly driven by permanent low consumption prospects (i.e., structural or poverty induced vulnerability) or by high consumption volatility (i.e., transitory or risk induced vulner- ability). 18 In other words, if the (estimated) expected mean consumption ln^ a high estimated variance in consumption ^r2 mated vulnerability that is greater than the set vulnerability threshold of 0.29, then the household is said to face risk in- duced vulnerability (Figure 1) (Günther and Harttgen 2009, 1229)

**Article**: (Hahn, Riederer, and Foster 2009)					
Construct	Defined?	Definition or further info	Directly	Indirectly	Operational text

			Operationalized ?	operationalized through:	
2 week illness	Yes	Percentage of households that report at least 1 family member who had to miss school of work due to illness in the last 2 weeks. (Hahn, Riederer, and Foster 2009, 77)	Yes	[name of construct]	Has anyone in your family been so sick in the past 2 weeks that they had to miss work or schoo (Hahn, Riederer, and Foster 2009, 77)
Adaptive capacity	Yes	adaptive capacity is the system's ability to withstand or recover from the exposure (Ebi et al., 2006). (Hahn, Riederer, and Foster 2009, 75)	No	Socio-demographic profile; livelihood strategies; social network	
agriculture dependend households	Yes	Percentage of households that report only agriculture as a source of income. (Hahn, Riederer, and Foster 2009, 77)	Yes		Do you or someone else in your household raise animals? Do you or someone else in your household grow crops? Do you or someone else in your household collect something from the bush, the forest, or lakes and rivers to sell? (Hahn, Riederer, and Foster 2009, 77)
average precipitation	Yes	Standard deviation of the average monthly precipitation between 1998 and 2003 was averaged for each province (Hahn, Riederer, and Foster 2009, 79)	Yes		1998–2003: provincial data; weather station based in the provincial capital (Hahn, Riederer, and Foster 2009, 79)
borrow-lend ratio	Yes	Ratio of a household borrowing money in the past month to a household lending money in the past month, e.g., If a household borrowed money but did not lend money, the ratio = 2:1 or 2 and if they lent money but did not borrow any, the ratio = 1:2 or 0.5. (Hahn, Riederer, and Foster 2009) (Hahn, Riederer, and Foster 2009, 78)	Yes		Did you borrow any money from relatives or friends in the past month? Did you lend any money to relatives or friends in the past month? (Hahn, Riederer, and Foster 2009) (Hahn, Riederer, and Foster 2009, 78)
crop diversity	Yes	The inverse of (the number of crops grown by a household +1). e.g., A household that grows pumpkin, maize,	Yes		What kind of crops does your household grow? (Hahn, Riederer, and Foster 2009)

		nhemba beans, and cassava will have a Crop Diversity Index = 1/(4 + 1) = 0.20. (Hahn, Riederer, and Foster 2009) (Hahn, Riederer, and Foster 2009, 78)			(Hahn, Riederer, and Foster 2009, 78)
dependency ratio	Yes	Ratio of the population under 15 and over 65 years of age to the population between 19 and 64 years of age. (Hahn, Riederer, and Foster 2009, 77)	Yes		Could you please list the ages and sexes of every person who eats and sleeps in this house? If you had a visitor who ate and slept here for the last 3 days, please include them as well. (Hahn, Riederer, and Foster 2009, 77)
don't save crops	Yes	Percentage of households that do not save crops from each harvest. (Hahn, Riederer, and Foster 2009) (Hahn, Riederer, and Foster 2009, 78)	Yes		Does your family save some of the crops you harvest to eat during a different time of year? (Hahn, Riederer, and Foster 2009) (Hahn, Riederer, and Foster 2009, 78)
don't save seeds	Yes	Percentage of households that do not have seeds from year to year. (Hahn, Riederer, and Foster 2009) (Hahn, Riederer, and Foster 2009, 78)	Yes		Does your family save seeds to grow the next year? (Hahn, Riederer, and Foster 2009)
Exposure	Yes	Exposure in this case is the magnitude and duration of the climate-related exposure such as a drought or change in precipitation, (Hahn, Riederer, and Foster 2009, 75)	No	Natural disaster and climate change	
family with cronic illness	Yes	Percentage of households that report at least 1 family member with chronic illness. Chronic illness was defined subjectively by respondent. (Hahn, Riederer, and Foster 2009, 77)	Yes		Is anybody in your family chronically ill (they get sick very often)? (Hahn, Riederer, and Foster 2009, 77)
flood, drought, cyclone events	Yes	Total number of floods, droughts, and cyclones that were reported by households in the past 6 years. (Hahn, Riederer, and Foster 2009, 79)	Yes		How many times has this area been affected by a flood/cyclone/drought in 2001–2007? (Hahn, Riederer, and Foster 2009, 79)

Food	Yes	Food from family farm; struggle for food; crop diversity; dont save crops; dont save seeds<br>(Hahn, Riederer, and Foster 2009)<br>(Hahn, Riederer, and Foster 2009, 78)	No	Food from family farm; struggle for food; crop diversity; dont save crops; dont save seeds	
food from family farm	Yes	Percentage of households that get their food primarily from their personal farms<br>(Hahn, Riederer, and Foster 2009)<br>(Hahn, Riederer, and Foster 2009, 78)	Yes		Where does your family get most of its food?<br>(Hahn, Riederer, and Foster 2009)<br>(Hahn, Riederer, and Foster 2009, 78)
Health	Yes	Proximity to health facility; 2 weeks illness; malaria-exposure-prevention<br>(Hahn, Riederer, and Foster 2009, 77)	No	Family with chronic illness; proximity to health facility; 2 weeks illness; malaria exposure-prevention	
households with orphans	Yes	Percentage of households that have at least 1 orphan living in their home. Orphans are children<18 years old who have lost one or both parents.<br>(Hahn, Riederer, and Foster 2009, 77)	Yes		Are there any children less than 18 years old from other families living in your house because one or both of their parents has died?<br>(Hahn, Riederer, and Foster 2009, 77)
households working elsewhere	Yes	Percentage of households that report at least 1 family member who works outside of the community for their primary work activity<br>(Hahn, Riederer, and Foster 2009, 77)	Yes		How many people in your family go to a different community to work?<br>(Hahn, Riederer, and Foster 2009, 77)
idendependent of local government	Yes	Percentage of households that reported that they have not asked their local government for any assistance in the past 12 months.<br>(Hahn, Riederer, and Foster 2009)<br>(Hahn, Riederer, and Foster 2009, 78)	Yes		In the past 12 months, have you or someone in your family gone to your community leader for help<br>(Hahn, Riederer, and Foster 2009)<br>(Hahn, Riederer, and Foster 2009, 78)
inconsistent water suply	Yes	Percentage of households that report that water is not available at their primary water source everyday<br>(Hahn, Riederer, and Foster 2009, 79)	Yes		Is this water available everyday?<br>(Hahn, Riederer, and Foster 2009, 79)
injury or death from	Yes	Percentage of households that reported	Yes		Was anyone in your family injured

disaster		either an injury to or death of one of their family members as a result of the most severe flood, drought, or cyclone in the past 6 years.<br>(Hahn, Riederer, and Foster 2009, 79)			in the flood/cyclone drought? Did anyone in your family die during the flood/cyclone/drought?<br>(Hahn, Riederer, and Foster 2009, 79)
inverse water stored	Yes	The inverse of (the average number of liters of water stored by each household + 1).<br>(Hahn, Riederer, and Foster 2009, 79)	Yes		What containers do you usually store water in? How many? How many liters are they?<br>(Hahn, Riederer, and Foster 2009, 79)
livelihood diversification	Yes	The inverse of (the number of agricultural livelihood activities +1) reported by a household, e.g., A household that farms, raises animals, and collects natural resources will have a Livelihood Diversification Index = 1/(3 + 1) = 0.25.<br>(Hahn, Riederer, and Foster 2009, 77)	Yes		Do you or someone else in your household raise animals? Do you or someone else in your household grow crops? Do you or someone else in your household collect something from the bush, the forest, or lakes and rivers to sell?<br>(Hahn, Riederer, and Foster 2009, 77)
Livelihood strategies	Yes	Household working elsewhere; agriculture dependent households; livlihood diversification<br>(Hahn, Riederer, and Foster 2009, 77)	No	Households working elsewhere; agriculture dependent household; livelihood diversification	
Livelihood vulnerability	Yes	The LVI includes seven major components: Socio-Demographic Profile, Livelihood Strategies, Social Networks, Health, Food, Water, and Natural Disasters and Climate Variability<br>(Hahn, Riederer, and Foster 2009, 76)	No	Socio-demographic profile; livelihood strategies; social networks; health; food; water; natural disaster and climate change	
malaria exposure-prevention	Yes	Months reported exposure to malaria*Owning at least one bednet indicator (have bednet = 0.5, no bednet = 1) (e.g., Respondent reported malaria	Yes		Which months of the year is malaria particularly bad? How many mosquito nets do you have?<br>(Hahn, Riederer, and Foster 2009,

		is a problem January–March and they do not own a bednet = 3*1 = 3).<br>(Hahn, Riederer, and Foster 2009, 77)			77)
maximum temperature	Yes	Standard deviation of the average daily maximum temperature by month between 1998 and 2003 was averaged for each provinceb<br>(Hahn, Riederer, and Foster 2009, 79)	Yes		1998–2003: provincial data; weather station based in the provincial capital<br>(Hahn, Riederer, and Foster 2009, 79)
minimum temperature	Yes	Standard deviation of the average daily minimum temperature by month between 1998 and 2003 was averaged for each province.<br>(Hahn, Riederer, and Foster 2009, 79)	Yes		1998–2003: provincial data; weather station based in the provincial capital<br>(Hahn, Riederer, and Foster 2009, 79)
Natural disasters and Climate variability	Yes	Sub-constructs: flood, drought, cyclone events; no warning of disaster; injury or death from disaster; maximum temperature; minimum temperature; average percipitatoin<br>(Hahn, Riederer, and Foster 2009, 79)	No	Flood, drought, cyclone events; injury or death from disaster; no warning of disaster; maximum temperature; minimum temperature; average precipitation	
natural water source	Yes	Percentage of households that report a creek, river, lake, pool, or hole as their primary water source.<br>(Hahn, Riederer, and Foster 2009, 79)	Yes		Where do you collect your water from?<br>(Hahn, Riederer, and Foster 2009, 79)
no warning of disaster	Yes	Percentage of households that did not receive a warning about the most severe flood, drought, and cyclone event in the past 6 years.<br>(Hahn, Riederer, and Foster 2009, 79)	Yes		Did you receive a warning about the flood/cyclone/drought before it happened?<br>(Hahn, Riederer, and Foster 2009, 79)
precent of female-headed households	Yes	Percentage of households where the primary adult is female. If a male head is away from the home >6 months per year the female is counted as the head of the household	Yes		Are you the head of the household?<br>(Hahn, Riederer, and Foster 2009, 77)

		(Hahn, Riederer, and Foster 2009, 77)			
proximity to health facility	Yes	Average time it takes the households to get to the nearest health facility.<br>(Hahn, Riederer, and Foster 2009, 77)	Yes		Howlong does it take you to get to a health facility?<br>(Hahn, Riederer, and Foster 2009, 77)
proximity to water source	Yes	Average time it takes the households to travel to their primary water source.<br>(Hahn, Riederer, and Foster 2009, 79)	Yes		How long does it take to get to your water source?<br>(Hahn, Riederer, and Foster 2009, 79)
receive-give ratio	Yes	Ratio of (the number of types of help received by a household in the past month + 1) to (the number of types of help given by a household to someone else in the past month + 1).<br>(Hahn, Riederer, and Foster 2009)<br>(Hahn, Riederer, and Foster 2009, 78)	Yes		In the past month, did relatives or friends help you and your family: (e.g., Get medical care or medicines, Sell animal products or other goods produced by family, Take care of children) In the past month, did you and your family help relatives or friends: (same choices as above)<br>(Hahn, Riederer, and Foster 2009)<br>(Hahn, Riederer, and Foster 2009, 78)
Sensitivity	Yes	sensitivity is the degree to which the system is affected by the exposure<br>(Hahn, Riederer, and Foster 2009, 75)	No	Food; health; water	
social networks	Yes	Receive-give ration; borrow-lend ration; independent of local government<br>(Hahn, Riederer, and Foster 2009)<br>(Hahn, Riederer, and Foster 2009, 78)	No	Receive-give ration; borrow-lend ration; indenpendent of loval government	
Socio-demographic profile	Yes	Dependency ratio; female headed households; uneducated headed households; households with orphans<br>(Hahn, Riederer, and Foster 2009, 77)	No	Dependency ratio; percent of female headed households; households with orphans; uneducated headed households	
struggle for food	Yes	Average number of months households struggle to obtain food for their family.<br>(Hahn, Riederer, and Foster 2009)	Yes		Does your family have adequate food the whole year, or are there times during the year that your

		(Hahn, Riederer, and Foster 2009, 78)			family does not have enough food? Howmanymonths a year does your family have trouble getting enough food?<br>(Hahn, Riederer, and Foster 2009)<br>(Hahn, Riederer, and Foster 2009, 78)
uneducated headed households	Yes	Percentage of households where the head of the household reports that they have attended 0 years of school<br>(Hahn, Riederer, and Foster 2009, 77)	Yes		Did you ever go to school?<br>(Hahn, Riederer, and Foster 2009)
Vulnerability ipcc	Yes	Many of these rely heavily on the IPCC working definition of vulnerability as a function of exposure, sensitivity, and adaptive capacity (IPCC, 2001).<br>(Hahn, Riederer, and Foster 2009, 75)	No	Exposure; Sensitivity; Adaptive capacity	
Water	Yes	Sub-constructs: water conflict; natural water source; proximity to water source; inconsistent water supply; inverse water storage<br>(Hahn, Riederer, and Foster 2009, 79)	No	Water conflict; natural water source; proximity to water source; inconsistent water supply; inverse water stored	
water conflict	Yes	Percentage of households that report having heard about conflicts over water in their community<br>(Hahn, Riederer, and Foster 2009, 79)	Yes		In the past year, have you heard about any conflicts over water in your community?<br>(Hahn, Riederer, and Foster 2009, 79)

**Article**: (Ionesco et al. 2009)					
Construct	Defined?	Definition or further info	Directly Operationalized?	Indirectly operationalized through:	Operational text[29]

[29] For the purposes of illustration, Ionesco et al operationalized their framework on two different data sets. Therefore, 2 operationalizations are coded for each directly operationalizable construct

adaptive capacity	no				
adaptive capacity as set	Yes	Definition (Adaptive capacity as a set) The adaptive capacity of a system f in state x subjected to an input e is represented by the set of its effective actions.<br>(Ionesco et al. 2009, 9)	Yes		The adaptive capacity index can be seen within our framework as an estimate of the size of the set of available actions Uk. The socio-economic data used to derive the index (e.g.,GDPper capita, literacy rate and labour participation rate of women) indicate the capac- ity of society to prepare for and respond to impacts of global change by choosing an appropriate action (i.e., ecosystem management strategy). The size of this set of actions can be assumed to be an indication of the size of the set of effective actions, since the latter is a subset of the former.<br>(Ionesco et al. 2009, 13)<br>[...]<br>In contrast toATEAM,the transition function of the<br>coupled human–environment system was known and has the form of Eq. 19. In addition to the input, controls (i.e., adaptation actions) were included in the model. The actions contained in the set of controls U were (1) do nothing, (2) build dikes, (3) move away and (4) nourish the beach or tidal basins.<br>(Ionesco et al. 2009, 13)
effective action	Yes	Definition (Effective action) An action u is effective for a system f in state x subjected to an input e if not (f (x, e,u) ≺ f (x, e∗,u∗)).<br>(Ionesco et al. 2009, 9)	No	adaptive capacity as set	
Entity	Yes	The mainstream mathematical interpretation of an entity is that of a dynamical system in a given state. This is the interpretation we will adopt here	Yes		When taking a closer look at ATEAM using the<br>formal framework of Section 3, we first need to identify the framework's three

		(Ionesco et al. 2009, 4)			primitives. ATEAM aimed "to assess where in Europe people may be vulnerable to the loss of particular ecosystem services, associated with the combined effects of climate change, land use change and atmospheric pollution" ([22], p. 3). Thus, the entity is a coupled human–ecological system: the people in Europe who rely on ecosystem services. The system receives both input (the stimuli) and controls (the human actions). The evolution of such a system can be given by<br>xk+1 = f (xk, ek,uk), (19)<br>where k denotes the time step and uk is an element of the set of available controls Uk, which are the man- agement actions people can apply to adapt to poten- tial impacts and, thus, maintain the ecosystem services on which they rely. These actions are usually specific to the ecosystem service considered.<br>(Ionesco et al. 2009, 12)<br>[...]<br>The first primitive, the vulnerable entity, is the coastal system.<br>(Ionesco et al. 2009, 13)
hazard potential impact	Yes	Definition (Hazard, potential impact) An input e ∈ E is a hazard for a system f in state x if ∃ u ∈ U : f (x, e,u) ≺ f (x, e∗,u∗). In this case, f (x, e,u) is called a potential impact.<br>(Ionesco et al. 2009, 8)	Not operationalized		
preference criteria	Yes	Preference criteria are used to ascertain whether<br>or not a possible evolution of the entity is "bad" or "good". In the examples we have considered, we have seen that this judgment is usually made by	Yes		The third primitive notion concerns the preference<br>criteria represented by a (partial) strict order ≺,which relate to the loss of ecosystem services.We will discuss<br>the preference criteria in more detail

		comparison with a “normal” evolution, or an evolution under a “zero input”. (Ionesco et al. 2009, 5)			below.<br>(Ionesco et al. 2009, 12)<br>[...]<br>The third primitive, the partial strict order was given<br>in the form of an impact function on the set of states. The function computes additional diagnostic properties such as people at risk of flooding, land loss, economic damages and the cost of protecting the coast.<br>(Ionesco et al. 2009, 13)
reference scenarios	Yes	The examples provided also have this “punctual” or “one-step” character. However, in many applications, it is more natural to consider an evolution of the system to be a sequence of states, and to consider scenarios and reference scenarios instead of punctual inputs for the vulnerability assessment. A scenario is just a sequence of inputs: es =[e1,<br>e2,..., en]. Corresponding to such a sequence, the sys- tem will undergo n transitions, xs =[x0, x1,..., xn]<br>(Ionesco et al. 2009, 7)	Yes		To allow for such comparisons was one of the main objectives of ATEAM. Depending on the purposes of the assessment, the reference input could be chosen to be “no input”, that is, the next state was compared to the current one, or one of the other inputs prepared in accordance to the SRES scenarios.<br>(Ionesco et al. 2009, 12)
relative hazards	Yes	Definition (Relative hazard) An input e ∈ E is a rela- tive hazard for a system f in state x relative to an action<br>u ∈ U if f (x, e,u) ≺ f (x, e∗,u∗).<br>(Ionesco et al. 2009, 8)	Not operationalized		
Stimulus	Yes	The stimuli to which such a system can be subjected are then naturally represented by the inputs to the system. The simplest kind of dynamical system with input is a discrete, deter- ministic one, given by a transition function (see [14]):<br>f : X × E→ X, (1)	yes		The second primitive is the stimulus or input e ∈ E,<br>to which the system’s vulnerability was assessed. This input was given by the scenarios of climate, land use and nitrogen deposition, which represent the pos- sible evolutions of the environment. The scenarios were based on the IPCC SRES

		(Ionesco et al. 2009, 4)			storylines (for details, see [22]).<br>(Ionesco et al. 2009, 12)<br>[...]<br>The second primitive, the stimulus or input to which the entity's vulnerability was assessed, was given in the form of climate, land-use and socio-economic scenarios. Similar to ATEAM, these were developed on the basis of the IPCC SRES storylines.<br>(Ionesco et al. 2009, 13)
unavoidable hazards	Yes	Definition (Unavoidable hazard) An input e is an<br>unavoidable hazard for a system f in state x if $\forall$ u $\in$ U : f (x, e,u) $\prec$ f (x, e$*$,u$*$).<br>(Ionesco et al. 2009, 8)	Not operationalized		
Vulnerability	Yes	Definition (Vulnerability with a reference input) A system f : X × E→ X in state x is vulnerable to e with respect to the strict partial order $\prec$ and the reference input e$*$ if<br>f (x, e) $\prec$ f (x, e$*$)<br>[...]<br>$\prec$ and the reference scenario es$*$ $\in$ En if xs $\prec$ xs$*$<br>Definition (Vulnerability with a reference scenario)A system f : X × E→ X in state x is vulnerable to input scenario es $\in$ En with respect to the strict partial order<br>(8)<br>where xs and xs$*$ are the trajectories induced by the input scenario and reference scenario, respectively.<br>(Ionesco et al. 2009, 6)	No	Entity; stimulus; preference criteria	

**Article**: (Jamir et al. 2013)

Construct	Defined?	Definition or further info	Directly Operationalized?	Indirectly operationalized through:	Operational text
Adaptive capacity	Yes	Asper theIPCC's definition and framework, vulnerability is understood as a function of three components—exposure, sensitivity and adaptive capacity. Vulnerability is defined as ''the degree to which a system is susceptible to or unable to cope with, adverse effects of climate change, including cli- mate variability and extremes'' (IPCC 2001).<br>(Jamir et al. 2013, 154)	Yes		Household questionnaire surveys and participatory rural appraisal (PRA) were conducted in all the five villages in order to quantify each of these indicators. A total of 150 households (30 households in each village) were randomly selected across the villages for the household questionnaire survey.<br>[...]<br>(Jamir et al. 2013, 156)<br>Table 2 continued<br>(Jamir et al. 2013, 158)
Agricultural	Yes	On the lines of Patnaik and Narayanan (2009),<br>(Jamir et al. 2013, 156)	Yes		Table 3 Indicators of sources of vulnerability Indicators<br>Source of vulnerability<br>(Jamir et al. 2013, 159)
Biophysical	Yes	On the lines of Patnaik and Narayanan (2009),<br>(Jamir et al. 2013, 156)	Yes		Table 3 Indicators of sources of vulnerability Indicators<br>Source of vulnerability<br>(Jamir et al. 2013, 159)
Climate-relted extrement events	No				
Demographic	Yes	On the lines of Patnaik and Narayanan (2009),<br>(Jamir et al. 2013, 156)	Yes		Table 3 Indicators of sources of vulnerability Indicators<br>Source of vulnerability<br>(Jamir et al. 2013, 159)
Drought	Yes	The India Meteorological Department (IMD) defines drought as a rainfall deficit of 25 % or more from the district-level long-period average (LPA)<br>(Jamir et al. 2013, 154)	Yes		Table 2 Description and rationale for indicators selected for the vulnerability assessment<br>(Jamir et al. 2013, 157)
Exposure	Yes	Asper theIPCC's definition and framework, vulnerability	Yes		Household questionnaire surveys and participatory rural

		is understood as a function of three components—exposure, sensitivity and adaptive capacity. Vulnerability is defined as ''the degree to which a system is susceptible to or unable to cope with, adverse effects of climate change, including cli- mate variability and extremes'' (IPCC 2001).<br>(Jamir et al. 2013, 154)			appraisal (PRA) were conducted in all the five villages in order to quantify each of these indicators. A total of 150 households (30 households in each village) were randomly selected across the villages for the household questionnaire survey.<br>[...]<br>(Jamir et al. 2013, 156)<br>Exposure<br>Component indicators<br>Extreme climate events<br>Drought duration<br>Extent of dryland<br>(Jamir et al. 2013, 157)
Sensitivity	Yes	Asper theIPCC's definition and framework, vulnerability<br>is understood as a function of three components—exposure, sensitivity and adaptive capacity. Vulnerability is defined as ''the degree to which a system is susceptible to or unable to cope with, adverse effects of climate change, including cli- mate variability and extremes'' (IPCC 2001).<br>(Jamir et al. 2013, 154)	Yes		Household questionnaire surveys and participatory rural<br>appraisal (PRA) were conducted in all the five villages in order to quantify each of these indicators. A total of 150 households (30 households in each village) were randomly selected across the villages for the household questionnaire survey.<br>[...]<br>(Jamir et al. 2013, 156)<br><br>Table 2 Description and rationale for indicators selected for the vulnerability assessment<br>(Jamir et al. 2013, 157)
Socio-economic	Yes	On the lines of Patnaik and Narayanan (2009),<br>(Jamir et al. 2013, 156)	Yes		Table 3 Indicators of sources of vulnerability Indicators<br>Source of vulnerability<br>(Jamir et al. 2013, 159)
Sources of vulnerability	Yes	On the lines of Patnaik and Narayanan (2009),<br>(Jamir et al. 2013, 156)	No	Agricultural; biophysical; demographic;	

				socio-economic	
Village level	No				
Vulnerability	Yes/no	Asper theIPCC's definition and framework, vulnerability is understood as a function of three components—exposure, sensitivity and adaptive capacity. Vulnerability is defined as ''the degree to which a system is susceptible to or unable to cope with, adverse effects of climate change, including cli- mate variability and extremes'' (IPCC 2001). (Jamir et al. 2013, 154)	No	Exposure; sensitivity; adaptive capacity	

**Article**: (Khan and Salman 2012)					
Construct	Defined?	Definition or further info	Directly Operationalized?	Indirectly operationalized through:	Operational text
Coping capacity	Yes	Therefore we define vulnerability as damage potential and coping capacity, that is, damage potential + coping capacity = regional vulnerability (McCarthy et al. 2001; Mustafa 1998). (Khan and Salman 2012, 164)	No	population density; lack of decent housing; lack of decend standard of living; lack of knowledge; livestock households and farm households	
Damage potential	Yes	Therefore we define vulnerability as damage potential and coping capacity, that is, damage potential + coping capacity = regional vulnerability (McCarthy et al. 2001; Mustafa 1998). (Khan and Salman 2012, 164)	No	population density; lack of decent housing; lack of decend standard of living; lack of knowledge; livestock	

				households and farm households	
Lack of decent housing	Yes	(3) Lack of decent housing: Lack of access to a proper housing facility, as measured by the weighted average of two variables, percentage of population having kacha (weighted 3/6) and semi-pacca (weighted 1/6) houses, is linked closely to vulnerability.iv (Khan and Salman 2012, 165)	Yes		as measured by the weighted average of two variables, percentage of population having kacha (weighted 3/6) and semi-pacca (weighted 1/6) houses, (Khan and Salman 2012, 165)
Lack of decent standard of living	Yes	(4) Lack of decent standard of living: Lack of access to overall socioeconomic provisions is measured by the average of two variables: the percentage of the population without access to piped water and the percentage of population with- out access to electricity (Khan and Salman 2012, 165)	Yes		Lack of access to overall socioeconomic provisions is measured by the average of two variables: the percentage of the population without access to piped water and the percentage of population with- out access to electricity. (Khan and Salman 2012, 165)
Lack of knowledge	Yes	(2) Lack of knowledge: Exclusion from the world of read- ing and communications, as measured by the adult illiteracy rate, is an additional factor affecting increased vulnerability. The ability to read and write and language skills improve access to information. Access to information is particularly important in times of disasters. (Khan and Salman 2012, 165)	Yes		as measured by the adult illiteracy rate, (Khan and Salman 2012, 165)
Livestock households and farm households	Yes	Therefore, households depending on agriculture and livestock are the most direct victims of floods and are highly vulnerable. Arif, Iqbal, and Farooq (2010), using the 2000 Agriculture Census, classify rural households into	Yes		In making the human vulnerability index we used two variables: percent of households classified as farm households and percent of households classified as livestock households in each district.

		three broad categories: farm households that operate land as owner-cultivator or tenants; livestock households that have at least one cow or buffalo, 5 sheep and/or goats, and operate no farm area; and non-agriculture households that do not fall into farm and livestock household categories. In making the human vulnerability index we used two variables: percent of households classified as farm households and percent of households classified as livestock households in each district. (Khan and Salman 2012, 165)			(Khan and Salman 2012, 165)
Population density	Yes	(1) Population density: Vulnerability to the effects of cli- mate change consists of vulnerability to death, displacement, trauma, and loss of assets and livelihoods. This is measured by population density. (Khan and Salman 2012, 165)	Yes		This is measured by population density. (Khan and Salman 2012, 165)
Regional vulnerability	Yes	Therefore we define vulnerability as damage potential and coping capacity, that is, damage potential + coping capacity = regional vulnerability (McCarthy et al. 2001; Mustafa 1998). (Khan and Salman 2012, 164)	No	Damage potential; coping capacity	

**Article**: (Luers et al. 2003)					
<u>Construct</u>	<u>Defined?</u>	<u>Definition or further info</u>	<u>Directly Operationalized?</u>	<u>Indirectly operationalized through:</u>	<u>Operational text</u>
Adaptive capacity	Yes	We define adaptive capacityas the extent to which a	Yes	[name of construct]	Management is the onlyone of these factors that farmers can

		system can modify its circumstances to move to a less vulnerable condition (Fig. 1c). We quantifyadaptive capacity(A) as the difference in the vulnerabilityunder existing conditions and under the less vulnerable condition to which the system could potentially shift:<br>A ¼ Vðexisting conditionsÞ<br>Vðmodified conditionsÞ:<br>(Luers et al. 2003)(Luers et al. 2003, 259)			potentiallymanipulate to move to a less vulnerable condition. Therefore, in our analysis we estimate adaptive capacity from our time series of yields as the extent to which a farm unit has exceeded its average management percentile over the studyperiod. We assumed that the highest relative yield, as represented bythe yield percentile, could be achieved everyyear with the appropriate management. We estimate the adaptive capacityas the difference between the vulnerabilitycalculated as above and the vulner- abilitycalculated for a yield temperature function where we assume the expected yield is equal to the maximum yield percentiles observed over the four years. To create a unitless measure we normalize this difference bythe average value of the difference calculated for all pixels over the Valley:<br>A ¼ ðVR mean VR maxÞpixeli ðVR mean VR maxÞvalleyav<br>(Luers et al. 2003, 261)
Exposure	Yes	Different communities and ecosystems are exposed to varying magnitudes and frequencies of disturbing forces, often resulting in differential vulnerabilities (IPCC, 2001; Turner et al., 2003a, b). We capture these differences in exposure bycalculating the expected value of the ratio of sensitivityto the state relative to a threshold based on the frequencydistribution of the	Yes		For each of the four years, we compute the distribu-<br>tion of yield within the entire Valley, and then rank yields by percentile for each year. We then use a linear least-squares regression of yield with average night-time temperature for January–April to define the average yield and sensitivity for each percentile. To define the vulnerabilitycorresponding to each percentile, we run a Monte Carlo simulation where temperature varies according to a normal distribution with

		stressors of concern:<br>(Luers et al. 2003, 258)			mean equal to 9.61?C and standard deviation equal to 0.99?C, as determined from 20 years of historical climate records.<br>(Luers et al. 2003, 261)
Sensitivity	Yes	In this example, the sensitivityis represented as the absolute value of the derivative of well-being with respect to the stressor, however, other measures of sensitivitycould be used, for example the coefficient of variations.<br>(Luers et al. 2003, 258)	Yes		For each of the four years, we compute the distribu-<br>tion of yield within the entire Valley, and then rank yields by percentile for each year. We then use a linear least-squares regression of yield with average night-time temperature for January–April to define the average yield and sensitivity for each percentile. To define the vulnerabilitycorresponding to each percentile, we run a Monte Carlo simulation where temperature varies according to a normal distribution with mean equal to 9.61?C and standard deviation equal to 0.99?C, as determined from 20 years of historical climate records.<br>(Luers et al. 2003, 261)
State of system relative to threshold of damage	Yes	identifying a threshold of human well- being at which the system is said to be ‘‘damaged.’’<br>(Luers et al. 2003, 257)	Yes		Our unit (or system) of analysis is the ‘‘farm unit’’—<br>that is an agricultural field and the farmer or farmers responsible for the field. For practical purposes, we define our agricultural field as a 30m?30m pixel as described below.<br>(Luers et al. 2003, 260)
Threshold of damage	Yes	W0 represents a threshold value of well-being below which the system is said to be damaged<br>(Luers et al. 2003, 258)	Yes		a threshold value of 4 t/ha, which is the approximate minimum yield required for farmer’s to ‘‘break-even’’ (i.e. zero net profit) based on the average management practices (Matson et al. 1998).<br>(Luers et al. 2003, 261)

Vulnerability as suceptability	Yes	we derive a generic vulnerabilitymetric bytranslating a general definition of vulnerability, the susceptibilityto damage, into a mathematical expres- sion. To do this we first define a threshold of damage and then measure susceptibility in terms of the system's sensitivityto and exposure to stressors.We then propose a framework for estimating a system's ability to modify its vulnerable conditions byadapting and responding to changing circumstances.<br>(Luers et al. 2003, 257)	No	State of system relative to threshold; sensitivity; exposure; adaptive capacity	
Well-being	Yes	human–environment system where some mea- sure of human well-being (W)<br>(Luers et al. 2003, 257)	Yes		. Of the manyoutcomes of concern to the Valleyfarmer, we focus on wheat yield as our measure of well-being (Luers et al. 2003, 260)

**Article**: (Marshall 2010)					
Construct	Defined?	Definition or further info	Directly Operationalized?	Indirectly operationalized through:	Operational text
ability to plan, learn, reorganise	Yes	A description of each dimension can be found in Marshall and Marshall (2007). (Marshall 2010, 38)	Yes		3.3. Planning, learning and reorganising for climate variability On a scale of 1–4, the mean response of graziers to questions about planning was 2.93 (s.e. = 0.03). Graziers were confident that they had the skills to plan and prepare for drought. Only a few graziers (21.2%), said that they ''just hope for the best...if there is a drought'' and only 28.8% believed that the, ''future will look after itself.'' Most graziers (83.5%) said that, ''at the onset of drought [they]

					plan a way to survive it''. All the same, some 52% said that<br>(Marshall 2010, 39)
Adpative capacity	Yes	It refers to the ability of individuals or communities to adapt to adversity and stressful life-events by 'reorganising' through networks or institutions that learn, store knowledge and experi- ence and are creative, flexible and novel in their approach to problem solving (Vayda and McCay, 1975; McCay, 1981; Sonn and Fisher, 1998).<br>(Marshall 2010, 37)	Yes		Survey questions were developed so as to quantify a grazier's<br>capacity to adapt to climate variability, their level of dependency on the resource and their likely uptake of seasonal climate forecasts (Marshall, 2008). Some questions within the survey, such as 'in what year were you born?', required simple answers. Some questions such as, 'are you employed as a land manager on someone else's land?' required a 'yes' or 'no' answer. Answers to most questions, however, were expressed as a statement and reflected an attitude, opinion or stance.<br>(Marshall 2010, 38)
interest n change	Yes	A description of each dimension can be found in Marshall and Marshall (2007).<br>(Marshall 2010, 38)	Yes		3.5. Interest in adapting to climate variability The mean response to questions about the level of interest in<br>change was 2.89 (s.e. = .06) on a scale of 1–4. This result reflects that 83.5% were, ''interested in learning how [they] could better prepare for drought.'' Some graziers (60.4%), ''attend workshops to get new ideas to better manage drought'' and 71.5%, ''talk about strategies to survive drought with others''.<br>(Marshall 2010, 39)
perception of risk	Yes	A description of each dimension can be found in Marshall and Marshall (2007).<br>(Marshall 2010, 38)	Yes		3.2. Perception of risk associated with climate variability On a scale of 1–4, where any value greater than 2 is considered<br>to be a positive response, the mean response of graziers to survey questions about risk was 2.9 (standard error = 0.03). Graziers in the Burdekin region positively perceived the risks associated with

					drought, but not overly. For example, 90.1% of graziers believed that they were more ‘‘likely to survive drought compared to other cattle producers’’. Most graziers were more positive towards approaching drought periods than they had been in their past since 82.5% were, ‘‘.. learning to survive drought periods more easily as [they] got older’’. Some 90.6% felt that they were prepared to, ‘‘...take advantage of a particularly good season’’, suggesting that they felt positive about the future. More than half (56.9%) of the grazier population also disagreed with the sentiment that, ‘‘I am too young to retire and too old to find work elsewhere’’, suggesting that they felt positive as to their long-term business outcomes on the rangelands. Most graziers (81.4%) were not worried about the financial impacts of drought, since they had, ‘‘planned for [their] financial security in the event of a drought’’.<br>(Marshall 2010, 39)
proximity to coping threshold	Yes	A description of each dimension can be found in Marshall and Marshall (2007). (Marshall 2010, 38)	Yes		3.4. Ability to cope with climate variability<br>Overall, themean response to questions about copingwas 2.98<br>(s.e. = 0.03) on a scale of 1–4. Over 55% of graziers thought that, ‘‘the uncertainty surrounding drought is worse than the drought event itself’’,where 75.5% said that their familywas, ‘‘used to bad times and [they know they] will survive future drought.’’ Some 82.9% believed that their, ‘‘good years help [them] to survive the bad years’’. Whilst 82.9% suggested that their, ‘‘stress levels greatly increase in [their] family during drought periods’’, only 23.4%

					of graziers believed that, ‘‘my partner and I have different opinions about how to manage drought’’, and only 29.5% suggested that their, ‘‘current level of debt means that drought will be especially difficult to recover from.’’Many graziers (58.8%) disagreed that their, ‘‘financial situation is a constant source of worry.’’ Instead, most graziers (90.9%) saw, ‘‘climate uncertainty as a normal part of [their] everyday life’’, where 79.1% say that, ‘‘regardless of what happens.. .have made sure that [they] are financially secure.’’ Only 11.2% of graziers suggested that they, ‘‘rely on drought assistance to get [them] through drought years.’’ All the same, 50.8% of graziers said that, ‘‘it was important for [them] to know how other graziers are coping in their business.’’ Interestingly, if droughtdid force people off the land, only 54.2%of people said that they were, ‘‘interested in learning new skills outside of the industry’’.<br>(Marshall 2010, 39)
Resilience	No				
resource dependency	Yes	Resource-dependent communities such as cattle-grazing commu- nities are more likely to be vulnerable to climate change since climate change is likely to significantly affect the grazing resource and the people dependent on it. However, resource dependency is a complex relationship since it has social, economic and environmental components (Jones, 2002).<br>(Marshall 2010, 37)	Yes		Survey questions were developed so as to quantify a grazier’s<br>capacity to adapt to climate variability, their level of dependency on the resource and their likely uptake of seasonal climate forecasts (Marshall, 2008). Some questions within the survey, such as ‘in what year were you born?’, required simple answers. Some questions such as, ‘are you employed as a land manager on someone else’s land?’ required a ‘yes’ or ‘no’ answer. Answers to most questions, however, were expressed as a statement and reflected an

					attitude, opinion or stance.<br>(Marshall 2010, 38)
use of forecasts	Yes	Seasonal climate forecasts are an example of a supportive technology that can, with variable accuracy, provide probabilistic information about future climate for a period of three to twelve months (Ash et al., 2007; Jones et al., 2000; Tompkins and Adger, 2005). Climate technology may be able to assist graziers to minimise losses in drought years and take advantage of favourable seasons (Hayman et al., 2007; Salinger et al., 2005; Hansen, 2002; Eto, 2003; Moss, 2007).<br>(Marshall 2010, 37)	Yes		Survey questions were developed so as to quantify a grazier's<br>capacity to adapt to climate variability, their level of dependency on the resource and their likely uptake of seasonal climate forecasts (Marshall, 2008). Some questions within the survey, such as 'in what year were you born?', required simple answers. Some questions such as, 'are you employed as a land manager on someone else's land?' required a 'yes' or 'no' answer. Answers to most questions, however, were expressed as a statement and reflected an attitude, opinion or stance.<br>(Marshall 2010, 38)

**Article**: (Mengistu 2011)					
Construct	Defined?	Definition or further info	Directly Operationalized?	Indirectly operationalized through:	Operational text
Adaptation strategies	no		Yes/no/ not operationalized	[name of construct]	
Climate change	no				
Climate forecast methods	no				
Coping strategies	no				
Drought early warning systems	no				
Knowledge of farmers	no				
Perception of Adiha farmers	Yes	Adaptation of people to different hazards vary from household to households and region to region based on existing support system	Yes		Respondents were systematically sampled from Adiha<br>tabia populations across all of the kueshets. One hundred forty four (144) respondents

		to increase the resilience of affected individuals. The assessment was aimed to generate primary information from the farming communities of Adiha related to cli- mate change. This report examined the perception of Adiha farmers on the trend of climate change and re- lated anomalities, existing coping strategies in place.<br>(Mengistu 2011, 139)			were sampled from popula- tion of the tabia. Various factors including gender (male/ female headed farm households), age, access to irriga- tion water and land holding size were considered during sampling.<br>2.2.1. Focus Group Discussion (FGD) Focus Group Discussion (FGD) was employed to generate information on the perception of the farmers on climate change, its related hazards, vulnerable groups of the community and existing coping strategies. Six FGDs, each consisting 24 participants, 12 male and 12 women, drawn from different kueshets, were held for climate re- lated hazard identification and characterization, identifi- cation and prioritization of coping mechanisms, identifi- cation and ranking of vulnerable groups and climate and weather forecasting. Tools such as hazard identification and characterization, hazard behavior story telling (time- line), hazard ranking matrix, vulnerability group ranking and experiential stories telling on indigenous technolo- gies and knowledge were used to acquire information on farmers' perception on climate change trends, existing hazards and their severity and vulnerable groups of the community. The different coping strategies used by the community were also identified and analyzed for their effectiveness. Effectiveness was rated as very satisfac- tory, satisfactory and not satisfactory and the rating number converted to percent to assess satisfaction level.<br>Data Management and Analysis<br>(Mengistu 2011, 139)

**Article**: (Misselhorn 2005)

Construct	Defined?	Definition or further info	Directly Operationalized?	Indirectly operationalized through:	Operational text
Access to food	No		not operationalized	[name of construct]	
Access to sufficient food	No		not operationalized		
Direct drivers	No		not operationalized		
Food insecurity	Yes	Food insecurityin the communities described bythe case studies maybe conceptualized as one element in an entrenched and escalating cycle of vulnerability (Fig. 3). (Misselhorn 2005, 38)	not operationalized		
Food production	No		not operationalized		
Household and community vulnerability	Yes	In general terms, vulner- abilityand social resilience have been similarlydefined as the abilityof a system or communityto resist or absorb adverse conditions.<br>[...]<br>Vulnerable commu- nities, where people are unable to buffer themselves from hazards for a number of reasons, have a low ability to cope with short-term shocks (such as drought) and to mitigate chronic stressors, which in turn means that the negative impacts on livelihoods resulting from coping and survival strategies are veryhigh. (Misselhorn 2005, 38)	not operationalized		
Indirect drivers	No		not		

			operationalized		
Livelihood level issues	No		not operationalized		
Livelihood strategies	yes	A livelihood maybe described as the capability, assets and activites required for a means of living. People everywhere pursue a range of livelihood strategies in attempting to increase their income and asset base ('accumulation strategies'), spread or reduce risk (in- crease securitythrough 'adaptive strategies'), mitigate the impact of shocks ('coping strategies'), and at the extreme, ensure survival through 'survival strategies' (Devereux, 1999; Scoones, 2000).<br>(Misselhorn 2005, 38)	not operationalized		

**Article**: (Mubaya et al. 2012)					
Construct	Defined?	Definition or further info	Directly Operationalized?	Indirectly operationalized through:	Operational text
Climate change	Yes	In this paper, the distinction between 'climate variability' and 'climate change' relates to differences in time-scale. On the one hand, 'climate variability' is conceptualised as variations in the climate system over short time scales such as months, years or decades and on the other hand 'climate change' is conceptualised as longer term	Yes		FGDs were used to first of all establish the general perceptions regarding climate change and variability and their causes and various stressors that confront farmers' livelihoods (see Appendix 2).<br>[...]<br>The questionnaire survey was used to collect household data and complement data generated through the qualitative methods. This

		trends in mean climate variables of periods of decades or longer. This is the suggested distinction in definitions of the concepts in question by the IPCC (2001). (Mubaya et al. 2012, 10)			survey collected data on changes in crops grown over a period of five years and reasons for these changes, indicators for good and bad crop production seasons and years considered to be good or bad over a ten year period. Questions in the survey also related to changes in weather patterns over a ten year period in relation to agriculture and what might have caused these changes. General household characteristics were also captured in this survey (see Appendix 1).<br>(Mubaya et al. 2012, 11)
Climate change and variability	Yes	In this paper, the distinction between 'climate variability' and 'climate change' relates to differences in time-scale. On the one hand, 'climate variability' is conceptualised as variations in the climate system over short time scales such as months, years or decades and on the other hand 'climate change' is conceptualised as longer term trends in mean climate variables of periods of decades or longer. This is the suggested distinction in definitions of the concepts in question by the IPCC (2001). (Mubaya et al. 2012, 10)	Yes		FGDs were used to first of all establish the general perceptions<br>regarding climate change and variability and their causes and various stressors that confront farmers' livelihoods (see Appendix 2).<br>[...]<br>The questionnaire survey was used to collect household data<br>and complement data generated through the qualitative methods. This survey collected data on changes in crops grown over a period of five years and reasons for these changes, indicators for good and bad crop production seasons and years considered to be good or bad over a ten year period. Questions in the survey also related to changes in weather patterns over a ten year period in relation to agriculture and what might have caused these changes. General household characteristics were also

					captured in this survey (see Appendix 1).<br>(Mubaya et al. 2012, 11)
Climate variability	Yes	In this paper, the distinction between 'climate variability' and 'climate change' relates to differences in time-scale. On the one hand, 'climate variability' is conceptualised as variations in the climate system over short time scales such as months, years or decades and on the other hand 'climate change' is conceptualised as longer term trends in mean climate variables of periods of decades or longer. This is the suggested distinction in definitions of the concepts in question by the IPCC (2001).<br>(Mubaya et al. 2012, 10)	Yes		FGDs were used to first of all establish the general perceptions<br>regarding climate change and variability and their causes and various stressors that confront farmers' livelihoods (see Appendix 2).<br>[...]<br>The questionnaire survey was used to collect household data<br>and complement data generated through the qualitative methods. This survey collected data on changes in crops grown over a period of five years and reasons for these changes, indicators for good and bad crop production seasons and years considered to be good or bad over a ten year period. Questions in the survey also related to changes in weather patterns over a ten year period in relation to agriculture and what might have caused these changes. General household characteristics were also captured in this survey (see Appendix 1).<br>(Mubaya et al. 2012, 11)
Farmer perceptions	Yes	there is an alternative approach which underscores how individuals perceive their environment and make decisions, with mal-adaptations attributed to problems in perception, cognition or the lack of available information (Diggs, 1991; Saarinen, 1966; Taylor et	Yes		To understand farmers' perceptions of climate and non-climate<br>risks, this study employed both qualitative and quantitative methodologies. The qualitative methods of data collection used include Participatory Rural Appraisal (PRA) techniques such as historical trend analysis and matrix scoring and ranking

		al., 1988). The main point is that from whatever level these adapta- tion measures are taken, the adaptation and coping measures depend on households' perceptions of extreme events and the problems associated with them (Davies, 1993).<br>(Mubaya et al. 2012, 10)			and Focus Group Discussions (FGDs). The quantitative method used is the household questionnaire survey. The sampling procedure and two approaches are presented in the following sections.<br>(Mubaya et al. 2012, 10)
Non-climatic stress	Yes	It is important to note though, that climate change amplifies already existing risks for farmers. This is the case as there are non-climatic risk factors such as economic instability, trade liberalisa- tion, conflicts and poor governance that may also be faced by farmers (Nyong and Niang-Diop, 2006). Other factors are impacts of diseases such as malaria and HIV and AIDS and lack of and limited access to climate and agricultural information (Gandure, 2005; Gandure and Marongwe, 2006). Africa is also characterised by institutional and legal frameworks that are, in some cases, insuffi- cient to deal with environmental degradation and disaster risks (Beg et al., 2002; Sokona and Denton, 2001).<br>(Mubaya et al. 2012, 10)	Yes		FGDs were used to first of all establish the general perceptions<br>regarding climate change and variability and their causes and various stressors that confront farmers' livelihoods (see Appendix 2).<br>[...]<br>The questionnaire survey was used to collect household data<br>and complement data generated through the qualitative methods. This survey collected data on changes in crops grown over a period of five years and reasons for these changes, indicators for good and bad crop production seasons and years considered to be good or bad over a ten year period. Questions in the survey also related to changes in weather patterns over a ten year period in relation to agriculture and what might have caused these changes. General household characteristics were also captured in this survey (see Appendix 1).<br>(Mubaya et al. 2012, 11)
Threat to livelihoods	No				

**Article**: (Mutsvangwa 2011)					
Construct	Defined?	Definition or further info	Directly Operationalized?	Indirectly operationalized through:	Operational text
Cereal production	Yes	Smallholder farmers in Zimbabwe commonly produce cereals such as maize, millet and sorghum; with maize being the staple food and most commonly grown cereal. The energy content of the three cereals is almost the same, with maize, millet and sorghum producing 358, 329 and 336 kilocalories per 100g of grain respectively (Leder, 2010). In this study maize, sorghum and millet produced by the household is added so as to determine how much per capita cereal is produced by the household.<br>[...]<br>In addition the Southern Africa Regional Poverty Network's (2003) report on the regional overview of the southern African food security crisis suggests that an average family of 6 people requires about 800 -1000kg annually of cereal to be food secure, which also suggests a per capita cereal requirement of approximately 165kg.<br>(Mutsvangwa 2011, 22)	Yes		In addition the Southern Africa Regional Poverty Network's (2003) report on the regional overview of the southern African food security crisis suggests that an average family of 6 people requires about 800 -1000kg annually of cereal to be food secure, which also suggests a per capita cereal requirement of approximately 165kg.<br>(Mutsvangwa 2011, 22)<br>[...]<br>Table 4: Data from the household questionnaires:<br>yields obtained;<br>(Mutsvangwa 2011, 40)
Climate change	No				
Expected food	Yes	Among other things, the	No	Welfare indicator;	

insecurity		vulnerability status of smallholder farmers in different locations will be influenced by the household’s ability to produce enough to ensure the household’s food security. (Mutsvangwa 2011)<br>(Mutsvangwa 2011, 21)		vulnerability threshold	
food insecurity	No				
small holder farmers	No				
Vulnerability	Yes	vulnerability as a starting point which focuses on the susceptibility of the household2 (Füssel., 2007). This study takes on the starting point interpretation, which takes the root problem as social vulnerability and examines the current vulnerability of the households as a measure of vulnerability to climate change. Households that are currently vulnerable to food insecurity will find it difficult to cope with adverse impacts of changes in climatic conditions. Thus measuring the likelihood of being food insecure provides a way to examine vulnerability to climate change.<br>(Mutsvangwa 2011, 2)<br>[...]<br>Vulnerability refers to the manner and degree to which a system is susceptible to conditions that negatively affect the well-being of the system. In		Climate change; small holder farmers; food insecurity	

		the climate change field, the IPCC Third Assessment Report defines vulnerability as “the degree to which a system is susceptible to, or unable to cope with, adverse effects of climate change, including climate variability and extremes” (McCarthy et al., 2001).<br>(Mutsvangwa 2011, 15)<br>[...]<br>The differences between these two interpretations of vulnerability are summarized in Table 1.<br>Vulnerability according to the end point interpretation represent the expected net impacts of a given level of global climate change, taking into account feasible adaptations. Vulnerability according to the starting point interpretation focuses on reducing internal socioeconomic vulnerability to any climatic hazard. This study takes on the starting point interpretation.<br>(Mutsvangwa 2011, 17)			
vulnerability threshold	Yes	The choice of the vulnerability threshold involves generating a sample that is classified into two groups, that is those that are vulnerable and those that are not vulnerable to food insecurity. It entails establishing a vulnerability threshold, such that a household is said to be	Yes	Yes	Thus a household is considered vulnerable food insecurity if the probability is equal or greater than 0.5 and less likely to be vulnerable to food insecurity if the probability is less than 0.5.<br>(Mutsvangwa 2011, 23)

		vulnerable if its vulnerability probability is greater or equal to v, i.e. vh ≥ v.<br>(Mutsvangwa 2011, 22)			
welfare indicator	Yes	This study uses the household's cereal production levels as a measure of welfare. Farmers in both Gweru and Lupane mainly depend on what they produce for household food security, thus what the<br>households produce is equated to consumptions levels for the household, in this study.<br>(Mutsvangwa 2011)<br>(Mutsvangwa 2011, 21)	No	Cereal production	

**Article**: (Nkondze, Masuku, and Manyatsi 2013)					
Construct	Defined?	Definition or further info	Directly Operationalized?	Indirectly operationalized through:	Operational text
Household vulnerability to climate change	No		Yes/no/ not operationalized	[name of construct]	
Factors affecting vulnerability	No				

**Article**: (Notenbaert et al. 2013)					
Construct	Defined?	Definition or further info	Directly Operationalized?	Indirectly operationalized through:	Operational text
Adaptive capacity	Yes/no	the risk response or the options that people have for managing these risks (Turner et al. 2003).<br>(Notenbaert et al. 2013, 460)	No	Response and management options	
Exposure		risks (or a chain of risky events) that	Yes		Differences in vulnerability, described as

		people confront in pursuit of their livelihoods, (Turner et al. 2003). (Notenbaert et al. 2013, 460)			outcomes of this exposure, are therefore attributed to differences in sensitivity and adaptive capacity only. (Notenbaert et al. 2013, 460)
Institutional environment		(Turner et al. 2003). (Notenbaert et al. 2013, 460)	Yes		As with the exposure, we therefore assume these are equal for all households in the same village. (Notenbaert et al. 2013, 462)
Livelihood assets		(Turner et al. 2003). (Notenbaert et al. 2013, 460)	Yes		The questionnaire was divided into the following five sections: (1) household composition, livelihood strategies and livestock assets; (2) household livestock ownership, herd dynamics and species; (3) livestock feeding tech- niques, management, products and markets, (4) welfare outcomes (income, food consumption and health) and (5) focused on the main concerns/challenges facing households and their coping strategies. In this last section, households were asked to list and rank their concerns and describe the coping strategies employed to counter these concerns. Furthermore, the households were asked to compare with other households (in the same village) the extent to which they have been coping. For each of the concerns they were facing, they were asked whether they had been coping either better than, worse than or similar to other households in their village. (Notenbaert et al. 2013) (Notenbaert et al. 2013, 461)
Livelihood strategies		(Turner et al. 2003). (Notenbaert et al. 2013, 460)	Yes		The questionnaire was divided into the following five sections: (1) household composition, livelihood strategies and livestock assets; (2) household livestock ownership, herd dynamics and species; (3) livestock feeding tech- niques,

					management, products and markets, (4) welfare outcomes (income, food consumption and health) and (5) focused on the main concerns/challenges facing households and their coping strategies. In this last section, households were asked to list and rank their concerns and describe the coping strategies employed to counter these concerns. Furthermore, the households were asked to compare with other households (in the same village) the extent to which they have been coping. For each of the concerns they were facing, they were asked whether they had been coping either better than, worse than or similar to other households in their village. (Notenbaert et al. 2013) (Notenbaert et al. 2013, 461)
Livelihoods		(Turner et al. 2003).<br>(Notenbaert et al. 2013, 460)	Yes		The questionnaire was divided into the following five sections: (1) household composition, livelihood strategies and livestock assets; (2) household livestock ownership, herd dynamics and species; (3) livestock feeding tech- niques, management, products and markets, (4) welfare outcomes (income, food consumption and health) and (5) focused on the main concerns/challenges facing households and their coping strategies. In this last section, households were asked to list and rank their concerns and describe the coping strategies employed to counter these concerns. Furthermore, the households were asked to compare with other households (in the same village) the extent to which they have been coping. For each of the concerns they were facing, they were asked whether they had been coping

					either better than, worse than or similar to other households in their village. (Notenbaert et al. 2013) (Notenbaert et al. 2013, 461)
Response and management options		(Turner et al. 2003). (Notenbaert et al. 2013, 460)	No	Livelihoods; livelihood assets; livelihood strategies; institutional environment	
Risks		(Turner et al. 2003). (Notenbaert et al. 2013, 460)	Yes		Differences in vulnerability, described as outcomes of this exposure, are therefore attributed to differences in sensitivity and adaptive capacity only. (Notenbaert et al. 2013, 460)
sensitivity		the sensitivity of the livelihood to these risks, (Turner et al. 2003). (Notenbaert et al. 2013, 460)	No	Risks; livelihoods	
Vulnerability		For the purpose of this paper, we work with the definition proposed by the Working Group II of the IPCC in the third assess- ment report. We will refer to (1) exposure to climate change impacts, (2) sensitivity to those impacts and (3) the capacity to cope with those impacts as the components of vulnerability. Vulnerability is thus comprised of risks (or a chain of risky events) that people confront in pursuit of their livelihoods, the sensitivity of the livelihood to these risks, the risk response or the options that people have for managing these risks and finally the outcomes that describe the loss in well-being (Turner et al. 2003). (Notenbaert et al. 2013, 460)	No	Exposure; sensitivity; adaptive capacity; vulnerability outcomes	
Vulnerability		the outcomes that describe the loss in	Yes		The questionnaire was divided into the

outcomes		well-being (Turner et al. 2003). (Notenbaert et al. 2013, 460)			following five sections: (1) household composition, livelihood strategies and livestock assets; (2) household livestock ownership, herd dynamics and species; (3) livestock feeding tech- niques, management, products and markets, (4) welfare outcomes (income, food consumption and health) and (5) focused on the main concerns/challenges facing households and their coping strategies. In this last section, households were asked to list and rank their concerns and describe the coping strategies employed to counter these concerns. Furthermore, the households were asked to compare with other households (in the same village) the extent to which they have been coping. For each of the concerns they were facing, they were asked whether they had been coping either better than, worse than or similar to other households in their village. (Notenbaert et al. 2013) (Notenbaert et al. 2013, 461)

**Article**: (Piya, Maharjan, and Joshi 2012)					
Construct	Defined?	Definition or further info	Directly Operationalized?	Indirectly operationalized through:	Operational text
Adaptive capacity	Yes/no	Adaptive capacity is the ability of a system to adjust to climate change including climate variability and extremes, to moderate the potential damage from it, to take advantage of its opportunities, or to cope with its consequences. Selection of indicators for adaptive capacity is based on the DFID sustainable livelihoods framework,	No	Livelihood assets	

		whereby adaptive capacity is taken to be a function of asset possession by the households (Jakobsen, 2011; Nelson, et al., 2010b).<br>(Piya, Maharjan, and Joshi 2012, 12)			
Exposure	Yes	Exposure is the nature and degree to which a system is exposed to significant climatic variations.<br>(Piya, Maharjan, and Joshi 2012, 11)	Yes		or this study, historical changes in climate variables and occurrence of extreme climatic events are taken as indicators of exposure (Table 1).<br>(Piya, Maharjan, and Joshi 2012, 12)
Financial capital	Yes	Ellis (2000) and DFID (1999)<br>(Piya, Maharjan, and Joshi 2012, 7)(Piya, Maharjan, and Joshi 2012)	Yes		Table 3. Indicators for adaptive capacity (Piya, Maharjan, and Joshi 2012, 14)(Piya, Maharjan, and Joshi 2012)
Human capital	Yes	Ellis (2000) and DFID (1999)<br>(Piya, Maharjan, and Joshi 2012, 7)(Piya, Maharjan, and Joshi 2012)	Yes		Table 3. Indicators for adaptive capacity (Piya, Maharjan, and Joshi 2012, 14)(Piya, Maharjan, and Joshi 2012)
Livelihood assets	Yes	The sustainable livelihoods approaches which views livelihood outcomes as a function of the ownership or access to livelihood assets is principally based on Nobel Laureate Amartya Sen's entitlements approach, where by households with sufficient range of entitlements, capabilities or assets have more choices of adopting strategies suitable to cope during the periods of adversities or minimize the associated risks (Jakobsen, 2011; Ludi & Slate, 2008). The lack of or limited access to livelihood assets increases the defenselessness or incapacity to avoid risks as well as increases the shocks and stresses to which an individual or household is exposed to (Shahbaz, 2008). On the other hand, households with diversified asset portfolio are more capable to reduce risks and to cope with or adapt to increased level of risks.	No	Physical capital; human capital; natural capital; financial capital; social capital	The first phase of the household survey was focused on collection of data related to demographics, livelihood assets (landholdings, livestock holdings, savings, loans, education, trainings, membership to CBOs, infrastructure, and physical assets), livelihood activities, income sources, and expenditures.<br>(Piya, Maharjan, and Joshi 2012, 10)

	Yes	Such households will have more options to substitute among alternative livelihood activities during the times of stress, thereby having more adaptive capacity. For instance, households with access to irrigation (physical assets) will face less risks of crop damage during droughts compared to those households depending entirely on rainfed agriculture. Similarly, households with higher savings (financial assets) or memberships in saving and credit institutions (social assets) have greater capability to minimize livelihood risks posed by crop failure due to bad weather. Finally, households having some non-farm sources in addition to farming will improve the adaptive capacity of the households against the climatic stresses through distribution of risks across various livelihoods sources.<br>(Piya, Maharjan, and Joshi 2012, 7)(Piya, Maharjan, and Joshi 2012)			
Local level vulnerability	Yes	Following the definition of vulnerability given by IPCC (2001), vulnerability in this<br>study is taken to be a function of exposure, sensitivity, and adaptive capacity.<br>(Piya, Maharjan, and Joshi 2012, 11)	No	Exposure; sensitivity; adaptive capacity	
Natural capital	Yes	Ellis (2000) and DFID (1999)<br>(Piya, Maharjan, and Joshi 2012, 7)(Piya, Maharjan, and Joshi 2012)	Yes		Table 3. Indicators for adaptive capacity (Piya, Maharjan, and Joshi 2012, 14)(Piya, Maharjan, and Joshi 2012)
Physical capital	Yes	Ellis (2000) and DFID (1999)<br>(Piya, Maharjan, and Joshi 2012, 7)(Piya, Maharjan, and Joshi 2012)	Yes		Table 3. Indicators for adaptive capacity (Piya, Maharjan, and Joshi 2012, 14)(Piya, Maharjan, and Joshi 2012)
Sensitivity	Yes	is the degree to which a system is	Yes		Livelihood impacts of climate related

		affected, either adversely or beneficially by climate-related stimuli.<br>(Piya, Maharjan, and Joshi 2012, 10)			disasters were taken as the sensitivity indicator following Daze, Ambrose, & Ehrhart (2009) and Marshall et al. (2009). Deaths of family members and loss of properties (viz. land, livestock, and crop) due to climate related disasters over the last ten years represent the sensitivity for the purpose of this study.<br>(Piya, Maharjan, and Joshi 2012, 12, 13)
Social capital	Yes	Ellis (2000) and DFID (1999)<br>(Piya, Maharjan, and Joshi 2012, 7)(Piya, Maharjan, and Joshi 2012)	yes		Table 3. Indicators for adaptive capacity<br>(Piya, Maharjan, and Joshi 2012, 14)(Piya, Maharjan, and Joshi 2012)

**Article**: (Sallu, Twyman, and Stringer 2010)					
Construct	Defined?	Definition or further info	Directly Operationalized?	Indirectly operationalized through:	Operational text
dynamic natural resource base	Yes	(see Sallu [2007] for a more detailed outline of the methodology and data)<br>(Sallu, Twyman, and Stringer 2010, 5)	Yes		Repeated vegetation and wild animal surveys were conducted before and after rains, and time-series sets of Landsat images and wild animal aerial count data records were collected from the Department of Surveys and Mapping and the Department of Wildlife and National Parks. Soil and climate data were collected from the Department of Surveys and Mapping and the Department of Meteorological Services, respectively (see Sallu [2007] for a more detailed outline of the methodology and data).<br>(Sallu, Twyman, and Stringer 2010, 5)
factors influencing resilience and vulnerability	Yes	Through comparative research we provide a rich contextual narrative and use it to explore those factors that in isolation and combination push livelihoods along particular	Yes		Methods used included oral histories and in-depth livelihood trajectory mapping exercises (n = 17), as well as household-level livelihood and resource use surveys (n = 98). These sought to identify the ways in

		"trajectories" towards vulnerability or resilience.<br>(Sallu, Twyman, and Stringer 2010, 2)			which households use their environment, how environmental changes (drought, land degradation, etc.) affect livelihood decisions, and how environmental factors interact with broader socioeconomic and political processes to determine resource use outcomes and impacts on livelihood systems.<br>(Sallu, Twyman, and Stringer 2010, 4, 5)
livelihood trajectories	Yes	Bagchi et al. (1998) use the term "livelihood trajectories" to describe and explain the direction and pattern of livelihoods of individuals or groups of people (e.g., households). A livelihood trajectory approach allows the examination of an individual household's "strategic behavior that is embedded in a historical repertoire, in social differentiation" (de Haan and Zoomers 2005), and in perceptions of risk. Such an approach is sensitive to life histories (an individual's own "story" of their changing livelihoods).<br>(Sallu, Twyman, and Stringer 2010, 2)	Yes		Methods used included oral histories and in-depth livelihood trajectory mapping exercises (n = 17), as well as household-level livelihood and resource use surveys (n = 98). These sought to identify the ways in which households use their environment, how environmental changes (drought, land degradation, etc.) affect livelihood decisions, and how environmental factors interact with broader socioeconomic and political processes to determine resource use outcomes and impacts on livelihood systems.<br>(Sallu, Twyman, and Stringer 2010, 4, 5)
resilience and vulnerability of rural livelihoods	yes	Fraser et al.'s (2010) vulnerability framework<br>(Sallu, Twyman, and Stringer 2010, 2)	No	Livelihood trajectories; factors influencing resilience and vulnerability	

**Article**: (Sarris and Karfakis 2010)					
Construct	Defined?	Definition or further info	Directly Operationalized?	Indirectly operationalized through:	Operational text
cash crop growing	No			[name of construct]	

households					
covariate shocks	Yes	The proposed methodology complements the applications by Chaudhuri. et. al. (2002) and Christiaensen and Subbarao (2005), through the inclusion of covariate risks (Sarris and Karfakis 2010, 3)	Yes		Shocks enumerated in the household survey fall into four broad categories: (1) climatic and agricultural, which includes drought, heavy rainfall, including flooding, hailstorm and major harvest losses due to pests; (2) health, comprising death of a household member and illness not resulting in death; (3) economic, including unemployment and negative price shocks; and (4) asset shocks, which include theft, loss of livestock, loss of land or eviction, and fire. Table 2 summarizes the incidence of shocks among cash and non-cash producing households in the two regions. (Sarris and Karfakis 2010, 12)
crop-growing households	No				
household consumption	Yes	consumption falling below a poverty threshold (Christiaensen and Subbarao 2004, Chaudhuri, et. al. 2002) (Sarris and Karfakis 2010, 4)	Yes		Table 1 presents the basic characteristics of rural households in the two regions in Tanzania for which we have data, as derived from the two surveys. (Sarris and Karfakis 2010, 10)
household socio-economic characteristics	No				
idiosyncratic shocks	Yes	Chistiaensen and Subbarao (2005) included covariate as well as idiosyncratic shocks (Sarris and Karfakis 2010, 6)	Yes		Shocks enumerated in the household survey fall into four broad categories: (1) climatic and agricultural, which includes drought, heavy rainfall, including flooding, hailstorm and major harvest losses due to pests; (2) health, comprising death of a household member and illness not resulting in death; (3) economic, including unemployment and negative price shocks; and (4) asset shocks, which include theft, loss of livestock, loss of land or eviction, and fire. Table 2 summarizes the incidence

					of shocks among cash and non-cash producing households in the two regions. (Sarris and Karfakis 2010, 12)
non-cash crop growing households	no				
Rural household vulnerability		Thus a household is said to be vulnerable to the outcome of a risk event, if it does not have sufficient resources to adequately contend with the risk event. In other words, the extent to which a household is vulnerable to a risk event, namely the extent to which the household can become and/or remain poor or food deprived, depends on the size of the risk event and how effective the household is in managing the risk event. (Sarris and Karfakis 2010, 1)<br>[...]<br>considers vulnerability as the probability of consumption falling below a poverty threshold (Christiaensen and Subbarao 2004, Chaudhuri, et. al. 2002),<br>(Sarris and Karfakis 2010, 4)	Not operationalized		

**Article**: (Sietz, Choque, and Lüdeke 2012)					
Construct	Defined?	Definition or further info	Directly Operationalized?	Indirectly operationalized through:	Operational text
access to food	Yes	Food security is often discussed in terms of four dimensions: food availability, access, stability of supply/ access and utilisation (FAO 2000).	not operationalized		

		(Sietz, Choque, and Lüdeke 2012, 490)			
adaptive capacity	Yes	the adaptive capacity of smallholders (the term as used in this study encompasses the coping capacity) describes the ability to adjust to weather extremes, manage damages or explore alternative livelihood opportunities.<br>(Sietz, Choque, and Lüdeke 2012, 490)	Yes		The ALTAGRO (2006) data base contains detailed<br>quantitative information for 527 smallholder households collected through household questionnaires. The data refer to the 2005/2006 agricultural campaign. Ten categories describe the smallholder households covering personal information about the family members (e.g. occupation, education level, age), production systems (e.g. crop and livestock assets, labour input, processing and commer- cialisation of produce), weather conditions, food reserves,<br>(Sietz, Choque, and Lüdeke 2012, 494)<br>[...]<br>The data given in Table 1 describe the attributes of 268<br>smallholder households located in our study region.<br>(Sietz, Choque, and Lüdeke 2012, 495)
cluster pattern analysis	Yes	Without such a pre-selection, alternative approaches investigate the structure of the data space spanned by selected vulnerability indicators using cluster analysis. They deliver useful insights into recurrent indicator com- binations based on similarities among units of analysis, in cases where such a grouping exists. For example, clustering revealed typical livelihood strategies employed by small- holders in Mexico and Botswana (Eakin 2005; Sallu et al. 2010).<br>(Sietz, Choque, and Lüdeke 2012, 492)	Yes		The cluster analysis was performed using a sequence of a common hierarchical and exchange algorithm, i.e., hclust and kmeans, using the statistics package R (MacQueen 1967; RDCT 2009). Based on stochastic initialisation, we calculated the reproducibility of partitions for a pre-given number of clusters to determine whether the algorithm detects stable or unstable (inappropriate) partitions.<br>(Sietz, Choque, and Lüdeke 2012, 498)
Exposure	Yes	expo- sure, sensitivity and	Yes		The climate exposure is determined by

		coping/adaptive capacity (IPCC 2007).<br>(Sietz, Choque, and Lüdeke 2012, 490)			precipitation and temperature conditions as main natural production factors. We refer to both the 2005/2006 and the preceding agri- cultural campaign. Weather conditions during these two campaigns influenced food production and available reserves in the campaign under investigation. Furthermore, we use a well-documented additional campaign to identify the conditions for drought and water stress. The necessary weather information is available in good quality for the 1996–2006 period for two stations located in Puno and Cabanillas (see Fig. 1). Table 2 shows the average pre- cipitation and temperature for both stations. ADDIN ZOTERO_ITEM {"citationID":"W8dCEV71","properties":{"formattedCitation":"{\\rtf (Sietz, Choque, and L\\uc0\\u252{}deke 2012)}","plainCitation":""},"citationItems":[{"id":676,"uris":["http://zotero.org/users/1986215/items/BS99PSWR"],"uri":["http://zotero.org/users/1986215/items/BS99PSWR"]}]} (Sietz, Choque, and Lüdeke 2012)<br>(Sietz, Choque, and Lüdeke 2012, 496)
food availability	Yes	Food security is often discussed in terms of four<br>dimensions: food availability, access, stability of supply/ access and utilisation (FAO 2000).<br>(Sietz, Choque, and Lüdeke 2012, 490)	No operationalized		
food security	Yes	Food security is often discussed in terms of four<br>dimensions: food availability, access, stability of supply/ access and utilisation (FAO 2000).	yes		For the outcome-oriented aspect of validation, we<br>assume that an increased purchase of food and fodder indicates damage since it forces the household to mobilise resources which

		(Sietz, Choque, and Lüdeke 2012, 490)			may have been earmarked for other pur- poses. We collected data on the purchase of food and fodder in 2005/2006 including monetary and in-kind exchange. The purchase was considered in relation to an average year to compare households in a standardised way. The average year indicates the necessary purchase which complements the household’s production and reserves to maintain the average nutritional status. We assume that changes in 2005/2006 were primarily caused by the iden- tified weather extremes given that the productive resources and agricultural management are relatively stable over time. As smallholders do not maintain records of their pur-<br>chase, the data collection drew on their memory recall. This approach provides good estimates in the absence of other reliable data sources, though some limitations need to be considered. Most importantly, this method does not account for memory biases. To reduce such biases, the survey referred to the purchase of a specific crop in a given year. Firstly, smallholders were asked to reflect on thecroptheyharvested last,startingwiththe previous campaign and successively moving backwards to the 2005/2006 campaign. ADDIN ZOTERO_ITEM {"citationID":"TmahHttY","properties":{"formattedCitation":"{\\rtf (Sietz, Choque, and L\\uc0\\u252{}deke 2012)}","plainCitation":""},"citationItems":[{"id":676,"uris":["http://zotero.org/users/1986215/items/BS99PSWR"],"uri":["http://zotero.org/users/1986215/items/BS99PSWR

					"]}]} (Sietz, Choque, and Lüdeke 2012)<br>(Sietz, Choque, and Lüdeke 2012, 499)
household characterisitcs	No				
Sensitivity	Yes	We consider the effects of weather disturbance on the agricultural systems as sensitivity.<br>(Sietz, Choque, and Lüdeke 2012, 490)	Yes		The ALTAGRO (2006) data base contains detailed<br>quantitative information for 527 smallholder households collected through household questionnaires. The data refer to the 2005/2006 agricultural campaign. Ten categories describe the smallholder households covering personal information about the family members (e.g. occupation, education level, age), production systems (e.g. crop and livestock assets, labour input, processing and commer- cialisation of produce), weather conditions, food reserves,<br>[...]<br>The data given in Table 1 describe the attributes of 268<br>smallholder households located in our study region. ADDIN ZOTERO_ITEM {"citationID":"OyC4m1MO","properties":{"formattedCitation":"{\\rtf (Sietz, Choque, and L\\uc0\\u252{}deke 2012)}","plainCitation":""},"citationItems":[{"id":676,"uris":["http://zotero.org/users/1986215/items/BS99PSWR"],"uri":["http://zotero.org/users/1986215/items/BS99PSWR"]}]} (Sietz, Choque, and Lüdeke 2012)<br>(Sietz, Choque, and Lüdeke 2012, 494)
Vulnerability	Yes	Climate vulnerability is considered as a function of expo- sure, sensitivity and coping/adaptive capacity (IPCC 2007).	No	Exposure; sensitivity; adaptive	

		(Sietz, Choque, and Lüdeke 2012, 490)		capacity; food security	
vulnerability creating mechanisms	No				
weather extremes	No				

**Article**: (Tesso, Emana, and Ketema 2012)					
Construct	Defined?	Definition or further info	Directly Operationalized?	Indirectly operationalized through:	Operational text
adaptive capacity	Yes	According to Füssel and Klein, the risk-hazard framework (biophysical approach) corresponds most closely to sensitivity in the IPCC ter- minology while the adaptive capacity (broader social development) is largely consistent with the socioeco- nomic approach [18].<br>[...]<br>In the framework, capacity is generated from the implementation of adaptation and mitigation intervene- tions [18].<br>(Tesso, Emana, and Ketema 2012, 873)	not operationalized		
Determinants of resilience	Yes	important determinants for resilience at household level in North Shewa zone of Ethiopia.<br>(Tesso, Emana, and Ketema 2012)(Tesso, Emana, and Ketema 2012, 872)	Yes		The independent variables included in the model were avail- ability of food stock(dummy), income diversification (number of enterprises), number of plots, number of de- pendent family members, age of household head (years), access to credit (dummy), social capital (number of in- stitutional involvement), area under perennial crops (ha), preparedness (dummy), propensity to invest on natural resources (percentage of area under conservation), pro- pensity to save

					(percentage of saving), access to irriga- tion (ha), geographic locations (dummy), etc. βs are pa- rameters estimated and Uij is the disturbance term<br>(Tesso, Emana, and Ketema 2012, 875)
Exposure	Yes	Furthermore, in the IPCC frame- work, exposure has an external dimension, whereas both sensitivity and adaptive capacity have an internal dimen- sion, which is implicitly assumed in the integrated vul- nerability assessment framework [13].<br>(Tesso, Emana, and Ketema 2012, 873)	not operationalized		
fast bouncing back	Yes	1) households that were fast in bouncing back; which means households that have gone back to their normal agricultural operation in the following production season;<br>(Tesso, Emana, and Ketema 2012, 874)	No	Household level resilience	
household level resilience	Yes	According to DFID, resil- ience at community level is explained as the ability of countries, communities and households to manage change, by maintaining or transforming living standards in the face of shocks or stresses—such as earthquakes, drought or violent conflict—without compromising their long- term prospects [10]. Similarly, resilience is the ability of a social or ecological system to absorb disturbances while retaining the same basic structure and ways of functioning, the capacity for self-organization, and the capacity to adapt to stress and change. This is a meas- urement of community's capacity to absorb external shocks. In the aftermath of occurrence of climate	Yes		In this research, a farmer is said to have fully bounced back, when it begins its lively- hood operation as time before the shock. The speed of bouncing back was measured by number of agricul- tural seasons taken to bounce back to their livelihood without external intervention by government or non- governmental organization.<br>(Tesso, Emana, and Ketema 2012, 874)

		change induced shocks, how do farmer bounce back to normal livelihood is about the resilience level of farming com- munity. A resilient community is able to respond to changes or stress in a positive way, and is able to main- tain its core functions as a community despite those stresses [11]. (Tesso, Emana, and Ketema 2012, 871, 872)			
household vulnerability to climate change	Yes	Therefore, vulner- ability is the degree to which a system is susceptible or unable to cope with the adverse effects of climate change, including climate variability and extremes. In this regard, vulnerability is a function of the character, magnitude, and rate of climate variation to which a system is ex- posed, its sensitivity, and its adaptive capacity [4]. (Tesso, Emana, and Ketema 2012) (Tesso, Emana, and Ketema 2012, 871)	Not operationalized		
moderate bouncing back	Yes	2) moderate in bouncing back; which means households which took one to two agricultural seasons to get back to normal operation as before the event; (Tesso, Emana, and Ketema 2012, 874)	No	Household level resilience	
Sensitivity	Yes	According to Füssel and Klein, the risk-hazard framework (biophysical approach) corresponds most closely to sensitivity in the IPCC ter- minology while the adaptive capacity (broader social development) is largely consistent with the socioeco- nomic approach [18]. (Tesso, Emana, and Ketema 2012, 873)	Not operationalized		
slow bouncing back	Yes	3) slow in bouncing back; which means households which were unable to	No	Household level resilience	

		bounce back within one to two agricultural seasons to their normal livelihood activities.<br>(Tesso, Emana, and Ketema 2012, 874)			

**Article**: (Westerhoff and Smit 2009)					
Construct	Defined?	Definition or further info	Directly Operationalized?	Indirectly operationalized through:	Operational text
adaptation strategy	Yes	Adaptations, or adaptive strategies, employed by individuals or groups are depicted as being mediated through their relative adaptive capacities, indicating that adaptations may or may not be accessed according to the distribution of various types of resources such as physical or social capital, as developed by Adger and Kelly (1999).<br>(Westerhoff and Smit 2009, 321)	Yes		This model of vulnerability was used as a guiding framework for the empirical assessment of the vulnerability of the case study community of Mimkyemfre in the Afram Plains (Kwahu North) district of Ghana. Current exposure-sensitivities, adaptive strategies and adaptive capacities of the community are documented and explained in order to provide a basis for understanding vulnerability to future changes in climate and other environments. These were determined using a community-based approach similar to those used by Burton et al. (2002), Ford and Smit (2004) and Schröter et al. (2005), in which the factors and forces relevant to the community vulnerability were sought via primary and secondary sources. A key element of the approach is to engage community members as necessary sources of information on the conditions to which they are exposed and how they are sensitive, the adaptive strategies they have employed, and the conditions that constrain or facilitate these strategies. This detailed analysis of current vulnerability identifies opportunities for adaptive interventions or initiatives, and provides a

					basis for estimating future vulnerabilities by extending processes of exposure-sensitivity and adaptive capacity, and by incorporating projections of future climate change and other conditions.<br>(Westerhoff and Smit 2009, 322)
Adaptive capacity	Yes	Adaptive capacity (broadly consistent with social resilience) is also reflective of both the natural resource base and the social, economic, cultural and political conditions that facilitate or constrain adaptations to changing environments.<br>(Westerhoff and Smit 2009, 321)	Yes		This model of vulnerability was used as a guiding framework for the empirical assessment of the vulnerability of the case study community of Mimkyemfre in the Afram Plains (Kwahu North) district of Ghana. Current exposure-sensitivities, adaptive strategies and adaptive capacities of the community are documented and explained in order to provide a basis for understanding vulnerability to future changes in climate and other environments. These were determined using a community-based approach similar to those used by Burton et al. (2002), Ford and Smit (2004) and Schröter et al. (2005), in which the factors and forces relevant to the community vulnerability were sought via primary and secondary sources. A key element of the approach is to engage community members as necessary sources of information on the conditions to which they are exposed and how they are sensitive, the adaptive strategies they have employed, and the conditions that constrain or facilitate these strategies. This detailed analysis of current vulnerability identifies opportunities for adaptive interventions or initiatives, and provides a basis for estimating future vulnerabilities by extending processes of exposure-sensitivity and adaptive capacity, and by incorporating projections of future climate

					change and other conditions.<br>(Westerhoff and Smit 2009, 322)
biophyisical conditions	No				
exposed and sensitive to climate change	Yes	People's exposures and sensitivities to external conditions are influenced by their occupancy and livelihood characteristics, and the nature and degree to which these are affected by the external stresses.<br>(Westerhoff and Smit 2009, 321)	Yes		This model of vulnerability was used as a guiding framework for the empirical assessment of the vulnerability of the case study community of Mimkyemfre in the Afram Plains (Kwahu North) district of Ghana. Current exposure-sensitivities, adaptive strategies and adaptive capacities of the community are documented and explained in order to provide a basis for understanding vulnerability to future changes in climate and other environments. These were determined using a community-based approach similar to those used by Burton et al. (2002), Ford and Smit (2004) and Schröter et al. (2005), in which the factors and forces relevant to the community vulnerability were sought via primary and secondary sources. A key element of the approach is to engage community members as necessary sources of information on the conditions to which they are exposed and how they are sensitive, the adaptive strategies they have employed, and the conditions that constrain or facilitate these strategies. This detailed analysis of current vulnerability identifies opportunities for adaptive interventions or initiatives, and provides a basis for estimating future vulnerabilities by extending processes of exposure-sensitivity and adaptive capacity, and by incorporating projections of future climate change and other conditions.<br>(Westerhoff and Smit 2009, 322)

local scale vulnerability	Yes	Vulnerability at a local scale is shown as nested within other scales, including the effects that broad-scale forces have on processes of local vulnerability and vice versa. The dynamic nature of vulnerability is indicated by the layers of the components of vulnerability and of the interacting biophysical and socioeconomic forces. (Westerhoff and Smit 2009, 322)	No	Adaptive capacity; Exposed and sensitive to climate change; adaptation strategy	
multiple underlying forces	Yes	n summary, research on practical adaptations to effectively address the vulnerability of people to climate change has recognized the need to identify the factors in addition to climate that contribute to vulnerability, including the multiple forces and dynamic processes that occur at both local and broader scales. (Westerhoff and Smit 2009, 320)	Yes		This model of vulnerability was used as a guiding framework for the empirical assessment of the vulnerability of the case study community of Mimkyemfre in the Afram Plains (Kwahu North) district of Ghana. Current exposure-sensitivities, adaptive strategies and adaptive capacities of the community are documented and explained in order to provide a basis for understanding vulnerability to future changes in climate and other environments. These were determined using a community-based approach similar to those used by Burton et al. (2002), Ford and Smit (2004) and Schröter et al. (2005), in which the factors and forces relevant to the community vulnerability were sought via primary and secondary sources. A key element of the approach is to engage community members as necessary sources of information on the conditions to which they are exposed and how they are sensitive, the adaptive strategies they have employed, and the conditions that constrain or facilitate these strategies. This detailed analysis of current vulnerability identifies opportunities for adaptive

					interventions or initiatives, and provides a basis for estimating future vulnerabilities by extending processes of exposure-sensitivity and adaptive capacity, and by incorporating projections of future climate change and other conditions. (Westerhoff and Smit 2009, 322)
socio-economic conditions	No				

Appendix D: Summary of article-specific constructs identified

Article	Constructs	Defined?	Directly operationalized?
(Antwi-Agyei et al. 2013)	Access to livelihood capital assets	Yes	Not operationalized
	Adaptive capacity	Yes	No
	Climatic risk	Yes	Not operationalized
	Community	Yes	Yes
	Diversified livelihood activities	Yes	Yes
	Drought	No	
	Exposure	Yes	Yes
	Financial capital	Yes	Yes
	Household	Yes	No
	Human Capital	Yes	Yes
	Livelihood capital assets	Yes	No
	Livelihoods	Yes	No
	Natural capital	Yes	Yes
	Physical capital	Yes	Yes
	Resilience	Yes	No
	Resilient and vulnerable communities	Yes	Yes
	Resilient and vulnerable households	No	
	Sensitivity	Yes	No
	Social capital	Yes	Yes
	Socio-economic, environmental, and community characteristics	No	
	Vulnerability	Yes	No
(Baca et al. 2014)	Adaptation strategies	No	
	Adaptive capacity	Yes	Yes
	Exposure	Yes	Yes
	Sensitivity	Yes	Yes
	Vulnerability of coffee farming communities	Yes	No
(Berkes and Ross 2013)	Adaptive capacity	Yes	not operationalized
	Agency	No	
	Community resilience	Yes	not operationalized
	Self-organising	No	
(Bogale, Taeb, and Endo 2006)	Common property	No	
	Household choice	No	
	Private property	No	
	Property rights	Yes	Not operationalized
	Property rights regime	Yes	Not operationalized
	Public property	No	
	Vulnerability	No	
(Calvo and Dercon 2013)	Aggregate vulnerability	Yes	No
	Covariant shocks	No	
	Idiosyncratic shocks	No	
	Individual vulnerability	Yes	No
	Possible states of the world	Yes	Yes

	Poverty line	Yes	Yes
	Probabilities of possible states of the world	Yes	Yes
	Shocks	No	
	Vulnerability to poverty	Yes	No
	vulnerability	Yes	No
(Capaldo et al. 2010)	Access to food	Yes	Not operationalized
	Chronically food insecure	Yes	No
	Current exposure to risk	YES	Yes
	Current socio-economic characteristics	YES	Yes
	Events	Yes	No
	Expected future food security status	Yes	No
	Food availability	Yes	Not operationalized
	Food consumption	Yes	Not operationalized
	Food security	Yes	No
	Food utilization	Yes	Not operationalized
	Future food security	Yes	Not operationalized
	Future nutritional status	Yes	Not operationalized
	Permanently food secure	Yes	No
	Present characteristics	Yes	No
	Present food security status	Yes	No
	Risk management	Yes	Yes
	Risks	Yes	Yes
	Transitory food insecure	Yes	No
	Transitory food secure	Yes	No
	Vulnerability	Yes	Not operationalized
	Vulnerability to future food insecurity	Yes	No
(CARE 2009)	Adaptation to climate change	Yes	Not operationalized
	adaptive capacity	Yes	Yes
	Climate change	Yes	Not operationalized
	community level	No	
	financial capital	No	
	Hazard	Yes	yes
	human capital	No	
	natural capital	No	
	physical capital	No	
	Resilience	Yes	Yes
	social cpaital	No	
	vulnerability to climate change	Yes	No
(Chhihn and Poch 2012)	Climate change	No	
	Current poverty status	Yes	Yes
	Environmental shocks	Yes	Yes
	Farmers	No	
	Household characteristics	Yes	Yes
	Household vulnerability as expected poverty	Yes	No
	Households	No	
	Natural hazards	No	
	Poverty	Yes	Yes
(Dasgupta and Baschieri 2010)	Asset vulnerability	Yes	no
	Climate shocks	Yes	No

	Communities	No	
	Communities at risk of climate shocks	No	
	Drought	Yes	Yes
	Household relations	Yes	Not operationalized
	Household vulnerability to climate change	Yes	No
	Human capital	Yes	Yes
	Labour	Yes	Yes
	Non-labour productive assets	Yes	Yes
	Prepared for adverse consequences	No	
	Risk of experiencing climate change shock	Yes	Yes
	Social capital	Yes	Yes
	Welfare of rural households	No	
(Deressa, Hassan, and Ringler 2009)	Climate and non-climate shocks	No	
	Ethiopean Farmers	No	
	Expected Poverty	Yes	No
	Household consumption (income)	No	
	Minimum consumption (income) level	Yes	Yes
	Vulnerability	Yes	No
(Eakin, Winkels, and Sendzimir 2009)	Cross- scalar teleconnection	Yes	No
	Exogenous drivers	Yes	Yes
	Geographically distant household vulnerability	Yes	Yes
	Geographically specific signals of change	Yes	Yes
	Household responses	Yes	Yes
	Livelihood vulnerability	Yes	No
	Nested and teleconnected livelihood vulnerability	Yes	No
	Nested system	Yes	Yes
	Response outcome	Yes	Yes
(Eakin et al. 2012)	Adaptiveness	Yes	No
	Disaster	No	
	Impacts & responses to Hurricane Stan by coffee farmers	Yes	yes
	Resilience	Yes	No
	Resilience of rural livelihoods	Yes	No
	Vulnerability	Yes	No
(Échevin 2011)	Community level	Yes	Yes
	Covariate shocks	No	
	Determinants of poverty and vulnerability	No	
	Economic well-being	Yes	No
	Household vulnerability to poverty	Yes	No
	Household level	Yes	Yes
	Idiosyncratic shocks	No	
	Observable covariate shocks	No	
	Observable idiosyncratic shocks	No	
	Poverty	Yes	No

	Unobservable covariate shocks	No	
	unobservable idiosyncratic shocks	No	
	vulnerability	Yes	No
(Ford and Smit 2004)	current adaptive capacity	Yes	No
	Current exposure	Yes	No
	Current vulnerability	Yes	yes
	future adaptive capacity	Yes	yes
	future climate probabilities	No	
	future exposure	Yes	yes
	future social probability	No	
	Future vulnerability	Yes	No
	vulnerability to climate risks	Yes	No
(Füssel and Klein 2006)	Adaptation	Yes	not operationalized
	Adaptatoin-Facilitation	Yes	not operationalized
	Adaptation-implmentation	Yes	not operationalized
	Adaptive capacity	Yes	not operationalized
	Climate change	Yes	not operationalized
	Climate variability	Yes	not operationalized
	concentrations	No	
	Emissions	No	
	Exposure	Yes	not operationalized
	Impacts	Yes	not operationalized
	Mitigation	Yes	not operationalized
	Mitigative capacity	Yes	not operationalized
	Mitigation Faciliation	Yes	not operationalized
	Mitigation-implementation	Yes	not operationalized
	Non-climatic drivers	Yes	not operationalized
	Non-climatic factors	Yes	not operationalized
	Sensitivity	Yes	not operationalized
	Vulnerability	Yes	not operationalized
(Gandure, Walker, and Botha 2013)	Actual meterological observation	Yes	Yes
	Adaptation to long term climate change	Yes	Yes
	Climatic risk factors	No	
	Experience of long term climate change	Yes	Not operationalized
	Livelihood risks	No	
	Non-climatic risk factors	No	
	Perception of long term climate change	Yes	Yes
(Günther and Harttgen 2009)	Community level	Yes	Yes
	Covariate shocks	Yes	yes
	Household level	Yes	Yes
	Household vulnerability to poverty	Yes	No
	Idiosyncratic shocks	Yes	Yes
	Risk-induced poverty	Yes	No
	Structural poverty	Yes	Yes
(Hahn, Riederer, and Foster 2009)	2 week illness	Yes	Yes
	Adaptive capacity	Yes	No
	agriculture dependend households	Yes	Yes
	average precipitation	Yes	Yes
	borrow-lend ratio	Yes	Yes
	crop diversity	Yes	Yes
	dependency ratio	Yes	Yes

	don't save crops	Yes	Yes
	don't save seeds	Yes	Yes
	Exposure	Yes	No
	family with cronic illness	Yes	Yes
	flood, drought, cyclone events	Yes	Yes
	Food	Yes	No
	food from family farm	Yes	Yes
	Health	Yes	No
	households with orphans	Yes	Yes
	households working elsewhere	Yes	Yes
	idendependent of local government	Yes	Yes
	inconsistent water suply	Yes	Yes
	injury or death from disaster	Yes	Yes
	inverse water stored	Yes	Yes
	livelihood diversification	Yes	Yes
	Livelihood strategies	Yes	No
	Livelihood vulnerability	Yes	No
	malaria exposure-prevention	Yes	Yes
	maximum temperature	Yes	Yes
	minimum temperature	Yes	Yes
	Natural disasters and Climate variability	Yes	No
	natural water source	Yes	Yes
	no warning of disaster	Yes	Yes
	precent of female-headed households	Yes	Yes
	proximity to health facility	Yes	Yes
	proximity to water source	Yes	Yes
	receive-give ratio	Yes	Yes
	Sensitivity	Yes	No
	social networks	Yes	No
	Socio-demographic profile	Yes	No
	struggle for food	Yes	Yes
	uneducated headed households	Yes	Yes
	Vulnerability ipcc	Yes	No
	Water	Yes	No
	water conflict	Yes	Yes
(Ionesco et al. 2009)	adaptive capacity	No	
	adaptive capacity as set	Yes	Yes
	effective action	Yes	No
	Entity	Yes	Yes
	hazard potential impact	Yes	Not operationalized
	preference criteria	Yes	Yes
	reference scenarios	Yes	Yes
	relative hazards	Yes	Not operationalized
	Stimulus	Yes	yes
	unavoidable hazards	Yes	Not operationalized
	Vulnerability	Yes	No
(Jamir et al. 2013)	Adaptive capacity	Yes	Yes
	Agricultural	Yes	Yes
	Biophysical	Yes	Yes
	Climate-relted extrement events	No	
	Demographic	Yes	Yes

	Drought	Yes	Yes
	Exposure	Yes	Yes
	Sensitivity	Yes	Yes
	Socio-economic	Yes	Yes
	Sources of vulnerability	Yes	No
	Village level	No	
	Vulnerability	Yes	No
(Khan and Salman 2012)	Coping capacity	Yes	No
	Damage potential	Yes	No
	Lack of decent housing	Yes	Yes
	Lack of decent standard of living	Yes	Yes
	Lack of knowledge	Yes	Yes
	Livestock households and farm households	Yes	Yes
	Population density	Yes	Yes
	Regional vulnerability	Yes	No
(Luers et al. 2003)	Adaptive capacity	Yes	Yes
	Exposure	Yes	Yes
	Sensitivity	Yes	Yes
	State of system relative to threshold of damage	Yes	Yes
	Threshold of damage	Yes	Yes
	Vulnerability as suceptability	Yes	No
	Well-being	Yes	Yes
(Marshall 2010)	ability to plan, learn, reorganise	Yes	Yes
	Adpative capacity	Yes	Yes
	interest n change	Yes	Yes
	perception of risk	Yes	Yes
	proximity to coping threshold	Yes	Yes
	Resilience	No	
	resource dependency	Yes	Yes
	use of forecasts	Yes	Yes
(Mengistu 2011)	Adaptation strategies	No	
	Climate change	No	
	Climate forecast methods	No	
	Coping strategies	No	
	Drought early warning systems	No	
	Knowledge of farmers	No	
	Perception of Adiha farmers	Yes	Yes
(Misselhorn 2005)	Access to food	No	
	Access to sufficient food	No	
	Direct drivers	No	
	Food insecurity	Yes	not operationalized
	Food production	No	
	Household and community vulnerability	Yes	not operationalized
	Indirect drivers	No	
	Livelihood level issues	No	
	Livelihood strategies	Yes	not operationalized
(Mubaya et al. 2012)	Climate change	Yes	Yes

	Climate change and variability	Yes	Yes
	Climate variability	Yes	Yes
	Farmer perceptions	Yes	Yes
	Non-climatic stress	Yes	Yes
	Threat to livelihoods	No	
(Mutsvangwa 2011)	Cereal production	Yes	Yes
	Climate change	No	
	Expected food insecurity	Yes	No
	food insecurity	No	
	small holder farmers	No	
	Vulnerability	Yes	Yes
	vulnerability threshold	Yes	Yes
	welfare indicator	Yes	No
(Nkondze, Masuku, and Manyatsi 2013)	Household vulnerability to climate change	No	
	Factors affecting vulnerability	No	
(Notenbaert et al. 2013)	Adaptive capacity	Yes	No
	Exposure	Yes	Yes
	Institutional environment	Yes	Yes
	Livelihood assets	Yes	Yes
	Livelihood strategies	Yes	Yes
	Livelihoods	Yes	Yes
	Response and management options	Yes	No
	Risks	Yes	Yes
	sensitivity	Yes	No
	Vulnerability	Yes	No
	Vulnerability outcomes	Yes	Yes
(Piya, Maharjan, and Joshi 2012)	Adaptive capacity	Yes	No
	Exposure	Yes	Yes
	Financial capital	Yes	Yes
	Human capital	Yes	Yes
	Livelihood assets	Yes	No
	Local level vulnerability	Yes	No
	Natural capital	Yes	Yes
	Physical capital	Yes	Yes
	Sensitivity	Yes	Yes
	Social capital	Yes	Yes
(Sallu, Twyman, and Stringer 2010)	dynamic natural resource base	Yes	Yes
	factors influencing resilience and vulnerability	Yes	Yes
	livelihood trajectories	Yes	Yes
	resilience and vulnerability of rural livelihoods	yes	No
(Sarris and Karfakis 2010)	cash crop growing households	No	
	covariate shocks	Yes	Yes
	crop-growing households	No	
	household consumption	Yes	Yes
	household socio-economic characteristics	No	
	idiosyncratic shocks	Yes	Yes
	non-cash crop growing households	no	

	Rural household vulnerability	Yes	Not operationalized
(Sietz, Choque, and Lüdeke 2012)	access to food	Yes	not operationalized
	adaptive capacity	Yes	Yes
	cluster pattern analysis	Yes	Yes
	Exposure	Yes	Yes
	food availability	Yes	No operationalized
	food security	Yes	yes
	household characterisitcs	No	
	Sensitivity	Yes	Yes
	Vulnerability	Yes	No
	vulnerability creating mechanisms	No	
	weather extremes	No	
(Tesso, Emana, and Ketema 2012)	adaptive capacity	Yes	not operationalized
	Determinants of resilience	Yes	Yes
	Exposure	Yes	not operationalized
	fast bouncing back	Yes	No
	household level resilience	Yes	Yes
	household vulnerability to climate change	Yes	Not operationalized
	moderate bouncing back	Yes	No
	Sensitivity	Yes	Not operationalized
	slow bouncing back	Yes	No
(Westerhoff and Smit 2009)	adaptation strategy	Yes	Yes
	Adaptive capacity	Yes	Yes
	biophyisical conditions	No	
	exposed and sensitive to climate change	Yes	Yes
	local scale vulnerability	Yes	No
	multiple underlying forces	Yes	Yes
	socio-economic conditions	No	
**Total**	358	281	154

Appendix E: Selection of framework-defining emic constructs

**Framework-defining emic constructs (Steps 2.8, 2.9)**							
Bridging Framework code	*Common constructs*	*Common bar 1*	*Articles omitting*	*Common bar 2*	*Articles omitting*	*Common bar 3*	*Articles omitting*
Merged [IPCC][Livelihoods integrated into IPCC][Residual7]	(1) Vulnerability; Vulnerability of coffee farming communities; vulnerability to climate change; Vulnerability ipcc; Vulnerability as suceptability; local level vulnerability; (2) Adaptive capacity	Exposure	(CARE 2009)				
		Sensitivity	(CARE 2009)				
unchanged [ie VEP]		Vulnerability; Rural household vulnerability	(Chhihn and Poch 2012)	household characteristics; household consumption	(Deressa, Hassan, and Ringler 2009); (Calvo and Dercon 2013)		
		Expected poverty; household vulnerability as expected poverty; vulnerability to poverty	(Sarris and Karfakis 2010)	covariant shocks; covariate shocks	(Deressa, Hassan, and Ringler 2009); (Chhihn and Poch 2012)		
		Household consumption (income);	(Calvo and Dercon	idiosyncratic shocks; idiosyncratic	(Deressa, Hassan, and		

		household consumption; current poverty status	2013)	shocks	Ringler 2009); (Chhihn and Poch 2012)	
		minimum consumption (income) level; poverty; Poverty line	(Sarris and Karfakis 2010)			
		Climate and non-climate shocks; environmental shocks; shocks	(Sarris and Karfakis 2010)			
Merged [Food Security][VE Food security]	(1) Expected food insecurity; expected future food security status; (2) food insecurity; vulnerability to future food insecurity; (3) future nutricional status; welfare indicator (4) vulnerability					
oth-VEP Extensions	Household vulnerability to poverty					
	household level					
	community level					
	risk-induced poverty;					

	Determinants of Poverty & Vulnerability						
	idiosyncratic shocks						
	covariate shocks						
Merged [Residual6][Residual 11] [Residual12][Residual16]	climate change and variability; biophyisical conditions; climatic risk factors; climate change	Farmer perceptions; Experience of long term climate change; Perception of Adida farmers	(Westerhoff and Smit 2009)	Perception of long term climate change; knowledge of farmers	(Mubaya et al. 2012); (Westerhoff and Smit 2009)		
		non-claimtic stress; socio-economic conditions; non-climatic risk factors	(Mengistu 2011)				
		threat to livelihoods; exposed and sensitive to climate change; livelihood risks	(Mengistu 2011)	actual meterological observations; climate forecast methods; drought early warning system	(Mubaya et al. 2012); (Westerhoff and Smit 2009)		
		adaptation strategy; adaptation ot long term climate change; coping strategies	(Mubaya et al. 2012)				
Residual 1 (Berkes & Ross)	Agency						
	Adaptive capacity						
	Community						

	Resilience	
	Sefl-organising	
Residual 2 Bogale et al	Propoerty rights	
	Propoerty rights regime	
	household choice	
	Vulnerability	
	private property	
	public property	
Residual 3 Dasgupta & baschieri	Welfare of rural housholds	
	household vulnerability to climate change	
	Asset vulnerability	
	communities at risk of climate shocks	
	prepared for adverse consequences	
	risk of experiencing climate change shock	
Residual 4 Eakin et al 2012	Adaptiveness	
	Disaster	
	Impacts & responses to Hurricane Stan by coffee farmers	
	Resilience	
	Resilience of rural livelihoods	
	vulnerability	
Residual 5 Ford & Smit	vulnerability to climate risks	
	Current vulnerability	
	Future vulnerability	

	Current exposure	
	current adaptive capacity	
	future exposure	
Residual 8 Ionesco et al	adaptive capacity	
	Vulnerability	
	Stimulus	
	Entity	
	preference criteria	
	reference scenarios	
Residual 9 Khan & Salman	regional vulnerability	
	damage potential	
	coping capacity	
	Population density	
	Lack of decent standard of living	
	Lack of decent housing	
Residual 10 Marshall	Resilience	
	Adpative capacity	
	use of forecasts	
	resource dependency	
	perception of risk	
	ability to plan, learn, reorganise	
Residual 13 Nkondze et al	factors afecting vulnerability	
	Household vulnerability to climate change	
Residual 14 Sietze et al	Vulnerability	
	Exposure	
	Sensitivity	
	Adaptive	

	capacity	
	cluster pattern analysis	
	Food security	
Residual 15 Tesso et al	household vulnerability to climate change	
	Exposure	
	Sensitivity	
	Adaptive capacity	
	Determinants of resilience	
	household level resilience	
Residual 17 Eakin et al 2008	Livelihood vulnerability	
	nested and teleconnected livelihood vulnerability	
	cross-scalar teleconnection	
	response outcomes	
	exogenous drivers	
	Nested system	
Food Security – Livelihoods Misselhorn	livelihood level issues	
	access to sufficient food	
	Food insecurity	
	Livelihood strategies	
	household and community vulnerability	
	Direct drivers	
Livelihoods A Hahn et al A	Livelihood vulnerability	
	Socio-demographic profile	
	Livelihood strategies	

	Health	
	Water	
	Natural disasters and Climate variability	
Livelihoods B – Sallu et al	resilience and vulnerability of rural livelihoods	
	factors influencing resilience and vulnerability	
	dynamic natural resource base	
	livelihood trajectories	

Appendix F: Record of within-comparison of emic constructs (Step 2.12)

**Record of within-comparison of emic constructs (Step 2.12)**				
**Emic construct**	**Appears in**	**Definitions**	**Decision**	**Selected representative for across-comparison**
ability to plan, learn, reorganise	(Marshall 2010)	A description of each dimension can be found in Marshall and Marshall (2007). (Marshall 2010, 38)	Default	Default
access to sufficient food	(Misselhorn 2005)	NOT DEFINED	Default	Default
adaptation ot long term climate change	(Gandure, Walker, and Botha 2013)	Unique in our study, is the use of individual perceptions in identifying and understanding the processes of adaptation in an area that has undergone significant political and socio-economic reformation resulting from a series of conflicts over land resources. (Gandure, Walker, and Botha 2013, 40)	Default	Default
adaptation strategy	(Westerhoff and Smit 2009)	Adaptations, or adaptive strategies, employed by individuals or groups are depicted as being mediated through their relative adaptive capacities, indicating that adaptations may or may not be accessed according to the distribution of various types of resources such as physical or social capital, as developed by Adger and Kelly (1999). (Westerhoff and Smit 2009, 321)	Default	Default
adaptive capacity	(Antwi-Agyei et al. 2013)	Adaptive capacity in the context of climate change has been defined by the IPCC (2007, p. 869)as ‘‘the ability of a system to adjust to climate change (including climate variability and extremes) to moderate potential damages, to take advantage of opportunities, or to cope with the consequences.’’ Adaptive capacity connotes some positive attributes of a system that enable it to reduce the adverse impacts (vulnerability) associated with climate change (Engle 2011). (Antwi-Agyei et al. 2013, 905)	Adaptive capacityA = {(Antwi-Agyei et al. 2013); (Baca et al. 2014); (CARE 2009); (Füssel and Klein 2006); (Hahn, Riederer, and Foster 2009); (Jamir et al. 2013); (Luers et al. 2003); (Notenbaert et al. 2013); (Piya, Maharjan, and Joshi	Adaptive Capacity A: (Füssel and Klein 2006)
	(Baca et al. 2014)	In contrast, adaptive capacity is defined as a system's ability to adjust to climate change in order to reduce or mitigate possible damage [3]. Adaptive capacity		Adaptive capacity B: (Marshall 2010)

		is dynamic, and depends partly on the society productive base, such as: natural and artificial assets, social benefits and networks, human capital and institutions, governance, national income, health and technology [2], and how much capability a society has to adapt to the changes so as to maintain, minimize loss of, or maximize gain in welfare.<br>(Baca et al. 2014, 2)	2012); (Tesso, Emana, and Ketema 2012)}<br>Adaptive capacityB = {(Berkes and Ross 2013); (Marshall 2010)}	
	(Berkes and Ross 2013)	Adaptive capacity is the capacity of actors in a system to influence resilience<br>(Folke et al. 2010), and often works through social networks and learning communi- ties (Goldstein 2012).<br>[...]<br>We view adaptive capacity as a latent pro- perty, which can be activated when people exercise their agency. The processes by which this occurs have not been well explored.<br>(Berkes and Ross 2013, 15)	Adaptive capacity C = {(Sietz, Choque, and Lüdeke 2012)}<br>Variance = {(Ionesco et al. 2009)}	Adaptive capacity C: (Sietz, Choque, and Lüdeke 2012)
	(CARE 2009)	The ability of a system to adjust to climate change (including climate variability and extremes) to moderate potential damages, to take advantage of opportunities, or to cope with the consequences.6<br>(CARE 2009, 5)		
	(Füssel and Klein 2006)	Adaptive capacity: The ability of a system to adjust to climate change (in- cluding climate variability and extremes) to moderate potential damages, to take advantage of opportunities, or to cope with the consequences.<br>(Füssel and Klein 2006, 319)		
	(Hahn, Riederer, and Foster 2009)	adaptive capacity is the system's ability to withstand or recover from the exposure (Ebi et al., 2006).<br>(Hahn, Riederer, and Foster 2009, 75)		
	(Ionesco et al. 2009)	NOT DEFINED		
	(Jamir et al. 2013)	Asper theIPCC's definition and framework, vulnerability<br>is understood as a function of three components—exposure, sensitivity and adaptive capacity. Vulnerability is defined as ''the degree to which a		

		system is susceptible to or unable to cope with, adverse effects of climate change, including cli- mate variability and extremes'' (IPCC 2001).<br>(Jamir et al. 2013, 154)		
	(Luers et al. 2003)	We define adaptive capacityas the extent to which a<br>system can modify its circumstances to move to a less vulnerable condition (Fig. 1c). We quantifyadaptive capacity(A) as the difference in the vulnerabilityunder existing conditions and under the less vulnerable condition to which the system could potentially shift:<br>A ¼ Vðexisting conditionsÞ Vðmodified conditionsÞ:<br>(Luers et al. 2003)(Luers et al. 2003, 259)		
	(Marshall 2010)	It refers to the ability of individuals or communities to adapt to adversity and stressful life-events by 'reorganising' through networks or institutions that learn, store knowledge and experi- ence and are creative, flexible and novel in their approach to problem solving (Vayda and McCay, 1975; McCay, 1981; Sonn and Fisher, 1998).<br>(Marshall 2010, 37)		
	(Notenbaert et al. 2013)	the risk response or the options that people have for managing these risks (Turner et al. 2003).<br>(Notenbaert et al. 2013, 460)		
	(Piya, Maharjan, and Joshi 2012)	Adaptive capacity is the ability of a system to adjust to climate change including climate variability and extremes, to moderate the potential damage from it, to take advantage of its opportunities, or to cope with its consequences. Selection of indicators for adaptive capacity is based on the DFID sustainable livelihoods framework, whereby adaptive capacity is taken to be a function of asset possession by the households (Jakobsen, 2011; Nelson, et al., 2010b).<br>(Piya, Maharjan, and Joshi 2012, 12)		
	(Sietz, Choque, and Lüdeke 2012)	the adaptive capacity of smallholders (the term as used in this study encompasses the coping capacity) describes the ability to adjust to weather extremes, manage damages or		

		explore alternative livelihood opportunities.<br>(Sietz, Choque, and Lüdeke 2012, 490)		
	(Tesso, Emana, and Ketema 2012)	According to Füssel and Klein, the risk-hazard framework (biophysical approach) corresponds most closely to sensitivity in the IPCC ter- minology while the adaptive capacity (broader social development) is largely consistent with the socioeco- nomic approach [18].<br>[...]<br>In the framework, capacity is generated from the implementation of adaptation and mitigation intervene- tions [18].<br>(Tesso, Emana, and Ketema 2012, 873)		
Adaptiveness	(Eakin et al. 2012)	Conceptually, the process of household adaptation could<br>be considered a function of the current state of the household (entitlements, assets, activities) and the biophysical, politi- cal, economic, institutional contexts in which decisions are made (determining the choice set for any household); the exposure and sensitivity of a household to stress and change; the decisions taken; and the outcome of those decisions. Adaptation is a decision process designed to ‘‘maintain capacities to deal with future change’’ and thus can involve actions that enhance adaptive capacities (Nelson et al. 2007). A household’s experience of an environmental shock or change—how it copes with the event—may result in a rel- atively dramatic change in livelihood activities with poten- tially negative welfare outcomes (e.g., increased poverty) or, alternatively, may provide opportunities for learning and welfare improvements and thus enhanced adaptive capaci- ties (McSweeney and Coomes 2011)<br>(Eakin et al. 2012, 477)	Default	Default
Agency	(Berkes and Ross 2013)	Not defined	Default	Default
Asset vulnerability	(Dasgupta and Baschieri 2010)	Using Moser’s (1998) asset vulnerability framework as guidance, we selected a range of variables to create an index of household vulnerability from GLSS 4. Each variable captures an aspect of vulnerability.	Default	Default

		(Dasgupta and Baschieri 2010, 807)		
biophyisical conditions	(Westerhoff and Smit 2009)	Not defined	Default	Default
Climate and non-climate shocks	(Deressa, Hassan, and Ringler 2009)	Not defined	Default	Default
climate change	(Mengistu 2011)	Not defined	Default	Default
climate change and variability	(Mubaya et al. 2012)	In this paper, the distinction between 'climate variability' and 'climate change' relates to differences in time-scale. On the one hand, 'climate variability' is conceptualised as variations in the climate system over short time scales such as months, years or decades and on the other hand 'climate change' is conceptualised as longer term trends in mean climate variables of periods of decades or longer. This is the suggested distinction in definitions of the concepts in question by the IPCC (2001). (Mubaya et al. 2012, 10)	Default	Default
climatic risk factors	(Gandure, Walker, and Botha 2013)	Not defined	Default	Default
cluster pattern analysis	(Sietz, Choque, and Lüdeke 2012)	Without such a pre-selection, alternative approaches investigate the structure of the data space spanned by selected vulnerability indicators using cluster analysis. They deliver useful insights into recurrent indicator com-binations based on similarities among units of analysis, in cases where such a grouping exists. For example, clustering revealed typical livelihood strategies employed by small- holders in Mexico and Botswana (Eakin 2005; Sallu et al. 2010). (Sietz, Choque, and Lüdeke 2012, 492)	Default	Default
communities at risk of climate shocks	(Dasgupta and Baschieri 2010)	NOT DEFINED	Default	Default
community level	(Échevin 2011)	an extension of this empirical framework will consist in using two-level (i.e. household and community levels) modelling of the impact of those shocks following Günther and Harttgen (2009)'s approach. (Échevin 2011, 3)	NO CHANGE	(Günther and Harttgen 2009)
	(Günther and Harttgen 2009)	Multilevel models are designed to analyze the relationship between variables that are measured at different		

		hierarchical levels (for an introduction see, e.g., Bryk & Raudenbush, 1992; Goldstein, 1999; Hox, 2002). We speak of ‘‘hierarchical” or ‘‘multilevel” data structure whenever variables are collected at different hierarchical levels with lower-levels (e.g., house- holds) nested within higher-levels (e.g., communities).<br>(Günther and Harttgen 2009, 1225)		
Community Resilience	(Berkes and Ross 2013)	Community resilience as a function of the strengths or characteristics that have been identified as important, leading to agency and self-organization. (Berkes and Ross 2013) 14 (Berkes and Ross 2013, 14)	Default	Default
coping capacity	(Khan and Salman 2012)	NOT DEFINED	Default	Default
coping strategies	(Mengistu 2011)	Not defined	Default	Default
covariate shocks	(Échevin 2011)	NOT DEFINED	NO CHANGE	(Günther and Harttgen 2009)
	(Günther and Harttgen 2009)	Households in developing countries are frequently hit by se-<br>vere idiosyncratic and covariate shocks resulting in high income volatility. 1<br>(Günther and Harttgen 2009, 1222)<br>[...]<br>1. Here, and in the following, idiosyncratic shocks refer to household-specific shocks (e.g., injury, birth, death, or job loss of a household member) that are only weakly correlated across households within a community. Covariate shocks refer to shocks that are correlated across households within communities but only weakly correlated across communities (e.g., natural disasters or epidemics).<br>(Günther and Harttgen 2009, 1231)		
cross-scalar teleconnection	(Eakin, Winkels, and Sendzimir 2009)	‘‘teleconnections’’, a term used in climatology in relation to ‘‘any transmission of a coherent effect beyond the location where the forcing occurred’’ (Chase et al., 2005). For example, one of the teleconnections associated with the El Nin<br>˜ o-Southern Oscillation effect is severe drought<br>in Northeastern Brazil. Teleconnections are also associated with other climate phenomena such as the North Atlantic	Default	Default

		Oscillation. The label of ‘‘teleconnection’’ is not explanatory in and of itself, but rather signifies the existence of a correlation in events, and highlights the need to explore the connecting mechanisms and drivers in order to anticipate outcomes.<br>(Eakin, Winkels, and Sendzimir 2009, 400)		
current adaptive capacity	(Ford and Smit 2004)	Adaptive capacity refers to a community’s potential or ability to address, plan for, or adapt to exposure (Smit and Pilifosova, 2003). Most communities can cope with normal climatic conditions and a range of deviations around norms. People have learned to modify their behaviour and their environment to manage and take advantage of their local climatic conditions (Jones and Boer, 2003). This ability to cope is referred to in the literature as the “coping range”; it reflects resource use options and risk management strategies to prepare for, avoid or moderate, and recover from exposure effects (Hewitt and Burton, 1971; Smit et al., 1999; Jones, 2001; Smit and Pilifosova, 2003). Adaptive capacity relates to communities’ resilience, resistance, flexibility, and ro-bustness (Smithers and Smit, 1997). It is influenced by economic wealth, social networks, infrastructure, social in-stitutions, social capital, experience with previous risk, the range of technological adaptation available, and equity of access to resources within the community, as well as by other stresses that contribute to the environment in which decisions are made (Adger and Kelly, 1999; Smit and Pilifosova, 2001; Smith et al., 2003).<br>(Ford and Smit 2004, 393)	Default	Default
Current exposure	(Ford and Smit 2004)	Exposure is a property of a community relative to climatic conditions. It reflects both the nature of the climatic conditions and nature of the community itself. Some communities may be exposed to a particular climate event whereas the same event may not affect another community. Climatic characteristics include magnitude, frequency, spatial dispersion, duration,	Default	Default

		speed of onset, and temporal spacing of climatic risks, relating to tem- peratures, precipitation, and wind. The nature of the com- munity concerns its location relative to the climatic risks (Ford and Smit 2004, 393)		
current poverty status	(Chhihn and Poch 2012)	This study adopts the approach to measuring household economic vulnerability posited and elaborated in Chaudhuri's (2003) study of household vulnerability (Chhihn and Poch 2012, 30)	Default	Default
Current vulnerability	(Ford and Smit 2004)	The assessment of current vulnerability requires analyzing and documenting communities' experiences with climatic risks (current exposure) and the adaptive options and resource management strategies employed to address these risks (current adaptive capacity). (Ford and Smit 2004, 395)	Default	Default
damage potential	(Khan and Salman 2012)	Not defined	Default	Default
Determinants of Poverty & Vulnerability	(Échevin 2011)	Not defined	Default	Default
Determinants of resilience	(Tesso, Emana, and Ketema 2012)	important determinants for resilience at household level in North Shewa zone of Ethiopia. (Tesso, Emana, and Ketema 2012)(Tesso, Emana, and Ketema 2012, 872)	Default	Default
Direct drivers	(Misselhorn 2005)	NOT DEFINED	Default	Default
Disaster	(Eakin et al. 2012)	NOT DEFINED	Default	Default
dynamic natural resource base	(Sallu, Twyman, and Stringer 2010)	NOT DEFINED	Default	Default
Entity	(Ionesco et al. 2009)	The mainstream mathematical interpretation of an entity is that of a dynamical system in a given state. This is the interpretation we will adopt here (Ionesco et al. 2009, 4)	Default	Default
environmental shocks	(Chhihn and Poch 2012)	This study adopts the approach to measuring household economic vulnerability posited and elaborated in Chaudhuri's (2003) study of household vulnerability (Chhihn and Poch 2012, 30)	Default	Default
exogenous drivers	(Eakin,	exogenous drivers (i.e. the risk and	Default	Default

	Winkels, and Sendzimir 2009)	stress factors)<br>(Eakin, Winkels, and Sendzimir 2009, 399)		
Expected food insecurity	(Mutsvangwa 2011)	Among other things, the vulnerability status of smallholder farmers in different locations will be influenced by the household's ability to produce enough to ensure the household's food security.<br>(Mutsvangwa 2011) (Mutsvangwa 2011, 21)	Default	Default
Expected future food-security status	(Capaldo et al. 2010)	conceptual framework drawn from it by Løvendal and Knowles (2005).<br>(Capaldo et al. 2010, 7)	Default	Default
Expected poverty	(Deressa, Hassan, and Ringler 2009)	This method is based on estimating the probability that a given shock or set of shocks will move household consumption below a given minimum level (such as a consumption poverty line) or force the consumption level to stay below the minimum if it is already below this level (Chaudhuri et al. 2002).<br>(Deressa, Hassan, and Ringler 2009, 3)	Default	Default
Experience of long term climate change	(Gandure, Walker, and Botha 2013)	The study relied on the experience and knowledge of farmers and community members in<br>Gladstone to characterise their livelihood risks fromclimatic and non-climatic risk factors.<br>(Gandure, Walker, and Botha 2013, 41)	Default	Default
exposed and sensitive to climate change	(Westerhoff and Smit 2009)	People's exposures and sensitivities to external conditions are influenced by their occupancy and livelihood characteristics, and the nature and degree to which these are affected by the external stresses.<br>(Westerhoff and Smit 2009, 321)	Default	Default
Exposure	(Antwi-Agyei et al. 2013)	Exposure relates to the extent to which a particular system may be exposed to climatic stresses or variations (IPCC 2007).<br>(Antwi-Agyei et al. 2013, 905)	NO CHANGE	(Füssel and Klein 2006)
	(Baca et al. 2014)	Exposure is the nature and extent of changes that a place's climate is subjected to with regard to variables such as temperature, precipitation, and extreme weather events.<br>(Baca et al. 2014, 2)		
	(Füssel and Klein 2006)	Exposure: The nature and degree to which a system is exposed to significant climatic variations.		

		The exposure of a system to climate stimuli depends on the level of global cli- mate change and, due to the spatial heterogeneity of anthropogenic climate change, on the system's location<br>(Füssel and Klein 2006, 313)		
	(Hahn, Riederer, and Foster 2009)	Exposure in this case is the magnitude and duration of the climate-related exposure such as a drought or change in precipitation,<br>(Hahn, Riederer, and Foster 2009, 75)		
	(Jamir et al. 2013)	Asper theIPCC's definition and framework, vulnerability<br>is understood as a function of three components—exposure, sensitivity and adaptive capacity. Vulnerability is defined as ''the degree to which a system is susceptible to or unable to cope with, adverse effects of climate change, including cli- mate variability and extremes'' (IPCC 2001).<br>(Jamir et al. 2013, 154)		
	(Luers et al. 2003)	Different communities and ecosystems are exposed to<br>varying magnitudes and frequencies of disturbing forces, often resulting in differential vulnerabilities (IPCC, 2001; Turner et al., 2003a, b). We capture these differences in exposure bycalculating the expected value of the ratio of sensitivityto the state relative to a threshold based on the frequencydistribution of the stressors of concern:<br>(Luers et al. 2003, 258)		
	(Notenbaert et al. 2013)	risks (or a chain of risky events) that people confront in pursuit of their livelihoods, (Turner et al. 2003).<br>(Notenbaert et al. 2013, 460)		
	(Piya, Maharjan, and Joshi 2012)	Exposure is the nature and degree to which a system is exposed to significant climatic variations.<br>(Piya, Maharjan, and Joshi 2012, 11)		
	(Sietz, Choque, and Lüdeke 2012)	expo- sure, sensitivity and coping/adaptive capacity (IPCC 2007).<br>(Sietz, Choque, and Lüdeke 2012, 490)		
	(Tesso, Emana, and Ketema 2012)	Furthermore, in the IPCC frame- work, exposure has an external dimension, whereas both sensitivity and adaptive capacity have an internal dimen- sion, which is implicitly assumed in the integrated vul- nerability assessment		

		framework [13].<br>(Tesso, Emana, and Ketema 2012, 873)		
factors afecting vulnerability	(Nkondze, Masuku, and Manyatsi 2013)	Not defined	Default	Default
factors influencing resilience and vulnerability	(Sallu, Twyman, and Stringer 2010)	Through comparative research we provide a rich contextual narrative and use it to explore those factors that in isolation and combination push livelihoods along particular "trajectories" towards vulnerability or resilience.<br>(Sallu, Twyman, and Stringer 2010, 2)	Default	Default
Farmer perceptions	(Mubaya et al. 2012)	there is an alternative approach which underscores how individuals perceive their environment and make decisions, with mal-adaptations attributed to problems in perception, cognition or the lack of available information (Diggs, 1991; Saarinen, 1966; Taylor et al., 1988). The main point is that from whatever level these adapta- tion measures are taken, the adaptation and coping measures depend on households' perceptions of extreme events and the problems associated with them (Davies, 1993).<br>(Mubaya et al. 2012, 10)	Default	Default
food insecurity	(Misselhorn 2005)	Food insecurityin the communities described bythe<br>case studies maybe conceptualized as one element in an entrenched and escalating cycle of vulnerability (Fig. 3).<br>(Misselhorn 2005, 38)	NO CHANGE	(Misselhorn 2005)
	(Mutsvangwa 2011)	NOT DEFINED		
food security	(Sietz, Choque, and Lüdeke 2012)	Food security is often discussed in terms of four<br>dimensions: food availability, access, stability of supply/ access and utilisation (FAO 2000).<br>(Sietz, Choque, and Lüdeke 2012, 490)	Default	Default
future exposure	(Ford and Smit 2004)	Future exposure also includes estimating the future state of the socioeco- nomic conditions, given that exposure is a property of the system relative to risk.<br>(Ford and Smit 2004, 396)	Default	Default
future nutricional status	(Capaldo et al. 2010)	conceptual framework drawn from it by Løvendal and Knowles (2005).<br>(Capaldo et al. 2010, 7)	Default	Default

Future vulnerability	(Ford and Smit 2004)	Future vulnerability is assessed by analyzing how cli- mate change will alter the nature of the climate-related risks and whether the communities' coping strategies will have the capacity to deal with these risks. Assessing future exposure involves collaboration with the climate science community to estimate the likelihood of changes in cli- matic attributes identified by the community<br>(Ford and Smit 2004, 396)	Default	Default
Health	(Hahn, Riederer, and Foster 2009)	Proximity to health facility; 2 weeks illness; malaria-exposure-prevention (Hahn, Riederer, and Foster 2009, 77)	Default	Default
household and community vulnerability	(Misselhorn 2005)	In general terms, vulner- abilityand social resilience have been similarlydefined as the abilityof a system or communityto resist or absorb adverse conditions.<br>[...]<br>Vulnerable commu- nities, where people are unable to buffer themselves from hazards for a number of reasons, have a low ability to cope with short-term shocks (such as drought) and to mitigate chronic stressors, which in turn means that the negative impacts on livelihoods resulting from coping and survival strategies are veryhigh.<br>(Misselhorn 2005, 38)	Default	Default
household choice	(Bogale, Taeb, and Endo 2006)	Not defined	Default	Default
household consumption	(Sarris and Karfakis 2010)	consumption falling below a poverty threshold (Christiaensen and Subbarao 2004, Chaudhuri, et. al. 2002)<br>(Sarris and Karfakis 2010, 4)	Default	Default
Household consumption(income)	(Deressa, Hassan, and Ringler 2009)	Not defined	Default	Default
household level	(Échevin 2011)	an extension of this empirical framework will consist in using two-level (i.e. household and community levels) modelling of the impact of those shocks following Günther and Harttgen (2009)'s approach.<br>(Échevin 2011, 3)	NO CHANGE	(Günther and Harttgen 2009)
	(Günther and Harttgen 2009)	Multilevel models are designed to analyze the relationship between variables that are measured at different		

		hierarchical levels (for an introduction see, e.g., Bryk & Raudenbush, 1992; Goldstein, 1999; Hox, 2002). We speak of ‘‘hierarchical” or ‘‘multilevel” data structure whenever variables are collected at different hierarchical levels with lower-levels (e.g., house- holds) nested within higher-levels (e.g., communities).<br>(Günther and Harttgen 2009, 1225)		
household level resilience	(Tesso, Emana, and Ketema 2012)	According to DFID, resil- ience at community level is explained as the ability of countries, communities and households to manage change, by maintaining or transforming living standards in the face of shocks or stresses—such as earthquakes, drought or violent conflict—without compromising their long- term prospects [10]. Similarly, resilience is the ability of a social or ecological system to absorb disturbances while retaining the same basic structure and ways of functioning, the capacity for self-organization, and the capacity to adapt to stress and change. This is a meas- urement of community's capacity to absorb external shocks. In the aftermath of occurrence of climate change induced shocks, how do farmer bounce back to normal livelihood is about the resilience level of farming com- munity. A resilient community is able to respond to changes or stress in a positive way, and is able to main- tain its core functions as a community despite those stresses [11].<br>(Tesso, Emana, and Ketema 2012, 871, 872)	Default	Default
household vulnerability as expected poverty	(Chhihn and Poch 2012)	Household vulnerability as ex- pected poverty is defined as the probability that households will move into poverty given certain environmental shocks, current poverty status and household characteristics of respondents.<br>(Chhihn and Poch 2012, 30)	Default	Default
household vulnerability to climate change	(Dasgupta and Baschieri 2010)	Using the GLSS 4, we applied the asset vulnerability framework developed by Moser (1996, 1998, 2007). We constructed an index of vulnerability to climate change, at the household level.<br>(Dasgupta and Baschieri 2010, 807)	NO CHANGE	(Tesso, Emana, and Ketema 2012)
	(Nkondze,	NOT DEFINED		

	Masuku, and Manyatsi 2013)			
	(Tesso, Emana, and Ketema 2012)	Therefore, vulner- ability is the degree to which a system is susceptible or unable to cope with the adverse effects of climate change, including climate variability and extremes. In this regard, vulnerability is a function of the character, magnitude, and rate of climate variation to which a system is ex- posed, its sensitivity, and its adaptive capacity [4].<br>(Tesso, Emana, and Ketema 2012)<br>(Tesso, Emana, and Ketema 2012, 871)		
Household vulnerability to poverty	(Échevin 2011)	we can define vulnerability to poverty as the probability of falling into poverty when one's consumption/income falls below a predefined poverty line.<br>(Échevin 2011, 5)	NO CHANGE	(Échevin 2011)
	(Günther and Harttgen 2009)	The suggested approach is an integration of multilevel analysis (e.g., Goldstein, 1999) into Chaudhuri's (2002) method to estimate vulnerabil- ity<br>(Günther and Harttgen 2009, 1223)		
idiosyncratic shocks	(Échevin 2011)	NOT DEFINED	NO CHANGE	(Günther and Harttgen 2009)
	(Günther and Harttgen 2009)	Households in developing countries are frequently hit by se-<br>vere idiosyncratic and covariate shocks resulting in high income volatility. 1<br>(Günther and Harttgen 2009, 1222)<br>[...]<br>1. Here, and in the following, idiosyncratic shocks refer to household-specific shocks (e.g., injury, birth, death, or job loss of a household member) that are only weakly correlated across households within a community. Covariate shocks refer to shocks that are correlated across households within communities but only weakly correlated across communities (e.g., natural disasters or epidemics).<br>(Günther and Harttgen 2009, 1231)		
Impacts & responses to Hurricane Stan by coffee farmers	(Eakin et al. 2012)	In this paper, we document household responses to a climatic shock, Stan, to gain insight into how natural resource-dependent communities move to secure their livelihoods following	Default	Default

		significant loss, the implications of household responses for coffee farming as a ''domain of attraction,'' as well as to highlight those aspects of household choices and perceptions that may be indicative of resilience at broader scales.<br>(Eakin et al. 2012, 477)		
Lack of decent housing	(Khan and Salman 2012)	(3) Lack of decent housing: Lack of access to a proper housing facility, as measured by the weighted average of two variables, percentage of population having kacha (weighted 3/6) and semi-pacca (weighted 1/6) houses, is linked closely to vulnerability.iv<br>(Khan and Salman 2012, 165)	Default	Default
Lack of decent standard of living	(Khan and Salman 2012)	(4) Lack of decent standard of living: Lack of access to overall socioeconomic provisions is measured by the average of two variables: the percentage of the population without access to piped water and the percentage of population with- out access to electricity<br>(Khan and Salman 2012, 165)	Default	Default
livelihood level issues	(Misselhorn 2005)	Not defined	Default	Default
livelihood risks	(Gandure, Walker, and Botha 2013)	Not defined	Default	Default
Livelihood strategies	(Hahn, Riederer, and Foster 2009)	Household working elsewhere; agriculture dependent households; livlihood diversification<br>(Hahn, Riederer, and Foster 2009, 77)	NO CHANGE	(Misselhorn 2005)
	(Misselhorn 2005)	A livelihood maybe described as the capability, assets<br>and activites required for a means of living. People everywhere pursue a range of livelihood strategies in attempting to increase their income and asset base ('accumulation strategies'), spread or reduce risk (in-crease securitythrough 'adaptive strategies'), mitigate the impact of shocks ('coping strategies'), and at the extreme, ensure survival through 'survival strategies' (Devereux, 1999; Scoones, 2000).<br>(Misselhorn 2005, 38)		
livelihood trajectories	(Sallu, Twyman, and Stringer 2010)	Bagchi et al. (1998) use the term "livelihood trajectories" to describe and explain the direction and pattern of livelihoods of individuals or groups of	Default	Default

		people (e.g., households). A livelihood trajectory approach allows the examination of an individual household’s “strategic behavior that is embedded in a historical repertoire, in social differentiation” (de Haan and Zoomers 2005), and in perceptions of risk. Such an approach is sensitive to life histories (an individual’s own “story” of their changing livelihoods).<br>(Sallu, Twyman, and Stringer 2010, 2)		
Livelihood vulnerability	(Hahn, Riederer, and Foster 2009)	The LVI includes seven major components: Socio-Demographic Profile, Livelihood Strategies, Social Networks, Health, Food, Water, and Natural Disasters and Climate Variability<br>(Hahn, Riederer, and Foster 2009, 76)	Livelihood vulnerability A: {(Hahn, Riederer, and Foster 2009)}<br>Livelihood vulnerability B: {(Eakin, Winkels, and Sendzimir 2009)}	(Hahn, Riederer, and Foster 2009)
	(Eakin, Winkels, and Sendzimir 2009)	By placing the household as the focus of analysis, livelihood approaches highlight both the exogenous drivers (i.e. the risk and stress factors) and the factors internal to the household (i.e. ability to mitigate and cope with stress) which together influence household security and well-being (Chambers and Conway, 1992; Ellis, 1998).<br>(Eakin, Winkels, and Sendzimir 2009, 399)		(Eakin, Winkels, and Sendzimir 2009)
local level vulnerability	(Piya, Maharjan, and Joshi 2012)	Following the definition of vulnerability given by IPCC (2001), vulnerability in this<br>study is taken to be a function of exposure, sensitivity, and adaptive capacity.<br>(Piya, Maharjan, and Joshi 2012, 11)	Default	Default
minimum consumption(income) level	(Deressa, Hassan, and Ringler 2009)	a given minimum level (such as a consumption poverty line)<br>(Deressa, Hassan, and Ringler 2009, 3)	Default	Default
Natural disasters and Climate variability	(Hahn, Riederer, and Foster 2009)	Sub-constructs: flood, drought, cyclone events; no warning of disaster; injury or death from disaster; maximum temperature; minimum temperature; average percipitatoin<br>(Hahn, Riederer, and Foster 2009, 79)	Default	Default
nested and teleconnected livelihood vulnerability	(Eakin, Winkels, and Sendzimir 2009)	In this article we use the concept of ‘‘nested and tele-<br>connected vulnerabilities’’ to illustrate how the vulnerabilities and responses of farm households in distinct geographic locations are linked through cross-scalar processes, as well as	Default	Default

		‘‘teleconnected’’ in space and time. In a nested system, profoundchanges inkeyvariablesthatoperatenormallyonly at one level, e.g., within a defined geographic region or admin- istrative domain, can have non-linear outcomes for processes operating at broader scales of analysis (Gunderson andHolling, 2001).<br>(Eakin, Winkels, and Sendzimir 2009, 400)		
Nested system	(Eakin, Winkels, and Sendzimir 2009)	In a nested system, profoundchanges inkeyvariablesthatoperatenormallyonly at one level, e.g., within a defined geographic region or admin- istrative domain, can have non-linear outcomes for processes operating at broader scales of analysis (Gunderson andHolling, 2001). Local level processes can episodically influence larger scale phenomena, and such explosive ‘‘upward cascades’’ can be sources of surprise at distant locations.<br>(Eakin, Winkels, and Sendzimir 2009, 400)	Default	Default
non-climatic stress	(Mubaya et al. 2012)	It is important to note though, that climate change amplifies<br>already existing risks for farmers. This is the case as there are non- climatic risk factors such as economic instability, trade liberalisa- tion, conflicts and poor governance that may also be faced by farmers (Nyong and Niang-Diop, 2006). Other factors are impacts of diseases such as malaria and HIV and AIDS and lack of and limited access to climate and agricultural information (Gandure, 2005; Gandure and Marongwe, 2006). Africa is also characterised by institutional and legal frameworks that are, in some cases, insuffi- cient to deal with environmental degradation and disaster risks (Beg et al., 2002; Sokona and Denton, 2001).<br>(Mubaya et al. 2012, 10)	Default	Default
non-climatic risk factors	(Gandure, Walker, and Botha 2013)	Not defined	Default	Default
Perception of Adida farmers	(Mengistu 2011)	Adaptation of people to different hazards vary from household to households and region to region based on existing support system to increase the resilience of affected individuals.	Default	Default

		The assessment was aimed to generate primary information from the farming communities of Adiha related to cli- mate change. This report examined the perception of Adiha farmers on the trend of climate change and re- lated anomalities, existing coping strategies in place.<br>(Mengistu 2011, 139)		
perception of risk	(Marshall 2010)	NOT DEFINED	Default	Default
Population density	(Khan and Salman 2012)	(1) Population density: Vulnerability to the effects of cli- mate change consists of vulnerability to death, displacement, trauma, and loss of assets and livelihoods. This is measured by population density.<br>(Khan and Salman 2012, 165)	Default	Default
Poverty	(Chhihn and Poch 2012)	Technically, the household vulnerability index is derived from the difference between the ex-<br>pected log per capita income and the minimum log per capita income threshold, with households having per capita incomes lower than the minimum per capita income defined as vulnerable (poor). The expected log per capita income is estimated using the three-step feasible generalised least squares (FGLS) method.<br>(Chhihn and Poch 2012, 30)	Default	Default
Poverty line	(Calvo and Dercon 2013)	Our aim is merely to make an ex-ante statement on the vulnerability of the individual to fall below a poverty norm z,<br>(Calvo and Dercon 2013, 724)	Default	Default
preference criteria	(Ionesco et al. 2009)	Preference criteria are used to ascertain whether<br>or not a possible evolution of the entity is "bad" or "good". In the examples we have considered, we have seen that this judgment is usually made by comparison with a "normal" evolution, or an evolution under a "zero input".<br>(Ionesco et al. 2009, 5)	Default	Default
prepared for adverse consequences	(Dasgupta and Baschieri 2010)	Not defined	Default	Default
private property	(Bogale, Taeb, and Endo 2006)	Not defined	Default	Default

Property rights	(Bogale, Taeb, and Endo 2006)	Property rights can be defined as bthe capacity to call upon the collective stand behind one's claim to a benefit stream (Bromley, 1991).Q Thus, property rights involve a relationship between the right holder, others, and an institution to back up the claim (Bogale, Taeb, and Endo 2006, 136)	Default	Default
Property rights regime	(Bogale, Taeb, and Endo 2006)	Property rights over land and other natural resources are often broadly classified as public, com- mon, and private or blegal individualsQ such as com- panies. (Bogale, Taeb, and Endo 2006, 136)	Default	Default
public property	(Bogale, Taeb, and Endo 2006)	Not defined	Default	Default
reference scenarios	(Ionesco et al. 2009)	The examples provided also have this "punctual" or "one-step" character. However, in many applications, it is more natural to consider an evolution of the system to be a sequence of states, and to consider scenarios and reference scenarios instead of punctual inputs for the vulnerability assessment. A scenario is just a sequence of inputs: es =[e1, e2,..., en]. Corresponding to such a sequence, the sys- tem will undergo n transitions, xs =[x0, x1,..., xn] (Ionesco et al. 2009, 7)	Default	Default
regional vulnerability	(Khan and Salman 2012)	Therefore we define vulnerability as damage potential and coping capacity, that is, damage potential + coping capacity = regional vulnerability (McCarthy et al. 2001; Mustafa 1998). (Khan and Salman 2012, 164)	Default	Default
Resilience	(Eakin et al. 2012)	A resilient system is one that maintains continued integrity of fundamental social–ecological services and functions under conditions of variability, surprise and stress (Carpenter et al. 2001; Folke et al. 2002). Learning, self- organization and adaptiveness have been proposed as core components of resilient communities. In this interpretation, adaptiveness refers to the ability of communities to ''col- lectively manage the resilience of the system'' (Walker et al. 2004) or, in other words, to actively manage how a system responds to change. Resilience is often evaluated with explicit reference to a desired	NO CHANGE	(Eakin et al. 2012)

		state or (in less nor- mative terms) a ''domain of attraction'' (Gallopin 2006). A given system can have multiple domains of attraction, shifting states once thresholds are crossed. Resilience research seeks to understand the conditions in which thresholds are surpassed and shifts in state occur and strives to relate those conditions to specific human inter- ventions that facilitate or inhibit such shifts in state (Walker and Meyers 2004).<br>(Eakin et al. 2012, 477)		
	(Marshall 2010)	Not defined		
resilience and vulnerability of rural livelihoods	(Sallu, Twyman, and Stringer 2010)	Fraser et al.'s (2010) vulnerability framework<br>(Sallu, Twyman, and Stringer 2010, 2)	Default	Default
Resilience of rural livelihoods	(Eakin et al. 2012)	In the next section, we briefly review the related con-<br>cepts of resilience and vulnerability, focusing on an attri- bute central to the definition of both concepts: ''adaptiveness'' and ''adaptive capacity.'<br>(Eakin et al. 2012, 476)	Default	Default
resource dependency	(Marshall 2010)	NOT DEFINED	Default	Default
response outcomes	(Eakin, Winkels, and Sendzimir 2009)	outcomes of these responses in terms of individual or household welfare.<br>(Eakin, Winkels, and Sendzimir 2009, 399)	Default	Default
risk of experiencing climate change shock	(Dasgupta and Baschieri 2010)	We use average annual rainfall data, which serves as a proxy for risk of climate-change-related shock.<br>(Dasgupta and Baschieri 2010, 810)	Default	Default
risk-induced poverty	(Günther and Harttgen 2009)	Here, and in the following, idiosyncratic shocks refer to household- specific shocks (e.g., injury, birth, death, or job loss of a household member) that are only weakly correlated across households within a community. Covariate shocks refer to shocks that are correlated across households within communities but only weakly correlated across communities (e.g., natural disasters or epidemics).<br>(Günther and Harttgen 2009, 1231)	Default	Default
Rural household vulnerability	(Sarris and Karfakis 2010)	Thus a household is said to be vulnerable to the outcome of a risk event, if it does not have sufficient	Default	Default

		resources to adequately contend with the risk event. In other words, the extent to which a household is vulnerable to a risk event, namely the extent to which the household can become and/or remain poor or food deprived, depends on the size of the risk event and how effective the household is in managing the risk event.<br>(Sarris and Karfakis 2010, 1)<br>[...]<br>considers vulnerability as the probability of consumption falling below a poverty threshold (Christiaensen and Subbarao 2004, Chaudhuri, et. al. 2002),<br>(Sarris and Karfakis 2010, 4)		
Sefl-organising	(Berkes and Ross 2013)	Not defined	Default	Default
Sensitivity	(Antwi-Agyei et al. 2013)	sensitivity determines the response of a given system to climate change and may be shaped by socioeconomic and ecological conditions of the system (IPCC 2007).<br>(Antwi-Agyei et al. 2013, 905)	Sensitivity A: {(Baca et al. 2014); (Füssel and Klein 2006); (Notenbaert et al. 2013); (Piya, Maharjan, and Joshi 2012); (Sietz, Choque, and Lüdeke 2012)}<br>Sensitivity B: {(Antwi-Agyei et al. 2013)}<br>Variance: {(Luers et al. 2003)}	Sensitivity A: (Füssel and Klein 2006)
	(Baca et al. 2014)	Sensitivity is a measure of how systems could be affected by the change in climate (e.g. how much crop yields change or how much human health might be affected).<br>(Baca et al. 2014, 2)		Sensitivity B: (Antwi-Agyei et al. 2013)
	(Füssel and Klein 2006)	Sensitivity: The degree to which a system is affected, either adversely or beneficially, by climate-related stimuli. [...] The effect may be direct [...]or indirect [...]<br>[...]<br>The sensitivity of a system denotes the (generally multi-factorial and dynamic) dose – response relationship between its exposure to climatic stimuli and the re- sulting impacts.<br>(Füssel and Klein 2006, 314)		
	(Hahn, Riederer, and Foster 2009)	sensitivity is the degree to which the system is affected by the exposure (Hahn, Riederer, and Foster 2009, 75)		
	(Jamir et al. 2013)	Asper theIPCC’s definition and framework, vulnerability<br>is understood as a function of three components—exposure, sensitivity and adaptive capacity. Vulnerability is defined as ‘‘the degree to which a		

		system is susceptible to or unable to cope with, adverse effects of climate change, including cli- mate variability and extremes'' (IPCC 2001).<br>(Jamir et al. 2013, 154)		
	(Luers et al. 2003)	In this example, the sensitivityis represented as the absolute value of the derivative of well-being with respect to the stressor, however, other measures of sensitivitycould be used, for example the coefficient of variations.<br>(Luers et al. 2003, 258)		
	(Notenbaert et al. 2013)	the sensitivity of the livelihood to these risks, (Turner et al. 2003).<br>(Notenbaert et al. 2013, 460)		
	(Piya, Maharjan, and Joshi 2012)	is the degree to which a system is affected, either adversely or beneficially by climate-related stimuli.<br>(Piya, Maharjan, and Joshi 2012, 10)		
	(Sietz, Choque, and Lüdeke 2012)	We consider the effects of weather disturbance on the agricultural systems as sensitivity.<br>(Sietz, Choque, and Lüdeke 2012, 490)		
Shocks	(Calvo and Dercon 2013)	Not defined	Default	Default
Socio-demographic profile	(Hahn, Riederer, and Foster 2009)	Dependency ratio; female headed households; uneducated headed households; households with orphans<br>(Hahn, Riederer, and Foster 2009, 77)	Default	Default
socio-economic conditions	(Westerhoff and Smit 2009)	Not defined	Default	Default
Stimulus	(Ionesco et al. 2009)	The stimuli to which such a system can be subjected are then naturally represented by the inputs to the system. The simplest kind of dynamical system with input is a discrete, deter- ministic one, given by a transition function (see [14]):<br>$f : X \times E \rightarrow X$, (1)<br>(Ionesco et al. 2009, 4)	Default	Default
threat to livelihoods	(Mubaya et al. 2012)	Not defined	Default	Default
use of forecasts	(Marshall 2010)	Seasonal climate forecasts are an example of a supportive technology that can, with variable accuracy, provide probabilistic information about future climate for a period of three to twelve months (Ash et al., 2007; Jones et al., 2000; Tompkins and Adger, 2005). Climate technology may be able	Default	Default

		to assist graziers to minimise losses in drought years and take advantage of favourable seasons (Hayman et al., 2007; Salinger et al., 2005; Hansen, 2002; Eto, 2003; Moss, 2007).<br>(Marshall 2010, 37)		
Vulnerability	(Antwi-Agyei et al. 2013)	Nevertheless, the most commonly accepted approach, which is the approach adopted in this paper, comes from the Intergovernmental Panel on Climate Change (IPCC)'s definition of vulnerability (to climate change) where vulnerability is ''the degree to which an environmental or social system is susceptible to, and unable to cope with, adverse effects of climate change, including climate variability and extremes''<br>(Antwi-Agyei et al. 2013, 904)	Vulnerability A: {(Antwi-Agyei et al. 2013); (Eakin, Winkels, and Sendzimir 2009); (Füssel and Klein 2006); (Ionesco et al. 2009); (Jamir et al. 2013); (Mutsvangwa 2011); (Notenbaert et al. 2013); (Sietz, Choque, and Lüdeke 2012)}<br>Vulnerability B: {(Calvo and Dercon 2013); (Deressa, Hassan, and Ringler 2009)}<br>Vulnerability C: { (Capaldo et al. 2010) }<br>Variance: {(Bogale, Taeb, and Endo 2006)}	Vulnerability A: (Füssel and Klein 2006)
	(Bogale, Taeb, and Endo 2006)	Not defined		Vulnerability B: (Calvo and Dercon 2013)
	(Calvo and Dercon 2013)	In this article, we explore the notion of vulnerability to poverty, closely linked with the magnitude of the threat of poverty, measured ex-ante, before uncertainty has been resolved.<br>[...]<br>To clarify how all these intuitions come together under the concept of vulnerability, this paper proposes an axiomatic approach to themeasurement of both individual and aggregate vulnerability.<br>(Calvo and Dercon 2013, 722)		Vulnerability C: (Capaldo et al. 2010)
	(Capaldo et al. 2010)	conceptual framework drawn from it by Løvendal and Knowles (2005).<br>(Capaldo et al. 2010, 7)		
	(Deressa, Hassan, and Ringler 2009)	Thus, vulnerability is seen as expected poverty, while consumption (income) is used as a proxy for well-being.<br>(Deressa, Hassan, and Ringler 2009, 3)		
	(Eakin et al. 2012)	The concept of vulnerability is closely linked to that of<br>resilience; however, the concepts emerged from different disciplinary traditions and have distinct applications, with implications for the utility of these concepts for different units of analysis (Eakin and Luers 2006; Turner 2010). Vulnerability generally		

		refers to the propensity of some unit of exposure to experience harm. In practice, house- holds are often a convenient unit of analysis for vulnera- bility assessments that aim to differentiate a population in terms of sensitivity to a particular stressor and capacities to effectively respond (Eakin and Luers 2006). At the household level, vulnerability is often evaluated by assessing exposure (the physical relation of the household to a stressor) and sensitivities to the losses experienced (e.g., what the impact means for the household's function and survival), as well as by the households' ability to cope and adapt, or its ''adaptive capacity,'' prior to and after experiencing loss.<br>(Eakin et al. 2012, 477)		
	(Füssel and Klein 2006)	Vulnerability: The degree to which a system is susceptible to, or unable to cope with, adverse effects of climate change, including climate variability and extremes. Vulnerability is a function of the character, magnitude, and rate of climate variation to which a system is exposed, its sensitivity, and its adaptive capacity.<br>(Füssel and Klein 2006, 306)		
	(Ionesco et al. 2009)	Definition (Vulnerability with a reference input) A system $f : X \times E \to X$ in state x is vulnerable to e with respect to the strict partial order $\prec$ and the reference input $e*$ if<br>$f(x, e) \prec f(x, e*)$<br>[...]<br>$\prec$ and the reference scenario $es* \in En$<br>if $xs \prec xs*$<br>Definition (Vulnerability with a reference scenario)A system $f : X \times E \to X$ in state x is vulnerable to input scenario $es \in En$ with respect to the strict partial order<br>(8)<br>where xs and $xs*$ are the trajectories induced by the input scenario and reference scenario, respectively.<br>(Ionesco et al. 2009, 6)		
	(Jamir et al. 2013)	Asper theIPCC's definition and framework, vulnerability<br>is understood as a function of three components—exposure, sensitivity and		

		adaptive capacity. Vulnerability is defined as ‘‘the degree to which a system is susceptible to or unable to cope with, adverse effects of climate change, including cli- mate variability and extremes’’ (IPCC 2001).<br>(Jamir et al. 2013, 154)		
	(Mutsvangwa 2011)	vulnerability as a starting point which focuses on the susceptibility of the household2 (Füssel., 2007). This study takes on the starting point interpretation, which takes the root problem as social vulnerability and examines the current vulnerability of the households as a measure of vulnerability to climate change. Households that are currently vulnerable to food insecurity will find it difficult to cope with adverse impacts of changes in climatic conditions. Thus measuring the likelihood of being food insecure provides a way to examine vulnerability to climate change.<br>(Mutsvangwa 2011, 2)<br>[...]<br>Vulnerability refers to the manner and degree to which a system is susceptible to conditions that negatively affect the well-being of the system. In the climate change field, the IPCC Third Assessment Report defines vulnerability as “the degree to which a system is susceptible to, or unable to cope with, adverse effects of climate change, including climate variability and extremes” (McCarthy et al., 2001).<br>(Mutsvangwa 2011, 15)<br>[...]<br>The differences between these two interpretations of vulnerability are summarized in Table 1.<br>Vulnerability according to the end point interpretation represent the expected net impacts of a given level of global climate change, taking into account feasible adaptations. Vulnerability according to the starting point interpretation focuses on reducing internal socioeconomic vulnerability to any climatic hazard. This study takes on the starting point interpretation.<br>(Mutsvangwa 2011, 17)		
	(Notenbaert	For the purpose of this paper, we work		

	et al. 2013)	with the definition proposed by the Working Group II of the IPCC in the third assess- ment report. We will refer to (1) exposure to climate change impacts, (2) sensitivity to those impacts and (3) the capacity to cope with those impacts as the components of vulnerability. Vulnerability is thus comprised of risks (or a chain of risky events) that people confront in pursuit of their livelihoods, the sensitivity of the livelihood to these risks, the risk response or the options that people have for managing these risks and finally the outcomes that describe the loss in well-being (Turner et al. 2003). (Notenbaert et al. 2013, 460)		
	(Sietz, Choque, and Lüdeke 2012)	Climate vulnerability is considered as a function of expo- sure, sensitivity and coping/adaptive capacity (IPCC 2007). (Sietz, Choque, and Lüdeke 2012, 490)		
Vulnerability as suceptability	(Luers et al. 2003)	we derive a generic vulnerabilitymetric bytranslating a general definition of vulnerability, the susceptibilityto damage, into a mathematical expres- sion. To do this we first define a threshold of damage and then measure susceptibility in terms of the system's sensitivityto and exposure to stressors.We then propose a framework for estimating a system's ability to modify its vulnerable conditions byadapting and responding to changing circumstances. (Luers et al. 2003, 257)	Default	
Vulnerability ipcc	(Hahn, Riederer, and Foster 2009)	Many of these rely heavily on the IPCC working definition of vulnerability as a function of exposure, sensitivity, and adaptive capacity (IPCC, 2001). (Hahn, Riederer, and Foster 2009, 75)	Default	
Vulnerability of coffee farming communities	(Baca et al. 2014)	For our methodology, vulnerability is defined as changes in climate variables that affect agricultural and natural systems over a timeframe. The vulnerability in the livelihoods of small coffee farmers is a function of three factors: exposure, sensitivity and adaptive capacity. (Baca et al. 2014, 2, 3)	Default	
vulnerability to climate change	(CARE 2009)	The degree to which a system is susceptible to, or unable to cope with, adverse effects of climate change, including climate variability and	Default	

		extremes. Vulnerability is a function of the character, magnitude, and rate of climate variation to which a system is exposed, its sensitivity, and its adaptive capacity.5<br>(CARE 2009, 5)		
vulnerability to climate risks	(Ford and Smit 2004)	The conceptual model of community vulnerability to climate change outlined here builds on the literature, conceptualizing vulnerability as a function of exposure of the community to climate-change effects and its adaptive capacity to deal with that exposure.<br>(Ford and Smit 2004, 393)<br>[...]<br>A research framework for empirically applying the model of vulnerability proposed above to Arctic commu- nities is illustrated in Figure 3. The first stage assesses current vulnerability by documenting current exposures and current adaptive strategies. The second stage assesses future vulnerability by estimating directional changes in exposure and predicting future adaptive capacity on the basis of past behavior.<br>(Ford and Smit 2004, 395)	Default	
vulnerability to poverty	(Calvo and Dercon 2013)	Remarking that we are interested in vulnerability to poverty will also be useful to<br>preempt any confusion with vulnerability to downfalls in wellbeing. Our reference point is an absolute poverty norm (e.g. as in Chaudhuri 2003; Suryahadi and Sumarto 2003,or Christiaensen and Subbarao 2005), and not the initial individual position.<br>(Calvo and Dercon 2013, 723)	Default	
Vulnerability to future food insecurity	(Capaldo et al. 2010)	conceptual framework drawn from it by Løvendal and Knowles (2005).<br>(Capaldo et al. 2010, 7)	Default	
Water	(Hahn, Riederer, and Foster 2009)	Sub-constructs: water conflict; natural water source; proximity to water source; inconsistent water supply; inverse water storage<br>(Hahn, Riederer, and Foster 2009, 79)	Default	
welfare indicator	(Mutsvangwa 2011)	This study uses the household’s cereal production levels as a measure of welfare. Farmers in both Gweru and Lupane mainly depend on what they	Default	

		produce for household food security, thus what the households produce is equated to consumptions levels for the household, in this study. (Mutsvangwa 2011) (Mutsvangwa 2011, 21)		
Welfare of rural housholds	(Dasgupta and Baschieri 2010)	Not defined	Default	

Appendix G: Construct mergers made in Step 2.13

Record of construct mergers made in Step 2.13		
**Name of emic construct**	**Representative definition**	**Bridging node**
adaptation strategy	Adaptations, or adaptive strategies, employed by individuals or groups are depicted as being mediated through their relative adaptive capacities, indicating that adaptations may or may not be accessed according to the distribution of various types of resources such as physical or social capital, as developed by Adger and Kelly (1999).<br>(Westerhoff and Smit 2009, 321)	Merged - [adaptation strategy]; [Impacts & responses to Hurricane Stan by coffee farmers]
Impacts & responses to Hurricane Stan by coffee farmers	In this paper, we document household responses to a climatic shock, Stan, to gain insight into how natural resource- dependent communities move to secure their livelihoods following significant loss, the implications of household responses for coffee farming as a ‘‘domain of attraction,’’ as well as to highlight those aspects of household choices and perceptions that may be indicative of resilience at broader scales.<br>(Eakin et al. 2012, 477)	
adaptation to long term climate change	Unique in our study, is the use of individual perceptions in identifying and understanding the processes of adaptation in an area that has undergone significant political and socio-economic reformation resulting from a series of conflicts over land resources.<br>(Gandure, Walker, and Botha 2013, 40)	Merged - [adaptation to long term climate change]; [Farmer perceptions]
Farmer perceptions	there is an alternative approach which underscores how individuals perceive their environment and make decisions, with mal-adaptations attributed to problems in perception, cognition or the lack of available information (Diggs, 1991; Saarinen, 1966; Taylor et al., 1988). The main point is that from whatever level these adapta- tion measures are taken, the adaptation and coping measures depend on households’ perceptions of extreme events and the problems associated with them (Davies, 1993).<br>(Mubaya et al. 2012, 10)	
Current exposure	Exposure is a property of a community relative to climatic conditions. It reflects both the nature of the climatic conditions and nature of the community itself. Some communities may be exposed to a particular climate event whereas the same event may not affect another community. Climatic characteristics include magnitude, frequency, spatial dispersion, duration, speed of onset, and temporal spacing of climatic risks, relating to tem- peratures, precipitation, and wind. The nature of the com- munity concerns its location relative to the climatic risks<br>(Ford and Smit 2004, 393)	Merged - [Current exposure]; [exposure]

exposure	Exposure: The nature and degree to which a system is exposed to significant climatic variations.<br>The exposure of a system to climate stimuli depends on the level of global cli- mate change and, due to the spatial heterogeneity of anthropogenic climate change, on the system's location<br>(Füssel and Klein 2006, 313)	
Expected poverty	This method is based on estimating the probability that a given shock or set of shocks will move household consumption below a given minimum level (such as a consumption poverty line) or force the consumption level to stay below the minimum if it is already below this level (Chaudhuri et al. 2002). (Deressa, Hassan, and Ringler 2009, 3)	Merged - [Expected poverty]; [household vulnerability as expected poverty]; [household vulnerability to poverty]; [rural household vulnerability]; [vulnerability B]; [vulnerability to poverty]
household vulnerability as expected poverty	Household vulnerability as ex- pected poverty is defined as the probability that households will move into poverty given certain environmental shocks, current poverty status and household characteristics of respondents.<br>(Chhihn and Poch 2012, 30)	
household vulnerability to poverty	we can define vulnerability to poverty as the probability of falling into<br>poverty when one's consumption/income falls below a predefined poverty line.<br>(Échevin 2011, 5)	
rural household vulnerability	Thus a household is said to be vulnerable to the outcome of a risk event, if it does not have sufficient resources to adequately contend with the risk event. In other words, the extent to which a household is vulnerable to a risk event, namely the extent to which the household can become and/or remain poor or food deprived, depends on the size of the risk event and how effective the household is in managing the risk event.<br>(Sarris and Karfakis 2010, 1)<br>[...]<br>considers vulnerability as the probability of consumption falling below a poverty threshold (Christiaensen and Subbarao 2004, Chaudhuri, et. al. 2002),<br>(Sarris and Karfakis 2010, 4)	
vulnerability B	In this article, we explore the notion of vulnerability to poverty, closely linked with the magnitude of the threat of poverty, measured ex-ante, before uncertainty has been resolved.<br>[...]<br>To clarify how all these intuitions come together under the concept of vulnerability, this paper proposes an axiomatic approach to themeasurement of both individual and aggregate vulnerability.<br>(Calvo and Dercon 2013, 722)	
vulnerability to poverty	Remarking that we are interested in vulnerability to poverty will also be useful to	

	preempt any confusion with vulnerability to downfalls in wellbeing. Our reference point is an absolute poverty norm (e.g. as in Chaudhuri 2003; Suryahadi and Sumarto 2003,or Christiaensen and Subbarao 2005), and not the initial individual position.<br>(Calvo and Dercon 2013, 723)	
future exposure	Future exposure also includes estimating the future state of the socioeco- nomic conditions, given that exposure is a property of the system relative to risk.<br>(Ford and Smit 2004, 396)	Merged - [future exposure]; [risk of experiencing climate change shock]
risk of experiencing climate change shock	We use average annual rainfall data, which serves as a proxy for risk of climate-change-related shock.<br>(Dasgupta and Baschieri 2010, 810)	
household consumption	consumption falling below a poverty threshold (Christiaensen and Subbarao 2004, Chaudhuri, et. al. 2002)<br>(Sarris and Karfakis 2010, 4)	Merged - [household consumption]; [minimum consumption(income) level]; [poverty]; [poverty line]; [welfare indicator]
minimum consumption(income) level	a given minimum level (such as a consumption poverty line)<br>(Deressa, Hassan, and Ringler 2009, 3)	
Poverty	Technically, the household vulnerability index is derived from the difference between the ex-<br>pected log per capita income and the minimum log per capita income threshold, with households having per capita incomes lower than the minimum per capita income defined as vulnerable (poor). The expected log per capita income is estimated using the three-step feasible generalised least squares (FGLS) method.<br>(Chhihn and Poch 2012, 30)	
poverty line	Our aim is merely to make an ex-ante statement on the vulnerability of the individual to fall below a poverty norm z,<br>(Calvo and Dercon 2013, 724)	
welfare indicator	This study uses the household’s cereal production levels as a measure of welfare. Farmers in both Gweru and Lupane mainly depend on what they produce for household food security, thus what the<br>households produce is equated to consumptions levels for the household, in this study.<br>(Mutsvangwa 2011) (Mutsvangwa 2011, 21)	
Household vulnerability to climate change	Therefore, vulner- ability is the degree to which a system is susceptible or unable to cope with the adverse effects of climate change, including climate variability and extremes. In this regard, vulnerability is a function of the character, magnitude, and rate of climate variation to which a system is ex- posed, its sensitivity, and its adaptive capacity [4].<br>(Tesso, Emana, and Ketema 2012) (Tesso, Emana, and Ketema 2012, 871)	Merged - [Household vulnerability to climate change]; [local level vulnerability]; [vulnreability A]; [vulnerability as suceptability]; [vulnerability ipcc]; [vulnerability of coffee farming communities]; [vulnerability to climate change]
local level vulnerability	Following the definition of vulnerability given by IPCC (2001), vulnerability in this	

	study is taken to be a function of exposure, sensitivity, and adaptive capacity.<br>(Piya, Maharjan, and Joshi 2012, 11)	
vulnreability A	Vulnerability: The degree to which a system is susceptible to, or unable to cope with, adverse effects of climate change, including climate variability and extremes. Vulnerability is a function of the character, magnitude, and rate of climate variation to which a system is exposed, its sensitivity, and its adaptive capacity.<br>(Füssel and Klein 2006, 306)	
vulnerability as suceptability	we derive a generic vulnerabilitymetric bytranslating a general definition of vulnerability, the susceptibilityto damage, into a mathematical expres- sion. To do this we first define a threshold of damage and then measure susceptibility in terms of the system's sensitivityto and exposure to stressors.We then propose a framework for estimating a system's ability to modify its vulnerable conditions byadapting and responding to changing circumstances.<br>(Luers et al. 2003, 257)	
vulnerability ipcc	Many of these rely heavily on the IPCC working definition of vulnerability as a function of exposure, sensitivity, and adaptive capacity (IPCC, 2001).<br>(Hahn, Riederer, and Foster 2009, 75)	
vulnerability of coffee farming communities	For our methodology, vulnerability is defined as changes in climate variables that affect agricultural and natural systems over a timeframe. The vulnerability in the livelihoods of small coffee farmers is a function of three factors: exposure, sensitivity and adaptive capacity.<br>(Baca et al. 2014, 2, 3)	
vulnerability to climate change	The degree to which a system is susceptible to, or unable to cope with, adverse effects of climate change, including climate variability and extremes. Vulnerability is a function of the character, magnitude, and rate of climate variation to which a system is exposed, its sensitivity, and its adaptive capacity.5<br>(CARE 2009, 5)	

Appendix H: Identification of framework-defining constructs (Step 2.14)

Identification of framework-defining bridging constructs (2.14)

IPCC

Baca et al (5)	Luers et al	Piya et al	Care	Fussel & Klein	Hahn et al	Jamir et al	Notenbaert et al	Antwi-Agyei
Vulnerability IPCC	Vulnerability IPCC	Vulnerability IPCC	Vulnerability IPCC	Vulnerability IPCC	Vulnerability IPCC	Vulnerability IPCC	Vulnerability IPCC	Vulnerability IPCC
Exposure (M)	Exposure (M)	Exposure (M)		Exposure (M)	Exposure (M)	Exposure (M)	Exposure (M)	Exposure (M)
Sensitivity A	Sensitivity (Var)	Sensitivity A		Sensitivity A	Sensitivity A	Sensitivity A	Sensitivity A	Sensitivity B
Adaptive Capacity A	Adaptive Capacity A	Adaptive Capacity A	Adaptive Capacity A	Adaptive Capacity A	Adaptive Capacity A	Adaptive Capacity A	Adaptive Capacity A	Adaptive Capacity A

Vulnerability as Expected Poverty

Deressa et al	Sarris & Karfakis	Chhihn & Poch	Calvo & Dercon
Vulnerability as Expected Poverty (M)	Vulnerability as Expected Poverty (M)	Vulnerability as Expected Poverty (M)	Vulnerability as Expected Poverty (M)
Poverty (M)	Poverty (M)	Poverty (M)	Poverty (M)
Climate and non-climate shocks			

Vulnerability as Expected food security

Mutsvangwa	Capaldo et al
Vulnerability as Expected Poverty	Vulnerability (Var)
Poverty	
Food insecurity	
	Expected future food security status
	Future nutritional status

Vulnerability as Expected Poverty – multi-level analysis

Günther & harttgen	Echevin
Vulnerability as Expected Poverty	Vulnerability as Expected Poverty
Idiosyncratic shocks	Idiosyncratic shocks
Covariate shocks	Covariate shocks
Household level	Household level
Community level	Community level

Perceptions of climate change

Mubaya et al	Westerhoff & Smit	Mengitsu	Gandure et al
Farmer perceptions	-		Farmer perceptions
Climate change and variability	-		
Threat to livelihoods	-		
Non-climatic stress	-		
	Adaptive strategy		
		Coping strategy	

Community Resilience (Residual)
default
Choice of property rights regime (Residual)
default
Asset vulnerability (Residual)
default
Disaster resilience of rural livelihoods (Residual)
default
Nested Vulnerability (Residual)
default
Current and future vulnerability (Residual)
default
Livelihood vulnerability index (Residual)
default
Mathematical formalisation of vulnerability (Residual)
default
Regional vulnerability (Residual)
default
Social Resilience (Residual)
default
Intensifying vulnerability to food insecurity (Residual)
default
Nkondze et al (2013) (Residual)
default
Patterns of smallholder vulnerability (Residual)
default
Livelihood trajectories and resilience and vulnerability (Residual)
default
Determinants of Resilience (Residual)
default

Appendix I: Review of frameworks by team member with expertise in field

Report of uniform and discreet Frameworks							
Framework	Description	Main constructs	Articles	Recognise ?	Distinct ?	Useful ?	Any comments
IPCC	This framework is guided by the definition and theory of the IPCC, which conceives of vulnerability to climate change as having three dimensions: Exposure to climate-induced shocks (a natural science phenomenon); the Sensitivity of the unit of analysis to such shocks (a social and natural science phenomenon); the adaptive capacity to deal with such shocks (a social science phenomenon). The framework often but not always creates a context-specific index of vulnerability from indicators of these three dimensions.	Vulnerability (IPCC); Exposure; Sensitivity (A,B); Adaptive Capacity (A)	Antwi-Agyei et al (2013); Baca et al (2004); CARE (2009); Fussel & Klein (2006); Hahn et al (2009); Jamir et al (2013); Luers et al (2013); Notenbaert et al (2013); Piya et al (2012).	Yes/no YES	Distinct/ to be merged	Retain / discard RETAIN	...
Vulnerability as Expected Poverty	This framework conceives of vulnerability as the potential of a unit of analysis (usually a household) becoming or remaining poor in the future. It is an econometric approach that makes forward projections based on cross-sectional data and associated risks of climatic (and sometimes non-climatic) stress. In some cases, assessments of vulnerability based on expected poverty are then regressed against	Vulnerability as Expected Poverty; Poverty	Calvo & Dercon (2012); Chhinh & Poch (2012); Deressa et al (2009);	YES	Merge the three highlighted as variations on a theme.	RETAIN	

	a series of socio-economic data to identify determinants of vulnerability.		Sarris & Karfakis (2010)				
Vulnerability as Expected food security	This is a variant of the framework 'Vulnerability as Expected Poverty' described above. The principal difference is that whereas the former takes its focus as that of current and projected future levels of *poverty*, usually measured through consumption, the current framework by contrast focusses on a household's current and projected future *food security* status.	Vulnerbaility as Expected Poverty;<br>Poverty;<br>Food insecurity;<br>Expected future food security status;<br>Future nutritional status	Capaldo et al (3020); Mutsvangwa (2011)	YES		RETAIN	
Vulnerability as Expected Poverty – multi-level analysis	Another extension of the 'Vulnerability as Expected Poverty' framework described above. This variant is characterised by its inclusion of multi-level analysis. That is, projections are made for units of analysis at two different scales (usually household and community/local), and analysis is done of differences between units at different scales.	Vulnerability as Expected Poverty;<br>Idiosyncratic shocks;<br>Covariate shocks;<br>Household level;<br>Community level	Echevin (2011); Günther & Harttgen (2009)	YES		RETAIN	
Perceptions of climate change	This category constitutes less a coherent framework and more of a collection of studies whose approach differs significantly from the majority of studies in this review in terms of epistemological orientation and position on the intervention cycle. A tentative general description of this category is that the approach focusses on articulating perceptions of people whose livelihoods are affected by climate change (often farmers), and in particular their perceptions of climate change as a physical phenomenon, perceptions of the impact climate change has on their livelihoods, and respondent reported strategies of coping or adaptation.	Farmer perceptions;<br>Adaptation strategy;<br>Coping strategy	Gandure et al (2013); Mengitsu (2011); Mubaya (2012); Westerhoff & Smit (2009)	YES		RETAIN	
Community Resilienc	This framework focusses on a concept of 'resilience' which is built on similar concepts in the fields of psychology and in	Community resilience;<br>Agency;<br>Self organising;	Berkes & Ross (2013)	YES			

e (Residual)	development studies. In particular it is a framework which looks at instances where communities display agency and self-organisation as key aspects of resilience	Adaptive Capacity (B)					
Choice of property rights regime (Residual)	This framework does not take vulnerability as its main focus. The focus instead is on household preferences for property rights regimes (as in, do they prefer private property, public property, or some form of common property), and looks in particular at the determinants, among them vulnerability, leading households to certain choices.	Property rights; Property rights regime; Household choice; Public property; Private property	Bogale et al (2006)	NO	No	No	Vulnerability not enough of a research focal point
Asset vulnerabi lity (Residual)	This framework conveives of household vulnerbility to climate change in terms of the management control that can be exercised over a series of assets. These assets include labour, human capital, non-labour productive assets, household relations, and social capital. A vulnerability index is created through a framework of weighted indicators representing each type of asset.	Household vulnerability to climate change; Asset vulnerability; Future exposure; Communities at risk of climate shocks; Welfare of rural households; Prepared for adverse consequences	Dasgupta & Baschieri (2010)	YES		Appears collapsable with the three vulnerability themed frameworks above	
Disaster resilience of rural livelihood s (Residual)	The focus here is on the adaptiveness of households in terms of their livelihoods and how such livelihoods are affected by disaster. The concept of adaptiveness is taken from the intersection between vulnerability frameworks and resilience frameworks. The framework is operationalised through a case study involving both quantitative (household survey) and qualitative (in-depth interviews) methods. Although the framework looks formally at resilience to *disaster*, it seems likely that the framework is transferable to other forms of climate-related extreme events.	Resilience of rural livelihoods; Resilience; Vulnerability IPCC; Disaster; Adaptation strategy; Adaptiveness;	Eakin et al (2012)	YES		Discard	Disasters vulnerability does not really provide clear indication of how to research long-term changes in CC vulnerability.
Nested	This framework is concerned with	Livelihood vulnerability	Eakin et	YES		Retain	

Vulnerabi lity (Residual)	'teleconnections' between households in geographically distant localities. It examines the mechanisms through which smallholders in distinct geographical contexts respond differently to exogenous shocks (climatic or not-climatic) and in so doing create a new set of influences on distant locations through connections in a nested globally interconnected system.	(B);<br>Nested and teleconnected livelihood vulnerability;<br>Nested system;<br>Cross scalar teleconnections;<br>Exogenous drivers;<br>Response outcomes	al (2008)				
Current and future vulnerabi lity (Residual)	The main characteristics of this framework is its comparison of current and future states of vulnerability. Vulnerability is conceived as being composed of two principal elements: exposure to climatic changes, and adaptive capacity. Multiple data sources are used to generate an assessment of current exposure and current adaptive capacity. On the basis of this data, and on historical social and physical trends, projections are made as to likely future states of exposure and future states of adaptive capacity.	Vulnerability to climate risks;<br>Current vulnerability;<br>Future vulnerability;<br>Current adaptive capacity;<br>Exposure;<br>Future exposure	Ford & Smit (2004)	YES		Retain	
Livelihoo d vulnerabi lity index (Residual)	This framework consists of an index to measure levels of vulnerability. The index is composed of a highly developed set of household-level indicators chosen to represent seven dimensions of a particular conception of 'livelihoods'. These seven dimensions are: socio-demographic profile; livelihood strategies; social network; health; food; water; and natural disaster and climate change.	Livelihood vulnerability (A);<br>Livelihood strategies;<br>Health;<br>Socio-demographic profile;<br>Water;<br>Natural disaster and climate change	Hahn et al (2009)	YES		Retain	
Mathema tical formalisa tion of vulnerabi lity (Residual)	The contribution of this framework is that of mathematical formalisation of the concept of vulnerability. In particular, the concept of vulnerability requires a specification of three elements: an entity that is vulnerable; a stimulus to which the entity is vulnerable; and a preference criteria against which to	Vulnerability IPCC;<br>Entity;<br>Stimulus;<br>Preference criteria;<br>Adaptive capacity (var)<br>Reference scenarios	Ionesco et al (2009)	YES	Merge with IPCC, if this isn't fundamen tally at odds with it. Does it	Yes	This sounds like an elaboration on IPCC, simply trying to design a concrete approach to measurement.

	normatively assess the outcomes of the entity affected by stimulus. The concept of adaptive capacity is also formalised. This framework is influenced by, although it does not closely resemble, the framework of the IPCC.				not resemble IPCC due to the formalizat ion?		
Regional vulnerabi lity (Residual)	The unit of analysis here is a spatial unit, or more precisely, administrative regions or districts that are conceived in geographical or spatial terms. A country-specific index is created with which to quantitatively compare vulnerability levels in different districts. Two interrelated concepts, damage potential and coping capacity, are deconstructed into 5 dimensions of human development: population density, knowledge level, housing standards, living standards, and importance of agriculture as a source of livelihoods. From these five dimensions, regional-level data are collected on a set of indicators, with which to draw conclusions about the vulnerability of a region or district.	Regional vulnerability; Damage potential; Coping capacity; Population density; Lack of decent housing; Lack of decent standard of living	Khan & Salman (2012)	YES	Yes	Discard	Unit of analysis is too far toward national/regional rather than addressing "local" as I understand it.
Social Resilienc e (Residual)	This framework assesses the capacity of units of analysis (e.g. grazers) to cope and adapt to climate variability. This is done through looking at four dimensions: perceptions of risk; capacity to reorganise; proximity to coping thresholds; and interest in change. In particular, the framework assesses adaptive capacity in terms of the use by (in this case grazers) of climate forecasting technology, and levels of dependency on natural resources.	Resilience; Adaptive Capacity (B); Use of forecasts; Resource dependency; Perception of risk; Ability to plan, learn, reorganise	Marshall (2010)	YES		Discard	
Intensifyi ng vulnerabi lity to food	Vulnerability is situated in a recursive framework which captures a cyclical nature of intensification of vulnerability principally through the negative impacts that coping strategies can have on food security.	Livelihood level issues; Access to sufficient food; Food insecurity; Household and	Misselho rn (2005)	YES		Retain	

insecurity (Residual)	Vulnerability is conceived principally in terms of food security, which in turn is conceived in terms of access to food and food productivity. When food security is negatively impacted through climatic and non-climatic drivers, vulnerable households and communities respond with particular coping strategies, which can have a recursive effect on future levels of food security.	community vulnerability;<br>Livelihood strategies;<br>Direct drivers						
Nkondze et al (2013) (Residual)	At a very general level, this framework investigates factors affecting household vulnerability. An index is constructed through which to measure vulnerability, which is then analysed against socio-economic data to determine the most significant factors influencing levels of household vulnerability.	Factors affecting vulnerability;<br>Household vulnerability to climate change	Nkondze et al (2013)	YES		Retain	
Patterns of smallholder vulnerability (Residual)	Cluster pattern analysis is employed in this framework to investigate whether there are, and which, characteristics that explain the causal structure of vulnerability to weather extremes. A measure of household/smallholder vulnerability is created using a combination of IPCC and Food Security household-level indicators. A cluster pattern analysis is then run relating measures of vulnerability to socio-economic and other household-level data to identify characterisitcs, and in particular combinations of characterisitcs that are related to concentrations of vulnerability.	Vulnerability IPCC;<br>Exposure;<br>Sensitivity (A);<br>Adaptive capacity (C);<br>Cluster patter analysis;<br>Food security	Sietz et al (2012)	YES		Retain	
Livelihood trajectories and resilience and vulnerability	On the basis of a mixed methods data collection methodology, the concept of 'livelihood trajectories' is explored among households over a period of (in this case) 30 years. With this long term approach, the framework seeks to generate narrative accounts of which livelihood strategies and trajectories lead to resilient and vulnerable	Resilience and vulnerability of rural livelihoods;<br>Livelihood trajectories;<br>Dynamic natural resource base;<br>Factors influencing resilience and	Sallu et al (2010)	YES		Retain	

(Residual)	states.	vulnerability					
Determinants of Resilience (Residual)	The focus is on identifying determinants of resilience to climate-related shocks. Resilience is conceptualised temporally in terms of the time taken to make a recovery after being impacted by shocks. A vulnerability index (in this case based on the framework of the IPCC) is created to compute measures of vulnerability based on household survey data. Classifications of resilience are then created based on the time taken to return to pre-shock states, which are then analysed against the vulnerability data to identify determinants of resilient housholds.	Vulnerability IPCC; Determinants of Resilience; Household level resilience; Exposure; Adaptive capacity (A);	Tesso et al (2012)	YES		Retain	

Appendix J: Scrutiny by first reviewer of construct splits suggested by expert reviewer

Suggested split	*Previous classificiation*	*Suggested classificiation*	*Sources*	*My conclusions*
Ionesco's 'vulnerability' to be split from 'vulnerability IPCC'	Vulnerability (IPCC)	Vulnerability (IPCC)	Antwi-Agyei et al; Baca et al; CARE (2009); Eakin et al (2012); Fussel & Klein; Hahn et al; Jamir et al; Luers et al; Notnbaert et al; Piya et al; Sietz et al; Tesso et al	Expert split is validated<br>Bridging nodes:<br>Merged - [Household vulnerability to climate change]; [local level vulnerability]; [vulnreability A]; [vulnerability as suceptability]; [vulnerability ipcc]; [vulnerability of coffee farming communities]; [vulnerability to climate change]<br>Vulnerability Ionesco et al
		Vulnerability Ionesco	Ionesco et al	
Jamir et al's & Sietz et al's 'Exposure' not sufficiently well defined to be verifiably of a kind with general concept of 'exposure'	Exposure	Exposure	Antwi-Agyei et al; Baca et al; Ford & Smit; Fussel & Klein; Hahn et al; Luers et al; Notnbaert et al; Piya et al; Tesso et al	Expert split refuted. Candidates are poorly defined. However they do refer to the work of the IPCC, so we may assume that their definitions correspond to those of the others.<br>No bridging nodes required.
		Exposure – Jamir et al	Jamir et al;	
		Exposure – Sietz et al	Sietz et al;	
Sensitivity A: definitions of Jamir et al and Notenbaert et al are not sufficiently well defined to be verifiably placed in the same category	Sensitivity A	Sensitivity A	Baca et al; Fussel & Klein; Hahn et al; Piya et al; Sietz et al;	Expert split partly accepted.<br>Notenbaert to be split; Jamir et al to be retained.<br>Following the logic of the previous construct, Jamir et al's (poor) definition refers to the IPCC and so can be assumed to be consistent with the others. The definition of Notenbaert however does not refer to any literature that the
		Sensitivity – Jamir et al	Jamir et al	
		Sensitivity – Notenbaert et al	Notenbaert et al	

				others do.<br>Bridging nodes:<br>Merged - [Sensitivity A][Sensitivity B]<br>Variance – poor definition \\ Sensitivity
Adaptive capacity A: Definitions of Jamir et al and Notenbaert et al are not sufficiently well defined to be verifiably placed in the same category	Adaptive Capacity A	Adaptive Capacity A	Antwi-Agyei et al; Baca et al; CARE (2009); Fussel & Klein; Hahn et al; Jamir et al; Luers et al; Piya et al; Tesso et al	Expert split partly accepted.<br>Notenbaert to be split; Jamir et al to be retained.<br>See above for reasoning.<br>Bridging nodes:<br>Merged - [Adaptive Capacity A][Adaptive Capacity B][Adaptive Capacity C];<br>Variance – poor definition \\ Adaptive Capacity
		Adaptive Capacity – Jamir et al	Jamir et al	
		Adaptive Capacity – Notenbaert et al	Notenbaert et al	
Vulnerability as Expected Poverty: Not enough information in defintion of Günther & Harttgen to confirm uniformity.	Vulnerability as Expected Poverty	Vulnerability as Expected Poverty	Calvo & Dercon; Chhihn & Poch; Deressa et al; Echevin; Mutsvangwa; Sarris & Karfakis	Expert split refuted.<br>The definition of Günther & Harttgen, although not very specific, does cite a reference that the others also cites (Chaudhuri 2002).<br>No Bridging nodes required
		Vulnerability as Expected Poverty – Günther & Harttgen	Günther & Harttgen	
Poverty: Definitions of Calvo & Dercon; and Deressa et al are too unspecific to confirm uniformity	Poverty	Poverty	Chhihn & Poch; Sarris & Karfakis	Expert split refuted<br>The expert noted poor definitions as the reason that uniformity cannot be verified. Going back to the original reasons for merging these constructs, 'I am aware that they are not all the same. Yet together they all form parts of a coherent construct. This is the nature of this Stage of research – to move from the specifics of author-reported constructs/frameworks, to analyst-generated synthesised representations of the field.' As the definitions do not support a definite split, then I
		Poverty – Calvo & Dercon	Calvo & Dercon	
		Poverty – Deressa et al	Deressa et al	

				consider the logic of merging them to still hold.<br>No bridging nodes required
Household level: Too little information in each of the two definitions	Household level	Household level – Echevin	Echevin	Expert split refuted. Expert judges that there is too little information in the definitions. However, the defintion of Echevin refers to the with or Günther & Harttgen. Therefore they can be considered the same.<br>No bridging nodes required
		Household level – Günther & harttgen	Günther & harttgen	
Community level: Too little information in each of the two definitions	Community level	Community level – Echevin	Echevin	Expert split refuted. Expert judges that there is too little information in the definitions. However, the defintion of Echevin refers to the with or Günther & Harttgen. Therefore they can be considered the same.<br>No bridging nodes required
		Community level - Günther & harttgen	Günther & harttgen	
Farmer perceptions: two examples; one definition is imprecise	Farmer perceptions	Adaptation to long-term climate change	Gandure et al	Expert split refuted. The suggestion to split is not based on a positive detection of divergence, rather on the basis that the definition of Gandure et al does not contain enough information for the classification to be confirmed. The original merger of these constructs was done in part with a view to collecting residuals that shared some broad characterisitics, including that they use broadly defined concepts so as to allow for conceptual development in qualitative research.<br>No bridging nodes
		Farmer perceptions	Mubaya et al	

				required.

Appendix K: Emic-Etic construct map

**Emic-Etic construct map**		
Emic constructs	Bridging construct GS-IT	Etic constructs
ability to plan, learn, reorganise		
access to sufficient food		
adaptation ot long term climate change	Merged - [adaptation to long term climate change]; [Farmer perceptions]	Farmer perceptions
adaptation strategy	Merged - [adaptation strategy]; [Impacts & responses to Hurricane Stan by coffee farmers]	Adaptation Strategy
adaptive capacity	Merged - [Adaptive Capacity A][Adaptive Capacity B][Adaptive Capacity C]	Adaptive Capacity
	Variance – poor definition \\ adaptive capacity	
Adaptiveness		
Agency		
Asset vulnerability		Asset vulnerability
biophyisical conditions		
Climate and non-climate shocks		
climate change		
climate change and variability		
climatic risk factors		
cluster pattern analysis		Cluster pattern analysis
communities at risk of climate shocks		
community level		Community level
Community Resilience		
coping capacity		
coping strategies		
covariate shocks		Covariate shocks
cross-scalar teleconnection		Cross scalar teleconnections
current adaptive capacity		Current Adaptive Capacity
Current exposure	Merged - [Current exposure]; [exposure]	Exposure
current poverty status		
Current vulnerability		Current vulnerability
damage potential		
Determinants of Poverty & Vulnerability		
Determinants of resilience		Determinants of Resilience
Direct drivers		
Disaster		
dynamic natural resource base		
Entity		
environmental shocks		
exogenous drivers		Exogenous drivers
Expected food insecurity		

Expected future food-security status		Expected future food security status
Expected poverty	Merged - [Expected poverty]; [household vulnerability as expected poverty]; [household vulnerability to poverty]; [rural household vulnerability]; [vulnerability B]; [vulnerability to poverty]	Vulnerability as Expected Poverty
Experience of long term climate change		
exposed and sensitive to climate change		
Exposure	Merged - [Current exposure]; [exposure]	Exposure
factors afecting vulnerability		
factors influencing resilience and vulnerability		Factors influencing resilience and vulnerability
Farmer perceptions	Merged - [adaptation to long term climate change]; [Farmer perceptions]	Farmer perceptions
food insecurity		Food Insecurity
food security		Food security
future exposure	Merged - [future exposure]; [risk of experiencing climate change shock]	Future exposure
future nutricional status		Future nutritional status
Future vulnerability		Future vulnerability
Health		Health
household and community vulnerability		Household and community vulnerability
household choice		
household consumption	Merged – [household consumption]; [minimum consumption(income) level]; [poverty]; [poverty line]; [welfare indicator]	Poverty
Household consumption(income)		
household level		Household level
household level resilience		Household level resilience
household vulnerability as expected poverty	Merged - [Expected poverty]; [household vulnerability as expected poverty]; [household vulnerability to poverty]; [rural household vulnerability]; [vulnerability B]; [vulnerability to poverty]	Vulnerability as Expected Poverty
household vulnerability to climate change	Merged - [Household vulnerability to climate change]; [local level vulnerability]; [vulnreability A]; [vulnerability as suceptability]; [vulnerability ipcc]; [vulnerability	Vulnerability IPCC

	of coffee farming communities]; [vulnerability to climate change]	
	Variance – poor definition \\ household vulnerability to climate change	
Household vulnerability to poverty	Merged - [Expected poverty]; [household vulnerability as expected poverty]; [household vulnerability to poverty]; [rural household vulnerability]; [vulnerability B]; [vulnerability to poverty]	Vulnerability as Expected Poverty
idiosyncratic shocks		Idiosyncratic shocks
Impacts & responses to Hurricane Stan by coffee farmers	Merged - [adaptation strategy]; [Impacts & responses to Hurricane Stan by coffee farmers]	Adaptation Strategy
Lack of decent housing		
Lack of decent standard of living		
livelihood level issues		
livelihood risks		
Livelihood strategies		Livelihood strategies
livelihood trajectories		Livelihood trajectories
Livelihood vulnerability	Livelihood vulnerability A	Livelihood vulnerability A
	Livelihood vulnerability B	Livelihood vulnerability B
local level vulnerability	Merged - [Household vulnerability to climate change]; [local level vulnerability]; [vulnreability A]; [vulnerability as suceptability]; [vulnerability ipcc]; [vulnerability of coffee farming communities]; [vulnerability to climate change]	Vulnerability IPCC
minimum consumption(income) level	Merged – [household consumption]; [minimum consumption(income) level]; [poverty]; [poverty line]; [welfare indicator]	Poverty
Natural disasters and Climate variability		Natural disaster and climate change
nested and teleconnected livelihood vulnerability		Nested and teleconnected livelihood vulnerability
Nested system		Nested system
non-claimtic stress		
non-climatic risk factors		
Perception of Adida farmers		
perception of risk		
Population density		
Poverty	Merged – [household consumption]; [minimum consumption(income) level]; [poverty]; [poverty line]; [welfare indicator]	Poverty

Poverty line	Merged – [household consumption]; [minimum consumption(income) level]; [poverty]; [poverty line]; [welfare indicator]	Poverty
preference criteria		
prepared for adverse consequences		
private property		
Propoerty rights		
Propoerty rights regime		
public property		
reference scenarios		
regional vulnerability		
Resilience		
resilience and vulnerability of rural livelihoods		Resilience and vulnerability of rural livelihoods
Resilience of rural livelihoods		
resource dependency		
response outcomes		Response outcomes
risk of experiencing climate change shock	Merged - [future exposure]; [risk of experiencing climate change shock]	Future exposure
risk-induced poverty		
Rural household vulnerability	Merged - [Expected poverty]; [household vulnerability as expected poverty]; [household vulnerability to poverty]; [rural household vulnerability]; [vulnerability B]; [vulnerability to poverty]	Vulnerability as Expected Poverty
Sefl-organising		
Sensitivity	Merged - [Sensitivity A][Sensitivity B]	Sensitivity
	Variance – poor definition \\ Sensitivity	
Shocks		
Socio-demographic profile		Socio-demographic Profile
socio-economic conditions		
Stimulus		
threat to livelihoods		
use of forecasts		
Vulnerability	Merged - [Household vulnerability to climate change]; [local level vulnerability]; [vulnreability A]; [vulnerability as suceptability]; [vulnerability ipcc]; [vulnerability of coffee farming communities]; [vulnerability to climate change]	Vulnerability IPCC
	Merged - [Expected poverty]; [household vulnerability as expected poverty]; [household	Vulnerability as Expected Poverty

	vulnerability to poverty]; [rural household vulnerability]; [vulnerability B]; [vulnerability to poverty]	
	Vulnerability Ionesco et al	
	Variance – poor definition \\ Vulnerability	
Vulnerability as suceptability	Merged - [Household vulnerability to climate change]; [local level vulnerability]; [vulnreability A]; [vulnerability as suceptability]; [vulnerability ipcc]; [vulnerability of coffee farming communities]; [vulnerability to climate change]	Vulnerability IPCC
Vulnerability ipcc	Merged - [Household vulnerability to climate change]; [local level vulnerability]; [vulnreability A]; [vulnerability as suceptability]; [vulnerability ipcc]; [vulnerability of coffee farming communities]; [vulnerability to climate change]	Vulnerability IPCC
Vulnerability of coffee farming communities	Merged - [Household vulnerability to climate change]; [local level vulnerability]; [vulnreability A]; [vulnerability as suceptability]; [vulnerability ipcc]; [vulnerability of coffee farming communities]; [vulnerability to climate change]	Vulnerability IPCC
vulnerability to climate change	Merged - [Household vulnerability to climate change]; [local level vulnerability]; [vulnreability A]; [vulnerability as suceptability]; [vulnerability ipcc]; [vulnerability of coffee farming communities]; [vulnerability to climate change]	Vulnerability IPCC
vulnerability to climate risks		Vulnerability to climate risks
vulnerability to poverty	Merged - [Expected poverty]; [household vulnerability as expected poverty]; [household vulnerability to poverty]; [rural household vulnerability]; [vulnerability B]; [vulnerability to poverty]	Vulnerability as Expected Poverty
vulnerbaility to future food insecurity		
Water		Water
welfare indicator	Merged – [household consumption]; [minimum consumption(income) level]; [poverty]; [poverty line]; [welfare indicator]	Poverty
Welfare of rural housholds		

Appendix L: Records of operationalizations assessed as not transparent or partially transparent

**Structured summary of operationalization – transparency assessment**		
**Construct:** Possible states of the world		
**Article:** Calvo & Dercon (2012)		
Criterion	Assessment	Quoted text or Rationale for negative assessment
Construct defined?	Yes	the probability of low outcomes or overall risk exposure (as defined in Rothschild and Stigliz 1970) increases.
Data collection methods reported?	Yes	To illustrate the insights that could be gained from our individual and aggregate mea- sures of vulnerability,we use three rounds (1994, 1999 and 2004) of a rural household panel data survey from Ethiopia, on 15 villages and about 1,400 households.18<br>[...]<br>Secondly, we identify shocks directly by using data on the historical rainfall distribution and reported shocks such as illness, price and market shocks, and asset losses. Details on specific features of the data (including on sampling, coverage and issues such as the low attrition in the data) can be found in Dercon et al. (2005).
Reporting of indicators/questions used to operationalise construct?	No	Some physical data consists of 2ndary data, and sufficient information is given. However, primary household data is not reported on sufficiently.<br>This is the report of 2ndary data:<br>"Secondly, we identify shocks directly by using data on the historical rainfall distribution and reported shocks such as illness, price and market shocks, and asset losses. Details on specific features of the data (including on sampling, coverage and issues such as the low attrition in the data) can be found in Dercon et al. (2005)."
Sampling strategies reported?	No	Some physical data consists of 2ndary data, and sufficient information is given. However, primary household data is not reported on sufficiently.<br>This is the report of 2ndary data:<br>"Secondly, we identify shocks directly by using data on the historical rainfall distribution and reported shocks such as illness, price and market shocks, and asset losses. Details on specific features of the data (including on sampling, coverage and issues such as the low attrition in the data) can be found in Dercon et al. (2005)."
Sampling sizes reported?	Yes	To illustrate the insights that could be gained from our individual and aggregate mea- sures of vulnerability,we use three rounds (1994, 1999 and 2004) of a rural household panel data survey from Ethiopia, on 15 villages and about 1,400 households.18<br>[...]<br>Secondly, we identify shocks directly by using data on the historical rainfall distribution and reported shocks such as illness, price and market shocks, and asset losses. Details on specific features of the data (including on sampling,

		coverage and issues such as the low attrition in the data) can be found in Dercon et al. (2005).
Data analysis methods reported?	Yes/no	
Conclusion		
Transparency Conclusion:	Not transparent	

**Structured summary of operationalization – transparency assessment**		
**Construct:** Poverty line		
**Article:** Calvo & Dercon (2012)		
Criterion	Assessment	Quoted text or Rationale for negative assessment
Construct defined?	Yes	Our aim is merely to make an ex-ante statement on the vulnerability of the individual to fall below a poverty norm z,
Data collection methods reported?	Yes/no	
Reporting of indicators/questions used to operationalise construct?	No	In their empirical example, they do not define what threshold they use for their poverty norm.
Sampling strategies reported?	Yes/no	
Sampling sizes reported?	Yes/no	
Data analysis methods reported?	Yes/no	
Conclusion		
Transparency Conclusion:	Not transparent	

**Structured summary of operationalization – transparency assessment**		
**Construct:** Probabilities of possible states of the world		
**Article:** Calvo & Dercon (2012)		
Criterion	Assessment	Quoted text or Rationale for negative assessment
Construct defined?	Yes	the probability of low outcomes or overall risk exposure (as defined in Rothschild and Stigliz 1970) increases.
Data collection methods reported?	Yes	To illustrate the insights that could be gained from our individual and aggregate mea- sures of vulnerability,we use three rounds (1994, 1999 and 2004) of a rural household panel data survey from Ethiopia, on 15 villages and about 1,400 households.18<br>[...]<br>Secondly, we identify shocks directly by using data on the historical rainfall distribution and reported shocks such as illness, price and market shocks, and asset losses. Details on specific features of the data (including on sampling, coverage and issues such as the low attrition in the data) can be found in Dercon et al. (2005).
Reporting of indicators/questions used to operationalise construct?	No	Some physical data consists of 2ndary data, and sufficient information is given. However, primary household data is not reported on sufficiently.<br>This is the report of 2ndary data:<br>“Secondly, we identify shocks directly by using data on the historical rainfall distribution and reported shocks such as illness, price and market shocks, and asset losses. Details on

		specific features of the data (including on sampling, coverage and issues such as the low attrition in the data) can be found in Dercon et al. (2005)."
Sampling strategies reported?	No	Some physical data consists of 2ndary data, and sufficient information is given. However, primary household data is not reported on sufficiently.<br>This is the report of 2ndary data:<br>"Secondly, we identify shocks directly by using data on the historical rainfall distribution and reported shocks such as illness, price and market shocks, and asset losses. Details on specific features of the data (including on sampling, coverage and issues such as the low attrition in the data) can be found in Dercon et al. (2005)."
Sampling sizes reported?	Yes	To illustrate the insights that could be gained from our individual and aggregate mea- sures of vulnerability,we use three rounds (1994, 1999 and 2004) of a rural household panel data survey from Ethiopia, on 15 villages and about 1,400 households.18<br>[...]<br>Secondly, we identify shocks directly by using data on the historical rainfall distribution and reported shocks such as illness, price and market shocks, and asset losses. Details on specific features of the data (including on sampling, coverage and issues such as the low attrition in the data) can be found in Dercon et al. (2005).
Data analysis methods reported?	Yes/no	
Conclusion		
Transparency Conclusion:	Not transparent	

**Structured summary of operationalization – transparency assessment**		
**Construct:** Risk management		
**Article:** Capaldo et al (2010)		
Criterion	Assessment	Quoted text or Rationale for negative assessment
Construct defined?	YES	conceptual framework drawn from it by Løvendal and Knowles (2005).
Data collection methods reported?	No	In the theoretical framework, 'Risk' and 'risk management' are subconstructs of 'events' that are to occur at time t_0-t_1. However, the authors only use ex-post data, implying that they only have data for t_0, ie current risk and risk managment:<br>"In this application, we are not able to complement this with information on future risks and risk management strategies."
Reporting of indicators/questions used to operationalise construct?	No	In the theoretical framework, 'Risk' and 'risk management' are subconstructs of 'events' that are to occur at time t_0-t_1. However, the authors only use ex-post data, implying that they only have data for t_0, ie current risk and risk managment:<br>"In this application, we are not able to complement this

		with information on future risks and risk management strategies.”
Sampling strategies reported?	Yes/no	
Sampling sizes reported?	Yes/no	
Data analysis methods reported?	Yes/no	
<u>Conclusion</u>		
Transparency Conclusion:	Not transparent	

**Structured summary of operationalization – transparency assessment**		
**Construct:** Risks		
**Article:** Capaldo et al (2010)		
<u>Criterion</u>	<u>Assessment</u>	<u>Quoted text or Rationale for negative assessment</u>
Construct defined?	YES	conceptual framework drawn from it by Løvendal and Knowles (2005).
Data collection methods reported?	No	In the theoretical framework, ‘Risk’ and ‘risk management’ are subconstructs of ‘events’ that are to occur at time t_0-t_1. However, the authors only use ex-post data, implying that they only have data for t_0, ie current risk and risk managment:<br>“In this application, we are not able to complement this with information on future risks and risk management strategies.”
Reporting of indicators/questions used to operationalise construct?	No	In the theoretical framework, ‘Risk’ and ‘risk management’ are subconstructs of ‘events’ that are to occur at time t_0-t_1. However, the authors only use ex-post data, implying that they only have data for t_0, ie current risk and risk managment:<br>“In this application, we are not able to complement this with information on future risks and risk management strategies.”
Sampling strategies reported?	Yes/no	
Sampling sizes reported?	Yes/no	
Data analysis methods reported?	Yes/no	
<u>Conclusion</u>		
Transparency Conclusion:	Not transparent	

**Structured summary of operationalization – transparency assessment**		
**Construct:** Current poverty status		
**Article:** Chhinh & Poch (2012)		
<u>Criterion</u>	<u>Assessment</u>	<u>Quoted text or Rationale for negative assessment</u>
Construct defined?	Yes	This study adopts the approach to measuring household economic vulnerability posited and elaborated in Chaudhuri’s (2003) study of household vulnerability
Data collection methods reported?	Yes	A total of 600 questionnaires were collected from households.
Reporting of indicators/questions used to operationalise construct?	No	The closest that the paper comes to reporting survey questions is this:<br>“Unlike Chaudhuri (2003), who analysed households’

		monthly per capita consumption expenditure, this study analyses households' monthly income to measure the household vulnerability index due to the lack of expenditure data."<br>However, we still don't know how data on households' monthly income was generated.
Sampling strategies reported?	No	
Sampling sizes reported?	Yes	A total of 600 questionnaires were collected from households.
Data analysis methods reported?	Yes/no	
Conclusion		
Transparency Conclusion:	Not Transparent	

**Structured summary of operationalization – transparency assessment**		
**Construct:** Environmental shocks		
**Article:** Chhinh & Poch (2012)		
Criterion	Assessment	Quoted text or Rationale for negative assessment
Construct defined?	Yes	This study adopts the approach to measuring household economic vulnerability posited and elaborated in Chaudhuri's (2003) study of household vulnerability
Data collection methods reported?	No	This is ambigious. On the one hand, the paper reports that data on natural disasters was obtained from key informant interviews:<br>"Three natural disasters were considered: flash flooding, drought, and windstorms. Areas were defined using Geographical Information Systems, which can be used to produce a Digital El- evation Model. Administrative boundaries were used to define provinces, districts and communes. Natural disaster occurrence was based on information given from key informant interviews from the Sub-national and local authorizes. A total of 600 questionnaires were collected from households."<br>However, later, they report that data on environmental shocks was obtained through asking households if they had experienced flood, windstorms, or drought in the previous 12 years. (see reporting of indicators)
Reporting of indicators/questions used to operationalise construct?	Yes/no	The three natural hazards that impact on people's livelihoods in Cambodia including flood, wind- storms and drought are investigated in this paper. It is important to note that the indicators of these events are measured as dummy, indicating whether the respondents have experienced drought, flood and windstorm over the last 12 years (1999-2010).<br>As indicated in Table 2, an overwhelming majority of respondents have reported experiencing<br>drought ranging in the last 12 years. In the rural communities of Morhasaing, Peang Lvea and Tasal, 100 % of the respondents reported experiencing drought. In contrast, the percentages of respond- ents who have experienced floods or windstorms in those 12 years are significantly lower than those who have experience

		drought.
Sampling strategies reported?	No	
Sampling sizes reported?	No	
Data analysis methods reported?	Yes/no	
Conclusion		
Transparency Conclusion:	NOT TRANSPARENT	

**Structured summary of operationalization – transparency assessment**		
**Construct:** Poverty		
**Article:** Chhinh & Poch (2012)		
Criterion	Assessment	Quoted text or Rationale for negative assessment
Construct defined?	Yes	Technically, the household vulnerability index is derived from the difference between the expected log per capita income and the minimum log per capita income threshold, with households having per capita incomes lower than the minimum per capita income defined as vulnerable (poor). The expected log per capita income is estimated using the three-step feasible generalised least squares (FGLS) method.
Data collection methods reported?	Yes	A total of 600 questionnaires were collected from households.
Reporting of indicators/questions used to operationalise construct?	No	The closest that the paper comes to reporting survey questions is this:<br>"Unlike Chaudhuri (2003), who analysed households' monthly per capita consumption expenditure, this study analyses households' monthly income to measure the household vulnerability index due to the lack of expenditure data."<br>However, we still don't know how data on households' monthly income was generated.
Sampling strategies reported?	No	
Sampling sizes reported?	Yes	A total of 600 questionnaires were collected from households.
Data analysis methods reported?	Yes/no	The expected log per capita income obtained from the above FGLS analysis was used to create vulnerability index at a US $1.00 daily threshold (Cambodia poverty line) and at a US $1.25 daily threshold.
Conclusion		
Transparency Conclusion:	Not transparent	

**Structured summary of operationalization – transparency assessment**		
**Construct:** Exogenous drivers		
**Article:** Eakin et al (2008)		
Criterion	Assessment	Quoted text or Rationale for negative assessment
Construct defined?	Yes	exogenous drivers (i.e. the risk and stress factors)
Data collection methods reported?	Yes/no	In the following sections we draw from a variety of primary and secondary sources to illustrate how the livelihood responses of coffee farmers in Mexico and Vietnam are

		linked through their integration into global markets and the social and environ- mental outcomes of their adaptation choices.<br>[...]<br>Specifically, we draw on two case studies of coffee-producing households and communities in Mexico and Vietnam<br>[...]<br>The Mexican case study took place in 2003, as farmers were emerging from the most recent coffee crisis. The research took place in two coffee-producing communities in the region of Coatepec, in Central Veracruz. In addition to interviews with public officials, coffee association leaders, academics and coffee processors and traders, a household survey collected data on the perceptions and responses of 60 households to the coffee situation (Eakin et al., 2006). The project was part of a broader study exploring the implications of climatic variability and change for coffee farming in Mexico (Gay Garcia et al., 2006). In Vietnam, a study of migrant livelihoods included many<br>coffee farmers, mostly migrants to the Central Highland region (Winkels, 2004). Livelihood surveys and interviews with 81 households originating from the overpopulated Red River Delta in the north provides important insights into both the opportunities andrisk of coffee farming at Vietnam's southern mountain frontier when the first signs of the looming coffee crisis became evident in 2000 and 2001.
Reporting of indicators/questions used to operationalise construct?	No	
Sampling strategies reported?	Yes/no	In this paper we therefore focus our examination on the causes for, and outcomes of, coffee farmers' vulnerability in Mexico and Vietnam in three periods during which global coffee production and trade underwent significant changes. The aim is to highlight not only how global changes affect smallholders in (dis)similar ways across different coffee-producing nations but also in describing how local responses and livelihood vulnerabilities are linked across space and time.
Sampling sizes reported?	Yes/no	two case studies of coffee-producing households and communities in Mexico and Vietnam<br>[...]<br>in three periods during which global coffee production and trade underwent significant changes.
Data analysis methods reported?	No	
Conclusion		
Transparency Conclusion:	No	

**Structured summary of operationalization – transparency assessment**
**Construct:** geographically distant household vulnerability
**Article:** Eakin et al (2008)

Criterion	Assessment	Quoted text or Rationale for negative assessment
Construct defined?	Yes	vulnerabilities and responses of farm households in distinct geographic locations
Data collection methods reported?	Yes/no	In the following sections we draw from a variety of primary and secondary sources to illustrate how the livelihood responses of coffee farmers in Mexico and Vietnam are linked through their integration into global markets and the social and environ- mental outcomes of their adaptation choices.<br>[...]<br>Specifically, we draw on two case studies of coffee-producing households and communities in Mexico and Vietnam<br>[...]<br>The Mexican case study took place in 2003, as farmers were emerging from the most recent coffee crisis. The research took place in two coffee-producing communities in the region of Coatepec, in Central Veracruz. In addition to interviews with public officials, coffee association leaders, academics and coffee processors and traders, a household survey collected data on the perceptions and responses of 60 households to the coffee situation (Eakin et al., 2006). The project was part of a broader study exploring the implications of climatic variability and change for coffee farming in Mexico (Gay Garcia et al., 2006). In Vietnam, a study of migrant livelihoods included many coffee farmers, mostly migrants to the Central Highland region (Winkels, 2004). Livelihood surveys and interviews with 81 households originating from the overpopulated Red River Delta in the north provides important insights into both the opportunities andrisk of coffee farming at Vietnam's southern mountain frontier when the first signs of the looming coffee crisis became evident in 2000 and 2001.
Reporting of indicators/questions used to operationalise construct?	No	
Sampling strategies reported?	Yes/no	In this paper we therefore focus our examination on the causes for, and outcomes of, coffee farmers' vulnerability in Mexico and Vietnam in three periods during which global coffee production and trade underwent significant changes. The aim is to highlight not only how global changes affect smallholders in (dis)similar ways across different coffee-producing nations but also in describing how local responses and livelihood vulnerabilities are linked across space and time.
Sampling sizes reported?	Yes/no	two case studies of coffee-producing households and communities in Mexico and Vietnam<br>[...]<br>in three periods during which global coffee production and trade underwent significant changes.
Data analysis methods reported?	No	

Conclusion		Conclusion
Transparency Conclusion:	No	

Structured summary of operationalization – transparency assessment		
**Construct:** Geographically specific signals of change		
**Article:** Eakin et al (2008)		
Criterion	Assessment	Quoted text or Rationale for negative assessment
Construct defined?	Yes	geographically specific signals of change – such as a shift in market opportunities, a drought, a change in public policy or new form of land use in a specific location –
Data collection methods reported?	Yes/no	In the following sections we draw from a variety of primary and secondary sources to illustrate how the livelihood responses of coffee farmers in Mexico and Vietnam are linked through their integration into global markets and the social and environ- mental outcomes of their adaptation choices.<br>[...]<br>Specifically, we draw on two case studies of coffee-producing households and communities in Mexico and Vietnam<br>[...]<br>The Mexican case study took place in 2003, as farmers were emerging from the most recent coffee crisis. The research took place in two coffee-producing communities in the region of Coatepec, in Central Veracruz. In addition to interviews with public officials, coffee association leaders, academics and coffee processors and traders, a household survey collected data on the perceptions and responses of 60 households to the coffee situation (Eakin et al., 2006). The project was part of a broader study exploring the implications of climatic variability and change for coffee farming in Mexico (Gay Garcia et al., 2006). In Vietnam, a study of migrant livelihoods included many coffee farmers, mostly migrants to the Central Highland region (Winkels, 2004). Livelihood surveys and interviews with 81 households originating from the overpopulated Red River Delta in the north provides important insights into both the opportunities andrisk of coffee farming at Vietnam's southern mountain frontier when the first signs of the looming coffee crisis became evident in 2000 and 2001.
Reporting of indicators/questions used to operationalise construct?	No	
Sampling strategies reported?	Yes/no	In this paper we therefore focus our examination on the causes for, and outcomes of, coffee farmers' vulnerability in Mexico and Vietnam in three periods during which global coffee production and trade underwent significant changes. The aim is to highlight not only how global changes affect smallholders in (dis)similar ways across different coffee-producing nations but also in describing how local responses and livelihood vulnerabilities are linked across

		space and time.
Sampling sizes reported?	Yes/no	two case studies of coffee-producing households and communities in Mexico and Vietnam<br>[...]<br>in three periods during which global coffee production and trade underwent significant changes.
Data analysis methods reported?	No	
Conclusion			Conclusion
Transparency Conclusion:	No	

**Structured summary of operationalization – transparency assessment**		
**Construct:** Household Response		
**Article:** Eakin et al (2008)		
Criterion	Assessment	Quoted text or Rationale for negative assessment
Construct defined?	Yes	factors internal to the household (i.e. ability to mitigate and cope with stress)
Data collection methods reported?	Yes/no	In the following sections we draw from a variety of primary and secondary sources to illustrate how the livelihood responses of coffee farmers in Mexico and Vietnam are linked through their integration into global markets and the social and environ- mental outcomes of their adaptation choices.<br>[...]<br>Specifically, we draw on two case studies of coffee-producing households and communities in Mexico and Vietnam<br>[...]<br>The Mexican case study took place in 2003, as farmers were emerging from the most recent coffee crisis. The research took place in two coffee-producing communities in the region of Coatepec, in Central Veracruz. In addition to interviews with public officials, coffee association leaders, academics and coffee processors and traders, a household survey collected data on the perceptions and responses of 60 households to the coffee situation (Eakin et al., 2006). The project was part of a broader study exploring the implications of climatic variability and change for coffee farming in Mexico (Gay Garcia et al., 2006). In Vietnam, a study of migrant livelihoods included many coffee farmers, mostly migrants to the Central Highland region (Winkels, 2004). Livelihood surveys and interviews with 81 households originating from the overpopulated Red River Delta in the north provides important insights into both the opportunities andrisk of coffee farming at Vietnam's southern mountain frontier when the first signs of the looming coffee crisis became evident in 2000 and 2001.
Reporting of indicators/questions used to operationalise construct?	No	
Sampling strategies reported?	Yes/no	In this paper we therefore focus our examination on the causes for, and outcomes of, coffee farmers' vulnerability in

		Mexico and Vietnam in three periods during which global coffee production and trade underwent significant changes. The aim is to highlight not only how global changes affect smallholders in (dis)similar ways across different coffee-producing nations but also in describing how local responses and livelihood vulnerabilities are linked across space and time.	
Sampling sizes reported?	Yes/no	two case studies of coffee-producing households and communities in Mexico and Vietnam<br>[...]<br>in three periods during which global coffee production and trade underwent significant changes.	
Data analysis methods reported?	No		
Conclusion			Conclusion
Transparency Conclusion:	No		

**Structured summary of operationalization – transparency assessment**		
**Construct:** nested System		
**Article:** Eakin et al (2008)		
Criterion	Assessment	Quoted text or Rationale for negative assessment
Construct defined?	Yes	In a nested system, profoundchanges inkeyvariablesthatoperatenormallyonly at one level, e.g., within a defined geographic region or admin- istrative domain, can have non-linear outcomes for processes operating at broader scales of analysis (Gunderson andHolling, 2001). Local level processes can episodically influence larger scale phenomena, and such explosive ‘‘upward cascades’’ can be sources of surprise at distant locations.
Data collection methods reported?	Yes/no	In the following sections we draw from a variety of primary and secondary sources to illustrate how the livelihood responses of coffee farmers in Mexico and Vietnam are linked through their integration into global markets and the social and environ- mental outcomes of their adaptation choices.<br>[...]<br>Specifically, we draw on two case studies of coffee-producing households and communities in Mexico and Vietnam<br>[...]<br>The Mexican case study took place in 2003, as farmers were emerging from the most recent coffee crisis. The research took place in two coffee-producing communities in the region of Coatepec, in Central Veracruz. In addition to interviews with public officials, coffee association leaders, academics and coffee processors and traders, a household survey collected data on the perceptions and responses of 60 households to the coffee situation (Eakin et al., 2006). The project was part of a broader study exploring the implications of climatic variability and change for coffee farming in Mexico (Gay Garcia et al., 2006). In Vietnam, a

		study of migrant livelihoods included many<br>coffee farmers, mostly migrants to the Central Highland region (Winkels, 2004). Livelihood surveys and interviews with 81 households originating from the overpopulated Red River Delta in the north provides important insights into both the opportunities andrisk of coffee farming at Vietnam's southern mountain frontier when the first signs of the looming coffee crisis became evident in 2000 and 2001.
Reporting of indicators/questions used to operationalise construct?	No	
Sampling strategies reported?	Yes/no	In this paper we therefore focus our examination on the causes for, and outcomes of, coffee farmers' vulnerability in Mexico and Vietnam in three periods during which global coffee production and trade underwent significant changes. The aim is to highlight not only how global changes affect smallholders in (dis)similar ways across different coffee-producing nations but also in describing how local responses and livelihood vulnerabilities are linked across space and time.
Sampling sizes reported?	Yes/no	two case studies of coffee-producing households and communities in Mexico and Vietnam<br>[...]<br>in three periods during which global coffee production and trade underwent significant changes.
Data analysis methods reported?	No	
<u>Conclusion</u>		<u>Conclusion</u>
Transparency Conclusion:	No	

**Structured summary of operationalization – transparency assessment**		
**Construct:** Response outcome		
**Article:** Eakin et al (2008)		
<u>Criterion</u>	<u>Assessment</u>	<u>Quoted text or Rationale for negative assessment</u>
Construct defined?	Yes	outcomes of these responses in terms of individual or household welfare.
Data collection methods reported?	Yes/no	In the following sections we draw from a variety of primary and secondary sources to illustrate how the livelihood responses of coffee farmers in Mexico and Vietnam are linked through their integration into global markets and the social and environ- mental outcomes of their adaptation choices.<br>[...]<br>Specifically, we draw on two case studies of coffee-producing households and communities in Mexico and Vietnam<br>[...]<br>The Mexican case study took place in 2003, as farmers were emerging from the most recent coffee crisis. The research took place in two coffee-producing communities in the region of Coatepec, in Central Veracruz. In addition to interviews with public officials, coffee association leaders,

		academics and coffee processors and traders, a household survey collected data on the perceptions and responses of 60 households to the coffee situation (Eakin et al., 2006). The project was part of a broader study exploring the implications of climatic variability and change for coffee farming in Mexico (Gay Garcia et al., 2006). In Vietnam, a study of migrant livelihoods included many coffee farmers, mostly migrants to the Central Highland region (Winkels, 2004). Livelihood surveys and interviews with 81 households originating from the overpopulated Red River Delta in the north provides important insights into both the opportunities andrisk of coffee farming at Vietnam's southern mountain frontier when the first signs of the looming coffee crisis became evident in 2000 and 2001.
Reporting of indicators/questions used to operationalise construct?	No	
Sampling strategies reported?	Yes/no	In this paper we therefore focus our examination on the causes for, and outcomes of, coffee farmers' vulnerability in Mexico and Vietnam in three periods during which global coffee production and trade underwent significant changes. The aim is to highlight not only how global changes affect smallholders in (dis)similar ways across different coffee-producing nations but also in describing how local responses and livelihood vulnerabilities are linked across space and time.
Sampling sizes reported?	Yes/no	two case studies of coffee-producing households and communities in Mexico and Vietnam<br>[...]<br>in three periods during which global coffee production and trade underwent significant changes.
Data analysis methods reported?	No	
Conclusion		Conclusion
Transparency Conclusion:	No	

Structured summary of operationalization – transparency assessment		
**Construct:** future exposure		
**Article:** Ford & Smit (2004)		
Criterion	Assessment	Quoted text or Rationale for negative assessment
Construct defined?	Yes	Future exposure also includes estimating the future state of the socioeco- nomic conditions, given that exposure is a property of the system relative to risk.
Data collection methods reported?	Yes	Assessing future exposure involves collaboration with the climate science community to estimate the likelihood of changes in cli- matic attributes identified by the community
Reporting of indicators/questions used to operationalise construct?	Yes	Assessing future exposure involves collaboration with the climate science community to estimate the likelihood of changes in cli- matic attributes identified by the community. For exam- ple, will extreme events or climatic variability

		continue to increase? Will the unexpected winds that have caused problems to hunters in many Nunavut communities be- come even stronger and less predictable? Will the storm surges that have damaged infrastructure and sea defenses increase in magnitude or frequency? Which areas will experience most exposure to erosion? Future exposure also includes estimating the future state of the socioeco- nomic conditions, given that exposure is a property of the system relative to risk.
Sampling strategies reported?	No	
Sampling sizes reported?	No	
Data analysis methods reported?	No	
Conclusion		
Transparency Conclusion:	No	

**Structured summary of operationalization – transparency assessment**		
**Construct:** Adaptation to long term climate change		
**Article:** Gandure et al (2013)		
Criterion	Assessment	Quoted text or Rationale for negative assessment
Construct defined?	Yes	Unique in our study, is the use of individual perceptions in identifying and understanding the processes of adaptation in an area that has undergone significant political and socio-economic reformation resulting from a series of conflicts over land resources.
Data collection methods reported?	Yes/no	The study relied on the experience and knowledge of farmers and community members in<br>Gladstone to characterise their livelihood risks fromclimatic and non-climatic risk factors. The groups brain stormed their risks and then ranked them. A total of 13 focus group discussions were organised comprising an average of ninemembers per group. One groupwas composed entirely of youth (6male and 5 female) aged between 20 and 36 years. In general, the groups represented various land and farming types and social groups in Gladstone. A deliberate attempt was made to include farmers from both the new and old land stands and those with and without access to piped water fromcommunity stand pipes within a distance 200m from the house. Two research assistants were selected from the Gladstone community and were trained in data capture and facilitation skills. They assisted in arranging themeetings and provided translation during the facilitation of the focus group discussions.<br>[...]<br>Open ended questions were used to seek information on actions farmers take to adapt to perceived<br>changes in temperature
Reporting of indicators/questions used to operationalise construct?	Yes	Open ended questions were used to seek information on actions farmers take to adapt to perceived<br>changes in temperature and rainfall and whether these

		actions were temporary or permanent. Firstly, farmerswere askedwhether they had changed theirway of life due to climate change. If the answerwas yes, then follow up questions of how they had changed andwhether they felt the changewas temporary or permanent were asked. If the answer was no, the reason(s) for not changing were then probed.
Sampling strategies reported?	No	
Sampling sizes reported?	No	
Data analysis methods reported?	No	Although the paper does report on results of the analysis of the data of this specific construct, nowhere do they mention any methods of data analysis:<br>"The primary adaptation strategies used by farmers in Gladstone include the use of water harvesting techniques; changes in crop planting dates, changes in agriculture practices, and changes in crops grown (Table 2). The use of the various strategies is driven by both climate and non-climatic factors"
Conclusion		
Transparency Conclusion:	No	

**Structured summary of operationalization – transparency assessment**		
**Construct:** Perception of long term climate change		
**Article:** Gandure et al (2013)		
Criterion	Assessment	Quoted text or Rationale for negative assessment
Construct defined?	Yes	Unique in our study, is the use of individual perceptions in identifying and understanding the processes of adaptation in an area that has undergone significant political and socio-economic reformation resulting from a series of conflicts over land resources.
Data collection methods reported?	Yes/no	The study relied on the experience and knowledge of farmers and community members in<br>Gladstone to characterise their livelihood risks fromclimatic and non-climatic risk factors. The groups brain stormed their risks and then ranked them. A total of 13 focus group discussions were organised comprising an average of ninemembers per group. One groupwas composed entirely of youth (6male and 5 female) aged between 20 and 36 years. In general, the groups represented various land and farming types and social groups in Gladstone. A deliberate attempt was made to include farmers from both the new and old land stands and those with and without access to piped water fromcommunity stand pipes within a distance 200m from the house. Two research assistants were selected from the Gladstone community and were trained in data capture and facilitation skills. They assisted in arranging themeetings and provided translation during the facilitation of the focus group discussions.<br>[...]<br>Farmers' perceptions were sought by means of open ended questions on their observations/ experiences of long-term

		changes in temperature and/or rainfall
Reporting of indicators/questions used to operationalise construct?	Yes/no	Farmers' perceptions were sought by means of open ended questions on their observations/ experiences of long-term changes in temperature and/or rainfall. For temperature, farmers' opinions were sought on whether it has become warmer, cooler, more extreme, or no change noted. They could also report any other characteristics noted or say they did not know. Similarly, rainfall could be perceived as wetter, drier, more extreme, no change noted, other characteristics noted or admit to having no knowledge. Additional questions were asked on the manner in which changes occurred and farmers' perceptions of these changes.
Sampling strategies reported?	No	
Sampling sizes reported?	No	
Data analysis methods reported?	No	Although the paper does report on results of the analysis of the data of this specific construct, nowhere do they mention any methods of data analysis:<br>"All groups regardless of age and gender agreed that Gladstone is experiencing long-term changes in rainfall and temperature (Table 1)."
<u>Conclusion</u>		
Transparency Conclusion:	No	

**Structured summary of operationalization – transparency assessment**		
**Construct:** Adaptive Capacity		
**Article:** Jamir et al (2013)		
<u>Criterion</u>	<u>Assessment</u>	<u>Quoted text or Rationale for negative assessment</u>
Construct defined?	Yes	Asper theIPCC's definition and framework, vulnerability is understood as a function of three components—exposure, sensitivity and adaptive capacity. Vulnerability is defined as ''the degree to which a system is susceptible to or unable to cope with, adverse effects of climate change, including cli- mate variability and extremes'' (IPCC 2001).
Data collection methods reported?	Yes	Household questionnaire surveys and participatory rural appraisal (PRA) were conducted in all the five villages in order to quantify each of these indicators. A total of 150 households (30 households in each village) were randomly selected across the villages for the household questionnaire survey. The PRA was in the form of focus group discussions and semi-structured interviews. The group discussions with the community, village council members and district offi- cials gave an insight into the local problems.<br>[...]<br>Based on the response of the farmers and the village council members during household surveys and PRA, the mean, minimum and maximum values for each of the indicators were obtained. Secondary data were used for those indicators that could not be quantified by this approach.
Reporting of indicators/questions used to operationalise	Yes	Table 2 Description and rationale for indicators selected for the vulnerability assessment<br>Component | Indicator | Indicator | Rationale

construct?

indicators	description	units	
Total annual crop production	Total annual crop production in the village of major crops including kharif and rabi crops	Tons/year	The total annual crop production in a village gives an overall indication of the agricultural suitability and growing conditions of crops (soil moisture, water availability, absence of pest attacks) and general food security
Literacy rate	Percentage of literate members in the household	Percentage	The literacy rate among the farmers is indicative of access to non-manual employment and to information regarding overall management in the face of extreme events
Farm income	Total amount of farm income from the agricultural activities carried out by the farmer	INR	Farm income from all agricultural production activities is indicative of the well-being and adaptive capacity of the farmer.
Farm holding size	Total size of the farm used for cultivation by the farmers	Area (ha/ acre/local unit)	Higher farm holding size is reflective of more agricultural production and higher

						adaptive capacity of the farmer
			Farm assets	Total number of tractors, farm equipments, storage facility, manure and pesticides used by the farmer	Number	The farm assets are indicative of the well-being of the farmers and hence adaptive capacity
			Access to health facilities	Distance travelled by the farmers to reach the nearest dispensary/public health centre or hospital	Distance (km)	Distance of the health centers is a major concern especially during a drought or an epidemic
			Access to market	The distance travelled by the farmers to the village or town markets to sell their farm products and procure farm inputs on their own or through some intermediaries.	Distance (km)	Access of farmers to the markets would ensure them proper returns from their agricultural produce as well as paying the required amount of money for procuring farm inputs. This is necessary to prevent the interference and usurping of the farmers money by intermediaries
			Access to banking facilities	Percentage of farmers having an account in the nearest rural banks	Distance (km)	This indicator is reflective of the access of farmers to agricultural credit
			Percentage of area under drought	The percentage of area drought-tolerant crop	Percentage	In those cases, where farmers use

		resistant crops	varieties (traditional ones or those supplied by the State agricultural departments)		drought-tolerant crops, the damage caused during water stressed conditions is minimized to a certain extent.
		Alternative livelihood options from forest, livestock, etc.	Sub-indicators addressing alternate means of earning livelihood (other than crop cultivation, etc.) such as dependence on forests, livestock, etc.		Having an alternate source of income apart from cultivation is necessary for farmers to earn their living during droughts when rainfall deficit affects agricultural yields
		Drinking water availability	Approximate amount of drinking water available during droughts irrespective of source	Liters/ individual	Drinking water is a major concern during droughts as surface water sources dry up and the groundwater tables also lower
		Percentage of households aware of drought preparedness and mitigation measures	Percentage of households having access to newspapers, radio, television, drought awareness programs, etc. taken as proxy	Percentage	High awareness level of the farmer about impending extreme events would give him an idea to make adjustments in the cropping pattern and type of crop to be sown.

<table>
<tr><td></td><td></td><td><table><tr><td>Compensation received from Government due to losses incurred during a drought/famine</td><td>Total amount of compensation received by the drought-affected farmers from the Government agencies, private donor organizations or NGOs</td><td>INR</td><td>This indicator also gives an idea about the institutional structure and Government interventions which are responsible to ensure whether the farmers have received adequate compensation or not</td></tr></table></td></tr>
<tr><td>Sampling strategies reported?</td><td>Yes</td><td>Household questionnaire surveys and participatory rural appraisal (PRA) were conducted in all the five villages in order to quantify each of these indicators. A total of 150 households (30 households in each village) were randomly selected across the villages for the household questionnaire survey.</td></tr>
<tr><td>Sampling sizes reported?</td><td>Yes</td><td>Household questionnaire surveys and participatory rural appraisal (PRA) were conducted in all the five villages in order to quantify each of these indicators. A total of 150 households (30 households in each village) were randomly selected across the villages for the household questionnaire survey.</td></tr>
<tr><td>Data analysis methods reported?</td><td>No</td><td>The construct adaptive capacity is only opertionalized up to the point of data collection. Afterwards, data is analysed according to a conceptual framework in which this construct is not included.</td></tr>
<tr><td colspan="3"><u>Conclusion</u></td></tr>
<tr><td>Transparency Conclusion:</td><td colspan="2">No</td></tr>
</table>

**Structured summary of operationalization – transparency assessment**		
**Construct:** Drought		
**Article:** Jamir et al (2013)		
<u>Criterion</u>	<u>Assessment</u>	<u>Quoted text or Rationale for negative assessment</u>
Construct defined?	Yes	The India Meteorological Department (IMD) defines drought as a rainfall deficit of 25 % or more from the district-level long-period average (LPA).
Data collection methods reported?	Yes	Household questionnaire surveys and participatory rural appraisal (PRA) were conducted in all the five villages in order to quantify each of these indicators. A total of 150 households (30 households in each village) were randomly selected across the villages for the household questionnaire survey. The PRA was in the form of focus group discussions and semi-structured interviews. The group discussions with the community, village council members and district offi-cials gave an insight into the local problems.<br>[...]

<table>
<tr><td></td><td></td><td>Based on the response of the farmers and the village council members during household surveys and PRA, the mean, minimum and maximum values for each of the indicators were obtained. Secondary data were used for those indicators that could not be quantified by this approach.</td></tr>
<tr><td>Reporting of indicators/questions used to operationalise construct?</td><td>Yes</td><td>Table 2 Description and rationale for indicators selected for the vulnerability assessment
<table>
<tr><th>Component indicators</th><th>Indicator description</th><th>Indicator units</th><th>Rationale</th></tr>
<tr><td>Drought duration</td><td>Total amount of time the drought-like conditions persist in the village</td><td>Months</td><td>If the drought-like conditions persist for more days, it would imply more damage in terms of water availability for drinking purposes and irrigation</td></tr>
</table></td></tr>
<tr><td>Sampling strategies reported?</td><td>Yes</td><td>Household questionnaire surveys and participatory rural appraisal (PRA) were conducted in all the five villages in order to quantify each of these indicators. A total of 150 households (30 households in each village) were randomly selected across the villages for the household questionnaire survey.</td></tr>
<tr><td>Sampling sizes reported?</td><td>Yes</td><td>Household questionnaire surveys and participatory rural appraisal (PRA) were conducted in all the five villages in order to quantify each of these indicators. A total of 150 households (30 households in each village) were randomly selected across the villages for the household questionnaire survey.</td></tr>
<tr><td>Data analysis methods reported?</td><td>No</td><td>The construct exposure is only operationalized up to the point of data collection. Afterwards, data is analyzed according to a conceptual framework in which this construct is not included.</td></tr>
<tr><td colspan="3"><u>Conclusion</u></td></tr>
<tr><td>Transparency Conclusion:</td><td colspan="2">No</td></tr>
</table>

<table>
<tr><td colspan="3">Structured summary of operationalization – transparency assessment</td></tr>
<tr><td colspan="3">Construct: Exposure</td></tr>
<tr><td colspan="3">Article: Jamir et al (2013)</td></tr>
<tr><td><u>Criterion</u></td><td><u>Assessment</u></td><td><u>Quoted text or Rationale for negative assessment</u></td></tr>
<tr><td>Construct defined?</td><td>Yes/no</td><td></td></tr>
<tr><td>Data collection methods reported?</td><td>Yes</td><td>Household questionnaire surveys and participatory rural appraisal (PRA) were conducted in all the five villages in</td></tr>
</table>

<table>
<tr><td></td><td></td><td>order to quantify each of these indicators. A total of 150 households (30 households in each village) were randomly selected across the villages for the household questionnaire survey. The PRA was in the form of focus group discussions and semi-structured interviews. The group discussions with the community, village council members and district officials gave an insight into the local problems.
[...]
Based on the response of the farmers and the village council members during household surveys and PRA, the mean, minimum and maximum values for each of the indicators were obtained. Secondary data were used for those indicators that could not be quantified by this approach.</td></tr>
<tr><td>Reporting of indicators/questions used to operationalise construct?</td><td>Yes</td><td>Table 2 Description and rationale for indicators selected for the vulnerability assessment
<table>
<tr><td>Component indicators</td><td>Indicator description</td><td>Indicator units</td><td>Rationale</td></tr>
<tr><td>Extreme climate events</td><td>Number of years experiencing rainfall deficit or droughts taken as a proxy</td><td>Number</td><td>The indicator is used to represent the existing exposure level to climate variability</td></tr>
<tr><td>Drought duration</td><td>Total amount of time the drought-like conditions persist in the village</td><td>Months</td><td>If the drought-like conditions persist for more days, it would imply more damage in terms of water availability for drinking purposes and irrigation</td></tr>
<tr><td>Extent of dryland</td><td>Ratio of the dryland area or non-irrigated agricultural land to the total geographical area of the village</td><td>Number</td><td>One of the most limiting natural resources in drylands is water, and therefore any form of disruption</td></tr>
</table></td></tr>
</table>

		in the normal rainfall pattern or water availability can trigger drought-like conditions. The higher the extent of drylands, higher the vulnerability of the agro-ecosystem
Sampling strategies reported?	Yes	Household questionnaire surveys and participatory rural appraisal (PRA) were conducted in all the five villages in order to quantify each of these indicators. A total of 150 households (30 households in each village) were randomly selected across the villages for the household questionnaire survey.
Sampling sizes reported?	Yes	Household questionnaire surveys and participatory rural appraisal (PRA) were conducted in all the five villages in order to quantify each of these indicators. A total of 150 households (30 households in each village) were randomly selected across the villages for the household questionnaire survey.
Data analysis methods reported?	No	The construct exposure is only operationalized up to the point of data collection. Afterwards, data is analyzed according to a conceptual framework in which this construct is not included.
<u>Conclusion</u>		
Transparency Conclusion:	No	

**Structured summary of operationalization – transparency assessment**		
**Construct:** Sensitivity		
**Article:** Jamir et al (2013)		
<u>Criterion</u>	<u>Assessment</u>	<u>Quoted text or Rationale for negative assessment</u>
Construct defined?	Yes/no	
Data collection methods reported?	Yes	Household questionnaire surveys and participatory rural appraisal (PRA) were conducted in all the five villages in order to quantify each of these indicators. A total of 150 households (30 households in each village) were randomly selected across the villages for the household questionnaire survey. The PRA was in the form of focus group discussions and semi-structured interviews. The group discussions with the community, village council members and district offi- cials gave an insight into the local problems.<br>[...]<br>Based on the response of the farmers and the village council members during household surveys and PRA, the mean,

<table>
<tr><td></td><td></td><td>minimum and maximum values for each of the indicators were obtained. Secondary data were used for those indicators that could not be quantified by this approach.</td></tr>
<tr><td>Reporting of indicators/questions used to operationalise construct?</td><td>Yes</td><td>Table 2 Description and rationale for indicators selected for the vulnerability assessment
<table>
<tr><th>Component indicators</th><th>Indicator description</th><th>Indicator units</th><th>Rationale</th></tr>
<tr><td>Area under shifting cultivation</td><td>Total area under shifting cultivation with less fallow periods (2–4 years)</td><td>Area (ha/ acre/ local unit)</td><td>Due to increasing requirement for cultivation of land in Northeast India, the fallow period in a shifting cultivation cycle has reduced from 20–30 to 2–3 years. This has adverse impacts on the ecosystem, and the land is increasingly deteriorating. Also, during the ‘slashing and burning, cycle, the forests emit carbon dioxide which can prove quite harmful in the long run unless the lost forests are replaced through plantation activities. Only that area under shifting cultivation has been considered where the fallow periods are less (2–4 years). The rationale for taking this is because the shortened fallow does not allow the recovery of nutrients necessary for crop production, and this intensification is causing shifting cultivation to become unsustainable. The net result is an increase in degraded lands that support neither crops nor</td></tr>
</table></td></tr>
</table>

						forests and a gradual reduction in carbon stocks
			Total area under rainfed agriculture	The total area cultivated by the farmers, which is dependent directly on rainfall for irrigation (whether under settled or shifting cultivation)	Area (ha/ acre/ local unit)	Greater the area under rainfed crops, greater is the dependence on rainfall. Hence, any change in rainfall pattern would influence the crop production thereby increasing the vulnerability
			Total area under irrigated crops	The total agriculture area under manually irrigated crops (during kharif as well as rabi seasons)	Area (ha/ acre/ local unit)	More the area under irrigated crops, lesser the dependence on rainfall for agricultural activities—it is assumed that irrigated crops are not directly dependant on rainfall variability or much affected by drought
			Irrigation availability	Total number of days irrigation available per year	Days/year	This indicator determines whether enough water (whether groundwater/surface water) is available for the irrigated crops. Lesser number of days for which irrigation water is available would indicate a water stress situation for the village
			Average crop diversity index	The inverse of (the number of crops grown by a household	Number	This gives an estimate whether mono-cropping is practiced or the farmer grows multiple crops. In a

				?1)		climate change scenario, multi-cropping is preferable
			Total number of kharif crops grown	Total number of crops grown during kharif or rainfed season of the year	Number	Kharif crops are grown with the onset of monsoon/ rainfall season, and these are harvested during September–October. Examples include paddy, soyabean. Since these crops are mostly rainfall dependent, any change in the rainfall pattern (deficit) is likely to affect Kharif crop yield and production
			Total number of rabi crops grown	Total number of crops grown during rabi and zaid seasons of the year (non-major cropping seasons)	Number	Rabi crops are mostly winter crops sown during October–December and harvested during April. Examples are wheat, gram, peas. Growing more of rabi crops is an alternative way to minimize crop production losses due to erratic rainfall pattern
			Rural population density	Total rural population of the village divided by the geographical area of the village	Percentage	Higher the rural population density within a region, greater will be the exposure to climate variability and change
			Percentage of small-scale farmers	Percentage of small-scale farmers (with land holding between 1.0 and 1.99 ha)	Percentage	The more is the number of small land holdings, higher the risk of damage to extreme events and subsequent losses
			Percentage	Percentage	Percentage	The more is the

		of marginal farmers	of marginal farmers (with land holding\1ha)		number of marginal land holdings, higher the risk of damage to extreme events and subsequent losses
		Crop area affected	Total area under cultivation affected by droughts	Area (ha/ acre/local unit)	More croplands affected by droughts would imply higher damage and losses in terms of agricultural produce
		Value of crops lost	The type and amount of crop sown and its market price during the time of crop loss taken as proxy	INR	Loss of agricultural produce due to droughts would mean reduction in farm income and higher vulnerability of the farmer
Sampling strategies reported?	Yes	Household questionnaire surveys and participatory rural appraisal (PRA) were conducted in all the five villages in order to quantify each of these indicators. A total of 150 households (30 households in each village) were randomly selected across the villages for the household questionnaire survey.			
Sampling sizes reported?	Yes	Household questionnaire surveys and participatory rural appraisal (PRA) were conducted in all the five villages in order to quantify each of these indicators. A total of 150 households (30 households in each village) were randomly selected across the villages for the household questionnaire survey.			
Data analysis methods reported?	No	The construct sensitivity is only operationalized up to the point of data collection. Afterwards, data is analyzed according to a conceptual framework in which this construct is not included.			
Conclusion					
Transparency Conclusion:	No				

**Structured summary of operationalization – transparency assessment**		
**Construct:** Adaptive Capacity		
**Article:** Marshall		
Criterion	Assessment	Quoted text or Rationale for negative assessment
Construct defined?	Yes	It refers to the ability of individuals or communities to adapt to adversity and stressful life-events by 'reorganising' through networks or institutions that learn, store knowledge and experience and are creative, flexible and novel in their approach to problem solving (Vayda and McCay, 1975; McCay, 1981; Sonn and Fisher, 1998).
Data collection methods reported?	Yes	Survey questions were developed so as to quantify a grazier's capacity to adapt to climate variability, their level of dependency

		on the resource and their likely uptake of seasonal climate forecasts (Marshall, 2008).
Reporting of indicators/questions used to operationalise construct?	No	"Some questions within the survey, such as 'in what year were you born?', required simple answers. Some questions such as, 'are you employed as a land manager on someone else's land?' required a 'yes' or 'no' answer. Answers to most questions, however, were expressed as a statement and reflected an attitude, opinion or stance. For example, one statement was, ''I do not talk about strategies to survive drought much with others''. Respondents were asked to rate how strongly they agreed with each statement using a 4-point rating scale (1 = strongly disagree, 2 = disagree, 3 = agree, 4 = strongly agree). This scale builds upon the Likert scale (Mueller, 1986; Likert, 1932) and is especially useful in quantifying and comparing attitudes,"<br>- Insufficient reporting of survey questions.
Sampling strategies reported?	Yes	2.2. Study site selection<br>In the Australian rangelands drought is a 'normal' characteristic for cattle producers (or graziers). In Queensland, for example, drought was declared 15 times between 1965 and 1989 and in some parts (e.g. the Burdekin region) drought can be a continual state for up to 34% of time (McKeon et al., 2000; Johnston et al., 2000). The survey, in this study, was conducted in the Upper Burdekin dry tropics region which is located in north eastern Queensland and covers an area of about 36,000 km2 (see Fig. 1). It is a sub-catchment of the Burdekin River, one of the largest rivers in the state. The high rainfall variability of the region is strongly correlated ahead of time with relatively well understood aspects of ENSO, making forecasting relatively beneficial for those who choose to use it (Ash et al., 2007). The climate is characterised by pronounced wet and dry seasons, with most rain falling between November and April. Average rainfall ranges between 650 and 1500 mm annually (Stokes et al., 2006). Other than some basalt soils, most soils in the region have low levels of nitrogen,

		organic<br>matter and fertility (Stokes et al., 2006).<br>[...]<br>2.4. Survey administration<br>An intensive media campaign commenced the survey administration<br>phase to introduce the research to the region. Next,<br>names, addresses and telephone numbers of graziers were obtained from the yellow pages; an online business directory.<br>All grazing families with the Dalrymple Shire received a personal<br>letter informing them of the research and inviting them to participate.<br>The final version of the survey was administered to 100 graziers<br>in their homes by two interviewers working as a team between<br>March 2007 and June 2007.
Sampling sizes reported?	Yes	The final version of the survey was administered to 100 graziers<br>in their homes<br>[...]<br>Of the 103 families that were contacted, 100 agreed to participate in the research. Hence a<br>response rate of 97% was achieved for the study. There are around<br>120–130 grazing families that live and work on the 230 properties<br>within the region (many properties are owned by the same grazing<br>family) so that results from this study represent at least 77% of the<br>region (Greiner et al., 2003).
Data analysis methods reported?	Yes	Quantitative data were analysed using standard statistical techniques (using SPSS1). Responses to each survey question are<br>described in the text and the overall resilience to climate variability on all four dimensions is presented as a mean of the<br>mean responses for each dimension. The influence of resource<br>dependency, and likely uptake on each component of adaptive<br>capacity was quantified using Pearson correlations. A ‘ weighted<br>mean’ or F-score was calculated for the set of relevant statements<br>for each component of resource dependency and social resilience.<br>Pearson correlations were made between uptake and the F-scores<br>for each conceptual variable. Bonferroni adjustments were made to<br>offset the chance of a false rejection of the null hypothesis in

		a large<br>number of separate t-tests.
Conclusion		
Transparency Conclusion:	No	

**Structured summary of operationalization – transparency assessment**		
**Construct:** Livelihood assets		
**Article:** Notenbaert et al (2013)		
Criterion	Assessment	Quoted text or Rationale for negative assessment
Construct defined?	Yes	(Turner et al. 2003).
Data collection methods reported?	Yes	A detailed household survey was used to elicit household responses (n = 184) about their available resources, live- lihood sources and coping strategies to climate variability.
Reporting of indicators/questions used to operationalise construct?	No	The text provides the following outline of the survey sections, but does not report the actual questions or indicators:<br>The questionnaire was divided into the following five sections: (1) household composition, livelihood strategies and livestock assets; (2) household livestock ownership, herd dynamics and species; (3) livestock feeding tech- niques, management, products and markets, (4) welfare outcomes (income, food consumption and health) and (5) focused on the main concerns/challenges facing households and their coping strategies. In this last section, households were asked to list and rank their concerns and describe the coping strategies employed to counter these concerns.
Sampling strategies reported?	No	
Sampling sizes reported?	Yes	household survey was used to elicit household responses (n = 184)
Data analysis methods reported?	Yes/no	These pieces of information taken all together, allowed us to come up with a household-level vulnerability index, assessing the degree of a household's vulnerability to climate change and variability in relation to other households in the same village. The index is not based on thresholds nor does it represent an absolute value. It is a relative measure, representing the households' own perception of how they have been coping in the past as compared to other households. For each of the concerns a household listed, an impact<br>factor (Ii) was established. This impact factor takes the value of ?1 if the household considered itself coping less well than the other households, -1 if it was doing better and 0 if they assessed themselves similar to the other households in the village. The rationale being that house- holds that are coping less than others, are more vulnerable, while the ones that are doing better than other households have a lower vulnerability. The concerns listed are not all of equal importance. To correct for this, we established a weight for each of the concerns based on the rank they were assigned across all the sampled households. If a household reported n concerns, the vulnerability of a household was then calculated following formula below. Formula 1:

		v ¼X n<br>i¼1<br>n = number of concerns, wi = weight of concerns, Ii = impact (?1: worse than/0: same/- 1:better).
Conclusion		
Transparency Conclusion:	NOT TRANSPARENT	

**Structured summary of operationalization – transparency assessment**		
**Construct:** Livelihoods		
**Article:** Notenbaert et al (2013)		
Criterion	Assessment	Quoted text or Rationale for negative assessment
Construct defined?	Yes	(Turner et al. 2003).
Data collection methods reported?	Yes	A detailed household survey was used to elicit household responses (n = 184) about their available resources, live- lihood sources and coping strategies to climate variability.
Reporting of indicators/questions used to operationalise construct?	No	The text provides the following outline of the survey sections, but does not report the actual questions or indicators:<br>The questionnaire was divided into the following five sections: (1) household composition, livelihood strategies and livestock assets; (2) household livestock ownership, herd dynamics and species; (3) livestock feeding tech- niques, management, products and markets, (4) welfare outcomes (income, food consumption and health) and (5) focused on the main concerns/challenges facing households and their coping strategies. In this last section, households were asked to list and rank their concerns and describe the coping strategies employed to counter these concerns.
Sampling strategies reported?	No	
Sampling sizes reported?	Yes	household survey was used to elicit household responses (n = 184)
Data analysis methods reported?	Yes/no	These pieces of information taken all together, allowed us to come up with a household-level vulnerability index, assessing the degree of a household's vulnerability to climate change and variability in relation to other households in the same village. The index is not based on thresholds nor does it represent an absolute value. It is a relative measure, representing the households' own perception of how they have been coping in the past as compared to other households. For each of the concerns a household listed, an impact<br>factor (Ii) was established. This impact factor takes the value of ?1 if the household considered itself coping less well than the other households, -1 if it was doing better and 0 if they assessed themselves similar to the other households in the village. The rationale being that house- holds that are coping less than others, are more vulnerable, while the ones that are doing better than other households have a lower vulnerability. The concerns listed are not all of equal importance. To correct for this, we established a weight for each of the concerns based on the rank they were assigned across all the sampled households. If a household reported n concerns, the vulnerability of a household

		was then calculated following formula below. Formula 1:<br>v ¼X n<br>i¼1<br>n = number of concerns, wi = weight of concerns, Ii = impact (?1: worse than/0: same/- 1:better).
Conclusion		
Transparency Conclusion:	NOT TRANSPARENT	

Structured summary of operationalization – transparency assessment		
**Construct:** Vulnerability Outcomes		
**Article:** Notenbaert et al (2013)		
Criterion	Assessment	Quoted text or Rationale for negative assessment
Construct defined?	Yes	the outcomes that describe the loss in well-being (Turner et al. 2003).
Data collection methods reported?	Yes	A detailed household survey was used to elicit household responses (n = 184) about their available resources, live- lihood sources and coping strategies to climate variability.
Reporting of indicators/questions used to operationalise construct?	No	The text provides the following outline of the survey sections, but does not report the actual questions or indicators:<br>The questionnaire was divided into the following five sections: (1) household composition, livelihood strategies and livestock assets; (2) household livestock ownership, herd dynamics and species; (3) livestock feeding tech- niques, management, products and markets, (4) welfare outcomes (income, food consumption and health) and (5) focused on the main concerns/challenges facing households and their coping strategies. In this last section, households were asked to list and rank their concerns and describe the coping strategies employed to counter these concerns.
Sampling strategies reported?	No	
Sampling sizes reported?	Yes	household survey was used to elicit household responses (n = 184)
Data analysis methods reported?	Yes/no	These pieces of information taken all together, allowed us to come up with a household-level vulnerability index, assessing the degree of a household's vulnerability to climate change and variability in relation to other households in the same village. The index is not based on thresholds nor does it represent an absolute value. It is a relative measure, representing the households' own perception of how they have been coping in the past as compared to other households. For each of the concerns a household listed, an impact<br>factor (Ii) was established. This impact factor takes the value of ?1 if the household considered itself coping less well than the other households, -1 if it was doing better and 0 if they assessed themselves similar to the other households in the village. The rationale being that house- holds that are coping less than others, are more vulnerable, while the ones that are doing better than other households have a lower vulnerability. The concerns listed are not all of equal importance. To correct for this, we established a weight for each of the concerns based on the rank they were assigned across all the sampled households. If a household reported n concerns, the vulnerability of a household was then calculated following formula below. Formula 1:<br>v ¼X n<br>i¼1

		n = number of concerns, wi = weight of concerns, Ii = impact (?1: worse than/0: same/- 1:better).
Conclusion		
Transparency Conclusion:	NOT TRANSPARENT	

Structured summary of operationalization – transparency assessment		
**Construct:** factors influencing resilience and vulnerability		
**Article:** Sallu et al		
Criterion	Assessment	Quoted text or Rationale for negative assessment
Construct defined?	Yes	Through comparative research we provide a rich contextual narrative and use it to explore those factors that in isolation and combination push livelihoods along particular "trajectories" towards vulnerability or resilience.
Data collection methods reported?	Yes	Methods used included oral histories and in-depth livelihood trajectory mapping exercises (n = 17), as well as household-level livelihood and resource use surveys (n = 98). These sought to identify the ways in which households use their environment, how environmental changes (drought, land degradation, etc.) affect livelihood decisions, and how environmental factors interact with broader socioeconomic and political processes to determine resource use outcomes and impacts on livelihood systems.
Reporting of indicators/questions used to operationalise construct?	No	"These sought to identify the ways in which households use their environment, how environmental changes (drought, land degradation, etc.) affect livelihood decisions, and how environmental factors interact with broader socioeconomic and political processes to determine resource use outcomes and impacts on livelihood systems."<br>- Insufficient documentation of household surveys
Sampling strategies reported?	Yes	Data were collected in 2004 and 2005 when fieldwork was carried out as part of a larger research project that considered environmental, socioeconomic, and institutional dynamics in two of Botswana's remote rural settlements, Khawa and Kedia settlements in Central and Kgalagadi Districts, respectively (Fig. 1). These settlements were chosen for comparison because they were of similarly low economic status and were classified by the government as "remote area dweller" settlements, yet were representative of distinct social-ecological systems with different environmental contexts, social compositions, and histories.
Sampling sizes reported?	Yes	in two of Botswana's

		remote rural settlements,<br>[...]<br>A mixed-method approach was taken in collecting the data. Methods used included oral histories and in-depth livelihood trajectory mapping exercises (n = 17), as well as household-level livelihood and resource use surveys (n = 98).
Data analysis methods reported?	Yes	Data analysis was conducted throughout the period of information gathering. Initially, this was at a descriptive level in order to note any trends in the data, but it progressed to a more detailed level as both qualitative and quantitative social and environmental information was drawn together. Qualitative data were coded through processes of indexing the data under emerging themes. This permitted the identification of the factors that played an important role in the construction of livelihood strategies. Consistent triangulation of the results highlighted any contradictions and similarities in the different data sources. Where contradictions were found, further iterative reflection took place in the form of focus groups in order to ascertain why and how the conflicts in information may have occurred. This became a circular process that led to inductive interpretation and explanation as the ecological information was gradually juxtaposed within the emergent socioeconomic context.
Conclusion		
Transparency Conclusion:	no	

**Structured summary of operationalization – transparency assessment**		
**Construct:** livelihood trajectories		
**Article:** Sallu et al		
Criterion	Assessment	Quoted text or Rationale for negative assessment
Construct defined?	Yes	Bagchi et al. (1998) use the term “livelihood trajectories” to describe and explain the direction and pattern of livelihoods of individuals or groups of people (e.g., households). A livelihood trajectory approach allows the examination of an individual household’s “strategic behavior that is embedded in a historical repertoire, in social differentiation” (de Haan and Zoomers 2005), and in perceptions of risk. Such an approach is sensitive to life histories (an individual’s own “story” of their changing livelihoods).
Data collection methods reported?	Yes/no	Methods used included oral histories and in-depth livelihood trajectory mapping exercises (n = 17), as well as household-level livelihood and resource use surveys (n = 98). These sought to identify the ways in which households use their environment, how environmental changes (drought,

		land degradation, etc.) affect livelihood decisions, and how environmental factors interact with broader<br>socioeconomic and political processes to determine<br>resource use outcomes and impacts on livelihood<br>systems.
Reporting of indicators/questions used to operationalise construct?	No	"These sought to<br>identify the ways in which households use their<br>environment, how environmental changes (drought,<br>land degradation, etc.) affect livelihood decisions, and how environmental factors interact with broader<br>socioeconomic and political processes to determine<br>resource use outcomes and impacts on livelihood<br>systems."<br>- Insufficient documentation of household surveys and livelihood trajectory mapping exercise
Sampling strategies reported?	Yes	Data were collected in 2004 and 2005 when<br>fieldwork was carried out as part of a larger research<br>project that considered environmental, socioeconomic,<br>and institutional dynamics in two of Botswana's<br>remote rural settlements, Khawa and Kedia<br>settlements in Central and Kgalagadi Districts,<br>respectively (Fig. 1). These settlements were chosen<br>for comparison because they were of similarly low<br>economic status and were classified by the government as "remote area dweller" settlements,<br>yet were representative of distinct social-ecological<br>systems with different environmental contexts,<br>social compositions, and histories.
Sampling sizes reported?	Yes	in two of Botswana's<br>remote rural settlements,<br>[...]<br>A mixed-method approach was taken in collecting<br>the data. Methods used included oral histories and<br>in-depth livelihood trajectory mapping exercises (n<br>= 17), as well as household-level livelihood and<br>resource use surveys (n = 98).
Data analysis methods reported?	Yes	Data analysis was conducted throughout the period<br>of information gathering. Initially, this was at a<br>descriptive level in order to note any trends in the<br>data, but it progressed to a more detailed level as<br>both qualitative and quantitative social and<br>environmental information was drawn together.<br>Qualitative data were coded through processes of<br>indexing the data under emerging themes. This<br>permitted the identification of the factors that played<br>an important role in the construction of livelihood<br>strategies. Consistent triangulation of the results<br>highlighted any contradictions and similarities in<br>the different data sources. Where contradictions<br>were found, further iterative reflection took place<br>in the form of focus groups in order to ascertain why<br>and how the conflicts in information may have

		occurred. This became a circular process that led to inductive interpretation and explanation as the ecological information was gradually juxtaposed within the emergent socioeconomic context.
Conclusion		
Transparency Conclusion:	no	

**Structured summary of operationalization – transparency assessment**		
**Construct:** Sensitivity		
**Article:** Baca et al (2004)		
Criterion	Assessment	Quoted text or Rationale for negative assessment
Construct defined?	Yes	Sensitivity is a measure of how systems could be affected by the change in climate (e.g. how much crop yields change or how much human health might be affected).
Data collection methods reported?	Yes	The indicators were used to assess the vulnerability of coffee farms in each country. From a population of 7,000 farmer members from 15 organizations across the four countries, 558 farmers were interviewed.
Reporting of indicators/questions used to operationalise construct?	Inconclusive – info to be requested from authors	Parameters were then constructed to evaluate each indicator as shown in Table S1 in File S1. To quantify the parameters scales from 1 to 5 were applied or a binary scale of 0 and 1, depending on the nature of the parameter. The final values for each indicator were calculated by averaging all the parameters and then transformed to a 0-1 continuous variable scale, with 0 being low and 1 being high sensitivity and adaptive capacity.<br>[...]<br>**Indicator** — **Parameter**<br>Conservation — Area of forest around the water source; Area of forest to keep in the farm<br>Soil and fertility — Soil type; Soil slope; Mulch of leaves; Soil depth<br>Access to and availability of water — Source of water for drinking or postharvest processing; Availability of water during the year; Distance to the water source; Water quality<br>Variability of annual coffee production — Average farm yield in four years compared to the local average<br>Road type — Time from the farm to the collection center; Time from the farm to the nearest market; Type of road from the farm to the collection center or nearest market<br>Transport of products — Type of transportation from the farm to the market; Time from the farm to the bus stop<br>Housing — Housing material

		quality	Basic services
		Health and food	Number of symptoms of human disease<br>Number of times that person is attended by a doctor<br>Dependency of external products
		Migration	Type and time

Sampling strategies reported?	Yes	From a population of 7,000 farmer members from 15 organizations across the four countries, 558 farmers were interviewed. The farmers may be considered representative of small-scale organized farmers, but should not be considered representative of the coffee farmers as a whole in each country. The sample size was defined using the formula for finite populations [20] and then individual farmers were selected randomly, stratified according to exposure level and country by 2050 (Table 1).
Sampling sizes reported?	Yes	From a population of 7,000 farmer members from 15 organizations across the four countries, 558 farmers were interviewed.
Data analysis methods reported?	Yes	A cluster analysis was carried out for each indicator of sensitivity and adaptive capacity based on the score of each family using the Ward method with Euclidean distance. Then an Analysis of Variance (ANOVA) was applied using the LSD-Fisher test to compare the averages for each indicator by cluster. The indicators in each cluster that obtained significantly different sample averages were classified in three levels on a scale of 0 to 1 (0–0.33=low, 0.34–0.66=medium, 0.67–1=high). Clusters with the greatest number of indicators with high, medium or low averages were classified as having high, medium or low sensitivity and adaptive capacity [21]. Each factor (exposure, sensitivity and adaptive capacity), as previously explained, and was classified into three levels (high, medium, low). To calculate the vulnerability equation we assigned each level a quantitative value: low=1, medium=2, high=3. With three factors and three levels per factor, we obtained 27 possible combinations. After applying the equation we obtained 7 values (–1,0,1,2,3,4,5), which we used to define low (–1,0), medium (1,2,3,) and high (4,5) levels of vulnerability (Figure 1). A Principal Components Analysis (PCA) was carried out to identify the indicators that most contribute to the sensitivity or adaptive capacity of families in different municipalities.
Conclusion		
Transparency Conclusion:	Inconclusive operationalization – info to be requested from authors.	

**Structured summary of operationalization – transparency assessment**		
**Construct:** Adaptive Capacity		
**Article:** CARE (2009)		
Criterion	Assessment	Quoted text or Rationale for negative assessment
Construct defined?	Yes	The ability of a system to adjust to climate change (including climate variability and extremes) to moderate potential damages, to take advantage of opportunities, or to cope with the consequences.6
Data collection methods reported?	Yes	Secondary Research An understanding of the livelihoods strategies, socio-economic situation, power dynamics and local governance in the target communities is critical to ensuring that facilitators are effective during the field work, and to identifying focus groups within the community.

		Secondary sources for community-level information would include:<br>• Assessment reports from NGOs or UN organizations<br>• Evaluations of past disaster response operations<br>Vulnerability monitoring programs (e.g. Famine Early Warning System (FEWS-Net)<br>Environmental screening reports for the target area<br>• Government documents including poverty reduction strategies, development plans, official statistics, etc.<br>Consultation with agencies (governmental and non-governmental) working in the target area<br>Seasonal forecasts<br>[...]<br>Policy Analysis<br>Depending on the degree of decentralization of decision-making in a particular country, local-level plans or policies may be important in shaping adaptive capacity of vulnerable households and individuals. Regional or district plans and/or sector strategies can give helpful information on priorities of local governments. Further, the process for developing these policies and strategies can provide insights into the level of participation of vulnerable people in establishing these priorities. The status of implementation can yield useful information on resource and capacity constraints faced by local actors.<br>[...]<br>Institutional Mapping<br>Institutions play a critical role in supporting or constraining people's capacity to adapt to climate change. In order to better understand which institutions are most important to people in the target communities, an institutional mapping exercise is useful. Institutional mapping involves examination of the following questions:<br>• Which organizations (governmental, non-governmental and • community-based) are involved in addressing key issues and problems related to climate change? What do they do?<br>• Where do they work?<br>• How do they interact with the target population? • Where are the overlaps with other organizations? • Where are the gaps in capacity?<br>• How might some organizations impede the work of others? • What are their longer term plans for working in the area? • What are the strengths and weaknesses of the institutions?13<br>• What is the institution's level of influence over planning and implementation of adaptation?<br>The mapping exercise assists in identifying the institutions that should be engaged in the CVCA process, as well as potential allies and opponents in addressing vulnerability at the community level.<br>Key Informant Interviews<br>Key informants can provide useful insights into local governance structures and status of implementation of local policies and programs. Power issues within and between communities and other stakeholders can also be surfaced through interviews with key actors. Again preserving their anonymity may allow them to speak more freely.<br>Key informants at the local government/community level would include: Local leaders (chiefs, mayors, elected representatives, etc.)<br>• •<br>Representatives of community-based organizations (CBOs) such as farmer's groups, water and sanitation committees, savings and credit groups, etc.

		Representatives of women's groups or other rights-based groups<br>Representatives of NGOs working on programs or advocacy in the target area •<br>Academic/research institutions engaged in the target area<br>[...]<br>Secondary Research In order to effectively plan the field work and to ensure that communities are not over-burdened with research and assessment teams, it is important to review existing information.<br>Sources of information on livelihoods would include: Assessment reports from NGOs or UN organizations Project/program baseline studies and/or evaluation reports<br>• • •<br>Vulnerability monitoring programs (e.g. Famine Early Warning System (FEWS-Net)) • Post-disaster assessments<br>• Consultation with agencies (governmental and non-governmental) working in the target area • Maps showing topography, agro-ecological regions, infrastructure, etc.<br>In some cases, it will be possible to answer many guiding questions using secondary sources, however this information must be verified by local stakeholders. Having more background information can allow the field work to focus specifically on climate change issues. In many cases, very little information may exist at the household/individual level, and so a deeper participatory analysis will be required to understand the dynamics of vulnerability.<br>Participatory Tools<br>Secondary research is complemented by collaborative learning employing typical participatory tools and discussions in focus groups (FGs).<br>FGs usually involve 5 – 12 people selected to be representative of different livelihood systems and/or vulnerable groups in the community. A single FG can include people selected by age and gender (e.g. teenage girls, or elderly women, or young married men), or by some other common characteristic (e.g. people with chronic illnesses, or members of farmer associations). At a minimum, it is suggested to conduct discussions with groups of men and women separately so that participants feel free to talk openly.<br>Participatory tools are designed to draw out issues which can then be examined further through semi-structured discussion. This is meant only as a guide; the field work must be tailored to the particular context and the objectives of the analysis. As well, the range of tools used will depend on the time and resources available for the field work. Fostering participatory processes, and balancing learning with information-gathering, relies on strong, thoughtful facilitation. The Field Guides at the end of this Handbook provide facilitation tips as well as detailed guidance on using participatory tools and facilitating discussions with focus groups.
Reporting of indicators/questions used to operationalise construct?	Yes	Guiding Questions Local Government/Community Level<br>Capacity Development<br>- What institutions (governmental and non-governmental) are involved in research, planning<br>and implementation of adaptation? What are the most important institutions in facilitating or constraining adaptation? - Do local institutions (governmental and non-governmental) have capacity to monitor and<br>- -<br>analyze information on current and future climate risks? Are mechanisms in place to disseminate this information?<br>- Do local institutions have capacity to plan and implement adaptation activities?<br>- Are resources allocated for implementation of adaptation-related policies?

		What is the budget?<br>Where are the resources coming from? What are the existing capacity and resource needs and/or gaps for climate change adaptation? - What new capacities may be needed to address changing circumstances due to climate change?<br><br>Addressing<br>Underlying Causes of Vulnerability<br>- What social groups within the community are most vulnerable to climate change? - Are local planning processes participatory?<br>- Do women and other marginalized groups have a voice in local planning processes? - Do local policies provide access to and control over critical livelihoods resources for all? - What are the other factors constraining adaptive capacity of the most vulnerable groups? Do<br>vulnerable communities and groups have any influence over these factors?<br>[...]<br>Guiding Questions Household/Individual Level<br>Capacity Development<br>Are social and economic safety nets available to households?<br>- Are financial services available to households?<br>- Do people have knowledge and skills to employ adaptation strategies? - Do people have access to seasonal forecasts and other climate information?<br>Addressing<br>Underlying Causes of Vulnerability<br>- Are men and women working together to address challenges? - Do households have control over critical livelihoods resources?<br>- Do women and other marginalized groups have equal access to information, skills and services? - Do women and other marginalized groups have equal rights and access to resources? - Are there other social, political or economic factors which make particular people within the<br>-<br>community more vulnerable than others? Do these vulnerable groups have any influence over these factors?
Sampling strategies reported?	(proposed methodology)	
Sampling sizes reported?	(proposed methodology)	
Data analysis methods reported?	Yes	Compiling and Analyzing the Data<br>After completing the field work, the teams should review the information gathered to identify any gaps in the information collected. Follow-up interviews or further research may be required to fill gaps.<br>Field teams from the same community should then sit together to analyze the information gained. Comparing the results for different groups within the community is an important part of the process, as this yields insights on differential vulnerability. The analysis may expose inequalities within the community which may not have been previously recognized. Follow-up discussions or interviews with particularly vulnerable groups may be needed to fully understand community or household dynamics.<br>Once information for specific communities has been analyzed, it can be helpful

		for teams who worked in different communities to come together to identify trends, common issues, differences, and to evaluate the process.<br>The community information should then be combined with the information gained using other tools in order to answer the guiding questions.<br>Validating the Analysis<br>After preliminary analysis of the data has been completed, a presentation of the findings should be made to community representatives to confirm the validity of the conclusions. A two-step approach is suggested for the validation process. The first step would be to present the analysis to the community focus groups themselves to ensure that the conclusions drawn are correct. Next, it is recommended that the results are presented to a wider community group and local organizations to facilitate dialogue on issues that have been raised by particular groups which may have implications for other groups. In particular, this provides an opportunity to make other groups in the community aware of the views of particularly vulnerable groups. Note that there may be sensitivities around some of the issues raised by different groups, and facilitators must be prepared to resolve conflicts that may arise. It must also be ensured that the sharing of views does not yield negative consequences for any members of the community. Local actions can provide guidance on this.<br>Feedback from stakeholders should be incorporated into the final analysis.
Conclusion		
Transparency Conclusion:	Partial	

**Structured summary of operationalization – transparency assessment**		
**Construct:** Hazard		
**Article:** CARE (2009)		
Criterion	Assessment	Quoted text or Rationale for negative assessment
Construct defined?	Yes	A dangerous phenomenon, substance, human activity or condition that may cause loss of life, injury or other health impacts, property damage, loss of livelihoods and services, social and economic disruption, or environmental damage.9
Data collection methods reported?	Yes	Secondary Research An understanding of the livelihoods strategies, socio-economic situation, power dynamics and local governance in the target communities is critical to ensuring that facilitators are effective during the field work, and to identifying focus groups within the community.<br>Secondary sources for community-level information would include:<br>• Assessment reports from NGOs or UN organizations<br>• Evaluations of past disaster response operations<br>Vulnerability monitoring programs (e.g. Famine Early Warning<br>System (FEWS-Net)<br>Environmental screening reports for the target area<br>• Government documents including poverty reduction strategies,<br>development plans, official statistics, etc.<br>Consultation with agencies (governmental and non-governmental)<br>working in the target area<br>Seasonal forecasts<br>[...]<br>Policy Analysis<br>Depending on the degree of decentralization of decision-making in a particular country, local-level plans or policies may be important in shaping adaptive capacity of vulnerable households and individuals. Regional or district plans and/or sector

		strategies can give helpful information on priorities of local governments. Further, the process for developing these policies and strategies can provide insights into the level of participation of vulnerable people in establishing these priorities. The status of implementation can yield useful information on resource and capacity constraints faced by local actors.<br>[...]<br>Institutional Mapping<br>Institutions play a critical role in supporting or constraining people's capacity to adapt to climate change. In order to better understand which institutions are most important to people in the target communities, an institutional mapping exercise is useful. Institutional mapping involves examination of the following questions:<br>• Which organizations (governmental, non-governmental and •<br>community-based) are involved in addressing key issues and problems related to climate change? What do they do?<br>• Where do they work?<br>• How do they interact with the target population? • Where are the overlaps with other organizations? • Where are the gaps in capacity?<br>• How might some organizations impede the work of others? • What are their longer term plans for working in the area? • What are the strengths and weaknesses of the institutions?13<br>• What is the institution's level of influence over planning and implementation of adaptation?<br>The mapping exercise assists in identifying the institutions that should be engaged in the CVCA process, as well as potential allies and opponents in addressing vulnerability at the community level.<br>Key Informant Interviews<br>Key informants can provide useful insights into local governance structures and status of implementation of local policies and programs. Power issues within and between communities and other stakeholders can also be surfaced through interviews with key actors. Again preserving their anonymity may allow them to speak more freely.<br>Key informants at the local government/community level would include: Local leaders (chiefs, mayors, elected representatives, etc.)<br>• •<br>Representatives of community-based organizations (CBOs) such as farmer's groups, water and sanitation committees, savings and credit groups, etc.<br>Representatives of women's groups or other rights-based groups<br>Representatives of NGOs working on programs or advocacy in the target area •<br>Academic/research institutions engaged in the target area<br>[...]<br>Secondary Research In order to effectively plan the field work and to ensure that communities are not over-burdened with research and assessment teams, it is important to review existing information.<br>Sources of information on livelihoods would include: Assessment reports from NGOs or UN organizations Project/program baseline studies and/or evaluation reports<br>• • •<br>Vulnerability monitoring programs (e.g. Famine Early Warning System (FEWS-Net))<br>• Post-disaster assessments<br>• Consultation with agencies (governmental and non-governmental) working in the target area • Maps showing topography, agro-ecological regions, infrastructure, etc.<br>In some cases, it will be possible to answer many guiding questions using

		secondary sources, however this information must be verified by local stakeholders. Having more background information can allow the field work to focus specifically on climate change issues. In many cases, very little information may exist at the household/individual level, and so a deeper participatory analysis will be required to understand the dynamics of vulnerability.<br>Participatory Tools<br>Secondary research is complemented by collaborative learning employing typical participatory tools and discussions in focus groups (FGs).<br>FGs usually involve 5 – 12 people selected to be representative of different livelihood systems and/or vulnerable groups in the community. A single FG can include people selected by age and gender (e.g. teenage girls, or elderly women, or young married men), or by some other common characteristic (e.g. people with chronic illnesses, or members of farmer associations). At a minimum, it is suggested to conduct discussions with groups of men and women separately so that participants feel free to talk openly.<br>Participatory tools are designed to draw out issues which can then be examined further through semi-structured discussion. This is meant only as a guide; the field work must be tailored to the particular context and the objectives of the analysis. As well, the range of tools used will depend on the time and resources available for the field work. Fostering participatory processes, and balancing learning with information-gathering, relies on strong, thoughtful facilitation. The Field Guides at the end of this Handbook provide facilitation tips as well as detailed guidance on using participatory tools and facilitating discussions with focus groups.
Reporting of indicators/questions used to operationalise construct?	Yes	Guiding Questions Local Government/Community Level<br>Disaster Risk Reduction<br>- What are the most important climate-related hazards the region and/or ecological zone faces?<br>Non-climate related? How are hazards likely to change over time as a result of climate change? - What groups within the community are most vulnerable to disasters? - Do local institutions have access to disaster risk information? - Are local disaster risk management plans being implemented? - Are functional early warning systems in place at the local level? - Does the local government have the capacity to respond to disasters? - Which other institutions are engaged disaster risk management at local level?<br><br>Guiding Questions Household/Individual Level<br>Disaster Risk Reduction<br>- What are the biggest climate-related hazards faced? Non-climate related hazards? - How are hazards likely to change over time as a result of climate change? - Do households have protected reserves of food and agricultural inputs? - Do households have secure shelter? - Are key assets protected from hazards?<br>- Do people have access to early warnings for climate hazards?<br>Do people have mobility to escape danger in the event of climate hazards?
Sampling strategies reported?	(proposed methodology)	
Sampling sizes reported?	(proposed methodology)	
Data analysis methods reported?	Yes	Compiling and Analyzing the Data<br>After completing the field work, the teams should review the information gathered

		to identify any gaps in the information collected. Follow-up interviews or further research may be required to fill gaps.<br>Field teams from the same community should then sit together to analyze the information gained. Comparing the results for different groups within the community is an important part of the process, as this yields insights on differential vulnerability. The analysis may expose inequalities within the community which may not have been previously recognized. Follow-up discussions or interviews with particularly vulnerable groups may be needed to fully understand community or household dynamics.<br>Once information for specific communities has been analyzed, it can be helpful for teams who worked in different communities to come together to identify trends, common issues, differences, and to evaluate the process.<br>The community information should then be combined with the information gained using other tools in order to answer the guiding questions.<br>Validating the Analysis<br>After preliminary analysis of the data has been completed, a presentation of the findings should be made to community representatives to confirm the validity of the conclusions. A two-step approach is suggested for the validation process. The first step would be to present the analysis to the community focus groups themselves to ensure that the conclusions drawn are correct. Next, it is recommended that the results are presented to a wider community group and local organizations to facilitate dialogue on issues that have been raised by particular groups which may have implications for other groups. In particular, this provides an opportunity to make other groups in the community aware of the views of particularly vulnerable groups. Note that there may be sensitivities around some of the issues raised by different groups, and facilitators must be prepared to resolve conflicts that may arise. It must also be ensured that the sharing of views does not yield negative consequences for any members of the community. Local actions can provide guidance on this.<br>Feedback from stakeholders should be incorporated into the final analysis.
<u>Conclusion</u>		
Transparency Conclusion:	Partial	

**Structured summary of operationalization – transparency assessment**		
**Construct:** Resilience		
**Article:** CARE (2009)		
<u>Criterion</u>	<u>Assessment</u>	<u>Quoted text or Rationale for negative assessment</u>
Construct defined?	Yes	The ability of a community to resist, absorb, and recover from the effects of hazards in a timely and efficient manner, preserving or restoring its essential basic structures, functions and identity.8
Data collection methods reported?	Yes	Secondary Research An understanding of the livelihoods strategies, socio-economic situation, power dynamics and local governance in the target communities is critical to ensuring that facilitators are effective during the field work, and to identifying focus groups within the community.<br>Secondary sources for community-level information would include:<br>• Assessment reports from NGOs or UN organizations<br>• Evaluations of past disaster response operations<br>Vulnerability monitoring programs (e.g. Famine Early Warning System (FEWS-Net)<br>Environmental screening reports for the target area

		• Government documents including poverty reduction strategies, development plans, official statistics, etc.<br>Consultation with agencies (governmental and non-governmental) working in the target area<br>Seasonal forecasts<br>[...]<br>Policy Analysis<br>Depending on the degree of decentralization of decision-making in a particular country, local-level plans or policies may be important in shaping adaptive capacity of vulnerable households and individuals. Regional or district plans and/or sector strategies can give helpful information on priorities of local governments. Further, the process for developing these policies and strategies can provide insights into the level of participation of vulnerable people in establishing these priorities. The status of implementation can yield useful information on resource and capacity constraints faced by local actors.<br>[...]<br>Institutional Mapping<br>Institutions play a critical role in supporting or constraining people's capacity to adapt to climate change. In order to better understand which institutions are most important to people in the target communities, an institutional mapping exercise is useful. Institutional mapping involves examination of the following questions:<br>• Which organizations (governmental, non-governmental and • community-based) are involved in addressing key issues and problems related to climate change? What do they do?<br>• Where do they work?<br>• How do they interact with the target population? • Where are the overlaps with other organizations? • Where are the gaps in capacity?<br>• How might some organizations impede the work of others? • What are their longer term plans for working in the area? • What are the strengths and weaknesses of the institutions?13<br>• What is the institution's level of influence over planning and implementation of adaptation?<br>The mapping exercise assists in identifying the institutions that should be engaged in the CVCA process, as well as potential allies and opponents in addressing vulnerability at the community level.<br>Key Informant Interviews<br>Key informants can provide useful insights into local governance structures and status of implementation of local policies and programs. Power issues within and between communities and other stakeholders can also be surfaced through interviews with key actors. Again preserving their anonymity may allow them to speak more freely.<br>Key informants at the local government/community level would include: Local leaders (chiefs, mayors, elected representatives, etc.)<br>• •<br>Representatives of community-based organizations (CBOs) such as farmer's groups, water and sanitation committees, savings and credit groups, etc.<br>Representatives of women's groups or other rights-based groups<br>Representatives of NGOs working on programs or advocacy in the target area •<br>Academic/research institutions engaged in the target area<br>[...]<br>Secondary Research In order to effectively plan the field work and to ensure that communities are not over-burdened with research and assessment teams, it is important to review existing information.

		Sources of information on livelihoods would include: Assessment reports from NGOs or UN organizations Project/program baseline studies and/or evaluation reports<br>• • •<br>Vulnerability monitoring programs (e.g. Famine Early Warning System (FEWS-Net))<br>• Post-disaster assessments<br>• Consultation with agencies (governmental and non-governmental) working in the target area • Maps showing topography, agro-ecological regions, infrastructure, etc.<br>In some cases, it will be possible to answer many guiding questions using secondary sources, however this information must be verified by local stakeholders. Having more background information can allow the field work to focus specifically on climate change issues. In many cases, very little information may exist at the household/individual level, and so a deeper participatory analysis will be required to understand the dynamics of vulnerability.<br>Participatory Tools<br>Secondary research is complemented by collaborative learning employing typical participatory tools and discussions in focus groups (FGs).<br>FGs usually involve 5 – 12 people selected to be representative of different livelihood systems and/or vulnerable groups in the community. A single FG can include people selected by age and gender (e.g. teenage girls, or elderly women, or young married men), or by some other common characteristic (e.g. people with chronic illnesses, or members of farmer associations). At a minimum, it is suggested to conduct discussions with groups of men and women separately so that participants feel free to talk openly.<br>Participatory tools are designed to draw out issues which can then be examined further through semi-structured discussion. This is meant only as a guide; the field work must be tailored to the particular context and the objectives of the analysis. As well, the range of tools used will depend on the time and resources available for the field work. Fostering participatory processes, and balancing learning with information-gathering, relies on strong, thoughtful facilitation. The Field Guides at the end of this Handbook provide facilitation tips as well as detailed guidance on using participatory tools and facilitating discussions with focus groups.
Reporting of indicators/questions used to operationalise construct?	Yes/no	
Sampling strategies reported?	(proposed methodology)	
Sampling sizes reported?	(proposed methodology)	
Data analysis methods reported?	Yes	Compiling and Analyzing the Data<br>After completing the field work, the teams should review the information gathered to identify any gaps in the information collected. Follow-up interviews or further research may be required to fill gaps.<br>Field teams from the same community should then sit together to analyze the information gained. Comparing the results for different groups within the community is an important part of the process, as this yields insights on

		differential vulnerability. The analysis may expose inequalities within the community which may not have been previously recognized. Follow-up discussions or interviews with particularly vulnerable groups may be needed to fully understand community or household dynamics.<br>Once information for specific communities has been analyzed, it can be helpful for teams who worked in different communities to come together to identify trends, common issues, differences, and to evaluate the process.<br>The community information should then be combined with the information gained using other tools in order to answer the guiding questions.<br>Validating the Analysis<br>After preliminary analysis of the data has been completed, a presentation of the findings should be made to community representatives to confirm the validity of the conclusions. A two-step approach is suggested for the validation process. The first step would be to present the analysis to the community focus groups themselves to ensure that the conclusions drawn are correct. Next, it is recommended that the results are presented to a wider community group and local organizations to facilitate dialogue on issues that have been raised by particular groups which may have implications for other groups. In particular, this provides an opportunity to make other groups in the community aware of the views of particularly vulnerable groups. Note that there may be sensitivities around some of the issues raised by different groups, and facilitators must be prepared to resolve conflicts that may arise. It must also be ensured that the sharing of views does not yield negative consequences for any members of the community. Local actions can provide guidance on this.<br>Feedback from stakeholders should be incorporated into the final analysis.
Conclusion		
Transparency Conclusion:	Partial	

**Structured summary of operationalization – transparency assessment**		
**Construct:** current vulnerability		
**Article:** Ford & Smit (2004)		
Criterion	Assess ment	Quoted text or Rationale for negative assessment
Construct defined?	Yes	The assessment of current vulnerability requires analyzing and documenting communities' experiences with climatic risks (current exposure) and the adaptive options and resource management strategies employed to address these risks (current adaptive capacity).
Data collection methods reported?	Yes	Such knowledge can be gained through several estab- lished ethnographic techniques, including focus groups, interviews, and participant observation<br>[...]<br>Information on risks and adaptation strategies can also be derived from content analysis of government reports, newspaper articles, Hudson Bay Company postal records, Distant Early Warning Site reports, and the insights of experienced land and resource use managers (Duerden, 2001). Solomon and Hart (1999) used Hudson Bay Com- pany postal records and ships' logbooks to examine storm frequency and severity in the Beaufort Sea. Fienup-Riordan (1999) used Catholic mission records and letters between government officials to assess the nature and impacts of a storm surge in 1931 in southwestern Alaska.
Reporting of	Yes/no	Indigenous populations possess detailed knowledge of their environ- ment built up

indicators/questions used to operationalise construct?		through personal observation and experience and from shared experience of members of the community (Duerden and Kuhn, 1998; Huntington, 1998; Usher, 2000). Knowledge about the environment and its use can be employed to identify and reconstruct events and condi- tions that represent climatic risks to the community and to provide insights into the resource-use options and risk-management strategies employed to prepare for, avoid or moderate, and recover from the effects of exposure
Sampling strategies reported?	No	
Sampling sizes reported?	No	
Data analysis methods reported?	Yes	The analysis of current vulnerability requires a timeframe to establish how far back in time the study should go when analyzing risks and community response. The timeframe depends in part on the extent to which past conditions that determined adaptability are relevant today, as well as on the availability of information. In setting the timeline, one must weigh the value of analyzing how previous genera-tions coped with hazards against the recent social, eco- nomic, political, and technological changes, which also determine adaptive capacity. Lim et al. (in press) suggest limiting historical analysis to one or two decades, although many of the traditional coping mechanisms, such as flex- ibility, detailed local knowledge, social networks, and intercommunity trade, have a much longer history and remain strong among Arctic communities (Berkes and Jolly, 2001).
Conclusion		
Transparency Conclusion:	Partial	

**Structured summary of operationalization – transparency assessment**		
**Construct:** future adaptive capcity		
**Article:** Ford & Smit (2004)		
Criterion	Assessment	Quoted text or Rationale for negative assessment
Construct defined?	Yes	Future adaptive capacity concerns the degree to which the community can deal with the estimated future exposures
Data collection methods reported?	Yes	Such knowledge can be gained through several estab- lished ethnographic techniques, including focus groups, interviews, and participant observation<br>[...]<br>Information on risks and adaptation strategies can also be derived from content analysis of government reports, newspaper articles, Hudson Bay Company postal records, Distant Early Warning Site reports, and the insights of experienced land and resource use managers (Duerden, 2001). Solomon and Hart (1999) used Hudson Bay Com- pany postal records and ships' logbooks to examine storm frequency and severity in the Beaufort Sea. Fienup-Riordan (1999) used Catholic mission records and letters between government officials to assess the nature and impacts of a storm surge in 1931 in southwestern Alaska.
Reporting of indicators/questions used to operationalise construct?	Yes	Indigenous populations possess detailed knowledge of their environ- ment built up through personal observation and experience and from shared experience of members of the community (Duerden and Kuhn, 1998; Huntington, 1998; Usher, 2000). Knowledge about the environment and its use can be employed to identify and reconstruct events and condi- tions that represent climatic risks to the community and to provide insights into the resource-use options and risk-management strategies employed to prepare for, avoid or moderate, and recover from the effects of exposure

		[...]<br>By examining past responses to climate variability and extremes and having the commu- nity identify its future adaptation options and constraints,
Sampling strategies reported?	No	
Sampling sizes reported?	No	
Data analysis methods reported?	Yes	Future adaptive capacity concerns the degree to which the community can deal with the estimated future exposures. By examining past responses to climate variability and extremes and having the commu- nity identify its future adaptation options and constraints, researchers can characterize a community's ability to cope with future changes and collaborate to identify adaptive strategies that will reduce risk.
Conclusion		
Transparency Conclusion:		Partial

**Structured summary of operationalization – transparency assessment**		
**Construct:** Livelihood strategies		
**Article:** Notenbaert et al (2013)		
Criterion	Assess ment	Quoted text or Rationale for negative assessment
Construct defined?	Yes	(Turner et al. 2003).
Data collection methods reported?	Yes	A detailed household survey was used to elicit household responses (n = 184) about their available resources, live- lihood sources and coping strategies to climate variability.
Reporting of indicators/questions used to operationalise construct?	Yes	The questionnaire was divided into the following five sections: (1) household composition, livelihood strategies and livestock assets; (2) household livestock ownership, herd dynamics and species; (3) livestock feeding tech- niques, management, products and markets, (4) welfare outcomes (income, food consumption and health) and (5) focused on the main concerns/challenges facing households and their coping strategies. In this last section, households were asked to list and rank their concerns and describe the coping strategies employed to counter these concerns. Furthermore, the households were asked to compare with other households (in the same village) the extent to which they have been coping. For each of the concerns they were facing, they were asked whether they had been coping either better than, worse than or similar to other households in their village.
Sampling strategies reported?	No	
Sampling sizes reported?	Yes	household survey was used to elicit household responses (n = 184)
Data analysis methods reported?	Yes/n o	These pieces of information taken all together, allowed us to come up with a household-level vulnerability index, assessing the degree of a household's vulnerability to climate change and variability in relation to other households in the same village. The index is not based on thresholds nor does it represent an absolute value. It is a relative measure, representing the households' own perception of how they have been coping in the past as compared to other households. For each of the concerns a household listed, an impact<br>factor (Ii) was established. This impact factor takes the value of ?1 if the household considered itself coping less well than the other households, -1 if it was doing better and 0 if they assessed themselves similar to the other households in the

		village. The rationale being that house- holds that are coping less than others, are more vulnerable, while the ones that are doing better than other households have a lower vulnerability. The concerns listed are not all of equal importance. To correct for this, we established a weight for each of the concerns based on the rank they were assigned across all the sampled households. If a household reported n concerns, the vulnerability of a household was then calculated following formula below. Formula 1:<br>v ¼X n<br>i¼1<br>n = number of concerns, wi = weight of concerns, Ii = impact (?1: worse than/0: same/- 1:better).<br>[...]<br>Analyzing determinants of coping strategies<br>In an attempt to understand the underlying mechanisms and processes through which these factors influence households' coping capacity, we also analyzed how they influence the choice of coping strategies. To this end, we applied a binary logit regression between the same geographic, demographic and household characteristics and each of the coping strategies. To assess the factors influencing a specific coping strategy, the respondents that utilized this coping strategy were given the value of 1 and 0 otherwise. As such we created dependent vari- ables for each of the coping strategies which were then each regressed against the independent variables from Table 1.

<u>Conclusion</u>	
Transparency Conclusion:	Partial

Appendix M: Validity Assessments

Research Questions

1. Is this operationalization valid?
2. Is this operationalization feasible?
3. (Which operationalizations are most useful?)

To operationalise the first question, we use two sub-questions:

1.1 Are the data collection methods used able to generate the **kind** of data required by the construct? Where 'kind' refers, non-exhaustively, to natural, social, critical realist, or interpretivist data etc.

1.2 If you put the data gathered through the operational questions all together, do you get a complete and valid understanding of the phenomena that is conceptually defined?

Answer question 1.1 on the basis of the construct definition and the data collection methods cells.

Answer 1.2 on the basis of the construct definition and the 'indicators/questions used to operationalise' cells (and if it is helpful, data analysis methods).

For a given operationalization, if the answer to 1.1 or 1.2 is no, then the answer for Question 1 is also 'no'

The second question is operationalized as follows:

2.1 Is this procedure feasible within CCAFS programs?

Question 2 is to be answered on the basis of all available information.

The questions are to be answered based on the information provided in the structured summaries below. If information in the relevant cells is understandable but not sufficient to answer the questions, answer 'can't tell'. In some cases, construct definitions contain references to previous work. If this work is familiar to you, that is, if you know how a construct is defined in the cited work, then use this knowledge; if the work is not familiar then the appropriate answer is 'can't tell'.

Equally, where a description is not understood, due for example to unclear writing, or to the use of dense terminology from an unfamiliar field, then a reliable answer cannot be given so please fill in 'unclear'.

Question 3 will get dealt with later. Once we have the low-level constructs that work identified, we will put them together. Question 3 will be used on those higher-order operationalizations.. Question 3 will ask you, after validity and feasibility assessments have been made, to select preferred candidates.

**Transparency Assessment Article summary**	
**Article**	Antwi-Agyei et al (2013)
**Transparent operationalizations**	Community; diversified livelihood activities; exposure; financial capital; human capital; natural capital; physical capital; resilience and vulnerable communities; social captial
**Partially transparent**	
**Not transparent**	

**Structured summary of operationalization – validity assessment**			**1.1 DCM Appropriate**	**1.2 valid empirical rep?**	**1. conclusion - Valid?**	**2. Feasible?**
**Construct:** Community						
**Article:** Antwi-Agyei et al (2013)						
Criterion	Assessment	Quoted text or Rationale for negative assessment				
Construct defined?	Yes	Nevertheless, households are connected to the wider community, which can greatly influence the decision-making process in relation to the use of pro- ductive resources of a particular household; hence, the need to explore vulnerability and adaptation strategies at the household level in relation to the wider socioeconomic and cultural processes occurring at the community level (Thomas et al. 2007).	Yes/ no/ can't tell	Yes/ no/ can't tell	Yes/ no/ can't tell<br><br>YES	Yes/ no/ can't tell<br><br>YES
Data collection methods reported?	Yes	Within one resilient and one vulnerable district, 6 specific resilient and vulnerable<br>farming communities (3 in each case) were selected for further research, based on infor- mation gained through interviews with experts and stakeholders (Antwi-Agyei et al. 2012). Three communities were selected from each district to allow comparisons to be made among communities within the same district without sacrificing	Yes			

		the opportunity for in- depth qualitative analysis; hence, three were deemed a suitable sample size. The resilient communities were Aframso, Babaso and Nyamebekyere located in the Ejura Sekyere- dumasi district of Ashanti region, while vulnerable communities were Adaboya, Ayelbia and Vea located in the Bongo district in the Upper East region (Fig. 1; Antwi-Agyei et al. 2012).				
Reporting of indicators/questions used to operationalise construct?	Yes	communities were Aframso, Babaso and Nyamebekyere located in the Ejura Sekyere- dumasi district of Ashanti region, while vulnerable communities were Adaboya, Ayelbia and Vea located in the Bongo district in the Upper East region (Fig. 1; Antwi-Agyei et al. 2012).		YES		
Sampling strategies reported?	Yes	Within one resilient and one vulnerable district, 6 specific resilient and vulnerable farming communities (3 in each case) were selected for further research, based on infor- mation gained through interviews with experts and stakeholders (Antwi-Agyei et al. 2012). Three communities were selected from each district to allow comparisons to be made among communities within the same district without sacrificing the opportunity for in- depth qualitative analysis; hence, three were deemed a suitable sample size.				
Sampling sizes reported?	Yes	6 specific resilient and vulnerable farming communities (3 in each case) were selected				
Data analysis methods reported?	Yes	Using Minitab, a one-way ANOVA was computed to compare the relative vulnerability among the various households and communities, and all differences resulting in p\0.05 were considered statistically significant. K-means cluster analysis using STATISTICA software was undertaken to group the households according to their vulnerability.				

**Structured summary of operationalization – validity assessment**	**1.1 DCM**	**1.2 valid**	**1.**	**2.**

**Construct:** Exposure			**Appropriate**	**empirical rep?**	**conclusion - Valid?**	**Feasible?**
**Article:** Antwi-Agyei et al (2013)						
Criterion	Assessment	Quoted text or Rationale for negative assessment				
Construct defined?	Yes	Exposure relates to the extent to which a particular system may be exposed to climatic stresses or variations (IPCC 2007).	Yes/ no/ can't tell	Yes/ no/ can't tell	Yes/ no/ can't tell	Yes/ no/ can't tell
Data collection methods reported?	Yes (2ndary data)	These two districts (and 6 communities) represent a range of different agroeco- logical and socioeconomic characteristics in Ghana. The Ejura Sekyeredumasi district (the resilient district) lies within the transitional agroecological zone and experiences bi-modal rainfall patterns with the major rainfall season from April to July and the minor rainfall season from September to October (EPA 2003). Average annual rainfall ranges from 1,200 to 1,500 mm with minimum and maximum temperatures of 20 and 32 C respectively (EPA 2003). Bongo district (the vulnerable district) lies within the Sudan savannah ag- roecological zone. The Bongo district experiences uni-modal rainfall from May/June - September/October, which constitutes the main farming season (EPA 2003). Average annual rainfall ranges from 800 to 1,000 mm with maximum temperatures of 35 C (EPA 2003).	NO			
Reporting of indicators/questions used to operationalise construct?	Yes (2ndary data)	These two districts (and 6 communities) represent a range of different agroeco- logical and socioeconomic characteristics in Ghana. The Ejura Sekyeredumasi district (the resilient district) lies within the transitional agroecological zone and experiences bi-modal rainfall patterns with the major rainfall season from April to July and the minor rainfall season from September to October (EPA 2003). Average annual rainfall ranges from 1,200 to 1,500 mm with minimum and maximum temperatures of 20 and 32 C respectively (EPA 2003). Bongo district (the vulnerable district) lies within the Sudan savannah ag- roecological zone. The Bongo district experiences uni-modal rainfall from May/June - September/October, which constitutes the main farming season (EPA 2003). Average annual rainfall ranges from 800 to 1,000 mm with maximum temperatures of 35 C (EPA 2003).		NO		

Sampling strategies reported?	Yes (2ndary data)	These two districts (and 6 communities) represent a range of different agroeco- logical and socioeconomic characteristics in Ghana. The Ejura Sekyeredumasi district (the resilient district) lies within the transitional agroecological zone and experiences bi-modal rainfall patterns with the major rainfall season from April to July and the minor rainfall season from September to October (EPA 2003). Average annual rainfall ranges from 1,200 to 1,500 mm with minimum and maximum temperatures of 20 and 32 C respectively (EPA 2003). Bongo district (the vulnerable district) lies within the Sudan savannah ag- roecological zone. The Bongo district experiences uni-modal rainfall from May/June - September/October, which constitutes the main farming season (EPA 2003). Average annual rainfall ranges from 800 to 1,000 mm with maximum temperatures of 35 C (EPA 2003).				
Sampling sizes reported?	Yes (2ndary data)	These two districts (and 6 communities) represent a range of different agroeco- logical and socioeconomic characteristics in Ghana. The Ejura Sekyeredumasi district (the resilient district) lies within the transitional agroecological zone and experiences bi-modal rainfall patterns with the major rainfall season from April to July and the minor rainfall season from September to October (EPA 2003). Average annual rainfall ranges from 1,200 to 1,500 mm with minimum and maximum temperatures of 20 and 32 C respectively (EPA 2003). Bongo district (the vulnerable district) lies within the Sudan savannah ag- roecological zone. The Bongo district experiences uni-modal rainfall from May/June - September/October, which constitutes the main farming season (EPA 2003). Average annual rainfall ranges from 800 to 1,000 mm with maximum temperatures of 35 C (EPA 2003).				
Data analysis methods reported?	Yes	In this regard, it is assumed that households within the same agroecological zone may be exposed to the same level of climate anomaly (drought in this case) (Eakin and Bojorquez-Tapia 2008). This paper focuses on drought because it is the major threat to African farming systems (UNDP 2007), with some studies predicting increased incidences of drought in				

		the future across sub-Saharan Africa (Boko et al. 2007).				

**Structured summary of operationalization – validity assessment**			**1.1 DCM Appropriate**	**1.2 valid empirical rep?**	**1. conclusion - Valid?**	**2. Feasible?**
**Construct:** Financial capital						
**Article:** Antwi-Agyei et al (2013)						
Criterion	Assessment	Quoted text or Rationale for negative assessment				
Construct defined?	Yes	Financial capital assets such as savings and remittances play a crucial role in cushioning households against drought-related food shortages. Eliciting information on financial assets was very problematic because of a lack of records on sales and memory lapses. Livestock were considered to offer readily available cash in times of crop failure due to erratic rainfall patterns in the study communities.	Yes/ no/ can't tell	Yes/ no/ can't tell	Yes/ no/ can't tell<br><br>YES	Yes/ no/ can't tell<br><br>YES
Data collection methods reported?	Yes	Data presented in this paper were collected using a mixture of participatory methods such as focus group discussions, household questionnaire surveys and key informant interviews. Data collection started with a rapid rural appraisal (Chambers 1994) during which com- munity gatherings and transect walks were conducted with community members including opinion leaders at each of the 6 villages. This provided an overview of the significant social and physical features of the selected communities that influenced their livelihood activities (Sallu et al. 2009). A household questionnaire survey was used to collect both qualitative and quantitative data. The questionnaire survey assessed households' capital assets (financial, human, natural, physical, and social). This information was used to develop a household livelihood vulnerability index (see Sect. 2.3). A total of 270 household ques- tionnaire surveys were conducted in the 6 farming communities (45 Questionnaires in each).	YES			
Reporting of indicators/questions used to	Yes	Eliciting information on financial assets was very problematic because of a lack of records on sales and memory lapses. Livestock were considered to offer readily available cash in		yes		

operationalise construct?		times of crop failure due to erratic rainfall patterns in the study communities.<br>[...]<br>Households without poultry or livestock scored 1 whilst those with livestock scored 2. In addition, financial assets were assessed by examining the remittances received by the household from family members or friends over the past 12 months.<br>[...]<br>Households that received remittances in the last 12 months scored 2 and those that did not receive any remittances scored 1. Access to credit may also influence adaptation to climate change including access to inputs such as improved cultivars of crops (e.g. Butt et al. 2006; Fosu-Mensah et al. 2012). Hence, it is assumed that households that have no access to credit will be more vulnerable and scored 1 whilst those with access to credit were given a score of 2.<br>[...]<br>Table 1 Indicators of household livelihood vulnerability index collected through a household survey across six communities in Ghana<br>[...]<br>Access to credit<br>Do you have access to credit for your agricultural activities?<br>Ownership of livestock<br>Do you have livestock or poultry? List the types and numbers of livestock.<br>Remittances received<br>Have you received remittances from family or friends in the last 12 months?				
Sampling strategies reported?	Yes	A random sampling approach was used for the selection of communities that partici-<br>pated in the study. Within communities, households were stratified into different wealth groups. A random sample of households was then surveyed. The criterion for wealth ranking was developed based on the perception of wealth and poverty by the communities' opinion leaders and individual households evaluated at the time of the survey.				

		Where there was an under representation of any wealth group, key informants were used to identify appropriate households to supplement the sample. At least one focus group discussion was conducted at each village with between 5 and 10 farmers of different socio-cultural backgrounds to further explore the main themes that emerged in the questionnaire surveys.				
Sampling sizes reported?	Yes	A total of 270 household ques- tionnaire surveys were conducted in the 6 farming communities (45 Questionnaires in each).<br>[...]<br>At least one focus group discussion was conducted at each village with between 5 and 10 farmers of different socio-cultural backgrounds to further explore the main themes that emerged in the questionnaire surveys.				
Data analysis methods reported?	Yes	To ensure the comparability of indicators that were used in the construction of the household livelihood vulnerability index, all indicators were standardised following the UNDP (2007) procedure of standardising indicators for life expectancy index (Eq. 1).<br>[...]<br>Having standardised the indicators, it was then necessary to elicit appropriate weights to them. An unequal weighting system, based on relative importance attached to each indi- cator by local households, extension officers, key informants and experts was used because it was deemed necessary to include the views of both local households and experts in the assessment. Hence, a five-point Likert scale was used where farmers, extension officers, key informants, and experts were asked to rank the five most important indicators that they considered to influence vulnerability at the household level (Table 2). The number of times a particular indicator was cited was used to generate the weighting system (Table 2). The following weights were assigned: 14 % to social capital, 11 % to human capital, 9 % to natural capital, 27 % to financial capital, 10 % to physical capital and 29 % to livelihood diversification (Table 2). The household livelihood vulnerability index for a household was then calculated using				

		the following model (Eq. 2) (Vincent 2004).<br>HLVI ¼ Ssvi□Wi<br>ðÞþðHsvi□WiiÞþ ðNsvi□WiiiÞþðFsvi□WivÞþðPsvi□WvÞ<br>þðLsvi□WviÞ<br>[...]<br>Quanti- tative data were transcribed and analysed using SPSS and Minitab (Edition 15). Using Minitab, a one-way ANOVA was computed to compare the relative vulnerability among the various households and communities, and all differences resulting in p\0.05 were considered statistically significant. K-means cluster analysis using STATISTICA software was undertaken to group the households according to their vulnerability. K-means cluster analysis, which seeks to group cases into distinct clusters by seeking groups that minimise variability within clusters and maximise variability between clusters (Levia and Page 2000), has been applied to spatial vulnerability assessment in dynamic systems (see Antwi-Agyei et al. 2012).				

**Structured summary of operationalization – validity assessment**			**1.1 DCM Appropriate**	**1.2 valid empirical rep?**	**1. conclusion - Valid?**	**2. Feasible?**
**Construct:** human capital						
**Article:** Antwi-Agyei et al (2013)						
Criterion	Assessment	Quoted text or Rationale for negative assessment				
Construct defined?	Yes	Human capital assets were represented by two indicators: the educational level of the head of the household (or the most educated person in the household) and the health status of the household (Table 1).			YES	YES
Data collection methods reported?	Yes	Data presented in this paper were collected using a mixture of participatory methods such as focus group discussions, household questionnaire surveys and key informant interviews. Data collection started with a rapid rural appraisal (Chambers 1994) during which com- munity gatherings and transect walks were conducted with community members including opinion leaders at each of the 6 villages. This provided an overview of the significant social	YES			

		and physical features of the selected communities that influenced their livelihood activities (Sallu et al. 2009). A household questionnaire survey was used to collect both qualitative and quantitative data. The questionnaire survey assessed households' capital assets (financial, human, natural, physical, and social). This information was used to develop a household livelihood vulnerability index (see Sect. 2.3). A total of 270 household ques- tionnaire surveys were conducted in the 6 farming communities (45 Questionnaires in each).				
Reporting of indicators/questions used to operationalise construct?	Yes	Human capital assets were represented by two indicators: the educational level of the head of the household (or the most educated person in the household) and the health status of the household (Table 1). No formal education was afforded a value of 1; 2 in the case of only primary education; 3 in the case of secondary education; and 4 for households that had tertiary education.<br>[...]<br>To assess health status, households were asked about the number of times they have been to the hospital (or hospitalised) within the last 12 months. House- holds with members that had been to the hospital were scored 1 whilst those with members that had not been to hospital as out patients (and those not needing any medical attention) within this period were scored 2. Also, situations where members of a household required hospital treatment but could not arrange transport and other resources needed were taken into consideration when scoring such a household.<br>[...]<br>Table 1 Indicators of household livelihood vulnerability index collected through a household survey across six communities in Ghana<br>[...]<br>Educational level<br>Could you please state the highest education attained?<br>Health status<br>Have any member of this household been ill in the last 12		YES		

		months?				
Sampling strategies reported?	Yes	A random sampling approach was used for the selection of communities that partici-<br>pated in the study. Within communities, households were stratified into different wealth groups. A random sample of households was then surveyed. The criterion for wealth ranking was developed based on the perception of wealth and poverty by the communities' opinion leaders and individual households evaluated at the time of the survey. Where there was an under representation of any wealth group, key informants were used to identify appropriate households to supplement the sample. At least one focus group discussion was conducted at each village with between 5 and 10 farmers of different socio-cultural backgrounds to further explore the main themes that emerged in the questionnaire surveys.				
Sampling sizes reported?	Yes	A total of 270 household ques- tionnaire surveys were conducted in the 6 farming communities (45 Questionnaires in each).<br>[...]<br>At least one focus group discussion was conducted at each village with between 5 and 10 farmers of different socio-cultural backgrounds to further explore the main themes that emerged in the questionnaire surveys.				
Data analysis methods reported?	Yes	To ensure the comparability of indicators that were used in the construction of the household livelihood vulnerability index, all indicators were standardised following the UNDP (2007) procedure of standardising indicators for life expectancy index (Eq. 1).<br>[...]<br>Having standardised the indicators, it was then necessary to elicit appropriate weights to them. An unequal weighting system, based on relative importance attached to each indi-cator by local households, extension officers, key informants and experts was used because it was deemed necessary to include the views of both local households and experts in the assessment. Hence, a five-point Likert scale was used where farmers, extension officers, key informants, and experts were				

		asked to rank the five most important indicators that they considered to influence vulnerability at the household level (Table 2). The number of times a particular indicator was cited was used to generate the weighting system (Table 2). The following weights were assigned: 14 % to social capital, 11 % to human capital, 9 % to natural capital, 27 % to financial capital, 10 % to physical capital and 29 % to livelihood diversification (Table 2). The household livelihood vulnerability index for a household was then calculated using the following model (Eq. 2) (Vincent 2004).<br>HLVI ¼ Ssvi□Wi<br>ðÞþðHsvi□WiiÞþ ðNsvi□WiiiÞþðFsvi□WivÞþðPsvi□WvÞ<br>þðLsvi□WviÞ<br>[...]<br>Quanti- tative data were transcribed and analysed using SPSS and Minitab (Edition 15). Using Minitab, a one-way ANOVA was computed to compare the relative vulnerability among the various households and communities, and all differences resulting in p\0.05 were considered statistically significant. K-means cluster analysis using STATISTICA software was undertaken to group the households according to their vulnerability. K-means cluster analysis, which seeks to group cases into distinct clusters by seeking groups that minimise variability within clusters and maximise variability between clusters (Levia and Page 2000), has been applied to spatial vulnerability assessment in dynamic systems (see Antwi-Agyei et al. 2012).				

**Structured summary of operationalization – validity assessment**			**1.1 DCM Appropriate**	**1.2 valid empirical rep?**	**1. conclusion - Valid?**	**2. Feasible?**
**Construct:** Natural Capital						
**Article:** Antwi-Agyei et al (2013)						
Criterion	Assessment	Quoted text or Rationale for negative assessment				
Construct defined?	Yes	Natural capital assets were assessed by two indicators. The first was the size of the farm holding under cultivation scored 3; those cultivating 16-20 acres scored 4, and			YES-ISH	YES

		households cultivating [20 acres scored 5. T				
Data collection methods reported?	Yes	Data presented in this paper were collected using a mixture of participatory methods such as focus group discussions, household questionnaire surveys and key informant interviews. Data collection started with a rapid rural appraisal (Chambers 1994) during which com- munity gatherings and transect walks were conducted with community members including opinion leaders at each of the 6 villages. This provided an overview of the significant social and physical features of the selected communities that influenced their livelihood activities (Sallu et al. 2009). A household questionnaire survey was used to collect both qualitative and quantitative data. The questionnaire survey assessed households' capital assets (financial, human, natural, physical, and social). This information was used to develop a household livelihood vulnerability index (see Sect. 2.3). A total of 270 household ques- tionnaire surveys were conducted in the 6 farming communities (45 Questionnaires in each).	YES			
Reporting of indicators/questions used to operationalise construct?	Yes	Natural capital assets were assessed by two indicators. The first was the size of the farm holding under cultivation (this was estimated as the average area of cultivated land over the past 5 years) (Table 1). It is assumed that the larger the farm holding, the greater the opportunity for the household to have more crops and yield, and hence the lower the vulnerability to climate change, though it is noted that labour availability and financial capital both affect the reality of how much land can be cultivated. Households which cultivated less than 5 acres scored 1; those cultivating between 5 and 10 acres scored 2; those cultivating between 11 and 15 acres scored 3; those cultivating 16-20 acres scored 4, and households cultivating [20 acres scored 5. The type of land tenure and level of security it provides may have serious implications for the management of agricultural soils and could indirectly affect crop productivity and environmental sustainability, conse- quently influencing household vulnerability (Butt et al. 2006). Three different tenure arrangements were identified in the study communities.		CAN'T TELL		

		These were ‘‘land inherited’’, ‘‘land purchased’’ and ‘‘land rented’’ by the household. A score of 1 was given to households who rented their farmlands; 2 for households who purchased their farmlands; and 3 for those who inherited their farmlands. Households that inherited their farm lands were given the highest score because it is assumed that they will have the most secure land tenure.<br>[...]<br>Table 1 Indicators of household livelihood vulnerability index collected through a household survey across six communities in Ghana<br>[...]<br>Farm holding size<br>Could you please state the size of farm holding in acres?<br>Tenure system<br>By what arrangements do you have access to your farm land for farming activities?				
Sampling strategies reported?	Yes	A random sampling approach was used for the selection of communities that partici-<br>pated in the study. Within communities, households were stratified into different wealth groups. A random sample of households was then surveyed. The criterion for wealth ranking was developed based on the perception of wealth and poverty by the communities’ opinion leaders and individual households evaluated at the time of the survey. Where there was an under representation of any wealth group, key informants were used to identify appropriate households to supplement the sample. At least one focus group discussion was conducted at each village with between 5 and 10 farmers of different socio-cultural backgrounds to further explore the main themes that emerged in the questionnaire surveys.				
Sampling sizes reported?	Yes	A total of 270 household ques- tionnaire surveys were conducted in the 6 farming communities (45 Questionnaires in each).<br>[...]<br>At least one focus group discussion was conducted at each village with between 5 and 10 farmers of different socio-				

		cultural backgrounds to further explore the main themes that emerged in the questionnaire surveys.				
Data analysis methods reported?	Yes	To ensure the comparability of indicators that were used in the construction of the household livelihood vulnerability index, all indicators were standardised following the UNDP (2007) procedure of standardising indicators for life expectancy index (Eq. 1).<br>[...]<br>Having standardised the indicators, it was then necessary to elicit appropriate weights to them. An unequal weighting system, based on relative importance attached to each indi- cator by local households, extension officers, key informants and experts was used because it was deemed necessary to include the views of both local households and experts in the assessment. Hence, a five-point Likert scale was used where farmers, extension officers, key informants, and experts were asked to rank the five most important indicators that they considered to influence vulnerability at the household level (Table 2). The number of times a particular indicator was cited was used to generate the weighting system (Table 2). The following weights were assigned: 14 % to social capital, 11 % to human capital, 9 % to natural capital, 27 % to financial capital, 10 % to physical capital and 29 % to livelihood diversification (Table 2). The household livelihood vulnerability index for a household was then calculated using the following model (Eq. 2) (Vincent 2004).<br>HLVI ¼ SsviWi<br>ðÞþðHsviWiiÞþ ðNsviWiiiÞþðFsviWivÞþðPsviWvÞ<br>þðLsviWviÞ<br>[...]<br>Quanti- tative data were transcribed and analysed using SPSS and Minitab (Edition 15). Using Minitab, a one-way ANOVA was computed to compare the relative vulnerability among the various households and communities, and all differences resulting in p\0.05 were considered statistically significant. K-means cluster analysis using STATISTICA software was undertaken to group the households according to their vulnerability. K-means cluster analysis, which seeks to group				

		cases into distinct clusters by seeking groups that minimise variability within clusters and maximise variability between clusters (Levia and Page 2000), has been applied to spatial vulnerability assessment in dynamic systems (see Antwi-Agyei et al. 2012).				

**Structured summary of operationalization – validity assessment**			**1.1 DCM Appropriate**	**1.2 valid empirical rep?**	**1. conclusion - Valid?**	**2. Feasible?**
**Construct:** Physical capital						
**Article:** Antwi-Agyei et al (2013)						
<u>Criterion</u>	<u>Assessment</u>	<u>Quoted text or Rationale for negative assessment</u>				
Construct defined?	Yes	Physical assets that were assessed included the presence of irrigation facilities and own- ership of radios, television or mobile phones by a household (Table 1).			YES	YES
Data collection methods reported?	Yes	Data presented in this paper were collected using a mixture of participatory methods such as focus group discussions, household questionnaire surveys and key informant interviews. Data collection started with a rapid rural appraisal (Chambers 1994) during which com- munity gatherings and transect walks were conducted with community members including opinion leaders at each of the 6 villages. This provided an overview of the significant social and physical features of the selected communities that influenced their livelihood activities (Sallu et al. 2009). A household questionnaire survey was used to collect both qualitative and quantitative data. The questionnaire survey assessed households' capital assets (financial, human, natural, physical, and social). This information was used to develop a household livelihood vulnerability index (see Sect. 2.3). A total of 270 household ques- tionnaire surveys were conducted in the 6 farming communities (45 Questionnaires in each).	YES			
Reporting of indicators/questions used to operationalise	Yes	Physical assets that were assessed included the presence of irrigation facilities and own- ership of radios, television or mobile phones by a household (Table 1). Irrigation facilities are crucial for rain-fed agriculture-dependent households, as		YES-ISH		

construct?		these facilities help farmers to practise dry season farming. It is assumed that households with irrigation facilities will be less vulnerable to changing rainfall patterns. Hence, households without irrigation facilities scored 1, whilst those with these facilities scored 2. The presence of radios, television or mobile phone in a rural household can be an effective tool for communication and accessing information on changing weather patterns (see Naab and Koranteng 2012). Here, households with any of these three assets scored 2, and those without any scored 1. Physical assets such as road networks and the availability of markets and health facilities may enhance the adaptive capacity of a household (see Zhang et al. 2007). These assets were not included in the vulnerability computation because field observations suggested that these physical assets did not significantly differ amongst either the resilient or vul- nerable communities.<br>[...]<br>Irrigation facilities<br>Do you have access to irrigation facilities for dry season farming?<br>Ownership of radio, television or mobile phone<br>Could you please list all communication gadgets that you have? These include TV, mobile phone or radios etc.				
Sampling strategies reported?	Yes	A random sampling approach was used for the selection of communities that partici-<br>pated in the study. Within communities, households were stratified into different wealth groups. A random sample of households was then surveyed. The criterion for wealth ranking was developed based on the perception of wealth and poverty by the communities' opinion leaders and individual households evaluated at the time of the survey. Where there was an under representation of any wealth group, key informants were used to identify appropriate households to supplement the sample. At least one focus group discussion was conducted at each village with between 5 and 10 farmers of different socio-cultural backgrounds to				

		further explore the main themes that emerged in the questionnaire surveys.				
Sampling sizes reported?	Yes	A total of 270 household ques- tionnaire surveys were conducted in the 6 farming communities (45 Questionnaires in each).<br>[...]<br>At least one focus group discussion was conducted at each village with between 5 and 10 farmers of different socio-cultural backgrounds to further explore the main themes that emerged in the questionnaire surveys.				
Data analysis methods reported?	Yes	To ensure the comparability of indicators that were used in the construction of the household livelihood vulnerability index, all indicators were standardised following the UNDP (2007) procedure of standardising indicators for life expectancy index (Eq. 1).<br>[...]<br>Having standardised the indicators, it was then necessary to elicit appropriate weights to them. An unequal weighting system, based on relative importance attached to each indi- cator by local households, extension officers, key informants and experts was used because it was deemed necessary to include the views of both local households and experts in the assessment. Hence, a five-point Likert scale was used where farmers, extension officers, key informants, and experts were asked to rank the five most important indicators that they considered to influence vulnerability at the household level (Table 2). The number of times a particular indicator was cited was used to generate the weighting system (Table 2). The following weights were assigned: 14 % to social capital, 11 % to human capital, 9 % to natural capital, 27 % to financial capital, 10 % to physical capital and 29 % to livelihood diversification (Table 2). The household livelihood vulnerability index for a household was then calculated using the following model (Eq. 2) (Vincent 2004).<br>HLVI ¼ Ssvi□Wi<br>ðÞþðHsvi□WiiÞþ ðNsvi□WiiiÞþðFsvi□WivÞþðPsvi□WvÞ<br>þðLsvi□WviÞ<br>[...]				

		Quanti- tative data were transcribed and analysed using SPSS and Minitab (Edition 15). Using Minitab, a one-way ANOVA was computed to compare the relative vulnerability among the various households and communities, and all differences resulting in p\0.05 were considered statistically significant. K-means cluster analysis using STATISTICA software was undertaken to group the households according to their vulnerability. K-means cluster analysis, which seeks to group cases into distinct clusters by seeking groups that minimise variability within clusters and maximise variability between clusters (Levia and Page 2000), has been applied to spatial vulnerability assessment in dynamic systems (see Antwi-Agyei et al. 2012).				

**Structured summary of operationalization – validity assessment**			**1.1 DCM Appropriate**	**1.2 valid empirical rep?**	**1. conclusion - Valid?**	**2. Feasible?**
**Construct:** Resilient and vulnerable communities						
**Article:** Antwi-Agyei et al (2013)						
Criterion	Assessment	Quoted text or Rationale for negative assessment				
Construct defined?	Yes	was based on a definition of ‘‘vulnerable’’ regions and districts as those where relatively minor perturbations in rainfall over the past 40 years had significant impacts on crop yields (Antwi-Agyei et al. 2012). Conversely, ‘‘resilient’’ regions and districts were defined as those where even large droughts were observed to have had only minor impacts on crop yields (Simelton e			CAN'T TELL	CAN'T TELL
Data collection methods reported?	Yes (2ndary data)	The Ejura Sekyeredumasi district of Ashanti region and Bongo district of the Upper East region of Ghana were selected for this study having been previously identified as the most resilient and vulnerable regions and districts respectively in Ghana (Antwi-Agyei et al. 2012). This was based on a definition of ‘‘vulnerable’’ regions and districts as those where relatively minor perturbations in rainfall over the past 40 years had significant impacts on crop yields (Antwi-Agyei et al. 2012). Conversely, ‘‘resilient’’ regions and districts were defined as those where even large droughts were observed	CAN'T TELL			

		to have had only minor impacts on crop yields (Simelton et al. 2009).				
Reporting of indicators/questions used to operationalise construct?	Yes (2ndary data)	The Ejura Sekyeredumasi district of Ashanti region and Bongo district of the Upper East region of Ghana were selected for this study having been previously identified as the most resilient and vulnerable regions and districts respectively in Ghana (Antwi-Agyei et al. 2012). This was based on a definition of ‘‘vulnerable’’ regions and districts as those where relatively minor perturbations in rainfall over the past 40 years had significant impacts on crop yields (Antwi-Agyei et al. 2012). Conversely, ‘‘resilient’’ regions and districts were defined as those where even large droughts were observed to have had only minor impacts on crop yields (Simelton et al. 2009).		CAN’T TELL		
Sampling strategies reported?	Yes	Advancing this work further, an assessment of livelihoods offers the opportunity to highlight the various adaptations that might be available to determine how rural communities can cope with declining crop yields due to drought, and also how such declining yields can affect livelihoods (see Ziervogel and Calder 2003). Within one resilient and one vulnerable district, 6 specific resilient and vulnerable farming communities (3 in each case) were selected for further research, based on infor- mation gained through interviews with experts and stakeholders (Antwi-Agyei et al. 2012). Three communities were selected from each district to allow comparisons to be made among communities within the same district without sacrificing the opportunity for in-depth qualitative analysis; hence, three were deemed a suitable sample size.<br>[...]<br>The resilient communities were Aframso, Babaso and Nyamebekyere located in the Ejura Sekyere- dumasi district of Ashanti region, while vulnerable communities were Adaboya, Ayelbia and Vea located in the Bongo district in the Upper East region (Fig. 1; Antwi-Agyei et al. 2012).				
Sampling sizes reported?	Yes	The Ejura Sekyeredumasi district of Ashanti region and Bongo district of the Upper East region of Ghana were selected for this study having been previously identified as the most				

		resilient and vulnerable regions and districts respectively in Ghana<br>[...]<br>Within one resilient and one vulnerable district, 6 specific resilient and vulnerable<br>farming communities (3 in each case) were selected for further research,				
Data analysis methods reported?	Yes	Using Minitab, a one-way ANOVA was computed to compare the relative vulnerability among the various households and communities, and all differences resulting in p\0.05 were considered statistically significant. K-means cluster analysis using STATISTICA software was undertaken to group the households according to their vulnerability.				

**Structured summary of operationalization – validity assessment**			**1.1 DCM Appropriate**	**1.2 valid empirical rep?**	**1. conclusion - Valid?**	**2. Feasible?**
**Construct:** Social Captial						
**Article:** Antwi-Agyei et al (2013)						
Criterion	Assessment	Quoted text or Rationale for negative assessment				
Construct defined?	Yes	Social capital—including connections to technical support and social resources such as networks, associations and affiliations—was assessed by counting the number of associations or groups to which the members of the household belong			NO	YES
Data collection methods reported?	Yes	Data presented in this paper were collected using a mixture of participatory methods such as focus group discussions, household questionnaire surveys and key informant interviews. Data collection started with a rapid rural appraisal (Chambers 1994) during which com- munity gatherings and transect walks were conducted with community members including opinion leaders at each of the 6 villages. This provided an overview of the significant social and physical features of the selected communities that influenced their livelihood activities (Sallu et al. 2009). A household questionnaire survey was used to collect both qualitative and quantitative data. The questionnaire survey	NO			

		assessed households' capital assets (financial, human, natural, physical, and social). This information was used to develop a household livelihood vulnerability index (see Sect. 2.3). A total of 270 household ques- tionnaire surveys were conducted in the 6 farming communities (45 Questionnaires in each).				
Reporting of indicators/questions used to operationalise construct?	Yes	Social capital—including connections to technical support and social resources such as networks, associations and affiliations—was assessed by counting the number of associations or groups to which the members of the household belong (Pretty and Ward 2001; Vincent 2007). It was assumed that households belonging to a high number of social groups and associations are better networked to cope with the impacts of climate change on their livelihoods activities (Adger 2003; Pretty 2003), as these represent the number of social safety nets and a form of informal grassroots insurance available to the household during climate-related crisis (e.g. Fraser 2007; Vincent 2007). Both bonding and bridging social capital were assessed. Bonding social capital is based on characteristics such as family kinship, ethnicity or nationality (Woolcock 2001). Bridging capital refers to ties to external groups and usually transcends different socioeconomic statuses, nationalities, religions, and ethnicities (Woolcock 2001). A scoring procedure for social capital followed the methods of Vincent (2007). A score of 1 was given to households that belonged to no identifiable group, 2 for those who were members of one group, 3 for membership of two groups and 4 for membership of more than three groups. While the level of interaction among the group members and the strength of the ties within such social groups could affect their usefulness, interaction and ties were beyond the scope of the assessment and were not considered.<br>[...]<br>Table 1 Indicators of household livelihood vulnerability index collected through a household survey across six communities in Ghana<br>Number of groups or associations households belong to		NO		

		Do you belong to any social groups? Could you please list them?				
Sampling strategies reported?	Yes	A random sampling approach was used for the selection of communities that partici-<br>pated in the study. Within communities, households were stratified into different wealth groups. A random sample of households was then surveyed. The criterion for wealth ranking was developed based on the perception of wealth and poverty by the communities' opinion leaders and individual households evaluated at the time of the survey. Where there was an under representation of any wealth group, key informants were used to identify appropriate households to supplement the sample. At least one focus group discussion was conducted at each village with between 5 and 10 farmers of different socio-cultural backgrounds to further explore the main themes that emerged in the questionnaire surveys.				
Sampling sizes reported?	Yes	A total of 270 household ques- tionnaire surveys were conducted in the 6 farming communities (45 Questionnaires in each).<br>[...]<br>At least one focus group discussion was conducted at each village with between 5 and 10 farmers of different socio-cultural backgrounds to further explore the main themes that emerged in the questionnaire surveys.				
Data analysis methods reported?	Yes	To ensure the comparability of indicators that were used in the construction of the household livelihood vulnerability index, all indicators were standardised following the UNDP (2007) procedure of standardising indicators for life expectancy index (Eq. 1).<br>[...]<br>Having standardised the indicators, it was then necessary to elicit appropriate weights to them. An unequal weighting system, based on relative importance attached to each indi-cator by local households, extension officers, key informants and experts was used because it was deemed necessary to include the views of both local households and experts in the assessment. Hence, a five-point Likert scale was used where				

		farmers, extension officers, key informants, and experts were asked to rank the five most important indicators that they considered to influence vulnerability at the household level (Table 2). The number of times a particular indicator was cited was used to generate the weighting system (Table 2). The following weights were assigned: 14 % to social capital, 11 % to human capital, 9 % to natural capital, 27 % to financial capital, 10 % to physical capital and 29 % to livelihood diversification (Table 2). The household livelihood vulnerability index for a household was then calculated using the following model (Eq. 2) (Vincent 2004).<br>HLVI ¼ Ssvi⁇Wi<br>ðÞþðHsvi⁇WiiÞþ ðNsvi⁇WiiiÞþðFsvi⁇WivÞþðPsvi⁇WvÞ<br>þðLsvi⁇WviÞ<br>[...]<br>Quanti- tative data were transcribed and analysed using SPSS and Minitab (Edition 15). Using Minitab, a one-way ANOVA was computed to compare the relative vulnerability among the various households and communities, and all differences resulting in p\0.05 were considered statistically significant. K-means cluster analysis using STATISTICA software was undertaken to group the households according to their vulnerability. K-means cluster analysis, which seeks to group cases into distinct clusters by seeking groups that minimise variability within clusters and maximise variability between clusters (Levia and Page 2000), has been applied to spatial vulnerability assessment in dynamic systems (see Antwi-Agyei et al. 2012).				

**Transparency Assessment Article summary**	
**Article**	Baca et al (2004)
**Transparent operationalizations**	Adaptive capacity; Exposure
**Partially transparent**	Inconclusive: Sensitivity
**Not transparent**	

**Structured summary of operationalization – validity assessment**			**1.1 DCM Appropriate**	**1.2 valid empirical rep?**	**1. conclusion - Valid?**	**2. Feasible?**
**Construct:** Adaptive Capacity						
**Article:** Baca et al (2004)						
Criterion	Assessment	Quoted text or Rationale for negative assessment				
Construct defined?	Yes	In contrast, adaptive capacity is defined as a system's ability to adjust to climate change in order to reduce or mitigate possible damage [3]. Adaptive capacity is dynamic, and depends partly on the society productive base, such as: natural and artificial assets, social benefits and networks, human capital and institutions, governance, national income, health and technology [2], and how much capability a society has to adapt to the changes so as to maintain, minimize loss of, or maximize gain in welfare.			NO	NO
Data collection methods reported?	Yes	The indicators were used to assess the vulnerability of coffee farms in each country. From a population of 7,000 farmer members from 15 organizations across the four countries, 558 farmers were interviewed.	CAN'T TELL			
Reporting of indicators/questions used to operationalise construct?	Yes	Parameters were then constructed to evaluate each indicator as shown in Table S1 in File S1. To quantify the parameters scales from 1 to 5 were applied or a binary scale of 0 and 1, depending on the nature of the parameter. The final values for each indicator were calculated by averaging all the parameters and then transformed to a 0-1 continuous variable scale, with 0 being low and 1 being high sensitivity and adaptive capacity.<br>[...]		NO		
		**Indicator** / **Parameter**				
		Management of shade trees and reforestation / Number of trees cut; Number of trees planted				
		Pollution / Waste management				

			Release of fermentation residues into water<br>Management of agrochemical containers<br>Coffee waste management<br>Area burning annually				
		Viability of post-harvest infra.	Types or forms to dry coffee				
		Access to credits	Term of credit<br>Interest rate of credit<br>Opportunity of credits				
		Income diversification	Number of sources of income				
		Access to specialty markets	Destined for sale<br>Special market access				
		Access to alternative technologies	Varieties<br>Drip irrigation<br>Water harvesting				
		Organization	Participation<br>Time<br>Benefits				
		Knowledge level of policies related to the coffee sector, environmental laws and other	Policies about coffee sector<br>Environmental laws<br>Land polices				
		Access to formal and informal education	Level of education<br>Quality of technical assistance<br>Crops for which receive technical assistance<br>Types of media accessed				
		Knowledge level of agro ecological system	Registration practices and activities				

		Coffee intercropping<br>Pests and diseases				
Sampling strategies reported?	Yes	From a population of 7,000 farmer members from 15 organizations across the four countries, 558 farmers were interviewed. The farmers may be considered representative of small-scale organized farmers, but should not be considered representative of the coffee farmers as a whole in each country. The sample size was defined using the formula for finite populations [20] and then individual farmers were selected randomly, stratified according to exposure level and country by 2050 (Table 1).				
Sampling sizes reported?	Yes	From a population of 7,000 farmer members from 15 organizations across the four countries, 558 farmers were interviewed.				
Data analysis methods reported?	Yes	A cluster analysis was carried out for each indicator of sensitivity and adaptive capacity based on the score of each family using the Ward method with Euclidean distance. Then an Analysis of Variance (ANOVA) was applied using the LSD-Fisher test to compare the averages for each indicator by cluster. The indicators in each cluster that obtained significantly different sample averages were classified in three levels on a scale of 0 to 1 (0–0.33=low, 0.34–0.66=medium, 0.67–1=high). Clusters with the greatest number of indicators with high, medium or low averages were classified as having high, medium or low sensitivity and adaptive capacity [21]. Each factor (exposure, sensitivity and adaptive capacity), as previously explained, and was classified into three levels (high, medium, low). To calculate the vulnerability equation we assigned each level a quantitative value: low=1, medium=2, high=3. With three factors and three levels per factor, we obtained 27 possible combinations. After applying the equation we obtained 7 values (–1,0,1,2,3,4,5), which we used to define low (–1,0), medium (1,2,3,) and high (4,5) levels of vulnerability (Figure 1). A Principal Components Analysis (PCA) was carried out to identify the indicators that most contribute to the sensitivity or adaptive capacity of families in different municipalities.				

**Structured summary of operationalization – validity assessment**			**1.1 DCM Appropriate**	**1.2 valid empirical rep?**	**1. conclusion - Valid?**	**2. Feasible?**
**Construct:** Exposure						
**Article:** Baca et al (2004)						
Criterion	Assessment	Quoted text or Rationale for negative assessment				
Construct defined?	Yes	Exposure is the nature and extent of changes that a place's climate is subjected to with regard to variables such as temperature, precipitation, and extreme weather events.			CAN'T TELL	

Data collection methods reported?	Yes (2ndary data)	The methodology combined current climate data with future climate change predictions. To map current climatic suitability, the historical climate database WorldClim (www.worldclim.org) was used<br>[...]<br>To predict future climate, the SRES-A2a scenario 19 IPCC Global Circulation Models were used. The Delta method was used to down-scale the climate change data, based on the sum of the anomalies interpolated with the WorldClim monthly high- resolution surfaces [15].	CAN'T TELL			
Reporting of indicators/questions used to operationalise construct?	Yes (2ndary data)	To map current climatic suitability, the historical climate database WorldClim (www.worldclim.org) was used. The variables included a total of 19 bioclimatic variables derived from monthly precipitation, monthly median temperature, minimum and maximum temper-ature [15]. Bioclimatic variables represent annual trends, season-ality, and extreme conditions.		CAN'T TELL		
Sampling strategies reported?	Yes (2ndary data)	The methodology combined current climate data with future climate change predictions. To map current climatic suitability, the historical climate database WorldClim (www.worldclim.org) was used<br>[...]<br>To predict future climate, the SRES-A2a scenario 19 IPCC Global Circulation Models were used. The Delta method was used to down-scale the climate change data, based on the sum of the anomalies interpolated with the WorldClim monthly high- resolution surfaces [15].				
Sampling sizes reported?	Yes (2ndary data)	The methodology combined current climate data with future climate change predictions. To map current climatic suitability, the historical climate database WorldClim (www.worldclim.org) was used<br>[...]<br>To predict future climate, the SRES-A2a scenario 19 IPCC Global Circulation Models were used. The Delta method was used to down-scale the climate change data, based on the sum of the anomalies interpolated with the WorldClim monthly high- resolution surfaces [15].				
Data analysis methods reported?	Yes	The Maximum entropy (MAXENT) method, a general-purpose method for making predictions or inferences based on incomplete information [17], was used to predict the future climatic suitability for coffee. The model requires calibration with climate data for current coffee production areas, which is provided by GPS				

		coordinates. The model assumes that a certain future climate at a given site is as suitable or unsuitable for the crop as is the same climate at another site in the present. This assumption is reasonable as long as crop genetics and cropping systems do not significantly change. It thus predicts what will happen in terms of relative climatic suitability for a crop if these factors do not change and helps identify those sites where adaptations in crops and cropping systems are necessary in order to avoid the consequences of a predicted decline in climatic suitability. This approach has previously been used for coffee [6], [18]. Two measures of uncertainty were calculated: (1) the agreement<br>of calculated models as a percentage of models that predict changes in the same direction and (2) the coefficient of variation (CV) among models.<br>[...]<br>For exposure, the relative decreases in climatic suitability according to the MAXENT model were divided into three classes of suitability loss (low, medium, high). For sensitivity and adaptive capacity, indicators were identified and quantified through interviews with the farming families.<br>[...]<br>Each factor (exposure, sensitivity and adaptive capacity), as previously explained, and was classified into three levels (high, medium, low). To calculate the vulnerability equation we assigned each level a quantitative value: low=1, medium=2, high=3. With three factors and three levels per factor, we obtained 27 possible combinations. After applying the equation we obtained 7 values (–1,0,1,2,3,4,5), which we used to define low (–1,0), medium (1,2,3,) and high (4,5) levels of vulnerability (Figure 1). A Principal Components Analysis (PCA) was carried out to identify the indicators that most contribute to the sensitivity or adaptive capacity of families in different municipalities.				

Transparency Assessment Article summary	
**Article**	Capaldo et al (2010)
**Transparent operationalizations**	Current exposure to risks; current socio-economic status
**Partially transparent**	
**Not transparent**	Risk management; risks

**Structured summary of operationalization – validity assessment**			**1.1 DCM Appropriate**	**1.2 valid empirical rep?**	**1. conclusion - Valid?**	**2. Feasible?**
**Construct:** Current exposure to risk						
**Article:** Capaldo et al (2010)						
Criterion	Assessment	Quoted text or Rationale for negative assessment				
Construct defined?	YES	conceptual framework drawn from it by Løvendal and Knowles (2005).			CAN'T TELL	
Data collection methods reported?	2ndary data	We analyze a sample of 1831 rural households from Nicaragua, surveyed in the 2001 Encuesta Nacional de Hogares Sobre Medición de Nivel de Vida, by the Instituto Nacional de Estadísticas y Censos INEC de Nicaragua. Constructed variables used in the analysis were prepared by the Rural Income Generating Activities (RIGA) project team at FAO.	CAN'T TELL			
Reporting of indicators/questions used to operationalise construct?	Yes/no	We use ex-post data on shocks and risk management strategies. These include information on the incidence of a covariate shock (such as drought) and an idiosyncratic shock (illness)<br>[...]<br>Table 1: Summary of variables<br>Drought shock<br>Illness shock		YES		
Sampling strategies reported?	2ndary data	We analyze a sample of 1831 rural households from Nicaragua, surveyed in the 2001 Encuesta Nacional de Hogares Sobre Medición de Nivel de Vida, by the Instituto Nacional de Estadísticas y Censos INEC de Nicaragua. Constructed variables used in the analysis were prepared by the Rural Income Generating Activities (RIGA) project team at FAO.				

Sampling sizes reported?	Yes	sample of 1831 rural households				
Data analysis methods reported?	Yes	After accounting for heteroskedasticity through the use of generalized least squares, we estimate vulnerability to food insecurity as the normal probability that the "individual minimum dietary energy requirement under light physical activity" exceeds the expected individual dietary energy consumption (measured in kilocalories). Since the main purpose of this paper is to propose a methodology to analyze and estimate vulnerability, we ignore possible econometric complications that are not directly relevant. However, by all means the results presented here are to be considered preliminary.				

**Structured summary of operationalization – validity assessment**			**1.1 DCM Appropriate**	**1.2 valid empirical rep?**	**1. conclusion - Valid?**	**2. Feasible?**
**Construct:** Current socio-economic characteristics						
**Article:** Capaldo et al (2010)						
Criterion	Assessment	Quoted text or Rationale for negative assessment				
Construct defined?	YES	conceptual framework drawn from it by Løvendal and Knowles (2005).			CAN'T TELL	
Data collection methods reported?	2ndary data	We analyze a sample of 1831 rural households from Nicaragua, surveyed in the 2001 Encuesta Nacional de Hogares Sobre Medición de Nivel de Vida, by the Instituto Nacional de Estadísticas y Censos INEC de Nicaragua. Constructed variables used in the analysis were prepared by the Rural Income Generating Activities (RIGA) project team at FAO.	CAN'T TELL			
Reporting of indicators/questions used to operationalise construct?	Yes	Information on the structure of a household includes the age of the head of household (which is also a proxy for working experience), gender, marital status, language spoken (as a proxy for households belonging to an indigenous group) and the share of female labor. The latter also approximates labor availability within the household. We observed a relatively high proportion of single- or female-headed households (23% and 18% respectively).		YES		

		Household assets are assessed in using education, as well as wealth-related variables (number of rooms, cement floor, telephone, access to safe water, bikes, radios, TV sets owned4), and social capital different<br>through participation of members in community organizations. Moreover, types of livestock and land assets are also taken into account to approximate<br>household wealth and potential credit-related constraints. We use access to a network for migration as a measure of the ability of a household to receive assistance from members living outside the location and as a proxy of a diversified income portfolio. Distance from a road, school, and health facilities, are variables used for measuring a household's access to infrastructure.<br>[...]<br>Table 1: Summary of variables<br>Kilocalories per capita<br>Age of hh head<br>Highest education in hh<br>Single head<br>Female head of hh widow<br>Female headed hh<br>Hh labor<br>Indigenous household<br>Hh size<br>Rooms<br>Cement floor in house<br>Telephone in hh<br>Hh members participating in comm. org.<br>Access to hh migration network<br>Access to safe water<br>Bikes owned<br>Radios owned<br>TVs owned<br>Distance to nearest primary school<br>Time to nearest health facility<br>Distance to nearest major road<br>Land owned				

		Cattle<br>Pigs<br>Horses<br>Land operated<br>Access to irrigation<br>Income from farming activities<br>Income from farm sales				
Sampling strategies reported?	2ndary data	We analyze a sample of 1831 rural households from Nicaragua, surveyed in the 2001 Encuesta Nacional de Hogares Sobre Medición de Nivel de Vida, by the Instituto Nacional de Estadísticas y Censos INEC de Nicaragua. Constructed variables used in the analysis were prepared by the Rural Income Generating Activities (RIGA) project team at FAO.				
Sampling sizes reported?	Yes	sample of 1831 rural households				
Data analysis methods reported?	Yes	After accounting for heteroskedasticity through the use of generalized least squares, we estimate vulnerability to food insecurity as the normal probability that the "individual minimum dietary energy requirement under light physical activity" exceeds the expected individual dietary energy consumption (measured in kilocalories). Since the main purpose of this paper is to propose a methodology to analyze and estimate vulnerability, we ignore possible econometric complications that are not directly relevant. However, by all means the results presented here are to be considered preliminary.				

Transparency Assessment Article summary	
**Article**	Chhinh & Poch (2012)
**Transparent operationalizations**	Household characteristics
**Partially transparent**	
**Not transparent**	Current poverty status; environmental shocks; poverty

**Structured summary of operationalization – validity assessment**			**1.1 DCM Appropriate**	**1.2 valid empirical rep?**	**1. conclusion - Valid?**	**2. Feasible?**
**Construct:** household characteristics						
**Article:** Chhinh & Poch (2012)						
Criterion	Assessment	Quoted text or Rationale for negative assessment				
Construct defined?	Yes	This study adopts the approach to measuring household economic vulnerability posited and elaborated in Chaudhuri's (2003) study of household vulnerability			YES	
Data collection methods reported?	Yes	A total of 600 questionnaires were collected from households.	YES			
Reporting of indicators/questions used to operationalise construct?	Yes	There were on average five people within a household within the surveyed areas. This is well above the national average household size (4.7) in 2008 (NIS, 2008). Rolang Chork has the small- est average household size (4.99 people per household on average) and the highest level of educa- tion among its population (9.6 years of schooling on average). The Kork and Chbar Mon com- munes had larger household sizes than the other selected communes (5.64 and 5.59 people per household on average, respectively). About 60% of respondents reported that their households have at least one motorcycle. There was a large variation in the proportion of households possessing motorcycles between communes, with the Chbar Mon (73%), Peang Lvea (74%) and Rolang Chork (68%) communes having a high- er percentage of motorcycle-possessing households than the Tasal (44%), Kork (50%) and Morhasaing (53%) communes. The survey also revealed that 11.7% of respondents live in households with at least one person with disability. Peang Lvea commune has the highest proportion of households containing a person with a disability		YES		

		(21%), followed by Rolang Chork (13%), Kork (12%), Tasal (11%), Morhasaing (7%) and Chbar Mon (6%).				
Sampling strategies reported?	No					
Sampling sizes reported?	Yes	A total of 600 questionnaires were collected from households.				
Data analysis methods reported?	Yes	The expected log per capita income is estimated using the three-step feasible generalised least squares (FGLS) method.<br>[...]<br>Table 3 presents the results of the FGLS analysis.<br>[...]<br>Household size, the possession of motor vehicle and a livelihood dependency on agriculture<br>are significantly and inversely associated with log per capita income. Specifically, the larger the household size, the lower the expected log per capita income (the coefficient is -0.182, p<0.001). In addition, the possession of a motor vehicle is positively related to expected per capita income (the coefficient is 0.312, p<0.001); while households who depend on agricultural work alone tend to have lower per capita income than those households who have an additional secondary occupation (the coefficient is -0.899, p<0.001). In addition, the education attainment of respondents has a posi- tive effect on log per capita income, although the effect is small (the coefficient is 0.044, p < 0.001). Access to credit and the presence of person living with disability in the household does not signifi- cantly affect log per capita income.				

**Transparency Assessment Article summary**	
**Article**	Dasgupta & Baschieri (2012)
**Transparent operationalizations**	Drought; human capital; labour; non-labour productive assets; risk of experiencing climate change; social capital
**Partially transparent**	
**Not transparent**	

**Structured summary of operationalization – validity assessment**			**1.1 DCM Appropriate**	**1.2 valid empirical rep?**	**1. conclusion - Valid?**	**2. Feasible?**
**Construct:** Drought						
**Article:** Dasgupta & Baschieri (2012)						
Criterion	Assessment	Quoted text or Rationale for negative assessment				
Construct defined?	Yes	We consider the first approach and use deficiency in rainfall as the definition of<br>drought in this study.<br>[...]<br>for some, drought is defined as a deficiency in rainfall, or rainfall which is lower than the expected amount in a certain period (van der Ge			NO	
Data collection methods reported?	2ndary data	We used amap fromtheUnited Nations Food and Agriculture Organization (FAO) (2007) showing bands of annual rainfall in Ghana to estimate annual rainfall by region. The regional rankings that we obtain using this definition appear to be confirmed by other studies (Dietz et al., 2004).	NO			
Reporting of indicators/questions used to operationalise construct?	2ndary data			NO		
Sampling strategies reported?	2ndary data					
Sampling sizes reported?	2ndary data					

Data analysis methods reported?	Yes	Alogistic regression model was estimated to investigate how(a) the risk of experiencing climate change (measured by the regions ranked by annual rainfall); and (b) poverty status, is associated with vulnerability to climate change, and this is shown in Table 8. Adjusted odds ratios (OR) are also displayed, controlling for either region or poverty status. The dependent variable is vulnerability group. Table 8 shows how as annual rainfall decreases (regions are ranked in order of decreasing rainfall), the crude odds of being in the most vulnerable group tends to increase. That is to say, the odds of being in the most vulnerable group increases as risk of experiencing a climate change shock increases.				

**Structured summary of operationalization – validity assessment**			**1.1 DCM Appropriate**	**1.2 valid empirical rep?**	**1. conclusion - Valid?**	**2. Feasible?**
**Construct:** Human capital						
**Article:** Dasgupta & Baschieri (2012)						
Criterion	Assessment	Quoted text or Rationale for negative assessment				
Construct defined?	Yes	The second asset Moser (1998) identified is human capital. Social services that offer education, health care and economic infrastructure for water, transport and electricity help to determine the ability of households to work and to profit from that work.				
Data collection methods reported?	2ndary data	We used data collected between April 1998 and March 1999 by the fourth round of the Ghana Living Standards Survey (GLSS 4), which was funded by theWorld Bank and the Republic of Ghana. The survey instruments were designed to monitor poverty and well- being in Ghana. The GLSS 4 contains information on the demographic characteristics of household members, their reported health status, education, employment, housing and income from wages, business activities and agricultural production and detailed records of consumption and expenditure data. The main data file contained household-level information and derived money-metric measures of poverty such as household income and	CAN'T TELL			

<table>
<tr><td></td><td></td><td>expenditure (Coulombe and McKay, 2000).</td><td></td><td></td><td></td><td></td></tr>
<tr><td>Reporting of indicators/questions used to operationalise construct?</td><td>Yes</td><td>We used the level of education of the heads of households and access to health care as proxies for human capital.
[...]
Education level was treated as binary where the household head either had achieved primary school education or less, or secondary education or higher.
[...]
We therefore included a variable to capture a household's ability to deal with increased morbidity in the vulnerability index, assuming that households without access to decent health facilities would be more likely to be affected by climate change shocks. Data of the existence of health facilities in the community were assigned to each household.We considered the existence of a hospital to be ideal, followed by that of a clinic. The third and lowest category was a household with access to neither a hospital nor clinic.
[...]
Table 2
<table>
<tr><td>ASSETS</td><td>Variable</td></tr>
<tr><td rowspan="2">Human Capital</td><td>Household head education level (N¼3679)
Primary or less
Secondary or more</td></tr>
<tr><td>Access to an hospital care (N¼3559)
No health clinic/hospital
Access to a clinic
Access to an hospital</td></tr>
</table></td><td></td><td>NO</td><td></td><td></td></tr>
<tr><td>Sampling strategies reported?</td><td>Yes</td><td>TheGLSS4 is a two-stage probability-proportional-to-size sample.</td><td></td><td></td><td></td><td></td></tr>
<tr><td>Sampling sizes reported?</td><td>Yes</td><td>The sample contains data for5998households, of which 3799 resided in rural areas, with 25 694 eligible individual household members. We excluded the Greater Accra area as it is semi-urban, leaving 3679 rural households. In addition to the household survey, the GLSS 4 team (supervisor and enumerator) administered a community questionnaire to community leaders of the rural enumeration areas that were</td><td></td><td></td><td></td><td></td></tr>
</table>

		surveyed. One questionnaire was administered to each of the 195 rural enumeration areas.				
Data analysis methods reported?	Yes	Table 4 shows the weight assigned to each variable derived from the first principal component. The data reduction that PCA performed on the data explained 25 per cent of the original variation of the data. The PCA factor loadings were examined, and the principal component tended to load positively on variables which contributed to lower vulnerability such as better education and better health, and negatively on variables which contributed to higher household vulnerability such as higher percentage of household income from agriculture. Therefore, the score is a measure of 'strength' or preparedness. A high score indicates a non-vulnerable household, and a low score indicates a vulnerable household.				

**Structured summary of operationalization – validity assessment**			**1.1 DCM Appropriate**	**1.2 valid empirical rep?**	**1. conclusion - Valid?**	**2. Feasible?**
**Construct:** Labour						
**Article:** Dasgupta & Baschieri (2012)						
Criterion	Assessment	Quoted text or Rationale for negative assessment				
Construct defined?	Yes	The first asset Moser identified is labour			NO	
Data collection methods reported?	2ndary data	We used data collected between April 1998 and March 1999 by the fourth round of the Ghana Living Standards Survey (GLSS 4), which was funded by theWorld Bank and the Republic of Ghana. The survey instruments were designed to monitor poverty and well- being in Ghana. The GLSS 4 contains information on the demographic characteristics of household members, their reported health status, education, employment, housing and income from wages, business activities and agricultural production and detailed records of consumption and expenditure data. The main data file contained household-level information and derived money-metric measures of poverty such as household income and expenditure (Coulombe and McKay, 2000).	CAN'T TELL			
Reporting of indicators/questions used to operationalise	Yes	The primary type of work in which the head of the household was engaged was included into the vulnerability index. This variable was binary, the categories being either in agricultural work or not. The		NO		

<table>
<tr><td>construct?</td><td></td><td>percentage of total income derived from agriculture was also included, with a high percentage being taken to indicate more vulnerable households.We created this variable by dividing household income from agriculture by the total household income.
[...]
We also considered the percentage of income derived from remittances.
[...]
A variable detailing the proportion of the household that is under 15 or over the age of 65 was included, to reflect how many dependents there are in a household who are less likely to be contributing economically. Finally, we considered the percentage of total household expenditure spent on food.
[...]
Table 2
<table>
<tr><td>ASSETS</td><td>Variable</td></tr>
<tr><td rowspan="5">Labour capital</td><td>Type of work of household head (N¼3546)
Household head works in agriculture
Household head does not work in agriculture</td></tr>
<tr><td>Percent of income that comes from agriculture (N¼3679)</td></tr>
<tr><td>Household income that comes from remittances (N¼3679)
Under 10%
Over 10%</td></tr>
<tr><td>Percent of expenditure on food (N¼3679)</td></tr>
<tr><td>Percent of household that are dependent (N¼3679)</td></tr>
</table></td><td></td><td></td><td></td><td></td></tr>
<tr><td>Sampling strategies reported?</td><td>Yes</td><td>TheGLSS4 is a two-stage probability-proportional-to-size sample.</td><td></td><td></td><td></td><td></td></tr>
<tr><td>Sampling sizes reported?</td><td>Yes</td><td>The sample contains data for5998households, of which 3799 resided in rural areas, with 25 694 eligible individual household members. We excluded the Greater Accra area as</td><td></td><td></td><td></td><td></td></tr>
</table>

		it is semi-urban, leaving 3679 rural households. In addition to the household survey, the GLSS 4 team (supervisor and enumerator) administered a community questionnaire to community leaders of the rural enumeration areas that were surveyed. One questionnaire was administered to each of the 195 rural enumeration areas.				
Data analysis methods reported?	Yes	Table 4 shows the weight assigned to each variable derived from the first principal<br>component. The data reduction that PCA performed on the data explained 25 per cent of the original variation of the data. The PCA factor loadings were examined, and the principal component tended to load<br>positively on variables which contributed to lower vulnerability such as better education and better health, and negatively on variables which contributed to higher household vulnerability such as higher percentage of household income from agriculture. Therefore, the score is a measure of 'strength' or preparedness. A high score indicates a non-vulnerable household, and a low score indicates a vulnerable household.				

**Structured summary of operationalization – validity assessment**			**1.1 DCM Appropriate**	**1.2 valid empirical rep?**	**1. conclusion - Valid?**	**2. Feasible?**
**Construct:** Non-labour productive assets						
**Article:** Dasgupta & Baschieri (2012)						
Criterion	Assessment	Quoted text or Rationale for negative assessment				
Construct defined?	Yes	The first asset Moser identified is labour				
Data collection methods reported?	Yes	Non-labour productive assets are the third type. Moser (1998) identified land, sewing<br>machines, radios, refrigerators and motor vehicles as important productive assets for rural households, which can either be used or sold in order to buffer short-term climatic shocks.	CAN'T TELL			
Reporting of indicators/questions used to	2ndary data	We used data collected between April 1998 and March 1999 by the fourth round of the Ghana Living Standards Survey (GLSS 4), which was funded by theWorld Bank and the		YES		

<table>
<tr><td>operationalise construct?</td><td></td><td>Republic of Ghana. The survey instruments were designed to monitor poverty and well- being in Ghana. The GLSS 4 contains information on the demographic characteristics of household members, their reported health status, education, employment, housing and income from wages, business activities and agricultural production and detailed records of consumption and expenditure data. The main data file contained household-level information and derived money-metric measures of poverty such as household income and expenditure (Coulombe and McKay, 2000).</td><td></td><td></td><td></td><td></td></tr>
<tr><td>Sampling strategies reported?</td><td>Yes</td><td>In order to measure the different degrees of productive assets between households we used the total number of productive assets owned by the household as a proxy. Among reproducible capital assets the questionnaire included furniture, sewing machines, stoves, refrigerator-freezers, air conditioners, fans, radios, radio-cassette players, record players, three-in-one radio-cassette players, video equipment, washing machines, TVs, cameras, electric irons, bicycles, motorcycles, cars, houses, land, shares, boats, canoes and outboard motors. Each asset was weighted equally.
[...]
Table 2<table><tr><td>ASSETS</td><td>Variable</td></tr><tr><td>Productive assets</td><td>Number of productive asset (N¼3679)</td></tr></table></td><td></td><td></td><td></td><td></td></tr>
<tr><td>Sampling sizes reported?</td><td>Yes</td><td>TheGLSS4 is a two-stage probability-proportional-to-size sample.</td><td></td><td></td><td></td><td></td></tr>
<tr><td>Data analysis methods reported?</td><td>Yes</td><td>The sample contains data for5998households, of which 3799 resided in rural areas, with 25 694 eligible individual household members. We excluded the Greater Accra area as it is semi-urban, leaving 3679 rural households. In addition to the household survey, the GLSS 4 team (supervisor and enumerator) administered a community questionnaire to community leaders of the rural enumeration areas that were surveyed. One questionnaire was administered to each of the 195 rural enumeration areas.</td><td></td><td></td><td></td><td></td></tr>
<tr><td></td><td>Yes</td><td>Table 4 shows the weight assigned to each variable derived</td><td></td><td></td><td></td><td></td></tr>
</table>

		from the first principal component. The data reduction that PCA performed on the data explained 25 per cent of the original variation of the data. The PCA factor loadings were examined, and the principal component tended to load positively on variables which contributed to lower vulnerability such as better education and better health, and negatively on variables which contributed to higher household vulnerability such as higher percentage of household income from agriculture. Therefore, the score is a measure of 'strength' or preparedness. A high score indicates a non-vulnerable household, and a low score indicates a vulnerable household.				

**Structured summary of operationalization – validity assessment**			**1.1 DCM Appropriate**	**1.2 valid empirical rep?**	**1. conclusion - Valid?**	**2. Feasible?**
**Construct:** risk of experiencing climate change						
**Article:** Dasgupta & Baschieri (2012)						
Criterion	Assessment	Quoted text or Rationale for negative assessment				
Construct defined?	Yes	We use average annual rainfall data, which serves as a proxy for risk of climate-change-related shock.			NO	
Data collection methods reported?	2ndary data	We used amap fromtheUnited Nations Food and Agriculture Organization (FAO) (2007) showing bands of annual rainfall in Ghana to estimate annual rainfall by region. The regional rankings that we obtain using this definition appear to be confirmed by other studies (Dietz et al., 2004).	NO			
Reporting of indicators/questions used to operationalise construct?	2ndary data			NO		
Sampling strategies reported?	2ndary data					
Sampling sizes reported?	2ndary data					
Data analysis	Yes	Alogistic regression model was estimated to investigate				

methods reported?		how(a) the risk of experiencing<br>climate change (measured by the regions ranked by annual rainfall); and (b) poverty status, is associated with vulnerability to climate change, and this is shown in Table 8. Adjusted odds ratios (OR) are also displayed, controlling for either region or poverty status. The dependent variable is vulnerability group. Table 8 shows how as annual rainfall decreases (regions are ranked in order of<br>decreasing rainfall), the crude odds of being in the most vulnerable group tends to increase. That is to say, the odds of being in the most vulnerable group increases as risk of experiencing a climate change shock increases.				
	Yes	Table 4 shows the weight assigned to each variable derived from the first principal<br>component. The data reduction that PCA performed on the data explained 25 per cent of the original variation of the data. The PCA factor loadings were examined, and the principal component tended to load<br>positively on variables which contributed to lower vulnerability such as better education and better health, and negatively on variables which contributed to higher household vulnerability such as higher percentage of household income from agriculture. Therefore, the score is a measure of 'strength' or preparedness. A high score indicates a non-vulnerable household, and a low score indicates a vulnerable household.				

**Structured summary of operationalization – validity assessment**			**1.1 DCM Appropriate**	**1.2 valid empirical rep?**	**1. conclusion - Valid?**	**2. Feasible?**
**Construct:** Social capital						
**Article:** Dasgupta & Baschieri (2012)						
Criterion	Assessment	Quoted text or Rationale for negative assessment				
Construct defined?	Yes	Social capital isMoser'sfifth asset as it reduces vulnerability and increases opportunities.<br>Moser and Felton (2007: p. 13) defined social capital as 'the rules, norms, obligations, reciprocity and trust embedded in				

		social relations, social structures and societies' institutional arrangements.' Social capital is generally provided through membership of social networks which can be bonded in a formal or informal nature. Social capital can also be enhanced through social learning and adaptive governance (Olsson et al., 2004; Folke et al., 2005; Pelling and High, 2005; Pelling, 2007). Adaptive governance as a dynamic management approach of social-ecological systems has proven itself particularly useful in periods of crisis as it utilises social sources and social learning, drawing on experiences and common understanding and policies of different groups. In the specific context of climate change a number of studies have identified social capital as important in enhancing the community adaptive capacity to climate change (Adger, 2003; van der Geest, 2004; Bryan et al., 2009)<br>[...]<br>We consider social capital in its widest sense as social-resource networks, social groups,<br>trust and reciprocity				
Data collection methods reported?	2ndary data	We used data collected between April 1998 and March 1999 by the fourth round of the Ghana Living Standards Survey (GLSS 4), which was funded by theWorld Bank and the Republic of Ghana. The survey instruments were designed to monitor poverty and well- being in Ghana. The GLSS 4 contains information on the demographic characteristics of household members, their reported health status, education, employment, housing and income from wages, business activities and agricultural production and detailed records of consumption and expenditure data. The main data file contained household-level information and derived money-metric measures of poverty such as household income and expenditure (Coulombe and McKay, 2000).	NO			
Reporting of indicators/questions used to operationalise construct?	Yes	social capital is often considered difficult to operationalise in a household survey as it can operate at different levels and scales.We used a variable from the community questionnaire to serve as a proxy. This variable iswhether a system of mutual aid forfieldworkexistedamongthe farmers of thehousehold'scommunity.		NO		

<table>
<tr><td></td><td></td><td>[...]
We consider social capital in its widest sense as social-resource networks, social groups,
trust and reciprocity. For this reason, we also include whether there is a road near the community to which its members have access, as it can be argued that roads are one type of proxy for the extent to which communities are able to interact with the outside world and potentially receive assistance (Sachs, 2005). This information was available in the community-level data.We divided this variable into three main categories: (a) Yes, always usable, (b) Yes, sometimes unusable, (c) No road.
[...]
Table 2
<table><tr><td>ASSETS</td><td>Variable</td></tr><tr><td rowspan="2">Social Capital</td><td>System of mutual aid amongst farmers (N¼3559)
There is a system of mutual aid
No System of mutual aid</td></tr><tr><td>Road nearby (N¼3559)
No
Yes sometime unusable
Yes always usable</td></tr></table></td><td></td><td></td><td></td><td></td></tr>
<tr><td>Sampling strategies reported?</td><td>Yes</td><td>TheGLSS4 is a two-stage probability-proportional-to-size sample.</td><td></td><td></td><td></td><td></td></tr>
<tr><td>Sampling sizes reported?</td><td>Yes</td><td>The sample contains data for5998households, of which 3799 resided in rural areas, with 25 694 eligible individual household members. We excluded the Greater Accra area as it is semi-urban, leaving 3679 rural households. In addition to the household survey, the GLSS 4 team (supervisor and enumerator) administered a community questionnaire to community leaders of the rural enumeration areas that were surveyed. One questionnaire was administered to each of the 195 rural enumeration areas.</td><td></td><td></td><td></td><td></td></tr>
<tr><td>Data analysis methods reported?</td><td>Yes</td><td>Table 4 shows the weight assigned to each variable derived from the first principal</td><td></td><td></td><td></td><td></td></tr>
</table>

		component. The data reduction that PCA performed on the data explained 25 per cent of the original variation of the data. The PCA factor loadings were examined, and the principal component tended to load positively on variables which contributed to lower vulnerability such as better education and better health, and negatively on variables which contributed to higher household vulnerability such as higher percentage of household income from agriculture. Therefore, the score is a measure of 'strength' or preparedness. A high score indicates a non-vulnerable household, and a low score indicates a vulnerable household.				
	Yes	Table 4 shows the weight assigned to each variable derived from the first principal component. The data reduction that PCA performed on the data explained 25 per cent of the original variation of the data. The PCA factor loadings were examined, and the principal component tended to load positively on variables which contributed to lower vulnerability such as better education and better health, and negatively on variables which contributed to higher household vulnerability such as higher percentage of household income from agriculture. Therefore, the score is a measure of 'strength' or preparedness. A high score indicates a non-vulnerable household, and a low score indicates a vulnerable household.				

**Transparency Assessment Article summary**	
**Article**	Deressa et al (2009)
**Transparent operationalizations**	Minimum consumption (income) level
**Partially transparent**	
**Not transparent**	

**Structured summary of operationalization – validity assessment**			**1.1 DCM Appropriate**	**1.2 valid empirical rep?**	**1. conclusion - Valid?**	**2. Feasible?**
**Construct:** Minimum consumption (income) level						
**Article:** Deressa et al (2009)						
Criterion	Assessment	Quoted text or Rationale for negative assessment				
Construct defined?	Yes	a given minimum level (such as a consumption poverty line)	Yes/ no/ can't tell	Yes/ no/ can't tell	Yes/ no/ can't tell<br><br>CAN'T TELL	Yes/ no/ can't tell
Data collection methods reported?	n/a	n/a	CAN'T TELL			
Reporting of indicators/questions used to operationalise construct?	Yes	Using the procedures discussed in Section 3 (applied through the STATA software), we estimate the probability of a household falling below a given level of income (poverty line), and perform a sensitivity analysis by examining this probability using four different minimum levels of income (poverty lines). The choice of minimum levels of income is based on different assumptions such as the international poverty line of 1.25 US per day (World Bank, 2008), average income of the surveyed households and arbitrary values above and below the average income of the surveyed households. The results are plotted in Figures 3 to 6.<br>[...]<br>Figure 3. Vulnerability (income at 2 USD per day or 6570 Ethiopian Birr per year) plotted against Ln (income)<br>[...]		CAN'T TELL		

		Figure 4. Vulnerability (income at 1.5 USD per day or 4928 Ethiopian Birr per year) plotted against Ln (income)<br>[...]<br>Figure 5. Vulnerability (income at 1.25 USD per day or 4471 Ethiopian Birr per year) plotted against Ln (income)<br>[...]<br>Figure 6. Vulnerability (income at 0.3 USD per day or 900 Ethiopian Birr per year) plotted against Ln (income)				
Sampling strategies reported?	n/a	n/a				
Sampling sizes reported?	n/a	n/a				
Data analysis methods reported?	Yes	Using the procedures discussed in Section 3 (applied through the STATA software), we estimate the probability of a household falling below a given level of income (poverty line), and perform a sensitivity analysis by examining this probability using four different minimum levels of income (poverty lines). The choice of minimum levels of income is based on different assumptions such as the international poverty line of 1.25 US per day (World Bank, 2008), average income of the surveyed households and arbitrary values above and below the average income of the surveyed households. The results are plotted in Figures 3 to 6.<br>[...]<br>Figure 3. Vulnerability (income at 2 USD per day or 6570 Ethiopian Birr per year) plotted against Ln (income)<br>[...]<br>Figure 4. Vulnerability (income at 1.5 USD per day or 4928 Ethiopian Birr per year) plotted against Ln (income)<br>[...]<br>Figure 5. Vulnerability (income at 1.25 USD per day or 4471 Ethiopian Birr per year) plotted against Ln (income)<br>[...]<br>Figure 6. Vulnerability (income at 0.3 USD per day or 900 Ethiopian Birr per year) plotted against Ln (income)				
	Yes	Table 4 shows the weight assigned to each variable derived from the first principal				

		component. The data reduction that PCA performed on the data explained 25 per cent of the original variation of the data. The PCA factor loadings were examined, and the principal component tended to load positively on variables which contributed to lower vulnerability such as better education and better health, and negatively on variables which contributed to higher household vulnerability such as higher percentage of household income from agriculture. Therefore, the score is a measure of 'strength' or preparedness. A high score indicates a non-vulnerable household, and a low score indicates a vulnerable household.				

Transparency Assessment Article summary	
**Article**	Eakin et al (2012)
**Transparent operationalizations**	Impacts & responses to Hurricane Stan by coffee farmers
**Partially transparent**	
**Not transparent**	

**Structured summary of operationalization – validity assessment**<br>**Construct:** Impacts & responses to Hurricane Stan by coffee farmers<br>**Article:** Eakin et al (2012)			**1.1 DCM Appropriate**	**1.2 valid empirical rep?**	**1. conclusion - Valid?**	**2. Feasible?**
Criterion	Assessment	Quoted text or Rationale for negative assessment				
Construct defined?	Yes	In this paper, we document household responses to a climatic shock, Stan, to gain insight into how natural resource- dependent communities move to secure their livelihoods following significant loss, the implications of household responses for coffee farming as a ''domain of attraction,'' as well as to highlight those aspects of household choices and perceptions that may be indicative of resilience at broader scales.	Yes/ no/ can't tell	Yes/ no/ can't tell	Yes/ no/ can't tell<br><br>YES	Yes/ no/ can't tell<br><br>YES
Data collection methods reported?	Yes	This study is based on 64 household surveys and additional in-depth expert and key-informant interviews, conducted in 2006 and 2007.	YES			
Reporting of indicators/questions used to operationalise construct?	Yes	The surveys, implemented 18 months following Stan, collected information regarding pre- and post-Hurricane Stan activities and income sources, house- hold demographics, land holdings, production attributes, hurricane impacts (to property, production and health and welfare), household assets before and after Stan and access to agricultural and emergency response services. As described later, the survey also captured households' per- ceptions and attitudes about the disaster and their suscep- tibility to damage.<br>[...]<br>Table 2 Household assets in 2005 by impact class		YE.		

		Land (in ha) in 2005<br>Percent land in coffee in 2005<br>Number of plots owned in 2005<br>Percentage of these plots in coffee in 2005<br>Coffee production in 2005 (kg)<br>Coffee yields in 2005 (kg/Ha)<br>Percentage of land in riverbed<br>Planted maize (subsistence) in 2005<br>Percentage of households reporting poultry as very important for livelihood in 2005<br><br>[...]<br>Table 3 Impacts of Stan by impact cluster<br>Coffee harvest loss<br>Soil loss<br>Average # of days with difficulty in acquiring basic needs following the hurricane<br>Percentage of households reporting<br>Total damages to the house<br>Loss of coffee production equipment<br>Impacts to their health due to the hurricane<br><br>[...]<br>Table 4 Income profiles pre- and post-Stan (2005 and 2007)<br>Percentage of household who received income from<br>Coffee<br>Other crops and/or cattle<br>Agricultural wage laborer<br>Non-farm activities<br>Subsidies, pensions or other governmental support<br>Remittances<br>Number of income sources<br><br>[...]<br>Table 5 Post-Stan actions by impact cluster				

		Bought or rented new land<br>New land for subsistence crops (maize and/or beans)<br>Invested in soil conservation<br>Shifted efforts to a new job<br>Invested in hurricane protection<br>Planted a new crop<br>Planted shade trees<br>[...]<br>Fig. 3 Recovery time for households and the community following Stan (Household was specifically asked: ''How much time do you feel is necessary for your household [community] to fully recover from Stan?'' Source: Authors' household survey)				
Sampling strategies reported?	Yes	Three of the most affected communities by Hurricane Stan in the municipio of Siltepec, Vega de Guerrero (pop. 410), Vicente Guerrero (pop. 151) and San Bartolo (pop. 185) were purposely selected for study on the basis of prior experience of one of the investigators in the region.1 Within each community, households were selected using a systematic random sample based on an estimation of the number of coffee producing households and the density of the population in each community. In addition to the surveys, interviews were conducted with community leaders, municipal authorities and local representatives of Civil Protection, the state and federal disaster management agency and the Chiapas Council for Coffee Development and Promotion (Comisio´n para el Desarrollo y Fomento del Cafe´ de Chiapas or COMCAFE).<br>1 Access to communities for research in Chiapas requires that the researchers have time to develop the necessary trust and collaborative relationships with community members. Because the focus of this research was on a specific disaster event, timeliness was of essence. We thus selected communities for our research that had been significantly affected by the event (as reported in official statistics) and where prior research activities permitted access.				

Sampling sizes reported?	Yes	64 household surveys				
Data analysis methods reported?	Yes	As a heuristic tool to aid in our interpretation of impacts and responses to Stan, we categorized households accord- ing to the exposure of their production systems to Hurri- cane Stan into impact clusters. The impact clusters were created using a two-step cluster method available through the statistical software, PASW 18. Two-step cluster anal- ysis uses a distance criterion (log-likelihood) to define optimal number of clusters and allows for handling a mixture of categorical and (standardized) continuous vari- ables (Zhang et al. 1996; Chiu et al. 2001).<br>[...]<br>We used two ‘‘loss’’ variables as the input data for the creation of clusters: percent of coffee harvest and soil lost due to Hurricane Stan. We chose these two variables because of the fundamental economic role played by coffee production for households in Siltepec in 2005.<br>[...]<br>We then used these clusters to explore two questions through a descriptive analysis of the remaining survey variables: What were the characteristics of house- holds that experienced specific degrees of loss? What were their responses?				
	Yes	Table 4 shows the weight assigned to each variable derived from the first principal<br>component. The data reduction that PCA performed on the data explained 25 per cent of the original variation of the data. The PCA factor loadings were examined, and the principal component tended to load<br>positively on variables which contributed to lower vulnerability such as better education and better health, and negatively on variables which contributed to higher household vulnerability such as higher percentage of household income from agriculture. Therefore, the score is a measure of ‘strength’ or preparedness. A high score indicates a non-vulnerable household, and a low score				

		indicates a vulnerable household.				

**Transparency Assessment Article summary**	
**Article**	Echevin (2011)
**Transparent operationalizations**	Community level; household level
**Partially transparent**	
**Not transparent**	

**Structured summary of operationalization – validity assessment**			**1.1 DCM Appropriate**	**1.2 valid empirical rep?**	**1. conclusion - Valid?**	**2. Feasible?**
**Construct:** Community level						
**Article:** Echevin (2011)						
Criterion	Assessment	Quoted text or Rationale for negative assessment				
Construct defined?	Yes	an extension of this empirical framework will consist in using two-level (i.e. household and community levels) modelling of the impact of those shocks following Günther and Harttgen (2009)'s approach.	Yes/ no/ can't tell	Yes/ no/ can't tell	Yes/ no/ can't tell<br><br>CAN'T TELL	Yes/ no/ can't tell<br><br>YES
Data collection methods reported?	2ndary	The vulnerability and food security survey was conducted in Haiti in October and<br>November 2007 on approximately 3,000 households living in 228 rural communities. This survey has been realized by the National Coordination of Food Security Unit with the partnership of the World Food Program. A community-related component was added to the household component of the survey, in connection with infrastructures and accessibility to basic social services.	CAN'T TELL			
Reporting of indicators/questions used to operationalise construct?	Yes	Table 2 presents summary statistics for variables used in the analysis. Consumption and<br>income are expressed in Gourdes.<br>[...]<br>The community index is a linear combination of community basic infrastructure and access to market		YES		

		variables (roads, access to elementary or secondary schools, health centres, markets, electricity and cell phone). A score of income diversity has also been built from the various income sources earned by the household. As four main income sources are declared by the household, the income diversity variable (ID) is defined as:<br>$ID_i = (1/2)[1- \sum^{4}_{k=1} (s_i^k)^2]$<br>Where s_i^k is the share of the kth income source in total income of<br>household i. This score equals 0 when only one source of income is declared by the household.<br>[...]<br><br>**Table 2. Descriptive statistics**<br>*Community variables*<br>Average years of schooling<br>Land owners<br>Community index				
Sampling strategies reported?	2ndary					
Sampling sizes reported?	Yes	3,000 households living in 228 rural communities.				
Data analysis methods reported?	Yes	We use self-reported shocks in order to estimate their impact on consumption and income.<br>Table 3 presents OLS estimates and GLLAMM estimates. Both models are estimated with log consumption and log income. Our preferred specification regroups a large set of explanatory variables such as household characteristics, regional dummies, community characteristics, interaction between household characteristics and community characteristics, shocks variables, interaction between shocks variables and household characteristics, interaction between shocks variables and community characteristics. Estimating the two-level linear random coefficient model (GLLAMM) allows us to decompose the variance of the residuals into				

		an idiosyncratic variance and a covariate variance.				
	Yes	Table 4 shows the weight assigned to each variable derived from the first principal component. The data reduction that PCA performed on the data explained 25 per cent of the original variation of the data. The PCA factor loadings were examined, and the principal component tended to load positively on variables which contributed to lower vulnerability such as better education and better health, and negatively on variables which contributed to higher household vulnerability such as higher percentage of household income from agriculture. Therefore, the score is a measure of 'strength' or preparedness. A high score indicates a non-vulnerable household, and a low score indicates a vulnerable household.				

**Structured summary of operationalization – validity assessment**			**1.1 DCM Appropriate**	**1.2 valid empirical rep?**	**1. conclusion - Valid?**	**2. Feasible?**
**Construct:** household level						
**Article:** Echevin (2011)						
Criterion	Assessment	Quoted text or Rationale for negative assessment				
Construct defined?	Yes	an extension of this empirical framework will consist in using two-level (i.e. household and community levels) modelling of the impact of those shocks following Günther and Harttgen (2009)'s approach.	Yes/ no/ can't tell	Yes/ no/ can't tell	Yes/ no/ can't tell<br><br>CAN'T TELL	Yes/ no/ can't tell<br><br>YES
Data collection methods reported?	2ndary	The vulnerability and food security survey was conducted in Haiti in October and November 2007 on approximately 3,000 households living in 228 rural communities. This survey has been realized by the National Coordination of Food Security Unit with the partnership of the World Food Program. A community-related component was added to the household component of the survey, in connection with infrastructures and accessibility to basic social services.	CAN'T TELL			
Reporting of indicators/questions	Yes	Table 2 presents summary statistics for variables used in the analysis. Consumption and		YES		

used to operationalise construct?		income are expressed in Gourdes. The agricultural index is a composite indicator which is a linear combination of categorical variables obtained from a multiple correspondence analysis (cf. Asselin, 2009). Variables considered in the analysis are the number of lands, animals and agricultural materials owned by the household.<br>[...]<br><br>**Table 2. Descriptive statistics**<br>*Household variables*<br><br>Log of consumption<br>Log of income<br>Agricultural index<br>Income diversity<br>Household size<br>Number of children<br>Age of head<br>Male head<br>Years of schooling (head)<br>Activity of head<br>No job<br>Agroalimentary<br>Industry<br>Construction<br>Trade<br>Services<br>Other activity				
Sampling strategies reported?	2ndary					
Sampling sizes reported?	Yes	3,000 households living in 228 rural communities.				
Data analysis methods reported?	Yes	We use self-reported shocks in order to estimate their impact on consumption and income.<br>Table 3 presents OLS estimates and GLLAMM estimates.				

		Both models are estimated with log consumption and log income. Our preferred specification regroups a large set of explanatory variables such as household characteristics, regional dummies, community characteristics, interaction between household characteristics and community characteristics, shocks variables, interaction between shocks variables and household characteristics, interaction between shocks variables and community characteristics. Estimating the two-level linear random coefficient model (GLLAMM) allows us to decompose the variance of the residuals into an idiosyncratic variance and a covariate variance.				
	Yes	Table 4 shows the weight assigned to each variable derived from the first principal component. The data reduction that PCA performed on the data explained 25 per cent of the original variation of the data. The PCA factor loadings were examined, and the principal component tended to load positively on variables which contributed to lower vulnerability such as better education and better health, and negatively on variables which contributed to higher household vulnerability such as higher percentage of household income from agriculture. Therefore, the score is a measure of 'strength' or preparedness. A high score indicates a non-vulnerable household, and a low score indicates a vulnerable household.				

**Transparency Assessment Article summary**	
**Article**	Gandure et al (2013)
**Transparent operationalizations**	Actual meteorological observation
**Partially transparent**	
**Not transparent**	adaptation ot long term climate change; Perception of long term climate change

**Structured summary of operationalization – validity assessment**			**1.1 DCM Appropriate**	**1.2 valid empirical rep?**	**1. conclusion - Valid?**	**2. Feasible?**
**Construct:** Actual meteorological observation						
**Article:** Gandure et al (2013)						
Criterion	Assessment	Quoted text or Rationale for negative assessment				
Construct defined?	Yes	actual meteorological observations, rainfall and temperature data obtained from the South Africa Weather Services were analysed. Rainfall and air temperature are routinely measured at various stations distributed across South Africa, although not all districts have weather stations.	Yes/ no/ can't tell	Yes/ no/ can't tell	Yes/ no/ can't tell<br><br>YES	Yes/ no/ can't tell<br><br>YES, WHERE MET STATION DATA ARE AVAILABLE
Data collection methods reported?	2ndary	Rainfall data for our analysis was obtained from the station at Thaba Nchu; for temperature; the Bloemfontein station data was used due to lack of such data for Thaba Nchu. Temperature data for Bloemfontein provided a near representation of climate conditions in Thaba Nchu. Trends of the recorded rainfall and temperature data over the last 49 years (1960–2009) were analysed	YES			
Reporting of indicators/questions used to operationalise construct?	2ndary			-		

Sampling strategies reported?	2ndary					
Sampling sizes reported?	2ndary					
Data analysis methods reported?	Yes	Rainfall data for Thaba Nchu (Fig. 1) reveals the inter-annual variability observed during 1960–2009. During 1960–2009, the district received above mean annual rainfall in half the years. In the other half of the years when there has been below mean average rainfall, the impact on water availability is likely to have been greater. In the three years (2007–2009) prior to data collection, the area has received below mean annual rainfall and these recent experiences are what the community was able to recall easily. Overall, from 1960 to 2009, the data does show high inter-annual variability which as it shall be discussed later has been experienced by the farmers. Figs. 2 and 3 show the average daily minimum (June, July, and August) and maximum (December, January, and February) temperatures from 1962–2009, respectively. Over 47 years, on average minimum temperatures display an increasing trend of 1–2 1C during June and July, with the month of June showing the greatestwarming tendencies.Maximum temperatures have remained fairly constant for December and January but have increased slightly for February by approximately 1 1Conaverage over thesameperiod.				
	Yes	Table 4 shows the weight assigned to each variable derived from the first principal component. The data reduction that PCA performed on the data explained 25 per cent of the original variation of the data. The PCA factor loadings were examined, and the principal component tended to load positively on variables which contributed to lower vulnerability such as better education and better health, and negatively on variables which contributed to higher household vulnerability such as higher percentage of household income from agriculture. Therefore, the score				

		is a measure of ‘strength’ or preparedness. A high score indicates a non-vulnerable household, and a low score indicates a vulnerable household.				

**Transparency Assessment Article summary**	
**Article**	Günther & Harttgen (2009)
**Transparent operationalizations**	Community level; covariate shocks; household level; idiosyncratic shocks; structural poverty
**Partially transparent**	
**Not transparent**	

<table>
<tr><td colspan="3">Structured summary of operationalization – validity assessment</td><td rowspan="4">1.1 DCM Appropriate</td><td rowspan="4">1.2 valid empirical rep?</td><td rowspan="4">1. conclusion - Valid?</td><td rowspan="4">2. Feasible?</td></tr>
<tr><td colspan="3">Construct: Community level</td></tr>
<tr><td colspan="3">Article: Günther & Harttgen (2009)</td></tr>
<tr><td><u>Criterion</u></td><td><u>Assessment</u></td><td><u>Quoted text or Rationale for negative assessment</u></td></tr>
<tr><td>Construct defined?</td><td>Yes</td><td>Multilevel models are designed to analyze the relationship between variables that are measured at different hierarchical levels (for an introduction see, e.g., Bryk & Raudenbush, 1992; Goldstein, 1999; Hox, 2002). We speak of ‘‘hierarchical” or ‘‘multilevel” data structure whenever variables are collected at different hierarchical levels with lower-levels (e.g., house- holds) nested within higher-levels (e.g., communities).</td><td>Yes/ no/ can’t tell</td><td>Yes/ no/ can’t tell</td><td rowspan="3">Yes/ no/ can’t tell

NO</td><td rowspan="3">Yes/ no/ can’t tell

YES</td></tr>
<tr><td>Data collection methods reported?</td><td>2ndary</td><td>The community census is the 2001 ILO/Cornell Commune Level census which covers 1,385 of the 1,395 communities in Mada- gascar. Both surveys do not have any time dimension.</td><td>CAN’T TELL</td><td></td></tr>
<tr><td>Reporting of indicators/questions used to operationalise construct?</td><td>Yes</td><td>Table 1. Summary statistics for households and communities

<table><tr><td>Table 1. Summary statistics for households and communities</td></tr><tr><td>Community characteristics</td></tr><tr><td>Bus stop (%)</td></tr><tr><td>Save water (%)</td></tr><tr><td>Electricity (%)</td></tr><tr><td>Hospital (%)</td></tr></table></td><td></td><td>NO</td></tr>
</table>

		Market (%)<br>Bank (%)<br>Fertilizer (%)<br>Community road (%)<br>Provincial road (%)<br>National road (%)<br>Secondary education facility (%)<br>Tertiary education facility (%)				
Sampling strategies reported?	2ndary					
Sampling sizes reported?	Yes	2001 ILO/Cornell Commune Level census which covers 1,385 of the 1,395 communities in Mada- gascar.				
Data analysis methods reported?	Yes	To estimate households' expected mean and variance in con- sumption, we first use the household characteristics in Table 1. In addition, we consider an agricultural asset index (composed of eight productive assets) estimated via principal component analysis (Filmer & Pritchett, 2001). At the community level, we include population density, mean educational level, the per- centage of households working in the formal sector and the percentage of households possessing an enterprise within the community. Moreover, we construct an infrastructure index, again based on principal component analysis, using fourteen characteristics reflecting the infrastructure of the community (see Table A.4 in Appendix).<br>[...]<br>As described in Section 3, we estimate the expected mean and variance per capita household (log) consumption using multilevel modeling. We also decompose the unexplained con- sumption variance into an idiosyncratic (household-level) and a covariate (community-level) component. The regression results of the multilevel model for the esti- mated mean of (log) consumption are presented in Table 2.				
	Yes	Table 4 shows the weight assigned to each variable derived from the first principal<br>component. The data reduction that PCA performed on				

		the data explained 25 per cent of the original variation of the data. The PCA factor loadings were examined, and the principal component tended to load<br>positively on variables which contributed to lower vulnerability such as better education and better health, and negatively on variables which contributed to higher household vulnerability such as higher percentage of household income from agriculture. Therefore, the score is a measure of 'strength' or preparedness. A high score indicates a non-vulnerable household, and a low score indicates a vulnerable household.				

**Structured summary of operationalization – validity assessment**			**1.1 DCM Appropriate**	**1.2 valid empirical rep?**	**1. conclusion - Valid?**	**2. Feasible?**
**Construct:** Covariate shocks						
**Article:** Günther & Harttgen (2009)						
<u>Criterion</u>	<u>Assessment</u>	<u>Quoted text or Rationale for negative assessment</u>				
Construct defined?	Yes	Households in developing countries are frequently hit by se-<br>vere idiosyncratic and covariate shocks resulting in high income volatility. 1<br>[...]<br>1. Here, and in the following, idiosyncratic shocks refer to household- specific shocks (e.g., injury, birth, death, or job loss of a household member) that are only weakly correlated across households within a community. Covariate shocks refer to shocks that are correlated across households within communities but only weakly correlated across communities (e.g., natural disasters or epidemics).	Yes/ no/ can't tell	Yes/ no/ can't tell	Yes/ no/ can't tell	Yes/ no/ can't tell
Data collection methods reported?	2ndary	Data on household characteristics are taken from the na- tional representative household survey of 2001 (Enqueˆte Aupre`s Des Me´nages), covering 5,080 households (1,778 ur- ban and 3,302 rural households) in 186 communities. The community census is the 2001 ILO/Cornell Commune Level census which covers 1,385 of the 1,395	CAN'T TELL			

		communities in Mada- gascar. Both surveys do not have any time dimension.				
Reporting of indicators/questions used to operationalise construct?	Yes	More precisely, for each community and for the three years preceding the survey (2001, 2000, 1999) it is reported whether the community was exposed to any of 16 covariate shocks (most of these are reported in Tables A.1 and A.2 in Appendix).<br>[...]<br>Table A.1. Households with exposure to shocks<br>Malaria<br>Tuberculosis<br>Typhoid<br>Cholera<br>Rice pest<br>Swineflu<br>Newcastle<br>Flooding<br>Impassible bridge or road<br>Drought<br>Cyclones		YES		
Sampling strategies reported?	2ndary					
Sampling sizes reported?	Yes	covering 5,080 households (1,778 ur- ban and 3,302 rural households) in 186 communities<br>[...]<br>2001 ILO/Cornell Commune Level census which covers 1,385 of the 1,395 communities in Mada- gascar.				
Data analysis methods reported?	Yes	The estimated average mean and variance in consumption<br>for the whole sample are presented in Table 3, also separately for rural and urban households. The expected per capita (log) consumption of rural households is below the (log) poverty line, whereas the expected per capita (log) consumption of ur- ban households lies above the (log) poverty line.<br>With regard to the estimated standard deviation in consumption, we show that the estimated standard deviation is slightly higher for rural households than for				

		urban households, with a standard deviation of 0.58 compared to 0.54 (Table 3). Idiosyn- cratic variance is much higher than covariate variance for ur- ban and only slightly higher for rural households. Hence, the relative importance of idiosyncratic variance is much higher for urban than for rural households. More precisely, whereas among urban households the estimated idiosyncratic standard deviation of consumption is 3.25 times as high as covariate standard deviation, the respective rate is only 1.57 for rural households. As a robustness check, we assume that half of the estimated idiosyncratic variance is measurement error. The idiosyncratic standard deviation is still 2.13 as high as covariate standard deviation for urban households and 1.14 as high for rural households (see Table A.3).				
	Yes	Table 4 shows the weight assigned to each variable derived from the first principal component. The data reduction that PCA performed on the data explained 25 per cent of the original variation of the data. The PCA factor loadings were examined, and the principal component tended to load positively on variables which contributed to lower vulnerability such as better education and better health, and negatively on variables which contributed to higher household vulnerability such as higher percentage of household income from agriculture. Therefore, the score is a measure of 'strength' or preparedness. A high score indicates a non-vulnerable household, and a low score indicates a vulnerable household.				

**Structured summary of operationalization – validity assessment**			**1.1 DCM Appropriate**	**1.2 valid empirical rep?**	**1. conclusion - Valid?**	**2. Feasible?**
**Construct:** Household level						
**Article:** Günther & Harttgen (2009)						
Criterion	Assessment	Quoted text or Rationale for negative assessment				
Construct defined?	Yes	Multilevel models are designed to analyze the	Yes/ no/ can't	Yes/ no/	Yes/ no/	Yes/ no/

		relationship between variables that are measured at different hierarchical levels (for an introduction see, e.g., Bryk & Raudenbush, 1992; Goldstein, 1999; Hox, 2002). We speak of ‘‘hierarchical” or ‘‘multilevel” data structure whenever variables are collected at different hierarchical levels with lower-levels (e.g., house- holds) nested within higher-levels (e.g., communities).	tell	can’t tell	can’t tell	can’t tell
Data collection methods reported?	2ndary	Data on household characteristics are taken from the na- tional representative household survey of 2001 (Enqueˆte Aupre`s Des Me´nages), covering 5,080 households (1,778 ur- ban and 3,302 rural households) in 186 communities.<br>[...]<br>Both surveys do not have any time dimension.	CAN’T TELL			
Reporting of indicators/questions used to operationalise construct?	Yes	Table 1. Summary statistics for households and communities<br><br>Table 1. Summary statistics for households and communities<br>*Household characteristics*<br>Age of HH head (years)<br>Number of children<br>Female headed households (%)<br>Household size Residence (%)<br>Years of schooling of HH head<br>Works in agriculture (HH head) (%)<br>Works in informal sector (HH head) (%)<br>Works in formal sector (HH head) (%)<br>Works in public sector (HH head) (%)<br>Enterprise owner (%)<br>Land owner (%)<br>Number of cattle		YES		
Sampling strategies reported?	Yes	na-<br>tional representative household survey of 2001				
Sampling sizes reported?	Yes	covering 5,080 households (1,778 ur- ban and 3,302 rural households) in 186 communities				
Data analysis	Yes	To estimate households’ expected mean and variance in				

methods reported?		con- sumption, we first use the household characteristics in Table 1. In addition, we consider an agricultural asset index (composed of eight productive assets) estimated via principal component analysis (Filmer & Pritchett, 2001).<br>[...]<br>As described in Section 3, we estimate the expected mean and variance per capita household (log) consumption using multilevel modeling. We also decompose the unexplained con- sumption variance into an idiosyncratic (household-level) and a covariate (community-level) component. The regression results of the multilevel model for the esti- mated mean of (log) consumption are presented in Table 2.				
	Yes	Table 4 shows the weight assigned to each variable derived from the first principal<br>component. The data reduction that PCA performed on the data explained 25 per cent of the original variation of the data. The PCA factor loadings were examined, and the principal component tended to load<br>positively on variables which contributed to lower vulnerability such as better education and better health, and negatively on variables which contributed to higher household vulnerability such as higher percentage of household income from agriculture. Therefore, the score is a measure of 'strength' or preparedness. A high score indicates a non-vulnerable household, and a low score indicates a vulnerable household.				

**Structured summary of operationalization – validity assessment**			**1.1 DCM Appropriate**	**1.2 valid empirical rep?**	**1. conclusion - Valid?**	**2. Feasible?**
**Construct:** Idiosyncratic shocks						
**Article:** Günther & Harttgen (2009)						
Criterion	Assessment	Quoted text or Rationale for negative assessment				
Construct defined?	Yes	Households in developing countries are frequently hit by se-<br>vere idiosyncratic and covariate shocks resulting in high income volatility. 1<br>[...]	Yes/ no/ can't tell	Yes/ no/ can't tell	Yes/ no/ can't tell	Yes/ no/ can't tell

		1. Here, and in the following, idiosyncratic shocks refer to household- specific shocks (e.g., injury, birth, death, or job loss of a household member) that are only weakly correlated across households within a community. Covariate shocks refer to shocks that are correlated across households within communities but only weakly correlated across communities (e.g., natural disasters or epidemics).				
Data collection methods reported?	2ndary	Data on household characteristics are taken from the na- tional representative household survey of 2001 (Enqueˆte Aupre`s Des Me´nages), covering 5,080 households (1,778 ur- ban and 3,302 rural households) in 186 communities. The community census is the 2001 ILO/Cornell Commune Level census which covers 1,385 of the 1,395 communities in Mada- gascar. Both surveys do not have any time dimension.	CAN'T TELL			
Reporting of indicators/questions used to operationalise construct?	2ndary					
Sampling strategies reported?	2ndary					
Sampling sizes reported?	Yes	covering 5,080 households (1,778 ur- ban and 3,302 rural households) in 186 communities<br>[...]<br>2001 ILO/Cornell Commune Level census which covers 1,385 of the 1,395 communities in Mada- gascar.				
Data analysis methods reported?	Yes	The estimated average mean and variance in consumption<br>for the whole sample are presented in Table 3, also separately for rural and urban households. The expected per capita (log) consumption of rural households is below the (log) poverty line, whereas the expected per capita (log) consumption of ur- ban households lies above the (log) poverty line.<br>With regard to the estimated standard deviation in consumption, we show that the estimated standard				

		deviation is slightly higher for rural households than for urban households, with a standard deviation of 0.58 compared to 0.54 (Table 3). Idiosyn- cratic variance is much higher than covariate variance for ur- ban and only slightly higher for rural households. Hence, the relative importance of idiosyncratic variance is much higher for urban than for rural households. More precisely, whereas among urban households the estimated idiosyncratic standard deviation of consumption is 3.25 times as high as covariate standard deviation, the respective rate is only 1.57 for rural households. As a robustness check, we assume that half of the estimated idiosyncratic variance is measurement error. The idiosyncratic standard deviation is still 2.13 as high as covariate standard deviation for urban households and 1.14 as high for rural households (see Table A.3).				
	Yes	Table 4 shows the weight assigned to each variable derived from the first principal component. The data reduction that PCA performed on the data explained 25 per cent of the original variation of the data. The PCA factor loadings were examined, and the principal component tended to load positively on variables which contributed to lower vulnerability such as better education and better health, and negatively on variables which contributed to higher household vulnerability such as higher percentage of household income from agriculture. Therefore, the score is a measure of 'strength' or preparedness. A high score indicates a non-vulnerable household, and a low score indicates a vulnerable household.				

**Structured summary of operationalization – validity assessment**			**1.1 DCM Appropriate**	**1.2 valid empirical rep?**	**1. conclusion - Valid?**	**2. Feasible?**
**Construct:** Structural poverty						
**Article:** Günther & Harttgen (2009)						
Criterion	Assessment	Quoted text or Rationale for negative assessment				

Construct defined?	Yes	Moreover, these poverty measures cannot assess whether high poverty rates are a cause of structural poverty (i.e., low endowments) or a cause of poverty risk (i.e., high uninsured income fluctuations), which is important to know from a policy perspective.	Yes/ no/ can't tell	Yes/ no/ can't tell	Yes/ no/ can't tell<br><br>NO	Yes/ no/ can't tell
Data collection methods reported?	2ndary	Data on household characteristics are taken from the na- tional representative household survey of 2001 (Enqueˆte Aupre`s Des Me´nages), covering 5,080 households (1,778 ur- ban and 3,302 rural households) in 186 communities. The community census is the 2001 ILO/Cornell Commune Level census which covers 1,385 of the 1,395 communities in Mada- gascar. Both surveys do not have any time dimension.	CAN'T TELL			
Reporting of indicators/questions used to operationalise construct?	Yes	Table 1. Summary statistics for households and communities<br><br>Table 1. Summary statistics for households and communities<br>*Household characteristics*<br>Age of HH head (years)<br>Number of children<br>Female headed households (%)<br>Household size Residence (%)<br>Years of schooling of HH head<br>Works in agriculture (HH head) (%)<br>Works in informal sector (HH head) (%)<br>Works in formal sector (HH head) (%)<br>Works in public sector (HH head) (%)<br>Enterprise owner (%)<br>Land owner (%)<br>Number of cattle<br>*Community characteristics*<br>Bus stop (%)<br>Save water (%)<br>Electricity (%)		NO		

		Hospital (%)<br>Market (%)<br>Bank (%)<br>Fertilizer (%)<br>Community road (%)<br>Provincial road (%)<br>National road (%)<br>Secondary education facility (%)<br>Tertiary education facility (%)				
Sampling strategies reported?	2ndary					
Sampling sizes reported?	Yes	covering 5,080 households (1,778 ur- ban and 3,302 rural households) in 186 communities<br>[...]<br>2001 ILO/Cornell Commune Level census which covers 1,385 of the 1,395 communities in Mada- gascar.				
Data analysis methods reported?	Yes	Last, we decompose vulnerability estimates into the sources<br>of vulnerability. We first analyze whether vulnerability is mainly driven by permanent low consumption prospects (i.e., structural or poverty induced vulnerability) or by high consumption volatility (i.e., transitory or risk induced vulner- ability). 18 In other words, if the (estimated) expected mean consumption ln^<br>a high estimated variance in consumption ^r2<br>mated vulnerability that is greater than the set vulnerability threshold of 0.29, then the household is said to face risk in- duced vulnerability (Figure 1). In Table 4, we see that rural vulnerability is mainly a cause<br>cij lies above the poverty line ln z, but ij leads to an esti-<br>cij of a household already lies below the pov-<br>erty line ln z, then the household is referred to as structural or poverty induced vulnerable (Figure 1). If the (estimated) ex- pected consumption ln^<br>of low expected mean in consumption whereas urban vulnera- bility is mainly driven by high consumption volatility. More precisely, 67.56% of rural households				

		have an expected per ca- pita consumption that already lies below the poverty line, and ‘‘only” 18.13% of rural households are vulnerable because of high consumption volatility. In contrast, only 7.32% of urban households face structural induced vulnerability, whereas 16.58% face risk induced vulnerability (because of high con- sumption fluctuations). Structural induced poverty is hence 3.78 times higher than risk induced poverty across rural house- holds. In contrast, urban households face more often risk in- duced than structural induced poverty (the ratio of structural to risk induced poverty is smaller one).				
	Yes	Table 4 shows the weight assigned to each variable derived from the first principal component. The data reduction that PCA performed on the data explained 25 per cent of the original variation of the data. The PCA factor loadings were examined, and the principal component tended to load positively on variables which contributed to lower vulnerability such as better education and better health, and negatively on variables which contributed to higher household vulnerability such as higher percentage of household income from agriculture. Therefore, the score is a measure of ‘strength’ or preparedness. A high score indicates a non-vulnerable household, and a low score indicates a vulnerable household.				

Transparency Assessment Article summary	
**Article**	Hahn et al (2009)
**Transparent operationalizations**	2 week illness; agriculture dependend households; average precipitation; borrow-lend ratio; crop diversity; dependency ratio; don't save crops; don't save seeds; family with cronic illness; flood, drought, cyclone events; food from family farm; households with orphans; households working elsewhere; idendependent of local government; inconsistent water suply; injury or death from disaster; inverse water stored; livelihood diversification; malaria exposure-prevention; maximum temperature; minimum temperature; natural water source; no warning of disaster; precent of female-headed households; proximity to health facility; proximity to water source; receive-give ratio; struggle for food; uneducated headed households; water conflict
**Partially transparent**	
**Not transparent**	

**Structured summary of operationalization – validity assessment**			**1.1 DCM Appropriate**	**1.2 valid empirical rep?**	**1. conclusion - Valid?**	**2. Feasible?**
**Construct:** 2 week illness						
**Article:** Hahn et al (2009)						
Criterion	Assessment	Quoted text or Rationale for negative assessment				
Construct defined?	Yes	Percentage of households that report at least 1 family member who had to miss school of work due to illness in the last 2 weeks.	Yes/ no/ can't tell	Yes/ no/ can't tell	Yes/ no/ can't tell<br><br>YES	Yes/ no/ can't tell<br><br>YES
Data collection methods reported?	Yes	household surveys	YES			
Reporting of indicators/questions used to operationalise construct?	Yes	Table 1 includes an explanation of how each sub-component was quantified, the survey question used to collect the data, the original source of the survey question, and potential sources of bias.<br>[...]<br><br>Table 1 Major components and sub-components comprising the Livelihood Vulnerability Index (LVI) developed for two districts of Mozambique.		YES		

		Sub-components | *Explanation of sub-components* | *Survey question*<br>Percent of households where a family member had to miss work or school in the last 2 weeks due to illness | Percentage of households that report at least 1 family member who had to miss school of work due to illness in the last 2 weeks. | Has anyone in your family been so sick in the past 2 weeks that they had to miss work or school?				
Sampling strategies reported?	Yes	We pilot tested the LVI and LVI–IPCC in the Moma and Mabote<br>Districts of Mozambique during 2007. These were selected by CARE-Mozambique as representative of coastal and inland communities, respectively, and the climate change issues con- fronting each.<br>[...]<br>Based on a sample size calculation (WHO, 2005) at the 95% confidence interval, [illegible]10% precision, 50% prevalence,1 and a design effect of 2 to account for cluster sampling, 200 households in each<br>district were surveyed.2 National 1997 census data that specified the total population in each village was used to select 20 villages in each district using the probability proportional to size method (WHO, 2005; UNICEF, 2008).<br>[...]<br>1 50% prevalence refers to the point prevalence of the indicators selected for the LVI. This is the default value for sample size calculations when the prevalence of the indicators is unknown.<br>2 Sample size formula: N = DEFF*[(Z2*p*q)/e2], where N = sample size, DEFF = 2; Z = 1.96 (95% CI), p = 0.5; q = 0.5; e = 0.10.				
Sampling sizes reported?	Yes	We pilot tested the LVI and LVI–IPCC in the Moma and Mabote<br>Districts<br>[...]<br>200 households in each				

		district were surveyed.2 National 1997 census data that specified the total population in each village was used to select 20 villages in each district				
Data analysis methods reported?	Yes	The LVI uses a balanced weighted average approach (Sullivan<br>et al., 2002) where each sub-component contributes equally to the overall index even though each major component is comprised of a different number of sub-components. Becausewe intended to develop an assessment tool accessible to a diverse set of users in resource-poor settings, the LVI formula uses the simple approach of applying equal weights to all major components. This weighting scheme could be adjusted by future users as needed. Because each of the sub-components is measured on a different<br>scale, it was first necessary to standardize each as an index. The equation used for this conversion was adapted from that used in the Human Development Index to calculate the life expectancy index, which is the ratio of the difference of the actual life expectancy and a pre-selected minimum, and the range of pre- determined maximum and minimum life expectancy (UNDP, 2007):<br>$index_{sd} = (s_d - s_{min})/(s_{max} - s_{min})$<br>where sd is the original sub-component for district d, and smin and smax are the minimum and maximum values, respectively, for each sub-component determined using data from both districts.				
	Yes	Table 4 shows the weight assigned to each variable derived from the first principal<br>component. The data reduction that PCA performed on the data explained 25 per cent of the original variation of the data. The PCA factor loadings were examined, and the principal component tended to load<br>positively on variables which contributed to lower vulnerability such as better education and better health, and negatively on variables which contributed to higher household vulnerability such as higher percentage of household income from agriculture. Therefore, the score				

		is a measure of 'strength' or preparedness. A high score indicates a non-vulnerable household, and a low score indicates a vulnerable household.				

Structured summary of operationalization – validity assessment			1.1 DCM Appropriate	1.2 valid empirical rep?	1. conclusion - Valid?	2. Feasible?
**Construct:** agriculture dependend households						
**Article:** Hahn et al (2009)						
Criterion	Assessment	Quoted text or Rationale for negative assessment				
Construct defined?	Yes	Percentage of households that report only agriculture as a source of income.	Yes/ no/ can't tell	Yes/ no/ can't tell	Yes/ no/ can't teLL<br><br>YES	Yes/ no/ can't tell<br>YES
Data collection methods reported?	Yes	household surveys	YES			
Reporting of indicators/questions used to operationalise construct?	Yes	Table 1 includes an explanation of how each sub-component was quantified, the survey question used to collect the data, the original source of the survey question, and potential sources of bias.<br>[...]<br><br>Table 1 Major components and sub-components comprising the Livelihood Vulnerability Index (LVI) developed for two districts of Mozambique.<br>*Sub-components* / *Explanation of sub-components* / *Survey question*<br>Percent of households dependent solely on agriculture as a source of income / Percentage of households that report only agriculture as a source of income. / Do you or someone else in your household raise animals? Do you or someone else in your household grow crops? Do you or someone else in your household collect		YES		

		something from the bush, the forest, or lakes and rivers to sell?				
Sampling strategies reported?	Yes	We pilot tested the LVI and LVI–IPCC in the Moma and Mabote<br>Districts of Mozambique during 2007. These were selected by CARE-Mozambique as representative of coastal and inland communities, respectively, and the climate change issues con- fronting each.<br>[...]<br>Based on a sample size calculation (WHO, 2005) at the 95% confidence interval, ±10% precision, 50% prevalence,1 and a design effect of 2 to account for cluster sampling, 200 households in each<br>district were surveyed.2 National 1997 census data that specified the total population in each village was used to select 20 villages in each district using the probability proportional to size method (WHO, 2005; UNICEF, 2008).<br>[...]<br>1 50% prevalence refers to the point prevalence of the indicators selected for the LVI. This is the default value for sample size calculations when the prevalence of the indicators is unknown.<br>2 Sample size formula: N = DEFF*[(Z2*p*q)/e2], where N = sample size, DEFF = 2; Z = 1.96 (95% CI), p = 0.5; q = 0.5; e = 0.10.				
Sampling sizes reported?	Yes	We pilot tested the LVI and LVI–IPCC in the Moma and Mabote<br>Districts<br>[...]<br>200 households in each<br>district were surveyed.2 National 1997 census data that specified the total population in each village was used to select 20 villages in each district				
Data analysis methods reported?	Yes	The LVI uses a balanced weighted average approach (Sullivan				

		et al., 2002) where each sub-component contributes equally to the overall index even though each major component is comprised of a different number of sub-components. Becausewe intended to develop an assessment tool accessible to a diverse set of users in resource-poor settings, the LVI formula uses the simple approach of applying equal weights to all major components. This weighting scheme could be adjusted by future users as needed. Because each of the sub-components is measured on a different<br>scale, it was first necessary to standardize each as an index. The equation used for this conversion was adapted from that used in the Human Development Index to calculate the life expectancy index, which is the ratio of the difference of the actual life expectancy and a pre-selected minimum, and the range of pre- determined maximum and minimum life expectancy (UNDP, 2007):<br>$index_{sd} = (s_d - s_{min})/(s_{max} - s_{min})$<br>where sd is the original sub-component for district d, and smin and smax are the minimum and maximum values, respectively, for each sub-component determined using data from both districts.				
	Yes	Table 4 shows the weight assigned to each variable derived from the first principal<br>component. The data reduction that PCA performed on the data explained 25 per cent of the original variation of the data. The PCA factor loadings were examined, and the principal component tended to load<br>positively on variables which contributed to lower vulnerability such as better education and better health, and negatively on variables which contributed to higher household vulnerability such as higher percentage of household income from agriculture. Therefore, the score is a measure of ‘strength’ or preparedness. A high score indicates a non-vulnerable household, and a low score indicates a vulnerable household.				

<table>
<tr><td colspan="3">Structured summary of operationalization – validity assessment
Construct: average precipitation
Article: Hahn et al (2009)</td><td rowspan="2">1.1 DCM Appropriate</td><td rowspan="2">1.2 valid empirical rep?</td><td rowspan="2">1. conclusion - Valid?</td><td rowspan="2">2. Feasible?</td></tr>
<tr><td><u>Criterion</u></td><td><u>Assessment</u></td><td><u>Quoted text or Rationale for negative assessment</u></td></tr>
<tr><td>Construct defined?</td><td>Yes</td><td>Standard deviation of the average monthly precipitation between 1998 and 2003 was averaged for each province</td><td>Yes/ no/ can't tell</td><td>Yes/ no/ can't tell</td><td rowspan="6">Yes/ no/ can't tell

YES, BUT ONLY FOR AREAS NEAR PROVINCIAL CAPITAL. GEOGRAPHIC VARIABILITY UNCLEAR</td><td rowspan="6">Yes/ no/ can't tell</td></tr>
<tr><td>Data collection methods reported?</td><td>2ndary data</td><td>provincial data; weather station based in the provincial capital</td><td>CAN'T TELL</td><td></td></tr>
<tr><td>Reporting of indicators/questions used to operationalise construct?</td><td>2ndary data</td><td><table><tr><td colspan="3">Table 1 Major components and sub-components comprising the Livelihood Vulnerability Index (LVI) developed for two districts of Mozambique.</td></tr><tr><td>Sub-components</td><td>Explanation of sub-components</td><td>Survey question</td></tr><tr><td>Mean standard deviation of average precipitation by month</td><td>Standard deviation of the average monthly precipitation between 1998 and 2003 was averaged for each province</td><td>1998–2003: provincial data; weather station based in the provincial capital</td></tr></table></td><td></td><td>YES</td></tr>
<tr><td>Sampling strategies reported?</td><td>2ndary data</td><td></td><td></td><td></td></tr>
<tr><td>Sampling sizes reported?</td><td>2ndary data</td><td></td><td></td><td></td></tr>
<tr><td>Data analysis methods reported?</td><td>Yes</td><td>The LVI uses a balanced weighted average approach (Sullivan
et al., 2002) where each sub-component contributes equally to the overall index even though each major component is comprised of a different number of sub-components. Becausewe intended to develop an assessment tool accessible to a diverse set of users in</td><td></td><td></td></tr>
</table>

		resource-poor settings, the LVI formula uses the simple approach of applying equal weights to all major components. This weighting scheme could be adjusted by future users as needed. Because each of the sub-components is measured on a different scale, it was first necessary to standardize each as an index. The equation used for this conversion was adapted from that used in the Human Development Index to calculate the life expectancy index, which is the ratio of the difference of the actual life expectancy and a pre-selected minimum, and the range of pre- determined maximum and minimum life expectancy (UNDP, 2007): $index_{sd} = (s_d - s_{min})/(s_{max} - s_{min})$ where sd is the original sub-component for district d, and smin and smax are the minimum and maximum values, respectively, for each sub-component determined using data from both districts.				
	Yes	Table 4 shows the weight assigned to each variable derived from the first principal component. The data reduction that PCA performed on the data explained 25 per cent of the original variation of the data. The PCA factor loadings were examined, and the principal component tended to load positively on variables which contributed to lower vulnerability such as better education and better health, and negatively on variables which contributed to higher household vulnerability such as higher percentage of household income from agriculture. Therefore, the score is a measure of 'strength' or preparedness. A high score indicates a non-vulnerable household, and a low score indicates a vulnerable household.				

**Structured summary of operationalization – validity assessment**	**1.1 DCM Appropriate**	**1.2 valid empirical rep?**	**1. conclusion - Valid?**	**2. Feasible?**
**Construct:** borrow-lend ratio				
**Article:** Hahn et al (2009)				

<table>
<tr><td><u>Criterion</u></td><td><u>Assessment</u></td><td><u>Quoted text or Rationale for negative assessment</u></td><td></td><td></td><td></td><td></td></tr>
<tr><td>Construct defined?</td><td>Yes</td><td>Ratio of a household borrowing money in the past month to a household lending money in the past month, e.g., If a household borrowed money but did not lend money, the ratio = 2:1 or 2 and if they lent money but did not borrow any, the ratio = 1:2 or 0.5.</td><td>Yes/ no/ can’t tell</td><td>Yes/ no/ can’t tell</td><td rowspan="3">Yes/ no/ can’t tell
YES</td><td rowspan="3">Yes/ no/ can’t tell
YES</td></tr>
<tr><td>Data collection methods reported?</td><td>Yes</td><td>household surveys</td><td>YES</td><td></td></tr>
<tr><td>Reporting of indicators/questions used to operationalise construct?</td><td>Yes</td><td>Table 1 includes an explanation of how each sub-component was quantified, the survey question used to collect the data, the original source of the survey question, and potential sources of bias.
[...]

<table>
<tr><td colspan="3">Table 1 Major components and sub-components comprising the Livelihood Vulnerability Index (LVI) developed for two districts of Mozambique.</td></tr>
<tr><td>Sub-components</td><td>Explanation of sub-components</td><td>Survey question</td></tr>
<tr><td>Average Borrow:Lend Money ratio (range: 0.5–2)</td><td>Ratio of a household borrowing money in the past month to a household lending money in the past month, e.g., If a household borrowed money but did not lend money, the ratio = 2:1 or 2 and if they lent money but did not borrow any, the ratio = 1:2 or 0.5.</td><td>Did you borrow any money from relatives or friends in the past month? Did you lend any money to relatives or friends in the past month?</td></tr>
</table></td><td></td><td>YES</td></tr>
</table>

Sampling strategies reported?	Yes	We pilot tested the LVI and LVI–IPCC in the Moma and Mabote<br>Districts of Mozambique during 2007. These were selected by CARE-Mozambique as representative of coastal and inland communities, respectively, and the climate change issues con- fronting each.<br>[...]<br>Based on a sample size calculation (WHO, 2005) at the 95% confidence interval, □10% precision, 50% prevalence,1 and a design effect of 2 to account for cluster sampling, 200 households in each<br>district were surveyed.2 National 1997 census data that specified the total population in each village was used to select 20 villages in each district using the probability proportional to size method (WHO, 2005; UNICEF, 2008).<br>[...]<br>1 50% prevalence refers to the point prevalence of the indicators selected for the LVI. This is the default value for sample size calculations when the prevalence of the indicators is unknown.<br>2 Sample size formula: N = DEFF*[(Z2*p*q)/e2], where N = sample size, DEFF = 2; Z = 1.96 (95% CI), p = 0.5; q = 0.5; e = 0.10.				
Sampling sizes reported?	Yes	We pilot tested the LVI and LVI–IPCC in the Moma and Mabote<br>Districts<br>[...]<br>200 households in each<br>district were surveyed.2 National 1997 census data that specified the total population in each village was used to select 20 villages in each district				
Data analysis methods reported?	Yes	The LVI uses a balanced weighted average approach (Sullivan<br>et al., 2002) where each sub-component contributes equally to the overall index even though each major component is comprised of a different number of sub-components. Becausewe intended to develop an assessment tool accessible to a diverse set of users in				

		resource-poor settings, the LVI formula uses the simple approach of applying equal weights to all major components. This weighting scheme could be adjusted by future users as needed. Because each of the sub-components is measured on a different scale, it was first necessary to standardize each as an index. The equation used for this conversion was adapted from that used in the Human Development Index to calculate the life expectancy index, which is the ratio of the difference of the actual life expectancy and a pre-selected minimum, and the range of pre- determined maximum and minimum life expectancy (UNDP, 2007): $index_{sd} = (s_d - s_{min})/(s_{max} - s_{min})$ where sd is the original sub-component for district d, and smin and smax are the minimum and maximum values, respectively, for each sub-component determined using data from both districts.				
	Yes	Table 4 shows the weight assigned to each variable derived from the first principal component. The data reduction that PCA performed on the data explained 25 per cent of the original variation of the data. The PCA factor loadings were examined, and the principal component tended to load positively on variables which contributed to lower vulnerability such as better education and better health, and negatively on variables which contributed to higher household vulnerability such as higher percentage of household income from agriculture. Therefore, the score is a measure of 'strength' or preparedness. A high score indicates a non-vulnerable household, and a low score indicates a vulnerable household.				

**Structured summary of operationalization – validity assessment**	**1.1 DCM Appropriate**	**1.2 valid empirical rep?**	**1. conclusion - Valid?**	**2. Feasible?**
**Construct:** crop diversity				
**Article:** Hahn et al (2009)				

<table>
<tr><td><u>Criterion</u></td><td><u>Assessment</u></td><td><u>Quoted text or Rationale for negative assessment</u></td><td></td><td></td><td></td><td></td></tr>
<tr><td>Construct defined?</td><td>Yes</td><td>The inverse of (the number of crops grown by a household +1). e.g., A household that grows pumpkin, maize, nhemba beans, and cassava will have a Crop Diversity Index = 1/(4 + 1) = 0.20.</td><td>Yes/ no/ can’t tell</td><td>Yes/ no/ can’t tell</td><td rowspan="4">Yes/ no/ can’t tell
YES</td><td rowspan="4">Yes/ no/ can’t tell
YES</td></tr>
<tr><td>Data collection methods reported?</td><td>Yes</td><td>household surveys</td><td>YES</td><td></td></tr>
<tr><td>Reporting of indicators/questions used to operationalise construct?</td><td>Yes</td><td>Table 1 includes an explanation of how each sub-component was quantified, the survey question used to collect the data, the original source of the survey question, and potential sources of bias.
[...]
<table><tr><td colspan="3">Table 1 Major components and sub-components comprising the Livelihood Vulnerability Index (LVI) developed for two districts of Mozambique.</td></tr><tr><td>Sub-components</td><td>Explanation of sub-components</td><td>Survey question</td></tr><tr><td>Average Crop Diversity Index (range: >0–1)a</td><td>The inverse of (the number of crops grown by a household +1). e.g., A household that grows pumpkin, maize, nhemba beans, and cassava will have a Crop Diversity</td><td>What kind of crops does your household grow?</td></tr></table></td><td></td><td>YES</td></tr>
<tr><td>Sampling strategies reported?</td><td>Yes</td><td>We pilot tested the LVI and LVI–IPCC in the Moma and Mabote
Districts of Mozambique during 2007. These were selected by CARE-Mozambique as representative of coastal and inland communities, respectively, and the climate change issues con- fronting each.
[...]
Based on a sample size calculation (WHO, 2005) at the</td><td></td><td></td></tr>
</table>

		95% confidence interval, [illegible]10% precision, 50% prevalence,1 and a design effect of 2 to account for cluster sampling, 200 households in each district were surveyed.2 National 1997 census data that specified the total population in each village was used to select 20 villages in each district using the probability proportional to size method (WHO, 2005; UNICEF, 2008).<br>[...]<br>1 50% prevalence refers to the point prevalence of the indicators selected for the LVI. This is the default value for sample size calculations when the prevalence of the indicators is unknown.<br>2 Sample size formula: N = DEFF*[(Z2*p*q)/e2], where N = sample size, DEFF = 2; Z = 1.96 (95% CI), p = 0.5; q = 0.5; e = 0.10.				
Sampling sizes reported?	Yes	We pilot tested the LVI and LVI–IPCC in the Moma and Mabote<br>Districts<br>[...]<br>200 households in each district were surveyed.2 National 1997 census data that specified the total population in each village was used to select 20 villages in each district				
Data analysis methods reported?	Yes	The LVI uses a balanced weighted average approach (Sullivan et al., 2002) where each sub-component contributes equally to the overall index even though each major component is comprised of a different number of sub-components. Becausewe intended to develop an assessment tool accessible to a diverse set of users in resource-poor settings, the LVI formula uses the simple approach of applying equal weights to all major components. This weighting scheme could be adjusted by future users as needed. Because each of the sub-components is measured on a different scale, it was first necessary to standardize each as an index. The equation used for this conversion was adapted from that used in the Human Development Index to				

		calculate the life expectancy index, which is the ratio of the difference of the actual life expectancy and a pre-selected minimum, and the range of pre- determined maximum and minimum life expectancy (UNDP, 2007): $index_{sd} = (s_d – s_{min})/(s_{max} – s_{min})$ where sd is the original sub-component for district d, and smin and smax are the minimum and maximum values, respectively, for each sub-component determined using data from both districts.				
	Yes	Table 4 shows the weight assigned to each variable derived from the first principal component. The data reduction that PCA performed on the data explained 25 per cent of the original variation of the data. The PCA factor loadings were examined, and the principal component tended to load positively on variables which contributed to lower vulnerability such as better education and better health, and negatively on variables which contributed to higher household vulnerability such as higher percentage of household income from agriculture. Therefore, the score is a measure of 'strength' or preparedness. A high score indicates a non-vulnerable household, and a low score indicates a vulnerable household.				

**Structured summary of operationalization – validity assessment**			**1.1 DCM Appropriate**	**1.2 valid empirical rep?**	**1. conclusion - Valid?**	**2. Feasible?**
**Construct:** dependency ratio						
**Article:** Hahn et al (2009)						
Criterion	Assessment	Quoted text or Rationale for negative assessment				
Construct defined?	Yes	Ratio of the population under 15 and over 65 years of age to the population between 19 and 64 years of age.	Yes/ no/ can't tell	Yes/ no/ can't tell	Yes/ no/ can't tell YES	Yes/ no/ can't tell CAN'T TELL
Data collection methods reported?	Yes	household surveys	YES			
Reporting of indicators/questions used to	Yes	Table 1 includes an explanation of how each sub-component was quantified, the survey question used to collect the data, the original source of the survey		YES		

<table>
<tr>
<td>operationalise construct?</td>
<td></td>
<td>question, and potential sources of bias.
[...]

<table>
<tr><td colspan="3">Table 1 Major components and sub-components comprising the Livelihood Vulnerability Index (LVI) developed for two districts of Mozambique.</td></tr>
<tr><td>Sub-components</td><td>Explanation of sub-components</td><td>Survey question</td></tr>
<tr><td>Dependency ratio</td><td>Ratio of the population under 15 and over 65 years of age to the population between 19 and 64 years of age.</td><td>Could you please list the ages and sexes of every person who eats and sleeps in this house? If you had a visitor who ate and slept here for the last 3 days, please include them as well.</td></tr>
</table>
</td>
<td></td>
<td></td>
<td></td>
<td></td>
</tr>
<tr>
<td>Sampling strategies reported?</td>
<td>Yes</td>
<td>We pilot tested the LVI and LVI–IPCC in the Moma and Mabote
Districts of Mozambique during 2007. These were selected by CARE-Mozambique as representative of coastal and inland communities, respectively, and the climate change issues con- fronting each.
[...]
Based on a sample size calculation (WHO, 2005) at the 95% confidence interval, [illegible]10% precision, 50% prevalence,1 and a design effect of 2 to account for cluster sampling, 200 households in each
district were surveyed.2 National 1997 census data that specified the total population in each village was used to select 20 villages in each district using the probability proportional to size method (WHO, 2005; UNICEF, 2008).
[...]
1 50% prevalence refers to the point prevalence of the</td>
<td></td>
<td></td>
<td></td>
<td></td>
</tr>
</table>

		indicators selected for the LVI. This is the default value for sample size calculations when the prevalence of the indicators is unknown.<br>2 Sample size formula: N = DEFF*[(Z2*p*q)/e2], where N = sample size, DEFF = 2; Z = 1.96 (95% CI), p = 0.5; q = 0.5; e = 0.10.				
Sampling sizes reported?	Yes	We pilot tested the LVI and LVI–IPCC in the Moma and Mabote<br>Districts<br>[...]<br>200 households in each<br>district were surveyed.2 National 1997 census data that specified the total population in each village was used to select 20 villages in each district				
Data analysis methods reported?	Yes	The LVI uses a balanced weighted average approach (Sullivan<br>et al., 2002) where each sub-component contributes equally to the overall index even though each major component is comprised of a different number of sub-components. Becausewe intended to develop an assessment tool accessible to a diverse set of users in resource-poor settings, the LVI formula uses the simple approach of applying equal weights to all major components. This weighting scheme could be adjusted by future users as needed. Because each of the sub-components is measured on a different<br>scale, it was first necessary to standardize each as an index. The equation used for this conversion was adapted from that used in the Human Development Index to calculate the life expectancy index, which is the ratio of the difference of the actual life expectancy and a pre-selected minimum, and the range of pre- determined maximum and minimum life expectancy (UNDP, 2007):<br>$index_{sd} = (s_d - s_{min})/(s_{max} - s_{min})$<br>where sd is the original sub-component for district d, and smin and smax are the minimum and maximum values, respectively, for each sub-component determined using data from both districts.				

	Yes	Table 4 shows the weight assigned to each variable derived from the first principal component. The data reduction that PCA performed on the data explained 25 per cent of the original variation of the data. The PCA factor loadings were examined, and the principal component tended to load positively on variables which contributed to lower vulnerability such as better education and better health, and negatively on variables which contributed to higher household vulnerability such as higher percentage of household income from agriculture. Therefore, the score is a measure of 'strength' or preparedness. A high score indicates a non-vulnerable household, and a low score indicates a vulnerable household.				

**Structured summary of operationalization – validity assessment**			**1.1 DCM Appropriate**	**1.2 valid empirical rep?**	**1. conclusion - Valid?**	**2. Feasible?**
**Construct:** don't save crops						
**Article:** Hahn et al (2009)						
<u>Criterion</u>	<u>Assessment</u>	<u>Quoted text or Rationale for negative assessment</u>				
Construct defined?	Yes	Percentage of households that do not save crops from each harvest.	Yes/ no/ can't tell	Yes/ no/ can't tell	Yes/ no/ can't tell	Yes/ no/ can't tell
Data collection methods reported?	Yes	household surveys	YES		YES	CAN'T TELL; RELEVANCE DEPENDS ON PRODUCTION SYSTEM, THUS NOT A
Reporting of indicators/questions used to operationalise construct?	Yes	Table 1 includes an explanation of how each sub-component was quantified, the survey question used to collect the data, the original source of the survey question, and potential sources of bias.<br>[...]<br><br>Table 1 Major components and sub-components comprising the Livelihood Vulnerability Index (LVI) developed for two districts of Mozambique.<br>*Sub-components* | *Explanation of sub-components* | *Survey question*<br>Percent of | Percentage of | Does your		YES		

		households that do not save crops | households that do not save crops from each harvest. | family save some of the crops you harvest to eat during a different time of year?				
Sampling strategies reported?	Yes	We pilot tested the LVI and LVI–IPCC in the Moma and Mabote<br>Districts of Mozambique during 2007. These were selected by CARE-Mozambique as representative of coastal and inland communities, respectively, and the climate change issues con- fronting each.<br>[...]<br>Based on a sample size calculation (WHO, 2005) at the 95% confidence interval, [illegible]10% precision, 50% prevalence,1 and a design effect of 2 to account for cluster sampling, 200 households in each<br>district were surveyed.2 National 1997 census data that specified the total population in each village was used to select 20 villages in each district using the probability proportional to size method (WHO, 2005; UNICEF, 2008).<br>[...]<br>1 50% prevalence refers to the point prevalence of the indicators selected for the LVI. This is the default value for sample size calculations when the prevalence of the indicators is unknown.<br>2 Sample size formula: N = DEFF*[(Z2*p*q)/e2], where N = sample size, DEFF = 2; Z = 1.96 (95% CI), p = 0.5; q = 0.5; e = 0.10.				
Sampling sizes reported?	Yes	We pilot tested the LVI and LVI–IPCC in the Moma and Mabote<br>Districts<br>[...]<br>200 households in each<br>district were surveyed.2 National 1997 census data that specified the total population in each village was used to select 20 villages in each district				

Data analysis methods reported?	Yes	The LVI uses a balanced weighted average approach (Sullivan et al., 2002) where each sub-component contributes equally to the overall index even though each major component is comprised of a different number of sub-components. Becausewe intended to develop an assessment tool accessible to a diverse set of users in resource-poor settings, the LVI formula uses the simple approach of applying equal weights to all major components. This weighting scheme could be adjusted by future users as needed. Because each of the sub-components is measured on a different scale, it was first necessary to standardize each as an index. The equation used for this conversion was adapted from that used in the Human Development Index to calculate the life expectancy index, which is the ratio of the difference of the actual life expectancy and a pre-selected minimum, and the range of pre- determined maximum and minimum life expectancy (UNDP, 2007): $index_{sd} = (s_d - s_{min})/(s_{max} - s_{min})$ where sd is the original sub-component for district d, and smin and smax are the minimum and maximum values, respectively, for each sub-component determined using data from both districts.				
	Yes	Table 4 shows the weight assigned to each variable derived from the first principal component. The data reduction that PCA performed on the data explained 25 per cent of the original variation of the data. The PCA factor loadings were examined, and the principal component tended to load positively on variables which contributed to lower vulnerability such as better education and better health, and negatively on variables which contributed to higher household vulnerability such as higher percentage of household income from agriculture. Therefore, the score is a measure of ‘strength’ or preparedness. A high score indicates a non-vulnerable household, and a low score indicates a vulnerable household.				

<table>
<tr><td colspan="3">Structured summary of operationalization – validity assessment</td><td rowspan="4">1.1 DCM Appropriate</td><td rowspan="4">1.2 valid empirical rep?</td><td rowspan="4">1. conclusion - Valid?</td><td rowspan="4">2. Feasible?</td></tr>
<tr><td colspan="3">Construct: don't save seeds</td></tr>
<tr><td colspan="3">Article: Hahn et al (2009)</td></tr>
<tr><td><u>Criterion</u></td><td><u>Assessment</u></td><td><u>Quoted text or Rationale for negative assessment</u></td></tr>
<tr><td>Construct defined?</td><td>Yes</td><td>Percentage of households that do not have seeds from year to year.</td><td>Yes/ no/ can’t tell</td><td>Yes/ no/ can’t tell</td><td rowspan="4">Yes/ no/ can’t tell
YES</td><td rowspan="4">Yes/ no/ can’t tell
YES</td></tr>
<tr><td>Data collection methods reported?</td><td>Yes</td><td>household surveys</td><td>YES</td><td></td></tr>
<tr><td>Reporting of indicators/questions used to operationalise construct?</td><td>Yes</td><td>Table 1 includes an explanation of how each sub-component was quantified, the survey question used to collect the data, the original source of the survey question, and potential sources of bias.
[...]
<table><tr><td colspan="3">Table 1 Major components and sub-components comprising the Livelihood Vulnerability Index (LVI) developed for two districts of Mozambique.</td></tr><tr><td>Sub-components</td><td>Explanation of sub-components</td><td>Survey question</td></tr><tr><td>Percent of households that do not save seeds</td><td>Percentage of households that do not have seeds from year to year.</td><td>Does your family save seeds to grow the next year?</td></tr></table></td><td></td><td>YES</td></tr>
<tr><td>Sampling strategies reported?</td><td>Yes</td><td>We pilot tested the LVI and LVI–IPCC in the Moma and Mabote
Districts of Mozambique during 2007. These were selected by CARE-Mozambique as representative of coastal and inland communities, respectively, and the climate change issues con- fronting each.
[...]
Based on a sample size calculation (WHO, 2005) at the 95% confidence interval, [illegible]10% precision, 50% prevalence,1 and a design effect of 2 to account for</td><td></td><td></td></tr>
</table>

		cluster sampling, 200 households in each<br>district were surveyed.2 National 1997 census data that specified the total population in each village was used to select 20 villages in each district using the probability proportional to size method (WHO, 2005; UNICEF, 2008).<br>[...]<br>1 50% prevalence refers to the point prevalence of the indicators selected for the LVI. This is the default value for sample size calculations when the prevalence of the indicators is unknown.<br>2 Sample size formula: N = DEFF*[(Z2*p*q)/e2], where N = sample size, DEFF = 2; Z = 1.96 (95% CI), p = 0.5; q = 0.5; e = 0.10.				
Sampling sizes reported?	Yes	We pilot tested the LVI and LVI–IPCC in the Moma and Mabote<br>Districts<br>[...]<br>200 households in each<br>district were surveyed.2 National 1997 census data that specified the total population in each village was used to select 20 villages in each district				
Data analysis methods reported?	Yes	The LVI uses a balanced weighted average approach (Sullivan<br>et al., 2002) where each sub-component contributes equally to the overall index even though each major component is comprised of a different number of sub-components. Becausewe intended to develop an assessment tool accessible to a diverse set of users in resource-poor settings, the LVI formula uses the simple approach of applying equal weights to all major components. This weighting scheme could be adjusted by future users as needed. Because each of the sub-components is measured on a different<br>scale, it was first necessary to standardize each as an index. The equation used for this conversion was adapted from that used in the Human Development Index to calculate the life expectancy index, which is the ratio of the difference of the actual life expectancy and a pre-				

		selected minimum, and the range of pre- determined maximum and minimum life expectancy (UNDP, 2007): $index_{sd} = (s_d – s_{min})/(s_{max} – s_{min})$ where sd is the original sub-component for district d, and smin and smax are the minimum and maximum values, respectively, for each sub-component determined using data from both districts.				
	Yes	Table 4 shows the weight assigned to each variable derived from the first principal component. The data reduction that PCA performed on the data explained 25 per cent of the original variation of the data. The PCA factor loadings were examined, and the principal component tended to load positively on variables which contributed to lower vulnerability such as better education and better health, and negatively on variables which contributed to higher household vulnerability such as higher percentage of household income from agriculture. Therefore, the score is a measure of 'strength' or preparedness. A high score indicates a non-vulnerable household, and a low score indicates a vulnerable household.				

**Structured summary of operationalization – validity assessment**			**1.1 DCM Appropriate**	**1.2 valid empirical rep?**	**1. conclusion - Valid?**	**2. Feasible?**
**Construct:** family with cronic illness						
**Article:** Hahn et al (2009)						
Criterion	Assessment	Quoted text or Rationale for negative assessment				
Construct defined?	Yes	Percentage of households that report at least 1 family member with chronic illness. Chronic illness was defined subjectively by respondent.	Yes/ no/ can't tell	Yes/ no/ can't tell	Yes/ no/ can't tell YES	Yes/ no/ can't tell YES
Data collection methods reported?	Yes	household surveys	YES			
Reporting of indicators/questions used to operationalise	Yes	Table 1 includes an explanation of how each sub-component was quantified, the survey question used to collect the data, the original source of the survey question, and potential sources of bias.		YES		

<table>
<tr><td>construct?</td><td></td><td>[...]
<table><tr><td colspan="3">Table 1 Major components and sub-components comprising the Livelihood Vulnerability Index (LVI) developed for two districts of Mozambique.</td></tr><tr><td>Sub-components</td><td>Explanation of sub-components</td><td>Survey question</td></tr><tr><td>Percent of households with family member with chronic illness</td><td>Percentage of households that report at least 1 family member with chronic illness. Chronic illness was defined subjectively by respondent.</td><td>Is anybody in your family chronically ill (they get sick very often)?</td></tr></table></td><td></td><td></td><td></td><td></td></tr>
<tr><td>Sampling strategies reported?</td><td>Yes</td><td>We pilot tested the LVI and LVI–IPCC in the Moma and Mabote
Districts of Mozambique during 2007. These were selected by CARE-Mozambique as representative of coastal and inland communities, respectively, and the climate change issues con- fronting each.
[...]
Based on a sample size calculation (WHO, 2005) at the 95% confidence interval, [illegible]10% precision, 50% prevalence,1 and a design effect of 2 to account for cluster sampling, 200 households in each
district were surveyed.2 National 1997 census data that specified the total population in each village was used to select 20 villages in each district using the probability proportional to size method (WHO, 2005; UNICEF, 2008).
[...]
1 50% prevalence refers to the point prevalence of the indicators selected for the LVI. This is the default value for sample size calculations when the prevalence of the indicators is unknown.
2 Sample size formula: N = DEFF*[(Z2*p*q)/e2], where N</td><td></td><td></td><td></td><td></td></tr>
</table>

		= sample size, DEFF = 2; Z = 1.96 (95% CI), p = 0.5; q = 0.5; e = 0.10.				
Sampling sizes reported?	Yes	We pilot tested the LVI and LVI–IPCC in the Moma and Mabote<br>Districts<br>[...]<br>200 households in each<br>district were surveyed.2 National 1997 census data that specified the total population in each village was used to select 20 villages in each district				
Data analysis methods reported?	Yes	The LVI uses a balanced weighted average approach (Sullivan<br>et al., 2002) where each sub-component contributes equally to the overall index even though each major component is comprised of a different number of sub-components. Becausewe intended to develop an assessment tool accessible to a diverse set of users in resource-poor settings, the LVI formula uses the simple approach of applying equal weights to all major components. This weighting scheme could be adjusted by future users as needed. Because each of the sub-components is measured on a different<br>scale, it was first necessary to standardize each as an index. The equation used for this conversion was adapted from that used in the Human Development Index to calculate the life expectancy index, which is the ratio of the difference of the actual life expectancy and a pre-selected minimum, and the range of pre- determined maximum and minimum life expectancy (UNDP, 2007):<br>$index_{sd} = (s_d - s_{min})/(s_{max} - s_{min})$<br>where sd is the original sub-component for district d, and smin and smax are the minimum and maximum values, respectively, for each sub-component determined using data from both districts.				
	Yes	Table 4 shows the weight assigned to each variable derived from the first principal<br>component. The data reduction that PCA performed on the data explained 25 per cent of the original variation of				

		the data. The PCA factor loadings were examined, and the principal component tended to load<br>positively on variables which contributed to lower vulnerability such as better education and better health, and negatively on variables which contributed to higher household vulnerability such as higher percentage of household income from agriculture. Therefore, the score is a measure of 'strength' or preparedness. A high score indicates a non-vulnerable household, and a low score indicates a vulnerable household.				

**Structured summary of operationalization – validity assessment**			**1.1 DCM Appropriate**	**1.2 valid empirical rep?**	**1. conclusion - Valid?**	**2. Feasible?**
**Construct:** flood, drought, cyclone events						
**Article:** Hahn et al (2009)						
Criterion	Assessment	Quoted text or Rationale for negative assessment				
Construct defined?	Yes	Total number of floods, droughts, and cyclones that were reported by households in the past 6 years.	Yes/ no/ can't tell	Yes/ no/ can't tell	Yes/ no/ can't tell<br>YES	Yes/ no/ can't tell<br>YES
Data collection methods reported?	Yes	household surveys	YES			
Reporting of indicators/questions used to operationalise construct?	Yes	Table 1 includes an explanation of how each sub-component was quantified, the survey question used to collect the data, the original source of the survey question, and potential sources of bias.<br>[...]		YES		

Table 1 Major components and sub-components comprising the Livelihood Vulnerability Index (LVI) developed for two districts of Mozambique.

Sub-components	*Explanation of sub-components*	*Survey question*
Average number of flood, drought, and	Total number of floods, droughts, and cyclones	How many times has this area been affected by a flood/cyclone/drought

		cyclone events in the past 6 years (range: 0–7)<br>that were reported by households in the past 6 years.<br>in 2001–2007?				
Sampling strategies reported?	Yes	We pilot tested the LVI and LVI–IPCC in the Moma and Mabote<br>Districts of Mozambique during 2007. These were selected by CARE-Mozambique as representative of coastal and inland communities, respectively, and the climate change issues con- fronting each.<br>[...]<br>Based on a sample size calculation (WHO, 2005) at the 95% confidence interval, ⍰10% precision, 50% prevalence,1 and a design effect of 2 to account for cluster sampling, 200 households in each<br>district were surveyed.2 National 1997 census data that specified the total population in each village was used to select 20 villages in each district using the probability proportional to size method (WHO, 2005; UNICEF, 2008).<br>[...]<br>1 50% prevalence refers to the point prevalence of the indicators selected for the LVI. This is the default value for sample size calculations when the prevalence of the indicators is unknown.<br>2 Sample size formula: N = DEFF*[(Z2*p*q)/e2], where N = sample size, DEFF = 2; Z = 1.96 (95% CI), p = 0.5; q = 0.5; e = 0.10.				
Sampling sizes reported?	Yes	We pilot tested the LVI and LVI–IPCC in the Moma and Mabote<br>Districts<br>[...]<br>200 households in each<br>district were surveyed.2 National 1997 census data that specified the total population in each village was used to select 20 villages in each district				
Data analysis methods reported?	Yes	The LVI uses a balanced weighted average approach (Sullivan				

		et al., 2002) where each sub-component contributes equally to the overall index even though each major component is comprised of a different number of sub-components. Becausewe intended to develop an assessment tool accessible to a diverse set of users in resource-poor settings, the LVI formula uses the simple approach of applying equal weights to all major components. This weighting scheme could be adjusted by future users as needed. Because each of the sub-components is measured on a different scale, it was first necessary to standardize each as an index. The equation used for this conversion was adapted from that used in the Human Development Index to calculate the life expectancy index, which is the ratio of the difference of the actual life expectancy and a pre-selected minimum, and the range of pre- determined maximum and minimum life expectancy (UNDP, 2007): $index_{sd} = (s_d - s_{min})/(s_{max} - s_{min})$ where sd is the original sub-component for district d, and smin and smax are the minimum and maximum values, respectively, for each sub-component determined using data from both districts.				
	Yes	Table 4 shows the weight assigned to each variable derived from the first principal component. The data reduction that PCA performed on the data explained 25 per cent of the original variation of the data. The PCA factor loadings were examined, and the principal component tended to load positively on variables which contributed to lower vulnerability such as better education and better health, and negatively on variables which contributed to higher household vulnerability such as higher percentage of household income from agriculture. Therefore, the score is a measure of 'strength' or preparedness. A high score indicates a non-vulnerable household, and a low score indicates a vulnerable household.				

<table>
<tr><th colspan="3">Structured summary of operationalization – validity assessment</th><th rowspan="4">1.1 DCM Appropriate</th><th rowspan="4">1.2 valid empirical rep?</th><th rowspan="4">1. conclusion - Valid?</th><th rowspan="4">2. Feasible?</th></tr>
<tr><td colspan="3">Construct: food from family farm</td></tr>
<tr><td colspan="3">Article: Hahn et al (2009)</td></tr>
<tr><td><u>Criterion</u></td><td><u>Assessment</u></td><td><u>Quoted text or Rationale for negative assessment</u></td></tr>
<tr><td>Construct defined?</td><td>Yes</td><td>Percentage of households that get their food primarily from their personal farms</td><td>Yes/ no/ can’t tell</td><td>Yes/ no/ can’t tell</td><td rowspan="4">Yes/ no/ can’t tell
YES</td><td rowspan="4">Yes/ no/ can’t tell
YES</td></tr>
<tr><td>Data collection methods reported?</td><td>Yes</td><td>household surveys</td><td>YES</td><td></td></tr>
<tr><td>Reporting of indicators/questions used to operationalise construct?</td><td>Yes</td><td>Table 1 includes an explanation of how each sub-component was quantified, the survey question used to collect the data, the original source of the survey question, and potential sources of bias.
[...]

Table 1 Major components and sub-components comprising the Livelihood Vulnerability Index (LVI) developed for two districts of Mozambique.
<table><tr><th>Sub-components</th><th>Explanation of sub-components</th><th>Survey question</th></tr><tr><td>Percent of households dependent on family farm for food</td><td>Percentage of households that get their food primarily from their personal farms.</td><td>Where does your family get most of its food?</td></tr></table></td><td></td><td>YES</td></tr>
<tr><td>Sampling strategies reported?</td><td>Yes</td><td>We pilot tested the LVI and LVI–IPCC in the Moma and Mabote
Districts of Mozambique during 2007. These were selected by CARE-Mozambique as representative of coastal and inland communities, respectively, and the climate change issues con- fronting each.
[...]
Based on a sample size calculation (WHO, 2005) at the 95% confidence interval, [illegible]10% precision, 50% prevalence,1 and a design effect of 2 to account for cluster sampling, 200 households in each</td><td></td><td></td></tr>
</table>

		district were surveyed.2 National 1997 census data that specified the total population in each village was used to select 20 villages in each district using the probability proportional to size method (WHO, 2005; UNICEF, 2008).<br>[...]<br>1 50% prevalence refers to the point prevalence of the indicators selected for the LVI. This is the default value for sample size calculations when the prevalence of the indicators is unknown.<br>2 Sample size formula: N = DEFF*[(Z2*p*q)/e2], where N = sample size, DEFF = 2; Z = 1.96 (95% CI), p = 0.5; q = 0.5; e = 0.10.				
Sampling sizes reported?	Yes	We pilot tested the LVI and LVI–IPCC in the Moma and Mabote<br>Districts<br>[...]<br>200 households in each<br>district were surveyed.2 National 1997 census data that specified the total population in each village was used to select 20 villages in each district				
Data analysis methods reported?	Yes	The LVI uses a balanced weighted average approach (Sullivan<br>et al., 2002) where each sub-component contributes equally to the overall index even though each major component is comprised of a different number of sub-components. Becausewe intended to develop an assessment tool accessible to a diverse set of users in resource-poor settings, the LVI formula uses the simple approach of applying equal weights to all major components. This weighting scheme could be adjusted by future users as needed. Because each of the sub-components is measured on a different<br>scale, it was first necessary to standardize each as an index. The equation used for this conversion was adapted from that used in the Human Development Index to calculate the life expectancy index, which is the ratio of the difference of the actual life expectancy and a pre-selected minimum, and the range of pre- determined				

		maximum and minimum life expectancy (UNDP, 2007):<br>$index_{sd} = (s_d - s_{min})/(s_{max} - s_{min})$<br>where sd is the original sub-component for district d, and smin and smax are the minimum and maximum values, respectively, for each sub-component determined using data from both districts.				
	Yes	Table 4 shows the weight assigned to each variable derived from the first principal<br>component. The data reduction that PCA performed on the data explained 25 per cent of the original variation of the data. The PCA factor loadings were examined, and the principal component tended to load<br>positively on variables which contributed to lower vulnerability such as better education and better health, and negatively on variables which contributed to higher household vulnerability such as higher percentage of household income from agriculture. Therefore, the score is a measure of 'strength' or preparedness. A high score indicates a non-vulnerable household, and a low score indicates a vulnerable household.				

**Structured summary of operationalization – validity assessment**			**1.1 DCM Appropriate**	**1.2 valid empirical rep?**	**1. conclusion - Valid?**	**2. Feasible?**
**Construct:** households with orphans						
**Article:** Hahn et al (2009)						
Criterion	Assessment	Quoted text or Rationale for negative assessment				
Construct defined?	Yes	Percentage of households that have at least 1 orphan living in their home. Orphans are children<18 years old who have lost one or both parents.	Yes/ no/ can't tell	Yes/ no/ can't tell	Yes/ no/ can't tell<br>YES	Yes/ no/ can't tell<br>YES
Data collection methods reported?	Yes	household surveys	YES			
Reporting of indicators/questions used to operationalise construct?	Yes	Table 1 includes an explanation of how each sub-component was quantified, the survey question used to collect the data, the original source of the survey question, and potential sources of bias.<br>[...]		YES		

<table>
<tr>
<td></td>
<td></td>
<td>
<table>
<tr><td colspan="3">Table 1 Major components and sub-components comprising the Livelihood Vulnerability Index (LVI) developed for two districts of Mozambique.</td></tr>
<tr><td>Sub-components</td><td>Explanation of sub-components</td><td>Survey question</td></tr>
<tr><td>Percent of households with orphans</td><td>Percentage of households that have at least 1 orphan living in their home. Orphans are children<18 years old who have lost one or both parents.</td><td>Are there any children less than 18 years old from other families living in your house because one or both of their parents has died?</td></tr>
</table>
</td>
<td></td>
<td></td>
<td></td>
<td></td>
</tr>
<tr>
<td>Sampling strategies reported?</td>
<td>Yes</td>
<td>We pilot tested the LVI and LVI–IPCC in the Moma and Mabote
Districts of Mozambique during 2007. These were selected by CARE-Mozambique as representative of coastal and inland communities, respectively, and the climate change issues con- fronting each.
[...]
Based on a sample size calculation (WHO, 2005) at the 95% confidence interval, [illegible]10% precision, 50% prevalence,1 and a design effect of 2 to account for cluster sampling, 200 households in each
district were surveyed.2 National 1997 census data that specified the total population in each village was used to select 20 villages in each district using the probability proportional to size method (WHO, 2005; UNICEF, 2008).
[...]
1 50% prevalence refers to the point prevalence of the indicators selected for the LVI. This is the default value for sample size calculations when the prevalence of the indicators is unknown.
2 Sample size formula: N = DEFF*[(Z2*p*q)/e2], where N = sample size, DEFF = 2; Z = 1.96 (95% CI), p = 0.5; q = 0.5;</td>
<td></td>
<td></td>
<td></td>
<td></td>
</tr>
</table>

		e = 0.10.				
Sampling sizes reported?	Yes	We pilot tested the LVI and LVI–IPCC in the Moma and Mabote<br>Districts<br>[...]<br>200 households in each<br>district were surveyed.2 National 1997 census data that specified the total population in each village was used to select 20 villages in each district				
Data analysis methods reported?	Yes	The LVI uses a balanced weighted average approach (Sullivan<br>et al., 2002) where each sub-component contributes equally to the overall index even though each major component is comprised of a different number of sub-components. Becausewe intended to develop an assessment tool accessible to a diverse set of users in resource-poor settings, the LVI formula uses the simple approach of applying equal weights to all major components. This weighting scheme could be adjusted by future users as needed. Because each of the sub-components is measured on a different<br>scale, it was first necessary to standardize each as an index. The equation used for this conversion was adapted from that used in the Human Development Index to calculate the life expectancy index, which is the ratio of the difference of the actual life expectancy and a pre-selected minimum, and the range of pre- determined maximum and minimum life expectancy (UNDP, 2007):<br>$index_{sd} = (s_d – s_{min})/(s_{max} – s_{min})$<br>where sd is the original sub-component for district d, and smin and smax are the minimum and maximum values, respectively, for each sub-component determined using data from both districts.				
	Yes	Table 4 shows the weight assigned to each variable derived from the first principal<br>component. The data reduction that PCA performed on the data explained 25 per cent of the original variation of the data. The PCA factor loadings were examined, and the				

		principal component tended to load positively on variables which contributed to lower vulnerability such as better education and better health, and negatively on variables which contributed to higher household vulnerability such as higher percentage of household income from agriculture. Therefore, the score is a measure of 'strength' or preparedness. A high score indicates a non-vulnerable household, and a low score indicates a vulnerable household.				

**Structured summary of operationalization – validity assessment**			**1.1 DCM Appropriate**	**1.2 valid empirical rep?**	**1. conclusion - Valid?**	**2. Feasible?**
**Construct:** households working elsewhere						
**Article:** Hahn et al (2009)						
Criterion	Assessment	Quoted text or Rationale for negative assessment				
Construct defined?	Yes	Percentage of households that report at least 1 family member who works outside of the community for their primary work activity.	Yes/ no/ can't tell	Yes/ no/ can't tell	Yes/ no/ can't tell YES	Yes/ no/ can't tell YES
Data collection methods reported?	Yes	household surveys	YES			
Reporting of indicators/questions used to operationalise construct?	Yes	Table 1 includes an explanation of how each sub-component was quantified, the survey question used to collect the data, the original source of the survey question, and potential sources of bias.<br>[...]<br>Table 1 Major components and sub-components comprising the Livelihood Vulnerability Index (LVI) developed for two districts of Mozambique.<br>*Sub-components* / *Explanation of sub-components* / *Survey question*<br>Percent of households with family member working in a different / Percentage of households that report at least 1 family member who works / How many people in your family go to a different community to		YES		

		community | outside of the community for their primary work activity. | work?				
Sampling strategies reported?	Yes	We pilot tested the LVI and LVI–IPCC in the Moma and Mabote<br>Districts of Mozambique during 2007. These were selected by CARE-Mozambique as representative of coastal and inland communities, respectively, and the climate change issues con- fronting each.<br>[...]<br>Based on a sample size calculation (WHO, 2005) at the 95% confidence interval, �10% precision, 50% prevalence,1 and a design effect of 2 to account for cluster sampling, 200 households in each<br>district were surveyed.2 National 1997 census data that specified the total population in each village was used to select 20 villages in each district using the probability proportional to size method (WHO, 2005; UNICEF, 2008).<br>[...]<br>1 50% prevalence refers to the point prevalence of the indicators selected for the LVI. This is the default value for sample size calculations when the prevalence of the indicators is unknown.<br>2 Sample size formula: N = DEFF*[(Z2*p*q)/e2], where N = sample size, DEFF = 2; Z = 1.96 (95% CI), p = 0.5; q = 0.5; e = 0.10.				
Sampling sizes reported?	Yes	We pilot tested the LVI and LVI–IPCC in the Moma and Mabote<br>Districts<br>[...]<br>200 households in each<br>district were surveyed.2 National 1997 census data that specified the total population in each village was used to select 20 villages in each district				
Data analysis methods reported?	Yes	The LVI uses a balanced weighted average approach (Sullivan<br>et al., 2002) where each sub-component contributes				

		equally to the overall index even though each major component is comprised of a different number of sub-components. Becausewe intended to develop an assessment tool accessible to a diverse set of users in resource-poor settings, the LVI formula uses the simple approach of applying equal weights to all major components. This weighting scheme could be adjusted by future users as needed. Because each of the sub-components is measured on a different scale, it was first necessary to standardize each as an index. The equation used for this conversion was adapted from that used in the Human Development Index to calculate the life expectancy index, which is the ratio of the difference of the actual life expectancy and a pre-selected minimum, and the range of pre- determined maximum and minimum life expectancy (UNDP, 2007): $index_{sd} = (s_d – s_{min})/(s_{max} – s_{min})$ where sd is the original sub-component for district d, and smin and smax are the minimum and maximum values, respectively, for each sub-component determined using data from both districts.				
	Yes	Table 4 shows the weight assigned to each variable derived from the first principal component. The data reduction that PCA performed on the data explained 25 per cent of the original variation of the data. The PCA factor loadings were examined, and the principal component tended to load positively on variables which contributed to lower vulnerability such as better education and better health, and negatively on variables which contributed to higher household vulnerability such as higher percentage of household income from agriculture. Therefore, the score is a measure of ‘strength’ or preparedness. A high score indicates a non-vulnerable household, and a low score indicates a vulnerable household.				

<table>
<tr><td colspan="3">Structured summary of operationalization – validity assessment</td><td rowspan="4">1.1 DCM Appropriate</td><td rowspan="4">1.2 valid empirical rep?</td><td rowspan="4">1. conclusion - Valid?</td><td rowspan="4">2. Feasible?</td></tr>
<tr><td colspan="3">Construct: idendependent of local government</td></tr>
<tr><td colspan="3">Article: Hahn et al (2009)</td></tr>
<tr><td><u>Criterion</u></td><td><u>Assessment</u></td><td><u>Quoted text or Rationale for negative assessment</u></td></tr>
<tr><td>Construct defined?</td><td>Yes</td><td>Percentage of households that reported that they have not asked their local government for any assistance in the past 12 months.</td><td>Yes/ no/ can't tell</td><td>Yes/ no/ can't tell</td><td rowspan="4">Yes/ no/ can't tell

YES</td><td rowspan="4">Yes/ no/ can't tell

YES</td></tr>
<tr><td>Data collection methods reported?</td><td>Yes</td><td>household surveys</td><td>YES</td><td></td></tr>
<tr><td>Reporting of indicators/questions used to operationalise construct?</td><td>Yes</td><td>Table 1 includes an explanation of how each sub-component was quantified, the survey question used to collect the data, the original source of the survey question, and potential sources of bias.
[...]
<table><tr><td colspan="3">Table 1 Major components and sub-components comprising the Livelihood Vulnerability Index (LVI) developed for two districts of Mozambique.</td></tr><tr><td>Sub-components</td><td>Explanation of sub-components</td><td>Survey question</td></tr><tr><td>Percent of households that have not gone to their local government for assistance in the past 12 months</td><td>Percentage of households that reported that they have not asked their local government for any assistance in the past 12 months.</td><td>In the past 12 months, have you or someone in your family gone to your community leader for help?</td></tr></table></td><td></td><td>YES</td></tr>
<tr><td>Sampling strategies reported?</td><td>Yes</td><td>We pilot tested the LVI and LVI–IPCC in the Moma and Mabote
Districts of Mozambique during 2007. These were selected by CARE-Mozambique as representative of coastal and inland communities, respectively, and the climate change issues con- fronting each.
[...]
Based on a sample size calculation (WHO, 2005) at the 95% confidence interval, ⍰10% precision, 50%</td><td></td><td></td></tr>
</table>

		prevalence,1 and a design effect of 2 to account for cluster sampling, 200 households in each<br>district were surveyed.2 National 1997 census data that specified the total population in each village was used to select 20 villages in each district using the probability proportional to size method (WHO, 2005; UNICEF, 2008).<br>[...]<br>1 50% prevalence refers to the point prevalence of the indicators selected for the LVI. This is the default value for sample size calculations when the prevalence of the indicators is unknown.<br>2 Sample size formula: N = DEFF*[(Z2*p*q)/e2], where N = sample size, DEFF = 2; Z = 1.96 (95% CI), p = 0.5; q = 0.5; e = 0.10.				
Sampling sizes reported?	Yes	We pilot tested the LVI and LVI–IPCC in the Moma and Mabote<br>Districts<br>[...]<br>200 households in each<br>district were surveyed.2 National 1997 census data that specified the total population in each village was used to select 20 villages in each district				
Data analysis methods reported?	Yes	The LVI uses a balanced weighted average approach (Sullivan<br>et al., 2002) where each sub-component contributes equally to the overall index even though each major component is comprised of a different number of sub-components. Becausewe intended to develop an assessment tool accessible to a diverse set of users in resource-poor settings, the LVI formula uses the simple approach of applying equal weights to all major components. This weighting scheme could be adjusted by future users as needed. Because each of the sub-components is measured on a different<br>scale, it was first necessary to standardize each as an index. The equation used for this conversion was adapted from that used in the Human Development Index to calculate the life expectancy index, which is the ratio of				

		the difference of the actual life expectancy and a pre-selected minimum, and the range of pre- determined maximum and minimum life expectancy (UNDP, 2007): index$_{sd}$ = (s_d – s_{min})/(s_{max} – s_{min}) where sd is the original sub-component for district d, and smin and smax are the minimum and maximum values, respectively, for each sub-component determined using data from both districts.				
	Yes	Table 4 shows the weight assigned to each variable derived from the first principal component. The data reduction that PCA performed on the data explained 25 per cent of the original variation of the data. The PCA factor loadings were examined, and the principal component tended to load positively on variables which contributed to lower vulnerability such as better education and better health, and negatively on variables which contributed to higher household vulnerability such as higher percentage of household income from agriculture. Therefore, the score is a measure of 'strength' or preparedness. A high score indicates a non-vulnerable household, and a low score indicates a vulnerable household.				

**Structured summary of operationalization – validity assessment**			**1.1 DCM Appropriate**	**1.2 valid empirical rep?**	**1. conclusion - Valid?**	**2. Feasible?**
**Construct:** inconsistent water suply						
**Article:** Hahn et al (2009)						
Criterion	Assessment	Quoted text or Rationale for negative assessment				
Construct defined?	Yes	Percentage of households that report that water is not available at their primary water source everyday	Yes/ no/ can't tell	Yes/ no/ can't tell	Yes/ no/ can't tell YES	Yes/ no/ can't tell YES
Data collection methods reported?	Yes	household surveys	YES			
Reporting of indicators/questions used to operationalise	Yes	Table 1 includes an explanation of how each sub-component was quantified, the survey question used to collect the data, the original source of the survey question, and potential sources of bias.		YES		

<table>
<tr>
<td>construct?</td>
<td></td>
<td>[...]
<table><tr><td colspan="3">Table 1 Major components and sub-components comprising the Livelihood Vulnerability Index (LVI) developed for two districts of Mozambique.</td></tr><tr><td>Sub-components</td><td>Explanation of sub-components</td><td>Survey question</td></tr><tr><td>Percent of households that do not have a consistent water supply</td><td>Percentage of households that report that water is not available at their primary water source everyday</td><td>Is this water available everyday?</td></tr></table></td>
<td></td>
<td></td>
<td></td>
<td></td>
</tr>
<tr>
<td>Sampling strategies reported?</td>
<td>Yes</td>
<td>We pilot tested the LVI and LVI–IPCC in the Moma and Mabote
Districts of Mozambique during 2007. These were selected by CARE-Mozambique as representative of coastal and inland communities, respectively, and the climate change issues con- fronting each.
[...]
Based on a sample size calculation (WHO, 2005) at the 95% confidence interval, [illegible]10% precision, 50% prevalence,1 and a design effect of 2 to account for cluster sampling, 200 households in each
district were surveyed.2 National 1997 census data that specified the total population in each village was used to select 20 villages in each district using the probability proportional to size method (WHO, 2005; UNICEF, 2008).
[...]
1 50% prevalence refers to the point prevalence of the indicators selected for the LVI. This is the default value for sample size calculations when the prevalence of the indicators is unknown.
2 Sample size formula: N = DEFF*[(Z2*p*q)/e2], where N = sample size, DEFF = 2; Z = 1.96 (95% CI), p = 0.5; q = 0.5; e = 0.10.</td>
<td></td>
<td></td>
<td></td>
<td></td>
</tr>
<tr>
<td>Sampling sizes</td>
<td>Yes</td>
<td>We pilot tested the LVI and LVI–IPCC in the Moma and</td>
<td></td>
<td></td>
<td></td>
<td></td>
</tr>
</table>

reported?		Mabote<br>Districts<br>[...]<br>200 households in each<br>district were surveyed.2 National 1997 census data that specified the total population in each village was used to select 20 villages in each district				
Data analysis methods reported?	Yes	The LVI uses a balanced weighted average approach (Sullivan<br>et al., 2002) where each sub-component contributes equally to the overall index even though each major component is comprised of a different number of sub-components. Becausewe intended to develop an assessment tool accessible to a diverse set of users in resource-poor settings, the LVI formula uses the simple approach of applying equal weights to all major components. This weighting scheme could be adjusted by future users as needed. Because each of the sub-components is measured on a different<br>scale, it was first necessary to standardize each as an index. The equation used for this conversion was adapted from that used in the Human Development Index to calculate the life expectancy index, which is the ratio of the difference of the actual life expectancy and a pre-selected minimum, and the range of pre- determined maximum and minimum life expectancy (UNDP, 2007):<br>$index_{sd} = (s_d - s_{min})/(s_{max} - s_{min})$<br>where sd is the original sub-component for district d, and smin and smax are the minimum and maximum values, respectively, for each sub-component determined using data from both districts.				
	Yes	Table 4 shows the weight assigned to each variable derived from the first principal<br>component. The data reduction that PCA performed on the data explained 25 per cent of the original variation of the data. The PCA factor loadings were examined, and the principal component tended to load<br>positively on variables which contributed to lower				

		vulnerability such as better education and better health, and negatively on variables which contributed to higher household vulnerability such as higher percentage of household income from agriculture. Therefore, the score is a measure of 'strength' or preparedness. A high score indicates a non-vulnerable household, and a low score indicates a vulnerable household.				

**Structured summary of operationalization – validity assessment**			**1.1 DCM Appropriate**	**1.2 valid empirical rep?**	**1. conclusion - Valid?**	**2. Feasible?**
**Construct:** injury or death from disaster						
**Article:** Hahn et al (2009)						
Criterion	Assessment	Quoted text or Rationale for negative assessment				
Construct defined?	Yes	Percentage of households that reported either an injury to or death of one of their family members as a result of the most severe flood, drought, or cyclone in the past 6 years.	Yes/ no/ can't tell	Yes/ no/ can't tell	Yes/ no/ can't tell YES	Yes/ no/ can't tell YESS
Data collection methods reported?	Yes	household surveys	YES			
Reporting of indicators/questions used to operationalise construct?	Yes	Table 1 includes an explanation of how each sub-component was quantified, the survey question used to collect the data, the original source of the survey question, and potential sources of bias. [...] (nested table below)		YES		

Table 1 Major components and sub-components comprising the Livelihood Vulnerability Index (LVI) developed for two districts of Mozambique.

Sub-components	*Explanation of sub-components*	*Survey question*
Percent of households with an injury or death as a	Percentage of households that reported	Was anyone in your family injured in the flood/cyclone drought? Did anyone in your family die during the

		result of the most severe natural disaster in the past 6 years | either an injury to or death of one of their family members as a result of the most severe flood, drought, or cyclone in the past 6 years. | flood/cyclone/drought?				
Sampling strategies reported?	Yes	We pilot tested the LVI and LVI–IPCC in the Moma and Mabote<br>Districts of Mozambique during 2007. These were selected by CARE-Mozambique as representative of coastal and inland communities, respectively, and the climate change issues con- fronting each.<br>[...]<br>Based on a sample size calculation (WHO, 2005) at the 95% confidence interval, ⍰10% precision, 50% prevalence,1 and a design effect of 2 to account for cluster sampling, 200 households in each<br>district were surveyed.2 National 1997 census data that specified the total population in each village was used to select 20 villages in each district using the probability proportional to size method (WHO, 2005; UNICEF, 2008).<br>[...]<br>1 50% prevalence refers to the point prevalence of the indicators selected for the LVI. This is the default value for sample size calculations when the prevalence of the indicators is unknown.<br>2 Sample size formula: N = DEFF*[(Z2*p*q)/e2], where N = sample size, DEFF = 2; Z = 1.96 (95% CI), p = 0.5; q = 0.5; e = 0.10.				
Sampling sizes reported?	Yes	We pilot tested the LVI and LVI–IPCC in the Moma and Mabote				

		Districts<br>[...]<br>200 households in each<br>district were surveyed.2 National 1997 census data that specified the total population in each village was used to select 20 villages in each district				
Data analysis methods reported?	Yes	The LVI uses a balanced weighted average approach (Sullivan<br>et al., 2002) where each sub-component contributes equally to the overall index even though each major component is comprised of a different number of sub-components. Becausewe intended to develop an assessment tool accessible to a diverse set of users in resource-poor settings, the LVI formula uses the simple approach of applying equal weights to all major components. This weighting scheme could be adjusted by future users as needed. Because each of the sub-components is measured on a different<br>scale, it was first necessary to standardize each as an index. The equation used for this conversion was adapted from that used in the Human Development Index to calculate the life expectancy index, which is the ratio of the difference of the actual life expectancy and a pre-selected minimum, and the range of pre- determined maximum and minimum life expectancy (UNDP, 2007):<br>$index_{sd} = (s_d - s_{min})/(s_{max} - s_{min})$<br>where sd is the original sub-component for district d, and smin and smax are the minimum and maximum values, respectively, for each sub-component determined using data from both districts.				
	Yes	Table 4 shows the weight assigned to each variable derived from the first principal<br>component. The data reduction that PCA performed on the data explained 25 per cent of the original variation of the data. The PCA factor loadings were examined, and the principal component tended to load<br>positively on variables which contributed to lower vulnerability such as better education and better health,				

		and negatively on variables which contributed to higher household vulnerability such as higher percentage of household income from agriculture. Therefore, the score is a measure of 'strength' or preparedness. A high score indicates a non-vulnerable household, and a low score indicates a vulnerable household.				

<table>
<tr><td colspan="3">Structured summary of operationalization – validity assessment</td><td rowspan="4">1.1 DCM Appropriate</td><td rowspan="4">1.2 valid empirical rep?</td><td rowspan="4">1. conclusion - Valid?</td><td rowspan="4">2. Feasible?</td></tr>
<tr><td colspan="3">Construct: inverse water stored</td></tr>
<tr><td colspan="3">Article: Hahn et al (2009)</td></tr>
<tr><td><u>Criterion</u></td><td><u>Assessment</u></td><td><u>Quoted text or Rationale for negative assessment</u></td></tr>
<tr><td>Construct defined?</td><td>Yes</td><td>The inverse of (the average number of liters of water stored by each household + 1).</td><td>Yes/ no/ can't tell</td><td>Yes/ no/ can't tell</td><td rowspan="4">Yes/ no/ can't tell

YES</td><td rowspan="4">Yes/ no/ can't tell

YES</td></tr>
<tr><td>Data collection methods reported?</td><td>Yes</td><td>household surveys</td><td>YES</td><td></td></tr>
<tr><td>Reporting of indicators/questions used to operationalise construct?</td><td>Yes</td><td>Table 1 includes an explanation of how each sub-component was quantified, the survey question used to collect the data, the original source of the survey question, and potential sources of bias.
[...]

Table 1 Major components and sub-components comprising the Livelihood Vulnerability Index (LVI) developed for two districts of Mozambique.
<table><tr><td>Sub-components</td><td>Explanation of sub-components</td><td>Survey question</td></tr><tr><td>Inverse of the average number of liters of water stored per household (range: >0–1)</td><td>The inverse of (the average number of liters of water stored by each household + 1).</td><td>What containers do you usually store water in? How many? How many liters are they?</td></tr></table></td><td></td><td>YES</td></tr>
<tr><td>Sampling strategies reported?</td><td>Yes</td><td>We pilot tested the LVI and LVI–IPCC in the Moma and Mabote
Districts of Mozambique during 2007. These were</td><td></td><td></td></tr>
</table>

		selected by CARE-Mozambique as representative of coastal and inland communities, respectively, and the climate change issues con- fronting each.<br>[...]<br>Based on a sample size calculation (WHO, 2005) at the 95% confidence interval, 10% precision, 50% prevalence,1 and a design effect of 2 to account for cluster sampling, 200 households in each<br>district were surveyed.2 National 1997 census data that specified the total population in each village was used to select 20 villages in each district using the probability proportional to size method (WHO, 2005; UNICEF, 2008).<br>[...]<br>1 50% prevalence refers to the point prevalence of the indicators selected for the LVI. This is the default value for sample size calculations when the prevalence of the indicators is unknown.<br>2 Sample size formula: N = DEFF*[(Z2*p*q)/e2], where N = sample size, DEFF = 2; Z = 1.96 (95% CI), p = 0.5; q = 0.5; e = 0.10.				
Sampling sizes reported?	Yes	We pilot tested the LVI and LVI–IPCC in the Moma and Mabote<br>Districts<br>[...]<br>200 households in each<br>district were surveyed.2 National 1997 census data that specified the total population in each village was used to select 20 villages in each district				
Data analysis methods reported?	Yes	The LVI uses a balanced weighted average approach (Sullivan<br>et al., 2002) where each sub-component contributes equally to the overall index even though each major component is comprised of a different number of sub-components. Becausewe intended to develop an assessment tool accessible to a diverse set of users in resource-poor settings, the LVI formula uses the simple approach of applying equal weights to all major components. This weighting scheme could be adjusted by				

		future users as needed. Because each of the sub-components is measured on a different scale, it was first necessary to standardize each as an index. The equation used for this conversion was adapted from that used in the Human Development Index to calculate the life expectancy index, which is the ratio of the difference of the actual life expectancy and a pre-selected minimum, and the range of pre- determined maximum and minimum life expectancy (UNDP, 2007): $index_{sd} = (s_d – s_{min})/(s_{max} – s_{min})$ where sd is the original sub-component for district d, and smin and smax are the minimum and maximum values, respectively, for each sub-component determined using data from both districts.				
	Yes	Table 4 shows the weight assigned to each variable derived from the first principal component. The data reduction that PCA performed on the data explained 25 per cent of the original variation of the data. The PCA factor loadings were examined, and the principal component tended to load positively on variables which contributed to lower vulnerability such as better education and better health, and negatively on variables which contributed to higher household vulnerability such as higher percentage of household income from agriculture. Therefore, the score is a measure of 'strength' or preparedness. A high score indicates a non-vulnerable household, and a low score indicates a vulnerable household.				

**Structured summary of operationalization – validity assessment**		
**Construct:** livelihood diversification		
**Article:** Hahn et al (2009)		
Criterion	Assessment	Quoted text or Rationale for negative assessment

<table>
<tr><td colspan="3">Structured summary of operationalization – validity assessment</td><td rowspan="4">1.1 DCM Appropriate</td><td rowspan="4">1.2 valid empirical rep?</td><td rowspan="4">1. conclusion - Valid?</td><td rowspan="4">2. Feasible?</td></tr>
<tr><td colspan="3">Construct: livelihood diversification</td></tr>
<tr><td colspan="3">Article: Hahn et al (2009)</td></tr>
<tr><td><u>Criterion</u></td><td><u>Assessment</u></td><td><u>Quoted text or Rationale for negative assessment</u></td></tr>
<tr><td>Construct defined?</td><td>Yes</td><td>The inverse of (the number of agricultural livelihood activities +1) reported by a household, e.g., A household that farms, raises animals, and collects natural resources will have a Livelihood Diversification Index = 1/(3 + 1) = 0.25.</td><td>Yes/ no/ can't tell</td><td>Yes/ no/ can't tell</td><td rowspan="3">Yes/ no/ can't tell

YES</td><td rowspan="3">Yes/ no/ can't tell
YES</td></tr>
<tr><td>Data collection methods reported?</td><td>Yes</td><td>household surveys</td><td>YES</td><td></td></tr>
<tr><td>Reporting of indicators/questions used to operationalise construct?</td><td>Yes</td><td>Table 1 includes an explanation of how each sub-component was quantified, the survey question used to collect the data, the original source of the survey question, and potential sources of bias.
[...]

<table><tr><td colspan="3">Table 1 Major components and sub-components comprising the Livelihood Vulnerability Index (LVI) developed for two districts of Mozambique.</td></tr><tr><td>Sub-components</td><td>Explanation of sub-components</td><td>Survey question</td></tr><tr><td>Average Agricultural Livelihood Diversification Index (range: 0.20–1)a</td><td>The inverse of (the number of agricultural livelihood activities +1) reported by a household, e.g., A household that farms, raises animals, and collects natural resources will have a</td><td>Do you or someone else in your household raise animals? Do you or someone else in your household grow crops? Do you or someone else in your household collect something from the bush, the</td></tr></table></td><td></td><td>YES</td></tr>
</table>

		Livelihood Diversification Index = 1/(3 + 1) = 0.25.<br>forest, or lakes and rivers to sell?				
Sampling strategies reported?	Yes	We pilot tested the LVI and LVI–IPCC in the Moma and Mabote<br>Districts of Mozambique during 2007. These were selected by CARE-Mozambique as representative of coastal and inland communities, respectively, and the climate change issues con- fronting each.<br>[...]<br>Based on a sample size calculation (WHO, 2005) at the 95% confidence interval, □10% precision, 50% prevalence,1 and a design effect of 2 to account for cluster sampling, 200 households in each<br>district were surveyed.2 National 1997 census data that specified the total population in each village was used to select 20 villages in each district using the probability proportional to size method (WHO, 2005; UNICEF, 2008).<br>[...]<br>1 50% prevalence refers to the point prevalence of the indicators selected for the LVI. This is the default value for sample size calculations when the prevalence of the indicators is unknown.<br>2 Sample size formula: N = DEFF*[(Z2*p*q)/e2], where N = sample size, DEFF = 2; Z = 1.96 (95% CI), p = 0.5; q = 0.5; e = 0.10.				
Sampling sizes reported?	Yes	We pilot tested the LVI and LVI–IPCC in the Moma and Mabote<br>Districts<br>[...]<br>200 households in each<br>district were surveyed.2 National 1997 census data that specified the total population in each village was used to select 20 villages in each district				
Data analysis methods reported?	Yes	The LVI uses a balanced weighted average approach (Sullivan<br>et al., 2002) where each sub-component contributes				

		equally to the overall index even though each major component is comprised of a different number of sub-components. Becausewe intended to develop an assessment tool accessible to a diverse set of users in resource-poor settings, the LVI formula uses the simple approach of applying equal weights to all major components. This weighting scheme could be adjusted by future users as needed. Because each of the sub-components is measured on a different scale, it was first necessary to standardize each as an index. The equation used for this conversion was adapted from that used in the Human Development Index to calculate the life expectancy index, which is the ratio of the difference of the actual life expectancy and a pre-selected minimum, and the range of pre- determined maximum and minimum life expectancy (UNDP, 2007): $index_{sd} = (s_d - s_{min})/(s_{max} - s_{min})$ where sd is the original sub-component for district d, and smin and smax are the minimum and maximum values, respectively, for each sub-component determined using data from both districts.				
	Yes	Table 4 shows the weight assigned to each variable derived from the first principal component. The data reduction that PCA performed on the data explained 25 per cent of the original variation of the data. The PCA factor loadings were examined, and the principal component tended to load positively on variables which contributed to lower vulnerability such as better education and better health, and negatively on variables which contributed to higher household vulnerability such as higher percentage of household income from agriculture. Therefore, the score is a measure of 'strength' or preparedness. A high score indicates a non-vulnerable household, and a low score indicates a vulnerable household.				

<table>
<tr><td colspan="3">Structured summary of operationalization – validity assessment</td></tr>
<tr><td colspan="3">Construct: maximum temperature</td></tr>
<tr><td colspan="3">Article: Hahn et al (2009)</td></tr>
<tr><td><u>Criterion</u></td><td><u>Assessment</u></td><td><u>Quoted text or Rationale for negative assessment</u></td></tr>
</table>

<table>
<tr><td colspan="3">Structured summary of operationalization – validity assessment</td><td rowspan="4">1.1 DCM Appropriate</td><td rowspan="4">1.2 valid empirical rep?</td><td rowspan="4">1. conclusion - Valid?</td><td rowspan="4">2. Feasible?</td></tr>
<tr><td colspan="3">Construct: maximum temperature</td></tr>
<tr><td colspan="3">Article: Hahn et al (2009)</td></tr>
<tr><td><u>Criterion</u></td><td><u>Assessment</u></td><td><u>Quoted text or Rationale for negative assessment</u></td></tr>
<tr><td>Construct defined?</td><td>Yes</td><td>Standard deviation of the average daily maximum temperature by month between 1998 and 2003 was averaged for each provinceb</td><td>Yes/ no/ can’t tell</td><td>Yes/ no/ can’t tell</td><td rowspan="5">Yes/ no/ can’t tell
YES</td><td rowspan="5">Yes/ no/ can’t tell
YES</td></tr>
<tr><td>Data collection methods reported?</td><td>2ndary data</td><td>provincial data; weather station based in the provincial capital</td><td>YES</td><td></td></tr>
<tr><td>Reporting of indicators/questions used to operationalise construct?</td><td>2ndary data</td><td><table>
<tr><td colspan="3">Table 1 Major components and sub-components comprising the Livelihood Vulnerability Index (LVI) developed for two districts of Mozambique.</td></tr>
<tr><td>Sub-components</td><td>Explanation of sub-components</td><td>Survey question</td></tr>
<tr><td>Mean standard deviation of the daily average maximum temperature by month</td><td>Standard deviation of the average daily maximum temperature by month between 1998 and 2003 was averaged for each provinceb</td><td>1998–2003: provincial data; weather station based in the provincial capital</td></tr>
</table></td><td></td><td>YES</td></tr>
<tr><td>Sampling strategies reported?</td><td>2ndary data</td><td></td><td></td><td></td></tr>
<tr><td>Sampling sizes</td><td>2ndary data</td><td></td><td></td><td></td></tr>
</table>

reported?						
Data analysis methods reported?	Yes	The LVI uses a balanced weighted average approach (Sullivan et al., 2002) where each sub-component contributes equally to the overall index even though each major component is comprised of a different number of sub-components. Becausewe intended to develop an assessment tool accessible to a diverse set of users in resource-poor settings, the LVI formula uses the simple approach of applying equal weights to all major components. This weighting scheme could be adjusted by future users as needed. Because each of the sub-components is measured on a different scale, it was first necessary to standardize each as an index. The equation used for this conversion was adapted from that used in the Human Development Index to calculate the life expectancy index, which is the ratio of the difference of the actual life expectancy and a pre-selected minimum, and the range of pre- determined maximum and minimum life expectancy (UNDP, 2007): $index_{sd} = (s_d - s_{min})/(s_{max} - s_{min})$ where sd is the original sub-component for district d, and smin and smax are the minimum and maximum values, respectively, for each sub-component determined using data from both districts.				
	Yes	Table 4 shows the weight assigned to each variable derived from the first principal component. The data reduction that PCA performed on the data explained 25 per cent of the original variation of the data. The PCA factor loadings were examined, and the principal component tended to load positively on variables which contributed to lower vulnerability such as better education and better health, and negatively on variables which contributed to higher household vulnerability such as higher percentage of household income from agriculture. Therefore, the score is a measure of 'strength' or preparedness. A high score indicates a non-vulnerable household, and a low score				

		indicates a vulnerable household.				

**Structured summary of operationalization – validity assessment**			**1.1 DCM Appropriate**	**1.2 valid empirical rep?**	**1. conclusion - Valid?**	**2. Feasible?**
**Construct:** natural water source						
**Article:** Hahn et al (2009)						
Criterion	Assessment	Quoted text or Rationale for negative assessment				
Construct defined?	Yes	Percentage of households that report a creek, river, lake, pool, or hole as their primary water source.	Yes/ no/ can't tell	Yes/ no/ can't tell	Yes/ no/ can't tell<br>YES	Yes/ no/ can't tell<br>YES
Data collection methods reported?	Yes	household surveys	YES			
Reporting of indicators/questions used to operationalise construct?	Yes	Table 1 includes an explanation of how each sub-component was quantified, the survey question used to collect the data, the original source of the survey question, and potential sources of bias.<br>[...]<br><br>Table 1 Major components and sub-components comprising the Livelihood Vulnerability Index (LVI) developed for two districts of Mozambique.<br>*Sub-components* / *Explanation of sub-components* / *Survey question*<br>Percent of households that utilize a natural water source / Percentage of households that report a creek, river, lake, pool, or hole as their primary water source. / Where do you collect your water from?		YES		
Sampling strategies reported?	Yes	We pilot tested the LVI and LVI–IPCC in the Moma and Mabote<br>Districts of Mozambique during 2007. These were selected by CARE-Mozambique as representative of coastal and inland communities, respectively, and the climate change issues con- fronting each.				

		[...]<br>Based on a sample size calculation (WHO, 2005) at the 95% confidence interval, 10% precision, 50% prevalence,1 and a design effect of 2 to account for cluster sampling, 200 households in each<br>district were surveyed.2 National 1997 census data that specified the total population in each village was used to select 20 villages in each district using the probability proportional to size method (WHO, 2005; UNICEF, 2008).<br>[...]<br>1 50% prevalence refers to the point prevalence of the indicators selected for the LVI. This is the default value for sample size calculations when the prevalence of the indicators is unknown.<br>2 Sample size formula: N = DEFF*[(Z2*p*q)/e2], where N = sample size, DEFF = 2; Z = 1.96 (95% CI), p = 0.5; q = 0.5; e = 0.10.				
Sampling sizes reported?	Yes	We pilot tested the LVI and LVI–IPCC in the Moma and Mabote<br>Districts<br>[...]<br>200 households in each<br>district were surveyed.2 National 1997 census data that specified the total population in each village was used to select 20 villages in each district				
Data analysis methods reported?	Yes	The LVI uses a balanced weighted average approach (Sullivan<br>et al., 2002) where each sub-component contributes equally to the overall index even though each major component is comprised of a different number of sub-components. Becausewe intended to develop an assessment tool accessible to a diverse set of users in resource-poor settings, the LVI formula uses the simple approach of applying equal weights to all major components. This weighting scheme could be adjusted by future users as needed. Because each of the sub-components is measured on a different<br>scale, it was first necessary to standardize each as an				

		index. The equation used for this conversion was adapted from that used in the Human Development Index to calculate the life expectancy index, which is the ratio of the difference of the actual life expectancy and a pre-selected minimum, and the range of pre- determined maximum and minimum life expectancy (UNDP, 2007): $index_{sd} = (s_d – s_{min})/(s_{max} – s_{min})$ where sd is the original sub-component for district d, and smin and smax are the minimum and maximum values, respectively, for each sub-component determined using data from both districts.				
	Yes	Table 4 shows the weight assigned to each variable derived from the first principal component. The data reduction that PCA performed on the data explained 25 per cent of the original variation of the data. The PCA factor loadings were examined, and the principal component tended to load positively on variables which contributed to lower vulnerability such as better education and better health, and negatively on variables which contributed to higher household vulnerability such as higher percentage of household income from agriculture. Therefore, the score is a measure of 'strength' or preparedness. A high score indicates a non-vulnerable household, and a low score indicates a vulnerable household.				

**Structured summary of operationalization – validity assessment**			**1.1 DCM Appropriate**	**1.2 valid empirical rep?**	**1. conclusion - Valid?**	**2. Feasible?**
**Construct:** no warning of disaster						
**Article:** Hahn et al (2009)						
Criterion	Assessment	Quoted text or Rationale for negative assessment				
Construct defined?	Yes	Percentage of households that did not receive a warning about the most severe flood, drought, and cyclone event in the past 6 years.	Yes/ no/ can't tell	Yes/ no/ can't tell	Yes/ no/ can't tell YES	Yes/ no/ can't tell YES
Data collection methods reported?	Yes	household surveys	YES			

<table>
<tr>
<td>Reporting of indicators/questions used to operationalise construct?</td>
<td>Yes</td>
<td>Table 1 includes an explanation of how each sub-component was quantified, the survey question used to collect the data, the original source of the survey question, and potential sources of bias.
[...]

<table>
<tr><td colspan="3">Table 1 Major components and sub-components comprising the Livelihood Vulnerability Index (LVI) developed for two districts of Mozambique.</td></tr>
<tr><td>Sub-components</td><td>Explanation of sub-components</td><td>Survey question</td></tr>
<tr><td>Percent of households that did not receive a warning about the pending natural disasters</td><td>Percentage of households that did not receive a warning about the most severe flood, drought, and cyclone event in the past 6 years.</td><td>Did you receive a warning about the flood/cyclone/drought before it happened?</td></tr>
</table>
</td>
<td></td>
<td>YES</td>
<td></td>
<td></td>
</tr>
<tr>
<td>Sampling strategies reported?</td>
<td>Yes</td>
<td>We pilot tested the LVI and LVI–IPCC in the Moma and Mabote
Districts of Mozambique during 2007. These were selected by CARE-Mozambique as representative of coastal and inland communities, respectively, and the climate change issues con- fronting each.
[...]
Based on a sample size calculation (WHO, 2005) at the 95% confidence interval, [illegible]10% precision, 50% prevalence,1 and a design effect of 2 to account for cluster sampling, 200 households in each
district were surveyed.2 National 1997 census data that specified the total population in each village was used to</td>
<td></td>
<td></td>
<td></td>
<td></td>
</tr>
</table>

		select 20 villages in each district using the probability proportional to size method (WHO, 2005; UNICEF, 2008).<br>[...]<br>1 50% prevalence refers to the point prevalence of the indicators selected for the LVI. This is the default value for sample size calculations when the prevalence of the indicators is unknown.<br>2 Sample size formula: N = DEFF*[(Z2*p*q)/e2], where N = sample size, DEFF = 2; Z = 1.96 (95% CI), p = 0.5; q = 0.5; e = 0.10.				
Sampling sizes reported?	Yes	We pilot tested the LVI and LVI–IPCC in the Moma and Mabote<br>Districts<br>[...]<br>200 households in each<br>district were surveyed.2 National 1997 census data that specified the total population in each village was used to select 20 villages in each district				
Data analysis methods reported?	Yes	The LVI uses a balanced weighted average approach (Sullivan<br>et al., 2002) where each sub-component contributes equally to the overall index even though each major component is comprised of a different number of sub-components. Becausewe intended to develop an assessment tool accessible to a diverse set of users in resource-poor settings, the LVI formula uses the simple approach of applying equal weights to all major components. This weighting scheme could be adjusted by future users as needed. Because each of the sub-components is measured on a different<br>scale, it was first necessary to standardize each as an index. The equation used for this conversion was adapted from that used in the Human Development Index to calculate the life expectancy index, which is the ratio of the difference of the actual life expectancy and a pre-selected minimum, and the range of pre- determined maximum and minimum life expectancy (UNDP, 2007):<br>$index_{sd} = (s_d - s_{min})/(s_{max} - s_{min})$				

		where sd is the original sub-component for district d, and smin and smax are the minimum and maximum values, respectively, for each sub-component determined using data from both districts.				
	Yes	Table 4 shows the weight assigned to each variable derived from the first principal component. The data reduction that PCA performed on the data explained 25 per cent of the original variation of the data. The PCA factor loadings were examined, and the principal component tended to load positively on variables which contributed to lower vulnerability such as better education and better health, and negatively on variables which contributed to higher household vulnerability such as higher percentage of household income from agriculture. Therefore, the score is a measure of 'strength' or preparedness. A high score indicates a non-vulnerable household, and a low score indicates a vulnerable household.				

**Structured summary of operationalization – validity assessment**			**1.1 DCM Appropriate**	**1.2 valid empirical rep?**	**1. conclusion - Valid?**	**2. Feasible?**
**Construct:** precent of female-headed households						
**Article:** Hahn et al (2009)						
Criterion	Assessment	Quoted text or Rationale for negative assessment				
Construct defined?	Yes	Percentage of households where the primary adult is female. If a male head is away from the home >6 months per year the female is counted as the head of the household	Yes/ no/ can't tell	Yes/ no/ can't tell	Yes/ no/ can't tell YES	Yes/ no/ can't tell YES
Data collection methods reported?	Yes	household surveys	YES			
Reporting of indicators/questions used to operationalise construct?	Yes	Table 1 includes an explanation of how each sub-component was quantified, the survey question used to collect the data, the original source of the survey question, and potential sources of bias. [...]		YES		

<table>
<tr>
<td></td>
<td></td>
<td>
<table>
<tr><td colspan="3">Table 1 Major components and sub-components comprising the Livelihood Vulnerability Index (LVI) developed for two districts of Mozambique.</td></tr>
<tr><td>Sub-components</td><td>Explanation of sub-components</td><td>Survey question</td></tr>
<tr><td>Percent of female-headed households</td><td>Percentage of households where the primary adult is female. If a male head is away from the home >6 months per year the female is counted as the head of the household.</td><td>Are you the head of the household?</td></tr>
</table>
</td>
<td></td>
<td></td>
<td></td>
<td></td>
</tr>
<tr>
<td>Sampling strategies reported?</td>
<td>Yes</td>
<td>We pilot tested the LVI and LVI–IPCC in the Moma and Mabote
Districts of Mozambique during 2007. These were selected by CARE-Mozambique as representative of coastal and inland communities, respectively, and the climate change issues con- fronting each.
[...]
Based on a sample size calculation (WHO, 2005) at the 95% confidence interval, [illegible]10% precision, 50% prevalence,1 and a design effect of 2 to account for cluster sampling, 200 households in each
district were surveyed.2 National 1997 census data that specified the total population in each village was used to select 20 villages in each district using the probability proportional to size method (WHO, 2005; UNICEF, 2008).
[...]
1 50% prevalence refers to the point prevalence of the indicators selected for the LVI. This is the default value for sample size calculations when the prevalence of the indicators is unknown.
2 Sample size formula: N = DEFF*[(Z2*p*q)/e2], where N</td>
<td></td>
<td></td>
<td></td>
<td></td>
</tr>
</table>

		= sample size, DEFF = 2; Z = 1.96 (95% CI), p = 0.5; q = 0.5; e = 0.10.				
Sampling sizes reported?	Yes	We pilot tested the LVI and LVI–IPCC in the Moma and Mabote<br>Districts<br>[...]<br>200 households in each<br>district were surveyed.2 National 1997 census data that specified the total population in each village was used to select 20 villages in each district				
Data analysis methods reported?	Yes	The LVI uses a balanced weighted average approach (Sullivan<br>et al., 2002) where each sub-component contributes equally to the overall index even though each major component is comprised of a different number of sub-components. Becausewe intended to develop an assessment tool accessible to a diverse set of users in resource-poor settings, the LVI formula uses the simple approach of applying equal weights to all major components. This weighting scheme could be adjusted by future users as needed. Because each of the sub-components is measured on a different<br>scale, it was first necessary to standardize each as an index. The equation used for this conversion was adapted from that used in the Human Development Index to calculate the life expectancy index, which is the ratio of the difference of the actual life expectancy and a pre-selected minimum, and the range of pre- determined maximum and minimum life expectancy (UNDP, 2007):<br>$index_{sd} = (s_d – s_{min})/(s_{max} – s_{min})$<br>where sd is the original sub-component for district d, and smin and smax are the minimum and maximum values, respectively, for each sub-component determined using data from both districts.				
	Yes	Table 4 shows the weight assigned to each variable derived from the first principal<br>component. The data reduction that PCA performed on the data explained 25 per cent of the original variation of				

		the data. The PCA factor loadings were examined, and the principal component tended to load positively on variables which contributed to lower vulnerability such as better education and better health, and negatively on variables which contributed to higher household vulnerability such as higher percentage of household income from agriculture. Therefore, the score is a measure of 'strength' or preparedness. A high score indicates a non-vulnerable household, and a low score indicates a vulnerable household.				

**Structured summary of operationalization – validity assessment**			**1.1 DCM Appropriate**	**1.2 valid empirical rep?**	**1. conclusion - Valid?**	**2. Feasible?**
**Construct:** proximity to health facility						
**Article:** Hahn et al (2009)						
Criterion	Assessment	Quoted text or Rationale for negative assessment				
Construct defined?	Yes	Average time it takes the households to get to the nearest health facility.	Yes/ no/ can't tell	Yes/ no/ can't tell	Yes/ no/ can't tell<br>YES	Yes/ no/ can't tell<br>YES
Data collection methods reported?	Yes	household surveys	YES			
Reporting of indicators/questions used to operationalise construct?	Yes	Table 1 includes an explanation of how each sub-component was quantified, the survey question used to collect the data, the original source of the survey question, and potential sources of bias.<br>[...]<br><br>Table 1 Major components and sub-components comprising the Livelihood Vulnerability Index (LVI) developed for two districts of Mozambique.<br>*Sub-components* / *Explanation of sub-components* / *Survey question*<br>Average time to health facility (minutes) / Average time it takes the households to get to the nearest health / Howlong does it take you to get to a health facility?		YES		

		facility.				
Sampling strategies reported?	Yes	We pilot tested the LVI and LVI–IPCC in the Moma and Mabote<br>Districts of Mozambique during 2007. These were selected by CARE-Mozambique as representative of coastal and inland communities, respectively, and the climate change issues con- fronting each.<br>[...]<br>Based on a sample size calculation (WHO, 2005) at the 95% confidence interval, 10% precision, 50% prevalence,1 and a design effect of 2 to account for cluster sampling, 200 households in each<br>district were surveyed.2 National 1997 census data that specified the total population in each village was used to select 20 villages in each district using the probability proportional to size method (WHO, 2005; UNICEF, 2008).<br>[...]<br>1 50% prevalence refers to the point prevalence of the indicators selected for the LVI. This is the default value for sample size calculations when the prevalence of the indicators is unknown.<br>2 Sample size formula: N = DEFF*[(Z2*p*q)/e2], where N = sample size, DEFF = 2; Z = 1.96 (95% CI), p = 0.5; q = 0.5; e = 0.10.				
Sampling sizes reported?	Yes	We pilot tested the LVI and LVI–IPCC in the Moma and Mabote<br>Districts<br>[...]<br>200 households in each<br>district were surveyed.2 National 1997 census data that specified the total population in each village was used to select 20 villages in each district				
Data analysis methods reported?	Yes	The LVI uses a balanced weighted average approach (Sullivan<br>et al., 2002) where each sub-component contributes equally to the overall index even though each major component is comprised of a different number of sub-components. Becausewe intended to develop an				

		assessment tool accessible to a diverse set of users in resource-poor settings, the LVI formula uses the simple approach of applying equal weights to all major components. This weighting scheme could be adjusted by future users as needed. Because each of the sub-components is measured on a different<br>scale, it was first necessary to standardize each as an index. The equation used for this conversion was adapted from that used in the Human Development Index to calculate the life expectancy index, which is the ratio of the difference of the actual life expectancy and a pre-selected minimum, and the range of pre- determined maximum and minimum life expectancy (UNDP, 2007):<br>$index_{sd} = (s_d - s_{min})/(s_{max} - s_{min})$<br>where sd is the original sub-component for district d, and smin and smax are the minimum and maximum values, respectively, for each sub-component determined using data from both districts.				
	Yes	Table 4 shows the weight assigned to each variable derived from the first principal<br>component. The data reduction that PCA performed on the data explained 25 per cent of the original variation of the data. The PCA factor loadings were examined, and the principal component tended to load<br>positively on variables which contributed to lower vulnerability such as better education and better health, and negatively on variables which contributed to higher household vulnerability such as higher percentage of household income from agriculture. Therefore, the score is a measure of 'strength' or preparedness. A high score indicates a non-vulnerable household, and a low score indicates a vulnerable household.				

**Structured summary of operationalization – validity assessment**	**1.1 DCM Appropriate**	**1.2 valid empirical**	**1. conclusion -**	**2. Feasible?**
**Construct:** proximity to water source				

<table>
<tr><td colspan="3">Article: Hahn et al (2009)</td><td rowspan="2"></td><td rowspan="2">rep?</td><td rowspan="2">Valid?</td><td rowspan="2"></td></tr>
<tr><td><u>Criterion</u></td><td><u>Assessment</u></td><td><u>Quoted text or Rationale for negative assessment</u></td></tr>
<tr><td>Construct defined?</td><td>Yes</td><td>Average time it takes the households to travel to their primary water source.</td><td>Yes/ no/ can’t tell</td><td>Yes/ no/ can’t tell</td><td rowspan="4">Yes/ no/ can’t tell
YES</td><td rowspan="4">Yes/ no/ can’t tell
YES</td></tr>
<tr><td>Data collection methods reported?</td><td>Yes</td><td>household surveys</td><td>YES</td><td></td></tr>
<tr><td>Reporting of indicators/questions used to operationalise construct?</td><td>Yes</td><td>Table 1 includes an explanation of how each sub-component was quantified, the survey question used to collect the data, the original source of the survey question, and potential sources of bias.
[...]
<table><tr><td colspan="3">Table 1 Major components and sub-components comprising the Livelihood Vulnerability Index (LVI) developed for two districts of Mozambique.</td></tr><tr><td>Sub-components</td><td>Explanation of sub-components</td><td>Survey question</td></tr><tr><td>Average time to water source (minutes)</td><td>Average time it takes the households to travel to their primary water source.</td><td>How long does it take to get to your water source?</td></tr></table></td><td></td><td>YES</td></tr>
<tr><td>Sampling strategies reported?</td><td>Yes</td><td>We pilot tested the LVI and LVI–IPCC in the Moma and Mabote
Districts of Mozambique during 2007. These were selected by CARE-Mozambique as representative of coastal and inland communities, respectively, and the climate change issues con- fronting each.
[...]
Based on a sample size calculation (WHO, 2005) at the 95% confidence interval, □10% precision, 50% prevalence,1 and a design effect of 2 to account for cluster sampling, 200 households in each
district were surveyed.2 National 1997 census data that specified the total population in each village was used to select 20 villages in each district using the probability proportional to size method (WHO, 2005; UNICEF, 2008).</td><td></td><td></td></tr>
</table>

		[...]<br>1 50% prevalence refers to the point prevalence of the indicators selected for the LVI. This is the default value for sample size calculations when the prevalence of the indicators is unknown.<br>2 Sample size formula: N = DEFF*[(Z2*p*q)/e2], where N = sample size, DEFF = 2; Z = 1.96 (95% CI), p = 0.5; q = 0.5; e = 0.10.				
Sampling sizes reported?	Yes	We pilot tested the LVI and LVI–IPCC in the Moma and Mabote<br>Districts<br>[...]<br>200 households in each<br>district were surveyed.2 National 1997 census data that specified the total population in each village was used to select 20 villages in each district				
Data analysis methods reported?	Yes	The LVI uses a balanced weighted average approach (Sullivan<br>et al., 2002) where each sub-component contributes equally to the overall index even though each major component is comprised of a different number of sub-components. Becausewe intended to develop an assessment tool accessible to a diverse set of users in resource-poor settings, the LVI formula uses the simple approach of applying equal weights to all major components. This weighting scheme could be adjusted by future users as needed. Because each of the sub-components is measured on a different<br>scale, it was first necessary to standardize each as an index. The equation used for this conversion was adapted from that used in the Human Development Index to calculate the life expectancy index, which is the ratio of the difference of the actual life expectancy and a pre-selected minimum, and the range of pre- determined maximum and minimum life expectancy (UNDP, 2007):<br>$index_{sd} = (s_d - s_{min})/(s_{max} - s_{min})$<br>where sd is the original sub-component for district d, and smin and smax are the minimum and maximum values,				

		respectively, for each sub-component determined using data from both districts.				
	Yes	Table 4 shows the weight assigned to each variable derived from the first principal component. The data reduction that PCA performed on the data explained 25 per cent of the original variation of the data. The PCA factor loadings were examined, and the principal component tended to load positively on variables which contributed to lower vulnerability such as better education and better health, and negatively on variables which contributed to higher household vulnerability such as higher percentage of household income from agriculture. Therefore, the score is a measure of 'strength' or preparedness. A high score indicates a non-vulnerable household, and a low score indicates a vulnerable household.				

**Structured summary of operationalization – validity assessment**			**1.1 DCM Appropriate**	**1.2 valid empirical rep?**	**1. conclusion - Valid?**	**2. Feasible?**
**Construct:** receive-give ratio						
**Article:** Hahn et al (2009)						
<u>Criterion</u>	<u>Assessment</u>	<u>Quoted text or Rationale for negative assessment</u>				
Construct defined?	Yes	Ratio of (the number of types of help received by a household in the past month + 1) to (the number of types of help given by a household to someone else in the past month + 1).	Yes/ no/ can't tell	Yes/ no/ can't tell	Yes/ no/ can't tell YES	Yes/ no/ can't tell YES
Data collection methods reported?	Yes	household surveys	YES			
Reporting of indicators/questions used to operationalise construct?	Yes	Table 1 includes an explanation of how each sub-component was quantified, the survey question used to collect the data, the original source of the survey question, and potential sources of bias.<br>[...]<br><br>Table 1 Major components and sub-components comprising the Livelihood Vulnerability Index (LVI) developed for two districts of Mozambique.<br>*Sub-components* | *Explanation of* | *Survey question*		YES		

<table>
<tr><td></td><td></td><td>
<table>
<tr><td></td><td>sub-components</td><td></td></tr>
<tr><td>Average Receive:Give ratio (range: 0–15)</td><td>Ratio of (the number of types of help received by a household in the past month + 1) to (the number of types of help given by a household to someone else in the past month + 1).</td><td>In the past month, did relatives or friends help you and your family: (e.g., Get medical care or medicines, Sell animal products or other goods produced by family, Take care of children)
In the past month, did you and your family help relatives or friends: (same choices as above)</td></tr>
</table>
</td><td></td><td></td><td></td><td></td><td></td></tr>
<tr><td>Sampling strategies reported?</td><td>Yes</td><td>We pilot tested the LVI and LVI–IPCC in the Moma and Mabote
Districts of Mozambique during 2007. These were selected by CARE-Mozambique as representative of coastal and inland communities, respectively, and the climate change issues con- fronting each.
[...]
Based on a sample size calculation (WHO, 2005) at the 95% confidence interval, ⍰10% precision, 50% prevalence,1 and a design effect of 2 to account for cluster sampling, 200 households in each
district were surveyed.2 National 1997 census data that specified the total population in each village was used to select 20 villages in each district using the probability proportional to size method (WHO, 2005; UNICEF, 2008).
[...]
1 50% prevalence refers to the point prevalence of the</td><td></td><td></td><td></td><td></td><td></td></tr>
</table>

		indicators selected for the LVI. This is the default value for sample size calculations when the prevalence of the indicators is unknown.<br>2 Sample size formula: N = DEFF*[(Z2*p*q)/e2], where N = sample size, DEFF = 2; Z = 1.96 (95% CI), p = 0.5; q = 0.5; e = 0.10.				
Sampling sizes reported?	Yes	We pilot tested the LVI and LVI–IPCC in the Moma and Mabote<br>Districts<br>[...]<br>200 households in each<br>district were surveyed.2 National 1997 census data that specified the total population in each village was used to select 20 villages in each district				
Data analysis methods reported?	Yes	The LVI uses a balanced weighted average approach (Sullivan<br>et al., 2002) where each sub-component contributes equally to the overall index even though each major component is comprised of a different number of sub-components. Becausewe intended to develop an assessment tool accessible to a diverse set of users in resource-poor settings, the LVI formula uses the simple approach of applying equal weights to all major components. This weighting scheme could be adjusted by future users as needed. Because each of the sub-components is measured on a different<br>scale, it was first necessary to standardize each as an index. The equation used for this conversion was adapted from that used in the Human Development Index to calculate the life expectancy index, which is the ratio of the difference of the actual life expectancy and a pre-selected minimum, and the range of pre- determined maximum and minimum life expectancy (UNDP, 2007):<br>$index_{sd} = (s_d - s_{min})/(s_{max} - s_{min})$<br>where sd is the original sub-component for district d, and smin and smax are the minimum and maximum values, respectively, for each sub-component determined using data from both districts.				

	Yes	Table 4 shows the weight assigned to each variable derived from the first principal component. The data reduction that PCA performed on the data explained 25 per cent of the original variation of the data. The PCA factor loadings were examined, and the principal component tended to load positively on variables which contributed to lower vulnerability such as better education and better health, and negatively on variables which contributed to higher household vulnerability such as higher percentage of household income from agriculture. Therefore, the score is a measure of 'strength' or preparedness. A high score indicates a non-vulnerable household, and a low score indicates a vulnerable household.				

**Structured summary of operationalization – validity assessment**			**1.1 DCM Appropriate**	**1.2 valid empirical rep?**	**1. conclusion - Valid?**	**2. Feasible?**
**Construct:** struggle for food						
**Article:** Hahn et al (2009)						
Criterion	Assessment	Quoted text or Rationale for negative assessment				
Construct defined?	Yes	Average number of months households struggle to obtain food for their family.	Yes/ no/ can't tell	Yes/ no/ can't tell	Yes/ no/ can't tell<br>YESS	Yes/ no/ can't tell<br>YES
Data collection methods reported?	Yes	household surveys	YES			
Reporting of indicators/questions used to operationalise construct?	Yes	Table 1 includes an explanation of how each sub-component was quantified, the survey question used to collect the data, the original source of the survey question, and potential sources of bias.<br>[...]<br><br>Table 1 Major components and sub-components comprising the Livelihood Vulnerability Index (LVI) developed for two districts of Mozambique.<br>*Sub-components* | *Explanation of sub-components* | *Survey question*		YES		

		Average number of months households struggle to find food (range: 0–12)<br><br>Average number of months households struggle to obtain food for their family.<br><br>Does your family have adequate food the whole year, or are there times during the year that your family does not have enough food? Howmanymonths a year does your family have trouble getting enough food?				
Sampling strategies reported?	Yes	We pilot tested the LVI and LVI–IPCC in the Moma and Mabote<br>Districts of Mozambique during 2007. These were selected by CARE-Mozambique as representative of coastal and inland communities, respectively, and the climate change issues con- fronting each.<br>[...]<br>Based on a sample size calculation (WHO, 2005) at the 95% confidence interval, ⍰10% precision, 50% prevalence,1 and a design effect of 2 to account for cluster sampling, 200 households in each<br>district were surveyed.2 National 1997 census data that specified the total population in each village was used to select 20 villages in each district using the probability proportional to size method (WHO, 2005; UNICEF, 2008).<br>[...]<br>1 50% prevalence refers to the point prevalence of the indicators selected for the LVI. This is the default value for sample size calculations when the prevalence of the indicators is unknown.<br>2 Sample size formula: N = DEFF*[(Z2*p*q)/e2], where N = sample size, DEFF = 2; Z = 1.96 (95% CI), p = 0.5; q = 0.5; e = 0.10.				
Sampling sizes	Yes	We pilot tested the LVI and LVI–IPCC in the Moma and				

reported?		Mabote<br>Districts<br>[...]<br>200 households in each<br>district were surveyed.2 National 1997 census data that specified the total population in each village was used to select 20 villages in each district				
Data analysis methods reported?	Yes	The LVI uses a balanced weighted average approach (Sullivan<br>et al., 2002) where each sub-component contributes equally to the overall index even though each major component is comprised of a different number of sub-components. Becausewe intended to develop an assessment tool accessible to a diverse set of users in resource-poor settings, the LVI formula uses the simple approach of applying equal weights to all major components. This weighting scheme could be adjusted by future users as needed. Because each of the sub-components is measured on a different<br>scale, it was first necessary to standardize each as an index. The equation used for this conversion was adapted from that used in the Human Development Index to calculate the life expectancy index, which is the ratio of the difference of the actual life expectancy and a pre-selected minimum, and the range of pre- determined maximum and minimum life expectancy (UNDP, 2007):<br>$index_{sd} = (s_d - s_{min})/(s_{max} - s_{min})$<br>where sd is the original sub-component for district d, and smin and smax are the minimum and maximum values, respectively, for each sub-component determined using data from both districts.				
	Yes	Table 4 shows the weight assigned to each variable derived from the first principal<br>component. The data reduction that PCA performed on the data explained 25 per cent of the original variation of the data. The PCA factor loadings were examined, and the principal component tended to load<br>positively on variables which contributed to lower				

<table>
<tr><td></td><td></td><td>vulnerability such as better education and better health, and negatively on variables which contributed to higher household vulnerability such as higher percentage of household income from agriculture. Therefore, the score is a measure of ‘strength’ or preparedness. A high score indicates a non-vulnerable household, and a low score indicates a vulnerable household.</td><td></td><td></td><td></td><td></td></tr>
</table>

<table>
<tr><td colspan="3">Structured summary of operationalization – validity assessment</td><td rowspan="4">1.1 DCM Appropriate</td><td rowspan="4">1.2 valid empirical rep?</td><td rowspan="4">1. conclusion - Valid?</td><td rowspan="4">2. Feasible?</td></tr>
<tr><td colspan="3">Construct: uneducated headed households</td></tr>
<tr><td colspan="3">Article: Hahn et al (2009)</td></tr>
<tr><td><u>Criterion</u></td><td><u>Assessment</u></td><td><u>Quoted text or Rationale for negative assessment</u></td></tr>
<tr><td>Construct defined?</td><td>Yes</td><td>Percentage of households where the head of the household reports that they have attended 0 years of school</td><td>Yes/ no/ can’t tell</td><td>Yes/ no/ can’t tell</td><td rowspan="3">Yes/ no/ can’t tell
NO</td><td rowspan="3">Yes/ no/ can’t tell
YES</td></tr>
<tr><td>Data collection methods reported?</td><td>Yes</td><td>household surveys</td><td>YES</td><td></td></tr>
<tr><td>Reporting of indicators/questions used to operationalise construct?</td><td>Yes</td><td>Table 1 includes an explanation of how each sub-component was quantified, the survey question used to collect the data, the original source of the survey question, and potential sources of bias.
[...]

<table>
<tr><td colspan="3">Table 1 Major components and sub-components comprising the Livelihood Vulnerability Index (LVI) developed for two districts of Mozambique.</td></tr>
<tr><td>Sub-components</td><td>Explanation of sub-components</td><td>Survey question</td></tr>
<tr><td>Percent of households where head of household has not attended school</td><td>Percentage of households where the head of the household reports that they have</td><td>Did you ever go to school?</td></tr>
</table></td><td></td><td>NO</td></tr>
</table>

		attended 0 years of school.				
Sampling strategies reported?	Yes	We pilot tested the LVI and LVI–IPCC in the Moma and Mabote<br>Districts of Mozambique during 2007. These were selected by CARE-Mozambique as representative of coastal and inland communities, respectively, and the climate change issues con- fronting each.<br>[...]<br>Based on a sample size calculation (WHO, 2005) at the 95% confidence interval, □10% precision, 50% prevalence,1 and a design effect of 2 to account for cluster sampling, 200 households in each<br>district were surveyed.2 National 1997 census data that specified the total population in each village was used to select 20 villages in each district using the probability proportional to size method (WHO, 2005; UNICEF, 2008).<br>[...]<br>1 50% prevalence refers to the point prevalence of the indicators selected for the LVI. This is the default value for sample size calculations when the prevalence of the indicators is unknown.<br>2 Sample size formula: N = DEFF*[(Z2*p*q)/e2], where N = sample size, DEFF = 2; Z = 1.96 (95% CI), p = 0.5; q = 0.5; e = 0.10.				
Sampling sizes reported?	Yes	We pilot tested the LVI and LVI–IPCC in the Moma and Mabote<br>Districts<br>[...]<br>200 households in each<br>district were surveyed.2 National 1997 census data that specified the total population in each village was used to select 20 villages in each district				
Data analysis methods reported?	Yes	The LVI uses a balanced weighted average approach (Sullivan<br>et al., 2002) where each sub-component contributes equally to the overall index even though each major component is comprised of a different number of sub-				

		components. Becausewe intended to develop an assessment tool accessible to a diverse set of users in resource-poor settings, the LVI formula uses the simple approach of applying equal weights to all major components. This weighting scheme could be adjusted by future users as needed. Because each of the sub-components is measured on a different scale, it was first necessary to standardize each as an index. The equation used for this conversion was adapted from that used in the Human Development Index to calculate the life expectancy index, which is the ratio of the difference of the actual life expectancy and a pre-selected minimum, and the range of pre- determined maximum and minimum life expectancy (UNDP, 2007): $index_{sd} = (s_d - s_{min})/(s_{max} - s_{min})$ where sd is the original sub-component for district d, and smin and smax are the minimum and maximum values, respectively, for each sub-component determined using data from both districts.				
	Yes	Table 4 shows the weight assigned to each variable derived from the first principal component. The data reduction that PCA performed on the data explained 25 per cent of the original variation of the data. The PCA factor loadings were examined, and the principal component tended to load positively on variables which contributed to lower vulnerability such as better education and better health, and negatively on variables which contributed to higher household vulnerability such as higher percentage of household income from agriculture. Therefore, the score is a measure of 'strength' or preparedness. A high score indicates a non-vulnerable household, and a low score indicates a vulnerable household.				

**Structured summary of operationalization – validity assessment**	**1.1 DCM**	**1.2 valid**	**1.**	**2. Feasible?**

<table>
<tr><td colspan="3">Construct: water conflict</td><td rowspan="3">Appropriate</td><td rowspan="3">empirical rep?</td><td rowspan="3">conclusion - Valid?</td><td rowspan="3"></td></tr>
<tr><td colspan="3">Article: Hahn et al (2009)</td></tr>
<tr><td><u>Criterion</u></td><td><u>Assessment</u></td><td><u>Quoted text or Rationale for negative assessment</u></td></tr>
<tr><td>Construct defined?</td><td>Yes</td><td>Percentage of households that report having heard about conflicts over water in their community</td><td>Yes/ no/ can’t tell</td><td>Yes/ no/ can’t tell</td><td rowspan="4">Yes/ no/ can’t tell
YES</td><td rowspan="4">Yes/ no/ can’t tell
YES</td></tr>
<tr><td>Data collection methods reported?</td><td>Yes</td><td>household surveys</td><td>YES</td><td></td></tr>
<tr><td>Reporting of indicators/questions used to operationalise construct?</td><td>Yes</td><td>Table 1 includes an explanation of how each sub-component was quantified, the survey question used to collect the data, the original source of the survey question, and potential sources of bias.
[...]
<table>
<tr><td colspan="3">Table 1 Major components and sub-components comprising the Livelihood Vulnerability Index (LVI) developed for two districts of Mozambique.</td></tr>
<tr><td>Sub-components</td><td>Explanation of sub-components</td><td>Survey question</td></tr>
<tr><td>Percent of households reporting water conflicts</td><td>Percentage of households that report having heard about conflicts over water in their community.</td><td>In the past year, have you heard about any conflicts over water in your community?</td></tr>
</table></td><td></td><td>YES</td></tr>
<tr><td>Sampling strategies reported?</td><td>Yes</td><td>We pilot tested the LVI and LVI–IPCC in the Moma and Mabote
Districts of Mozambique during 2007. These were selected by CARE-Mozambique as representative of coastal and inland communities, respectively, and the climate change issues con- fronting each.
[...]
Based on a sample size calculation (WHO, 2005) at the 95% confidence interval, 10% precision, 50% prevalence,1 and a design effect of 2 to account for cluster sampling, 200 households in each
district were surveyed.2 National 1997 census data that specified the total population in each village was used to</td><td></td><td></td></tr>
</table>

		select 20 villages in each district using the probability proportional to size method (WHO, 2005; UNICEF, 2008).<br>[...]<br>1 50% prevalence refers to the point prevalence of the indicators selected for the LVI. This is the default value for sample size calculations when the prevalence of the indicators is unknown.<br>2 Sample size formula: N = DEFF*[(Z2*p*q)/e2], where N = sample size, DEFF = 2; Z = 1.96 (95% CI), p = 0.5; q = 0.5; e = 0.10.				
Sampling sizes reported?	Yes	We pilot tested the LVI and LVI–IPCC in the Moma and Mabote<br>Districts<br>[...]<br>200 households in each<br>district were surveyed.2 National 1997 census data that specified the total population in each village was used to select 20 villages in each district				
Data analysis methods reported?	Yes	The LVI uses a balanced weighted average approach (Sullivan<br>et al., 2002) where each sub-component contributes equally to the overall index even though each major component is comprised of a different number of sub-components. Becausewe intended to develop an assessment tool accessible to a diverse set of users in resource-poor settings, the LVI formula uses the simple approach of applying equal weights to all major components. This weighting scheme could be adjusted by future users as needed. Because each of the sub-components is measured on a different<br>scale, it was first necessary to standardize each as an index. The equation used for this conversion was adapted from that used in the Human Development Index to calculate the life expectancy index, which is the ratio of the difference of the actual life expectancy and a pre-selected minimum, and the range of pre- determined maximum and minimum life expectancy (UNDP, 2007):<br>$index_{sd} = (s_d - s_{min})/(s_{max} - s_{min})$				

		where sd is the original sub-component for district d, and smin and smax are the minimum and maximum values, respectively, for each sub-component determined using data from both districts.				
	Yes	Table 4 shows the weight assigned to each variable derived from the first principal component. The data reduction that PCA performed on the data explained 25 per cent of the original variation of the data. The PCA factor loadings were examined, and the principal component tended to load positively on variables which contributed to lower vulnerability such as better education and better health, and negatively on variables which contributed to higher household vulnerability such as higher percentage of household income from agriculture. Therefore, the score is a measure of 'strength' or preparedness. A high score indicates a non-vulnerable household, and a low score indicates a vulnerable household.				

**Transparency Assessment Article summary**	
**Article**	Ionesco et al (2009)
**Transparent operationalizations**	Adaptive capacity as set (ATEAM); entity (ATEAM); preference criteria (ATEAM); reference scenarios (ATEAM); stimulus (ATEAM); adaptive capacity as set (DINAS); entity (DINAS); preference criteria (DINAS); reference scenario (DINAS); stimulus (DINAS).
**Partially transparent**	
**Not transparent**	

**Structured summary of operationalization – validity assessment**			**1.1 DCM Appropriate**	**1.2 valid empirical rep?**	**1. conclusion - Valid?**	**2. Feasible?**
**Construct:** adaptive capacity as set						
**Article:** Ionesco et al (2009)						
Criterion	Assessment	Quoted text or Rationale for negative assessment				
Construct defined?	Yes	Definition (Adaptive capacity as a set) The adaptive capacity of a system f in state x subjected to an input e is represented by the set of its effective actions.	Yes/ no/ can't tell	Yes/ no/ can't tell	Yes/ no/ can't tell CAN'T TELL	Yes/ no/ can't tell CAN'T TELL
Data collection methods reported?	ATEAM: 2ndary data	The objective of this section is to relate the framework developed in Section 3 to the IPCCconceptualisation of vulnerability (see Fig. 1) and to two recent vulnerability assessments:Advanced Terrestrial EcosystemAnalysis and Modelling (ATEAM) and Dynamic and Inter- active Assessment of National, Regional and Global Vulnerability of Coastal Zones to Climate Change and Sea-Level Rise (DINAS-COAST).<br>[...]<br>4.2Advanced Terrestrial EcosystemAnalysis andModelling<br>[...]<br>Socio-economic data were used to assess adaptive capacity on a sub-national scale, in a way that allowed it to be projected into the future using the same set of scenarios as for the assessment of po- tential impacts.<br>[...]	CAN'T TELL			

Reporting of indicators/questions used to operationalise construct?	ATEAM: 2ndary data			-		
Sampling strategies reported?	ATEAM: 2ndary data					
Sampling sizes reported?	ATEAM: 2ndary data					
Data analysis methods reported?	ATEAM: Yes	4.2Advanced Terrestrial EcosystemAnalysis andModelling [...] Adaptive capacity was modelled as an index that was chosen to be a real number between 0 and 1. It was developed by building a statistical model from observed socio-economic data, which was then applied to the IPCC SRES scenarios to produce future projections of adaptive capacity. The adaptive capacity index can be seen within our framework as an estimate of the size of the set of available actions Uk. The socio-economic data used to derive the index (e.g.,GDPper capita, literacy rate and labour participation rate of women) indicate the capac-ity of society to prepare for and respond to impacts of global change by choosing an appropriate action (i.e., ecosystem management strategy). The size of this set of actions can be assumed to be an indication of the size of the set of effective actions, since the latter is a subset of the former.				

**Structured summary of operationalization – validity assessment**			**1.1 DCM Appropriate**	**1.2 valid empirical rep?**	**1. conclusion - Valid?**	**2. Feasible?**
**Construct:** adaptive capacity as set						
**Article:** Ionesco et al (2009)						
Criterion	Assessment	Quoted text or Rationale for negative assessment				
Construct defined?	Yes	Definition (Adaptive capacity as a set) The adaptive	Yes/ no/ can't	Yes/ no/	Yes/ no/	Yes/ no/

		capacity of a system f in state x subjected to an input e is represented by the set of its effective actions.	tell	can’t tell	can’t tell	can’t tell
Data collection methods reported?	DINAS: 2ndary data	The objective of this section is to relate the framework developed in Section 3 to the IPCCconceptualisation of vulnerability (see Fig. 1) and to two recent vulnerability assessments:Advanced Terrestrial EcosystemAnalysis and Modelling (ATEAM) and Dynamic and Inter- active Assessment of National, Regional and Global Vulnerability of Coastal Zones to Climate Change and Sea-Level Rise (DINAS-COAST).<br>[...]<br>4.3 Dynamic and InteractiveAssessment of National, Regional and Global Vulnerability of Coastal Zones to Climate Change and Sea-Level Rise<br>[...]<br>The project DINAS-COAST (http://www.dinas-coast. net) was also funded by the Research Directorate- General of the European Commission from 2001 to 2004. Five partners and two subcontractors worked together to develop the dynamic, interactive and flexi- ble tool Dynamic and Interactive VulnerabilityAssess- ment (DIVA, [5]). DIVA enables its users to assess coastal vulnerability to sea-level rise and to explore possible adaptation policies.	CAN’T TELL		CAN’T TELL	
Reporting of indicators/questions used to operationalise construct?	DINAS: 2ndary data			-		
Sampling strategies reported?	DINAS: 2ndary data					
Sampling sizes reported?	DINAS: 2ndary data					
Data analysis methods reported?	DINAS: Yes	4.3 Dynamic and InteractiveAssessment of National, Regional and Global Vulnerability of Coastal Zones to				

		Climate Change and Sea-Level Rise<br>[...]<br>In contrast toATEAM,the transition function of the coupled human–environment system was known and has the form of Eq. 19. In addition to the input, controls (i.e., adaptation actions) were included in the model. The actions contained in the set of controls U were (1) do nothing, (2) build dikes, (3) move away and (4) nourish the beach or tidal basins. Given f ,U andasetofscenarios E, the vulnerability<br>of the system could have been assessed by computing the transition of the system for every adaptation action<br>$u \in U$ and comparing the resulting set of possible states X_{k+1} with the previous state x_k. However, doing so would be computationally expensive. Instead, DIVA<br>introduced adaptation policies. An adaptation policy is a function that returns an adaptation action u for every state of the system and input it receives from the environment:<br>$\phi : X \times E \rightarrow U, \phi(x_k, e_k) = u_k$. (21)<br>The following adaptation policies were considered:<br>• No adaptation: the model computes only potential impacts.<br>• Full protection: raise dikes or nourish beaches as much as is necessary to preserve the status quo<br>(i.e., x_0).<br>• Optimal protection: optimisation based on the comparison of the monetary costs and benefits of adaptation actions and potential impacts.<br>• User-defined protection: the user defines a flood return period against which to protect.<br>The composition of the adaptation policy ϕ with<br>the state transition function f transforms the non-deterministic system into a deterministic one:<br>$x_{k+1} = f(x_k, e_k, u_k) = f(x_k, e_k, \phi(x_k, e_k)) = f$ [illegible](x_k, e_k). (22)				

**Structured summary of operationalization – validity assessment**			**1.1 DCM Appropriate**	**1.2 valid empirical rep?**	**1. conclusion - Valid?**	**2. Feasible?**
**Construct:** Entity						
**Article:** Ionesco et al (2009)						
Criterion	Assessment	Quoted text or Rationale for negative assessment				
Construct defined?	Yes	The mainstream mathematical interpretation of an entity is that of a dynamical system in a given state. This is the interpretation we will adopt here	Yes/ no/ can't tell	Yes/ no/ can't tell	Yes/ no/ can't tell<br><br>CAN'T TELL	Yes/ no/ can't tell
Data collection methods reported?	ATEAM: 2ndary data	The objective of this section is to relate the framework developed in Section 3 to the IPCCconceptualisation of vulnerability (see Fig. 1) and to two recent vulnerability assessments:Advanced Terrestrial EcosystemAnalysis and Modelling (ATEAM) and Dynamic and Inter- active Assessment of National, Regional and Global Vulnerability of Coastal Zones to Climate Change and Sea-Level Rise (DINAS-COAST).<br>[...]<br>4.2Advanced Terrestrial EcosystemAnalysis andModelling<br>[...]<br>It involved 13 partners and six subcontractors, whose joint activities resulted in the development of a vulnerability mapping tool [21]. The project adopted the IPCC conceptualisation of vul- nerability, which required combining information on potential impactswith information on adaptive capacity (see Fig. 1). Socio-economic data were used to assess adaptive capacity on a sub-national scale, in a way that allowed it to be projected into the future using the same set of scenarios as for the assessment of po- tential impacts. The information on potential impacts and adaptive capacity was then combined in a series of vulnerability maps [25].	CAN'T TELL			
Reporting of indicators/questions used to operationalise construct?	ATEAM: 2ndary data			-		
Sampling strategies reported?	ATEAM: 2ndary data					

Sampling sizes reported?	ATEAM: 2ndary data					
Data analysis methods reported?	ATEAM: Yes	4.2Advanced Terrestrial EcosystemAnalysis andModelling [...] ATEAM aimed "to assess where in Europe people may be vulnerable to the loss of particular ecosystem services, associated with the combined effects of climate change, land use change and atmospheric pollution" ([22], p. 3). Thus, the entity is a coupled human–ecological system: the people in Europe who rely on ecosystem services. The system receives both input (the stimuli) and controls (the human actions). The evolution of such a system can be given by xk+1 = f (xk, ek,uk), (19) where k denotes the time step and uk is an element of the set of available controls Uk, which are the man- agement actions people can apply to adapt to poten- tial impacts and, thus, maintain the ecosystem services on which they rely. These actions are usually specific to the ecosystem service considered. For example, a management action for ensuring the ecosystem service "agriculture" could be to irrigate the land.				

**Structured summary of operationalization – validity assessment**			**1.1 DCM Appropriate**	**1.2 valid empirical rep?**	**1. conclusion - Valid?**	**2. Feasible?**
**Construct:** Entity						
**Article:** Ionesco et al (2009)						
Criterion	Assessment	Quoted text or Rationale for negative assessment				
Construct defined?	Yes	The mainstream mathematical interpretation of an entity is that of a dynamical system in a given state. This is the interpretation we will adopt here	Yes/ no/ can't tell	Yes/ no/ can't tell	Yes/ no/ can't tell<br><br>CAN'T TELL	Yes/ no/ can't tell
Data collection methods reported?	DINAS:	The objective of this section is to relate the framework developed in Section 3 to the IPCCconceptualisation of	CAN'T TELL			

	2ndary data	vulnerability (see Fig. 1) and to two recent vulnerability assessments:Advanced Terrestrial EcosystemAnalysis and Modelling (ATEAM) and Dynamic and Inter- active Assessment of National, Regional and Global Vulnerability of Coastal Zones to Climate Change and Sea-Level Rise (DINAS-COAST).<br>[...]<br>4.2Advanced Terrestrial EcosystemAnalysis andModelling<br>[...]<br>It involved 13 partners and six subcontractors, whose joint activities resulted in the development of a vulnerability mapping tool [21]. The project adopted the IPCC conceptualisation of vul- nerability, which required combining information on potential impactswith information on adaptive capacity (see Fig. 1). Socio-economic data were used to assess adaptive capacity on a sub-national scale, in a way that allowed it to be projected into the future using the same set of scenarios as for the assessment of po- tential impacts. The information on potential impacts and adaptive capacity was then combined in a series of vulnerability maps [25].<br>[...]<br>4.3 Dynamic and InteractiveAssessment of National, Regional and Global Vulnerability of Coastal Zones to Climate Change and Sea-Level Rise<br>[...]<br>The project DINAS-COAST (http://www.dinas-coast. net) was also funded by the Research Directorate- General of the European Commission from 2001 to 2004. Five partners and two subcontractors worked together to develop the dynamic, interactive and flexi- ble tool Dynamic and Interactive VulnerabilityAssess- ment (DIVA, [5]). DIVA enables its users to assess coastal vulnerability to sea-level rise and to explore possible adaptation policies.				
Reporting of indicators/questions used to	DINAS:<br>2ndary data			-		

operationalise construct?						
Sampling strategies reported?	DINAS: 2ndary data					
Sampling sizes reported?	DINAS: 2ndary data					
Data analysis methods reported?	DINAS: Yes	4.2Advanced Terrestrial EcosystemAnalysis andModelling<br>[...]<br>ATEAM aimed "to assess where in Europe people may be vulnerable to the loss of particular ecosystem services, associated with the combined effects of climate change, land use change and atmospheric pollution" ([22], p. 3). Thus, the entity is a coupled human–ecological system: the people in Europe who rely on ecosystem services. The system receives both input (the stimuli) and controls (the human actions). The evolution of such a system can be given by<br>xk+1 = f (xk, ek,uk), (19)<br>where k denotes the time step and uk is an element of the set of available controls Uk, which are the man- agement actions people can apply to adapt to poten- tial impacts and, thus, maintain the ecosystem services on which they rely. These actions are usually specific to the ecosystem service considered. For example, a management action for ensuring the ecosystem service "agriculture" could be to irrigate the land.<br>[...]<br>4.3 Dynamic and InteractiveAssessment of National, Regional and Global Vulnerability of Coastal Zones to Climate Change and Sea-Level Rise<br>[...]<br>The first primitive, the vulnerable entity, is the coastal system.				

**Structured summary of operationalization – validity assessment**			**1.1 DCM Appropriate**	**1.2 valid empirical rep?**	**1. conclusion - Valid?**	**2. Feasible?**
**Construct:** preference criteria						
**Article:** Ionesco et al (2009)						
Criterion	Assessment	Quoted text or Rationale for negative assessment				
Construct defined?	Yes	Definition (Hazard, potential impact) An input e ∈ E is a hazard for a system f in state x if ∃ u ∈ U : f (x, e,u) ≺ f (x, e∗,u∗). In this case, f (x, e,u) is called a potential impact.	Yes/ no/ can't tell	Yes/ no/ can't tell	Yes/ no/ can't tell CAN'T TELL	Yes/ no/ can't tell
Data collection methods reported?	ATEAM: 2ndary data	The objective of this section is to relate the framework developed in Section 3 to the IPCCconceptualisation of vulnerability (see Fig. 1) and to two recent vulnerability assessments:Advanced Terrestrial EcosystemAnalysis and Modelling (ATEAM) and Dynamic and Inter- active Assessment of National, Regional and Global Vulnerability of Coastal Zones to Climate Change and Sea-Level Rise (DINAS-COAST).<br>[...]<br>4.2Advanced Terrestrial EcosystemAnalysis andModelling<br>[...]<br>It involved 13 partners and six subcontractors, whose joint activities resulted in the development of a vulnerability mapping tool [21]. The project adopted the IPCC conceptualisation of vul- nerability, which required combining information on potential impactswith information on adaptive capacity (see Fig. 1). Socio-economic data were used to assess adaptive capacity on a sub-national scale, in a way that allowed it to be projected into the future using the same set of scenarios as for the assessment of po- tential impacts. The information on potential impacts and adaptive capacity was then combined in a series of vulnerability maps [25].	CAN'T TELL			
Reporting of indicators/questions used to operationalise	ATEAM: 2ndary data			-		

construct?						
Sampling strategies reported?	ATEAM: 2ndary data					
Sampling sizes reported?	ATEAM: 2ndary data					
Data analysis methods reported?	ATEAM: Yes	4.2Advanced Terrestrial EcosystemAnalysis andModelling<br>[...]<br>The third primitive notion concerns the preference criteria represented by a (partial) strict order ≺,which relate to the loss of ecosystem services.We will discuss the preference criteria in more detail below.<br>[...]<br>The (partial) strict order was therefore developed in consultation with stakeholders in the form of an impact function on the set of states (also referred to as output or indicator function), in a similar way as shown in Example 3. The impact function reduces the thematic components of the state vector to a single real number between 0 and 1 for each ecosystem service.<br>[...]				

Structured summary of operationalization – validity assessment			1.1 DCM Appropriate	1.2 valid empirical rep?	1. conclusion - Valid?	2. Feasible?
**Construct:** preference criteria						
**Article:** Ionesco et al (2009)						
Criterion	Assessment	Quoted text or Rationale for negative assessment				
Construct defined?	Yes	Definition (Hazard, potential impact) An input e ∈ E is a hazard for a system f in state x if ∃ u ∈ U : f (x, e,u) ≺ f (x, e∗,u∗). In this case, f (x, e,u) is called<br>a potential impact.	Yes/ no/ can't tell	Yes/ no/ can't tell	Yes/ no/ can't tell CAN'T TELL	Yes/ no/ can't tell
Data collection methods reported?	DINAS: 2ndary data	The objective of this section is to relate the framework developed in Section 3 to the IPCCconceptualisation of vulnerability (see Fig. 1) and to two recent vulnerability	CAN'T TELL			

		assessments:Advanced Terrestrial EcosystemAnalysis and Modelling (ATEAM) and Dynamic and Inter- active Assessment of National, Regional and Global Vulnerability of Coastal Zones to Climate Change and Sea-Level Rise (DINAS-COAST).<br>[...]<br>[...]<br>4.3 Dynamic and InteractiveAssessment of National, Regional and Global Vulnerability of Coastal Zones to Climate Change and Sea-Level Rise<br>[...]<br>The project DINAS-COAST (http://www.dinas-coast. net) was also funded by the Research Directorate- General of the European Commission from 2001 to 2004. Five partners and two subcontractors worked together to develop the dynamic, interactive and flexi- ble tool Dynamic and Interactive VulnerabilityAssess- ment (DIVA, [5]). DIVA enables its users to assess coastal vulnerability to sea-level rise and to explore possible adaptation policies.				
Reporting of indicators/questions used to operationalise construct?	DINAS:<br>2ndary data			-		
Sampling strategies reported?	DINAS:<br>2ndary data					
Sampling sizes reported?	DINAS:<br>2ndary data					
Data analysis methods reported?	DINAS:<br>Yes	[...]<br>4.3 Dynamic and InteractiveAssessment of National, Regional and Global Vulnerability of Coastal Zones to Climate Change and Sea-Level Rise<br>[...]<br>The third primitive, the partial strict order was given in the form of an impact function on the set of states. The				

		function computes additional diagnostic properties such as people at risk of flooding, land loss, economic damages and the cost of protecting the coast. In con- trast to ATEAM, the impact function does not re- duce and normalise the dimensions of the state vector. One could say that DINAS-COAST provides a sparser partial strict order than ATEAM. Only the vector's monetary components can be directly compared, which is also the basis for the optimal protection policy. The comparison of the vector's non-monetary components is left to the individual user, as is the choice of a reference scenario and reference control policy. For this purpose the model is providedwith a graphical user interface that allows for the visual comparison of the outputs for different regions, time steps, scenarios and adaptation policies in form of graphs, tables and maps.				

**Structured summary of operationalization – validity assessment**			**1.1 DCM Appropriate**	**1.2 valid empirical rep?**	**1. conclusion - Valid?**	**2. Feasible?**
**Construct:** reference scenarios						
**Article:** Ionesco et al (2009)						
<u>Criterion</u>	<u>Assessment</u>	<u>Quoted text or Rationale for negative assessment</u>				
Construct defined?	Yes	The examples provided also have this "punctual" or "one-step" character. However, in many applications, it is more natural to consider an evolution of the system to be a sequence of states, and to consider scenarios and reference scenarios instead of punctual inputs for the vulnerability assessment. A scenario is just a sequence of inputs: es =[e1,<br>e2,..., en]. Corresponding to such a sequence, the sys- tem will undergo n transitions, xs =[x0, x1,..., xn]	Yes/ no/ can't tell	Yes/ no/ can't tell	Yes/ no/ can't tell CAN'T TELL	Yes/ no/ can't tell
Data collection methods reported?	ATEAM: 2ndary data	The objective of this section is to relate the framework developed in Section 3 to the IPCCconceptualisation of vulnerability (see Fig. 1) and to two recent vulnerability assessments:Advanced Terrestrial EcosystemAnalysis and Modelling (ATEAM) and Dynamic and Inter- active	CAN'T TELL			

		Assessment of National, Regional and Global Vulnerability of Coastal Zones to Climate Change and Sea-Level Rise (DINAS-COAST).<br>[...]<br>4.2Advanced Terrestrial EcosystemAnalysis andModelling<br>[...]<br>It involved 13 partners and six subcontractors, whose joint activities resulted in the development of a vulnerability mapping tool [21]. The project adopted the IPCC conceptualisation of vul- nerability, which required combining information on potential impactswith information on adaptive capacity (see Fig. 1). Socio-economic data were used to assess adaptive capacity on a sub-national scale, in a way that allowed it to be projected into the future using the same set of scenarios as for the assessment of po- tential impacts. The information on potential impacts and adaptive capacity was then combined in a series of vulnerability maps [25].<br>[...]				
Reporting of indicators/questions used to operationalise construct?	ATEAM: 2ndary data			-		
Sampling strategies reported?	ATEAM: 2ndary data					
Sampling sizes reported?	ATEAM: 2ndary data					
Data analysis methods reported?	ATEAM: Yes	4.2Advanced Terrestrial EcosystemAnalysis andModelling<br>[...]<br>To allow for such comparisons was one of the main objectives of ATEAM. Depending on the purposes of the assessment, the reference input could be chosen to be “no input”, that is, the next state was compared to the current one, or one of the other inputs prepared in				

		accordance to the SRES scenarios.<br>[...]<br>.				

Structured summary of operationalization – validity assessment			1.1 DCM Appropriate	1.2 valid empirical rep?	1. conclusion - Valid?	2. Feasible?
**Construct:** reference scenarios						
**Article:** Ionesco et al (2009)						
Criterion	Assessment	Quoted text or Rationale for negative assessment				
Construct defined?	Yes	The examples provided also have this “punctual” or “one-step” character. However, in many applications, it is more natural to consider an evolution of the system to be a sequence of states, and to consider scenarios and reference scenarios instead of punctual inputs for the vulnerability assessment. A scenario is just a sequence of inputs: es =[e1,<br>e2,..., en]. Corresponding to such a sequence, the sys- tem will undergo n transitions, xs =[x0, x1,..., xn]	Yes/ no/ can’t tell	Yes/ no/ can’t tell	Yes/ no/ can’t tell<br>CAN’T TELL	Yes/ no/ can’t tell
Data collection methods reported?	DINAS: 2ndary data	The objective of this section is to relate the framework developed in Section 3 to the IPCCconceptualisation of vulnerability (see Fig. 1) and to two recent vulnerability assessments:Advanced Terrestrial EcosystemAnalysis and Modelling (ATEAM) and Dynamic and Inter- active Assessment of National, Regional and Global Vulnerability of Coastal Zones to Climate Change and Sea-Level Rise (DINAS-COAST).<br>[...]<br>4.3 Dynamic and InteractiveAssessment of National, Regional and Global Vulnerability of Coastal Zones to Climate Change and Sea-Level Rise<br>[...]<br>The project DINAS-COAST (http://www.dinas-coast. net) was also funded by the Research Directorate- General of the European Commission from 2001 to 2004. Five partners and two subcontractors worked together to develop the dynamic, interactive and flexi- ble tool	CAN’T TELL			

		Dynamic and Interactive VulnerabilityAssess- ment (DIVA, [5]). DIVA enables its users to assess coastal vulnerability to sea-level rise and to explore possible adaptation policies.				
Reporting of indicators/questions used to operationalise construct?	DINAS: 2ndary data			-		
Sampling strategies reported?	DINAS: 2ndary data					
Sampling sizes reported?	DINAS: 2ndary data					
Data analysis methods reported?	DINAS: Yes	4.3 Dynamic and InteractiveAssessment of National, Regional and Global Vulnerability of Coastal Zones to Climate Change and Sea-Level Rise<br>[...]<br>The comparison of the vector's non-monetary components is left to the individual user, as is the choice of a reference scenario and reference control policy. For this purpose the model is providedwith a graphical user interface that allows for the visual comparison of the outputs for different regions, time steps, scenarios and adaptation policies in form of graphs, tables and maps.				

**Structured summary of operationalization – validity assessment**			**1.1 DCM Appropriate**	**1.2 valid empirical rep?**	**1. conclusion - Valid?**	**2. Feasible?**
**Construct:** Stimulus						
**Article:** Ionesco et al (2009)						
Criterion	Assessment	Quoted text or Rationale for negative assessment				
Construct defined?	Yes	The stimuli to which such a system can be subjected are then naturally represented by the inputs to the system. The simplest kind of dynamical system with input is a discrete, deter- ministic one, given by a transition	Yes/ no/ can't tell	Yes/ no/ can't tell	Yes/ no/ can't tell CAN'T TELL	Yes/ no/ can't tell

		function (see [14]):<br>$f : X \times E \rightarrow X$, (1)				
Data collection methods reported?	ATEAM:<br>Yes	The objective of this section is to relate the framework developed in Section 3 to the IPCCconceptualisation of vulnerability (see Fig. 1) and to two recent vulnerability assessments:Advanced Terrestrial EcosystemAnalysis and Modelling (ATEAM) and Dynamic and Inter- active Assessment of National, Regional and Global Vulnerability of Coastal Zones to Climate Change and Sea-Level Rise (DINAS-COAST).<br>[...]<br>4.2Advanced Terrestrial EcosystemAnalysis andModelling<br>[...]<br>It involved 13 partners and six subcontractors, whose joint activities resulted in the development of a vulnerability mapping tool [21]. The project adopted the IPCC conceptualisation of vul- nerability, which required combining information on potential impactswith information on adaptive capacity (see Fig. 1). Socio-economic data were used to assess adaptive capacity on a sub-national scale, in a way that allowed it to be projected into the future using the same set of scenarios as for the assessment of po- tential impacts. The information on potential impacts and adaptive capacity was then combined in a series of vulnerability maps [25].	CAN'T TELL			
Reporting of indicators/questions used to operationalise construct?	ATEAM:<br>2ndary data			-		
Sampling strategies reported?	ATEAM:<br>2ndary data					
Sampling sizes reported?	ATEAM:<br>2ndary data					
Data analysis methods reported?	ATEAM:<br>Yes	4.2Advanced Terrestrial EcosystemAnalysis andModelling<br>[...]				

		The second primitive is the stimulus or input e ∈ E,<br>to which the system's vulnerability was assessed. This input was given by the scenarios of climate, land use and nitrogen deposition, which represent the pos- sible evolutions of the environment. The scenarios were based on the IPCC SRES storylines (for details, see [22]).<br>[...]<br>The transition function of the deterministic system can then be given by<br>xk+1 = f˜ u(xk, ek). (20) This equation now allows for the computation of<br>possible future states (i.e., xk+1) for the given scenarios. However, to assert that an entity is vulnerable, the third primitive, a (partial) strict order, is needed to compare different states (e.g., future states with present states, states determined by different scenarios or states of different regional sub-systems). In the case ofATEAM, the elements of the set of states X are vectors, so it is not trivial to provide an appropriate order relation.<br>[...]				

**Structured summary of operationalization – validity assessment**			**1.1 DCM Appropriate**	**1.2 valid empirical rep?**	**1. conclusion - Valid?**	**2. Feasible?**
**Construct:** Stimulus						
**Article:** Ionesco et al (2009)						
Criterion	Assessment	Quoted text or Rationale for negative assessment				
Construct defined?	Yes	The stimuli to which such a system can be subjected are then naturally represented by the inputs to the system. The simplest kind of dynamical system with input is a discrete, deter- ministic one, given by a transition function (see [14]):<br>f : X × E→ X, (1)	Yes/ no/ can't tell	Yes/ no/ can't tell	Yes/ no/ can't tell<br>CAN'T TELL	Yes/ no/ can't tell
Data collection methods reported?	DINAS: Yes	The objective of this section is to relate the framework developed in Section 3 to the IPCCconceptualisation of vulnerability (see Fig. 1) and to two recent vulnerability	CAN'T TELL			

		assessments:Advanced Terrestrial EcosystemAnalysis and Modelling (ATEAM) and Dynamic and Inter- active Assessment of National, Regional and Global Vulnerability of Coastal Zones to Climate Change and Sea-Level Rise (DINAS-COAST).<br>[...]<br>4.3 Dynamic and InteractiveAssessment of National, Regional and Global Vulnerability of Coastal Zones to Climate Change and Sea-Level Rise<br>[...]<br>The project DINAS-COAST (http://www.dinas-coast. net) was also funded by the Research Directorate- General of the European Commission from 2001 to 2004. Five partners and two subcontractors worked together to develop the dynamic, interactive and flexi- ble tool Dynamic and Interactive VulnerabilityAssess- ment (DIVA, [5]). DIVA enables its users to assess coastal vulnerability to sea-level rise and to explore possible adaptation policies.				
Reporting of indicators/questions used to operationalise construct?	DINAS: 2ndary data			-		
Sampling strategies reported?	DINAS: 2ndary data					
Sampling sizes reported?	DINAS: 2ndary data					
Data analysis methods reported?	DINAS: Yes	4.3 Dynamic and InteractiveAssessment of National, Regional and Global Vulnerability of Coastal Zones to Climate Change and Sea-Level Rise<br>[...]<br>The second primitive, the stimulus or input to which the entity's vulnerability was assessed, was given in the form				

		of climate, land-use and socio-economic scenarios				

Transparency Assessment Article summary	
**Article**	Jamir et al (2013)
**Transparent operationalizations**	Agricultural; biophysical; demographic; socio-economic
**Partially transparent**	
**Not transparent**	Adaptive capacity ; Drought; Exposure; Sensitivity

<table>
<tr><td colspan="3">Structured summary of operationalization – validity assessment</td><td rowspan="4">1.1 DCM Appropriate</td><td rowspan="4">1.2 valid empirical rep?</td><td rowspan="4">1. conclusion - Valid?</td><td rowspan="4">2. Feasible?</td></tr>
<tr><td colspan="3">Construct: Agricultural</td></tr>
<tr><td colspan="3">Article: Jamir et al (2013)</td></tr>
<tr><td><u>Criterion</u></td><td><u>Assessment</u></td><td><u>Quoted text or Rationale for negative assessment</u></td></tr>
<tr><td>Construct defined?</td><td>Defined by reference</td><td>On the lines of Patnaik and Narayanan (2009),</td><td>Yes/ no/ can't tell</td><td>Yes/ no/ can't tell</td><td rowspan="3">Yes/ no/ can't tell
YES</td><td rowspan="3">Yes/ no/ can't tell
YES</td></tr>
<tr><td>Data collection methods reported?</td><td>Yes</td><td>Household questionnaire surveys and participatory rural appraisal (PRA) were conducted in all the five villages in order to quantify each of these indicators. A total of 150 households (30 households in each village) were randomly selected across the villages for the household questionnaire survey. The PRA was in the form of focus group discussions and semi-structured interviews. The group discussions with the community, village council members and district offi- cials gave an insight into the local problems.
[...]
Based on the response of the farmers and the village council members during household surveys and PRA, the mean, minimum and maximum values for each of the indicators were obtained. Secondary data were used for those indicators that could not be quantified by this approach.</td><td>YES</td><td></td></tr>
<tr><td>Reporting of indicators/questions used to operationalise</td><td>Yes</td><td>Table 3 Indicators of sources of vulnerability<table><tr><td>Component indicators</td><td>Indicator description (Table 2)</td><td>Indicator units (Table 2)</td></tr></table></td><td></td><td>YES</td></tr>
</table>

construct?			Area under shifting cultivation	Total area under shifting cultivation with less fallow periods (2–4 years)	Area (ha/ acre/ local unit)					
			Total area under rainfed agriculture	The total area cultivated by the farmers, which is dependent directly on rainfall for irrigation (whether under settled or shifting cultivation)	Area (ha/ acre/ local unit)					
			Total area under irrigated crops	The total agriculture area under manually irrigated crops (during kharif as well as rabi seasons)	Area (ha/ acre/ local unit)					
			Irrigation availability	Total number of days irrigation available per year	Days/year					
			Average crop diversity index	The inverse of (the number of crops grown by a household ?1)	Number					
			Total number of kharif crops grown	Total number of crops grown during kharif or rainfed season	Number					

			of the year					
		Total number of rabi crops grown	Total number of crops grown during rabi and zaid seasons of the year (non-major cropping seasons)	Number				
		Total annual crop production	Total annual crop production in the village of major crops including kharif and rabi crops	Tons/year				
		Extent of dryland	Ratio of the dryland area or non-irrigated agricultural land to the total geographical area of the village	Number				
		Crop area affected	Total area under cultivation affected by droughts	Area (ha/ acre/local unit)				
		Value of crops lost	The type and amount of crop sown and its market price during the time of crop loss taken as proxy	INR				
Sampling strategies reported?	Yes	Household questionnaire surveys and participatory rural appraisal (PRA) were conducted in all the five villages in						

		order to quantify each of these indicators. A total of 150 households (30 households in each village) were randomly selected across the villages for the household questionnaire survey.				
Sampling sizes reported?	Yes	Household questionnaire surveys and participatory rural appraisal (PRA) were conducted in all the five villages in order to quantify each of these indicators. A total of 150 households (30 households in each village) were randomly selected across the villages for the household questionnaire survey.				
Data analysis methods reported?	Yes	Based on the data obtained from the household survey and the PRA, four indices, corresponding to the four sources of vulnerability—biophysical, agricultural, demographic and socio-economic, were computed for all the villages. Data gaps were bridged by using secondary data, where avail- able. Table 4 shows the average biophysical, agricultural, demographic and socio-economic vulnerability indices for these villages.<br>Following the calculation of the indices, they were assigned weights based on the ranks given to the sources of vulnerability by the farmers themselves during the PRA. A rank of 4 indicates very high contribution to vulnerability. Ranks 3, 2 and 1 indicate high, moderate and low contri- bution of a particular source to overall vulnerability, respectively. The final weight to be apportioned was cal- culated by the following formula:<br>Average of the ranks assigned in the five villages Sumof the ranks<br>Table 5 shows the weights apportioned to the average vulnerability indices for calculatingvillage-levelvulnerability.<br>After weights were assigned to the biophysical, agri-cultural, socio-economic and demographic indices, a composite vulnerability index, representative of the climate variability-induced drought vulnerability of the resident farmers, was calculated (as per Patnaik and Narayanan 2009). The weighted biophysical, agricultural and socio- economic vulnerability indices of the villages				

		were calcu- lated by multiplying the apportioned weight by the average index calculated for each source of vulnerability.<br>[...]<br>The composite vulnerability indices for each of the villages were calculated using the following formula: V ¼ hi1=4 4<br>Pn i¼1 ðAverage indexi WeightiÞ				

**Structured summary of operationalization – validity assessment**			**1.1 DCM Appropriate**	**1.2 valid empirical rep?**	**1. conclusion - Valid?**	**2. Feasible?**
**Construct:** Biophysical						
**Article:** Jamir et al (2013)						
Criterion	Assessment	Quoted text or Rationale for negative assessment				
Construct defined?	Defined by reference	On the lines of Patnaik and Narayanan (2009),	Yes/ no/ can't tell	Yes/ no/ can't tell	Yes/ no/ can't tell<br>YES	Yes/ no/ can't tell<br>YES
Data collection methods reported?	Yes	Household questionnaire surveys and participatory rural appraisal (PRA) were conducted in all the five villages in order to quantify each of these indicators. A total of 150 households (30 households in each village) were randomly selected across the villages for the household questionnaire survey. The PRA was in the form of focus group discussions and semi-structured interviews. The group discussions with the community, village council members and district offi- cials gave an insight into the local problems.<br>[...]<br>Based on the response of the farmers and the village council members during household surveys and PRA, the mean, minimum and maximum values for each of the indicators were obtained. Secondary data were used for those indicators that could not be quantified by this approach.	YES			
Reporting of indicators/questions used to	Yes/no	able 3 Indicators of sources of vulnerability<br>Component indicators | Indicator description | Indicator units (Table 2)		YES		

<table>
<tr><td>operationalise construct?</td><td></td><td><table>
<tr><td></td><td>(Table 2)</td><td></td></tr>
<tr><td>Extreme climate events</td><td>Number of years experiencing rainfall deficit or droughts taken as a proxy</td><td>Number</td></tr>
<tr><td>Drought duration</td><td>Total amount of time the drought-like conditions persist in the village</td><td>Months</td></tr>
<tr><td>Drinking water availability</td><td>Approximate amount of drinking water available during droughts irrespective of source</td><td>Liters/ individual</td></tr>
</table></td><td></td><td></td><td></td><td></td></tr>
<tr><td>Sampling strategies reported?</td><td>Yes</td><td>Household questionnaire surveys and participatory rural appraisal (PRA) were conducted in all the five villages in order to quantify each of these indicators. A total of 150 households (30 households in each village) were randomly selected across the villages for the household questionnaire survey.</td><td></td><td></td><td></td><td></td></tr>
<tr><td>Sampling sizes reported?</td><td>Yes</td><td>Household questionnaire surveys and participatory rural appraisal (PRA) were conducted in all the five villages in order to quantify each of these indicators. A total of 150 households (30 households in each village) were randomly selected across the villages for the household questionnaire survey.</td><td></td><td></td><td></td><td></td></tr>
<tr><td>Data analysis methods reported?</td><td>Yes</td><td>Based on the data obtained from the household survey and the PRA, four indices, corresponding to the four sources of vulnerability—biophysical, agricultural, demographic and socio-economic, were computed for all the villages. Data gaps were bridged by using secondary</td><td></td><td></td><td></td><td></td></tr>
</table>

		data, where avail- able. Table 4 shows the average biophysical, agricultural, demographic and socio-economic vulnerability indices for these villages.<br>Following the calculation of the indices, they were assigned weights based on the ranks given to the sources of vulnerability by the farmers themselves during the PRA. A rank of 4 indicates very high contribution to vulnerability. Ranks 3, 2 and 1 indicate high, moderate and low contri- bution of a particular source to overall vulnerability, respectively. The final weight to be apportioned was cal- culated by the following formula:<br>Average of the ranks assigned in the five villages Sumof the ranks<br>Table 5 shows the weights apportioned to the average vulnerability indices for calculatingvillage-levelvulnerability.<br>After weights were assigned to the biophysical, agri- cultural, socio-economic and demographic indices, a composite vulnerability index, representative of the climate variability-induced drought vulnerability of the resident farmers, was calculated (as per Patnaik and Narayanan 2009). The weighted biophysical, agricultural and socio- economic vulnerability indices of the villages were calcu- lated by multiplying the apportioned weight by the average index calculated for each source of vulnerability.<br>[...]<br>The composite vulnerability indices for each of the villages were calculated using the following formula: V ¼ hi1=4 4<br>Pn i¼1 ðAverage indexi WeightiÞ				

Structured summary of operationalization – validity assessment	1.1 DCM Appropriate	1.2 valid empirical rep?	1. conclusion - Valid?	2. Feasible?
**Construct:** Demographic				
**Article:** Jamir et al (2013)				

<table>
<tr><td><u>Criterion</u></td><td><u>Assessment</u></td><td><u>Quoted text or Rationale for negative assessment</u></td><td></td><td></td><td></td><td></td></tr>
<tr><td>Construct defined?</td><td>Defined by reference</td><td>On the lines of Patnaik and Narayanan (2009),</td><td>Yes/ no/ can’t tell</td><td>Yes/ no/ can’t tell</td><td>Yes/ no/ can’t tell</td><td>Yes/ no/ can’t tell</td></tr>
<tr><td>Data collection methods reported?</td><td>Yes</td><td>Household questionnaire surveys and participatory rural appraisal (PRA) were conducted in all the five villages in order to quantify each of these indicators. A total of 150 households (30 households in each village) were randomly selected across the villages for the household questionnaire survey. The PRA was in the form of focus group discussions and semi-structured interviews. The group discussions with the community, village council members and district offi- cials gave an insight into the local problems.
[...]
Based on the response of the farmers and the village council members during household surveys and PRA, the mean, minimum and maximum values for each of the indicators were obtained. Secondary data were used for those indicators that could not be quantified by this approach.</td><td>YES</td><td></td><td rowspan="2">YESS</td><td rowspan="2">YESS</td></tr>
<tr><td>Reporting of indicators/questions used to operationalise construct?</td><td>Yes</td><td>Table 3 Indicators of sources of vulnerability<table>
<tr><td>Component indicators</td><td>Indicator description (Table 2)</td><td>Indicator units (Table 2)</td></tr>
<tr><td>Rural population density</td><td>Total rural population of the village divided by the geographical area of the village</td><td>Percentage</td></tr>
<tr><td>Percentage of small- scale farmers</td><td>Percentage of small-scale farmers (with land holding between 1.0 and 1.99 ha)</td><td>Percentage</td></tr>
<tr><td>Percentage of</td><td>Percentage of</td><td>Percentage</td></tr>
</table></td><td></td><td>YES</td></tr>
</table>

<table>
<tr><td></td><td></td><td><table><tr><td>marginal farmers</td><td>marginal farmers (with land holding\1ha)</td><td></td></tr><tr><td>Literacy rate</td><td>Percentage of literate members in the household</td><td>Percentage</td></tr></table></td><td></td><td></td><td></td><td></td></tr>
<tr><td>Sampling strategies reported?</td><td>Yes</td><td>Household questionnaire surveys and participatory rural appraisal (PRA) were conducted in all the five villages in order to quantify each of these indicators. A total of 150 households (30 households in each village) were randomly selected across the villages for the household questionnaire survey.</td><td></td><td></td><td></td><td></td></tr>
<tr><td>Sampling sizes reported?</td><td>Yes</td><td>Household questionnaire surveys and participatory rural appraisal (PRA) were conducted in all the five villages in order to quantify each of these indicators. A total of 150 households (30 households in each village) were randomly selected across the villages for the household questionnaire survey.</td><td></td><td></td><td></td><td></td></tr>
<tr><td>Data analysis methods reported?</td><td>Yes</td><td>Based on the data obtained from the household survey and the PRA, four indices, corresponding to the four sources of vulnerability—biophysical, agricultural, demographic and socio-economic, were computed for all the villages. Data gaps were bridged by using secondary data, where avail- able. Table 4 shows the average biophysical, agricultural, demographic and socio-economic vulnerability indices for these villages. Following the calculation of the indices, they were assigned weights based on the ranks given to the sources of vulnerability by the farmers themselves during the PRA. A rank of 4 indicates very high contribution to vulnerability. Ranks 3, 2 and 1 indicate high, moderate and low contri- bution of a particular source to overall vulnerability, respectively. The final weight to be apportioned was cal- culated by the following formula: Average of the ranks assigned in the five villages Sumof the ranks</td><td></td><td></td><td></td><td></td></tr>
</table>

		Table 5 shows the weights apportioned to the average vulnerability indices for calculatingvillage-levelvulnerability.<br>After weights were assigned to the biophysical, agri- cultural, socio-economic and demographic indices, a composite vulnerability index, representative of the climate variability-induced drought vulnerability of the resident farmers, was calculated (as per Patnaik and Narayanan 2009). The weighted biophysical, agricultural and socio- economic vulnerability indices of the villages were calcu- lated by multiplying the apportioned weight by the average index calculated for each source of vulnerability.<br>[...]<br>The composite vulnerability indices for each of the villages were calculated using the following formula: V ¼ hi1=4 4<br>Pn i¼1 ðAverage indexi ⍰WeightiÞ				

**Structured summary of operationalization – validity assessment**			**1.1 DCM Appropriate**	**1.2 valid empirical rep?**	**1. conclusion - Valid?**	**2. Feasible?**
**Construct:** Socio-economic						
**Article:** Jamir et al (2013)						
<u>Criterion</u>	<u>Assessment</u>	<u>Quoted text or Rationale for negative assessment</u>				
Construct defined?	Defined by reference	On the lines of Patnaik and Narayanan (2009),	Yes/ no/ can't tell	Yes/ no/ can't tell	Yes/ no/ can't tell	Yes/ no/ can't tell
Data collection methods reported?	Yes	Household questionnaire surveys and participatory rural appraisal (PRA) were conducted in all the five villages in order to quantify each of these indicators. A total of 150 households (30 households in each village) were randomly selected across the villages for the household questionnaire survey. The PRA was in the form of focus group discussions and semi-structured interviews. The group discussions with the community, village council	YES		YES	YES

<table>
<tr>
<td></td>
<td></td>
<td>members and district offi- cials gave an insight into the local problems.
[...]
Based on the response of the farmers and the village council members during household surveys and PRA, the mean, minimum and maximum values for each of the indicators were obtained. Secondary data were used for those indicators that could not be quantified by this approach.</td>
<td></td>
<td></td>
<td></td>
<td></td>
</tr>
<tr>
<td>Reporting of indicators/questions used to operationalise construct?</td>
<td>Yes</td>
<td>Table 3 Indicators of sources of vulnerability
<table>
<tr><td>Component indicators</td><td>Indicator description (Table 2)</td><td>Indicator units (Table 2)</td></tr>
<tr><td>Net Farm income</td><td>Total amount of farm income from the agricultural activities carried out by the farmer</td><td>INR</td></tr>
<tr><td>Average Farm size</td><td>Total size of the farm used for cultivation by the farmers</td><td>Area (ha/ acre/local unit)</td></tr>
<tr><td>Farm assets</td><td>Total number of tractors, farm equipments, storage facility, manure and pesticides used by the farmer</td><td>Number</td></tr>
<tr><td>Access to market</td><td>The distance travelled by the farmers to the village or town markets to sell their farm products and procure farm inputs on their</td><td>Distance (km)</td></tr>
</table>
</td>
<td></td>
<td>YES</td>
<td></td>
<td></td>
</tr>
</table>

			own or through some intermediaries.					
		Access to health facilities	Distance travelled by the farmers to reach the nearest dispensary/public health centre or hospital	Distance (km)				
		Access to bank	Percentage of farmers having an account in the nearest rural banks	Distance (km)				
		Alternative livelihood options	Sub-indicators addressing alternate means of earning livelihood (other than crop cultivation, etc.) such as dependence on forests, livestock, etc.					
		Awareness of drought preparedness and mitigation measures	Percentage of households having access to newspapers, radio, television, drought awareness programs, etc. taken as proxy	Percentage				
		Compensation received from Government due to losses	Total amount of compensation received by the drought-affected	INR				

		incurred during a drought/famine | farmers from the Government agencies, private donor organizations or NGOs |				
Sampling strategies reported?	Yes	Household questionnaire surveys and participatory rural appraisal (PRA) were conducted in all the five villages in order to quantify each of these indicators. A total of 150 households (30 households in each village) were randomly selected across the villages for the household questionnaire survey.				
Sampling sizes reported?	Yes	Household questionnaire surveys and participatory rural appraisal (PRA) were conducted in all the five villages in order to quantify each of these indicators. A total of 150 households (30 households in each village) were randomly selected across the villages for the household questionnaire survey.				
Data analysis methods reported?	Yes	Based on the data obtained from the household survey and the PRA, four indices, corresponding to the four sources of vulnerability—biophysical, agricultural, demographic and socio-economic, were computed for all the villages. Data gaps were bridged by using secondary data, where avail- able. Table 4 shows the average biophysical, agricultural, demographic and socio-economic vulnerability indices for these villages. Following the calculation of the indices, they were assigned weights based on the ranks given to the sources of vulnerability by the farmers themselves during the PRA. A rank of 4 indicates very high contribution to vulnerability. Ranks 3, 2 and 1 indicate high, moderate and low contri- bution of a particular source to overall vulnerability, respectively. The final weight to be apportioned was cal- culated by the following formula: Average of the ranks assigned in the five villages Sumof the ranks<br>Table 5 shows the weights apportioned to the average vulnerability indices for calculatingvillage-				

		levelvulnerability.<br>After weights were assigned to the biophysical, agri- cultural, socio-economic and demographic indices, a composite vulnerability index, representative of the climate variability-induced drought vulnerability of the resident farmers, was calculated (as per Patnaik and Narayanan 2009). The weighted biophysical, agricultural and socio- economic vulnerability indices of the villages were calcu- lated by multiplying the apportioned weight by the average index calculated for each source of vulnerability.<br>[...]<br>The composite vulnerability indices for each of the villages were calculated using the following formula: V ¼ hi1=4 4<br>Pn i¼1 ðAverage indexi Weighti Þ				

Transparency Assessment Article summary	
**Article**	Luers et al (2003)
**Transparent operationalizations**	Adaptive capacity; exposure; sensitivity; State of system relative to threshold of damage; threshold of damage; Well-being
**Partially transparent**	
**Not transparent**	

**Structured summary of operationalization – validity assessment**			**1.1 DCM Appropriate**	**1.2 valid empirical rep?**	**1. conclusion - Valid?**	**2. Feasible?**
**Construct:** Adaptive capacity						
**Article:** Luers et al (2003)						
Criterion	Assessment	Quoted text or Rationale for negative assessment				
Construct defined?	Yes	We define adaptive capacityas the extent to which a system can modify its circumstances to move to a less vulnerable condition (Fig. 1c). We quantifyadaptive capacity(A) as the difference in the vulnerabilityunder existing conditions and under the less vulnerable condition to which the system could potentially shift: A ¼ Vðexisting conditionsÞ� Vðmodified conditionsÞ:	Yes/ no/ can't tell	Yes/ no/ can't tell	Yes/ no/ can't tell NO	Yes/ no/ can't tell
Data collection methods reported?	Yes (2ndary data)	To illustrate an application of the proposed metric, we utilize remotely sensed estimates of yields in the Yaqui Valley for four years: 1994, 2000, 2001, and 2002. Yield estimates are derived from Landsat TM and ETM+ data, as described in detail by Lobell et al. (2003). [...] For each of the four years, we compute the distribution of yield within the entire Valley, and then rank yields by percentile for each year. We then use a linear least-squares regression of yield with average night-time temperature for January–April to define the average yield and sensitivity for each percentile.	CAN'T TELL			
Reporting of indicators/questions used to operationalise construct?	Yes (2ndary data)	To illustrate an application of the proposed metric, we utilize remotely sensed estimates of yields in the Yaqui Valley for four years: 1994, 2000, 2001, and 2002. Yield estimates are derived from Landsat TM and ETM+ data, as described in detail by Lobell et al. (2003).		NO		

		[...]<br>For each of the four years, we compute the distribution of yield within the entire Valley, and then rank yields by percentile for each year. We then use a linear least-squares regression of yield with average night-time temperature for January–April to define the average yield and sensitivity for each percentile.				
Sampling strategies reported?	Yes (2ndary data)	To illustrate an application of the proposed metric, we utilize remotely sensed estimates of yields in the Yaqui Valley for four years: 1994, 2000, 2001, and 2002. Yield estimates are derived from Landsat TM and ETM+ data, as described in detail by Lobell et al. (2003).<br>[...]<br>For each of the four years, we compute the distribution of yield within the entire Valley, and then rank yields by percentile for each year. We then use a linear least-squares regression of yield with average night-time temperature for January–April to define the average yield and sensitivity for each percentile.				
Sampling sizes reported?	Yes (2ndary data)	To illustrate an application of the proposed metric, we utilize remotely sensed estimates of yields in the Yaqui Valley for four years: 1994, 2000, 2001, and 2002. Yield estimates are derived from Landsat TM and ETM+ data, as described in detail by Lobell et al. (2003).<br>[...]<br>For each of the four years, we compute the distribution of yield within the entire Valley, and then rank yields by percentile for each year. We then use a linear least-squares regression of yield with average night-time temperature for January–April to define the average yield and sensitivity for each percentile.				
Data analysis methods reported?	Yes/no	For each of the four years, we compute the distribu-tion of yield within the entire Valley, and then rank yields by percentile for each year. We then use a linear least-squares regression of yield with average night-time temperature for January–April to define the average yield and sensitivity for each percentile. To define the vulnerabilitycorresponding to each percentile, we run a				

		Monte Carlo simulation where temperature varies according to a normal distribution with mean equal to 9.61□C and standard deviation equal to 0.99□C, as determined from 20 years of historical climate records. We then calculate the vulnerabilityaccording to Eq. (2) using a threshold value of 4 t/ha, which is the approximate minimum yield required for farmer's to ''break-even'' (i.e. zero net profit) based on the average management practices (Matson et al. 1998). We normal-ize these vulnerabilityvalues bythe average vulner-abilitycalculated for the entire Valley.<br>[...]<br>V ¼ Expected Value ðsensitivity=state relative to a thresholdÞ;<br>V ¼ Z jqW=qXj W=W0<br>□<br>where W0 represents a threshold value of well-being below which the system is said to be damaged.<br>where PX refers to the probabilityof the occurrence of stressor X:<br>[...]<br>Management is the onlyone of these factors that farmers can potentiallymanipulate to move to a less vulnerable condition. Therefore, in our analysis we estimate adaptive capacity from our time series of yields as the extent to which a farm unit has exceeded its average management percentile over the studyperiod. We assumed that the highest relative yield, as represented bythe yield percentile, could be achieved everyyear with the appropriate management. We estimate the adaptive capacityas the difference between the vulnerabilitycalculated as above and the vulner-abilitycalculated for a yield temperature function where we assume the expected yield is equal to the maximum yield percentiles observed over the four years. To create a unitless measure we normalize this difference bythe average value of the difference calculated for all pixels over the Valley:				

		A ¼ ðVR mean □VR maxÞpixel□i ðVR mean □VR maxÞvalleyave<br>;<br>where R refers to the relative yield percentile.				

**Structured summary of operationalization – validity assessment**			**1.1 DCM Appropriate**	**1.2 valid empirical rep?**	**1. conclusion - Valid?**	**2. Feasible?**
**Construct:** Exposure						
**Article:** Luers et al (2003)						
Criterion	Assessment	Quoted text or Rationale for negative assessment				
Construct defined?	Yes	Different communities and ecosystems are exposed to varying magnitudes and frequencies of disturbing forces, often resulting in differential vulnerabilities (IPCC, 2001; Turner et al., 2003a, b). We capture these differences in exposure bycalculating the expected value of the ratio of sensitivityto the state relative to a threshold based on the frequencydistribution of the stressors of concern:	Yes/ no/ can't tell	Yes/ no/ can't tell	Yes/ no/ can't tell<br>NO	Yes/ no/ can't tell
Data collection methods reported?	Yes (2ndary data)	To illustrate an application of the proposed metric, we utilize remotely sensed estimates of yields in the Yaqui Valley for four years: 1994, 2000, 2001, and 2002. Yield estimates are derived from Landsat TM and ETM+ data, as described in detail by Lobell et al. (2003).<br>[...]<br>For each of the four years, we compute the distribution of yield within the entire Valley, and then rank yields by percentile for each year. We then use a linear least-squares regression of yield with average night-time temperature for January–April to define the average yield and sensitivity for each percentile.	CAN'T TELL			
Reporting of indicators/questions used to operationalise construct?	Yes (2ndary data)	To illustrate an application of the proposed metric, we utilize remotely sensed estimates of yields in the Yaqui Valley for four years: 1994, 2000, 2001, and 2002. Yield estimates are derived from Landsat TM and ETM+ data, as described in detail by Lobell et al. (2003).<br>[...]<br>For each of the four years, we compute the distribution of		NO		

		yield within the entire Valley, and then rank yields by percentile for each year. We then use a linear least-squares regression of yield with average night-time temperature for January–April to define the average yield and sensitivity for each percentile.				
Sampling strategies reported?	Yes (2ndary data)	To illustrate an application of the proposed metric, we utilize remotely sensed estimates of yields in the Yaqui Valley for four years: 1994, 2000, 2001, and 2002. Yield estimates are derived from Landsat TM and ETM+ data, as described in detail by Lobell et al. (2003).<br>[...]<br>For each of the four years, we compute the distribution of yield within the entire Valley, and then rank yields by percentile for each year. We then use a linear least-squares regression of yield with average night-time temperature for January–April to define the average yield and sensitivity for each percentile.				
Sampling sizes reported?	Yes (2ndary data)	To illustrate an application of the proposed metric, we utilize remotely sensed estimates of yields in the Yaqui Valley for four years: 1994, 2000, 2001, and 2002. Yield estimates are derived from Landsat TM and ETM+ data, as described in detail by Lobell et al. (2003).<br>[...]<br>For each of the four years, we compute the distribution of yield within the entire Valley, and then rank yields by percentile for each year. We then use a linear least-squares regression of yield with average night-time temperature for January–April to define the average yield and sensitivity for each percentile.				
Data analysis methods reported?	Yes	For each of the four years, we compute the distribution of yield within the entire Valley, and then rank yields by percentile for each year. We then use a linear least-squares regression of yield with average night-time temperature for January–April to define the average yield and sensitivity for each percentile. To define the vulnerabilitycorresponding to each percentile, we run a Monte Carlo simulation where temperature varies according to a normal distribution with mean equal to				

		9.61C and standard deviation equal to 0.99C, as determined from 20 years of historical climate records. We then calculate the vulnerabilityaccording to Eq. (2) using a threshold value of 4 t/ha, which is the approximate minimum yield required for farmer's to ''break-even'' (i.e. zero net profit) based on the average management practices (Matson et al. 1998). We normal-ize these vulnerabilityvalues bythe average vulner-abilitycalculated for the entire Valley.<br>[...]<br>V ¼ Expected Value ðsensitivity=state relative to a thresholdÞ;<br>V ¼ Z jqW=qXj W=W0<br><br>where W0 represents a threshold value of well-being below which the system is said to be damaged.<br>where PX refers to the probabilityof the occurrence of stressor X:				

**Structured summary of operationalization – validity assessment**			**1.1 DCM Appropriate**	**1.2 valid empirical rep?**	**1. conclusion - Valid?**	**2. Feasible?**
**Construct:** Sensitivity						
**Article:** Luers et al (2003)						
Criterion	Assessment	Quoted text or Rationale for negative assessment				
Construct defined?	Yes	In this example, the sensitivityis represented as the absolute value of the derivative of well-being with respect to the stressor, however, other measures of sensitivitycould be used, for example the coefficient of variations.	Yes/ no/ can't tell	Yes/ no/ can't tell	Yes/ no/ can't tell<br>NO	Yes/ no/ can't tell
Data collection methods reported?	Yes (2ndary data)	To illustrate an application of the proposed metric,<br>we utilize remotely sensed estimates of yields in the Yaqui Valley for four years: 1994, 2000, 2001, and 2002. Yield estimates are derived from Landsat TM and ETM+ data, as described in detail by Lobell et al. (2003).<br>[...]	CAN'T TELL			

		For each of the four years, we compute the distribution of yield within the entire Valley, and then rank yields by percentile for each year. We then use a linear least-squares regression of yield with average night-time temperature for January–April to define the average yield and sensitivity for each percentile.				
Reporting of indicators/questions used to operationalise construct?	Yes (2ndary data)	To illustrate an application of the proposed metric, we utilize remotely sensed estimates of yields in the Yaqui Valley for four years: 1994, 2000, 2001, and 2002. Yield estimates are derived from Landsat TM and ETM+ data, as described in detail by Lobell et al. (2003). [...] For each of the four years, we compute the distribution of yield within the entire Valley, and then rank yields by percentile for each year. We then use a linear least-squares regression of yield with average night-time temperature for January–April to define the average yield and sensitivity for each percentile.		NO		
Sampling strategies reported?	Yes (2ndary data)	To illustrate an application of the proposed metric, we utilize remotely sensed estimates of yields in the Yaqui Valley for four years: 1994, 2000, 2001, and 2002. Yield estimates are derived from Landsat TM and ETM+ data, as described in detail by Lobell et al. (2003). [...] For each of the four years, we compute the distribution of yield within the entire Valley, and then rank yields by percentile for each year. We then use a linear least-squares regression of yield with average night-time temperature for January–April to define the average yield and sensitivity for each percentile.				
Sampling sizes reported?	Yes (2ndary data)	To illustrate an application of the proposed metric, we utilize remotely sensed estimates of yields in the Yaqui Valley for four years: 1994, 2000, 2001, and 2002. Yield estimates are derived from Landsat TM and ETM+ data, as described in detail by Lobell et al. (2003). [...] For each of the four years, we compute the distribution of yield within the entire Valley, and then rank yields by				

		percentile for each year. We then use a linear least-squares regression of yield with average night-time temperature for January–April to define the average yield and sensitivity for each percentile.				
Data analysis methods reported?	Yes	For each of the four years, we compute the distribu-tion of yield within the entire Valley, and then rank yields by percentile for each year. We then use a linear least-squares regression of yield with average night-time temperature for January–April to define the average yield and sensitivity for each percentile.				

**Structured summary of operationalization – validity assessment**			**1.1 DCM Appropriate**	**1.2 valid empirical rep?**	**1. conclusion - Valid?**	**2. Feasible?**
**Construct:** State of system relative to threshold of damage						
**Article:** Luers et al (2003)						
Criterion	Assessment	Quoted text or Rationale for negative assessment				
Construct defined?	Yes	identifying a threshold of human well- being at which the system is said to be ‘‘damaged.’’	Yes/ no/ can’t tell	Yes/ no/ can’t tell	Yes/ no/ can’t tell<br>NO	Yes/ no/ can’t tell
Data collection methods reported?	Yes	Our unit (or system) of analysis is the ‘‘farm unit’’—that is an agricultural field and the farmer or farmers responsible for the field. For practical purposes, we define our agricultural field as a 30m30m pixel as described below. Of the manyoutcomes of concern to the Valleyfarmer, we focus on wheat yield as our measure of well-being. Wheat yield alone obviously does not fullycapture the well-being of Valleyfarmers, however, we use it here to illustrate the proposed methodology.<br>[...]<br>To illustrate an application of the proposed metric, we utilize remotely sensed estimates of yields in the Yaqui Valley for four years: 1994, 2000, 2001, and 2002. Yield estimates are derived from Landsat TM and ETM+ data, as described in detail by Lobell et al. (2003).<br>[...]<br>For each of the four years, we compute the distribution of yield within the entire Valley, and then rank yields by	CAN’T TELL			

		percentile for each year. We then use a linear least-squares regression of yield with average average yield and sensitivity for each percentile.				
Reporting of indicators/questions used to operationalise construct?	Yes	Our unit (or system) of analysis is the ‘‘farm unit’’—that is an agricultural field and the farmer or farmers responsible for the field. For practical purposes, we define our agricultural field as a 30m30m pixel as described below. Of the manyoutcomes of concern to the Valleyfarmer, we focus on wheat yield as our measure of well-being. Wheat yield alone obviously does not fullycapture the well-being of Valleyfarmers, however, we use it here to illustrate the proposed methodology.<br>[...]<br>To illustrate an application of the proposed metric, we utilize remotely sensed estimates of yields in the Yaqui Valley for four years: 1994, 2000, 2001, and 2002. Yield estimates are derived from Landsat TM and ETM+ data, as described in detail by Lobell et al. (2003).<br>[...]<br>For each of the four years, we compute the distribution of yield within the entire Valley, and then rank yields by percentile for each year. We then use a linear least-squares regression of yield with average night-time temperature for January–April to define the average yield and sensitivity for each percentile.		NO		
Sampling strategies reported?	Yes (2ndary data)	To illustrate an application of the proposed metric, we utilize remotely sensed estimates of yields in the Yaqui Valley for four years: 1994, 2000, 2001, and 2002. Yield estimates are derived from Landsat TM and ETM+ data, as described in detail by Lobell et al. (2003).<br>[...]<br>For each of the four years, we compute the distribution of yield within the entire Valley, and then rank yields by percentile for each year. We then use a linear least-squares regression of yield with average night-time temperature for January–April to define the average yield and sensitivity for each percentile.				
Sampling sizes	Yes (2ndary	To illustrate an application of the proposed metric,				

reported?	data)	we utilize remotely sensed estimates of yields in the Yaqui Valley for four years: 1994, 2000, 2001, and 2002. Yield estimates are derived from Landsat TM and ETM+ data, as described in detail by Lobell et al. (2003).<br>[...]<br>For each of the four years, we compute the distribution of yield within the entire Valley, and then rank yields by percentile for each year. We then use a linear least-squares regression of yield with average night-time temperature for January–April to define the average yield and sensitivity for each percentile.				
Data analysis methods reported?	Yes	For each of the four years, we compute the distribu-tion of yield within the entire Valley, and then rank yields by percentile for each year. We then use a linear least-squares regression of yield with average night-time temperature for January–April to define the average yield and sensitivity for each percentile.<br>[...]<br>We then calculate the vulnerabilityaccording to Eq. (2) using a threshold value of 4 t/ha, which is the approximate minimum yield required for farmer's to ''break-even'' (i.e. zero net profit) based on the average management practices (Matson et al. 1998).				

**Structured summary of operationalization – validity assessment**			**1.1 DCM Appropriate**	**1.2 valid empirical rep?**	**1. conclusion - Valid?**	**2. Feasible?**
**Construct:** threshold of damage						
**Article:** Luers et al (2003)						
Criterion	Assessment	Quoted text or Rationale for negative assessment				
Construct defined?	Yes	W0 represents a threshold value of well-being below which the system is said to be damaged	Yes/ no/ can't tell	Yes/ no/ can't tell	Yes/ no/ can't tell<br>NO	Yes/ no/ can't tell
Data collection methods reported?	Yes (2ndary data)	To illustrate an application of the proposed metric, we utilize remotely sensed estimates of yields in the Yaqui Valley for four years: 1994, 2000, 2001, and 2002. Yield estimates are derived from Landsat TM and ETM+ data, as described in detail by Lobell et al. (2003).	CAN'T TELL			

		[...]<br>For each of the four years, we compute the distribution of yield within the entire Valley, and then rank yields by percentile for each year. We then use a linear least-squares regression of yield with average night-time temperature for January–April to define the average yield and sensitivity for each percentile.				
Reporting of indicators/questions used to operationalise construct?	Yes (2ndary data)	To illustrate an application of the proposed metric, we utilize remotely sensed estimates of yields in the Yaqui Valley for four years: 1994, 2000, 2001, and 2002. Yield estimates are derived from Landsat TM and ETM+ data, as described in detail by Lobell et al. (2003).<br>[...]<br>For each of the four years, we compute the distribution of yield within the entire Valley, and then rank yields by percentile for each year. We then use a linear least-squares regression of yield with average night-time temperature for January–April to define the average yield and sensitivity for each percentile.		NO		
Sampling strategies reported?	Yes (2ndary data)	To illustrate an application of the proposed metric, we utilize remotely sensed estimates of yields in the Yaqui Valley for four years: 1994, 2000, 2001, and 2002. Yield estimates are derived from Landsat TM and ETM+ data, as described in detail by Lobell et al. (2003).<br>[...]<br>For each of the four years, we compute the distribution of yield within the entire Valley, and then rank yields by percentile for each year. We then use a linear least-squares regression of yield with average night-time temperature for January–April to define the average yield and sensitivity for each percentile.				
Sampling sizes reported?	Yes (2ndary data)	To illustrate an application of the proposed metric, we utilize remotely sensed estimates of yields in the Yaqui Valley for four years: 1994, 2000, 2001, and 2002. Yield estimates are derived from Landsat TM and ETM+ data, as described in detail by Lobell et al. (2003).<br>[...]<br>For each of the four years, we compute the distribution of				

		yield within the entire Valley, and then rank yields by percentile for each year. We then use a linear least-squares regression of yield with average night-time temperature for January–April to define the average yield and sensitivity for each percentile.				
Data analysis methods reported?	Yes	using a threshold value of 4 t/ha, which is the approximate minimum yield required for farmer's to ''break-even'' (i.e. zero net profit) based on the average management practices (Matson et al. 1998).				

**Structured summary of operationalization – validity assessment**			**1.1 DCM Appropriate**	**1.2 valid empirical rep?**	**1. conclusion - Valid?**	**2. Feasible?**
**Construct:** Well-being						
**Article:** Luers et al (2003)						
Criterion	Assessment	Quoted text or Rationale for negative assessment				
Construct defined?	Yes	human–environment system where some mea- sure of human well-being (W)	Yes/ no/ can't tell	Yes/ no/ can't tell	Yes/ no/ can't tell<br>NO	Yes/ no/ can't tell
Data collection methods reported?	Yes (2ndary data)	To illustrate an application of the proposed metric, we utilize remotely sensed estimates of yields in the Yaqui Valley for four years: 1994, 2000, 2001, and 2002. Yield estimates are derived from Landsat TM and ETM+ data, as described in detail by Lobell et al. (2003).<br>[...]<br>For each of the four years, we compute the distribution of yield within the entire Valley, and then rank yields by percentile for each year. We then use a linear least-squares regression of yield with average night-time temperature for January–April to define the average yield and sensitivity for each percentile.	NO			
Reporting of indicators/questions used to operationalise construct?	Yes	Of the manyoutcomes of concern to the Valleyfarmer, we focus on wheat yield as our measure of well-being. Wheat yield alone obviously does not fullycapture the well-being of Valleyfarmers, however, we use it here to illustrate the proposed methodology.		NO		
Sampling strategies reported?	Yes (2ndary data)	To illustrate an application of the proposed metric, we utilize remotely sensed estimates of yields in the				

		Yaqui Valley for four years: 1994, 2000, 2001, and 2002. Yield estimates are derived from Landsat TM and ETM+ data, as described in detail by Lobell et al. (2003). [...] For each of the four years, we compute the distribution of yield within the entire Valley, and then rank yields by percentile for each year. We then use a linear least-squares regression of yield with average night-time temperature for January–April to define the average yield and sensitivity for each percentile.				
Sampling sizes reported?	Yes (2ndary data)	To illustrate an application of the proposed metric, we utilize remotely sensed estimates of yields in the Yaqui Valley for four years: 1994, 2000, 2001, and 2002. Yield estimates are derived from Landsat TM and ETM+ data, as described in detail by Lobell et al. (2003). [...] For each of the four years, we compute the distribution of yield within the entire Valley, and then rank yields by percentile for each year. We then use a linear least-squares regression of yield with average night-time temperature for January–April to define the average yield and sensitivity for each percentile.				
Data analysis methods reported?	Yes	For each of the four years, we compute the distribution of yield within the entire Valley, and then rank yields by percentile for each year.				

Transparency Assessment Article summary	
**Article**	Mengitsu (2011)
**Transparent operationalizations**	Perception of Adiha farmers
**Partially transparent**	
**Not transparent**	

**Structured summary of operationalization – validity assessment**			**1.1 DCM Appropriate**	**1.2 valid empirical rep?**	**1. conclusion - Valid?**	**2. Feasible?**
**Construct:** Perception of Adiha farmers						
**Article:** Mengitsu (2011)						
Criterion	Assessment	Quoted text or Rationale for negative assessment				
Construct defined?	Yes	Adaptation of people to different hazards vary from household to households and region to region based on existing support system to increase the resilience of affected individuals. The assessment was aimed to generate primary information from the farming communities of Adiha related to cli- mate change. This report examined the perception of Adiha farmers on the trend of climate change and re- lated anomalities, existing coping strategies in place.	Yes/ no/ can't tell	Yes/ no/ can't tell	Yes/ no/ can't tell YES	Yes/ no/ can't tell CAN'T TELL
Data collection methods reported?	Yes	2.2.1. Focus Group Discussion (FGD) Focus Group Discussion (FGD) was employed to generate information on the perception of the farmers on climate change, its related hazards, vulnerable groups of the community and existing coping strategies. Six FGDs, each consisting 24 participants, 12 male and 12 women, drawn from different kueshets, were held for climate re- lated hazard identification and characterization, identifi- cation and prioritization of coping mechanisms, identifi- cation and ranking of vulnerable groups and climate and weather forecasting.	YES			
Reporting of indicators/questions used to	Yes	Six FGDs, each consisting 24 participants, 12 male and 12 women, drawn from different kueshets, were held for climate re- lated hazard identification and		YES		

operationalise construct?		characterization, identifi- cation and prioritization of coping mechanisms, identifi- cation and ranking of vulnerable groups and climate and weather forecasting. Tools such as hazard identification and characterization, hazard behavior story telling (time- line), hazard ranking matrix, vulnerability group ranking and experiential stories telling on indigenous technolo- gies and knowledge were used to acquire information on farmers' perception on climate change trends, existing hazards and their severity and vulnerable groups of the community. The different coping strategies used by the community were also identified and analyzed for their effectiveness. Effectiveness was rated as very satisfac- tory, satisfactory and not satisfactory and the rating number converted to percent to assess satisfaction level.				
Sampling strategies reported?	Yes	Respondents were systematically sampled from Adiha tabia populations across all of the kueshets. One hundred forty four (144) respondents were sampled from popula- tion of the tabia. Various factors including gender (male/ female headed farm households), age, access to irriga- tion water and land holding size were considered during sampling.				
Sampling sizes reported?	Yes	One hundred forty four (144) respondents were sampled from popula- tion of the tabia.				
Data analysis methods reported?	Yes	Information was recorded using worksheets prepared for each category of discussion. Data collected on each parameter was expressed as percent of respondents. Farmer's perceptions on changes in long-term tempera- ture and precipitation as well as various coping strategies being used by farmers were analyzed and presented us- ing simple descriptive statistics (tables and figures).				

**Transparency Assessment Article summary**	
**Article**	Mubaya et al (2012)
**Transparent operationalizations**	Climate change; climate change and variability; climate variability; Farmer perceptions; non-climatic stress
**Partially transparent**	
**Not transparent**	

**Structured summary of operationalization – validity assessment**			**1.1 DCM Appropriate**	**1.2 valid empirical rep?**	**1. conclusion - Valid?**	**2. Feasible?**
**Construct:** Climate change						
**Article:** Mubaya et al (2012)						
Criterion	Assessment	Quoted text or Rationale for negative assessment				
Construct defined?	Yes	In this paper, the distinction between 'climate variability' and 'climate change' relates to differences in time-scale. On the one hand, 'climate variability' is conceptualised as variations in the climate system over short time scales such as months, years or decades and on the other hand 'climate change' is conceptualised as longer term trends in mean climate variables of periods of decades or longer. This is the suggested distinction in definitions of the concepts in question by the IPCC (2001).	Yes/ no/ can't tell	Yes/ no/ can't tell	Yes/ no/ can't tell NO	Yes/ no/ can't tell
Data collection methods reported?	Yes	The qualitative methods of data collection used include Participatory Rural Appraisal (PRA) techniques such as historical trend analysis and matrix scoring and ranking and Focus Group Discussions (FGDs). The quantitative method used is the household questionnaire survey.	NO			
Reporting of indicators/questions used to operationalise construct?	Yes	2.2.2. Qualitative assessments FGDs were used to first of all establish the general perceptions regarding climate change and variability and their causes and various stressors that confront farmers' livelihoods (see Appendix 2). Following this, it was considered important for this study to factor in how farmers regard climate change and variability as an obstacle to their livelihoods among the multiple stressors that they had		NO		

		identified. Among these stressors are climate variability in different forms, issues of financial capital, issues related to cattle pests and diseases, inadequate draught power, marketing issues and HIV and AIDS. A matrix scoring and ranking exercise was then facilitated for<br>farmers. Farmers were asked as a group to select from the long list of stressors the ones they considered critical for the purposes of scoring and ranking. The second step involved participants defining criteria that they would use to evaluate these stressors. These criteria include food security, income generation, crop production and livelihood security. Through group consensus, farmers then decided how much to allocate each shock out of a total of 20 points, based on the group defined criteria. Historical trend lines were used to elicit information on specific historical trends in farmers' perceptions regarding changes in climate over a period of 20 years and as far back as they could recall. Specifically, participants were asked to recall major occurrences that had a bearing on climate and weather, community resources, and even the political situation. They were then asked to indicate what occurrences had the greatest impact on their livelihoods among the cited events.<br>[...]<br>2.2.3. Quantitative assessments The questionnaire survey was used to collect household data<br>and complement data generated through the qualitative methods. This survey collected data on changes in crops grown over a period of five years and reasons for these changes, indicators for good and bad crop production seasons and years considered to be good or bad over a ten year period. Questions in the survey also related to changes in weather patterns over a ten year period in relation to agriculture and what might have caused these changes. General household characteristics were also captured in this survey (see Appendix 1).				
Sampling strategies reported?	Yes	A sample of 720 households across countries was selected for				

		the survey, 180 households per each of the four districts. Specifi- cally, systematic random sampling was employed to come up with six villages per district (making them 24 across countries) and 30 households per each of these villages, making a total of 380 households per country (this study was part of a big inter- institutional research-based development project). For FGDs and PRA workshops, a group of eight to 15 participants was selected to represent the three villages per district, with approximately five representatives from each of the three villages per district. In coming up with this group, factors such as age and gender were used. In terms of gender, separate PRA workshops were held for men and women in order not to compromise the amount and quality of information that can be generated from the less confident if they were to be combined. Specifically, old men and women were incorporated into the sample for the group discussions in order to capture information related to historical trends in climate. Itwas envisaged that they would be able to recall as far back as they could and provide rich information on these trends. In the same context, youths were incorporated into the sample in order to validate some of the recent trends on climate suggested by the elderly.				
Sampling sizes reported?	Yes	A sample of 720 households across countries was selected for<br>the survey, 180 households per each of the four districts. [...]<br>For FGDs and PRA workshops, a group of eight to 15 participants<br>was selected to represent the three villages per district, with approximately five representatives from each of the three villages per district.				
Data analysis methods reported?	Yes	Qualitative data were categorised and analysed in four distinct themes. These themes are<br>Perceptions regarding changes in weather patterns, Perceptions regarding causes of changes and variability in climate, Perceptions regarding other stressors among				

		farmers and Perceptions regarding climate change in relation to other stressors.<br>These perceptions were established in historical trend lines,<br>FGDs and matrix scoring and ranking and they are presented in this manner in the sections under results and discussion.<br>[...]<br>Data from the questionnaire survey were entered into the Statistical Package for the Social Sciences (SPSS) and analysed by running descriptive frequencies in relation to the distinct themes highlighted in this section. These themes include perceptions regarding changes in weather patterns in general and for specific seasons and regarding causes of these changes. These frequencies were disaggregated by district and country.				

**Structured summary of operationalization – validity assessment**			**1.1 DCM Appropriate**	**1.2 valid empirical rep?**	**1. conclusion - Valid?**	**2. Feasible?**
**Construct:** Climate change and variability						
**Article:** Mubaya et al (2012)						
Criterion	Assessment	Quoted text or Rationale for negative assessment				
Construct defined?	Yes	In this paper, the distinction between ‘climate variability’ and ‘climate change’ relates to differences in time-scale. On the one hand, ‘climate variability’ is conceptualised as variations in the climate system over short time scales such as months, years or decades and on the other hand ‘climate change’ is conceptualised as longer term trends in mean climate variables of periods of decades or longer. This is the suggested distinction in definitions of the concepts in question by the IPCC (2001).	Yes/ no/ can’t tell	Yes/ no/ can’t tell	Yes/ no/ can’t tell<br>NO	Yes/ no/ can’t tell
Data collection methods reported?	Yes	The qualitative methods of data collection used include Participatory Rural Appraisal (PRA) techniques such as historical trend analysis and matrix scoring and ranking and Focus Group Discussions (FGDs). The quantitative method used is the household questionnaire survey.	NO			

Reporting of indicators/questions used to operationalise construct?	Yes	2.2.2. Qualitative assessments FGDs were used to first of all establish the general perceptions<br>regarding climate change and variability and their causes and various stressors that confront farmers' livelihoods (see Appendix 2). Following this, it was considered important for this study to factor in how farmers regard climate change and variability as an obstacle to their livelihoods among the multiple stressors that they had identified. Among these stressors are climate variability in different forms, issues of financial capital, issues related to cattle pests and diseases, inadequate draught power, marketing issues and HIV and AIDS. A matrix scoring and ranking exercise was then facilitated for<br>farmers. Farmers were asked as a group to select from the long list of stressors the ones they considered critical for the purposes of scoring and ranking. The second step involved participants defining criteria that they would use to evaluate these stressors. These criteria include food security, income generation, crop production and livelihood security. Through group consensus, farmers then decided how much to allocate each shock out of a total of 20 points, based on the group defined criteria. Historical trend lines were used to elicit information on specific historical trends in farmers' perceptions regarding changes in climate over a period of 20 years and as far back as they could recall. Specifically, participants were asked to recall major occurrences that had a bearing on climate and weather, community resources, and even the political situation. They were then asked to indicate what occurrences had the greatest impact on their livelihoods among the cited events.<br>[...]<br>2.2.3. Quantitative assessments The questionnaire survey was used to collect household data<br>and complement data generated through the qualitative methods. This survey collected data on changes in crops grown over a period of five years and reasons for these changes, indicators for good and bad crop production		NO		

		seasons and years considered to be good or bad over a ten year period. Questions in the survey also related to changes in weather patterns over a ten year period in relation to agriculture and what might have caused these changes. General household characteristics were also captured in this survey (see Appendix 1).				
Sampling strategies reported?	Yes	A sample of 720 households across countries was selected for<br>the survey, 180 households per each of the four districts. Specifi- cally, systematic random sampling was employed to come up with six villages per district (making them 24 across countries) and 30 households per each of these villages, making a total of 380 households per country (this study was part of a big inter- institutional research-based development project). For FGDs and PRA workshops, a group of eight to 15 participants<br>was selected to represent the three villages per district, with approximately five representatives from each of the three villages per district. In coming up with this group, factors such as age and gender were used. In terms of gender, separate PRA workshops were held for men and women in order not to compromise the amount and quality of information that can be generated from the less confident if they were to be combined. Specifically, old men and women were incorporated into the sample for the group discussions in order to capture information related to historical trends in climate. Itwas envisaged that they would be able to recall as far back as they could and provide rich information on these trends. In the same context, youths were incorporated into the sample in order to validate some of the recent trends on climate suggested by the elderly.				
Sampling sizes reported?	Yes	A sample of 720 households across countries was selected for<br>the survey, 180 households per each of the four districts.<br>[...]<br>For FGDs and PRA workshops, a group of eight to 15 participants				

		was selected to represent the three villages per district, with approximately five representatives from each of the three villages per district.				
Data analysis methods reported?	Yes	Qualitative data were categorised and analysed in four distinct themes. These themes are<br>Perceptions regarding changes in weather patterns, Perceptions regarding causes of changes and variability in climate, Perceptions regarding other stressors among farmers and Perceptions regarding climate change in relation to other stressors.<br>These perceptions were established in historical trend lines,<br>FGDs and matrix scoring and ranking and they are presented in this manner in the sections under results and discussion.<br>[...]<br>Data from the questionnaire survey were entered into the Statistical Package for the Social Sciences (SPSS) and analysed by running descriptive frequencies in relation to the distinct themes highlighted in this section. These themes include perceptions regarding changes in weather patterns in general and for specific seasons and regarding causes of these changes. These frequencies were disaggregated by district and country.				

**Structured summary of operationalization – validity assessment**			**1.1 DCM Appropriate**	**1.2 valid empirical rep?**	**1. conclusion - Valid?**	**2. Feasible?**
**Construct:** Climate variability						
**Article:** Mubaya et al (2012)						
Criterion	Assessment	Quoted text or Rationale for negative assessment				
Construct defined?	Yes	In this paper, the distinction between ‘climate variability’ and ‘climate change’ relates to differences in time-scale. On the one hand, ‘climate variability’ is conceptualised as variations in the climate system over short time scales such as months, years or decades and on the other hand ‘climate change’ is conceptualised as longer term trends	Yes/ no/ can’t tell	Yes/ no/ can’t tell	Yes/ no/ can’t tell<br><br>NO	Yes/ no/ can’t tell

		in mean climate variables of periods of decades or longer. This is the suggested distinction in definitions of the concepts in question by the IPCC (2001).				
Data collection methods reported?	Yes	The qualitative methods of data collection used include Participatory Rural Appraisal (PRA) techniques such as historical trend analysis and matrix scoring and ranking and Focus Group Discussions (FGDs). The quantitative method used is the household questionnaire survey.	NO			
Reporting of indicators/questions used to operationalise construct?	Yes	2.2.2. Qualitative assessments FGDs were used to first of all establish the general perceptions regarding climate change and variability and their causes and various stressors that confront farmers' livelihoods (see Appendix 2). Following this, it was considered important for this study to factor in how farmers regard climate change and variability as an obstacle to their livelihoods among the multiple stressors that they had identified. Among these stressors are climate variability in different forms, issues of financial capital, issues related to cattle pests and diseases, inadequate draught power, marketing issues and HIV and AIDS. A matrix scoring and ranking exercise was then facilitated for farmers. Farmers were asked as a group to select from the long list of stressors the ones they considered critical for the purposes of scoring and ranking. The second step involved participants defining criteria that they would use to evaluate these stressors. These criteria include food security, income generation, crop production and livelihood security. Through group consensus, farmers then decided how much to allocate each shock out of a total of 20 points, based on the group defined criteria. Historical trend lines were used to elicit information on specific historical trends in farmers' perceptions regarding changes in climate over a period of 20 years and as far back as they could recall. Specifically, participants were asked to recall major occurrences that had a bearing on climate and weather, community resources, and even the political situation. They were then asked to indicate what occurrences had the greatest impact on their livelihoods		NO		

		among the cited events.<br>[...]<br>2.2.3. Quantitative assessments The questionnaire survey was used to collect household data<br>and complement data generated through the qualitative methods. This survey collected data on changes in crops grown over a period of five years and reasons for these changes, indicators for good and bad crop production seasons and years considered to be good or bad over a ten year period. Questions in the survey also related to changes in weather patterns over a ten year period in relation to agriculture and what might have caused these changes. General household characteristics were also captured in this survey (see Appendix 1).				
Sampling strategies reported?	Yes	A sample of 720 households across countries was selected for<br>the survey, 180 households per each of the four districts. Specifi- cally, systematic random sampling was employed to come up with six villages per district (making them 24 across countries) and 30 households per each of these villages, making a total of 380 households per country (this study was part of a big inter- institutional research-based development project). For FGDs and PRA workshops, a group of eight to 15 participants<br>was selected to represent the three villages per district, with approximately five representatives from each of the three villages per district. In coming up with this group, factors such as age and gender were used. In terms of gender, separate PRA workshops were held for men and women in order not to compromise the amount and quality of information that can be generated from the less confident if they were to be combined. Specifically, old men and women were incorporated into the sample for the group discussions in order to capture information related to historical trends in climate. Itwas envisaged that they would be able to recall as far back as they could and provide rich information on these trends. In the same context, youths were incorporated into the sample in				

		order to validate some of the recent trends on climate suggested by the elderly.				
Sampling sizes reported?	Yes	A sample of 720 households across countries was selected for<br>the survey, 180 households per each of the four districts.<br>[...]<br>For FGDs and PRA workshops, a group of eight to 15 participants<br>was selected to represent the three villages per district, with approximately five representatives from each of the three villages per district.				
Data analysis methods reported?	Yes	Qualitative data were categorised and analysed in four distinct themes. These themes are<br>Perceptions regarding changes in weather patterns, Perceptions regarding causes of changes and variability in climate, Perceptions regarding other stressors among farmers and Perceptions regarding climate change in relation to other stressors.<br>These perceptions were established in historical trend lines,<br>FGDs and matrix scoring and ranking and they are presented in this manner in the sections under results and discussion.<br>[...]<br>Data from the questionnaire survey were entered into the Statistical Package for the Social Sciences (SPSS) and analysed by running descriptive frequencies in relation to the distinct themes highlighted in this section. These themes include perceptions regarding changes in weather patterns in general and for specific seasons and regarding causes of these changes. These frequencies were disaggregated by district and country.				

**Structured summary of operationalization – validity assessment**	**1.1 DCM Appropriate**	**1.2 valid empirical**	**1. conclusion -**	**2. Feasible?**
**Construct:** Farmer perceptions				

**Article:** Mubaya et al (2012)				**rep?**	**Valid?**	
Criterion	Assessment	Quoted text or Rationale for negative assessment				
Construct defined?	Yes	there is an alternative approach which underscores how individuals perceive their environment and make decisions, with mal-adaptations attributed to problems in perception, cognition or the lack of available information (Diggs, 1991; Saarinen, 1966; Taylor et al., 1988). The main point is that from whatever level these adapta- tion measures are taken, the adaptation and coping measures depend on households' perceptions of extreme events and the problems associated with them (Davies, 1993).	Yes/ no/ can't tell	Yes/ no/ can't tell	Yes/ no/ can't tell<br>YES	Yes/ no/ can't tell
Data collection methods reported?	Yes	The qualitative methods of data collection used include Participatory Rural Appraisal (PRA) techniques such as historical trend analysis and matrix scoring and ranking and Focus Group Discussions (FGDs). The quantitative method used is the household questionnaire survey.	YES			
Reporting of indicators/questions used to operationalise construct?	Yes	2.2.2. Qualitative assessments FGDs were used to first of all establish the general perceptions<br>regarding climate change and variability and their causes and various stressors that confront farmers' livelihoods (see Appendix 2). Following this, it was considered important for this study to factor in how farmers regard climate change and variability as an obstacle to their livelihoods among the multiple stressors that they had identified. Among these stressors are climate variability in different forms, issues of financial capital, issues related to cattle pests and diseases, inadequate draught power, marketing issues and HIV and AIDS. A matrix scoring and ranking exercise was then facilitated for<br>farmers. Farmers were asked as a group to select from the long list of stressors the ones they considered critical for the purposes of scoring and ranking. The second step involved participants defining criteria that they would use to evaluate these stressors. These criteria include food security, income generation, crop production and livelihood security. Through group consensus, farmers then decided how much to allocate each shock out of a total of 20 points, based on the group defined criteria.		YES		

		Historical trend lines were used to elicit information on specific historical trends in farmers' perceptions regarding changes in climate over a period of 20 years and as far back as they could recall. Specifically, participants were asked to recall major occurrences that had a bearing on climate and weather, community resources, and even the political situation. They were then asked to indicate what occurrences had the greatest impact on their livelihoods among the cited events.<br>[...]<br>2.2.3. Quantitative assessments The questionnaire survey was used to collect household data<br>and complement data generated through the qualitative methods. This survey collected data on changes in crops grown over a period of five years and reasons for these changes, indicators for good and bad crop production seasons and years considered to be good or bad over a ten year period. Questions in the survey also related to changes in weather patterns over a ten year period in relation to agriculture and what might have caused these changes. General household characteristics were also captured in this survey (see Appendix 1).				
Sampling strategies reported?	Yes	A sample of 720 households across countries was selected for<br>the survey, 180 households per each of the four districts. Specifi- cally, systematic random sampling was employed to come up with six villages per district (making them 24 across countries) and 30 households per each of these villages, making a total of 380 households per country (this study was part of a big inter- institutional research-based development project). For FGDs and PRA workshops, a group of eight to 15 participants<br>was selected to represent the three villages per district, with approximately five representatives from each of the three villages per district. In coming up with this group, factors such as age and gender were used. In terms of gender, separate PRA workshops were held for men and women in order not to compromise the amount and				

		quality of information that can be generated from the less confident if they were to be combined. Specifically, old men and women were incorporated into the sample for the group discussions in order to capture information related to historical trends in climate. Itwas envisaged that they would be able to recall as far back as they could and provide rich information on these trends. In the same context, youths were incorporated into the sample in order to validate some of the recent trends on climate suggested by the elderly.				
Sampling sizes reported?	Yes	A sample of 720 households across countries was selected for<br>the survey, 180 households per each of the four districts.<br>[...]<br>For FGDs and PRA workshops, a group of eight to 15 participants<br>was selected to represent the three villages per district, with approximately five representatives from each of the three villages per district.				
Data analysis methods reported?	Yes	Qualitative data were categorised and analysed in four distinct themes. These themes are<br>Perceptions regarding changes in weather patterns, Perceptions regarding causes of changes and variability in climate, Perceptions regarding other stressors among farmers and Perceptions regarding climate change in relation to other stressors.<br>These perceptions were established in historical trend lines,<br>FGDs and matrix scoring and ranking and they are presented in this manner in the sections under results and discussion.<br>[...]<br>Data from the questionnaire survey were entered into the Statistical Package for the Social Sciences (SPSS) and analysed by running descriptive frequencies in relation to the distinct themes highlighted in this section. These themes include perceptions regarding changes in weather patterns in general and for specific seasons and regarding				

		causes of these changes. These frequencies were disaggregated by district and country.				

**Structured summary of operationalization – validity assessment**			**1.1 DCM Appropriate**	**1.2 valid empirical rep?**	**1. conclusion - Valid?**	**2. Feasible?**
**Construct:** Non-climatic stress						
**Article:** Mubaya et al (2012)						
Criterion	Assessment	Quoted text or Rationale for negative assessment				
Construct defined?	Yes	It is important to note though, that climate change amplifies<br>already existing risks for farmers. This is the case as there are non- climatic risk factors such as economic instability, trade liberalisa- tion, conflicts and poor governance that may also be faced by farmers (Nyong and Niang-Diop, 2006). Other factors are impacts of diseases such as malaria and HIV and AIDS and lack of and limited access to climate and agricultural information (Gandure, 2005; Gandure and Marongwe, 2006). Africa is also characterised by institutional and legal frameworks that are, in some cases, insuffi- cient to deal with environmental degradation and disaster risks (Beg et al., 2002; Sokona and Denton, 2001).	Yes/ no/ can't tell	Yes/ no/ can't tell	Yes/ no/ can't tell<br>YES	Yes/ no/ can't tell
Data collection methods reported?	Yes	The qualitative methods of data collection used include Participatory Rural Appraisal (PRA) techniques such as historical trend analysis and matrix scoring and ranking and Focus Group Discussions (FGDs). The quantitative method used is the household questionnaire survey.	YES			
Reporting of indicators/questions used to operationalise construct?	Yes	2.2.2. Qualitative assessments FGDs were used to first of all establish the general perceptions<br>regarding climate change and variability and their causes and various stressors that confront farmers' livelihoods (see Appendix 2). Following this, it was considered important for this study to factor in how farmers regard climate change and variability as an obstacle to their livelihoods among the multiple stressors that they had identified. Among these stressors are climate variability in		YES		

		different forms, issues of financial capital, issues related to cattle pests and diseases, inadequate draught power, marketing issues and HIV and AIDS. A matrix scoring and ranking exercise was then facilitated for<br>farmers. Farmers were asked as a group to select from the long list of stressors the ones they considered critical for the purposes of scoring and ranking. The second step involved participants defining criteria that they would use to evaluate these stressors. These criteria include food security, income generation, crop production and livelihood security. Through group consensus, farmers then decided how much to allocate each shock out of a total of 20 points, based on the group defined criteria. Historical trend lines were used to elicit information on specific historical trends in farmers' perceptions regarding changes in climate over a period of 20 years and as far back as they could recall. Specifically, participants were asked to recall major occurrences that had a bearing on climate and weather, community resources, and even the political situation. They were then asked to indicate what occurrences had the greatest impact on their livelihoods among the cited events.<br>[...]<br>2.2.3. Quantitative assessments The questionnaire survey was used to collect household data<br>and complement data generated through the qualitative methods. This survey collected data on changes in crops grown over a period of five years and reasons for these changes, indicators for good and bad crop production seasons and years considered to be good or bad over a ten year period. Questions in the survey also related to changes in weather patterns over a ten year period in relation to agriculture and what might have caused these changes. General household characteristics were also captured in this survey (see Appendix 1).				
Sampling strategies reported?	Yes	A sample of 720 households across countries was selected for<br>the survey, 180 households per each of the four districts.				

		Specifi- cally, systematic random sampling was employed to come up with six villages per district (making them 24 across countries) and 30 households per each of these villages, making a total of 380 households per country (this study was part of a big inter- institutional research-based development project). For FGDs and PRA workshops, a group of eight to 15 participants was selected to represent the three villages per district, with approximately five representatives from each of the three villages per district. In coming up with this group, factors such as age and gender were used. In terms of gender, separate PRA workshops were held for men and women in order not to compromise the amount and quality of information that can be generated from the less confident if they were to be combined. Specifically, old men and women were incorporated into the sample for the group discussions in order to capture information related to historical trends in climate. Itwas envisaged that they would be able to recall as far back as they could and provide rich information on these trends. In the same context, youths were incorporated into the sample in order to validate some of the recent trends on climate suggested by the elderly.				
Sampling sizes reported?	Yes	A sample of 720 households across countries was selected for<br>the survey, 180 households per each of the four districts.<br>[...]<br>For FGDs and PRA workshops, a group of eight to 15 participants<br>was selected to represent the three villages per district, with approximately five representatives from each of the three villages per district.				
Data analysis methods reported?	Yes	Qualitative data were categorised and analysed in four distinct themes. These themes are<br>Perceptions regarding changes in weather patterns, Perceptions regarding causes of changes and variability in climate, Perceptions regarding other stressors among farmers and Perceptions regarding climate change in				

		relation to other stressors.<br>These perceptions were established in historical trend lines,<br>FGDs and matrix scoring and ranking and they are presented in this manner in the sections under results and discussion.<br>[...]<br>Data from the questionnaire survey were entered into the Statistical Package for the Social Sciences (SPSS) and analysed by running descriptive frequencies in relation to the distinct themes highlighted in this section. These themes include perceptions regarding changes in weather patterns in general and for specific seasons and regarding causes of these changes. These frequencies were disaggregated by district and country.				

Transparency Assessment Article summary	
**Article**	Mutsvangwa (2011)
**Transparent operationalizations**	Cereal production; vulnerability threshold
**Partially transparent**	
**Not transparent**	

**Structured summary of operationalization – validity assessment**			**1.1 DCM Appropriate**	**1.2 valid empirical rep?**	**1. conclusion - Valid?**	**2. Feasible?**
**Construct:** Cereal Production						
**Article:** Mutsvangwa (2011)						
Criterion	Assessment	Quoted text or Rationale for negative assessment				
Construct defined?	Yes	Smallholder farmers in Zimbabwe commonly produce cereals such as maize, millet and sorghum; with maize being the staple food and most commonly grown cereal. The energy content of the three cereals is almost the same, with maize, millet and sorghum producing 358, 329 and 336 kilocalories per 100g of grain respectively (Leder, 2010). In this study maize, sorghum and millet produced by the household is added so as to determine how much per capita cereal is produced by the household.<br>[...]<br>In addition the Southern Africa Regional Poverty Network's (2003) report on the regional overview of the southern African food security crisis suggests that an average family of 6 people requires about 800 -1000kg annually of cereal to be food secure, which also suggests a per capita cereal requirement of approximately 165kg.	Yes/ no/ can't tell	Yes/ no/ can't tell	Yes/ no/ can't tell<br><br>YES	Yes/ no/ can't tell<br><br>YES
Data collection methods reported?	Yes	The primary data used in this study was obtained from a survey carried out in September 2009. The survey gathered qualitative and quantitative data pertaining to social, demographic and economic aspects of the households, agriculture activities, farmers' perceptions of	YES			

		climate change and the role of local organizations in helping smallholder farmers develop strategies to mitigate against the negative climate change.				
Reporting of indicators/questions used to operationalise construct?	Yes	Data on production/acquisition of cereals, household size and asset ownership was gathered, as summarized in Table 5. This data was gathered using the household questionnaire.<br><br>**Type of data**: Agriculture production — **Specific data collected**: Arable land owned; crops grown and areas allocated to the crops; yields obtained; farming implements available; availability of draft power; livestock owned; crop management practices		YES		
Sampling strategies reported?	Yes	The selection of sites was done at the BACCC project level. The aim was to select areas that are marginal in terms of the climate experienced so as to assess how inhabitants of such communities are being affected or are going to be affected by climate change.<br>[...]<br>The selection for the study sites was done strategically to meet the objectives of the project and one of the main objectives was to look at smallholder farmers in marginal areas.				
Sampling sizes reported?	Yes	The sampling procedure involved selecting from each district, two wards; three villages from each ward; and 15 households from each village. A total of 180 households were selected from the two districts, 90 per district, 45 per ward and 15 households in each village. The description of the study sites is also summarized in Table 4.				
Data analysis methods reported?	Yes	The cleaned data was then analyzed by running descriptive statistics; mainly frequencies, descriptive and crosstabs.<br>[...]<br>The other analyses carried out involved running the 2 stage least squares regression model using SPSS to find				

		estimates for the vulnerability model. This involved a double regression of the per capita cereal production levels against household observable characteristics such as age, gender, education status of the household head, assess to extension services and other factors that were considered pertinent in influencing cereal production. The estimates obtained from the 2 stage least regression was used to measure the degree of each household's vulnerability to food insecurity. The estimated probability was given by:				

Structured summary of operationalization – validity assessment			1.1 DCM Appropriate	1.2 valid empirical rep?	1. conclusion - Valid?	2. Feasible?
**Construct:** Vulnerability threshold						
**Article:** Mutsvangwa (2011)						
Criterion	Assessment	Quoted text or Rationale for negative assessment				
Construct defined?	Yes	The choice of the vulnerability threshold involves generating a sample that is classified into two groups, that is those that are vulnerable and those that are not vulnerable to food insecurity. It entails establishing a vulnerability threshold, such that a household is said to be vulnerable if its vulnerability probability is greater or equal to v, i.e. vh ≥ v.	Yes/ no/ can't tell	Yes/ no/ can't tell	Yes/ no/ can't tell CAN'T TELL	Yes/ no/ can't tell
Data collection methods reported?	n/a	This is a threshold construct. Therefore it is operationalized through specification	CAN'T TELL			
Reporting of indicators/questions used to operationalise construct?	Yes/no	The outcome of the above model measures the degree of vulnerability to food insecurity for each household. The probability of a household being vulnerable to food insecurity is ≥ 0.5 and the probability a household not being vulnerable to food insecurity is < 0.5, thus a threshold of 0.5 was used.		CAN'T TELL		
Sampling strategies reported?	n/a	This is a threshold construct. Therefore it is operationalized through specification				
Sampling sizes reported?	n/a	This is a threshold construct. Therefore it is operationalized through specification				

Data analysis methods reported?	Yes/no	The other analyses carried out involved running the 2 stage least squares regression model using SPSS to find estimates for the vulnerability model. This involved a double regression of the per capita cereal production levels against household observable characteristics such as age, gender, education status of the household head, assess to extension services and other factors that were considered pertinent in influencing cereal production. The estimates obtained from the 2 stage least regression was used to measure the degree of each household's vulnerability to food insecurity. The estimated probability was given by:<br>[...]<br>The outcome of the above model measures the degree of vulnerability to food insecurity for each household. The probability of a household being vulnerable to food insecurity is ≥ 0.5 and the probability a household not being vulnerable to food insecurity is < 0.5, thus a threshold of 0.5 was used. Food insecurity increases the chances of being negatively impacted by climate change. Thus a household with a probability of < 0.5 has less chances of being negatively impacted by climate change and a household with a probability ≥ 0.5 has greater chances of being impacted by climate change.				

Transparency Assessment Article summary	
**Article**	Notenbaert et al (2013)
**Transparent operationalizations**	Exposure; Institutional environment; Risks
**Partially transparent**	Livelihood strategies;
**Not transparent**	Livelihood assets; Livelihoods; Vulnerability Outcomes

**Structured summary of operationalization – validity assessment**			**1.1 DCM Appropriate**	**1.2 valid empirical rep?**	**1. conclusion - Valid?**	**2. Feasible?**
**Construct:** Exposure						
**Article:** Notenbaert et al (2013)						
Criterion	Assessment	Quoted text or Rationale for negative assessment				
Construct defined?	Yes	risks (or a chain of risky events) that people confront in pursuit of their livelihoods, (Turner et al. 2003).	Yes/ no/ can't tell	Yes/ no/ can't tell	Yes/ no/ can't tell<br><br>YES	Yes/ no/ can't tell
Data collection methods reported?	Yes	In this study, we assume the exposure to climate change and variability of households in the same village to be equal. Differences in vulnerability, described as outcomes of this exposure, are therefore attributed to differences in sensitivity and adaptive capacity only.<br>[...]<br>We also believe that the overall assumption of equal exposure is sensible in the fairly homogenous landscape of Mabalane district and the relatively clustered villages as our unit of analysis. When analyzing communities spread out in more complex land- scapes or in rugged terrain, this assumption will not nec- essarily hold true	YES			
Reporting of indicators/questions used to operationalise construct?	Yes	In this study, we assume the exposure to climate change and variability of households in the same village to be equal. Differences in vulnerability, described as outcomes of this exposure, are therefore attributed to differences in sensitivity and adaptive capacity only.<br>[...]<br>We also believe that the overall assumption of equal exposure is sensible in the fairly homogenous landscape of Mabalane district and the relatively clustered villages		YES		

		as our unit of analysis. When analyzing communities spread out in more complex land- scapes or in rugged terrain, this assumption will not nec- essarily hold true				
Sampling strategies reported?	Yes	In this study, we assume the exposure to climate change and variability of households in the same village to be equal. Differences in vulnerability, described as outcomes of this exposure, are therefore attributed to differences in sensitivity and adaptive capacity only.<br>[...]<br>We also believe that the overall assumption of equal exposure is sensible in the fairly homogenous landscape of Mabalane district and the relatively clustered villages as our unit of analysis. When analyzing communities spread out in more complex land- scapes or in rugged terrain, this assumption will not nec- essarily hold true				
Sampling sizes reported?	Yes	In this study, we assume the exposure to climate change and variability of households in the same village to be equal. Differences in vulnerability, described as outcomes of this exposure, are therefore attributed to differences in sensitivity and adaptive capacity only.<br>[...]<br>We also believe that the overall assumption of equal exposure is sensible in the fairly homogenous landscape of Mabalane district and the relatively clustered villages as our unit of analysis. When analyzing communities spread out in more complex land- scapes or in rugged terrain, this assumption will not nec- essarily hold true				
Data analysis methods reported?	Yes	In this study, we assume the exposure to climate change and variability of households in the same village to be equal. Differences in vulnerability, described as outcomes of this exposure, are therefore attributed to differences in sensitivity and adaptive capacity only.<br>[...]<br>We also believe that the overall assumption of equal exposure is sensible in the fairly homogenous landscape of Mabalane district and the relatively clustered villages as our unit of analysis. When analyzing communities spread out in more complex land- scapes or in rugged				

		terrain, this assumption will not nec- essarily hold true				

**Structured summary of operationalization – validity assessment**			**1.1 DCM Appropriate**	**1.2 valid empirical rep?**	**1. conclusion - Valid?**	**2. Feasible?**
**Construct:** Institutional environment						
**Article:** Notenbaert et al (2013)						
Criterion	Assessment	Quoted text or Rationale for negative assessment				
Construct defined?	Yes	(Turner et al. 2003).	Yes/ no/ can't tell	Yes/ no/ can't tell	Yes/ no/ can't tell<br><br>NO	Yes/ no/ can't tell
Data collection methods reported?	Yes	The factors that make up these policies, institutions and processes can be changed, but will usually require action at ''higher'' levels (Messer and Townsley 2003). As with the exposure, we therefore assume these are equal for all households in the same village.	NO			
Reporting of indicators/questions used to operationalise construct?	Yes	The factors that make up these policies, institutions and processes can be changed, but will usually require action at ''higher'' levels (Messer and Townsley 2003). As with the exposure, we therefore assume these are equal for all households in the same village.		NO		
Sampling strategies reported?	Yes	The factors that make up these policies, institutions and processes can be changed, but will usually require action at ''higher'' levels (Messer and Townsley 2003). As with the exposure, we therefore assume these are equal for all households in the same village.				
Sampling sizes reported?	Yes	The factors that make up these policies, institutions and processes can be changed, but will usually require action at ''higher'' levels (Messer and Townsley 2003). As with the exposure, we therefore assume these are equal for all households in the same village.				
Data analysis methods reported?	Yes	The factors that make up these policies, institutions and processes can be changed, but will usually require action at ''higher'' levels (Messer and Townsley 2003). As with the exposure, we therefore assume these are equal for all households in the same village.				

Structured summary of operationalization – validity assessment			1.1 DCM Appropriate	1.2 valid empirical rep?	1. conclusion - Valid?	2. Feasible?
**Construct:** Risks						
**Article:** Notenbaert et al (2013)						
Criterion	Assessment	Quoted text or Rationale for negative assessment				
Construct defined?	Yes	(Turner et al. 2003).	Yes/ no/ can't tell	Yes/ no/ can't tell	Yes/ no/ can't tell	Yes/ no/ can't tell
Data collection methods reported?	Yes	In this study, we assume the exposure to climate change and variability of households in the same village to be equal. Differences in vulnerability, described as outcomes of this exposure, are therefore attributed to differences in sensitivity and adaptive capacity only.<br>[...]<br>We also believe that the overall assumption of equal exposure is sensible in the fairly homogenous landscape of Mabalane district and the relatively clustered villages as our unit of analysis. When analyzing communities spread out in more complex land- scapes or in rugged terrain, this assumption will not nec- essarily hold true	NO		NO	
Reporting of indicators/questions used to operationalise construct?	Yes	In this study, we assume the exposure to climate change and variability of households in the same village to be equal. Differences in vulnerability, described as outcomes of this exposure, are therefore attributed to differences in sensitivity and adaptive capacity only.<br>[...]<br>We also believe that the overall assumption of equal exposure is sensible in the fairly homogenous landscape of Mabalane district and the relatively clustered villages as our unit of analysis. When analyzing communities spread out in more complex land- scapes or in rugged terrain, this assumption will not nec- essarily hold true		NO		
Sampling strategies reported?	Yes	In this study, we assume the exposure to climate change and variability of households in the same village to be equal. Differences in vulnerability, described as outcomes of this exposure, are therefore attributed to differences in				

		sensitivity and adaptive capacity only.<br>[...]<br>We also believe that the overall assumption of equal exposure is sensible in the fairly homogenous landscape of Mabalane district and the relatively clustered villages as our unit of analysis. When analyzing communities spread out in more complex land- scapes or in rugged terrain, this assumption will not nec- essarily hold true				
Sampling sizes reported?	Yes	In this study, we assume the exposure to climate change and variability of households in the same village to be equal. Differences in vulnerability, described as outcomes of this exposure, are therefore attributed to differences in sensitivity and adaptive capacity only.<br>[...]<br>We also believe that the overall assumption of equal exposure is sensible in the fairly homogenous landscape of Mabalane district and the relatively clustered villages as our unit of analysis. When analyzing communities spread out in more complex land- scapes or in rugged terrain, this assumption will not nec- essarily hold true				
Data analysis methods reported?	Yes	In this study, we assume the exposure to climate change and variability of households in the same village to be equal. Differences in vulnerability, described as outcomes of this exposure, are therefore attributed to differences in sensitivity and adaptive capacity only.<br>[...]<br>We also believe that the overall assumption of equal exposure is sensible in the fairly homogenous landscape of Mabalane district and the relatively clustered villages as our unit of analysis. When analyzing communities spread out in more complex land- scapes or in rugged terrain, this assumption will not nec- essarily hold true				

**Transparency Assessment Article summary**	
**Article**	Piya et al (2012)
**Transparent operationalizations**	Exposure; Financial capital; Human capital; natural capital; physical capital; sensitivity; social capital
**Partially transparent**	
**Not transparent**	

**Structured summary of operationalization – validity assessment**			**1.1 DCM Appropriate**	**1.2 valid empirical rep?**	**1. conclusion - Valid?**	**2. Feasible?**
**Construct:** Exposure						
**Article:** Piya et al (2012)						
Criterion	Assessment	Quoted text or Rationale for negative assessment				
Construct defined?	Yes	Exposure is the nature and degree to which a system is exposed to significant climatic variations.	Yes/ no/ can't tell	Yes/ no/ can't tell	Yes/ no/ can't tell<br><br>YES	Yes/ no/ can't tell<br><br>CAN'T TELL
Data collection methods reported?	Yes	This study is based on the primary data collected by household survey conducted in<br>two phases. The first phase of household survey was conducted in February-March 2010 and the second phase in May-June 2011.<br>[...]<br>The latitude, longitude and altitude of the sample households were recorded during the second phase of field visit. This paper also makes use of raw monthly minimum and maximum temperature and<br>monthly precipitation data obtained from Department of Hydrology and Meteorology (DHM) in Kathmandu, Nepal for the time period of 32 years, from 1977-2008. Temperature data was obtained from 49 stations and precipitation data from 218 stations distributed all over the country. The temperature and precipitation at the household level was interpolated for each year from the weather stations using the latitude-longitude-altitude information of each household by ordinary kriging method in ArcGIS 10.	YES			
Reporting of indicators/questions	Yes	3.2.1 Exposure For this study, historical changes in climate variables and occurrence of extreme		YES		

<table>
<tr>
<td>used to operationalise construct?</td>
<td></td>
<td>climatic events are taken as indicators of exposure (Table 1). Rate of change in average annual maximum temperature, average annual minimum temperature and average annual precipitation for the time period of 1977–2008 represent the historical climate changes. The temperature and precipitation for individual household was interpolated for each year from the station level data (49 temperature stations and 218 precipitation stations) using the latitude, longitude, and altitude information of the stations and the households by ordinary kriging method in ArcGIS10. The coefficient of the trends of climate variables is calculated separately for each household. Floods/landslides, droughts and hailstorms are the most commonly occurring natural disasters in the study area. Number of occurrence of these extreme events for the last ten years was obtained for each household from the household survey (Appendix 1). It was hypothesized that higher the rate of change of the climate variables and higher the frequency of natural disasters, higher will be the exposure of the households to climate change and extremes.
Table 1. Indicators for exposure
<table>
<tr><th>Component Indicators</th><th>Description of the Indicators</th><th>Unit</th><th>Hypothesized relation</th></tr>
<tr><td rowspan="2">Historical change in climate Variables</td><td>Rate of change in average annual minimum temperature (1977 – 2008)</td><td>Coefficient of trend</td><td>+</td></tr>
<tr><td>Rate of change in average annual</td><td>Coefficient of trend</td><td>+</td></tr>
</table>
</td>
<td></td>
<td></td>
<td></td>
<td></td>
</tr>
</table>

			maximum temperature (1977 – 2008)						
			Rate of change in average annual precipitation (1977 – 2008)	Coefficient of trend	+				
		Extreme climate events	Frequency of climate related natural disasters (floods, landslides, droughts and hailstorms) over the last 10 years	Number	+				
Sampling strategies reported?	Yes	Sixty randomly selected households from each VDC form the sample for the household survey.<br>[...]<br>Out of the total 240 households covered in 2010 field survey, 58 households in Chitwan, 56 households in Makwanpur, 54 household in Dhading, and 53 households in Gorkha could be revisited in 2011 survey;							
Sampling sizes reported?	Yes	Sixty randomly selected households from each VDC form the sample for the household survey.<br>[...]<br>Out of the total 240 households covered in 2010 field survey, 58 households in Chitwan, 56 households in Makwanpur, 54 household in Dhading, and 53 households							

		in Gorkha could be revisited in 2011 survey; thus the final sample constitutes a total of 221 households.<br>[...]<br>temperature and<br>monthly precipitation data obtained from Department of Hydrology and Meteorology (DHM) in Kathmandu, Nepal for the time period of 32 years, from 1977-2008. Temperature data was obtained from 49 stations and precipitation data from 218 stations distributed all over the country.				
Data analysis methods reported?	Yes	Having chosen the suitable indicators, now these need to be normalized so as to bring<br>the values of the indicators within the comparable range (Nelson, et al., 2010b; Gbetibouo & Ringler, 2009; Vincent, 2004). Normalization is done by subtracting the mean from the observed value and dividing by the standard deviation for each indicator.<br>$Normalized\ value\ = Observed\ Value\ /\ Mean\ standard\ deviation$<br>Next, weights should be assigned to these indicators.<br>[...]<br><br>The normalized variables are then multiplied with the assigned weights to construct the indices (for exposure, sensitivity, and adaptive capacity separately) using the following formulae:<br>$Ij = \sum^{k}_{i=1} b_i[(a_{ji} – x_i)/s_i]$<br>where, 'I' is the respective index value, 'b' is the loadings from first component from PCA (PCA1) taken as weights for respective indicators, 'a' is the indicator value, 'x' is the mean indicator value, and 's' is the standard deviation of the indicators. Finally, vulnerability index for each household is calculated as: V = E + S – AC, where, V is the vulnerability index, E the exposure index, S is the sensitivity index and AC is the adaptive capacity index for respective household.				

Structured summary of operationalization – validity assessment			1.1 DCM Appropriate	1.2 valid empirical rep?	1. conclusion - Valid?	2. Feasible?
**Construct:** Financial Capital						
**Article:** Piya et al (2012)						
Criterion	Assessment	Quoted text or Rationale for negative assessment				
Construct defined?	Yes	Ellis (2000) and DFID (1999)	Yes/ no/ can't tell	Yes/ no/ can't tell	Yes/ no/ can't tell	Yes/ no/ can't tell
Data collection methods reported?	Yes	This study is based on the primary data collected by household survey conducted in two phases. The first phase of household survey was conducted in February-March 2010 and the second phase in May-June 2011.	YES		YES	
Reporting of indicators/questions used to operationalise construct?	Yes	Gross household annual income, livelihood diversification index, household savings, and ownership of small livestock (goat, poultry, and pig) are taken as the indicators of financial assets. These indicators of financial assets are not specific to climate shocks only. Gross annual income of the household is the sum total of the cash and non-cash income from 11 different sources shown in Appendix 2. Higher income means greater availability of resources at disposal to maximize positive livelihood outcomes. Besides the amount of annual income, the sources from which the income is derived also need to be considered. If all of the income is derived from farming alone, then such income will be adversely affected during the years of bad weather. On the other hand, if the income is derived from more than one source, then risk will be distributed among the sources. In order to capture this aspect of income, Livelihood Diversification Index (LDI) is calculated; higher diversification indicating better ability of the household to switch among the activities when needed. Herfindahl index of diversification is used (Kimenju & Tschirley, 2009), which is calculated as $Dk = 1 - \sum_{i=1..N} (S_{i,k})^2$ where, Dk is the diversification index, i is the specific livelihood activity, N is the total number of activities being considered, k is the particular household, and Si,k is the		YES		

<table>
<tr><td></td><td></td><td>share of ith activity to the total household income for kth household (see Appendix 2). In addition to income at disposal, households which are able to make some savings out of their income will be able to make productive investments like family education or use the savings as buffer during the times of need. For Chepangs, small livestock are also important sources of cash income; they keep these livestock as buffer to sell during the times of stress or to pay back the loan that they take from moneylenders.
<table>
<tr><td>Component Indicators</td><td>Description of the Indicators</td><td>Unit</td><td>Hypothesized relation</td></tr>
<tr><td>Financial Assets</td><td>Gross household annual income</td><td>NRs</td><td>+</td></tr>
<tr><td></td><td>Livelihood Diversification Index</td><td>-</td><td>+</td></tr>
<tr><td></td><td>Total household savings</td><td>NRs</td><td>+</td></tr>
<tr><td></td><td>Ownership of goat, poultry, and pig</td><td>LSU</td><td>+</td></tr>
<tr><td></td><td>Memberships in CBOs</td><td>Number</td><td>+</td></tr>
</table></td><td></td><td></td><td></td><td></td></tr>
<tr><td>Sampling strategies reported?</td><td>Yes</td><td>Sixty randomly selected households from each VDC form the sample for the household survey.
[...]
Out of the total 240 households covered in 2010 field survey, 58 households in Chitwan, 56 households in Makwanpur, 54 household in Dhading, and 53 households in Gorkha could be revisited in 2011 survey;</td><td></td><td></td><td></td><td></td></tr>
<tr><td>Sampling sizes reported?</td><td>Yes</td><td>Sixty randomly selected households from each VDC form the sample for the household survey.
[...]</td><td></td><td></td><td></td><td></td></tr>
</table>

		Out of the total 240 households covered in 2010 field survey, 58 households in Chitwan, 56 households in Makwanpur, 54 household in Dhading, and 53 households in Gorkha could be revisited in 2011 survey; thus the final sample constitutes a total of 221 households.				
Data analysis methods reported?	Yes	Having chosen the suitable indicators, now these need to be normalized so as to bring<br>the values of the indicators within the comparable range (Nelson, et al., 2010b; Gbetibouo & Ringler, 2009; Vincent, 2004). Normalization is done by subtracting the mean from the observed value and dividing by the standard deviation for each indicator.<br>$Normalized\ value\ = Observed\ Value\ /\ Mean\ standard\ deviation$<br>Next, weights should be assigned to these indicators.<br>[...]<br>Assigning weight by Principal Component Analysis (PCA) following Filmer and Pritchett (2001) is thus preferred compared to the former two methods (Nelson et al., 2010b; Gbetibouo & Ringler, 2009; Cutter, Boruff, & Shirley, 2003). PCA was run for the selected indicators of exposure, sensitivity, and adaptive capacity separately in Data Analysis and Statistical Software (STATA10) software for assigning the weights. The loadings from the first component of PCA are used as the weights for the indicators. The weights assigned for each indicator varies between -1 and +1, sign of the indicators denoting the direction of relationship with other indicators used to construct the respective index. The magnitude of the weights describes the contribution of each indicator to the value of the index. PCA was run separately for the indicators of exposure, sensitivity and adaptive capacity. Stepwise PCA was run for the indicators of adaptive capacity. The first-step PCA was run for the indicators of each asset group separately to observe the relative importance of indicators within each asset category. From the weights obtained from first-step PCA, individual index				

		values for each asset type was calculated. Second-step PCA was run using the index values for each of the five asset types to analyze which asset group contributes the most to the total adaptive capacity. Overall adaptive capacity index was calculated using the weights (loadings) obtained from the second step PCA run for the five asset categories.<br>The normalized variables are then multiplied with the assigned weights to construct the indices (for exposure, sensitivity, and adaptive capacity separately) using the following formulae:<br>$Ij = \sum^{k}_{i=1} b_i[(a_{ji} - x_i)/s_i]$<br>where, 'I' is the respective index value, 'b' is the loadings from first component from PCA (PCA1) taken as weights for respective indicators, 'a' is the indicator value, 'x' is the mean indicator value, and 's' is the standard deviation of the indicators. Finally, vulnerability index for each household is calculated as: V = E + S – AC, where, V is the vulnerability index, E the exposure index, S is the sensitivity index and AC is the adaptive capacity index for respective household.				

**Structured summary of operationalization – validity assessment**			**1.1 DCM Appropriate**	**1.2 valid empirical rep?**	**1. conclusion - Valid?**	**2. Feasible?**
**Construct:** Human Capital						
**Article:** Piya et al (2012)						
Criterion	Assessment	Quoted text or Rationale for negative assessment				
Construct defined?	Yes	Ellis (2000) and DFID (1999)	Yes/ no/ can't tell	Yes/ no/ can't tell	Yes/ no/ can't tell	Yes/ no/ can't tell
Data collection methods reported?	Yes	This study is based on the primary data collected by household survey conducted in<br>two phases. The first phase of household survey was conducted in February-March 2010 and the second phase in May-June 2011.	YES		YES	YES
Reporting of indicators/questions used to	Yes	Human asset is represented by highest qualification in the family; trainings or<br>vocational courses attended by the family members; and		YES		

<table>
<tr>
<td>operationalise construct?</td>
<td></td>
<td>dependency ratio. These indicators are not directly related climate shocks; however they are still relevant because development of human capabilities through vocational trainings or formal education enable households to increase their income by undertaking skilled non-farm activities, which are less climate- sensitive compared to farming and gathering, thereby helping the households to avert climate risks. Furthermore, it also diversifies household livelihood sources which help to buffer the risks posed by climate on farm income. Households with higher dependency ratio will have more burdens on the earning members thereby reducing the adaptive capacity. The implication of dependency ratio is common to any types of shocks including climate.

<table>
<tr><th>Component Indicators</th><th>Description of the Indicators</th><th>Unit</th><th>Hypothesized relation</th></tr>
<tr><td rowspan="3">Human Assets</td><td>Highest qualification in the family</td><td>Number of schooling years</td><td>+</td></tr>
<tr><td>Dependency Ratio</td><td>-</td><td>+</td></tr>
<tr><td>Trainings or vocational course attended by family members</td><td>Number</td><td>-</td></tr>
</table>
</td>
<td></td>
<td></td>
<td rowspan="2"></td>
<td rowspan="2"></td>
</tr>
<tr>
<td>Sampling strategies reported?</td>
<td>Yes</td>
<td>Sixty randomly selected households from each VDC form the sample for the household survey.
[...]
Out of the total 240 households covered in 2010 field survey, 58 households in Chitwan, 56 households in Makwanpur, 54 household in Dhading, and 53 households</td>
<td></td>
<td></td>
</tr>
</table>

		in Gorkha could be revisited in 2011 survey;				
Sampling sizes reported?	Yes	Sixty randomly selected households from each VDC form the sample for the household survey.<br>[...]<br>Out of the total 240 households covered in 2010 field survey, 58 households in Chitwan, 56 households in Makwanpur, 54 household in Dhading, and 53 households in Gorkha could be revisited in 2011 survey; thus the final sample constitutes a total of 221 households.				
Data analysis methods reported?	Yes	Having chosen the suitable indicators, now these need to be normalized so as to bring<br>the values of the indicators within the comparable range (Nelson, et al., 2010b; Gbetibouo & Ringler, 2009; Vincent, 2004). Normalization is done by subtracting the mean from the observed value and dividing by the standard deviation for each indicator.<br>$Normalized\ value\ = Observed\ Value\ /\ Mean\ standard\ deviation$<br>Next, weights should be assigned to these indicators.<br>[...]<br>Assigning weight by Principal Component Analysis (PCA) following Filmer and Pritchett (2001) is thus preferred compared to the former two methods (Nelson et al., 2010b; Gbetibouo & Ringler, 2009; Cutter, Boruff, & Shirley, 2003). PCA was run for the selected indicators of exposure, sensitivity, and adaptive capacity separately in Data Analysis and Statistical Software (STATA10) software for assigning the weights. The loadings from the first component of PCA are used as the weights for the indicators. The weights assigned for each indicator varies between -1 and +1, sign of the indicators denoting the direction of relationship with other indicators used to construct the respective index. The magnitude of the weights describes the contribution of each indicator to the value of the index. PCA was run separately for the indicators of exposure, sensitivity and adaptive capacity. Stepwise PCA was run for the indicators of adaptive				

		capacity. The first-step PCA was run for the indicators of each asset group separately to observe the relative importance of indicators within each asset category. From the weights obtained from first-step PCA, individual index values for each asset type was calculated. Second-step PCA was run using the index values for each of the five asset types to analyze which asset group contributes the most to the total adaptive capacity. Overall adaptive capacity index was calculated using the weights (loadings) obtained from the second step PCA run for the five asset categories.<br>The normalized variables are then multiplied with the assigned weights to construct the indices (for exposure, sensitivity, and adaptive capacity separately) using the following formulae:<br>$Ij = \sum^{k}_{i=1} b_i[(a_{ji} - x_i)/s_i]$<br>where, 'I' is the respective index value, 'b' is the loadings from first component from PCA (PCA1) taken as weights for respective indicators, 'a' is the indicator value, 'x' is the mean indicator value, and 's' is the standard deviation of the indicators. Finally, vulnerability index for each household is calculated as: V = E + S – AC, where, V is the vulnerability index, E the exposure index, S is the sensitivity index and AC is the adaptive capacity index for respective household.				

**Structured summary of operationalization – validity assessment**			**1.1 DCM Appropriate**	**1.2 valid empirical rep?**	**1. conclusion - Valid?**	**2. Feasible?**
**Construct:** Natural Capital						
**Article:** Piya et al (2012)						
Criterion	Assessment	Quoted text or Rationale for negative assessment				
Construct defined?	Yes	Ellis (2000) and DFID (1999)	Yes/ no/ can't tell	Yes/ no/ can't tell	Yes/ no/ can't tell<br>YES	Yes/ no/ can't tell<br>YES
Data collection methods reported?	Yes	This study is based on the primary data collected by household survey conducted in<br>two phases. The first phase of household survey was conducted in February-March 2010 and the second phase	YES			

<table>
<tr><td></td><td></td><td>in May-June 2011.</td><td></td><td></td><td></td><td></td></tr>
<tr><td>Reporting of indicators/questions used to operationalise construct?</td><td>Yes</td><td>The quality of land possessed by the households is taken as an indicator of natural
assets. Chepangs possess three categories of land. Paddyland (khet) is the most productive category of land, usually having an irrigation source. Bari is terraced upland, which may or may not be irrigated, and is less productive than khet, but more productive than the third category, khoriya, which is unterraced sloppy land-plot. Natural assets, by their own nature, are more vulnerable to climate shocks than other types of assets. While terraced land types (khet and bari) are less prone to erosion, khoriya face greater risks of landslides and loss of top-soil due to run-off during rains. Households possessing higher share of khet and bari compared to khoriya will suffer less from climate disasters. Higher share of more productive land (khet and bari) also means higher food self-sufficiency, thus higher adaptive capacity. Higher share of khoriya indicates the opposite. Besides land, possession of bullock, which is the only means of ploughing fields in the hills, is another indicator of household natural assets.
<table>
<tr><td>Component Indicators</td><td>Description of the Indicators</td><td>Unit</td><td>Hypothesized relation</td></tr>
<tr><td rowspan="3">Natural Assets</td><td>Share of more productive land (khet + bari) possessed</td><td>% of total</td><td>+</td></tr>
<tr><td>Share of less productive land (khoriya) possessed</td><td>% of total</td><td>+</td></tr>
<tr><td>Have bullock (0 = No, 1 = Yes)</td><td>Ordinal</td><td>+</td></tr>
</table></td><td></td><td>YES</td><td></td><td></td></tr>
<tr><td>Sampling strategies reported?</td><td>Yes</td><td>Sixty randomly selected households from each VDC form the sample for the household survey.</td><td></td><td></td><td></td><td></td></tr>
</table>

		[...]<br>Out of the total 240 households covered in 2010 field survey, 58 households in Chitwan, 56 households in Makwanpur, 54 household in Dhading, and 53 households in Gorkha could be revisited in 2011 survey;				
Sampling sizes reported?	Yes	Sixty randomly selected households from each VDC form the sample for the household survey.<br>[...]<br>Out of the total 240 households covered in 2010 field survey, 58 households in Chitwan, 56 households in Makwanpur, 54 household in Dhading, and 53 households in Gorkha could be revisited in 2011 survey; thus the final sample constitutes a total of 221 households.				
Data analysis methods reported?	Yes	Having chosen the suitable indicators, now these need to be normalized so as to bring<br>the values of the indicators within the comparable range (Nelson, et al., 2010b; Gbetibouo & Ringler, 2009; Vincent, 2004). Normalization is done by subtracting the mean from the observed value and dividing by the standard deviation for each indicator.<br>$Normalized\ value\ = Observed\ Value\ /\ Mean\ standard\ deviation$<br>Next, weights should be assigned to these indicators.<br>[...]<br>Assigning weight by Principal Component Analysis (PCA) following Filmer and Pritchett (2001) is thus preferred compared to the former two methods (Nelson et al., 2010b; Gbetibouo & Ringler, 2009; Cutter, Boruff, & Shirley, 2003). PCA was run for the selected indicators of exposure, sensitivity, and adaptive capacity separately in Data Analysis and Statistical Software (STATA10) software for assigning the weights. The loadings from the first component of PCA are used as the weights for the indicators. The weights assigned for each indicator varies between -1 and +1, sign of the indicators denoting the direction of relationship with other indicators used to construct the respective index. The magnitude of the				

		weights describes the contribution of each indicator to the value of the index. PCA was run separately for the indicators of exposure, sensitivity and adaptive capacity. Stepwise PCA was run for the indicators of adaptive capacity. The first-step PCA was run for the indicators of each asset group separately to observe the relative importance of indicators within each asset category. From the weights obtained from first-step PCA, individual index values for each asset type was calculated. Second-step PCA was run using the index values for each of the five asset types to analyze which asset group contributes the most to the total adaptive capacity. Overall adaptive capacity index was calculated using the weights (loadings) obtained from the second step PCA run for the five asset categories. The normalized variables are then multiplied with the assigned weights to construct the indices (for exposure, sensitivity, and adaptive capacity separately) using the following formulae: $Ij = \sum^{k}_{i=1} b_i[(a_{ji} – x_i)/s_i]$ where, 'I' is the respective index value, 'b' is the loadings from first component from PCA (PCA1) taken as weights for respective indicators, 'a' is the indicator value, 'x' is the mean indicator value, and 's' is the standard deviation of the indicators. Finally, vulnerability index for each household is calculated as: V = E + S – AC, where, V is the vulnerability index, E the exposure index, S is the sensitivity index and AC is the adaptive capacity index for respective household.				

**Structured summary of operationalization – validity assessment**			**1.1 DCM Appropriate**	**1.2 valid empirical rep?**	**1. conclusion - Valid?**	**2. Feasible?**
**Construct:** Physical Capital						
**Article:** Piya et al (2012)						
Criterion	Assessment	Quoted text or Rationale for negative assessment				
Construct defined?	Yes	Ellis (2000) and DFID (1999)	Yes/ no/ can't tell	Yes/ no/ can't tell	Yes/ no/ can't tell	Yes/ no/ can't tell

<table>
<tr>
<td>Data collection methods reported?</td>
<td>Yes</td>
<td>This study is based on the primary data collected by household survey conducted in
two phases. The first phase of household survey was conducted in February-March 2010 and the second phase in May-June 2011.</td>
<td>YES</td>
<td></td>
<td rowspan="2">YES</td>
<td rowspan="2"></td>
</tr>
<tr>
<td>Reporting of indicators/questions used to operationalise construct?</td>
<td>Yes</td>
<td>Indicators for the physical assets are type of house, ownership of devices to access
information (mobile phone and radio), walking distance to the nearest road, and irrigated land. Out of these, only house quality and irrigation are directly related to climate risks. Possession of better quality house will improve the capacity to withstand the risks from extreme climate events. Type of house was indicated from a value of 1-3, 3 indicating the most durable type of house (see Table 3). Ownership of mobile phone and radio will increase the adaptive capacity through access to weather related information. Better access to information enables a household in planning proactive adaptation measures against climate risks. Walking distance to the nearest motor road, which in this case is also equivalent to the nearest marketplace, is assumed to be inversely related to adaptive capacity as household located far away from the markets will be in a disadvantageous position for lacking the opportunity of income generation from alternative sources like non-farm labor, which help in securing livelihoods during the periods of food shortage or crop failure. Farther distance from the roads also symbolizes poor access to inputs as the service centers are located at the road-heads. In addition, greater distance from the motor roads also means limited access to information as the marketplace acts as informal gathering centers where information exchange takes place, and also the formal institutions providing extension services are located there. Irrigation is directly related to climate shocks as it minimizes risks posed by droughts. Higher percentage of irrigated land means lesser dependence on natural rain for agricultural purposes, which is becoming more unpredictable with climate change.
<table>
<tr><td>Component Indicators</td><td>Description of the Indicators</td><td>Unit</td><td>Hypothesized relation</td></tr>
<tr><td>Physical Assets</td><td>Type of house (1 = thatch roof,</td><td>Ordinal value</td><td>+</td></tr>
</table>
</td>
<td></td>
<td>YES</td>
</tr>
</table>

<table>
<tr><td></td><td></td><td>
<table>
<tr><td></td><td>thatch/wooden wall; 2 = thatch roof, stone+mud wall; 3 = stone/tin/tile roof, stone/wood/brick+mud wall)</td><td></td><td></td></tr>
<tr><td></td><td>Have devices to access information (mobile, radio) (0 = No, 1 = Yes)</td><td>Ordinal value</td><td>+</td></tr>
<tr><td></td><td>Walking distance to nearest motor road</td><td>Hours</td><td>+</td></tr>
<tr><td></td><td>Irrigated land</td><td>% of total</td><td>-</td></tr>
</table>
</td><td></td><td></td><td></td><td></td></tr>
<tr><td>Sampling strategies reported?</td><td>Yes</td><td>Sixty randomly selected households from each VDC form the sample for the household survey.
[...]
Out of the total 240 households covered in 2010 field survey, 58 households in Chitwan, 56 households in Makwanpur, 54 household in Dhading, and 53 households in Gorkha could be revisited in 2011 survey;</td><td></td><td></td><td></td><td></td></tr>
<tr><td>Sampling sizes reported?</td><td>Yes</td><td>Sixty randomly selected households from each VDC form the sample for the household survey.
[...]
Out of the total 240 households covered in 2010 field survey, 58 households in Chitwan, 56 households in Makwanpur, 54 household in Dhading, and 53 households in Gorkha could be revisited in 2011 survey; thus the final sample constitutes a total of 221 households.</td><td></td><td></td><td></td><td></td></tr>
<tr><td>Data analysis methods reported?</td><td>Yes</td><td>Having chosen the suitable indicators, now these need to be normalized so as to bring
the values of the indicators within the comparable range (Nelson, et al., 2010b; Gbetibouo & Ringler, 2009; Vincent, 2004).
Normalization is done by subtracting the mean from the observed value and dividing by the standard deviation for each indicator.
$Normalized\ value\ = Observed\ Value\ /\ Mean\ standard$</td><td></td><td></td><td></td><td></td></tr>
</table>

		deviation<br>Next, weights should be assigned to these indicators.<br>[...]<br>Assigning weight by Principal Component Analysis (PCA) following Filmer and Pritchett (2001) is thus preferred compared to the former two methods (Nelson et al., 2010b; Gbetibouo & Ringler, 2009; Cutter, Boruff, & Shirley, 2003). PCA was run for the selected indicators of exposure, sensitivity, and adaptive capacity separately in Data Analysis and Statistical Software (STATA10) software for assigning the weights. The loadings from the first component of PCA are used as the weights for the indicators. The weights assigned for each indicator varies between -1 and +1, sign of the indicators denoting the direction of relationship with other indicators used to construct the respective index. The magnitude of the weights describes the contribution of each indicator to the value of the index. PCA was run separately for the indicators of exposure, sensitivity and adaptive capacity. Stepwise PCA was run for the indicators of adaptive capacity. The first-step PCA was run for the indicators of each asset group separately to observe the relative importance of indicators within each asset category. From the weights obtained from first-step PCA, individual index values for each asset type was calculated. Second-step PCA was run using the index values for each of the five asset types to analyze which asset group contributes the most to the total adaptive capacity. Overall adaptive capacity index was calculated using the weights (loadings) obtained from the second step PCA run for the five asset categories.<br>The normalized variables are then multiplied with the assigned weights to construct the indices (for exposure, sensitivity, and adaptive capacity separately) using the following formulae:<br>$Ij = \sum^{k}_{i=1} b_i[(a_{ji} – x_i)/s_i]$<br>where, ‘I’ is the respective index value, ‘b’ is the loadings from first component from PCA (PCA1) taken as weights for respective indicators, ‘a’ is the indicator value, ‘x’ is the mean indicator value, and ‘s’ is the standard deviation of the indicators. Finally, vulnerability index for each household is calculated as: V = E + S – AC, where, V is the vulnerability index, E the exposure index, S is the sensitivity index and AC is the adaptive capacity index for				

		respective household.				

Structured summary of operationalization – validity assessment			1.1 DCM Appropriate	1.2 valid empirical rep?	1. conclusion - Valid?	2. Feasible?
**Construct:** Sensitivity						
**Article:** Piya et al (2012)						
Criterion	Assessment	Quoted text or Rationale for negative assessment				
Construct defined?	Yes	is the degree to which a system is affected, either adversely or beneficially by climate-related stimuli.	Yes/ no/ can't tell	Yes/ no/ can't tell	Yes/ no/ can't tell	Yes/ no/ can't tell
Data collection methods reported?	Yes	This study is based on the primary data collected by household survey conducted in<br>two phases. The first phase of household survey was conducted in February-March 2010 and the second phase in May-June 2011.	CAN'T TELL		YES	YES
Reporting of indicators/questions used to operationalise construct?	Yes	3.2.2 Sensitivity<br>Sensitivity is given by the degree to which a system is modified or affected by an internal or external disturbance or set of disturbances (Gallopin, 2003). Livelihood impacts of climate related disasters were taken as the sensitivity indicator following Daze, Ambrose, & Ehrhart (2009) and Marshall et al. (2009). Deaths of family members and loss of properties (viz. land, livestock, and crop) due to climate related disasters over the last ten years represent the sensitivity for the purpose of this study. It is hypothesized that higher impacts of past climatic hazards will increase the sensitivity of the households to such events. The income structure will also determine the household sensitivity. Higher share of natural resource based income (composed of agriculture, livestock, forest, honey and handicrafts) will increase the sensitivity of the household as these sources are more dependent on climate; while higher share of non-natural resource based remunerative income sources (composed of salaried jobs, non-farm skilled jobs, and remittances from abroad) will reduce the sensitivity. These three income sources are categorized as remunerative sources because the return from these sources is comparatively higher than other sources of income. It was found that the annual income of the households having any of these three sources is higher compared to other households with no income from any of these three sources (Piya, Maharjan, & Joshi,		YES		

2011b). The detailed breakdown of the share of various income sources are given in Appendix 2.

Table 2. Indicators for sensitivity

Component Indicators	Description of the Indicators	Unit	Hypothesized relation
Fatalities	Death of family members due to climate related disasters (floods, landslides) over the last 10 years	Number of family members	+
Damage to properties	Total land damaged by flood/landslides over the last 10 years	Area in local units (Kattha4)	+
	Total livestock death due to flood/landslides/drought/hail over the last 10 years	Livestock Standard Unit (LSU5)	+
	Total crop damage due to flood/ landslides/ drought/ hail over the last 10 years	Value in Nepali Rupees (NRs6)	+
Income structure	Share of natural resource based income (agriculture, livestock, forest, honey, and handicraft) to total income	%	+
	Share of non-natural based remunerative income (salaried job, remittance, skilled non-farm job) to total income	%	-

4 1 Kattha = 0.033 ha

5 LSU is aggregates of different types of livestock kept at kept at household in standard unit calculated using the following equivalents; 1 adult buffalo = 1 LSU, 1 immature buffalo = 0.5 LSU, 1 Cow = 0.8 LSU, 1 calf = 0.4

		LSU, 1 pig = 0.3 LSU, 1 sheep or goat = 0.2 LSU and 1 poultry = 0.1 LSU (CBS, 2003; Baral, 2005).<br>6 73 NRs = 1 US $ at the time of field survey.				
Sampling strategies reported?	Yes	Sixty randomly selected households from each VDC form the sample for the household survey.<br>[...]<br>Out of the total 240 households covered in 2010 field survey, 58 households in Chitwan, 56 households in Makwanpur, 54 household in Dhading, and 53 households in Gorkha could be revisited in 2011 survey;				
Sampling sizes reported?	Yes	Sixty randomly selected households from each VDC form the sample for the household survey.<br>[...]<br>Out of the total 240 households covered in 2010 field survey, 58 households in Chitwan, 56 households in Makwanpur, 54 household in Dhading, and 53 households in Gorkha could be revisited in 2011 survey; thus the final sample constitutes a total of 221 households.				
Data analysis methods reported?	Yes	Having chosen the suitable indicators, now these need to be normalized so as to bring<br>the values of the indicators within the comparable range (Nelson, et al., 2010b; Gbetibouo & Ringler, 2009; Vincent, 2004). Normalization is done by subtracting the mean from the observed value and dividing by the standard deviation for each indicator.<br>$Normalized\ value\ = Observed\ Value\ /\ Mean\ standard\ deviation$<br>Next, weights should be assigned to these indicators.<br>[...]<br><br>The normalized variables are then multiplied with the assigned weights to construct the indices (for exposure, sensitivity, and adaptive capacity separately) using the following formulae:<br>$Ij = \sum^{k}_{i=1} b_i[(a_{ji} – x_i)/s_i]$<br>where, 'I' is the respective index value, 'b' is the loadings from first component from PCA (PCA1) taken as weights for respective indicators, 'a' is the indicator value, 'x' is the mean indicator value, and 's' is the standard deviation of the indicators. Finally, vulnerability index for each household is calculated as: V = E + S – AC, where, V is the vulnerability				

		index, E the exposure index, S is the sensitivity index and AC is the adaptive capacity index for respective household.				

**Structured summary of operationalization – validity assessment**			**1.1 DCM Appropriate**	**1.2 valid empirical rep?**	**1. conclusion - Valid?**	**2. Feasible?**
**Construct:** Social Capital						
**Article:** Piya et al (2012)						
Criterion	Assessment	Quoted text or Rationale for negative assessment				
Construct defined?	Yes	Ellis (2000) and DFID (1999)	Yes/ no/ can't tell	Yes/ no/ can't tell	Yes/ no/ can't tell<br>YES	Yes/ no/ can't tell<br>YES
Data collection methods reported?	Yes	This study is based on the primary data collected by household survey conducted in<br>two phases. The first phase of household survey was conducted in February-March 2010 and the second phase in May-June 2011.	YES			
Reporting of indicators/questions used to operationalise construct?	Yes	Finally, social asset is represented by the number of membership in formal<br>community based organizations (CBOs) and access to credit. Membership in CBOs will improve the households' social networks and access to information through their constant contact with the outsiders during the meetings in CBOs. Also, management of resources like water collection tanks and forests is done jointly by the members of these CBOs. Such activities help in pooling risks across the households in a community. Access to credit is also taken as social assets because for the Chepangs, taking loans from social contacts is one of the most important strategies to cope with seasonal food shortages, which they repay by selling agricultural produce, livestock, or forest products. Thus, access to credits in this community is equivalent to the social safety nets against all types of shocks. Also, some semi-formal saving and credit organizations in the community have recently started providing interest-free loans for productive investment like vegetable farming, and rearing cattle. Thus, access to productive loans denotes the access of the households to existing credit providing organizations in the locality. Better the access to credit, higher will be the adaptive capacity of the households.		YES,<br>MEH		

Component Indicators	Description of the Indicators	Unit	Hypothesized relation
Social Assets	Memberships in CBOs	Number	+
	Access to credit (1 = needed, but no access; 2 = credit used only for subsistence purposes; 3 = credit used for productive investment +/- subsistence; 4 = no need)	Ordinal Value	+

Sampling strategies reported?	Yes	Sixty randomly selected households from each VDC form the sample for the household survey.<br>[...]<br>Out of the total 240 households covered in 2010 field survey, 58 households in Chitwan, 56 households in Makwanpur, 54 household in Dhading, and 53 households in Gorkha could be revisited in 2011 survey;				
Sampling sizes reported?	Yes	Sixty randomly selected households from each VDC form the sample for the household survey.<br>[...]<br>Out of the total 240 households covered in 2010 field survey, 58 households in Chitwan, 56 households in Makwanpur, 54 household in Dhading, and 53 households in Gorkha could be revisited in 2011 survey; thus the final sample constitutes a total of 221 households.				
Data analysis methods reported?	Yes	Having chosen the suitable indicators, now these need to be normalized so as to bring<br>the values of the indicators within the comparable range (Nelson, et al., 2010b; Gbetibouo & Ringler, 2009; Vincent, 2004). Normalization is done by subtracting the mean from the observed value and dividing by the standard deviation for each indicator.<br>$Normalized\ value\ = Observed\ Value\ /\ Mean\ standard\ deviation$				

		Next, weights should be assigned to these indicators.<br>[...]<br>Assigning weight by Principal Component Analysis (PCA) following Filmer and Pritchett (2001) is thus preferred compared to the former two methods (Nelson et al., 2010b; Gbetibouo & Ringler, 2009; Cutter, Boruff, & Shirley, 2003). PCA was run for the selected indicators of exposure, sensitivity, and adaptive capacity separately in Data Analysis and Statistical Software (STATA10) software for assigning the weights. The loadings from the first component of PCA are used as the weights for the indicators. The weights assigned for each indicator varies between -1 and +1, sign of the indicators denoting the direction of relationship with other indicators used to construct the respective index. The magnitude of the weights describes the contribution of each indicator to the value of the index. PCA was run separately for the indicators of exposure, sensitivity and adaptive capacity. Stepwise PCA was run for the indicators of adaptive capacity. The first-step PCA was run for the indicators of each asset group separately to observe the relative importance of indicators within each asset category. From the weights obtained from first-step PCA, individual index values for each asset type was calculated. Second-step PCA was run using the index values for each of the five asset types to analyze which asset group contributes the most to the total adaptive capacity. Overall adaptive capacity index was calculated using the weights (loadings) obtained from the second step PCA run for the five asset categories.<br>The normalized variables are then multiplied with the assigned weights to construct the indices (for exposure, sensitivity, and adaptive capacity separately) using the following formulae:<br>$Ij = \sum^{k}_{i=1} b_i[(a_{ji} – x_i)/s_i]$<br>where, 'I' is the respective index value, 'b' is the loadings from first component from PCA (PCA1) taken as weights for respective indicators, 'a' is the indicator value, 'x' is the mean indicator value, and 's' is the standard deviation of the indicators. Finally, vulnerability index for each household is calculated as: V = E + S – AC, where, V is the vulnerability index, E the exposure index, S is the sensitivity index and AC is the adaptive capacity index for respective household.				

Transparency Assessment Article summary	
**Article**	Sarris & Karfakis (2010)
**Transparent operationalizations**	Covariate shocks; household consumption; idiosyncratic shocks
**Partially transparent**	
**Not transparent**	

**Structured summary of operationalization – validity assessment**			**1.1 DCM Appropriate**	**1.2 valid empirical rep?**	**1. conclusion - Valid?**	**2. Feasible?**
**Construct:** covariate shocks						
**Article:** Sarris & Karfakis (2010)						
Criterion	Assessment	Quoted text or Rationale for negative assessment				
Construct defined?	Yes	The proposed methodology complements the applications by Chaudhuri. et. al. (2002) and Christiaensen and Subbarao (2005), through the inclusion of covariate risks	Yes/ no/ can't tell	Yes/ no/ can't tell	Yes/ no/ can't tell<br><br>YES	Yes/ no/ can't tell<br><br>YES
Data collection methods reported?	Yes	The analysis of the paper will be based on a representative survey of 957 rural households in 45 villages done in the Kilimanjaro region, in November 2003, and a representative survey of 892 rural households in 36 villages done in the Ruvuma region in February-March 2004. The survey was repeated a year later<br>[...]<br>The questionnaire was designed to investigate the complete socio-economic characteristics of households with a particular emphasis on their vulnerability to a variety of risks.	YES			
Reporting of indicators/questions used to operationalise construct?	Yes	Table 2: Percentage of households affected by various shocks between 1999 and 2003, by region and status as cash crop grower or not.<br><br>**Health** | .. | ..<br>Death<br>Illness<br>**Climatic**		YES		

		<table><tr><td>Drought<br>Excessive rains</td><td></td><td></td></tr><tr><td>**Agricultural production**<br>Harvest loss<br>Livestock loss<br>Post harvest cereal loss</td><td></td><td></td></tr><tr><td>**Economic**<br>Cash crop price shock<br>Cereal price shock<br>Unemployment</td><td></td><td></td></tr><tr><td>**Property**<br>Theft<br>Fire/house destroyed<br>Land loss</td><td></td><td></td></tr></table>				
Sampling strategies reported?	Yes	based on a representative survey of 957 rural households in 45 villages done in the Kilimanjaro region, in November 2003, and a representative survey of 892 rural households in 36 villages done in the Ruvuma region in February-March 2004. The survey was repeated a year later for each region and was designed to be representative of rural farm households, and among them of cash crop (coffee in Kilimanjaro, coffee, tobacco and cashew nuts in Ruvuma) as well as non-cash crop producing households. The survey was not designed to sample the large-scale public and private coffee estates but only smallholders.				
Sampling sizes reported?	Yes	The analysis of the paper will be based on a representative survey of 957 rural households in 45 villages done in the Kilimanjaro region, in November 2003, and a representative survey of 892 rural households in 36 villages done in the Ruvuma region in February-March 2004.				
Data analysis methods reported?	Yes	Table 5 exhibits the results of the (instrumental variable) regressions on consumption and the squared residuals of consumption as per equations (15) and (17). The key variable for the vulnerability analysis is the coefficient in the consumption regressions of crop income per acre.				

		Concerning the consumption per equivalent adult, it can be seen that it depends positively and significantly on aggregate crop productivity, the size of land, the size of household, several wealth variables such as the lagged value of the number of animals owned and the lagged value of consumer durables, the age of the household head (significant in Ruvuma), access to credit variables, and some education variables.<br>The Durbin-Wu-Hausman test of the exogeneity of the crop productivity strongly rejects the hypothesis of exogeneity, so IV is appropriate. Table 6 presents the first stage regressions for the IV estimates. We use as instruments a variety of exogenous land characteristics, as well as weather shock variables, and lagged dummies for whether the farm household used fertilizer and chemicals, as well as the lagged number of coffee and cashew trees. The Sargan test does not invalidate the use of these instruments.<br>It must be mentioned that in the consumption regressions the IV regression coefficient of crop income per acre is significantly larger in the IV regressions compared to the OLS estimates (the OLS estimates for these coefficients are 0.028 for Kilimanjaro and 0.174 for Ruvuma, compared to 0.144 and 0.411 for the IV regressions in table 5 for the two regions).<br>The consumption regressions explain about 47 and 51 percent of the variance of consumption in Kilimanjaro and Ruvuma respectively. The regressions of the squared residuals from the consumption regressions on the same explanatory variables as the ones in the consumption regressions (excluding the variables that are related to covariate and idiosyncratic shocks) reveal that fewer of the variables are significant. In Kilimanjaro the dependency ratio, the value of the dwelling, the number of small animals, and the membership in a social group are significant, while in Ruvuma, the only two significant variables are the dummies for whether the household receives remittances and whether the household has easy				

		access to seasonal credit. The regressions explain a rather small proportion of the error less than 10 percent in both regions). This suggests that unexplained components of consumption variability dominate any parts that maybe due to structural household specific factors.<br>Tables 7 and 8 indicate the average vulnerability index in Kilimanjaro and Ruvuma by district, along with the proportions of the variance of consumption that are due to covariate factors, the average consumption per capita and the average headcount measures of poverty rates in both years of the survey. The first observation is that average vulnerability in Kilimanjaro is much lower than in Ruvuma (31 percent versus 60 percent). This is in line with the much larger poverty incidence in Ruvuma compared to Kilimanjaro that was indicated earlier (63.3 percent versus 39.5 percent).				

**Structured summary of operationalization – validity assessment**			**1.1 DCM Appropriate**	**1.2 valid empirical rep?**	**1. conclusion - Valid?**	**2. Feasible?**
**Construct:** household consumption						
**Article:** Sarris & Karfakis (2010)						
Criterion	Assessment	Quoted text or Rationale for negative assessment				
Construct defined?	Yes	consumption falling below a poverty threshold (Christiaensen and Subbarao 2004, Chaudhuri, et. al. 2002)	Yes/ no/ can't tell	Yes/ no/ can't tell	Yes/ no/ can't tell<br>YES	Yes/ no/ can't tell<br><br>YES
Data collection methods reported?	Yes	The analysis of the paper will be based on a representative survey of 957 rural households in 45 villages done in the Kilimanjaro region, in November 2003, and a representative survey of 892 rural households in 36 villages done in the Ruvuma region in February-March 2004. The survey was repeated a year later<br>[...]<br>The questionnaire was designed to investigate the complete socio-economic characteristics of households with a particular emphasis on their vulnerability to a	YES			

		variety of risks.				
Reporting of indicators/questions used to operationalise construct?	Yes	Table 1: General characteristics of rural households in Kilimanjaro and Ruvuma<br>Annual per capita total expenditure<br>Annual per capita total income		YES		
Sampling strategies reported?	Yes	based on a representative survey of 957 rural households in 45 villages done in the Kilimanjaro region, in November 2003, and a representative survey of 892 rural households in 36 villages done in the Ruvuma region in February-March 2004. The survey was repeated a year later for each region and was designed to be representative of rural farm households, and among them of cash crop (coffee in Kilimanjaro, coffee, tobacco and cashew nuts in Ruvuma) as well as non-cash crop producing households. The survey was not designed to sample the large-scale public and private coffee estates but only smallholders.				
Sampling sizes reported?	Yes	The analysis of the paper will be based on a representative survey of 957 rural households in 45 villages done in the Kilimanjaro region, in November 2003, and a representative survey of 892 rural households in 36 villages done in the Ruvuma region in February-March 2004.				
Data analysis methods reported?	Yes	Table 5 exhibits the results of the (instrumental variable) regressions on consumption and the squared residuals of consumption as per equations (15) and (17). The key variable for the vulnerability analysis is the coefficient in the consumption regressions of crop income per acre. Concerning the consumption per equivalent adult, it can be seen that it depends positively and significantly on aggregate crop productivity, the size of land, the size of household, several wealth variables such as the lagged value of the number of animals owned and the lagged value of consumer durables, the age of the household head (significant in Ruvuma), access to credit variables, and some education variables.<br>The Durbin-Wu-Hausman test of the exogeneity of the crop productivity strongly rejects the hypothesis of				

		exogeneity, so IV is appropriate. Table 6 presents the first stage regressions for the IV estimates. We use as instruments a variety of exogenous land characteristics, as well as weather shock variables, and lagged dummies for whether the farm household used fertilizer and chemicals, as well as the lagged number of coffee and cashew trees. The Sargan test does not invalidate the use of these instruments.<br>It must be mentioned that in the consumption regressions the IV regression coefficient of crop income per acre is significantly larger in the IV regressions compared to the OLS estimates (the OLS estimates for these coefficients are 0.028 for Kilimanjaro and 0.174 for Ruvuma, compared to 0.144 and 0.411 for the IV regressions in table 5 for the two regions).<br>The consumption regressions explain about 47 and 51 percent of the variance of consumption in Kilimanjaro and Ruvuma respectively. The regressions of the squared residuals from the consumption regressions on the same explanatory variables as the ones in the consumption regressions (excluding the variables that are related to covariate and idiosyncratic shocks) reveal that fewer of the variables are significant. In Kilimanjaro the dependency ratio, the value of the dwelling, the number of small animals, and the membership in a social group are significant, while in Ruvuma, the only two significant variables are the dummies for whether the household receives remittances and whether the household has easy access to seasonal credit. The regressions explain a rather small proportion of the error less than 10 percent in both regions). This suggests that unexplained components of consumption variability dominate any parts that maybe due to structural household specific factors.<br>Tables 7 and 8 indicate the average vulnerability index in Kilimanjaro and Ruvuma by district, along with the proportions of the variance of consumption that are due to covariate factors, the average consumption per capita and the average headcount measures of poverty rates in				

		both years of the survey. The first observation is that average vulnerability in Kilimanjaro is much lower than in Ruvuma (31 percent versus 60 percent). This is in line with the much larger poverty incidence in Ruvuma compared to Kilimanjaro that was indicated earlier (63.3 percent versus 39.5 percent).				

**Structured summary of operationalization – validity assessment**			**1.1 DCM Appropriate**	**1.2 valid empirical rep?**	**1. conclusion - Valid?**	**2. Feasible?**
**Construct:** idiosyncratic shocks						
**Article:** Sarris & Karfakis (2010)						
Criterion	Assessment	Quoted text or Rationale for negative assessment				
Construct defined?	Yes	Chistiaensen and Subbarao (2005) included covariate as well as idiosyncratic shocks	Yes/ no/ can't tell	Yes/ no/ can't tell	Yes/ no/ can't tell<br>YES	Yes/ no/ can't tell<br>YES
Data collection methods reported?	Yes	The analysis of the paper will be based on a representative survey of 957 rural households in 45 villages done in the Kilimanjaro region, in November 2003, and a representative survey of 892 rural households in 36 villages done in the Ruvuma region in February-March 2004. The survey was repeated a year later<br>[...]<br>The questionnaire was designed to investigate the complete socio-economic characteristics of households with a particular emphasis on their vulnerability to a variety of risks.	YES			
Reporting of indicators/questions used to operationalise construct?	Yes	Table 2: Percentage of households affected by various shocks between 1999 and 2003, by region and status as cash crop grower or not.<br><br>**Health** | .. | ..<br>Death<br>Illness<br>**Climatic**<br>Drought<br>Excessive rains		YES		

		**Agricultural production**<br>Harvest loss<br>Livestock loss<br>Post harvest cereal loss<br>**Economic**<br>Cash crop price shock<br>Cereal price shock<br>Unemployment<br>**Property**<br>Theft<br>Fire/house destroyed<br>Land loss				
Sampling strategies reported?	Yes	based on a representative survey of 957 rural households in 45 villages done in the Kilimanjaro region, in November 2003, and a representative survey of 892 rural households in 36 villages done in the Ruvuma region in February-March 2004. The survey was repeated a year later for each region and was designed to be representative of rural farm households, and among them of cash crop (coffee in Kilimanjaro, coffee, tobacco and cashew nuts in Ruvuma) as well as non-cash crop producing households. The survey was not designed to sample the large-scale public and private coffee estates but only smallholders.				
Sampling sizes reported?	Yes	The analysis of the paper will be based on a representative survey of 957 rural households in 45 villages done in the Kilimanjaro region, in November 2003, and a representative survey of 892 rural households in 36 villages done in the Ruvuma region in February-March 2004.				
Data analysis methods reported?	Yes	Table 5 exhibits the results of the (instrumental variable) regressions on consumption and the squared residuals of consumption as per equations (15) and (17). The key variable for the vulnerability analysis is the coefficient in the consumption regressions of crop income per acre. Concerning the consumption per equivalent adult, it can be seen that it depends positively and significantly on				

		aggregate crop productivity, the size of land, the size of household, several wealth variables such as the lagged value of the number of animals owned and the lagged value of consumer durables, the age of the household head (significant in Ruvuma), access to credit variables, and some education variables.<br>The Durbin-Wu-Hausman test of the exogeneity of the crop productivity strongly rejects the hypothesis of exogeneity, so IV is appropriate. Table 6 presents the first stage regressions for the IV estimates. We use as instruments a variety of exogenous land characteristics, as well as weather shock variables, and lagged dummies for whether the farm household used fertilizer and chemicals, as well as the lagged number of coffee and cashew trees. The Sargan test does not invalidate the use of these instruments.<br>It must be mentioned that in the consumption regressions the IV regression coefficient of crop income per acre is significantly larger in the IV regressions compared to the OLS estimates (the OLS estimates for these coefficients are 0.028 for Kilimanjaro and 0.174 for Ruvuma, compared to 0.144 and 0.411 for the IV regressions in table 5 for the two regions).<br>The consumption regressions explain about 47 and 51 percent of the variance of consumption in Kilimanjaro and Ruvuma respectively. The regressions of the squared residuals from the consumption regressions on the same explanatory variables as the ones in the consumption regressions (excluding the variables that are related to covariate and idiosyncratic shocks) reveal that fewer of the variables are significant. In Kilimanjaro the dependency ratio, the value of the dwelling, the number of small animals, and the membership in a social group are significant, while in Ruvuma, the only two significant variables are the dummies for whether the household receives remittances and whether the household has easy access to seasonal credit. The regressions explain a rather small proportion of the error less than 10 percent in both				

		regions). This suggests that unexplained components of consumption variability dominate any parts that maybe due to structural household specific factors.<br>Tables 7 and 8 indicate the average vulnerability index in Kilimanjaro and Ruvuma by district, along with the proportions of the variance of consumption that are due to covariate factors, the average consumption per capita and the average headcount measures of poverty rates in both years of the survey. The first observation is that average vulnerability in Kilimanjaro is much lower than in Ruvuma (31 percent versus 60 percent). This is in line with the much larger poverty incidence in Ruvuma compared to Kilimanjaro that was indicated earlier (63.3 percent versus 39.5 percent).				

Transparency Assessment Article summary	
**Article**	Sietz et al (2012)
**Transparent operationalizations**	Adaptive capacity; cluster pattern analysis; exposure; food security; sensitivity
**Partially transparent**	
**Not transparent**	

**Structured summary of operationalization – validity assessment**			**1.1 DCM Appropriate**	**1.2 valid empirical rep?**	**1. conclusion - Valid?**	**2. Feasible?**
**Construct:** adaptive capacity						
**Article:** Sietz et al (2012)						
Criterion	Assessment	Quoted text or Rationale for negative assessment				
Construct defined?	Yes	the adaptive capacity of smallholders (the term as used in this study encompasses the coping capacity) describes the ability to adjust to weather extremes, manage damages or explore alternative livelihood opportunities.	Yes/ no/ can't tell	Yes/ no/ can't tell	Yes/ no/ can't tell CAN'T TELL	Yes/ no/ can't tell
Data collection methods reported?	2ndary data	The ALTAGRO (2006) data base contains detailed quantitative information for 527 smallholder households collected through household questionnaires.	CAN'T TELL			
Reporting of indicators/questions used to operationalise construct?	Yes	Ten categories describe the smallholder households covering personal information about the family members (e.g. occupation, education level, age), production systems (e.g. crop and livestock assets, labour input, processing and commer- cialisation of produce), weather conditions, food reserves, income, some expenses and credits.<br>[...]<br>The following data are taken from the ALTAGRO (2006) data base to indicate the mechanisms relevant in this study. As the first dimension, the harvest failure risk is indicated by the number of production zones used for crop and pasture cultivation. The indicator considers plains, hillsides and hills. The second dimension of the area con- straint is measured by the crop area as an		YES		

		important pre- requisite for food production. The pasture area highly correlates to livestock keeping and is therefore reflected in the livestock measure. The third dimension, the livestock constraint, is characterised by the number and types of animals. To compare various animal species, we calculated standardised livestock units in relation to an improved cattle variety based on the livestock-specific metabolism (Kleiber 1961). Average livestock weights were estimated using 20 representative animals of each species in the study region. Since fodder production is an essential condition for livestock keeping, the respective indicator contains a reference to the area and productivity of pasture land. Furthermore, the productivity constraint as the fourth dimension is provided for the major food crops potatoes and quinua. It averages the household's productivity across species, varieties and production zones for each crop. Again, we concentrate on food crops since the productivity of pastures is already included in the livestock measure. The fifth dimension of education deprivation relates to the number of years that a household head attended school. School attendance is classified according to the four levels: no formal education, primary, secondary and higher edu- cation. Finally, the lack of alternative income as the sixth dimension is quantified by the sum of annual monetary income from local off-farm activities and remittances. People usually receive remittances from household<br>495<br>members who migrate for climate-independent labour, for example mining and commerce. Table 1 summarises the indicators used to assess vulnerability.<br>[...]<br>Table 1 Indicators of households' sensitivity and adaptive capacity. The range of the area and livestock constraints as well as lack of alternative income is provided following winsorisation, see description in text. (Data source: ALTAGRO 2006)				

<table>
<tr><td></td><td></td><td><table>
<tr><td>Dimension of sensitivity and adaptive capacity</td><td>Indicator</td><td>Range</td></tr>
<tr><td>Harvest failure risk</td><td>Number of production zones used for cultivation</td><td>1–3</td></tr>
<tr><td>Area constraint</td><td>Crop area</td><td>0.1–1.3 ha/person[a]</td></tr>
<tr><td>Livestock constraint</td><td>Livestock units</td><td>0.1–8.0 livestock units/person</td></tr>
<tr><td>Productivity constraint</td><td>Potato productivity
Quinua productivity</td><td>0.1–10.0 t/ha
0.2–1.8 t/ha</td></tr>
<tr><td>Education deprivation</td><td>Education level of household head</td><td>1–4</td></tr>
<tr><td>Lack of alternative income</td><td>Local off-farm income and remittances</td><td>0–2400 Soles/year*person</td></tr>
<tr><td colspan="3">a Average: 4 persons per household</td></tr>
</table></td><td></td><td></td><td></td><td></td></tr>
<tr><td>Sampling strategies reported?</td><td>Yes</td><td>The households were randomly selected in four areas across the administrative Region of Puno reflecting representative smallholder live- lihood conditions.</td><td></td><td></td><td></td><td></td></tr>
<tr><td>Sampling sizes reported?</td><td>Yes</td><td>527 smallholder households</td><td></td><td></td><td></td><td></td></tr>
<tr><td>Data analysis methods reported?</td><td>Yes</td><td>In preparing the further analysis, we adjusted data sets with only a few extreme values to increase the influence of these data sets on the cluster partitions. For example, the majority of households possess eight or fewer units of livestock. The few households with up to 39 livestock units can be formally interpreted as single outliers which skew the overall data distribution of this indicator. To deskew such data sets and thus adequately focus on the majority of households, we winsorised the data sets, i.e.,</td><td></td><td></td><td></td><td></td></tr>
</table>

		replaced the outlying observations (4%) with the next available less extreme observation (Barnett and Lewis 1994). This pro- cedure was applied to the area and livestock constraints as well as the alternative income. All indicators were then normalised to a 0–1 range using the minimum–maximum values. Prior to the cluster analysis, we determined correlations<br>between the selected indicators and the variance distribu- tion in the data space. Firstly, the correlation coefficients reached average absolute values of 0.11. The crop area and livestock units correlate most strongly here (0.46) reflec- ting the mixed production systems. Furthermore, variables showing a large variance may be intuitively expected to contain most of the structure information. Therefore, we explored the variance of the selected indicators using a principal component analysis (PCA). The PCA was per- formed using the open source statistics package R (RDCT 2009) following standard procedure based on Pearson correlations.				

**Structured summary of operationalization – validity assessment**			**1.1 DCM Appropriate**	**1.2 valid empirical rep?**	**1. conclusion - Valid?**	**2. Feasible?**
**Construct:** cluster pattern analysis						
**Article:** Sietz et al (2012)						
<u>Criterion</u>	<u>Assessment</u>	<u>Quoted text or Rationale for negative assessment</u>				
Construct defined?	Yes	Without such a pre-selection, alternative approaches investigate the structure of the data space spanned by selected vulnerability indicators using cluster analysis. They deliver useful insights into recurrent indicator com- binations based on similarities among units of analysis, in cases where such a grouping exists. For example, clustering revealed typical livelihood strategies employed by small- holders in Mexico and Botswana (Eakin 2005; Sallu et al. 2010).	Yes/ no/ can't tell	Yes/ no/ can't tell	Yes/ no/ can't tell CAN'T TELL	Yes/ no/ can't tell
Data collection methods reported?	2ndary data	The ALTAGRO (2006) data base contains detailed<br>quantitative information for 527 smallholder households collected through household questionnaires.<br>[...]	CAN'T TELL			

		The necessary weather information is available in good quality for the 1996–2006 period for two stations located in Puno and Cabanillas (see Fig. 1). Table 2 shows the average pre- cipitation and temperature for both stations.				
Reporting of indicators/questions used to operationalise construct?	Yes	Ten categories describe the smallholder households covering personal information about the family members (e.g. occupation, education level, age), production systems (e.g. crop and livestock assets, labour input, processing and commer- cialisation of produce), weather conditions, food reserves, income, some expenses and credits.<br>[...]<br>The following data are taken from the ALTAGRO<br>(2006) data base to indicate the mechanisms relevant in this study. As the first dimension, the harvest failure risk is indicated by the number of production zones used for crop and pasture cultivation. The indicator considers plains, hillsides and hills. The second dimension of the area con- straint is measured by the crop area as an important pre- requisite for food production. The pasture area highly correlates to livestock keeping and is therefore reflected in the livestock measure. The third dimension, the livestock constraint, is characterised by the number and types of animals. To compare various animal species, we calculated standardised livestock units in relation to an improved cattle variety based on the livestock-specific metabolism (Kleiber 1961). Average livestock weights were estimated using 20 representative animals of each species in the study region. Since fodder production is an essential condition for livestock keeping, the respective indicator contains a reference to the area and productivity of pasture land. Furthermore, the productivity constraint as the fourth dimension is provided for the major food crops potatoes and quinua. It averages the household's productivity across species, varieties and production zones for each crop. Again, we concentrate on food crops since the productivity of pastures is already included in the livestock measure. The fifth dimension of education deprivation relates to the number of years that a household head attended school. School attendance is classified according to the four levels: no formal education, primary, secondary and higher edu- cation. Finally, the lack of alternative income as the sixth dimension is quantified by the sum of annual monetary income from local off-farm activities and remittances. People usually receive remittances from household<br>495<br>members who migrate for climate-independent labour, for example mining and commerce. Table 1 summarises the indicators used to assess vulnerability.<br>[...]<br>Table 1 Indicators of households' sensitivity and adaptive capacity. The range of the area and livestock constraints as well as lack of alternative income is provided following		CAN'T TELL		

		winsorisation, see description in text. (Data source: ALTAGRO 2006)					

Dimension of sensitivity and adaptive capacity	*Indicator*	*Range*
Harvest failure risk	Number of production zones used for cultivation	1–3
Area constraint	Crop area	0.1–1.3 ha/person[a]
Livestock constraint	Livestock units	0.1–8.0 livestock units/person
Productivity constraint	Potato productivity<br>Quinua productivity	0.1–10.0 t/ha<br>0.2–1.8 t/ha
Education deprivation	Education level of household head	1–4
Lack of alternative income	Local off-farm income and remittances	0–2400 Soles/year*person
a Average: 4 persons per household		

[...]
The necessary weather information is available in good quality for the 1996–2006 period for two stations located in Puno and Cabanillas (see Fig. 1). Table 2 shows the average pre- cipitation and temperature for both stations.
[...]

Table 2 Mean precipitation and temperature for 1996–2006 at Puno and Cabanillas stations (Data source: Servicio Nacional de Meteorologı´ae Hidrologı´a del Peru´, SENAMHI)

	Mean values for 1996–2006												
	Jan	Fe b	Ma r	A pr	Ma y	Ju n	Ju l	Au g	Se pt	Oc t	No v	De c	Tot al

Precipitation (mm)													
Puno	201	161	138	60	7	3	4	14	27	51	48	88	801
Cabanillas	166	165	112	56	6	1	3	11	19	54	55	91	738
Mean temperature (°C)													
Puno	10.8	10.7	10.6	9.7	8.1	6.8	6.8	7.9	9.3	10.4	11.0	11.5	9.5
Cabanillas	10.6	10.5	10.5	9.8	8.6	7.3	6.9	8.1	9.6	10.6	11.1	11.3	9.6
Minimum temperature (°C)													
Puno	5.7	5.8	5.4	3.8	0.8	-0.9	-1.1	0.4	1.9	3.6	4.3	5.4	2.9
Cabanillas	5.3	5.5	5.2	3.7	1.1	-0.8	-1.5	0.3	2.1	3.7	4.2	5.1	2.8

Sampling strategies reported?	2ndary data					
Sampling sizes reported?	2ndary data					
Data analysis methods reported?	Yes	The cluster analysis was performed using a sequence of a common hierarchical and exchange algorithm, i.e., hclust and kmeans, using the statistics package R (MacQueen 1967; RDCT 2009). Based on stochastic initialisation, we calculated the reproducibility of partitions for a pre-given number of clusters to determine whether the algorithm detects stable or unstable (inappropriate) partitions. The share of households that were categorised in the same cluster in two partitions is expressed as ‘‘consistency measure’’. The higher this measure, the more reliable the cluster results. We calculated the consistency measure as the average of 200 pairwise comparisons of partitions with				

		a given number of clusters. Ultimately, the consistency measure enables us to identify the optimal number of clusters to be analysed. Further methodological details are outlined in a previous application of the cluster approach to dryland vulnerability on a global scale (Sietz et al. 2011).				

**Structured summary of operationalization – validity assessment**			**1.1 DCM Appropriate**	**1.2 valid empirical rep?**	**1. conclusion - Valid?**	**2. Feasible?**
**Construct:** Exposure						
**Article:** Sietz et al (2012)						
Criterion	Assessment	Quoted text or Rationale for negative assessment				
Construct defined?	Yes	expo- sure, sensitivity and coping/adaptive capacity (IPCC 2007).	Yes/ no/ can't tell	Yes/ no/ can't tell	Yes/ no/ can't tell	Yes/ no/ can't tell
Data collection methods reported?	2ndary data	The necessary weather information is available in good quality for the 1996–2006 period for two stations located in Puno and Cabanillas (see Fig. 1). Table 2 shows the average pre- cipitation and temperature for both stations.	YES		YESS	YES, WHEN DATA IS AVAILABLE
Reporting of indicators/questions used to operationalise construct?	2ndary data	The necessary weather information is available in good quality for the 1996–2006 period for two stations located in Puno and Cabanillas (see Fig. 1). Table 2 shows the average pre- cipitation and temperature for both stations. [...]		YES		

Table 2 Mean precipitation and temperature for 1996–2006 at Puno and Cabanillas stations (Data source: Servicio Nacional de Meteorologı´ae Hidrologı´a del Peru´, SENAMHI)

	Mean values for 1996–2006												
	Jan	Feb	Mar	Apr	May	Jun	Jul	Aug	Sept	Oct	Nov	Dec	Total
Precipitation (mm)													
Puno	201	161	138	60	7	3	4	14	27	51	48	88	801
Cabanillas	166	165	112	56	6	1	3	11	19	54	55	91	738

Mean temperature (°C)													
Puno	10.8	10.7	10.6	9.7	8.1	6.8	6.8	7.9	9.3	10.4	11.0	11.5	9.5
Cabanillas	10.6	10.5	10.5	9.8	8.6	7.3	6.9	8.1	9.6	10.6	11.1	11.3	9.6
Minimum temperature (°C)													
Puno	5.7	5.8	5.4	3.8	0.8	-0.9	-1.1	0.4	1.9	3.6	4.3	5.4	2.9
Cabanillas	5.3	5.5	5.2	3.7	1.1	-0.8	-1.5	0.3	2.1	3.7	4.2	5.1	2.8

Sampling strategies reported?	2ndary data					
Sampling sizes reported?	2ndary data					
Data analysis methods reported?	Yes	To make the two stations comparable, we determined relative anomalies compared to the average precipitation course over the period 1996–2006 through precipitation ranking. This ranking was then used to identify driest and wettest periods which caused production damage. Since soil water content integrates previous precipitation events to some extent, we cumulated the daily precipitation records in a 20-day window. This window was moved as a running mean by steps of one decade (10 days). This choice is supported by the calibration campaign 2003/2004 descri- bed below. Covering the rainy season from December to March, we obtained cumulated precipitation values for 12 time segments (Fig. 2). This number of time segments still allows for sufficient resolution of intra-seasonal anomalies.<br>[...]<br>In conclusion, climate exposure was precipitation-driven<br>during the relevant campaigns. Similar precipitation and temperature conditions at both stations indicate a similar climate exposure throughout the study region. Therefore, a potential spatial variation in the exposure does not have to be considered				

		in the further vulnerability analysis.				

Structured summary of operationalization – validity assessment			1.1 DCM Appropriate	1.2 valid empirical rep?	1. conclusion - Valid?	2. Feasible?
**Construct:** food security						
**Article:** Sietz et al (2012)						
Criterion	Assessment	Quoted text or Rationale for negative assessment				
Construct defined?	Yes	Food security is often discussed in terms of four dimensions: food availability, access, stability of supply/ access and utilisation (FAO 2000).	Yes/ no/ can't tell	Yes/ no/ can't tell	Yes/ no/ can't tell YES	Yes/ no/ can't tell<br><br>YES
Data collection methods reported?	Yes	Therefore, we conducted a Household Validation Survey (HVS) in collaboration with CIRNMA technicians.	YES			
Reporting of indicators/questions used to operationalise construct?	Yes	We collected data on the purchase of food and fodder in 2005/2006 including monetary and in-kind exchange. The purchase was considered in relation to an average year to compare households in a standardised way. The average year indicates the necessary purchase which complements the household's production and reserves to maintain the average nutritional status. We assume that changes in 2005/2006 were primarily caused by the iden- tified weather extremes given that the productive resources and agricultural management are relatively stable over time. As smallholders do not maintain records of their pur-<br>chase, the data collection drew on their memory recall. This approach provides good estimates in the absence of other reliable data sources, though some limitations need to be considered. Most importantly, this method does not account for memory biases. To reduce such biases, the survey referred to the purchase of a specific crop in a given year. Firstly, smallholders were asked to reflect on thecroptheyharvested last,startingwiththe previous campaign and successively moving backwards to the 2005/2006 campaign. This part of the survey was con- ducted with the aid of an abacus. Starting with the given number of 10 beads indicating the average purchase, household heads or other adult family members removed or added beads to quantify their relative purchase in 2005/2006. The survey considered the five major food and fodder crops: potatoes, quinua, broad beans, barley and oat. The second part of the HVS focused on		YES		

		information<br>about aspects of the smallholder livelihoods that help explain important causes for differences in purchase to support the interpretation and validation of the vulnera- bility clusters. This part involved semi-structured inter- views exploring effects of weather extremes on the smallholders' livelihoods, access to land, production zones and income, availability of labour as well as social and economic opportunities to cope with production failure. Overall, each interview took around 45 min and was car- ried out in Spanish or Quechua according to the native language of the interviewees.				
Sampling strategies reported?	Yes	It was carried out in 33 ran- domly chosen households (12%) in February 2009. The engagement of local smallholders is a key component of this study. They are considered a necessary information source for providing details on the local conditions of climate sensitivity as well as constraints and opportunities for coping with adverse effects.				
Sampling sizes reported?	Yes	It was carried out in 33 ran- domly chosen households (12%) in February 2009.				
Data analysis methods reported?	Yes	Recognising the sensitivity of any vulnerability analysis<br>to the choice of indicators, we empirically examine whe- ther the formal entities provide specific evidence about damages under the identified climate exposure. For this, the data on households' purchase collected in the HVS are related to the cluster membership of households. Figure 4 shows that each cluster corresponds to a relatively small range of the damage measure. Therefore, the similarities among the households revealed by the cluster analysis hold true with regard to the outcomes of the climate exposure.				

**Structured summary of operationalization – validity assessment**			**1.1 DCM Appropriate**	**1.2 valid empirical rep?**	**1. conclusion - Valid?**	**2. Feasible?**
**Construct:** Sensitivity						
**Article:** Sietz et al (2012)						
Criterion	Assessment	Quoted text or Rationale for negative assessment				
Construct defined?	Yes	We consider the effects of weather disturbance on the agricultural systems as sensitivity.	Yes/ no/ can't tell	Yes/ no/ can't tell	Yes/ no/ can't tell<br>CAN'T TELL	Yes/ no/ can't tell
Data collection	2ndary data	The ALTAGRO (2006) data base contains detailed	YES			

methods reported?		quantitative information for 527 smallholder households collected through household questionnaires.				
Reporting of indicators/questions used to operationalise construct?	Yes	Ten categories describe the smallholder households covering personal information about the family members (e.g. occupation, education level, age), production systems (e.g. crop and livestock assets, labour input, processing and commer- cialisation of produce), weather conditions, food reserves, income, some expenses and credits.<br>[...]<br>The following data are taken from the ALTAGRO (2006) data base to indicate the mechanisms relevant in this study. As the first dimension, the harvest failure risk is indicated by the number of production zones used for crop and pasture cultivation. The indicator considers plains, hillsides and hills. The second dimension of the area con- straint is measured by the crop area as an important pre- requisite for food production. The pasture area highly correlates to livestock keeping and is therefore reflected in the livestock measure. The third dimension, the livestock constraint, is characterised by the number and types of animals. To compare various animal species, we calculated standardised livestock units in relation to an improved cattle variety based on the livestock-specific metabolism (Kleiber 1961). Average livestock weights were estimated using 20 representative animals of each species in the study region. Since fodder production is an essential condition for livestock keeping, the respective indicator contains a reference to the area and productivity of pasture land. Furthermore, the productivity constraint as the fourth dimension is provided for the major food crops potatoes and quinua. It averages the household's productivity across species, varieties and production zones for each crop. Again, we concentrate on food crops since the productivity of pastures is already included in the livestock measure. The fifth dimension of education deprivation relates to the number of years that a household head attended school.		CAN'T TELL		

School attendance is classified according to the four levels: no formal education, primary, secondary and higher edu- cation. Finally, the lack of alternative income as the sixth dimension is quantified by the sum of annual monetary income from local off-farm activities and remittances. People usually receive remittances from household
495
members who migrate for climate-independent labour, for example mining and commerce. Table 1 summarises the indicators used to assess vulnerability.
[...]

Table 1 Indicators of households’ sensitivity and adaptive capacity. The range of the area and livestock constraints as well as lack of alternative income is provided following winsorisation, see description in text. (Data source: ALTAGRO 2006)

Dimension of sensitivity and adaptive capacity	*Indicator*	*Range*
Harvest failure risk	Number of production zones used for cultivation	1–3
Area constraint	Crop area	0.1–1.3 ha/person[a]
Livestock constraint	Livestock units	0.1–8.0 livestock units/person
Productivity constraint	Potato productivity Quinua productivity	0.1–10.0 t/ha 0.2–1.8 t/ha
Education deprivation	Education level of household head	1–4
Lack of	Local off-farm	0–2400

<table>
<tr><td></td><td></td><td><table><tr><td>alternative income</td><td>income and remittances</td><td>Soles/year*person</td></tr><tr><td colspan="3">a Average: 4 persons per household</td></tr></table></td><td></td><td></td><td></td><td></td></tr>
<tr><td>Sampling strategies reported?</td><td>Yes</td><td>The households were randomly selected in four areas across the administrative Region of Puno reflecting representative smallholder live- lihood conditions.</td><td></td><td></td><td></td><td></td></tr>
<tr><td>Sampling sizes reported?</td><td>Yes</td><td>527 smallholder households</td><td></td><td></td><td></td><td></td></tr>
<tr><td>Data analysis methods reported?</td><td>Yes</td><td>In preparing the further analysis, we adjusted data sets with only a few extreme values to increase the influence of these data sets on the cluster partitions. For example, the majority of households possess eight or fewer units of livestock. The few households with up to 39 livestock units can be formally interpreted as single outliers which skew the overall data distribution of this indicator. To deskew such data sets and thus adequately focus on the majority of households, we winsorised the data sets, i.e., replaced the outlying observations (4%) with the next available less extreme observation (Barnett and Lewis 1994). This pro- cedure was applied to the area and livestock constraints as well as the alternative income. All indicators were then normalised to a 0–1 range using the minimum–maximum values. Prior to the cluster analysis, we determined correlations
between the selected indicators and the variance distribu- tion in the data space. Firstly, the correlation coefficients reached average absolute values of 0.11. The crop area and livestock units correlate most strongly here (0.46) reflec- ting the mixed production systems. Furthermore, variables showing a large variance may be intuitively expected to contain most of the structure information. Therefore, we explored the variance of the selected indicators using a principal component analysis (PCA). The PCA was per- formed using the open source statistics package R (RDCT 2009) following standard procedure based on Pearson correlations.</td><td></td><td></td><td></td><td></td></tr>
</table>

**Transparency Assessment Article summary**	
**Article**	Tesso et al (2012)
**Transparent operationalizations**	Determinants of resilience; household level resilience
**Partially transparent**	
**Not transparent**	

**Structured summary of operationalization – validity assessment**			**1.1 DCM Appropriate**	**1.2 valid empirical rep?**	**1. conclusion - Valid?**	**2. Feasible?**
**Construct:** Determinants of resilience						
**Article:** Tesso et al (2012)						
Criterion	Assessment	Quoted text or Rationale for negative assessment				
Construct defined?	Yes	important determinants for resilience at household level in North Shewa zone of Ethiopia.	Yes/ no/ can't tell	Yes/ no/ can't tell	Yes/ no/ can't tell<br><br>YES	Yes/ no/ can't tell<br><br>YES
Data collection methods reported?	Yes	The data for the research was obtained from a survey of 452 farm households in three districts of the Zone in 2011/2012.<br>[...]<br>A structured ques- tionnaire was used to interview the farmers.<br>[...]<br>In addition, secondary data relevant for this analysis was obtained from the National Meteorological Service Agency (NMSA), Central Statistical Authority (CSA), and Zonal and district agricultural offices.<br>In order to understand the research questions at community level, qualitative data were collected through focused group discussion using checklist prepared for the purpose.	YESS			
Reporting of indicators/questions used to operationalise construct?	Yes	Data col- lected from the farmers include household character- istics, landholding, crops and livestock production, dis- aster occurrence, perception level (on precipitation, tem- perature, soil moisture, air moisture and wind direction), adaptation strategies pursued, different coping strategies pursued, level of resilience, and other		YES		

		relevant informa- tion.<br>[...]<br>Table 1. Social, economic and environmental vulnerability indicators for the study area.<br>**I. Social Vulnerability Variables**<br>Sex: Female headed<br>Education: illiterate and less than grade 2<br>Marital status: Single (including divorce and widow)<br>No. of relatives: relative to less than 5 households<br>No. institutions: Participation in less than 2.35 institutions<br>Dependency: High dependency of 4 person and more<br>Farm to farm ext: No access to farmer to farmer extension<br>Year Ag. Experience: Lack of farm experience if < 3 years<br>Access to indigenous early warning information: Having no access<br>**II. Economic Vulnerability Variables**<br>Livestock ownership: Own less than 2 tropical livestock unit<br>Access to information: Having no access to<br>Ownership of perennial crops: no area under perennial crops<br>Land size: own less than 0.5ha of land<br>Land fragmentation: own only one plots<br>Non-farm income: Have no non-farm income<br>Soil and water conservation structures: More than 50% is not conserved<br>Income level: Having less than minimum requirement<br>Consumption expenditure: Spending less than minimum requirement<br>Crop diversity: less than 50% of the 8 major crops grown in the area<br>Land under irrigation: no access to irrigation at all<br>Land under improved seed: area not covered with improved seed (average of high yielding, drought				

		tolerant, early maturing)<br>Land under commercial fertilizer: Having no access to fertilizer at all<br>Cash reserve: Having no cash saving at all<br>Food reserve: Having no food reserve for next year<br>Credit: Having no access to credit at all<br>**III. Environmental Vulnerability Variables (Measures of Sensitivity and Exposure)**<br>Land topography: Slope greater than 15% and 0% slope<br>Fertility: Poor fertility and cannot produce without heavy fertilizer use<br>Vegetation cover: Bare land<br>Frequency of hazards: People facing more than two natural hazards in a year<br>Rainfall: Receiving below average<br>Temperature: Experiencing above average<br>Change in wind direction: Encountering change in wind direction than usual				
Sampling strategies reported?	Yes	The specific study sites within the dis- tricts were selected based on a multi stage random sam- pling procedure. Consequently, 19 Kebeles were selected from which the sample households were selected ran- domly proportional to population size.				
Sampling sizes reported?	Yes	452 farm households in three districts of the Zone				
Data analysis methods reported?	Yes	Ordered probit regression model was used to iden-<br>tify and analyze the determinants of households' re-<br>silience to climate change induced shocks.<br>[...]<br>comparison was done based on certain defined characteristics. Thus, resilience in this measurment involved ordered out- come. This is with the basic hypothesis that a given natural shock will have differencial impact on house-holds' resilience.<br>$Y_j^* = X^1_jB + U_{1j}$				

		Y = 0 if Y^{*} _< 0<br>Y = 1 if 0 < Y^{*} _< 1<br>Y = 2 if 1 < Y^{*} _< 2<br>Y* is level of resilience and involves ordered outcome, that is Y = 0 was given to households taking more than two years to bounce back, Y = 1 was given households taking greater than one year and less than or equals to two years; and Y = 2, was given to households taking less than or equals to one year. The Xij are the explanatory variables determining the time taken to bounce back. The independent variables included in the model were avail- ability of food stock(dummy), income diversification (number of enterprises), number of plots, number of de- pendent family members, age of household head (years), access to credit (dummy), social capital (number of in- stitutional involvement), area under perennial crops (ha), preparedness (dummy), propensity to invest on natural resources (percentage of area under conservation), pro- pensity to save (percentage of saving), access to irriga- tion (ha), geographic locations (dummy), etc. βs are pa- rameters estimated and Uij is the disturbance term.				

**Structured summary of operationalization – validity assessment**			**1.1 DCM Appropriate**	**1.2 valid empirical rep?**	**1. conclusion - Valid?**	**2. Feasible?**
**Construct:** household level resilience						
**Article:** Tesso et al (2012)						
Criterion	Assessment	Quoted text or Rationale for negative assessment				
Construct defined?	Yes	According to DFID, resilience at community level is explained as the ability of countries, communities and households to manage change, by maintaining or transforming living standards in the face of shocks or stresses—such as earthquakes, drought or violent conflict—without compromising their long- term prospects [10]. Similarly, resilience is the ability of a social or ecological system to absorb disturbances while retaining the same basic structure and ways of functioning, the capacity for	Yes/ no/ can't tell	Yes/ no/ can't tell	Yes/ no/ can't tell YES	Yes/ no/ can't tell

		self-organization, and the capacity to adapt to stress and change. This is a measurement of community’s capacity to absorb external shocks. In the aftermath of occurrence of climate change induced shocks, how do farmer bounce back to normal livelihood is about the resilience level of farming com- munity. A resilient community is able to respond to changes or stress in a positive way, and is able to maintain its core functions as a community despite those stresses [11].				
Data collection methods reported?	Yes	The data for the research was obtained from a survey of 452 farm households in three districts of the Zone in 2011/2012.<br>[...]<br>A structured ques- tionnaire was used to interview the farmers.<br>[...]<br>In addition, secondary data relevant for this analysis was obtained from the National Meteorological Service Agency (NMSA), Central Statistical Authority (CSA), and Zonal and district agricultural offices.<br>In order to understand the research questions at community level, qualitative data were collected through focused group discussion using checklist prepared for the purpose.	YES			
Reporting of indicators/questions used to operationalise construct?	Yes	Data collected from the farmers include household characteristics, landholding, crops and livestock production, disaster occurrence, perception level (on precipitation, temperature, soil moisture, air moisture and wind direction), adaptation strategies pursued, different coping strategies pursued, level of resilience, and other relevant informa- tion.<br>[...]<br>Table 1. Social, economic and environmental vulnerability indicators for the study area.<br>**I. Social Vulnerability Variables**<br>Sex: Female headed<br>Education: illiterate and less than grade 2		YES		

			Marital status: Single (including divorce and widow)<br>No. of relatives: relative to less than 5 households<br>No. institutions: Participation in less than 2.35 institutions<br>Dependency: High dependency of 4 person and more<br>Farm to farm ext: No access to farmer to farmer extension<br>Year Ag. Experience: Lack of farm experience if < 3 years<br>Access to indigenous early warning information: Having no access				
			**II. Economic Vulnerability Variables**				
			Livestock ownership: Own less than 2 tropical livestock unit<br>Access to information: Having no access to<br>Ownership of perennial crops: no area under perennial crops<br>Land size: own less than 0.5ha of land<br>Land fragmentation: own only one plots<br>Non-farm income: Have no non-farm income<br>Soil and water conservation structures: More than 50% is not conserved<br>Income level: Having less than minimum requirement<br>Consumption expenditure: Spending less than minimum requirement<br>Crop diversity: less than 50% of the 8 major crops grown in the area<br>Land under irrigation: no access to irrigation at all<br>Land under improved seed: area not covered with improved seed (average of high yielding, drought tolerant, early maturing)<br>Land under commercial fertilizer: Having no access to fertilizer at all<br>Cash reserve: Having no cash saving at all<br>Food reserve: Having no food reserve for next year<br>Credit: Having no access to credit at all				
			**III. Environmental Vulnerability Variables (Measures of Sensitivity and Exposure)**				

		Land topography: Slope greater than 15% and 0% slope<br>Fertility: Poor fertility and cannot produce without heavy fertilizer use<br>Vegetation cover: Bare land<br>Frequency of hazards: People facing more than two natural hazards in a year<br>Rainfall: Receiving below average<br>Temperature: Experiencing above average<br>Change in wind direction: Encountering change in wind direction than usual				
Sampling strategies reported?	Yes	The specific study sites within the dis- tricts were selected based on a multi stage random sam- pling procedure. Consequently, 19 Kebeles were selected from which the sample households were selected ran- domly proportional to population size.				
Sampling sizes reported?	Yes	452 farm households in three districts of the Zone				
Data analysis methods reported?	Yes	In this analysis, the level of resilience was classified into three categories: 1) households that were fast in bouncing back; which means households that have gone back to their normal agricultural operation in the following production season; 2) moderate in bouncing back; which means households which took one to two agricultural seasons to get back to normal operation as before the event; and 3) slow in bouncing back; which means households which were unable to bounce back within one to two agricultural seasons to their normal livelihood activities. In this research, a farmer is said to have fully bounced back, when it begins its lively- hood operation as time before the shock. The speed of bouncing back was measured by number of agricul- tural seasons taken to bounce back to their livelihood without external intervention by government or non- governmental organization.<br>[...]<br>Table 3 presents the statistical measure of the different				

variables of resilience in the study area. From the statistical analysis result, the time taken to
bounce back after climate change induced shocks ranges from 1 agricultural year to more than 5 years
[...]

Table 3. Statistical values of factors of resilience to climate change induced shocks.				
**Variables**	**Mean**	**Maximum**	**Minimum**	**St Deviation**
Time taken to bounce back (Agr. seasons)	3	4	1	1.3898
Source: Own computation from household survey of 2011/2012.				

Transparency Assessment Article summary	
**Article**	Westerhoff & Smit (2009)
**Transparent operationalizations**	Adaptation strategy; adaptive capacity; exposed and sensitive to climate change; multiple underlying forces
**Partially transparent**	
**Not transparent**	

**Structured summary of operationalization – validity assessment**			**1.1 DCM Appropriate**	**1.2 valid empirical rep?**	**1. conclusion - Valid?**	**2. Feasible?**
**Construct:** adaptation strategy						
**Article:** Westerhoff & Smit (2009)						
Criterion	Assessment	Quoted text or Rationale for negative assessment				
Construct defined?	Yes	Adaptations, or adaptive strategies, employed by individuals or groups are depicted as being mediated through their relative adaptive capacities, indicating that adaptations may or may not be accessed according to the distribution of various types of resources such as physical or social capital, as developed by Adger and Kelly (1999).	Yes/ no/ can't tell	Yes/ no/ can't tell	Yes/ no/ can't tell YES	Yes/ no/ can't tell YES
Data collection methods reported?	Yes	These were determined using a community-based approach similar to those used by Burton et al. (2002), Ford and Smit (2004) and Schröter et al. (2005), in which the factors and forces relevant to the community vulnerability were sought via primary and secondary sources. [...] Most of the primary data were derived from 22 semi-structured, in-depth interviews with 11 male and 11 female community members, [...] These community-member interviews were complemented by an additional 22 in-depth interviews with key informants from various governmental and non-governmental institutions in the area [...] In addition, five focus groups were conducted with members	YES			

		of the community,<br>[...]<br>The community-based data collection was complemented by a review of documents and records to extract information on the biophysical and socioeconomic forces contributing to vulnerability. Documents comprised existing studies completed in the area, government reports, climate data, and all other pertinent information.				
Reporting of indicators/questions used to operationalise construct?	Yes	22 semi-structured, in-depth interviews with 11 male and 11 female community members, who were asked to describe and explain in local terminology the various exposure-sensitivities, adaptations and adaptive capacities of importance to them.<br>[...]<br>interviews with key informants from various governmental and non-governmental institutions in the area for the purposes of obtaining further information on the relevant contributing biophysical/socioeconomic forces, exposure-sensitivities, adaptations, and adaptive capac- ities in Mimkyemfre.<br>[...]<br>five focus groups were conducted with members of the community, through<br>which data on the experience of vulnerability by residents engaged in primary livelihood activities were gathered. Information on methods of farming, charcoal production and fishing, the stresses on these livelihoods, and the means for overcoming these stresses was compiled. Focus groups were used to investigate interactions between community members and other aspects of vulnerability that became evident in the group dynamic, an effect often referred to as 'synergism' (Morgan 1996).		YES		
Sampling strategies reported?	Yes	Interviewees were selected using purposeful and "typical case" sampling methods in order to obtain an illustrative sample of gender and age groups (Bradshaw and Stratford 2000). Members of the community who were engaged in farming or other commonly- practiced activities were included, as were typically marginalized groups such as				

		women and the elderly, to gain insight on different experiences within the community. [...] Key informant interviewees were selected based on their expertise on and/or experience with the community and its environment, and ranged from community members to members of relevant institutions, including local NGOs, the Ministry of Food and Agriculture, the National Health Insurance Scheme, the Department of Forestry and several others. [...] Participants for focus groups were selected primarily using a combination of purposeful and typical case sampling methods in order to identify members that were representative of the community so as to ensure a typical characterization of the community was obtained.				
Sampling sizes reported?	Yes	22 semi-structured, in-depth interviews with 11 male and 11 female [...] 22 in-depth interviews with key informants [...] five focus groups				
Data analysis methods reported?	Yes	Data from these multiple sources were coded, sorted and analyzed (in part using qualitative data analysis software) according to the themes of the conceptual model of vulnerability in order to identify relevant forces, exposure-sensitivities, adaptations and adaptive capacities experienced at the individual, household and community levels.				

**Structured summary of operationalization – validity assessment**			**1.1 DCM Appropriate**	**1.2 valid empirical rep?**	**1. conclusion - Valid?**	**2. Feasible?**
**Construct:** Adaptive capacity						
**Article:** Westerhoff & Smit (2009)						
<u>Criterion</u>	<u>Assessment</u>	<u>Quoted text or Rationale for negative assessment</u>				
Construct defined?	Yes	Adaptive capacity (broadly consistent with social	Yes/ no/ can't	Yes/ no/	Yes/ no/	Yes/ no/

		resilience) is also reflective of both the natural resource base and the social, economic, cultural and political conditions that facilitate or constrain adaptations to changing environments.	tell	can't tell	can't tell<br><br>YES	can't tell<br><br>YES
Data collection methods reported?	Yes	These were determined using a community-based approach similar to those used by Burton et al. (2002), Ford and Smit (2004) and Schröter et al. (2005), in which the factors and forces relevant to the community vulnerability were sought via primary and secondary sources.<br>[...]<br>Most of the primary data were derived from 22 semi-structured, in-depth interviews with 11 male and 11 female community members,<br>[...]<br>These community-member interviews were complemented by an additional 22 in-depth interviews with key informants from various governmental and non-governmental institutions in the area<br>[...]<br>In addition, five focus groups were conducted with members of the community,<br>[...]<br>The community-based data collection was complemented by a review of documents and records to extract information on the biophysical and socioeconomic forces contributing to vulnerability. Documents comprised existing studies completed in the area, government reports, climate data, and all other pertinent information.	YES			
Reporting of indicators/questions used to operationalise construct?	Yes	22 semi-structured, in-depth interviews with 11 male and 11 female community members, who were asked to describe and explain in local terminology the various exposure-sensitivities, adaptations and adaptive capacities of importance to them.<br>[...]<br>interviews with key informants from various governmental and non-governmental institutions in the area for the purposes of obtaining further information on		YES		

		the relevant contributing biophysical/socioeconomic forces, exposure-sensitivities, adaptations, and adaptive capac- ities in Mimkyemfre.<br>[...]<br>five focus groups were conducted with members of the community, through<br>which data on the experience of vulnerability by residents engaged in primary livelihood activities were gathered. Information on methods of farming, charcoal production and fishing, the stresses on these livelihoods, and the means for overcoming these stresses was compiled. Focus groups were used to investigate interactions between community members and other aspects of vulnerability that became evident in the group dynamic, an effect often referred to as 'synergism' (Morgan 1996).				
Sampling strategies reported?	Yes	Interviewees were selected using purposeful and "typical case" sampling methods in order to obtain an illustrative sample of gender and age groups (Bradshaw and Stratford 2000). Members of the community who were engaged in farming or other commonly- practiced activities were included, as were typically marginalized groups such as women and the elderly, to gain insight on different experiences within the community.<br>[...]<br>Key informant interviewees were selected based on their expertise on and/or experience with the community and its environment, and ranged from community members to members of relevant institutions, including local NGOs, the Ministry of Food and Agriculture, the National Health Insurance Scheme, the Department of Forestry and several others.<br>[...]<br>Participants for focus groups were selected primarily using a combination of purposeful and typical case sampling methods in order to identify members that were representative of the community so as to ensure a typical characterization of the community was obtained.				

Sampling sizes reported?	Yes	22 semi-structured, in-depth interviews with 11 male and 11 female<br>[...]<br>22 in-depth interviews with key informants<br>[...]<br>five focus groups				
Data analysis methods reported?	Yes	Data from these multiple sources were coded, sorted and analyzed (in part using qualitative data analysis software) according to the themes of the conceptual model of vulnerability in order to identify relevant forces, exposure-sensitivities, adaptations and adaptive capacities experienced at the individual, household and community levels.				

**Structured summary of operationalization – validity assessment**			**1.1 DCM Appropriate**	**1.2 valid empirical rep?**	**1. conclusion - Valid?**	**2. Feasible?**
**Construct:** exposed and sensitive to climate change						
**Article:** Westerhoff & Smit (2009)						
Criterion	Assessment	Quoted text or Rationale for negative assessment				
Construct defined?	Yes	People's exposures and sensitivities to external conditions are influenced by their occupancy and livelihood characteristics, and the nature and degree to which these are affected by the external stresses.	Yes/ no/ can't tell	Yes/ no/ can't tell	Yes/ no/ can't tell<br><br>YES	Yes/ no/ can't tell<br><br>YES
Data collection methods reported?	Yes	These were determined using a community-based approach similar to those used by Burton et al. (2002), Ford and Smit (2004) and Schröter et al. (2005), in which the factors and forces relevant to the community vulnerability were sought via primary and secondary sources.<br>[...]<br>Most of the primary data were derived from 22 semi-structured, in-depth interviews with 11 male and 11 female community members,<br>[...]<br>These community-member interviews were complemented by an additional 22 in-depth interviews	YES			

		with key informants from various governmental and non-governmental institutions in the area<br>[...]<br>In addition, five focus groups were conducted with members of the community,<br>[...]<br>The community-based data collection was complemented by a review of documents and records to extract information on the biophysical and socioeconomic forces contributing to vulnerability. Documents comprised existing studies completed in the area, government reports, climate data, and all other pertinent information.				
Reporting of indicators/questions used to operationalise construct?	Yes	22 semi-structured, in-depth interviews with 11 male and 11 female community members, who were asked to describe and explain in local terminology the various exposure-sensitivities, adaptations and adaptive capacities of importance to them.<br>[...]<br>interviews with key informants from various governmental and non-governmental institutions in the area for the purposes of obtaining further information on the relevant contributing biophysical/socioeconomic forces, exposure-sensitivities, adaptations, and adaptive capac- ities in Mimkyemfre.<br>[...]<br>five focus groups were conducted with members of the community, through<br>which data on the experience of vulnerability by residents engaged in primary livelihood activities were gathered. Information on methods of farming, charcoal production and fishing, the stresses on these livelihoods, and the means for overcoming these stresses was compiled. Focus groups were used to investigate interactions between community members and other aspects of vulnerability that became evident in the group dynamic, an effect often referred to as 'synergism' (Morgan 1996).		YES		
Sampling strategies reported?	Yes	Interviewees were selected using purposeful and "typical case" sampling methods in order to obtain an illustrative				

		sample of gender and age groups (Bradshaw and Stratford 2000). Members of the community who were engaged in farming or other commonly- practiced activities were included, as were typically marginalized groups such as women and the elderly, to gain insight on different experiences within the community.<br>[...]<br>Key informant interviewees were selected based on their expertise on and/or experience with the community and its environment, and ranged from community members to members of relevant institutions, including local NGOs, the Ministry of Food and Agriculture, the National Health Insurance Scheme, the Department of Forestry and several others.<br>[...]<br>Participants for focus groups were selected primarily using a combination of purposeful and typical case sampling methods in order to identify members that were representative of the community so as to ensure a typical characterization of the community was obtained.				
Sampling sizes reported?	Yes	22 semi-structured, in-depth interviews with 11 male and 11 female<br>[...]<br>22 in-depth interviews with key informants<br>[...]<br>five focus groups				
Data analysis methods reported?	Yes	Data from these multiple sources were coded, sorted and analyzed (in part using qualitative data analysis software) according to the themes of the conceptual model of vulnerability in order to identify relevant forces, exposure-sensitivities, adaptations and adaptive capacities experienced at the individual, household and community levels.				

Structured summary of operationalization – validity assessment			1.1 DCM Appropriate	1.2 valid empirical rep?	1. conclusion - Valid?	2. Feasible?
**Construct:** multiple underlying forces						
**Article:** Westerhoff & Smit (2009)						
Criterion	Assessment	Quoted text or Rationale for negative assessment				
Construct defined?	Yes	n summary, research on practical adaptations to effectively address the vulnerability of people to climate change has recognized the need to identify the factors in addition to climate that contribute to vulnerability, including the multiple forces and dynamic processes that occur at both local and broader scales.	Yes/ no/ can't tell	Yes/ no/ can't tell	Yes/ no/ can't tell YES	Yes/ no/ can't tell
Data collection methods reported?	Yes	These were determined using a community-based approach similar to those used by Burton et al. (2002), Ford and Smit (2004) and Schröter et al. (2005), in which the factors and forces relevant to the community vulnerability were sought via primary and secondary sources. [...] Most of the primary data were derived from 22 semi-structured, in-depth interviews with 11 male and 11 female community members, [...] These community-member interviews were complemented by an additional 22 in-depth interviews with key informants from various governmental and non-governmental institutions in the area [...] In addition, five focus groups were conducted with members of the community, [...] The community-based data collection was complemented by a review of documents and records to extract information on the biophysical and socioeconomic forces contributing to vulnerability. Documents comprised existing studies completed in the area, government reports, climate data, and all other pertinent information.	YES			
Reporting of indicators/questions used to	Yes	22 semi-structured, in-depth interviews with 11 male and 11 female community members, who were asked to describe and explain in local terminology the various		YES		

operationalise construct?		exposure-sensitivities, adaptations and adaptive capacities of importance to them.<br>[...]<br>interviews with key informants from various governmental and non-governmental institutions in the area for the purposes of obtaining further information on the relevant contributing biophysical/socioeconomic forces, exposure-sensitivities, adaptations, and adaptive capac- ities in Mimkyemfre.<br>[...]<br>five focus groups were conducted with members of the community, through<br>which data on the experience of vulnerability by residents engaged in primary livelihood activities were gathered. Information on methods of farming, charcoal production and fishing, the stresses on these livelihoods, and the means for overcoming these stresses was compiled. Focus groups were used to investigate interactions between community members and other aspects of vulnerability that became evident in the group dynamic, an effect often referred to as 'synergism' (Morgan 1996).				
Sampling strategies reported?	Yes	Interviewees were selected using purposeful and "typical case" sampling methods in order to obtain an illustrative sample of gender and age groups (Bradshaw and Stratford 2000). Members of the community who were engaged in farming or other commonly- practiced activities were included, as were typically marginalized groups such as women and the elderly, to gain insight on different experiences within the community.<br>[...]<br>Key informant interviewees were selected based on their expertise on and/or experience with the community and its environment, and ranged from community members to members of relevant institutions, including local NGOs, the Ministry of Food and Agriculture, the National Health Insurance Scheme, the Department of Forestry and several others.<br>[...]				

		Participants for focus groups were selected primarily using a combination of purposeful and typical case sampling methods in order to identify members that were representative of the community so as to ensure a typical characterization of the community was obtained.				
Sampling sizes reported?	Yes	22 semi-structured, in-depth interviews with 11 male and 11 female<br>[...]<br>22 in-depth interviews with key informants<br>[...]<br>five focus groups				
Data analysis methods reported?	Yes	Data from these multiple sources were coded, sorted and analyzed (in part using qualitative data analysis software) according to the themes of the conceptual model of vulnerability in order to identify relevant forces, exposure-sensitivities, adaptations and adaptive capacities experienced at the individual, household and community levels.				

Appendix N: Report of selected operationalizations of retained frameworks

IPCC

Constructs

- Vulnerability (IPCC);

Constructs in report of frameworks	Represented in list by	Coded text indicating relationship
Vulnerability (IPCC)	Vulnerability (IPCC)	
Exposure	Vulnerability (IPCC)	Asper theIPCC's definition and framework, vulnerability is understood as a function of three components—exposure, sensitivity and adaptive capacity.
Sensitivity (A,B)	Vulnerability (IPCC)	Asper theIPCC's definition and framework, vulnerability is understood as a function of three components—exposure, sensitivity and adaptive capacity.
Adaptive Capacity (A)	Vulnerability (IPCC)	Asper theIPCC's definition and framework, vulnerability is understood as a function of three components—exposure, sensitivity and adaptive capacity.
Entity	Vulnerability (IPCC)	An important result of the grammatical investigation is that the concept of vulnerability is a relative one: it is the vulnerability of an entity to a specific stimulus with respect to certain preference criteria
Stimulus	Vulnerability (IPCC)	An important result of the grammatical investigation is that the concept of vulnerability is a relative one: it is the vulnerability of an entity to a specific stimulus with respect to certain preference criteria
Preference criteria	Vulnerability (IPCC)	An important result of the grammatical investigation is that the concept of vulnerability is a relative one: it is the vulnerability of an entity to a specific stimulus with respect to certain preference criteria
Adaptive capacity (var)		No operationalized representative
Reference scenarios		

Operationalization of constructs			
**Construct operationalized:** Vulnerability IPCC			
**Source article(s):**			
**Selected by:**		expert selection [justification]	
Sub-constructs		Intermediate constructs	
		Directly operationalized constructs	
Conceptual framework			
Operationalization of sub-constructs			
1.	•	Data collection	
		Operational questions	
		Sampling strategies	
		Sample sizes	
		Data analysis	
2.	•	Data collection	
		Operational questions	
		Sampling strategies	
		Sample sizes	
		Data analysis	
3.	•	Data collection	
		Operational questions	
		Sampling strategies	
		Sample sizes	
		Data analysis	
4.	•	Data collection	
		Operational questions	
		Sampling strategies	
		Sample sizes	
		Data analysis	
Candidate-level Analysis			

Vulnerability as Expected poverty (with extensions)

Constructs:

- Vulnerability as Expected Poverty
- Food insecurity
- Expected future food security status
- Idiosyncratic shocks
- Covariate shocks
- Household level
- Community level

Constructs in report of frameworks	Represented in list by	Coded text indicating relationship
Vulnerability as Expected Poverty	Vulnerability as Expected Poverty	Directly represented
Poverty	Vulnerability as Expected Poverty	Thus, vulnerability is seen as expected poverty, while consumption (income) is used as a proxy for well-being. This method is based on estimating the probability that a given shock or set of shocks will move household consumption below a given minimum level (such as a consumption poverty line) or force the consumption level to stay below the minimum if it is already below this level (Chaudhuri et al. 2002).
Food insecurity		No operationalized representative
Expected future food security status	Expected future food security status	Directly represented
Future nutritional status		No operationalized representative
Idiosyncratic shocks	Idiosyncratic shocks	Directly represented
Covariate shocks	Covariate shocks	Directly represented
Household level	Household level	Directly represented
Community level	Community level	Directly represented

**Operationalization of constructs**			
**Construct operationalized:** Vulnerability as Expected Poverty			
**Source article(s):**			
**Selected by:**		expert selection [justification]	
Sub-constructs		Intermediate constructs	
		Directly operationalized constructs	
Conceptual framework			
Operationalization of sub-constructs			
5.	•	Data collection	
		Operational questions	
		Sampling strategies	
		Sample sizes	

		Data analysis	
6.	•	Data collection	
		Operational questions	
		Sampling strategies	
		Sample sizes	
		Data analysis	
7.	•	Data collection	
		Operational questions	
		Sampling strategies	
		Sample sizes	
		Data analysis	
8.	•	Data collection	
		Operational questions	
		Sampling strategies	
		Sample sizes	
		Data analysis	
Candidate-level Analysis			

**Operationalization of constructs**			
**Construct operationalized:** Expected future food security status			
**Source article(s):** Capaldo et al (2010)			
**Selected by:**		default	
Sub-constructs		Intermediate constructs	Present food security status; events
		Directly operationalized constructs	Current socio-economic characteristics; current exposure to risks; Risks; risk management
Conceptual framework		Our model is based on the Social Risk Management approach (Holzmann and Jørgensen 2000; World Bank 2000) and, more specifically, on the conceptual framework drawn from it by Løvendal and Knowles (2005). In this framework vulnerability is the result of a recursive process: current socio-economic characteristics and exposure to risks determine households' future characteristics and their risk-management capacity. At every point in time households' current food security status is affected by their past status and affects their future status. Figure 1 represents graphically this recursive connection.	
Operationalization of sub-constructs			
9.	Current socio-economic characteristics	Data collection	We analyze a sample of 1831 rural households from Nicaragua, surveyed in the 2001 Encuesta Nacional de Hogares Sobre Medición de Nivel de Vida, by the Instituto Nacional de Estadísticas y Censos INEC de Nicaragua. Constructed variables used in the analysis were prepared by the Rural Income Generating Activities (RIGA) project team at FAO.
		Operational questions	Information on the structure of a household includes the age of the head of household (which is also a proxy for working experience), gender, marital status, language spoken (as a proxy for households belonging to an indigenous group) and the share of female labor. The latter also approximates labor availability within the household. We observed a relatively high proportion of

			single- or female-headed households (23% and 18% respectively). Household assets are assessed in using education, as well as wealth-related variables (number of rooms, cement floor, telephone, access to safe water, bikes, radios, TV sets owned4), and social capital different<br>through participation of members in community organizations. Moreover, types of livestock and land assets are also taken into account to approximate<br>household wealth and potential credit-related constraints. We use access to a network for migration as a measure of the ability of a household to receive assistance from members living outside the location and as a proxy of a diversified income portfolio. Distance from a road, school, and health facilities, are variables used for measuring a household's access to infrastructure.<br>[...]<br>Table 1: Summary of variables<br>Kilocalories per capita<br>Age of hh head<br>Highest education in hh<br>Single head<br>Female head of hh widow<br>Female headed hh<br>Hh labor<br>Indigenous household<br>Hh size<br>Rooms<br>Cement floor in house<br>Telephone in hh<br>Hh members participating in comm. org.<br>Access to hh migration network<br>Access to safe water<br>Bikes owned<br>Radios owned<br>TVs owned<br>Distance to nearest primary school<br>Time to nearest health facility<br>Distance to nearest major road<br>Land owned<br>Cattle<br>Pigs<br>Horses<br>Land operated<br>Access to irrigation<br>Income from farming activities<br>Income from farm sales
		Sampling strategies	We analyze a sample of 1831 rural households from Nicaragua, surveyed in the 2001 Encuesta Nacional de Hogares Sobre Medición de Nivel de Vida, by the Instituto Nacional de Estadísticas y Censos INEC de Nicaragua. Constructed variables used in the analysis were prepared by the Rural Income Generating Activities (RIGA) project team at FAO.
		Sample sizes	sample of 1831 rural households

		Data analysis	After accounting for heteroskedasticity through the use of generalized least squares, we estimate vulnerability to food insecurity as the normal probability that the "individual minimum dietary energy requirement under light physical activity" exceeds the expected individual dietary energy consumption (measured in kilocalories). Since the main purpose of this paper is to propose a methodology to analyze and estimate vulnerability, we ignore possible econometric complications that are not directly relevant. However, by all means the results presented here are to be considered preliminary.
10.	current exposure to risks	Data collection	Same as previous construct
		Operational questions	We use ex-post data on shocks and risk management strategies. These include information on the incidence of a covariate shock (such as drought) and an idiosyncratic shock (illness)<br>[...]<br>Table 1: Summary of variables<br>Drought shock<br>Illness shock
		Sampling strategies	Same as previous construct
		Sample sizes	Same as previous construct
		Data analysis	Same as previous construct
11.	Risks	Data collection	NOT TRANSPARENT
		Operational questions	
		Sampling strategies	
		Sample sizes	
		Data analysis	
12.	risk management	Data collection	NOT TRANSPARENT
		Operational questions	
		Sampling strategies	
		Sample sizes	
		Data analysis	
13.		Data collection	
		Operational questions	
		Sampling strategies	
		Sample sizes	
		Data analysis	
Candidate-level Analysis		After accounting for heteroskedasticity through the use of generalized least squares, we estimate vulnerability to food insecurity as the normal probability that the "individual minimum dietary energy requirement under light physical activity" exceeds the expected individual dietary energy consumption (measured in kilocalories). Since the main purpose of this paper is to propose a methodology to analyze and estimate vulnerability, we ignore possible econometric complications that are not directly relevant. However, by all means the results presented here are to be considered preliminary.	

**Operationalization of constructs**
**Construct operationalized:** Idiosyncratic shocks
**Source article(s):**

**Selected by:**		expert selection [justification]	
Sub-constructs		Intermediate constructs	
		Directly operationalized constructs	
Conceptual framework			
Operationalization of sub-constructs			
14.	•	Data collection	
		Operational questions	
		Sampling strategies	
		Sample sizes	
		Data analysis	
15.	•	Data collection	
		Operational questions	
		Sampling strategies	
		Sample sizes	
		Data analysis	
16.	•	Data collection	
		Operational questions	
		Sampling strategies	
		Sample sizes	
		Data analysis	
17.	•	Data collection	
		Operational questions	
		Sampling strategies	
		Sample sizes	
		Data analysis	
Candidate-level Analysis			

**Operationalization of constructs**			
**Construct operationalized:** Covariate shocks			
**Source article(s):**			
**Selected by:**		expert selection [justification]	
Sub-constructs		Intermediate constructs	
		Directly operationalized constructs	
Conceptual framework			
Operationalization of sub-constructs			
18.	•	Data collection	
		Operational questions	
		Sampling strategies	
		Sample sizes	
		Data analysis	
19.	•	Data collection	
		Operational questions	

		Sampling strategies	
		Sample sizes	
		Data analysis	
20.	•	Data collection	
		Operational questions	
		Sampling strategies	
		Sample sizes	
		Data analysis	
21.	•	Data collection	
		Operational questions	
		Sampling strategies	
		Sample sizes	
		Data analysis	
Candidate-level Analysis			

**Operationalization of constructs**			
**Construct operationalized:** Household level			
**Source article(s):**			
**Selected by:**		expert selection [justification]	
Sub-constructs		Intermediate constructs	
		Directly operationalized constructs	
Conceptual framework			
Operationalization of sub-constructs			
22.	•	Data collection	
		Operational questions	
		Sampling strategies	
		Sample sizes	
		Data analysis	
23.	•	Data collection	
		Operational questions	
		Sampling strategies	
		Sample sizes	
		Data analysis	
24.	•	Data collection	
		Operational questions	
		Sampling strategies	
		Sample sizes	
		Data analysis	
25.	•	Data collection	
		Operational questions	
		Sampling strategies	
		Sample sizes	
		Data analysis	
Candidate-level Analysis			

**Operationalization of constructs**			
**Construct operationalized:** Community level			
**Source article(s):**			
**Selected by:**		expert selection [justification]	
Sub-constructs		Intermediate constructs	
		Directly operationalized constructs	
Conceptual framework			
Operationalization of sub-constructs			
26.	•	Data collection	
		Operational questions	
		Sampling strategies	
		Sample sizes	
		Data analysis	
27.	•	Data collection	
		Operational questions	
		Sampling strategies	
		Sample sizes	
		Data analysis	
28.	•	Data collection	
		Operational questions	
		Sampling strategies	
		Sample sizes	
		Data analysis	
29.	•	Data collection	
		Operational questions	
		Sampling strategies	
		Sample sizes	
		Data analysis	
Candidate-level Analysis			

Perceptions of Climate Change

Constructs:

- Farmer perceptions
- Adaptation strategy

Constructs in report of frameworks	Represented in list by	Coded text indicating relationship
Farmer perceptions	Farmer perceptions	Directly represented
Adaptation strategy	Adaptation strategy	Directly represented
Coping strategy		No operationalized representative

<table>
<tr><td colspan="4">Operationalization of constructs</td></tr>
<tr><td colspan="4">Construct operationalized: Farmer perceptions</td></tr>
<tr><td colspan="4">Source article(s): Mubaya et al</td></tr>
<tr><td colspan="2">Selected by:</td><td colspan="2">default</td></tr>
<tr><td colspan="2" rowspan="2">Sub-constructs</td><td>Intermediate constructs</td><td></td></tr>
<tr><td>Directly operationalized constructs</td><td></td></tr>
<tr><td colspan="2">Conceptual framework</td><td colspan="2">DIRECETLY OPERATIONALIZED</td></tr>
<tr><td colspan="2">Operationalization of sub-constructs</td><td colspan="2"></td></tr>
<tr><td rowspan="2">30.</td><td rowspan="2">•</td><td>Data collection</td><td>The qualitative methods of data collection used include Participatory Rural Appraisal (PRA) techniques such as historical trend analysis and matrix scoring and ranking and Focus Group Discussions (FGDs). The quantitative method used is the household questionnaire survey.</td></tr>
<tr><td>Operational questions</td><td>2.2.2. Qualitative assessments FGDs were used to first of all establish the general perceptions
regarding climate change and variability and their causes and various stressors that confront farmers' livelihoods (see Appendix 2). Following this, it was considered important for this study to factor in how farmers regard climate change and variability as an obstacle to their livelihoods among the multiple stressors that they had identified. Among these stressors are climate variability in different forms, issues of financial capital, issues related to cattle pests and diseases, inadequate draught power, marketing issues and HIV and AIDS. A matrix scoring and ranking exercise was then facilitated for
farmers. Farmers were asked as a group to select from the long list of stressors the ones they considered critical for the purposes of scoring and ranking. The second step involved participants defining criteria that they would use to evaluate these stressors. These criteria include food security, income generation, crop production and livelihood security. Through group consensus, farmers then decided how much to allocate each shock out of a total of 20 points, based on the</td></tr>
</table>

			group defined criteria. Historical trend lines were used to elicit information on specific historical trends in farmers' perceptions regarding changes in climate over a period of 20 years and as far back as they could recall. Specifically, participants were asked to recall major occurrences that had a bearing on climate and weather, community resources, and even the political situation. They were then asked to indicate what occurrences had the greatest impact on their livelihoods among the cited events.<br>[...]<br>2.2.3. Quantitative assessments The questionnaire survey was used to collect household data<br>and complement data generated through the qualitative methods. This survey collected data on changes in crops grown over a period of five years and reasons for these changes, indicators for good and bad crop production seasons and years considered to be good or bad over a ten year period. Questions in the survey also related to changes in weather patterns over a ten year period in relation to agriculture and what might have caused these changes. General household characteristics were also captured in this survey (see Appendix 1).
		Sampling strategies	A sample of 720 households across countries was selected for<br>the survey, 180 households per each of the four districts. Specifi- cally, systematic random sampling was employed to come up with six villages per district (making them 24 across countries) and 30 households per each of these villages, making a total of 380 households per country (this study was part of a big inter- institutional research-based development project). For FGDs and PRA workshops, a group of eight to 15 participants<br>was selected to represent the three villages per district, with approximately five representatives from each of the three villages per district. In coming up with this group, factors such as age and gender were used. In terms of gender, separate PRA workshops were held for men and women in order not to compromise the amount and quality of information that can be generated from the less confident if they were to be combined. Specifically, old men and women were incorporated into the sample for the group discussions in order to capture information related to historical trends in climate. Itwas envisaged that they would be able to recall as far back as they could and provide rich information on these trends. In the same context, youths were incorporated into the sample in order to validate some of the recent trends on climate suggested by the elderly.
		Sample sizes	A sample of 720 households across countries was selected for<br>the survey, 180 households per each of the four districts.<br>[...]<br>For FGDs and PRA workshops, a group of eight to 15 participants

			was selected to represent the three villages per district, with approximately five representatives from each of the three villages per district.
		Data analysis	Qualitative data were categorised and analysed in four distinct themes. These themes are<br>Perceptions regarding changes in weather patterns, Perceptions regarding causes of changes and variability in climate, Perceptions regarding other stressors among farmers and Perceptions regarding climate change in relation to other stressors.<br>These perceptions were established in historical trend lines, FGDs and matrix scoring and ranking and they are presented in this manner in the sections under results and discussion.<br>[...]<br>Data from the questionnaire survey were entered into the Statistical Package for the Social Sciences (SPSS) and analysed by running descriptive frequencies in relation to the distinct themes highlighted in this section. These themes include perceptions regarding changes in weather patterns in general and for specific seasons and regarding causes of these changes. These frequencies were disaggregated by district and country.
Candidate-level Analysis		DIRECTLY OPERATIONALIZED	

**Operationalization of constructs**			
**Construct operationalized:** Adaptation strategy			
**Source article(s):**			
**Selected by:**		expert selection [justification]	
Sub-constructs		Intermediate constructs	
		Directly operationalized constructs	
Conceptual framework			
Operationalization of sub-constructs			
31.	•	Data collection	
		Operational questions	
		Sampling strategies	
		Sample sizes	
		Data analysis	
32.	•	Data collection	
		Operational questions	
		Sampling strategies	
		Sample sizes	
		Data analysis	
33.	•	Data collection	
		Operational questions	
		Sampling strategies	
		Sample sizes	

		Data analysis	
34.	•	Data collection	
		Operational questions	
		Sampling strategies	
		Sample sizes	
		Data analysis	
Candidate-level Analysis			

Asset Vulnerability

- Household vulnerability to climate change
- Future exposure;

Constructs in report of frameworks	Represented in list by	Coded text indicating relationship
Household vulnerability to climate change	Household vulnerability to climate change	Directly represented
Asset vulnerability	Household vulnerability to climate change	Using the GLSS 4, we applied the asset vulnerability framework developed by Moser (1996, 1998, 2007). We constructed an index of vulnerability to climate change, at the household level.
Future exposure	Future exposure	Directly represented
Communities at risk of climate shocks		No operationalized representative
Welfare of rural households		No operationalized representative
Prepared for adverse consequences		No operationalized representative

<table>
<tr><td colspan="4">Operationalization of constructs</td></tr>
<tr><td colspan="4">Construct operationalized: Household vulnerability to climate change</td></tr>
<tr><td colspan="4">Source article(s): Dasgupta & bashieri</td></tr>
<tr><td colspan="2">Selected by:</td><td colspan="2">default</td></tr>
<tr><td colspan="2" rowspan="2">Sub-constructs</td><td>Intermediate constructs</td><td>Asset vulnerability</td></tr>
<tr><td>Directly operationalized constructs</td><td>Labour; human capital; non-labour productive assets; social capital</td></tr>
<tr><td colspan="2">Conceptual framework</td><td colspan="2">Using the GLSS 4, we applied the asset vulnerability framework developed by Moser (1996, 1998, 2007). We constructed an index of vulnerability to climate change, at the household level.
[...]
The first asset Moser identified is labour.
[...]
The second asset Moser (1998) identified is human capital.
[...]
Non-labour productive assets are the third type.
[...]
Moser (1998) identified household relations
[...]
Social capital isMoser’sfifth asset</td></tr>
<tr><td colspan="2">Operationalization of sub-constructs</td><td colspan="2"></td></tr>
<tr><td rowspan="5">35.</td><td rowspan="5">Labour</td><td>Data collection</td><td>NOT valid/feasible</td></tr>
<tr><td>Operational questions</td><td></td></tr>
<tr><td>Sampling strategies</td><td></td></tr>
<tr><td>Sample sizes</td><td></td></tr>
<tr><td>Data analysis</td><td></td></tr>
</table>

<table>
<tr><td rowspan="7">36.</td><td rowspan="7">human capital</td><td>Data collection</td><td>NOT valid/feasible</td></tr>
<tr><td>Operational questions</td><td></td></tr>
<tr><td>Sampling strategies</td><td></td></tr>
<tr><td>Sample sizes</td><td></td></tr>
<tr><td>Data analysis</td><td></td></tr>
<tr><td>Sample sizes</td><td></td></tr>
<tr><td>Data analysis</td><td></td></tr>
<tr><td rowspan="5">37.</td><td rowspan="5">non-labour productive assets</td><td>Data collection</td><td>We used data collected between April 1998 and March 1999 by the fourth round of the Ghana Living Standards Survey (GLSS 4), which was funded by theWorld Bank and the Republic of Ghana. The survey instruments were designed to monitor poverty and well- being in Ghana. The GLSS 4 contains information on the demographic characteristics of household members, their reported health status, education, employment, housing and income from wages, business activities and agricultural production and detailed records of consumption and expenditure data. The main data file contained household-level information and derived money-metric measures of poverty such as household income and expenditure (Coulombe and McKay, 2000).</td></tr>
<tr><td>Operational questions</td><td>In order to measure the different degrees of productive assets between households we used the total number of productive assets owned by the household as a proxy. Among reproducible capital assets the questionnaire included furniture, sewing machines, stoves, refrigerator-freezers, air conditioners, fans, radios, radio-cassette players, record players, three-in-one radio-cassette players, video equipment, washing machines, TVs, cameras, electric irons, bicycles, motorcycles, cars, houses, land, shares, boats, canoes and outboard motors. Each asset was weighted equally.
[...]
Table 2
<table><tr><td>ASSETS</td><td>Variable</td></tr><tr><td>Productive assets</td><td>Number of productive asset (N¼3679)</td></tr></table></td></tr>
<tr><td>Sampling strategies</td><td>TheGLSS4 is a two-stage probability-proportional-to-size sample.</td></tr>
<tr><td>Sample sizes</td><td>The sample contains data for5998households, of which 3799 resided in rural areas, with 25 694 eligible individual household members. We excluded the Greater Accra area as it is semi-urban, leaving 3679 rural households. In addition to the household survey, the GLSS 4 team (supervisor and enumerator) administered a community questionnaire to community leaders of the rural enumeration areas that were surveyed. One questionnaire was administered to each of the 195 rural enumeration areas.</td></tr>
<tr><td>Data analysis</td><td>Table 4 shows the weight assigned to each variable derived from the first principal
component. The data reduction that PCA performed on the data explained 25 per cent of the original variation of the data. The PCA factor loadings were examined, and the</td></tr>
</table>

			principal component tended to load<br>positively on variables which contributed to lower vulnerability such as better education and better health, and negatively on variables which contributed to higher household vulnerability such as higher percentage of household income from agriculture. Therefore, the score is a measure of 'strength' or preparedness. A high score indicates a non-vulnerable household, and a low score indicates a vulnerable household.
38.	social capital	Data collection	NOT VALID/FEASIBLE
		Operational questions	
		Sampling strategies	
		Sample sizes	
		Data analysis	
Candidate-level Analysis		Table 4 shows the weight assigned to each variable derived from the first principal component. The data reduction that PCA performed on the data explained 25 per cent of the original variation of the data. The PCA factor loadings were examined, and the principal component tended to load<br>positively on variables which contributed to lower vulnerability such as better education and better health, and negatively on variables which contributed to higher household vulnerability such as higher percentage of household income from agriculture. Therefore, the score is a measure of 'strength' or preparedness. A high score indicates a non-vulnerable household, and a low score indicates a vulnerable household.	

**Operationalization of constructs**			
**Construct operationalized:** Future Exposure			
**Source article(s): NO TRANSPARENT (FORD & SMIT) OR VALID (DASGUPTA & BASHCIERI) OPERATIONALIZATION**			
**Selected by:**		default	
Sub-constructs		Intermediate constructs	
		Directly operationalized constructs	
Conceptual framework			
Operationalization of sub-constructs			
39.	•	Data collection	
		Operational questions	
		Sampling strategies	
		Sample sizes	
		Data analysis	
40.	•	Data collection	
		Operational questions	
		Sampling strategies	
		Sample sizes	
		Data analysis	
41.	•	Data collection	
		Operational questions	
		Sampling strategies	
		Sample sizes	

		Data analysis	
42.	•	Data collection	
		Operational questions	
		Sampling strategies	
		Sample sizes	
		Data analysis	
Candidate-level Analysis			

Nested Vulnerability

Constructs:

- Nested and teleconnected livelihood vulnerability

Constructs in report of frameworks	Represented in list by	Coded text indicating relationship
Livelihood vulnerability (B)	Nested and teleconnected livelihood vulnerability	Directly represented
Nested and teleconnected livelihood vulnerability	Nested and teleconnected livelihood vulnerability	In the following sections, we use the case of the responses of farmers in Vietnam and Mexico to the evolution of the global coffeemarket over the past three decades to illustrate the insights that can be gained fromemploying a concept of nestedandteleconnectedlivelihoodvulnerability. Inthe case we present here, we argue that the vulnerability of individual farmers to the experience of welfare loss is connected not only through the structure of the global coffee commodity chain, but also through global ideological shifts affecting national policy, the movement of labor, the material flow of coffee stocks, channels of information, and, in reverse, through the broader environmental and institutional impli- cations of local adaptive action.
Nested system	Nested and teleconnected livelihood vulnerability	In this article we use the concept of ''nested and tele-connected vulnerabilities'' to illustrate how the vulnerabilities and responses of farm households in distinct geographic locations are linked through cross-scalar processes, as well as ''teleconnected'' in space and time. In a nested system, profoundchanges inkeyvariablesthatoperatenormallyonly at one level, e.g., within a defined geographic region or admin- istrative domain, can have non-linear outcomes for processes operating at broader scales of analysis (Gunderson andHolling, 2001)

Operationalization of constructs		
**Construct operationalized:** Nested and teleconnected livelihood vulnerability		
**Source article(s):** Eakin et al (2008)		
**Selected by:**	default	
Sub-constructs	Intermediate constructs	Livelihood vulnerability
	Directly operationalized	Nested Systems; Exogenous drivers; geographically specific

		constructs	signals of change; geographically distant household vulnerability; household responses; response outcomes
Conceptual framework		In the following sections, we use the case of the responses<br>of farmers in Vietnam and Mexico to the evolution of the global coffeemarket over the past three decades to illustrate the insights that can be gained fromemploying a concept of nestedandteleconnectedlivelihoodvulnerability. Inthe case we present here, we argue that the vulnerability of individual farmers to the experience of welfare loss is connected not only through the structure of the global coffee commodity chain, but also through global ideological shifts affecting national policy, the movement of labor, the material flow of coffee stocks, channels of information, and, in reverse, through the broader environmental and institutional impli- cations of local adaptive action.<br>[...]<br>In this article we use the concept of ‘‘nested and tele-<br>connected vulnerabilities’’ to illustrate how the vulnerabilities and responses of farm households in distinct geographic locations are linked through cross-scalar processes, as well as ‘‘teleconnected’’ in space and time. In a nested system, profoundchanges inkeyvariablesthatoperatenormallyonly at one level, e.g., within a defined geographic region or admin- istrative domain, can have non-linear outcomes for processes operating at broader scales of analysis (Gunderson andHolling, 2001)<br>[...]<br>Livelihood vulner- ability is composed of exogenous risks, household responses to risks, and the outcomes of these responses in terms of individual or household welfare.<br>[...]<br>In our case, we argue that geographically specific signals of change – such as a shift in market opportunities, a drought, a change in public policy or new form of land use in a specific location – can create risks and opportunities	
Operationalization of sub-constructs			
43.	Nested Systems	Data collection	NOT TRANSPARENT
		Operational questions	
		Sampling strategies	
		Sample sizes	
		Data analysis	
44.	Exogenous drivers	Data collection	NOT TRANSPARENT
		Operational questions	
		Sampling strategies	
		Sample sizes	
		Data analysis	
45.	geographically specific signals of change	Data collection	NOT TRANSPARENT
		Operational questions	
		Sampling strategies	
		Sample sizes	
		Data analysis	
46.	geographically distant household vulnerability	Data collection	NOT TRANSPARENT
		Operational questions	
		Sampling strategies	
		Sample sizes	
		Data analysis	
47.	household	Data collection	NOT TRANSPARENT

	responses	Operational questions	
		Sampling strategies	
		Sample sizes	
		Data analysis	
48.	response outcomes	Data collection	NOT TRANSPARENT
		Operational questions	
		Sampling strategies	
		Sample sizes	
		Data analysis	
Candidate-level Analysis			

Current and future vulnerability

Constructs:

- Vulnerability to climate risks

Constructs in report of frameworks	Represented in list by	Coded text indicating relationship
Vulnerability to climate risks		
Current vulnerability	Vulnerability to climate risks	A research framework for empirically applying the model of vulnerability proposed above to Arctic commu- nities is illustrated in Figure 3. The first stage assesses current vulnerability by documenting current exposures and current adaptive strategies. The second stage assesses future vulnerability by estimating directional changes in exposure and predicting future adaptive capacity on the basis of past behavior.
Future vulnerability	Vulnerability to climate risks	A research framework for empirically applying the model of vulnerability proposed above to Arctic commu- nities is illustrated in Figure 3. The first stage assesses current vulnerability by documenting current exposures and current adaptive strategies. The second stage assesses future vulnerability by estimating directional changes in exposure and predicting future adaptive capacity on the basis of past behavior.
Current adaptive capacity	Vulnerability to climate risks	The assessment of current vulnerability requires analyzing and documenting communities' experiences with climatic risks (current exposure) and the adaptive options and resource management strategies employed to address these risks (current adaptive capacity).
Exposure	Vulnerability to climate risks	The assessment of current vulnerability requires analyzing and documenting communities' experiences with climatic risks (current exposure) and the adaptive options and resource management strategies employed to address these risks (current adaptive capacity).

Future exposure	Vulnerability to climate risks	Future Exposure<br>Future Vulnerability<br>Future Adaptive Capacity

**Operationalization of constructs**			
**Construct operationalized:** Vulnerability to climate risks			
**Source article(s):** Ford & Smit (2004); Dasgupta & bashieri			
**Selected by:**		default	
Sub-constructs		Intermediate constructs	future vulnerability
		Directly operationalized constructs	Current vulnerability; Future exposure; future adaptive capacity
Conceptual framework		A research framework for empirically applying the model of vulnerability proposed above to Arctic commu- nities is illustrated in Figure 3. The first stage assesses current vulnerability by documenting current exposures and current adaptive strategies. The second stage assesses future vulnerability by estimating directional changes in exposure and predicting future adaptive capacity on the basis of past behavior.<br>[...]<br>FIG. 3. Analytical framework for vulnerability assessment.<br>Future Exposure<br>Future Vulnerability<br>Future Adaptive Capacity	
Operationalization of sub-constructs			
49.	Current vulnerability	Data collection	NOT TRANSPARENT
		Operational questions	
		Sampling strategies	
		Sample sizes	
		Data analysis	
50.	Future exposure	Data collection	NOT TRANSPARENT (Ford & Smit); Not Valid (Dasgupta & bashieri)
		Operational questions	
		Sampling strategies	
		Sample sizes	
		Data analysis	
51.	future adaptive capacity	Data collection	NOT TRANSPARENT
		Operational questions	
		Sampling strategies	
		Sample sizes	
		Data analysis	
52.	•	Data collection	
		Operational questions	
		Sampling strategies	
		Sample sizes	
		Data analysis	
Candidate-level Analysis			

Livelihood vulnerability index

Constructs:

- Livelihood vulnerability (A)

Constructs in report of frameworks	Represented in list by	Coded text indicating relationship
Livelihood vulnerability (A);	Livelihood vulnerability (A);	Directly represented
Livelihood strategies	Livelihood vulnerability (A);	The LVI includes seven major components: Socio-Demographic Profile, Livelihood Strategies, Social Networks, Health, Food, Water, and Natural Disasters and Climate Variability.
Health	Livelihood vulnerability (A);	The LVI includes seven major components: Socio-Demographic Profile, Livelihood Strategies, Social Networks, Health, Food, Water, and Natural Disasters and Climate Variability.
Socio-demographic profile	Livelihood vulnerability (A);	The LVI includes seven major components: Socio-Demographic Profile, Livelihood Strategies, Social Networks, Health, Food, Water, and Natural Disasters and Climate Variability.
Water	Livelihood vulnerability (A);	The LVI includes seven major components: Socio-Demographic Profile, Livelihood Strategies, Social Networks, Health, Food, Water, and Natural Disasters and Climate Variability.
Natural disaster and climate change	Livelihood vulnerability (A);	The LVI includes seven major components: Socio-Demographic Profile, Livelihood Strategies, Social Networks, Health, Food, Water, and Natural Disasters and Climate Variability.

<table>
<tr><th colspan="3">Operationalization of constructs</th></tr>
<tr><td colspan="3">Construct operationalized: Livelihood vulnerability (A)</td></tr>
<tr><td colspan="3">Source article(s): Hahn et al</td></tr>
<tr><td>Selected by:</td><td colspan="2">default</td></tr>
<tr><td rowspan="2">Sub-constructs</td><td>Intermediate constructs</td><td>Socio-demographic profile; livelihood strategies; social networks; health; food; water; natural disaster and climate change</td></tr>
<tr><td>Directly operationalized constructs</td><td>Dependency ratio; percent of female headed households; households with orphans; uneducated headed households; Households working elsewhere; agriculture dependent</td></tr>
</table>

		household; livelihood diversification; Receive-give ration; borrow-lend ration; independent of local government; Family with chronic illness; proximity to health facility; 2 weeks illness; malaria exposure-prevention; Food from family farm; struggle for food; crop diversity; dont save crops; dont save seeds; Water conflict; natural water source; proximity to water source; inconsistent water supply; inverse water stored; Flood, drought, cyclone events; injury or death from disaster; no warning of disaster; maximum temperature; minimum temperature; average precipitation
Conceptual framework	The LVI includes seven major components: Socio-Demographic Profile, Livelihood Strategies, Social Networks, Health, Food, Water, and Natural Disasters and Climate Variability.<br>[...]<br>Socio-demographic profile<br>Explanation of sub-components<br>Ratio of the population under 15 and over 65 years of age to the population between 19 and 64 years of age.<br>Percent of female-headed households<br>Percent of households where head of household has not attended school<br>Percent of households with orphans<br>[...]<br>Livelihood<br>Percent of households with family member working in a different community<br>Percent of households dependent solely on agriculture as a source of income<br>Percentage of households where the head of the household reports that they have attended 0 years of school.<br>Percentage of households that have at least 1 orphan living in their home. Orphans are children<18 years old who have lost one or both parents.<br>Percentage of households that report at least 1 family member who works outside of the community for their primary work activity.<br>Percentage of households that report only agriculture as a source of income.<br>[...]<br>Social Networks<br>Average Receive:Give ratio (range: 0–15)<br>Average Borrow:Lend Money ratio (range: 0.5–2)<br>[...]<br>Health<br>Average time to health facility (minutes)<br>Percent of households with family member with chronic illness<br>Percent of households where a family member had to miss work or school in the last 2 weeks due to illness<br>Average Malaria Exposure*Prevention Index (range: 0–12)<br>[...]<br>Food<br>Percent of households dependent on family farm for food<br>Average number of months households struggle to find food (range: 0–12)<br>[...]<br>Water<br>Percent of households reporting water conflicts<br>Percent of households that utilize a natural water source<br>Average time to water source (minutes)<br>Percent of households that do not have a consistent water supply<br>Inverse of the average number of liters of water stored per household (range: >0–1)	

<table>
<tr><td colspan="2"></td><td colspan="2">[...]
Natural disasters and climate variability
Average number of flood, drought, and cyclone events in the past 6 years (range: 0–7)
Percent of households that did not receive a warning about the pending natural disasters
Percent of households with an injury or death as a result of the most severe natural disaster in the past 6 years
Mean standard deviation of the daily average maximum temperature by month
Mean standard deviation of the daily average minimum temperature by month
Mean standard deviation of average precipitation by month</td></tr>
<tr><td colspan="2">Operationalization of sub-constructs</td><td colspan="2"></td></tr>
<tr><td rowspan="3">53.</td><td rowspan="3">Dependency ratio</td><td>Data collection</td><td>household surveys</td></tr>
<tr><td>Operational questions</td><td>Table 1 includes an explanation of how each sub-component was quantified, the survey question used to collect the data, the original source of the survey question, and potential sources of bias.
[...]
<table>
<tr><td colspan="3">Table 1 Major components and sub-components comprising the Livelihood Vulnerability Index (LVI) developed for two districts of Mozambique.</td></tr>
<tr><td>Sub-components</td><td>Explanation of sub-components</td><td>Survey question</td></tr>
<tr><td>Dependency ratio</td><td>Ratio of the population under 15 and over 65 years of age to the population between 19 and 64 years of age.</td><td>Could you please list the ages and sexes of every person who eats and sleeps in this house? If you had a visitor who ate and slept here for the last 3 days, please include them as well.</td></tr>
</table></td></tr>
<tr><td>Sampling strategies</td><td>We pilot tested the LVI and LVI–IPCC in the Moma and Mabote
Districts of Mozambique during 2007. These were selected by CARE-Mozambique as representative of coastal and inland communities, respectively, and the climate change issues con- fronting each.
[...]
Based on a sample size calculation (WHO, 2005) at the 95% confidence interval, □10% precision, 50% prevalence,1 and a design effect of 2 to account for cluster sampling, 200 households in each
district were surveyed.2 National 1997 census data that specified the total population in each village was used to select 20 villages in each district using the probability proportional to size method (WHO, 2005; UNICEF, 2008).</td></tr>
</table>

<table>
<tr><td></td><td></td><td></td><td>[...]
1 50% prevalence refers to the point prevalence of the indicators selected for the LVI. This is the default value for sample size calculations when the prevalence of the indicators is unknown.
2 Sample size formula: N = DEFF*[(Z2*p*q)/e2], where N = sample size, DEFF = 2; Z = 1.96 (95% CI), p = 0.5; q = 0.5; e = 0.10.</td></tr>
<tr><td></td><td></td><td>Sample sizes</td><td>We pilot tested the LVI and LVI–IPCC in the Moma and Mabote
Districts
[...]
200 households in each
district were surveyed.2 National 1997 census data that specified the total population in each village was used to select 20 villages in each district</td></tr>
<tr><td></td><td></td><td>Data analysis</td><td>The LVI uses a balanced weighted average approach (Sullivan et al., 2002) where each sub-component contributes equally to the overall index even though each major component is comprised of a different number of sub-components. Becausewe intended to develop an assessment tool accessible to a diverse set of users in resource-poor settings, the LVI formula uses the simple approach of applying equal weights to all major components. This weighting scheme could be adjusted by future users as needed. Because each of the sub-components is measured on a different
scale, it was first necessary to standardize each as an index. The equation used for this conversion was adapted from that used in the Human Development Index to calculate the life expectancy index, which is the ratio of the difference of the actual life expectancy and a pre-selected minimum, and the range of pre- determined maximum and minimum life expectancy (UNDP, 2007):
$index_{sd} = (s_d - s_{min})/(s_{max} - s_{min})$
where sd is the original sub-component for district d, and smin and smax are the minimum and maximum values, respectively, for each sub-component determined using data from both districts.</td></tr>
<tr><td>54.</td><td>percent of female headed households</td><td>Data collection</td><td>Same as previous construct</td></tr>
<tr><td></td><td></td><td>Operational questions</td><td>Table 1 includes an explanation of how each sub-component was quantified, the survey question used to collect the data, the original source of the survey question, and potential sources of bias.
[...]
<table><tr><td colspan="3">Table 1 Major components and sub-components comprising the Livelihood Vulnerability Index (LVI) developed for two districts of Mozambique.</td></tr><tr><td>Sub-components</td><td>Explanation of sub-components</td><td>Survey question</td></tr><tr><td>Percent of female-headed</td><td>Percentage of households</td><td>Are you the head of the</td></tr></table></td></tr>
</table>

<table>
<tr><td></td><td></td><td></td><td><table><tr><td>households</td><td>where the primary adult is female. If a male head is away from the home >6 months per year the female is counted as the head of the household.</td><td>household?</td></tr></table></td></tr>
<tr><td></td><td></td><td>Sampling strategies</td><td>Same as previous construct</td></tr>
<tr><td></td><td></td><td>Sample sizes</td><td>Same as previous construct</td></tr>
<tr><td></td><td></td><td>Data analysis</td><td>Same as previous construct</td></tr>
<tr><td>55.</td><td>households with orphans</td><td>Data collection</td><td>Same as previous construct</td></tr>
<tr><td></td><td></td><td>Operational questions</td><td>Table 1 includes an explanation of how each sub-component was quantified, the survey question used to collect the data, the original source of the survey question, and potential sources of bias.
[...]
<table><tr><td colspan="3">Table 1 Major components and sub-components comprising the Livelihood Vulnerability Index (LVI) developed for two districts of Mozambique.</td></tr><tr><td>Sub-components</td><td>Explanation of sub-components</td><td>Survey question</td></tr><tr><td>Percent of households with orphans</td><td>Percentage of households that have at least 1 orphan living in their home. Orphans are children<18 years old who have lost one or both parents.</td><td>Are there any children less than 18 years old from other families living in your house because one or both of their parents has died?</td></tr></table></td></tr>
<tr><td></td><td></td><td>Sampling strategies</td><td>Same as previous construct</td></tr>
<tr><td></td><td></td><td>Sample sizes</td><td>Same as previous construct</td></tr>
<tr><td></td><td></td><td>Data analysis</td><td>Same as previous construct</td></tr>
<tr><td>56.</td><td>uneducated headed households</td><td>Data collection</td><td>NOT TRANSPARENT</td></tr>
<tr><td></td><td></td><td>Operational questions</td><td></td></tr>
<tr><td></td><td></td><td>Sampling strategies</td><td></td></tr>
<tr><td></td><td></td><td>Sample sizes</td><td></td></tr>
<tr><td></td><td></td><td>Data analysis</td><td></td></tr>
<tr><td>57.</td><td>Households working elsewhere</td><td>Data collection</td><td>Same as for ‘dependency ratio’</td></tr>
<tr><td></td><td></td><td>Operational questions</td><td>Table 1 includes an explanation of how each sub-component was quantified, the survey question used to collect the data, the original source of the survey question, and potential sources of bias.
[...]
<table><tr><td>Table 1 Major components and sub-components comprising the Livelihood Vulnerability Index (LVI)</td></tr></table></td></tr>
</table>

<table>
<tr><td rowspan="4"></td><td rowspan="4"></td><td></td><td><table><tr><td colspan="3">developed for two districts of Mozambique.</td></tr><tr><td>Sub-components</td><td>Explanation of sub-components</td><td>Survey question</td></tr><tr><td>Percent of households with family member working in a different community</td><td>Percentage of households that report at least 1 family member who works outside of the community for their primary work activity.</td><td>How many people in your family go to a different community to work?</td></tr></table></td></tr>
<tr><td>Sampling strategies</td><td>Same as for ‘dependency ratio’</td></tr>
<tr><td>Sample sizes</td><td>Same as for ‘dependency ratio’</td></tr>
<tr><td>Data analysis</td><td>Same as for ‘dependency ratio’</td></tr>
<tr><td rowspan="5">58.</td><td rowspan="5">agriculture dependent household</td><td>Data collection</td><td>Same as previous construct</td></tr>
<tr><td>Operational questions</td><td>Table 1 includes an explanation of how each sub-component was quantified, the survey question used to collect the data, the original source of the survey question, and potential sources of bias.
[...]
<table><tr><td colspan="3">Table 1 Major components and sub-components comprising the Livelihood Vulnerability Index (LVI) developed for two districts of Mozambique.</td></tr><tr><td>Sub-components</td><td>Explanation of sub-components</td><td>Survey question</td></tr><tr><td>Percent of households dependent solely on agriculture as a source of income</td><td>Percentage of households that report only agriculture as a source of income.</td><td>Do you or someone else in your household raise animals? Do you or someone else in your household grow crops? Do you or someone else in your household collect something from the bush, the forest, or lakes and rivers to sell?</td></tr></table></td></tr>
<tr><td>Sampling strategies</td><td>Same as previous construct</td></tr>
<tr><td>Sample sizes</td><td>Same as previous construct</td></tr>
<tr><td>Data analysis</td><td>Same as previous construct</td></tr>
<tr><td rowspan="2">59.</td><td rowspan="2">livelihood diversification</td><td>Data collection</td><td>Same as previous construct</td></tr>
<tr><td>Operational questions</td><td>Table 1 includes an explanation of how each sub-component was quantified, the survey question used to collect the data, the original source of the survey question, and potential sources of bias.</td></tr>
</table>

<table>
<tr><td></td><td></td><td></td><td>[...]
<table><tr><td colspan="3">Table 1 Major components and sub-components comprising the Livelihood Vulnerability Index (LVI) developed for two districts of Mozambique.</td></tr><tr><td>Sub-components</td><td>Explanation of sub-components</td><td>Survey question</td></tr><tr><td>Average Agricultural Livelihood Diversification Index (range: 0.20–1)a</td><td>The inverse of (the number of agricultural livelihood activities +1) reported by a household, e.g., A household that farms, raises animals, and collects natural resources will have a Livelihood Diversification Index = 1/(3 + 1) = 0.25.</td><td>Do you or someone else in your household raise animals? Do you or someone else in your household grow crops? Do you or someone else in your household collect something from the bush, the forest, or lakes and rivers to sell?</td></tr></table></td></tr>
<tr><td></td><td></td><td>Sampling strategies</td><td>Same as previous construct</td></tr>
<tr><td></td><td></td><td>Sample sizes</td><td>Same as previous construct</td></tr>
<tr><td></td><td></td><td>Data analysis</td><td>Same as previous construct</td></tr>
<tr><td>60.</td><td>Receive-give ratio</td><td>Data collection</td><td>Same as previous construct</td></tr>
<tr><td></td><td></td><td>Operational questions</td><td>Table 1 includes an explanation of how each sub-component was quantified, the survey question used to collect the data, the original source of the survey question, and potential sources of bias.
[...]
<table><tr><td colspan="3">Table 1 Major components and sub-components comprising the Livelihood Vulnerability Index (LVI) developed for two districts of Mozambique.</td></tr><tr><td>Sub-components</td><td>Explanation of sub-components</td><td>Survey question</td></tr><tr><td>Average Receive:Give ratio (range: 0–15)</td><td>Ratio of (the number of types of help received by a household in the past month + 1) to (the number of types of help given by a household to someone else in the past month + 1).</td><td>In the past month, did relatives or friends help you and your family: (e.g., Get medical care or medicines, Sell animal products or other goods produced by family, Take care of children) In</td></tr></table></td></tr>
</table>

<table>
<tr><td></td><td></td><td></td><td><table><tr><td></td><td></td><td>the past month, did you and your family help relatives or friends: (same choices as above)</td></tr></table></td></tr>
<tr><td></td><td></td><td>Sampling strategies</td><td>Same as previous construct</td></tr>
<tr><td></td><td></td><td>Sample sizes</td><td>Same as previous construct</td></tr>
<tr><td></td><td></td><td>Data analysis</td><td>Same as previous construct</td></tr>
<tr><td>61.</td><td>borrow-lend ratio</td><td>Data collection</td><td>Same as previous construct</td></tr>
<tr><td></td><td></td><td>Operational questions</td><td>Table 1 includes an explanation of how each sub-component was quantified, the survey question used to collect the data, the original source of the survey question, and potential sources of bias.
[...]
<table><tr><td colspan="3">Table 1 Major components and sub-components comprising the Livelihood Vulnerability Index (LVI) developed for two districts of Mozambique.</td></tr><tr><td>Sub-components</td><td>Explanation of sub-components</td><td>Survey question</td></tr><tr><td>Average Borrow:Lend Money ratio (range: 0.5–2)</td><td>Ratio of a household borrowing money in the past month to a household lending money in the past month, e.g., If a household borrowed money but did not lend money, the ratio = 2:1 or 2 and if they lent money but did not borrow any, the ratio = 1:2 or 0.5.</td><td>Did you borrow any money from relatives or friends in the past month? Did you lend any money to relatives or friends in the past month?</td></tr></table></td></tr>
<tr><td></td><td></td><td>Sampling strategies</td><td>Same as previous construct</td></tr>
<tr><td></td><td></td><td>Sample sizes</td><td>Same as previous construct</td></tr>
<tr><td></td><td></td><td>Data analysis</td><td>Same as previous construct</td></tr>
<tr><td>62.</td><td>independent of local government</td><td>Data collection</td><td>Same as previous construct</td></tr>
<tr><td></td><td></td><td>Operational questions</td><td>Table 1 includes an explanation of how each sub-component was quantified, the survey question used to collect the data, the original source of the survey question, and potential sources of bias.
[...]
<table><tr><td>Table 1 Major components and sub-components</td></tr></table></td></tr>
</table>

<table>
<tr><td></td><td></td><td></td><td><table>
<tr><td colspan="3">comprising the Livelihood Vulnerability Index (LVI) developed for two districts of Mozambique.</td></tr>
<tr><td>Sub-components</td><td>Explanation of sub-components</td><td>Survey question</td></tr>
<tr><td>Percent of households that have not gone to their local government for assistance in the past 12 months</td><td>Percentage of households that reported that they have not asked their local government for any assistance in the past 12 months.</td><td>In the past 12 months, have you or someone in your family gone to your community leader for help?</td></tr>
</table></td></tr>
<tr><td></td><td></td><td>Sampling strategies</td><td>Same as previous construct</td></tr>
<tr><td></td><td></td><td>Sample sizes</td><td>Same as previous construct</td></tr>
<tr><td></td><td></td><td>Data analysis</td><td>Same as previous construct</td></tr>
<tr><td>63.</td><td>Family with chronic illness</td><td>Data collection</td><td>Same as previous construct</td></tr>
<tr><td></td><td></td><td>Operational questions</td><td>Table 1 includes an explanation of how each sub-component was quantified, the survey question used to collect the data, the original source of the survey question, and potential sources of bias.
[...]
<table>
<tr><td colspan="3">Table 1 Major components and sub-components comprising the Livelihood Vulnerability Index (LVI) developed for two districts of Mozambique.</td></tr>
<tr><td>Sub-components</td><td>Explanation of sub-components</td><td>Survey question</td></tr>
<tr><td>Percent of households with family member with chronic illness</td><td>Percentage of households that report at least 1 family member with chronic illness. Chronic illness was defined subjectively by respondent.</td><td>Is anybody in your family chronically ill (they get sick very often)?</td></tr>
</table></td></tr>
<tr><td></td><td></td><td>Sampling strategies</td><td>Same as previous construct</td></tr>
<tr><td></td><td></td><td>Sample sizes</td><td>Same as previous construct</td></tr>
<tr><td></td><td></td><td>Data analysis</td><td>Same as previous construct</td></tr>
<tr><td>64.</td><td>proximity to health facility</td><td>Data collection</td><td>Same as previous construct</td></tr>
<tr><td></td><td></td><td>Operational questions</td><td>Table 1 includes an explanation of how each sub-component was quantified, the survey question used to collect the data, the original source of the survey question, and potential sources of bias.
[...]
<table>
<tr><td colspan="3">Table 1 Major components and sub-components comprising the Livelihood Vulnerability Index (LVI) developed for two districts of Mozambique.</td></tr>
<tr><td>Sub-components</td><td>Explanation of</td><td>Survey question</td></tr>
</table></td></tr>
</table>

<table>
<tr><td rowspan="4"></td><td rowspan="4"></td><td></td><td><table><tr><td></td><td>sub-components</td><td></td></tr><tr><td>Average time to health facility (minutes)</td><td>Average time it takes the households to get to the nearest health facility.</td><td>Howlong does it take you to get to a health facility?</td></tr></table></td></tr>
<tr><td>Sampling strategies</td><td>Same as previous construct</td></tr>
<tr><td>Sample sizes</td><td>Same as previous construct</td></tr>
<tr><td>Data analysis</td><td>Same as previous construct</td></tr>
<tr><td rowspan="5">65.</td><td rowspan="5">2 weeks illness</td><td>Data collection</td><td>Same as previous construct</td></tr>
<tr><td>Operational questions</td><td>Table 1 includes an explanation of how each sub-component was quantified, the survey question used to collect the data, the original source of the survey question, and potential sources of bias.
[...]
<table><tr><td colspan="3">Table 1 Major components and sub-components comprising the Livelihood Vulnerability Index (LVI) developed for two districts of Mozambique.</td></tr><tr><td>Sub-components</td><td>Explanation of sub-components</td><td>Survey question</td></tr><tr><td>Percent of households where a family member had to miss work or school in the last 2 weeks due to illness</td><td>Percentage of households that report at least 1 family member who had to miss school of work due to illness in the last 2 weeks.</td><td>Has anyone in your family been so sick in the past 2 weeks that they had to miss work or school?</td></tr></table></td></tr>
<tr><td>Sampling strategies</td><td>Same as previous construct</td></tr>
<tr><td>Sample sizes</td><td>Same as previous construct</td></tr>
<tr><td>Data analysis</td><td>Same as previous construct</td></tr>
<tr><td rowspan="2">66.</td><td rowspan="2">malaria exposure-prevention</td><td>Data collection</td><td>Same as previous construct</td></tr>
<tr><td>Operational questions</td><td>Table 1 includes an explanation of how each sub-component was quantified, the survey question used to collect the data, the original source of the survey question, and potential sources of bias.
[...]
<table><tr><td colspan="3">Table 1 Major components and sub-components comprising the Livelihood Vulnerability Index (LVI) developed for two districts of Mozambique.</td></tr><tr><td>Sub-components</td><td>Explanation of sub-components</td><td>Survey question</td></tr><tr><td>Average Malaria Exposure*Prevention Index (range: 0–12)</td><td>Months reported exposure to malaria*Owning at least one bednet indicator (have bednet =</td><td>Which months of the year is malaria particularly bad? How many mosquito nets</td></tr></table></td></tr>
</table>

<table>
<tr><td></td><td></td><td></td><td><table><tr><td></td><td>0.5, no bednet = 1) (e.g., Respondent reported malaria is a problem January–March and they do not own a bednet = 3*1 = 3).</td><td>do you have?</td></tr></table></td></tr>
<tr><td></td><td></td><td>Sampling strategies</td><td>Same as previous construct</td></tr>
<tr><td></td><td></td><td>Sample sizes</td><td>Same as previous construct</td></tr>
<tr><td></td><td></td><td>Data analysis</td><td>Same as previous construct</td></tr>
<tr><td>67.</td><td>Food from family farm</td><td>Data collection</td><td>Same as previous construct</td></tr>
<tr><td></td><td></td><td>Operational questions</td><td>Table 1 includes an explanation of how each sub-component was quantified, the survey question used to collect the data, the original source of the survey question, and potential sources of bias.
[...]

<table><tr><td colspan="3">Table 1 Major components and sub-components comprising the Livelihood Vulnerability Index (LVI) developed for two districts of Mozambique.</td></tr><tr><td>Sub-components</td><td>Explanation of sub-components</td><td>Survey question</td></tr><tr><td>Percent of households dependent on family farm for food</td><td>Percentage of households that get their food primarily from their personal farms.</td><td>Where does your family get most of its food?</td></tr></table></td></tr>
<tr><td></td><td></td><td>Sampling strategies</td><td>Same as previous construct</td></tr>
<tr><td></td><td></td><td>Sample sizes</td><td>Same as previous construct</td></tr>
<tr><td></td><td></td><td>Data analysis</td><td>Same as previous construct</td></tr>
<tr><td>68.</td><td>struggle for food</td><td>Data collection</td><td>Same as previous construct</td></tr>
<tr><td></td><td></td><td>Operational questions</td><td>Table 1 includes an explanation of how each sub-component was quantified, the survey question used to collect the data, the original source of the survey question, and potential sources of bias.
[...]

<table><tr><td colspan="3">Table 1 Major components and sub-components comprising the Livelihood Vulnerability Index (LVI) developed for two districts of Mozambique.</td></tr><tr><td>Sub-components</td><td>Explanation of sub-components</td><td>Survey question</td></tr><tr><td>Average number of months households struggle to find food (range: 0–12)</td><td>Average number of months households struggle to obtain food for their family.</td><td>Does your family have adequate food the whole year, or are there times during the year that your family does not</td></tr></table></td></tr>
</table>

<table>
<tr><td></td><td></td><td></td><td><table><tr><td></td><td></td><td>have enough food? Howmanymonths a year does your family have trouble getting enough food?</td></tr></table></td></tr>
<tr><td></td><td></td><td>Sampling strategies</td><td>Same as previous construct</td></tr>
<tr><td></td><td></td><td>Sample sizes</td><td>Same as previous construct</td></tr>
<tr><td></td><td></td><td>Data analysis</td><td>Same as previous construct</td></tr>
<tr><td>69.</td><td>crop diversity</td><td>Data collection</td><td>Same as previous construct</td></tr>
<tr><td></td><td></td><td>Operational questions</td><td>Table 1 includes an explanation of how each sub-component was quantified, the survey question used to collect the data, the original source of the survey question, and potential sources of bias.
[...]
<table><tr><td colspan="3">Table 1 Major components and sub-components comprising the Livelihood Vulnerability Index (LVI) developed for two districts of Mozambique.</td></tr><tr><td>Sub-components</td><td>Explanation of sub-components</td><td>Survey question</td></tr><tr><td>Average Crop Diversity Index (range: >0–1)a</td><td>The inverse of (the number of crops grown by a household +1). e.g., A household that grows pumpkin, maize, nhemba beans, and cassava will have a Crop Diversity</td><td>What kind of crops does your household grow?</td></tr></table></td></tr>
<tr><td></td><td></td><td>Sampling strategies</td><td>Same as previous construct</td></tr>
<tr><td></td><td></td><td>Sample sizes</td><td>Same as previous construct</td></tr>
<tr><td></td><td></td><td>Data analysis</td><td>Same as previous construct</td></tr>
<tr><td>70.</td><td>dont save crops</td><td>Data collection</td><td>Same as previous construct</td></tr>
<tr><td></td><td></td><td>Operational questions</td><td>Table 1 includes an explanation of how each sub-component was quantified, the survey question used to collect the data, the original source of the survey question, and potential sources of bias.
[...]
<table><tr><td colspan="3">Table 1 Major components and sub-components comprising the Livelihood Vulnerability Index (LVI) developed for two districts of Mozambique.</td></tr><tr><td>Sub-components</td><td>Explanation of sub-components</td><td>Survey question</td></tr><tr><td>Percent of households that do not save crops</td><td>Percentage of households that do not save crops from each</td><td>Does your family save some of the crops you harvest to eat</td></tr></table></td></tr>
</table>

<table>
<tr><td></td><td></td><td></td><td><table><tr><td></td><td>harvest.</td><td>during a different time of year?</td></tr></table></td></tr>
<tr><td></td><td></td><td>Sampling strategies</td><td>Same as previous construct</td></tr>
<tr><td></td><td></td><td>Sample sizes</td><td>Same as previous construct</td></tr>
<tr><td></td><td></td><td>Data analysis</td><td>Same as previous construct</td></tr>
<tr><td>71.</td><td>dont save seeds</td><td>Data collection</td><td>Same as previous construct</td></tr>
<tr><td></td><td></td><td>Operational questions</td><td>Table 1 includes an explanation of how each sub-component was quantified, the survey question used to collect the data, the original source of the survey question, and potential sources of bias.
[...]
<table><tr><td colspan="3">Table 1 Major components and sub-components comprising the Livelihood Vulnerability Index (LVI) developed for two districts of Mozambique.</td></tr><tr><td>Sub-components</td><td>Explanation of sub-components</td><td>Survey question</td></tr><tr><td>Percent of households that do not save seeds</td><td>Percentage of households that do not have seeds from year to year.</td><td>Does your family save seeds to grow the next year?</td></tr></table></td></tr>
<tr><td></td><td></td><td>Sampling strategies</td><td>Same as previous construct</td></tr>
<tr><td></td><td></td><td>Sample sizes</td><td>Same as previous construct</td></tr>
<tr><td></td><td></td><td>Data analysis</td><td>Same as previous construct</td></tr>
<tr><td>72.</td><td>Water conflict</td><td>Data collection</td><td>Same as previous construct</td></tr>
<tr><td></td><td></td><td>Operational questions</td><td>Table 1 includes an explanation of how each sub-component was quantified, the survey question used to collect the data, the original source of the survey question, and potential sources of bias.
[...]
<table><tr><td colspan="3">Table 1 Major components and sub-components comprising the Livelihood Vulnerability Index (LVI) developed for two districts of Mozambique.</td></tr><tr><td>Sub-components</td><td>Explanation of sub-components</td><td>Survey question</td></tr><tr><td>Percent of households reporting water conflicts</td><td>Percentage of households that report having heard about conflicts over water in their community.</td><td>In the past year, have you heard about any conflicts over water in your community?</td></tr></table></td></tr>
<tr><td></td><td></td><td>Sampling strategies</td><td>Same as previous construct</td></tr>
<tr><td></td><td></td><td>Sample sizes</td><td>Same as previous construct</td></tr>
<tr><td></td><td></td><td>Data analysis</td><td>Same as previous construct</td></tr>
<tr><td></td><td></td><td>Sampling strategies</td><td>Same as previous construct</td></tr>
<tr><td></td><td></td><td>Sample sizes</td><td>Same as previous construct</td></tr>
<tr><td></td><td></td><td>Data analysis</td><td>Same as previous construct</td></tr>
<tr><td>73.</td><td>natural water</td><td>Data collection</td><td>Same as previous construct</td></tr>
</table>

<table>
<tr><td rowspan="4"></td><td rowspan="4">source</td><td>Operational questions</td><td>Table 1 includes an explanation of how each sub-component was quantified, the survey question used to collect the data, the original source of the survey question, and potential sources of bias.
[...]
<table>
<tr><td colspan="3">Table 1 Major components and sub-components comprising the Livelihood Vulnerability Index (LVI) developed for two districts of Mozambique.</td></tr>
<tr><td>Sub-components</td><td>Explanation of sub-components</td><td>Survey question</td></tr>
<tr><td>Percent of households that utilize a natural water source</td><td>Percentage of households that report a creek, river, lake, pool, or hole as their primary water source.</td><td>Where do you collect your water from?</td></tr>
</table></td></tr>
<tr><td>Sampling strategies</td><td>Same as previous construct</td></tr>
<tr><td>Sample sizes</td><td>Same as previous construct</td></tr>
<tr><td>Data analysis</td><td>Same as previous construct</td></tr>
<tr><td rowspan="5">74.</td><td rowspan="5">proximity to water source</td><td>Data collection</td><td>Same as previous construct</td></tr>
<tr><td>Operational questions</td><td>Table 1 includes an explanation of how each sub-component was quantified, the survey question used to collect the data, the original source of the survey question, and potential sources of bias.
[...]
<table>
<tr><td colspan="3">Table 1 Major components and sub-components comprising the Livelihood Vulnerability Index (LVI) developed for two districts of Mozambique.</td></tr>
<tr><td>Sub-components</td><td>Explanation of sub-components</td><td>Survey question</td></tr>
<tr><td>Average time to water source (minutes)</td><td>Average time it takes the households to travel to their primary water source.</td><td>How long does it take to get to your water source?</td></tr>
</table></td></tr>
<tr><td>Sampling strategies</td><td>Same as previous construct</td></tr>
<tr><td>Sample sizes</td><td>Same as previous construct</td></tr>
<tr><td>Data analysis</td><td>Same as previous construct</td></tr>
<tr><td rowspan="2">75.</td><td rowspan="2">inconsistent water supply</td><td>Data collection</td><td>Same as previous construct</td></tr>
<tr><td>Operational questions</td><td>Table 1 includes an explanation of how each sub-component was quantified, the survey question used to collect the data, the original source of the survey question, and potential sources of bias.
[...]
<table>
<tr><td>Table 1 Major components and sub-components comprising the Livelihood Vulnerability Index (LVI)</td></tr>
</table></td></tr>
</table>

<table>
<tr><td></td><td></td><td></td><td><table><tr><td colspan="3">developed for two districts of Mozambique.</td></tr><tr><td>Sub-components</td><td>Explanation of sub-components</td><td>Survey question</td></tr><tr><td>Percent of households that do not have a consistent water supply</td><td>Percentage of households that report that water is not available at their primary water source everyday</td><td>Is this water available everyday?</td></tr></table></td></tr>
<tr><td></td><td></td><td>Sampling strategies</td><td>Same as previous construct</td></tr>
<tr><td></td><td></td><td>Sample sizes</td><td>Same as previous construct</td></tr>
<tr><td></td><td></td><td>Data analysis</td><td>Same as previous construct</td></tr>
<tr><td>76.</td><td>inverse water stored</td><td>Data collection</td><td>Same as previous construct</td></tr>
<tr><td></td><td></td><td>Operational questions</td><td>Table 1 includes an explanation of how each sub-component was quantified, the survey question used to collect the data, the original source of the survey question, and potential sources of bias.
[...]<table><tr><td colspan="3">Table 1 Major components and sub-components comprising the Livelihood Vulnerability Index (LVI) developed for two districts of Mozambique.</td></tr><tr><td>Sub-components</td><td>Explanation of sub-components</td><td>Survey question</td></tr><tr><td>Inverse of the average number of liters of water stored per household (range: >0–1)</td><td>The inverse of (the average number of liters of water stored by each household + 1).</td><td>What containers do you usually store water in? How many? How many liters are they?</td></tr></table></td></tr>
<tr><td></td><td></td><td>Sampling strategies</td><td>Same as previous construct</td></tr>
<tr><td></td><td></td><td>Sample sizes</td><td>Same as previous construct</td></tr>
<tr><td></td><td></td><td>Data analysis</td><td>Same as previous construct</td></tr>
<tr><td>77.</td><td>Flood, drought, cyclone events</td><td>Data collection</td><td>Same as previous construct</td></tr>
<tr><td></td><td></td><td>Operational questions</td><td>Table 1 includes an explanation of how each sub-component was quantified, the survey question used to collect the data, the original source of the survey question, and potential sources of bias.
[...]<table><tr><td colspan="3">Table 1 Major components and sub-components comprising the Livelihood Vulnerability Index (LVI) developed for two districts of Mozambique.</td></tr><tr><td>Sub-components</td><td>Explanation of sub-components</td><td>Survey question</td></tr><tr><td>Average number of flood, drought, and cyclone events in the</td><td>Total number of floods, droughts, and cyclones that were reported</td><td>How many times has this area been affected by a flood/cyclone/drought in 2001–2007?</td></tr></table></td></tr>
</table>

<table>
<tr><td></td><td></td><td></td><td><table><tr><td>past 6 years (range: 0–7)</td><td>by households in the past 6 years.</td><td></td></tr></table></td></tr>
<tr><td></td><td></td><td>Sampling strategies</td><td>Same as previous construct</td></tr>
<tr><td></td><td></td><td>Sample sizes</td><td>Same as previous construct</td></tr>
<tr><td></td><td></td><td>Data analysis</td><td>Same as previous construct</td></tr>
<tr><td>78.</td><td>injury or death from disaster</td><td>Data collection</td><td>Same as previous construct</td></tr>
<tr><td></td><td></td><td>Operational questions</td><td>Table 1 includes an explanation of how each sub-component was quantified, the survey question used to collect the data, the original source of the survey question, and potential sources of bias.
[...]

<table><tr><td colspan="3">Table 1 Major components and sub-components comprising the Livelihood Vulnerability Index (LVI) developed for two districts of Mozambique.</td></tr><tr><td>Sub-components</td><td>Explanation of sub-components</td><td>Survey question</td></tr><tr><td>Percent of households with an injury or death as a result of the most severe natural disaster in the past 6 years</td><td>Percentage of households that reported either an injury to or death of one of their family members as a result of the most severe flood, drought, or cyclone in the past 6 years.</td><td>Was anyone in your family injured in the flood/cyclone drought?
Did anyone in your family die during the flood/cyclone/drought?</td></tr></table></td></tr>
<tr><td></td><td></td><td>Sampling strategies</td><td>Same as previous construct</td></tr>
<tr><td></td><td></td><td>Sample sizes</td><td>Same as previous construct</td></tr>
<tr><td></td><td></td><td>Data analysis</td><td>Same as previous construct</td></tr>
<tr><td>79.</td><td>no warning of disaster</td><td>Data collection</td><td>Same as previous construct</td></tr>
<tr><td></td><td></td><td>Operational questions</td><td>Table 1 includes an explanation of how each sub-component was quantified, the survey question used to collect the data, the original source of the survey question, and potential sources of bias.
[...]

<table><tr><td colspan="3">Table 1 Major components and sub-components comprising the Livelihood Vulnerability Index (LVI) developed for two districts of Mozambique.</td></tr><tr><td>Sub-components</td><td>Explanation of sub-components</td><td>Survey question</td></tr><tr><td>Percent of households that did not</td><td>Percentage of households that did not</td><td>Did you receive a warning about the flood/cyclone/drought</td></tr></table></td></tr>
</table>

<table>
<tr><td></td><td></td><td></td><td><table><tr><td>receive a warning about the pending natural disasters</td><td>receive a warning about the most severe flood, drought, and cyclone event in the past 6 years.</td><td>before it happened?</td></tr></table></td></tr>
<tr><td></td><td></td><td>Sampling strategies</td><td>Same as previous construct</td></tr>
<tr><td></td><td></td><td>Sample sizes</td><td>Same as previous construct</td></tr>
<tr><td></td><td></td><td>Data analysis</td><td>Same as previous construct</td></tr>
<tr><td>80.</td><td>maximum temperature</td><td>Data collection</td><td>provincial data; weather station based in the provincial capital</td></tr>
<tr><td></td><td></td><td>Operational questions</td><td><table><tr><td colspan="3">Table 1 Major components and sub-components comprising the Livelihood Vulnerability Index (LVI) developed for two districts of Mozambique.</td></tr><tr><td>Sub-components</td><td>Explanation of sub-components</td><td>Survey question</td></tr><tr><td>Mean standard deviation of the daily average maximum temperature by month</td><td>Standard deviation of the average daily maximum temperature by month between 1998 and 2003 was averaged for each provinceb</td><td>1998–2003: provincial data; weather station based in the provincial capital</td></tr></table></td></tr>
<tr><td></td><td></td><td>Sampling strategies</td><td></td></tr>
<tr><td></td><td></td><td>Sample sizes</td><td></td></tr>
<tr><td></td><td></td><td>Data analysis</td><td>Same as previous construct</td></tr>
<tr><td>81.</td><td>minimum temperature</td><td>Data collection</td><td>provincial data; weather station based in the provincial capital</td></tr>
<tr><td></td><td></td><td>Operational questions</td><td><table><tr><td colspan="3">Table 1 Major components and sub-components comprising the Livelihood Vulnerability Index (LVI) developed for two districts of Mozambique.</td></tr><tr><td>Sub-components</td><td>Explanation of sub-components</td><td>Survey question</td></tr><tr><td>Mean standard deviation of the daily average minimum temperature by month</td><td>Standard deviation of the average daily minimum temperature by month between 1998 and 2003 was averaged for each province.</td><td>1998–2003: provincial data; weather station based in the provincial capital</td></tr></table></td></tr>
<tr><td></td><td></td><td>Sampling strategies</td><td></td></tr>
<tr><td></td><td></td><td>Sample sizes</td><td></td></tr>
</table>

<table>
<tr><td></td><td></td><td>Data analysis</td><td></td></tr>
<tr><td rowspan="5">82.</td><td rowspan="5">average precipitation</td><td>Data collection</td><td>provincial data; weather station based in the provincial capital</td></tr>
<tr><td>Operational questions</td><td>
<table>
<tr><td colspan="3">Table 1 Major components and sub-components comprising the Livelihood Vulnerability Index (LVI) developed for two districts of Mozambique.</td></tr>
<tr><td>Sub-components</td><td>Explanation of sub-components</td><td>Survey question</td></tr>
<tr><td>Mean standard deviation of average precipitation by month</td><td>Standard deviation of the average monthly precipitation between 1998 and 2003 was averaged for each province</td><td>1998–2003: provincial data; weather station based in the provincial capital</td></tr>
</table>
</td></tr>
<tr><td>Sampling strategies</td><td></td></tr>
<tr><td>Sample sizes</td><td></td></tr>
<tr><td>Data analysis</td><td>Same as previous construct</td></tr>
<tr><td colspan="2">Candidate-level Analysis</td><td colspan="2">Same as ‘dependency ratio’</td></tr>
</table>

Intensifying vulnerability to food insecurity

Constructs:

- Livelihood strategies

Constructs in report of frameworks	Represented in list by	Coded text indicating relationship
Livelihood level issues		No operationalized representative
Access to sufficient food		No operationalized representative
Food insecurity		No operationalized representative
Household and community vulnerability		No operationalized representative
Livelihood strategies	Livelihood strategies	Direct representation
Direct drivers		No operationalized representative

<table>
<tr><td colspan="4">Operationalization of constructs</td></tr>
<tr><td colspan="4">Construct operationalized: Livelihood strategies</td></tr>
<tr><td colspan="4">Source article(s): Hahn et al</td></tr>
<tr><td>Selected by:</td><td colspan="3">Default</td></tr>
<tr><td rowspan="2">Sub-constructs</td><td>Intermediate constructs</td><td colspan="2"></td></tr>
<tr><td>Directly operationalized constructs</td><td colspan="2">Households working elsewhere; agriculture dependent household; livelihood diversification</td></tr>
<tr><td>Conceptual framework</td><td colspan="3">Livelihood
Percent of households with family member working in a different community
Percent of households dependent solely on agriculture as a source of income
Percentage of households where the head of the household reports that they have attended 0 years of school.
Percentage of households that have at least 1 orphan living in their home. Orphans are children<18 years old who have lost one or both parents.
Percentage of households that report at least 1 family member who works outside of the community for their primary work activity.
Percentage of households that report only agriculture as a source of income.</td></tr>
<tr><td>Operationalization of sub-constructs</td><td colspan="3"></td></tr>
<tr><td rowspan="2">83. Households working elsewhere</td><td>Data collection</td><td colspan="2">Household survey</td></tr>
<tr><td>Operational questions</td><td colspan="2">Table 1 includes an explanation of how each sub-component was quantified, the survey question used to collect the data, the original source of the survey question, and potential sources of bias.
[...]

Table 1 Major components and sub-components comprising the Livelihood Vulnerability Index (LVI) developed for two districts of Mozambique.
<table><tr><td>Sub-components</td><td>Explanation of sub-components</td><td>Survey question</td></tr><tr><td>Percent of households with family member working in a</td><td>Percentage of households that report at least 1 family member</td><td>How many people in your family go to a different</td></tr></table></td></tr>
</table>

			different community / who works outside of the community for their primary work activity. / community to work?
		Sampling strategies	We pilot tested the LVI and LVI–IPCC in the Moma and Mabote<br>Districts of Mozambique during 2007. These were selected by CARE-Mozambique as representative of coastal and inland communities, respectively, and the climate change issues con- fronting each.<br>[...]<br>Based on a sample size calculation (WHO, 2005) at the 95% confidence interval, □10% precision, 50% prevalence,1 and a design effect of 2 to account for cluster sampling, 200 households in each<br>district were surveyed.2 National 1997 census data that specified the total population in each village was used to select 20 villages in each district using the probability proportional to size method (WHO, 2005; UNICEF, 2008).<br>[...]<br>1 50% prevalence refers to the point prevalence of the indicators selected for the LVI. This is the default value for sample size calculations when the prevalence of the indicators is unknown.<br>2 Sample size formula: N = DEFF*[(Z2*p*q)/e2], where N = sample size, DEFF = 2; Z = 1.96 (95% CI), p = 0.5; q = 0.5; e = 0.10.
		Sample sizes	We pilot tested the LVI and LVI–IPCC in the Moma and Mabote<br>Districts<br>[...]<br>200 households in each<br>district were surveyed.2 National 1997 census data that specified the total population in each village was used to select 20 villages in each district
		Data analysis	The LVI uses a balanced weighted average approach (Sullivan et al., 2002) where each sub-component contributes equally to the overall index even though each major component is comprised of a different number of sub-components. Becausewe intended to develop an assessment tool accessible to a diverse set of users in resource-poor settings, the LVI formula uses the simple approach of applying equal weights to all major components. This weighting scheme could be adjusted by future users as needed. Because each of the sub-components is measured on a different<br>scale, it was first necessary to standardize each as an index. The equation used for this conversion was adapted from that used in the Human Development Index to calculate the life expectancy index, which is the ratio of the difference of the actual life expectancy and a pre-selected minimum, and the range of pre- determined maximum and minimum life expectancy (UNDP, 2007):

<table>
<tr><td></td><td></td><td></td><td>$index_{sd} = (s_d - s_{min})/(s_{max} - s_{min})$
where sd is the original sub-component for district d, and smin and smax are the minimum and maximum values, respectively, for each sub-component determined using data from both districts.</td></tr>
<tr><td rowspan="5">84.</td><td rowspan="5">agriculture dependent household</td><td>Data collection</td><td>Same as previous construct</td></tr>
<tr><td>Operational questions</td><td>Table 1 includes an explanation of how each sub-component was quantified, the survey question used to collect the data, the original source of the survey question, and potential sources of bias.
[...]
<table>
<tr><td colspan="3">Table 1 Major components and sub-components comprising the Livelihood Vulnerability Index (LVI) developed for two districts of Mozambique.</td></tr>
<tr><td>Sub-components</td><td>Explanation of sub-components</td><td>Survey question</td></tr>
<tr><td>Percent of households dependent solely on agriculture as a source of income</td><td>Percentage of households that report only agriculture as a source of income.</td><td>Do you or someone else in your household raise animals? Do you or someone else in your household grow crops? Do you or someone else in your household collect something from the bush, the forest, or lakes and rivers to sell?</td></tr>
</table></td></tr>
<tr><td>Sampling strategies</td><td>Same as previous construct</td></tr>
<tr><td>Sample sizes</td><td>Same as previous construct</td></tr>
<tr><td>Data analysis</td><td>Same as previous construct</td></tr>
<tr><td rowspan="2">85.</td><td rowspan="2">livelihood diversification</td><td>Data collection</td><td>Same as previous construct</td></tr>
<tr><td>Operational questions</td><td>Table 1 includes an explanation of how each sub-component was quantified, the survey question used to collect the data, the original source of the survey question, and potential sources of bias.
[...]
<table>
<tr><td colspan="3">Table 1 Major components and sub-components comprising the Livelihood Vulnerability Index (LVI) developed for two districts of Mozambique.</td></tr>
<tr><td>Sub-components</td><td>Explanation of sub-components</td><td>Survey question</td></tr>
<tr><td>Average Agricultural Livelihood</td><td>The inverse of (the number of agricultural</td><td>Do you or someone else in your household</td></tr>
</table></td></tr>
</table>

<table>
<tr><td rowspan="5"></td><td rowspan="5"></td><td></td><td><table><tr><td>Diversification Index (range: 0.20–1)a</td><td>livelihood activities +1) reported by a household, e.g., A household that farms, raises animals, and collects natural resources will have a Livelihood Diversification Index = 1/(3 + 1) = 0.25.</td><td>raise animals? Do you or someone else in your household grow crops? Do you or someone else in your household collect something from the bush, the forest, or lakes and rivers to sell?</td></tr></table></td></tr>
<tr><td>Sampling strategies</td><td>Same as previous construct</td></tr>
<tr><td>Sample sizes</td><td>Same as previous construct</td></tr>
<tr><td>Data analysis</td><td>Same as previous construct</td></tr>
<tr><td>Data analysis</td><td></td></tr>
<tr><td colspan="2">Candidate-level Analysis</td><td colspan="2">The LVI uses a balanced weighted average approach (Sullivan
et al., 2002) where each sub-component contributes equally to the overall index even though each major component is comprised of a different number of sub-components. Becausewe intended to develop an assessment tool accessible to a diverse set of users in resource-poor settings, the LVI formula uses the simple approach of applying equal weights to all major components. This weighting scheme could be adjusted by future users as needed. Because each of the sub-components is measured on a different scale, it was first necessary to standardize each as an index. The equation used for this conversion was adapted from that used in the Human Development Index to calculate the life expectancy index, which is the ratio of the difference of the actual life expectancy and a pre-selected minimum, and the range of pre- determined maximum and minimum life expectancy (UNDP, 2007):
$index_{sd} = (s_d - s_{min})/(s_{max} - s_{min})$
where sd is the original sub-component for district d, and smin and smax are the minimum and maximum values, respectively, for each sub-component determined using data from both districts.</td></tr>
</table>

Nkondze et al (2013)

Constructs:

Constructs in report of frameworks	Represented in list by	Coded text indicating relationship
Factors affecting vulnerability		No operationalized representative
Household vulnerability to climate change		No operationalized representative

Patterns of smallholder vulnerability

Constructs:

- Vulnerability IPCC
- Cluster pattern analysis

Constructs in report of frameworks	Represented in list by	Coded text indicating relationship
Vulnerability IPCC	Vulnerability IPCC	Directly represented
Exposure	Vulnerability IPCC	Climate vulnerability is considered as a function of expo- sure, sensitivity and coping/adaptive capacity (IPCC 2007).
Sensitivity (A)	Vulnerability IPCC	Climate vulnerability is considered as a function of expo- sure, sensitivity and coping/adaptive capacity (IPCC 2007).
Adaptive capacity (C)	Vulnerability IPCC	Climate vulnerability is considered as a function of expo- sure, sensitivity and coping/adaptive capacity (IPCC 2007).
Cluster pattern analysis	Cluster pattern analysis	Directly represented
Food security	Vulnerability IPCC	Therefore,we investigate as to whether there are typical characteristics of smallholder households that help to explain the causal structure of their vulnerability to weather extremes in relation to food security.

<table>
<tr><td colspan="4">Operationalization of constructs</td></tr>
<tr><td colspan="4">Construct operationalized: Vulnerability IPCC</td></tr>
<tr><td colspan="4">Source article(s):</td></tr>
<tr><td colspan="2">Selected by:</td><td colspan="2">expert selection [justification]</td></tr>
<tr><td colspan="2" rowspan="2">Sub-constructs</td><td>Intermediate constructs</td><td></td></tr>
<tr><td>Directly operationalized constructs</td><td></td></tr>
<tr><td colspan="2">Conceptual framework</td><td colspan="2"></td></tr>
<tr><td colspan="2">Operationalization of sub-constructs</td><td colspan="2"></td></tr>
<tr><td rowspan="5">86.</td><td rowspan="5">•</td><td>Data collection</td><td></td></tr>
<tr><td>Operational questions</td><td></td></tr>
<tr><td>Sampling strategies</td><td></td></tr>
<tr><td>Sample sizes</td><td></td></tr>
<tr><td>Data analysis</td><td></td></tr>
<tr><td rowspan="5">87.</td><td rowspan="5">•</td><td>Data collection</td><td></td></tr>
<tr><td>Operational questions</td><td></td></tr>
<tr><td>Sampling strategies</td><td></td></tr>
<tr><td>Sample sizes</td><td></td></tr>
<tr><td>Data analysis</td><td></td></tr>
</table>

88.	•	Data collection	
		Operational questions	
		Sampling strategies	
		Sample sizes	
		Data analysis	
89.	•	Data collection	
		Operational questions	
		Sampling strategies	
		Sample sizes	
		Data analysis	
Candidate-level Analysis			

**Operationalization of constructs**			
**Construct operationalized:** Cluster Pattern analysis			
**Source article(s):** Sietz et al			
**Selected by:**	default/expert selection [justification]		
Sub-constructs	Intermediate constructs		
	Directly operationalized constructs		
Conceptual framework	DIRECT OPERATIONALIZATION		
Operationalization of sub-constructs			
90.		Data collection	The ALTAGRO (2006) data base contains detailed quantitative information for 527 smallholder households collected through household questionnaires.<br>[...]<br>The necessary weather information is available in good quality for the 1996–2006 period for two stations located in Puno and Cabanillas (see Fig. 1). Table 2 shows the average pre- cipitation and temperature for both stations.
		Operational questions	Ten categories describe the smallholder households covering personal information about the family members (e.g. occupation, education level, age), production systems (e.g. crop and livestock assets, labour input, processing and commer- cialisation of produce), weather conditions, food reserves, income, some expenses and credits.<br>[...]<br>The following data are taken from the ALTAGRO (2006) data base to indicate the mechanisms relevant in this study. As the first dimension, the harvest failure risk is indicated by the number of production zones used for crop and pasture cultivation. The indicator considers plains, hillsides and hills. The second dimension of the area con- straint is measured by the crop area as an important pre- requisite for food production. The pasture area highly correlates to livestock keeping and is therefore reflected in the livestock measure. The third dimension, the livestock constraint, is characterised by the number and types of

animals. To compare various animal species, we calculated standardised livestock units in relation to an improved cattle variety based on the livestock-specific metabolism (Kleiber 1961). Average livestock weights were estimated using 20 representative animals of each species in the study region. Since fodder production is an essential condition for livestock keeping, the respective indicator contains a reference to the area and productivity of pasture land. Furthermore, the productivity constraint as the fourth dimension is provided for the major food crops potatoes and quinua. It averages the household's productivity across species, varieties and production zones for each crop. Again, we concentrate on food crops since the productivity of pastures is already included in the livestock measure. The fifth dimension of education deprivation relates to the number of years that a household head attended school. School attendance is classified according to the four levels: no formal education, primary, secondary and higher edu- cation. Finally, the lack of alternative income as the sixth dimension is quantified by the sum of annual monetary income from local off-farm activities and remittances. People usually receive remittances from household
495
members who migrate for climate-independent labour, for example mining and commerce. Table 1 summarises the indicators used to assess vulnerability.
[...]

Table 1 Indicators of households' sensitivity and adaptive capacity. The range of the area and livestock constraints as well as lack of alternative income is provided following winsorisation, see description in text. (Data source: ALTAGRO 2006)

Dimension of sensitivity and adaptive capacity	*Indicator*	*Range*
Harvest failure risk	Number of production zones used for cultivation	1–3
Area constraint	Crop area	0.1–1.3 ha/person[a]
Livestock constraint	Livestock units	0.1–8.0 livestock units/person
Productivity constraint	Potato productivity<br>Quinua productivity	0.1–10.0 t/ha<br>0.2–1.8 t/ha
Education deprivation	Education level of household head	1–4
Lack of alternative income	Local off-farm income and remittances	0–2400 Soles/year*person

a Average: 4 persons per household

[...]
The necessary weather information is available in good quality for the 1996–2006 period for two stations located in Puno and Cabanillas (see Fig. 1). Table 2 shows the

average pre- cipitation and temperature for both stations.
[...]

Table 2 Mean precipitation and temperature for 1996–2006 at Puno and Cabanillas stations (Data source: Servicio Nacional de Meteorologı´ae Hidrologı´a del Peru´, SENAMHI)

	Mean values for 1996–2006												
	Jan	Feb	Mar	Apr	May	Jun	Jul	Aug	Sept	Oct	Nov	Dec	Total
Precipitation (mm)													
Puno	201	161	138	60	7	3	4	14	27	51	48	88	801
Cabanillas	166	165	112	56	6	1	3	11	19	54	55	91	738
Mean temperature (□C)													
Puno	10.8	10.7	10.6	9.7	8.1	6.8	6.8	7.9	9.3	10.4	11.0	11.5	9.5
Cabanillas	10.6	10.5	10.5	9.8	8.6	7.3	6.9	8.1	9.6	10.6	11.1	11.3	9.6
Minimum temperature (□C)													
Puno	5.7	5.8	5.4	3.8	0.8	-0.9	-1.1	0.4	1.9	3.6	4.3	5.4	2.9
Cabanillas	5.3	5.5	5.2	3.7	1.1	-0.8	-1.5	0.3	2.1	3.7	4.2	5.1	2.8

Sampling strategies	
Sample sizes	
Data analysis	The cluster analysis was performed using a sequence of a common hierarchical and exchange algorithm, i.e., hclust and kmeans, using the statistics package R (MacQueen 1967; RDCT 2009). Based on stochastic initialisation, we calculated the reproducibility of partitions for a pre-given number of clusters to determine whether the algorithm detects stable or unstable (inappropriate) partitions. The share of households that were categorised in the same cluster in two partitions is expressed as ‘‘consistency measure’’. The higher this measure, the more reliable the cluster results. We calculated the consistency measure as the average of 200 pairwise comparisons of partitions with a given number of clusters. Ultimately, the consistency measure enables us to identify the optimal number of clusters to be analysed. Further methodological details are outlined in a previous application of the cluster approach to dryland vulnerability on a global scale (Sietz et al. 2011).
Candidate-	DIRECT OPERATIONALIZATION

level Analysis	

Livelihood trajectories and resilience and vulnerability

Constructs

- resilience and vulnerability of rural livelihoods

Constructs in report of frameworks	Represented in list by	Coded text indicating relationship
dynamic natural resource base	resilience and vulnerability of rural livelihoods	
factors influencing resilience and vulnerability	resilience and vulnerability of rural livelihoods	
livelihood trajectories	resilience and vulnerability of rural livelihoods	
resilience and vulnerability of rural livelihoods	resilience and vulnerability of rural livelihoods	Directly represented

<table>
<tr><td colspan="4">Operationalization of constructs</td></tr>
<tr><td colspan="4">Construct operationalized: resilience and vulnerability of rural livelihoods</td></tr>
<tr><td colspan="4">Source article(s):</td></tr>
<tr><td colspan="2">Selected by:</td><td colspan="2">default</td></tr>
<tr><td colspan="2" rowspan="2">Sub-constructs</td><td>Intermediate constructs</td><td></td></tr>
<tr><td>Directly operationalized constructs</td><td>dynamic natural resource base; factors influencing resilience and vulnerability; livelihood trajectories</td></tr>
<tr><td colspan="2">Conceptual framework</td><td colspan="2"></td></tr>
<tr><td colspan="2">Operationalization of sub-constructs</td><td colspan="2"></td></tr>
<tr><td rowspan="5">91.</td><td rowspan="5">dynamic natural resource base</td><td>Data collection</td><td>Repeated vegetation and wild animal surveys were conducted before and after rains, and time-series sets of Landsat images and wild animal aerial count data records were collected from the Department of Surveys and Mapping and the Department of Wildlife and National Parks. Soil and climate data were collected from the Department of Surveys and Mapping and the Department of Meteorological Services, respectively (see Sallu [2007] for a more detailed outline of the methodology and data). Environmental change data were then analyzed in conjunction with livelihood trajectory results in order to elucidate the key dynamics of relationships between livelihoods and the natural resource base.</td></tr>
<tr><td>Operational questions</td><td>2ndary data</td></tr>
<tr><td>Sampling strategies</td><td>2ndary data</td></tr>
<tr><td>Sample sizes</td><td>2ndary data</td></tr>
<tr><td>Data analysis</td><td>Quantitative data sets were analyzed using multivariate statistics. Livelihood and environmental data were classified using cluster analysis, and correlations were tested using principal components</td></tr>
</table>

			analysis. Landsat images were classified using ERDAS Imagine V.9 software and landscape-level changes were detected from raster attribute comparison (see Sallu [2007] for a more detailed outline of data analysis procedures).
92.	factors influencing resilience and vulnerability	Data collection	Not Transparent
		Operational questions	
		Sampling strategies	
		Sample sizes	
		Data analysis	
93.	livelihood trajectories	Data collection	Not Transparent
		Operational questions	
		Sampling strategies	
		Sample sizes	
		Data analysis	
Candidate-level Analysis		Data analysis was conducted throughout the period of information gathering. Initially, this was at a descriptive level in order to note any trends in the data, but it progressed to a more detailed level as both qualitative and quantitative social and environmental information was drawn together. Qualitative data were coded through processes of indexing the data under emerging themes. This permitted the identification of the factors that played an important role in the construction of livelihood strategies. Consistent triangulation of the results highlighted any contradictions and similarities in the different data sources. Where contradictions were found, further iterative reflection took place in the form of focus groups in order to ascertain why and how the conflicts in information may have occurred. This became a circular process that led to inductive interpretation and explanation as the ecological information was gradually juxtaposed within the emergent socioeconomic context.<br>Quantitative data sets were analyzed using multivariate statistics. Livelihood and environmental data were classified using cluster analysis, and correlations were tested using principal components analysis. Landsat images were classified using ERDAS Imagine V.9 software and landscape-level changes were detected from raster attribute comparison (see Sallu [2007] for a more detailed outline of data analysis procedures). Based on this analysis, we aimed to identify contemporary strategies and the nature of trajectories to which they led. In doing this, we also identified the key changes to the vulnerability context and the combination of factors that have led to more resilient or vulnerable livelihood outcomes.	

Determinants of Resilience

Constructs:

- Vulnerability IPCC
- Household level resilience
- Determinants of resilience

Constructs in report of frameworks	Represented in list by	Coded text indicating relationship
Vulnerability IPCC	Vulnerability IPCC	directly represented
Determinants of Resilience	Determinants of Resilience	directly represented
Household level resilience	Household level resilience	directly represented
Exposure	Vulnerability IPCC	In this regard, vulnerability is a function of the character, magnitude, and rate of climate variation to which a system is exposed, its sensitivity, and its adaptive capacity [4].
Adaptive capacity (A);	Vulnerability IPCC	In this regard, vulnerability is a function of the character, magnitude, and rate of climate variation to which a system is exposed, its sensitivity, and its adaptive capacity [4].

**Operationalization of constructs**			
**Construct operationalized:** Vulnerability IPCC			
**Source article(s):**			
**Selected by:**		expert selection [justification]	
Sub-constructs		Intermediate constructs	
		Directly operationalized constructs	
Conceptual framework			
Operationalization of sub-constructs			
94.	•	Data collection	
		Operational questions	
		Sampling strategies	
		Sample sizes	
		Data analysis	
95.	•	Data collection	
		Operational questions	
		Sampling strategies	
		Sample sizes	
		Data analysis	

96.	•	Data collection	
		Operational questions	
		Sampling strategies	
		Sample sizes	
		Data analysis	
97.	•	Data collection	
		Operational questions	
		Sampling strategies	
		Sample sizes	
		Data analysis	
Candidate-level Analysis			

**Operationalization of constructs**			
**Construct operationalized:** Household level resilience			
**Source article(s):** Tesso et al (2012)			
**Selected by:**		default	
Sub-constructs		Intermediate constructs	
		Directly operationalized constructs	
Conceptual framework		DIRECT OPERATIONALIZATION	
Operationalization of sub-constructs			
98.	•	Data collection	The data for the research was obtained from a survey of 452 farm households in three districts of the Zone in 2011/2012.<br>[...]<br>A structured ques- tionnaire was used to interview the farmers.<br>[...]<br>In addition, secondary data relevant for this analysis was obtained from the National Meteorological Service Agency (NMSA), Central Statistical Authority (CSA), and Zonal and district agricultural offices.<br>In order to understand the research questions at community level, qualitative data were collected through focused group discussion using checklist prepared for the purpose.
		Operational questions	Data collected from the farmers include household characteristics, landholding, crops and livestock production, disaster occurrence, perception level (on precipitation, temperature, soil moisture, air moisture and wind direction), adaptation strategies pursued, different coping strategies pursued, level of resilience, and other relevant informa- tion.<br>[...]<br>Table 1. Social, economic and environmental vulnerability indicators for the study area.<br>**I. Social Vulnerability Variables**<br>Sex: Female headed

Education: illiterate and less than grade 2<br>Marital status: Single (including divorce and widow)<br>No. of relatives: relative to less than 5 households<br>No. institutions: Participation in less than 2.35 institutions<br>Dependency: High dependency of 4 person and more<br>Farm to farm ext: No access to farmer to farmer extension<br>Year Ag. Experience: Lack of farm experience if < 3 years<br>Access to indigenous early warning information: Having no access
**II. Economic Vulnerability Variables**
Livestock ownership: Own less than 2 tropical livestock unit<br>Access to information: Having no access to<br>Ownership of perennial crops: no area under perennial crops<br>Land size: own less than 0.5ha of land<br>Land fragmentation: own only one plots<br>Non-farm income: Have no non-farm income<br>Soil and water conservation structures: More than 50% is not conserved<br>Income level: Having less than minimum requirement<br>Consumption expenditure: Spending less than minimum requirement<br>Crop diversity: less than 50% of the 8 major crops grown in the area<br>Land under irrigation: no access to irrigation at all<br>Land under improved seed: area not covered with improved seed (average of high yielding, drought tolerant, early maturing)<br>Land under commercial fertilizer: Having no access to fertilizer at all<br>Cash reserve: Having no cash saving at all<br>Food reserve: Having no food reserve for next year<br>Credit: Having no access to credit at all
**III. Environmental Vulnerability Variables (Measures of Sensitivity and Exposure)**
Land topography: Slope greater than 15% and 0% slope<br>Fertility: Poor fertility and cannot produce without heavy fertilizer use<br>Vegetation cover: Bare land<br>Frequency of hazards: People facing more than two natural hazards in a year<br>Rainfall: Receiving below average<br>Temperature: Experiencing above average<br>Change in wind direction: Encountering change in wind direction than usual

		Sampling strategies	The specific study sites within the dis- tricts were selected based on a multi stage random sam- pling procedure. Consequently, 19 Kebeles were selected from which the sample households were selected ran- domly proportional to population size.
		Sample sizes	452 farm households in three districts of the Zone
		Data analysis	In this analysis, the level of resilience was classified into three categories: 1) households that were fast in bouncing back; which means households that have gone back to their normal agricultural operation in the following production season; 2) moderate in bouncing back; which means households which took one to two agricultural seasons to get back to normal operation as before the event; and 3) slow in bouncing back; which means households which were unable to bounce back within one to two agricultural seasons to their normal livelihood activities. In this research, a farmer is said to have fully bounced back, when it begins its lively- hood operation as time before the shock. The speed of bouncing back was measured by number of agricul- tural seasons taken to bounce back to their livelihood without external intervention by government or non- governmental organization.<br>[...]<br>Table 3 presents the statistical measure of the different variables of resilience in the study area. From the statistical analysis result, the time taken to<br>bounce back after climate change induced shocks ranges from 1 agricultural year to more than 5 years<br>[...]
Candidate-level Analysis		DIRECT OPERATIONALIZATION	

Table 3. Statistical values of factors of resilience to climate change induced shocks.

**Variables**	**Mean**	**Maximum**	**Minimum**	**St Deviation**
Time taken to bounce back (Agr. seasons)	3	4	1	1.3898

Source: Own computation from household survey of 2011/2012.

**Operationalization of constructs**		
**Construct operationalized:** Determinants of resilience		
**Source article(s):** Tesso et al (2012)		
**Selected by:**	default	
Sub-constructs	Intermediate constructs	
	Directly operationalized	

		constructs	
Conceptual framework		DIRECT OPERATIONALIZATION	
Operationalization of sub-constructs			
99.	•	Data collection	The data for the research was obtained from a survey of 452 farm households in three districts of the Zone in 2011/2012.<br>[...]<br>A structured ques- tionnaire was used to interview the farmers.<br>[...]<br>In addition, secondary data relevant for this analysis was obtained from the National Meteorological Service Agency (NMSA), Central Statistical Authority (CSA), and Zonal and district agricultural offices.<br>In order to understand the research questions at community level, qualitative data were collected through focused group discussion using checklist prepared for the purpose.
		Operational questions	Data col- lected from the farmers include household character- istics, landholding, crops and livestock production, dis- aster occurrence, perception level (on precipitation, tem- perature, soil moisture, air moisture and wind direction), adaptation strategies pursued, different coping strategies pursued, level of resilience, and other relevant informa- tion.<br>[...]

Table 1. Social, economic and environmental vulnerability indicators for the study area.
**I. Social Vulnerability Variables**
Sex: Female headed<br>Education: illiterate and less than grade 2<br>Marital status: Single (including divorce and widow)<br>No. of relatives: relative to less than 5 households<br>No. institutions: Participation in less than 2.35 institutions<br>Dependency: High dependency of 4 person and more<br>Farm to farm ext: No access to farmer to farmer extension<br>Year Ag. Experience: Lack of farm experience if < 3 years<br>Access to indigenous early warning information: Having no access
**II. Economic Vulnerability Variables**
Livestock ownership: Own less than 2 tropical livestock unit<br>Access to information: Having no access to<br>Ownership of perennial crops: no area under perennial crops<br>Land size: own less than 0.5ha of land<br>Land fragmentation: own only one plots<br>Non-farm income: Have no non-farm income<br>Soil and water conservation structures: More than 50% is

			not conserved<br>Income level: Having less than minimum requirement<br>Consumption expenditure: Spending less than minimum requirement<br>Crop diversity: less than 50% of the 8 major crops grown in the area<br>Land under irrigation: no access to irrigation at all<br>Land under improved seed: area not covered with improved seed (average of high yielding, drought tolerant, early maturing)<br>Land under commercial fertilizer: Having no access to fertilizer at all<br>Cash reserve: Having no cash saving at all<br>Food reserve: Having no food reserve for next year<br>Credit: Having no access to credit at all<br>**III. Environmental Vulnerability Variables (Measures of Sensitivity and Exposure)**<br>Land topography: Slope greater than 15% and 0% slope<br>Fertility: Poor fertility and cannot produce without heavy fertilizer use<br>Vegetation cover: Bare land<br>Frequency of hazards: People facing more than two natural hazards in a year<br>Rainfall: Receiving below average<br>Temperature: Experiencing above average<br>Change in wind direction: Encountering change in wind direction than usual
		Sampling strategies	The specific study sites within the dis- tricts were selected based on a multi stage random sam- pling procedure. Consequently, 19 Kebeles were selected from which the sample households were selected ran- domly proportional to population size.
		Sample sizes	452 farm households in three districts of the Zone
		Data analysis	Ordered probit regression model was used to iden-<br>tify and analyze the determinants of households' re- silience to climate change induced shocks.<br>[...]<br>comparison was done based on certain defined characteristics. Thus, resilience in this measurment involved ordered out- come. This is with the basic hypothesis that a given natural shock will have differencial impact on house- holds' resilience.<br>$Y_j^* = X^1{}_j B + U_{1j}$<br>$Y = 0$ if $Y^* \leq 0$<br>$Y = 1$ if $0 < Y^* \leq 1$<br>$Y = 2$ if $1 < Y^* \leq 2$<br>Y* is level of resilience and involves ordered outcome, that is Y = 0 was given to households taking more than two years to bounce back, Y = 1 was given households taking greater than

			one year and less than or equals to two years; and Y = 2, was given to households taking less than or equals to one year. The Xij are the explanatory variables determining the time taken to bounce back. The independent variables included in the model were avail- ability of food stock(dummy), income diversification (number of enterprises), number of plots, number of de- pendent family members, age of household head (years), access to credit (dummy), social capital (number of in- stitutional involvement), area under perennial crops (ha), preparedness (dummy), propensity to invest on natural resources (percentage of area under conservation), pro- pensity to save (percentage of saving), access to irriga- tion (ha), geographic locations (dummy), etc. βs are pa- rameters estimated and Uij is the disturbance term.
Candidate-level Analysis		DIRECT OPERATIONALIZATION	

Appendix O: Results – Framework summaries, constructs, and operationalizations

Asset vulnerability

**Framework summary**
**Name of framework:** Asset vulnerability
**Description of framework:**<br>This framework conveives of household vulnerbility to climate change in terms of the management control that can be exercised over a series of assets. These assets include labour, human capital, non-labour productive assets, household relations, and social capital. A vulnerability index is created through a framework of weighted indicators representing each type of asset.
**Key constructs and definitions:**<br><u>Household vulnerability to climate change:</u> NOT DEFINED<br><u>Asset vulnerability:</u> Using Moser's (1998) asset vulnerability framework as guidance, we selected a range of variables to create an index of household vulnerability from GLSS 4. Each variable captures an aspect of vulnerability. (Dasgupta & Bashieri 2012)<br><br><u>Future exposure</u>: Future exposure also includes estimating the future state of the socioeconomic conditions, given that exposure is a property of the system relative to risk. (Ford & Smit 2004)<br><u>Communities at risk of climate shocks:</u> NOT DEFINED<br><u>Welfare of rural households:</u> NOT DEFINED<br><u>Prepared for adverse consequences:</u> NOT DEFINED
Ideal type model:<br>Not enough defined constructs to generate model.
**Articles using framework:** (Dasgupta & Bashieri 2012)
**Operationalization of key constructs:**

**Operationalization of constructs**		
**Construct operationalized:** Household vulnerability to climate change		
**Source article(s):** Dasgupta & bashieri		
**Selected by:**	default	
Sub-constructs	Intermediate constructs	Asset vulnerability
	Directly operationalized constructs	Labour; human capital; non-labour productive assets; social capital
Conceptual framework	Using the GLSS 4, we applied the asset vulnerability framework developed by Moser (1996, 1998, 2007). We constructed an index of vulnerability to climate change, at the household level.<br>[...]<br>The first asset Moser identified is labour.<br>[...]<br>The second asset Moser (1998) identified is human capital.<br>[...]<br>Non-labour productive assets are the third type.<br>[...]<br>Moser (1998) identified household relations<br>[...]<br>Social capital isMoser'sfifth asset	
Operationalization of		

<table>
<tr><td colspan="2">sub-constructs</td><td colspan="2"></td></tr>
<tr><td rowspan="5">100</td><td rowspan="5">Labour</td><td>Data collection</td><td>NOT valid/feasible</td></tr>
<tr><td>Operational questions</td><td></td></tr>
<tr><td>Sampling strategies</td><td></td></tr>
<tr><td>Sample sizes</td><td></td></tr>
<tr><td>Data analysis</td><td></td></tr>
<tr><td rowspan="7">101</td><td rowspan="7">human capital</td><td>Data collection</td><td>NOT valid/feasible</td></tr>
<tr><td>Operational questions</td><td></td></tr>
<tr><td>Sampling strategies</td><td></td></tr>
<tr><td>Sample sizes</td><td></td></tr>
<tr><td>Data analysis</td><td></td></tr>
<tr><td>Sample sizes</td><td></td></tr>
<tr><td>Data analysis</td><td></td></tr>
<tr><td rowspan="4">102</td><td rowspan="4">non-labour productive assets</td><td>Data collection</td><td>We used data collected between April 1998 and March 1999 by the fourth round of the Ghana Living Standards Survey (GLSS 4), which was funded by theWorld Bank and the Republic of Ghana. The survey instruments were designed to monitor poverty and well- being in Ghana. The GLSS 4 contains information on the demographic characteristics of household members, their reported health status, education, employment, housing and income from wages, business activities and agricultural production and detailed records of consumption and expenditure data. The main data file contained household-level information and derived money-metric measures of poverty such as household income and expenditure (Coulombe and McKay, 2000).</td></tr>
<tr><td>Operational questions</td><td>In order to measure the different degrees of productive assets between households we used the total number of productive assets owned by the household as a proxy. Among reproducible capital assets the questionnaire included furniture, sewing machines, stoves, refrigerator-freezers, air conditioners, fans, radios, radio-cassette players, record players, three-in-one radio-cassette players, video equipment, washing machines, TVs, cameras, electric irons, bicycles, motorcycles, cars, houses, land, shares, boats, canoes and outboard motors. Each asset was weighted equally.
[...]
Table 2<table><tr><td>ASSETS</td><td>Variable</td></tr><tr><td>Productive assets</td><td>Number of productive asset (N¼3679)</td></tr></table></td></tr>
<tr><td>Sampling strategies</td><td>TheGLSS4 is a two-stage probability-proportional-to-size sample.</td></tr>
<tr><td>Sample sizes</td><td>The sample contains data for5998households, of which 3799 resided in rural areas, with 25 694 eligible individual household members. We excluded the Greater Accra area as it is semi-urban, leaving 3679 rural households. In addition to the household survey, the GLSS 4 team (supervisor and enumerator) administered a community questionnaire to community leaders of the rural</td></tr>
</table>

			enumeration areas that were surveyed. One questionnaire was administered to each of the 195 rural enumeration areas.
		Data analysis	Table 4 shows the weight assigned to each variable derived from the first principal<br>component. The data reduction that PCA performed on the data explained 25 per cent of the original variation of the data. The PCA factor loadings were examined, and the principal component tended to load<br>positively on variables which contributed to lower vulnerability such as better education and better health, and negatively on variables which contributed to higher household vulnerability such as higher percentage of household income from agriculture. Therefore, the score is a measure of 'strength' or preparedness. A high score indicates a non-vulnerable household, and a low score indicates a vulnerable household.
103	social capital	Data collection	NOT VALID/FEASIBLE
		Operational questions	
		Sampling strategies	
		Sample sizes	
		Data analysis	
Candidate-level Analysis		Table 4 shows the weight assigned to each variable derived from the first principal component. The data reduction that PCA performed on the data explained 25 per cent of the original variation of the data. The PCA factor loadings were examined, and the principal component tended to load<br>positively on variables which contributed to lower vulnerability such as better education and better health, and negatively on variables which contributed to higher household vulnerability such as higher percentage of household income from agriculture. Therefore, the score is a measure of 'strength' or preparedness. A high score indicates a non-vulnerable household, and a low score indicates a vulnerable household.	

**Operationalization of constructs**			
**Construct operationalized:** Future Exposure			
**Source article(s): NO TRANSPARENT (FORD & SMIT) OR VALID (DASGUPTA & BASHCIERI) OPERATIONALIZATION**			
**Selected by:**		default	
Sub-constructs		Intermediate constructs	
		Directly operationalized constructs	
Conceptual framework			
Operationalization of sub-constructs			
104	•	Data collection	
		Operational questions	
		Sampling strategies	
		Sample sizes	
		Data analysis	
105	•	Data collection	

		Operational questions	
		Sampling strategies	
		Sample sizes	
		Data analysis	
106	•	Data collection	
		Operational questions	
		Sampling strategies	
		Sample sizes	
		Data analysis	
107	•	Data collection	
		Operational questions	
		Sampling strategies	
		Sample sizes	
		Data analysis	
Candidate-level Analysis			

Information relating to further development of framework		
Constructs with no adequate operationalizations<br>NO ADEQUATE OPERATIONALIZATIONS:<br>- Communities at risk of climate shocks (no operationalizations)<br>- Welfare of rural households (no operationalizations)<br>- Prepared for adverse consequences (no operationalizations)<br>- Future Exposure (no adequate operationalization)<br>- Household vulnerability to climate change (partial operationalization)		
Constructs with more than one adequate operationalizations		
Construct	Preference rank	Summary of operationalization

Current and future vulnerability

**Framework summary**
**Name of framework:** Current and future vulnerability
**Description of framework:**<br>The main characteristics of this framework is its comparison of current and future states of vulnerability. Vulnerability is conceived as being composed of two principal elements: exposure to climatic changes, and adaptive capacity. Multiple data sources are used to generate an assessment of current exposure and current adaptive capacity. On the basis of this data, and on historical social and physical trends, projections are made as to likely future states of exposure and future states of adaptive capacity.
**Key constructs and definitions:**<br>Vulnerability to climate risks: The conceptual model of community vulnerability to climate change outlined here builds on the literature, conceptualizing vulnerability as a function of exposure of the community to climate-change effects and its adaptive capacity to deal with that exposure.<br>[...]<br>A research framework for empirically applying the model of vulnerability proposed above to Arctic communities is illustrated in Figure 3. The first stage assesses current vulnerability by documenting current exposures and current adaptive strategies. The second stage assesses future vulnerability by estimating directional changes in exposure and predicting future adaptive capacity on the basis of past behavior. **(Ford & Smit 2004)**<br>Current vulnerability: The assessment of current vulnerability requires analyzing and documenting communities' experiences with climatic risks (current exposure) and the adaptive options and resource management strategies employed to address these risks (current adaptive capacity). **(Ford & Smit 2004)**<br><br>Future vulnerability: Future vulnerability is assessed by analyzing how cli- mate change will alter the nature of the climate-related risks and whether the communities' coping strategies will have the capacity to deal with these risks. Assessing future exposure involves collaboration with the climate science community to estimate the likelihood of changes in climatic attributes identified by the community **(Ford & Smit 2004)**<br><br>Current adaptive capacity: Adaptive capacity refers to a community's potential or ability to address, plan for, or adapt to exposure (Smit and Pilifosova, 2003). Most communities can cope with normal climatic conditions and a range of deviations around norms. People have learned to modify their behaviour and their environment to manage and take advantage of their local climatic conditions (Jones and Boer, 2003). This ability to cope is referred to in the literature as the "coping range"; it reflects resource use options and risk management strategies to prepare for, avoid or moderate, and recover from exposure effects (Hewitt and Burton, 1971; Smit et al., 1999; Jones, 2001; Smit and Pilifosova, 2003). Adaptive capacity relates to communities' resilience, resistance, flexibility, and robustness (Smithers and Smit, 1997). It is influenced by economic wealth, social networks, infrastructure, social in- stitutions, social capital, experience with previous risk, the range of technological adaptation available, and equity of access to resources within the community, as well as by other stresses that contribute to the environment in which decisions are made (Adger and Kelly, 1999; Smit and Pilifosova, 2001; Smith et al., 2003). **(Ford & Smit 2004)**<br><br>Exposure: The nature and degree to which a system is exposed to significant climatic variations.<br>The exposure of a system to climate stimuli depends on the level of global cli- mate change and, due to the spatial heterogeneity of anthropogenic climate change, on the system's location **(Fussel & Klein 2006)**<br>Future exposure: Future exposure also includes estimating the future state of the socioeconomic conditions, given that exposure is a property of the system relative to risk. **(Ford & Smit 2004)**
**Ideal type model:** (uneven)

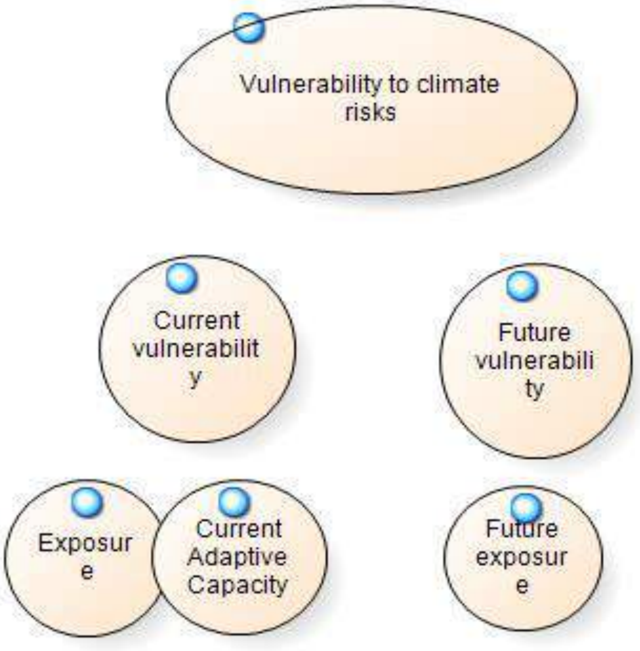

Articles using framework: (Ford & Smit 2004)

Operationalization of key constructs:

**Operationalization of constructs**			
**Construct operationalized:** Vulnerability to climate risks			
**Source article(s):** Ford & Smit (2004); Dasgupta & bashieri			
**Selected by:**		default	
Sub-constructs		Intermediate constructs	future vulnerability
		Directly operationalized constructs	Current vulnerability; Future exposure; future adaptive capacity
Conceptual framework		A research framework for empirically applying the model of vulnerability proposed above to Arctic commu- nities is illustrated in Figure 3. The first stage assesses current vulnerability by documenting current exposures and current adaptive strategies. The second stage assesses future vulnerability by estimating directional changes in exposure and predicting future adaptive capacity on the basis of past behavior.<br>[...]<br>FIG. 3. Analytical framework for vulnerability assessment.<br>Future Exposure<br>Future Vulnerability<br>Future Adaptive Capacity	
Operationalization of sub-constructs			
108	Current vulnerability	Data collection	NOT TRANSPARENT
		Operational questions	
		Sampling strategies	
		Sample sizes	
		Data analysis	
109	Future exposure	Data collection	NOT TRANSPARENT (Ford & Smit); Not Valid (Dasgupta &

			bashieri)
		Operational questions	
		Sampling strategies	
		Sample sizes	
		Data analysis	
110	future adaptive capacity	Data collection	NOT TRANSPARENT
		Operational questions	
		Sampling strategies	
		Sample sizes	
		Data analysis	
111	•	Data collection	
		Operational questions	
		Sampling strategies	
		Sample sizes	
		Data analysis	
Candidate-level Analysis			

**Information relating to further development of framework**		
Constructs with no adequate operationalizations:<br>NO ADEQUATE OPERATIONALIZATIONS		
Constructs with more than one adequate operationalizations		
Construct	Preference rank	Summary of operationalization

Determinants of Resilience

**Framework summary**
**Name of framework:** Determinants of Resilience
**Description of framework:**<br>The focus is on identifying determinants of resilience to climate-related shocks. Resilience is conceptualised temporally in terms of the time taken to make a recovery after being impacted by shocks. A vulnerability index (in this case based on the framework of the IPCC) is created to compute measures of vulnerability based on household survey data. Classifications of resilience are then created based on the time taken to return to pre-shock states, which are then analysed against the vulnerability data to identify determinants of resilient households.
**Key constructs and definitions:**<br>Vulnerability IPCC: The degree to which a system is susceptible to, or unable to cope with, adverse effects of climate change, including climate variability and extremes. Vulnerability is a function of the character, magnitude, and rate of climate variation to which a system is exposed, its sensitivity, and its adaptive capacity. **(Fussel & Klein 2006)**<br>Determinants of Resilience: important determinants for resilience at household level in North Shewa zone of Ethiopia. **(Tesso et al 2012)**<br>Household level resilience: According to DFID, resilience at community level is explained as the ability of countries, communities and households to manage change, by maintaining or transforming living standards in the face of shocks or stresses—such as earthquakes, drought or violent conflict—without compromising their long- term prospects [10]. Similarly, resilience is the ability of a social or ecological system to absorb disturbances while retaining the same basic structure and ways of functioning, the capacity for self-organization, and the capacity to adapt to stress and change. This is a measurement of community's capacity to absorb external shocks. In the aftermath of occurrence of climate change induced shocks, how do farmer bounce back to normal livelihood is about the resilience level of farming com- munity. A resilient community is able to respond to changes or stress in a positive way, and is able to maintain its core functions as a community despite those stresses [11]. **(Tesso et al 2012)**<br>Exposure: The nature and degree to which a system is exposed to significant climatic variations.<br>The exposure of a system to climate stimuli depends on the level of global cli- mate change and, due to the spatial heterogeneity of anthropogenic climate change, on the system's location **(Fussel & Klein 2006)**<br>Adaptive capacity: The ability of a system to adjust to climate change (in- cluding climate variability and extremes) to moderate potential damages, to take advantage of opportunities, or to cope with the consequences. **(Fussel & Klein 2006)**
**Ideal type model:** (uneven)<br>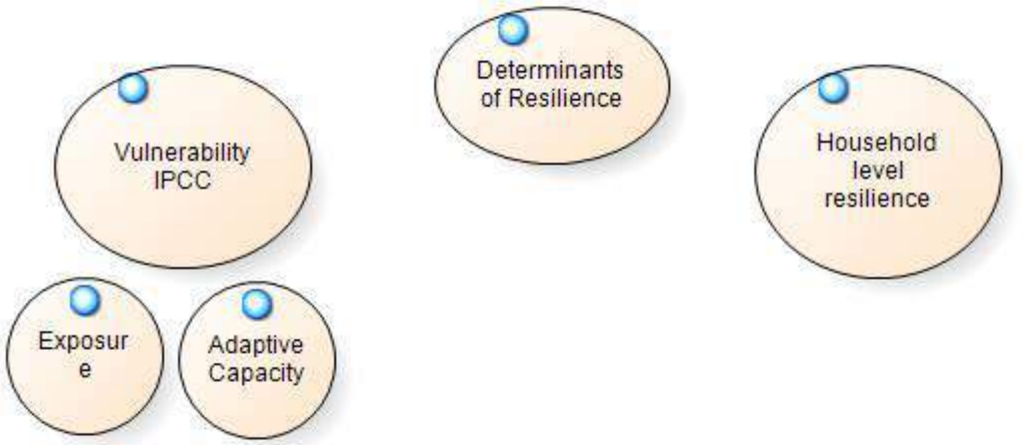
**Articles using framework:** (Tesso et al 2012)

Operationalization of key constructs:

**Operationalization of constructs**		
**Construct operationalized:** Vulnerability IPCC		
**Source article(s):**		
**Selected by:**	expert selection [justification]	
Sub-constructs	Intermediate constructs	
	Directly operationalized constructs	
Conceptual framework		
Operationalization of sub-constructs		
112 •	Data collection	
	Operational questions	
	Sampling strategies	
	Sample sizes	
	Data analysis	
113 •	Data collection	
	Operational questions	
	Sampling strategies	
	Sample sizes	
	Data analysis	
114 •	Data collection	
	Operational questions	
	Sampling strategies	
	Sample sizes	
	Data analysis	
115 •	Data collection	
	Operational questions	
	Sampling strategies	
	Sample sizes	
	Data analysis	
Candidate-level Analysis		

**Operationalization of constructs**		
**Construct operationalized:** Household level resilience		
**Source article(s):** Tesso et al (2012)		
**Selected by:**	default	
Sub-constructs	Intermediate constructs	
	Directly operationalized constructs	
Conceptual framework	DIRECT OPERATIONALIZATION	
Operationalization of		

<table>
<tr><td colspan="2">sub-constructs</td><td colspan="2"></td></tr>
<tr><td rowspan="2">116</td><td rowspan="2">•</td><td>Data collection</td><td>The data for the research was obtained from a survey of 452 farm households in three districts of the Zone in 2011/2012.
[...]
A structured ques- tionnaire was used to interview the farmers.
[...]
In addition, secondary data relevant for this analysis was obtained from the National Meteorological Service Agency (NMSA), Central Statistical Authority (CSA), and Zonal and district agricultural offices.
In order to understand the research questions at community level, qualitative data were collected through focused group discussion using checklist prepared for the purpose.</td></tr>
<tr><td>Operational questions</td><td>Data collected from the farmers include household characteristics, landholding, crops and livestock production, disaster occurrence, perception level (on precipitation, temperature, soil moisture, air moisture and wind direction), adaptation strategies pursued, different coping strategies pursued, level of resilience, and other relevant informa- tion.
[...]
<table>
<tr><td>Table 1. Social, economic and environmental vulnerability indicators for the study area.</td></tr>
<tr><td>I. Social Vulnerability Variables</td></tr>
<tr><td>Sex: Female headed
Education: illiterate and less than grade 2
Marital status: Single (including divorce and widow)
No. of relatives: relative to less than 5 households
No. institutions: Participation in less than 2.35 institutions
Dependency: High dependency of 4 person and more
Farm to farm ext: No access to farmer to farmer extension
Year Ag. Experience: Lack of farm experience if < 3 years
Access to indigenous early warning information: Having no access</td></tr>
<tr><td>II. Economic Vulnerability Variables</td></tr>
<tr><td>Livestock ownership: Own less than 2 tropical livestock unit
Access to information: Having no access to
Ownership of perennial crops: no area under perennial crops
Land size: own less than 0.5ha of land
Land fragmentation: own only one plots
Non-farm income: Have no non-farm income
Soil and water conservation structures: More than 50% is not conserved
Income level: Having less than minimum requirement
Consumption expenditure: Spending less than minimum</td></tr>
</table></td></tr>
</table>

			requirement<br>Crop diversity: less than 50% of the 8 major crops grown in the area<br>Land under irrigation: no access to irrigation at all<br>Land under improved seed: area not covered with improved seed (average of high yielding, drought tolerant, early maturing)<br>Land under commercial fertilizer: Having no access to fertilizer at all<br>Cash reserve: Having no cash saving at all<br>Food reserve: Having no food reserve for next year<br>Credit: Having no access to credit at all<br>**III. Environmental Vulnerability Variables (Measures of Sensitivity and Exposure)**<br>Land topography: Slope greater than 15% and 0% slope<br>Fertility: Poor fertility and cannot produce without heavy fertilizer use<br>Vegetation cover: Bare land<br>Frequency of hazards: People facing more than two natural hazards in a year<br>Rainfall: Receiving below average<br>Temperature: Experiencing above average<br>Change in wind direction: Encountering change in wind direction than usual
		Sampling strategies	The specific study sites within the dis- tricts were selected based on a multi stage random sam- pling procedure. Consequently, 19 Kebeles were selected from which the sample households were selected ran- domly proportional to population size.
		Sample sizes	452 farm households in three districts of the Zone
		Data analysis	In this analysis, the level of resilience was classified into three categories: 1) households that were fast in bouncing back; which means households that have gone back to their normal agricultural operation in the following production season; 2) moderate in bouncing back; which means households which took one to two agricultural seasons to get back to normal operation as before the event; and 3) slow in bouncing back; which means households which were unable to bounce back within one to two agricultural seasons to their normal livelihood activities. In this research, a farmer is said to have fully bounced back, when it begins its lively- hood operation as time before the shock. The speed of bouncing back was measured by number of agricul- tural seasons taken to bounce back to their livelihood without external intervention by government or non- governmental organization.<br>[...]<br>Table 3 presents the statistical measure of the different variables of resilience in the study area. From the statistical analysis result, the time taken to

			bounce back after climate change induced shocks ranges from 1 agricultural year to more than 5 years<br>[...]

Table 3. Statistical values of factors of resilience to climate change induced shocks.				
**Variables**	**Mean**	**Maximum**	**Minimum**	**St Deviation**
Time taken to bounce back (Agr. seasons)	3	4	1	1.3898
Source: Own computation from household survey of 2011/2012.				

Candidate-level Analysis	DIRECT OPERATIONALIZATION

**Operationalization of constructs**		
**Construct operationalized:** Determinants of resilience		
**Source article(s):** Tesso et al (2012)		
**Selected by:**	default	
Sub-constructs	Intermediate constructs	
	Directly operationalized constructs	
Conceptual framework	DIRECT OPERATIONALIZATION	
Operationalization of sub-constructs		
117 •	Data collection	The data for the research was obtained from a survey of 452 farm households in three districts of the Zone in 2011/2012.<br>[...]<br>A structured ques- tionnaire was used to interview the farmers.<br>[...]<br>In addition, secondary data relevant for this analysis was obtained from the National Meteorological Service Agency (NMSA), Central Statistical Authority (CSA), and Zonal and district agricultural offices.<br>In order to understand the research questions at community level, qualitative data were collected through focused group discussion using checklist prepared for the purpose.
	Operational questions	Data col- lected from the farmers include household character- istics, landholding, crops and livestock production, dis- aster occurrence, perception level (on precipitation, tem- perature, soil moisture, air moisture and wind direction), adaptation strategies pursued, different coping

			strategies pursued, level of resilience, and other relevant informa- tion.<br>[...]

Table 1. Social, economic and environmental vulnerability indicators for the study area.
**I. Social Vulnerability Variables**
Sex: Female headed<br>Education: illiterate and less than grade 2<br>Marital status: Single (including divorce and widow)<br>No. of relatives: relative to less than 5 households<br>No. institutions: Participation in less than 2.35 institutions<br>Dependency: High dependency of 4 person and more<br>Farm to farm ext: No access to farmer to farmer extension<br>Year Ag. Experience: Lack of farm experience if < 3 years<br>Access to indigenous early warning information: Having no access
**II. Economic Vulnerability Variables**
Livestock ownership: Own less than 2 tropical livestock unit<br>Access to information: Having no access to<br>Ownership of perennial crops: no area under perennial crops<br>Land size: own less than 0.5ha of land<br>Land fragmentation: own only one plots<br>Non-farm income: Have no non-farm income<br>Soil and water conservation structures: More than 50% is not conserved<br>Income level: Having less than minimum requirement<br>Consumption expenditure: Spending less than minimum requirement<br>Crop diversity: less than 50% of the 8 major crops grown in the area<br>Land under irrigation: no access to irrigation at all<br>Land under improved seed: area not covered with improved seed (average of high yielding, drought tolerant, early maturing)<br>Land under commercial fertilizer: Having no access to fertilizer at all<br>Cash reserve: Having no cash saving at all<br>Food reserve: Having no food reserve for next year<br>Credit: Having no access to credit at all
**III. Environmental Vulnerability Variables (Measures of Sensitivity and Exposure)**
Land topography: Slope greater than 15% and 0% slope<br>Fertility: Poor fertility and cannot produce without heavy fertilizer use<br>Vegetation cover: Bare land

			Frequency of hazards: People facing more than two natural hazards in a year<br>Rainfall: Receiving below average<br>Temperature: Experiencing above average<br>Change in wind direction: Encountering change in wind direction than usual
		Sampling strategies	The specific study sites within the dis- tricts were selected based on a multi stage random sam- pling procedure. Consequently, 19 Kebeles were selected from which the sample households were selected ran- domly proportional to population size.
		Sample sizes	452 farm households in three districts of the Zone
		Data analysis	Ordered probit regression model was used to iden-<br>tify and analyze the determinants of households' re- silience to climate change induced shocks.<br>[...]<br>comparison was done based on certain defined characteristics. Thus, resilience in this measurment involved ordered out- come. This is with the basic hypothesis that a given natural shock will have differencial impact on house- holds' resilience.<br>$Y_j^* = X^1{}_j B + U_{1j}$<br>$Y = 0$ if Y^* _< 0<br>$Y = 1$ if $0 < Y^*$ _< 1<br>$Y = 2$ if $1 < Y^*$ _< 2<br>Y* is level of resilience and involves ordered outcome, that is Y = 0 was given to households taking more than two years to bounce back, Y = 1 was given households taking greater than one year and less than or equals to two years; and Y = 2, was given to households taking less than or equals to one year. The Xij are the explanatory variables determining the time taken to bounce back. The independent variables included in the model were avail- ability of food stock(dummy), income diversification (number of enterprises), number of plots, number of de- pendent family members, age of household head (years), access to credit (dummy), social capital (number of in- stitutional involvement), area under perennial crops (ha), preparedness (dummy), propensity to invest on natural resources (percentage of area under conservation), pro- pensity to save (percentage of saving), access to irriga- tion (ha), geographic locations (dummy), etc. βs are pa- rameters estimated and Uij is the disturbance term.
Candidate-level Analysis		DIRECT OPERATIONALIZATION	

Information relating to further development of framework

Constructs with no adequate operationalizations<br>ALL ADEQUATE		
Constructs with more than one adequate operationalizations		
Construct	Preference rank	Summary of operationalization
Vulnerability IPCC		

Intensifying vulnerability to food insecurity

**Framework summary**
**Name of framework:** Intensifying vulnerability to food insecurity
**Description of framework:**<br>Vulnerability is situated in a recursive framework which captures a cyclical nature of intensification of vulnerability principally through the negative impacts that coping strategies can have on food security. Vulnerability is conceived principally in terms of food security, which in turn is conceived in terms of access to food and food productivity. When food security is negatively impacted through climatic and non-climatic drivers, vulnerable households and communities respond with particular coping strategies, which can have a recursive effect on future levels of food security.
**Key constructs and definitions:**<br><br><u>Household and community vulnerability</u>: In general terms, vulnerability and social resilience have been similarly defined as the ability of a system or community to resist or absorb adverse conditions.<br>[...]<br>Vulnerable communities, where people are unable to buffer themselves from hazards for a number of reasons, have a low ability to cope with short-term shocks (such as drought) and to mitigate chronic stressors, which in turn means that the negative impacts on livelihoods resulting from coping and survival strategies are very high.<br>**Misselhorn (2005)**<br><u>Livelihood strategies</u>: A livelihood maybe described as the capability, assets<br>and activites required for a means of living. People everywhere pursue a range of livelihood strategies in attempting to increase their income and asset base ('accumulation strategies'), spread or reduce risk (in- crease securitythrough 'adaptive strategies'), mitigate the impact of shocks ('coping strategies'), and at the extreme, ensure survival through 'survival strategies' (Devereux, 1999; Scoones, 2000). **Misselhorn (2005)**<br><u>Food insecurity</u>: Food insecurityin the communities described bythe<br>case studies maybe conceptualized as one element in an entrenched and escalating cycle of vulnerability (Fig. 3).<br>**Misselhorn (2005)**
**Ideal type model:** (insufficient defined constructs)<br>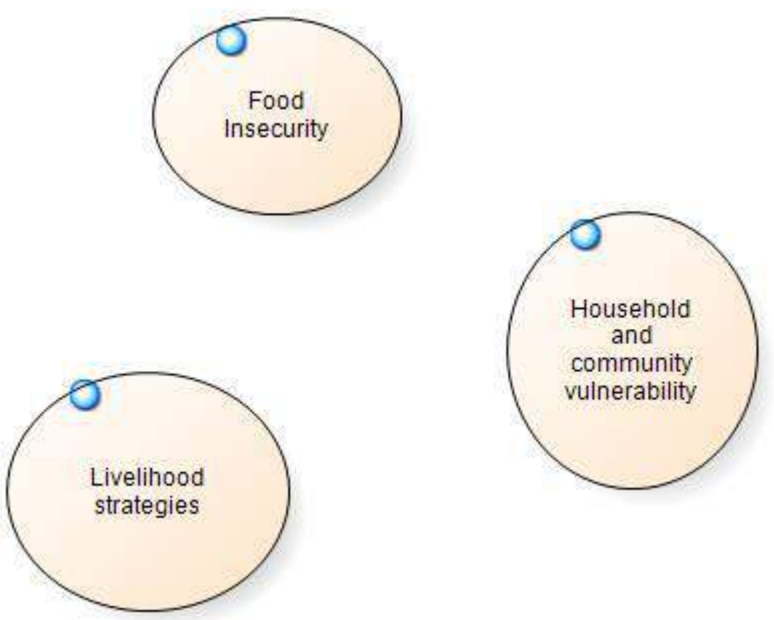

Articles using framework: Misselhorn (2005)

Operationalization of key constructs:

Operationalization of constructs

Construct operationalized: Livelihood strategies

Source article(s): Hahn et al

**Selected by:**	Default	
Sub-constructs	Intermediate constructs	
	Directly operationalized constructs	Households working elsewhere; agriculture dependent household; livelihood diversification
Conceptual framework	Livelihood<br>Percent of households with family member working in a different community<br>Percent of households dependent solely on agriculture as a source of income<br>Percentage of households where the head of the household reports that they have attended 0 years of school.<br>Percentage of households that have at least 1 orphan living in their home. Orphans are children<18 years old who have lost one or both parents.<br>Percentage of households that report at least 1 family member who works outside of the community for their primary work activity.<br>Percentage of households that report only agriculture as a source of income.	
Operationalization of sub-constructs		

118	Households working elsewhere	Data collection	Household survey
		Operational questions	Table 1 includes an explanation of how each sub-component was quantified, the survey question used to collect the data, the original source of the survey question, and potential sources of bias.<br>[...]
		Sampling strategies	We pilot tested the LVI and LVI–IPCC in the Moma and Mabote<br>Districts of Mozambique during 2007. These were selected by CARE-Mozambique as representative of coastal and inland communities, respectively, and the climate change

Table 1 Major components and sub-components comprising the Livelihood Vulnerability Index (LVI) developed for two districts of Mozambique.

Sub-components	*Explanation of sub-components*	*Survey question*
Percent of households with family member working in a different community	Percentage of households that report at least 1 family member who works outside of the community for their primary work activity.	How many people in your family go to a different community to work?

			issues con- fronting each.<br>[...]<br>Based on a sample size calculation (WHO, 2005) at the 95% confidence interval, □10% precision, 50% prevalence,1 and a design effect of 2 to account for cluster sampling, 200 households in each<br>district were surveyed.2 National 1997 census data that specified the total population in each village was used to select 20 villages in each district using the probability proportional to size method (WHO, 2005; UNICEF, 2008).<br>[...]<br>1 50% prevalence refers to the point prevalence of the indicators selected for the LVI. This is the default value for sample size calculations when the prevalence of the indicators is unknown.<br>2 Sample size formula: N = DEFF*[(Z2*p*q)/e2], where N = sample size, DEFF = 2; Z = 1.96 (95% CI), p = 0.5; q = 0.5; e = 0.10.
		Sample sizes	We pilot tested the LVI and LVI–IPCC in the Moma and Mabote<br>Districts<br>[...]<br>200 households in each<br>district were surveyed.2 National 1997 census data that specified the total population in each village was used to select 20 villages in each district
		Data analysis	The LVI uses a balanced weighted average approach (Sullivan<br>et al., 2002) where each sub-component contributes equally to the overall index even though each major component is comprised of a different number of sub-components. Becausewe intended to develop an assessment tool accessible to a diverse set of users in resource-poor settings, the LVI formula uses the simple approach of applying equal weights to all major components. This weighting scheme could be adjusted by future users as needed. Because each of the sub-components is measured on a different<br>scale, it was first necessary to standardize each as an index. The equation used for this conversion was adapted from that used in the Human Development Index to calculate the life expectancy index, which is the ratio of the difference of the actual life expectancy and a pre-selected minimum, and the range of pre- determined maximum and minimum life expectancy (UNDP, 2007):<br>$index_{sd} = (s_d - s_{min})/(s_{max} - s_{min})$<br>where sd is the original sub-component for district d, and smin and smax are the minimum and maximum values, respectively, for each sub-component determined using data from both districts.
11[illegible]	agriculture dependent	Data collection	Same as previous construct
		Operational questions	Table 1 includes an explanation of how each sub-component was quantified, the survey question used to

<table>
<tr><td></td><td>household</td><td></td><td>collect the data, the original source of the survey question, and potential sources of bias.
[...]

<table>
<tr><td colspan="3">Table 1 Major components and sub-components comprising the Livelihood Vulnerability Index (LVI) developed for two districts of Mozambique.</td></tr>
<tr><td>Sub-components</td><td>Explanation of sub-components</td><td>Survey question</td></tr>
<tr><td>Percent of households dependent solely on agriculture as a source of income</td><td>Percentage of households that report only agriculture as a source of income.</td><td>Do you or someone else in your household raise animals? Do you or someone else in your household grow crops? Do you or someone else in your household collect something from the bush, the forest, or lakes and rivers to sell?</td></tr>
</table></td></tr>
<tr><td></td><td></td><td>Sampling strategies</td><td>Same as previous construct</td></tr>
<tr><td></td><td></td><td>Sample sizes</td><td>Same as previous construct</td></tr>
<tr><td></td><td></td><td>Data analysis</td><td>Same as previous construct</td></tr>
<tr><td>12[illegible]</td><td>livelihood diversification</td><td>Data collection</td><td>Same as previous construct</td></tr>
<tr><td></td><td></td><td>Operational questions</td><td>Table 1 includes an explanation of how each sub-component was quantified, the survey question used to collect the data, the original source of the survey question, and potential sources of bias.
[...]

<table>
<tr><td colspan="3">Table 1 Major components and sub-components comprising the Livelihood Vulnerability Index (LVI) developed for two districts of Mozambique.</td></tr>
<tr><td>Sub-components</td><td>Explanation of sub-components</td><td>Survey question</td></tr>
<tr><td>Average Agricultural Livelihood Diversification Index (range: 0.20–1)a</td><td>The inverse of (the number of agricultural livelihood activities +1) reported by a household, e.g., A household that farms, raises animals, and collects natural</td><td>Do you or someone else in your household raise animals? Do you or someone else in your household grow crops? Do you or someone else in your household</td></tr>
</table></td></tr>
</table>

			resources will have a Livelihood Diversification Index = 1/(3 + 1) = 0.25.	collect something from the bush, the forest, or lakes and rivers to sell?
	Sampling strategies	Same as previous construct		
	Sample sizes	Same as previous construct		
	Data analysis	Same as previous construct		
	Data analysis			
Candidate-level Analysis	The LVI uses a balanced weighted average approach (Sullivan et al., 2002) where each sub-component contributes equally to the overall index even though each major component is comprised of a different number of sub-components. Becausewe intended to develop an assessment tool accessible to a diverse set of users in resource-poor settings, the LVI formula uses the simple approach of applying equal weights to all major components. This weighting scheme could be adjusted by future users as needed. Because each of the sub-components is measured on a different scale, it was first necessary to standardize each as an index. The equation used for this conversion was adapted from that used in the Human Development Index to calculate the life expectancy index, which is the ratio of the difference of the actual life expectancy and a pre-selected minimum, and the range of pre- determined maximum and minimum life expectancy (UNDP, 2007): $index_{sd} = (s_d - s_{min})/(s_{max} - s_{min})$ where sd is the original sub-component for district d, and smin and smax are the minimum and maximum values, respectively, for each sub-component determined using data from both districts.			

Information relating to further development of framework

<u>Constructs with no adequate operationalizations</u>

- Livelihood level issues (no operationalization)
- Access to sufficient food (no operationalization)
- Food insecurity (no operationalization)
- Household and community vulnerability (no operationalization)
- Direct drivers (no operationalization)

<u>Constructs with more than one adequate operationalizations</u>

Construct	Preference rank	Summary of operationalization

IPCC

Framework summary

Name of framework: IPCC

Description of framework:
This framework is guided by the definition and theory of the IPCC, which conceives of vulnerability to climate change as having three dimensions: Exposure to climate-induced shocks (a natural science phenomenon); the Sensitivity of the unit of analysis to such shocks (a social and natural science phenomenon); the adaptive capacity to deal with such shocks (a social science phenomenon). The framework often but not always creates a context-specific index of vulnerability from indicators of these three dimensions.

Key constructs and definitions:
Vulnerability (IPCC): The degree to which a system is susceptible to, or unable to cope with, adverse effects of climate change, including climate variability and extremes. Vulnerability is a function of the character, magnitude, and rate of climate variation to which a system is exposed, its sensitivity, and its adaptive capacity. **(Fussel & Klein 2006)**
Exposure: The nature and degree to which a system is exposed to significant climatic variations.
The exposure of a system to climate stimuli depends on the level of global cli- mate change and, due to the spatial heterogeneity of anthropogenic climate change, on the system's location **(Fussel & Klein 2006)**
Sensitivity: The degree to which a system is affected, either adversely or beneficially, by climate-related stimuli. [...] The effect may be direct [...]or indirect [...]
[...]
The sensitivity of a system denotes the (generally multi-factorial and dynamic) dose – response relationship between its exposure to climatic stimuli and the re- sulting impacts. **(Fussel & Klein 2006)**
Adaptive Capacity: The ability of a system to adjust to climate change (in- cluding climate variability and extremes) to moderate potential damages, to take advantage of opportunities, or to cope with the consequences. **(Fussel & Klein 2006)**

Ideal type model:

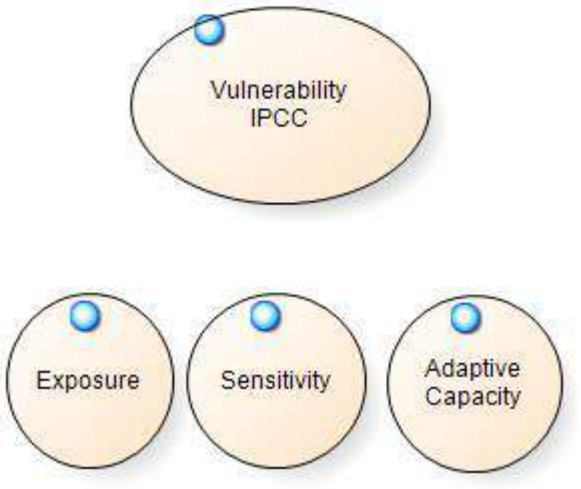

Articles using framework:
Antwi-Agyei et al (2013); Baca et al (2004); CARE (2009); Fussel & Klein (2006); Hahn et al (2009); Ionesco et al (2009); Jamir et al (2013); Luers et al (2013); Notenbaert et al (2013); Piya et al (2012).

Operationalization of key constructs:

Operationalization of constructs
**Construct operationalized:** Vulnerability IPCC

**Source article(s):**		
**Selected by:**	expert selection [justification]	
Sub-constructs	Intermediate constructs	
	Directly operationalized constructs	
Conceptual framework		
Operationalization of sub-constructs		
121 •	Data collection	
	Operational questions	
	Sampling strategies	
	Sample sizes	
	Data analysis	
122 •	Data collection	
	Operational questions	
	Sampling strategies	
	Sample sizes	
	Data analysis	
123 •	Data collection	
	Operational questions	
	Sampling strategies	
	Sample sizes	
	Data analysis	
124 •	Data collection	
	Operational questions	
	Sampling strategies	
	Sample sizes	
	Data analysis	
Candidate-level Analysis		

**Information relating to further development of framework**		
Constructs with no adequate operationalizations<br>FULLY AND ADEQUATELY OPERATIONALIZED		
Constructs with more than one adequate operationalizations		
Construct	Preference rank	Summary of operationalization

Livelihood trajectories and resilience and vulnerability

Framework summary

Name of framework: Livelihood trajectories and resilience and vulnerability

Description of framework:
On the basis of a mixed methods data collection methodology, the concept of 'livelihood trajectories' is explored among households over a period of (in this case) 30 years. With this long term approach, the framework seeks to generate narrative accounts of which livelihood strategies and trajectories lead to resilient and vulnerable states.

Key constructs and definitions:
Resilience and vulnerability of rural livelihoods: Fraser et al.'s (2010) vulnerability framework **Sallu et al (2010)**
Livelihood trajectories: Bagchi et al. (1998) use the term "livelihood trajectories" to describe and explain the direction and pattern of livelihoods of individuals or groups of people (e.g., households). A livelihood trajectory approach allows the examination of an individual household's "strategic behavior that is embedded in a historical repertoire, in social differentiation" (de Haan and Zoomers 2005), and in perceptions of risk. Such an approach is sensitive to life histories (an individual's own "story" of their changing livelihoods). **Sallu et al (2010)**
Dynamic natural resource base: NO DEFINITION
Factors influencing resilience and vulnerability: Through comparative research we provide a rich contextual narrative and use it to explore those factors that in isolation and combination push livelihoods along particular "trajectories" towards vulnerability or resilience. **Sallu et al (2010)**

Ideal type model: (incomplete – insufficient defined constructs)

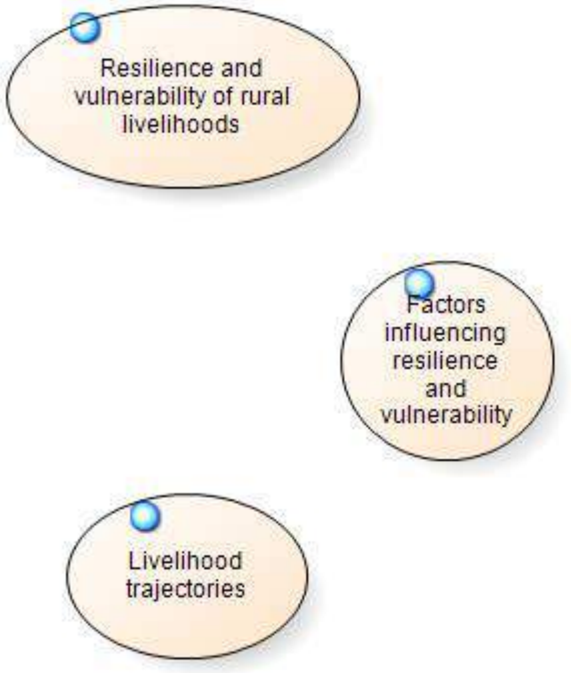

Articles using framework: Sallu et al (2010)

Operationalization of key constructs:

Operationalization of constructs
**Construct operationalized:** resilience and vulnerability of rural livelihoods
**Source article(s):**

**Selected by:**		default	
Sub-constructs		Intermediate constructs	
		Directly operationalized constructs	dynamic natural resource base; factors influencing resilience and vulnerability; livelihood trajectories
Conceptual framework			
Operationalization of sub-constructs			
125	dynamic natural resource base	Data collection	Repeated vegetation and wild animal surveys were conducted before and after rains, and time-series sets of Landsat images and wild animal aerial count data records were collected from the Department of Surveys and Mapping and the Department of Wildlife and National Parks. Soil and climate data were collected from the Department of Surveys and Mapping and the Department of Meteorological Services, respectively (see Sallu [2007] for a more detailed outline of the methodology and data). Environmental change data were then analyzed in conjunction with livelihood trajectory results in order to elucidate the key dynamics of relationships between livelihoods and the natural resource base.
		Operational questions	2ndary data
		Sampling strategies	2ndary data
		Sample sizes	2ndary data
		Data analysis	Quantitative data sets were analyzed using multivariate statistics. Livelihood and environmental data were classified using cluster analysis, and correlations were tested using principal components analysis. Landsat images were classified using ERDAS Imagine V.9 software and landscape-level changes were detected from raster attribute comparison (see Sallu [2007] for a more detailed outline of data analysis procedures).
126	factors influencing resilience and vulnerability	Data collection	Not Transparent
		Operational questions	
		Sampling strategies	
		Sample sizes	
		Data analysis	
127	livelihood trajectories	Data collection	Not Transparent
		Operational questions	
		Sampling strategies	
		Sample sizes	
		Data analysis	
Candidate-level Analysis		Data analysis was conducted throughout the period of information gathering. Initially, this was at a descriptive level in order to note any trends in the data, but it progressed to a more detailed level as both qualitative and quantitative social and environmental information was drawn together. Qualitative data were coded through processes of indexing the data under emerging themes. This permitted the identification of the factors that played an important role in the	

	construction of livelihood strategies. Consistent triangulation of the results highlighted any contradictions and similarities in the different data sources. Where contradictions were found, further iterative reflection took place in the form of focus groups in order to ascertain why and how the conflicts in information may have occurred. This became a circular process that led to inductive interpretation and explanation as the ecological information was gradually juxtaposed within the emergent socioeconomic context.<br>Quantitative data sets were analyzed using multivariate statistics. Livelihood and environmental data were classified using cluster analysis, and correlations were tested using principal components analysis. Landsat images were classified using ERDAS Imagine V.9 software and landscape-level changes were detected from raster attribute comparison (see Sallu [2007] for a more detailed outline of data analysis procedures). Based on this analysis, we aimed to identify contemporary strategies and the nature of trajectories to which they led. In doing this, we also identified the key changes to the vulnerability context and the combination of factors that have led to more resilient or vulnerable livelihood outcomes.

**Information relating to further development of framework**		
Constructs with no adequate operationalizations<br>- factors influencing resilience and vulnerability (no transparent operationalization)<br>- livelihood trajectories (no transparent operationalization)<br>•		
Constructs with more than one adequate operationalizations		
Construct	Preference rank	Summary of operationalization

Livelihood vulnerability index

Framework summary
**Name of framework:** Livelihood vulnerability index
**Description of framework:**<br>This framework consists of an index to measure levels of vulnerability. The index is composed of a highly developed set of household-level indicators chosen to represent seven dimensions of a particular conception of 'livelihoods'. These seven dimensions are: socio-demographic profile; livelihood strategies; social network; health; food; water; and natural disaster and climate change.
**Key constructs and definitions:**<br>Livelihood vulnerability (A): The LVI includes seven major components: Socio-Demographic Profile, Livelihood Strategies, Social Networks, Health, Food, Water, and Natural Disasters and Climate Variability **Hahn et al (2009)**<br>Livelihood strategies: Household working elsewhere; agriculture dependent households; livelihood diversification **Hahn et al (2009)**<br>Health: Proximity to health facility; 2 weeks illness; malaria-exposure-prevention **Hahn et al (2009)**<br>Socio-demographic profile: Dependency ratio; female headed households; uneducated headed households; households with orphans **Hahn et al (2009)**<br>Water: Sub-constructs: water conflict; natural water source; proximity to water source; inconsistent water supply; inverse water storage **Hahn et al (2009)**<br>Natural disaster and climate variability: Sub-constructs: flood, drought, cyclone events; no warning of disaster; injury or death from disaster; maximum temperature; minimum temperature; average percipitation **Hahn et al (2009)**
**Ideal type model:** (uneven)

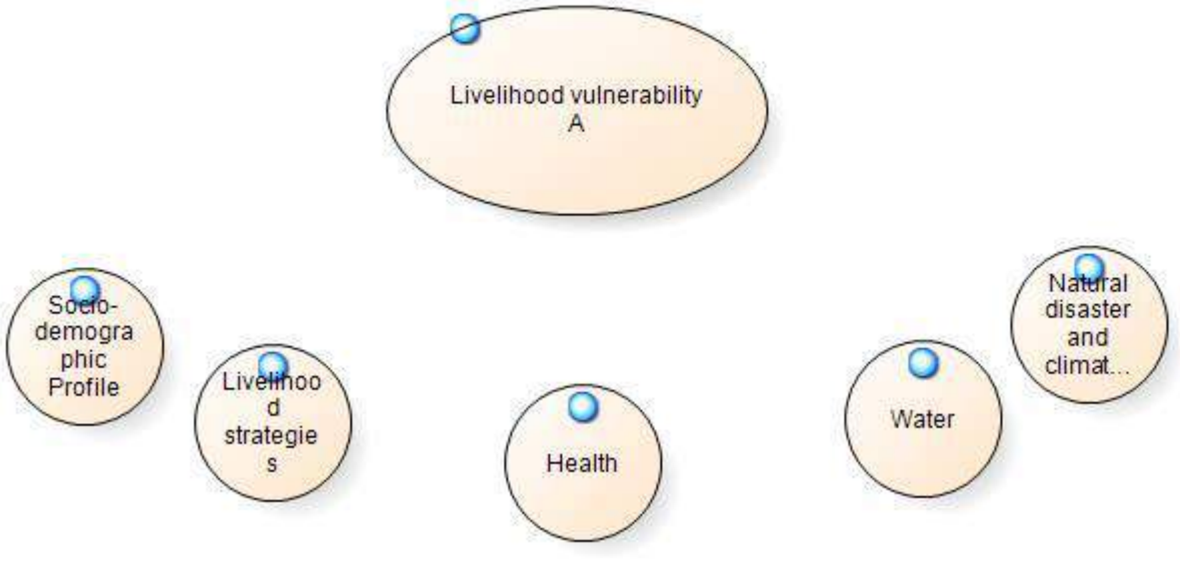

**Articles using framework:** Hahn et al (2009)
**Operationalization of key constructs:**

Operationalization of constructs	
**Construct operationalized:** Livelihood vulnerability (A)	
**Source article(s):** Hahn et al	
**Selected by:**	default

Sub-constructs	Intermediate constructs	Socio-demographic profile; livelihood strategies; social networks; health; food; water; natural disaster and climate change
	Directly operationalized constructs	Dependency ratio; percent of female headed households; households with orphans; uneducated headed households; Households working elsewhere; agriculture dependent household; livelihood diversification; Receive-give ration; borrow-lend ration; independent of local government; Family with chronic illness; proximity to health facility; 2 weeks illness; malaria exposure-prevention; Food from family farm; struggle for food; crop diversity; dont save crops; dont save seeds; Water conflict; natural water source; proximity to water source; inconsistent water supply; inverse water stored; Flood, drought, cyclone events; injury or death from disaster; no warning of disaster; maximum temperature; minimum temperature; average precipitation
Conceptual framework	The LVI includes seven major components: Socio-Demographic Profile, Livelihood Strategies, Social Networks, Health, Food, Water, and Natural Disasters and Climate Variability.<br>[...]<br>Socio-demographic profile<br>Explanation of sub-components<br>Ratio of the population under 15 and over 65 years of age to the population between 19 and 64 years of age.<br>Percent of female-headed households<br>Percent of households where head of household has not attended school<br>Percent of households with orphans<br>[...]<br>Livelihood<br>Percent of households with family member working in a different community<br>Percent of households dependent solely on agriculture as a source of income<br>Percentage of households where the head of the household reports that they have attended 0 years of school.<br>Percentage of households that have at least 1 orphan living in their home. Orphans are children<18 years old who have lost one or both parents.<br>Percentage of households that report at least 1 family member who works outside of the community for their primary work activity.<br>Percentage of households that report only agriculture as a source of income.<br>[...]<br>Social Networks<br>Average Receive:Give ratio (range: 0–15)<br>Average Borrow:Lend Money ratio (range: 0.5–2)<br>[...]<br>Health<br>Average time to health facility (minutes)<br>Percent of households with family member with chronic illness<br>Percent of households where a family member had to miss work or school in the last 2 weeks due to illness<br>Average Malaria Exposure*Prevention Index (range: 0–12)<br>[...]<br>Food<br>Percent of households dependent on family farm for food<br>Average number of months households struggle to find food (range: 0–12)	

<table>
<tr><td colspan="2"></td><td colspan="2">[...]
Water
Percent of households reporting water conflicts
Percent of households that utilize a natural water source
Average time to water source (minutes)
Percent of households that do not have a consistent water supply
Inverse of the average number of liters of water stored per household (range: >0–1)
[...]
Natural disasters and climate variability
Average number of flood, drought, and cyclone events in the past 6 years (range: 0–7)
Percent of households that did not receive a warning about the pending natural disasters
Percent of households with an injury or death as a result of the most severe natural disaster in the past 6 years
Mean standard deviation of the daily average maximum temperature by month
Mean standard deviation of the daily average minimum temperature by month
Mean standard deviation of average precipitation by month</td></tr>
<tr><td colspan="2">Operationalization of sub-constructs</td><td colspan="2"></td></tr>
<tr><td rowspan="3">128</td><td rowspan="3">Dependency ratio</td><td>Data collection</td><td>household surveys</td></tr>
<tr><td>Operational questions</td><td>Table 1 includes an explanation of how each sub-component was quantified, the survey question used to collect the data, the original source of the survey question, and potential sources of bias.
[...]
<table>
<tr><td colspan="3">Table 1 Major components and sub-components comprising the Livelihood Vulnerability Index (LVI) developed for two districts of Mozambique.</td></tr>
<tr><td>Sub-components</td><td>Explanation of sub-components</td><td>Survey question</td></tr>
<tr><td>Dependency ratio</td><td>Ratio of the population under 15 and over 65 years of age to the population between 19 and 64 years of age.</td><td>Could you please list the ages and sexes of every person who eats and sleeps in this house? If you had a visitor who ate and slept here for the last 3 days, please include them as well.</td></tr>
</table></td></tr>
<tr><td>Sampling strategies</td><td>We pilot tested the LVI and LVI–IPCC in the Moma and Mabote
Districts of Mozambique during 2007. These were selected by CARE-Mozambique as representative of coastal and inland communities, respectively, and the climate change issues con- fronting each.
[...]
Based on a sample size calculation (WHO, 2005) at the 95%</td></tr>
</table>

			confidence interval, 10% precision, 50% prevalence,1 and a design effect of 2 to account for cluster sampling, 200 households in each<br>district were surveyed.2 National 1997 census data that specified the total population in each village was used to select 20 villages in each district using the probability proportional to size method (WHO, 2005; UNICEF, 2008).<br>[...]<br>1 50% prevalence refers to the point prevalence of the indicators selected for the LVI. This is the default value for sample size calculations when the prevalence of the indicators is unknown.<br>2 Sample size formula: N = DEFF*[(Z2*p*q)/e2], where N = sample size, DEFF = 2; Z = 1.96 (95% CI), p = 0.5; q = 0.5; e = 0.10.
		Sample sizes	We pilot tested the LVI and LVI–IPCC in the Moma and Mabote<br>Districts<br>[...]<br>200 households in each<br>district were surveyed.2 National 1997 census data that specified the total population in each village was used to select 20 villages in each district
		Data analysis	The LVI uses a balanced weighted average approach (Sullivan<br>et al., 2002) where each sub-component contributes equally to the overall index even though each major component is comprised of a different number of sub-components. Becausewe intended to develop an assessment tool accessible to a diverse set of users in resource-poor settings, the LVI formula uses the simple approach of applying equal weights to all major components. This weighting scheme could be adjusted by future users as needed. Because each of the sub-components is measured on a different<br>scale, it was first necessary to standardize each as an index. The equation used for this conversion was adapted from that used in the Human Development Index to calculate the life expectancy index, which is the ratio of the difference of the actual life expectancy and a pre-selected minimum, and the range of pre- determined maximum and minimum life expectancy (UNDP, 2007):<br>$index_{sd} = (s_d – s_{min})/(s_{max} – s_{min})$<br>where sd is the original sub-component for district d, and smin and smax are the minimum and maximum values, respectively, for each sub-component determined using data from both districts.
129	percent of female headed households	Data collection	Same as previous construct
		Operational questions	Table 1 includes an explanation of how each sub-component was quantified, the survey question used to collect the data, the original source of the survey question, and potential sources of bias.<br>[...]

<table>
<tr><td></td><td></td><td></td><td>
<table>
<tr><td colspan="3">Table 1 Major components and sub-components comprising the Livelihood Vulnerability Index (LVI) developed for two districts of Mozambique.</td></tr>
<tr><td>Sub-components</td><td>Explanation of sub-components</td><td>Survey question</td></tr>
<tr><td>Percent of female-headed households</td><td>Percentage of households where the primary adult is female. If a male head is away from the home >6 months per year the female is counted as the head of the household.</td><td>Are you the head of the household?</td></tr>
</table>
</td></tr>
<tr><td></td><td></td><td>Sampling strategies</td><td>Same as previous construct</td></tr>
<tr><td></td><td></td><td>Sample sizes</td><td>Same as previous construct</td></tr>
<tr><td></td><td></td><td>Data analysis</td><td>Same as previous construct</td></tr>
<tr><td>130</td><td>households with orphans</td><td>Data collection</td><td>Same as previous construct</td></tr>
<tr><td></td><td></td><td>Operational questions</td><td>Table 1 includes an explanation of how each sub-component was quantified, the survey question used to collect the data, the original source of the survey question, and potential sources of bias.
[...]
<table>
<tr><td colspan="3">Table 1 Major components and sub-components comprising the Livelihood Vulnerability Index (LVI) developed for two districts of Mozambique.</td></tr>
<tr><td>Sub-components</td><td>Explanation of sub-components</td><td>Survey question</td></tr>
<tr><td>Percent of households with orphans</td><td>Percentage of households that have at least 1 orphan living in their home. Orphans are children<18 years old who have lost one or both parents.</td><td>Are there any children less than 18 years old from other families living in your house because one or both of their parents has died?</td></tr>
</table>
</td></tr>
<tr><td></td><td></td><td>Sampling strategies</td><td>Same as previous construct</td></tr>
<tr><td></td><td></td><td>Sample sizes</td><td>Same as previous construct</td></tr>
<tr><td></td><td></td><td>Data analysis</td><td>Same as previous construct</td></tr>
<tr><td>131</td><td>uneducated headed households</td><td>Data collection</td><td>NOT TRANSPARENT</td></tr>
<tr><td></td><td></td><td>Operational questions</td><td></td></tr>
<tr><td></td><td></td><td>Sampling strategies</td><td></td></tr>
<tr><td></td><td></td><td>Sample sizes</td><td></td></tr>
<tr><td></td><td></td><td>Data analysis</td><td></td></tr>
<tr><td>132</td><td>Households</td><td>Data collection</td><td>Same as for ‘dependency ratio’</td></tr>
</table>

<table>
<tr><td></td><td>working elsewhere</td><td>Operational questions</td><td>Table 1 includes an explanation of how each sub-component was quantified, the survey question used to collect the data, the original source of the survey question, and potential sources of bias.
[...]
<table><tr><td colspan="3">Table 1 Major components and sub-components comprising the Livelihood Vulnerability Index (LVI) developed for two districts of Mozambique.</td></tr><tr><td>Sub-components</td><td>Explanation of sub-components</td><td>Survey question</td></tr><tr><td>Percent of households with family member working in a different community</td><td>Percentage of households that report at least 1 family member who works outside of the community for their primary work activity.</td><td>How many people in your family go to a different community to work?</td></tr></table></td></tr>
<tr><td></td><td></td><td>Sampling strategies</td><td>Same as for ‘dependency ratio’</td></tr>
<tr><td></td><td></td><td>Sample sizes</td><td>Same as for ‘dependency ratio’</td></tr>
<tr><td></td><td></td><td>Data analysis</td><td>Same as for ‘dependency ratio’</td></tr>
<tr><td>13[illegible]</td><td>agriculture dependent household</td><td>Data collection</td><td>Same as previous construct</td></tr>
<tr><td></td><td></td><td>Operational questions</td><td>Table 1 includes an explanation of how each sub-component was quantified, the survey question used to collect the data, the original source of the survey question, and potential sources of bias.
[...]
<table><tr><td colspan="3">Table 1 Major components and sub-components comprising the Livelihood Vulnerability Index (LVI) developed for two districts of Mozambique.</td></tr><tr><td>Sub-components</td><td>Explanation of sub-components</td><td>Survey question</td></tr><tr><td>Percent of households dependent solely on agriculture as a source of income</td><td>Percentage of households that report only agriculture as a source of income.</td><td>Do you or someone else in your household raise animals? Do you or someone else in your household grow crops? Do you or someone else in your household collect something from the bush, the forest, or lakes and rivers to sell?</td></tr></table></td></tr>
</table>

<table>
<tr><td></td><td></td><td>Sampling strategies</td><td>Same as previous construct</td></tr>
<tr><td></td><td></td><td>Sample sizes</td><td>Same as previous construct</td></tr>
<tr><td></td><td></td><td>Data analysis</td><td>Same as previous construct</td></tr>
<tr><td>134</td><td>livelihood diversification</td><td>Data collection</td><td>Same as previous construct</td></tr>
<tr><td></td><td></td><td>Operational questions</td><td>Table 1 includes an explanation of how each sub-component was quantified, the survey question used to collect the data, the original source of the survey question, and potential sources of bias.
[...]
<table>
<tr><td colspan="3">Table 1 Major components and sub-components comprising the Livelihood Vulnerability Index (LVI) developed for two districts of Mozambique.</td></tr>
<tr><td>Sub-components</td><td>Explanation of sub-components</td><td>Survey question</td></tr>
<tr><td>Average Agricultural Livelihood Diversification Index (range: 0.20–1)a</td><td>The inverse of (the number of agricultural livelihood activities +1) reported by a household, e.g., A household that farms, raises animals, and collects natural resources will have a Livelihood Diversification Index = 1/(3 + 1) = 0.25.</td><td>Do you or someone else in your household raise animals? Do you or someone else in your household grow crops? Do you or someone else in your household collect something from the bush, the forest, or lakes and rivers to sell?</td></tr>
</table></td></tr>
<tr><td></td><td></td><td>Sampling strategies</td><td>Same as previous construct</td></tr>
<tr><td></td><td></td><td>Sample sizes</td><td>Same as previous construct</td></tr>
<tr><td></td><td></td><td>Data analysis</td><td>Same as previous construct</td></tr>
<tr><td>135</td><td>Receive-give ratio</td><td>Data collection</td><td>Same as previous construct</td></tr>
<tr><td></td><td></td><td>Operational questions</td><td>Table 1 includes an explanation of how each sub-component was quantified, the survey question used to collect the data, the original source of the survey question, and potential sources of bias.
[...]
<table>
<tr><td colspan="3">Table 1 Major components and sub-components comprising the Livelihood Vulnerability Index (LVI) developed for two districts of Mozambique.</td></tr>
<tr><td>Sub-components</td><td>Explanation of sub-components</td><td>Survey question</td></tr>
<tr><td>Average Receive:Give ratio (range: 0–15)</td><td>Ratio of (the number of types of help received by a household in the past</td><td>In the past month, did relatives or friends help you and your family:</td></tr>
</table></td></tr>
</table>

			<table><tr><td></td><td>month + 1) to (the number of types of help given by a household to someone else in the past month + 1).</td><td>(e.g., Get medical care or medicines, Sell animal products or other goods produced by family, Take care of children) In the past month, did you and your family help relatives or friends: (same choices as above)</td></tr></table>
		Sampling strategies	Same as previous construct
		Sample sizes	Same as previous construct
		Data analysis	Same as previous construct
136	borrow-lend ratio	Data collection	Same as previous construct
		Operational questions	Table 1 includes an explanation of how each sub-component was quantified, the survey question used to collect the data, the original source of the survey question, and potential sources of bias.<br>[...]<br><table><tr><td colspan="3">Table 1 Major components and sub-components comprising the Livelihood Vulnerability Index (LVI) developed for two districts of Mozambique.</td></tr><tr><td>Sub-components</td><td>Explanation of sub-components</td><td>Survey question</td></tr><tr><td>Average Borrow:Lend Money ratio (range: 0.5–2)</td><td>Ratio of a household borrowing money in the past month to a household lending money in the past month, e.g., If a household borrowed money but did not lend money, the ratio = 2:1 or 2 and if they lent money but did not borrow any, the ratio = 1:2 or 0.5.</td><td>Did you borrow any money from relatives or friends in the past month? Did you lend any money to relatives or friends in the past month?</td></tr></table>
		Sampling strategies	Same as previous construct
		Sample sizes	Same as previous construct
		Data analysis	Same as previous construct

<table>
<tr><td rowspan="5">137</td><td rowspan="5">independent of local government</td><td>Data collection</td><td>Same as previous construct</td></tr>
<tr><td>Operational questions</td><td>Table 1 includes an explanation of how each sub-component was quantified, the survey question used to collect the data, the original source of the survey question, and potential sources of bias.
[...]
<table>
<tr><td colspan="3">Table 1 Major components and sub-components comprising the Livelihood Vulnerability Index (LVI) developed for two districts of Mozambique.</td></tr>
<tr><td>Sub-components</td><td>Explanation of sub-components</td><td>Survey question</td></tr>
<tr><td>Percent of households that have not gone to their local government for assistance in the past 12 months</td><td>Percentage of households that reported that they have not asked their local government for any assistance in the past 12 months.</td><td>In the past 12 months, have you or someone in your family gone to your community leader for help?</td></tr>
</table></td></tr>
<tr><td>Sampling strategies</td><td>Same as previous construct</td></tr>
<tr><td>Sample sizes</td><td>Same as previous construct</td></tr>
<tr><td>Data analysis</td><td>Same as previous construct</td></tr>
<tr><td rowspan="5">138</td><td rowspan="5">Family with chronic illness</td><td>Data collection</td><td>Same as previous construct</td></tr>
<tr><td>Operational questions</td><td>Table 1 includes an explanation of how each sub-component was quantified, the survey question used to collect the data, the original source of the survey question, and potential sources of bias.
[...]
<table>
<tr><td colspan="3">Table 1 Major components and sub-components comprising the Livelihood Vulnerability Index (LVI) developed for two districts of Mozambique.</td></tr>
<tr><td>Sub-components</td><td>Explanation of sub-components</td><td>Survey question</td></tr>
<tr><td>Percent of households with family member with chronic illness</td><td>Percentage of households that report at least 1 family member with chronic illness. Chronic illness was defined subjectively by respondent.</td><td>Is anybody in your family chronically ill (they get sick very often)?</td></tr>
</table></td></tr>
<tr><td>Sampling strategies</td><td>Same as previous construct</td></tr>
<tr><td>Sample sizes</td><td>Same as previous construct</td></tr>
<tr><td>Data analysis</td><td>Same as previous construct</td></tr>
<tr><td rowspan="2">139</td><td rowspan="2">proximity to health facility</td><td>Data collection</td><td>Same as previous construct</td></tr>
<tr><td>Operational questions</td><td>Table 1 includes an explanation of how each sub-component was quantified, the survey question used to collect the data,</td></tr>
</table>

<table>
<tr><td></td><td></td><td></td><td>the original source of the survey question, and potential sources of bias.
[...]
<table><tr><td colspan="3">Table 1 Major components and sub-components comprising the Livelihood Vulnerability Index (LVI) developed for two districts of Mozambique.</td></tr><tr><td>Sub-components</td><td>Explanation of sub-components</td><td>Survey question</td></tr><tr><td>Average time to health facility (minutes)</td><td>Average time it takes the households to get to the nearest health facility.</td><td>Howlong does it take you to get to a health facility?</td></tr></table></td></tr>
<tr><td></td><td></td><td>Sampling strategies</td><td>Same as previous construct</td></tr>
<tr><td></td><td></td><td>Sample sizes</td><td>Same as previous construct</td></tr>
<tr><td></td><td></td><td>Data analysis</td><td>Same as previous construct</td></tr>
<tr><td>140</td><td>2 weeks illness</td><td>Data collection</td><td>Same as previous construct</td></tr>
<tr><td></td><td></td><td>Operational questions</td><td>Table 1 includes an explanation of how each sub-component was quantified, the survey question used to collect the data, the original source of the survey question, and potential sources of bias.
[...]
<table><tr><td colspan="3">Table 1 Major components and sub-components comprising the Livelihood Vulnerability Index (LVI) developed for two districts of Mozambique.</td></tr><tr><td>Sub-components</td><td>Explanation of sub-components</td><td>Survey question</td></tr><tr><td>Percent of households where a family member had to miss work or school in the last 2 weeks due to illness</td><td>Percentage of households that report at least 1 family member who had to miss school of work due to illness in the last 2 weeks.</td><td>Has anyone in your family been so sick in the past 2 weeks that they had to miss work or school?</td></tr></table></td></tr>
<tr><td></td><td></td><td>Sampling strategies</td><td>Same as previous construct</td></tr>
<tr><td></td><td></td><td>Sample sizes</td><td>Same as previous construct</td></tr>
<tr><td></td><td></td><td>Data analysis</td><td>Same as previous construct</td></tr>
<tr><td>141</td><td>malaria exposure-prevention</td><td>Data collection</td><td>Same as previous construct</td></tr>
<tr><td></td><td></td><td>Operational questions</td><td>Table 1 includes an explanation of how each sub-component was quantified, the survey question used to collect the data, the original source of the survey question, and potential sources of bias.
[...]
<table><tr><td colspan="3">Table 1 Major components and sub-components comprising the Livelihood Vulnerability Index (LVI) developed for two districts of Mozambique.</td></tr><tr><td>Sub-components</td><td>Explanation of</td><td>Survey</td></tr></table></td></tr>
</table>

<table>
<tr><td></td><td></td><td></td><td><table>
<tr><td></td><td>sub-components</td><td>question</td></tr>
<tr><td>Average Malaria Exposure*Prevention Index (range: 0–12)</td><td>Months reported exposure to malaria*Owning at least one bednet indicator (have bednet = 0.5, no bednet = 1) (e.g., Respondent reported malaria is a problem January–March and they do not own a bednet = 3*1 = 3).</td><td>Which months of the year is malaria particularly bad? How many mosquito nets do you have?</td></tr>
</table></td></tr>
<tr><td></td><td></td><td>Sampling strategies</td><td>Same as previous construct</td></tr>
<tr><td></td><td></td><td>Sample sizes</td><td>Same as previous construct</td></tr>
<tr><td></td><td></td><td>Data analysis</td><td>Same as previous construct</td></tr>
<tr><td>142</td><td>Food from family farm</td><td>Data collection</td><td>Same as previous construct</td></tr>
<tr><td></td><td></td><td>Operational questions</td><td>Table 1 includes an explanation of how each sub-component was quantified, the survey question used to collect the data, the original source of the survey question, and potential sources of bias.
[...]

<table>
<tr><td colspan="3">Table 1 Major components and sub-components comprising the Livelihood Vulnerability Index (LVI) developed for two districts of Mozambique.</td></tr>
<tr><td>Sub-components</td><td>Explanation of sub-components</td><td>Survey question</td></tr>
<tr><td>Percent of households dependent on family farm for food</td><td>Percentage of households that get their food primarily from their personal farms.</td><td>Where does your family get most of its food?</td></tr>
</table></td></tr>
<tr><td></td><td></td><td>Sampling strategies</td><td>Same as previous construct</td></tr>
<tr><td></td><td></td><td>Sample sizes</td><td>Same as previous construct</td></tr>
<tr><td></td><td></td><td>Data analysis</td><td>Same as previous construct</td></tr>
<tr><td>143</td><td>struggle for food</td><td>Data collection</td><td>Same as previous construct</td></tr>
<tr><td></td><td></td><td>Operational questions</td><td>Table 1 includes an explanation of how each sub-component was quantified, the survey question used to collect the data, the original source of the survey question, and potential sources of bias.
[...]

<table>
<tr><td colspan="3">Table 1 Major components and sub-components comprising the Livelihood Vulnerability Index (LVI) developed for two districts of Mozambique.</td></tr>
<tr><td>Sub-components</td><td>Explanation of</td><td>Survey question</td></tr>
</table></td></tr>
</table>

<table>
<tr><td></td><td></td><td></td><td><table><tr><td></td><td>sub-components</td><td></td></tr><tr><td>Average number of months households struggle to find food (range: 0–12)</td><td>Average number of months households struggle to obtain food for their family.</td><td>Does your family have adequate food the whole year, or are there times during the year that your family does not have enough food? Howmanymonths a year does your family have trouble getting enough food?</td></tr></table></td></tr>
<tr><td></td><td></td><td>Sampling strategies</td><td>Same as previous construct</td></tr>
<tr><td></td><td></td><td>Sample sizes</td><td>Same as previous construct</td></tr>
<tr><td></td><td></td><td>Data analysis</td><td>Same as previous construct</td></tr>
<tr><td>144</td><td>crop diversity</td><td>Data collection</td><td>Same as previous construct</td></tr>
<tr><td></td><td></td><td>Operational questions</td><td>Table 1 includes an explanation of how each sub-component was quantified, the survey question used to collect the data, the original source of the survey question, and potential sources of bias.
[...]
<table><tr><td colspan="3">Table 1 Major components and sub-components comprising the Livelihood Vulnerability Index (LVI) developed for two districts of Mozambique.</td></tr><tr><td>Sub-components</td><td>Explanation of sub-components</td><td>Survey question</td></tr><tr><td>Average Crop Diversity Index (range: >0–1)a</td><td>The inverse of (the number of crops grown by a household +1). e.g., A household that grows pumpkin, maize, nhemba beans, and cassava will have a Crop Diversity</td><td>What kind of crops does your household grow?</td></tr></table></td></tr>
<tr><td></td><td></td><td>Sampling strategies</td><td>Same as previous construct</td></tr>
<tr><td></td><td></td><td>Sample sizes</td><td>Same as previous construct</td></tr>
<tr><td></td><td></td><td>Data analysis</td><td>Same as previous construct</td></tr>
<tr><td>145</td><td>dont save crops</td><td>Data collection</td><td>Same as previous construct</td></tr>
<tr><td></td><td></td><td>Operational questions</td><td>Table 1 includes an explanation of how each sub-component was quantified, the survey question used to collect the data, the original source of the survey question, and potential sources of bias.
[...]
<table><tr><td>Table 1 Major components and sub-components</td></tr></table></td></tr>
</table>

<table>
<tr><td></td><td></td><td></td><td><table><tr><td colspan="3">comprising the Livelihood Vulnerability Index (LVI) developed for two districts of Mozambique.</td></tr><tr><td>Sub-components</td><td>Explanation of sub-components</td><td>Survey question</td></tr><tr><td>Percent of households that do not save crops</td><td>Percentage of households that do not save crops from each harvest.</td><td>Does your family save some of the crops you harvest to eat during a different time of year?</td></tr></table></td></tr>
<tr><td></td><td></td><td>Sampling strategies</td><td>Same as previous construct</td></tr>
<tr><td></td><td></td><td>Sample sizes</td><td>Same as previous construct</td></tr>
<tr><td></td><td></td><td>Data analysis</td><td>Same as previous construct</td></tr>
<tr><td>146</td><td>dont save seeds</td><td>Data collection</td><td>Same as previous construct</td></tr>
<tr><td></td><td></td><td>Operational questions</td><td>Table 1 includes an explanation of how each sub-component was quantified, the survey question used to collect the data, the original source of the survey question, and potential sources of bias.
[...]
<table><tr><td colspan="3">Table 1 Major components and sub-components comprising the Livelihood Vulnerability Index (LVI) developed for two districts of Mozambique.</td></tr><tr><td>Sub-components</td><td>Explanation of sub-components</td><td>Survey question</td></tr><tr><td>Percent of households that do not save seeds</td><td>Percentage of households that do not have seeds from year to year.</td><td>Does your family save seeds to grow the next year?</td></tr></table></td></tr>
<tr><td></td><td></td><td>Sampling strategies</td><td>Same as previous construct</td></tr>
<tr><td></td><td></td><td>Sample sizes</td><td>Same as previous construct</td></tr>
<tr><td></td><td></td><td>Data analysis</td><td>Same as previous construct</td></tr>
<tr><td>147</td><td>Water conflict</td><td>Data collection</td><td>Same as previous construct</td></tr>
<tr><td></td><td></td><td>Operational questions</td><td>Table 1 includes an explanation of how each sub-component was quantified, the survey question used to collect the data, the original source of the survey question, and potential sources of bias.
[...]
<table><tr><td colspan="3">Table 1 Major components and sub-components comprising the Livelihood Vulnerability Index (LVI) developed for two districts of Mozambique.</td></tr><tr><td>Sub-components</td><td>Explanation of sub-components</td><td>Survey question</td></tr><tr><td>Percent of households reporting water conflicts</td><td>Percentage of households that report having heard about conflicts over water in their</td><td>In the past year, have you heard about any conflicts over water in your community?</td></tr></table></td></tr>
</table>

<table>
<tr><td></td><td></td><td></td><td><table><tr><td></td><td>community.</td><td></td></tr></table></td></tr>
<tr><td></td><td></td><td>Sampling strategies</td><td>Same as previous construct</td></tr>
<tr><td></td><td></td><td>Sample sizes</td><td>Same as previous construct</td></tr>
<tr><td></td><td></td><td>Data analysis</td><td>Same as previous construct</td></tr>
<tr><td></td><td></td><td>Sampling strategies</td><td>Same as previous construct</td></tr>
<tr><td></td><td></td><td>Sample sizes</td><td>Same as previous construct</td></tr>
<tr><td></td><td></td><td>Data analysis</td><td>Same as previous construct</td></tr>
<tr><td>148</td><td>natural water source</td><td>Data collection</td><td>Same as previous construct</td></tr>
<tr><td></td><td></td><td>Operational questions</td><td>Table 1 includes an explanation of how each sub-component was quantified, the survey question used to collect the data, the original source of the survey question, and potential sources of bias.
[...]
<table><tr><td colspan="3">Table 1 Major components and sub-components comprising the Livelihood Vulnerability Index (LVI) developed for two districts of Mozambique.</td></tr><tr><td>Sub-components</td><td>Explanation of sub-components</td><td>Survey question</td></tr><tr><td>Percent of households that utilize a natural water source</td><td>Percentage of households that report a creek, river, lake, pool, or hole as their primary water source.</td><td>Where do you collect your water from?</td></tr></table></td></tr>
<tr><td></td><td></td><td>Sampling strategies</td><td>Same as previous construct</td></tr>
<tr><td></td><td></td><td>Sample sizes</td><td>Same as previous construct</td></tr>
<tr><td></td><td></td><td>Data analysis</td><td>Same as previous construct</td></tr>
<tr><td>149</td><td>proximity to water source</td><td>Data collection</td><td>Same as previous construct</td></tr>
<tr><td></td><td></td><td>Operational questions</td><td>Table 1 includes an explanation of how each sub-component was quantified, the survey question used to collect the data, the original source of the survey question, and potential sources of bias.
[...]
<table><tr><td colspan="3">Table 1 Major components and sub-components comprising the Livelihood Vulnerability Index (LVI) developed for two districts of Mozambique.</td></tr><tr><td>Sub-components</td><td>Explanation of sub-components</td><td>Survey question</td></tr><tr><td>Average time to water source (minutes)</td><td>Average time it takes the households to travel to their primary water source.</td><td>How long does it take to get to your water source?</td></tr></table></td></tr>
<tr><td></td><td></td><td>Sampling strategies</td><td>Same as previous construct</td></tr>
<tr><td></td><td></td><td>Sample sizes</td><td>Same as previous construct</td></tr>
<tr><td></td><td></td><td>Data analysis</td><td>Same as previous construct</td></tr>
<tr><td>150</td><td>inconsistent water</td><td>Data collection</td><td>Same as previous construct</td></tr>
</table>

<table>
<tr><td></td><td>supply</td><td>Operational questions</td><td>Table 1 includes an explanation of how each sub-component was quantified, the survey question used to collect the data, the original source of the survey question, and potential sources of bias.
[...]
<table><tr><td colspan="3">Table 1 Major components and sub-components comprising the Livelihood Vulnerability Index (LVI) developed for two districts of Mozambique.</td></tr><tr><td>Sub-components</td><td>Explanation of sub-components</td><td>Survey question</td></tr><tr><td>Percent of households that do not have a consistent water supply</td><td>Percentage of households that report that water is not available at their primary water source everyday</td><td>Is this water available everyday?</td></tr></table></td></tr>
<tr><td></td><td></td><td>Sampling strategies</td><td>Same as previous construct</td></tr>
<tr><td></td><td></td><td>Sample sizes</td><td>Same as previous construct</td></tr>
<tr><td></td><td></td><td>Data analysis</td><td>Same as previous construct</td></tr>
<tr><td>151</td><td>inverse water stored</td><td>Data collection</td><td>Same as previous construct</td></tr>
<tr><td></td><td></td><td>Operational questions</td><td>Table 1 includes an explanation of how each sub-component was quantified, the survey question used to collect the data, the original source of the survey question, and potential sources of bias.
[...]
<table><tr><td colspan="3">Table 1 Major components and sub-components comprising the Livelihood Vulnerability Index (LVI) developed for two districts of Mozambique.</td></tr><tr><td>Sub-components</td><td>Explanation of sub-components</td><td>Survey question</td></tr><tr><td>Inverse of the average number of liters of water stored per household (range: >0–1)</td><td>The inverse of (the average number of liters of water stored by each household + 1).</td><td>What containers do you usually store water in? How many? How many liters are they?</td></tr></table></td></tr>
<tr><td></td><td></td><td>Sampling strategies</td><td>Same as previous construct</td></tr>
<tr><td></td><td></td><td>Sample sizes</td><td>Same as previous construct</td></tr>
<tr><td></td><td></td><td>Data analysis</td><td>Same as previous construct</td></tr>
<tr><td>152</td><td>Flood, drought, cyclone events</td><td>Data collection</td><td>Same as previous construct</td></tr>
<tr><td></td><td></td><td>Operational questions</td><td>Table 1 includes an explanation of how each sub-component was quantified, the survey question used to collect the data, the original source of the survey question, and potential sources of bias.
[...]
<table><tr><td>Table 1 Major components and sub-components comprising the Livelihood Vulnerability Index (LVI) developed for two districts of Mozambique.</td></tr></table></td></tr>
</table>

<table>
<tr><td></td><td></td><td></td><td><table><tr><td>Sub-components</td><td>Explanation of sub-components</td><td>Survey question</td></tr><tr><td>Average number of flood, drought, and cyclone events in the past 6 years (range: 0–7)</td><td>Total number of floods, droughts, and cyclones that were reported by households in the past 6 years.</td><td>How many times has this area been affected by a flood/cyclone/drought in 2001–2007?</td></tr></table></td></tr>
<tr><td></td><td></td><td>Sampling strategies</td><td>Same as previous construct</td></tr>
<tr><td></td><td></td><td>Sample sizes</td><td>Same as previous construct</td></tr>
<tr><td></td><td></td><td>Data analysis</td><td>Same as previous construct</td></tr>
<tr><td>153</td><td>injury or death from disaster</td><td>Data collection</td><td>Same as previous construct</td></tr>
<tr><td></td><td></td><td>Operational questions</td><td>Table 1 includes an explanation of how each sub-component was quantified, the survey question used to collect the data, the original source of the survey question, and potential sources of bias.
[...]
<table><tr><td colspan="3">Table 1 Major components and sub-components comprising the Livelihood Vulnerability Index (LVI) developed for two districts of Mozambique.</td></tr><tr><td>Sub-components</td><td>Explanation of sub-components</td><td>Survey question</td></tr><tr><td>Percent of households with an injury or death as a result of the most severe natural disaster in the past 6 years</td><td>Percentage of households that reported either an injury to or death of one of their family members as a result of the most severe flood, drought, or cyclone in the past 6 years.</td><td>Was anyone in your family injured in the flood/cyclone drought? Did anyone in your family die during the flood/cyclone/drought?</td></tr></table></td></tr>
<tr><td></td><td></td><td>Sampling strategies</td><td>Same as previous construct</td></tr>
<tr><td></td><td></td><td>Sample sizes</td><td>Same as previous construct</td></tr>
<tr><td></td><td></td><td>Data analysis</td><td>Same as previous construct</td></tr>
<tr><td>154</td><td>no warning of disaster</td><td>Data collection</td><td>Same as previous construct</td></tr>
<tr><td></td><td></td><td>Operational questions</td><td>Table 1 includes an explanation of how each sub-component was quantified, the survey question used to collect the data, the original source of the survey question, and potential sources of bias.
[...]
<table><tr><td>Table 1 Major components and sub-components</td></tr></table></td></tr>
</table>

<table>
<tr><td></td><td></td><td></td><td><table><tr><td colspan="3">comprising the Livelihood Vulnerability Index (LVI) developed for two districts of Mozambique.</td></tr><tr><td>Sub-components</td><td>Explanation of sub-components</td><td>Survey question</td></tr><tr><td>Percent of households that did not receive a warning about the pending natural disasters</td><td>Percentage of households that did not receive a warning about the most severe flood, drought, and cyclone event in the past 6 years.</td><td>Did you receive a warning about the flood/cyclone/drought before it happened?</td></tr></table></td></tr>
<tr><td></td><td></td><td>Sampling strategies</td><td>Same as previous construct</td></tr>
<tr><td></td><td></td><td>Sample sizes</td><td>Same as previous construct</td></tr>
<tr><td></td><td></td><td>Data analysis</td><td>Same as previous construct</td></tr>
<tr><td>155</td><td>maximum temperature</td><td>Data collection</td><td>provincial data; weather station based in the provincial capital</td></tr>
<tr><td></td><td></td><td>Operational questions</td><td><table><tr><td colspan="3">Table 1 Major components and sub-components comprising the Livelihood Vulnerability Index (LVI) developed for two districts of Mozambique.</td></tr><tr><td>Sub-components</td><td>Explanation of sub-components</td><td>Survey question</td></tr><tr><td>Mean standard deviation of the daily average maximum temperature by month</td><td>Standard deviation of the average daily maximum temperature by month between 1998 and 2003 was averaged for each provinceb</td><td>1998–2003: provincial data; weather station based in the provincial capital</td></tr></table></td></tr>
<tr><td></td><td></td><td>Sampling strategies</td><td></td></tr>
<tr><td></td><td></td><td>Sample sizes</td><td></td></tr>
<tr><td></td><td></td><td>Data analysis</td><td>Same as previous construct</td></tr>
<tr><td>156</td><td>minimum temperature</td><td>Data collection</td><td>provincial data; weather station based in the provincial capital</td></tr>
<tr><td></td><td></td><td>Operational questions</td><td><table><tr><td colspan="3">Table 1 Major components and sub-components comprising the Livelihood Vulnerability Index (LVI) developed for two districts of Mozambique.</td></tr><tr><td>Sub-components</td><td>Explanation of sub-components</td><td>Survey question</td></tr><tr><td>Mean standard deviation of the daily average</td><td>Standard deviation of the average daily</td><td>1998–2003: provincial data; weather station</td></tr></table></td></tr>
</table>

<table>
<tr><td></td><td></td><td></td><td><table><tr><td>minimum temperature by month</td><td>minimum temperature by month between 1998 and 2003 was averaged for each province.</td><td>based in the provincial capital</td></tr></table></td></tr>
<tr><td></td><td></td><td>Sampling strategies</td><td></td></tr>
<tr><td></td><td></td><td>Sample sizes</td><td></td></tr>
<tr><td></td><td></td><td>Data analysis</td><td></td></tr>
<tr><td>157</td><td>average precipitation</td><td>Data collection</td><td>provincial data; weather station based in the provincial capital</td></tr>
<tr><td></td><td></td><td>Operational questions</td><td><table><tr><td colspan="3">Table 1 Major components and sub-components comprising the Livelihood Vulnerability Index (LVI) developed for two districts of Mozambique.</td></tr><tr><td>Sub-components</td><td>Explanation of sub-components</td><td>Survey question</td></tr><tr><td>Mean standard deviation of average precipitation by month</td><td>Standard deviation of the average monthly precipitation between 1998 and 2003 was averaged for each province</td><td>1998–2003: provincial data; weather station based in the provincial capital</td></tr></table></td></tr>
<tr><td></td><td></td><td>Sampling strategies</td><td></td></tr>
<tr><td></td><td></td><td>Sample sizes</td><td></td></tr>
<tr><td></td><td></td><td>Data analysis</td><td>Same as previous construct</td></tr>
<tr><td colspan="2">Candidate-level Analysis</td><td colspan="2">Same as ‘dependency ratio’</td></tr>
</table>

<table>
<tr><td colspan="3">Information relating to further development of framework</td></tr>
<tr><td colspan="3"><u>Constructs with no adequate operationalizations</u>
FULLY AND ADEQUATELY OPERATIONALIZED</td></tr>
<tr><td colspan="3">Constructs with more than one adequate operationalizations</td></tr>
<tr><td>Construct</td><td>Preference rank</td><td>Summary of operationalization</td></tr>
</table>

Nested vulnerability

Framework summary
**Name of framework:** Nested Vulnerability
**Description of framework:**<br>This framework is concerned with 'teleconnections' between households in geographically distant localities. It examines the mechanisms through which smallholders in distinct geographical contexts respond differently to exogenous shocks (climatic or not-climatic) and in so doing create a new set of influences on distant locations through connections in a nested globally interconnected system.
**Key constructs and definitions:**<br>Livelihood vulnerability (B): By placing the household as the focus of analysis, livelihood approaches highlight both the exogenous drivers (i.e. the risk and stress factors) and the factors internal to the household (i.e. ability to mitigate and cope with stress) which together influence household security and well-being (Chambers and Conway, 1992; Ellis, 1998). **Eakin et al (2008)**<br>Nested and teleconnected livelihood vulnerability: In this article we use the concept of ''nested and teleconnected vulnerabilities'' to illustrate how the vulnerabilities and responses of farm households in distinct geographic locations are linked through cross-scalar processes, as well as ''teleconnected'' in space and time. In a nested system, profound changes in key variables that operate normally only at one level, e.g., within a defined geographic region or administrative domain, can have non-linear outcomes for processes operating at broader scales of analysis (Gunderson and Holling, 2001). **Eakin et al (2008)**<br>Nested system: In a nested system, profound changes in key variables that operate normally only at one level, e.g., within a defined geographic region or administrative domain, can have non-linear outcomes for processes operating at broader scales of analysis (Gunderson andHolling, 2001). Local level processes can episodically influence larger scale phenomena, and such explosive ''upward cascades'' can be sources of surprise at distant locations. **Eakin et al (2008)**<br>Cross scalar teleconnections: ''teleconnections'', a term used in climatology in relation to ''any transmission of a coherent effect beyond the location where the forcing occurred'' (Chase et al., 2005). For example, one of the teleconnections associated with the El Nino-Southern Oscillation effect is severe drought in Northeastern Brazil. Teleconnections are also associated with other climate phenomena such as the North Atlantic Oscillation. The label of ''teleconnection'' is not explanatory in and of itself, but rather signifies the existence of a correlation in events, and highlights the need to explore the connecting mechanisms and drivers in order to anticipate outcomes. **Eakin et al (2008)**<br>Exogenous drivers: exogenous drivers (i.e. the risk and stress factors) **Eakin et al (2008)**<br>Response outcomes: outcomes of these responses in terms of individual or household welfare. **Eakin et al (2008)**
**Ideal type model:** (uneven)

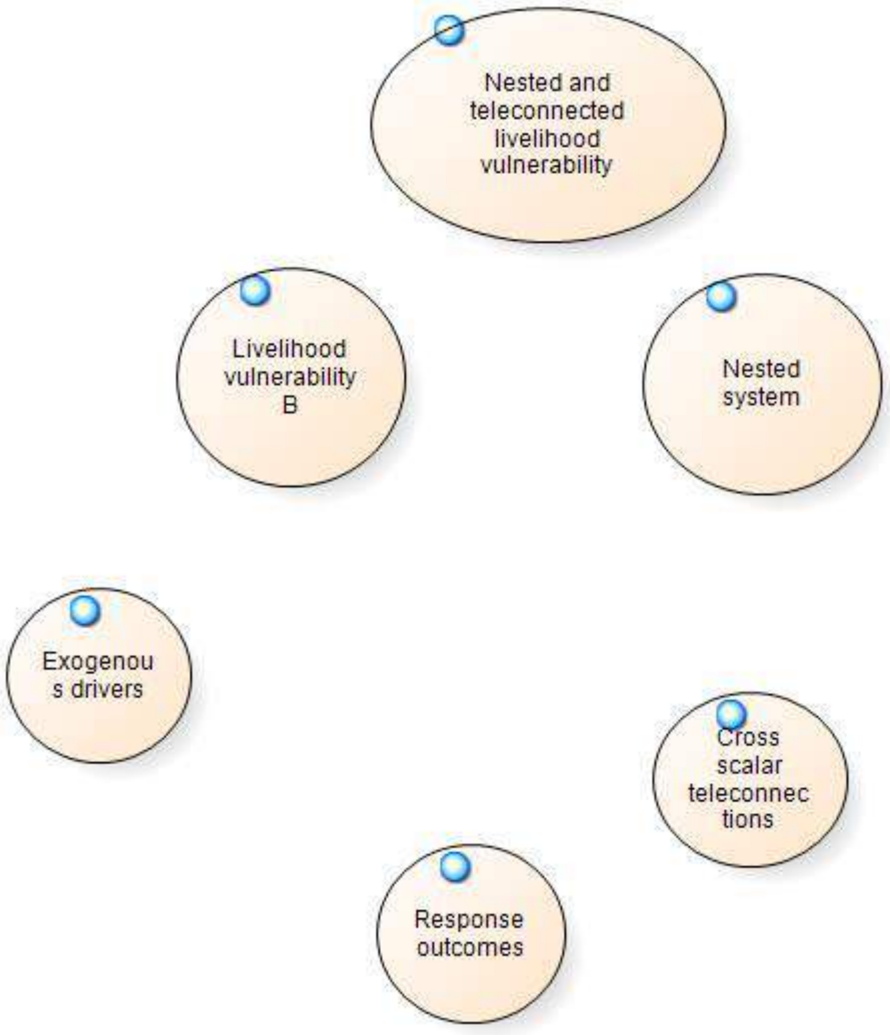

Articles using framework: Eakin et al (2008)

Operationalization of key constructs:

**Operationalization of constructs**		
**Construct operationalized:** Nested and teleconnected livelihood vulnerability		
**Source article(s):** Eakin et al (2008)		
**Selected by:**	default	
Sub-constructs	Intermediate constructs	Livelihood vulnerability
	Directly operationalized constructs	Nested Systems; Exogenous drivers; geographically specific signals of change; geographically distant household vulnerability; household responses; response outcomes
Conceptual framework	In the following sections, we use the case of the responses of farmers in Vietnam and Mexico to the evolution of the global coffeemarket over the past three decades to illustrate the insights that can be gained fromemploying a concept of nestedandteleconnectedlivelihoodvulnerability. Inthe case we present here, we argue that the vulnerability of individual farmers to the experience of welfare loss is connected not only through the structure of the global coffee	

		commodity chain, but also through global ideological shifts affecting national policy, the movement of labor, the material flow of coffee stocks, channels of information, and, in reverse, through the broader environmental and institutional impli- cations of local adaptive action.<br>[...]<br>In this article we use the concept of ‘‘nested and tele-<br>connected vulnerabilities’’ to illustrate how the vulnerabilities and responses of farm households in distinct geographic locations are linked through cross-scalar processes, as well as ‘‘teleconnected’’ in space and time. In a nested system, profoundchanges inkeyvariablesthatoperatenormallyonly at one level, e.g., within a defined geographic region or admin- istrative domain, can have non-linear outcomes for processes operating at broader scales of analysis (Gunderson andHolling, 2001)<br>[...]<br>Livelihood vulner- ability is composed of exogenous risks, household responses to risks, and the outcomes of these responses in terms of individual or household welfare.<br>[...]<br>In our case, we argue that geographically specific signals of change – such as a shift in market opportunities, a drought, a change in public policy or new form of land use in a specific location – can create risks and opportunities	
Operationalization of sub-constructs			
158	Nested Systems	Data collection	NOT TRANSPARENT
		Operational questions	
		Sampling strategies	
		Sample sizes	
		Data analysis	
159	Exogenous drivers	Data collection	NOT TRANSPARENT
		Operational questions	
		Sampling strategies	
		Sample sizes	
		Data analysis	
160	geographically specific signals of change	Data collection	NOT TRANSPARENT
		Operational questions	
		Sampling strategies	
		Sample sizes	
		Data analysis	
161	geographically distant household vulnerability	Data collection	NOT TRANSPARENT
		Operational questions	
		Sampling strategies	
		Sample sizes	
		Data analysis	
162	household responses	Data collection	NOT TRANSPARENT
		Operational questions	
		Sampling strategies	
		Sample sizes	
		Data analysis	
163	response outcomes	Data collection	NOT TRANSPARENT
		Operational questions	
		Sampling strategies	

		Sample sizes	
		Data analysis	
Candidate-level Analysis			

Information relating to further development of framework		
Constructs with no adequate operationalizations<br>NO ADEQUATE OPERATIONALIZATIONS<br>- Nested and teleconnected livelihood vulnerability (not adequately operationalized)		
Constructs with more than one adequate operationalizations		
Construct	Preference rank	Summary of operationalization

Nkondze et al

Framework summary
**Name of framework:** Nkondze et al (2013)
**Description of framework:**<br>At a very general level, this framework investigates factors affecting household vulnerability. An index is constructed through which to measure vulnerability, which is then analysed against socio-economic data to determine the most significant factors influencing levels of household vulnerability.
**Key constructs and definitions:**<br>Factors affecting vulnerability: No definition<br>Household vulnerability to climate change: No definition
**Ideal type model:**<br>No defined constructs
**Articles using framework:** Nkondze et al (2013)
**Operationalization of key constructs:**

**Information relating to further development of framework**		
Constructs with no adequate operationalizations<br>NO OPERATIONALIZATIONS		
Constructs with more than one adequate operationalizations		
Construct	Preference rank	Summary of operationalization

Patterns of smallholder vulnerability

**Framework summary**
**Name of framework:** Patterns of smallholder vulnerability
**Description of framework:**<br>Cluster pattern analysis is employed in this framework to investigate whether there are, and which, characteristics that explain the causal structure of vulnerability to weather extremes. A measure of household/smallholder vulnerability is created using a combination of IPCC and Food Security household-level indicators. A cluster pattern analysis is then run relating measures of vulnerability to socio-economic and other household-level data to identify characterisitcs, and in particular combinations of characterisitcs that are related to concentrations of vulnerability.
**Key constructs and definitions:**<br>Vulnerability (IPCC): The degree to which a system is susceptible to, or unable to cope with, adverse effects of climate change, including climate variability and extremes. Vulnerability is a function of the character, magnitude, and rate of climate variation to which a system is exposed, its sensitivity, and its adaptive capacity. **(Fussel & Klein 2006)**<br>Exposure: The nature and degree to which a system is exposed to significant climatic variations.<br>The exposure of a system to climate stimuli depends on the level of global cli- mate change and, due to the spatial heterogeneity of anthropogenic climate change, on the system's location **(Fussel & Klein 2006)**<br>Sensitivity: The degree to which a system is affected, either adversely or beneficially, by climate-related stimuli. [...] The effect may be direct [...]or indirect [...]<br>[...]<br>The sensitivity of a system denotes the (generally multi-factorial and dynamic) dose – response relationship between its exposure to climatic stimuli and the re- sulting impacts. **(Fussel & Klein 2006)**<br>Adaptive capacity: the adaptive capacity of smallholders (the term as used in this study encompasses the coping capacity) describes the ability to adjust to weather extremes, manage damages or explore alternative livelihood opportunities. **(Sietz et al 2012)**<br>Cluster pattern analysis: Without such a pre-selection, alternative approaches investigate the structure of the data space spanned by selected vulnerability indicators using cluster analysis. They deliver useful insights into recurrent indicator combinations based on similarities among units of analysis, in cases where such a grouping exists. For example, clustering revealed typical livelihood strategies employed by small- holders in Mexico and Botswana (Eakin 2005; Sallu et al. 2010). **(Sietz et al 2012)**<br>Food security: Food security is often discussed in terms of four<br>dimensions: food availability, access, stability of supply/ access and utilisation (FAO 2000). **(Sietz et al 2012)**
**Ideal type model:**

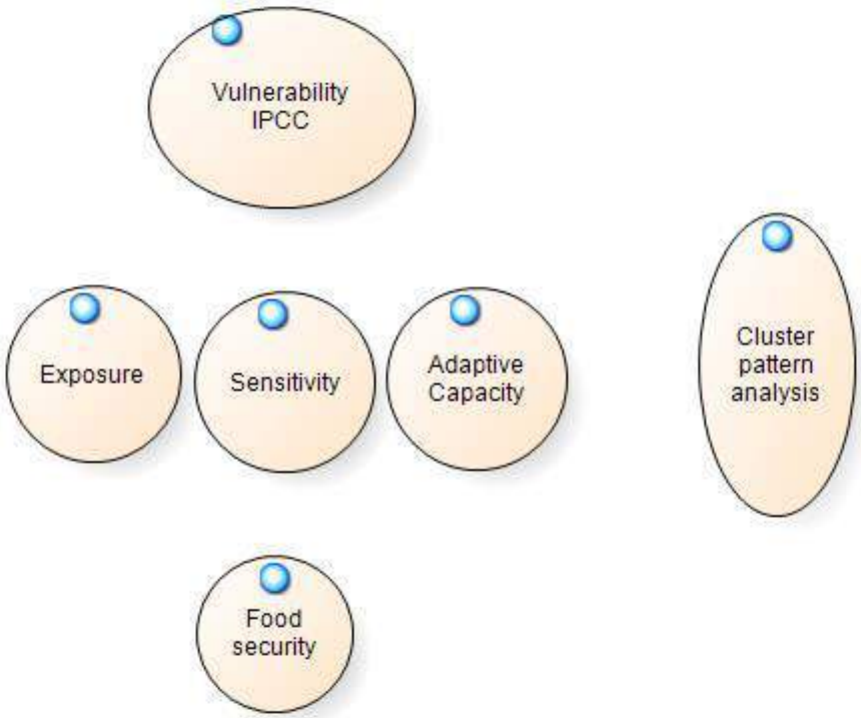

Articles using framework: (Sietz et al 2012)

Operationalization of key constructs:

**Operationalization of constructs**		
**Construct operationalized:** Vulnerability IPCC		
**Source article(s):**		
**Selected by:**	expert selection [justification]	
Sub-constructs	Intermediate constructs	
	Directly operationalized constructs	
Conceptual framework		
Operationalization of sub-constructs		
164 •	Data collection	
	Operational questions	
	Sampling strategies	
	Sample sizes	
	Data analysis	
165 •	Data collection	
	Operational questions	
	Sampling strategies	
	Sample sizes	
	Data analysis	
166 •	Data collection	
	Operational questions	

		Sampling strategies	
		Sample sizes	
		Data analysis	
167	•	Data collection	
		Operational questions	
		Sampling strategies	
		Sample sizes	
		Data analysis	
Candidate-level Analysis			

**Operationalization of constructs**			
**Construct operationalized:** Cluster Pattern analysis			
**Source article(s):** Sietz et al			
**Selected by:**	default/expert selection [justification]		
Sub-constructs	Intermediate constructs		
	Directly operationalized constructs		
Conceptual framework	DIRECT OPERATIONALIZATION		
Operationalization of sub-constructs			
168.		Data collection	The ALTAGRO (2006) data base contains detailed<br>quantitative information for 527 smallholder households collected through household questionnaires.<br>[...]<br>The necessary weather information is available in good quality for the 1996–2006 period for two stations located in Puno and Cabanillas (see Fig. 1). Table 2 shows the average pre- cipitation and temperature for both stations.
		Operational questions	Ten categories describe the smallholder households covering personal information about the family members (e.g. occupation, education level, age), production systems (e.g. crop and livestock assets, labour input, processing and commer- cialisation of produce), weather conditions, food reserves, income, some expenses and credits.<br>[...]<br>The following data are taken from the ALTAGRO<br>(2006) data base to indicate the mechanisms relevant in this study. As the first dimension, the harvest failure risk is indicated by the number of production zones used for crop and pasture cultivation. The indicator considers plains, hillsides and hills. The second dimension of the area con- straint is measured by the crop area as an important pre- requisite for food production. The pasture area highly correlates to livestock keeping and is therefore reflected in the livestock measure. The third dimension, the livestock constraint, is characterised by the number and types of animals. To compare various animal species, we calculated standardised livestock units in relation to an improved cattle variety based on the livestock-specific

metabolism (Kleiber 1961). Average livestock weights were estimated using 20 representative animals of each species in the study region. Since fodder production is an essential condition for livestock keeping, the respective indicator contains a reference to the area and productivity of pasture land. Furthermore, the productivity constraint as the fourth dimension is provided for the major food crops potatoes and quinua. It averages the household's productivity across species, varieties and production zones for each crop. Again, we concentrate on food crops since the productivity of pastures is already included in the livestock measure. The fifth dimension of education deprivation relates to the number of years that a household head attended school. School attendance is classified according to the four levels: no formal education, primary, secondary and higher edu- cation. Finally, the lack of alternative income as the sixth dimension is quantified by the sum of annual monetary income from local off-farm activities and remittances. People usually receive remittances from household
495
members who migrate for climate-independent labour, for example mining and commerce. Table 1 summarises the indicators used to assess vulnerability.
[...]

Table 1 Indicators of households' sensitivity and adaptive capacity. The range of the area and livestock constraints as well as lack of alternative income is provided following winsorisation, see description in text. (Data source: ALTAGRO 2006)

Dimension of sensitivity and adaptive capacity	*Indicator*	*Range*
Harvest failure risk	Number of production zones used for cultivation	1–3
Area constraint	Crop area	0.1–1.3 ha/person[a]
Livestock constraint	Livestock units	0.1–8.0 livestock units/person
Productivity constraint	Potato productivity<br>Quinua productivity	0.1–10.0 t/ha<br>0.2–1.8 t/ha
Education deprivation	Education level of household head	1–4
Lack of alternative income	Local off-farm income and remittances	0–2400 Soles/year*person

a Average: 4 persons per household

[...]
The necessary weather information is available in good quality for the 1996–2006 period for two stations located in Puno and Cabanillas (see Fig. 1). Table 2 shows the average pre- cipitation and temperature for both stations.
[...]

Table 2 Mean precipitation and temperature for 1996–2006 at Puno and Cabanillas stations (Data source: Servicio Nacional de Meteorologı´ae Hidrologı´a del Peru´, SENAMHI)

	Mean values for 1996–2006												
	Jan	Feb	Mar	Apr	May	Jun	Jul	Aug	Sept	Oct	Nov	Dec	Total
Precipitation (mm)													
Puno	201	161	138	60	7	3	4	14	27	51	48	88	801
Cabanillas	166	165	112	56	6	1	3	11	19	54	55	91	738
Mean temperature (□C)													
Puno	10.8	10.7	10.6	9.7	8.1	6.8	6.8	7.9	9.3	10.4	11.0	11.5	9.5
Cabanillas	10.6	10.5	10.5	9.8	8.6	7.3	6.9	8.1	9.6	10.6	11.1	11.3	9.6
Minimum temperature (□C)													
Puno	5.7	5.8	5.4	3.8	0.8	-0.9	-1.1	0.4	1.9	3.6	4.3	5.4	2.9
Cabanillas	5.3	5.5	5.2	3.7	1.1	-0.8	-1.5	0.3	2.1	3.7	4.2	5.1	2.8

Sampling strategies	
Sample sizes	
Data analysis	The cluster analysis was performed using a sequence of a common hierarchical and exchange algorithm, i.e., hclust and kmeans, using the statistics package R (MacQueen 1967; RDCT 2009). Based on stochastic initialisation, we calculated the reproducibility of partitions for a pre-given number of clusters to determine whether the algorithm detects stable or unstable (inappropriate) partitions. The share of households that were categorised in the same cluster in two partitions is expressed as ‘‘consistency measure’’. The higher this measure, the more reliable the cluster results. We calculated the consistency measure as the average of 200 pairwise comparisons of partitions with a given number of clusters. Ultimately, the consistency measure enables us to identify the optimal number of clusters to be analysed. Further methodological details are outlined in a previous application of the cluster approach to dryland vulnerability on a global scale (Sietz et al. 2011).
Candidate-level Analysis	DIRECT OPERATIONALIZATION

**Information relating to further development of framework**		
Constructs with no adequate operationalizations<br>FULLY AND ADEQUATELY OPERATIONALIZED		
Constructs with more than one adequate operationalizations		
Construct	Preference rank	Summary of operationalization

Perceptions of climate change

Framework summary

Name of framework: Perceptions of climate change

Description of framework:
This category constitutes less a coherent framework and more of a collection of studies whose approach differs significantly from the majority of studies in this review in terms of epistemological orientation and position on the intervention cycle. A tentative general description of this category is that the approach focusses on articulating perceptions of people whose livelihoods are affected by climate change (often farmers), and in particular their perceptions of climate change as a physical phenomenon, perceptions of the impact climate change has on their livelihoods, and respondent reported strategies of coping or adaptation.

Key constructs and definitions:
Farmer perceptions: there is an alternative approach which underscores how individuals perceive their environment and make decisions, with mal-adaptations attributed to problems in perception, cognition or the lack of available information (Diggs, 1991; Saarinen, 1966; Taylor et al., 1988). The main point is that from whatever level these adaptation measures are taken, the adaptation and coping measures depend on households' perceptions of extreme events and the problems associated with them (Davies, 1993). **(Mubaya et al 2012)**
Adaptation strategy: Adaptations, or adaptive strategies, employed by individuals or groups are depicted as being mediated through their relative adaptive capacities, indicating that adaptations may or may not be accessed according to the distribution of various types of resources such as physical or social capital, as developed by Adger and Kelly (1999). **(Westerhoff & Smit 2009)**
Coping strategy: No definition

Ideal type model:
Insufficient defined constructs.

Articles using framework: Gandure et al (2013); Mengitsu (2011); Mubaya (2012); Westerhoff & Smit (2009)

Operationalization of key constructs:

**Operationalization of constructs**			
**Construct operationalized:** Farmer perceptions			
**Source article(s):** Mubaya et al			
**Selected by:**		default	
Sub-constructs		Intermediate constructs	
		Directly operationalized constructs	
Conceptual framework		DIRECETLY OPERATIONALIZED	
Operationalization of sub-constructs			
169	•	Data collection	The qualitative methods of data collection used include Participatory Rural Appraisal (PRA) techniques such as historical trend analysis and matrix scoring and ranking and Focus Group Discussions (FGDs). The quantitative method used is the household questionnaire survey.
		Operational questions	2.2.2. Qualitative assessments FGDs were used to first of all establish the general perceptions regarding climate change and variability and their causes and various stressors that confront farmers' livelihoods (see

			Appendix 2). Following this, it was considered important for this study to factor in how farmers regard climate change and variability as an obstacle to their livelihoods among the multiple stressors that they had identified. Among these stressors are climate variability in different forms, issues of financial capital, issues related to cattle pests and diseases, inadequate draught power, marketing issues and HIV and AIDS. A matrix scoring and ranking exercise was then facilitated for<br>farmers. Farmers were asked as a group to select from the long list of stressors the ones they considered critical for the purposes of scoring and ranking. The second step involved participants defining criteria that they would use to evaluate these stressors. These criteria include food security, income generation, crop production and livelihood security. Through group consensus, farmers then decided how much to allocate each shock out of a total of 20 points, based on the group defined criteria. Historical trend lines were used to elicit information on specific historical trends in farmers' perceptions regarding changes in climate over a period of 20 years and as far back as they could recall. Specifically, participants were asked to recall major occurrences that had a bearing on climate and weather, community resources, and even the political situation. They were then asked to indicate what occurrences had the greatest impact on their livelihoods among the cited events.<br>[...]<br>2.2.3. Quantitative assessments The questionnaire survey was used to collect household data<br>and complement data generated through the qualitative methods. This survey collected data on changes in crops grown over a period of five years and reasons for these changes, indicators for good and bad crop production seasons and years considered to be good or bad over a ten year period. Questions in the survey also related to changes in weather patterns over a ten year period in relation to agriculture and what might have caused these changes. General household characteristics were also captured in this survey (see Appendix 1).
		Sampling strategies	A sample of 720 households across countries was selected for<br>the survey, 180 households per each of the four districts. Specifi- cally, systematic random sampling was employed to come up with six villages per district (making them 24 across countries) and 30 households per each of these villages, making a total of 380 households per country (this study was part of a big inter- institutional research-based development project). For FGDs and PRA workshops, a group of eight to 15 participants<br>was selected to represent the three villages per district, with approximately five representatives from each of the three villages per district. In coming up with this group,

			factors such as age and gender were used. In terms of gender, separate PRA workshops were held for men and women in order not to compromise the amount and quality of information that can be generated from the less confident if they were to be combined. Specifically, old men and women were incorporated into the sample for the group discussions in order to capture information related to historical trends in climate. Itwas envisaged that they would be able to recall as far back as they could and provide rich information on these trends. In the same context, youths were incorporated into the sample in order to validate some of the recent trends on climate suggested by the elderly.
		Sample sizes	A sample of 720 households across countries was selected for<br>the survey, 180 households per each of the four districts.<br>[...]<br>For FGDs and PRA workshops, a group of eight to 15 participants<br>was selected to represent the three villages per district, with approximately five representatives from each of the three villages per district.
		Data analysis	Qualitative data were categorised and analysed in four distinct themes. These themes are<br>Perceptions regarding changes in weather patterns, Perceptions regarding causes of changes and variability in climate, Perceptions regarding other stressors among farmers and Perceptions regarding climate change in relation to other stressors.<br>These perceptions were established in historical trend lines, FGDs and matrix scoring and ranking and they are presented in this manner in the sections under results and discussion.<br>[...]<br>Data from the questionnaire survey were entered into the Statistical Package for the Social Sciences (SPSS) and analysed by running descriptive frequencies in relation to the distinct themes highlighted in this section. These themes include perceptions regarding changes in weather patterns in general and for specific seasons and regarding causes of these changes. These frequencies were disaggregated by district and country.
Candidate-level Analysis		DIRECTLY OPERATIONALIZED	

**Operationalization of constructs**		
**Construct operationalized:** Adaptation strategy		
**Source article(s):**		
**Selected by:**	expert selection [justification]	
Sub-constructs	Intermediate constructs	

	Directly operationalized constructs	
Conceptual framework		
Operationalization of sub-constructs		
170 •	Data collection	
	Operational questions	
	Sampling strategies	
	Sample sizes	
	Data analysis	
171 •	Data collection	
	Operational questions	
	Sampling strategies	
	Sample sizes	
	Data analysis	
172 •	Data collection	
	Operational questions	
	Sampling strategies	
	Sample sizes	
	Data analysis	
173 •	Data collection	
	Operational questions	
	Sampling strategies	
	Sample sizes	
	Data analysis	
Candidate-level Analysis		

**Information relating to further development of framework**		
Constructs with no adequate operationalizations<br>- Coping strategies (no operationalizations)		
Constructs with more than one adequate operationalizations		
Construct	Preference rank	Summary of operationalization

Vulnerability as expected poverty, with extensions

**Framework summary**
**Name of framework:** Vulnerability as expected poverty, with extensions
**Description of framework:**<br>This framework conceives of vulnerability as the potential of a unit of analysis (usually a household) becoming or remaining poor in the future. It is an econometric approach that makes forward projections based on cross-sectional data and associated risks of climatic (and sometimes non-climatic) stress. In some cases, assessments of vulnerability based on expected poverty are then regressed against a series of socio-economic data to identify determinants of vulnerability.<br>Extension 1: This is a variant of the framework 'Vulnerability as Expected Poverty' described above. The principal difference is that whereas the former takes its focus as that of current and projected future levels of *poverty*, usually measured through consumption, the current framework by contrast focusses on a household's current and projected future *food security* status.<br>Extension 2: Another extension of the 'Vulnerability as Expected Poverty' framework described above. This variant is characterised by its inclusion of multi-level analysis. That is, projections are made for units of analysis at two different scales (usually household and community/local), and analysis is done of differences between units at different scales.
**Key constructs and definitions:**<br><u>Vulnerability as Expected Poverty</u>: In this article, we explore the notion of vulnerability to poverty, closely linked with the magnitude of the threat of poverty, measured ex-ante, before uncertainty has been resolved.<br>[...]<br>To clarify how all these intuitions come together under the concept of vulnerability, this paper proposes an axiomatic approach to themeasurement of both individual and aggregate vulnerability. **(Calvo & Dercon 2012)**<br><u>Poverty</u>: This study adopts the approach to measuring household economic vulnerability posited and elaborated in Chaudhuri's (2003) study of household vulnerability<br>[...]<br>Technically, the household vulnerability index is derived from the difference between the expected log per capita income and the minimum log per capita income threshold, with households having per capita incomes lower than the minimum per capita income defined as vulnerable (poor). The expected log per capita income is estimated using the three-step feasible generalised least squares (FGLS) method. **(Chhihn & Poch 2012)**<br><u>Food insecurity</u>: Food insecurityin the communities described bythe<br>case studies maybe conceptualized as one element in an entrenched and escalating cycle of vulnerability (Fig. 3). **(Misselhorn 2005)**<br><u>Expected future food security status</u>: conceptual framework drawn from it by Løvendal and Knowles (2005). **(Capaldo et al 2010)**<br><u>Future nutritional status</u>: conceptual framework drawn from it by Løvendal and Knowles (2005). **(Capaldo et al 2010)**<br><br><u>Idiosyncratic shocks</u>: Households in developing countries are frequently hit by severe idiosyncratic and covariate shocks resulting in high income volatility. 1<br>[...]<br>1. Here, and in the following, idiosyncratic shocks refer to household- specific shocks (e.g., injury, birth, death, or job loss of a household member) that are only weakly correlated across households within a community. Covariate shocks refer to shocks that are correlated across households within communities but only weakly correlated across communities (e.g., natural disasters or epidemics). **(Gunther & harttgen 2009)**<br><u>Covariate shocks</u>: Households in developing countries are frequently hit by severe idiosyncratic and covariate shocks resulting in high income volatility. 1<br>[...]<br>1. Here, and in the following, idiosyncratic shocks refer to household- specific shocks (e.g., injury, birth, death, or job loss of a household member) that are only weakly correlated across households within a community. Covariate

shocks refer to shocks that are correlated across households within communities but only weakly correlated across communities (e.g., natural disasters or epidemics). **(Gunther & harttgen 2009)**

Household level: Multilevel models are designed to analyze the relationship between variables that are measured at different hierarchical levels (for an introduction see, e.g., Bryk & Raudenbush, 1992; Goldstein, 1999; Hox, 2002). We speak of ‘‘hierarchical’’ or ‘‘multilevel’’ data structure whenever variables are collected at different hierarchical levels with lower-levels (e.g., house- holds) nested within higher-levels (e.g., communities). **(Gunther & harttgen 2009)**

Community level: Multilevel models are designed to analyze the relationship between variables that are measured at different hierarchical levels (for an introduction see, e.g., Bryk & Raudenbush, 1992; Goldstein, 1999; Hox, 2002). We speak of ‘‘hierarchical’’ or ‘‘multilevel’’ data structure whenever variables are collected at different hierarchical levels with lower-levels (e.g., house- holds) nested within higher-levels (e.g., communities). **(Gunther & harttgen 2009)**

Ideal type model:

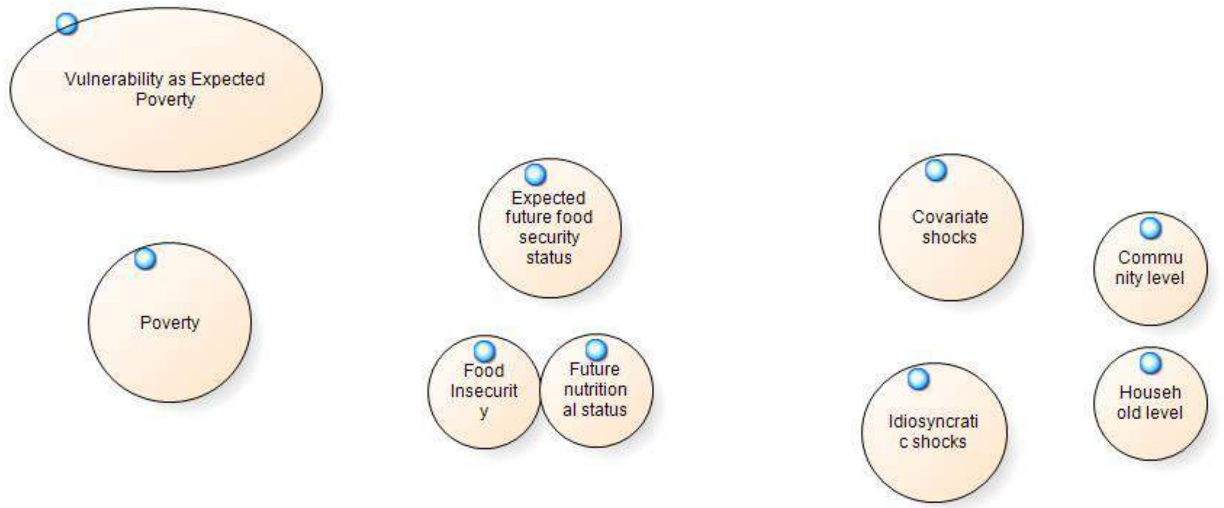

Articles using framework: Calvo & Dercon (2012); Capaldo et al (3020); Chhinh & Poch (2012); Deressa et al (2009); Echevin (2011); Günther & Harttgen (2009); Mutsvangwa (2011); Sarris & Karfakis (2010)

Operationalization of key constructs:

**Operationalization of constructs**		
**Construct operationalized:** Vulnerability as Expected Poverty		
**Source article(s):**		
**Selected by:**	expert selection [justification]	
Sub-constructs	Intermediate constructs	
	Directly operationalized constructs	
Conceptual framework		
Operationalization of sub-constructs		
174 •	Data collection	
	Operational questions	
	Sampling strategies	
	Sample sizes	

		Data analysis	
175	•	Data collection	
		Operational questions	
		Sampling strategies	
		Sample sizes	
		Data analysis	
176	•	Data collection	
		Operational questions	
		Sampling strategies	
		Sample sizes	
		Data analysis	
177	•	Data collection	
		Operational questions	
		Sampling strategies	
		Sample sizes	
		Data analysis	
Candidate-level Analysis			

**Operationalization of constructs**			
**Construct operationalized:** Expected future food security status			
**Source article(s):** Capaldo et al (2010)			
**Selected by:**		default	
Sub-constructs		Intermediate constructs	Present food security status; events
		Directly operationalized constructs	Current socio-economic characteristics; current exposure to risks; Risks; risk management
Conceptual framework		Our model is based on the Social Risk Management approach (Holzmann and Jørgensen 2000; World Bank 2000) and, more specifically, on the conceptual framework drawn from it by Løvendal and Knowles (2005). In this framework vulnerability is the result of a recursive process: current socio-economic characteristics and exposure to risks determine households' future characteristics and their risk-management capacity. At every point in time households' current food security status is affected by their past status and affects their future status. Figure 1 represents graphically this recursive connection.	
Operationalization of sub-constructs			
178	Current socio-economic characteristics	Data collection	We analyze a sample of 1831 rural households from Nicaragua, surveyed in the 2001 Encuesta Nacional de Hogares Sobre Medición de Nivel de Vida, by the Instituto Nacional de Estadísticas y Censos INEC de Nicaragua. Constructed variables used in the analysis were prepared by the Rural Income Generating Activities (RIGA) project team at FAO.
		Operational questions	Information on the structure of a household includes the age of the head of household (which is also a proxy for working experience), gender, marital status, language spoken (as a proxy for households belonging to an indigenous group) and the share of female labor. The latter also approximates labor availability within the household.

				We observed a relatively high proportion of single- or female-headed households (23% and 18% respectively). Household assets are assessed in using education, as well as wealth-related variables (number of rooms, cement floor, telephone, access to safe water, bikes, radios, TV sets owned4), and social capital different<br>through participation of members in community organizations. Moreover, types of livestock and land assets are also taken into account to approximate<br>household wealth and potential credit-related constraints. We use access to a network for migration as a measure of the ability of a household to receive assistance from members living outside the location and as a proxy of a diversified income portfolio. Distance from a road, school, and health facilities, are variables used for measuring a household's access to infrastructure.<br>[...]<br>Table 1: Summary of variables<br>Kilocalories per capita<br>Age of hh head<br>Highest education in hh<br>Single head<br>Female head of hh widow<br>Female headed hh<br>Hh labor<br>Indigenous household<br>Hh size<br>Rooms<br>Cement floor in house<br>Telephone in hh<br>Hh members participating in comm. org.<br>Access to hh migration network<br>Access to safe water<br>Bikes owned<br>Radios owned<br>TVs owned<br>Distance to nearest primary school<br>Time to nearest health facility<br>Distance to nearest major road<br>Land owned<br>Cattle<br>Pigs<br>Horses<br>Land operated<br>Access to irrigation<br>Income from farming activities<br>Income from farm sales	
			Sampling strategies	We analyze a sample of 1831 rural households from Nicaragua, surveyed in the 2001 Encuesta Nacional de Hogares Sobre Medición de Nivel de Vida, by the Instituto Nacional de Estadísticas y Censos INEC de Nicaragua. Constructed variables used in the analysis were prepared by	

			the Rural Income Generating Activities (RIGA) project team at FAO.
		Sample sizes	sample of 1831 rural households
		Data analysis	After accounting for heteroskedasticity through the use of generalized least squares, we estimate vulnerability to food insecurity as the normal probability that the “individual minimum dietary energy requirement under light physical activity” exceeds the expected individual dietary energy consumption (measured in kilocalories). Since the main purpose of this paper is to propose a methodology to analyze and estimate vulnerability, we ignore possible econometric complications that are not directly relevant. However, by all means the results presented here are to be considered preliminary.
179	current exposure to risks	Data collection	Same as previous construct
		Operational questions	We use ex-post data on shocks and risk management strategies. These include information on the incidence of a covariate shock (such as drought) and an idiosyncratic shock (illness)<br>[...]<br>Table 1: Summary of variables<br>Drought shock<br>Illness shock
		Sampling strategies	Same as previous construct
		Sample sizes	Same as previous construct
		Data analysis	Same as previous construct
180	Risks	Data collection	NOT TRANSPARENT
		Operational questions	
		Sampling strategies	
		Sample sizes	
		Data analysis	
181	risk management	Data collection	NOT TRANSPARENT
		Operational questions	
		Sampling strategies	
		Sample sizes	
		Data analysis	
182		Data collection	
		Operational questions	
		Sampling strategies	
		Sample sizes	
		Data analysis	
Candidate-level Analysis		After accounting for heteroskedasticity through the use of generalized least squares, we estimate vulnerability to food insecurity as the normal probability that the “individual minimum dietary energy requirement under light physical activity” exceeds the expected individual dietary energy consumption (measured in kilocalories). Since the main purpose of this paper is to propose a methodology to analyze and estimate vulnerability, we ignore possible econometric complications that are not directly relevant. However, by all means the results presented here are to be considered preliminary.	

**Operationalization of constructs**

**Construct operationalized:** Idiosyncratic shocks			
**Source article(s):**			
**Selected by:**		expert selection [justification]	
Sub-constructs		Intermediate constructs	
		Directly operationalized constructs	
Conceptual framework			
Operationalization of sub-constructs			
183	•	Data collection	
		Operational questions	
		Sampling strategies	
		Sample sizes	
		Data analysis	
184	•	Data collection	
		Operational questions	
		Sampling strategies	
		Sample sizes	
		Data analysis	
185	•	Data collection	
		Operational questions	
		Sampling strategies	
		Sample sizes	
		Data analysis	
186	•	Data collection	
		Operational questions	
		Sampling strategies	
		Sample sizes	
		Data analysis	
Candidate-level Analysis			

**Operationalization of constructs**			
**Construct operationalized:** Covariate shocks			
**Source article(s):**			
**Selected by:**		expert selection [justification]	
Sub-constructs		Intermediate constructs	
		Directly operationalized constructs	
Conceptual framework			
Operationalization of sub-constructs			
187	•	Data collection	
		Operational questions	
		Sampling strategies	

		Sample sizes	
		Data analysis	
188	•	Data collection	
		Operational questions	
		Sampling strategies	
		Sample sizes	
		Data analysis	
189	•	Data collection	
		Operational questions	
		Sampling strategies	
		Sample sizes	
		Data analysis	
190	•	Data collection	
		Operational questions	
		Sampling strategies	
		Sample sizes	
		Data analysis	
Candidate-level Analysis			

**Operationalization of constructs**			
**Construct operationalized:** Household level			
**Source article(s):**			
**Selected by:**		expert selection [justification]	
Sub-constructs		Intermediate constructs	
		Directly operationalized constructs	
Conceptual framework			
Operationalization of sub-constructs			
191	•	Data collection	
		Operational questions	
		Sampling strategies	
		Sample sizes	
		Data analysis	
192	•	Data collection	
		Operational questions	
		Sampling strategies	
		Sample sizes	
		Data analysis	
193	•	Data collection	
		Operational questions	
		Sampling strategies	
		Sample sizes	
		Data analysis	
194	•	Data collection	
		Operational questions	
		Sampling strategies	

		Sample sizes	
		Data analysis	
Candidate-level Analysis			

**Operationalization of constructs**			
**Construct operationalized:** Community level			
**Source article(s):**			
**Selected by:**		expert selection [justification]	
Sub-constructs		Intermediate constructs	
		Directly operationalized constructs	
Conceptual framework			
Operationalization of sub-constructs			
195	•	Data collection	
		Operational questions	
		Sampling strategies	
		Sample sizes	
		Data analysis	
196	•	Data collection	
		Operational questions	
		Sampling strategies	
		Sample sizes	
		Data analysis	
197	•	Data collection	
		Operational questions	
		Sampling strategies	
		Sample sizes	
		Data analysis	
198	•	Data collection	
		Operational questions	
		Sampling strategies	
		Sample sizes	
		Data analysis	
Candidate-level Analysis			

Information relating to further development of framework

Constructs with no adequate operationalizations

- Food insecurity (no operationalizations)
- Future nutritional status (no operationalizations)
- Expected future food security status (partially adequate operaiontalations)

Constructs with more than one adequate operationalizations		
Construct	Preference rank	Summary of operationalization

Appendix P: Questionnaire – candidate operationalizations

Instructions for review:

Each section of this questionnaire is concerned with a construct. Each section gathers together the different ways in which the given construct has been operationalized in the different papers. These are summarised in structured summary tables of candidate operationalizations, which contain information on the sub-constructs used to operationalize the main construct, the relationships between the constructs, data collection methods and operational questions used to generate raw data for each of the sub-constructs, and methods of analysis to draw findings at the level of the main construct.

The question you are asked to respond to is: for each construct, among the candidate operationalizations, choose three which you subjectively consider to be the most useful for CCAFS' study of vulnerability. Fill in your ordered preferences in the 'selection of most useful operationalizations' tables at the top of each section.

If you wish, you can compose a choice by merging elements from different candidates.

Adaptation strategy

Selection of most useful operationalizations (to be completed by expert reviewer)	
**Construct**	**Adaptation strategy**
1st preference	[Author (year)]
2nd preference	[Author (year)]
3rd preference	[Author (year)]

<table>
<tr><td colspan="5">Structured summary of candidate operationalizations</td></tr>
<tr><td colspan="5">Candidate article: Eakin et al (2012)</td></tr>
<tr><td colspan="5">Construct operationalized: Adaptation strategy</td></tr>
<tr><td colspan="2" rowspan="2">Sub-constructs</td><td colspan="2">Intermediate constructs</td><td></td></tr>
<tr><td colspan="2">Directly operationalized constructs</td><td></td></tr>
<tr><td colspan="2">Conceptual framework</td><td colspan="3">DIRECT OPERATIONALIZATION’</td></tr>
<tr><td colspan="2">Operationalization of sub-constructs</td><td colspan="3"></td></tr>
<tr><td rowspan="2">1.</td><td rowspan="2">•</td><td>Data collection</td><td colspan="2">This study is based on 64 household surveys and additional in-depth expert and key-informant interviews, conducted in 2006 and 2007.</td></tr>
<tr><td>Operational questions</td><td colspan="2">The surveys, implemented 18 months following Stan, collected information regarding pre- and post-Hurricane Stan activities and income sources, house- hold demographics, land holdings, production attributes, hurricane impacts (to property, production and health and welfare), household assets before and after Stan and access to agricultural and emergency response services. As described later, the survey also captured households’ per- ceptions and attitudes about the disaster and their suscep- tibility to damage.
[...]
<table><tr><td>Table 2 Household assets in 2005 by impact class</td></tr><tr><td>Land (in ha) in 2005
Percent land in coffee in 2005
Number of plots owned in 2005
Percentage of these plots in coffee in 2005
Coffee production in 2005 (kg)
Coffee yields in 2005 (kg/Ha)
Percentage of land in riverbed
Planted maize (subsistence) in 2005
Percentage of households reporting poultry as very important for livelihood in 2005</td></tr></table>
[...]
<table><tr><td>Table 3 Impacts of Stan by impact cluster</td></tr><tr><td>Coffee harvest loss
Soil loss
Average # of days with difficulty in acquiring basic needs following the hurricane
Percentage of households reporting
Total damages to the house
Loss of coffee production equipment</td></tr></table></td></tr>
</table>

<table>
<tr><td></td><td></td><td></td><td>
<table><tr><td>Impacts to their health due to the hurricane</td></tr></table>

[...]
<table>
<tr><td>Table 4 Income profiles pre- and post-Stan (2005 and 2007)</td></tr>
<tr><td>Percentage of household who received income from</td></tr>
<tr><td>Coffee
Other crops and/or cattle
Agricultural wage laborer
Non-farm activities
Subsidies, pensions or other governmental support
Remittances
Number of income sources</td></tr>
</table>

[...]
<table>
<tr><td>Table 5 Post-Stan actions by impact cluster</td></tr>
<tr><td>Bought or rented new land
New land for subsistence crops (maize and/or beans)
Invested in soil conservation
Shifted efforts to a new job
Invested in hurricane protection
Planted a new crop
Planted shade trees</td></tr>
</table>
[...]
Fig. 3 Recovery time for households and the community following Stan (Household was specifically asked: ‘‘How much time do you feel is necessary for your household [community] to fully recover from Stan?’’ Source: Authors’ household survey)
</td></tr>
<tr><td colspan="2">Candidate-level Analysis</td><td colspan="2">As a heuristic tool to aid in our interpretation of impacts
and responses to Stan, we categorized households accord- ing to the exposure of their production systems to Hurri- cane Stan into impact clusters. The impact clusters were created using a two-step cluster method available through the statistical software, PASW 18. Two-step cluster anal- ysis uses a distance criterion (log-likelihood) to define optimal number of clusters and allows for handling a mixture of categorical and (standardized) continuous vari- ables (Zhang et al. 1996; Chiu et al. 2001).
[...]
We used two ‘‘loss’’ variables as the input data for the
creation of clusters: percent of coffee harvest and soil lost due to Hurricane Stan. We chose these two variables because of the fundamental economic role played by coffee production for households in Siltepec in 2005.
[...]
We then used these clusters to explore two questions through a descriptive analysis of the remaining survey variables: What were the characteristics of house- holds that experienced specific degrees of loss? What were their responses?</td></tr>
</table>

<table>
<tr><td colspan="3">Structured summary of candidate operationalizations</td></tr>
<tr><td colspan="3">Candidate article: Westerhoff & Smit</td></tr>
<tr><td colspan="3">Construct operationalized: Adaptation strategy</td></tr>
<tr><td>Sub-constructs</td><td>Intermediate constructs</td><td></td></tr>
</table>

		Directly operationalized constructs	
Conceptual framework		DIRECT OPERATIONALIZATION’	
Operationalization of sub-constructs			
1.	•	Data collection	These were determined using a community-based approach similar to those used by Burton et al. (2002), Ford and Smit (2004) and Schröter et al. (2005), in which the factors and forces relevant to the community vulnerability were sought via primary and secondary sources.<br>[...]<br>Most of the primary data were derived from 22 semi-structured, in-depth interviews with 11 male and 11 female community members,<br>[...]<br>These community-member interviews were complemented by an additional 22 in-depth interviews with key informants from various governmental and non-governmental institutions in the area<br>[...]<br>In addition, five focus groups were conducted with members of the community,<br>[...]<br>The community-based data collection was complemented by a review of documents and records to extract information on the biophysical and socioeconomic forces contributing to vulnerability. Documents comprised existing studies completed in the area, government reports, climate data, and all other pertinent information.
		Operational questions	22 semi-structured, in-depth interviews with 11 male and 11 female community members, who were asked to describe and explain in local terminology the various exposure-sensitivities, adaptations and adaptive capacities of importance to them.<br>[...]<br>interviews with key informants from various governmental and non-governmental institutions in the area for the purposes of obtaining further information on the relevant contributing biophysical/socioeconomic forces, exposure-sensitivities, adaptations, and adaptive capac- ities in Mimkyemfre.<br>[...]<br>five focus groups were conducted with members of the community, through<br>which data on the experience of vulnerability by residents engaged in primary livelihood activities were gathered. Information on methods of farming, charcoal production and fishing, the stresses on these livelihoods, and the means for overcoming these stresses was compiled. Focus groups were used to investigate interactions between community members and other aspects of vulnerability that became evident in the group dynamic, an effect often referred to as ‘synergism’ (Morgan 1996).

Candidate-level Analysis	Data from these multiple sources were coded, sorted and analyzed (in part using qualitative data analysis software) according to the themes of the conceptual model of vulnerability in order to identify relevant forces, exposure-sensitivities, adaptations and adaptive capacities experienced at the individual, household and community levels.

Adaptive capacity

Selection of most useful operationalizations (to be completed by expert reviewer)	
**Construct**	**Adaptive capacity**
1st preference	[Author (year)]
2nd preference	[Author (year)]
3rd preference	[Author (year)]

Structured summary of candidate operationalizations			
**Candidate article:** Antwi-Agyei et al			
**Construct operationalized:** Adaptive capacity			
Sub-constructs		Intermediate constructs	Livelihoods; Livelihood capital assets
		Directly operationalized constructs	Social capital; financial capital; natural capital; physical capital; human capital
Conceptual framework		Traditionally, the SLA has been applied by considering the five livelihood capital assets—human, financial, natural, physical and social—as well as their links to an overall vulnerability context, processes, institutions (both formal and informal) and policies that govern people's access to these capital assets (Scoones 1998).	
Operationalization of sub-constructs			
1.	Social capital	Data collection	not valid/feasible
		Operational questions	
2.	Financial capital	Data collection	Data presented in this paper were collected using a mixture of participatory methods such as focus group discussions, household questionnaire surveys and key informant interviews. Data collection started with a rapid rural appraisal (Chambers 1994) during which com- munity gatherings and transect walks were conducted with community members including opinion leaders at each of the 6 villages. This provided an overview of the significant social and physical features of the selected communities that influenced their livelihood activities (Sallu et al. 2009). A household questionnaire survey was used to collect both qualitative and quantitative data. The questionnaire survey assessed households' capital assets (financial, human, natural, physical, and social). This information was used to develop a household livelihood vulnerability index (see Sect. 2.3). A total of 270 household ques- tionnaire surveys were conducted in the 6 farming communities (45 Questionnaires in each).
		Operational questions	Eliciting information on financial assets was very problematic because of a lack of records on sales and memory lapses. Livestock were considered to offer readily available cash in times of crop failure due to erratic rainfall patterns in the study communities.<br>[...]<br>Households without poultry or livestock scored 1 whilst those with livestock scored 2. In addition, financial assets were assessed by examining the remittances received by the household from family members or friends over the past 12

			months.<br>[...]<br>Households that received remittances in the last 12 months scored 2 and those that did not receive any remittances scored 1. Access to credit may also influence adaptation to climate change including access to inputs such as improved cultivars of crops (e.g. Butt et al. 2006; Fosu-Mensah et al. 2012). Hence, it is assumed that households that have no access to credit will be more vulnerable and scored 1 whilst those with access to credit were given a score of 2.<br>[...]<br>Table 1 Indicators of household livelihood vulnerability index collected through a household survey across six communities in Ghana<br>[...]<br>Access to credit<br>Do you have access to credit for your agricultural activities?<br>Ownership of livestock<br>Do you have livestock or poultry? List the types and numbers of livestock.<br>Remittances received<br>Have you received remittances from family or friends in the last 12 months?
3.	Natural capital	Data collection	same as for 'financial capital'
		Operational questions	Natural capital assets were assessed by two indicators. The first was the size of the farm holding under cultivation (this was estimated as the average area of cultivated land over the past 5 years) (Table 1). It is assumed that the larger the farm holding, the greater the opportunity for the household to have more crops and yield, and hence the lower the vulnerability to climate change, though it is noted that labour availability and financial capital both affect the reality of how much land can be cultivated. Households which cultivated less than 5 acres scored 1; those cultivating between 5 and 10 acres scored 2; those cultivating between 11 and 15 acres scored 3; those cultivating 16-20 acres scored 4, and households cultivating [20 acres scored 5. The type of land tenure and level of security it provides may have serious implications for the management of agricultural soils and could indirectly affect crop productivity and environmental sustainability, conse- quently influencing household vulnerability (Butt et al. 2006). Three different tenure arrangements were identified in the study communities. These were ''land inherited'', ''land purchased'' and ''land rented'' by the household. A score of 1 was given to households who rented their farmlands; 2 for households who purchased their farmlands; and 3 for those who inherited their farmlands. Households that inherited their farm lands were given the highest score because it is assumed that they will have the most secure land tenure.<br>[...]<br>Table 1 Indicators of household livelihood vulnerability index collected through a household survey across six communities

			in Ghana<br>[...]<br>Farm holding size<br>Could you please state the size of farm holding in acres?<br>Tenure system<br>By what arrangements do you have access to your farm land for farming activities?
4.	Physical capital	Data collection	same as for ‘financial capital’
		Operational questions	Physical assets that were assessed included the presence of irrigation facilities and own- ership of radios, television or mobile phones by a household (Table 1). Irrigation facilities are crucial for rain-fed agriculture-dependent households, as these facilities help farmers to practise dry season farming. It is assumed that households with irrigation facilities will be less vulnerable to changing rainfall patterns. Hence, households without irrigation facilities scored 1, whilst those with these facilities scored 2. The presence of radios, television or mobile phone in a rural household can be an effective tool for communication and accessing information on changing weather patterns (see Naab and Koranteng 2012). Here, households with any of these three assets scored 2, and those without any scored 1. Physical assets such as road networks and the availability of markets and health facilities may enhance the adaptive capacity of a household (see Zhang et al. 2007). These assets were not included in the vulnerability computation because field observations suggested that these physical assets did not significantly differ amongst either the resilient or vul- nerable communities.<br>[...]<br>Irrigation facilities<br>Do you have access to irrigation facilities for dry season farming?<br>Ownership of radio, television or mobile phone<br>Could you please list all communication gadgets that you have? These include TV, mobile phone or radios etc.
5.	Human capital	Data collection	same as for ‘financial capital’
		Operational questions	Human capital assets were represented by two indicators: the educational level of the head of the household (or the most educated person in the household) and the health status of the household (Table 1). No formal education was afforded a value of 1; 2 in the case of only primary education; 3 in the case of secondary education; and 4 for households that had tertiary education.<br>[...]<br>To assess health status, households were asked about the number of times they have been to the hospital (or hospitalised) within the last 12 months. House- holds with members that had been to the hospital were scored 1 whilst those with members that had not been to hospital as out patients (and those not needing any medical attention) within this period were scored 2. Also, situations where members of a household required hospital treatment but could not

			arrange transport and other resources needed were taken into consideration when scoring such a household.<br>[...]<br>Table 1 Indicators of household livelihood vulnerability index collected through a household survey across six communities in Ghana<br>[...]<br>Educational level<br>Could you please state the highest education attained?<br>Health status<br>Have any member of this household been ill in the last 12 months?
Candidate-level Analysis		To ensure the comparability of indicators that were used in the construction of the household livelihood vulnerability index, all indicators were standardised following the UNDP (2007) procedure of standardising indicators for life expectancy index (Eq. 1).<br>[...]<br>Having standardised the indicators, it was then necessary to elicit appropriate weights to them. An unequal weighting system, based on relative importance attached to each indicator by local households, extension officers, key informants and experts was used because it was deemed necessary to include the views of both local households and experts in the assessment. Hence, a five-point Likert scale was used where farmers, extension officers, key informants, and experts were asked to rank the five most important indicators that they considered to influence vulnerability at the household level (Table 2). The number of times a particular indicator was cited was used to generate the weighting system (Table 2). The following weights were assigned: 14 % to social capital, 11 % to human capital, 9 % to natural capital, 27 % to financial capital, 10 % to physical capital and 29 % to livelihood diversification (Table 2). The household livelihood vulnerability index for a household was then calculated using the following model (Eq. 2) (Vincent 2004).<br>HLVI = (Ssvi X Wi) + (Hsvi X Wii) + (Nsvi X Wiii) + (Fsvi X Wiv) + (Psvi X Wv) + (Lsvi X Wvi)<br>where HLVI = household livelihood vulnerability index, Ssvi = standardised value of social capital asset sub-index, Hsvi = standardised value of human capital asset sub-index, Nsvi = standardised value of natural capital asset sub-index, Fsvi = standardised value of financial capital asset sub-index, Psvi = standardised value of physical capital asset sub-index, and Lsvi = standardised value of livelihood diversification sub-index. The Wi terms refer to the weighting that was applied to each standardised value: Wi = 0.14, Wii = 0.11, Wiii = 0.09, Wiv = 0.27, Wv = 0.10, and Wvi = 0.29<br>[...]<br>Quanti- tative data were transcribed and analysed using SPSS and Minitab (Edition 15). Using Minitab, a one-way ANOVA was computed to compare the relative vulnerability among the various households and communities, and all differences resulting in p\0.05 were considered statistically significant. K-means cluster analysis using STATISTICA software was undertaken to group the households according to their vulnerability. K-means cluster analysis, which seeks to group cases into distinct clusters by seeking groups that minimise variability within clusters and maximise variability between clusters (Levia and Page 2000), has been applied to spatial vulnerability assessment in dynamic systems (see Antwi- Agyei et al. 2012).	

**Structured summary of candidate operationalizations**
**Candidate article:** Hahn et al

<table>
<tr><td colspan="4">Construct operationalized: Adaptive capacity</td></tr>
<tr><td colspan="2" rowspan="2">Sub-constructs</td><td>Intermediate constructs</td><td>Socio-demographic profile; livelihood strategies; social network</td></tr>
<tr><td>Directly operationalized constructs</td><td>Households working elsewhere; agriculture dependent household; livelihood diversification; Dependency ratio; percent of female headed households; households with orphans; uneducated headed households; Receive-give ratio; borrow-lend ratio; independent of local government</td></tr>
<tr><td colspan="2">Conceptual framework</td><td colspan="2">Adaptive capacity is quantified by the demographic profile of a district (e.g., percent of female-headed households), the types of livelihood strategies employed (e.g., predominately agricultural, or also collect natural resources to sell in the market), and the strength of social networks (e.g., percent of residents assisting neighbors with chores).</td></tr>
<tr><td colspan="2">Operationalization of sub-constructs</td><td colspan="2"></td></tr>
<tr><td rowspan="2">1.</td><td rowspan="2">Households working elsewhere</td><td>Data collection</td><td>household surveys</td></tr>
<tr><td>Operational questions</td><td>Table 1 includes an explanation of how each sub-component was quantified, the survey question used to collect the data, the original source of the survey question, and potential sources of bias.
[...]
<table><tr><td colspan="3">Table 1 Major components and sub-components comprising the Livelihood Vulnerability Index (LVI) developed for two districts of Mozambique.</td></tr><tr><td>Sub-components</td><td>Explanation of sub-components</td><td>Survey question</td></tr><tr><td>Percent of households with family member working in a different community</td><td>Percentage of households that report at least 1 family member who works outside of the community for their primary work activity.</td><td>How many people in your family go to a different community to work?</td></tr></table></td></tr>
<tr><td rowspan="2">2.</td><td rowspan="2">agriculture dependent household</td><td>Data collection</td><td>household surveys</td></tr>
<tr><td>Operational questions</td><td>Table 1 includes an explanation of how each sub-component was quantified, the survey question used to collect the data, the original source of the survey question, and potential sources of bias.
[...]</td></tr>
</table>

<table>
<tr><td></td><td></td><td></td><td>
<table>
<tr><td colspan="3">Table 1 Major components and sub-components comprising the Livelihood Vulnerability Index (LVI) developed for two districts of Mozambique.</td></tr>
<tr><td>Sub-components</td><td>Explanation of sub-components</td><td>Survey question</td></tr>
<tr><td>Percent of households dependent solely on agriculture as a source of income</td><td>Percentage of households that report only agriculture as a source of income.</td><td>Do you or someone else in your household raise animals? Do you or someone else in your household grow crops? Do you or someone else in your household collect something from the bush, the forest, or lakes and rivers to sell?</td></tr>
</table>
</td></tr>
<tr><td rowspan="2">3.</td><td rowspan="2">livelihood diversification</td><td>Data collection</td><td>household surveys</td></tr>
<tr><td>Operational questions</td><td>Table 1 includes an explanation of how each sub-component was quantified, the survey question used to collect the data, the original source of the survey question, and potential sources of bias.
[...]
<table>
<tr><td colspan="3">Table 1 Major components and sub-components comprising the Livelihood Vulnerability Index (LVI) developed for two districts of Mozambique.</td></tr>
<tr><td>Sub-components</td><td>Explanation of sub-components</td><td>Survey question</td></tr>
<tr><td>Average Agricultural Livelihood Diversification Index (range: 0.20–1)a</td><td>The inverse of (the number of agricultural livelihood activities +1) reported by a</td><td>Do you or someone else in your household raise</td></tr>
</table>
</td></tr>
</table>

<table>
<tr><td></td><td></td><td></td><td><table><tr><td></td><td>household, e.g., A household that farms, raises animals, and collects natural resources will have a Livelihood Diversification Index = 1/(3 + 1) = 0.25.</td><td>animals? Do you or someone else in your household grow crops? Do you or someone else in your household collect something from the bush, the forest, or lakes and rivers to sell?</td></tr></table></td></tr>
<tr><td rowspan="2">4.</td><td rowspan="2">Dependency ratio</td><td>Data collection</td><td>household surveys</td></tr>
<tr><td>Operational questions</td><td>Table 1 includes an explanation of how each sub-component was quantified, the survey question used to collect the data, the original source of the survey question, and potential sources of bias.
[...]
<table><tr><td colspan="3">Table 1 Major components and sub-components comprising the Livelihood Vulnerability Index (LVI) developed for two districts of Mozambique.</td></tr><tr><td>Sub-components</td><td>Explanation of sub-components</td><td>Survey question</td></tr><tr><td>Dependency ratio</td><td>Ratio of the population under 15 and over 65 years of age to the population between 19 and 64 years of age.</td><td>Could you please list the ages and sexes of every person who eats and sleeps in this house? If you had a visitor who ate and slept here for the last 3 days, please</td></tr></table></td></tr>
</table>

<table>
<tr><td></td><td></td><td></td><td><table><tr><td></td><td></td><td>include them as well.</td></tr></table></td></tr>
<tr><td rowspan="2"></td><td rowspan="2">percent of female headed households</td><td>Data collection</td><td>household surveys</td></tr>
<tr><td>Operational questions</td><td>Table 1 includes an explanation of how each sub-component was quantified, the survey question used to collect the data, the original source of the survey question, and potential sources of bias.
[...]
<table><tr><td colspan="3">Table 1 Major components and sub-components comprising the Livelihood Vulnerability Index (LVI) developed for two districts of Mozambique.</td></tr><tr><td>Sub-components</td><td>Explanation of sub-components</td><td>Survey question</td></tr><tr><td>Percent of female-headed households</td><td>Percentage of households where the primary adult is female. If a male head is away from the home >6 months per year the female is counted as the head of the household.</td><td>Are you the head of the household?</td></tr></table></td></tr>
<tr><td rowspan="2"></td><td rowspan="2">households with orphans</td><td>Data collection</td><td>household surveys</td></tr>
<tr><td>Operational questions</td><td>Table 1 includes an explanation of how each sub-component was quantified, the survey question used to collect the data, the original source of the survey question, and potential sources of bias.
[...]
<table><tr><td colspan="3">Table 1 Major components and sub-components comprising the Livelihood Vulnerability Index (LVI) developed for two districts of Mozambique.</td></tr><tr><td>Sub-components</td><td>Explanation of sub-components</td><td>Survey question</td></tr><tr><td>Percent of households with orphans</td><td>Percentage of households that have at</td><td>Are there any children</td></tr></table></td></tr>
</table>

<table>
<tr><td></td><td></td><td></td><td><table><tr><td></td><td>least 1 orphan living in their home. Orphans are children<18 years old who have lost one or both parents.</td><td>less than 18 years old from other families living in your house because one or both of their parents has died?</td></tr></table></td></tr>
<tr><td rowspan="2"></td><td rowspan="2">uneducated headed households</td><td>Data collection</td><td>Not valid/feasible</td></tr>
<tr><td>Operational questions</td><td></td></tr>
<tr><td rowspan="2"></td><td rowspan="2">Receive-give ratio</td><td>Data collection</td><td>household surveys</td></tr>
<tr><td>Operational questions</td><td>Table 1 includes an explanation of how each sub-component was quantified, the survey question used to collect the data, the original source of the survey question, and potential sources of bias.
[...]

<table><tr><td colspan="3">Table 1 Major components and sub-components comprising the Livelihood Vulnerability Index (LVI) developed for two districts of Mozambique.</td></tr><tr><td>Sub-components</td><td>Explanation of sub-components</td><td>Survey question</td></tr><tr><td>Average Receive:Give ratio (range: 0–15)</td><td>Ratio of (the number of types of help received by a household in the past month + 1) to (the number of types of help given by a household to someone else in the past month + 1).</td><td>In the past month, did relatives or friends help you and your family: (e.g., Get medical care or medicines, Sell animal products or other goods produced by family, Take care of children) In the past month, did you and your family help relatives or</td></tr></table></td></tr>
</table>

<table>
<tr><td></td><td></td><td></td><td><table><tr><td></td><td></td><td>friends: (same choices as above)</td></tr></table></td></tr>
<tr><td rowspan="2"></td><td rowspan="2">borrow-lend ratio</td><td>Data collection</td><td>household surveys</td></tr>
<tr><td>Operational questions</td><td>Table 1 includes an explanation of how each sub-component was quantified, the survey question used to collect the data, the original source of the survey question, and potential sources of bias.
[...]
<table><tr><td colspan="3">Table 1 Major components and sub-components comprising the Livelihood Vulnerability Index (LVI) developed for two districts of Mozambique.</td></tr><tr><td>Sub-components</td><td>Explanation of sub-components</td><td>Survey question</td></tr><tr><td>Average Borrow:Lend Money ratio (range: 0.5–2)</td><td>Ratio of a household borrowing money in the past month to a household lending money in the past month, e.g., If a household borrowed money but did not lend money, the ratio = 2:1 or 2 and if they lent money but did not borrow any, the ratio = 1:2 or 0.5.</td><td>Did you borrow any money from relatives or friends in the past month? Did you lend any money to relatives or friends in the past month?</td></tr></table></td></tr>
<tr><td rowspan="2"></td><td rowspan="2">independent of local government</td><td>Data collection</td><td>household surveys</td></tr>
<tr><td>Operational questions</td><td>Table 1 includes an explanation of how each sub-component was quantified, the survey question used to collect the data, the original source of the survey question, and potential sources of bias.
[...]
<table><tr><td>Table 1 Major components and sub-components comprising the Livelihood Vulnerability Index (LVI) developed for two</td></tr></table></td></tr>
</table>

			districts of Mozambique.		
			Sub-components	*Explanation of sub-components*	*Survey question*
			Percent of households that have not gone to their local government for assistance in the past 12 months	Percentage of households that reported that they have not asked their local government for any assistance in the past 12 months.	In the past 12 months, have you or someone in your family gone to your community leader for help?
Candidate-level Analysis		Rather than merge the major components into the LVI in one step, they are first combined according to the categorization scheme in Table 2 using the following equation:<br>$CF_d = [\sum^n_{i=1} w_{Mj} M_{di}]/[\sum^n_{i=1} w_{Mi}]$<br>where CFd is an IPCC-defined contributing factor (exposure, sensitivity, or adaptive capacity) for district d, Mdi are the major components for district d indexed by i, wMi is the weight of each major component, and n is the number of major components in each contributing factor.			

**Structured summary of candidate operationalizations**			
**Candidate article:** Piya et al			
**Construct operationalized:** Adaptive capacity			
Sub-constructs		Intermediate constructs	Livelihood assets
		Directly operationalized constructs	Physical capital; human capital; natural capital; financial capital; social capital
Conceptual framework		adaptive capacity of a household is taken to be an emergent property of the five types of livelihood assets viz. physical, human, natural, financial, and social	
Operationalization of sub-constructs			
5.	Physical capital	Data collection	This study is based on the primary data collected by household survey conducted in<br>two phases. The first phase of household survey was conducted in February-March 2010 and the second phase in May-June 2011.
		Operational questions	Indicators for the physical assets are type of house, ownership of devices to access<br>information (mobile phone and radio), walking distance to the nearest road, and irrigated land. Out of these, only house quality and irrigation are directly related to climate risks. Possession of better quality house will improve the capacity to withstand the

<table>
<tr><td></td><td></td><td></td><td>risks from extreme climate events. Type of house was indicated from a value of 1-3, 3 indicating the most durable type of house (see Table 3). Ownership of mobile phone and radio will increase the adaptive capacity through access to weather related information. Better access to information enables a household in planning proactive adaptation measures against climate risks. Walking distance to the nearest motor road, which in this case is also equivalent to the nearest marketplace, is assumed to be inversely related to adaptive capacity as household located far away from the markets will be in a disadvantageous position for lacking the opportunity of income generation from alternative sources like non-farm labor, which help in securing livelihoods during the periods of food shortage or crop failure. Farther distance from the roads also symbolizes poor access to inputs as the service centers are located at the road-heads. In addition, greater distance from the motor roads also means limited access to information as the marketplace acts as informal gathering centers where information exchange takes place, and also the formal institutions providing extension services are located there. Irrigation is directly related to climate shocks as it minimizes risks posed by droughts. Higher percentage of irrigated land means lesser dependence on natural rain for agricultural purposes, which is becoming more unpredictable with climate change.

<table>
<tr><th>Component Indicators</th><th>Description of the Indicators</th><th>Unit</th><th>Hypothesized relation</th></tr>
<tr><td>Physical Assets</td><td>Type of house (1 = thatch roof, thatch/wooden wall; 2 = thatch roof, stone+mud wall; 3 = stone/tin/tile roof, stone/wood/brick+mud wall)</td><td>Ordinal value</td><td>+</td></tr>
<tr><td></td><td>Have devices to access information (mobile, radio) (0 = No, 1 = Yes)</td><td>Ordinal value</td><td>+</td></tr>
<tr><td></td><td>Walking distance to nearest motor road</td><td>Hours</td><td>+</td></tr>
<tr><td></td><td>Irrigated land</td><td>% of total</td><td>-</td></tr>
</table>
</td></tr>
<tr><td>6.</td><td>human capital</td><td>Data collection</td><td>Same as ‘physical capital’</td></tr>
<tr><td></td><td></td><td>Operational questions</td><td>Human asset is represented by highest qualification in the family; trainings or
vocational courses attended by the family members; and dependency ratio. These indicators are not directly related climate shocks; however they are still relevant because development of human capabilities through vocational trainings or formal education enable households to increase their income by undertaking skilled non-farm</td></tr>
</table>

			activities, which are less climate- sensitive compared to farming and gathering, thereby helping the households to avert climate risks. Furthermore, it also diversifies household livelihood sources which help to buffer the risks posed by climate on farm income. Households with higher dependency ratio will have more burdens on the earning members thereby reducing the adaptive capacity. The implication of dependency ratio is common to any types of shocks including climate.
7.	natural capital	Data collection	Same as ‘physical capital
		Operational questions	The quality of land possessed by the households is taken as an indicator of natural assets. Chepangs possess three categories of land. Paddyland (khet) is the most productive category of land, usually having an irrigation source. Bari is terraced upland, which may or may not be irrigated, and is less productive than khet, but more productive than the third category, khoriya, which is unterraced sloppy land-plot. Natural assets, by their own nature, are more vulnerable to climate shocks than other types of assets. While terraced land types (khet and bari) are less prone to erosion, khoriya face greater risks of landslides and loss of top-soil due to run-off during rains. Households possessing higher share of khet and bari compared to khoriya will suffer less from climate disasters. Higher share of more productive land (khet and bari) also means higher food self-sufficiency, thus higher adaptive capacity. Higher share of khoriya indicates the opposite. Besides land, possession of bullock, which is the only means of ploughing fields in the hills, is another indicator of household natural assets.

Component Indicators	Description of the Indicators	Unit	Hypothesized relation
Human Assets	Highest qualification in the family	Number of schooling years	+
	Dependency Ratio	-	+
	Trainings or vocational course attended by family members	Number	-

Component Indicators	Description of the Indicators	Unit	Hypothesized relation
Natural	Share of more	% of	+

<table>
<tr><td></td><td></td><td></td><td><table>
<tr><td>Assets</td><td>productive land (khet + bari) possessed</td><td>total</td><td></td></tr>
<tr><td></td><td>Share of less productive land (khoriya) possessed</td><td>% of total</td><td>+</td></tr>
<tr><td></td><td>Have bullock (0 = No, 1 = Yes)</td><td>Ordinal</td><td>+</td></tr>
</table></td></tr>
<tr><td>8.</td><td>financial capital</td><td>Data collection</td><td>Same as ‘physical capital’</td></tr>
<tr><td></td><td></td><td>Operational questions</td><td>Gross household annual income, livelihood diversification index, household savings,
and ownership of small livestock (goat, poultry, and pig) are taken as the indicators of financial assets. These indicators of financial assets are not specific to climate shocks only. Gross annual income of the household is the sum total of the cash and non-cash income from 11 different sources shown in Appendix 2. Higher income means greater availability of resources at disposal to maximize positive livelihood outcomes. Besides the amount of annual income, the sources from which the income is derived also need to be considered. If all of the income is derived from farming alone, then such income will be adversely affected during the years of bad weather. On the other hand, if the income is derived from more than one source, then risk will be distributed among the sources. In order to capture this aspect of income, Livelihood Diversification Index (LDI) is calculated; higher diversification indicating better ability of the household to switch among the activities when needed. Herfindahl index of diversification is used (Kimenju & Tschirley, 2009), which is calculated as
$Dk = 1 - \sum_{i=1..N} (S_{i,k})^2$
where, Dk is the diversification index, i is the specific livelihood activity, N is the total number of activities being considered, k is the particular household, and Si,k is the share of ith activity to the total household income for kth household (see Appendix 2). In addition to income at disposal, households which are able to make some savings out of their income will be able to make productive investments like family education or use the savings as buffer during the times of need. For Chepangs, small livestock are also important sources of cash income; they keep these livestock as buffer to sell during the times of stress or to pay back the loan that they take from moneylenders.

<table>
<tr><td>Component Indicators</td><td>Description of the Indicators</td><td>Unit</td><td>Hypothesized relation</td></tr>
<tr><td>Financial Assets</td><td>Gross household</td><td>NRs</td><td>+</td></tr>
</table></td></tr>
</table>

<table>
<tr><td></td><td></td><td></td><td>
<table>
<tr><td></td><td>annual income</td><td></td><td></td></tr>
<tr><td></td><td>Livelihood Diversification Index</td><td>-</td><td>+</td></tr>
<tr><td></td><td>Total household savings</td><td>NRs</td><td>+</td></tr>
<tr><td></td><td>Ownership of goat, poultry, and pig</td><td>LSU</td><td>+</td></tr>
<tr><td></td><td>Memberships in CBOs</td><td>Number</td><td>+</td></tr>
</table>
</td></tr>
<tr><td rowspan="2"></td><td rowspan="2">social capital</td><td>Data collection</td><td>Same as ‘physical capital’</td></tr>
<tr><td>Operational questions</td><td>Finally, social asset is represented by the number of membership in formal community based organizations (CBOs) and access to credit. Membership in CBOs will improve the households’ social networks and access to information through their constant contact with the outsiders during the meetings in CBOs. Also, management of resources like water collection tanks and forests is done jointly by the members of these CBOs. Such activities help in pooling risks across the households in a community. Access to credit is also taken as social assets because for the Chepangs, taking loans from social contacts is one of the most important strategies to cope with seasonal food shortages, which they repay by selling agricultural produce, livestock, or forest products. Thus, access to credits in this community is equivalent to the social safety nets against all types of shocks. Also, some semi-formal saving and credit organizations in the community have recently started providing interest-free loans for productive investment like vegetable farming, and rearing cattle. Thus, access to productive loans denotes the access of the households to existing credit providing organizations in the locality. Better the access to credit, higher will be the adaptive capacity of the households.
<table>
<tr><th>Component Indicators</th><th>Description of the Indicators</th><th>Unit</th><th>Hypothesized relation</th></tr>
<tr><td>Social Assets</td><td>Memberships in CBOs</td><td>Number</td><td>+</td></tr>
<tr><td></td><td>Access to credit (1 = needed, but no access; 2 = credit used only for subsistence purposes; 3 = credit used for productive investment +/- subsistence; 4 = no need)</td><td>Ordinal Value</td><td>+</td></tr>
</table>
</td></tr>
<tr><td colspan="2">Candidate-level</td><td colspan="2">Having chosen the suitable indicators, now these need to be normalized so as to bring</td></tr>
</table>

Analysis	the values of the indicators within the comparable range (Nelson, et al., 2010b; Gbetibouo & Ringler, 2009; Vincent, 2004). Normalization is done by subtracting the mean from the observed value and dividing by the standard deviation for each indicator.<br>$Normalized\ value\ = Observed\ Value\ /\ Mean\ standard\ deviation$<br>Next, weights should be assigned to these indicators.<br>[...]<br>Assigning weight by Principal Component Analysis (PCA) following Filmer and Pritchett (2001) is thus preferred compared to the former two methods (Nelson et al., 2010b; Gbetibouo & Ringler, 2009; Cutter, Boruff, & Shirley, 2003). PCA was run for the selected indicators of exposure, sensitivity, and adaptive capacity separately in Data Analysis and Statistical Software (STATA10) software for assigning the weights. The loadings from the first component of PCA are used as the weights for the indicators. The weights assigned for each indicator varies between -1 and +1, sign of the indicators denoting the direction of relationship with other indicators used to construct the respective index. The magnitude of the weights describes the contribution of each indicator to the value of the index. PCA was run separately for the indicators of exposure, sensitivity and adaptive capacity. Stepwise PCA was run for the indicators of adaptive capacity. The first-step PCA was run for the indicators of each asset group separately to observe the relative importance of indicators within each asset category. From the weights obtained from first-step PCA, individual index values for each asset type was calculated. Second-step PCA was run using the index values for each of the five asset types to analyze which asset group contributes the most to the total adaptive capacity. Overall adaptive capacity index was calculated using the weights (loadings) obtained from the second step PCA run for the five asset categories. The normalized variables are then multiplied with the assigned weights to construct the indices (for exposure, sensitivity, and adaptive capacity separately) using the following formulae:<br>$Ij = \sum^{k}_{i=1} b_i[(a_{ji} - x_i)/s_i]$<br>where, 'I' is the respective index value, 'b' is the loadings from first component from PCA (PCA1) taken as weights for respective indicators, 'a' is the indicator value, 'x' is the mean indicator value, and 's' is the standard deviation of the indicators. Finally, vulnerability index for each household is calculated as: V = E + S – AC, where, V is the vulnerability index, E the exposure index, S is the sensitivity index and AC is the adaptive capacity index for respective household.

**Structured summary of candidate operationalizations**			
**Candidate article:** Sietze et al			
**Construct operationalized:** Adaptive Capacity			
Sub-constructs	Intermediate constructs		
	Directly operationalized constructs		
Conceptual framework	DIRECTLY OPERATIONALIZED		
Operationalization of sub-constructs			
2. •	Data collection	The ALTAGRO (2006) data base contains detailed quantitative information for 527 smallholder households collected through household questionnaires.	
	Operational questions	Ten categories describe the smallholder households covering personal information about the family members (e.g. occupation, education level, age), production systems (e.g. crop and livestock assets, labour input, processing and commer- cialisation of produce), weather conditions, food	

reserves, income, some expenses and credits.
[...]
The following data are taken from the ALTAGRO (2006) data base to indicate the mechanisms relevant in this study. As the first dimension, the harvest failure risk is indicated by the number of production zones used for crop and pasture cultivation. The indicator considers plains, hillsides and hills. The second dimension of the area constraint is measured by the crop area as an important prerequisite for food production. The pasture area highly correlates to livestock keeping and is therefore reflected in the livestock measure. The third dimension, the livestock constraint, is characterised by the number and types of animals. To compare various animal species, we calculated standardised livestock units in relation to an improved cattle variety based on the livestock-specific metabolism (Kleiber 1961). Average livestock weights were estimated using 20 representative animals of each species in the study region. Since fodder production is an essential condition for livestock keeping, the respective indicator contains a reference to the area and productivity of pasture land. Furthermore, the productivity constraint as the fourth dimension is provided for the major food crops potatoes and quinua. It averages the household's productivity across species, varieties and production zones for each crop. Again, we concentrate on food crops since the productivity of pastures is already included in the livestock measure. The fifth dimension of education deprivation relates to the number of years that a household head attended school. School attendance is classified according to the four levels: no formal education, primary, secondary and higher edu- cation. Finally, the lack of alternative income as the sixth dimension is quantified by the sum of annual monetary income from local off-farm activities and remittances. People usually receive remittances from household
495
members who migrate for climate-independent labour, for example mining and commerce. Table 1 summarises the indicators used to assess vulnerability.
[...]

Table 1 Indicators of households' sensitivity and adaptive capacity. The range of the area and livestock constraints as well as lack of alternative income is provided following winsorisation, see description in text. (Data source: ALTAGRO 2006)

Dimension of sensitivity and adaptive capacity	*Indicator*	*Range*
Harvest failure risk	Number of production zones used for	1–3

	cultivation	
Area constraint	Crop area	0.1–1.3 ha/person[a]
Livestock constraint	Livestock units	0.1–8.0 livestock units/person
Productivity constraint	Potato productivity<br>Quinua productivity	0.1–10.0 t/ha<br>0.2–1.8 t/ha
Education deprivation	Education level of household head	1–4
Lack of alternative income	Local off-farm income and remittances	0–2400 Soles/year*person
a Average: 4 persons per household		

Candidate-level Analysis	In preparing the further analysis, we adjusted data sets with only a few extreme values to increase the influence of these data sets on the cluster partitions. For example, the majority of households possess eight or fewer units of livestock. The few households with up to 39 livestock units can be formally interpreted as single outliers which skew the overall data distribution of this indicator. To deskew such data sets and thus adequately focus on the majority of households, we winsorised the data sets, i.e., replaced the outlying observations (4%) with the next available less extreme observation (Barnett and Lewis 1994). This pro- cedure was applied to the area and livestock constraints as well as the alternative income. All indicators were then normalised to a 0–1 range using the minimum–maximum values. Prior to the cluster analysis, we determined correlations<br>between the selected indicators and the variance distribu- tion in the data space. Firstly, the correlation coefficients reached average absolute values of 0.11. The crop area and livestock units correlate most strongly here (0.46) reflec- ting the mixed production systems. Furthermore, variables showing a large variance may be intuitively expected to contain most of the structure information. Therefore, we explored the variance of the selected indicators using a principal component analysis (PCA). The PCA was per- formed using the open source statistics package R (RDCT 2009) following standard procedure based on Pearson correlations.

Community level

Selection of most useful operationalizations (to be completed by expert reviewer)	
**Construct**	**Community level**
1st preference	[Author (year)]
2nd preference	[Author (year)]
3rd preference	[Author (year)]

Structured summary of candidate operationalizations				
**Candidate article:** Echevin				
**Construct operationalized:** community level				
Sub-constructs		Intermediate constructs		
		Directly operationalized constructs		
Conceptual framework		DIRECTLY OPERATIONALIZED		
Operationalization of sub-constructs				
9.	•	Data collection		Having chosen the suitable indicators, now these need to be normalized so as to bring<br>the values of the indicators within the comparable range (Nelson, et al., 2010b; Gbetibouo & Ringler, 2009; Vincent, 2004). Normalization is done by subtracting the mean from the observed value and dividing by the standard deviation for each indicator.<br>$Normalized\ value\ = Observed\ Value\ /\ Mean\ standard\ deviation$<br>Next, weights should be assigned to these indicators.<br>[...]<br>Assigning weight by Principal Component Analysis (PCA) following Filmer and Pritchett (2001) is thus preferred compared to the former two methods (Nelson et al., 2010b; Gbetibouo & Ringler, 2009; Cutter, Boruff, & Shirley, 2003). PCA was run for the selected indicators of exposure, sensitivity, and adaptive capacity separately in Data Analysis and Statistical Software (STATA10) software for assigning the weights. The loadings from the first component of PCA are used as the weights for the indicators. The weights assigned for each indicator varies between -1 and +1, sign of the indicators denoting the direction of relationship with other indicators used to construct the respective index. The magnitude of the weights describes the contribution of each indicator to the value of the index. PCA was run separately for the indicators of exposure, sensitivity and adaptive capacity. Stepwise PCA was run for the indicators of adaptive capacity. The first-step PCA was run for the indicators of each asset group separately to observe the relative importance of indicators within each asset category. From the weights obtained from first-step PCA, individual index values for each asset type was calculated. Second-step PCA

				was run using the index values for each of the five asset types to analyze which asset group contributes the most to the total adaptive capacity. Overall adaptive capacity index was calculated using the weights (loadings) obtained from the second step PCA run for the five asset categories.<br>The normalized variables are then multiplied with the assigned weights to construct the indices (for exposure, sensitivity, and adaptive capacity separately) using the following formulae:<br>$Ij = \sum^{k}_{i=1} b_i[(a_{ji} – x_i)/s_i]$<br>where, 'I' is the respective index value, 'b' is the loadings from first component from PCA (PCA1) taken as weights for respective indicators, 'a' is the indicator value, 'x' is the mean indicator value, and 's' is the standard deviation of the indicators. Finally, vulnerability index for each household is calculated as: V = E + S – AC, where, V is the vulnerability index, E the exposure index, S is the sensitivity index and AC is the adaptive capacity index for respective household.
		Operational questions		Having chosen the suitable indicators, now these need to be normalized so as to bring<br>the values of the indicators within the comparable range (Nelson, et al., 2010b; Gbetibouo & Ringler, 2009; Vincent, 2004). Normalization is done by subtracting the mean from the observed value and dividing by the standard deviation for each indicator.<br>$Normalized\ value\ = Observed\ Value\ /\ Mean\ standard\ deviation$<br>Next, weights should be assigned to these indicators.<br>[...]<br>Assigning weight by Principal Component Analysis (PCA) following Filmer and Pritchett (2001) is thus preferred compared to the former two methods (Nelson et al., 2010b; Gbetibouo & Ringler, 2009; Cutter, Boruff, & Shirley, 2003). PCA was run for the selected indicators of exposure, sensitivity, and adaptive capacity separately in Data Analysis and Statistical Software (STATA10) software for assigning the weights. The loadings from the first component of PCA are used as the weights for the indicators. The weights assigned for each indicator varies between -1 and +1, sign of the indicators denoting the direction of relationship with other indicators used to construct the respective index. The magnitude of the weights describes the contribution of each indicator to the value of the index. PCA was run separately for the indicators of exposure, sensitivity and adaptive capacity. Stepwise PCA was run for the indicators of adaptive capacity. The first-step PCA was run for the indicators of each asset group separately to observe the relative importance of indicators within each asset category. From the weights obtained from first-step PCA, individual index values for each asset type was calculated. Second-step PCA was run using the index values for each of the five asset

			types to analyze which asset group contributes the most to the total adaptive capacity. Overall adaptive capacity index was calculated using the weights (loadings) obtained from the second step PCA run for the five asset categories.<br>The normalized variables are then multiplied with the assigned weights to construct the indices (for exposure, sensitivity, and adaptive capacity separately) using the following formulae:<br>$Ij = \sum_{i=1}^{k} b_i[(a_{ji} – x_i)/s_i]$<br>where, 'I' is the respective index value, 'b' is the loadings from first component from PCA (PCA1) taken as weights for respective indicators, 'a' is the indicator value, 'x' is the mean indicator value, and 's' is the standard deviation of the indicators. Finally, vulnerability index for each household is calculated as: V = E + S – AC, where, V is the vulnerability index, E the exposure index, S is the sensitivity index and AC is the adaptive capacity index for respective household.
Candidate-level Analysis	We use self-reported shocks in order to estimate their impact on consumption and income.<br>Table 3 presents OLS estimates and GLLAMM estimates. Both models are estimated with log consumption and log income. Our preferred specification regroups a large set of explanatory variables such as household characteristics, regional dummies, community characteristics, interaction between household characteristics and community characteristics, shocks variables, interaction between shocks variables and household characteristics, interaction between shocks variables and community characteristics. Estimating the two-level linear random coefficient model (GLLAMM) allows us to decompose the variance of the residuals into an idiosyncratic variance and a covariate variance.		

**Structured summary of candidate operationalizations**		
**Candidate article:** Günther & Harttgen		
**Construct operationalized:** community level		
Sub-constructs	Intermediate constructs	
	Directly operationalized constructs	
Conceptual framework	DIRECTLY OPERATIONALIZED	
Operationalization of sub-constructs		
10. •	Data collection	Not valid/feasible
	Operational questions	
Candidate-level Analysis		

Covariate shocks

Selection of most useful operationalizations (to be completed by expert reviewer)	
**Construct**	**Covariate shocks**
1st preference	[Author (year)]
2nd preference	[Author (year)]
3rd preference	[Author (year)]

**Structured summary of candidate operationalizations**		
**Candidate article:** Günther & Harttgen		
**Construct operationalized:** Covariate shocks		
Sub-constructs	Intermediate constructs	
	Directly operationalized constructs	
Conceptual framework	DIRECTLY OPERATIONALIZED	
Operationalization of sub-constructs		
11. •	Data collection	Data on household characteristics are taken from the na-tional representative household survey of 2001 (Enqueˆte Aupre`s Des Me´nages), covering 5,080 households (1,778 ur-ban and 3,302 rural households) in 186 communities. The community census is the 2001 ILO/Cornell Commune Level census which covers 1,385 of the 1,395 communities in Mada-gascar. Both surveys do not have any time dimension.
	Operational questions	More precisely, for each community and for the three years preceding the survey (2001, 2000, 1999) it is reported whether the community was exposed to any of 16 covariate shocks (most of these are reported in Tables A.1 and A.2 in Appendix).<br>[...]<br>Table A.1. Households with exposure to shocks<br>Malaria<br>Tuberculosis<br>Typhoid<br>Cholera<br>Rice pest<br>Swineflu<br>Newcastle<br>Flooding<br>Impassible bridge or road<br>Drought<br>Cyclones
Candidate-level Analysis	The estimated average mean and variance in consumption for the whole sample are presented in Table 3, also separately for rural and urban households. The expected per capita (log) consumption of rural households is below the (log) poverty line, whereas the expected per capita (log) consumption of ur- ban households lies above the (log) poverty line.<br>With regard to the estimated standard deviation in consumption, we show that the	

	estimated standard deviation is slightly higher for rural households than for urban households, with a standard deviation of 0.58 compared to 0.54 (Table 3). Idiosyn- cratic variance is much higher than covariate variance for ur- ban and only slightly higher for rural households. Hence, the relative importance of idiosyncratic variance is much higher for urban than for rural households. More precisely, whereas among urban households the estimated idiosyncratic standard deviation of consumption is 3.25 times as high as covariate standard deviation, the respective rate is only 1.57 for rural households. As a robustness check, we assume that half of the estimated idiosyncratic variance is measurement error. The idiosyncratic standard deviation is still 2.13 as high as covariate standard deviation for urban households and 1.14 as high for rural households (see Table A.3).

**Structured summary of candidate operationalizations**			
**Candidate article:** Sarris & karfakis			
**Construct operationalized:** Covariate shocks			
Sub-constructs		Intermediate constructs	
		Directly operationalized constructs	
Conceptual framework		DDIRECLY OPERATIONALIZED	
Operationalization of sub-constructs			
12.	•	Data collection	The analysis of the paper will be based on a representative survey of 957 rural households in 45 villages done in the Kilimanjaro region, in November 2003, and a representative survey of 892 rural households in 36 villages done in the Ruvuma region in February-March 2004. The survey was repeated a year later<br>[...]<br>The questionnaire was designed to investigate the complete socio-economic characteristics of households with a particular emphasis on their vulnerability to a variety of risks.
		Operational questions	Table 2: Percentage of households affected by various shocks between 1999 and 2003, by region and status as cash crop grower or not.

**Health**<br>Death<br>Illness	..	..
**Climatic**<br>Drought<br>Excessive rains		
**Agricultural production**<br>Harvest loss<br>Livestock loss<br>Post harvest cereal loss		
**Economic**<br>Cash crop price shock<br>Cereal price shock<br>Unemployment		
**Property**<br>Theft		

			Fire/house destroyed<br>Land loss
Candidate-level Analysis	Table 5 exhibits the results of the (instrumental variable) regressions on consumption and the squared residuals of consumption as per equations (15) and (17). The key variable for the vulnerability analysis is the coefficient in the consumption regressions of crop income per acre. Concerning the consumption per equivalent adult, it can be seen that it depends positively and significantly on aggregate crop productivity, the size of land, the size of household, several wealth variables such as the lagged value of the number of animals owned and the lagged value of consumer durables, the age of the household head (significant in Ruvuma), access to credit variables, and some education variables.<br>The Durbin-Wu-Hausman test of the exogeneity of the crop productivity strongly rejects the hypothesis of exogeneity, so IV is appropriate. Table 6 presents the first stage regressions for the IV estimates. We use as instruments a variety of exogenous land characteristics, as well as weather shock variables, and lagged dummies for whether the farm household used fertilizer and chemicals, as well as the lagged number of coffee and cashew trees. The Sargan test does not invalidate the use of these instruments.<br>It must be mentioned that in the consumption regressions the IV regression coefficient of crop income per acre is significantly larger in the IV regressions compared to the OLS estimates (the OLS estimates for these coefficients are 0.028 for Kilimanjaro and 0.174 for Ruvuma, compared to 0.144 and 0.411 for the IV regressions in table 5 for the two regions).<br>The consumption regressions explain about 47 and 51 percent of the variance of consumption in Kilimanjaro and Ruvuma respectively. The regressions of the squared residuals from the consumption regressions on the same explanatory variables as the ones in the consumption regressions (excluding the variables that are related to covariate and idiosyncratic shocks) reveal that fewer of the variables are significant. In Kilimanjaro the dependency ratio, the value of the dwelling, the number of small animals, and the membership in a social group are significant, while in Ruvuma, the only two significant variables are the dummies for whether the household receives remittances and whether the household has easy access to seasonal credit. The regressions explain a rather small proportion of the error less than 10 percent in both regions). This suggests that unexplained components of consumption variability dominate any parts that maybe due to structural household specific factors.<br>Tables 7 and 8 indicate the average vulnerability index in Kilimanjaro and Ruvuma by district, along with the proportions of the variance of consumption that are due to covariate factors, the average consumption per capita and the average headcount measures of poverty rates in both years of the survey. The first observation is that average vulnerability in Kilimanjaro is much lower than in Ruvuma (31 percent versus 60 percent). This is in line with the much larger poverty incidence in Ruvuma compared to Kilimanjaro that was indicated earlier (63.3 percent versus 39.5 percent).		

Exposure

Selection of most useful operationalizations (to be completed by expert reviewer)	
**Construct**	**Exposure**
1st preference	[Author (year)]
2nd preference	[Author (year)]
3rd preference	[Author (year)]

Structured summary of candidate operationalizations			
**Candidate article:** Baca et al			
**Construct operationalized:** Exposure			
Sub-constructs	Intermediate constructs		
	Directly operationalized constructs		
Conceptual framework	DIRECTLY OPERATIONALIZED		
Operationalization of sub-constructs			
13. •	Data collection	The methodology combined current climate data with future climate change predictions. To map current climatic suitability, the historical climate database WorldClim (www.worldclim.org) was used<br>[...]<br>To predict future climate, the SRES-A2a scenario 19 IPCC Global Circulation Models were used. The Delta method was used to down-scale the climate change data, based on the sum of the anomalies interpolated with the WorldClim monthly high- resolution surfaces [15].	
	Operational questions	To map current climatic suitability, the historical climate database WorldClim (www.worldclim.org) was used. The variables included a total of 19 bioclimatic variables derived from monthly precipitation, monthly median temperature, minimum and maximum temper- ature [15]. Bioclimatic variables represent annual trends, season- ality, and extreme conditions.	
Candidate-level Analysis	The Maximum entropy (MAXENT) method, a general-purpose<br>method for making predictions or inferences based on incomplete information [17], was used to predict the future climatic suitability for coffee. The model requires calibration with climate data for current coffee production areas, which is provided by GPS coordinates. The model assumes that a certain future climate at a given site is as suitable or unsuitable for the crop as is the same climate at another site in the present. This assumption is reasonable as long as crop genetics and cropping systems do not significantly change. It thus predicts what will happen in terms of relative climatic suitability for a crop if these factors do not change and helps identify those sites where adaptations in crops and cropping systems are necessary in order to avoid the consequences of a predicted decline in climatic suitability. This approach has previously been used for coffee [6], [18]. Two measures of uncertainty were calculated: (1) the agreement<br>of calculated models as a percentage of models that predict changes in the same		

	direction and (2) the coefficient of variation (CV) among models.<br>[...]<br>For exposure, the relative decreases in climatic suitability according to the MAXENT model were divided into three classes of suitability loss (low, medium, high). For sensitivity and adaptive capacity, indicators were identified and quantified through interviews with the farming families.<br>[...]<br>Each factor (exposure, sensitivity and adaptive capacity), as previously explained, and was classified into three levels (high, medium, low). To calculate the vulnerability equation we assigned each level a quantitative value: low=1, medium=2, high=3. With three factors and three levels per factor, we obtained 27 possible combinations. After applying the equation we obtained 7 values (–1,0,1,2,3,4,5), which we used to define low (–1,0), medium (1,2,3,) and high (4,5) levels of vulnerability (Figure 1). A Principal Components Analysis (PCA) was carried out to identify the indicators that most contribute to the sensitivity or adaptive capacity of families in different municipalities.

<table>
<tr><td colspan="5">Structured summary of candidate operationalizations</td></tr>
<tr><td colspan="5">Candidate article: Hahn et al</td></tr>
<tr><td colspan="5">Construct operationalized: Exposure</td></tr>
<tr><td colspan="2" rowspan="2">Sub-constructs</td><td>Intermediate constructs</td><td colspan="2">Natural disaster and climate change</td></tr>
<tr><td>Directly operationalized constructs</td><td colspan="2">Flood, drought, cyclone events; injury or death from disaster; no warning of disaster; maximum temperature; minimum temperature; average precipitation</td></tr>
<tr><td colspan="2">Conceptual framework</td><td colspan="3">Exposure of the study population is measured by the number of natural disasters that have occurred in the past 6 years, while climate variability is measured by the average standard deviation of the maximum and minimum monthly temperatures and monthly precipitation over a 6-year period.</td></tr>
<tr><td colspan="2">Operationalization of sub-constructs</td><td colspan="3"></td></tr>
<tr><td rowspan="2">14.</td><td rowspan="2">Flood, drought, cyclone events</td><td>Data collection</td><td colspan="2">household surveys</td></tr>
<tr><td>Operational questions</td><td colspan="2">Table 1 includes an explanation of how each sub-component was quantified, the survey question used to collect the data, the original source of the survey question, and potential sources of bias.
[...]
<table>
<tr><td colspan="3">Table 1 Major components and sub-components comprising the Livelihood Vulnerability Index (LVI) developed for two districts of Mozambique.</td></tr>
<tr><td>Sub-components</td><td>Explanation of sub-components</td><td>Survey question</td></tr>
<tr><td>Average number of flood, drought, and cyclone events in the</td><td>Total number of floods, droughts, and cyclones that were reported by</td><td>How many times has this area been affected by a flood/cyclone/drought in 2001–2007?</td></tr>
</table></td></tr>
</table>

<table>
<tr><td></td><td></td><td></td><td><table><tr><td>past 6 years (range: 0–7)</td><td>households in the past 6 years.</td><td></td></tr></table></td></tr>
<tr><td rowspan="2">15.</td><td rowspan="2">injury or death from disaster</td><td>Data collection</td><td>household surveys</td></tr>
<tr><td>Operational questions</td><td>Table 1 includes an explanation of how each sub-component was quantified, the survey question used to collect the data, the original source of the survey question, and potential sources of bias.
[...]

<table><tr><td colspan="3">Table 1 Major components and sub-components comprising the Livelihood Vulnerability Index (LVI) developed for two districts of Mozambique.</td></tr><tr><td>Sub-components</td><td>Explanation of sub-components</td><td>Survey question</td></tr><tr><td>Percent of households with an injury or death as a result of the most severe natural disaster in the past 6 years</td><td>Percentage of households that reported either an injury to or death of one of their family members as a result of the most severe flood, drought, or cyclone in the past 6 years.</td><td>Was anyone in your family injured in the flood/cyclone drought? Did anyone in your family die during the flood/cyclone/drought?</td></tr></table></td></tr>
<tr><td rowspan="2">16.</td><td rowspan="2">no warning of disaster</td><td>Data collection</td><td>household surveys</td></tr>
<tr><td>Operational questions</td><td>Table 1 includes an explanation of how each sub-component was quantified, the survey question used to collect the data, the original source of the survey question, and potential sources of bias.
[...]

<table><tr><td colspan="3">Table 1 Major components and sub-components comprising the Livelihood Vulnerability Index (LVI) developed for two districts of Mozambique.</td></tr><tr><td>Sub-components</td><td>Explanation of sub-components</td><td>Survey question</td></tr><tr><td>Percent of households that did not receive a warning</td><td>Percentage of households that did not receive a warning</td><td>Did you receive a warning about the flood/cyclone/drought before it happened?</td></tr></table></td></tr>
</table>

<table>
<tr><td></td><td></td><td></td><td><table><tr><td>about the pending natural disasters</td><td>about the most severe flood, drought, and cyclone event in the past 6 years.</td><td></td></tr></table></td></tr>
<tr><td rowspan="2">17.</td><td rowspan="2">maximum temperature</td><td>Data collection</td><td>provincial data; weather station based in the provincial capital</td></tr>
<tr><td>Operational questions</td><td><table><tr><td colspan="3">Table 1 Major components and sub-components comprising the Livelihood Vulnerability Index (LVI) developed for two districts of Mozambique.</td></tr><tr><td>Sub-components</td><td>Explanation of sub-components</td><td>Survey question</td></tr><tr><td>Mean standard deviation of the daily average maximum temperature by month</td><td>Standard deviation of the average daily maximum temperature by month between 1998 and 2003 was averaged for each provinceb</td><td>1998–2003: provincial data; weather station based in the provincial capital</td></tr></table></td></tr>
<tr><td rowspan="2"></td><td rowspan="2">minimum temperature</td><td>Data collection</td><td>provincial data; weather station based in the provincial capital</td></tr>
<tr><td>Operational questions</td><td><table><tr><td colspan="3">Table 1 Major components and sub-components comprising the Livelihood Vulnerability Index (LVI) developed for two districts of Mozambique.</td></tr><tr><td>Sub-components</td><td>Explanation of sub-components</td><td>Survey question</td></tr><tr><td>Mean standard deviation of the daily average maximum temperature by month</td><td>Standard deviation of the average daily maximum temperature by month between 1998 and 2003 was averaged for each provinceb</td><td>1998–2003: provincial data; weather station based in the provincial capital</td></tr></table></td></tr>
<tr><td rowspan="2"></td><td rowspan="2">average precipitation</td><td>Data collection</td><td>provincial data; weather station based in the provincial capital</td></tr>
<tr><td>Operational questions</td><td><table><tr><td>Table 1 Major components and sub-components comprising the Livelihood Vulnerability Index (LVI) developed for two districts of Mozambique.</td></tr></table></td></tr>
</table>

Sub-components	Explanation of sub-components	Survey question
Sub-components	*Explanation of sub-components*	*Survey question*
Mean standard deviation of the daily average maximum temperature by month	Standard deviation of the average daily maximum temperature by month between 1998 and 2003 was averaged for each provinceb	1998–2003: provincial data; weather station based in the provincial capital

Candidate-level Analysis	Rather than merge the major components into the LVI in one step, they are first combined according to the categorization scheme in Table 2 using the following equation: $CF_d = [\sum^{n}_{i=1} w_{Mj}\, M_{di}]/[\sum^{n}_{i=1} w_{Mi}]$ where CFd is an IPCC-defined contributing factor (exposure, sensitivity, or adaptive capacity) for district d, Mdi are the major components for district d indexed by i, wMi is the weight of each major component, and n is the number of major components in each contributing factor.

**Structured summary of candidate operationalizations**			
**Candidate article:** Notenbaert et al			
**Construct operationalized:** Exposure			
Sub-constructs	Intermediate constructs		
	Directly operationalized constructs		
Conceptual framework	DIRECTLY OPERATAIONALIZED		
Operationalization of sub-constructs			
18. •	Data collection	In this study, we assume the exposure to climate change and variability of households in the same village to be equal. Differences in vulnerability, described as outcomes of this exposure, are therefore attributed to differences in sensitivity and adaptive capacity only. [...] We also believe that the overall assumption of equal exposure is sensible in the fairly homogenous landscape of Mabalane district and the relatively clustered villages as our unit of analysis. When analyzing communities spread out in more complex land- scapes or in rugged terrain, this assumption will not nec- essarily hold true	
	Operational questions	In this study, we assume the exposure to climate change and variability of households in the same village to be equal. Differences in vulnerability, described as outcomes of this exposure, are therefore attributed to differences in sensitivity and adaptive capacity only. [...] We also believe that the overall assumption of equal exposure	

			is sensible in the fairly homogenous landscape of Mabalane district and the relatively clustered villages as our unit of analysis. When analyzing communities spread out in more complex land- scapes or in rugged terrain, this assumption will not nec- essarily hold true
Candidate-level Analysis	In this study, we assume the exposure to climate change<br>and variability of households in the same village to be equal. Differences in vulnerability, described as outcomes of this exposure, are therefore attributed to differences in sensitivity and adaptive capacity only.<br>[...]<br>We also believe that the overall assumption of equal exposure is sensible in the fairly homogenous landscape of Mabalane district and the relatively clustered villages as our unit of analysis. When analyzing communities spread out in more complex land- scapes or in rugged terrain, this assumption will not nec- essarily hold true		

**Structured summary of candidate operationalizations**			
**Candidate article:** Piya et al			
**Construct operationalized:** Exposure			
Sub-constructs	Intermediate constructs		
	Directly operationalized constructs		
Conceptual framework	DIRECLY OPERATIONALIZED		
Operationalization of sub-constructs			
19. •	Data collection	This study is based on the primary data collected by household survey conducted in<br>two phases. The first phase of household survey was conducted in February-March 2010 and the second phase in May-June 2011.<br>[...]<br>The latitude, longitude and altitude of the sample households were recorded during the second phase of field visit. This paper also makes use of raw monthly minimum and maximum temperature and<br>monthly precipitation data obtained from Department of Hydrology and Meteorology (DHM) in Kathmandu, Nepal for the time period of 32 years, from 1977-2008. Temperature data was obtained from 49 stations and precipitation data from 218 stations distributed all over the country. The temperature and precipitation at the household level was interpolated for each year from the weather stations using the latitude-longitude-altitude information of each household by ordinary kriging method in ArcGIS 10.	
	Operational questions	3.2.1 Exposure For this study, historical changes in climate variables and occurrence of extreme<br>climatic events are taken as indicators of exposure (Table 1). Rate of change in average annual maximum temperature, average annual minimum temperature and average annual precipitation for the time period of 1977–2008 represent the historical climate changes. The temperature and precipitation for individual household was interpolated for each year from	

			the station level data (49 temperature stations and 218 precipitation stations) using the latitude, longitude, and altitude information of the stations and the households by ordinary kriging method in ArcGIS10. The coefficient of the trends of climate variables is calculated separately for each household. Floods/landslides, droughts and hailstorms are the most commonly occurring natural disasters in the study area. Number of occurrence of these extreme events for the last ten years was obtained for each household from the household survey (Appendix 1). It was hypothesized that higher the rate of change of the climate variables and higher the frequency of natural disasters, higher will be the exposure of the households to climate change and extremes.

Table 1. Indicators for exposure

Component Indicators	Description of the Indicators	Unit	Hypothesized relation
Historical change in climate Variables	Rate of change in average annual minimum temperature (1977 – 2008)	Coefficient of trend	+
	Rate of change in average annual maximum temperature (1977 – 2008)	Coefficient of trend	+
	Rate of change in average annual precipitation (1977 – 2008)	Coefficient of trend	+

<table>
<tr><td></td><td></td><td></td><td>
<table>
<tr><td>Extreme climate events</td><td>Frequency of climate related natural disasters (floods, landslides, droughts and hailstorms) over the last 10 years</td><td>Number</td><td>+</td></tr>
</table>
</td></tr>
<tr><td colspan="2">Candidate-level Analysis</td><td colspan="2">Having chosen the suitable indicators, now these need to be normalized so as to bring the values of the indicators within the comparable range (Nelson, et al., 2010b; Gbetibouo & Ringler, 2009; Vincent, 2004). Normalization is done by subtracting the mean from the observed value and dividing by the standard deviation for each indicator.
$Normalized\ value\ = Observed\ Value\ /\ Mean\ standard\ deviation$
Next, weights should be assigned to these indicators.
[...]

The normalized variables are then multiplied with the assigned weights to construct the indices (for exposure, sensitivity, and adaptive capacity separately) using the following formulae:
$Ij = \sum^{k}_{i=1} b_i[(a_{ji} – x_i)/s_i]$
where, ‘I’ is the respective index value, ‘b’ is the loadings from first component from PCA (PCA1) taken as weights for respective indicators, ‘a’ is the indicator value, ‘x’ is the mean indicator value, and ‘s’ is the standard deviation of the indicators. Finally, vulnerability index for each household is calculated as: V = E + S – AC, where, V is the vulnerability index, E the exposure index, S is the sensitivity index and AC is the adaptive capacity index for respective household.</td></tr>
</table>

<table>
<tr><td colspan="5">Structured summary of candidate operationalizations</td></tr>
<tr><td colspan="5">Candidate article: Sietz et al</td></tr>
<tr><td colspan="5">Construct operationalized: Exposure</td></tr>
<tr><td colspan="2" rowspan="2">Sub-constructs</td><td colspan="2">Intermediate constructs</td><td></td></tr>
<tr><td colspan="2">Directly operationalized constructs</td><td></td></tr>
<tr><td colspan="2">Conceptual framework</td><td colspan="3">Directly operationalized</td></tr>
<tr><td colspan="2">Operationalization of sub-constructs</td><td colspan="3"></td></tr>
<tr><td rowspan="2">20</td><td rowspan="2">•</td><td>Data collection</td><td colspan="2">The necessary weather information is available in good quality for the 1996–2006 period for two stations located in Puno and Cabanillas (see Fig. 1). Table 2 shows the average pre- cipitation and temperature for both stations.</td></tr>
<tr><td>Operational questions</td><td colspan="2">The necessary weather information is available in good quality for the 1996–2006 period for two stations located in Puno and Cabanillas (see Fig. 1). Table 2 shows the average pre- cipitation and temperature for both stations.
[...]</td></tr>
</table>

Table 2 Mean precipitation and temperature for 1996–2006 at Puno and Cabanillas stations (Data source: Servicio Nacional de Meteorologı´ae Hidrologı´a del Peru´, SENAMHI)

	Mean values for 1996–2006												
	Jan	Feb	Mar	Apr	May	Jun	Jul	Aug	Sept	Oct	Nov	Dec	Total
Precipitation (mm)													
Puno	201	161	138	60	7	3	4	14	27	51	48	88	801
Cabanillas	166	165	112	56	6	1	3	11	19	54	55	91	738
Mean temperature (□C)													
Puno	10.8	10.7	10.6	9.7	8.1	6.8	6.8	7.9	9.3	10.4	11.0	11.5	9.5
Cabanillas	10.6	10.5	10.5	9.8	8.6	7.3	6.9	8.1	9.6	10.6	11.1	11.3	9.6
Minimum temperature (□C)													
Puno	5.7	5.8	5.4	3.8	0.8	-0.9	-1.1	0.4	1.9	3.6	4.3	5.4	2.9
Cabanillas	5.3	5.5	5.2	3.7	1.1	-0.8	-1.5	0.3	2.1	3.7	4.2	5.1	2.8

Candidate-level Analysis	To make the two stations comparable, we determined relative anomalies compared to the average precipitation course over the period 1996–2006 through precipitation ranking. This ranking was then used to identify driest and wettest periods which caused production damage. Since soil water content integrates previous precipitation events to some extent, we cumulated the daily precipitation records in a 20-day window. This window was moved as a running mean by steps of one decade (10 days). This choice is supported by the calibration campaign 2003/2004 descri- bed below. Covering the rainy season from December to March, we obtained cumulated precipitation values for 12 time segments (Fig. 2). This number of time segments still allows for sufficient resolution of intra-seasonal anomalies.<br>[...]<br>In conclusion, climate exposure was precipitation-driven<br>during the relevant campaigns. Similar precipitation and temperature conditions at both stations indicate a similar climate exposure throughout the study region. Therefore, a potential spatial variation in the exposure does not have to be considered in the further vulnerability analysis.

Household level

Selection of most useful operationalizations (to be completed by expert reviewer)	
**Construct**	**Household level**
1st preference	[Author (year)]
2nd preference	[Author (year)]
3rd preference	[Author (year)]

**Structured summary of candidate operationalizations**			
**Candidate article:** Echevin			
**Construct operationalized:** Household level			
Sub-constructs		Intermediate constructs	
		Directly operationalized constructs	
Conceptual framework		DIRECLY OPERATIONALIZED	
Operationalization of sub-constructs			
21.	•	Data collection	The vulnerability and food security survey was conducted in Haiti in October and<br>November 2007 on approximately 3,000 households living in 228 rural communities. This survey has been realized by the National Coordination of Food Security Unit with the partnership of the World Food Program. A community-related component was added to the household component of the survey, in connection with infrastructures and accessibility to basic social services.
		Operational questions	Table 2 presents summary statistics for variables used in the analysis. Consumption and<br>income are expressed in Gourdes. The agricultural index is a composite indicator which is a linear combination of categorical variables obtained from a multiple correspondence analysis (cf. Asselin, 2009). Variables considered in the analysis are the number of lands, animals and agricultural materials owned by the household.<br>[...]<br><br>**Table 2. Descriptive statistics**<br>*Household variables*<br><br>Log of consumption<br>Log of income<br>Agricultural index<br>Income diversity<br>Household size<br>Number of children<br>Age of head<br>Male head<br>Years of schooling (head)

			Activity of head<br>No job<br>Agroalimentary<br>Industry<br>Construction<br>Trade<br>Services<br>Other activity	
Candidate-level Analysis	We use self-reported shocks in order to estimate their impact on consumption and income.<br>Table 3 presents OLS estimates and GLLAMM estimates. Both models are estimated with log consumption and log income. Our preferred specification regroups a large set of explanatory variables such as household characteristics, regional dummies, community characteristics, interaction between household characteristics and community characteristics, shocks variables, interaction between shocks variables and household characteristics, interaction between shocks variables and community characteristics. Estimating the two-level linear random coefficient model (GLLAMM) allows us to decompose the variance of the residuals into an idiosyncratic variance and a covariate variance.			

**Structured summary of candidate operationalizations**		
**Candidate article:** Günther & Harttgen		
**Construct operationalized:** Household level		
Sub-constructs	Intermediate constructs	
	Directly operationalized constructs	
Conceptual framework	DIRECTLY OPERATIONALIZED	
Operationalization of sub-constructs		

22.	•	Data collection	Data on household characteristics are taken from the national representative household survey of 2001 (Enqueˆte Aupre`s Des Me´nages), covering 5,080 households (1,778 urban and 3,302 rural households) in 186 communities.<br>[...]<br>Both surveys do not have any time dimension.
		Operational questions	Table 1. Summary statistics for households and communities

Table 1. Summary statistics for households and communities
Household characteristics
Age of HH head (years)
Number of children
Female headed households (%)
Household size Residence (%)
Years of schooling of HH head
Works in agriculture (HH head) (%)
Works in informal sector (HH head) (%)
Works in formal sector (HH head) (%)
Works in public sector (HH head) (%)

			Enterprise owner (%)
			Land owner (%)
			Number of cattle
Candidate-level Analysis	To estimate households' expected mean and variance in con- sumption, we first use the household characteristics in Table 1. In addition, we consider an agricultural asset index (composed of eight productive assets) estimated via principal component analysis (Filmer & Pritchett, 2001).<br>[...]<br>As described in Section 3, we estimate the expected mean<br>and variance per capita household (log) consumption using multilevel modeling. We also decompose the unexplained con- sumption variance into an idiosyncratic (household-level) and a covariate (community-level) component. The regression results of the multilevel model for the esti- mated mean of (log) consumption are presented in Table 2.		

Idiosyncratic shocks

Selection of most useful operationalizations (to be completed by expert reviewer)	
**Construct**	Idiosyncratic shocks
1st preference	[Author (year)]
2nd preference	[Author (year)]
3rd preference	[Author (year)]

**Structured summary of candidate operationalizations**			
**Candidate article:** Günther & Harttgen			
**Construct operationalized:** Idiosyncratic shocks			
Sub-constructs		Intermediate constructs	
		Directly operationalized constructs	
Conceptual framework		DIRECTLY OPERATIONALIZED	
Operationalization of sub-constructs			
23.	•	Data collection	Data on household characteristics are taken from the na- tional representative household survey of 2001 (Enqueˆte Aupre`s Des Me´nages), covering 5,080 households (1,778 ur- ban and 3,302 rural households) in 186 communities. The community census is the 2001 ILO/Cornell Commune Level census which covers 1,385 of the 1,395 communities in Mada- gascar. Both surveys do not have any time dimension.
		Operational questions	
Candidate-level Analysis		The estimated average mean and variance in consumption<br>for the whole sample are presented in Table 3, also separately for rural and urban households. The expected per capita (log) consumption of rural households is below the (log) poverty line, whereas the expected per capita (log) consumption of ur- ban households lies above the (log) poverty line.<br>With regard to the estimated standard deviation in consumption, we show that the estimated standard deviation is slightly higher for rural households than for urban households, with a standard deviation of 0.58 compared to 0.54 (Table 3). Idiosyn- cratic variance is much higher than covariate variance for ur- ban and only slightly higher for rural households. Hence, the relative importance of idiosyncratic variance is much higher for urban than for rural households. More precisely, whereas among urban households the estimated idiosyncratic standard deviation of consumption is 3.25 times as high as covariate standard deviation, the respective rate is only 1.57 for rural households. As a robustness check, we assume that half of the estimated idiosyncratic variance is measurement error. The idiosyncratic standard deviation is still 2.13 as high as covariate standard deviation for urban households and 1.14 as high for rural households (see Table A.3).	

**Structured summary of candidate operationalizations**		
**Candidate article:** Sarris & Karfakis		
**Construct operationalized:** Idiosyncratic shocks		
Sub-constructs	Intermediate constructs	

	Directly operationalized constructs	
Conceptual framework	DIRECTLY OPERATIONALIZED	
Operationalization of sub-constructs		
24. •	Data collection	The analysis of the paper will be based on a representative survey of 957 rural households in 45 villages done in the Kilimanjaro region, in November 2003, and a representative survey of 892 rural households in 36 villages done in the Ruvuma region in February-March 2004. The survey was repeated a year later<br>[...]<br>The questionnaire was designed to investigate the complete socio-economic characteristics of households with a particular emphasis on their vulnerability to a variety of risks.
	Operational questions	Table 2: Percentage of households affected by various shocks between 1999 and 2003, by region and status as cash crop grower or not.

**Health**<br>Death<br>Illness	..	..
**Climatic**<br>Drought<br>Excessive rains		
**Agricultural production**<br>Harvest loss<br>Livestock loss<br>Post harvest cereal loss		
**Economic**<br>Cash crop price shock<br>Cereal price shock<br>Unemployment		
**Property**<br>Theft<br>Fire/house destroyed<br>Land loss		

Candidate-level Analysis	Table 5 exhibits the results of the (instrumental variable) regressions on consumption and the squared residuals of consumption as per equations (15) and (17). The key variable for the vulnerability analysis is the coefficient in the consumption regressions of crop income per acre. Concerning the consumption per equivalent adult, it can be seen that it depends positively and significantly on aggregate crop productivity, the size of land, the size of household, several wealth variables such as the lagged value of the number of animals owned and the lagged value of consumer durables, the age of the household head (significant in Ruvuma), access to credit variables, and some education variables.<br>The Durbin-Wu-Hausman test of the exogeneity of the crop productivity strongly rejects the hypothesis of exogeneity, so IV is appropriate. Table 6 presents the first stage regressions for the IV estimates. We use as instruments a variety of exogenous land characteristics, as well as weather shock variables, and lagged dummies for whether the

	farm household used fertilizer and chemicals, as well as the lagged number of coffee and cashew trees. The Sargan test does not invalidate the use of these instruments.<br>It must be mentioned that in the consumption regressions the IV regression coefficient of crop income per acre is significantly larger in the IV regressions compared to the OLS estimates (the OLS estimates for these coefficients are 0.028 for Kilimanjaro and 0.174 for Ruvuma, compared to 0.144 and 0.411 for the IV regressions in table 5 for the two regions).<br>The consumption regressions explain about 47 and 51 percent of the variance of consumption in Kilimanjaro and Ruvuma respectively. The regressions of the squared residuals from the consumption regressions on the same explanatory variables as the ones in the consumption regressions (excluding the variables that are related to covariate and idiosyncratic shocks) reveal that fewer of the variables are significant. In Kilimanjaro the dependency ratio, the value of the dwelling, the number of small animals, and the membership in a social group are significant, while in Ruvuma, the only two significant variables are the dummies for whether the household receives remittances and whether the household has easy access to seasonal credit. The regressions explain a rather small proportion of the error less than 10 percent in both regions). This suggests that unexplained components of consumption variability dominate any parts that maybe due to structural household specific factors.<br>Tables 7 and 8 indicate the average vulnerability index in Kilimanjaro and Ruvuma by district, along with the proportions of the variance of consumption that are due to covariate factors, the average consumption per capita and the average headcount measures of poverty rates in both years of the survey. The first observation is that average vulnerability in Kilimanjaro is much lower than in Ruvuma (31 percent versus 60 percent). This is in line with the much larger poverty incidence in Ruvuma compared to Kilimanjaro that was indicated earlier (63.3 percent versus 39.5 percent).

Poverty

Selection of most useful operationalizations (to be completed by expert reviewer)	
**Construct**	**Poverty**
1st preference	[Author (year)]
2nd preference	[Author (year)]
3rd preference	[Author (year)]

**Structured summary of candidate operationalizations**			
**Candidate article:** Deressa et al			
**Construct operationalized:** Poverty			
Sub-constructs	Intermediate constructs		
	Directly operationalized constructs		
Conceptual framework	DIRECTLY OPERATIONALIZED		
Operationalization of sub-constructs			
25. •	Data collection	n/a	
	Operational questions	Using the procedures discussed in Section 3 (applied through the STATA software), we estimate the probability of a household falling below a given level of income (poverty line), and perform a sensitivity analysis by examining this probability using four different minimum levels of income (poverty lines). The choice of minimum levels of income is based on different assumptions such as the international poverty line of 1.25 US per day (World Bank, 2008), average income of the surveyed households and arbitrary values above and below the average income of the surveyed households. The results are plotted in Figures 3 to 6.<br>[...]<br>Figure 3. Vulnerability (income at 2 USD per day or 6570 Ethiopian Birr per year) plotted against Ln (income)<br>[...]<br>Figure 4. Vulnerability (income at 1.5 USD per day or 4928 Ethiopian Birr per year) plotted against Ln (income)<br>[...]<br>Figure 5. Vulnerability (income at 1.25 USD per day or 4471 Ethiopian Birr per year) plotted against Ln (income)<br>[...]<br>Figure 6. Vulnerability (income at 0.3 USD per day or 900 Ethiopian Birr per year) plotted against Ln (income)	
Candidate-level Analysis	Using the procedures discussed in Section 3 (applied through the STATA software), we estimate the probability of a household falling below a given level of income (poverty line), and perform a sensitivity analysis by examining this probability using four different minimum levels of income (poverty lines). The choice of minimum levels of income is based on different assumptions such as the international poverty line of 1.25 US per day (World Bank, 2008), average income of the surveyed households and arbitrary values above and below the average income of the surveyed households. The results are		

	plotted in Figures 3 to 6.<br>[...]<br>Figure 3. Vulnerability (income at 2 USD per day or 6570 Ethiopian Birr per year) plotted against Ln (income)<br>[...]<br>Figure 4. Vulnerability (income at 1.5 USD per day or 4928 Ethiopian Birr per year) plotted against Ln (income)<br>[...]<br>Figure 5. Vulnerability (income at 1.25 USD per day or 4471 Ethiopian Birr per year) plotted against Ln (income)<br>[...]<br>Figure 6. Vulnerability (income at 0.3 USD per day or 900 Ethiopian Birr per year) plotted against Ln (income)

<table>
<tr><td colspan="5">Structured summary of candidate operationalizations</td></tr>
<tr><td colspan="5">Candidate article: Mutsvangwa</td></tr>
<tr><td colspan="5">Construct operationalized: Poverty</td></tr>
<tr><td colspan="2" rowspan="2">Sub-constructs</td><td>Intermediate constructs</td><td colspan="2"></td></tr>
<tr><td>Directly operationalized constructs</td><td colspan="2">Cereal Production</td></tr>
<tr><td colspan="2">Conceptual framework</td><td colspan="3">This study uses the household’s cereal production levels as a measure of welfare.</td></tr>
<tr><td colspan="2">Operationalization of sub-constructs</td><td colspan="3"></td></tr>
<tr><td rowspan="2">26.</td><td rowspan="2">Cereal production</td><td>Data collection</td><td colspan="2">The primary data used in this study was obtained from a survey carried out in September 2009. The survey gathered qualitative and quantitative data pertaining to social, demographic and economic aspects of the households, agriculture activities, farmers’ perceptions of climate change and the role of local organizations in helping smallholder farmers develop strategies to mitigate against the negative climate change.</td></tr>
<tr><td>Operational questions</td><td colspan="2">Data on production/acquisition of cereals, household size and asset ownership was gathered, as summarized in Table 5. This data was gathered using the household questionnaire.

<table><tr><th>Type of data</th><th>Specific data collected</th></tr><tr><td>Agriculture production</td><td>Arable land owned; crops grown and areas allocated to the crops; yields obtained; farming implements available; availability of draft power; livestock owned; crop management practices</td></tr></table></td></tr>
<tr><td colspan="2">Candidate-level Analysis</td><td colspan="3">The cleaned data was then analyzed by running descriptive statistics; mainly frequencies, descriptive and crosstabs.
[...]
The other analyses carried out involved running the 2 stage least squares regression model using SPSS to find estimates for the vulnerability model. This involved a double regression of the per capita cereal production levels against household observable characteristics such as age, gender, education status of the household head, assess to extension services and other factors that were considered pertinent in influencing cereal production. The estimates obtained from the 2 stage least regression was used to</td></tr>
</table>

<table>
<tr><td></td><td>measure the degree of each household's vulnerability to food insecurity. The estimated probability was given by:</td></tr>
</table>

<table>
<tr><td colspan="5">Structured summary of candidate operationalizations</td></tr>
<tr><td colspan="5">Candidate article: Sarris & karfakis</td></tr>
<tr><td colspan="5">Construct operationalized: Poverty</td></tr>
<tr><td colspan="2" rowspan="2">Sub-constructs</td><td colspan="2">Intermediate constructs</td><td></td></tr>
<tr><td colspan="2">Directly operationalized constructs</td><td></td></tr>
<tr><td colspan="2">Conceptual framework</td><td colspan="3">DIRECTLY OPERATIONALIZED</td></tr>
<tr><td colspan="2">Operationalization of sub-constructs</td><td colspan="3"></td></tr>
<tr><td rowspan="2">27.</td><td rowspan="2">•</td><td>Data collection</td><td colspan="2">The analysis of the paper will be based on a representative survey of 957 rural households in 45 villages done in the Kilimanjaro region, in November 2003, and a representative survey of 892 rural households in 36 villages done in the Ruvuma region in February-March 2004. The survey was repeated a year later
[...]
The questionnaire was designed to investigate the complete socio-economic characteristics of households with a particular emphasis on their vulnerability to a variety of risks.</td></tr>
<tr><td>Operational questions</td><td colspan="2">Table 1: General characteristics of rural households in Kilimanjaro and Ruvuma
Annual per capita total expenditure
Annual per capita total income</td></tr>
<tr><td colspan="2">Candidate-level Analysis</td><td colspan="3">Table 5 exhibits the results of the (instrumental variable) regressions on consumption and the squared residuals of consumption as per equations (15) and (17). The key variable for the vulnerability analysis is the coefficient in the consumption regressions of crop income per acre. Concerning the consumption per equivalent adult, it can be seen that it depends positively and significantly on aggregate crop productivity, the size of land, the size of household, several wealth variables such as the lagged value of the number of animals owned and the lagged value of consumer durables, the age of the household head (significant in Ruvuma), access to credit variables, and some education variables.
The Durbin-Wu-Hausman test of the exogeneity of the crop productivity strongly rejects the hypothesis of exogeneity, so IV is appropriate. Table 6 presents the first stage regressions for the IV estimates. We use as instruments a variety of exogenous land characteristics, as well as weather shock variables, and lagged dummies for whether the farm household used fertilizer and chemicals, as well as the lagged number of coffee and cashew trees. The Sargan test does not invalidate the use of these instruments.
It must be mentioned that in the consumption regressions the IV regression coefficient of crop income per acre is significantly larger in the IV regressions compared to the OLS estimates (the OLS estimates for these coefficients are 0.028 for Kilimanjaro and 0.174 for Ruvuma, compared to 0.144 and 0.411 for the IV regressions in table 5 for the two regions).
The consumption regressions explain about 47 and 51 percent of the variance of consumption in Kilimanjaro and Ruvuma respectively. The regressions of the squared residuals from the consumption regressions on the same explanatory variables as the ones in the consumption regressions (excluding the variables that are related to</td></tr>
</table>

	covariate and idiosyncratic shocks) reveal that fewer of the variables are significant. In Kilimanjaro the dependency ratio, the value of the dwelling, the number of small animals, and the membership in a social group are significant, while in Ruvuma, the only two significant variables are the dummies for whether the household receives remittances and whether the household has easy access to seasonal credit. The regressions explain a rather small proportion of the error less than 10 percent in both regions). This suggests that unexplained components of consumption variability dominate any parts that maybe due to structural household specific factors.<br>Tables 7 and 8 indicate the average vulnerability index in Kilimanjaro and Ruvuma by district, along with the proportions of the variance of consumption that are due to covariate factors, the average consumption per capita and the average headcount measures of poverty rates in both years of the survey. The first observation is that average vulnerability in Kilimanjaro is much lower than in Ruvuma (31 percent versus 60 percent). This is in line with the much larger poverty incidence in Ruvuma compared to Kilimanjaro that was indicated earlier (63.3 percent versus 39.5 percent).

Sensitivity

Selection of most useful operationalizations (to be completed by expert reviewer)	
**Construct**	**Sensitivity**
1st preference	[Author (year)]
2nd preference	[Author (year)]
3rd preference	[Author (year)]

<table>
<tr><td colspan="5">Structured summary of candidate operationalizations</td></tr>
<tr><td colspan="5">Candidate article: Antwi-Agyei et al</td></tr>
<tr><td colspan="5">Construct operationalized: Sensitivity</td></tr>
<tr><td colspan="2" rowspan="2">Sub-constructs</td><td>Intermediate constructs</td><td colspan="2">Livelihoods; Livelihood capital assets;</td></tr>
<tr><td>Directly operationalized constructs</td><td colspan="2">Social capital; financial capital; natural capital; physical capital; human capital</td></tr>
<tr><td colspan="2">Conceptual framework</td><td colspan="3"></td></tr>
<tr><td colspan="2">Operationalization of sub-constructs</td><td colspan="3"></td></tr>
<tr><td rowspan="2">3.</td><td rowspan="2">Social capital</td><td>Data collection</td><td colspan="2">Data presented in this paper were collected using a mixture of participatory methods such as focus group discussions, household questionnaire surveys and key informant interviews. Data collection started with a rapid rural appraisal (Chambers 1994) during which com- munity gatherings and transect walks were conducted with community members including opinion leaders at each of the 6 villages. This provided an overview of the significant social and physical features of the selected communities that influenced their livelihood activities (Sallu et al. 2009). A household questionnaire survey was used to collect both qualitative and quantitative data. The questionnaire survey assessed households’ capital assets (financial, human, natural, physical, and social). This information was used to develop a household livelihood vulnerability index (see Sect. 2.3). A total of 270 household ques- tionnaire surveys were conducted in the 6 farming communities (45 Questionnaires in each).</td></tr>
<tr><td>Operational questions</td><td colspan="2">Social capital—including connections to technical support and social resources such as networks, associations and affiliations—was assessed by counting the number of associations or groups to which the members of the household belong (Pretty and Ward 2001; Vincent 2007). It was assumed that households belonging to a high number of social groups and associations are better networked to cope with the impacts of climate change on their livelihoods activities (Adger 2003; Pretty 2003), as these represent the number of social safety nets and a form of informal grassroots insurance available to the household during climate-related crisis (e.g. Fraser 2007; Vincent 2007). Both bonding and bridging social capital were assessed. Bonding social capital is based on characteristics such as family kinship, ethnicity or nationality (Woolcock 2001). Bridging capital refers to ties to external groups and usually transcends different</td></tr>
</table>

			socioeconomic statuses, nationalities, religions, and ethnicities (Woolcock 2001). A scoring procedure for social capital followed the methods of Vincent (2007). A score of 1 was given to households that belonged to no identifiable group, 2 for those who were members of one group, 3 for membership of two groups and 4 for membership of more than three groups. While the level of interaction among the group members and the strength of the ties within such social groups could affect their usefulness, interaction and ties were beyond the scope of the assessment and were not considered.<br>[...]<br>Table 1 Indicators of household livelihood vulnerability index collected through a household survey across six communities in Ghana<br>Number of groups or associations households belong to<br>Do you belong to any social groups? Could you please list them?
4.	Financial capital	Data collection	Data presented in this paper were collected using a mixture of participatory methods such as focus group discussions, household questionnaire surveys and key informant interviews. Data collection started with a rapid rural appraisal (Chambers 1994) during which com- munity gatherings and transect walks were conducted with community members including opinion leaders at each of the 6 villages. This provided an overview of the significant social and physical features of the selected communities that influenced their livelihood activities (Sallu et al. 2009). A household questionnaire survey was used to collect both qualitative and quantitative data. The questionnaire survey assessed households' capital assets (financial, human, natural, physical, and social). This information was used to develop a household livelihood vulnerability index (see Sect. 2.3). A total of 270 household ques- tionnaire surveys were conducted in the 6 farming communities (45 Questionnaires in each).
		Operational questions	Eliciting information on financial assets was very problematic because of a lack of records on sales and memory lapses. Livestock were considered to offer readily available cash in times of crop failure due to erratic rainfall patterns in the study communities.<br>[...]<br>Households without poultry or livestock scored 1 whilst those with livestock scored 2. In addition, financial assets were assessed by examining the remittances received by the household from family members or friends over the past 12 months.<br>[...]<br>Households that received remittances in the last 12 months scored 2 and those that did not receive any remittances scored 1. Access to credit may also influence adaptation to climate change including access to inputs such as improved cultivars of crops (e.g. Butt et al. 2006; Fosu-Mensah et al. 2012). Hence, it is assumed that households that have no

			access to credit will be more vulnerable and scored 1 whilst those with access to credit were given a score of 2.<br>[...]<br>Table 1 Indicators of household livelihood vulnerability index collected through a household survey across six communities in Ghana<br>[...]<br>Access to credit<br>Do you have access to credit for your agricultural activities?<br>Ownership of livestock<br>Do you have livestock or poultry? List the types and numbers of livestock.<br>Remittances received<br>Have you received remittances from family or friends in the last 12 months?
5.	Natural capital	Data collection	Data presented in this paper were collected using a mixture of participatory methods such as focus group discussions, household questionnaire surveys and key informant interviews. Data collection started with a rapid rural appraisal (Chambers 1994) during which com- munity gatherings and transect walks were conducted with community members including opinion leaders at each of the 6 villages. This provided an overview of the significant social and physical features of the selected communities that influenced their livelihood activities (Sallu et al. 2009). A household questionnaire survey was used to collect both qualitative and quantitative data. The questionnaire survey assessed households' capital assets (financial, human, natural, physical, and social). This information was used to develop a household livelihood vulnerability index (see Sect. 2.3). A total of 270 household ques- tionnaire surveys were conducted in the 6 farming communities (45 Questionnaires in each).
		Operational questions	Natural capital assets were assessed by two indicators. The first was the size of the farm holding under cultivation (this was estimated as the average area of cultivated land over the past 5 years) (Table 1). It is assumed that the larger the farm holding, the greater the opportunity for the household to have more crops and yield, and hence the lower the vulnerability to climate change, though it is noted that labour availability and financial capital both affect the reality of how much land can be cultivated. Households which cultivated less than 5 acres scored 1; those cultivating between 5 and 10 acres scored 2; those cultivating between 11 and 15 acres scored 3; those cultivating 16-20 acres scored 4, and households cultivating [20 acres scored 5. The type of land tenure and level of security it provides may have serious implications for the management of agricultural soils and could indirectly affect crop productivity and environmental sustainability, conse- quently influencing household vulnerability (Butt et al. 2006). Three different tenure arrangements were identified in the study communities. These were ''land inherited'', ''land purchased'' and ''land rented'' by the household. A score of 1 was given to

			households who rented their farmlands; 2 for households who purchased their farmlands; and 3 for those who inherited their farmlands. Households that inherited their farm lands were given the highest score because it is assumed that they will have the most secure land tenure.<br>[...]<br>Table 1 Indicators of household livelihood vulnerability index collected through a household survey across six communities in Ghana<br>[...]<br>Farm holding size<br>Could you please state the size of farm holding in acres?<br>Tenure system<br>By what arrangements do you have access to your farm land for farming activities?
6.	Physical capital	Data collection	Data presented in this paper were collected using a mixture of participatory methods such as focus group discussions, household questionnaire surveys and key informant interviews. Data collection started with a rapid rural appraisal (Chambers 1994) during which com- munity gatherings and transect walks were conducted with community members including opinion leaders at each of the 6 villages. This provided an overview of the significant social and physical features of the selected communities that influenced their livelihood activities (Sallu et al. 2009). A household questionnaire survey was used to collect both qualitative and quantitative data. The questionnaire survey assessed households' capital assets (financial, human, natural, physical, and social). This information was used to develop a household livelihood vulnerability index (see Sect. 2.3). A total of 270 household ques- tionnaire surveys were conducted in the 6 farming communities (45 Questionnaires in each).
		Operational questions	Physical assets that were assessed included the presence of irrigation facilities and own- ership of radios, television or mobile phones by a household (Table 1). Irrigation facilities are crucial for rain-fed agriculture-dependent households, as these facilities help farmers to practise dry season farming. It is assumed that households with irrigation facilities will be less vulnerable to changing rainfall patterns. Hence, households without irrigation facilities scored 1, whilst those with these facilities scored 2. The presence of radios, television or mobile phone in a rural household can be an effective tool for communication and accessing information on changing weather patterns (see Naab and Koranteng 2012). Here, households with any of these three assets scored 2, and those without any scored 1. Physical assets such as road networks and the availability of markets and health facilities may enhance the adaptive capacity of a household (see Zhang et al. 2007). These assets were not included in the vulnerability computation because field observations suggested that these physical assets did not significantly differ amongst either the resilient or vul- nerable communities.<br>[...]

			Irrigation facilities<br>Do you have access to irrigation facilities for dry season farming?<br>Ownership of radio, television or mobile phone<br>Could you please list all communication gadgets that you have? These include TV, mobile phone or radios etc.
7.	Human capital	Data collection	Data presented in this paper were collected using a mixture of participatory methods such as focus group discussions, household questionnaire surveys and key informant interviews. Data collection started with a rapid rural appraisal (Chambers 1994) during which com- munity gatherings and transect walks were conducted with community members including opinion leaders at each of the 6 villages. This provided an overview of the significant social and physical features of the selected communities that influenced their livelihood activities (Sallu et al. 2009). A household questionnaire survey was used to collect both qualitative and quantitative data. The questionnaire survey assessed households' capital assets (financial, human, natural, physical, and social). This information was used to develop a household livelihood vulnerability index (see Sect. 2.3). A total of 270 household ques- tionnaire surveys were conducted in the 6 farming communities (45 Questionnaires in each).
		Operational questions	Human capital assets were represented by two indicators: the educational level of the head of the household (or the most educated person in the household) and the health status of the household (Table 1). No formal education was afforded a value of 1; 2 in the case of only primary education; 3 in the case of secondary education; and 4 for households that had tertiary education.<br>[...]<br>To assess health status, households were asked about the number of times they have been to the hospital (or hospitalised) within the last 12 months. House- holds with members that had been to the hospital were scored 1 whilst those with members that had not been to hospital as out patients (and those not needing any medical attention) within this period were scored 2. Also, situations where members of a household required hospital treatment but could not arrange transport and other resources needed were taken into consideration when scoring such a household.<br>[...]<br>Table 1 Indicators of household livelihood vulnerability index collected through a household survey across six communities in Ghana<br>[...]<br>Educational level<br>Could you please state the highest education attained?<br>Health status<br>Have any member of this household been ill in the last 12 months?
Candidate-level		To ensure the comparability of indicators that were used in the construction of the	

Analysis	household livelihood vulnerability index, all indicators were standardised following the UNDP (2007) procedure of standardising indicators for life expectancy index (Eq. 1).<br>[...]<br>Having standardised the indicators, it was then necessary to elicit appropriate weights to them. An unequal weighting system, based on relative importance attached to each indicator by local households, extension officers, key informants and experts was used because it was deemed necessary to include the views of both local households and experts in the assessment. Hence, a five-point Likert scale was used where farmers, extension officers, key informants, and experts were asked to rank the five most important indicators that they considered to influence vulnerability at the household level (Table 2). The number of times a particular indicator was cited was used to generate the weighting system (Table 2). The following weights were assigned: 14 % to social capital, 11 % to human capital, 9 % to natural capital, 27 % to financial capital, 10 % to physical capital and 29 % to livelihood diversification (Table 2). The household livelihood vulnerability index for a household was then calculated using the following model (Eq. 2) (Vincent 2004).<br>HLVI ¼ Ssvi□Wi<br>ðÞþðHsvi□WiiÞþ ðNsvi□WiiiÞþðFsvi□WivÞþðPsvi□WvÞ þðLsvi□WviÞ<br>[...]<br>Quanti- tative data were transcribed and analysed using SPSS and Minitab (Edition 15). Using Minitab, a one-way ANOVA was computed to compare the relative vulnerability among the various households and communities, and all differences resulting in p\0.05 were considered statistically significant. K-means cluster analysis using STATISTICA software was undertaken to group the households according to their vulnerability. K-means cluster analysis, which seeks to group cases into distinct clusters by seeking groups that minimise variability within clusters and maximise variability between clusters (Levia and Page 2000), has been applied to spatial vulnerability assessment in dynamic systems (see Antwi- Agyei et al. 2012).

**Structured summary of candidate operationalizations**				
**Candidate article:** Hahn et al				
**Construct operationalized:** Sensitivity				
Sub-constructs		Intermediate constructs	Food; health; water	
		Directly operationalized constructs	Food from family farm; struggle for food; crop diversity; dont save crops; dont save seeds; Family with chronic illness; proximity to health facility; 2 weeks illness; malaria exposure-prevention; Water conflict; natural water source; proximity to water source; inconsistent water supply; inverse water stored	
Conceptual framework		sensitivity is measured by assessing the current state of a district's food and water security and health status.		
Operationalization of sub-constructs				
28	Food from family farm	Data collection	household surveys	
		Operational questions	Table 1 includes an explanation of how each sub-component was quantified, the survey question used to collect the data, the original source of the survey question, and potential sources of bias.	

<table>
<tr><td></td><td></td><td></td><td>[...]
<table><tr><td colspan="3">Table 1 Major components and sub-components comprising the Livelihood Vulnerability Index (LVI) developed for two districts of Mozambique.</td></tr><tr><td>Sub-components</td><td>Explanation of sub-components</td><td>Survey question</td></tr><tr><td>Percent of households dependent on family farm for food</td><td>Percentage of households that get their food primarily from their personal farms.</td><td>Where does your family get most of its food?</td></tr></table></td></tr>
<tr><td rowspan="2">29</td><td rowspan="2">struggle for food</td><td>Data collection</td><td>household surveys</td></tr>
<tr><td>Operational questions</td><td>Table 1 includes an explanation of how each sub-component was quantified, the survey question used to collect the data, the original source of the survey question, and potential sources of bias.
[...]
<table><tr><td colspan="3">Table 1 Major components and sub-components comprising the Livelihood Vulnerability Index (LVI) developed for two districts of Mozambique.</td></tr><tr><td>Sub-components</td><td>Explanation of sub-components</td><td>Survey question</td></tr><tr><td>Average number of months households struggle to find food (range: 0–12)</td><td>Average number of months households struggle to obtain food for their family.</td><td>Does your family have adequate food the whole year, or are there times during the year that your family does not have enough food? Howmanymonths a year does your family have trouble getting enough food?</td></tr></table></td></tr>
<tr><td rowspan="2">30</td><td rowspan="2">crop diversity</td><td>Data collection</td><td>household surveys</td></tr>
<tr><td>Operational questions</td><td>Table 1 includes an explanation of how each sub-component was quantified, the survey question used to collect the data, the original source of the survey question, and potential sources of bias.
[...]
<table><tr><td colspan="3">Table 1 Major components and sub-components comprising the Livelihood Vulnerability Index (LVI) developed for two districts of Mozambique.</td></tr><tr><td>Sub-components</td><td>Explanation of sub-components</td><td>Survey question</td></tr><tr><td>Average Crop</td><td>The inverse of</td><td>What kind of</td></tr></table></td></tr>
</table>

<table>
<tr><td></td><td></td><td></td><td><table><tr><td>Diversity Index (range: >0–1)a</td><td>(the number of crops grown by a household +1). e.g., A household that grows pumpkin, maize, nhemba beans, and cassava will have a Crop Diversity</td><td>crops does your household grow?</td></tr></table></td></tr>
<tr><td rowspan="2">31</td><td rowspan="2">dont save crops</td><td>Data collection</td><td>household surveys</td></tr>
<tr><td>Operational questions</td><td>Table 1 includes an explanation of how each sub-component was quantified, the survey question used to collect the data, the original source of the survey question, and potential sources of bias.
[...]
<table><tr><td colspan="3">Table 1 Major components and sub-components comprising the Livelihood Vulnerability Index (LVI) developed for two districts of Mozambique.</td></tr><tr><td>Sub-components</td><td>Explanation of sub-components</td><td>Survey question</td></tr><tr><td>Percent of households that do not save crops</td><td>Percentage of households that do not save crops from each harvest.</td><td>Does your family save some of the crops you harvest to eat during a different time of year?</td></tr></table></td></tr>
<tr><td rowspan="2"></td><td rowspan="2">dont save seeds</td><td>Data collection</td><td>household surveys</td></tr>
<tr><td>Operational questions</td><td>Table 1 includes an explanation of how each sub-component was quantified, the survey question used to collect the data, the original source of the survey question, and potential sources of bias.
[...]
<table><tr><td colspan="3">Table 1 Major components and sub-components comprising the Livelihood Vulnerability Index (LVI) developed for two districts of Mozambique.</td></tr><tr><td>Sub-components</td><td>Explanation of sub-components</td><td>Survey question</td></tr><tr><td>Percent of households that do not save seeds</td><td>Percentage of households that do not have seeds from year to year.</td><td>Does your family save seeds to grow the next year?</td></tr></table></td></tr>
<tr><td rowspan="2"></td><td rowspan="2">Family with chronic illness</td><td>Data collection</td><td></td></tr>
<tr><td>Operational questions</td><td></td></tr>
<tr><td rowspan="2"></td><td rowspan="2">proximity to health facility</td><td>Data collection</td><td>household surveys</td></tr>
<tr><td>Operational questions</td><td>Table 1 includes an explanation of how each sub-component was quantified, the survey question used to</td></tr>
</table>

<table>
<tr><td></td><td></td><td></td><td>collect the data, the original source of the survey question, and potential sources of bias.
[...]
<table><tr><td colspan="3">Table 1 Major components and sub-components comprising the Livelihood Vulnerability Index (LVI) developed for two districts of Mozambique.</td></tr><tr><td>Sub-components</td><td>Explanation of sub-components</td><td>Survey question</td></tr><tr><td>Average time to health facility (minutes)</td><td>Average time it takes the households to get to the nearest health facility.</td><td>Howlong does it take you to get to a health facility?</td></tr></table></td></tr>
<tr><td rowspan="2"></td><td rowspan="2">2 weeks illness</td><td>Data collection</td><td>household surveys</td></tr>
<tr><td>Operational questions</td><td>Table 1 includes an explanation of how each sub-component was quantified, the survey question used to collect the data, the original source of the survey question, and potential sources of bias.
[...]
<table><tr><td colspan="3">Table 1 Major components and sub-components comprising the Livelihood Vulnerability Index (LVI) developed for two districts of Mozambique.</td></tr><tr><td>Sub-components</td><td>Explanation of sub-components</td><td>Survey question</td></tr><tr><td>Percent of households where a family member had to miss work or school in the last 2 weeks due to illness</td><td>Percentage of households that report at least 1 family member who had to miss school of work due to illness in the last 2 weeks.</td><td>Has anyone in your family been so sick in the past 2 weeks that they had to miss work or school?</td></tr></table></td></tr>
<tr><td rowspan="2"></td><td rowspan="2">malaria exposure-prevention</td><td>Data collection</td><td>household surveys</td></tr>
<tr><td>Operational questions</td><td>Table 1 includes an explanation of how each sub-component was quantified, the survey question used to collect the data, the original source of the survey question, and potential sources of bias.
[...]
<table><tr><td colspan="3">Table 1 Major components and sub-components comprising the Livelihood Vulnerability Index (LVI) developed for two districts of Mozambique.</td></tr><tr><td>Sub-components</td><td>Explanation of sub-components</td><td>Survey question</td></tr><tr><td>Average Malaria Exposure*Prevention Index (range: 0–12)</td><td>Months reported exposure to malaria*Owning at least one</td><td>Which months of the year is malaria particularly</td></tr></table></td></tr>
</table>

<table>
<tr><td></td><td></td><td></td><td><table><tr><td></td><td>bednet indicator (have bednet = 0.5, no bednet = 1) (e.g., Respondent reported malaria is a problem January–March and they do not own a bednet = 3*1 = 3).</td><td>bad? How many mosquito nets do you have?</td></tr></table></td></tr>
<tr><td rowspan="2"></td><td rowspan="2">Water conflict</td><td>Data collection</td><td>household surveys</td></tr>
<tr><td>Operational questions</td><td>Table 1 includes an explanation of how each sub-component was quantified, the survey question used to collect the data, the original source of the survey question, and potential sources of bias.
[...]
<table><tr><td colspan="3">Table 1 Major components and sub-components comprising the Livelihood Vulnerability Index (LVI) developed for two districts of Mozambique.</td></tr><tr><td>Sub-components</td><td>Explanation of sub-components</td><td>Survey question</td></tr><tr><td>Percent of households reporting water conflicts</td><td>Percentage of households that report having heard about conflicts over water in their community.</td><td>In the past year, have you heard about any conflicts over water in your community?</td></tr></table></td></tr>
<tr><td rowspan="2"></td><td rowspan="2">natural water source</td><td>Data collection</td><td>household surveys</td></tr>
<tr><td>Operational questions</td><td>Table 1 includes an explanation of how each sub-component was quantified, the survey question used to collect the data, the original source of the survey question, and potential sources of bias.
[...]
<table><tr><td colspan="3">Table 1 Major components and sub-components comprising the Livelihood Vulnerability Index (LVI) developed for two districts of Mozambique.</td></tr><tr><td>Sub-components</td><td>Explanation of sub-components</td><td>Survey question</td></tr><tr><td>Percent of households that utilize a natural water source</td><td>Percentage of households that report a creek, river, lake, pool, or hole as their primary water source.</td><td>Where do you collect your water from?</td></tr></table></td></tr>
<tr><td></td><td>proximity to water source</td><td>Data collection</td><td>household surveys</td></tr>
</table>

<table>
<tr><td></td><td></td><td>Operational questions</td><td>Table 1 includes an explanation of how each sub-component was quantified, the survey question used to collect the data, the original source of the survey question, and potential sources of bias.
[...]
<table><tr><td colspan="3">Table 1 Major components and sub-components comprising the Livelihood Vulnerability Index (LVI) developed for two districts of Mozambique.</td></tr><tr><td>Sub-components</td><td>Explanation of sub-components</td><td>Survey question</td></tr><tr><td>Average time to water source (minutes)</td><td>Average time it takes the households to travel to their primary water source.</td><td>How long does it take to get to your water source?</td></tr></table></td></tr>
<tr><td rowspan="2"></td><td rowspan="2">inconsistent water supply</td><td>Data collection</td><td>household surveys</td></tr>
<tr><td>Operational questions</td><td>Table 1 includes an explanation of how each sub-component was quantified, the survey question used to collect the data, the original source of the survey question, and potential sources of bias.
[...]
<table><tr><td colspan="3">Table 1 Major components and sub-components comprising the Livelihood Vulnerability Index (LVI) developed for two districts of Mozambique.</td></tr><tr><td>Sub-components</td><td>Explanation of sub-components</td><td>Survey question</td></tr><tr><td>Percent of households that do not have a consistent water supply</td><td>Percentage of households that report that water is not available at their primary water source everyday</td><td>Is this water available everyday?</td></tr></table></td></tr>
<tr><td rowspan="2"></td><td rowspan="2">inverse water stored</td><td>Data collection</td><td>household surveys</td></tr>
<tr><td>Operational questions</td><td>Table 1 includes an explanation of how each sub-component was quantified, the survey question used to collect the data, the original source of the survey question, and potential sources of bias.
[...]
<table><tr><td colspan="3">Table 1 Major components and sub-components comprising the Livelihood Vulnerability Index (LVI) developed for two districts of Mozambique.</td></tr><tr><td>Sub-components</td><td>Explanation of sub-components</td><td>Survey question</td></tr><tr><td>Inverse of the average number of liters of water stored per</td><td>The inverse of (the average number of liters of water stored</td><td>What containers do you usually store water in? How many?</td></tr></table></td></tr>
</table>

			household (range: >0–1)	by each household + 1).	How many liters are they?

Candidate-level Analysis	Rather than merge the major components into the LVI in one step, they are first combined according to the categorization scheme in Table 2 using the following equation:<br>$CF_d = [\sum^n_{i=1} w_{Mj} M_{di}]/[\sum^n_{i=1} w_{Mi}]$<br>where CFd is an IPCC-defined contributing factor (exposure, sensitivity, or adaptive capacity) for district d, Mdi are the major components for district d indexed by i, wMi is the weight of each major component, and n is the number of major components in each contributing factor.

**Structured summary of candidate operationalizations**			
**Candidate article:** Piya et al			
**Construct operationalized:** Sensitivity			
Sub-constructs	Intermediate constructs		
	Directly operationalized constructs		
Conceptual framework	DIRECTLY OPERATIONALIZED		
Operationalization of sub-constructs			
32.	•	Data collection	This study is based on the primary data collected by household survey conducted in<br>two phases. The first phase of household survey was conducted in February-March 2010 and the second phase in May-June 2011.
		Operational questions	3.2.2 Sensitivity<br>Sensitivity is given by the degree to which a system is modified or affected by an internal or external disturbance or set of disturbances (Gallopin, 2003). Livelihood impacts of climate related disasters were taken as the sensitivity indicator following Daze, Ambrose, & Ehrhart (2009) and Marshall et al. (2009). Deaths of family members and loss of properties (viz. land, livestock, and crop) due to climate related disasters over the last ten years represent the sensitivity for the purpose of this study. It is hypothesized that higher impacts of past climatic hazards will increase the sensitivity of the households to such events. The income structure will also determine the household sensitivity. Higher share of natural resource based income (composed of agriculture, livestock, forest, honey and handicrafts) will increase the sensitivity of the household as these sources are more dependent on climate; while higher share of non-natural resource based remunerative income sources (composed of salaried jobs, non-farm skilled jobs, and remittances from abroad) will reduce the sensitivity. These three income sources are categorized as remunerative sources because the return from these sources is comparatively higher than other sources of income. It was found that the annual income of the households having any of these three sources is higher compared to other households with no income from any of these three sources (Piya, Maharjan, & Joshi, 2011b). The detailed breakdown of the share of various income sources are given in Appendix 2.<br>Table 2. Indicators for sensitivity

Component Indicators	Description of the Indicators	Unit	Hypothesized relation
Fatalities	Death of family members due to climate related disasters (floods, landslides) over the last 10 years	Number of family members	+
Damage to properties	Total land damaged by flood/landslides over the last 10 years	Area in local units (Kattha4)	+
	Total livestock death due to flood/landslides/drought/hail over the last 10 years	Livestock Standard Unit (LSU5)	+
	Total crop damage due to flood/ landslides/ drought/ hail over the last 10 years	Value in Nepali Rupees (NRs6)	+
Income structure	Share of natural resource based income (agriculture, livestock, forest, honey, and handicraft) to total income	%	+
	Share of non-natural based remunerative income (salaried job, remittance, skilled non-farm job) to total income	%	-

4 1 Kattha = 0.033 ha
5 LSU is aggregates of different types of livestock kept at kept at household in standard unit calculated using the following equivalents; 1 adult buffalo = 1 LSU, 1 immature buffalo = 0.5 LSU, 1 Cow = 0.8 LSU, 1 calf = 0.4 LSU, 1 pig = 0.3 LSU, 1 sheep or goat = 0.2 LSU and 1 poultry = 0.1 LSU (CBS, 2003; Baral, 2005).
6 73 NRs = 1 US $ at the time of field survey.

Candidate-level Analysis

Having chosen the suitable indicators, now these need to be normalized so as to bring the values of the indicators within the comparable range (Nelson, et al., 2010b; Gbetibouo & Ringler, 2009; Vincent, 2004). Normalization is done by subtracting the mean from the observed value and dividing by the standard deviation for each indicator.

$Normalized\ value\ = Observed\ Value\ /\ Mean\ standard\ deviation$

Next, weights should be assigned to these indicators.

[...]

The normalized variables are then multiplied with the assigned weights to construct the indices (for exposure, sensitivity, and adaptive capacity separately) using the following formulae:

$Ij = \sum^{k}_{i=1} b_i[(a_{ji} - x_i)/s_i]$

where, ‘I’ is the respective index value, ‘b’ is the loadings from first component from PCA (PCA1) taken as weights for respective indicators, ‘a’ is the indicator value, ‘x’ is the mean indicator value, and ‘s’ is the standard deviation of the indicators. Finally, vulnerability index for each household is calculated as: V = E + S – AC, where, V is the vulnerability index, E the

	exposure index, S is the sensitivity index and AC is the adaptive capacity index for respective household.

**Structured summary of candidate operationalizations**			
**Candidate article:** Sietz et al			
**Construct operationalized:** Sensitivity			
Sub-constructs	Intermediate constructs		
	Directly operationalized constructs		
Conceptual framework	DIRECTLY OPERATIONALIZED		
Operationalization of sub-constructs			
33. •	Data collection	The ALTAGRO (2006) data base contains detailed quantitative information for 527 smallholder households collected through household questionnaires.	
	Operational questions	Ten categories describe the smallholder households covering personal information about the family members (e.g. occupation, education level, age), production systems (e.g. crop and livestock assets, labour input, processing and commer- cialisation of produce), weather conditions, food reserves, income, some expenses and credits.<br>[...]<br>The following data are taken from the ALTAGRO (2006) data base to indicate the mechanisms relevant in this study. As the first dimension, the harvest failure risk is indicated by the number of production zones used for crop and pasture cultivation. The indicator considers plains, hillsides and hills. The second dimension of the area con- straint is measured by the crop area as an important pre- requisite for food production. The pasture area highly correlates to livestock keeping and is therefore reflected in the livestock measure. The third dimension, the livestock constraint, is characterised by the number and types of animals. To compare various animal species, we calculated standardised livestock units in relation to an improved cattle variety based on the livestock-specific metabolism (Kleiber 1961). Average livestock weights were estimated using 20 representative animals of each species in the study region. Since fodder production is an essential condition for livestock keeping, the respective indicator contains a reference to the area and productivity of pasture land. Furthermore, the productivity constraint as the fourth dimension is provided for the major food crops potatoes and quinua. It averages the household's productivity across species, varieties and production zones for each crop. Again, we concentrate on food crops since the productivity of pastures is already included in the livestock measure. The fifth dimension of education deprivation relates to the number of years that a household head attended school. School attendance is classified according to the four levels: no formal education, primary, secondary and higher edu- cation. Finally, the lack of	

alternative income as the sixth dimension is quantified by the sum of annual monetary income from local off-farm activities and remittances. People usually receive remittances from household
495
members who migrate for climate-independent labour, for example mining and commerce. Table 1 summarises the indicators used to assess vulnerability.
[...]

Table 1 Indicators of households' sensitivity and adaptive capacity. The range of the area and livestock constraints as well as lack of alternative income is provided following winsorisation, see description in text. (Data source: ALTAGRO 2006)

Dimension of sensitivity and adaptive capacity	*Indicator*	*Range*
Harvest failure risk	Number of production zones used for cultivation	1–3
Area constraint	Crop area	0.1–1.3 ha/person[a]
Livestock constraint	Livestock units	0.1–8.0 livestock units/person
Productivity constraint	Potato productivity<br>Quinua productivity	0.1–10.0 t/ha<br>0.2–1.8 t/ha
Education deprivation	Education level of household head	1–4
Lack of alternative income	Local off-farm income and remittances	0–2400 Soles/year*person

a Average: 4 persons per household

Candidate-level Analysis

In preparing the further analysis, we adjusted data sets with only a few extreme values to increase the influence of these data sets on the cluster partitions. For example, the majority of households possess eight or fewer units of livestock. The few households with up to 39 livestock units can be formally interpreted as single outliers which skew the overall data distribution of this indicator. To deskew such data sets and thus adequately focus on the majority of households, we winsorised the data sets, i.e., replaced the outlying observations (4%) with the next available less extreme observation (Barnett and Lewis 1994). This pro- cedure was applied to the area and livestock constraints as well as the alternative income. All indicators were then normalised to a 0–1 range using the minimum–maximum values. Prior to the cluster analysis, we determined correlations
between the selected indicators and the variance distribu- tion in the data space. Firstly, the correlation coefficients reached average absolute values of 0.11. The crop area and

	livestock units correlate most strongly here (0.46) reflec- ting the mixed production systems. Furthermore, variables showing a large variance may be intuitively expected to contain most of the structure information. Therefore, we explored the variance of the selected indicators using a principal component analysis (PCA). The PCA was per- formed using the open source statistics package R (RDCT 2009) following standard procedure based on Pearson correlations.

Vulnerability (IPCC)

Selection of most useful operationalizations (to be completed by expert reviewer)	
**Construct**	**Vulnerability (IPCC)**
1st preference	[Author (year)]
2nd preference	[Author (year)]
3rd preference	[Author (year)]

An explanatory note: In this section, many operationalizations are built on constructs that are dealt with elsewhere in this document. When choosing among candidates here, the choice is effectively between choosing between a Vulnerability IPCC framework that is operationalized in a specific way here, or one that is operationalized using constructs for which themselves there are multiple candidate operationalizations. This section is therefore best completed after choices have been made for the sub-constructs, and when making a choice here, consider your first preference from those sub-constructs.

**Structured summary of candidate operationalizations**			
**Candidate article:** Antwi-Agyei et al			
**Construct operationalized:** Vulnerability (IPCC)			
Sub-constructs		Intermediate constructs	Sensitivity; Livelihoods; Livelihood capital assets
		Directly operationalized constructs	diversified livelihood activities; exposure-SEE CANDIDATE SECTION; Social capital; financial capital; natural capital; physical capital; human capital
Conceptual framework			
Operationalization of sub-constructs			
34.	diversified livelihood activities	Data collection	Data presented in this paper were collected using a mixture of participatory methods such as focus group discussions, household questionnaire surveys and key informant interviews. Data collection started with a rapid rural appraisal (Chambers 1994) during which com- munity gatherings and transect walks were conducted with community members including opinion leaders at each of the 6 villages. This provided an overview of the significant social and physical features of the selected communities that influenced their livelihood activities (Sallu et al. 2009). A household questionnaire survey was used to collect both qualitative and quantitative data. The questionnaire survey assessed households' capital assets (financial, human, natural, physical, and social). This information was used to develop a household livelihood vulnerability index (see Sect. 2.3). A total of 270 household ques- tionnaire surveys were conducted in the 6 farming communities (45 Questionnaires in each).
		Operational questions	In addition to exploring the five capital assets, this study also examined whether house- holds in resilient and vulnerable communities diversified their livelihood activities. This is important because diversification has been reported as one of

			the main strategies for reducing household vulnerability to the impacts of climate change and variability (see Ellis 1998; Barrett et al. 2001). Therefore, the number of livelihood activities that a household was engaged in was also assessed.<br>[...]<br>A score of 1 was therefore given to households that had only one livelihood activity, 2 for households having two livelihood activities, 3 for those with three livelihood activities, 4 for those with four livelihood activities, and households with[4 livelihood activities scored 5.<br>[...]<br>Table 1 Indicators of household livelihood vulnerability index collected through a household survey across six communities in Ghana<br>[...]<br>Livelihood diversity index What are your main livelihood activities? Could you rank these in terms of their contribution to household income?
35.	Exposure	Data collection	-SEE CANDIDATE SECTION
		Operational questions	
36.	Social capital	Data collection	Not valid/feasible
		Operational questions	
	financial capital	Data collection	Same as 'diversified livelihood activites'
		Operational questions	Eliciting information on financial assets was very problematic because of a lack of records on sales and memory lapses. Livestock were considered to offer readily available cash in times of crop failure due to erratic rainfall patterns in the study communities.<br>[...]<br>Households without poultry or livestock scored 1 whilst those with livestock scored 2. In addition, financial assets were assessed by examining the remittances received by the household from family members or friends over the past 12 months.<br>[...]<br>Households that received remittances in the last 12 months scored 2 and those that did not receive any remittances scored 1. Access to credit may also influence adaptation to climate change including access to inputs such as improved cultivars of crops (e.g. Butt et al. 2006; Fosu-Mensah et al. 2012). Hence, it is assumed that households that have no access to credit will be more vulnerable and scored 1 whilst those with access to credit were given a score of 2.<br>[...]<br>Table 1 Indicators of household livelihood vulnerability index collected through a household survey across six communities in Ghana<br>[...]<br>Access to credit<br>Do you have access to credit for your agricultural activities?<br>Ownership of livestock<br>Do you have livestock or poultry? List the types and numbers

			of livestock.<br>Remittances received<br>Have you received remittances from family or friends in the last 12 months?
	natural capital	Data collection	Same as ‘diversified livelihood activites’
		Operational questions	Natural capital assets were assessed by two indicators. The first was the size of the farm holding under cultivation (this was estimated as the average area of cultivated land over the past 5 years) (Table 1). It is assumed that the larger the farm holding, the greater the opportunity for the household to have more crops and yield, and hence the lower the vulnerability to climate change, though it is noted that labour availability and financial capital both affect the reality of how much land can be cultivated. Households which cultivated less than 5 acres scored 1; those cultivating between 5 and 10 acres scored 2; those cultivating between 11 and 15 acres scored 3; those cultivating 16-20 acres scored 4, and households cultivating [20 acres scored 5. The type of land tenure and level of security it provides may have serious implications for the management of agricultural soils and could indirectly affect crop productivity and environmental sustainability, conse- quently influencing household vulnerability (Butt et al. 2006). Three different tenure arrangements were identified in the study communities. These were ‘‘land inherited’’, ‘‘land purchased’’ and ‘‘land rented’’ by the household. A score of 1 was given to households who rented their farmlands; 2 for households who purchased their farmlands; and 3 for those who inherited their farmlands. Households that inherited their farm lands were given the highest score because it is assumed that they will have the most secure land tenure.<br>[...]<br>Table 1 Indicators of household livelihood vulnerability index collected through a household survey across six communities in Ghana<br>[...]<br>Farm holding size<br>Could you please state the size of farm holding in acres?<br>Tenure system<br>By what arrangements do you have access to your farm land for farming activities?
	physical capital	Data collection	Same as ‘diversified livelihood activites’
		Operational questions	Physical assets that were assessed included the presence of irrigation facilities and own- ership of radios, television or mobile phones by a household (Table 1). Irrigation facilities are crucial for rain-fed agriculture-dependent households, as these facilities help farmers to practise dry season farming. It is assumed that households with irrigation facilities will be less vulnerable to changing rainfall patterns. Hence, households without irrigation facilities scored 1, whilst those with these facilities scored 2. The presence of radios, television or mobile phone in a rural household can be an effective tool for communication and accessing information

			on changing weather patterns (see Naab and Koranteng 2012). Here, households with any of these three assets scored 2, and those without any scored 1. Physical assets such as road networks and the availability of markets and health facilities may enhance the adaptive capacity of a household (see Zhang et al. 2007). These assets were not included in the vulnerability computation because field observations suggested that these physical assets did not significantly differ amongst either the resilient or vul- nerable communities.<br>[...]<br>Irrigation facilities<br>Do you have access to irrigation facilities for dry season farming?<br>Ownership of radio, television or mobile phone<br>Could you please list all communication gadgets that you have? These include TV, mobile phone or radios etc.
	human capital	Data collection	Same as ‘diversified livelihood activites’
		Operational questions	Human capital assets were represented by two indicators: the educational level of the head of the household (or the most educated person in the household) and the health status of the household (Table 1). No formal education was afforded a value of 1; 2 in the case of only primary education; 3 in the case of secondary education; and 4 for households that had tertiary education.<br>[...]<br>To assess health status, households were asked about the number of times they have been to the hospital (or hospitalised) within the last 12 months. House- holds with members that had been to the hospital were scored 1 whilst those with members that had not been to hospital as out patients (and those not needing any medical attention) within this period were scored 2. Also, situations where members of a household required hospital treatment but could not arrange transport and other resources needed were taken into consideration when scoring such a household.<br>[...]<br>Table 1 Indicators of household livelihood vulnerability index collected through a household survey across six communities in Ghana<br>[...]<br>Educational level<br>Could you please state the highest education attained?<br>Health status<br>Have any member of this household been ill in the last 12 months?
Candidate-level Analysis		To ensure the comparability of indicators that were used in the construction of the household livelihood vulnerability index, all indicators were standardised following the UNDP (2007) procedure of standardising indicators for life expectancy index (Eq. 1).<br>[...]<br>Having standardised the indicators, it was then necessary to elicit appropriate weights to them. An unequal weighting system, based on relative importance attached to each indi- cator by local households, extension officers, key informants and experts was used	

	because it was deemed necessary to include the views of both local households and experts in the assessment. Hence, a five-point Likert scale was used where farmers, extension officers, key informants, and experts were asked to rank the five most important indicators that they considered to influence vulnerability at the household level (Table 2). The number of times a particular indicator was cited was used to generate the weighting system (Table 2). The following weights were assigned: 14 % to social capital, 11 % to human capital, 9 % to natural capital, 27 % to financial capital, 10 % to physical capital and 29 % to livelihood diversification (Table 2). The household livelihood vulnerability index for a household was then calculated using the following model (Eq. 2) (Vincent 2004).<br>HLVI ¼ Ssvi□Wi<br>ðÞþðHsvi□WiiÞþ ðNsvi□WiiiÞþðFsvi□WivÞþðPsvi□WvÞ þðLsvi□WviÞ<br>[...]<br>Quanti- tative data were transcribed and analysed using SPSS and Minitab (Edition 15). Using Minitab, a one-way ANOVA was computed to compare the relative vulnerability among the various households and communities, and all differences resulting in p\0.05 were considered statistically significant. K-means cluster analysis using STATISTICA software was undertaken to group the households according to their vulnerability. K-means cluster analysis, which seeks to group cases into distinct clusters by seeking groups that minimise variability within clusters and maximise variability between clusters (Levia and Page 2000), has been applied to spatial vulnerability assessment in dynamic systems (see Antwi- Agyei et al. 2012).

**Structured summary of candidate operationalizations**			
**Candidate article:** Baca et al			
**Construct operationalized:** Vulnerability (IPCC)			
Sub-constructs		Intermediate constructs	Exposure-SEE CANDIDATE SECTION; sensitivity-SEE CANDIDATE SECTION; adaptive capacity-SEE CANDIDATE SECTION’
		Directly operationalized constructs	
Conceptual framework		The vulnerability in the livelihoods of small coffee farmers is a function of three factors: exposure, sensitivity and adaptive capacity.	
Operationalization of sub-constructs			
37.	• Exposure	Data collection	SEE CANDIDATE SECTION’
		Operational questions	
38.	• Sensitivity	Data collection	SEE CANDIDATE SECTION’
		Operational questions	
39.	• Adaptive capacity	Data collection	SEE CANDIDATE SECTION’
		Operational questions	
40.	•	Data collection	
		Operational questions	
Candidate-level Analysis		For exposure, the relative decreases in climatic suitability according to the MAXENT model were divided into three classes of suitability loss (low, medium, high). For sensitivity and adaptive capacity, indicators were identified and quantified through interviews with the farming families.<br>[...]<br>Each factor (exposure, sensitivity and adaptive capacity), as previously explained, and was classified into three levels (high, medium, low). To calculate the vulnerability equation we assigned each level a quantitative value: low=1, medium=2, high=3. With	

	three factors and three levels per factor, we obtained 27 possible combinations. After applying the equation we obtained 7 values (–1,0,1,2,3,4,5), which we used to define low (–1,0), medium (1,2,3,) and high (4,5) levels of vulnerability (Figure 1). A Principal Components Analysis (PCA) was carried out to identify the indicators that most contribute to the sensitivity or adaptive capacity of families in different municipalities.

**Structured summary of candidate operationalizations**				
**Candidate article:** CARE (2009)				
**Construct operationalized:** Vulnerability (IPCC)				
Sub-constructs		Intermediate constructs		
		Directly operationalized constructs		Adaptive capacity- SEE CANDIDATE SECTION
Conceptual framework		Vulnerability to climate change has been defined as:<br>The degree to which a system is susceptible to, or unable to cope with, adverse effects of climate change, including climate variability and extremes. Vulnerability is a function of the character, magnitude, and rate of climate variation to which a system is exposed, its sensitivity, and its adaptive capacity.5		
Operationalization of sub-constructs				
41.	• Adaptive capacity	Data collection	SEE CANDIDATE SECTION	
		Operational questions		
Candidate-level Analysis				

**Structured summary of candidate operationalizations**				
**Candidate article:** Eakin et al (2012)				
**Construct operationalized:** Vulnerability (IPCC)				
Sub-constructs		Intermediate constructs		Adaptiveness
		Directly operationalized constructs		Adaptation strategy- SEE CANDIDATE SECTION
Conceptual framework				
Operationalization of sub-constructs				
42.	Adaptation strategy	Data collection	SEE CANDIDATE SECTION	
		Operational questions		
Candidate-level Analysis		As a heuristic tool to aid in our interpretation of impacts<br>and responses to Stan, we categorized households accord- ing to the exposure of their production systems to Hurri- cane Stan into impact clusters. The impact clusters were created using a two-step cluster method available through the statistical software, PASW 18. Two-step cluster anal- ysis uses a distance criterion (log-likelihood) to define optimal number of clusters and allows for handling a mixture of categorical and (standardized) continuous vari- ables (Zhang et al. 1996; Chiu et al. 2001).<br>[...]<br>We used two ‘‘loss’’ variables as the input data for the<br>creation of clusters: percent of coffee harvest and soil lost due to Hurricane Stan. We chose these two variables because of the fundamental economic role played by coffee production for households in Siltepec in 2005.<br>[...]<br>We then used these clusters to explore two questions through a descriptive analysis of the remaining survey variables: What were the characteristics of house- holds that		

	experienced specific degrees of loss? What were their responses?

**Structured summary of candidate operationalizations**		
**Candidate article:** Hahn et al		
**Construct operationalized:** Vulnerability (IPCC)		
Sub-constructs	Intermediate constructs	Exposure- SEE CANDIDATE SECTION; Sensitivity- SEE CANDIDATE SECTION; Adaptive capacity- SEE CANDIDATE SECTION
	Directly operationalized constructs	
Conceptual framework		
Operationalization of sub-constructs		
43. •	Data collection	
	Operational questions	
44. •	Data collection	
	Operational questions	
45. •	Data collection	
	Operational questions	
46. •	Data collection	
	Operational questions	
Candidate-level Analysis	Rather than merge the major components into the LVI in one step, they are first combined according to the categorization scheme in Table 2 using the following equation:<br>$CF_d = [\sum^{n}_{i=1} w_{Mj} M_{di}]/[\sum^{n}_{i=1} w_{Mi}]$<br>where CFd is an IPCC-defined contributing factor (exposure, sensitivity, or adaptive capacity) for district d, Mdi are the major components for district d indexed by i, wMi is the weight of each major component, and n is the number of major components in each contributing factor. Once exposure, sensitivity, and adaptive capacity were calculated, the three contributing factors were combined using the following equation:<br>$LVI - IPCC_d = (e_d - a_d)*s_d$<br>where LVI–IPCCd is the LVI for district d expressed using the IPCC vulnerability framework, e is the calculated exposure score for district d (equivalent to the Natural Disaster and Climate Variability major component), a is the calculated adaptive capacity score for district d (weighted average of the Socio-Demographic, Livelihood Strategies, and Social Networks major components), and s is the calculated sensitivity score for district d (weighted average of the Heath, Food, and Water major components). We scaled the LVI–IPCC from □1 (least vulnerable) to 1 (most vulnerable).	

**Structured summary of candidate operationalizations**		
**Candidate article:** Jamir et al		
**Construct operationalized:** Vulnerability (IPCC)		
Sub-constructs	Intermediate constructs	
	Directly operationalized constructs	Exposure- SEE CANDIDATE SECTION; sensitivity- SEE CANDIDATE SECTION; adaptive capacity- SEE

		CANDIDATE SECTION
Conceptual framework	Asper theIPCC's definition and framework, vulnerability is understood as a function of three components—exposure, sensitivity and adaptive capacity.	
Operationalization of sub-constructs		
47. Exposure	Data collection	- SEE CANDIDATE SECTION
	Operational questions	
48. Sensitivity	Data collection	- SEE CANDIDATE SECTION
	Operational questions	
49. adaptive capacity	Data collection	SEE CANDIDATE SECTION
	Operational questions	
Candidate-level Analysis		

**Structured summary of candidate operationalizations**		
**Candidate article:** Luers et al		
**Construct operationalized:** Vulnerability (IPCC)		
Sub-constructs	Intermediate constructs	
	Directly operationalized constructs	State of system relative to threshold; sensitivity; exposure-SEE CANDIDATE SECTION; adaptive capacity- SEE CANDIDATE SECTION
Conceptual framework		
Operationalization of sub-constructs		
50. State of system relative to threshold	Data collection	Not valid/feasible
	Operational questions	
51. Sensitivity	Data collection	Not valid/feasible
	Operational questions	
52. Exposure	Data collection	-SEE CANDIDATE SECTION
	Operational questions	
53. adaptive capacity	Data collection	SEE CANDIDATE SECTION
	Operational questions	
Candidate-level Analysis		

**Structured summary of candidate operationalizations**		
**Candidate article:** Notenbaert et al		
**Construct operationalized:** Vulnerability (IPCC)		
Sub-constructs	Intermediate constructs	
	Directly operationalized constructs	Exposure- SEE CANDIDATE SECTION; sensitivity- SEE CANDIDATE SECTION; adaptive capacity- SEE CANDIDATE SECTION; vulnerability outcomes
Conceptual framework		
Operationalization of sub-constructs		
54. Exposure	Data collection	SEE CANDIDATE SECTION
	Operational questions	

55.	Sensitivity	Data collection	SEE CANDIDATE SECTION
		Operational questions	
56.	adaptive capacity	Data collection	SEE CANDIDATE SECTION
		Operational questions	
57.	vulnerability outcomes	Data collection	Not transparent
		Operational questions	
Candidate-level Analysis			

**Structured summary of candidate operationalizations**				
**Candidate article:** Piya et al				
**Construct operationalized:** Vulnerability (IPCC)				
Sub-constructs		Intermediate constructs		adaptive capacity- SEE CANDIDATE SECTION
		Directly operationalized constructs		Exposure- SEE CANDIDATE SECTION; sensitivity- SEE CANDIDATE SECTION
Conceptual framework				
Operationalization of sub-constructs				
58.	Exposure	Data collection	SEE CANDIDATE SECTION	
		Operational questions		
59.	Sensitivity	Data collection	SEE CANDIDATE SECTION	
		Operational questions		
60.	adaptive capacity	Data collection	SEE CANDIDATE SECTION	
		Operational questions		
Candidate-level Analysis		Assigning weight by Principal Component Analysis (PCA) following Filmer and Pritchett (2001) is thus preferred compared to the former two methods (Nelson et al., 2010b; Gbetibouo & Ringler, 2009; Cutter, Boruff, & Shirley, 2003). PCA was run for the selected indicators of exposure, sensitivity, and adaptive capacity separately in Data Analysis and Statistical Software (STATA10) software for assigning the weights. The loadings from the first component of PCA are used as the weights for the indicators. The weights assigned for each indicator varies between -1 and +1, sign of the indicators denoting the direction of relationship with other indicators used to construct the respective index. The magnitude of the weights describes the contribution of each indicator to the value of the index. PCA was run separately for the indicators of exposure, sensitivity and adaptive capacity. Stepwise PCA was run for the indicators of adaptive capacity. The first-step PCA was run for the indicators of each asset group separately to observe the relative importance of indicators within each asset category. From the weights obtained from first-step PCA, individual index values for each asset type was calculated. Second-step PCA was run using the index values for each of the five asset types to analyze which asset group contributes the most to the total adaptive capacity. Overall adaptive capacity index was calculated using the weights (loadings) obtained from the second step PCA run for the five asset categories.<br>The normalized variables are then multiplied with the assigned weights to construct the indices (for exposure, sensitivity, and adaptive capacity separately) using the following formulae:<br>$Ij = \sum^{k}_{i=1} b_i[(a_{ji} - x_i)/s_i]$<br>where, 'I' is the respective index value, 'b' is the loadings from first component from PCA (PCA1) taken as weights for respective indicators, 'a' is the indicator value, 'x' is the mean indicator value, and 's' is the standard deviation of the indicators. Finally, vulnerability index for each household is calculated as: V = E + S – AC, where, V is the vulnerability index, E the exposure index, S is the sensitivity index and AC is the adaptive		

	capacity index for respective household.

**Structured summary of candidate operationalizations**				
**Candidate article:** Sietz et al				
**Construct operationalized:** Vulnerability (IPCC)				
Sub-constructs		Intermediate constructs		
		Directly operationalized constructs		Exposure- SEE CANDIDATE SECTION; sensitivity- SEE CANDIDATE SECTION; adaptive capacity; food security
Conceptual framework				
Operationalization of sub-constructs				
61.	Exposure	Data collection	SEE CANDIDATE SECTION	
		Operational questions		
62.	Sensitivity	Data collection	SEE CANDIDATE SECTION	
		Operational questions		
63.	adaptive capacity	Data collection	SEE CANDIDATE SECTION	
		Operational questions		
64.	food security	Data collection	Therefore, we conducted a Household Validation Survey (HVS) in collaboration with CIRNMA technicians.	
		Operational questions	We collected data on the purchase of food and fodder in 2005/2006 including monetary and in-kind exchange. The purchase was considered in relation to an average year to compare households in a standardised way. The average year indicates the necessary purchase which complements the household’s production and reserves to maintain the average nutritional status. We assume that changes in 2005/2006 were primarily caused by the iden- tified weather extremes given that the productive resources and agricultural management are relatively stable over time. As smallholders do not maintain records of their pur-<br>chase, the data collection drew on their memory recall. This approach provides good estimates in the absence of other reliable data sources, though some limitations need to be considered. Most importantly, this method does not account for memory biases. To reduce such biases, the survey referred to the purchase of a specific crop in a given year. Firstly, smallholders were asked to reflect on thecroptheyharvested last,startingwiththe previous campaign and successively moving backwards to the 2005/2006 campaign. This part of the survey was con- ducted with the aid of an abacus. Starting with the given number of 10 beads indicating the average purchase, household heads or other adult family members removed or added beads to quantify their relative purchase in 2005/2006. The survey considered the five major food and fodder crops: potatoes, quinua, broad beans, barley and oat. The second part of the HVS focused on information<br>about aspects of the smallholder livelihoods that help explain important causes for differences in purchase to support the interpretation and validation of the vulnera- bility clusters. This part involved semi-structured inter- views exploring	

			effects of weather extremes on the smallholders' livelihoods, access to land, production zones and income, availability of labour as well as social and economic opportunities to cope with production failure. Overall, each interview took around 45 min and was car- ried out in Spanish or Quechua according to the native language of the interviewees.
Candidate-level Analysis		The cluster analysis was performed using a sequence of a common hierarchical and exchange algorithm, i.e., hclust and kmeans, using the statistics package R (MacQueen 1967; RDCT 2009). Based on stochastic initialisation, we calculated the reproducibility of partitions for a pre-given number of clusters to determine whether the algorithm detects stable or unstable (inappropriate) partitions. The share of households that were categorised in the same cluster in two partitions is expressed as ''consistency measure''. The higher this measure, the more reliable the cluster results. We calculated the consistency measure as the average of 200 pairwise comparisons of partitions with a given number of clusters. Ultimately, the consistency measure enables us to identify the optimal number of clusters to be analysed. Further methodological details are outlined in a previous application of the cluster approach to dryland vulnerability on a global scale (Sietz et al. 2011).<br>[...]<br>Recognising the sensitivity of any vulnerability analysis<br>to the choice of indicators, we empirically examine whe- ther the formal entities provide specific evidence about damages under the identified climate exposure. For this, the data on households' purchase collected in the HVS are related to the cluster membership of households. Figure 4 shows that each cluster corresponds to a relatively small range of the damage measure. Therefore, the similarities among the households revealed by the cluster analysis hold true with regard to the outcomes of the climate exposure.	

Vulnerability as Expected Poverty

Selection of most useful operationalizations (to be completed by expert reviewer)	
**Construct**	Vulnerability as Expected Poverty
1st preference	[Author (year)]
2nd preference	[Author (year)]
3rd preference	[Author (year)]

**Structured summary of candidate operationalizations**			
**Candidate article:** Calvo & Dercon			
**Construct operationalized:** Vulnerability as Expected poverty			
Sub-constructs		Intermediate constructs	Individual vulnerability; aggregate vulnerability
		Directly operationalized constructs	Poverty-SEE CANDIDATE SECTION; possible states of the world; probabilities of possible states of the world.
Conceptual framework			
Operationalization of sub-constructs			
65.	Poverty	Data collection	SEE CANDIDATE SECTION
		Operational questions	
66.	possible states of the world;	Data collection	NOT TRANSPARENT
		Operational questions	
67.	probabilities of possible states of the world	Data collection	NOT TRANSPARENT
		Operational questions	
Candidate-level Analysis			

**Structured summary of candidate operationalizations**			
**Candidate article:** Chhihn & Poch			
**Construct operationalized:** Vulnerability as Expected poverty			
Sub-constructs		Intermediate constructs	
		Directly operationalized constructs	Environmental shocks; current poverty status; household characteristics; poverty- SEE CANDIDATE SECTION
Conceptual framework			
Operationalization of sub-constructs			
68.	Environmental shocks	Data collection	NOT TRANSPARENT
		Operational questions	
69.	current poverty status	Data collection	NOT TRANSPARENT
		Operational questions	
70.	household characteristics	Data collection	A total of 600 questionnaires were collected from households.
		Operational questions	There were on average five people within a household within the surveyed areas. This is well above the national average household size (4.7) in 2008 (NIS, 2008). Rolang Chork has the small- est average household size (4.99 people per household on average) and the highest level of educa- tion among its

<table>
<tr><td></td><td></td><td></td><td>population (9.6 years of schooling on average). The Kork and Chbar Mon com- munes had larger household sizes than the other selected communes (5.64 and 5.59 people per household on average, respectively). About 60% of respondents reported that their households have at least one motorcycle. There
was a large variation in the proportion of households possessing motorcycles between communes, with the Chbar Mon (73%), Peang Lvea (74%) and Rolang Chork (68%) communes having a high- er percentage of motorcycle-possessing households than the Tasal (44%), Kork (50%) and Morhasaing (53%) communes. The survey also revealed that 11.7% of respondents live in households with at least one person with disability. Peang Lvea commune has the highest proportion of households containing a person with a disability (21%), followed by Rolang Chork (13%), Kork (12%), Tasal (11%), Morhasaing (7%) and Chbar Mon (6%).</td></tr>
<tr><td rowspan="2">71.</td><td rowspan="2">Poverty</td><td>Data collection</td><td>SEE CANDIDATE SECTION</td></tr>
<tr><td>Operational questions</td><td></td></tr>
<tr><td colspan="2">Candidate-level Analysis</td><td colspan="2">The expected log per capita income is estimated using the three-step feasible generalised least squares (FGLS) method.
[...]
Table 3 presents the results of the FGLS analysis.
[...]
Household size, the possession of motor vehicle and a livelihood dependency on agriculture
are significantly and inversely associated with log per capita income. Specifically, the larger the household size, the lower the expected log per capita income (the coefficient is -0.182, p<0.001). In addition, the possession of a motor vehicle is positively related to expected per capita income (the coefficient is 0.312, p<0.001); while households who depend on agricultural work alone tend to have lower per capita income than those households who have an additional secondary occupation (the coefficient is -0.899, p<0.001). In addition, the education attainment of respondents has a posi- tive effect on log per capita income, although the effect is small (the coefficient is 0.044, p < 0.001). Access to credit and the presence of person living with disability in the household does not signifi- cantly affect log per capita income.</td></tr>
</table>

<table>
<tr><td colspan="4">Structured summary of candidate operationalizations</td></tr>
<tr><td colspan="4">Candidate article: Deressa et al</td></tr>
<tr><td colspan="4">Construct operationalized: Vulnerability as Expected poverty</td></tr>
<tr><td colspan="2" rowspan="2">Sub-constructs</td><td>Intermediate constructs</td><td></td></tr>
<tr><td>Directly operationalized constructs</td><td>Poverty- SEE CANDIDATE SECTION</td></tr>
<tr><td colspan="2">Conceptual framework</td><td colspan="2"></td></tr>
<tr><td colspan="2">Operationalization of sub-constructs</td><td colspan="2"></td></tr>
<tr><td rowspan="2">72.</td><td rowspan="2">Poverty</td><td>Data collection</td><td>SEE CANDIDATE SECTION</td></tr>
<tr><td>Operational questions</td><td></td></tr>
<tr><td colspan="2">Candidate-level Analysis</td><td colspan="2"></td></tr>
</table>

Structured summary of candidate operationalizations				
**Candidate article:** Echevin				
**Construct operationalized:** Vulnerability as Expected poverty				
Sub-constructs		Intermediate constructs		Economic well-being
		Directly operationalized constructs		Household level- SEE CANDIDATE SECTION; community level- SEE CANDIDATE SECTION
Conceptual framework				
Operationalization of sub-constructs				
73.	Household level	Data collection	SEE CANDIDATE SECTION	
		Operational questions		
74.	community level-	Data collection	SEE CANDIDATE SECTION	
		Operational questions		
Candidate-level Analysis				

Structured summary of candidate operationalizations				
**Candidate article:** Günther & harttgen				
**Construct operationalized:** Vulnerability as Expected poverty				
Sub-constructs		Intermediate constructs		Risk-induced vulnerability
		Directly operationalized constructs		household level- SEE CANDIDATE SECTION; Idiosyncratic shocks- SEE CANDIDATE SECTION; covariate shocks- SEE CANDIDATE SECTION
Conceptual framework				
Operationalization of sub-constructs				
75.	household level	Data collection	SEE CANDIDATE SECTION	
		Operational questions		
76.	Idiosyncratic shocks	Data collection	SEE CANDIDATE SECTION	
		Operational questions		
77.	covariate shocks-	Data collection	SEE CANDIDATE SECTION	
		Operational questions		
Candidate-level Analysis				

Appendix Q: Order of measures and constructs

This appendix presents the results of an assessment that attempted to determine if the data gathered in a given operationalisation was of the same order as the construct it supported. For instance, given the construct ‘crop yield’, the data collection method of ‘remote sensing‘ or records of sale’ would match while ‘survey of farmers’ would not. The construct ‘crop yield’ requires objective measurement of a valid indicator. Asking farmers about their crop yield provides their espoused recall of a displaced external object. The point here is not that subjective measures are necessarily suspect. For example, remote sensing data may be objective but it might not support valid measurements of crop yield. The point, rather, is that assessments of vulnerability appear to mix the results of direct measurement with subjectively mediated measurement in a manner that assumes their equivalence and we could find no evidence in the articles reviewed that this equivalence had been established. Rather than including all of the data from appendix Q, this appendix presents a summary table of the results. Judgements found in the table below can be reviewed by consulting the data presented in Appendix Q.

Research questions: given the data presented in Appendix Q:

1. What order of data does this operationalization provide
    a. Respondent[|s]’ espoused recall of an a displaced external object (ER, DO)
    b. Respondent[|s]’ espoused recall of a displaced subjective condition (ER, DSC)
    c. Respondent[|s]’ espousal of a displaced external object (E, O)
    d. Respondent[|s]’ espousal of a displaced subjective condition (E, SC)
    e. Respondent[|s]’ espousal of immediate circumstances (E)
    f. Objective measurement of indicator of unit of analysis (O, I)
    g. Objective measurement of validated indicator of unit of analysis (O, VI)
2. What order of data does the corresponding construct require?
    a. Respondent[|s]’ espoused recall of an a displaced external object
    b. Respondent[|s]’ espoused recall of a displaced subjective condition
    c. Respondent[|s]’ espousal of a displaced external object
    d. Respondent[|s]’ espousal of a displaced subjective condition
    e. Respondent[|s]’ espousal of immediate circumstances
    f. Objective measurement of indicator of unit of analysis
    g. Objective measurement of validated indicator of unit of analysis
3. Does 1=2?
4. If 1 ≠ 2, is there justification for inference?
5. If there is justification for inference, is that justification adequate? (not done)

Match of Measures and Constucts				Measure is		
construct	source	Order DCM	Order Concept	subjective	objective	unclear
Actual meterological Observations	Gandure et al (2013)	O-I	O-VI		yes	
Adaptation Strategy	Westerhoff & Smit (2009)	E-DO	O-VI	yes	yes	
Adaptive Capacity	Westerhoff & Smit (2009)	E-DO	O-VI	yes	yes	
Adaptive Capacity	Baca et al (2004)	E-DO	O-VI	yes		
Adaptive Capacity	Sietz et al (2012)	E-DO	O-VI	yes		
Adaptive Capacity	Luers et al (2003)	O-I	O-VI		yes	
Adaptive Capacity	Ionesco et al (2009)	N/I	N/I			yes
agricultural vulnerabilty	Jamir et al (2013)	E-DO	O-VI	likely		
agriculture dependend households	Hahn et al (2009)	ER-DO	ER-DO	yes		
averge precipitation	Hahn et al (2009)	O-I	O-VI	yes		
Biphysical vulnerability	Jamir et al (2013)	E-DO	O-VI	likely		
borrow-lend ratio	Hahn et al (2009)	E-DO	O-VI	yes		
cereal production	Mutsvangwa (2011)	E-DO	O-VI	yes		
Climate Change	Mubaya et al (2012)	E-DO	O-VI	yes		
Climate change and Variability	Mubaya et al (2012)	E-DO	O-VI	yes		
Climate Variability	Mubaya et al (2012)	E-DO	O-VI	yes		
cluster pattern analysis	Sietz et al (2012)	N/I	N/I			
Community	Antwi-Agyei et al (2013)	E-DO	O-VI	yes		
Community Level	Echevin (2011)	E-DO	O-VI	yes		
Community Level	Günther & Harttgen	N/I	N/I			yes

Match of Measures and Constucts				Measure is		
construct	source	Order DCM	Order Concept	subjective	objective	unclear
	(2009)					
Covariate Shocks	*Sarris & Karfakis (2010)*	*E-DO*	*O-VI*	*yes*		
Covariate Shocks	*Günther & Harttgen (2009)*	*E-DO*	*O-VI*			*yes*
crop diversity	*Hahn et al (2009)*	*E-DO*	*O-VI*	*yes*		
Current exposure to risk	*Capaldo et al (2010)*	*E-DO*	*O-VI*	*likely*		
Current socio-economic characteristics	*Capaldo et al (2010)*	*E-DO*	*O-VI*	*likely*		
Demographic vulnerability	*Jamir et al (2013)*	*E-DO*	*O-VI*	*likely*		
dependency ratio	*Hahn et al (2009)*	*E-DO*	*O-VI*	*yes*		
determinants of resiliance	*Tesso et al (2012)*	*E-DO*	*O-VI*	*yes*		
don't save crops	*Hahn et al (2009)*	*E-DO*	*O-VI*	*yes*		
don't save seeds	*Hahn et al (2009)*	*E-DO*	*O-VI*	*yes*		
Drought	*Dasgupta & Baschieri (2012)*	*O-I*	*O-VI*		*yes*	
Entity	*Ionesco et al (2009)*	*N/I*	*N/I*			*yes*
exposed and sensitive to climate change	*Westerhoff & Smit (2009)*	*E-DO*	*O-VI*	*yes*	*yes*	
Exposure	*Antwi-Agyei et al (2013)*	*O-I*	*O-VI*		*yes*	
Exposure	*Baca et al (2004)*	*O-I*	*O-VI*		*yes*	
Exposure	*Luers et al (2003)*	*O-I*	*O-VI*		*yes*	
Exposure	*Piya et al (2012)*	*O-VI*	*O-VI*		*yes*	
exposure	*Sietz et al (2012)*	*O-I*	*O-VI*		*yes*	
Exposure	*Notenbaert et al (2013)*					*yes*

Match of Measures and Constucts				Measure is		
construct	source	Order DCM	Order Concept	subjective	objective	unclear
Family with chronic illness	Hahn et al (2009)	ER-DO	ER-DO	yes		
farmer perceptions	Mubaya et al (2012)	E-DO	E-DO	yes		
Financial Capital	Piya et al (2012)	E-DO	O-VI	likely		
Financial Capital	Antwi-Agyei et al (2013)	ER-DO	O-VI	yes	yes	
flood, drought, cyclone events	Hahn et al (2009)	ER-DO	ER-DO	yes		
food from family farm	Hahn et al (2009)	E-DO	O-VI	yes		
food security	Sietz et al (2012)	E-DO	O-VI	yes		
household characteristics	Chhinh & Poch (2012)	E-DO	O-VI	yes		
Household Consumption	Sarris & Karfakis (2010)	E-DO	O-VI	yes		
household level	Echevin (2011)	E-DO	O-VI	yes		
household level	Günther & Harttgen (2009)	E-DO	O-VI			yes
household level resilience	Tesso et al (2012)	E-DO	O-VI	yes		
households with orphans	Hahn et al (2009)	E-DO	O-VI	yes		
households working elsewhere	Hahn et al (2009)	ER-DO	ER-Do	yes		
Human Capital	Piya et al (2012)	E-DO	O-VI	likely		
Human Capital	Antwi-Agyei et al (2013)	E-DO	O-VI	yes		
Human Capital	Dasgupta & Baschieri (2012)	E-DO	O-VI	yes		

Match of Measures and Constucts				Measure is		
construct	source	Order DCM	Order Concept	subjective	objective	unclear
idiosyncratic shocks	Sarris & Karfakis (2010)	E-DO	O-VI	yes		
idiosyncratic shocks	Günther & Harttgen (2009)	N/I	N/I			yes
Impacts & responses to Hurricane Stan by coffee farmers	Eakin et al (2012)	E-DO	O-VI	yes		
inconsistent water supply	Hahn et al (2009)	ER-DO	ER-Do	yes		
independent of local government	Hahn et al (2009)	ER-DO	ER-Do	yes		
injury or death from disaster	Hahn et al (2009)	ER-DO	ER-Do	yes		
Institutional Environmnent	Notenbaert et al (2013)	N/I	N/I			yes
inverse water stored	Hahn et al (2009)	E-DO	O-VI	yes		
Labour	Dasgupta & Baschieri (2012)	E-DO	O-VI	yes		
lifelihood diversification	Hahn et al (2009)	E-DO	O-VI	yes		
maximum temperature	Hahn et al (2009)	O-I	O-VI	yes		
minimum consumption (income) level	Deressa et al (2009)	N/I	N/I			
multiple underlying forces	Westerhoff & Smit (2009)					
Natural Capital	Piya et al (2012)	E-DO	O-VI	likely		
Natural Capital	Antwi-Agyei et al (2013)	E-DO	O-VI	yes		
natural water source	Hahn et al (2009)	ER-DO	ER-Do	yes		
no warning of disaster	Hahn et al (2009)	E-DO	O-VI	yes		
non-climatic stress	Mubaya et al (2012)	E-DO	O-VI	yes		

Match of Measures and Constucts				Measure is		
construct	source	Order DCM	Order Concept	subjective	objective	unclear
non-labour productive assets	*Dasgupta & Baschieri (2012)*	*E-DO*	*O-VI*	*yes*		
percent of female-headed households	*Hahn et al (2009)*	*E-DO*	*O-VI*	*yes*		
perception of adiha farmers	*Mengitsu (2011)*	*E-DO*	*E-DO*	*yes*		
Physical Capital	*Piya et al (2012)*	*E-DO*	*O-VI*	*likely*		
Physical Capital	*Antwi-Agyei et al (2013)*	*E-DO*	*O-VI*	*yes*		
preference criteria	*Ionesco et al (2009)*	*N/I*	*N/I*			*yes*
proximity to health facility	*Hahn et al (2009)*	*E-DO*	*O-VI*	*yes*		
proximity to water source	*Hahn et al (2009)*	*E-DO*	*O-VI*	*yes*		
receive-give ratio	*Hahn et al (2009)*	*E-DO*	*O-VI*	*yes*		
reference scenarios	*Ionesco et al (2009)*	*N/I*	*N/I*			*yes*
resillient and vulnerable communities	*Antwi-Agyei et al (2013)*	*O-VI*	*O-VI*		*yes*	
Risk of experiencing climate change	*Dasgupta & Baschieri (2012)*	*O-I*	*O-VI*		*yes*	
Sensitivity	*Piya et al (2012)*	*E-DO*	*O-VI*	*likely*		
Sensitivity	*Luers et al (2003)*	*O-I*	*O-VI*		*yes*	
Sensitivity	*Sietz et al (2012)*	*O-I*	*O-VI*		*yes*	
Social Capital	*Piya et al (2012)*	*E-DO*	*O-VI*	*likely*		
Social Capital	*Antwi-Agyei et al (2013)*	*E-DO*	*O-VI*	*yes*		
Social Capital	*Dasgupta & Baschieri (2012)*	*E-DO*	*O-VI*	*yes*		
Socio-economic vulnerability	*Jamir et al (2013)*	*E-DO*	*O-VI*	*likely*		

Match of Measures and Constucts				Measure is		
construct	source	Order DCM	Order Concept	subjective	objective	unclear
State of system relative to threshold of damage	*Luers et al (2003)*	*O-I*	*O-VI*		*yes*	
stimulus	*Ionesco et al (2009)*	*N/I*	*N/I*			*yes*
Structural Poverty	*Günther & Harttgen (2009)*	*E-DO*	*O-VI*			*yes*
struggle for food	*Hahn et al (2009)*	*E-DO*	*O-VI*	*yes*		
Threshold to damage	*Luers et al (2003)*	*O-I*	*O-VI*		*yes*	
uneduated headed households	*Hahn et al (2009)*	*ER-DO*	*ER-DO*	*yes*		
vulnerability threshold	*Mutsvangwa (2011)*	*E-DO*	*O-VI*	*likely*		
water conflict	*Hahn et al (2009)*	*ER-DO*	*ER-Do*	*yes*		
week illness	*Hahn et al (2009)*	*ER-DO*	*ER-Do*	*yes*		
Wellbeing	*Luers et al (2003)*	*O-I*	*O-VI*		*yes*	

References

Acosta, Lilibeth, Richard J.T. Klein, Pytrik Reidsma, Marc J. Metzger, Mark D.A. Rounsevell, Rik Leemans, and Dagmar Schröter. 2013. "A Spatially Explicit Scenario-Driven Model of Adaptive Capacity to Global Change in Europe." *Global Environmental Change* 23 (5): 1211–24.

Antwi-Agyei, Philip, Andrew J. Dougill, Evan D. G. Fraser, and Lindsay C. Stringer. 2013. "Characterising the Nature of Household Vulnerability to Climate Variability: Empirical Evidence from Two Regions of Ghana." *Environment, Development and Sustainability* 15 (4): 903–26. doi:10.1007/s10668-012-9418-9.

Baca, María, Peter Läderach, Jeremy Haggar, Götz Schroth, and Oriana Ovalle. 2014. "An Integrated Framework for Assessing Vulnerability to Climate Change and Developing Adaptation Strategies for Coffee Growing Families in Mesoamerica." *PLoS ONE* 9 (2): e88463. doi:10.1371/journal.pone.0088463.

Berkes, Fikret, and Helen Ross. 2013. "Community Resilience: Toward an Integrated Approach." *Society & Natural Resources: An International Journal* 26 (1): 5–20. doi:10.1080/08941920.2012.736605.

Bogale, Ayalneh, Mohammed Taeb, and Mitsugi Endo. 2006. "Land Ownership and Conflicts over the Use of Resources: Implication for Household Vulnerability in Eastern Ethiopia." *Ecological Economics* 58: 134–45.

Borgatti, Stephen P. 1994. "Cultural Domain Analysis." *Journal of Quantitative Anthropology* 4: 261–78.

Calvo, Cesar, and Stefan Dercon. 2013. "Vulnerability to Individual and Aggregate Poverty." *Social Choice and Welfare* 41 (4): 721–40. doi:10.1007/s00355-012-0706-y.

Capaldo, Jeronim, Panagiotis Karfakis, Marco Knowles, and Mark Smulders. 2010. "A Model of Vulnerability to Food Insecurity." *ESA Working Paper* 10 (03). http://bvsan.uni.edu.ni:8080/48/1/model_vulnerability.pdf.

CARE. 2009. *Climate Vulnerability and Capacity Analysis: Handbook*. http://www.careclimatechange.org.

Carroll, Christopher, Andrew Booth, Joanna Leaviss, and Jo Rick. 2013. "'Best Fit' Framework Synthesis: Refining the Method." *BMC Medical Research Methodology* 13: 37.

Carroll, Christopher, Jo Rick, Joanna Leaviss, and Andrew Booth. 2013. "A Qualitative Evidence Synthesis of Employees' Views of Workplace Smoking Reduction or Cessation Interventions." *BMC Public Health* 13: 1095.

Chhihn, Nyda, and Bunnak Poch. 2012. "Climate Change Impacts on Agriculture and Vulnerability as Expected Poverty of Kampong Speu Province, Cambodia." *IJERD – International Journal of Environmental and Rural Development* 3 (2): 28–37.

Dasgupta, Aisha, and Angela Baschieri. 2010. "Vulnerability to Climate Change in Rural Ghana: Mainstreaming Climate Change in Poverty-Reduction Strategies." *Journal of International Development* 22: 803–20.

Da Silva, Sven. 2014. "Assessment of Transparency, Structure, and Coherence: A Systematic Review of Empirical Research Articles on Social Movements in Latin America." Wageningen: Wageningen University.

Deressa, Temesgen T., Rashid M. Hassan, and Claudia Ringler. 2009. "Assessing Household Vulnerability To Climate Change: The Case Of Farmers In The Nile Basin Of Ethiopia." *IFPRI Discussion Paper 00935*.

Dhamija, Nidhi, and Shashanka Bhide. 2011. "DYNAMICS OF CHRONIC POVERTY: VARIATIONS IN FACTORS INFLUENCING ENTRY AND EXIT OF CHRONIC POOR." *CPRC-IIPA Working Paper No. 38*. http://www.chronicpoverty.org/uploads/publication_files2/CPRC-IIPA%2039-new.pdf.

Eakin, Hailli, Karina Benessaiah, Juan F. Baretta, Gustavo M. Cruz-Bello, and Helda Morales. 2012. "Livelihoods and Landscapes at the Threshold of Change: Disaster and Resilience in a Chiapas Coffee Community." *Regional Environmental Change* 12: 475–88. doi:DOI 10.1007/s10113-011-0263-4.

Eakin, Hailli, Alexandra Winkels, and Jan Sendzimir. 2009. "Nested Vulnerability: Exploring Cross-Scale Linkages and Vulnerability Teleconnections in Mexican and Vietnamese Coffee Systems." *Environmental Science and Policy* 12: 398–412.

Échevin, Damien. 2011. "Characterizing Poverty and Vulnerability in Rural Haiti: A Multilevel Decomposition Approach." *Munich Personal RePEc Archive* 35659. http://mpra.ub.uni-muenchen.de/35659/.

Ford, James, and Barry Smit. 2004. "A Framework for Assessing the Vulnerability of Communities in the Canadian Arctic to Risks Associated with Climate Change." *Arctic* 57 (4): 389–400.

Füssel, Hans-Martin, and Richard J.T. Klein. 2006. “Climate Change Vulnerability Assessments: An Evolution of Conceptual Thinking.” *Climate Change* 75: 301–29. doi:DOI: 10.1007/s10584-006-0329-3.
Gandure, S., S. Walker, and J.J. Botha. 2013. “Farmers’ Perceptions of Adaptation to Climate Change and Water Stress in a South African Rural Community.” *Environmental Development* 5: 39–53.
Glaser, Barney G. 1965. “The Constant Comparative Method of Qualitative Analysis.” *Social Problems* 12 (4): 436–45.
Günther, Isabel, and Kenneth Harttgen. 2009. “Estimating Households Vulnerability to Idiosyncratic and Covariate Shocks: A Novel Method Applied in Madagascar.” *World Development* 37 (7): 1222–34. doi:doi:10.1016/j.worlddev.2008.11.006.
Hahn, Micah B., Anne Riederer, and Stanley Foster. 2009. “The Livelihood Vulnerability Index: A Pragmatic Approach to Assessing Risks from Climate Variability and change—A Case Study in Mozambique.” *Global Environmental Change* 19: 74–88.
Ionesco, Cezar, Richard J.T. Klein, Jochen Hinkel, K. S. Kavi Kumar, and Rupert Klein. 2009. “Towards a Formal Framework of Vulnerability to Climate Change.” *Environmental Modeling and Assessment* 14 (1): 1–16.
Jamir, Chubanaro, Nitasha Sharma, Asmita Sengupta, and N. H. Ravindranath. 2013. “Farmers’ Vulnerability to Climate Variability in Dimapur District of Nagaland, India.” *Regional Environmental Change* 13 (1): 153–64. doi:10.1007/s10113-012-0324-3.
Kampen, Jarl K., and Peter Tamás. 2014. “Should I Take This Seriously? A Simple Checklist for Calling Bullshit on Policy Supporting Research.” *Quality & Quantity* 48: 1213–23. doi:DOI 10.1007/s11135-013-9830-8.
Khan, Fazal Ali, and Ali Salman. 2012. “A Simple Human Vulnerability Index to Climate Change Hazards for Pakistan.” *International Journal of Disaster Risk Science* 3 (3): 163–76. doi:10.1007/s13753-012-0017-z.
Luers, Amy, David B. Lobell, Leonard S. Sklar, C. Lee Addams, and Pamela A. Matson. 2003. “A Method for Quantifying Vulnerability, Applied to the Agricultural System of the Yaqui Valley, Mexico.” *Global Environmental Change* 13: 255–67.
Marshall, Nadine A. 2010. “Understanding Social Resilience to Climate Variability in Primary Enterprises and Industries.” *Global Environmental Change* 20: 36–43. doi:doi:10.1016/j.gloenvcha.2009.10.003.
Mengistu, Dejene. 2011. “Farmers’ Perception and Knowledge of Climate Change and Their Coping Strategies to the Related Hazards: Case Study from Adiha, Central Tigray, Ethiopia.” *Agricultural Sciences* 2 (2): 138–45. doi:doi:10.4236/as.2011.22020.
Misselhorn, Alison A. 2005. “What Drives Food Insecurityin Southern Africa? A Meta-Analysis of Household Economy Studies.” *Global Environmental Change* 15: 33–43.
Morse, Janice M. 2004. “Constructing Qualitatively Derived Theory: Concept Construction and Concept Typologies.” *Qualitative Health Research* 14 (10): 1387–95. doi:10.1177/1049732304269676.
Mubaya, Chipo Plaxedes, Jemimah Njuki, Eness Paidamoyo Mutsvangwa, Francis Temba Mugabe, and Durton Nanja. 2012. “Climate Variability and Change or Multiple Stressors? Farmer Perceptions Regarding Threats to Livelihoods in Zimbabwe and Zambia.” *Journal of Environmental Management* 102: 9–17.
Mutsvangwa, Eness P. 2011. “Climate Change and Vulnerability to Food Insecurity among Smallholder Farmers: A Case Study of Gweru and Lupane Districts in Zimbabwe.” South Africa: University of Free State Bloemfontein. http://etd.uovs.ac.za/ETD-db/theses/available/etd-08182011-105132/unrestricted/MutsvangwaEP.pdf.
Nkondze, Majahodvwa S., Micah B. Masuku, and Absalom Manyatsi. 2013. “Factors Affecting Households Vulnerability to Climate Change in Swaziland: A Case of Mpolonjeni Area Development Programme (ADP).” *Journal of Agricultural Science* 5 (10): p108. doi:10.5539/jas.v5n10p108.
Notenbaert, An, Stanley Nganga Karanja, Mario Herrero, Maute Felisberto, and Siboniso Moyo. 2013. “Derivation of a Household-Level Vulnerability Index for Empirically Testing Measures of Adaptive Capacity and Vulnerability.” *Regional Environmental Change* 13 (2): 459–70. doi:10.1007/s10113-012-0368-4.
Piya, Luni, Keshav Lall Maharjan, and Niraj Prakash Joshi. 2012. “Vulnerability of Rural Households to Climate Change and Extremes: Analysis of Chepang Households in the Mid-Hills of Nepal.” In . Foz do Iguaçu, Brazil.
Sallu, Susannah, Chasca Twyman, and Lindsay C. Stringer. 2010. “Resilient or Vulnerable Livelihoods? Assessing Livelihood Dynamics and Trajectories in Rural Botswana.” *Ecology and Society* 15 (4): 3.
Sandoval, Jennifer A., Julie Lucero, John Oetzel, Magdalena Avila, Lorenda Belone, Marjorie Mau, Cynthia Pearson, et al. 2012. “Process and Outcome Constructs for Evaluating Community-Based Participatory Research Projects: A Matrix of Existing Measures.” *Health Education Research* 27 (4): 680–90.

Sarris, Alexander, and Panayiotis Karfakis. 2010. "Vulnerability to Covariate and Idiosyncratic Shocks and Safety Net Targeting of Rural Households with an Application to Rural Tanzania." In . Paris. http://erd.eui.eu/media/2010/Sarris.pdf.
Sietz, Diana, Sabino Edgar Mamani Choque, and Matthias K. B. Lüdeke. 2012. "Typical Patterns of Smallholder Vulnerability to Weather Extremes with Regard to Food Security in the Peruvian Altiplano." *Regional Environmental Change* 12 (3): 489–505. doi:10.1007/s10113-011-0246-5.
Tesso, Gutu, Bezabih Emana, and Mengistu Ketema. 2012. "Analysis of Vulnerability and Resilience to Climate Change Induced Shocks in North Shewa, Ethiopia." *Agricultural Sciences* 3 (6): 871–88.
Westerhoff, Lisa, and Barry Smit. 2009. "The Rains Are Disappointing Us: Dynamic Vulnerability and Adaptation to Multiple Stressors in the Afram Plains, Ghana." *Mitigation and Adaptation Strategies for Global Change* 14 (4): 317–37. doi:10.1007/s11027-008-9166-1.
