# Supplementary material for: Systematic Review of Methods in Low-Consensus Fields: Supporting Commensuration through `Construct-Centered Methods Aggregation’ in the Case of Climate Change Vulnerability Research
Source: PLoS One. 2016 Feb 22;11(2):e0149071. doi: 10.1371/journal.pone.0149071 (PMC4762661; doi:10.1371/journal.pone.0149071)
Supplement: S2 File — Source articles and bibliography. (ZIP) [file pone.0149071.s002.zip › data requirements/Berkes & Ross.pdf]

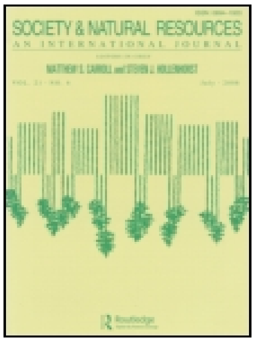

Routledge
Taylor & Francis Group

Society & Natural Resources
An International Journal

ISSN: 0894-1920 (Print) 1521-0723 (Online) Journal homepage: http://www.tandfonline.com/loi/usnr20

Community Resilience: Toward an Integrated Approach

Fikret Berkes & Helen Ross

To cite this article: Fikret Berkes & Helen Ross (2013) Community Resilience: Toward an Integrated Approach, Society & Natural Resources, 26:1, 5-20, DOI: 10.1080/08941920.2012.736605

To link to this article: http://dx.doi.org/10.1080/08941920.2012.736605

Published online: 30 Nov 2012.

Submit your article to this journal

Article views: 6483

View related articles

Citing articles: 72 View citing articles

Full Terms & Conditions of access and use can be found at
http://www.tandfonline.com/action/journalInformation?journalCode=usnr20

Download by: [212.233.56.181] **Date:** 16 December 2015, At: 02:53

Society and Natural Resources, 26:5–20
Copyright © 2013 Taylor & Francis Group, LLC
ISSN: 0894-1920 print/1521-0723 online
DOI: 10.1080/08941920.2012.736605

Routledge
Taylor & Francis Group

Downloaded by [212.233.56.181] at 02:53 16 December 2015

Featured Articles

Community Resilience: Toward an Integrated Approach

FIKRET BERKES

Natural Resources Institute, The University of Manitoba, Winnipeg, Manitoba, Canada

HELEN ROSS

School of Agriculture and Food Sciences, The University of Queensland, Gatton, Queensland, Australia

We explore opportunities for an integrated approach in community resilience to inform new research directions and practice, using the productive common ground between two strands of literature on community resilience, one from social–ecological systems and the other from the psychology of development and mental health. The first strand treats resilience as a systems concept, dealing with adaptive relationships and learning in social–ecological systems across nested levels, with attention to feedbacks, nonlinearity, unpredictability, scale, renewal cycles, drivers, system memory, disturbance events, and windows of opportunity. The second strand emphasizes identifying and developing community strengths, and building resilience through agency and self-organization, with attention to people–place connections, values and beliefs, knowledge and learning, social networks, collaborative governance, economic diversification, infrastructure, leadership, and outlook. An integrative approach seated in the complex adaptive system and ecological understanding can incorporate the identification of explicit social strengths and connections to place, activated by agency and self-organizing.

Keywords adaptive management, agency, community development, community health, complex adaptive systems, panarchy, resilience, social learning, social–ecological systems

Received 12 February 2012; accepted 31 July 2012.

Berkes's work has been supported by the Canada Research Chairs program (http://www.chairs.gc.ca). Ross's work has been supported by the Australian Research Council Discovery Program, and the Australian Government's Marine and Tropical Sciences Research Facility (MTSRF), through the Department of the Environment, Water, Heritage and the Arts. She thanks Desley Hegney, Elizabeth Buikstra, Michael Cuthill, Kirsten Maclean, and other team members and partners for their contributions to our studies of social resilience.

Address correspondence to Fikret Berkes, Natural Resources Institute, The University of Manitoba, 303-70 Dysart Rd, Winnipeg, Manitoba R3T 2N2, Canada. E-mail: Fikret.Berkes@ad.umanitoba.ca

An era of rapid change brings the challenge of rapid and flexible response at all levels. Resilience thinking (resilience theory) is one of the major conceptual tools in the environment literature to deal with change (Scheffer 2009; Chapin et al. 2009) at multiple levels of organization from local to global (Gunderson and Holling 2002). Resilience literature at the level of ecosystems is well developed, but the same cannot be said for the local and community level. Here, one finds another cluster of resilience literature from health and developmental psychology (Brown and Westaway 2011).

Two strands of literature have been converging toward an appreciation of community resilience. One strand originally comes from ecology and addresses the resilience of ecosystems or integrated social–ecological systems (Holling 1973; Chapin et al. 2009). Based on some four decades of conceptual development, this literature is strong in the biophysical sciences, but the social science component is relatively new and weakly developed (Davidson 2010; Berkes and Folke 1998). The other strand originates in the psychology of personal development and mental health, which normally deal with the individual level. This provides the conceptual background of the disaster literature (Brown and Westaway 2011) and has connected with a range of social science disciplines interested in factors that allow communities to deal with adversity, such as community development (Paton and Johnston 2001) and community self-organization (Norris et al. 2008; Chaskin 2008). Conceptual development of community-level resilience in this literature is relatively new. Here we identify the productive common ground between these two strands of literature.

Community-level resilience is of special interest because of the overlaps and complementarities between the two strands of literature, and opportunities of mutual enrichment. These considerations indicate the need to develop an integrated concept of community resilience. We argue two points. The first is that the panarchy concept (Gunderson and Holling 2002) indicates that attention should be paid to all levels. The community level is relatively neglected and therefore requires more attention. The second is that the set of ideas from the emerging community resilience literature from community health and development can help us develop an enriched and integrated concept of community resilience, and inform new research directions and practice.

There are competing notions and definitions of resilience. In the social–ecological systems strand, resilience is the capacity of the system to continually change and adapt and yet remain within critical thresholds. It may be formally defined as the capacity of a system to absorb disturbance and reorganize while undergoing change so as to still retain essentially the same function, structure, identity, and feedbacks (Walker et al. 2004). Other definitions of resilience include those that focus on the return to equilibrium after a disturbance, in contrast to Holling's (1973) resilience that says nothing about returning to the original state, as it assumes constant change (Folke et al. 2010).

A common definition from health and psychology focuses on the ability of individuals to recover from adversity (Buikstra et al. 2010). As defined by Magis (2010, 401) community resilience is the "existence, development and engagement of community resources by community members to thrive in an environment characterized by change, uncertainty, unpredictability and surprise." A community's resilience is often understood as the capacity of its social system to come together to work toward a communal objective. The Canadian Centre for Community Renewal (2000, 1–5) defines a resilient community as "one that takes intentional action to enhance the personal and collective capacity of its citizens and institutions to respond to and influence the course of social and economic change."

Downloaded by [212.233.56.181] at 02:53 16 December 2015

These definitions from the two strands are consistent in their focus on the adaptive capacity of a system (individuals, communities, larger societies, corporations, social–ecological systems, ecosystems) in the face of change. Hence, resilience building can be accomplished by actively developing and engaging the capacity to thrive in an environment characterized by change (Magis 2010; Armitage et al. 2011). However, there are aspects of the social–ecological resilience concept that are not used in community resilience. Much of the disaster resilience literature uses resilience in its dictionary sense (Manyena 2006) or as a metaphor (Norris et al. 2008), rather than as an analytic concept producing new insights. The strand derived from psychology builds theory by examining components and processes of community resilience. Norris et al. (2008) emphasize the understanding of stress, adaptation, wellness, and resource dynamics; Magis (2010) emphasizes dimensions of resilience such as community resources, collective action, and strategic action. Although both strands of literature go back many decades, there are few citations across the two and almost no overlap; exceptions include Adger (2000), Ommer (2007), and Brown and Westaway (2011).

To develop an integrated concept of community resilience, we first examine some of the key insights from the two strands of literature. We then deal with characteristics of resilient communities, supplementing the idea of components of resilience (from the health, psychology, and disaster strand) with stronger systems concepts from the social–ecological resilience strand, toward building a synthesis.

Downloaded by [212.233.56.181] at 02:53 16 December 2015

Insights from the Analysis of Social–Ecological System Resilience

Ecological resilience literature starts with C. S. (Buzz) Holling (1973), whose notion of resilience was based on the observations of the dynamics of the boreal forest ecosystem, with its uncertainties, abrupt shifts, and renewal cycles. Recognizing that ecosystems often exhibit multiple stable states, Holling sought a way to characterize the capacity of a system for self-renewal and maintenance in the face of disturbance. He described how a changing system could remain within critical thresholds in a given stable state or else flip into a new system (Gunderson and Holling 2002; Folke et al. 2010). Resilience theory deals with system dynamics and envisions ecosystems as continuously changing, sometimes abruptly and unpredictably. It focuses on renewal cycles and disturbance events, such as fire, that are essential to renew the ecosystem before another cycle of growth and development can proceed.

In its broader context, resilience is about ecosystems and people together as integrated social–ecological systems in which social systems and ecosystems are recognized as coupled, interdependent, and coevolving (Berkes and Folke 1998; Folke 2006). Studies that have critically examined social–ecological systems have found them to be something more than the sum of social systems and ecological systems (Liu et al. 2007). Much of the resilience literature from about 2000 onward adopted the term *social–ecological system*. Making the social side explicit has been consistent with efforts to deal with global issues. Indeed, many of the drivers leading to abrupt and unexpected change are social and economic in nature (Scheffer 2009). A key assumption is that there is no balance of nature, but nonequilibrium or multi-equilibrium conditions, unpredictable systems, subject to cycles of continuous change and renewal (Norberg and Cumming 2008; Chapin et al. 2009).

Table 1 provides a selection of examples that provide community resilience insights from social–ecological systems resilience studies. Rather than assuming that

Table 1. A selection of examples showing use of social–ecological system resilience concepts

Case	Question	Findings and lessons
Two Huaorani communities in Ecuador's Amazon (Lu 2010)	What does attention to scale contribute to studies of hunting sustainability?	Linear thinking and data from snapshots in time suggest hunters drive prey species to depletion. But situating the study in larger temporal and spatial scales and taking into account adaptive processes such as switching prey species, the observed hunting sustainability can be modeled.
Coastal Cambodia (Marschke and Berkes 2006)	How can resilience thinking help understand livelihood challenges and how people deal with them?	People at the community level are continuously "doing something" as a response to various shocks and stresses to their livelihood systems. Livelihood strategies often, but not always, produce community resilience.
California fires of 2003 (Goldstein 2008)	Is there an alternative to increasingly centralized fire management agencies?	San Diego Fire Recovery Network, an informal learning organization, contributed to community resilience by diversifying possible responses to environmental change and uncertainty.
Inuvialuit of Canadian Western Arctic (Berkes and Jolly 2001)	How do the Inuvialuit respond to climate change impacts at household and community levels? What is the source of responses?	Indigenous coping responses to climate change at household and community levels are an extension of traditional adaptive strategies to deal with the variability of the Arctic environment; over time, coping responses can become adaptive strategies.
Gabra pastoralists of northern Kenya	What can be done when existing community	New knowledge, technology, and adaptations can be

(*Continued*)

Downloaded by [212.233.56.181] at 02:53 16 December 2015

Table 1. Continued

Case	Question	Findings and lessons
(Robinson and Berkes 2011)	adaptations are insufficient to deal with new kinds of global change?	accessed by contact with higher levels of organization. Through networks, deliberation, and inclusivity, NGOs can facilitate multilevel interactions, which, in turn, leads to social learning and resilience building.

Downloaded by [212.233.56.181] at 02:53 16 December 2015

human hunters simply act on prey species in a linear relationship to deplete them, Lu (2010) used long-term data to analyze hunter–prey dynamics as a social–ecological system characterized by feedbacks, nonlinearities, uncertainty, and social learning. Other studies show that community institutions have the ability to persist through change and learn from challenges to deal with stresses such as resource depletion and conflicts, and shocks such as forest fires and border closing. Analyzed at multiple levels (household, community, national), adaptive responses and social learning may be interpreted as evidence of resilience through increased livelihood options and flexibility (Marschke and Berkes 2006).

Some scholars have emphasized the role of social learning in transforming an existing social–ecological system, rather than perpetuating it (Wilson 2012). In resilience theory, transformability is the capacity "to create untried beginnings from which to evolve a new way of living" (Walker et al. 2004, 7). Goldstein (2008) analyzed the notion that disasters may provide windows of opportunity to transform social–ecological systems. He studied the role of informal and community-based knowledge networks (skunkworks) to challenge the narrative that "enhancement of the technological capacity and authority of government [was] the only reasonable response to fire disaster" (Goldstein 2008, 24).

Analyzing community adaptation in the face of climate, Berkes and Jolly (2001) found that Canadian western Arctic indigenous people were able to build on their traditional resilience to their highly variable Arctic ecosystems, with coping strategies to adjust "when, where and how" of hunting, and minimizing risk, especially with regard to increasingly unreliable sea ice (Berkes and Jolly 2001). Community adaptive capacity to deal with change relies not only on existing cultural adaptations but also on the ability to put together knowledge from different sources to make a new synthesis, co-producing knowledge (Armitage et al. 2011). Bringing in new sources of knowledge to access options developed at regional, national, and international levels may require bridging organizations, such as development nongovernmental organizations (NGOs), for resilience-based development policies that incorporate multiple levels of participation (Robinson and Berkes 2011).

Resilience concepts are thought to apply to all levels, from individual to earth system. In any given case, resilience phenomena are occurring simultaneously at nested and interacting levels, as illustrated by Table 1. However, many resilience scholars tend to look for social–ecological systems, rather than communities. A

resource-dependent community or an indigenous community that affiliates culturally with a local environment and relies heavily on local resources can be treated as a social–ecological system (Lu 2010). However, most communities are too diffuse, boundaries are much too porous, and many people make their livelihoods outside the immediate geographic area. In many cases, a community is not a natural choice for a researcher who has a resource focus. Nevertheless, we argue that the community level is a vital one to understand within the panarchy. Attention to the community level also provides a healthy opportunity for convergence between the understandings developed in the two strands of resilience science.

Insights from Mental Health, Developmental Psychology, and Community Development

Our second resilience strand evolved from a mental health and personal development (developmental psychology) tradition, interested in the factors that lead some individuals to cope better than others through major disturbances in their lives. The work on individual resilience, especially in relation to child development, has recently been extended to community level, particularly in relation to disaster management and recovery (Paton and Johnston 2001).

The central principle in this strand of research on resilience is identifying and building on an individual's or community's strengths, rather than focusing primarily on identifying and overcoming deficits (Luthar 2006; Buikstra et al. 2010). Resilience is seen as a continual personal development process in facing adversity and adaptation, rather than a stable outcome that is reached and maintained (Luthar and Cicchetti 2000; Almedom et al. 2007). The process is nonlinear: Personal psychosocial transitions occur (Almedom et al. 2007). While people may become more resilient through coping with successive experiences, even the most resilient individuals may be set back if further hardships cross their personal tolerance threshold (Masten and Obradović 2006).

Research is concerned with identifying the factors involved, the processes of developing resilience, and providing an evidence base for interventions (Luthar and Cicchetti 2000; Buikstra et al. 2010). The processes involve dynamic interactions between the person and his or her social and physical environment, influenced by a range of personal cognitive, emotional, and spiritual factors (such as optimism) and personal goals (Kumpfer 1999; Brown and Westaway 2011; Buikstra et al. 2011). Protective factors such as social support are recognized as playing important roles. The goal is to understand the fundamental adaptive systems involved as individuals confront and transcend adversity, both adapting to the environment and maintaining internal integration (Masten and Obradovic 2006).

Questions arising from this literature include the criteria for judging good adaptation, and the nature of processes underpinning the resilience-promoting factors. Can an individual be resilient in one context or at one time and not another, for one kind of stressor and not another, and for one kind of adaptive domain and not another (Masten and Obradovic 2006)? The focus here is on the outcome for the individual, whereas social–ecological resilience is fundamentally interested in the system (rather than its components) and its dynamics.

The extension of this work to community level focuses on identifying the nature of a community's strengths, and how these contribute within a collective process of facing challenges and developing resilience (Kulig 2000; Kulig et al. 2008; Kulig et al.

Downloaded by [212.233.56.181] at 02:53 16 December 2015

2010; Hegney et al. 2008a; 2008b; Norris et al. 2008; Buikstra et al. 2010). Studies agree on a key set of community strengths: social networks, and the communications, social support, and the social inclusion and sense of belonging they foster (social capital); leadership; outlook on life, including readiness to accept change; and learning (Norris et al. 2008; Kulig et al 2010; Buikstra et al. 2010; Ross et al. 2010). Some studies also recognize the roles of the natural and built environment, of the lifestyles and livelihoods these enable, and of having a diverse and innovative economy to manage through agricultural failures and economic downturns in particular sectors (Hegney et al. 2008a; Buikstra et al. 2010; Gooch et al. 2010; Ross et al. 2010). The roles of infrastructure and support services are particularly important from a disaster recovery respective, as the loss of infrastructure and the services relying on it inhibits use of the other strengths (Kulig et al. 2010). Some provide narratives explaining how communities draw on their strengths in order to organize (Kulig et al. 2010; Ross et al. 2010). Almedom (2004) identifies the combined supporting roles of macro (government) and micro (family, community) factors to assist people suffering displacement to adjust emotionally.

Four studies of community resilience using the health and development approach are summarized in Table 2, showing many common themes across the four cases with regard to attributes of community and individual resilience. Community action through self-organizing and a sense of agency are vital themes across much of this literature and that of others (Goldstein 2008; Magis 2010; Brown and Westaway 2011; Wilson 2012). Kulig et al. (2010) emphasize action, especially problem solving, as part of community resilience building. An understanding of resilience at the community level can therefore benefit from actions and processes for building community strengths, as recognized in the body of work in community development (Zautra et al. 2008; Paton and Johnston 2001).

Taken in combination, the health and disaster strand of literature, combined with community development, contributes ideas that can usefully elaborate the systems thinking contributed by the social–ecological resilience literature. The idea of resilience building as a process, rather than an outcome, sits well with the dynamics of resilience in social–ecological systems. The idea of identifying and building on strengths could enhance social–ecological resilience thinking. The literature identifies an important set of strengths, with considerable consistency across countries, cultures, and types of threats (Table 2). The strengths match well to findings from the social–ecological systems literature (Table 1). They are not purely social or ecological but also involve infrastructure, livelihoods, and economies. The important point, however, is the way in which communities draw on their strengths in combination, leading to agency and self-organization, which appear to be important aspects of generating community level resilience.

Developing an Integrated Concept of Community Resilience

We have strived to identify a core set of ideas from the two strands of literature, using those directly relevant to community resilience. We have found much overlap but also some key differences, to help point out where the two strands can learn from each other toward improved scholarship and practice in the area of community resilience. We seek an “integrated concept” in that limited sense, and not in the sense of somehow integrating large and disparate bodies of literature, given that the two strands are rooted in two very different scholarly traditions.

Downloaded by [212.233.56.181] at 02:53 16 December 2015

Table 2. Selected studies of community resilience elaborating from psychology of development and mental health theory

Context, research questions, and references	Strengths, processes, and contributions to practice
Multiple hazards in a rural area, Southern Queensland, Australia<br>What are the characteristics of individual and community resilience, and how can these be fostered? (Hegney et al. 2008a; Buikstra et al. (2010)	Identified 11 attributes that apply both to community and individual resilience:<br>Social networks and support<br>Positive outlook<br>Learning<br>Early experience<br>Environment and lifestyle<br>Infrastructure and support services<br>Sense of purpose<br>Diverse and innovative economy<br>Embracing differences<br>Beliefs<br>Leadership<br>Note: Developed a toolkit toward building community resilience.
Rural and urban, protected areas, Queensland Wet Tropics, Australia<br>What are the characteristics of social resilience, and how can community and regional-level managers of natural resources and social services enhance them? (Gooch et al. 2010; Ross et al. 2010)	Identified 6 attributes of social resilience:<br>People–place connections<br>Knowledge, skills, and learning<br>Community networks<br>Engaged governance<br>Diverse and innovative economy<br>Community infrastructure<br>Note: Ross et al. (2010) argued that organizations can manage for social resilience by acknowledging, using, or seeking to enhance these attributes, and developed indicators of social resilience.
Rural well-being, Alberta, Canada<br>What is the relationship between health status and community resilience? How can health professionals assist? (Kulig et al. 2010; Kulig et al. 2008; Kulig 2000)	Key attributes of community resilience are:<br>Leadership<br>Community problem solving<br>Sense of belonging<br>Community togetherness<br>Mentality/outlook<br>Ability to cope with divisions<br>Getting along<br>Networks<br>Ability to cope with change<br>Note: Kulig et al. (2010) argued that resilience involves relationships

(*Continued*)

Downloaded by [212.233.56.181] at 02:53 16 December 2015

Table 2. Continued

Context, research questions, and references	Strengths, processes, and contributions to practice
	between community interactions as a collective unit; expressions of a sense of community (e.g., pride, belonging); internal and external influences/ impacts (e.g., a mine closure, economic downturn, or health); and community action in response to influences.
Model building for disaster management, based on review<br>How does resilience enhance disaster readiness? (Norris et al. 2008)	Portrays community resilience as a network of adaptive capacities, centered on:<br>Social capital (including linkages)<br>Economic development (including level and diversity of economic resources and fairness of distribution)<br>Information and communication<br>Community competence: taking action, critical reflection, collective efficacy and empowerment, and political partnerships.

Downloaded by [212.233.56.181] at 02:53 16 December 2015

Community resilience concepts apply best to place-based communities (Maida 2007), and less fully to communities where people make their living outside the geographic area. However, some communities of interest have resource ties (e.g., vegetable farmers) and displaced communities may retain social bonds (Almedom 2004). In the case of communities that have historical intimacy with their lands, as with many Indigenous groups, the health of the people may be closely related to the health of the land, a notion captured in the Australian Indigenous maxim "healthy country, healthy people" (Ross et al. 2010). Even though there are no doubt many factors involved in such relationships, many Indigenous people and other rural communities, such as forest-based communities (Kelly and Bliss 2009), see a correspondence between the health and the resilience of communities and their environments.

Communities do not control all of the conditions that affect them, but they have the ability to change many of the conditions that can increase their resilience. They can build resilience through their responses to shocks and stresses, and actively develop resilience through capacity building and social learning—but up to a point. Control is at best partial and outcomes always uncertain (Goldstein 2009). Community strengths that assist the development of resilience obviously vary from community to community, but cases indicate a set of characteristics as playing key roles (Table 2). The most important of these are summarized in Figure 1: people–place connections; values and beliefs; knowledge, skills and learning; social networks; engaged governance (involving collaborative institutions); a diverse and innovative

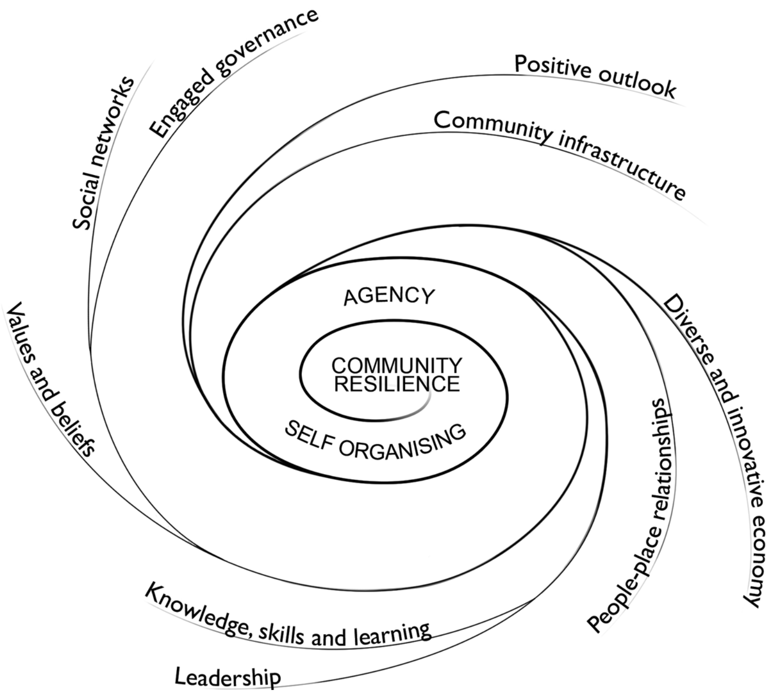

Figure 1. Community resilience as a function of the strengths or characteristics that have been identified as important, leading to agency and self-organization.

economy; community infrastructure; leadership; and a positive outlook, including readiness to accept change. As Figure 1 expresses, these strengths are drawn into combined influence through agency and self-organizing. Thus, the characteristics that feature in resilience, and processes of agency and self-organizing, are important foci for developing an integrated concept of community resilience. Just as social–ecological resilience provides a timely contribution to those social sciences that devote minimal attention to ecological considerations (Wilkinson 2011), health and development resilience provides a timely reminder to those natural sciences with positivistic tendencies that devote minimal attention to social considerations such as agency.

The resilience of any and all parts of a system to all kinds of shocks and stresses, known as general resilience (Folke et al. 2010), may be contrasted with specified resilience, in which one asks the question, resilience of what, to what (Carpenter et al. 2001)? Both kinds of resilience are no doubt important, and an integrated approach would consider how they can be combined fruitfully. In some fields, such as disaster management, the question of what to what becomes particularly important, as for example in the case of a community that is flood prone, as compared to one that is fire prone. However, specified resilience risks becoming too focused; increasing resilience of particular parts of a system to specific disturbances may cause the system to lose resilience in other ways (Folke et al. 2010). Resilience is a systems concept, and the social–ecological system, as an integrated and interdependent unit, may itself be considered a complex adaptive system (Norberg and Cumming 2008). As such, the analysis of community resilience would be expected

Downloaded by [212.233.56.181] at 02:53 16 December 2015

to be sensitive to the various principles of complexity, such as feedbacks, nonlinearity, unpredictability, and scale (Berkes et al. 2003; Liu et al. 2007).

Social–ecological systems literature alerts researchers to look for renewal cycles, memory in the system that can help restart cycles, disturbance events, drivers of change, and the significance of windows of opportunity during which innovative changes can be made in the system (Scheffer 2009; Chapin et al. 2009). It emphasizes slow-onset hazards associated with slow drivers of change (Wilson 2012; Haque and Etkin 2012). Resilience at the level of the individual, the household, and the community is all interrelated, even though resilience building mechanisms of each, and the actual set of principles applying to each, may be different (Berkes et al. 2003). The resilience of individuals and households is linked to that of the community. A community with individuals who are personally resilient in the face of physical disasters is likely to be resilient as a community as well. Similarly, households with livelihood resilience will contribute to community and regional livelihood resilience. However, the correspondence between levels may not always hold. For example, loss of the family head's labor may be disastrous for the household but not necessarily for the community as a whole (Marschke and Berkes 2006).

A further conceptual need is to improve the understanding of the community level within nested levels of the panarchy, and how the resilience of a community relates to that of social–ecological resilience as a whole. Communities are more than the sum of their individuals, households, and groups, are not necessarily cohesive, and comprise dynamic combinations of actors and groupings with multiple interests and shifting alliances (Agrawal and Gibson 1999). Communities of place interweave in complex ways with regions, nations, and the globe. For instance, a farming community's food production assists the food security of neighboring and distant regions, and the economy of its nation. Households and businesses that have many options and strategies provide a more diverse and hence resilient local economy, providing alternate opportunities where one part of the economy may fail. Enhancing capacity at the community level could thus assist resilience across many levels. As the community development literature has come to recognize (Flora et al. 2003), building a community involves building ecological as well as all other "capitals." Indigenous peoples worldwide have long understood this interdependency, wherein a healthy ecosystem supports human well-being, and a thriving society is best able to care for its environment.

Adaptive capacity is the capacity of actors in a system to influence resilience (Folke et al. 2010), and often works through social networks and learning communities (Goldstein 2012). Adaptive capacity is seen as an attribute at multiple levels, from the individual to community, and sometimes to higher levels of organization (Brown and Westaway 2011). Some of the social–ecological systems literature uses adaptive capacity in much the same sense as the terms *agency*, the capacity of an individual to act independently and to make one's own free choices, and *self-efficacy*, the belief in one's own ability to perform a task and to manage prospective situations (Brown and Westaway 2011, 325, 326). We view adaptive capacity as a latent property, which can be activated when people exercise their agency. The processes by which this occurs have not been well explored.

The notion of adaptive capacity, widely used in the social–ecological systems strand and the environmental change literature in general, covers some of the same ground as the notion of agency used elsewhere. Adaptive capacity is a property of the social part of the social–ecological system. Animals and plants show adaptations

Downloaded by [212.233.56.181] at 02:53 16 December 2015

in an evolutionary sense and at an evolutionary time scale, but only humans anticipate change and use social, political, and cultural means to influence resilience (Folke et al. 2010). An integrated analysis of community resilience should consider the interaction between adaptive capacity and agency on the one hand, and community characteristics (Figure 1) on the other. An integrated approach is concerned with how these community characteristics combine to produce the process of resilience. Adaptive capacity (as ability to influence resilience) and agency (as capacity of an individual or group to act independently) are both highly important. Resilient communities organize themselves and take action through activating characteristics such as those in Figure 1.

Community development processes tend to use practical and achievable participatory projects (e.g., improving a neighborhood or service), which community groups select and carry out themselves, as vehicles to empower the group or community through a series of small successes and learning experiences. Such processes build cohesion and a sense of community while achieving tangible outcomes. Problem-solving approaches to build social strengths and agency, resulting in outcomes such as infrastructure improvements and economic diversification, can be seen as resilience-building strategies. Such strategies incorporate, but go beyond, the spontaneous self-organizing as portrayed in the social–ecological systems literature. Self-organizing appears to activate capacities already inherent in a community; these could be improved further through community resilience-enhancing processes by building on core strengths by iterative processes.

Resilience is not always positive or desirable. For example, poverty cycles in inner-city environments are often very persistent (resilient) though hardly desirable. In these instances change agents seek transformation. Loss of livelihoods is another situation in which transformation may become desirable. For example, a small-scale fishing community that suffers from resource depletion may lose its ability to make a livelihood. If the option is available, converting the local economy to rely primarily on tourism income may make that community viable once again. It is likely that such transformations, and the related transformability of the system (Walker et al. 2004), involve building on community strengths (Figure 1).

Transformational change at lower levels may enable resilience at higher levels. The evidence from the social–ecological systems literature indicates that small-scale disturbances at lower levels, such as small forest fires, are beneficial in keeping away large-scale disturbances at higher levels, such as disastrous regional forest fires (Berkes and Folke 1998). Similarly, transformational changes in certain communities (e.g., from fishing to tourism) may help the region as a whole to become more resilient through increasing the diversity of livelihoods. Where deliberate transformational change at the regional scale is too costly or socially unacceptable, local transformational change in a sequential way can lead to feedback effects improving resilience at the level of the whole system (Walker et al. 2009).

Conclusions

In analyzing the productive common ground between the two strands of literature on community resilience, one from social–ecological systems and the other from psychology and mental health, we find that the first provides a richer set of analytical concepts and the second a deeper understanding and use of social science. The social–ecological systems strand is most relevant for communities of place (Maida

Downloaded by [212.233.56.181] at 02:53 16 December 2015

Downloaded by [212.233.56.181] at 02:53 16 December 2015

2007), particularly those communities interacting closely with their environments. Here the integrated social–ecological system is the appropriate unit for study. However, in other situations, the social–ecological system may not be as relevant to the resilience of a community as a social entity. The psychology/mental health strand has wider applicability for cases that do not involve resource dependence, or *require analysis of disasters and other impacts which are not primarily about the natural environment*. However, for many studies of community resilience, combining the strengths of the two strands through an integrated concept of community resilience may provide insights that may not otherwise be available.

An integrated concept of community resilience is not only about theory; it is equally about practice: How can adaptive capacity, self-organization and agency be supported and fostered through processes such as community development and community-based planning? Adaptive capacity and agency (Brown and Westaway 2011) can be facilitated by community members themselves through social learning (Goldstein 2008) or by external change agents, using well-known approaches in community development, such as building community strengths and relationships.

An integrated concept of community resilience would need to deal constructively with some dichotomies in social–ecological systems theory. One of these is between resilience and transformation. Although analytically useful, resilient change and transformation is more likely a continuum than a dichotomy; what constitutes an actual transformation of one social–ecological system into another is not always clear. General versus specified resilience is another problematic dichotomy. Some ecological problems require focusing on specified resilience, and the disaster management literature is definitely about specified resilience—to an earthquake, fire, flood, or landslides. Both concepts are valuable and yet the two kinds of resilience can be interdependent, either in being mutually supportive or in competing for management attention (Folke et al. 2010). In community resilience, we expect that generalized resilience is highly desirable, providing flexibility to cope with a wide range of crises, underpinning specified resilience where required. For instance, social networks are generically useful for general resilience, but also for some kinds of specified resilience (e.g., Almedom 2004, for communities displaced by war).

A critical analysis requires that we identify potentially relevant social science concepts about communities that have been overlooked or underappreciated in social–ecological resilience thinking. Social capital and networks, sense of place, values, and social identity all appear across the strengths identified earlier. One relative silence in the community resilience literature is the relevance of power relationships. While the literature recognizes the importance of the ability to cope with divisions within the community (Kulig 2000; Hegney et al. 2008a), the ability to deal with power will be most relevant in self-organizing ability. Migration, changes in age structure, and gender balance are clearly important in community functioning and thus presumably in community resilience as well. There has been little systematic interrogation of dimensions such as agency, self-efficacy, empowerment, optimism, and self-esteem in determining how people deal with shocks and stresses (Brown and Westaway 2011). More work is needed about the values and behavior that bond communities and cultures with their environments, and cross-cultural resilience. We know about sense of place, formation of social identity, and stewardship, but we do not know their significance for community resilience. Also, there is little in the literature, in either strand of community resilience, about methodologies. These are areas for further work that can inform new research

directions and practice toward improving the resilience of human–environment systems at the community level.

References

Adger, W. N. 2000. Social and ecological resilience: Are they related? *Progr. Hum. Geogr.* 23:347–364.

Agrawal, A., and C. C. Gibson. 1999. Enchantment and disenchantment: The role of community in natural resource conservation. *World Dev.* 27:629–649.

Almedom, A. 2004. Factors that mitigate war-induced anxiety and mental distress. *J. Biosocial Sci.* 36:445–461.

Almedom, A. M., B. Tesfamichael, Z. S. Mohammed, C. G. N. Mascie-Taylor, and Z. Alemu. 2007. Use of 'sense of coherence (soc)' scale to measure resilience in Eritrea: Interrogating both the data and the scale. *J. Biosocial Sci.* 39:91–107.

Armitage, D., F. Berkes, A. Dale, E. Kocho-Schellenberg, and E. Patton. 2011. Co-management and the co-production of knowledge: Learning to adapt in Canada's Arctic. *Global Environ. Change* 21:995–1004.

Berkes, F., J. Colding, and C. Folke, eds. 2003. *Navigating social–ecological systems: Building resilience for complexity and change*. Cambridge, UK: Cambridge University Press.

Berkes, F., and C. Folke, eds. 1998. *Linking social and ecological systems. Management practices and social mechanisms for building resilience*. Cambridge, UK: Cambridge University Press.

Berkes, F., and D. Jolly. 2001. Adapting to climate change: Social–ecological resilience in a Canadian western Arctic community. *Conserv. Ecol.* 5(2):18. http://www.consecol.org/vol5/iss2/art18 (accessed 18 September 2012).

Brown, K., and E. Westaway. 2011. Agency, capacity, and resilience to environmental change: Lessons from human development, well-being, and disasters. *Annu. Rev. Environ. Resources* 36:321–342.

Buikstra, E., C. Rogers-Clark, H. Ross, D. Hegney, C. King, P. Baker, and K. McLachlan. 2011. Ego-resilience and psychological wellness in rural people. In *Continuity versus creative response to challenge: The primacy of resilience and resourcefulness in life and therapy*, ed. M. J. Celinski and K. M. Gow, 273–290. New York, NY: Nova Science Publishers.

Buikstra, E., H. Ross, C. A. King, P. G. Baker, D. Hegney, K. McLachlan, and C. Rogers-Clark. 2010. The components of resilience: Perceptions of an Australian rural community. *J. Commun. Psychol.* 38:975–991.

Canadian Centre for Community Renewal. 2000. *The community resilience manual. A resource for rural recovery and renewal*. Port Alberni, Canada: Centre for Community Enterprise. http://communityrenewal.ca/sites/all/files/resource/P200_0.pdf (accessed 28 December 2011).

Carpenter, S. R., B. H. Walker, J. M. Anderies, and N. Abel. 2001. From metaphor to measurement: Resilience of what to what? *Ecosystems* 4:765–781.

Chapin, F. S. III, G. P. Kofinas, and C. Folke, eds. 2009. *Principles of ecosystem stewardship: Resilience-based resource management in a changing world*. New York, NY: Springer-Verlag.

Chaskin, R. J. 2008. Resilience, community, and resilient communities: Conditioning contexts and collective action. *Child Care Pract.* 14:65–74.

Davidson, D. J. 2010. The applicability of the concept of resilience to social systems: Some sources of optimism and nagging doubts. *Society Nat. Resources* 23:1135–1149.

Flora, C. B., J. L. Flora, and S. Fey. 2003. *Rural communities: Legacy and change*. Boulder, CO: Westview Press.

Folke, C. 2006. Resilience: The emergence of a perspective for social–ecological systems analyses. *Global Environ. Change* 16:253–267.

Downloaded by [212.233.56.181] at 02:53 16 December 2015

Folke, C., S. R. Carpenter, B. Walker, M. Scheffer, T. Chapin, and J. Rockström. 2010. Resilience thinking: Integrating resilience, adaptability and transformability. *Ecol. Society* 15(4):20. http://www.ecologyandsociety.org/vol15/iss4/art20 (accessed 18 September 2012).

Goldstein, B. E. 2008. Skunkworks in the embers of the Cedar fire: Enhancing resilience in the aftermath of disaster. *Hum. Ecol.* 36:15–28.

Goldstein, B. 2009. Resilience to surprises through communicative planning. *Ecol. Society* 14(2):33. http://www.ecologyandsociety.org/vol14/iss2/art33 (accessed 18 September 2012).

Goldstein, B. E., ed. 2012. *Collaborative resilience*. Cambridge, MA: MIT Press.

Gooch, M., D. Rigano, J. Butler, and L. Cullen. 2010. *Developing and refining indicators of community-scale resilience to changing water quality*. Unpublished report prepared for the Marine and Tropical Sciences Research Facility (MTSRF), Cairns, Australia. [Quoted in Johnson and Martin (2011, 15).]

Gunderson, L. H., and C. S. Holling, eds. 2002. *Panarchy. Understanding transformations in human and natural systems*. Washington, DC: Island Press.

Haque, C. E., and D. Etkin, eds. 2012. *Disaster risk and vulnerability*. Montreal and Kingston, Canada: McGill-Queens University Press.

Hegney, D., H. Ross, P. Baker, C. Rogers-Clark, C. King, E. Buikstra, A. Watson-Luke, K. McLachlan, and L. Stallard. 2008a. *Identification of personal and community resilience that enhance psychological wellness: A Stanthorpe study*. Toowoomba, Australia: Centre for Rural and Remote Area Health, The University of Queensland and University of Southern Queensland.

Hegney, D., H. Ross, P. Baker, C. Rogers-Clark, C. King, and E. Buikstra. 2008b. *Building resilience in rural communities toolkit*. Toowoomba, Australia: University of Queensland and University of Southern Queensland.

Holling, C. S. 1973. Resilience and stability of ecological systems. *Annu. Rev. Ecol. Systematics* 4:1–23.

Johnson, J., and K. Martin. 2011. *Managing for resilience of the Great Barrier Reef: Socio-economic influences*. Synthesis report prepared for the Marine and Tropical Sciences Research Facility (MTSRF). Cairns, Australia: Reef and Rainforest Research Centre. http://www.rrrc.org.au/publications/downloads/Theme-5-C2O-Johnson-J-et-al-2011-Managing-for-GBR-resilience-synthesis.pdf (accessed 26 September 2012).

Kelly, E. C., and J. C. Bliss. 2009. Healthy forests, healthy communities: An emerging paradigm for natural resource-dependent communities? *Society Nat. Resources* 22:519–537.

Kulig, J. C. 2000. Community resiliency: The potential for community health nursing theory development. *Public Health Nursing* 17:374–385.

Kulig, J. C., D. Edge, and B. Joyce. 2008. Understanding community resiliency in rural communities through multimethod research. *J. Rural Commun. Dev.* 3:76–94.

Kulig, J. C., D. Hegney, and D. S. Edge. 2010. Community resiliency and rural nursing: Canadian and Australian perspectives. In *Rural nursing: Concepts, theory and practice*, 3rd ed., ed. C. A. Winters and H. J. Lee, 385–400. New York, NY: Springer.

Kumpfer, K. L. 1999. Factors and processes contributing to resilience. In *Resilience and development: Positive life adaptations*, ed. M. D. Glantz and J. L. Johnson, 179–244. New York, NY: Kluwer Academic/Plenum.

Liu, J., T. Dietz, S. R. Carpenter, M. Alberti, C. Folke, E. Moran, A. N. Pell, P. Deadman, T. Kratz, J. Lubchenko, E. Ostrom, Z. Ouyang, W. Provencher, C. L. Redman, S. H. Schneider, and W. W. Taylor. 2007. Complexity of coupled human and natural systems. *Science* 317:1513–1516.

Lu, F. 2010. Patterns of indigenous resilience in the Amazon: A case study of Huaorani hunting in Ecuador. *J. Ecol. Anthropol.* 14:5–21.

Luthar, S. S. 2006. Resilience in development: A synthesis of research across five decades. In *Developmental psychopathology, Vol. 3, Risk, disorder, and adaptation*, 2nd ed., ed. D. Cicchetti and D. J. Cohen, 739–795. Hoboken, NJ: Wiley.

Downloaded by [212.233.56.181] at 02:53 16 December 2015

Luthar, S. S., and D. Cicchetti. 2000. The construct of resilience: Implications for interventions and social policies. *Dev. Psychopathol.* 12:857–885.

Maida, C. A., ed. 2007. *Sustainability and communities of place*. New York, NY: Berghahn.

Magis, K. 2010. Community resilience: An indicator of social sustainability. *Society Nat. Resources* 23:401–416.

Manyena, S. B. 2006. The concept of resilience revisited. *Disasters* 30:433–450.

Marschke, M., and F. Berkes. 2006. Exploring strategies that build livelihood resilience: A case from Cambodia. *Ecol. Society* 11(1):42. http://www.ecologyandsociety.org/vol11/iss1/art42 (accessed 18 September 2012).

Masten, A. S., and J. Obradović. 2006. Competence and resilience in development. *Ann. NY Acad. Sci.* 1094:13–27.

Norberg, J., and G. Cumming, eds. 2008. *Complexity theory for a sustainable future*. New York, NY: Columbia University Press.

Norris, F. H., S. P. Stevens, B. Pfefferbaum, K. F. Wyche, and R. L. Pfefferbaum. 2008. Community resilience as a metaphor, theory, set of capabilities, and strategy for disaster readiness. *Am. J. Commun. Psychol.* 41:127–150.

Ommer, R. 2007. *Coasts under stress. Restructuring and social–ecological health*. Montreal and Kingston: McGill-Queen's University Press.

Paton, D., and D. Johnston. 2001. Disasters and communities: vulnerability, resilience and preparedness. *Disaster Prevent. Manage.* 10:270–277.

Robinson, L. W., and F. Berkes. 2011. Multi-level participation for building adaptive capacity: Formal agency-community interactions in northern Kenya. *Global Environ. Change* 21:1185–1194.

Ross H., M. Cuthill, K. Maclean, D. Jansen, and B. Witt. 2010. *Understanding, enhancing and managing for social resilience at the regional scale: Opportunities in North Queensland*. Cairns, Australia: Marine and Tropical Sciences Research Facility, Reef and Rainforest Research Centre. http://www.rrrc.org.au/publications/social_resilience_northqueensland.html (accessed 2 January 2012).

Scheffer, M. 2009. *Critical transitions in nature and society*. Princeton, NJ: Princeton University Press.

Walker, B. H., N. Abel, J. M. Anderies, and P. Ryan. 2009. Resilience, adaptability, and transformability in the Goulburn-Broken Catchment, Australia. *Ecol. Society* 14(1):12. http://www.ecologyandsociety.org/vol14/iss1/art12 (accessed 18 September 2012).

Walker, B., C. S. Holling, S. R. Carpenter, and A. Kinzig. 2004. Resilience, adaptability and transformability in social–ecological systems. *Ecol. Society* 9(2):5. http://www.ecologyandsociety.org/vol9/iss2/art5 (accessed 18 September 2012).

Wilkinson, C. 2011. Social–ecological resilience: Insights and issues for planning theory. *Plan. Theory* 11:148–169.

Wilson, G. 2012. *Community resilience and environmental transitions*. Oxford, UK: Earthscan.

Zautra, A., J. Hall, and K. Murray. 2008. Community development, and community resilience: An integrative approach. *Commun. Dev.* 39: 130–147.

Downloaded by [212.233.56.181] at 02:53 16 December 2015
