# Supplementary material for: Systematic Review of Methods in Low-Consensus Fields: Supporting Commensuration through `Construct-Centered Methods Aggregation’ in the Case of Climate Change Vulnerability Research
Source: PLoS One. 2016 Feb 22;11(2):e0149071. doi: 10.1371/journal.pone.0149071 (PMC4762661; doi:10.1371/journal.pone.0149071)
Supplement: S2 File — Source articles and bibliography. (ZIP) [file pone.0149071.s002.zip › data requirements/Capaldo et al.pdf]

A model of vulnerability to food insecurity

Jeronim Capaldo, Panagiotis Karfakis,

Marco Knowles and Mark Smulders

ESA Working paper No. 10-03

August 2010

Agricultural Development Economics Division

Food and Agriculture Organization of the United Nations

www.fao.org/economic/esa

A Model of Vulnerability to Food Insecurity[1]

Jeronim Capaldo, jeronim.capaldo@fao.org

Panagiotis Karfakis, panagiotis.karfakis@fao.org

Marco Knowles, marco.knowles@fao.org

Mark Smulders mark.smulders@fao.org

Food and Agriculture Organization

Economic and Social Development Department

Agricultural Development Economics Division

April, 2010

Abstract

Empirical findings show that access to adequate and sufficient food in developing countries is unstable, suggesting that whether a household or individual is food secure at any point in time is best thought of in a dynamic sense. The more widely used food security analysis methods mainly consider *current* access to food, failing to provide policy makers with forward-looking information. While drawing on household survey data, the vulnerability analysis model presented here provides estimates of the probability that a given household will lose or gain access to sufficient food in the near future. We propose a model of vulnerability analysis that can improve policy design and targeting. We test the model with data from a survey of Nicaraguan households.

Keywords: vulnerability, food security, policy planning, policy targeting, Nicaragua

JEL Classification: I32, O2, Q18

[1] The authors are Economists in the Agricultural Development Economics Division (ESA) of the Food and Agricultural Organization of the United Nations (FAO), based in Rome, Italy. We thank Sumiter Broca, David Dawe, James Tefft and Alberto Zezza of FAO for their comments and suggestions. The findings, interpretations, and conclusions expressed in this paper are entirely those of the authors. They do not necessarily represent the views of FAO.

1. Introduction

Widely accepted empirical findings show that access to adequate and sufficient food in many countries is unstable (FAO, 2009). That is, regardless of the specific measurement adopted, many households frequently move in and out of a state of undernutrition, suggesting that the notion of *food insecurity* is best thought of in a dynamic sense. Not surprisingly the first World Food Summit led to defining food security as universal and *permanent* access to sufficient, safe and nutritious food (FAO, 1996).

An immediate practical consequence that can be drawn from this observation is that food security policies should be based as much on the assessment of households' *current* conditions as on the expectation of their *future* access to food. In order to reduce the *threat* of future undernutrition, policy design should address the uncertainty that households face alongside their risk-management options. In contrast to this logic, however, widely used food security analyses mainly consider current access to food.

Vulnerability analysis (VA) offers a solution to this problem by providing a quantitative estimate of the probability that a given household will lose access to sufficient food in the near future. In two decades of applications many subfields of economics, from finance to poverty, have developed their own standard model of VA. In the field of food security analysis, a standard model has not arisen yet and different analytical methods coexist. The starting point of this paper is a review of some of the methods employed.

In search of a suitable standard model that supports policy making, we have adapted analytical advances made in the field of poverty analysis to our area of interest, namely food security analysis. A welcome by-product of this endeavor is that, by setting a common language for the analysis of poverty and food insecurity, we maximize comparability between the two fields.

The present proposal originates from the need to overcome the widespread lack of time series data in developing countries. We construct an estimation procedure that can be easily replicated using data from a single household survey and whose results have a straightforward interpretation. Furthermore, in order to answer specific policy questions, our procedure can be adapted to exploit available information expressed in quantitative variables.

Compared to static food security analyses that categorize households into either “food secure” or “food insecure”, the present model allows households to be classified into four categories of food security. Relying on estimates of future calorie consumption (and its probability distribution), we disaggregate households into “chronically food insecure”, “transitory food insecure, “permanently food secure” and “transitory food secure”. This categorization improves the targeting of scarce resources since it enables policy makers to distinguish currently food insecure households that are able to improve their situation without external assistance (i.e. the transitory food insecure) from currently food insecure households that are not able to improve their situation without external assistance (i.e. chronically food insecure).

Once chronic and transitory lack of access to food, are separated, the question arises whether their underlying causes and remedies are different. If they are, and if we can identify and locate food insecure households, the model could also be useful for policy formulation. For example, while one-time transfers may be appropriate for transitory and potentially food insecure households, chronic food insecurity conditions call for a mix of transfers and asset-building incentives.

This forward looking model identifies the risks that households are exposed to, while also estimating the magnitude of the impact of these risks on household food security, depending on the risk management strategies that are adopted. This is particularly useful for preparedness planning since it allows decision makers to prioritize and design interventions based on (i) the

likelihood that different risks will materialize; (ii) the expected food security impact of these risks on different types of households; and (iii) the effectiveness of different risk management options.

The insight into risk and risk management, together with the identification of the chronically food insecure, helps identify households that may be in a "hunger trap" and define strategies to overcome their predicament. A common concern is that exposure to risks and the difficulties in managing them may induce food insecure households to choose low-risk income-earning strategies, which however may also result in low-mean returns. Through interventions that reduce exposure to risks or improve risk-management (e.g. through provision of transfers under certain conditions), households can make productivity-enhancing investments and gradually escape the hunger trap.

The remainder of the paper is organized as follows. In section 2, we review key contributions to the quantitative analysis of vulnerability and identify the current analytical gap. In section 3, we describe the conceptual foundations and the analytical structure of our model. Section 4 contains a description of the data we use in our empirical application, while section 5 contains our quantitative results and examples of profiling based on the vulnerability indicator. In section 6 we present our conclusions.

2. Economic Literature on Vulnerability

In the 1980s, following Sen's seminal works (Sen, 1983), economic research on poverty began to acknowledge that risks affect households in different ways, depending on their degree of exposure to them and their risk-management ability. The concept of vulnerability, that had found fertile ground in finance (Fishburn, 1977) after its importation from natural sciences, was then introduced in economics as way of approaching uncertainty. Theorists soon took up explaining how hunger can be generated in competitive markets fueling a line of research in general equilibrium theory known as "the survival problem" (Hashimzade and Majumdar, 2005). Recently the theoretical framework has privileged inter-temporal optimization with fully rational agents constrained by market imperfections (Dercon 2001).

Empirical applications to the analysis of poverty and deprivation in general, at both the micro and macro level, made vulnerability analysis increasingly popular in academic and policy circles (Lybbert et al 2004). In these applications the analysis essentially consists of calculating the probability that a household's performance indicator, such as total consumption or food consumption, will fall below a given threshold in the future. Common assumptions in statistical inference allow interpreting this probability as an estimate of the future poverty ratio (i.e. the share of the population who will "underperform").

Existing models of vulnerability can be grouped into two large categories: (a) models that analyze vulnerability to stochastic events – usually shocks, hazards or risks – and (b) models that analyze vulnerability to the outcomes of those events. The former, typically concerned with short-term disaster management, are based on strong ad hoc assumptions. The latter focus on the longer term patterns of poverty and deprivation and can be further split into models that measure outcomes with statistical indicators (consumption, land ownership, human development index, etc.) and models that measure outcomes in terms of utility (see Ligon and Schecter, 2004, for a review).

Whenever policy formulation requires quantitative information, statistical outcome-based models are most appropriate for vulnerability analysis. Indeed they have produced many interesting empirical results (Ravallion, 1988; Chaudhuri, 2001; Chaudhuri *et al* 2002; Christiaensen and Boisvert, 2000; and Kamanou and Morduch, 2002; Christiaensen, and

Subbarao, 2005; Sarris and Karfakis, 2007; Gunther and Harttgen 2010).

Since there is no established functional relationship between food consumption (e.g. measured in kilocalories or in money terms) and total household consumption, the analysis of vulnerability to poverty cannot be immediately applied to food insecurity. Even when this problem is sidestepped with an *ad hoc* assumption of linearity, the application of this model for analyzing vulnerability to food insecurity in developing countries is problematic due to the scarcity of relevant time-series and panel data on which these models rely. Hence, analyzing vulnerability to food insecurity requires a model that provides the useful results obtained from poverty analysis, but that addresses the specific determinants of food insecurity and can be estimated from cross-sectional data.

Although a mainstream model has not yet arisen in the research on vulnerability to food insecurity, a few interesting models exist. FAO has added to the diversity of approaches through the introduction of the analysis of resilience (Alinovi *et al*, 2008) to assess how households adjust their livelihoods after a series of shocks have occurred. This analysis assesses longer term patterns with non-parametric methods.

Christiaensen and Boisvert (2001) have proposed the main economic model of vulnerability to food insecurity to date by drawing on the analysis of vulnerability to poverty. They define vulnerability as the probability V_t that the household's expected dietary energy consumption x_{t+1}, measured in Kilocalories, will fall below a threshold *z*:

$$V_{t,\alpha} = F(z) \int_{\alpha}^{z} \left(z - x_{t+1}\right)^{\alpha} \frac{f\left(X_{t+1}\right)}{F(z)} dx_{t+1} \qquad (1)$$

Vulnerability, in this formulation, is null whenever $x_{t+1} \geq z$. When, instead, expected dietary energy consumption is below the threshold, the index depends on α. Interestingly, for $\alpha = 0$, vulnerability does not depend on the extent of the shortfall. To overcome this, the authors consider vulnerable only those households whose index falls below a vulnerability threshold called θ.

The index is used to evaluate the future nutritional adequacy of a two-period consumption plan stemming from inter-temporal optimization in the presence of imperfect capital markets. Uncertainty and risks enter this model in the form of an uncertain future income (and, therefore, consumption), whose value is predicted through assumptions on the stochastic properties of the environment. However, even though two periods are considered, the households' problem is solved with static optimization. This is possible because there is no endogenous state variable in the model. As a result the model's applicability is limited to scenarios in which there are no assets lasting more than one period. If one were to introduce such assets in order to account, for example, for livestock or machinery, static optimization techniques would no longer be applicable and the model would change significantly.

Christiaensen and Boisvert estimate levels of vulnerability in Northern Mali and compare the effects of an investment policy (irrigation projects and inclusion of more households into the irrigated areas) to the effects of encouraging migration out of the drought-prone region. In line with Lipton and Ravallion (1995) their simulations suggest that investment policies are the best way to reduce vulnerability to food insecurity.

Christiaensen and Boisvert, use panel data and employ two-stage least square regression. Unfortunately this hinders the model's applicability for two reasons. Firstly, the availability of panel data on food consumption and shocks for developing countries is limited and, secondly,

linear regression techniques are not optimal, although very common, for estimating relationships in the absence of a compatible underlying theory. Since there is not yet an established theory of food consumption and of its distribution across different stochastic scenarios, non-parametric estimation models would be more suitable.

In a companion paper, Christiaensen et al. (2000) propose to combine their vulnerability index linearly with other indices of current deprivation defined over a $[0,1]$ interval and respecting the usual axioms of poverty indicators. The authors define the *j*-th dimension of individual *i*'s deprivation as:

$$
\begin{aligned}
P_j(x_{ij} \mid z) &= 1 \qquad if \quad x_{ij} = 0 \\
&= 0 \qquad\qquad x_{ij} < z \\
&= f(x) \qquad\quad x_{ij} \geq z
\end{aligned}
\qquad (2)
$$

Aggregating the indices of different dimensions of deprivation they obtain:

$$
fis_i = \sum_j a_j P_j(x_{ij} \mid z), \quad \sum_j a_j = 1, \quad a_j < 1 \qquad (3)
$$

In this index, that can be averaged to obtain a population index, the relative importance of current deprivation and vulnerability to future deprivation are given by the weights α_j. The authors determine future food consumption assuming a loglinear distribution and using a regression that generates heteroskedastic residuals. They then use the estimates to calculate their vulnerability index and combine it linearly, with equal weights, with an index of current food deprivation. Applying this procedure to Mali, the authors argue that "three quarters of the bi-dimensional food security index depends on vulnerability". This means that food insecurity – as it is estimated considering both current and future deprivation – is mainly due to its prospective component, i.e. vulnerability.

Although useful in empirical analysis, the index could be strengthened from the logical point of view. Indeed the shares of each dimension (present and future) depend on the arbitrary weights of the linear combination and the fifty-fifty choice may impose on households a larger relative weight of the future than is reflected in their consumption choices. Perhaps a choice more in line with the index's theoretical foundations would be to combine the two dimensions using the inter-temporal rate of substitution between future and present consumption. In this case, however, the weight assigned to vulnerability would be smaller, exhibiting myopia, as shown by a long tradition of general equilibrium calibrations.

A similar approach is taken by Scaramozzino (2006) who goes back to the financial roots of vulnerability analysis, suggesting that the notion of *Value at Risk* (VaR) could be a suitable basis for measuring the likelihood of future food insecurity.

3. A model of vulnerability to food insecurity

3.1. Conceptual underpinnings

Our model is based on the Social Risk Management approach (Holzmann and Jørgensen 2000; World Bank 2000) and, more specifically, on the conceptual framework drawn from it by Løvendal and Knowles (2005). In this framework vulnerability is the result of a recursive process: *current* socio-economic characteristics and exposure to risks determine households' *future* characteristics and their risk-management capacity. At every point in time households' current food security status is affected by their past status and affects their future status. Figure 1 represents graphically this recursive connection.

Figure 1. Vulnerability to future food insecurity: Conceptual Framework

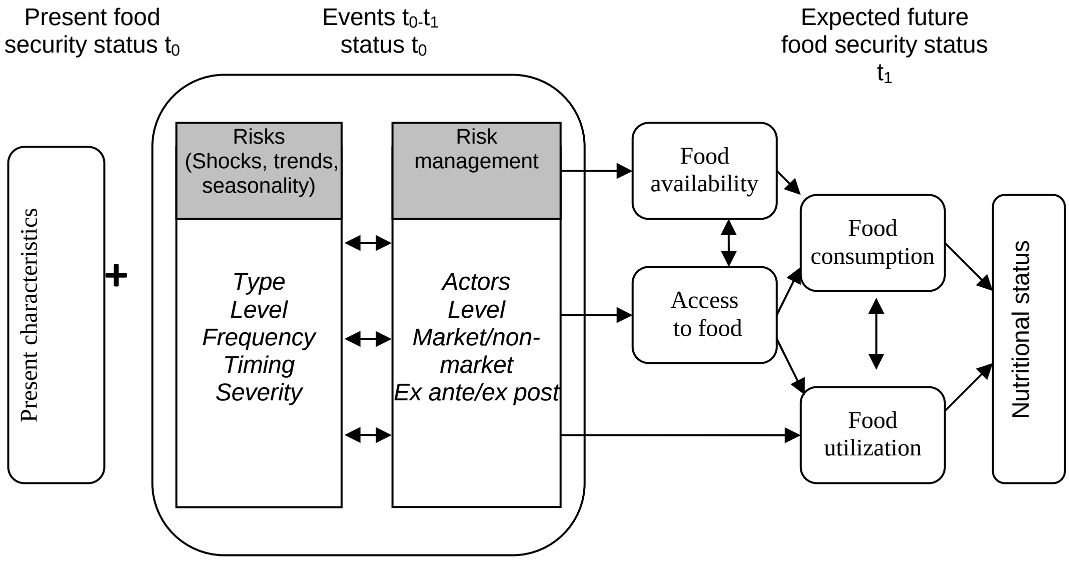

Source: Løvendal -Knowles, 2005

In this conceptual framework, as in the family of economic models with "overlapping generations", households have a two-period lifetime consisting of the present (t_0) and the future (t_1). Present characteristics are known to households and policymakers and determine households' current food security status. Future characteristics, on the other hand, are unknown to households and policymakers. Between the present and the future ($t_0 - t_1$), a number of previously unknown factors (i.e. risks of different kinds) manifest themselves and determine, depending on households' risk management abilities, the future food security status (as measured through various dimensions, including e.g. food consumption and nutritional status). Both the current food security status and the expectation of the future status determine the overall household food security situation over a period of time[2]. Given that each household's food security status is assessed in the present, but includes an expectation of the future, vulnerability to food insecurity in this model is dynamic and forward-looking.

[2] The period over which the food security status is estimated may vary depending on a case by case basis.

The analytical model proposed here captures the conceptual framework's recursive structure in two ways: on the one hand it specifies econometrically the relationship between a measure of food security status (food consumption expressed in kilocalories) and a set of household characteristics; on the other hand it explains how current characteristics, risks and risk management capacities affect the likelihood of a favorable (or unfavorable) future food security status. However, in the form presented in this paper, the model does not offer an explicit description of households' dynamic decision problem. Such description requires specifying the process of asset accumulation (or exhaustion), which we leave to our future work.

3.2. Algebraic Structure

In the model presented here, we follow Chaudhuri *et al*'s suggestion and assume that all the cross-sectional variability of our crucial variable – dietary energy consumption, measured through kilocalorie – depends on the household's observable characteristics. The same assumption underlies all time series econometrics, although it is not always explicit. This assumption allows us to estimate vulnerability using cross-sectional data from a single point in time, thereby limiting data requirements. The analytical methodology is similar with Chistiaensen and Boisvert in that food consumption is approximated by kilocalorie consumption. However we employ here a cross section of data allowing for wider applicability of the model given the increasing availability of one-off household surveys in developing countries. Chistiaensen and Boisvert use panel data from Mali instead.

Christiaensen and Subbarao, employ a pseudo-panel for Kenya, to study vulnerability to consumption poverty. The authors recognize that even with long and balanced panels is difficult to describe the stochastic character of consumption and, in order to circumvent the problem they include information on the incidence of past shocks in their analysis. Such information assists in estimating the impact of covariate or idiosyncratic shocks on consumption. This approach, even though imperfect, is followed in the present study as well.

We follow a three-step process. In order to project future consumption, we first estimate a model of calorie consumption whereby the latter is a function of a number of household characteristics. We find that the residuals generated by this estimation are correlated to each other and exhibit different variances (i.e. they are heteroskedastic). These are two statistically undesirable properties, signaling that the model is unable to capture all the systematic variability of the dependent variable. To address this, we take a second step which involves estimating, via weighted least squares, a model of the residuals that explains their variability. This second step gives us estimates of the residual variance. Lastly, we use the estimate of variance of the residuals to calculate the probabilities that kilocalorie consumption, which we assume normally distributed, may be lower than an acceptable threshold.

For a generic household h let c_h indicate kilocalorie consumption and X_h be a vector of characteristics assumed constant over time, such as household size, location, etc. Assuming for simplicity a linear dependence, we can express each household's calorie consumption as follows:

$$c_h = X'_h\beta = \beta_1 x_{h1} + \dots + \beta_2 x_{h2} + \dots + \beta_J x_{hJ} \quad (7)$$

where β is a vector of parameters that are the same for all households (note the absence of the h index).

Considering all households in one multivariate equation, we have:

$$C = X\beta = \begin{bmatrix} \beta_1 x_{11} + \ldots + \beta_2 x_{12} + \ldots + \beta_J x_{1J} \\ \vdots \\ \beta_1 x_{h1} + \ldots + \beta_2 x_{h2} + \ldots + \beta_J x_{hJ} \\ \vdots \\ \beta_1 x_{H1} + \ldots + \beta_2 x_{H2} + \ldots + \beta_J x_{HJ} \end{bmatrix} \quad (8)$$

where $C = [c_1 \quad \ldots \quad c_h \quad \ldots \quad c_H]'$ and $X = [X_1' \quad \ldots \quad X_h' \quad \ldots \quad X_H']'$.

The first step of our 3GLS procedure consists of estimating the multivariate equation obtaining estimates $\hat{\beta}$ of the parameters that explain calorie consumption but for a residual component $u = [u_1 \quad \ldots \quad u_h \quad \ldots \quad u_H]$:

$$C = X\hat{\beta} + u \quad (9)$$

As we anticipated above, the predicted residuals from (9), are correlated and heteroskedastic; therefore, as a second step, we assess their dependence on the same explanatory variables through a set of parameters γ. We estimate the equation:

$$u = X\hat{\gamma} + \varepsilon \quad (10)$$

where ε is the vector of residuals of this second estimation, showing all the desirable properties of residuals that u does not have.

From the deterministic part of equation (10) and after correcting again for heteroskedasticity, we derive a consistent estimate of the household variance of food consumption $\hat{\sigma}_u^2$. We use $\hat{\sigma}_u^2$, in the last step of our procedure, to compute each household's vulnerability to food insecurity. Assuming that vulnerability v distributes normally, each household's probability of food insecurity is given by a determination of:

$$v_h \sim N(E(u_h), \sigma_h^2) \quad (11)$$

The ultimate outcome of our calculations is a set of estimates v_h (one for every household h) of the probability that each household faces of falling below the minimum energy requirement in the future. Each estimate takes values in the interval $[0,1]$. The extremes of the interval represent two opposite certainties: when $v_h = 0$, household h will consume in the future with certainty *at least* the minimum amount of calories prescribed by the threshold; when $v_h = 1$

household h will consume *less* calories in the future then prescribed by the threshold. In all intermediate cases, when $0 < v_h < 1$, no particular outcome is anticipated *ex ante*.

Since we can attach an index v_h to all households, the question arises which households should be considered vulnerable in between the two extremes. This is particularly important for the design on any mitigating interventions and associated policy formulation. It makes sense to consider households that have an estimated vulnerability close or equal to unity as "vulnerable" and those with a vulnerability index close or equal to zero as "non vulnerable". But, as we move towards the center of the spectrum, the distinction becomes less obvious and the need for an arbitrary[3] cut-off point arises. Among the many choices of cut-off points, two are the most widely used: the median and the 0.5 value.

Choosing the median probability of vulnerability to food insecurity implicitly emphasizes the importance of inequality: the vulnerable are those who exhibit a relatively high level of vulnerability, regardless of the absolute value of the index. If the cutoff is the median only those with the highest relative levels of vulnerability will be considered vulnerable even if most households exhibit a very high probability of undernutrition in absolute terms. Also, households with a very low absolute probability of undernutrition may be considered vulnerable if the rest of the population has even lower values.

On the other hand, choosing the 0.5 value as a cutoff point emphasizes the absolute likelihood of undernutrition: the vulnerable are those who are more likely to be undernourished than not. With this cutoff, all or no households can be considered undernourished, which is an impossible event when the median is selected. The choice of the cutoff depends on the purposes of the analysis. The median would be more appropriate when designing policies to redress inequality, whereas the 0.5 value is more appropriate when planning interventions to address absolute deprivation. In this paper we adopt the 0.5 value, considering 'vulnerable' those households whose probability of future undernutrition is higher than the probability of sufficient nutrition.

In the following sections, after estimating vulnerability to food insecurity of rural households in Nicaragua, we analyze co-movements of the vulnerability index with other household characteristics to identify different types of vulnerable households – in accordance with their socio-economic characteristics – and to assess the impact of risk and risk management strategies.

[3] So far no analytical method has been developed to select endogenously the vulnerability threshold.

4. Data

We analyze a sample of 1831 rural households from Nicaragua, surveyed in the 2001 *Encuesta Nacional de Hogares Sobre Medición de Nivel de Vida*, by the *Instituto Nacional de Estadísticas y Censos - INEC de* Nicaragua. Constructed variables used in the analysis were prepared by the Rural Income Generating Activities (RIGA) project team at FAO.

We estimate daily per capita kilocalorie consumption as a function of several variables representing the households' demographic and social characteristics, asset holdings, liquidity constraints, access to infrastructure, occurrence of shocks and geographic location. Special attention is given to households that are linked to - or earn a significant proportion of their livelihoods from the agricultural sector. Table 1 provides a list of all variables, including their mean value and standard deviation. We have omitted from Table 1 the dummy variables for household location.

Table 1: Summary of variables

Variable	Unit	Mean	Standard deviation
Kilocalories per capita	Kilocalories	1958.54	5474.31
Age of hh head	Years	46.15	16.26
Highest education in hh	Years	5.18	3.60
Single head	Proportion	0.23	0.42
Female head of hh widow	Proportion	0.07	0.26
Female headed hh	Proportion	0.18	0.39
Hh labor	Proportion	0.47	0.23
Indigenous household	Proportion	0.04	0.20
Hh size	Units	5.84	2.85
Rooms	Units	1.94	1.09
Cement floor in house	Proportion	0.14	0.35
Telephone in hh	Proportion	0.01	0.11
Hh members participating in comm. org.	Units	0.38	0.70
Access to hh migration network	Proportion	0.05	0.22
Access to safe water	Proportion	0.53	0.50
Bikes owned	Units	0.30	0.61
Radios owned	Units	0.57	0.54
TVs owned	Units	0.19	0.39
Distance to nearest primary school	Km	1.70	3.65
Time to nearest health facility	Min	13.79	14.66
Distance to nearest major road	Km	47.01	93.77
Land owned	Hectares	7.09	18.14
Cattle	Units	3.55	13.77
Pigs	Units	1.29	2.86
Horses	Units	0.30	2.02
Drought shock	Proportion	0.24	0.43
Illness shock	Proportion	0.13	0.34
Government assistance programs	Units	1.32	1.54
Non-government assistance programs	Units	0.34	0.72

Land operated	Hectares	7.55	18.54
Access to irrigation	Proportion	0.01	0.09
Income from farming activities	Proportion	0.42	0.43
Income from farm sales	Proportion	0.12	0.22

Information on the structure of a household includes the age of the head of household (which is also a proxy for working experience), gender, marital status, language spoken (as a proxy for households belonging to an indigenous group) and the share of female labor. The latter also approximates labor availability within the household. We observed a relatively high proportion of single- or female-headed households (23% and 18% respectively).

Household assets are assessed in using education, as well as wealth-related variables (number of rooms, cement floor, telephone, access to safe water, bikes, radios, TV sets owned[4]), and social capital through participation of members in community organizations. Moreover, different types of livestock and land assets are also taken into account to approximate household wealth and potential credit-related constraints. We use access to a network for migration as a measure of the ability of a household to receive assistance from members living outside the location and as a proxy of a diversified income portfolio. Distance from a road, school, and health facilities, are variables used for measuring a household's access to infrastructure.

We use ex-post data on shocks and risk management strategies. These include information on the incidence of a covariate shock (such as drought) and an idiosyncratic shock (illness), as well as the number of government and non-governmental programs from which households received assistance. In this application, we are not able to complement this with information on future risks and risk management strategies. We note that nearly a quarter of the rural households report being affected by drought.

Finally, we also use a set of variables that reflect the agricultural characteristics of a household (land cultivated, irrigation, and shares of income from farm activities and farm sales). More than 40 percent of household income stems from agricultural activities in rural Nicaragua; however most of it is used for home consumption, while only 12 percent of income is derived from farm sales. Almost all agricultural households rely on rain-fed agriculture, with only 1 percent of households reporting the use of irrigation.

5. Results

After accounting for heteroskedasticity through the use of generalized least squares, we estimate vulnerability to food insecurity as the normal probability that the "individual minimum dietary energy requirement under light physical activity" exceeds the expected individual dietary energy consumption (measured in kilocalories). Since the main purpose of this paper is to propose a methodology to analyze and estimate vulnerability, we ignore possible econometric complications that are not directly relevant. However, by all means the results presented here are to be considered preliminary.

Endogeneity concerns for some of the explanatory variables are fully acknowledged. In this exercise, however, econometric analysis serves as a vehicle to estimate relative vulnerability and not to identify direct causes of inadequate food consumption. A discussion on some of the

[4] Bikes, radios and TV sets are also proxies for transaction and information related constraints.

correlations, which preliminarily trace causes of insufficient dietary energy consumption, follows in the next few paragraphs.

Our estimates of the model of calorie consumption (equation 9) and the variance of consumption (equation 10) are reported in Table 2. We employ two regression specifications in order to estimate expected per capita calorie consumption and its variance. The first specification (left-hand panel) is a generic one in which household preferences, assets, access to infrastructure, incidence of shocks and coping capacity are used as explanatory variables for per capita food consumption. Such specification may be applied in different contexts (i.e. urban versus rural areas), with minor adjustments.

Table 2: Regression results (all variables in logs except shares and binaries)

	**Without variables specific to agricultural livelihoods**				**With variables related to agricultural livelihoods**			
	**Log pc kcal consumption**		**Variance of consumption**		**Log pc kcal consumption**		**Variance of consumption**	
	Coef.	t-stat	Coef.	t-stat	Coef.	t-stat	Coef.	t-stat
Log age of hh head	-0.0205	(-1.18)	-0.118	(-0.67)	-0.0194	(-1.13)	-0.262	(-1.39)
Log highest years of education in hh	0.0221**	(2.45)	0.128	(1.53)	0.0205**	(2.31)	0.236***	(2.67)
Single head	-0.0305	(-1.43)	-0.449**	(-2.23)	-0.0417*	(-1.92)	-0.139	(-0.65)
Female head of hh is widow	0.00240	(0.09)	-0.488*	(-1.92)	0.0115	(0.42)	-0.459	(-1.64)
Female headed hh	-0.00871	(-0.37)	0.383*	(1.72)	-0.0112	(-0.47)	0.116	(0.50)
Share of female hh labor	0.00144	(0.05)	0.288	(1.01)	-0.00375	(-0.13)	-0.0510	(-0.17)
Indigenous household	0.0436	(1.39)	0.153	(0.59)	0.0646**	(2.18)	-0.338	(-1.27)
Log hh size	-0.144***	(-10.98)	-1.583***	(-11.59)	-0.141***	(-10.92)	-1.480***	(-10.35)
Log no of rooms	0.0504***	(4.04)	0.179	(1.51)	0.0460***	(3.76)	-0.0930	(-0.74)
Cement floor	0.0278	(1.54)	-0.00609	(-0.03)	0.0281	(1.50)	0.162	(0.81)
Telephone in hh	0.0809	(1.06)	0.0991	(0.12)	0.102	(1.31)	0.179	(0.18)
Log members participating in comm. org.	0.0130	(0.49)	-0.229	(-0.77)	0.00303	(0.10)	0.541	(1.59)
Access to hh migration network	0.0115	(0.39)	0.260	(0.99)	0.00872	(0.36)	-0.806***	(-3.34)
Access to safe water	-0.0206	(-1.57)	0.268**	(2.16)	-0.0245*	(-1.89)	0.173	(1.33)
Log no of bikes owned	0.00789	(0.21)	0.306	(0.77)	0.00840	(0.23)	0.250	(0.59)
Log no of radios owned	-0.0379	(-0.68)	0.414	(0.79)	-0.0177	(-0.32)	0.342	(0.62)
Log no of TVs owned	-0.0625	(-0.34)	-1.283	(-0.65)	-0.0207	(-0.11)	-1.360	(-0.63)
Log distance to nearest primary school	-0.00167	(-0.38)	-0.0252	(-0.60)	-0.000561	(-0.13)	0.0288	(0.65)
Log time to nearest health facility	-0.000255	(-0.13)	0.0326*	(1.81)	-0.00046	(-0.25)	0.0323*	(1.71)
Log distance to nearest major road	-0.0160***	(-3.38)	-0.122***	(-2.71)	-0.0150***	(-3.18)	-0.120**	(-2.49)
Log land owned (ha)	-0.0106**	(-2.12)	-0.127***	(-2.90)	0.000886	(0.14)	0.00135	(0.02)
Log no of cattle	0.00632	(0.84)	0.0359	(0.53)	0.0109	(1.43)	0.0930	(1.27)
Log no of pigs	-0.0139	(-1.42)	0.0855	(1.08)	-0.00796	(-0.82)	0.170**	(2.07)
Log no of horses	0.00338	(0.18)	-0.0264	(-0.16)	0.00164	(0.08)	0.0678	(0.38)
Drought shock	-0.0116	(-0.72)	0.351**	(2.19)	-0.00282	(-0.18)	0.338**	(2.03)
Illness shock	-0.0174	(-1.13)	-0.329**	(-2.40)	-0.00622	(-0.38)	0.174	(1.17)
Log no of gov't assistance programs	0.00559	(0.41)	0.365***	(2.64)	0.0112	(0.81)	0.266*	(1.81)
Log no of non-gov't. assistance programs	-0.0345	(-1.35)	-0.293	(-1.11)	-0.0312	(-1.18)	-0.288	(-1.03)
Log no of land operated					-0.0111	(-1.64)	-0.130**	(-2.16)
Access to irrigation					-0.0237	(-0.42)	-0.214	(-0.52)
Share of income from farming activities					-0.098***	(-4.83)	-0.566***	(-2.87)
Share of income from farm sales					0.0855**	(2.54)	-0.198	(-0.60)
Constant	7.807***	(104.64)	-1.670**	(-2.24)	7.835***	(104.29)	-0.869	(-1.07)
R squared	0.17		0.17		0.19		0.17	
No of cases	1831		1831		1831		1831	

Significant at 0.10: *, 0.05: **, 0.01: ***. Department dummies included but their coefficients are not reported.

The second specification (right-hand panel), incorporates variables that capture agriculture-specific features since this is the dominant livelihood activity in rural areas. Both specifications lead to similar estimates of vulnerability thus confirming the robustness of our methodological approach. Nevertheless, we prefer the second specification since it provides richer contextual information while resolving specific estimation and interpretation issues that are discussed below.

In both specifications, variables related to household demographics and assets perform as expected. Assets and human capital positively contribute to higher levels of calorie consumption. In particular, education (an asset from an economic perspective), and the number of rooms in a house (an approximation of household wealth), have the largest positive correlation with the level of food consumption. Single-headed households are more vulnerable and thus more likely to face reduced food consumption in the future, whereas indigenous households are likely to consume more kilocalories per capita.

Households with migrant family members face a significantly smaller variance in their food consumption. This possibly indicates the positive impact of remittances (in other words, it reflects the effect on food consumption resulting from a more diversified household income).

Better access to public infrastructure also positively correlates with food consumption; increased distance from a public road is strongly linked to a reduction in the level of food consumption. However, the greater distance from a public road is also associated with lower variance in food consumption, possibly indicating low transmission of market volatility. This result is robust across both regression specifications. Finally, increased distance from health services appears to cause an increase in the variance of consumption. The result is significant at 10 % level in both specifications and may indicate an indirect impact of health risks on food security.

As expected, we observe that 'drought' and 'illness' have a negative impact on the level of food consumption. The results are not statistically significant, possibly because of the binary specification of the shock variables, which means that there is limited information on the intensity of the shocks that each household has experienced. Nevertheless, across both specifications the incidence of a drought has a robust positive effect on the variance of food consumption. On the other hand, while in the first specification 'illness' increases the variance of consumption, the effect disappears in the second specification.

Due to a lack of specific data, we approximate 'shock coping capacity' in using the number of government and non-government programs from which households receive assistance. This approximation is not ideal, given that such projects are not necessarily related to the above-mentioned shocks and may constitute social programs that are irrelevant to the shocks. While considering these imperfections, the regression results are not entirely surprising. The positive association between 'government assistance programs' and variance in 'food consumption' may reflect poor targeting or design imperfections, as frequently discussed in the literature.

The next paragraphs discuss agriculture-related variables and their impact on food consumption and its variance. We employ five such variables, as mentioned above. 'Land ownership' is used to approximate assets as well as access to credit since land may be used as collateral. In the first specification, land ownership reflects the degree of households' reliance on agriculture-based income generating activities. This explains the strong negative impact of land ownership on food consumption and its variance.

When the characteristics of agriculture-based livelihoods are explicitly considered, as in the second specification, some important features of agricultural households in rural Nicaragua become evident. Firstly, a greater dependence on agriculture does not automatically lead to a household being food secure – evidence shows that the greater the share of income from agriculture and related activities, the lower the level of per capita calorie consumption. However, a greater share of income from agriculture appears to reduce volatility in food consumption as the significant negative impact on its variance indicates. Consistent with the above, the larger the size of cultivated land the lower the volatility in food consumption[5].

These results suggest that a household livelihood largely dependent on agriculture is correlated with lower, but more stable levels of food consumption. The statistically insignificant impact of land ownership on food consumption also suggests the presence of non-binding credit constraints for these households. On the other hand, the share of income from farm sales has a significantly positive impact on food consumption, suggesting that households that earn part of their livelihoods from marketing their agricultural produce are less vulnerable to becoming food insecure.

Following the regression analysis, the vulnerability indicator (equation 11) is computed using predicted kilocalorie consumption and its variance for each household in each of the two specifications. Table 3 shows that between these two specifications, vulnerability levels change only marginally, whereby the average probability for a household to fall below the food insecurity threshold is about 36 percent.

Table 3: Probability of falling into a state of food insecurity in Nicaragua

	**Without agriculture variables**		**Including agriculture variables**	
	Average	Standard deviation	Average	Standard deviation
**Vulnerability (%):**	36.4	27.7	35.9	28.7

5.1. How many are vulnerable and how vulnerable are they?

Vulnerability estimates, described and analyzed in more detail in the following sections, show that one third of the sampled rural population is in a transitory condition, falling in and out of food insecurity, while two thirds are found to be in a stable condition, being either food secure or food insecure. Food security-oriented policies based on a static analysis of food security (emphasizing *current* vulnerability) may not capture the imminent needs of a large share of the population, while targeting households whose needs are of a temporary nature only. The results also show a positive relationship between vulnerability to food insecurity and households' dependence on farming activities. Asset ownership only seems to provide these households with limited insurance against the risks involved in farming activities, suggesting that additional risk management strategies are needed.

Confirming the empirical evidence that motivated this paper, our estimates suggest that there is no biunivocal correspondence between undernourished households and vulnerable ones. The two groups overlap but are not identical. Consequently, policy measures based on static food security analysis would include errors of exclusion and of inclusion; resources would be directed to undernourished households, a large proportion of which are unlikely to remain food

[5] Inclusion of land owned and land operated variables together, raises collinearity concerns. Testing for the issue does not indicate a serious collinearity problem as the tolerance factor is 0.46 (tolerance smaller than 0.1 implies a valid collinearity threat). Deininger et al, 2003, in their farm profit function, using Nicaraguan survey data, also include both variables.

insecure even without assistance, while those households currently sufficiently well-nourished are vulnerable to future food insecurity.

As table 4 shows, only 44.3% of households enjoy *stable* levels of *food security* in our sample; that is they are food secure and not vulnerable. On the other hand, 20.3% of the population is undernourished (*food insecure*) while also being vulnerable; these are considered *chronically food insecure*. 29.2% of households are currently undernourished but only temporarily (*transient food insecure*). Most importantly, about 6% of households in our sample are food secure at present, while being at risk of being undernourished *(food insecure)* in the future. Therefore, in the case of Nicaragua a targeting error could potentially affect more than one third of the population (29.2%+6.2%=35.4%). Overall, in Nicaragua 26.5% of households are vulnerable to food insecurity, exhibiting an average vulnerability of 77%.

Table 4: Current food security status and vulnerability to future food insecurity (population shares and, in brackets, average probability of a household's future food insecurity status, v_h)

**Current status:**	**"vulnerable" (food insecure in future)** ($v_h > 0.5$)		**"non-vulnerable" (food secure in future)** ($v_h < 0.5$)		Total	
**Food insecure**	20.3%	[.79]	29.2%	[.26]	49.5%	[.48]
**Food secure**	6.2%	[.70]	44.3%	[.18]	50.5%	[.24]
Total	26.5%	[.77]	73.5%	[.21]	100%	[.36]

Figure 2: Nicaragua: Proportions of food insecure and vulnerable population are uneven across departments

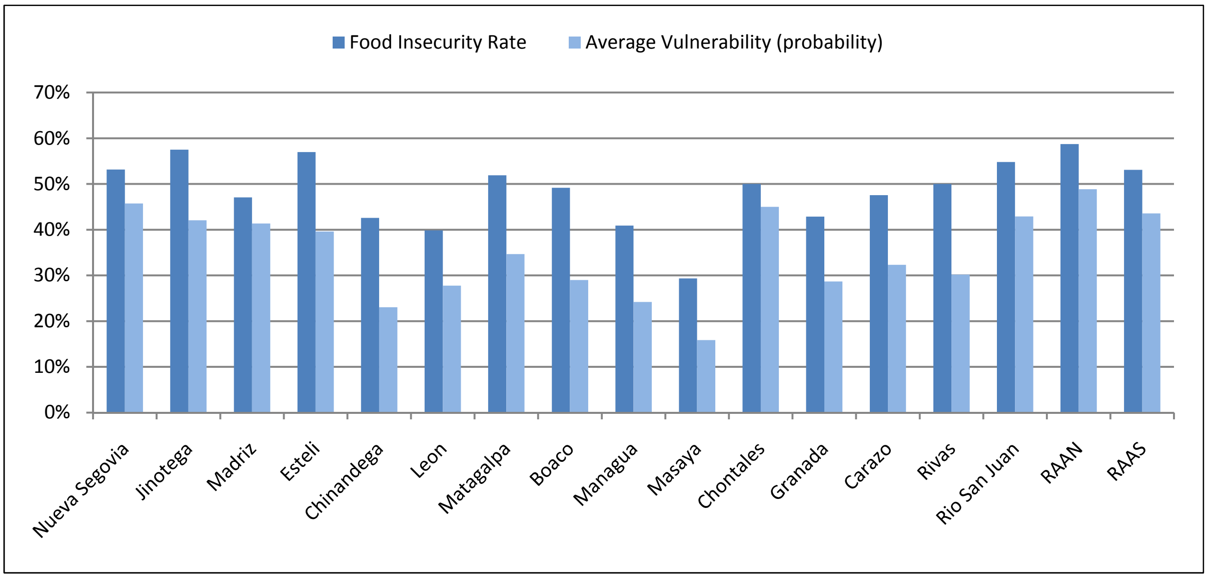

In figure 2, we see the average probability of becoming food insecure as well as the food insecurity headcount, by department. The RAAN department performs worst as a typical household in the region has nearly a 50% probability to become food deprived in the near future. On the other hand, average vulnerability in the region of Chinandega is 23%, the second lowest in the rural Nicaragua (average probability in Masaya is 16%). In general, the food

poverty headcount in each department is always higher than average vulnerability to fall into food insecurity; this outcome indicates probably the incidence of a transitory shock which has an impact on food insecurity now, but is not expected to last.

5.2. What are the characteristics of the most vulnerable households?

In order to identify vulnerable households we have examined the way vulnerability associates with other variables in our model across different households. In Table 5, we have tabulated the values that some household characteristics exhibit on average in five different classes of vulnerability. A few patterns emerge:

1. Higher levels of vulnerability are associated with lower current calorie consumption. This means that on average, across the vulnerability classes we have selected, vulnerability and food consumption generally move in the same direction. However, as we have seen, this average co-movement is loose enough for a substantial difference to arise between vulnerable – and currently food insecure households;
2. Demographic variables (household size, education and age of household head) have a monotonic relationship with vulnerability;
3. Livestock assets have a monotonic relationship with vulnerability. In all cases they are smaller for the least vulnerable class than for the most vulnerable one;
4. Larger land operation is associated with higher levels of vulnerability; this relationship is weaker when land ownership is considered;
5. Higher levels of vulnerability are associated with a higher share of income derived from on-farm activities.
6. Higher levels of vulnerability are associated with a higher share of agricultural produce sold on the market.

Results under points 3 and 4 above are puzzling, since asset ownership is usually thought of as a mitigating factor, decreasing the risk of food insecurity. However, point 5 suggests that the correlation between asset holdings and vulnerability may be spurious. Recalling that not all rural households rely entirely on farming, it is plausible that those who are more dependent on farming are also more vulnerable to food insecurity, especially where agricultural production is erratic. It is also plausible that, due to their dependence on agriculture, these households have higher ownership of land and livestock. The interpretation of results 3 and 4, therefore, requires great caution. They cannot be interpreted as correlations unless they are observed while keeping all other variables, including the share of on-farm income, constant. Similarly puzzling results are not uncommon (see, for example, Deininger *et al*, 2003, who find negative correlation between profit and land operation/ownership).

Table 5 analyzes the way some characters change *across* classes of vulnerability. We have distinguished seven different classes of vulnerability, two below and five above the 50% vulnerability threshold. For each class of vulnerability we have indicated the average levels of the variables listed in the first column. Hence, for example, the average features of the tenth decile of the vulnerability distribution are found in the rightmost column. Households belonging to this class exhibit low per capita calorie consumption, relatively high age of the head of household, low access to infrastructure and a large share of income derived from on-farm activities. At the other end of the chart, we see that least vulnerable households are characterized by higher per capita calorie consumption; a younger head of household; better access to infrastructure and lower dependence on on-farm activities.

In Table 5a results by different classes of vulnerability are presented. As can be seen, the non-vulnerable food secure households (second column) are younger, better educated and more often headed by a male than households considered transitory food secure. In line with finding

4 above, transitory food security (first column) is associated with larger land holdings and a higher reliance on on-farm activities than those in a stable state of food security (second column).

Table 5: Levels of vulnerability to food insecurity – relationships with selected variables

	vulnerability to food insecurity						
	**0-20%**	**20.1-50%**	**50.1-60%**	**60.1-70%**	**70.1-80%**	**80.1-90%**	**90.1-100%**
**Kilocalories**	2048.75	2085.02	1934.14	1846.58	1690.41	1780.33	1618.36
**Kcal. Requirement**	1577.92	1781.89	1869.20	1846.51	1832.33	1829.07	1822.60
**Food share**	0.50	0.48	0.51	0.49	0.52	0.49	0.60
**Farm income**	0.30	0.36	0.53	0.53	0.62	0.71	0.81
**Farm sales**	0.10	0.10	0.14	0.15	0.19	0.19	0.22
**Household size**	5.43	5.25	5.60	6.71	6.95	7.66	8.61
**Age**	39.41	49.48	49.80	49.80	50.09	51.84	52.86
**Education**	5.28	5.70	4.56	4.51	4.49	4.64	3.86
**Single head**	0.38	0.41	0.39	0.36	0.49	0.45	0.49
**Female headed**	0.16	0.18	0.17	0.22	0.25	0.22	0.23
**Widow head**	0.06	0.06	0.06	0.08	0.09	0.12	0.14
**Female labor**	1.28	1.31	1.28	1.57	1.60	1.97	1.97
**Indigenous**	0.05	0.02	0.02	0.03	0.08	0.05	0.11
**Land, operated**	3.02	3.80	6.25	5.56	7.05	9.41	11.06
**Land, owned**	3.61	6.90	10.66	7.89	9.87	13.85	16.20
**Cattle**	1.65	4.65	5.83	3.66	5.60	4.95	4.16
**Pigs**	0.76	1.16	2.06	1.73	2.43	2.40	2.43
**Horse**	0.15	0.36	0.38	0.50	0.35	0.37	0.49
**Rooms**	1.97	1.91	2.02	1.84	2.03	2.00	1.89
**Cement floor**	0.17	0.17	0.07	0.08	0.03	0.11	0.05
**Home phone**	0.02	0.01	0.00	0.00	0.00	0.00	0.00
**Motorcycle**	0.01	0.00	0.00	0.00	0.00	0.00	0.00
**Bikes**	0.34	0.35	0.17	0.26	0.14	0.25	0.11
**Radios**	0.48	0.55	0.54	0.67	0.82	0.85	0.76
**TV**	0.22	0.22	0.15	0.13	0.08	0.12	0.03
**Access to Irrig.**	0.01	0.01	0.00	0.00	0.00	0.01	0.01
**Comm. Org.**	0.31	0.43	0.45	0.57	0.37	0.45	0.35
**Migr. Network**	0.09	0.02	0.04	0.05	0.02	0.03	0.05
**Safe water**	0.49	0.62	0.55	0.48	0.54	0.51	0.32
**Dist. to school**	1.76	1.47	1.80	1.69	1.43	2.05	2.28
**Time to medic.**	13.08	15.11	13.11	16.76	13.65	12.27	10.84
**Dist. to road**	45.29	29.99	37.96	46.59	81.71	59.99	113.72
**Gov't programs**	1.37	1.54	1.13	1.23	0.82	0.99	0.68
**Non-gov't Progr.**	0.30	0.37	0.43	0.37	0.42	0.34	0.33
**Drought**	0.19	0.29	0.33	0.28	0.37	0.15	0.16
**Illness**	0.08	0.10	0.16	0.21	0.18	0.32	0.31

Similar results apply to the food insecure households. Transitory food insecure households (the currently food insecure who are likely to escape this condition as depicted in column four) are younger and better educated than chronically food insecure ones. On the other hand, the difference in terms of gender of the household head is not significant. In this case, confirming again point 4 above, chronic food insecurity is associated with higher land ownership and a higher share of income from on-farm activities, suggesting that farming exposes households to shocks with lasting negative consequences.

Both tables 5 and 5a show a positive association between vulnerability and the share of income coming from the sale of agricultural production on the market. This share typically represents an inverse measure of liquidity constraints, with higher absolute values associated with loser constraints. On the other hand, the unexpected result showed by tables 5 and 5a may suggest that, in the specific context of Nicaragua, the market for agricultural products may be a source of instability for rural households exposing them to economic shocks that we are not considering explicitly here.

In table 6 households are disaggregated according to the gender of the head of household. Female- and male-headed households, representing 18% and 82% of the total respectively, on average exhibit relatively different levels of vulnerability; in female-headed households the average probability to fall below the undernourishment threshold is 4 percentage points higher than for male-headed households (39% relative to 35%). Similarly the share of female-headed households whose probability of food insecurity exceeds 50% is different to that of male-headed households (31% and 25%, respectively).

Table 5a: Presentation of variables by class of vulnerability to food insecurity

	currently food secure		currently food insecure	
	**vulnerable**	**non-vulnerable**	**vulnerable**	**non-vulnerable**
**Calories**	2461.29	2429.29	1551.97	1515.64
**Cal. Requirement**	1862.29	1652.51	1832.69	1716.74
**Food share in cons.**	0.51	0.50	0.54	0.48
**Share of inc. from farm**	0.61	0.31	0.67	0.36
**Share of inc. from farm**	2770.04	1736.64	3735.09	2166.17
**Household size**	6.19	5.30	7.55	5.40
**Age**	50.00	42.21	51.44	47.60
**Education**	3.96	5.61	4.45	5.29
**Single head**	0.35	0.39	0.47	0.39
**Female headed**	0.26	0.16	0.20	0.18
**Widow head**	0.07	0.06	0.11	0.07
**Female labor**	1.40	1.27	1.79	1.32
**Indigenous**	0.03	0.03	0.07	0.03
**Land, op.**	6.28	3.16	8.81	3.77
**Land, owned**	8.86	4.90	13.32	5.71
**Cattle**	4.04	2.77	4.98	3.65
**Pigs**	1.74	0.86	2.36	1.10
**Horse**	0.46	0.21	0.42	0.32
**Rooms**	1.91	1.97	1.96	1.90
**Cement floor**	0.02	0.19	0.08	0.15
**Home phone**	0.00	0.02	0.00	0.01
**Motorcycle**	0.00	0.00	0.00	0.01
**Bikes**	0.16	0.37	0.18	0.30
**Radios**	0.72	0.50	0.72	0.53
**TV**	0.09	0.24	0.10	0.19
**Access to Irrigation**	0.01	0.01	0.01	0.01
**Comm. Org.**	0.50	0.36	0.41	0.38
**Migr. Network**	0.04	0.06	0.04	0.05
**Safe water**	0.49	0.55	0.45	0.56
**Dist. to school**	2.36	1.46	1.79	1.87
**Time to medic.**	10.56	14.24	13.73	13.82
**Dist. to road**	52.81	37.11	78.37	38.82
**Gov't programs**	0.88	1.49	0.96	1.39
**Non-gov't Progr.**	0.31	0.33	0.39	0.33
**Drought**	0.29	0.22	0.23	0.27
**Illness**	0.18	0.08	0.26	0.10

Table 6: Female-headed households are more vulnerable

	**relative group size**	**vulnerable ($v_h > 0.5$)**	**avg. vulnerability prob.**
Female headed HH	18%	31.3%	.39
Male headed HH	82%	25.4%	.35

Table 7: Levels of vulnerability to food insecurity in the three most vulnerable Departments

		Department		
		**Chinandega**	**Jinotega**	**Río San Juan**
**Vulnerability:**	**mean**	.23	.42	.43
	**std err**	.16	.29	.33
**Kilocalories**		2216.77	1868.40	1896.38
**Kcal. Requirement**		1726.05	1709.22	1707.62
**Food share in consumption**		0.51	0.48	0.51
**Share of income from farm**		0.29	0.67	0.56
**Share of inc. from farm sales**		0.09	0.32	0.19
**Household Size**		6.01	5.95	5.28
**Age of head**		49.45	44.64	43.67
**Education**		5.98	3.97	3.89
**Single head**		0.47	0.48	0.39
**Female headed**		0.12	0.16	0.17
**Widow head**		0.06	0.05	0.06
**Female labor**		1.43	1.29	1.31
**Indigenous**		0.00	0.04	0.00
**Land, operated (ha)**		2.91	7.18	5.57
**Land, owned (ha)**		4.08	8.73	6.94
**Cattle**		2.63	2.16	4.13
**Pigs**		1.52	1.27	1.42
**Horse**		0.22	0.39	0.14
**Rooms**		1.61	1.80	2.13
**Cement floor**		0.17	0.14	0.15
**Home phone**		0.02	0.00	0.01
**Motorcycle**		0.02	0.01	0.01
**Bikes**		0.66	0.07	0.03
**Radios**		0.36	0.74	0.76
**TV**		0.36	0.08	0.10
**Access to Irrigation**		0.02	0.02	0.00
**Comm. Org.**		0.37	0.36	0.35
**Migration Network**		0.09	0.04	0.10
**Safe water**		0.57	0.61	0.46
**Dist. to school**		1.11	2.67	4.91
**Time to medic.**		16.26	11.86	14.98
**Dist. to road**		8.99	48.92	99.56
**Gov't programs**		1.65	1.16	0.50
**Non-gov't Progr.**		0.77	0.47	0.54
**Drought**		0.39	0.14	0.13
**Illness**		0.11	0.18	0.18

The analysis of vulnerable households' characteristics can include a number of variables deemed relevant. Table 7 shows how the average values of several household characters change in the three Nicaraguan Departments (out of fifteen), that exhibit the highest vulnerability.

5.3. What has been the impact of shocks on the proportion of vulnerable households?

As a last step, in order to complete the description of our empirical analysis of vulnerability to food insecurity in Nicaragua, we consider the relation of the proportion of vulnerable households to two different shocks (Table 8). Data show that mainly idiosyncratic shocks are highly correlated with vulnerability. Households that have been exposed to a shock have higher levels of vulnerability compared to households that have not, suggesting a difficulty in recovering from these and a need to strengthen risk management capacities. In particular, an illness of a member of the household has the worst outcome in terms of increasing the average probability that a household will be undernourished - households that have experienced illness have a 52 % probability to be food insecure in the near future, which is 18 percentage points higher than households that have not experienced an illness. Drought affected one quarter of the population, taking their vulnerability to the relatively higher levels; the average probability is 37 %.

Table 8: Shares of the population affected by selected shocks (in brackets: average vulnerability probability V_h)

	yes		no	
**drought**	24%	[.37]	76%	[.35]
**illness**	13%	[.52]	87%	[.34]

6. Conclusions

Empirical findings show that in many countries, households repeatedly fall in and out of food insecurity, making it difficult for policy makers to address their needs with well-targeted development programmes, interventions or safety nets. In the literature, several theories on how best to assess possible levels of future food insecurity are discussed; however, none of these have evolved into an accepted baseline model. We contribute to this line of research by adapting from poverty analysis a methodology that allows future levels of food insecurity to be estimated without having to draw on historical (time series) data.

The model allows the relative vulnerability to food insecurity of each typology of households to be estimated in that the probability that any given household's food consumption will fall below a specified threshold in the future can be computed. As a first step, we study the relationship between kilocalorie consumption and a set of key household characteristics, such as demographic information, asset ownership, access to infrastructure, location and exposure to shocks. Subsequently, estimates from the model are used to identify the probability distribution of kilocalorie consumption focusing on the likelihood of household consumption falling below the threshold. Finally, the model allows for an analysis on how vulnerability relates with various household characteristics, in order to profile the most vulnerable households and to draw conclusions on possible causes of vulnerability.

The application shows that more than half of the vulnerable households in Nicaragua are currently food secure, while almost one third of the currently food insecure only face temporary food deprivation. Overall, one third of rural households face an unstable food security situation. Past exposure to shocks that affect agricultural production, and illness in the family, drought,

and market-related shocks increase the probability that households will be food insecure in the future. Education, access to public infrastructure and other assets represent positive factors that reduce overall vulnerability to food insecurity.

From a policy perspective, our results have a number of implications. Policies and food security interventions based on static food security analyses do not capture the imminent needs of a potentially large share of the population that is likely to change its food security status in the near future. These include currently food secure households that may become food insecure in the near future and, on the other hand, households that are likely to overcome a currently food insecure situation without external assistance. Forward-looking analysis of vulnerability to food insecurity allows correcting these potential errors in policy design.

In this paper, we have shown that it is possible to associate a robust vulnerability index to each household. This index can be meaningfully related to other household characteristics in order to identify the characteristics of groups at risk of future food insecurity. In upcoming work, policy simulations will explore options for reducing vulnerability as it stems from different sources such as climate change and imported inflation.

Finally, richer information on risks and risk management capacities would allow a more detailed analysis of future food insecurity. Extensions of the present paper, employ historical data and projections of climate-related information in Nicaragua in order to assess the likely repercussions of expected climate change on household food security.

References

Alinovi, L., E. Mane, D. Romano, 2009. "Measuring Household Resilience to Food Insecurity: Application to Palestinian Households". FAO-ESA Working Paper. FAO, Agricultural and Development Economics Division, Rome.

Barrett, C.B. (2002), "Food Security and Food Assistance Programs," in B.L. Gardner and G.C. Rausser, eds., Handbook of Agricultural Economics (Amsterdam: Elsevier).

Calvo, C., and S. Dercon, 2005. "Measuring Individual Vulnerability", Economics Series Working Papers 229, University of Oxford, Department of Economics.

Chaudhuri, S., 2003. "Assessing Vulnerability to Poverty: Concepts, Empirical Methods, and Illustrative Examples", mimeo, Columbia University.

Chaudhuri, S., J. Jalan, and A. Suryahadi, 2002. "Assessing Household Vulnerability to Poverty from Cross-Sectional Data: A Methodology and Estimates from Indonesia". Discussion Paper Series 0102-52. New York: Department of Economics, Columbia University.

Christiaensen, L. J. and R. N. Boisvert, 2000. "On measuring household food vulnerability: Case evidence from Northern Mali". Working Paper No. 2000-05, Ithaca, NY: Cornell University.

Christiaensen L. J. and K. Subbarao, 2005. "Towards an Understanding of Household Vulnerability in Rural Kenya", Journal of African Economies, vol 14, no 4, pp. 520–558

Deininger, K., E. Zegarra and I. Lavadenz, 2003, "Determinants and Impacts of Rural Land Market Activity: Evidence from Nicaragua", World Development, Vol. 31, no. 8, pp 1385-1404.

Dercon S., 2005. "Risk, Poverty and Vulnerability in Africa," Journal of African Economies, Oxford University Press, vol. 14(4), pages 483-488, December

Dercon, S., 2001. "Assessing vulnerability to poverty". Report prepared for the Department for International Development (DFID). Available at: www.economics.ox.ac.uk/members/stefan.dercon/assessingvulnerability. Empirical Methods, and Illustrative Examples", mimeo, Columbia University.

Fishburn, P.C., 1977. "Mean-Risk Analysis with Risk Associated with Below-Target Returns". The American Economic Review, Vol.67, No 2 (March), pp.116-126.

Holzmann, R., and S. Jørgensen (2000). 'Social Risk Management: a New Conceptual Framework for Social Protection and Beyond'. Social Protection Discussion Paper No. 0006. Washington, DC: World Bank.

Kamanou, G., and J. Morduch, 2002. "Measuring Vulnerability to Poverty". WIDER Discussion Paper 2002/58. Helsinki: UNU-WIDER.

Ligon E. and L. Schechter, 2004. "Evaluating Different Approaches to Estimating Vulnerability", Social Protection Discussion Paper Series, World Bank.

Lipton, M. and M. Ravallion, 1995. "Poverty and policy", Handbook of Development Economics, in: Chenery H. andT.N. Srinivasan (ed.), Handbook of Development Economics, edition 1, volume 3, chapter 41, pages 2551-2657 Elsevier.

Løvendal C.R and M. Knowles, 2005. "Tomorrow's hunger: a framework for analysing vulnerability to food insecurity". FAO-ESA Working Paper No. 05-07. FAO, Agricultural and Development Economics Division, Rome.

Lybbert T, C. B. Barrett, S. Desta, and D. L. Coppock, "Stochastic Wealth Dynamics and Risk Management Among A Poor Population," Economic Journal, vol. 114, no. 498 (October 2004): pp. 750- 7.

Ravallion, M., 1988. "Expected Poverty under Risk-Induced Welfare Variability," Economic Journal, Royal Economic Society, vol. 98(393), pages 1171-82, December.

Scaramozzino, P., 2006. “Measuring vulnerability to food insecurity” FAO-ESA Working Paper No. 06-12. FAO, Agricultural and Development Economics Division, Rome.

Seaman J., P. Clarke, T. Boudreau, J. Holt, 2000. “The Household Economy Approach”. Save the Children, London UK.

Sen, Amartya, 1982. “Poverty and Famines: An Essay on Entitlements and Deprivation”, Oxford, Clarendon Press.

WFP & FAO. 2007. “Comprehensive Food Security and Vulnerability Analysis (CFSVA). West Bank and Gaza Strip”.

World Bank (2000). World Development Report 2000/01: Attacking Poverty. Washington, DC: World Bank.

ESA Working Papers

WORKING PAPERS

The ESA Working Papers are produced by the Agriculture and Economic Development Analysis Division (ESA) of the Economic and Social Department of the United Nations Food and Agriculture Organization (FAO). The series presents ESA's ongoing research. Working papers are circulated to stimulate discussion and comments. They are made available to the public through the Division's website. The analysis and conclusions are those of the authors and do not indicate concurrence by FAO.

AGRICULTURAL DEVELOPMENT ECONOMICS

Agricultural Development Economics (ESA) is FAO's focal point for economic research and policy analysis on issues relating to world food security and sustainable development. ESA contributes to the generation of knowledge and evolution of scientific thought on hunger and poverty alleviation through its economic studies publications which include this working paper series as well as periodic and occasional publications.

Agricultural Development Economics (ESA)
The Food and Agriculture Organization
Viale delle Terme di Caracalla
00153 Rome, Italy

Contact:
Office of the Director
Telephone: +39 06 57054368
Facsimile: + 39 06 57055522
Website: www.fao.org/economic/esa
e-mail: ESA@fao.org
