# Supplementary material for: Systematic Review of Methods in Low-Consensus Fields: Supporting Commensuration through `Construct-Centered Methods Aggregation’ in the Case of Climate Change Vulnerability Research
Source: PLoS One. 2016 Feb 22;11(2):e0149071. doi: 10.1371/journal.pone.0149071 (PMC4762661; doi:10.1371/journal.pone.0149071)
Supplement: S2 File — Source articles and bibliography. (ZIP) [file pone.0149071.s002.zip › data requirements/CARE.pdf]

Climate Vulnerability and Capacity Analysis

Handbook

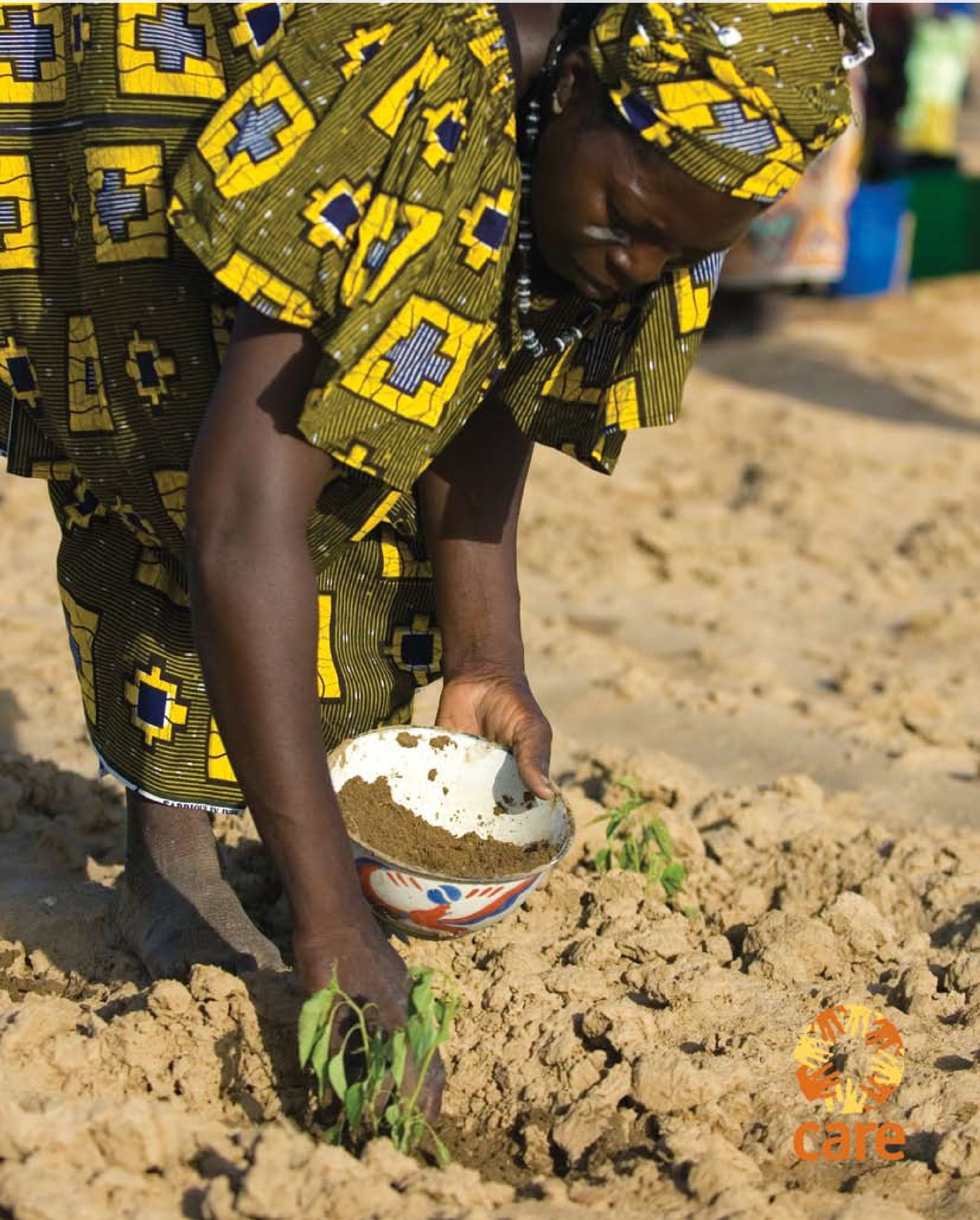

care

Climate Vulnerability and Capacity Analysis

Handbook

care®

Foreword

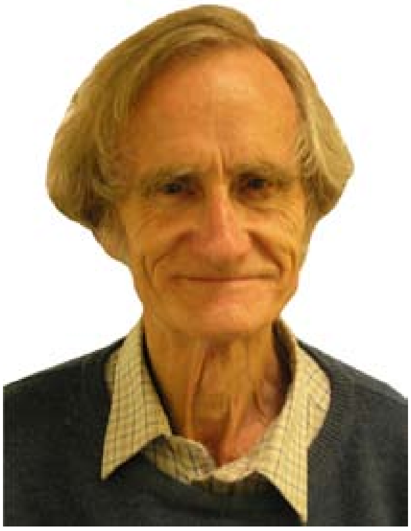

Prof. Robert Chambers
Research Associate
Institute of Development Studies
University of Sussex, UK

Climate change has already impacted on innumerable communities, exposing them to increasing hazards and making them more vulnerable; and we can expect this to become more marked, and for some communities catastrophic, in coming years. In order to plan effective adaptation actions, scientific climate change analysis is vital for broad context. However, at the local level, the most relevant information and knowledge often already exists or can be generated through local stakeholders' own analysis. Local knowledge also has a credible authority for informing and influencing policy.

So this Handbook, which presents a new participatory methodology for Climate Vulnerability and Capacity Analysis, is very timely. Its focus on the community level is sharp and salutary. It stresses that communities are not homogeneous. It underlines the need to pay special attention to those, especially women and the marginalised, who are more at risk and less able to adapt. It is about facilitating analysis of vulnerability and adaptive capacity by members of communities themselves. In doing this it applies participatory values, processes and methods, to enable local people to articulate and enhance their own knowledge and understanding, and to plan action. It is based on the premise, so often validated, that 'they can do it' - that local people have knowledge and capabilities greater than outsider professionals often believe.

This Handbook is clear and practical. It is to the point in stressing the importance of empowerment and of sensitive facilitation: for this, the behaviour and attitudes of facilitators are key, as is their ability to 'hand over the stick'. As a guide and source of ideas for field workers, it should be invaluable. It has been designed as a living document. If widely used, and updated and further developed through experience, it will contribute more and more. May its good effects multiply. May it be applied so that very many of those so easily overlooked, whose lives, livelihoods and whole communities are threatened by climate change, will be better able to adapt to the challenges they face.

Acknowledgements

Prepared by Angie Dazé, Kaia Ambrose and Charles Ehrhart

©Copyright CARE

1st Edition

May 2009

Cover photo by Valenda Campbell/CARE

CARE grants permission to all not-for-profit organizations to reproduce this work, in whole or in part. The following notice shall appear conspicuously with any reproduction: "Climate Vulnerability and Capacity Analysis Handbook" © 2009 by CARE International. Used by permission.

The Handbook can be downloaded from CARE's climate change website at **http://www.careclimatechange.org**. The CVCA Handbook is a living document. Please send feedback and suggestions to **cvca@careclimatechange.org**. We would be particularly pleased to hear about your experiences using the Handbook and suggestions for its improvement.

The authors would like to thank the following CARE colleagues for providing helpful feedback and suggestions to make this Handbook more relevant to development actors: Cynthia Awuor, Shafiqul Islam, Amilcar Lucas, Marcos Athias-Neto, Richard Paterson, Morten Fauerby Thomsen, and Nguyen Thi Yen. We are grateful to Sam Boardley (CHF – Partners in Rural Development), Gina Castillo (Oxfam America), Anne Hammill (International Institute for Sustainable Development), Mark Janz (World Vision International), Mary Morris (World Vision International), Kimberly Rafuse (Canadian Red Cross), and Tom Tanner (Institute of Development Studies) for useful suggestions.

During the field tests, invaluable contributions were made by CARE staff, partner organizations, and community members in Ghana, Niger, and Nepal. These contributors are too numerous to mention by name, but we are hopeful that this final product reflects their views and meets their expectations.

Table of Contents

Introduction **1**
- About the Climate Vulnerability and Capacity Analysis (CVCA) Methodology 2
- What's new and different about the CVCA? 2
- Who is this Handbook for? 3
- How do I use the CVCA Handbook? 4
- What the CVCA will NOT do 4

Key Concepts **5**
- Climate Change 5
- Vulnerability to Climate Change 5
- Resilience 6
- Adaptation to Climate Change 7

The CVCA Process **9**
- A Framework for Community-Based Adaptation 9
- CVCAs: Where and When? 11
- Determining the Scope and Depth of the Analysis 11
- Assembling the Team 12
- Balancing Research with Learning 12

Doing the Analysis **13**
- National Level 13
- Local Government/Community Level 16
- Household/Individual Level 18
- Compiling and Analyzing the Data 20
- Validating the Analysis 20
- Documenting and Disseminating the Analysis 21

Using the Analysis **23**
- Adaptation Advocacy 23
- Integrating Adaptation into Development Projects 23
- Community-Based Adaptation Projects 24
- Further Research 28

Field Guides for Participatory Tools **29**
- Field Guide 1: Facilitation Tips 30
- Field Guide 2: Hazard Mapping 33
- Field Guide 3: Seasonal Calendar 35
- Field Guide 4: Historical Timeline 37
- Field Guide 5: Vulnerability Matrix 39
- Field Guide 6: Venn Diagram 41

List of Acronyms

CBA	Community-Based Adaptation
CBO	Community-Based Organization
CIDA	Canadian International Development Agency
CVCA	Climate Vulnerability and Capacity Analysis
EWS	Early Warning System
FAO	Food and Agriculture Organization of the United Nations
FEWS Net	Famine Early Warning System Network
FG	Focus Group
HIV&AIDS	Human Immunodeficiency Virus & Acquired Immunodeficiency Syndrome
IISD	International Institute for Sustainable Development
IUCN	International Union for the Conservation of Nature
IPCC	Intergovernmental Panel on Climate Change
M&E	Monitoring and Evaluation
NAP	National Action Programme to Combat Drought and Desertification
NAPA	National Adaptation Programme of Action
NGO	Non-Governmental Organization
PLA	Participatory Learning for Action
RBA	Rights-Based Approaches
SEI	Stockholm Environment Institute
UN	United Nations
UNCCD	United Nations Convention to Combat Desertification
UNDP	United Nations Development Program
UNFCCC	United Nations Framework Convention on Climate Change
UNISDR	United Nations International Strategy for Disaster Reduction
VCA	Vulnerability and Capacity Assessment
WRI	World Resources Institute

Introduction

Climate change presents additional obstacles to ending poverty and achieving social justice. Rising temperatures, increasingly erratic rainfall, and more frequent and severe floods, cyclones and droughts all have significant consequences for the livelihood security of poor people; and development professionals are seeing first-hand the effects of a changing climate on their work around the world.

In order to ensure that development programs reduce people's vulnerability to climate change, we must understand who is vulnerable to its effects and why. Then, we must apply this information to the design, implementation, monitoring and evaluation of activities.

CARE's approach to climate change adaptation is grounded in the knowledge that people must be empowered to transform and secure their rights and livelihoods. It also recognizes the critical role that local and national institutions, as well as public policies, play in shaping people's adaptive capacity.

The Climate Vulnerability and Capacity Analysis (CVCA) methodology helps us to understand the implications of climate change for the lives and livelihoods of the people we serve. By combining local knowledge with scientific data, the process builds people's understanding about climate risks and adaptation strategies. It provides a framework for dialogue within communities, as well as between communities and other stakeholders. The results provide a solid foundation for the identification of practical strategies to facilitate community-based adaptation to climate change.

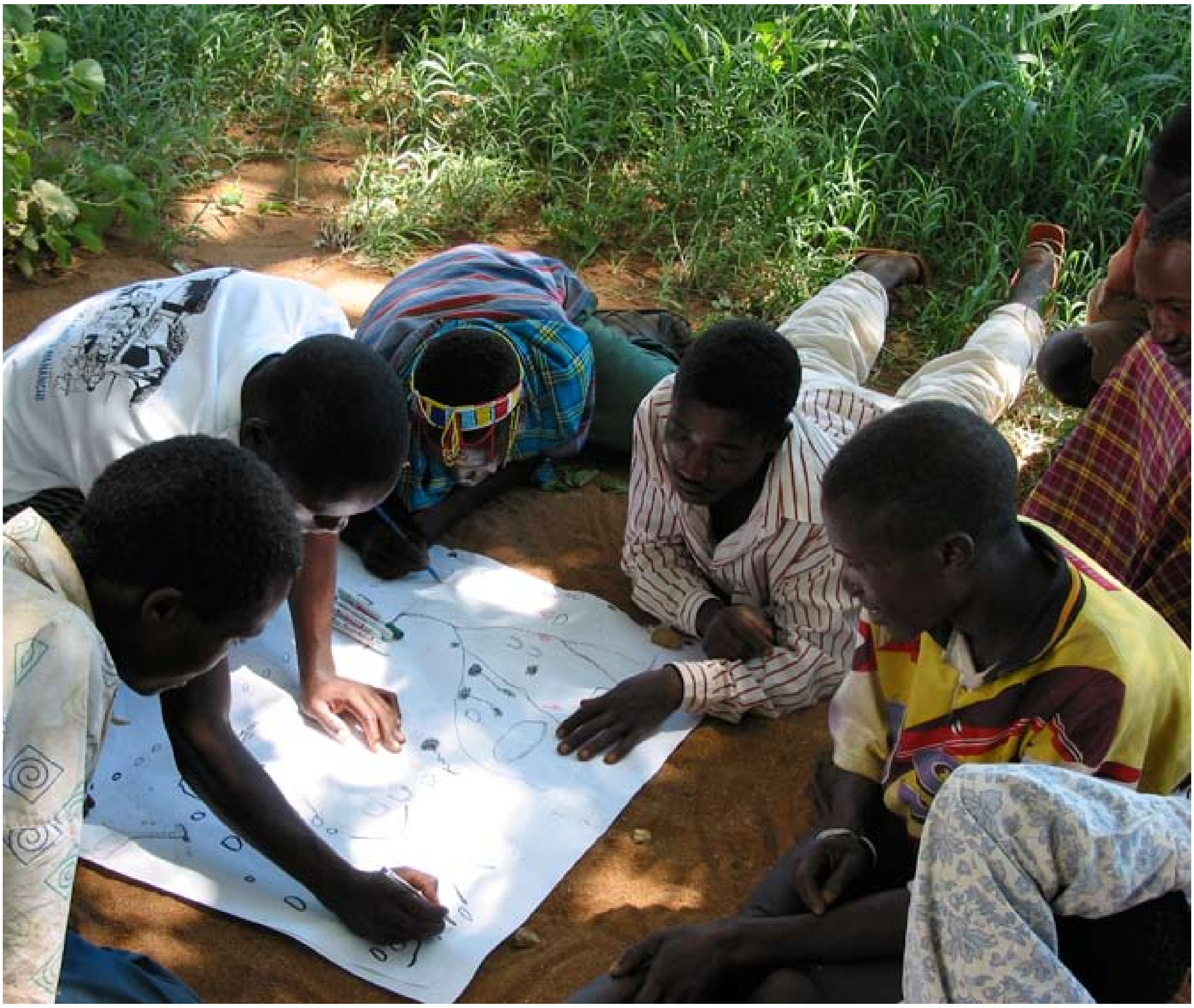

About the Climate Vulnerability and Capacity Analysis (CVCA) Methodology

The CVCA methodology provides a framework for analyzing vulnerability and capacity to adapt to climate change at the community level. Recognizing that local actors must drive their own future, the CVCA prioritises local knowledge on climate risks and adaptation strategies in the data gathering and analysis process.

The main objectives of the CVCA are to:

- ***Analyze vulnerability to climate change and adaptive capacity at the community level:*** The CVCA is a methodology for gathering, organizing and analyzing information on the vulnerability and adaptive capacity of communities, households and individuals. It provides guidance and tools for participatory research, analysis and learning. It also takes into account the role of local and national institutions and policies in facilitating adaptation.

- ***Combine community knowledge and scientific data to yield greater understanding about local impacts of climate change:*** One of the challenges of working at the local level on climate change adaptation is the lack of scaled-down information on impacts. This is coupled with inadequate data and information on weather and climate predictions. The process of gathering and analyzing information with communities serves to build local knowledge on climate issues and appropriate strategies to adapt. The participatory exercises and associated discussions provide opportunities to link community knowledge to available scientific information on climate change. This will help local stakeholders to understand the implications of climate change for their livelihoods, so that they are better able to analyze risks and plan for adaptation.

The CVCA methodology is based on a framework of "enabling factors" for Community-Based Adaptation (CBA). The Handbook presents a set of guiding questions for analysis of information at national, local and household/individual levels. It provides guidance on facilitating a participatory process for multi-stakeholder analysis and collaborative learning. The CVCA is designed to feed into and strengthen planning processes by providing vital, context-specific information about the impacts of climate change and local vulnerability. The process of gathering, analyzing and validating this information promotes invaluable dialogue within communities, and between communities and other stakeholders.

The CVCA methodology may be used and adapted to gather and analyze information to design climate change adaptation initiatives, as well as to integrate climate change adaptation issues into livelihoods and natural resource management programs. It can also provide practical evidence for advocacy on climate change issues. This Handbook provides an overview of the methodology, as well as practical guidance for using it in the design and implementation of adaptation actions.

What's new and different about the CVCA?

There are a number of characteristics which make the CVCA process different from other forms of participatory learning and analysis. These include:

- **Focus on climate change:** The CVCA focuses on understanding how climate change will affect the lives and livelihoods of target populations. It examines hazards, vulnerability to climate change and adaptive capacity with a view to building resilience for the future. The types of tools suggested are tried-and-true Participatory Learning for Action (PLA) tools, but with a climate "lens". The tools are used to draw out issues, which are then examined in the context of climate change through guided discussion.

- **Analyzing conditions and hazards:** The CVCA attempts to combine good practices from analyses done for development initiatives, which tend to focus on conditions of poverty and vulnerability[1], and those done within the context of disaster risk reduction (DRR), which tend to focus on hazards[2]. The framework of the CVCA facilitates analysis of the information gained from both types of assessments from a climate change perspective. It examines both hazards and conditions, and analyzes the interactions between the two.

- **Emphasis on multi-stakeholder analysis, collaborative learning and dialogue:** While the primary purpose of the CVCA is to analyze information, the methodology is designed to balance the research agenda with a process of learning and dialogue among local stakeholders. This can yield a greater understanding within communities of the resources available to them to support adaptation, and can promote dialogue among stakeholders on adaptation actions that make sense.

1 For example, CARE's Household Livelihood Security (HLS) Assessment approach.
2 A notable example is the International Federation of Red Cross and Red Crescent Societies (IFRC) Vulnerability and Capacity Assessment (VCA) Methodology. An introduction can be found at: http://www.ifrc.org/Docs/pubs/disasters/resources/preparing-disasters/vca/whats-vca-en.pdf

- **Focus on communities but also examines enabling environment:** Vulnerability to climate change can vary within countries, communities and even households. Therefore, adaptation requires context-specific activities, with strategies targeted to meet the needs of different vulnerable groups. At the same time, local and national policies and institutions play a critical role in shaping people's capacity to adapt to climate change. Thus, the CVCA process focuses on the community level but incorporates analysis of issues at regional and national level in an effort to foster an enabling environment for community-based adaptation.

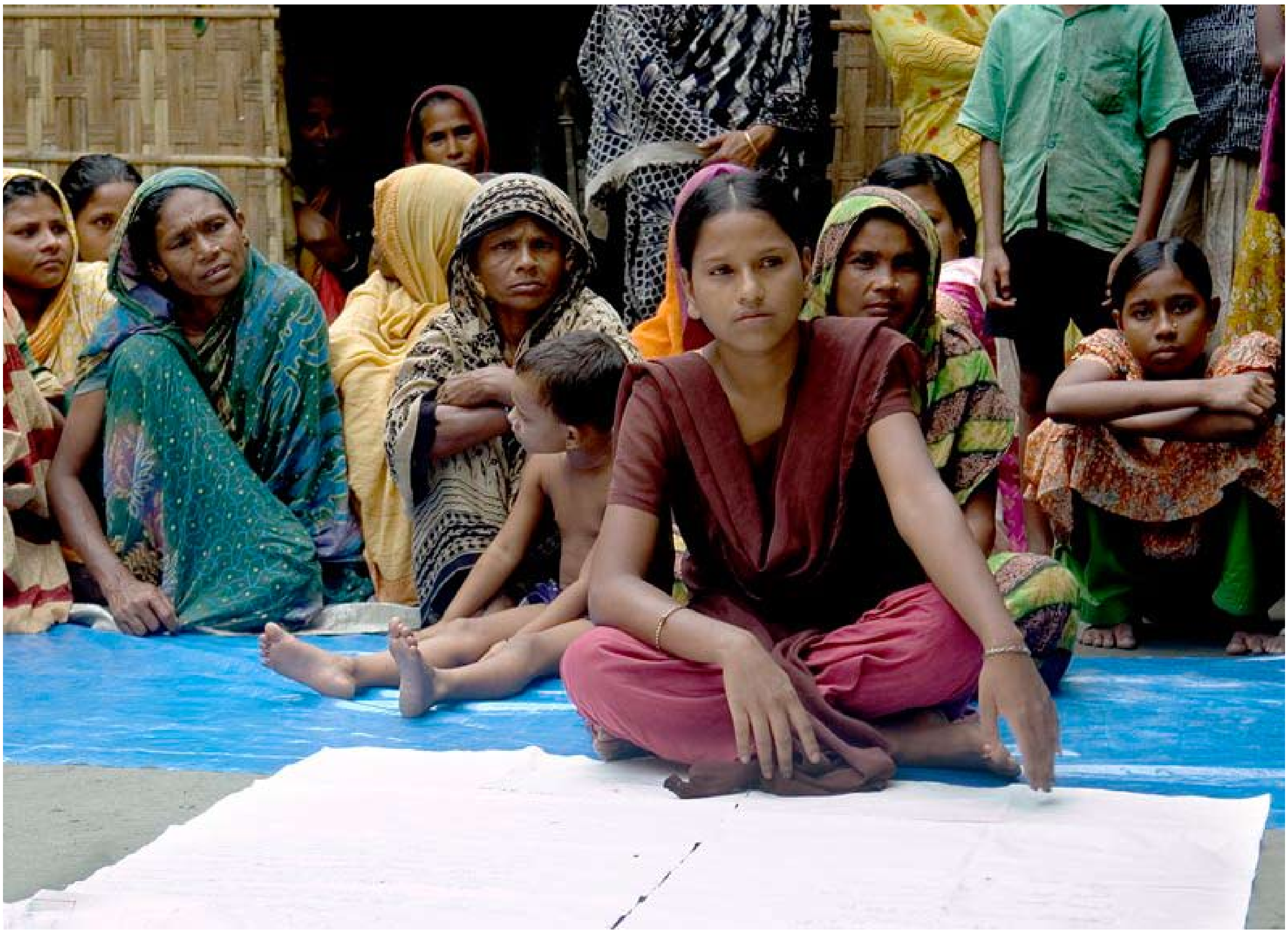

©CARE/Rick Perera

Who is this Handbook for?

The CVCA Handbook is designed for use by:

- **Project managers and field staff:** Staff that are engaged in livelihoods projects or community-based adaptation projects will find useful guidelines on how to collect and analyze information from community, government and scientific perspectives. This information can be used to design locally appropriate climate change adaptation activities.

- **Local partners (government and NGOs):** The tools and process are designed to be used and replicated by a wide range of stakeholders. Local governmental and non-governmental organizations can use the CVCA to assist in integrating vulnerability and adaptation issues into planning and programs.

- **Communities:** Communities can use the tools in this Handbook to support their own process of investigation and learning. The results can be used to plan collective action on adaptation, or to lobby local government or NGOs for appropriate interventions to support them in adapting to the changing climate.

This Handbook is intended as a starting point. We hope that practitioners will experiment with the CVCA methodology by adapting it to their circumstances and enriching it with their experiences. Your feedback will help us evolve the methodology and will form the basis for future updates.

How do I use the CVCA Handbook?

The CVCA Handbook is designed to stimulate analysis and dialogue about climate change and human vulnerability. It uses guiding questions to examine factors at multiple levels using a variety of tools to gather information. It is designed to be flexible so that the learning process can be adapted to suit the needs of particular users.

The Handbook begins with a basic overview of key concepts and CARE's approach to community-based adaptation. It then describes how to plan for and conduct a CVCA. This is followed by a detailed outline of the analytical framework, with suggestions for tools that can be used at different levels to answer the guiding questions. Finally, the Handbook provides suggestions for applying the results, using examples and short case studies. Detailed guidance on using participatory tools in a CVCA analysis is provided in the Field Guides at the end of the Handbook. In an effort to keep the Handbook concise and focused, it is designed to be used in conjunction with other resources, tools, and analytical frameworks. Links to complementary resources are provided throughout the Handbook.

What the CVCA will NOT do.....

The CVCA Handbook is not meant to guide the entire process of developing a project or designing an advocacy campaign. Rather, it is intended to guide the analysis which is generally the first step in either of these undertakings, and to provide suggestions for how this analysis can be used to take action on adaptation to climate change. It is also important to note that the CVCA methodology is not designed to quantify vulnerability or provide results that can be generalised to regional or national levels. However, qualitative information from the CVCA can be used to design quantitative surveys, if desired.

Key Concepts

The CVCA methodology builds on the following interpretations of climate change, vulnerability to climate change, adaptive capacity, resilience, hazard and adaptation:

Climate Change

The Intergovernmental Panel on Climate Change (IPCC)[3] defines climate change as:

Any change in climate over time, whether due to natural variability or as a result of human activity.[4]

CARE uses this definition because it encompasses both natural variability and anthropogenic changes.

When we discuss climate change in this Handbook, we are referring to the observed and projected increase in average global temperature, and the associated impacts, including: an increase in extreme weather events; melting of icebergs, glaciers and permafrost; sea level rise; and changes in the timing and amount of rainfall. From the perspective of reducing vulnerability, it is unnecessary to separate "climate change" caused by humans from natural "climate variability".

Vulnerability to Climate Change

Vulnerability to climate change has been defined as:

The degree to which a system is susceptible to, or unable to cope with, adverse effects of climate change, including climate variability and extremes. Vulnerability is a function of the character, magnitude, and rate of climate variation to which a system is exposed, its sensitivity, and its adaptive capacity.[5]

In the context of the CVCA, the systems we are referring to are communities (recognizing that communities are not homogeneous, so particular households or individuals within communities may have differing degrees of vulnerability).

Exposure to climate variation is primarily a function of geography. For example, coastal communities will have higher exposure to sea level rise and cyclones, while communities in semi-arid areas may be most exposed to drought.

Sensitivity is the degree to which the community is affected by climatic stresses. A community dependent on rain-fed agriculture is much more sensitive than one where the main livelihood strategy is labour in a mining facility, for instance.

Adaptive Capacity

Adaptive capacity is defined as:

The ability of a system to adjust to climate change (including climate variability and extremes) to moderate potential damages, to take advantage of opportunities, or to cope with the consequences.[6]

One of the most important factors shaping the adaptive capacity of individuals, households and communities is their access to and control over natural, human, social, physical, and financial resources. Examples of resources that may be important to adaptive capacity would be:

**Human**	Knowledge of climate risks, conservation agriculture skills, good health to enable labour
**Social**	Women's savings and loans groups, farmer-based organizations
**Physical**	Irrigation infrastructure, seed and grain storage facilities
**Natural**	Reliable water source, productive land
**Financial**	Micro-insurance, diversified income sources

3 The Intergovernmental Panel on Climate Change (IPCC) is a body set up to provide scientific, technical and socio-economic information in a policy-relevant but policy neutral way to decision makers.
4 IPCC, 2007. Climate Change 2007: Impacts, Adaptation and Vulnerability. Contribution of Working Group II to the Fourth Assessment Report of the Intergovernmental Panel on Climate Change, Annex I., M.L. Parry, O.F. Canziani, J.P. Palutikof, P.J. van der Linden and C.E. Hanson, Eds., Cambridge University Press, Cambridge, UK, 976pp.
5 Intergovernmental Panel on Climate Change (IPCC) Working Group 2, 2001. Third Assessment Report, Annex B: Glossary of Terms.
6 Intergovernmental Panel on Climate Change (IPCC) Working Group 2, 2001. Third Assessment Report, Annex B: Glossary of Terms.

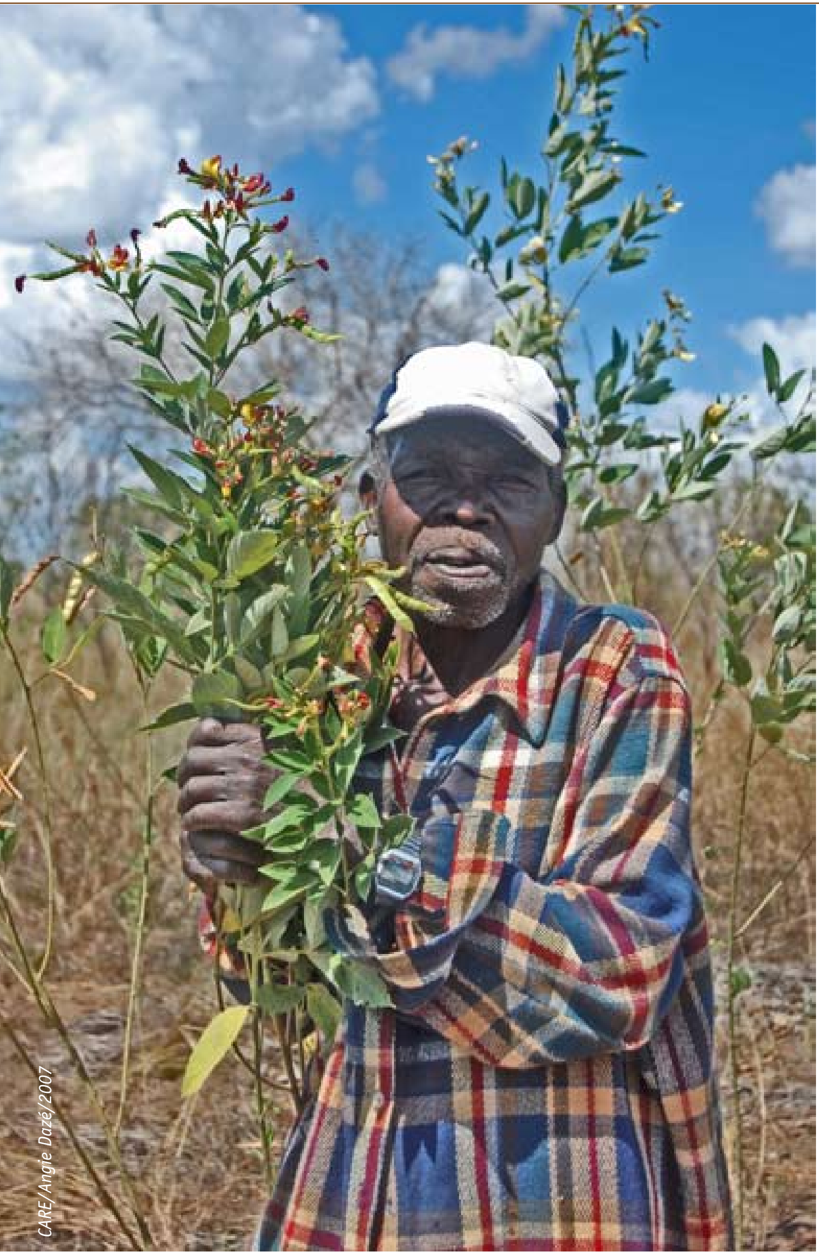

A man in Nampula, Mozambique shows the fruits of his labour in using conservation agriculture techniques to conserve soil moisture and fertility.

Access to and control over the resources necessary for adaptation varies within countries, communities and even households. It is influenced by external factors such as policies, institutions and power structures.[7] Adaptive capacity can vary over time based on changing conditions, and may differ in relation to particular hazards.

In general, the world's poorest people are also the most vulnerable to climate change. This is often because they have limited access to those resources that would facilitate adaptation. For instance, women are often particularly vulnerable to the impacts of climate change due to their responsibilities in the home and their limited access to information, resources and services. Other groups such as pastoralists, persons living with HIV&AIDS and the elderly may also represent highly vulnerable populations. The CVCA facilitates identification of vulnerable groups and targeting of adaptation strategies depending on the context.

Resilience

Resilience can be defined as:

> *The ability of a community to resist, absorb, and recover from the effects of hazards in a timely and efficient manner, preserving or restoring its essential basic structures, functions and identity.*[8]

Resilience is a familiar concept in the context of disaster risk reduction (DRR), and is increasingly being discussed in the realm of adaptation. A resilient community is well-placed to manage hazards to minimize their effects and/or to recover quickly from any negative impacts, resulting in a similar or improved state as compared to before the hazard occurred. There are strong linkages between resilience and adaptive capacity; consequently, resilience also varies greatly for different groups within a community.

Hazard

In the context of disaster risk reduction, a hazard is defined as:

> *A dangerous phenomenon, substance, human activity or condition that may cause loss of life, injury or other health impacts, property damage, loss of livelihoods and services, social and economic disruption, or environmental damage.*[9]

When we discuss hazards in the context of the CVCA, we are referring both to shocks, such as droughts or floods (rapid onset), and to stresses, such as changing rainfall patterns (slow onset).

It is important to distinguish between the hazard - for example a flood, and the effects of the hazard - for example death of livestock. Some effects, such as food shortages, may be the result of a combination of hazards, including climate shocks and stresses, declining soil fertility, and insecure access to markets. To effectively analyze vulnerability, we must understand the dynamic nature and interactions of hazards.

7 In some livelihoods frameworks, political capital is recognized as a sixth category of resources.
8 Adapted from: UNISDR, 2009. Terminology: Basic terms of disaster risk reduction and IISD et al, 2007. Community-based Risk Screening – Adaptation and Livelihoods (CRiSTAL) User's Manual, Version 3.0.
9 UNISDR, 2009. Terminology: Basic terms of disaster risk reduction.

Adaptation to Climate Change

In order to reduce vulnerability to climate change, we must focus on building adaptive capacity, particularly of the most vulnerable people; and, in some cases, on reducing exposure or sensitivity to climate impacts. We must also ensure that development initiatives don't inadvertently increase vulnerability. We call this process adaptation. Adaptation is defined as:

> *Adjustment in natural or human systems in response to actual or expected climatic stimuli or their effects, which moderates harm or exploits beneficial opportunities.*[10]

The adaptation of human systems is a process which requires the engagement of a wide range of stakeholders at multiple levels and in multiple sectors. It requires analysis of current exposure to climate shocks and stresses, and model-based analysis of future climate impacts. It demands an understanding of the existing vulnerability of individuals, households, and communities. With this information, adaptation strategies can be designed and implemented. Monitoring and evaluating the effectiveness of activities, as well as sharing knowledge and lessons learnt, are critical components of the process.

How is adaptation different from coping?

The terms "adaptation" and "coping" are sometimes used interchangeably, leading to confusion about the similarities and differences between these two important concepts. The following lists of characteristics are a compilation of brainstorming sessions by groups of development practitioners in Ghana, Niger and Nepal.

Coping	*Adaptation*
• Short-term and immediate<br>• Oriented towards survival<br>• Not continuous<br>• Motivated by crisis, reactive<br>• Often degrades resource base<br>• Prompted by a lack of alternatives	• Oriented towards longer term livelihoods security<br>• A continuous process<br>• Results are sustained<br>• Uses resources efficiently and sustainably<br>• Involves planning<br>• Combines old and new strategies and knowledge<br>• Focused on finding alternatives

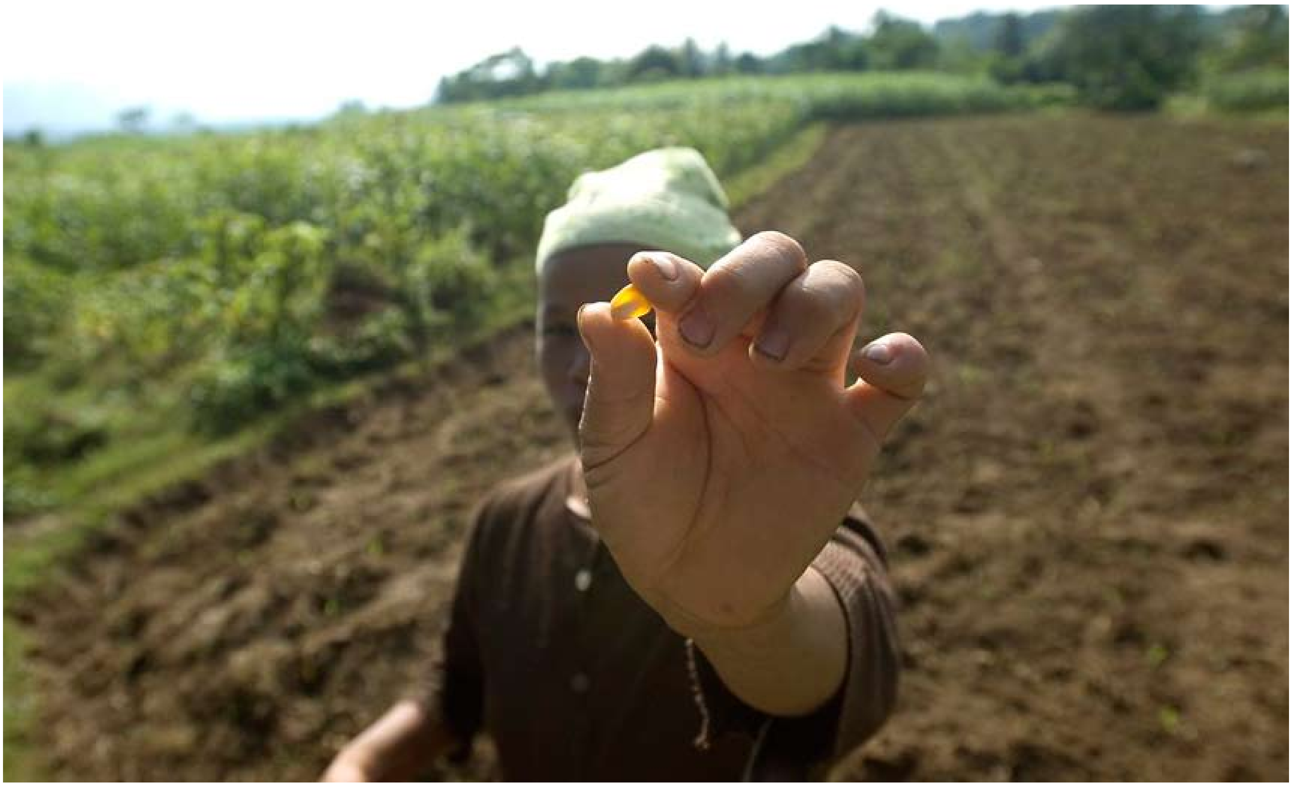

10 IPCC, 2007: Climate Change 2007: Impacts, Adaptation and Vulnerability. Contribution of Working Group II to the Fourth Assessment Report of the Intergovernmental Panel on Climate Change, Annex I., M.L. Parry, O.F. Canziani, J.P. Palutikof, P.J. van der Linden and C.E. Hanson, Eds., Cambridge University Press, Cambridge, UK, 976pp.

Linkages with Other Frameworks

The CVCA methodology is designed to complement analysis guided by other frameworks. Users are encouraged to explore these complementary frameworks to facilitate an integrated analysis.

Gender and Diversity - The success of development initiatives depends on equal relations between women and men and between different social groups. From a climate change perspective, this begins with an understanding of the differences in adaptive capacity between different groups and between men and women, and the design of adaptation strategies that ensure that vulnerable people have equal access to resources, rights and opportunities. It is increasingly recognized that women may be more vulnerable to climate impacts than men for a variety of reasons. On average, women are poorer and they typically lack secure access to the resources needed for adaptation. Women rarely have an equal say in decision-making in households, communities or in national politics. At the same time, experience has shown that women are central to permanently improving the lives of their families and communities, and therefore must play a pivotal role in community-based adaptation initiatives. Similarly, marginalized groups tend to have less security in access to and control over resources, and this contributes to their vulnerability to climate change. These underlying causes of vulnerability must be addressed in order to have a sustainable impact in reducing vulnerability to climate shocks. The CVCA methodology emphasizes differential vulnerability within communities and households to identify who is vulnerable and why.

Livelihoods Frameworks - Livelihoods frameworks guide users in a systematic process of understanding how people use diverse assets or resources (both tangible and intangible) to undertake a range of activities, in order to achieve important outcomes in their lives. These outcomes include satisfaction of basic rights, as well as sustainable access to basic needs like water, shelter, and food. Livelihoods analysis involves understanding how people access and control various mixes of resources and activities, and how these differ within and among households in ways that affect their ability to achieve the outcomes they desire in their lives. The analysis also helps us to determine how these are influenced by external factors such as gender and other social norms, policy frameworks, economic trends, and the physical environment. The CVCA is designed to apply a climate "lens" to livelihoods analysis. It examines the influence of the physical environment, but also helps in understanding how people are using resources and what aspects of livelihoods are most vulnerable.

Rights-Based Approaches - The focus of the CVCA on underlying causes of vulnerability to climate change is consistent with a rights-based approach to development. A rights-based approach (RBA) deliberately and explicitly focuses on people achieving the minimum conditions for living with dignity (i.e. achieving their human rights). It does so by exposing the roots of vulnerability and marginalization and expanding the range of responses. It empowers people to claim and exercise their rights and fulfill their responsibilities. A rights-based approach recognizes poor, displaced, and war-affected people as having inherent rights essential to livelihood security – rights that are validated by international law.[11]

11 Jones, Andrew. 2001. CARE's Program Cycle: Incorporation of a Rights-Based Approach, CARE USA, Atlanta.

The CVCA Process

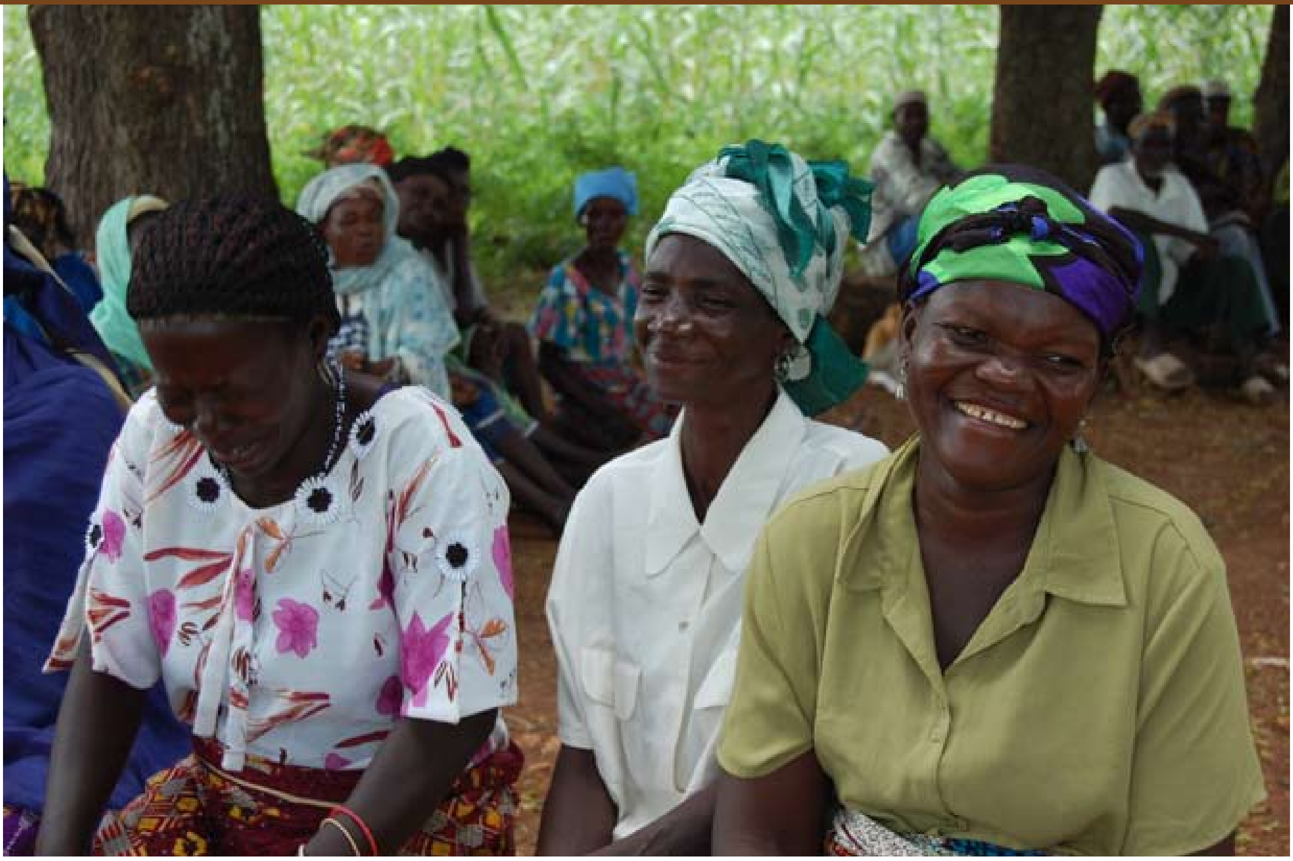

©CARE/Dazé, 2007

A group of women in Bansi Village in Bawku District in Northern Ghana enjoy a lighter moment during a participatory exercise.

The CVCA methodology provides a starting point for engaging stakeholders, assessing current vulnerability and understanding future climate risks. Its results provide an excellent foundation for designing, implementing and evaluating adaptation strategies through a participatory learning and planning process. The following sections describe the analytical framework and the steps in the CVCA process.

A Framework for Community-Based Adaptation

Climate change is only one of many challenges facing poor people. In order to effectively reduce vulnerability, climate change adaptation must form part of a holistic response which aims to build resilience of communities to withstand the range of shocks and stresses that they are exposed to.

From CARE's perspective, CBA requires an integrated approach which combines traditional knowledge with innovative strategies to address current vulnerability while building adaptive capacity to face new and dynamic challenges. The process of CBA involves four inter-related strategies:

- Promotion of climate-resilient livelihoods strategies in combination with income diversification and capacity building for planning and improved risk management;
- Disaster risk reduction strategies to reduce the impact of hazards, particularly on vulnerable households and individuals;
- Capacity development for local civil society and governmental institutions so that they can provide better support to communities, households and individuals in their adaptation efforts; and
- Advocacy and social mobilization to address the underlying causes of vulnerability, such as poor governance, lack of control over resources, or limited access to basic services.

Recognizing the importance of an enabling environment for effective CBA, our strategy is not limited to promoting change at the community level. CARE's approach also endeavours to influence policies at regional, national and international levels with community-based experience. This involves evidence-based advocacy as well as constructive engagement in key decision-making processes.

The analytical framework of the CVCA is based on CARE's CBA Framework. This Framework presents a range of "enabling factors" which must be in place at household/individual, community/local and national levels in order for effective community-based adaptation to take place. The CVCA process facilitates analysis of the existing situation with respect to these enabling factors. This helps to identify actions which can be taken to put the factors in place, creating an enabling environment for adaptation. These enabling factors are linked to the four strategies outlined above. The framework is presented below.

CARE's Framework for Community-Based Adaptation

	Climate-Resilient Livelihoods	Disaster Risk Reduction	Capacity Development	Addressing Underlying Causes of Vulnerability
**National Level**	• Government is monitoring, analyzing and disseminating current and future climate information related to livelihoods<br>• Climate change is integrated into relevant sectoral policies<br>• Climate change is integrated into poverty reduction strategy and/or other development policies	• Government is monitoring, analyzing and disseminating disaster risk information<br>• Government is engaged in planning and implementing disaster risk management (including prevention, preparedness, response and recovery)<br>• Functional early warning systems in place<br>• Government has capacity to respond to disasters	• Government has capacity to monitor, analyze and disseminate information on current and future climate risks<br>• Government has mandate to integrate climate change into policies<br>• National policies are rolled out at regional and local levels<br>• Resources are allocated for implementation of adaptation-related policies	• Government recognizes specific vulnerability of women and other marginalized groups to climate change<br>• Policy and implementation is focused on reducing these vulnerabilities<br>• Civil society is involved in planning and implementation of adaptation activities
**Local Government/ Community Level**	• Local institutions have access to climate information<br>• Local plans or policies support climate-resilient livelihoods<br>• Local government and NGO extension workers understand climate risks and are promoting adaptation strategies	• Local institutions have access to disaster risk information<br>• Local disaster risk management plans being implemented<br>• Functional early warning systems in place<br>• Local government has capacity to respond to disasters	• Local institutions have capacity to monitor, analyze and disseminate information on current and future climate risks<br>• Local institutions have capacity and resources to plan and implement adaptation activities	• Local planning processes are participatory<br>• Women and other marginalized groups have a voice in local planning processes<br>• Local policies provide access to and control over critical livelihoods resources for all
**Household/ Individual Level**	• People are generating and using climate information for planning<br>• Households are employing climate-resilient agricultural practices<br>• Households have diversified livelihoods, including non-agricultural strategies<br>• People are managing risk by planning for and investing in the future	• Households have protected reserves of food and agricultural inputs<br>• Households have secure shelter<br>• Key assets are protected<br>• People have access to early warnings for climate hazards<br>• People have mobility to escape danger in the event of climate hazardsHos	• Social and economic safety nets are available to households<br>• Financial services are available to households<br>• People have knowledge and skills to employ adaptation strategies<br>• People have access to seasonal forecasts and other climate information	• Men and women are working together to address challenges<br>• Households have control over critical livelihoods resources<br>• Women and other marginalized groups have equal access to information, skills and services<br>• Women and other marginalized groups have equal rights and access to critical livelihoods resources

NOTE: *Local institutions refers to both government and civil society organizations at the local level.*

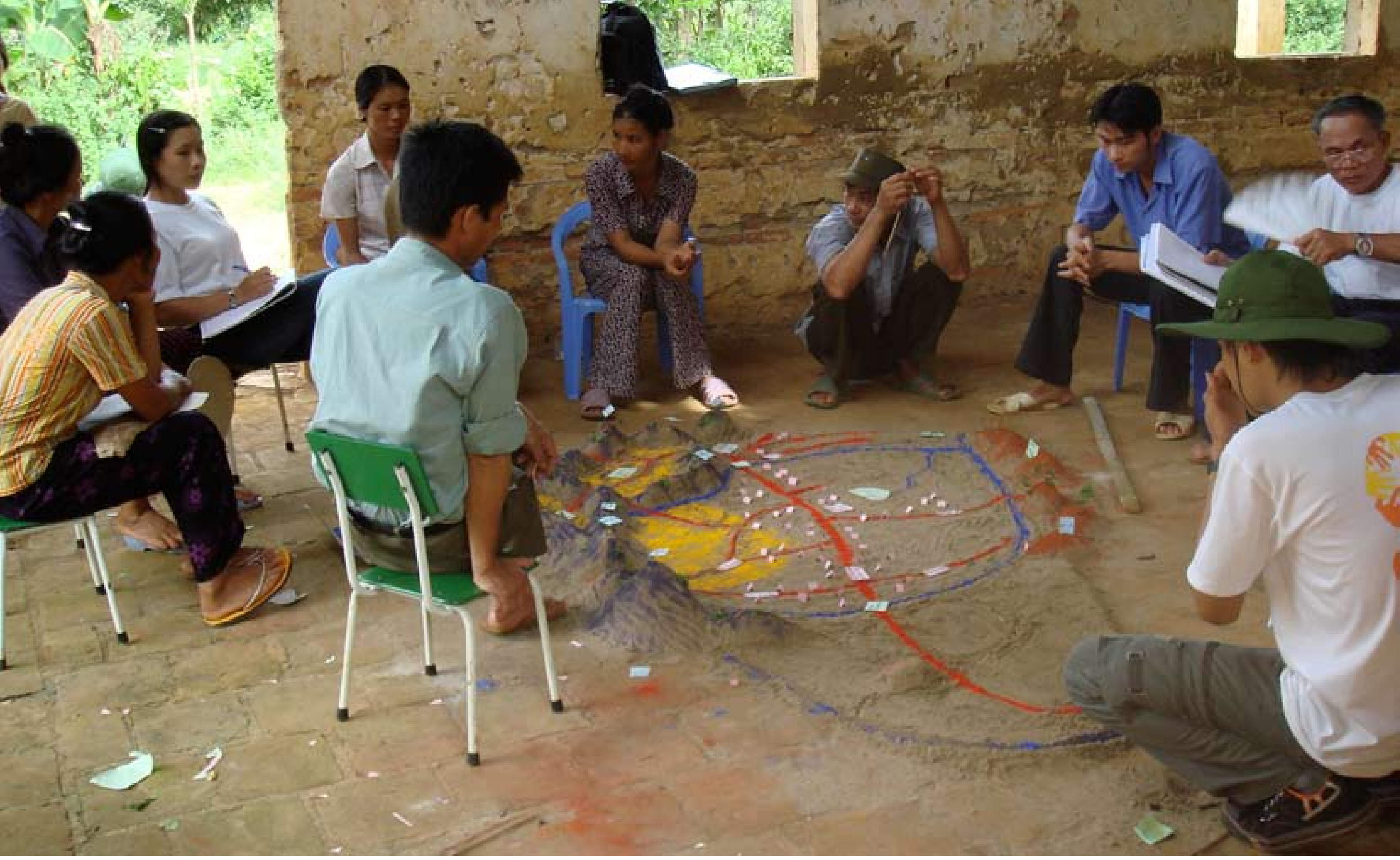

CVCAs: Where and When?

The CVCA process can be used in any community where a deeper understanding of vulnerability to climate change is desired. It may be particularly relevant for communities in areas or regions that have been identified as particularly vulnerable.[12] The process described in this Handbook is targeted towards rural communities due to their sensitivity to climate impacts; however it could easily be used and adapted for urban communities.

Ideally, the CVCA approach will be integrated into the analytical process undertaken at the beginning of a project cycle, so that the analysis can be used in the design of the project. This would apply regardless of whether the objective is to develop a targeted community-based adaptation project, or to integrate adaptation into a development project (see page 23 for further details). It could also be used in the initial stages of designing an advocacy campaign to develop the evidence base and identify allies and opponents.

Given the dynamic nature of vulnerability to climate change, it may be appropriate to update the CVCA analysis over the course of a project. The approaches suggested may also be incorporated into the monitoring and evaluation system of a project, to track changes in vulnerability resulting from project interventions and changing climate conditions.

Determining the Scope and Depth of the Analysis

The CVCA process can easily be adapted to reflect specific needs and constraints. The process described in this Handbook assumes that time and resources are available to undertake a relatively "deep" analysis. In this scenario, the desire is to gain a detailed understanding of the dynamics of vulnerability for different groups within the community. It requires significant engagement with communities and local stakeholders over a period of time to gather information, analyze the data, validate the analysis, and use it in a broader participatory planning process.

If resources are limited, or if the goal is to analyze a wider geographic area, a sampling approach may be used. Within the geographic boundaries, the analysis can target communities that have been pre-identified as particularly vulnerable, or a sample of communities which represent different agro-ecological zones and/or socio-economic profiles. Consultation with local government and NGO representatives is important in selecting target communities.

12 See, for example: Ehrhart, C. et al, 2009. Humanitarian Impacts of Climate Change: Mapping emerging trends and risk hotspots. CARE, UN OCHA and Maplecroft.

The amount of time it takes to do a CVCA really depends on the scope of analysis, number of stakeholders involved (community groups, households, government institutions, etc.) and the amount of additional/secondary information available in the target area. It will also depend on whether the analysis builds on an existing presence in the community. If not, more time will be needed to identify appropriate entry points and establish trust.

The CVCA guiding questions and tools can be easily integrated into other types of analyses such as livelihoods analysis, providing a climate "lens" for approaches already in use. This would be particularly relevant when the aim is to integrate adaptation into a development project.

Assembling the Team

Key skills and experience that would be helpful to have on an analytical team would include:

- Research skills – *for background research*
- Knowledge of climate change – *to analyze and summarize available climate information*
- Policy and institution*al analysis – to analyze the enabling environment*
- Scientific expertise – *in agriculture, water, and other relevant sectors*
- Facilitation of participatory processes –*to animate and balance the participation of everyone in the group, keep the group on track and to construct an environment of trust and openness*
- Gender and diversity – *to ensure gender and diversity-sensitive facilitation and to analyze differential vulnerability*
- Conflict management – *to help the group understand diverse perspectives and opinions, and to come to conclusions and/or consensus*
- Qualitative interviewing – *to listen actively and push for deeper reflection/additional information*
- Writing skills – *to present a convincing, clear and robust argument to various audiences for incorporating adaptation strategies within projects or as new activities*

When planning a CVCA, it is important to choose an analytical team with diverse backgrounds. A multidisciplinary team will be better positioned to undertake a holistic analysis. In order to ensure appropriate entry points into communities, and to increase trust during the field exercises, the team should include people who are known in the area, such as local NGO or CBO representatives. Team leaders should be aware that some people may have a vested interest in the outcomes of the analysis.

To identify potential team members for the analysis, an inventory of local organizations is helpful (see page 15). Ideally, local government representatives, either from the planning and administrative unit, or from line agencies such as agriculture or water, will also be members of the team. This will increase ownership of the process, as well as accountability for taking action on the results. It also serves to facilitate linkages between service providers and vulnerable groups who might not have an opportunity to interact with these organizations under normal circumstances.

Balancing Research with Learning

Connected to concepts and approaches to participatory development, as well as participatory action research, those applying the CVCA approach must be cautious of being "extractive". Although the user may be acting in a research capacity, he or she must also be a facilitator – of dialogue, perspectives, opinions, and thus, learning. The CVCA process is an opportunity to gather valuable information, but it is also an opportunity to enable multi-stakeholder learning – around new, or un-surfaced, issues. Ideally, this learning will be action-oriented whereby communities (and others) identify commitments towards adaptation. Facilitating critical analysis among a group will better enable them to be able to repeat the exercise in the future, and encourage momentum for the learning process.

Doing the Analysis

Building on CARE's CBA Framework, the CVCA process uses a series of guiding questions to analyse information at national, local government/community, and household/individual levels. The idea is to combine the information gained at different levels using the various tools in order to the guiding questions. With this information, users should be well-positioned to draw conclusions about adaptive capacity in the target communities, and to design appropriate interventions to support adaptation.

Guiding questions and suggested tools for gathering and analyzing data are presented below.

National Level

	Guiding Questions National Level
Resilient Livelihoods	• Is the government monitoring and analyzing current and future climate information related to livelihoods?<br>• If so, is this information being disseminated? How? To whom?<br>• What are the observed and predicted impacts of climate change for the country?<br>• What livelihood groups or economic sectors are most vulnerable to climate change?<br>• Is climate change integrated into relevant sectoral policies?<br>• Is climate change integrated into poverty reduction strategy and/or other development policies and programs?
Disaster Risk Reduction	• What are the most important climate-related hazards the country faces? Non-climate related?<br>• Are there particular parts of the country that are vulnerable?<br>• How are hazards likely to change over time as a result of climate change?<br>• Is the government monitoring and analyzing disaster risk information?<br>• If so, is this information being disseminated? How? To whom?<br>• Is the government engaged in planning and implementation of disaster risk management? If so, which ministries and/or government agencies are actively involved?<br>• Is climate change integrated into planning for disaster risk management?<br>• Are functional early warning systems (EWS) in place at the national level?<br>• Does the government have the capacity to respond to disasters?<br>• Which other institutions are engaged disaster risk management at national level?
Capacity Development	• What institutions are involved in research, planning and implementation of adaptation?<br>• What are the most important institutions in facilitating or constraining adaptation?<br>• Does the government have capacity to monitor and analyze information on current and future climate risks?<br>• Are there mechanisms in place to disseminate this information?<br>• Is an appropriate structure in place within the Government with a mandate to integrate climate information into relevant policies?<br>• Is this information being integrated into relevant policies?<br>• Are national policies rolled out at regional and local levels? Is the government responsive to local priorities?<br>• Are resources allocated for implementation of adaptation-related policies? What is the budget? Where are the resources coming from?<br>• What are the existing capacity and resource needs and/or gaps for climate change adaptation?<br>• What new capacities may be needed to address changing circumstances due to climate change?
Addressing Underlying Causes of Vulnerability	• Do those responsible for climate change policies and programs demonstrate understanding of the link between poverty and climate change vulnerability?<br>• Do those responsible for climate change policies and programs recognize the specific vulnerability of women and other marginalized groups to climate change?<br>• Is this knowledge and recognition translated into policy and implementation of programs? Do policies and programs support empowerment of vulnerable groups?<br>• Do vulnerable groups have advocates at national level?<br>• Is civil society involved in planning for adaptation?

Analytical Tools

Secondary Research

Before starting work in communities, it is important to know the bigger picture. Scientific information on climate change is generally available at the country level. This can help in identifying which climate-related shocks and stresses are likely to affect communities. It is important to know what information is available and re-package it in a way that will be interesting, relevant and easily understood in communities.

Helpful sources of information may include:

- National Communications to the United Nations Framework Convention on Climate Change (UNFCCC)
- Intergovernmental Panel on Climate Change (IPCC) Reports
- National Adaptation Programme of Action (NAPA) documents
- National Action Programme to Combat Drought and Desertification (NAP) documents
- Professional and academic journals
- Meteorological data on current climate trends
- Seasonal forecasts
- Maps showing topography, agro-ecological regions, infrastructure, etc.
- National census and poverty data

Most of these resources can be found on the internet and through national meteorological services.

FOR MORE INFO

The Intergovernmental Panel on Climate Change (IPCC) produces assessment reports which summarize information on climate change science, mitigation, impacts and adaptation: http://www.ipcc.ch/

National Communications to the UNFCCC can be downloaded at:
http://unfccc.int/national_reports/non-annex_i_natcom/submitted_natcom/items/653.php

The UNFCCC website also has completed NAPAs available for download: http://unfccc.int/adaptation/napas/items/4585.php

The World Resources Institute (WRI) EarthTrends fact sheets give handy country-level statistics on climate and energy: http://earthtrends.wri.org/#

The United Nations Development Program (UNDP) and the School of Geography and Environment at Oxford University have generated country-level studies of climate observations and multi-model projections for 52 developing countries, which are available at: http://country-profiles.geog.ox.ac.uk/

The United Nations International Strategy for Disaster Reduction (UNISDR) provides helpful information on disaster trends and disaster risk reduction: http://www.unisdr.org/

Institutional Mapping

To understand the context at the national level and to guide further analysis, it can be useful to undertake an institutional mapping exercise. Key issues to consider in the analysis would include:

- Which organizations (governmental and non-governmental) are involved in addressing key issues and problems related to climate change?
- What are the policy or strategy documents that guide their work?
- What are their activities that are relevant to adaptation?
- Do they have a mandate to address climate change issues?
- What is the institution's level of influence in addressing adaptation?
- What are their relationships with other organizations?
- What are the strengths and weaknesses of the institutions?

The institutional analysis provides useful information to plan the scope of the policy analysis, and to identify key stakeholders for further investigation.

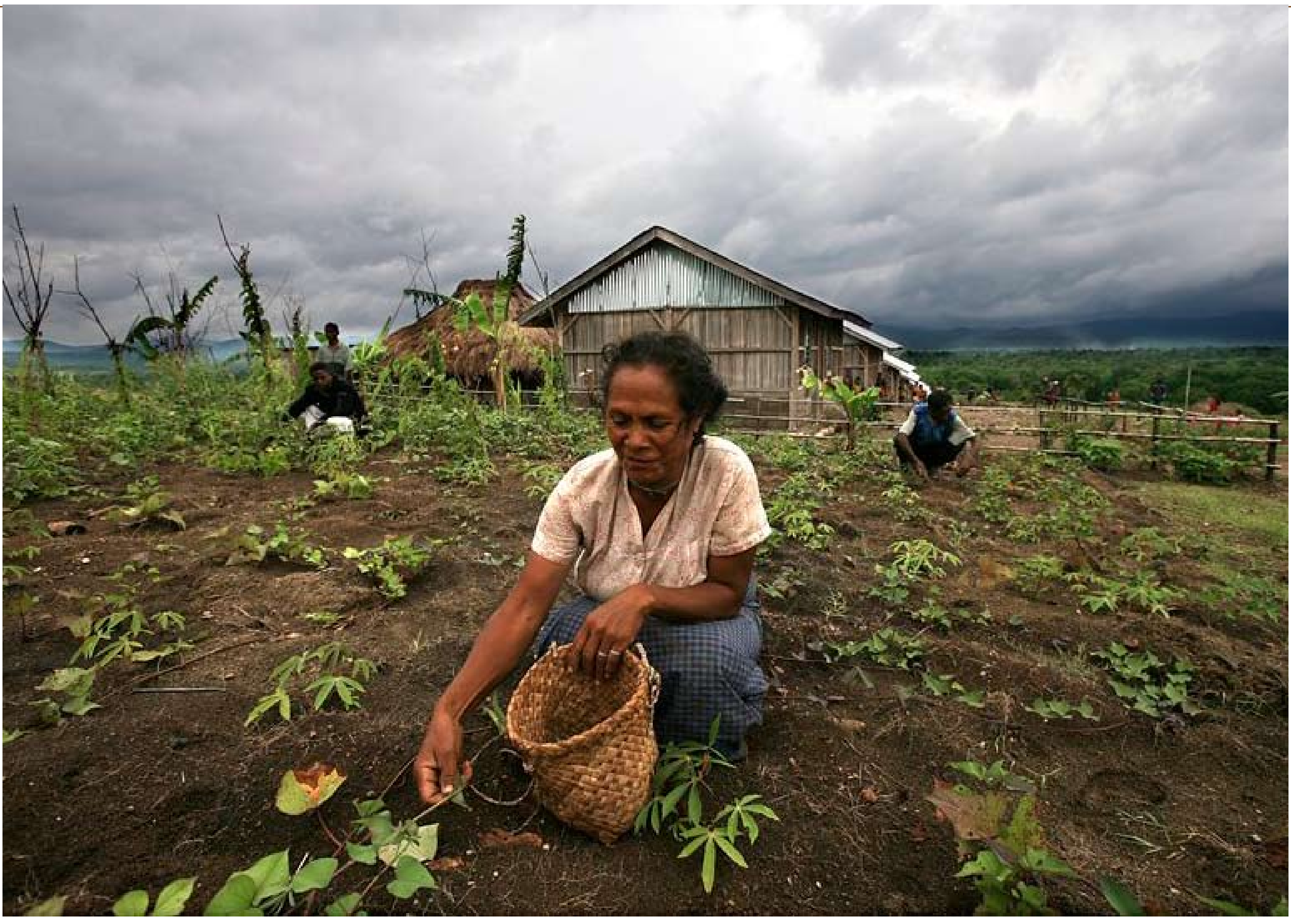

Policy Analysis

Decisions made by central governments can have a profound effect on the ability of communities to adapt to climate change. Policies in sectors such as water, agriculture, health, infrastructure, and economic development can facilitate or constrain adaptation. Integration of climate change considerations into these policies can ensure that they contribute to adaptive capacity from national to local levels. In some cases, existing policies provide opportunities to address climate change – as long as the capacities, resources and political will are in place to ensure they are implemented.

It is important to understand these dynamics and how they may affect adaptive capacity at the local government/ community, and household/individual levels. Therefore, the CVCA process should analyze relevant policies, focusing on the integration of climate change issues into policies, and on openings and barriers to facilitating adaptation in target communities.

Key Informant Interviews

Often policy documents present an optimistic scenario which is quite different from the reality of implementation. Therefore, it can be very useful to speak with key informants who can provide information and analysis on the implementation of relevant policies. Note that these informants may want to remain anonymous.

Key informants at the national level would include:

- Persons engaged in developing NAPAs or other climate change policies and plans
- Persons engaged in developing or implementing disaster risk management policies
- Persons leading decision-making in relevant sectors such as water, agriculture, economic development, etc.
- Civil society representatives engaged in advocacy on environment and natural resource management issues and/or rights of vulnerable people
- Representatives of donor agencies
- Academic or policy research experts in relevant sectors

Local Government/Community Level

Guiding Questions<br>*Local Government/Community Level*	
Resilient Livelihoods	- Are scaled-down climate projections available?<br>- If so, what are the observed and predicted impacts of climate change for the region and/or ecological zone?<br>- Do local institutions have access to information on current and future climate risks?<br>- What livelihood groups or economic sectors are most vulnerable to climate change?<br>- Do local plans or policies support climate-resilient livelihoods?<br>- Do local government and NGO extension workers understand climate risks and promote adaptation strategies?
Disaster Risk Reduction	- What are the most important climate-related hazards the region and/or ecological zone faces? Non-climate related?<br>- How are hazards likely to change over time as a result of climate change?<br>- What groups within the community are most vulnerable to disasters?<br>- Do local institutions have access to disaster risk information?<br>- Are local disaster risk management plans being implemented?<br>- Are functional early warning systems in place at the local level?<br>- Does the local government have the capacity to respond to disasters?<br>- Which other institutions are engaged disaster risk management at local level?
Capacity Development	- What institutions (governmental and non-governmental) are involved in research, planning and implementation of adaptation?<br>- What are the most important institutions in facilitating or constraining adaptation?<br>- Do local institutions (governmental and non-governmental) have capacity to monitor and analyze information on current and future climate risks?<br>- Are mechanisms in place to disseminate this information?<br>- Do local institutions have capacity to plan and implement adaptation activities?<br>- Are resources allocated for implementation of adaptation-related policies? What is the budget? Where are the resources coming from?<br>- What are the existing capacity and resource needs and/or gaps for climate change adaptation?<br>- What new capacities may be needed to address changing circumstances due to climate change?
Addressing Underlying Causes of Vulnerability	- What social groups within the community are most vulnerable to climate change?<br>- Are local planning processes participatory?<br>- Do women and other marginalized groups have a voice in local planning processes?<br>- Do local policies provide access to and control over critical livelihoods resources for all?<br>- What are the other factors constraining adaptive capacity of the most vulnerable groups? Do vulnerable communities and groups have any influence over these factors?

Analytical Tools

Secondary Research

An understanding of the livelihoods strategies, socio-economic situation, power dynamics and local governance in the target communities is critical to ensuring that facilitators are effective during the field work, and to identifying focus groups within the community.

Secondary sources for community-level information would include:

- Assessment reports from NGOs or UN organizations
- Evaluations of past disaster response operations

- Vulnerability monitoring programs (e.g. Famine Early Warning System (FEWS-Net)
- Environmental screening reports for the target area
- Government documents including poverty reduction strategies, development plans, official statistics, etc.
- Consultation with agencies (governmental and non-governmental) working in the target area
- Seasonal forecasts

Policy Analysis

Depending on the degree of decentralization of decision-making in a particular country, local-level plans or policies may be important in shaping adaptive capacity of vulnerable households and individuals. Regional or district plans and/or sector strategies can give helpful information on priorities of local governments. Further, the process for developing these policies and strategies can provide insights into the level of participation of vulnerable people in establishing these priorities. The status of implementation can yield useful information on resource and capacity constraints faced by local actors.

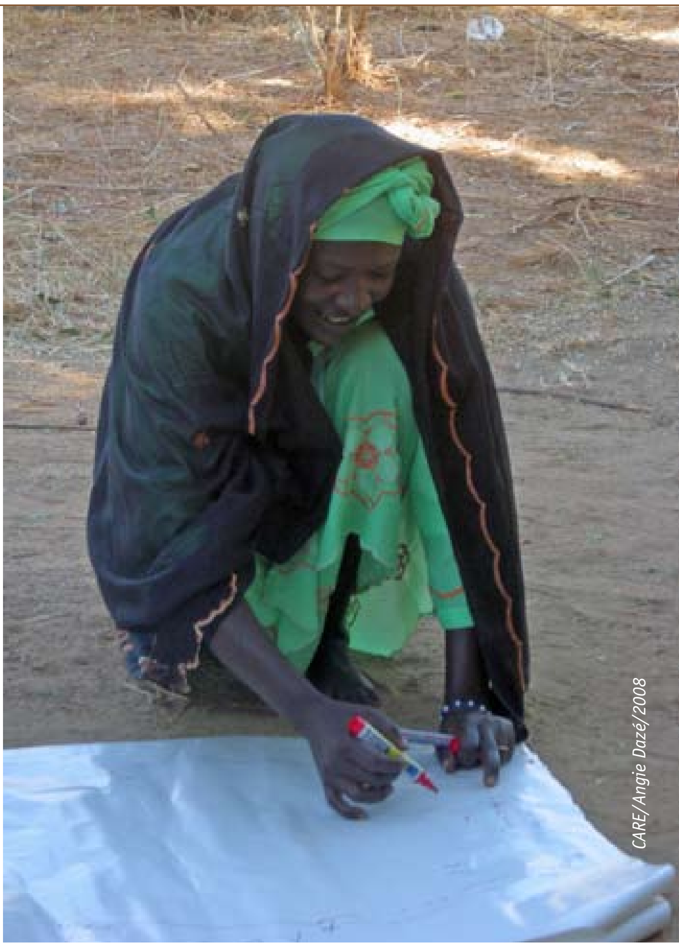

In Soudoure Village in Niger, a group of women work collectively to draw a hazard map for their community.

Institutional Mapping

Institutions play a critical role in supporting or constraining people's capacity to adapt to climate change. In order to better understand which institutions are most important to people in the target communities, an institutional mapping exercise is useful. Institutional mapping involves examination of the following questions:

- Which organizations (governmental, non-governmental and community-based) are involved in addressing key issues and problems related to climate change?
- What do they do?
- Where do they work?
- How do they interact with the target population?
- Where are the overlaps with other organizations?
- Where are the gaps in capacity?
- How might some organizations impede the work of others?
- What are their longer term plans for working in the area?
- What are the strengths and weaknesses of the institutions?[13]
- What is the institution's level of influence over planning and implementation of adaptation?

The mapping exercise assists in identifying the institutions that should be engaged in the CVCA process, as well as potential allies and opponents in addressing vulnerability at the community level.

Key Informant Interviews

Key informants can provide useful insights into local governance structures and status of implementation of local policies and programs. Power issues within and between communities and other stakeholders can also be surfaced through interviews with key actors. Again preserving their anonymity may allow them to speak more freely.

Key informants at the local government/community level would include:

- Local leaders (chiefs, mayors, elected representatives, etc.)
- Representatives of community-based organizations (CBOs) such as farmer's groups, water and sanitation committees, savings and credit groups, etc.

13 CARE, 2002. Household Livelihood Security Assessment Toolkit for Practitioners.

- Representatives of women's groups or other rights-based groups
- Representatives of NGOs working on programs or advocacy in the target area
- Academic/research institutions engaged in the target area

FOR MORE INFO

CARE's Household Livelihood Security Assessment Toolkit provides helpful advice on planning assessments, doing key informant interviews, and other relevant approaches:
http://www.proventionconsortium.org/themes/default/pdfs/CRA/HLSA2002_meth.pdf

The Institute of Development Studies: Livelihoods Connect website has links to several useful tools for policy and institutional analysis: http://www.livelihoods.org/info/info_toolbox.html

Tearfund's Advocacy Toolkit includes a number of different tools for researching and analyzing the broad country context:
http://tilz.tearfund.org/webdocs/Tilz/Roots/English/Advocacy%20toolkit/Advocacy%20toolkit_E_Part%20C_2_Research%20and%20analysis.pdf

Provoking change: A Toolkit for African NGOs includes guidance on tools for advocacy work, including stakeholder analysis:
http://www.fern.org/media/documents/document_3914_3917.pdf

Household/Individual Level

Guiding Questions Household/Individual Level	
Resilient Livelihoods	- What are the most important livelihoods resources to different groups within the community?<br>- What changes in climate is the community observing? Are traditional weather prediction systems working?<br>- What coping strategies are currently employed to deal with shocks and stresses?<br>- Are people generating and using climate information for planning?<br>- Are households employing climate-resilient agricultural practices?<br>- Do households have diversified livelihoods strategies? Does this include non-agricultural strategies?<br>- Are people managing risk by planning for and investing in the future?
Disaster Risk Reduction	- What are the biggest climate-related hazards faced? Non-climate related hazards?<br>- How are hazards likely to change over time as a result of climate change?<br>- Do households have protected reserves of food and agricultural inputs?<br>- Do households have secure shelter?<br>- Are key assets protected from hazards?<br>- Do people have access to early warnings for climate hazards?<br>- Do people have mobility to escape danger in the event of climate hazards?
Capacity Development	- Are social and economic safety nets available to households?<br>- Are financial services available to households?<br>- Do people have knowledge and skills to employ adaptation strategies?<br>- Do people have access to seasonal forecasts and other climate information?
Addressing Underlying Causes of Vulnerability	- Are men and women working together to address challenges?<br>- Do households have control over critical livelihoods resources?<br>- Do women and other marginalized groups have equal access to information, skills and services?<br>- Do women and other marginalized groups have equal rights and access to resources?<br>- Are there other social, political or economic factors which make particular people within the community more vulnerable than others?<br>- Do these vulnerable groups have any influence over these factors?

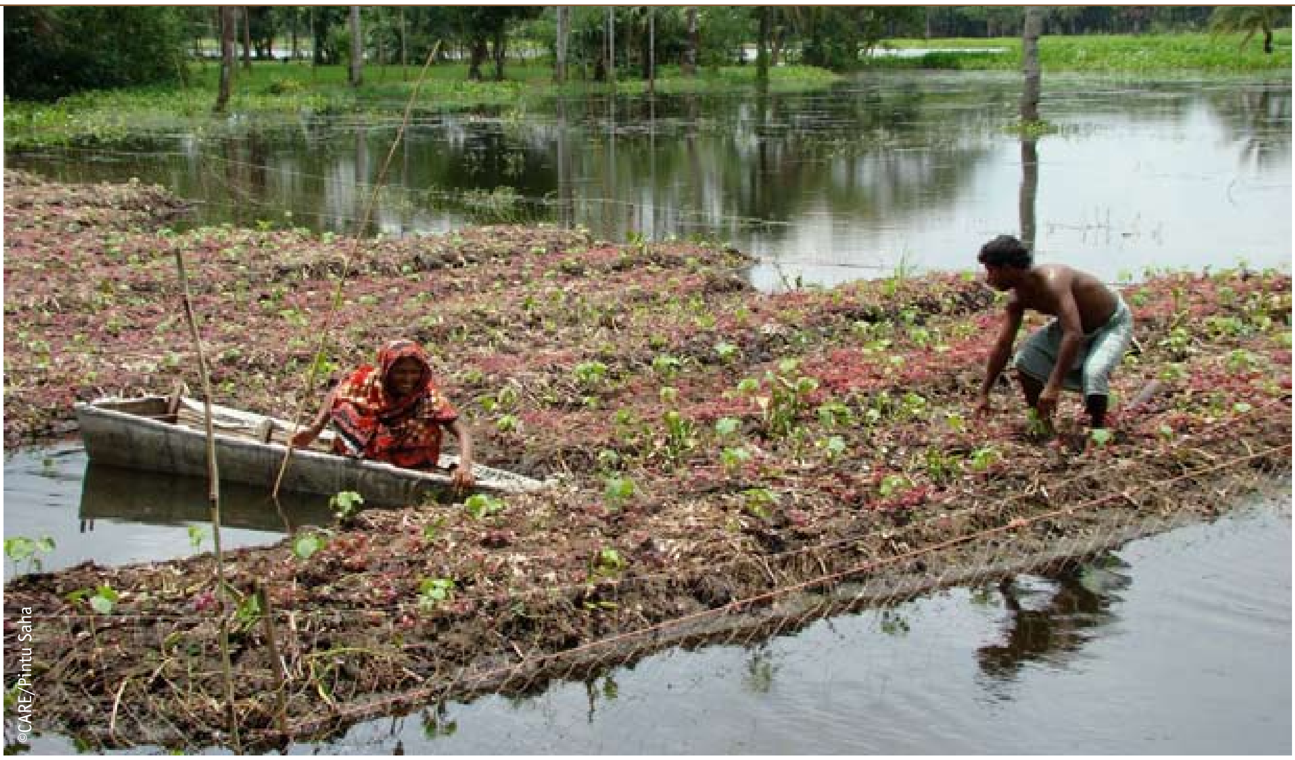

Farmers working in floating gardens in Chadra village Jessore Bangladesh

Analytical Tools

Secondary Research

In order to effectively plan the field work and to ensure that communities are not over-burdened with research and assessment teams, it is important to review existing information.

Sources of information on livelihoods would include:

- Assessment reports from NGOs or UN organizations
- Project/program baseline studies and/or evaluation reports
- Vulnerability monitoring programs (e.g. Famine Early Warning System (FEWS-Net))
- Post-disaster assessments
- Consultation with agencies (governmental and non-governmental) working in the target area
- Maps showing topography, agro-ecological regions, infrastructure, etc.

In some cases, it will be possible to answer many guiding questions using secondary sources, however this information must be verified by local stakeholders. Having more background information can allow the field work to focus specifically on climate change issues. In many cases, very little information may exist at the household/individual level, and so a deeper participatory analysis will be required to understand the dynamics of vulnerability.

Participatory Tools

Secondary research is complemented by collaborative learning employing typical participatory tools and discussions in focus groups (FGs).

FGs usually involve 5 – 12 people selected to be representative of different livelihood systems and/or vulnerable groups in the community. A single FG can include people selected by age and gender (e.g. teenage girls, or elderly women, or young married men), or by some other common characteristic (e.g. people with chronic illnesses, or members of farmer associations). At a minimum, it is suggested to conduct discussions with groups of men and women separately so that participants feel free to talk openly.

Participatory tools are designed to draw out issues which can then be examined further through semi-structured discussion. This is meant only as a guide; the field work must be tailored to the particular context and the objectives of the analysis. As well, the range of tools used will depend on the time and resources available for the field work.

Fostering participatory processes, and balancing learning with information-gathering, relies on strong, thoughtful facilitation. The Field Guides at the end of this Handbook provide facilitation tips as well as detailed guidance on using participatory tools and facilitating discussions with focus groups.

	Field Guide	Purpose
1	*Facilitation Tips*	- To guide planning and preparation for community visits<br>- To provide general guidance on effective facilitation
2	*Hazard Mapping*	- To become familiar with the community, and to see how the place is perceived by different groups within the community<br>- To identify important livelihoods resources in the community, and who has access and control over them<br>- To identify areas and resources at risk from climate hazards<br>- To analyze changes in hazards and planning for risk reduction
3	*Seasonal Calendars*	- To identify periods of stress, hazards, diseases, hunger, debt, vulnerability, etc.<br>- To understand livelihoods and coping strategies<br>- To analyze changes in seasonal activities<br>- To evaluate use of climate information for planning
4	*Historical Timeline*	- To get an insight into past hazards, changes in their nature, intensity and behaviour<br>- To make people aware of trends and changes over time<br>- To evaluate extent of risk analysis, planning and investment for the future
5	*Vulnerability Matrix*	- To determine the hazards that have the most serious impact on important livelihoods resources<br>- To determine which livelihoods resources are most vulnerable<br>- To identify coping strategies currently used to address the hazards identified
6	*Venn Diagram*	- To understand which institutions are most important to communities<br>- To analyze engagement of different groups in local planning processes<br>- To evaluate access to services and availability of social safety nets

Compiling and Analyzing the Data

After completing the field work, the teams should review the information gathered to identify any gaps in the information collected. Follow-up interviews or further research may be required to fill gaps.

Field teams from the same community should then sit together to analyze the information gained. Comparing the results for different groups within the community is an important part of the process, as this yields insights on differential vulnerability. The analysis may expose inequalities within the community which may not have been previously recognized. Follow-up discussions or interviews with particularly vulnerable groups may be needed to fully understand community or household dynamics.

Once information for specific communities has been analyzed, it can be helpful for teams who worked in different communities to come together to identify trends, common issues, differences, and to evaluate the process.

The community information should then be combined with the information gained using other tools in order to answer the guiding questions.

Validating the Analysis

After preliminary analysis of the data has been completed, a presentation of the findings should be made to community representatives to confirm the validity of the conclusions. A two-step approach is suggested for the validation process. The first step would be to present the analysis to the community focus groups themselves to ensure that the conclusions drawn are correct. Next, it is recommended that the results are presented to a wider community group and local organizations to facilitate dialogue on issues that have been raised by particular groups which may have implications for other groups. In particular, this provides an opportunity to make other groups in the community aware of the views of particularly vulnerable groups. Note that there may be sensitivities around some of the issues raised by different groups, and facilitators must

be prepared to resolve conflicts that may arise. It must also be ensured that the sharing of views does not yield negative consequences for any members of the community. Local actions can provide guidance on this.

Feedback from stakeholders should be incorporated into the final analysis.

People-centred development

People-centred development builds relationships with individuals and communities as a foundation for creative collaboration around an emergent agenda – such as climate change and adaptation. Development actors (e.g. farmers, women's groups, individual households, local NGOs, governments, women, men, communities) are viewed as knowledgeable and capable of solving their own problems. With this in mind, the CVCA methodology seeks to build new relationships between multiple stakeholders and their environment and among people themselves, depending largely on, and celebrating, local perceptions and creativity. This necessitates a more iterative and open-ended process where it is impossible to predict thematic details or timelines, but where the facilitator must carefully observe when and how these emerge. Overall, the central factor driving people-centred interventions is common understanding with communities, e.g., knowledge on what people do and why. Participation in the CVCA process can be viewed as a means to help local actors gain further control over knowledge and resources in ways that enable them to advance individual and collective interests on their own, as well as in collaborative arrangement (for example, with other entities in their enabling environment, such as local governments).

However, time and pre-determined plans can often dictate supposedly "participatory" processes. Different types of participation are outlined below. The CVCA encourages users to carefully consider the type of participation they are promoting, and to strive for those which emphasize empowerment.

- *Passive Participation:* People participate by being told what has been decided or has already happened. Information being shared belongs only to external professionals.
- *Participation by Consultation:* People participate by being consulted or by answering questions. Process does not concede any share in decision-making, and professionals are under no obligation to take on board people's views.
- *Bought Participation:* People participate in return for food, cash or other material incentives. Local people have no stake in prolonging technologies or practices when the incentives end.
- *Functional Participation:* Participation seen by external agencies as a means to achieve their goals, especially reduced costs. People participate by forming groups to meet predetermined objectives.
- *Interactive Participation:* People participate in joint analysis, development of action plans and formation or strengthening of local groups or institutions. Learning methodologies used to seek multiple perspectives, and groups determine how available resources are used.
- *Self-Mobilisation and Connectedness:* People participate by taking initiatives independently to change systems. They develop contacts with external institutions for resources and technical advice they need, but retain control over how resources are used.[14]

Documenting and Disseminating the Analysis

Documentation is a key part of the CVCA process. If the analysis is used in the design of a project, documenting the process and the analysis will help to ensure that the basis for decisions on the project is clear in the future. It may also be useful to integrate the CVCA analysis into the M&E of the project, in which case the initial analysis would form part of the project baseline. Finally, this type of analysis can be very useful to other stakeholders working in the project area, including local government authorities, other NGOs, and the communities themselves. The report from the CVCA process should be shared with these other actors to help them in integrating climate change adaptation issues into their work.

The following is a suggested outline for a report on a CVCA analysis:

CVCA Process

- Provide details of the overall process undertaken: timeline, membership of analytical team, objectives of the analysis, etc.
- Note the sources of secondary information and the names of key informants (if they agree to be identified, otherwise just note the number of people interviewed), etc.

14 Pretty, Jules, 1995. Typology of Participation. Participatory Learning for Sustainable Agriculture in World Development, Vol.23, No.8.

- Provide details of participatory research: number of focus group discussions, location, number and characteristics of participants, names and designations of facilitators, etc.
- Describe the process of interpreting and validating the analysis.

Climate Context

- Provide analysis of integration of climate change issues in relevant policies and programs.
- Identify current climate hazards (events and conditions) facing target area (country, region, community).
- Describe how climate hazards (events and conditions) are likely to change based on available climate change predictions.
- Document community observations of climate change.

Livelihoods-Climate Linkages

- Provide analysis of the contribution to resilience of relevant sectoral policies.
- Identify vulnerable livelihood groups or economic sectors.
- Identify resources important to livelihoods and adaptation.
- Describe impact of current and future climate hazards (including changing conditions) on resources and livelihoods.
- Identify current coping strategies identified and evaluate their effectiveness and sustainability.

Changing Disaster Risks

- Provide analysis of current and future disaster risks (climate-related and non-climate-related).
- Provide analysis of disaster risk management policies.
- Describe and evaluate monitoring and dissemination systems for disaster risk information.
- Describe and assess capacity at national, local and community level to respond to disasters, including evaluation of past responses.
- Provide analysis of household/individual vulnerability to hazards.

Institutional Context Related to Climate Change

- Describe government structures to address climate change.
- Describe and assess capacity of relevant institutions to integrate climate change considerations into their work.
- Provide analysis of linkages between national policies and local implementation.
- Provide analysis of resource allocation for adaptation-related activities at national and local levels.

Underlying Causes of Vulnerability

- Provide analysis of impact of policies and programs on access to and control over critical livelihoods resources.
- Provide analysis of impacts of policies and programs on women and other marginalized groups.
- Describe and evaluate participation (particularly of vulnerable groups) in policy decisions at national and local levels.
- Provide analysis of inequalities within communities or households which exacerbate vulnerability (such as access to services, control over resources, mobility, etc.).

In addition to sharing a written report, it can also be helpful to hold a workshop or meeting to present the community-validated results to local and national governmental and non-governmental organizations, particularly those who have not been involved in the analysis. Ideally the information will be presented by community representatives to demonstrate their ownership of the process and to facilitate dialogue between communities and other stakeholders. Again, facilitators must be prepared for conflict around sensitive issues, and must work with local actors to facilitate constructive dialogue.

Using the Analysis

The results of the CVCA provide us with insights into the policy and institutional issues that constrain the ability of vulnerable communities to adapt to climate change. They also help us to understand inequalities within communities and households that make certain groups or people more vulnerable. Finally, they provide us with "first-hand" accounts of the impacts of climate change on vulnerable people.

The framework for CBA presented on page 8 provides a general view of the enabling factors that must be in place in order for adaptation to take place. In order to increase adaptive capacity, actions must be taken to facilitate the attainment of these conditions. This can occur through advocacy on climate change issues, by integrating climate change vulnerability issues into programs in other sectors, or through targeted adaptation programs.

Adaptation Advocacy

A rights-based approach to adaptation requires us to challenge existing policies and power structures to facilitate adaptation by vulnerable people. The understanding gained through the CVCA provides us with a platform for designing advocacy campaigns to address these issues. These campaigns may be locally-based and targeted at facilitating rights of vulnerable people to livelihoods resources. They may be targeted at national-level policies which are not supportive of adaptation or do not recognize specific needs and priorities of particularly vulnerable groups. Or, they may have an international focus, highlighting the consequences of the international climate change policy framework for vulnerable people. In any of these cases, the CVCA helps us to identify allies and targets, decide on messaging, and generate evidence for advocacy campaigns.

Integrating Adaptation into Development Projects

Integrating or "mainstreaming" climate change adaptation into development projects can increase the sustainability and impact of interventions in sectors such as water, agriculture, livelihoods, and health. Climate change impacts can seriously affect development results, in some cases completely setting back gains that have been made. At the same time, development projects have the potential to increase the resilience of target populations to a range of threats including climate change or, if designed incorrectly, to constrain it.

When we talk about integrating adaptation at the project level, we are describing a process of considering climate risks to development projects, and of adjusting project activities and approaches to address those risks. The assumption is that the project has a goal related to poverty reduction, livelihood security, or improved well-being for target populations, and that the sustainability and impact of the initiative can be increased by integrating climate change. This is different from a "targeted" community-based adaptation project, where the explicit goal is to reduce vulnerability to climate change.

Integrating adaptation can achieve two main objectives:

- Reducing the risks posed by climate change to project activities, stakeholders, and results, sometimes referred to as "climate-proofing".
- Ensuring that project activities reduce the vulnerability of target populations to climate change, through interventions designed to build their adaptive capacity while achieving development goals.[15]

"Climate-proofing" is primarily concerned with protecting investments in, and results from, development initiatives from the impacts of climate change. It increases the sustainability of projects by analyzing the risks posed by climate change to project activities, stakeholders and results, and by making adjustments to the project design or implementation plan to mitigate those risks.

The second objective of integrating adaptation into projects recognizes that activities that aim to reduce poverty can inadvertently increase the vulnerability of target populations, and that adjustments to project activities and approaches can yield greater increases in adaptive capacity. By analyzing vulnerability of target populations to climate change, and adjusting project activities to maximize their contribution to adaptive capacity, the impact of development projects can be significantly increased.

It should be noted that in some areas, there is significant uncertainty in predictions of climate change impacts. When this is the case, adaptation actions should focus on "no regrets" approaches that will increase capacity to adapt to a range of likely scenarios. As well, no project will ever be truly "climate-proof". The best we can do is to understand the range of risks that the

15 Klein, R.J.T. et al. 2007. Portfolio Screening to Support the Mainstreaming of Adaptation to Climate Change into Development Assistance. Tyndall Centre Working Paper 102. Tyndall Centre for Climate Change Research, University of East Anglia, Norwich.

project may be exposed to, make our best efforts to mitigate and monitor those risks, and regularly adapt project parameters as conditions change or as our understanding improves.

For community-level projects, the most useful tool for integrating adaptation is *CRiSTAL: Community-based Risk Screening Tool – Adaptation & Livelihoods*. CRiSTAL is designed to assist project planners and managers with integrating risk reduction and climate change adaptation into livelihoods projects. Developed by the International Institute for Sustainable Development (IISD) in partnership with the International Union for the Conservation of Nature (IUCN), Stockholm Environment Institute (SEI) and Intercooperation, the tool is designed to help users to: systematically understand the links between local livelihoods and climate risks; assess a project's impact on community level adaptive capacity; and make adjustments to improve a project's impact on adaptive capacity. The tool is highly compatible with the CVCA. The information gained from the CVCA can feed directly into CRiSTAL, which facilitates analysis of implications for project activities.

The CRiSTAL tool can be downloaded at: www.cristaltool.org.

> FOR MORE INFO
>
> In addition to CRiSTAL, there are a number of other tools and methodologies available to support integration of adaptation into development programs at both project level and strategic planning level.
>
> For a summary of climate adaptation tools, see: http://www.iisd.org/pdf/2007/sharing_climate_adaptation_tools.pdf

Community-Based Adaptation Projects

In some contexts, targeted action on adaptation may be the best approach. A community-based adaptation (CBA) project is one where the explicit objective is to reduce vulnerability to climate change. In line with the CBA framework, action on adaptation must target multiple levels and incorporate a range of different strategies. The following sections present examples of the types of activities that may be undertaken within a CBA project to put in place the "enabling factors" for adaptation. Note that these are simply examples - they may not be appropriate in all contexts, and do not represent an exhaustive overview of the types of activities that comprise adaptation. As well, as noted above, when the potential consequences of climate change are unclear, the best approach is to focus on actions which will increase capacity to adapt in the face of uncertainty.

National Level

National policies and programs can play an important role in determining local-level adaptive capacity. In order for adaptation policies to be fair and effective, decision-making on the design and implementation of adaptation activities must be inclusive and transparent, with active and meaningful participation of vulnerable groups. Consequently, community-based adaptation projects can and should endeavour to influence the development and implementation of policies and programs related to adaptation. CBA projects can also facilitate the two-way flow of information between the community level and the national level, to ensure that local people have access to information needed for adaptation, and that national decisions are grounded in local reality.

The table below provides examples of the types of national-level activities which may support community-based adaptation:

	Creating an Enabling Environment for CBA<br>National Level
Resilient Livelihoods	• Summarizing climate data in user-friendly formats<br>• Disseminating climate change information to sectoral actors<br>• Review of national sectoral policies with climate "lens"<br>• Integration of climate vulnerability issues into poverty reduction strategies and/or other development plans
Disaster Risk Reduction	• Support for planning for disaster risk management<br>• Build capacity of disaster risk management actors on climate change<br>• Support development of early warning systems<br>• Build government capacity to respond to disasters
Capacity Development	• Map climate change adaptation capacity among national institutions<br>• Support roll out of national policies at regional and local levels<br>• Advocate for inclusive and transparent decision-making on adaptation
Addressing Underlying Causes of Vulnerability	• Raise awareness of vulnerability of women and other marginalized groups to climate change<br>• Strengthen civil society involvement in planning for adaptation<br>• Support advocacy for rights of vulnerable people

Adaptation in Action: Advocating for Rights to Safe Water in Bangladesh

In Bangladesh, CARE's analysis of the national context, combined with participatory research data, led to a logical focus on the right to safe water for the advocacy component of the project. Safe water is already a serious challenge in the area, and the impacts of climate change will only exacerbate the problem. The goal of the campaign was to get the government to provide safe water to vulnerable populations, and to address the problem of saline intrusion, in the south-western part of the country. CARE and civil society partner the *Pani* Committee launched a grassroots campaign to mobilize citizen support of the issue. This was a multifaceted effort involving a number of activities, including a letter campaign to the Prime Minister which led to over 10,000 letters from residents describing their struggles to access water, and demanding action to provide safe water; district rallies that attracted hundreds of residents; and a symbolic strike attended by national political leaders and Members of Parliament, who urged the government to take action on the water crisis.

The pleas for safe water found a receptive audience, evidenced by the resulting commitments of government leaders. Following the actions by CARE, partners and communities, the Prime Minister's Office issued a directive to relevant ministries to address the potable water issue in the southwest region. A second example of the campaign's success is the allocation of resources by the Prime Minister for the public health department to work on water supply in the region. From a strategic perspective, the project illustrates how efforts to mobilize grassroots action and to constructively engage with policymakers can work in effecting change. Through this collaboration, combined with the strengthened capacity of the local partner organizations, CARE and its partners have made great headway in creating an enabling environment for adaptation.

The Reducing Vulnerability to Climate Change (RVCC) Project in Bangladesh was implemented with funding gratefully received from the Canadian International Development Agency (CIDA).

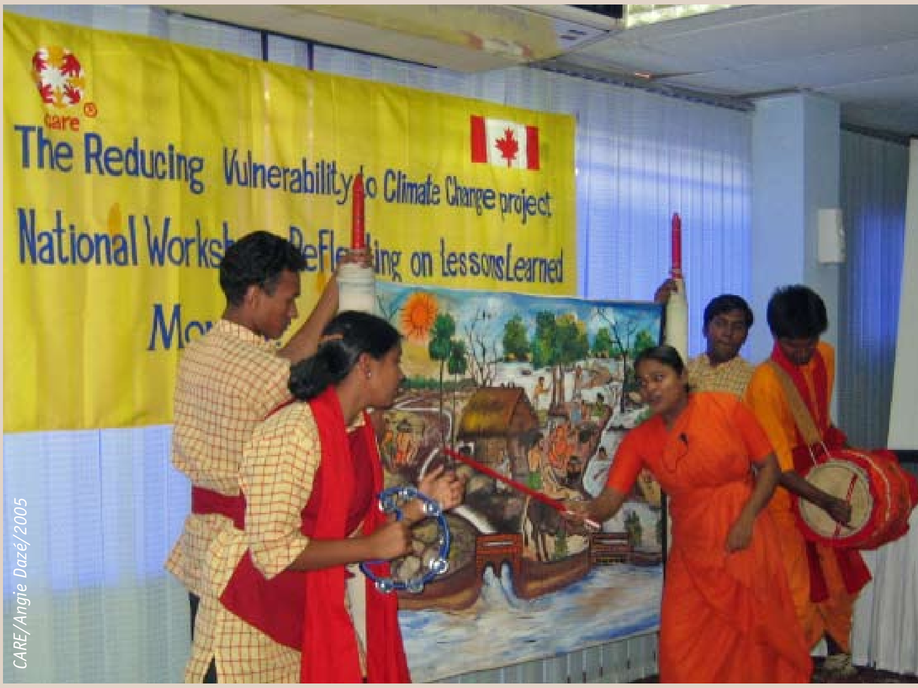

CARE partner Rupantar perform a song and dance about climate change and adaptation at a National Workshop in Dhaka, Bangladesh.

Local Government/Community Level

The CVCA process can act as a catalyst for dialogue in communities on issues of vulnerability. The approach is designed to help people articulate their experience and to draw conclusions that may inform future decision-making. Viewing their experience through a different lens may lead to the identification of new approaches in their livelihoods strategies, or new alliances within the community to try to solve common problems.

Ideally, local institutions (governmental and non-governmental) will have been engaged in the process of gathering data and facilitating dialogue in the communities. If this is the case, the hope is that they will internalize the information, and that it will be factored into future planning and programs. The process can also facilitate linkages between vulnerable people and local organizations that may not have occurred previously. If it has not been possible for local organizations to participate in the process, it is important to present the results in a way that will be useful for local institutions, highlighting the priorities and needs of the most vulnerable which may not normally be considered in local planning processes.

The following table suggests activities which may be appropriate at the local government/community level:

	Creating an Enabling Environment for CBA<br>*Local Government/Community Level*
Resilient Livelihoods	- Scaling down climate projections<br>- Review of local plans/policies using climate change "lens"<br>- Training for local government and NGO extension workers on vulnerability analysis and adaptation<br>- Promoting climate-resilient agricultural practices<br>- Supporting income diversification, including non-agricultural livelihoods strategies
Disaster Risk Reduction	- Supporting implementation of local disaster risk management plans<br>- Facilitating establishment of locally-appropriate early warning systems<br>- Building local government and community capacity to respond to disasters
Capacity Development	- Promoting participatory planning processes at local levels<br>- Building capacity of local institutions to analyze climate risks and plan for appropriate actions<br>- Establishing mechanisms for communication of climate information
Addressing Underlying Causes of Vulnerability	- Supporting voices of women and other marginalized groups in local planning processes<br>- Advocacy on access to and control over critical livelihoods resources in local policies

Adaptation in Action: Integrating climate change into district planning in Ghana

In Ghana, CARE is working with local communities to promote the integration of climate change adaptation issues into the Medium Term Plans (2010-2015) for two districts in northern Ghana – East Mamprusi and Bawku. Using the CVCA process, key vulnerability issues were identified in target communities, with a particular emphasis on vulnerable groups. The process demonstrated the impacts of climate change in the region, with floods, droughts and erratic rainfall identified as key issues facing target communities. The analysis also yielded information on the particular vulnerability of women, who are largely responsible for maintaining family well-being, are often left behind by male family members who migrate in search of work, and who have insecure access to important resources such as land. This analysis is being used as the basis for the development of Community Action Plans which identify the priority actions to reduce vulnerability to climate change.

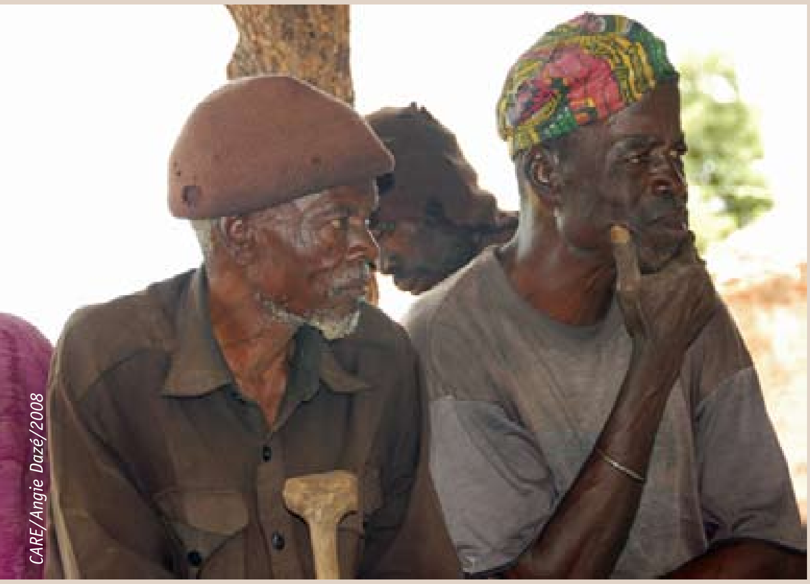

CARE/Angie Dazé/2008

Senior members of the community in Bowku Village, East Manprusi District, Ghana discuss how climate change is affecting their livelihoods and what action they are taking.

The project is combining bottom-up and top-down approaches by strengthening communities' capacity to communicate their needs and priorities to decision-makers while also working with District officials to promote participatory planning processes. The project is placing particular focus on ensuring that women play a leading role in local governance by promoting their engagement in Area Councils and community-based organizations, and by strengthening their capacity to advocate for women's rights.

The expected outcomes of the project are District Plans that incorporate the climate change adaptation priorities of vulnerable people, and increased capacity to adopt a participatory approach to local governance. Promoting district-level action to reduce vulnerability, while addressing some of the systemic inequalities that increase vulnerability of women, will build adaptive capacity to address future climate impacts.

The Community Land Use Responses to Climate Change (CLURCC) Project is implemented with the support of CIDA's Canadian Partnership Branch.

Household/Individual Level

Despite the global nature of the climate change challenge, its impacts are locally-specific. The CVCA process is designed to build understanding of local impacts and of existing adaptive capacity. The following table presents examples of interventions that can contribute to building adaptive capacity of vulnerable individuals and households.

	CBA Actions<br>*Household/Individual Level*
Resilient Livelihoods	• Promoting climate-resilient agricultural practices<br>• Supporting diversification of livelihoods, including non-agricultural livelihoods strategies<br>• Building capacity to analyze risks<br>• Promoting savings and building capacity to plan for risk management
Disaster Risk Reduction	• Establishing food and seed banks in places safe from hazards<br>• Improving shelter to withstand hazards<br>• Strengthening access to early warnings<br>• Facilitating evacuation planning<br>• Protection of assets
Capacity Development	• Strengthening social protection schemes<br>• Facilitating access to financial services<br>• Building knowledge and skills on adaptation strategies<br>• Facilitating access to climate information
Addressing Underlying Causes of Vulnerability	• Empowerment of women and other marginalized groups<br>• Promoting equitable division of labour within households<br>• Advocacy on rights to livelihoods resources

Adaptation in Action: Food security in a changing climate in Tajikistan

To better understand the complexity of climate change impacts in mountainous Tajikistan, CARE used a participatory process to determine how climate-related risks were affecting residents' well-being. The major changes observed by surveyed households included increasing snow pack, a shifting and lengthening of the winter season, and increasingly erratic rainfall. All of these observations are consistent with the meteorological data for the area, providing an excellent opening to introduce the topic of climate change to target communities.

Armed with a clear understanding of climate impacts on household livelihoods, the project identified those household-level adaptation strategies most likely to reduce the impact of climate-related shocks and stresses. When designing adaptation strategies, we focused on women, because of their vital contribution to family well-being, and their greater vulnerability.

Cold frames were distributed to especially vulnerable households in the target communities. These simple wood and glass frame structures act as small-scale greenhouses for growing herbs and vegetables. They are ideal for parsley, basilica, green onions, radishes, carrots, peppers, tomatoes, cucumbers, cabbages and turnips—even strawberries. By sheltering seedlings from inclement weather, cold frames help community members start seedlings earlier in the spring and increase the growing season. Some households are successfully growing cold-hardy vegetables such as greens all year round, achieving up to four harvests per year. The project also offered training and technical support in food preservation. The result is increased food security for vulnerable households during the difficult winter season.

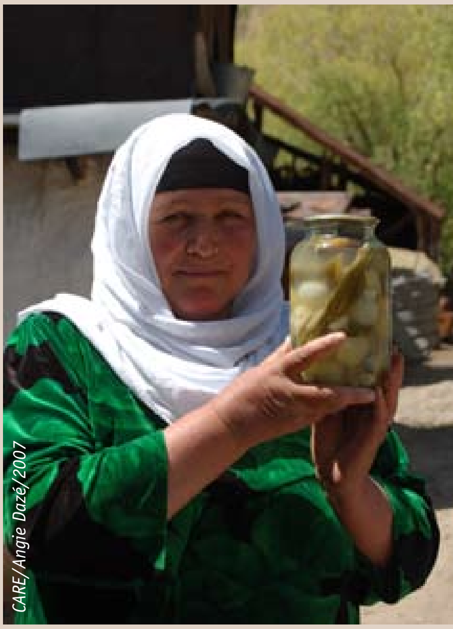

In Panjhok Village in central Tajikistan, a woman holds up a jar of vegetables, preserved to provide some food variety during the long winter season.

CARE is grateful to the Canadian International Development Agency (CIDA) for supporting The Adaptation to Climate Change in Tajikistan (ACCT) Project.

FOR MORE INFO

The Adaptation Learning Mechanism is a collaborative knowledge-sharing platform with a wide range of resources including country profiles, case studies and lessons learned. http://www.adaptationlearning.net/

WeADAPT is an online platform that offers a range of innovative tools to help users access, share and synthesize knowledge on adaptation. http://www.weadapt.org/

The Linking Climate Adaptation Network is an online community of practitioners and researchers on climate change adaptation: http://community.eldis.org/.599266eb/

The Nairobi Work Programme on impacts, vulnerability and adaptation to climate change has developed a compendium on methods and tools as well as an adaptation practices interface:
http://unfccc.int/adaptation/sbsta_agenda_item_adaptation/items/3633.php

The International Institute for Sustainable Development (IISD) has helpful tools and policy analysis on climate change: http://www.iisd.org/climate/vulnerability/

The Institute of Development Studies (IDS) website includes useful briefs and analysis on climate change adaptation issues: http://www.ids.ac.uk/go/browse-by-subject/climate-change

International Institute for Environment and Development (IIED) generates commentary and analysis on climate change: http://www.iied.org/CC/index.html

The Red Cross/Red Crescent Climate Centre has produced a useful guide for helping communities to prepare for climate change: http://www.climatecentre.org/index.php?page=news_ext&pub_id=85&type=4&view=more

Further Research

Participatory research is valuable for gathering information and understanding a range of perceptions at a local level, and for designing local level interventions that may help hundreds of people. However, it is time-consuming, and can't easily be generalized to other locations. Participatory research alone is seldom sufficient to influence national policies or convince decision-makers to change things that can affect millions of people; the results can be seen as "just stories" or anecdotes.

In order to broaden our understanding of different situations, and to strengthen our ability to influence policies and programs more widely, participatory research can form the basis for design of large-scale, survey-based studies. Information and perceptions gained by using the guiding questions and tools outlined above can help us sharpen our thinking, and to identify more specific questions that can be used in surveys covering many people in a large number of locations. This can improve our understanding of the various ways in which climate change may affect different parts of a country. Such broader perspectives can be valuable as the basis for influencing a range of national policies and practices impacting millions of people, such as agricultural research and development priorities, standards for surface water utilization, or development of infrastructure for densely populated areas.

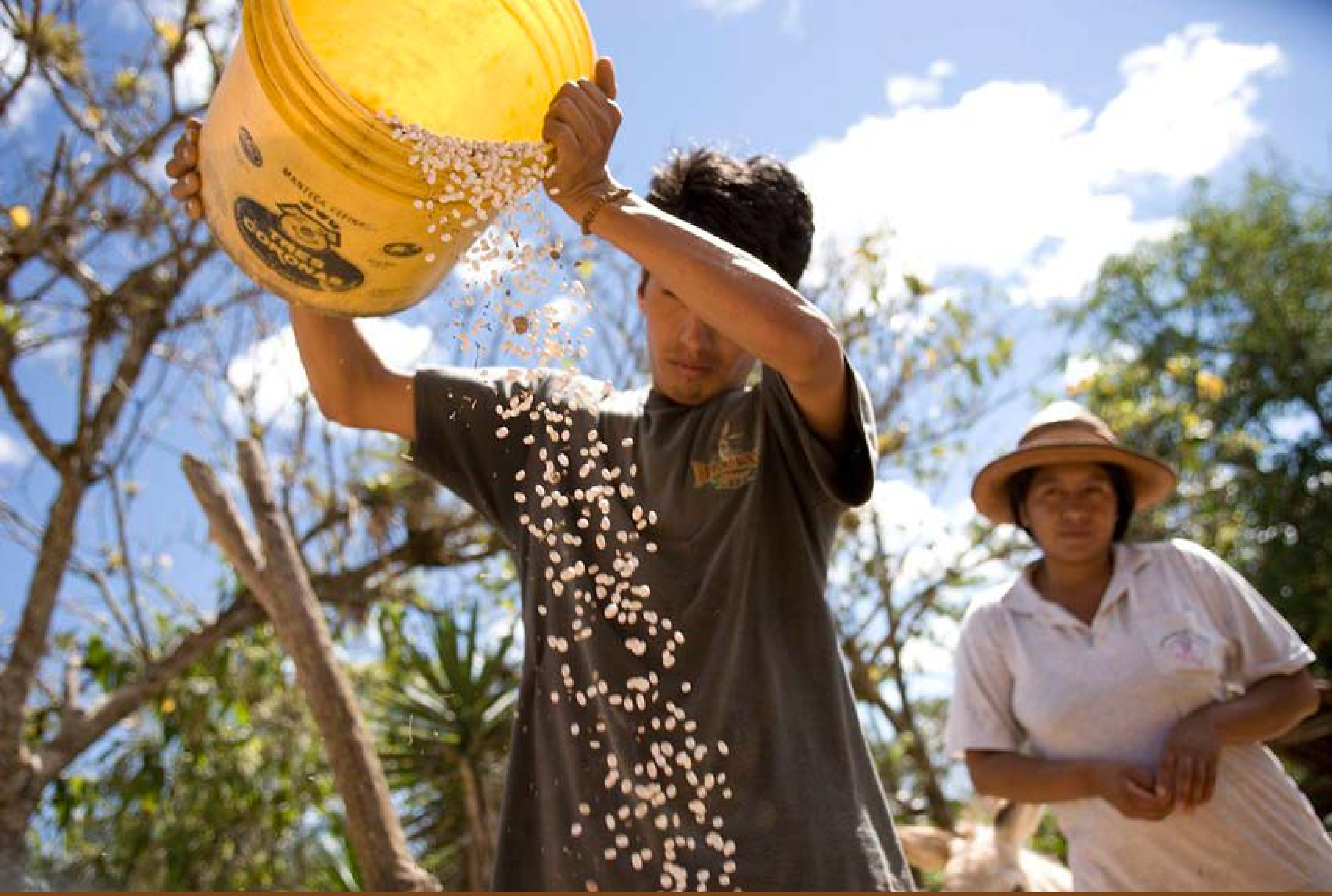

Field Guides for Participatory Tools

Before Going to the Community

Plan carefully

- Acquire background information before going to the field. Be aware of community or group history, past or present conflicts and power dynamics which may be important in selecting focus groups or in facilitating dialogue.
- Prepare the agenda for the community visits. The agenda should ensure that participants are able to move at their own pace, but that the required ground will be covered in the available time.
- Find out about literacy levels in advance if possible to ensure that you plan exercises accordingly.
- Don't forget to allow time for clarification, questions and answers, discussion and 'learning moments'.
- Keep in mind that community members are very busy, so visits should be kept short and be spaced out over time as much as possible so as not to take too much time away from their regular activities.
- Plan to provide refreshments when appropriate.
- Decide on focus groups.
- Ensure that facilitators are functional in local dialects.

Get support of community leaders

- Explain purpose of fieldwork and get their permission to work in the community.
- It may be helpful to have a preparatory meeting bringing together local stakeholders, including community leaders, local government representatives, CBOs, and other local organizations to explain the approach and its benefits and to plan the timing of the community visits.
- Review the agenda with stakeholders to clarify objectives, how much time will be needed, and where the discussions will take place (ensure that this is an appropriate venue which is accessible and comfortable for women or other less mobile community members).
- Agree on focus groups. If enough facilitators are available, it may be helpful to have concurrent sessions in the same community to allow participants in different groups to speak freely without being concerned about being heard by other groups.
- Decide how information on focus group discussions will be communicated to participants.
- Agree who will introduce the facilitators to the communities.

Be prepared

- Ensure the objectives of the community visits are agreed among all members of the analytical team.
- All facilitators must have a good grasp on the tools and analytical methodology. It may be helpful to practice facilitating the tools before going to the communities.
- If you are working as a facilitator team, decide who will actively facilitate which parts of the agenda, and who will take notes.
- The facilitation team should include both men and women, and should be trained in gender-sensitive facilitation. In some contexts it is very important to have female facilitators work with women's groups to increase comfort.
- Agree with co-facilitators on how concepts such as hazard, livelihood resources, etc. will be described in local languages. Note that the concept of climate change may be difficult to explain. Community members may be more comfortable talking about seasons, weather, the environment, etc.

Be ready to handle conflict.

- The process can draw out issues of inequality that need to be addressed in order to reduce vulnerability. With these issues, facilitators must tread carefully, as there are generally established levels of power and influence within communities, or between communities and other groups.
- Having a grasp on conflict resolution techniques will help facilitators manage the process should any conflicts arise.
- Engaging a wide range of stakeholders in the gathering and analysis of information can assist in mitigating conflicts.

Ensure you have the materials you need.

- These may include:
  - Flipchart paper
  - Thick-tipped markers in a variety of colours
  - Coloured paper
  - Masking tape
  - Local materials such as stones, sticks, seeds, etc.
  - Recording device
  - Camera to document the process (ensure that this is culturally appropriate)
  - Notebook and clipboard
  - Snacks/lunch/water (depending on how much time the meeting will take, and where it will take place)

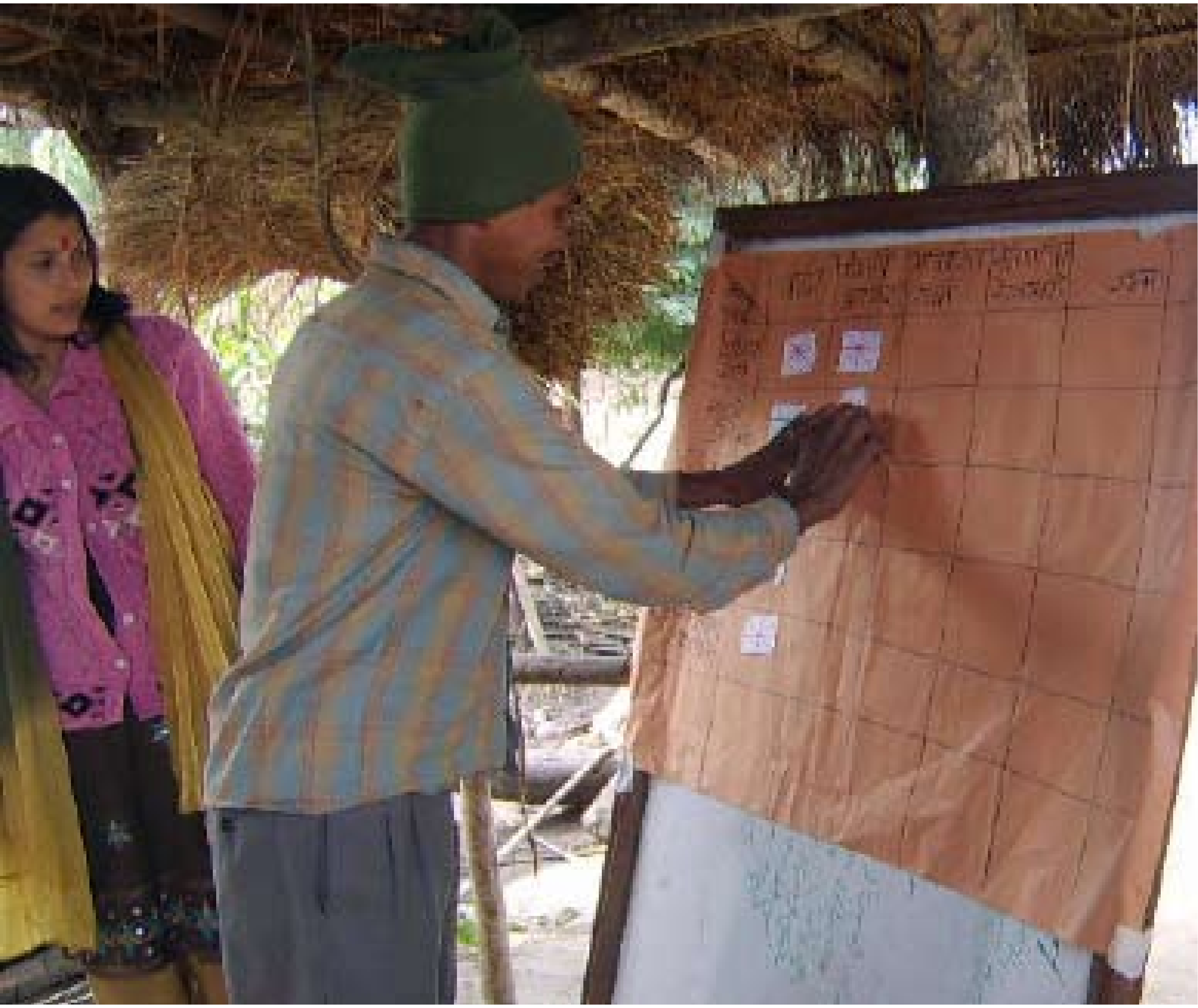

A participant in Nepal fixes the group ranking on the Vulnerability Matrix as the facilitator looks on.

During the Focus Group Discussions

Manage expectations

- It is important to manage expectations during the fieldwork. Communities have often been "assessed" many times for different projects, and may have expectations that the fieldwork will lead to a project or program.
- Facilitators should be aware of this, both because it may influence the issues that are raised in the discussions, and to ensure that expectations are not being raised for follow-up projects.

Create and maintain a trusting and 'safe' space

- Allow a trusted community member or local representative to introduce the team.
- Be gracious and welcoming.
- Allow everyone to introduce themselves.
- Ask permission to take photographs or video, and refrain if participants are uncomfortable with it.
- Provide refreshments if appropriate.
- Value participants' knowledge and experience.
- Interrupt any "attacks".
- Admit to and correct your errors.
- Be impartial.
- Allow time for participants to ask questions.

Animate and balance participation

- Ensure that the venue is conducive to participation.
- Develop ground rules with the participants.
- Explain the process and ensure that all understand instructions and questions.
- Support those that are timid, and gently silence those that take the floor too much or consider themselves "experts".
- Find ways to allow people to drive the process (e.g. building the map themselves, marking symbols on the matrix).
- Allow participants to raise issues, but keep the process on track. Ensure that you are moving quickly enough to cover the necessary ground in the time allocated.
- Probe for more information if the discussion is lagging, but try not to lead participants.

Finish gracefully

- Explain what the next steps are.
- Schedule a time to return to validate the analysis.
- Thank the group for their participation, and give an opportunity to ask questions.
- If the participants would like to keep the products of the focus group discussions (e.g. hazard maps), make a copy and leave the original behind.

FIELD GUIDE 2: **Hazard Mapping**

Objectives

- To become familiar with the community, and to see how the place is perceived by different groups within the community
- To identify important livelihoods resources in the community, and who has access and control over them
- To identify areas and resources at risk from climate hazards
- To analyze changes in hazards and planning for risk reduction

How to Facilitate

This activity should take approximately 1 hour and 30 minutes including discussion: 45 minutes for the map, and 45 for discussion.

1. Explain to the participants that you would like to build a map of their community.
2. Choose a suitable place (ground, floor, paper) and medium (sticks, stones, seeds, pencils, chalk) for the map. If the map is made on the ground or floor, the note taker will then have to copy the map on a flipchart or in his/her notebook. A photo can also be helpful.
3. First, build the community map. Ask the community members to identify a landmark in the community.
4. Put a mark or a stone to stand for the landmark. NOTE: The facilitator should help the participants get started but let them draw the map by themselves.
5. Ask the community members to draw the boundaries of the community.
6. Ask community members to draw the location of settled areas, critical facilities and resources in the community. This should include houses (the map doesn't need to show every house, but the general area where houses are located), facilities such as churches/mosques, health clinics, schools, and resources such as forested areas and water bodies.
7. When the community members have agreed that the map is representative of their community, begin the second step: identifying the hazards.
8. Ask the community members to identify the areas at risk from different types of hazards. These should include:
    - Natural disasters
    - Health crises such as HIV/AIDS or malaria
    - Socio-political issues such as conflict or redistribution of land, etc.

Hazards that are mentioned that are not location-specific should be noted on the report.

Learning and Discussion

When the map is complete, ask the group members the following questions:

- Who has access to the resources shown on the map? Who controls this access?
- What are the impacts of the hazards identified?
- Are the hazards different now than they were 10/20/30 years ago (depending on age of participants)? How?
- Are there places in the community that are safe from the hazards?
- Are these safe places used to protect from hazards (e.g. to store food and inputs, or to shelter livestock)?
- Who are the members of the community who are most at risk from the different hazards? Why?
- How do people in the community currently cope with the impacts of the specific hazards identified? Are the current coping strategies working? Are they sustainable?

Communicating Climate Change

During the discussion, note any observations by communities that may be in line with the meteorological data that is available for the region, and communicate this information in order to validate their observations. This can provide an opening to present the predicted future trends for the particular hazards that have been identified.

The note taker should carefully transcribe the key points of the discussion.

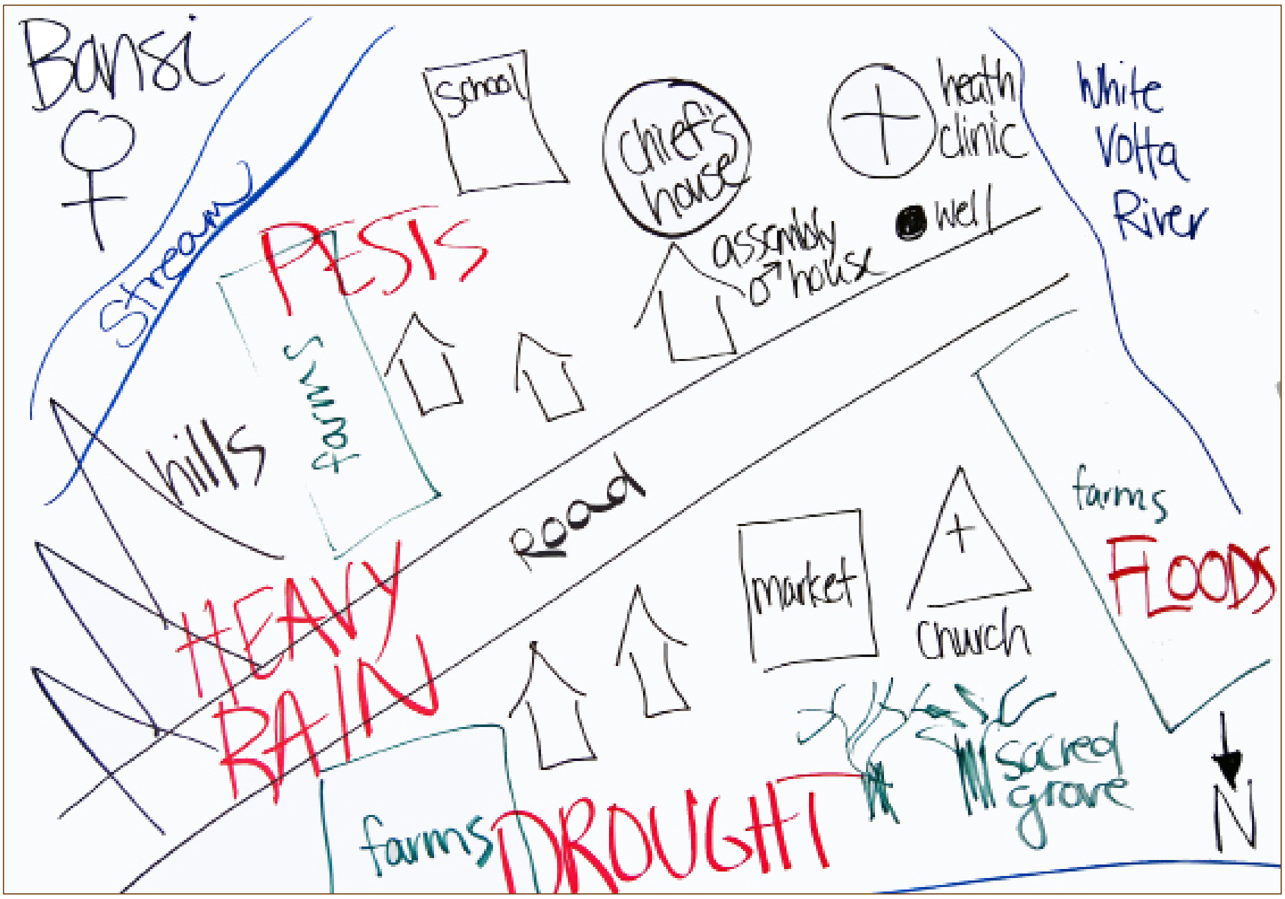

CARE/Angie Dazé/2007

A Hazard Map for Bansi Village in Northern Ghana showing the key resources in the village and the hazards affecting them.

ADAPTED FROM:

Community-Based Disaster Risk Management Field Practitioners' Handbook, Imelda Abarquez and Zubair Murshed, Asian Disaster Preparedness Center (2004). http://www.adpc.net/PDR-SEA/publications/12Handbk.pdf

Make that change: community-based disaster management, International Federation of Red Cross and Red Crescent Societies (undated). http://www.proventionconsortium.org/themes/default/pdfs/CRA/IFRC2003_meth.pdf

FIELD GUIDE 3: **Seasonal Calendar**

Objectives

- To identify periods of stress, hazards, diseases, hunger, debt, vulnerability, etc.
- To understand livelihoods and coping strategies
- To analyze changes in seasonal activities
- To evaluate use of climate information for planning

How to Facilitate

This activity should take approximately 1 hour and 15 minutes including discussion: 30 minutes for the calendar, and 45 minutes for the discussion.

1. Use the ground or large sheets of paper. Mark off the months of the year on the horizontal axis.
2. Explain to the participants that you would like to develop a calendar to show key events and activities that occur during the year.
3. Ask people to list seasons, events, conditions, etc., and arrange these along the vertical axis. The list should include:
   - Holidays and festivals
   - Planting and harvest seasons
   - Periods of food scarcity
   - Times of migration
   - Timing of hazards/disasters such as cyclones, droughts and floods
   - When common seasonal illnesses occur
   - Etc.
4. When the key events have been listed, plot the timing of them in the table based on agreement among the participants. The note taker should note any events for which the group has difficulty deciding on timing.

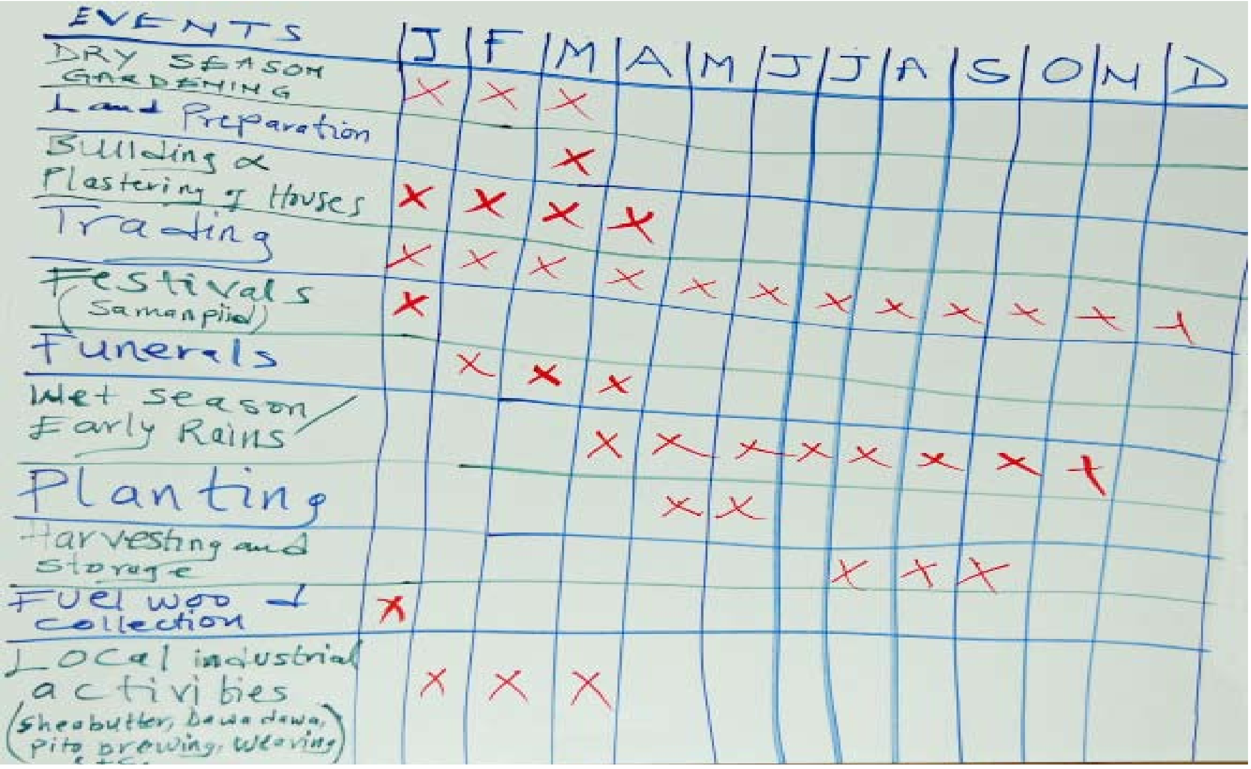

CARE/Angie Dazé/2007

The result of a Seasonal Calendar exercise with a group of women in Zaago Village, Upper East Region in Ghana.

Learning and Discussion

When the calendar is complete, ask the group members the following questions:

- What are the most important livelihoods strategies employed at different points of the year?
- What are current strategies to cope during the difficult times? Are they working?
- Are there any differences in the timing of seasons and events as compared to 10/20/30 years ago?
- Have livelihoods/coping strategies changed based on the changing seasons or events?
- How are decisions made on timing of livelihoods strategies?

Communicating Climate Change

When discussing coping strategies and changes, there may be opportunities to examine whether existing coping strategies are working in the context of the changing environment and/or to identify innovative strategies that have emerged as a result of the changes. It can provide an opening to discuss the need for new strategies in the context of climate change, and to introduce the concept of adaptation.

The note taker should carefully transcribe the key points of the discussion.

ADAPTED FROM:

Community-Based Disaster Risk Management Field Practitioners' Handbook, Imelda Abarquez and Zubair Murshed, Asian Disaster Preparedness Center (2004). http://www.adpc.net/PDR-SEA/publications/12Handbk.pdf

Make that change: community-based disaster management, International Federation of Red Cross and Red Crescent Societies (undated). http://www.proventionconsortium.org/themes/default/pdfs/CRA/IFRC2003_meth.pdf

Objectives

- To get an insight into past hazards, changes in their nature, intensity and behaviour
- To make people aware of trends and changes over time
- To evaluate extent of risk analysis, planning and investment for the future

How to Facilitate

This activity should take approximately 1 hour and 15 minutes including discussion: 45 minutes for the timeline, and 30 minutes for the discussion.

1. The facilitator should consult with the group to decide on a table format or a graphical line format.
2. Ask people if they can recall major events in the community such as:
   - major hazards and their effects
   - changes in land use (crops, forest cover, houses etc.)
   - changes in land tenure
   - changes in food security and nutrition
   - changes in administration and organization
   - major political events
3. The facilitator can write the stories down on a blackboard or large sheets of paper in chronological order.
4. Periodically run back through the events already reported to prompt recall and help the informant to fill in gaps. Just concentrate on key events.

NOTE: It must be kept in mind that there may be a bias in the timeline as events in recent memory are more likely to be noted.

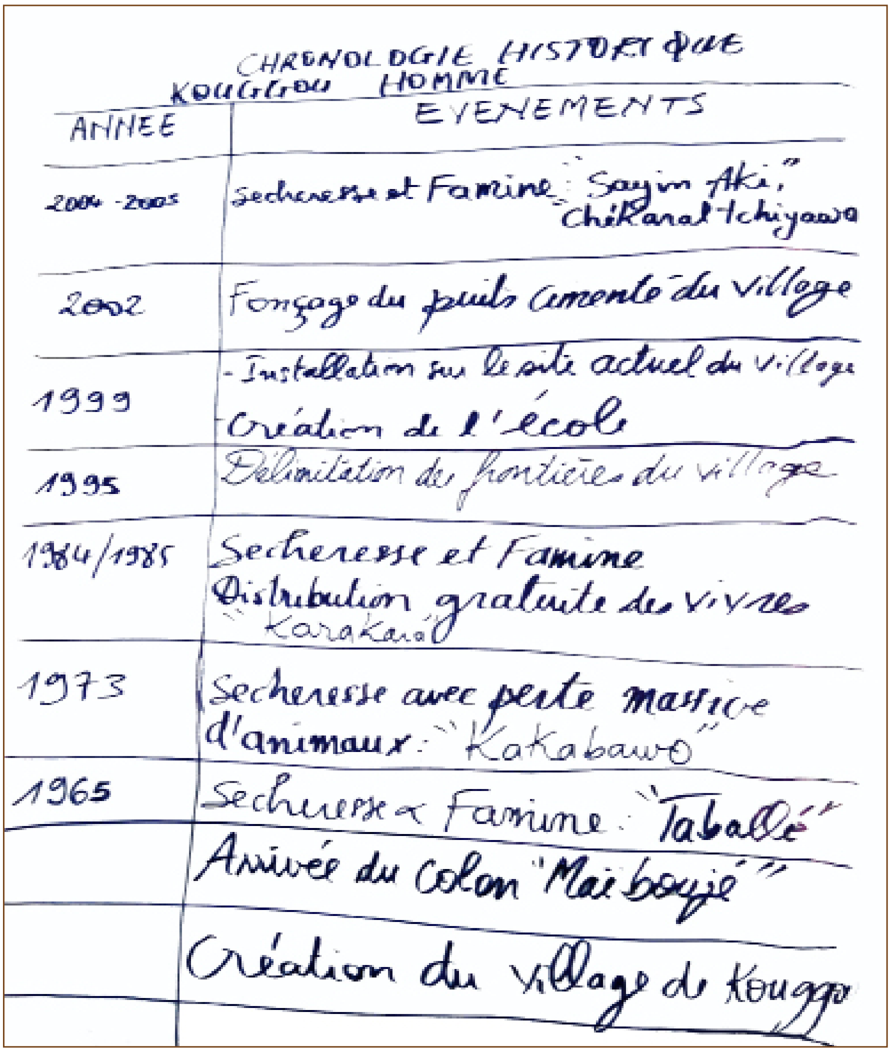

CARE/Angie Dazé/2007

Historical timeline for Kouggou Village in the Department of Dakoro in Niger. The timeline was created by a group of men, and shows events such as famines, the construction of a well, and the delineation of the village borders.

Discussion Questions

When the timeline is complete, ask the group members the following questions:

- Are there any trends or changes in the frequency of events over time?
- What are current strategies to cope during the difficult events? Are they working?
- Have coping strategies changed based on the changing frequency of events?
- What events do you expect will occur in the future? When?
- Does this perception of future events affect your plans for the future?

Communicating Climate Change

Discussing trends or changes in the frequency of events is an excellent opportunity to validate community observations that are in line with climate data. Focusing the discussion on the future can help in understanding community aspirations and extent of planning for the future. It also presents an opening to facilitate communication of available information on predicted future trends.

The note taker should carefully transcribe the key points of the discussion.

ADAPTED FROM:

Community-Based Disaster Risk Management Field Practitioners' Handbook, Imelda Abarquez and Zubair Murshed, Asian Disaster Preparedness Center (2004). http://www.adpc.net/PDR-SEA/publications/12Handbk.pdf

Objectives

- To determine the hazards that have the most serious impact on important livelihoods resources
- To determine which livelihoods resources are most vulnerable
- To identify coping strategies currently used to address the hazards identified

How to Facilitate

This activity should take approximately 1 hour and 30 minutes including discussion: 45 minutes for the matrix, and 45 minutes for the discussion.

1. Prepare a matrix in advance. This can be done on the ground or on flip chart paper.
2. Ask the group to identify their most important livelihoods resources. These do not have to be resources that they currently have, but those that they consider to be most important in achieving well-being. They may create a long list of resources. You may want to organize the list based on the different categories of resources – human, social, physical, natural and financial.
3. Ask the group to identify the four resources that they consider to be MOST important in achieving well-being. List these priority resources down the left side of the matrix on the vertical. Use symbols if this will help participants to better understand.
4. Then ask the group to identify the greatest hazards to their livelihoods. Hazards may be natural or man-made. Do not limit the discussion to only climate-related hazards, but you may want to prompt the group if they are not identifying environmental hazards.

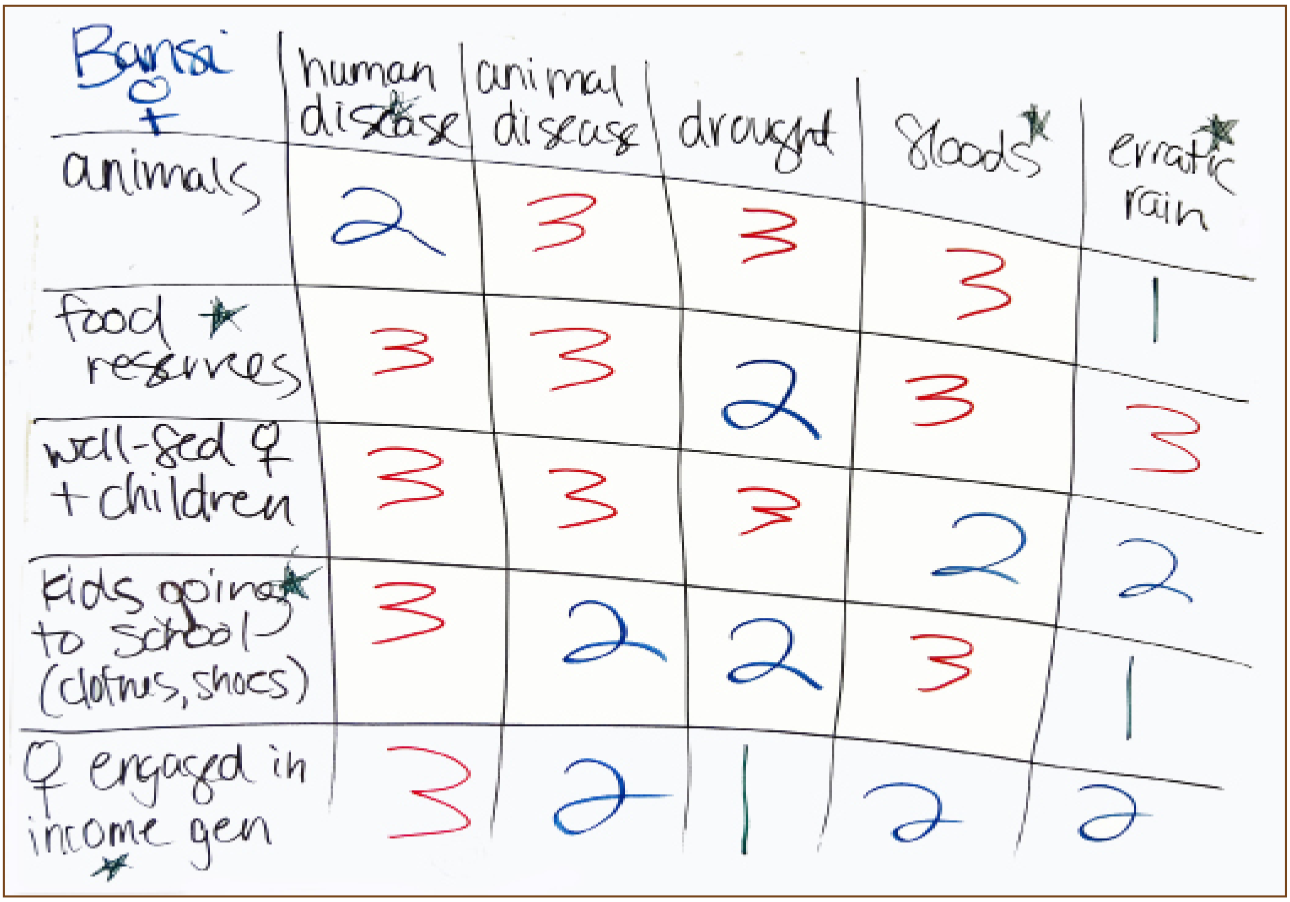

CARE/Angie Dazé/2007

An example of a Vulnerability Matrix from a group of women in Bansi Village in Bawku District in Northern Ghana.

NOTE: It is important to be specific in the hazards, and to ensure that the issues identified are actually hazards. Participants may identify conditions such as "food insecurity" as hazards. It is up to the facilitator to ask the group to break down these conditions to determine if they are caused by hazards (e.g. food insecurity may be the result of a drought, which is a hazard). Similarly, some groups may identify scarcity of resources, such as "lack of money", as a hazard. In this case, it should be determined whether the lack of a resource is the result of a hazard, or in some cases, whether the resource should be added to the list of priority resources identified in the previous step.

5. The four most important hazards should be listed horizontally across the top of the matrix, again using symbols if necessary.

6. Ask the community to decide on a scoring system for the hazards against the livelihoods resources, identifying significant, medium, low and no hazard. The scoring system should be as follows:

   3 = significant impact on the resource

   2 = medium impact on the resource

   1 = low impact on the resource

   0 = no impact on the resource

   You can use stones, symbols or different colours of markers (e.g. red = significant risk to resource, orange = medium risk, green = low risk, blue = no risk). Ensure that all members of the group understand the scoring system.

7. Ask the participants to decide on the degree of impact that each of the hazards has on each of the resources. This will involve coming to consensus as a group. The note taker should note key points of discussion that lead to the scores assigned, and any disagreements on the scores.

Discussion Questions

When the matrix is complete, ask the group members the following questions:

- What coping strategies are currently used to deal with the hazards identified? Are they working?
- Are there different strategies that you would like to adopt which would reduce the impact of hazards on your livelihoods?
- What resources do you have that would help you to adopt these new strategies?
- What are the constraints to adopting these new strategies?

The note taker should carefully transcribe the key points of the discussion.

FIELD GUIDE 6: **Venn Diagram**

Objectives

- To understand which institutions are most important to communities
- To analyze engagement of different groups in local planning processes
- To evaluate access to services and availability of social safety nets

How to facilitate

This activity should take approximately 1 hour and 30 minutes including discussion: 1 hour for the diagram, and 30 minutes for the discussion.

1. There are a number of different ways to do the Venn Diagram. You can draw and write with a stick on a soft ground or you can work on paper. If you decide to use paper, people should first use a pencil in order to be able to make changes. Another option is to cut circles of different sizes from coloured paper and let participants decide which size of circle represents the different institutions.
2. If people find it difficult to understand this tool, it may be helpful to draw a simple example for them.
3. Ask the participants which organisations/institutions/groups are found in the village and which other ones from elsewhere are working with them. Encourage them to also think about informal groups and community-based organizations.
4. Write down all the institutions that are mentioned and give each organisation a symbol which everybody can understand.
5. Ask the participants to draw a big circle in the centre of the paper or on the ground that represents them.
6. Ask them to discuss for each organization how important it is for them. The most important ones are then drawn as a big circle and the less important ones as smaller circles. Ask the participants to compare the sizes of the circles and to adjust them so that the sizes of the circles represent the relative importance of the institution, organization or group.
7. Every organization/group should be marked with the name or symbol.
8. Ask them to discuss in which way they benefit from the different organizations.
9. The note taker should transcribe the discussion, noting why the different organizations are considered important or less important.
10. Ask them to show the degree of contact/co-operation between themselves and those institutions by distance between the circles. Institutions which they do not have much contact with should be far away from their own big circle. Institutions that are in close contact with the participants and with whom they co-operate most, should be inside their own circle.

Discussion Questions

When the diagram is complete, ask the group members the following questions:

- Are any of the organizations shown only open to membership by men or women? Do any only offer services to men or women?
- Are there any other groups that are excluded from membership or service for the organizations identified?
- Do any of the organizations offer support in times of crisis?
- How do you receive information from the different organizations?
- How do you communicate information to the different organizations?

The note taker should carefully transcribe the key points of the discussion.

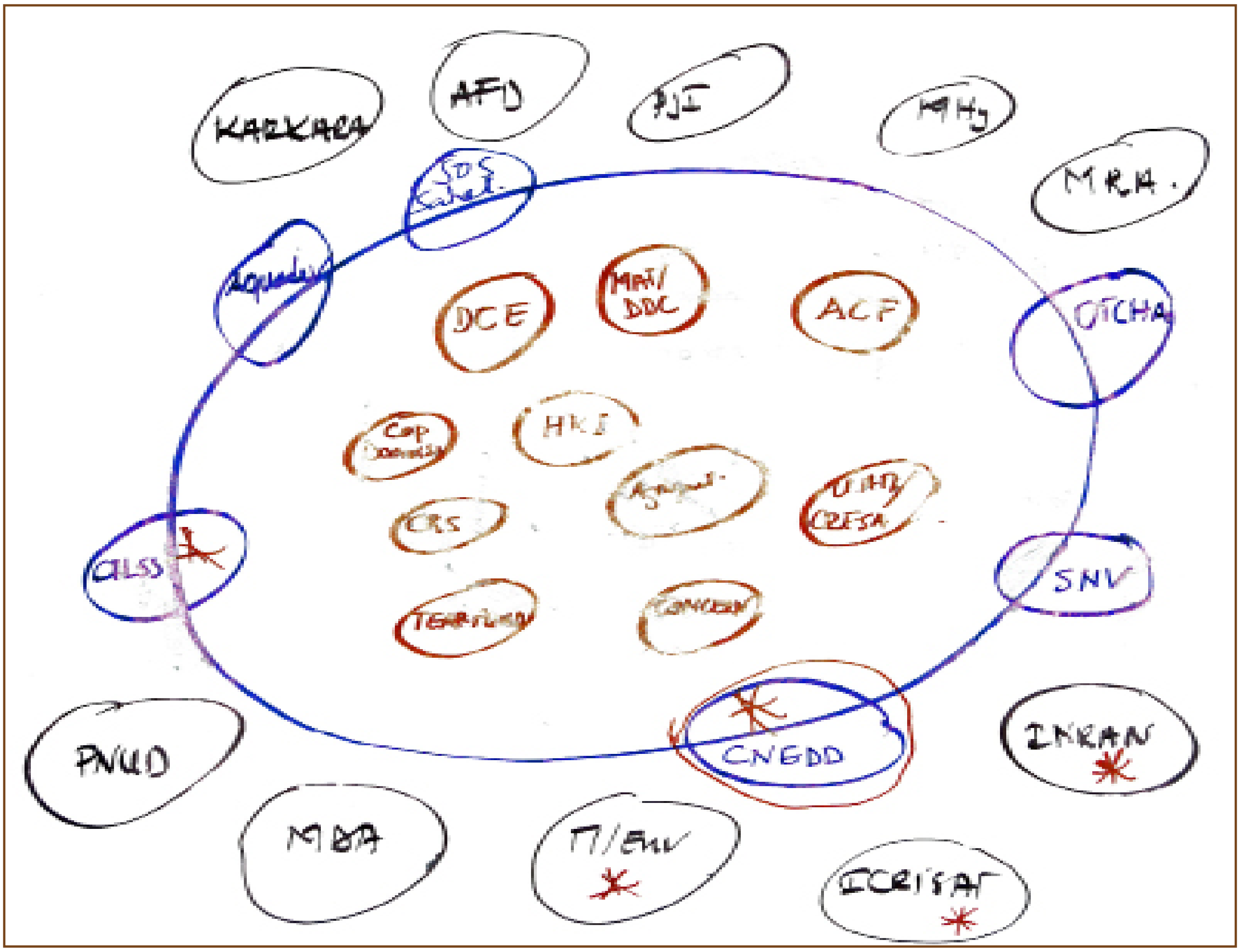

CARE/Angie Dazé/2007

A Venn Diagram for Niger created as part of a CVCA training exercise. The example shows national and international NGOs as well as governmental organizations that are relevant from a climate change perspective.

ADAPTED FROM:

PRA Toolbox, Food and Agriculture Organization of the United Nations (FAO). http://www.fao.org/docrep/003/x5996e/x5996e06.htm

Make that change: community-based disaster management, International Federation of Red Cross and Red Crescent Societies (undated). http://www.proventionconsortium.org/themes/default/pdfs/CRA/IFRC2003_meth.pdf

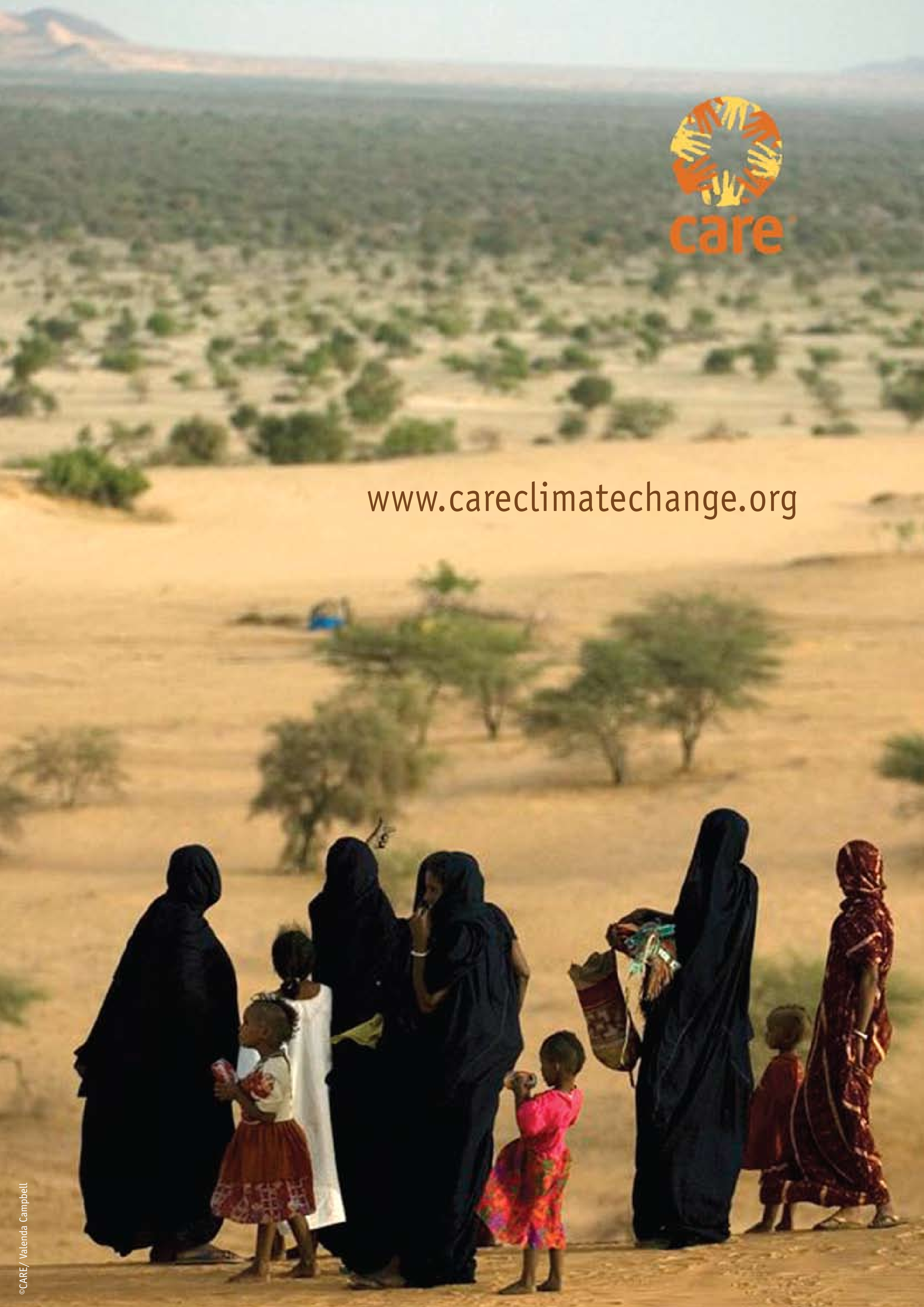

www.careclimatechange.org

©CARE/ Valenda Campbell
