# Supplementary material for: Systematic Review of Methods in Low-Consensus Fields: Supporting Commensuration through `Construct-Centered Methods Aggregation’ in the Case of Climate Change Vulnerability Research
Source: PLoS One. 2016 Feb 22;11(2):e0149071. doi: 10.1371/journal.pone.0149071 (PMC4762661; doi:10.1371/journal.pone.0149071)
Supplement: S2 File — Source articles and bibliography. (ZIP) [file pone.0149071.s002.zip › data requirements/Deressa et al.pdf]

INTERNATIONAL FOOD
POLICY RESEARCH INSTITUTE
*sustainable solutions for ending hunger and poverty*
IFPRI®
Supported by the CGIAR

**IFPRI Discussion Paper 00935**

November 2009

# Assessing Household Vulnerability To Climate Change

## The Case Of Farmers In The Nile Basin Of Ethiopia

**Temesgen T. Deressa**

**Rashid M. Hassan**

**Claudia Ringler**

Environment and Production Technology Division

## INTERNATIONAL FOOD POLICY RESEARCH INSTITUTE

The International Food Policy Research Institute (IFPRI) was established in 1975. IFPRI is one of 15 agricultural research centers that receive principal funding from governments, private foundations, and international and regional organizations, most of which are members of the Consultative Group on International Agricultural Research (CGIAR).

## FINANCIAL CONTRIBUTORS AND PARTNERS

IFPRI's research, capacity strengthening, and communications work is made possible by its financial contributors and partners. IFPRI receives its principal funding from governments, private foundations, and international and regional organizations, most of which are members of the Consultative Group on International Agricultural Research (CGIAR). IFPRI gratefully acknowledges the generous unrestricted funding from Australia, Canada, China, Finland, France, Germany, India, Ireland, Italy, Japan, Netherlands, Norway, South Africa, Sweden, Switzerland, United Kingdom, United States, and World Bank.

## AUTHORS

**Temesgen T. Deressa, University of Pretoria**
PhD Candidate, Centre for Environmental Economics and Policy for Africa
ttderessa@yahoo.com

**Rashid M. Hassan, University of Pretoria**
Director, Centre for Environmental Economics and Policy for Africa

**Claudia Ringler, International Food Policy Research Institute**
Senior Research Fellow, Environment and Production Technology Division

**Notices**

[1] Effective January 2007, the Discussion Paper series within each division and the Director General's Office of IFPRI were merged into one IFPRI–wide Discussion Paper series. The new series begins with number 00689, reflecting the prior publication of 688 discussion papers within the dispersed series. The earlier series are available on IFPRI's website at www.ifpri.org/pubs/otherpubs.htm#dp.

[2] IFPRI Discussion Papers contain preliminary material and research results, and have been peer reviewed by at least two reviewers—internal and/or external. They are circulated in order to stimulate discussion and critical comment.

Copyright 2009 International Food Policy Research Institute. All rights reserved. Sections of this document may be reproduced for noncommercial and not-for-profit purposes without the express written permission of, but with acknowledgment to, the International Food Policy Research Institute. For permission to republish, contact ifpri-copyright@cgiar.org.

# Contents

Acknowledgments v
Abstract vi
Abbreviations and Acronyms vii
1. Introduction 1
2. Review of Literature 2
3. Empirical Model, Study Area and Data 4
4. Results and Discussion 8
5. Conclusions and Policy Implications 15
References 16

## List of Tables


1. Climatic zones and their physical characteristics 5
2. Surveyed districts and peasant associations 7
3. Major shocks encountered by surveyed farmers 8
4. Effects of shocks by surveyed farmers 8
5. Coping strategies by surveyed farmers 8
6. Description of the utilized dependent and independent variables 10
7. Sensitivity analysis at the agro-ecological level: percent of farmers in each agro ecology 13
8. Sensitivity analysis at the regional-state level: percent of farmers in each region 14


## List of Figures


1: Nile Basin of Ethiopia with its agro-ecological classifications and survey districts 6

2: Coping strategies used to deal with major environmental shocks 9

3 : Vulnerability (income at 2 USD per day or 6570 Ethiopian Birr per year) plotted against Ln (income) 11

4: ( Vulnerability (income at 1.5 USD per day or 4928 Ethiopian Birr per year) plotted against Ln income) 12

5: Vulnerability (income at 1.25 USD per day or 4471 Ethiopian Birr per year) plotted against Ln (income) 12

6: Vulnerability (income at 0.3 USD per day or 900 Ethiopian Birr per year) plotted against Ln (income) 13

# ACKNOWLEDGMENTS

This work was supported by the Federal Ministry for Economic Cooperation and Development (Germany) under the project “Food and Water Security Under Global Change: Developing Adaptive Capacity with a Focus on Rural Africa,” which forms part of the Consultative Group on International Agricultural Research (CGIAR)’s Challenge Program on Water and Food (CPWF). Special thanks go to Tekie Alemu and Mahmud Yesuf for creating an ideal environment for this research, especially during data collection. The views expressed here are solely those of the authors.

## ABSTRACT

This study measures the vulnerability of farmers to climatic extremes such as droughts, floods and hailstorms, by employing the "vulnerability as expected poverty" approach. This approach is based on estimating the probability that a given shock or set of shocks will move household consumption below a given minimum level (such as the consumption poverty line) or force the consumption level to stay below the given minimum if it is already below this level. The utilized data come from a household survey of farmers performed during the 2004/2005 production year in the Nile Basin of Ethiopia. The results show that the farmers' vulnerability is highly sensitive to their minimum daily requirement (poverty line). For instance, when the daily minimum income is fixed at 0.3 United States dollars (USD) per day, only 12.4 percent of farmers are vulnerable to climate extremes, whereas 99 percent of farmers are vulnerable when the minimum requirement is fixed at 2 USD per day. The results further indicate that farmers in kola agro-ecological zones (which are warm and semi-arid) are the most vulnerable to extreme climatic events. Policy-wise, these preliminary results indicate that, keeping other factors constant, increasing the incomes of farmers (with special emphasis on those in kola agro-ecological zones) and enabling them to meet their daily minimum requirements will reduce their vulnerability to climatic extremes.

Keywords: vulnerability to climate extremes, Nile Basin of Ethiopia, minimum daily income

## ABBREVIATIONS AND ACRONYMS

| | |
|---|---|
| GDP | gross domestic product |
| GNP | gross national product |
| USD | United States dollars |
| IPCC | Intergovernmental Panel on Climate Change |
| VEP | vulnerability as expected poverty |
| VEU | vulnerability as low expected utility |
| VER | vulnerability as uninsured exposure to risk |
| SNNP | Southern Nations Nationalities and Peoples |
| EDRI | Ethiopian Development Research Institute |

# 1. INTRODUCTION

Ethiopia has a population of 70 million, but remains one of the least developed countries in the world. Agriculture, which is the main sector of the Ethiopian economy, contributes about 52 percent of the country's gross domestic product (GDP), generates more than 85 percent of the foreign exchange earnings and employs about 80 percent of the population. The real per capita Gross National Product (GNP) is about 100 United States dollars (USD) in Ethiopia, and most residents find it hard to meet their daily basic needs. About 50 percent of the population lives under absolute poverty, and the average life expectancy in the country is about 43 years (CSA 2005).

The agricultural sector is dominated by small-scale mixed crop-and-livestock production with very low productivity. The major factors responsible for this low productivity include: reliance on obsolete farming techniques; soil degradation caused by overgrazing and deforestation; poor complementary services such as extension services, credit, markets and infrastructure; and climatic factors such as drought and flood (Deressa 2007). These factors reduce the farmers' adaptive capacity and/or increase their vulnerability to future changes, negatively affecting the performance of the already weak agricultural sector.

Despite the fact that Ethiopia has a long history of drought, studies have shown that the frequency and spatial coverage of droughts have increased over the past few decades (Lautze et al. 2003). Moreover, over the past 50 years, the average annual minimum and maximum temperatures across the country have increased by about 0.25oC and about 0.1oC, respectively, per decade, and precipitation has shown a decreasing trend throughout the country (NMSA 2001). This trend of increasing temperature, decreasing precipitation and increasingly frequent drought is predicted to continue in the tropics (which include Ethiopia) through the future (World Bank 2003; Mitchell and Tanner 2006; IPCC 2001). Thus, the country's agricultural sector should be considered vulnerable to future climate change.

Attempts have been made to analyze the vulnerability of Ethiopian farmers to climatic and non-climatic shocks in studies using panel datasets (Dercon 2004; Dercon et al. 2005; Skoufias and Quisumbing 2003; Dercon and Krishnan 2000), and policy options have been suggested to reduce vulnerability. The studies by Dercon (2004), Dercon et al. (2005) and Dercon and Krishnan (2000) used ex ante vulnerability assessment approaches to analyze the vulnerability of Ethiopian farmers by taking maximum of only 15 villages. The Skoufias and Quisumbing (2003) study used an ex post approach to analyze vulnerability in the same 15 villages. While these studies are informative and methodologically sound, their use of a relatively small data pool means that they are unlikely to accurately represent the vast agro-ecological and socio-economic diversity of the country. This represents an important limitation, since the results from these studies cannot be generalized to farming communities that do not share the same socio-economic and environmental attributes.

The present study addresses this knowledge gap by using a cross-sectional dataset collected from 162 villages in the Nile Basin of Ethiopia, representing diverse socio-economic and environmental settings. The paper is organized as follows. Section 2 reviews the literature on approaches to vulnerability assessment. Section 3 describes the empirical method employed in this study. Section 4 discusses the results, and Section 5 provides conclusions.

# 2. REVIEW OF LITERATURE

## 2.1. Definitions of Vulnerability

The term "vulnerability" has no universally accepted definition, largely because different disciplines use the term differently to explain their areas of concern. Studies on natural hazards and epidemiology define vulnerability as the degree to which an exposed unit is susceptible to being harmed by exposure to a perturbation or stress, in conjunction with its ability (or lack thereof) to cope, recover or fundamentally adapt (become a new system or go extinct) (Kasperson et al. 2001). In contrast, the poverty and development literature, which focuses on social, economic and political conditions, defines vulnerability as an aggregate measure of human welfare that integrates environmental, social, economic, and political exposure to a range of harmful perturbations (Bohle et al. 1994). According to Yamin et al. (2005), the disaster community defines vulnerability as conditions that are determined by physical, social, economic, and environmental factors or processes, and that increase the susceptibility of a community to the impact of a hazard. In the resilience community, vulnerability is defined as a loss of resilience (Franklin and Downing 2004).

Adger (1999) defines social vulnerability as the exposure of groups or individuals to stress as a result of social and environmental change, where "stress" refers to unexpected changes and disruptions to livelihoods. Reilly and Schimmelpfennig (1999) define vulnerability as a probability-weighted mean of damages and benefits, and give examples of crop yield vulnerability, farmer or farm sector vulnerability, regional sector vulnerability, regional economic vulnerability, and vulnerability to hunger. The Intergovernmental Panel on Climate Change (IPCC 2001) defines vulnerability to climate change as: "The degree to which a system is susceptible, or unable to cope with adverse effects of climate change, including climate variability and extremes, and vulnerability is a function of the character, magnitude and rate of climate variation to which a system is exposed, its sensitivity, and its adaptive capacity."

## 2.2. Approaches to Estimating Vulnerability

Two types of analytical methods for measuring vulnerability are discussed in this section, namely indicator and econometric approaches.

### *Indicator Approaches*

The indicator approaches are based on developing a wide range of indicators and selecting some of them through expert judgment (Kaly and Pratt 2000; Kaly et al. 1999), principal component analysis (Easter 1999; Cutter et al. 2003), or correlation with past disaster events (Brooks et al. 2005). Each of these selection procedures is used to choose the indicators that account for the largest proportion of vulnerability. The selected indicators may be used at the local (Adger 1999; Leon-Vasquez et al. 2003; Morrow 1999), national (O'Brien et al. 2004), regional (Leichenko and O'Brien, 2001; Vincent 2004) or global (Brooks et al. 2005; Moss et al. 2001) scales. According to Luers et al. (2003), the indicator approaches are valuable for monitoring trends and exploring conceptual frameworks. However, these approaches are limited by: 1) considerable subjectivity in the selection of variables and their relative weights, 2) the availability of data at various scales, and 3) the difficulty of testing or validating the different metrics.

### *Econometric Approaches*

The econometric methods, which use household-level socio-economic survey data to analyze the vulnerability levels of different social groups, include three assessments: vulnerability as expected poverty (VEP), vulnerability as low expected utility (VEU) and vulnerability as uninsured exposure to risk (VER) (Hoddinott and Quisumbing 2003). All of these methods construct measures of welfare loss attributed to shocks, but differ in that VEP and VEU measure the ex ante probability of a household's

consumption or utility falling below a given minimum level in the future due to current or past shocks, while VER measures ex post welfare loss due to shocks. The most commonly cited shocks resulting in welfare loss include climatic, economic, political, social, legal, crime and health shocks (Hoddinott and Quisumbing 2003).

*Vulnerability as expected poverty*

In the expected poverty framework, an individual's vulnerability is the prospect of that person becoming poor in the future if currently not poor, or the prospect of him/her continuing to be poor if currently poor (Christiaensen and Subbarao 2004). Thus, vulnerability is seen as expected poverty, while consumption (income) is used as a proxy for well-being. This method is based on estimating the probability that a given shock or set of shocks will move household consumption below a given minimum level (such as a consumption poverty line) or force the consumption level to stay below the minimum if it is already below this level (Chaudhuri  et al. 2002).

Using this method on cross-sectional survey data obtained in 1998, Chaudhuri et al. (2002) found that while only 22 percent of the Indonesian population was poor, as much as 45 percent of the population could be considered vulnerable to poverty. Tesliuc and Lindert (2002) examined cross-sectional survey data obtained from Guatemala in 2000 and showed that three-quarters of the total poor had a vulnerability index in excess of 0.67, indicating that two out of three of the then-poor households would still be poor in the coming period. Similarly, Sarris and Karfakis (2006) measured the vulnerability of rural households in Tanzania, and found that poorer regions were considerably more vulnerable to poverty.

One of the disadvantages of this method, however, is that the use of estimations made across a single cross-section requires the strong assumption that the cross-sectional variability captures temporal variability (Hoddinott and Quisumbing 2003).

*Vulnerability as low expected utility*

Ligon and Schechter (2002, 2003) defined vulnerability as the difference between the utility derived from some level of certainty-equivalent consumption (at and above which the household would not be considered vulnerable) and the expected utility of consumption. Ligon and Schechter (2003) applied this method to a panel dataset obtained from Bulgaria in 1994, and found that poverty and risk played roughly equal roles in reducing welfare. The disadvantage of this method is that it is difficult to account for an individual's risk preference, given that individuals are ill-informed about their preferences, especially those related to uncertain events (Kanbur 1987).

*Vulnerability as uninsured exposure to risk*

This method is based on as ex post assessment of the extent to which a negative shock causes welfare loss (Hoddinott and Quisumbing 2003).  Here, the shock impact is assessed by using panel data to quantify shock-induced changes in consumption. Skoufias (2003) employed this approach to analyze the impact of shocks on Russia. In the absence of risk-management tools, shocks impose a welfare loss that materializes through reductions in consumption. The amount of loss incurred due to shocks equals the amount paid as insurance to keep a household as well off as it had been prior to any shock.

This approach, which is mainly based on regressions of panel datasets containing the consumption levels of specific households before and after a specific shock, analyzes how households mange to smooth their consumptions over time, and categorizes households as vulnerable or less vulnerable. In the absence of panel data, it is typically impossible to measure the impact of shocks such as droughts, floods and hailstorms, as ex ante and ex post consumption and income data are generally not included in cross-sectional household-level datasets. Moreover, an attempt to compare the predicted incomes between households that did and did not experience shocks will result in a biased estimate of the shock impacts, largely because household income levels do not vary only due to shocks. Instead, households vary across many different attributes and hence may have different income levels even in the absence of shock. Thus, these exercises cannot precisely quantify shock-specific losses.

# 3. EMPIRICAL MODEL, STUDY AREA AND DATA

## 3.1. Empirical Model

Here, the probability of a farmer falling below a given consumption (income) level due to climatic shocks (droughts, floods or hailstorms) was measured with the vulnerability as expected poverty approach. This allows estimation of the proportion of people that are vulnerable to shocks, and hence may be used to support policies aimed at implementing safety nets or adaptation strategies.

Following Chaudhuri et al. (2002), the stochastic process generating the consumption of a household h is given by:

$$\ln C_h = X_h \beta + e_h \tag{1}$$

where $C_h$ is per capita consumption[1] expenditure, $X_h$ represents a bundle of observable household characteristics (household size, location, educational attainment of the household head, etc.) and climatic shocks (droughts, floods and hailstorms), β is a vector of parameters, and eh is a mean-zero disturbance term. The dependent and independent variables are described in Table 6.

We assume that the variance of eh is given by:

$$\sigma_{e,h}{}^2 = X_h \theta \tag{2}$$

where β and θ are parameter estimates obtained from the three-step feasible generalized least squares (FGLS) procedure suggested by Amemiya (1977).

Using the estimates β and θ, the expected log of consumption and the variance of log consumption for each household h are, respectively, estimated as:

$$\hat{E}\left[\ln C_h \mid X_h\right] = X_h \hat{\beta} \tag{3}$$

$$\hat{V}\left[\ln C_h \mid X_h = \sigma^{\wedge 2}{}_{e,h} = X_h \hat{\theta}\right] \tag{4}$$

By assuming that consumption is log-normally distributed (i.e. that $\ln C_h$ is normally distributed), the above equations allow us to estimate the probability that a household with characteristics $X_h$ will be poor (i.e., the household's vulnerability level). If $\Phi(.)$ denotes the cumulative density of the standard normal, the estimated probability will be given by:

$$\hat{V}_h = \hat{\Pr}(\ln C_h < \ln z \mid X_h) = \Phi\left(\frac{\ln z - X_h \hat{\beta}}{\sqrt{X_h \hat{\theta}}}\right) \tag{5}$$

where ln *z* is the log of the minimum consumption/income level beyond which a household would be called vulnerable.

This analysis is based on the assumption that experiencing climatic shocks such as a drought, flood and/or hailstorm will increase the probability of a farmer falling below a given consumption/income level, or force him/her to stay under such a level if already below it.

[1] This study considers farmers' incomes rather than their consumption. In poor countries, including Ethiopia, it is assumed that most or all of the farmers' incomes are consumed, and the farmers do not save.

### 3.2. Study Area

The study area for this research is the Nile basin of Ethiopia. The Nile basin of Ethiopia covers a total area of about 358,889 km$^2$, which is equivalent to 34 percent of Ethiopia's total geographic area, and contains about 40 percent of the country's population. Portions of six different regional states of Ethiopia are contained within the basin, namely: 38 percent of the total land area of Amhara, 24 percent of Oromiya, 15 percent of Benishangul-Gumuz, 11 percent of Tigray, 7 percent of Gambella and 5 percent of Southern Nations Nationalities and Peoples (SNNP) (MoWR 1998).

The basin contains three major rivers: the Abbay River, which originates from the central highlands; the Tekeze River, which originates from the north-western parts of the country and the Baro-Akobo River, which originates from the south-western part of the country. The total annual surface runoff of the three rivers is estimated at 80.83 billion cubic meters per year, which amounts to nearly 74 percent of the total runoff from Ethiopia's 12 river basins (MoWR 1998). Of the five agro-ecological zones found in Ethiopia (Table 1), the surveyed districts all fall under three: namely dega, weynadega and kola (Figure 1).

**Table 1. Climatic zones and their physical characteristics**

| Zone | Altitude (meters) | Rainfall (mm/year) | Average annual temperature (°C) |
|---|---|---|---|
| Wurch<br>(cold and moist) | 3200 plus | 900 – 2200 | >11.5 |
| Dega<br>(cool and humid) | 2300 – 3200 | 900 – 1200 | 17.5/16.0 – 11.5 |
| Weynadega<br>(cool and sub-humid) | 1500 – 2300 | 800 – 1200 | 20.0 – 17.5/16.0 |
| Kola<br>(warm and semi-arid) | 500 – 1500 | 200 – 800 | 27.5 – 20 |
| Berha<br>(hot and arid) | under 500 | under 200 | >27.5 |

Source: MoA (2000)

**Figure 1. Nile Basin of Ethiopia with its agro-ecological classifications and survey districts**

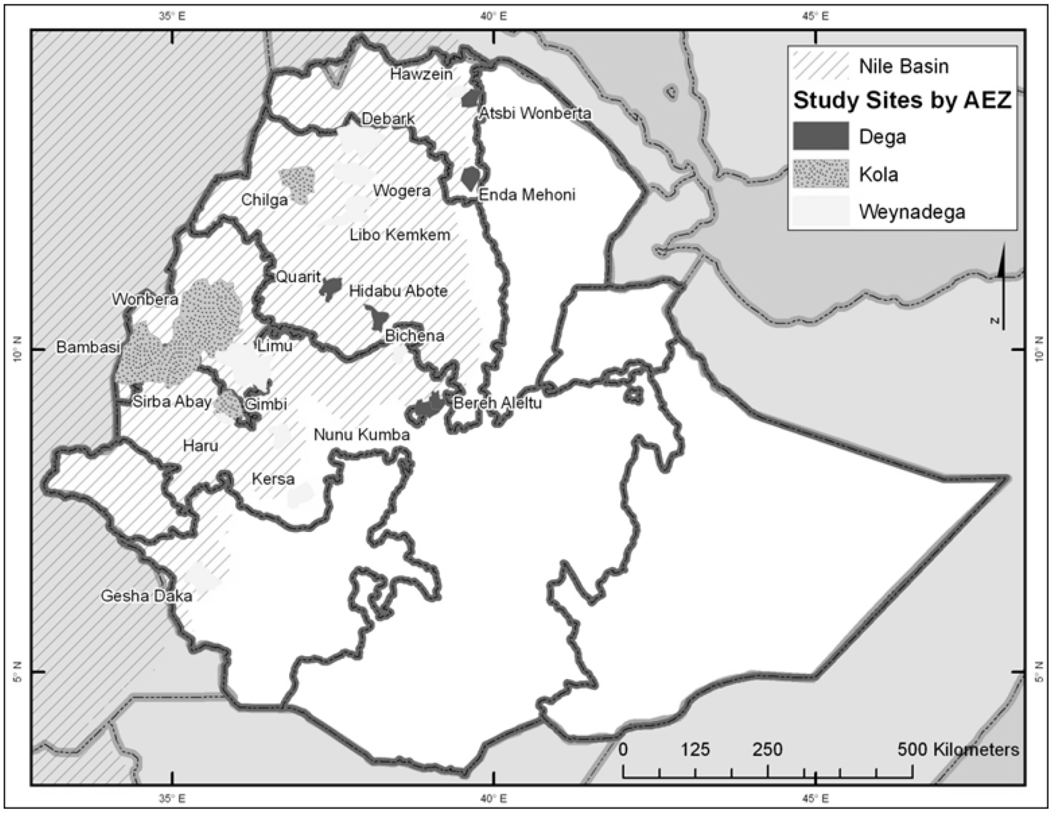


Source: MOA (2000)

## 3.3. Data

The data used for this study come from a farmers' household survey performed during the 2004/2005 production year in the Nile Basin of Ethiopia. The International Food Policy Research Institute (IFPRI) conducted this cross-sectional survey in collaboration with the Ethiopian Development Research Institute (EDRI). The sampled districts were selected to represent the different attributes of the basin, including the typologies of the regions' agro-ecological zones (dega, weynadega and kola), the degree of irrigation activity (percent of cultivated land), the average annual rainfall, the rainfall variability, and the vulnerability (food aid-dependent population).

Peasant associations (administrative units lower than districts) were also purposively selected to include households that irrigate their farms. One peasant association was selected from each of 20 sampled districts, for a total of 20 sampled peasant associations. Once the peasant associations were chosen, 50 farming households were randomly selected from each peasant association (Peasant associations have more than one village) for a total of 1000 interviewed households. Table 2 lists the surveyed districts and peasant associations.

**Table 2. Surveyed districts and peasant associations**

| Regional state | Zone | District | Peasant association | Number of households |
|---|---|---|---|---|
| Tigray | East Tigray | Hawzein | Selam | 50 |
| | | Atsbi Wonberta | Felege Woinie | 50 |
| | South Tigray | Endamehoni | Mehan | 50 |
| Amhara | North Gondar | Debark | Mekara | 50 |
| | | Chilga | Teber Serako | 50 |
| | | Wogera | Sak Debir | 50 |
| | South Gondar | Libo Kemkem | Angot | 50 |
| | East Gojam | Bichena | Aratband Bichena | 50 |
| | West Gojam | Quarit | Gebez | 50 |
| Oromiya | West Wellega | Gimbi | Were Sayo | 50 |
| | | Haru | Genti Abo | 50 |
| | East Shoa | Bereh Aleltu | Welgewo | 50 |
| | East Shoa | Hidabu Abote | Sira marase | 50 |
| | East Wellega | Limu | Areb Gebeya | 50 |
| | | Nunu Kumba | Bachu | 50 |
| | Jimma | Kersa | Merewa | 50 |
| Benishangul Gumuz | Metekel | Wonbera | Addis Alem | 50 |
| | Asosa | Bambasi | Sonka | 50 |
| | Kamashi | Sirba Abay | Koncho | 50 |
| SNNP | Zone 1 | Gesha Daka | Kicho | 50 |
| Total | | | | 1,000 |

Source: Authors' calculations from the IFPRI/EDRI survey data

The collected dataset includes the following: household characteristics; the incidence of different climatic and other shocks over the previous five years; food aid; land tenure; machinery ownership; rain-fed and irrigated agriculture; livestock production; access to credit, markets and extension services; income and food expenditures; perceptions of climate change; adaptation options; and social capital. Moreover, temperature and rainfall data for the surveyed households during the relevant production seasons were obtained from IFPRI.

# 4. RESULTS AND DISCUSSION

## 4.1. Descriptive Statistics

The average income of the sampled farmers was equal to Ethiopian Birr 4356.20 per year (about 1.2 USD per day). The percentages of households that reported droughts, floods and hailstorms over the prior five years were 31, 12 and 18 percent, respectively (Table 3). The relatively high frequency of drought-affected households is consistent with Ethiopia being a drought-prone country. These shocks resulted in a variety of reported losses, primarily consisting of crop yield declines and asset/income losses (Table 4). The majority of farmers did nothing to respond to these shocks, mainly due to poverty. Those farmers who attempted to cope with the negative impacts of the shocks responded mainly by selling their livestock, borrowing from their relatives, participating in food-for-work programs, and/or obtaining food aid (Table 5).

**Table 3. Major shocks encountered by surveyed farmers**

| Shock | Number of farmers | Percent of farmers |
|---|---|---|
| Drought | 380 | 31.0 |
| Hailstorm | 225 | 18.3 |
| Flood | 142 | 11.6 |
| Animal disease | 112 | 9.1 |
| Pest damage to crops before harvest | 84 | 6.8 |
| Illness of family member | 71 | 5.8 |

Source: Authors' calculations from the IFPRI/EDRI survey data

**Table 4. Effects of shocks by surveyed farmers**

| Result | Number of farmers | Percent of farmers |
|---|---|---|
| Decline in crop yield | 403 | 32.8 |
| Loss of assets | 213 | 17.4 |
| Loss of income | 201 | 16.4 |
| Food insecurity/shortage | 140 | 11.4 |
| Death of livestock | 128 | 10.4 |
| Decline in consumption | 124 | 10.1 |

Source: Authors' calculations from the IFPRI/EDRI survey data

**Table 5. Coping strategies by surveyed farmers**

| Response to shocks | Number of farmers | Percent of farmers |
|---|---|---|
| Did nothing | 503 | 41.0 |
| Sold livestock | 438 | 35.7 |
| Borrowed from relatives | 106 | 8.6 |
| Participated in food-for-work | 34 | 2.8 |
| Received food aid | 21 | 1.7 |
| Ate less | 35 | 2.9 |
| Sought off-farm employment | 18 | 1.5 |

Source: Authors' calculations from the IFPRI/EDRI survey data

Closer examination of the coping strategies used to deal with the major environmental shocks (droughts, floods and hailstorms) reveals that most of the surveyed farmers who reported taking action to deal with shocks experienced over the prior five years coped by selling livestock. This suggests that in addition to serving as source of power for farming (e.g., oxen) and manure for fertilizing soil, livestock can serve as assets and insurance against shocks (Yirga, 2007). The other utilized coping strategies include borrowing from relatives, eating less, depending on food aid and food-for-work, and looking for off-farm employment. Figure 2 describes the types of coping strategies employed under different climatic shocks by percent of farmers who used a coping strategy.

**Figure 2. Coping strategies used to deal with major environmental shocks**

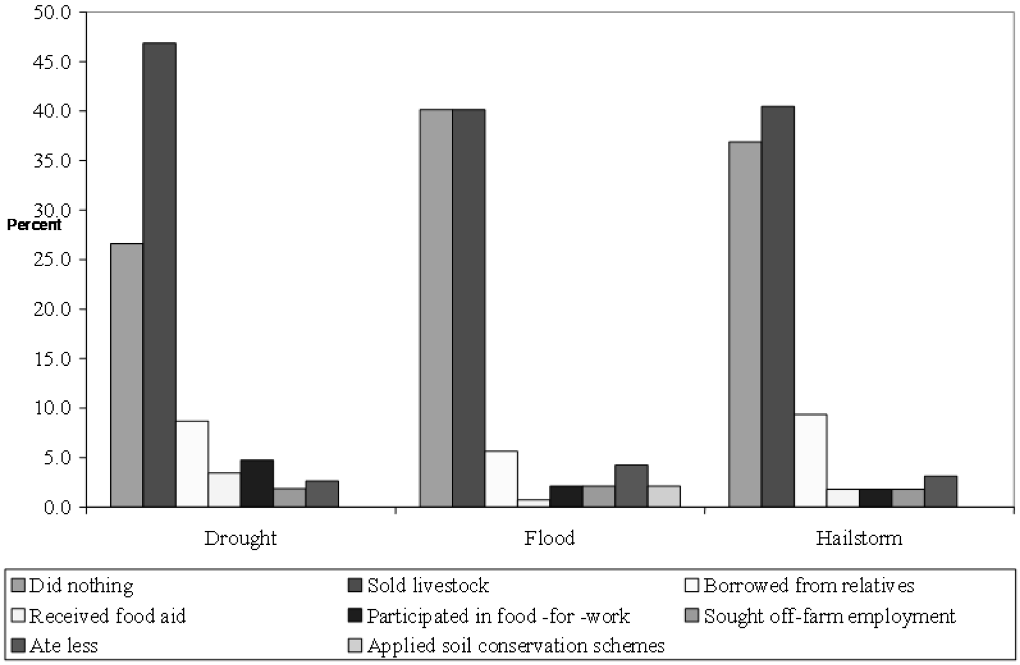


Source: Created by the authors from the IFPRI/EDRI data

Across the household survey, 90 percent of the households were male-headed; the average years of education for the household head was 1.7 years; the average age of the household head was 44.3 years; and the average household size was 6.15 individuals. As indicated in the previous section (section 3.1), the logarithm of farm income has been used as a dependent variable where as different socio-economic and environmental factors have been used as independent variables. Table 6 gives the means and standard deviations of the dependent and independent variables included in the analysis from the surveyed households.

**Table 6. Description of the utilized dependent and independent variables**

| Dependent variable | Mean value | Std. dev. | Description |
|---|---|---|---|
| Farm income | 4356.169 | 7276.695 | Continuous |

| Explanatory variables | Mean | Std. dev. | Description |
|---|---|---|---|
| Drought | 0.3099511 | 0.4626619 | Dummy, takes the value of 1 if occurred during the prior five years, and 0 otherwise |
| Flood | 0.1158238 | 0.3201441 | Dummy, takes the value of 1 if occurred during the prior five years, and 0 otherwise |
| Hailstorm | 0.1835237 | 0.3872532 | Dummy, takes the value of 1 if occurred during the prior five years, and 0 otherwise |
| Years of education for household head | 1.7035 | 2.7777 | Continuous |
| Size of household | 6.1493 | 2.2206 | Continuous |
| Gender of household head | 0.8963 | 0.3051 | Dummy, takes the value of 1 if male and 0 otherwise |
| Age of household head | 44.2915 | 12.6248 | Continuous |
| Livestock ownership | 0.9488 | 0.2205 | Dummy, takes the value of 1 if owned and 0 otherwise |
| Use of crop and livestock extension services | 0.5455 | 0.4982 | Dummy, takes the value of 1 if visited and 0 otherwise |
| Credit access | 0.2191 | 0.4138 | Dummy, takes the value of 1 if there is access and 0 otherwise |
| Farm size in hectares | 2.02 | 1.18 | Continuous |
| Distance to output market in kilometers | 5.70 | 4.14 | Continuous |
| Amhara region | 0.4380098 | 0.4963448 | Dummy, takes the value of 1 if Amhara region and 0 otherwise |
| Oromia region | 0.3026101 | 0.4595754 | Dummy, takes the value of 1 if Oromia region and 0 otherwise |
| Beneshangul region | 0.1272431 | 0.333381 | Dummy, takes the value of 1 if Beneshangul region and 0 otherwise |
| South Peoples' region | 0.0228385 | 0.1494494 | Dummy, takes the value of 1 if South Peoples' region and 0 otherwise |
| Average temperature | 18.63101 | 2.578172 | Continuous, annual average over the 2004–2005 survey period |
| Average rainfall | 111.4413 | 36.58814 | Continuous, annual average over the 2004–2005 survey period |
| Local agro-ecology is Kola | 0.25 | 0.43 | Dummy, takes the value of 1 if Kola and 0 otherwise |
| Local agro-ecology is Weynadega | 0.50 | 0.50 | Dummy, takes the value of 1 if Weynadega and 0 otherwise |
| Local agro-ecology is Dega | 0.25 | 0.43 | Dummy, takes the value of 1 if Dega and 0 otherwise |

Source: Authors' calculations from the IFPRI/EDRI survey data.

## 4.2. Model Results

Using the procedures discussed in Section 3 (applied through the STATA software), we estimate the probability of a household falling below a given level of income (poverty line), and perform a sensitivity analysis by examining this probability using four different minimum levels of income (poverty lines). The choice of minimum levels of income is based on different assumptions such as the international poverty line of 1.25 US per day (World Bank, 2008), average income of the surveyed households and arbitrary values above and below the average income of the surveyed households.

The results are plotted in Figures 3 to 6. The x-axis shows the observed and imputed values for the natural log of income, while the y-axis shows the computed estimates of vulnerability. Each graph is broken in to four sections. Those in the upper left are poor today and likely to be poor tomorrow, and those in the bottom left are poor today, but have characteristics suggesting they have a less than 50 ( 0.5 probability level is taken as a cutoff point) percent chance of being poor in the future. Those in the upper right corner are not below the income threshold at present, but are likely to become so in the future, while those in the bottom left are above the income threshold and are likely to remain above it in the future.

In Figure 3, where the poverty line is fixed at 2USD per day, most farmers are poor today and more likely to be poor tomorrow. As Figure 6 shows, when the poverty line is fixed at 0.3USD per day, most people have the characteristics that they are not poor today and are likely to remain above the poverty line in the future. Figures 4-6 depict that the number of people poor today and likely to be poor in the future increases with increasing the minimum income level required to sustain daily life.

The analysis undertaken to compare the vulnerability of households across different agro-ecologies over different scenarios of poverty line indicate that farmers living in kola are the most vulnerable to climatic extremes. Percent of farmers vulnerable under each agro-ecology in conjunction with the different scenarios is presented in Table 7.

**Figure 3. Vulnerability (income at 2 USD per day or 6570 Ethiopian Birr per year) plotted against Ln (income)**

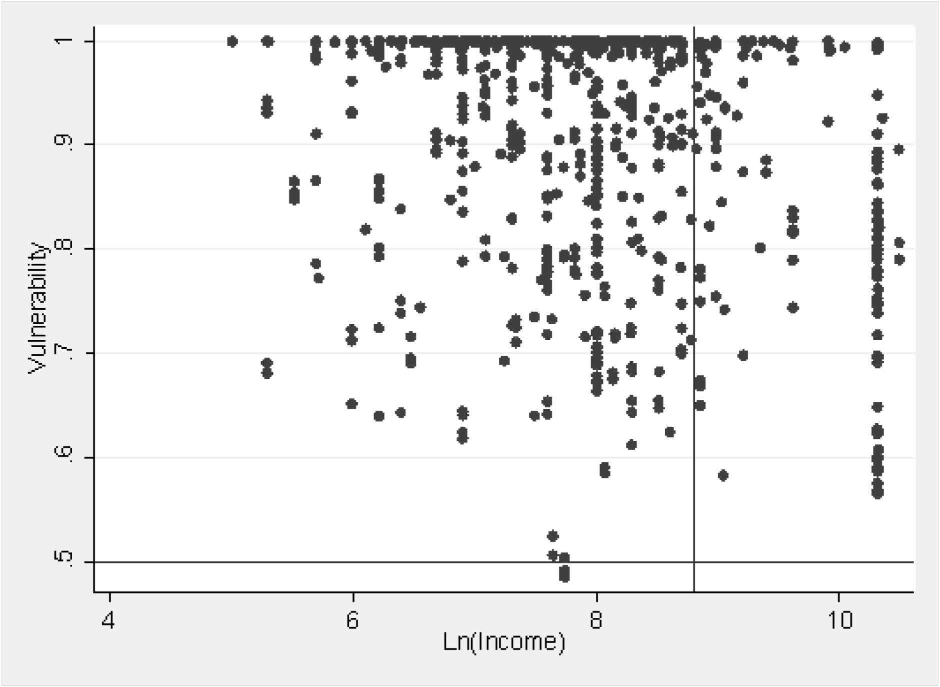


Source: Created by the authors from the IFPRI/EDRI data.

**Figure 4. Vulnerability (income at 1.5 USD per day or 4928 Ethiopian Birr per year) plotted against Ln (income)**

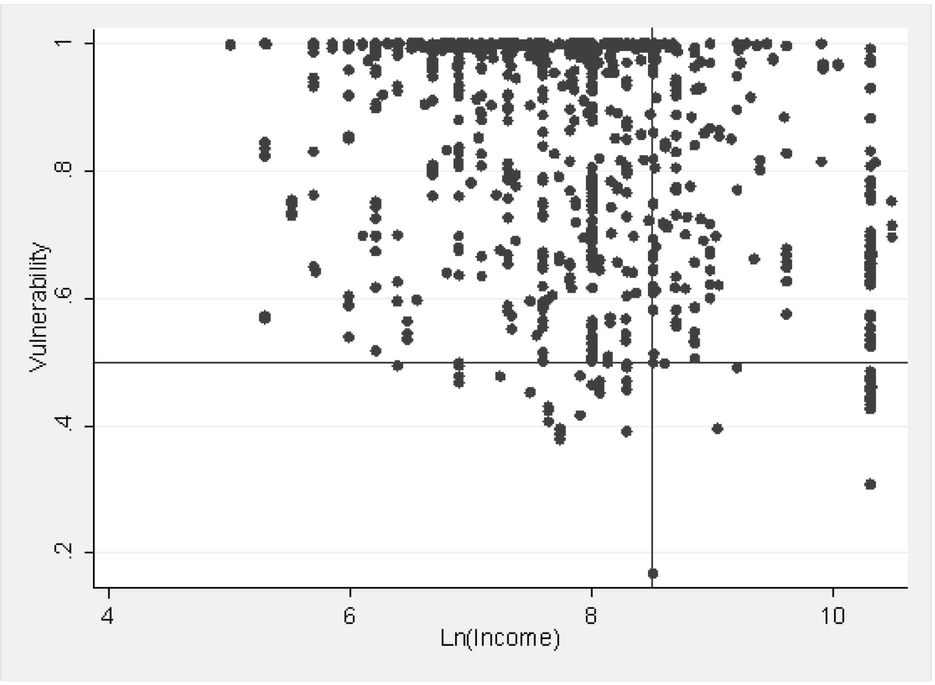


Source: Created by the authors from the IFPRI/EDRI data.

**Figure 5. Vulnerability (income at 1.25 USD per day or 4471 Ethiopian Birr per year) plotted against Ln (income)**

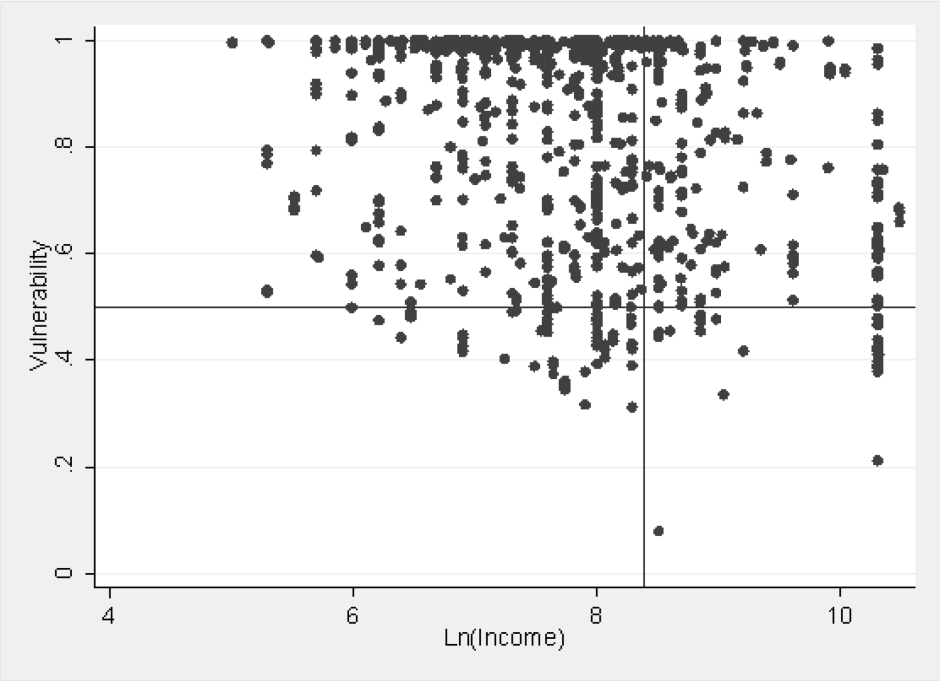


Source: created by the Authors from the IFPRI/EDRI data.

**Figure 6. Vulnerability (income at 0.3 USD per day or 900 Ethiopian Birr per year) plotted against Ln (income)**

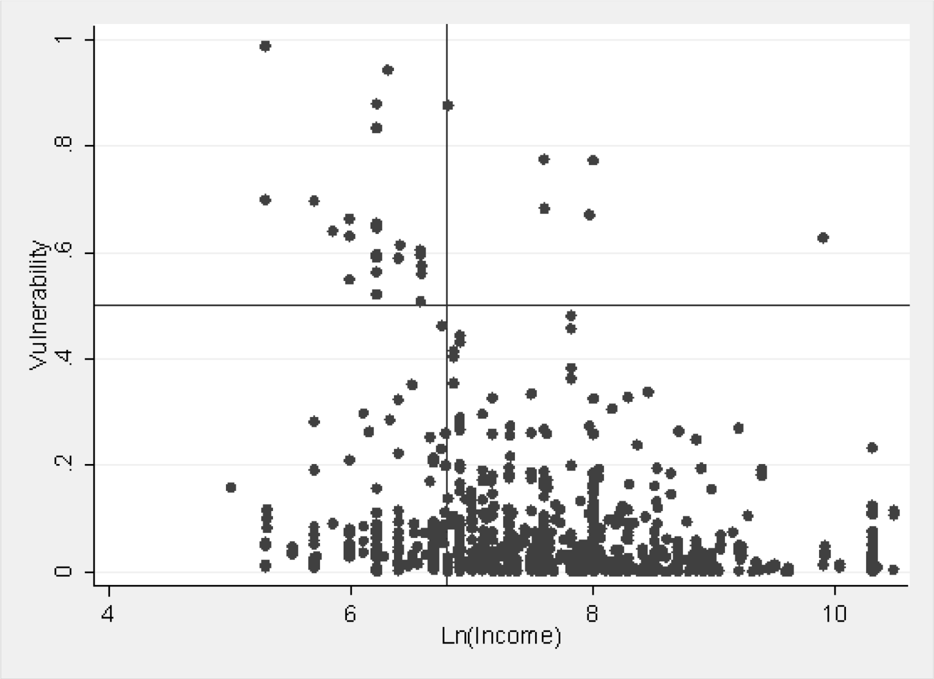


Source: Created by the authors from the IFPRI/EDRI data.

**Table 7. Sensitivity analysis at the agro-ecological level: percent of farmers in each agro ecology**

| Agro-ecological zone | 2 USD per day | | 1.5 USD per day | | 1.25 USD per day | | 0.3 USD per day | |
|---|---|---|---|---|---|---|---|---|
| | p > 0.5 | p < 0.5 | p >0.5 | p< 0.5 | p > 0.5 | p < 0.5 | p > 0.5 | p < 0.5 |
| Kola | 100.00 | 0.00 | 99.64 | 0.36 | 98.57 | 1.43 | 17.20 | 82.80 |
| Dega | 100.00 | 0.00 | 97.69 | 2.31 | 88.65 | 11.35 | 9.26 | 90.74 |
| Weynadega | 99.73 | 0.27 | 93.57 | 6.43 | 93.98 | 6.02 | 12.72 | 87.28 |

Source: Authors' calculations from the IFPRI/EDRI survey data.

Out of the total households surveyed in kola, 99.4 percent of the farmers are vulnerable at present or will be vulnerable in the future (fall above the 50 percent cutoff line); where as the remaining 0.36 percent of the farmers are not vulnerable at present or will not be vulnerable in the near future when the scenario of minimum daily income is fixed at 1.25 USD per day. The same line of explanation also holds for the rest of the scenarios across different agro-ecologies as depicted in Table 7.

As indicated in Section 3.2, the sampled districts were selected to represent the different attributes of the basin, not those of the different regional states represented in the basin. Thus, the following results do not reflect the vulnerability levels of regional states, but rather the vulnerability of the surveyed districts. The results of the sensitivity analysis indicate that the surveyed districts in Beneshangul Gumz and SNNP are the most vulnerable under all of the tested scenarios, while the districts in Oromia are the least vulnerable under all scenarios (Table 8).

**Table 8. Sensitivity analysis at the regional-state level: Percent of farmers in each region**

| Regional state | 2 USD per day | | 1.5 USD per day | | 1.25 USD per day | | 0.3 USD per day | |
|---|---|---|---|---|---|---|---|---|
| | p > 0.5 | p < 0.5 | p> 0.5 | p< 0.5 | p > 0.5 | p < 0.5 | p > 0.5 | p <0.5 |
| Amhara | 100.00 | 0.00 | 99.63 | 0.37 | 89.77 | 10.23 | 14.71 | 85.29 |
| Oromia | 99.46 | 0.54 | 87.33 | 12.67 | 76.82 | 23.18 | 6.47 | 93.53 |
| Beneshangul Gumz | 100.00 | 0.00 | 100.00 | 0.00 | 100.00 | 0.00 | 10.90 | 89.10 |
| SNNP | 100.00 | 0.00 | 100.00 | 0.00 | 100.00 | 0.00 | 5.60 | 94.40 |
| Tigray | 100.00 | 0.00 | 97.01 | 2.99 | 96.27 | 3.73 | 9.70 | 90.30 |

Source: Authors' calculations from the IFPRI/EDRI survey data.

## 5. CONCLUSIONS AND POLICY IMPLICATIONS

Based on household-level survey data from the Nile Basin of Ethiopia, we herein use the vulnerability as expected poverty approach to analyze the probability of farmers falling below a given consumption (income) level due to climatic shocks (droughts, floods and hailstorms). The logarithm of income is assumed to substitute for the logarithm of consumption, as most farmers in Ethiopia consume most of their farm incomes.

A sensitivity analysis, applied by fixing the minimum daily income at different levels, is used to examine the proportion of households vulnerable to climate extremes when the minimum daily income is 1, 1.5, 2, and 0.3 USD per day. When the minimum income is 2 USD per day, 99 percent of the surveyed farmers fall below the minimum income level. In contrast, when the minimum income is 0.3 UDS per day, only 12.4 percent of the surveyed farmers fall below the vulnerability line. Moreover, farmers living in different agro-ecological settings have different levels of vulnerability under the four scenarios.

This study shows that farmers' vulnerability is highly sensitive to their minimum per day income requirement (poverty line) and the agro-ecological setting. When the minimum requirement is higher, most people will be vulnerable poverty due to climate extremes, whereas this vulnerability is lower when the minimum requirement is lower. Furthermore, farmers living in kola zones were relatively more vulnerable to extreme climate events than farmers living in the other agro-ecological zones.

Notably, these preliminary results indicate that increasing farmers' incomes, with special emphasis on farmers in kola agro-ecologies, and enabling them to meet the daily minimum requirement will reduce their vulnerability to climate extremes. Thus, policy interventions should focus on strengthening both household- and public-level climate risk management, through mitigation and coping practices aimed at reducing the damages from climate change. The risk-mitigation strategies that should be addressed at the household level should include those that encourage crop and livestock diversification, the use of drought-tolerant crop varieties and livestock species, the mixing of crop and livestock production, and membership in rotating credit groups. Policies that support coping strategies at the household level should encourage income generation and asset holding, both of which will enable consumption smoothening during and immediately after harsh climatic events.

Public-level risk mitigation strategies might include water harvesting, resource conservation and management, irrigation, voluntary resettlement programs, provision of household and agro-ecological extension packages, inception of productive safety net programs, provision of weather-indexed drought insurance, and the development of well coordinated drought early warning systems. Some helpful public-level coping strategies might be those focusing on the efficient administration of foreign emergency relief aid and effective food-for-work programs.

# REFERENCES


Adger, W. N. 1999. Social vulnerability to climate change and extremes in coastal Vietnam. *World Development* 27 (2): 249–269.

Amemiya, T. 1977. The maximum likelihood estimator and the nonlinear three-stage least squares estimator in the general nonlinear simultaneous equation model. *Econometrica* 45: 955–968.

Bohle, H. G., T. E. Downing, and M. J. Watts. 1994. Climate change and social vulnerability: The sociology and geography of food insecurity. *Global Environmental Change* 4(1): 37–48.

Brooks, N., W. N. Adger, and P. M. Kelly. 2005. The determinants of vulnerability and adaptive capacity at the national level and the implications for adaptation. *Global Environmental Change* 15(2): 151–163.

Chaudhuri, S., J. Jalan, and A. Suryahadi. 2002. *Assessing household vulnerability to poverty: A methodology and estimates for Indonesia*. Department of Economics Discussion Paper No. 0102-52. New York: Columbia University.

Christiaensen, J., and K. Subbarao. 2004. *Toward an understanding of household vulnerability in rural Kenya*. World Bank Policy Research Working Paper 3326. Washington D.C.: World Bank.

CSA (Central Statistical Authority). 2005. *Agricultural survey of farm management practices*. Addis Ababa, Ethiopia: CSA.

Cutter, S. L., B. J. Boruff, and W. L. Shirley. 2003. Social vulnerability to environmental hazards. *Social Sciences Quarterly* 84(2): 243–261.

Deressa, T. 2007. *Measuring the economic impact of climate change on Ethiopian agriculture: Ricardian approach.* World Bank Policy Research Paper No. 4342. Washington D.C.: World Bank.

Dercon, S. 2004. Growth and shocks: Evidence from rural Ethiopia. *Journal of Development Economics* 74: 309–329.

Dercon, S., and P. Krishnan. 2000. Vulnerability, seasonality and poverty in Ethiopia. *Journal of Development Studies* 36(6): 25–53.

Dercon, S., J. Hoddinott, and T. Woldehanna. 2005. Vulnerability and shocks in 15 Ethiopian villages, 1999-2004. *Journal of African Economies* 14: 559–585.

Easter, C. 1999. Small states development: A commonwealth vulnerability index. *The Round Table* 351(1): 403–422.

Franklin, S., and T. Downing. 2004. Resilience and vulnerability. Poverty and Vulnerability Programme: GECAFS Project. Stockholm Environmental Institute. <http://www.sei.e-collaboration.co.uk/OPMS/getfile.php>. Accessed October 2006.

Hoddinott, J., and A. Quisumbing. 2003. *Methods for microeconometric risk and vulnerability assessments*. Social Protection Discussion Paper Series No. 0324. Social Protection Unit, Human Development Network. Washington D.C.: World Bank.

IPCC (Intergovernmental Panel on Climate Change). 2001. Climate change: The scientific basis. <http://www.ipcc.ch/>. Accessed June 2006.

Kaly, U., and C. Pratt 2000. *Environmental vulnerability index: Development and provisional indices and profiles for Fiji, Samoa, Tuvalu and Vanuatu. Phase II report for NZODA.* SOPAC Technical Report 306: 89.

Kaly, U., L. Brigugilo, H. McLeod, S. Schmsall, C. Pratt, and R. Pal. 1999. *Environmental vulnerability index (EVI) to summarize national environmental vulnerability profiles*. SOPAC Technical Report 275.

Kanbur, R. 1987. The standard of living: Uncertainty, inequality and opportunity. In The Standard of Living. G. Hawtorn (ed). New York: Cambridge University Press.

Kasperson, R. E., J. X. Kasperson, and K. Dow. 2001. Equity, vulnerability and global environmental change. In *Global Environmental Risk,* J. X. Kasperson and R. E. Kasperson (eds). Tokyo: United Nations University Press. 247-272.

Lautze, S., Y. Aklilu, A. Raven-Roberts, H. Young, G. Kebede, and J. Learning. 2003. *Risk and vulnerability in Ethiopia: Learning from the past, responding to the present, preparing for the future. Report for the U.S. Agency for International Development.* Addis Ababa, Ethiopia.

Leon-Vasquez, M., T. West, and J. Finan. 2003. A comparative assessment of climate vulnerability: Agriculture and ranching on both sides of the US- Mexico border. *Global Environmental Change* 13(3): 159–173.

Leichenko, R. M., and K. L. O'Brien. 2001. The dynamics of rural vulnerability to global change: The case of Southern Africa. *Mitigation and adaptation strategies for global change* 7 (1): 1–18.

Ligon, E., and L. Schechter. 2002. *Measuring vulnerability*. Manuscript. California: University of California, Berkeley.

_____ 2003. Measuring vulnerability. *Economic Journal* 113: 95–102.

Luers, A. L., D. B. Lobell, L. S. Skar, C. L. Adams, and P. A. Matson. 2003. A method for quantifying vulnerability, applied to the agricultural system of the Yaqui Valley, Mexico. *Global Environmental Change* 13: 255–267.

Mitchell, T., and T. Tanner. 2006. *Adapting to climate change-challenges and opportunities for the development community.* Teddington, UK: Institute of Development Studies.

MoA (Ministry of Agriculture). 2000. *Agro-ecological zonations of Ethiopia*. Addis Ababa, Ethiopia.

Morrow, B. H. 1999. Identifying and mapping community vulnerability. Disasters 23(1): 1–8.

Moss, R., A. Brenkert, and E. Malone. 2001. Vulnerability to climate change, a quantitative Approach. <http://www.ntis.gov.html>. Accessed June 12, 2006.

MoWR (Ministry of Water Resources). 1998. *Tekeze River Basin Integrated Development Master Plan Project.* Executive Summary, Federal Democratic Republic of Ethiopia (FDRE), Addis Ababa.

NMSA (National Meteorological Services Agency). 2001. *Initial national communication of Ethiopia to the United Nations Framework Convention on Climate Change (UNFCCC),* Addis Ababa, Ethiopia.

O'Brien, K., R. Leichenko, U. Kelkar, H. Venema, G. Aandahl, H. Tompkins, A. Javed, S. Bhadwal, S. Barg, N. Nygaard, and J. West. 2004. Mapping vulnerability to multiple stressors: Climate change and globalization in India. *Global Environmental Change* 14(4): 303–313.

Reilly, J. M., and D. Schimmelpfennig. 1999. Agricultural impact assessment, vulnerability, and the scope for adaptation. *Climatic Change* 43(4): 745–788.

Skoufias, E. 2003. Consumption smoothing in Russia. *Economics of Transition* 11(1): 67–91.

Skoufias, E., and A. R. Quisumbing. 2003. Consumption insurance and vulnerability to poverty: A synthesis of the evidence from Bangladesh, Ethiopia, Mali, Mexico and Russia. Paper presented at the conference "Staying Poor: Chronic Poverty and Development Policy" at the University of Manchester, 7 to 9 April 2003. United Kingdom.

Sarris, A., and P. Karfakis. 2006. *Household vulnerability in rural Tanzania*. FAO Commodities and Trade Policy Research Working Paper No. 17. Rome, Italy.

Tesliuc, E., and K. Lindert. 2002. *Vulnerability: A quantitative and qualitative assessment. Guatemala Poverty Assessment Program.* Washington D.C.: World Bank.

Vincent, K. 2004. *Creating an index of social vulnerability to climate change for Africa.* Technical Report 56. Norwich, U.K.: Tyndall Centre for Climate Change Research, University of East Anglia.

World Bank. 2003. *Africa rainfall and temperature evaluation system*. Washington D.C.: World Bank.

World Bank. 2008. Updates of Poverty Estimates for the Developing World. Washington D.C.: World Bank. http://go.worldbank.org/C9GR27WRJ0. Accessed October, 2009

Yamin, F., A. Rahman, and S. Huq. 2005. Vulnerability, adaptation and climate disasters: A conceptual overview. *IDS Bulletin* 36: 1–14.

Yirga, C. T. 2007. The dynamics of soil degradation and incentives for optimal management in Central Highlands of Ethiopia. Ph.D. Thesis. South Africa: Department of Agricultural Economics, Extension, and Rural Development, University of Pretoria.

# RECENT IFPRI DISCUSSION PAPERS

**For earlier discussion papers, please go to www.ifpri.org/pubs/pubs.htm#dp.**
**All discussion papers can be downloaded free of charge.**

933. I*mpacts of prenatal and environmental factors on child growth:Eevidence from Indonesia*. Futoshi Yamauchi, Katsuhiko Higuchi, and Rita Nur Suhaeti, 2009.

932. R*ich consumers and poor producers: Quality and rent distribution in global value chains*. Johan F.M. Swinnen and Anneleen Vandeplas, 2009.

931. *Economic growth and distribution of income: A Growth model to fit Ghanaian data. Harumi* T. Nelson , Terry L. Roe, and Xinshen Diao, 2009.

930. *Trade liberalization, poverty, and food security in India*. Manoj Panda and A. Ganesh-Kumar, 2009.

929. *Improving the proof: Evolution of and emerging trends in impact assessment methods and approaches in agricultural development.* Mywish K. Maredia, 2009.

928. *Improving diet quality and micronutrient nutrition: Homestead food production in Bangladesh.* Lora Iannotti, Kenda Cunningham, and Marie Ruel, 2009.

927. *Land-tenure policy reforms: Decollectivization and the Doi Moi System in Vietnam.* Michael Kirk and Tuan Nguyen, 2009.

926. *"Crossing the river while feeling the rocks:" Incremental land reform and its impact on rural welfare in China.* John W. Bruce and Zongmin Li, 2009.

925. *Rich food for poor people: Genetically improved tilapia in the Philippines*. Sivan Yosef, 2009.

924 *.Rural and urban linkages: Operation Flood's role in India's dairy development.* Kenda Cunningham, 2009.

923. *The global effort to eradicate rinderpest*. Peter Roeder and Karl Rich, 2009.

922. *The mungbean transformation: Diversifying crops, defeating malnutrition.* Subramanyan Shanmugasundaram, J. D. H. Keatinge, and Jacqueline d'Arros Hughes, 2009.

921. *Private sector responses to public investments and policy reforms: The case of fertilizer and maize market development in Kenya.* Joshua Ariga and T. S. Jayne, 2009.

920. *Institutional reform in the Burkinabè cotton sector and its impacts on incomes and food security: 1996–2006.* Jonathan Kaminski, Derek Headey, and Tanguy Bernard, 2009.

919. *Pearl millet and sorghum improvement in India.* Carl E. Pray and Latha Nagarajan, 2009.910. *Combating stem and leaf rust of wheat: Historical perspective, impacts, and lessons learned.* H. J. Dubin and John P. Brennan, 2009.

918. *Hybrid rice technology development: Ensuring China's food security.* Jiming Li, Yeyun Xin, and Longping Yuan, 2009.

917. *The impact of shallow tubewells and boro rice on food security in Bangladesh.* Mahabub Hossain, 2009.

916. *Zero tillage in the rice-wheat systems of the Indo-Gangetic plains: A review of impacts and sustainability implications.* Olaf Erenstein, 2009.

915. *The case of zero-tillage technology in Argentina.* Eduardo Trigo, Eugenio Cap, Valeria Malach, and Federico Villarreal, 2009.

914. *Agro-environmental transformation in the Sahel: Another kind of "Green Revolution."* Chris Reij, Gray Tappan, and Melinda Smale, 2009.

913. *Community forestry in Nepal: A policy innovation for local livelihoods.* Hemant Ojha, Lauren Persha, and Ashwini Chhatre, 2009.

912. *Controlling cassava mosaic virus and cassava mealybug in Sub-Saharan Africa.* Felix Nweke, 2009.

911. *The Asian Green Revolution*. Peter B. R. Hazell, 2009.

910. *Combating stem and leaf rust of wheat: Historical perspective, impacts, and lessons learned.* H. J. Dubin and John P. Brennan, 2009.

**INTERNATIONAL FOOD POLICY RESEARCH INSTITUTE**

**www.ifpri.org**

**IFPRI HEADQUARTERS**

2033 K Street, NW
Washington, DC 20006-1002 USA
Tel.: +1-202-862-5600
Fax: +1-202-467-4439
Email: ifpri@cgiar.org

**IFPRI ADDIS ABABA**

P. O. Box 5689
Addis Ababa, Ethiopia
Tel.: +251 11 6463215
Fax: +251 11 6462927
Email: ifpri-addisababa@cgiar.org

**IFPRI NEW DELHI**

CG Block, NASC Complex, PUSA
New Delhi 110-012 India
Tel.: 91 11 2584-6565
Fax: 91 11 2584-8008 / 2584-6572
Email: ifpri-newdelhi@cgiar.org
