# Supplementary material for: Systematic Review of Methods in Low-Consensus Fields: Supporting Commensuration through `Construct-Centered Methods Aggregation’ in the Case of Climate Change Vulnerability Research
Source: PLoS One. 2016 Feb 22;11(2):e0149071. doi: 10.1371/journal.pone.0149071 (PMC4762661; doi:10.1371/journal.pone.0149071)
Supplement: S2 File — Source articles and bibliography. (ZIP) [file pone.0149071.s002.zip › data requirements/Echevin.pdf]

MPRA
Munich Personal RePEc Archive

Characterizing poverty and vulnerability in rural Haiti: a multilevel decomposition approach

Damien Echevin

Sherbrooke University, CRCELB, CIRPÉE

2011

Online at https://mpra.ub.uni-muenchen.de/35659/
MPRA Paper No. 35659, posted 31. December 2011 22:05 UTC

Characterizing Poverty and Vulnerability in Rural Haiti: A Multilevel Decomposition Approach*

Damien Échevin†

Abstract: This paper aims at characterizing poverty and vulnerability in Haiti based on a unique survey conducted in 2007 in rural areas. Using two-level modelling of consumption/income, we assess the impact of both observable and unobservable idiosyncratic and covariate shocks on households' economic well-being. Empirical findings show that idiosyncratic shocks, in particular health-related shocks, have larger impact on vulnerability to poverty than covariate shocks. These results are in line with the fact that many households reported idiosyncratic health shocks as being the worst shocks they experienced. Also, unobservable idiosyncratic shocks appear to have generally more influence on households' vulnerability than unobservable covariate shocks. Geographic disparities exist and should be considered for policy and program implementation purposes.

Keywords: Vulnerability; Poverty; Observable and unobservable shocks; Multilevel modelling; Haiti.

JEL Codes: D31; I32; O12; O15.

---

*I thankfully acknowledge Gary Mathieu from CNSA-Haiti for providing the data used in this paper. Francesca Lamanna and Ana Maria Oviedo provided very useful comments and suggestions. All errors and opinions expressed in this paper remain mine.

† Université de Sherbrooke, CRCELB and CIRPÉE. Email: damien.echevin@usherbrooke.ca.

1. Introduction

The nature of shocks and the way people cope with them (or get insured) are major matters of consideration when the focus for governments and agencies is to design policies and programs to alleviate vulnerability to poverty in developing countries (see, *e.g.*, Alderman and Paxson, 1994, Townsend, 1995, Morduch, 1999, and Dercon, 2002). Vulnerable people are those with low opportunities and who encounter difficulties when shocks occur. For instance: disabled people, children, female headed families and elderly people can generally be considered as vulnerable groups. People living in rural areas are also particularly vulnerable to shocks, due to poor environment (poor soils, poor infrastructures, lower access to markets, etc.) or because they do not have the capacity to diversify their income sources. By contrast, people can adopt efficient coping strategies and behaviours against adverse shocks such as: diversification of income, accumulation of assets, and so forth. Such behaviours generally allow households to pursue activities with better returns and, thus, avoid poverty traps (see, *e.g.*, Zimmerman and Carter, 2003, and Carter and Barrett, 2006).

Households may also provide mutual insurance to one another in order to smooth their consumption in the face of risk (Townsend, 1995). So, current income and shocks should play only a minor role in determining households' consumption. In reality, "excess sensitivity" of consumption to shocks generally occurs when shocks are aggregated covariate ones, whereas households can better insure consumption against idiosyncratic shocks (see, *e.g.*, Mace, 1991, Cochrane, 1991). Hence, governments should be more efficiently alarmed by covariate or aggregate shocks (be they economic, climate or other) and also better armed to protect/secure people from these shocks. However, in developing countries, in the presence of imperfect markets for credit and insurance, few households are able to smooth their consumption when facing individual income shocks (see empirical evidences by Rosenzweig and Wolpin, 1993, Morduch, 1995, Fafchamp *et al.*, 1998, Kazianga and Udry, 2006, and Hoddinott, 2006). In such conditions, vulnerability to shocks gets higher in a society. Furthermore, if not well targeted, social transfers may crowd out private transfers, since incentives are reduced to share private risk (see, *e.g.*, Attanasio and Rios Rull, 2000, and Dercon and Krishnan, 2003).

For policy and program implementation purposes, the following issues have to be addressed. First, we should be able to tell transitorily poor households (who may be vulnerable to poverty because consumption or income fluctuates over time) from structurally poor households. Second, it appears necessary to understand why transitorily or structurally poor households are exposed to consumption or income volatility that may contribute to vulnerability. Why can't they cope with these risks? Is it because households mostly face covariate shocks so that they can't be insured against? Or is it because they do not have the ability to cope with even moderate levels of idiosyncratic shocks?

In order to fully characterize the determinants of poverty and vulnerability in rural Haiti, a unique survey can be used to assess the impact of idiosyncratic and covariate shocks on economic well-being (such as household consumption or income). This household survey on Haitian food security and vulnerability has been conducted in 2007 in rural areas. The number of households is around 3,000 distributed in 228 communities. It contains quantitative information on household consumption, production, income and assets as well as a good deal of qualitative information on perceived shocks, coping strategies, response capacity and other risks.

This paper will firstly apply the methodology proposed by Datt and Hoogeveen (2003) who derive counterfactual consumption of households in the absence of shocks from the estimates of a single equation linking household living standard to household characteristics and shocks variables. Counterfactual estimates of consumption (resp. income) can then be used to assess the impact of various observable shocks on poverty, be they idiosyncratic or covariate shocks. Secondly, an extension of this empirical framework will consist in using two-level (*i.e.* household and community levels) modelling of the impact of those shocks following Günther and Harttgen (2009)'s approach. The methodology will allow us to estimate the variance of household-level and community-level unobservable shocks. Finally, the variance of shocks can be decomposed into various components: observable idiosyncratic shocks, observable covariate shocks, unobservable idiosyncratic shocks, and unobservable covariate shocks. Following

Chaudhuri *et al.* (2002) or Christiaensen and Subbarao (2005), it will be possible to provide estimates of household vulnerability to poverty considering these various components.[1]

This paper is organized as follows. Section two presents the methodology. Section three presents the data. Section four provides regression results together with some simulations of the impact of shocks on both poverty and vulnerability. The last section concludes.

2. Methodology

Vulnerability to shocks

In this section, we explore the relationships between consumption or income, on the one hand, and various idiosyncratic and aggregate covariates on the other hand. We suppose that households are imperfectly insured against shocks and have limited access to credit. So, assuming uninsured exposure to risk, we can write:

$$\ln y_{ij} = X_{ij}\beta + S_{ij}\gamma + S_{ij}\delta' X'_{ij} + \theta_j + \varepsilon_{ij}\,, \tag{1}$$

where y_{ij} is the consumption of household *i* in community *j*, X_{ij} is a vector of household characteristics, S_{ij} is a vector of observable shocks, θ_j is a community specific effect and ε_{ij} is the error term.

In the above equation, two parameters are of particular interest. First, we should assess whether γ is significantly different from zero that is whether observable shocks have significant impact on economic well-being. Second, in order to ascertain whether observable shocks have different impacts depending on household and community characteristics, we should also assess whether δ is significantly different from zero.

[1] Other recent contributions to the literature of vulnerability to poverty are, among others, Ligon and Schechter (2003), Kamanou and Murdoch (2004), Calvo and Dercon (2005), Duclos et al. (2011).

Community specific effect θ_j can be modelled either as a fixed effect or a random effect. In what follows, we will see how to model this unobservable component within a two-level linear random coefficient model. Finally, we should take into account the possibility that the error term ε_{ij} can be correlated with observable household characteristics and shocks so that parameters estimates might be biased.

Following Datt and Hoogeveen (2003), equation (1) parameters estimates are used to measure the impact of the observable shocks on poverty. First, the counterfactual welfare index (y_{ij}^*) is derived from the difference between actual consumption (y_{ij}) and the estimated impact of observable shocks that is:

$$y_{ij}^* = y_{ij} - [\exp(\ln \hat{y}_{ij}) - \exp(\ln \hat{y}_{ij} \mid S_{ij} = 0)]\,. \tag{2}$$

Second, we measure the impact of shocks on poverty by a *poverty gap* (*PG*):

$$PG = \Pr(\ln y_{ij} < \ln z) - \Pr(\ln y_{ij}^* < \ln z)\,. \tag{3}$$

This poverty gap will inform us about the extent to which shocks affect poverty so that policy should be implemented to reduce the impact of shocks on social welfare.

Parameters estimates in equation (1) can be used as measures of vulnerability since they inform us about coping mechanisms. However, we don’t learn much from these parameters about the variability of shocks, so we do not know their vulnerability incidence.

Vulnerability as expected poverty

One step further, we can define vulnerability to poverty as the probability of falling into poverty when one’s consumption/income falls below a predefined poverty line. Furthermore, households will be considered as vulnerable when the probability to be poor in the future is below a chosen vulnerability threshold. In order to estimate such a probability, Chaudhuri *et al.*

(2002) proposed to estimate the expected mean and variance in consumption using cross-sectional data or short panel data.

Let us define vulnerability for individual *i* in community *j* by:

$$\hat{v}_{ij} = \Pr(\ln y_{ij} < \ln z \mid X_{ij}) = \Phi\left(\frac{\ln z - \ln \hat{y}_{ij}}{\hat{\sigma}_{ij}}\right), \tag{4}$$

where $\Phi(.)$ denotes the cumulative density of the standard normal; *z* is the poverty line; $\ln \hat{y}_{ij}$ is the expected mean of log per capita consumption and $\hat{\sigma}_{ij}^2$ is the estimated variance of log per capita consumption.

As in Christiaensen and Subbarao (2005), the conditional mean and variance could be expressed from equation (1) as:

$$E\left(\ln y_{ij} \middle| X_{ij}\right) = X_{ij}\beta + E\left(S_{ij}\right)\left[\gamma + \delta' X'_{ij}\right] + E\left(\theta_j\right), \tag{6}$$

$$V\left(\ln y_{ij} \middle| X_{ij}\right) = \left[\gamma + \delta' X'_{ij}\right]' V\left(S_{ij}\right)\left[\gamma + \delta' X'_{ij}\right] + \sigma_\theta^2 + \sigma_\varepsilon^2 . \tag{7}$$

One of the main strong points of Chaudhuri's approach resides in the fact that it is rather straightforward to implement on various types of datasets. One limitation of this approach when it is applied to a single cross-section is that it cannot take the temporal variability of parameters into account. Moreover, vulnerability estimates using cross-sections usually prove to rely on partial observation of the local covariate and idiosyncratic shocks experienced by households (*cf.* Christiansen and Subbarao, 2005), which implies omitted variables or reverse causality biases. By taking into account both observable and unobservable shocks our approach thus builds on previous literature by providing a larger spectra of possible shocks endured by households. What is more, a two-level modelling approach will allow us to assess the impact of shocks at a community level which is an appropriate level to analyse risk-sharing behaviours (*cf.* Suri, 2010).

A multilevel decomposition analysis

Our methodological approach is based on a two-level linear random coefficient model where y_{ij} is the consumption of household *i* in community *j*, x_{ij} is a vector of household covariates (such as households characteristics, self-reported shocks and their interactions) and w_j is a vector of community covariates. One writes:

$$\begin{aligned} \ln y_{ij} &= \eta_{0j} + \eta_{1j} x_{ij} + u_{ij}, \\ \eta_{0j} &= \gamma_{00} + \gamma_{01} w_j + \zeta_{0j}, \\ \eta_{1j} &= \gamma_{10} + \gamma_{11} w_j + \zeta_{1j}, \end{aligned} \tag{8}$$

where the error term u_{ij} reflects unobserved heterogeneity of household consumption and the error terms ζ_{0j} and ζ_{1j} represent unobserved heterogeneity of consumption across communities. Given previous equations we get:

$$\ln y_{ij} = \gamma_{00} + \gamma_{01} w_j + (\gamma_{10} + \gamma_{11} w_j) x_{ij} + \zeta_{0j} + \zeta_{1j} x_{ij} + u_{ij}, \tag{9}$$

where the equation can be decomposed into a fixed part and a random part. For identification purposes, we assume that the covariates x_{ij} and w_j are exogenous with $E(\zeta_{0j} | x_{ij}, w_j) = 0$, $E(\zeta_{1j} | x_{ij}, w_j) = 0$ and $E(u_{ij} | x_{ij}, w_j, \zeta_{0j}, \zeta_{1j}) = 0$. This model can be estimated using standard statistical software such as Stata's *gllamm* command (Rabe-Hesketh and Skrondal, 2008).

In contrast with Günther and Harttgen (2009), we will both consider observable and unobservable shocks as sources of vulnerability, whereas Günther and Harttgen do not consider observable shocks in their analysis.

Using this multilevel random coefficient model, we can decompose the total conditional variance into two spatial levels: household and community. So, using equation (9) and following Chaudhuri *et al.* (2002), we estimate the expected unobservable idiosyncratic variance $\hat{\sigma}^2_{u_{ij}}$, covariate variance $\hat{\sigma}^2_{\varsigma_{0j}}$ and total variance $\hat{\sigma}^2_{u_{ij}+\varsigma_{0j}}$ of household consumption using the estimated coefficients from the following regressions:

$$u_{ij}^2 = \alpha_0 + \alpha_1 x_{ij} + \alpha_2 w_j + \alpha_3 x_{ij} w_j,$$
$$\zeta_{0j}^2 = \delta_0 + \delta_1 w_j, \tag{10}$$
$$(u_{ij} + \zeta_{0j})^2 = \theta_0 + \theta_1 x_{ij} + \theta_2 w_j + \theta_3 x_{ij} w_j.$$

Using variance estimates from the above equations, we will provide measures of vulnerability according to the different sources of vulnerability. First, we are concerned with vulnerability induced by structural (or permanent) poverty, that is the fraction of vulnerable households whose expected mean consumption $\ln \hat{y}_{ij}$ is already below the poverty line $\ln z$. Second, we will measure vulnerability induced by risk, that is the fraction of vulnerable households whose expected mean consumption $\ln \hat{y}_{ij}$ lies above the poverty line $\ln z$. As in Chaudhuri *et al.* (2002), a household is considered as vulnerable if the estimated vulnerability index is greater than the vulnerability threshold of 0.29.

Identification issues

A problem associated with the estimation of equations (1) and (9) is that idiosyncratic and covariate observable shocks are potentially endogenous for at least three reasons. First, since the shocks are self-reported by the households in the questionnaire, it might be reported with errors. Hence, it is possible that households with a certain level of consumption or welfare consider an event to be a shock, while others with a different level of consumption or welfare may not. Second, if consumption levels influence the likelihood of exposure to the shock then reverse causality may arise. For instance, health shock has not the same probability of occurring depending on household consumption/income level. This problem is most likely to happen with idiosyncratic shocks. Community shocks are less likely to be influenced by household consumption or income. Third, shocks may be correlated to the error term because of unobserved heterogeneity. Unobserved factors may indeed influence both exposure to shocks and consumption/income. For example, richer households may better irrigate their lands. If irrigation is not observed, the estimated impact of drought on consumption declines may be upwardly biased.

These sources of estimation bias are difficult to take into account with cross-sectional data. However, as proposed in Datt and Hoogeveen (2003), a potential solution is the use of instrumental variables (IV) estimation. Instrumental variables are constructed as community means of shock variables leaving out the current household. These instruments are valid if households are more likely to report a shock when neighbours also report that shock, although neighbours affected by the shock do not influence other household's economic well-being in another way than through the household's self-reported shock.

3. Data

The vulnerability and food security survey was conducted in Haiti in October and November 2007 on approximately 3,000 households living in 228 rural communities. This survey has been realized by the National Coordination of Food Security Unit with the partnership of the World Food Program. A community-related component was added to the household component of the survey, in connection with infrastructures and accessibility to basic social services. So, this survey contains quantitative information on household consumption expenditures, production, income and assets as well as a good deal of qualitative information on perceived shocks, coping strategies and other hazards. Our empirical study will thus try to assess vulnerability by using both sets of data –quantitative and qualitative.

Prior to the 2010 earthquake which has struck western—mostly urban—Haiti, the rural population of Haiti represented about 60% of the total population. These households are particularly vulnerable to natural shocks such as droughts, floods and hurricanes. They also face other risks and shocks such as economic and health shocks, animal disease, crime and violence. When looking at the shocks faced by rural households in Haiti in Table 1, we find that many households face covariate shocks such as: increase in food prices, cyclones, floods, droughts and irregular rainfall; many of those shocks have an impact upon income or upon both income and assets, and less often upon assets only. On the other hand, among the worst shocks declared by the households, most of them are idiosyncratic shocks: they have to do with disease, casualties or death of a household member (for 42.5% of them) or animal diseases (14.0%); the worst

covariate shocks are cyclones, floods, droughts and increase in food prices which concern around 26.3% of the households.

Table 2 presents summary statistics for variables used in the analysis. Consumption and income are expressed in Gourdes. The agricultural index is a composite indicator which is a linear combination of categorical variables obtained from a multiple correspondence analysis (*cf.* Asselin, 2009). Variables considered in the analysis are the number of lands, animals and agricultural materials owned by the household. The community index is a linear combination of community basic infrastructure and access to market variables (roads, access to elementary or secondary schools, health centres, markets, electricity and cell phone). A score of income diversity has also been built from the various income sources earned by the household. As four main income sources are declared by the household, the income diversity variable (*ID*) is defined as $ID_i = \frac{1}{2}\left(1 - \sum_{k=1}^{4}\left(s_i^k\right)^2\right)$, where s_i^k is the share of the kth income source in total income of household *i*. This score equals 0 when only one source of income is declared by the household. It averages 0.17 in the studied population.

As reported in Table 2, many heads of household are working in agricultural activities (54%) and about one quarter of them have no job. Another important source of income is trade. Note also that about three quarters of households are land owners.

4. Results

Regression results

We use self-reported shocks in order to estimate their impact on consumption and income. Table 3 presents OLS estimates and GLLAMM estimates. Both models are estimated with log consumption and log income. Our preferred specification regroups a large set of explanatory variables such as household characteristics, regional dummies, community characteristics, interaction between household characteristics and community characteristics, shocks variables, interaction between shocks variables and household characteristics, interaction between shocks variables and community characteristics. Estimating the two-level linear random coefficient

model (GLLAMM) allows us to decompose the variance of the residuals into an idiosyncratic variance and a covariate variance.

In the regressions, shocks variables were regrouped into broad categories: idiosyncratic health shocks (disease/accident or death of a household member), idiosyncratic disease shocks (animal and crop diseases), new household member, loss of income (drop in wages, cessation of transfers from relatives/friends, loss of job or bankruptcy, equipment/tool breakdown), covariate climate shocks (cyclone, flood, drought and irregular rainfall), covariate health shocks (human epidemia), covariate economic shocks (increase in food prices, rarity of basic foodstuffs on the market, increase in seed prices, drop in relative agricultural prices, increase in fertilizer prices, drop in demand), health shocks (human epidemia), insecurity shocks (theft, kidnapping).

In Table 3, OLS estimates without shocks interacting with characteristics shows the mean impact of shocks. Their impact is generally negative except when hosting new household members (positive impact). In particular, idiosyncratic and covariate health shocks have large and significant negative effects on both consumption and income.

Regression results in Table 3 also help us characterizing vulnerable groups by differentiating the impact of shocks on well-being according to different household and community characteristics.

Idiosyncratic health shocks. The negative impact of this shock is reduced in absolute term when many households own lands in the community. This may be due to the fact that idiosyncratic health shock can be mutually insured within richer communities.

Idiosyncratic disease shocks. The significant positive parameter on the number of more than 15 years old household members shows that the idiosyncratic disease shock concerning crops or animals significantly increases the productivity of adults who may have to compensate for this kind of losses. In other words, the presence of a larger number of 15+ year old has a positive effect in reducing the impact from an animal/plant disease shock. Furthermore, idiosyncratic disease shock significantly decreases the benefits of income diversity for household economic well-being.

New household member. On the one hand, the positive impact of accommodating a new member in the household is reduced when the head of the household is higher educated or for greater diversity of income. The positive impact also decreases with the community index (access to basic infrastructures). On the other hand, the household benefit more from a new member in case of land ownership.

Loss of income. The negative impact of a loss of income appears to be significantly reduced when the head of the family is older.

Covariate climate shocks. The negative impact of covariate climate shock is significantly reduced with income diversity. The impact of this shock is further negative when many households own lands in the community.

Covariate health shocks. The negative impact of covariate health shock is significantly reduced when the household owns a land and when the head is older.

Covariate economic shocks. The negative impact of aggregate economic shock is significantly reduced when the household owns a land.

Insecurity shocks. The negative impact of insecurity shock is significantly increased in absolute term when the household owns a land. The impact of this shock is significantly less negative when many households own lands in the community.

Simulation results

Previous estimates of equation (9) with GLLAMM are used to simulate the impact of self-reported idiosyncratic and covariate shocks on both poverty and vulnerability. Table 4 presents the results. We define two poverty thresholds: one is chosen so that 80% of the households are poor; another one is chosen so that 40% are considered as *extremely* poor. What is more, a household is considered as vulnerable if the estimated vulnerability index is greater than the vulnerability threshold of 0.29. People are thus considered as vulnerable to poverty when they are more likely to fall into poverty in any period over two consecutive periods than to not be poor,

that is $(1–P)^2 \leq 0.5$, where P is the probability to fall below the poverty line. So, previous condition can be rewritten as $P \geq 0.29$.

For simulation purposes, the poverty line is chosen so that 80% (resp. 40%) of households have *expected* mean consumption/income below it. As a result, mean vulnerability appears to be respectively 63% and 46% for consumption and 67% and 45% for income. Using a vulnerability threshold of 0.29, vulnerability rates are respectively 98% and 87% for consumption and 96% and 76% for income.

Simulations exercises first consist in estimating the poverty rate and the vulnerability rate without observable idiosyncratic shocks (column 2 in Table 4) or without covariate shocks (column 3). As reported in Table 3, shocks which have the largest impact on consumption and income are health shocks, be they household or community shocks. So, most of the impact of observable shocks could be attributed to these particular shocks. On the contrary, loss of income has very little impact on poverty and vulnerability to poverty.

Without observable idiosyncratic shocks (column 2 in Table 4), the consumption-poverty rate falls to 28% and the consumption-extreme poverty rate is estimated to be 6%. So, the poverty gap, as it is defined by equation (3), corresponds to 52 percentage points, whereas the extreme poverty gap is 34 percentage points. Without observable covariate shocks, poverty decreases less: the poverty gap is 10 percentage points and the extreme poverty gap is 11 percentage points.

We also simulate the impact of observable shocks on the vulnerability rates. This impact is twofold: observable shocks have an impact on the mean (as stated in equation (6)) as well as on the variance of consumption/income (as stated in equation (7)). On the one hand, the percentage of households with mean consumption/income below the poverty line is what we call poverty induced vulnerability. On the other hand, the percentage of households with mean consumption/income above the poverty line that would fall into poverty due to consumption/income variability is what we call risk induced vulnerability.

Simulations results of the impact of shocks on vulnerability are as follows. Firstly, the impact of observable idiosyncratic shocks (in particular, observable idiosyncratic health shocks)

on the vulnerability rate is large, whereas covariate shocks have little impact on it. Without observable idiosyncratic shocks, the rate of vulnerability to poverty (resp. to extreme poverty) is estimated to be 64% (resp. 28%), compared to 98% (resp. 87%) with these shocks, which represents a 34 percentage points (resp. 58 percentage points) fall. Without observable covariate shocks, the rate of vulnerability to poverty (resp. extreme poverty) is estimated to be 95% (resp. 73%), compared to 98% (resp. 87%) with these shocks, which represents a 3 percentage points (resp. 14 percentage points) fall. We also have simulated the impact of idiosyncratic and covariate observable shocks on household income. The results are very similar to previous ones (see Table 4).

Secondly, Table 4 shows that observable idiosyncratic and covariate shocks have larger impact on the mean than on the variance of consumption/income. This is particularly true when considering observable idiosyncratic shocks. Indeed, the ratio between poverty induced and risk induced vulnerability that equals 4.42 with shocks is sharply decreased in the absence of observable shocks. This ratio is even lower without observable idiosyncratic shocks (0.35) than without observable idiosyncratic shocks (0.77). So, one possible interpretation of those results is that the main impact of shocks is to increase poverty permanently rather than transitorily.

Finally, one should estimate the impact of unobservable idiosyncratic or covariate shocks on vulnerability. By construction, unobservable shocks have no impact on mean consumption or mean income. However, they influence the variability of both consumption and income. So, we estimate vulnerability rates using either unobservable shocks or observable shocks as sources of consumption/income variability. Table 4 indicates that unobservable idiosyncratic shocks have more influence on households' vulnerability than unobservable covariate shocks. Indeed, 96% of households are vulnerable to unobservable idiosyncratic shocks (80% when considering vulnerability to extreme poverty), whereas they are 82% to be vulnerable to unobservable covariate shocks (44% when considering vulnerability to extreme poverty). By contrast, observable idiosyncratic shocks have the same influence on households' vulnerability than observable covariate shocks. Indeed, the ratio of idiosyncratic to covariate vulnerability is 1.00 (1.04 when considering vulnerability to extreme poverty) for observable shocks, whereas it is 1.17 (respectively 1.83) for unobservable shocks.

Regional profile

Previous results have shown that observable idiosyncratic shocks (in particular health related shocks) have large impact on vulnerability, especially poverty induced vulnerability. Furthermore, the variability of consumption or income induced by observable idiosyncratic shocks is comparable to that induced by observable covariate shocks. These findings suggest that households are not well insured against idiosyncratic shocks and, at least, not better than against covariate shocks. Idiosyncratic shocks may also have permanent impact on household living standard.

These findings are important for policy interventions in rural Haiti. However, as budget is necessarily constrained, programs should be targeted toward the most vulnerable people. To do so, regional profiles can provide valuable information on the impact of both idiosyncratic and covariate shocks on vulnerability, be they observable or unobservable shocks.

Tables 5 and 6 present such regional profiles. We first consider per capita consumption as an indicator of well-being (Table 5). We find that poverty induced vulnerability is generally higher when the poverty rate is high. This is the case in the North, North-east, Artibonite or Grand'Anse regions. In the West, Nippes, South and South-east regions it is lower. Observable idiosyncratic shocks have generally the same influence on households' vulnerability as observable covariate shocks in all the regions. Unobservable idiosyncratic shocks have more influence on households' vulnerability than unobservable covariate shocks in most of the regions, especially in the West, Nippes, South and South-east regions. Results are comparable when considering per capita income as an indicator of well-being (Table 6). Consequently, government and agencies should be conscious of these geographical disparities when designing policies and programs so that they could better target the poor and vulnerable people.

5. Discussion and Policy Implications

This paper aims at characterizing poverty and vulnerability in rural Haiti. Using a unique survey conducted in 2007 in rural areas, this paper decomposes vulnerability into several sources. Based on the estimates of a two-level linear random coefficient model the effects of shocks are

divided into idiosyncratic and covariate components, on the one hand, and observable and unobservable components on the other hand.

These sources of vulnerability can have various impacts upon households' well-being depending on (1) households' coping strategies, (2) self-insurance and (3) the ability of households in community to share risks. Empirical findings demonstrate that, overall, observable idiosyncratic shocks, in particular health related shocks, have larger impact upon vulnerability to poverty than observable covariate shocks. Also, observable shocks have greater impact on the expected mean rather than on the variance of consumption/income. That is, the main impact of those shocks is to increase poverty induced vulnerability rather than risk induced vulnerability. According to these results, poverty reduction policies may partly succeed in reducing vulnerability to poverty. However, due to the imperfect overlap between poverty and vulnerability, there is also scope for social programs and policies to focus on vulnerability and foster coping mechanisms.

One step further, focusing on the specific effects of shocks on the variability of consumption/income, we find that observable idiosyncratic and covariate shocks have comparable influence on well-being. Unobservable idiosyncratic shocks have generally more influence on households' vulnerability than unobservable covariate shocks. What is more, regional disparities exist and should be accounted for in order to better target vulnerable people in rural areas.

These findings raise important issues regarding the design of policies and programs aiming at reducing vulnerability in rural areas. Are insurance mechanisms really deficient or have idiosyncratic shocks particular adverse effects on income or assets? According to our results, households might not be fully able to share risks within communities or, at least, not more able than across communities. Several studies have already pointed out the fact that insurance mechanisms are insufficient in developing countries. A specificity of our study is that this interpretation is in line with the fact that many households in rural areas reported idiosyncratic health shocks as being the worst shocks they experienced. Nevertheless, rural households were more frequently hit by covariate shocks (such as increase of food prices, cyclone, flood, drought and irregular rainfall) in 2007. As these shocks are typically more frequent in rural areas,

households may also be better armed against them. As there are difficulties to disentangle the various causes of vulnerability, further studies based on other data on shocks and insurance mechanisms should thus be undertaken. A possible extension of our study could be to use such surveys at two points in time, before and after the 2010 earthquake in Haiti in order to assess vulnerability to poverty in this context and in relation with other shocks and threats faced by households and communities.

REFERENCES

Alderman, H., and Paxson, C. (1994) Do the poor insure? A synthesis of the literature on risk and consumption in developing countries, in: D. Bacha (ed.) *Economics in a Changing World*, vol. 4: Development, Trade and the Environment (London: Macmillan).

Asselin, L.-M. (2009) *Analysis of multidimensional poverty: Theory and case studies.* Springer.

Attanasio, O.P., and Rios Rull, J.-V. (2000) Consumption smoothing in island economies: Can public insurance reduce welfare? *European Economic Review*, 44(7), 1225-1258.

Calvo, C., and Dercon, S. (2005). Measuring individual vulnerability. Oxford Economics Discussion Paper, 229.

Carter, M.R., and Barrett, C.B. (2006) The economics of poverty traps and persistent poverty: An asset-based approach. *Journal of Development Studies*, 42(2), 178–199.

Chaudhuri S., Jalan, J. and Suryahadi, A. (2002) Assessing household vulnerability to poverty from cross-sectional data: a methodology and estimates from Indonesia. Discussion Paper 0102-02, Department of Economics, Columbia University.

Christiaensen, L., and Subbarao, K. (2005) Towards an understanding of vulnerability in rural Kenya. *Journal of African Economies*, 14(4), 520-558.

Cochrane, J.H. (1991) A Simple Test of Consumption Insurance. *Journal of Political Economy*, 99(5), 957–976.

Datt, G. and Hoogeveen, H. (2003) El Niño or El Peso? Crisis, poverty and income distribution in the Philippines. *World Development*, 31(7), 1103-1124.

Dercon, D. (2002) Income risk, coping strategies, and safety nets," *World Bank Research Observer*, 17(2), 141-166.

Dercon, S. and Krishnan, P. (2003) Risk sharing and public transfers. *Economic Journal*, 113(486), C86-C94.

Duclos, J-Y., Araar, A. and Giles, J. (2010). Chronic and transient poverty: Measurement and estimation, with evidence from China. Journal of development Economics, 91(2), 266-277.

Fafchamps, M., Udry, C. and Czukas, K. (1998) Drought and saving in West Africa: Are livestock a buffer stock? *Journal of Development Economics*, 55(2), 273-305.

Günther, I. and Harttgen, K. (2009) Estimating households vulnerability to idiosyncratic and covariate shocks: A novel method applied in Madagascar, *World Development*, 37(7), 1222-1234.

Hoddinott, J. (2006) Shocks and their consequences across and within households in rural Zimbabwe. *Journal of Development Studies*, 42(2), 301-321.

Kamanou, G. and Morduch, J. (2004). *Measuring vulnerability to poverty*. Oxford University Press, Oxford.

Kazianga, H., and Udry, C. (2006) Consumption smoothing? Livestock, insurance and drought in rural Burkina Faso. *Journal of Development Economics*, 79(2), 413-446.

Ligon, E., and Schechter, L. (2003). Measuring vulnerability. *Economic Journal*, 113(486), 95-102.

Mace, B.J. (1991) Full insurance in the presence of aggregate uncertainty. *Journal of Political Economy*, 99(5), 928–956.

Morduch, J. (1995) Income smoothing and consumption smoothing. *Journal of Economic Perspectives*, 9(3), 103-114.

Morduch, J. (1999) Between the state and the market: Can informal insurance patch the safety net? *World Bank Research Observer*, 14(2), 187-207.

Rabe-Hesketh, S. and Skrondal, A. (2008) Multilevel and longitudinal modeling using Stata. Stata Press.

Rosenzweig, M., and Wolpin, H. (1993) Credit market constraints, consumption smoothing, and the accumulation of durable production assets in low-income countries: investment in bullocks in India. *Journal of Political Economy*, 101(2), 223-244.

Suri, T. (2010) Estimating the extent of risk sharing between households. Working Paper, MIT.

Townsend, R.M. (1994) Risk and Insurance in Village India. *Econometrica*, 62(3), 539-91.

Townsend, R.M. (1995) Consumption insurance: An evaluation of risk-bearing systems in low-income economies. *Journal of Economic Perspectives*, 9(3), 83-102.

Zimmerman, F.J., and Carter, M.R. (2003) Asset smoothing, consumption smoothing and the reproduction of inequality under risk and subsistence constraints. *Journal of Development Economics*, 71(2), 233-260.

Table 1. Shocks in rural Haiti

%	% affected by this shock	Income only	Assets only	Both	% reporting this shock as the worst shock*	Income only	Assets only	Both
Increase in food prices	70.7	50.9	0.7	37.1	10.1	53.2	1.8	30.1
Cyclone, Flood	63.9	31.7	1.3	63.2	11.4	37.9	0.9	58.6
Drought	54.6	40.3	1.2	55.2	4.8	45.8	0.0	50.8
Irregular rainfall	49.6	37.9	0.3	56.7	1.7	27.3	2.3	63.6
Disease/Accident of a household member	47.6	42.0	1.1	54.1	30.8	41.2	1.0	51.8
Animal diseases	47.1	4.4	4.1	90.7	9.5	3.1	1.5	90.8
Crop diseases	37.6	45.1	0.5	51.6	4.5	38.8	0.9	54.3
Rarity of basic foodstuffs on the market	29.1	42.6	1.1	43.9	2.1	25.5	0.0	61.8
Increase in seed prices	27.7	48.5	0.8	42.0	1.0	51.9	0.0	29.6
Drop in relative agricultural prices	25.3	60.3	0.6	35.5	1.1	52.9	0.0	35.3
Drop in wages	22.6	48.6	0.6	47.9	1.6	25.5	0.0	63.8
Human epidemia	22.1	47.8	0.5	40.5	2.2	41.5	0.0	45.3
Death of an household member	21.9	33.0	0.4	63.3	11.7	30.6	0.7	64.2
Increase in fertilizer prices	12.9	43.9	1.0	44.5	0.9	56.3	0.0	31.3
Drop in demand	12.7	54.9	2.1	38.7	0.3	28.6	14.3	42.9
Insecurity (theft, kidnapping)	11.1	23.5	6.9	64.2	2.1	27.8	1.9	68.5
New household member	10.0	47.1	0.7	36.8	0.5	50.0	0.0	37.5
Cessation of transfers from relatives/friends	4.7	38.9	0.0	54.3	0.3	33.3	0.0	16.7
Loss of job or bankruptcy	3.9	39.0	1.5	57.6	0.9	35.0	0.0	50.0
Equipment, tool breakdown	2.7	49.7	1.6	27.5	0.0	100.0	0.0	0.0
Others	2.7	32.0	0.0	64.3	1.0	41.7	0.0	20.8

Source: Own computations using *Haitian Vulnerability and Food Security Survey*, 2007.
Notes: The sum of the three columns "income only", "assets only" and "both" do not sum to 100% due to non response or don't know or no impact. *Do not sum to 100% due to non response or don't know.

Table 2. Descriptive statistics

	Mean	SE
Household variables		
Log of consumption	7.30	1.06
Log of income	7.99	1.28
Agricultural index	0.24	0.13
Income diversity	0.17	0.13
Household size	5.2	2.3
Number of children	1.9	1.7
Age of head	49.8	16.4
Male head	0.71	0.45
Years of schooling (head)	2.6	3.8
Activity of head		
No job	0.23	0.42
Agroalimentary	0.54	0.50
Industry	0.03	0.18
Construction	0.00	0.05
Trade	0.12	0.33
Services	0.05	0.21
Other activity	0.03	0.17
Community variables		
Average years of schooling	4.0	1.6
Land owners	0.76	0.24
Community index	0.38	0.31

Source: Own computations using *Haitian Vulnerability and Food Security Survey*, 2007.

Table 3. Regression results

	Consumption (in log)						Income (in log)					
	OLS				GLLAMM		OLS				GLLAMM	
	Coeff.	P-value	Coeff.	P-value	Coeff.	P-value	Coeff.	P-value	Coeff.	P-value	Coeff.	P-value
Intercept	7.79	0.00	8.94	0.00	9.09	0.00	7.44	0.00	7.80	0.00	7.56	0.00
Household variables												
Agricultural index	-0.37	0.66	-0.12	0.89	-0.16	0.91	1.30	0.18	1.50	0.13	1.18	0.22
Number of adults over 50 years	-0.17	0.00	-0.10	0.06	-0.09	0.52	-0.13	0.03	-0.09	0.18	-0.08	0.19
Number of adults 25-50 years	-0.10	0.04	-0.04	0.47	-0.03	0.54	-0.10	0.09	-0.05	0.40	-0.05	0.39
Number of adults 15-24 years	-0.03	0.46	0.03	0.50	0.04	0.54	-0.07	0.16	-0.03	0.58	-0.03	0.57
Number of children 12-14 years	0.01	0.87	0.07	0.19	0.08	0.65	-0.12	0.03	-0.08	0.19	-0.07	0.25
Number of children 6-11 years	0.03	0.33	0.01	0.91	0.01	0.97	-0.04	0.19	-0.02	0.87	-0.02	0.84
Number of infants 3-5 years	0.03	0.36	0.00	0.99	0.00	1.00	-0.03	0.46	0.00	0.97	-0.01	0.96
Number of infants 0-2 years	-0.01	0.80	-0.01	0.89	-0.02	0.92	-0.05	0.24	-0.02	0.88	-0.02	0.84
Age of head	0.01	0.06	0.01	0.54	0.01	0.64	0.02	0.05	0.02	0.18	0.01	0.26
Age of head^2/100	-0.02	0.00	-0.02	0.00	-0.02	0.00	-0.02	0.00	-0.03	0.00	-0.02	0.00
Male head	0.00	0.93	-0.02	0.79	-0.04	0.55	0.12	0.06	0.15	0.04	0.14	0.05
Years of schooling of head	0.04	0.00	-0.02	0.49	-0.02	0.65	0.04	0.00	0.06	0.13	0.05	0.19
No job	0.16	0.02	0.16	0.03	0.14	0.59	0.16	0.05	0.17	0.04	0.16	0.07
Income diversity	0.25	0.00	0.33	0.06	0.34	0.30	0.57	0.00	0.64	0.00	0.60	0.00
Land owner	-0.09	0.62	-0.76	0.14	-0.68	0.51	0.05	0.80	-0.13	0.83	0.07	0.90
Region												
North West	-0.16	0.35	-0.27	0.14	-0.30	0.72	-0.37	0.06	-0.28	0.18	-0.33	0.13
North	-0.35	0.09	-0.39	0.08	-0.37	0.75	-0.02	0.93	0.27	0.31	0.25	0.35
North East	-0.98	0.00	-0.91	0.00	-0.90	0.22	-0.27	0.22	-0.23	0.35	-0.19	0.44
Artibonite	-0.31	0.03	-0.33	0.05	-0.36	0.30	0.31	0.06	0.31	0.12	0.29	0.16
Centre	-0.84	0.00	-0.63	0.00	-0.64	0.02	-0.13	0.54	-0.15	0.53	-0.17	0.50
West	0.13	0.42	0.03	0.87	-0.02	0.95	0.49	0.01	0.57	0.01	0.57	0.01
Grande'Anse	-0.85	0.00	-0.74	0.00	-0.79	0.45	-0.81	0.00	-0.68	0.00	-0.72	0.00
Nippes	-0.17	0.30	-0.11	0.58	-0.11	0.64	-0.07	0.72	-0.05	0.84	0.04	0.86
South	0.34	0.02	0.24	0.15	0.19	0.58	0.10	0.55	0.21	0.29	0.09	0.68
Southeast	ref		ref		ref		ref		ref		ref	
Community variables												
% Land owners	0.33	0.38	0.47	0.60	0.27	0.90	1.24	0.00	1.32	0.20	1.68	0.14
Community index	-0.17	0.17	-0.60	0.22	-0.55	0.77	-0.09	0.50	-0.72	0.21	-0.49	0.49
Average years of schooling	0.06	0.07	0.06	0.06	0.06	0.51	0.03	0.38	0.06	0.15	0.04	0.27

*Household * Community variables*												
Average years of schooling * Agricultural index	0.17	0.08	0.20	0.04	0.20	0.24	0.01	0.92	0.02	0.89	0.04	0.70
Average years of schooling * Number of children	0.00	0.55	0.00	0.92	0.00	0.87	0.00	0.88	0.00	0.91	0.00	0.89
% Land owner * Agricultural index	-1.03	0.12	-1.44	0.04	-1.45	0.17	-2.11	0.01	-2.32	0.01	-2.05	0.01
% Land owner * Household size	-0.04	0.19	-0.10	0.01	-0.10	0.49	-0.11	0.00	-0.17	0.00	-0.16	0.00
% Land owner * Age of head	0.01	0.09	0.01	0.41	0.01	0.56	0.01	0.18	0.00	0.95	0.00	0.71
Community index * Agricultural index	0.60	0.19	0.48	0.31	0.54	0.31	0.54	0.31	0.45	0.42	0.41	0.45
Shock variables												
Idiosyncratic health shock	-1.31	0.00	-3.17	0.00	-3.18	0.27	-1.66	0.00	-4.13	0.00	-4.12	0.00
Idiosyncratic disease shock	-0.64	0.12	-0.18	0.83	-0.25	0.93	-1.34	0.00	-1.70	0.09	-1.63	0.11
New household member	1.72	0.03	6.33	0.01	5.51	0.61	3.54	0.00	8.57	0.00	8.46	0.00
Loss of income	0.12	0.76	-2.19	0.02	-1.88	0.38	0.08	0.86	-0.04	0.97	-0.64	0.57
Covariate climate shock	0.25	0.35	-0.51	0.55	-0.62	0.70	0.27	0.37	1.70	0.08	1.78	0.08
Covariate health shock	-1.43	0.02	-3.75	0.03	-3.40	0.71	-1.40	0.04	-1.79	0.36	-0.96	0.66
Covariate economic shock	-0.53	0.12	0.04	0.97	-0.13	0.97	-0.34	0.39	-1.55	0.23	-1.09	0.44
Insecurity shock	-0.59	0.08	-2.20	0.10	-2.15	0.76	-0.47	0.22	-2.16	0.16	-2.18	0.16
*Shock * Household variables*												
Idiosyncratic health shock * Nb adults 15 and more			-0.07	0.51	-0.07	0.53			0.04	0.73	0.04	0.75
Idiosyncratic health shock * Age of head			-0.01	0.62	0.00	0.77			0.00	0.92	0.00	0.88
Idiosyncratic health shock * Years of schooling of head			0.07	0.15	0.07	0.16			0.02	0.72	0.04	0.45
Idiosyncratic health shock * Income diversity			-0.07	0.71	-0.05	0.87			0.19	0.39	0.28	0.20
Idiosyncratic health shock * Land owner			0.64	0.21	0.60	0.55			-0.23	0.70	-0.30	0.62
Idiosyncratic disease shock * Nb adults 15 and more			0.30	0.00	0.30	0.00			0.33	0.00	0.32	0.00
Idiosyncratic disease shock * Age of head			-0.01	0.35	-0.01	0.28			-0.01	0.39	-0.01	0.26
Idiosyncratic disease shock * Years of schooling of head			0.03	0.41	0.02	0.75			-0.04	0.25	-0.04	0.26
Idiosyncratic disease shock * Income diversity			-0.48	0.00	-0.47	0.00			-0.16	0.35	-0.11	0.52
Idiosyncratic disease shock * Land owner			0.85	0.10	0.80	0.63			0.58	0.33	0.46	0.44
New household member * Nb adults 15 and more			0.16	0.40	0.14	0.64			0.21	0.32	0.26	0.23
New household member * Age of head			-0.04	0.10	-0.03	0.46			-0.03	0.22	-0.03	0.26
New household member * Years of schooling of head			-0.21	0.04	-0.22	0.21			-0.11	0.34	-0.11	0.36
New household member * Income diversity			-0.77	0.07	-0.81	0.17			-1.53	0.00	-1.48	0.00
New household member * Land owner			0.31	0.80	0.21	0.87			2.60	0.07	3.12	0.03
Loss of income * Nb adults 15 and more			0.03	0.73	0.07	0.87			0.02	0.86	0.04	0.73
Loss of income * Age of head			0.03	0.00	0.03	0.01			0.01	0.34	0.01	0.27
Loss of income * Years of schooling of head			0.03	0.56	0.02	0.68			-0.02	0.71	-0.01	0.76
Loss of income * Income diversity			0.32	0.10	0.35	0.32			0.02	0.93	0.03	0.90
Loss of income * Land owner			0.50	0.27	0.50	0.32			-0.72	0.17	-0.50	0.35
Covariate climate shock * Nb adults 15 and more			-0.16	0.12	-0.19	0.38			-0.22	0.06	-0.22	0.06

Covariate climate shock * Age of head	0.01	0.15	0.02	0.54	0.00	0.90	0.00	0.94
Covariate climate shock * Years of schooling of head	-0.03	0.48	-0.02	0.78	-0.03	0.44	-0.04	0.39
Covariate climate shock * Income diversity	0.54	0.00	0.52	0.34	0.13	0.53	0.12	0.57
Covariate climate shock * Land owner	-0.71	0.10	-0.71	0.61	1.02	0.04	1.06	0.04
Covariate health shock * Nb adults 15 and more	0.06	0.71	0.02	0.95	-0.05	0.80	-0.02	0.92
Covariate health shock * Age of head	0.03	0.04	0.03	0.12	0.01	0.63	0.01	0.64
Covariate health shock * Years of schooling of head	0.04	0.60	0.04	0.60	-0.08	0.34	-0.10	0.23
Covariate health shock * Income diversity	0.61	0.05	0.62	0.07	0.90	0.01	0.68	0.07
Covariate health shock * Land owner	0.54	0.49	0.55	0.62	-0.27	0.77	0.11	0.91
Covariate economic shock * Nb adults 15 and more	-0.03	0.83	0.02	0.85	-0.08	0.56	-0.10	0.48
Covariate economic shock * Age of head	0.00	0.98	0.00	0.85	0.02	0.27	0.01	0.31
Covariate economic shock * Years of schooling of head	0.06	0.25	0.05	0.35	0.07	0.21	0.07	0.20
Covariate economic shock * Income diversity	-0.30	0.15	-0.32	0.13	-0.26	0.29	-0.23	0.34
Covariate economic shock * Land owner	0.48	0.37	0.56	0.33	-0.84	0.16	-1.21	0.05
Insecurity shock * Nb adults 15 and more	0.14	0.23	0.15	0.37	-0.03	0.82	-0.01	0.94
Insecurity shock * Age of head	0.01	0.30	0.02	0.30	0.02	0.08	0.03	0.07
Insecurity shock * Years of schooling of head	-0.02	0.77	-0.01	0.90	-0.04	0.55	-0.03	0.60
Insecurity shock * Income diversity	-0.33	0.20	-0.32	0.66	-0.18	0.55	-0.25	0.40
Insecurity shock * Land owner	-1.58	0.01	-1.60	0.28	-0.81	0.27	-0.82	0.26
*Shock * Community variables*								
Idiosyncratic health shock * % Land owners	2.45	0.01	2.50	0.02	2.46	0.03	2.48	0.04
Idiosyncratic health shock * Community index	0.01	0.98	-0.15	0.83	0.46	0.44	0.33	0.62
Idiosyncratic disease shock * % Land owners	-1.11	0.11	-1.16	0.66	-0.03	0.97	-0.04	0.96
Idiosyncratic disease shock * Community index	-0.30	0.47	-0.30	0.61	-0.29	0.56	-0.36	0.48
New household member * % Land owners	-1.30	0.55	-0.90	0.91	-1.59	0.53	-2.99	0.25
New household member * Community index	-2.57	0.04	-2.49	0.05	-1.90	0.18	-2.09	0.16
Loss of income * % Land owners	-0.43	0.64	-0.77	0.69	-0.88	0.40	-0.46	0.68
Loss of income * Community index	0.47	0.40	0.47	0.67	0.70	0.28	1.07	0.12
Covariate climate shock * % Land owners	-0.14	0.86	0.07	0.98	-2.40	0.01	-2.71	0.01
Covariate climate shock * Community index	0.64	0.21	0.62	0.57	0.97	0.10	0.96	0.13
Covariate health shock * % Land owners	-1.16	0.44	-1.27	0.92	-2.25	0.19	-2.93	0.14
Covariate health shock * Community index	0.57	0.52	0.80	0.51	0.88	0.39	0.78	0.54
Covariate economic shock * % Land owners	-0.21	0.84	-0.03	1.00	2.20	0.08	2.04	0.14
Covariate economic shock * Community index	0.22	0.73	0.18	0.94	-0.46	0.53	-0.71	0.42
Insecurity shock * % Land owners	3.17	0.01	2.81	0.53	2.03	0.14	1.85	0.18
Insecurity shock * Community index	-0.10	0.89	-0.05	0.98	0.23	0.78	0.46	0.60
Idiosyncratic variance			0.69	0.00			0.92	0.00
Covariate variance			0.03	0.93			1.69	0.02

Number of households	2585	2585	2585	2612	2612	2612
Number of communities	228	228	228	228	228	228
R2 or Pseudo-R2	0.32	0.36	0.92	0.36	0.38	0.91

Source: Own computations using *Haitian Vulnerability and Food Security Survey*, 2007.

Table 4. Vulnerability decomposition and simulations

	Consumption			Income		
	Factual (1)	Without idiosyncratic shocks (2)	Without covariate shocks (3)	Factual (1)	Without idiosyncratic shocks (2)	Without covariate shocks (3)
Poverty rate*	0.80	0.28	0.70	0.80	0.24	0.63
Mean vulnerability	0.63	0.39	0.58	0.67	0.33	0.57
Vulnerability rate**	0.98	0.64	0.95	0.96	0.46	0.86
Poverty induced vulnerability	0.80	0.28	0.70	0.80	0.24	0.63
Risk induced vulnerability	0.18	0.36	0.25	0.16	0.22	0.23
Poverty induced/Risk induced vulnerability	4.42	0.77	2.75	5.11	1.10	2.70
Idiosyncratic vulnerability (unobserved)	0.96	0.60	0.94	0.92	0.41	0.83
Covariate vulnerability (unobserved)	0.82	0.30	0.73	0.82	0.27	0.67
Idiosyncratic/Covariate vulnerability (unobserved)	1.17	2.01	1.28	1.12	1.56	1.22
Idiosyncratic vulnerability (observed)	0.91	0.00	0.84	0.89	0.00	0.77
Covariate vulnerability (observed)	0.91	0.39	0.00	0.90	0.32	0.00
Idiosyncratic/Covariate vulnerability (unobserved)	1.00	-	-	1.00	-	-
(Extreme) Poverty rate*	0.40	0.06	0.29	0.40	0.06	0.24
Mean vulnerability	0.46	0.23	0.41	0.45	0.16	0.34
Vulnerability rate**	0.87	0.28	0.73	0.76	0.17	0.54
Poverty induced vulnerability	0.40	0.06	0.29	0.40	0.06	0.24
Risk induced vulnerability	0.47	0.22	0.44	0.36	0.11	0.30
Poverty induced/Risk induced vulnerability	0.85	0.29	0.66	1.10	0.60	0.82
Idiosyncratic vulnerability (unobserved)	0.80	0.27	0.69	0.66	0.15	0.47
Covariate vulnerability (unobserved)	0.44	0.07	0.32	0.45	0.08	0.28
Idiosyncratic/Covariate vulnerability (unobserved)	1.83	3.79	2.16	1.47	1.93	1.68
Idiosyncratic vulnerability (observed)	0.62	0.00	0.50	0.58	0.00	0.40
Covariate vulnerability (observed)	0.60	0.11	0.00	0.56	0.10	0.00
Idiosyncratic/Covariate vulnerability (observed)	1.04	-	-	1.03	-	-

Source: Own computations using *Haitian Vulnerability and Food Security Survey*, 2007.

Notes: *The poverty line is chosen so that 80% (resp. 40%) of households have expected mean consumption below it. The poverty rate is the percentage of households whose expected mean consumption is below the poverty line. **The vulnerability threshold is 29%.

Table 5. Regional profile (consumption)

	North-West			North			North-East			Artibonite			Centre		
	(1)	(2)	(3)	(1)	(2)	(3)	(1)	(2)	(3)	(1)	(2)	(3)	(1)	(2)	(3)
Poverty rate*	0.83	0.29	0.79	0.98	0.48	0.84	1.00	0.74	0.97	0.97	0.36	0.83	0.93	0.28	0.88
Mean vulnerability	0.63	0.41	0.60	0.75	0.50	0.62	0.79	0.60	0.74	0.72	0.45	0.63	0.65	0.42	0.64
Vulnerability rate	1.00	0.70	0.97	1.00	0.84	0.99	1.00	0.97	1.00	1.00	0.81	1.00	1.00	0.79	1.00
Poverty induced vulnerability	0.83	0.29	0.79	0.98	0.48	0.84	1.00	0.74	0.97	0.97	0.36	0.83	0.93	0.28	0.88
Risk induced vulnerability	0.17	0.41	0.19	0.02	0.36	0.15	0.00	0.23	0.03	0.03	0.46	0.17	0.07	0.51	0.12
Poverty induced/Risk induced vulnerability	*4.84*	*0.72*	*4.22*	*39.25*	*1.35*	*5.45*	-	*3.14*	*38.94*	*36.87*	*0.78*	*5.03*	*13.38*	*0.56*	*7.04*
Idiosyncratic vulnerability (unobserved)	1.00	0.62	0.96	1.00	0.82	0.98	1.00	0.95	1.00	1.00	0.77	0.99	1.00	0.77	1.00
Covariate vulnerability (unobserved)	0.85	0.30	0.81	0.98	0.55	0.85	1.00	0.77	0.99	0.98	0.38	0.87	0.95	0.30	0.90
Idiosyncratic/Covariate vulnerability (unobserved)	*1.18*	*2.07*	*1.18*	*1.03*	*1.49*	*1.16*	*1.00*	*1.24*	*1.01*	*1.02*	*2.02*	*1.14*	*1.05*	*2.55*	*1.11*
Idiosyncratic vulnerability (observed)	0.95	0.00	0.94	1.00	0.00	0.98	1.00	0.00	1.00	1.00	0.00	0.97	0.97	0.00	0.94
Covariate vulnerability (observed)	0.97	0.42	0.00	0.99	0.67	0.00	1.00	0.87	0.00	1.00	0.52	0.00	0.96	0.38	0.00
Idiosyncratic/Covariate vulnerability (observed)	*0.98*	-	-	*1.01*	-	-	*1.00*	-	-	*1.00*	-	-	*1.01*	-	-
(Extreme) Poverty rate*	0.36	0.07	0.29	0.71	0.12	0.33	0.94	0.33	0.73	0.67	0.07	0.33	0.51	0.02	0.41
Mean vulnerability	0.46	0.25	0.42	0.59	0.30	0.43	0.65	0.43	0.58	0.55	0.27	0.45	0.49	0.26	0.47
Vulnerability rate	0.92	0.29	0.81	0.98	0.46	0.82	1.00	0.83	0.98	0.99	0.36	0.88	0.95	0.34	0.90
Poverty induced vulnerability	0.36	0.07	0.29	0.71	0.12	0.33	0.94	0.33	0.73	0.67	0.07	0.33	0.51	0.02	0.41
Risk induced vulnerability	0.57	0.22	0.51	0.27	0.34	0.48	0.06	0.50	0.26	0.32	0.29	0.54	0.44	0.32	0.49
Poverty induced/Risk induced vulnerability	*0.63*	*0.30*	*0.58*	*2.67*	*0.36*	*0.68*	-	*0.65*	*2.82*	*2.05*	*0.23*	*0.61*	*1.15*	*0.07*	*0.84*
Idiosyncratic vulnerability (unobserved)	0.84	0.27	0.75	0.95	0.41	0.78	1.00	0.81	0.98	0.97	0.33	0.82	0.95	0.33	0.89
Covariate vulnerability (unobserved)	0.41	0.07	0.33	0.75	0.13	0.38	0.95	0.35	0.76	0.71	0.07	0.36	0.58	0.03	0.45
Idiosyncratic/Covariate vulnerability (unobserved)	*2.06*	*3.97*	*2.26*	*1.28*	*3.22*	*2.05*	*1.06*	*2.28*	*1.29*	*1.36*	*4.87*	*2.26*	*1.65*	*10.07*	*1.98*
Idiosyncratic vulnerability (observed)	0.62	0.00	0.59	0.91	0.00	0.66	0.98	0.00	0.86	0.89	0.00	0.61	0.74	0.00	0.65
Covariate vulnerability (observed)	0.61	0.13	0.00	0.87	0.19	0.00	0.98	0.49	0.00	0.85	0.11	0.00	0.67	0.04	0.00
Idiosyncratic/Covariate vulnerability (observed)	*1.01*	-	-	*1.05*	-	-	*1.00*	-	-	*1.04*	-	-	*1.11*	-	-

Source: Own computations using *Haitian Vulnerability and Food Security Survey*, 2007.

Notes: *The poverty line is chosen so that 80% (resp. 40%) of households have expected mean consumption below it. The poverty rate is the percentage of households whose expected mean consumption is below the poverty line. **The vulnerability threshold is 29%. Note: (1) Factual (2) Without idiosyncratic self-reported shocks (3) Without covariate self-reported shocks.

Table 5 (bis). Regional profile (consumption)

	West			Grand'Anse			Nippes			South			Southeast		
	(1)	(2)	(3)	(1)	(2)	(3)	(1)	(2)	(3)	(1)	(2)	(3)	(1)	(2)	(3)
Poverty rate*	0.58	0.10	0.39	0.95	0.58	0.92	0.77	0.19	0.59	0.56	0.14	0.36	0.66	0.05	0.83
Mean vulnerability	0.52	0.27	0.46	0.71	0.57	0.70	0.57	0.35	0.52	0.52	0.31	0.46	0.55	0.22	0.65
Vulnerability rate	0.93	0.41	0.89	1.00	0.88	1.00	1.00	0.59	0.97	0.96	0.47	0.79	0.97	0.25	1.00
Poverty induced vulnerability	0.58	0.10	0.39	0.95	0.58	0.92	0.77	0.19	0.59	0.56	0.14	0.36	0.66	0.05	0.83
Risk induced vulnerability	0.36	0.30	0.50	0.05	0.31	0.08	0.23	0.40	0.38	0.41	0.33	0.43	0.31	0.20	0.17
Poverty induced/Risk induced vulnerability	*1.63*	*0.34*	*0.77*	*17.35*	*1.86*	*11.98*	*3.39*	*0.47*	*1.53*	*1.37*	*0.42*	*0.86*	*2.14*	*0.24*	*5.00*
Idiosyncratic vulnerability (unobserved)	0.89	0.37	0.85	1.00	0.85	1.00	0.99	0.56	0.96	0.92	0.44	0.74	0.93	0.18	0.98
Covariate vulnerability (unobserved)	0.61	0.11	0.42	0.96	0.61	0.94	0.83	0.20	0.65	0.58	0.16	0.40	0.72	0.05	0.85
Idiosyncratic/Covariate vulnerability (unobserved)	*1.45*	*3.41*	*2.01*	*1.04*	*1.39*	*1.06*	*1.20*	*2.76*	*1.49*	*1.58*	*2.67*	*1.85*	*1.28*	*3.74*	*1.16*
Idiosyncratic vulnerability (observed)	0.76	0.00	0.60	0.99	0.00	0.99	0.90	0.00	0.79	0.80	0.00	0.57	0.85	0.00	0.96
Covariate vulnerability (observed)	0.75	0.17	0.00	1.00	0.73	0.00	0.91	0.26	0.00	0.78	0.22	0.00	0.90	0.12	0.00
Idiosyncratic/Covariate vulnerability (observed)	*1.01*	-	-	*0.99*	-	-	*0.99*	-	-	*1.03*	-	-	*0.95*	-	-
(Extreme) Poverty rate*	0.13	0.02	0.09	0.50	0.18	0.43	0.12	0.01	0.10	0.10	0.02	0.14	0.18	0.00	0.45
Mean vulnerability	0.36	0.16	0.30	0.51	0.36	0.47	0.40	0.20	0.35	0.33	0.15	0.29	0.40	0.11	0.47
Vulnerability rate	0.71	0.13	0.48	0.96	0.56	0.88	0.90	0.21	0.68	0.62	0.09	0.37	0.83	0.04	0.86
Poverty induced vulnerability	0.13	0.02	0.09	0.50	0.18	0.43	0.12	0.01	0.10	0.10	0.02	0.14	0.18	0.00	0.45
Risk induced vulnerability	0.59	0.11	0.38	0.46	0.37	0.45	0.78	0.20	0.58	0.52	0.08	0.24	0.65	0.03	0.42
Poverty induced/Risk induced vulnerability	*0.21*	*0.22*	*0.25*	*1.09*	*0.50*	*0.96*	*0.15*	*0.03*	*0.17*	*0.19*	*0.25*	*0.58*	*0.27*	*0.14*	*1.07*
Idiosyncratic vulnerability (unobserved)	0.61	0.12	0.42	0.91	0.53	0.85	0.83	0.19	0.64	0.47	0.08	0.34	0.70	0.04	0.83
Covariate vulnerability (unobserved)	0.16	0.03	0.11	0.56	0.22	0.47	0.16	0.01	0.12	0.12	0.02	0.15	0.22	0.00	0.48
Idiosyncratic/Covariate vulnerability (unobserved)	*3.70*	*4.50*	*3.92*	*1.63*	*2.43*	*1.82*	*5.19*	*36.42*	*5.57*	*4.01*	*3.56*	*2.26*	*3.21*	*8.20*	*1.73*
Idiosyncratic vulnerability (observed)	0.34	0.00	0.21	0.73	0.00	0.71	0.38	0.00	0.27	0.30	0.00	0.20	0.51	0.00	0.66
Covariate vulnerability (observed)	0.33	0.06	0.00	0.70	0.24	0.00	0.38	0.03	0.00	0.24	0.03	0.00	0.54	0.01	0.00
Idiosyncratic/Covariate vulnerability (observed)	*1.04*	-	-	*1.04*	-	-	*0.99*	-	-	*1.25*	-	-	*0.95*	-	-

Source: Own computations using *Haitian Vulnerability and Food Security Survey*, 2007.
Notes: *The poverty line is chosen so that 80% (resp. 40%) of households have expected mean consumption below it. The poverty rate is the percentage of households whose expected mean consumption is below the poverty line. **The vulnerability threshold is 29%. Note: (1) Factual (2) Without idiosyncratic self-reported shocks (3) Without covariate self-reported shocks.

Table 6. Regional profile (income)

	North-West			North			North-East			Artibonite			Centre		
	(1)	(2)	(3)	(1)	(2)	(3)	(1)	(2)	(3)	(1)	(2)	(3)	(1)	(2)	(3)
Poverty rate*	0.94	0.45	0.88	0.98	0.35	0.89	0.96	0.43	0.74	0.92	0.21	0.66	0.74	0.11	0.63
Mean vulnerability	0.73	0.48	0.68	0.84	0.40	0.72	0.77	0.47	0.63	0.72	0.30	0.58	0.62	0.23	0.58
Vulnerability rate	1.00	0.72	0.98	1.00	0.63	0.99	1.00	0.67	0.90	0.99	0.40	0.92	0.94	0.30	0.90
Poverty induced vulnerability	0.94	0.45	0.88	0.98	0.35	0.89	0.96	0.43	0.74	0.92	0.21	0.66	0.74	0.11	0.63
Risk induced vulnerability	0.06	0.27	0.10	0.02	0.28	0.10	0.04	0.24	0.16	0.07	0.19	0.27	0.20	0.19	0.27
Poverty induced/Risk induced vulnerability	*16.30*	*1.63*	*8.92*	*65.49*	*1.27*	*9.38*	-	*1.80*	*4.57*	*13.68*	*1.12*	*2.48*	*3.71*	*0.56*	*2.32*
Idiosyncratic vulnerability (unobserved)	0.98	0.65	0.97	0.99	0.60	0.98	0.98	0.59	0.89	0.97	0.35	0.89	0.90	0.26	0.85
Covariate vulnerability (unobserved)	0.95	0.48	0.90	0.99	0.40	0.91	0.97	0.46	0.79	0.93	0.24	0.73	0.77	0.13	0.67
Idiosyncratic/Covariate vulnerability (unobserved)	*1.04*	*1.37*	*1.07*	*1.00*	*1.51*	*1.08*	*1.02*	*1.29*	*1.12*	*1.05*	*1.49*	*1.22*	*1.17*	*2.01*	*1.26*
Idiosyncratic vulnerability (observed)	0.98	0.00	0.95	0.99	0.00	0.96	0.98	0.00	0.87	0.96	0.00	0.84	0.88	0.00	0.80
Covariate vulnerability (observed)	0.98	0.58	0.00	0.99	0.45	0.00	1.00	0.57	0.00	0.96	0.28	0.00	0.84	0.14	0.00
Idiosyncratic/Covariate vulnerability (observed)	*0.99*	-	-	*1.00*	-	-	*0.98*	-	-	*1.01*	-	-	*1.04*	-	-
(Extreme) Poverty rate*	0.60	0.16	0.41	0.87	0.07	0.52	0.53	0.14	0.23	0.47	0.03	0.17	0.22	0.02	0.22
Mean vulnerability	0.55	0.29	0.46	0.68	0.19	0.50	0.51	0.23	0.33	0.49	0.12	0.32	0.37	0.08	0.32
Vulnerability rate	0.95	0.41	0.84	0.98	0.23	0.84	0.90	0.29	0.54	0.87	0.12	0.53	0.64	0.06	0.52
Poverty induced vulnerability	0.60	0.16	0.41	0.87	0.07	0.52	0.53	0.14	0.23	0.47	0.03	0.17	0.22	0.02	0.22
Risk induced vulnerability	0.35	0.25	0.44	0.11	0.16	0.32	0.37	0.16	0.31	0.40	0.09	0.36	0.42	0.04	0.30
Poverty induced/Risk induced vulnerability	*1.70*	*0.64*	*0.93*	*8.26*	*0.41*	*1.62*	-	*0.87*	*0.74*	*1.20*	*0.33*	*0.47*	*0.52*	*0.48*	*0.73*
Idiosyncratic vulnerability (unobserved)	0.86	0.37	0.77	0.96	0.21	0.77	0.79	0.24	0.46	0.74	0.11	0.42	0.51	0.06	0.44
Covariate vulnerability (unobserved)	0.64	0.20	0.44	0.91	0.09	0.61	0.62	0.15	0.28	0.54	0.05	0.19	0.28	0.03	0.25
Idiosyncratic/Covariate vulnerability (unobserved)	*1.35*	*1.83*	*1.74*	*1.06*	*2.30*	*1.26*	*1.26*	*1.57*	*1.67*	*1.39*	*2.20*	*2.17*	*1.81*	*2.26*	*1.78*
Idiosyncratic vulnerability (observed)	0.77	0.00	0.64	0.95	0.00	0.75	0.75	0.00	0.40	0.69	0.00	0.38	0.43	0.00	0.35
Covariate vulnerability (observed)	0.80	0.24	0.00	0.95	0.11	0.00	0.76	0.19	0.00	0.65	0.07	0.00	0.34	0.03	0.00
Idiosyncratic/Covariate vulnerability (observed)	*0.96*	-	-	*1.00*	-	-	*0.98*	-	-	*1.05*	-	-	*1.26*	-	-

Source: Own computations using *Haitian Vulnerability and Food Security Survey*, 2007.

Notes: *The poverty line is chosen so that 80% (resp. 40%) of households have expected mean consumption below it. The poverty rate is the percentage of households whose expected mean consumption is below the poverty line. **The vulnerability threshold is 29%. Note: (1) Factual (2) Without idiosyncratic self-reported shocks (3) Without covariate self-reported shocks.

Table 6 (bis). Regional profile (income)

	West			Grand'Anse			Nippes			South			Southeast		
	(1)	(2)	(3)	(1)	(2)	(3)	(1)	(2)	(3)	(1)	(2)	(3)	(1)	(2)	(3)
Poverty rate*	0.53	0.10	0.25	0.95	0.49	0.91	0.67	0.28	0.39	0.87	0.37	0.64	0.75	0.07	0.84
Mean vulnerability	0.51	0.23	0.36	0.77	0.52	0.74	0.57	0.35	0.43	0.69	0.42	0.57	0.62	0.23	0.70
Vulnerability rate	0.86	0.29	0.63	0.99	0.73	0.97	0.92	0.47	0.71	0.98	0.66	0.89	0.99	0.27	0.98
Poverty induced vulnerability	0.53	0.10	0.25	0.95	0.49	0.91	0.67	0.28	0.39	0.87	0.37	0.64	0.75	0.07	0.84
Risk induced vulnerability	0.34	0.19	0.38	0.04	0.23	0.06	0.25	0.19	0.32	0.11	0.28	0.24	0.24	0.21	0.14
Poverty induced/Risk induced vulnerability	*1.56*	*0.52*	*0.66*	*22.76*	*2.12*	*15.17*	*2.72*	*1.46*	*1.21*	*7.57*	*1.31*	*2.64*	*3.08*	*0.32*	*5.95*
Idiosyncratic vulnerability (unobserved)	0.78	0.25	0.54	0.99	0.68	0.97	0.87	0.43	0.68	0.98	0.60	0.87	0.92	0.23	0.96
Covariate vulnerability (unobserved)	0.57	0.11	0.30	0.95	0.52	0.93	0.71	0.29	0.44	0.90	0.41	0.68	0.79	0.08	0.87
Idiosyncratic/Covariate vulnerability (unobserved)	*1.38*	*2.18*	*1.79*	*1.04*	*1.32*	*1.04*	*1.23*	*1.47*	*1.57*	*1.09*	*1.46*	*1.27*	*1.17*	*2.98*	*1.10*
Idiosyncratic vulnerability (observed)	0.71	0.00	0.45	0.98	0.00	0.96	0.78	0.00	0.56	0.96	0.00	0.79	0.89	0.00	0.93
Covariate vulnerability (observed)	0.70	0.16	0.00	0.99	0.58	0.00	0.84	0.34	0.00	0.96	0.47	0.00	0.94	0.13	0.00
Idiosyncratic/Covariate vulnerability (observed)	*1.01*	-	-	*0.99*	-	-	*0.93*	-	-	*1.00*	-	-	*0.95*	-	-
(Extreme) Poverty rate*	0.12	0.03	0.01	0.51	0.20	0.47	0.19	0.07	0.05	0.42	0.10	0.25	0.36	0.01	0.49
Mean vulnerability	0.30	0.10	0.17	0.52	0.28	0.46	0.34	0.18	0.21	0.47	0.23	0.36	0.43	0.10	0.48
Vulnerability rate	0.48	0.07	0.18	0.88	0.35	0.78	0.58	0.20	0.28	0.85	0.27	0.57	0.76	0.06	0.78
Poverty induced vulnerability	0.12	0.03	0.01	0.51	0.20	0.47	0.19	0.07	0.05	0.42	0.10	0.25	0.36	0.01	0.49
Risk induced vulnerability	0.36	0.04	0.16	0.37	0.16	0.31	0.39	0.14	0.23	0.43	0.18	0.32	0.40	0.04	0.29
Poverty induced/Risk induced vulnerability	*0.33*	*0.76*	*0.08*	*1.38*	*1.23*	*1.51*	*0.49*	*0.48*	*0.22*	*0.98*	*0.54*	*0.79*	*0.89*	*0.34*	*1.67*
Idiosyncratic vulnerability (unobserved)	0.35	0.06	0.12	0.79	0.32	0.72	0.49	0.19	0.23	0.79	0.24	0.50	0.66	0.05	0.74
Covariate vulnerability (unobserved)	0.14	0.03	0.03	0.58	0.22	0.52	0.23	0.10	0.06	0.48	0.12	0.27	0.41	0.02	0.54
Idiosyncratic/Covariate vulnerability (unobserved)	*2.44*	*2.15*	*3.68*	*1.37*	*1.44*	*1.39*	*2.15*	*1.99*	*3.77*	*1.65*	*2.06*	*1.83*	*1.61*	*2.74*	*1.37*
Idiosyncratic vulnerability (observed)	0.28	0.00	0.08	0.70	0.00	0.64	0.30	0.00	0.12	0.66	0.00	0.39	0.60	0.00	0.69
Covariate vulnerability (observed)	0.25	0.04	0.00	0.71	0.25	0.00	0.30	0.13	0.00	0.62	0.14	0.00	0.64	0.03	0.00
Idiosyncratic/Covariate vulnerability (observed)	*1.14*	-	-	*0.99*	-	-	*1.01*	-	-	*1.06*	-	-	*0.94*	-	-

Source: Own computations using *Haitian Vulnerability and Food Security Survey*, 2007.
Notes: *The poverty line is chosen so that 80% (resp. 40%) of households have expected mean consumption below it. The poverty rate is the percentage of households whose expected mean consumption is below the poverty line. **The vulnerability threshold is 29%. Note: (1) Factual (2) Without idiosyncratic self-reported shocks (3) Without covariate self-reported shocks.
