# Supplementary material for: Systematic Review of Methods in Low-Consensus Fields: Supporting Commensuration through `Construct-Centered Methods Aggregation’ in the Case of Climate Change Vulnerability Research
Source: PLoS One. 2016 Feb 22;11(2):e0149071. doi: 10.1371/journal.pone.0149071 (PMC4762661; doi:10.1371/journal.pone.0149071)
Supplement: S2 File — Source articles and bibliography. (ZIP) [file pone.0149071.s002.zip › data requirements/Ford&Smit.pdf]

ARCTIC
VOL. 57, NO. 4 (DECEMBER 2004) P. 389–400

A Framework for Assessing the Vulnerability of Communities in the Canadian Arctic to Risks Associated with Climate Change

JAMES D. FORD[1] and BARRY SMIT[2]

(*Received 17 November 2003; accepted in revised form 14 April 2004*)

ABSTRACT. Adaptation to climate change is recognized as an important policy issue by international bodies such as the United Nations and by various national governments. Initiatives to identify adaptation needs and to improve adaptive capacity increasingly start with an assessment of the vulnerability of the system of interest, in terms of who and what are vulnerable, to what stresses, in what way, and what capacity exists to adapt to changing risks. Notwithstanding the scholarship on climate change itself, there are few studies on the nature of Arctic communities' vulnerability to climate-change risks. We review existing literature on implications of climate change for Arctic communities, develop a conceptual model of vulnerability, and present an analytical approach to assessing climate hazards and coping strategies in Arctic communities. Vulnerability is conceptualized as a function of exposure to climatic stresses and the adaptive capacity to cope with these stresses. The analytical framework employs place-specific case studies involving community residents and integrates information from multiple sources, both to document current exposures and adaptations and to characterize future exposures and adaptive capacity.

Key words: vulnerability, adaptation, adaptive capacity, climate change, environmental change, climatic risks, communities, Inuit, Canadian Arctic

RÉSUMÉ. L'adaptation au changement climatique est perçue comme un enjeu crucial par les organes internationaux tels que les Nations unies et plusieurs gouvernements nationaux. Des initiatives visant l'identification des besoins en matière d'adaptation, ainsi que l'amélioration de la capacité adaptative, débutent de plus en plus par un bilan de la vulnérabilité du système en cause, c'est-à-dire qui et quoi est vulnérable à quels stress et de quelle manière, et quelle capacité existe pour une adaptation aux risques changeants. Malgré les travaux de recherche sur le changement climatique même, il n'existe que peu d'études sur la nature de la vulnérabilité des communautés arctiques aux risques découlant du changement climatique. On passe en revue la documentation qui existe sur les implications que représente le changement climatique pour les collectivités arctiques, on élabore un modèle conceptuel de vulnérabilité et on présente une approche analytique à l'évaluation des dangers dus au climat et des stratégies d'adaptation dans les collectivités arctiques. La vulnérabilité est conceptualisée sous la forme d'une fonction de l'exposition aux stress climatiques et de la capacité adaptative permettant de composer avec ces stress. Le cadre d'analyse fait appel à des études de cas spécifiques à un lieu, mettant en cause des résidents de la communauté, et il intègre de l'information venant de sources multiples afin de documenter les expositions et adaptations actuelles et de caractériser les futures expositions ainsi que la capacité d'adaptation correspondante.

Mots clés: vulnérabilité, adaptation, capacité adaptative, changement climatique, changement environnemental, risques climatiques, collectivités, Inuit, Arctique canadien

Traduit pour la revue *Arctic* par Nésida Loyer.

INTRODUCTION

It is now widely accepted that the global climate is changing, at least in part as a result of human modification of the atmosphere (Weaver, 2003). Temperatures have increased at an unprecedented rate in the last 100 years, and warming trends are particularly pronounced in the higher latitudes (Houghton et al., 2001). Climate models predict that future climatic conditions are likely to be without precedent in the last 10 000 years, and it is anticipated that the effects will be felt earliest and strongest in the Arctic (Serreze et al., 2000; Houghton et al., 2001). Important changes in key climatic parameters are already evident in the Arctic, and climate models suggest that greater changes are forthcoming. As a consequence, climate-related risks that already pose challenges to Arctic communities, including greater unpredictability of environmental conditions, geophysical hazards, and changes to marine and terrestrial ecosystems, are expected to increase.

The response to climate change by the United Nations Framework Convention on Climate Change (UNFCCC) and national governments has been to focus on reducing emissions of greenhouse gases (known as "mitigation" in the climate-change community). Given the slow progress

[1] Department of Geography, University of Guelph, Guelph, Ontario N1G 2W1, Canada; jford01@uoguelph.ca
[2] Department of Geography, University of Guelph, Guelph, Ontario N1G 2W1, Canada; bsmit@uoguelph.ca
© The Arctic Institute of North America

towards reducing emissions, and with evidence suggesting that climate is already changing, measures to increase the ability of nations, sectors, and communities to cope with climatic changes (called "adaptation") are increasingly proposed by bodies such as the Intergovernmental Panel on Climate Change (IPCC), the parties to the UNFCCC, and in the Canadian Arctic, by regional organizations and territorial governments. To facilitate adaptation, these bodies need to know the nature of vulnerability, in terms of who and what are vulnerable, to what stresses, in what way, and what capacity exists to adapt to changing risks. While it is commonly stated that Arctic communities, especially indigenous communities following traditional lifestyles, are vulnerable to climate-related risks (Cohen, 1997; Maxwell, 1997; Anisimov and Fitzharris, 2001), other studies have identified considerable adaptability in these communities (Newton, 1995; Berkes and Jolly, 2001).

We present a conceptual model of vulnerability to climate risks and outline an analytical framework for applying the model to the case of Arctic communities. The approach, which draws on scholarship and practice from several fields, is designed to assist in analyzing community vulnerability and adaptability to climate. To provide a context for the methodology, we review the literature on climate-change implications for the Arctic, and we provide a critique of climate-change policy, noting the role of adaptation.

CLIMATE CHANGE AND THE ARCTIC

Instrumental and observational records have demonstrated that climatic and environmental conditions in the Arctic are already changing. Significant warming, increased precipitation, and changes in climatic variability and the occurrence of extremes have been recorded over extensive land areas (Maxwell, 1997; Serreze et al., 2000; Anisimov and Fitzharris, 2001; Krupnik and Jolly, 2002). According to the Inuvialuit at Sachs Harbour, Northwest Territories, the climatic changes observed in the 1990s were without precedent and outside the range of variation they consider normal (Berkes and Jolly, 2001). Inuit throughout Nunavut have also observed changes—some of them dramatic—in temperature, precipitation, the length of the seasons, the direction and strength of the prevailing wind, and the predictability of the weather (NTI, 2001; DSD, 2003). Instrumental and observational records also indicate changes in various aspects of the biophysical environment, including sea-ice extent, permafrost distribution and depth, river hydrology, geophysical processes, and the distribution of marine and terrestrial species (Table 1).

These climate-related changes have had implications for human systems (Table 2). As a result of the thawing of ice-rich permafrost, coastal erosion and retreat have damaged infrastructure and heritage sites along the Beaufort Sea coast (Shaw et al., 1998). Reductions in the extent, stability, and seasonal duration of sea ice and increased unpredictability of the weather have altered traditional hunting and subsistence strategies (Krupnik and Jolly, 2002). For example, Inuit at Baker Lake in Nunavut have had to adjust the timing and location of their hunting activities in response to lowering water levels in lakes and rivers, which have made traditional hunting grounds increasingly inaccessible (Fox, 2002). Inuit in the Nunavik region of northern Quebec have had to make similar adjustments in response to the increasing unpredictability of the weather and changes in ice and snow distribution (Furgal et al., 2002).

These observed changes are a portent of things to come. Future climate change in the polar regions is expected to be among the greatest anywhere on earth because of amplification by positive feedbacks in the climate system (Holland and Bitz, 2003). Models indicate that land areas in the Arctic will experience significant warming. Kattsov (2004) calculates a mean warming over the Arctic region by the end of the 21st century of 5–7˚C (twice the global average) using the output from five different climate models. Carter et al. (2000) indicate an increase in temperature by 2080 of 4–7.5˚C in the summer and 2.5–14˚C in the winter. Increased precipitation and alterations in the frequency, magnitude, and geographic distribution of climate-related events are also predicted (Houghton et al., 2001). These predicted changes are expected to have implications for communities and infrastructure in the Arctic (Anisimov and Fitzharris, 2001).

Many of these changes relate to risks that Arctic communities currently have to address. Smith and Burgess (1999) predict that permafrost could disappear from half of its present Canadian distribution. Indeed, in many areas permafrost is close to its limiting temperature and is therefore inherently unstable (Nelson et al., 2001). Increased amounts of open water in the Arctic Ocean, combined with rising sea levels, are likely to increase coastal erosion (Shaw et al., 1998). Together these changes bring the potential for severe disruption to human infrastructure, including transportation routes, pipelines, and housing, as well as to traditional subsistence practices. Shifts in the abundance and distribution of marine and terrestrial species such as salmon, cod, seal, caribou, and moose, already documented in the 20th century (Tynan and DeMaster, 1997; Babaluk et al., 2000), will necessitate further changes in traditional hunting practices. Increased unpredictability of sea-ice conditions and weather patterns will pose risks to communities for whom traditional knowledge of past environmental conditions is less dependable under changed climatic conditions. An event in July 2000, off Arctic Bay, Nunavut, where a large section of nearshore ice bearing 52 hunters and family members broke loose from landfast ice, is indicative of the risks posed (George, 2000). While all 52 marooned hunters were rescued, the incident highlights the difficulty of relying on traditional knowledge as a basis for adaptation under changing conditions.

Concern about such risks in communities along the Beaufort Sea coast was expressed at the Beaufort Sea Conference

TABLE 1. Instrumental records and community observations of changes in biophysical systems in the Arctic.

Biophysical System	Instrumental Records	Community Observations
Sea Ice	Reduced thickness and areal extent (Johannessen et al., 1999; Kerr, 2002)	Later ice freeze and breakup reported (Krupnik and Jolly, 2002)
Permafrost	Northward shift in distribution (Beilman et al., 2001)	Increased rate of spring melt; increased depth of active layer (Jolly et al., 2002)
River hydrology	No obvious trends in North American Arctic (Serreze et al., 2000)	Reduced water levels recorded by Inuit of Baker Lake, Nunavut (Fox, 2002)
Slopes	Increase in landslide frequency (Cohen, 1997; Aylsworth et al., 2000); increased coastal erosion (Shaw et al., 1998)	Increased coastal erosion and coastal slumping reported by Inuvialuit on Beaufort Sea coast (Berkes and Jolly, 2001; Nickels et al., 2002)
Terrestrial and marine ecosystems	Range extension of some fish species (Babaluk et al., 2000); northward movement of the tree line (Weller and Lange, 1999)	Changes in the timing of intra-island caribou migration on Banks Island and fewer polar bears (Jolly et al., 2002); geese arriving earlier in the year and leaving later and beluga having abandoned some traditional areas in Hudson Bay (McDonald et al., 1997); extension of the range of southern water fish

2000 in Inuvik, where participants from six western Arctic communities identified climate change as one of two primary areas of concern facing their communities (Ayles et al., 2002). Inuit in Nunavut have voiced similar concerns (McDonald et al., 1997; DSD, 2003; NTI, 2003).

CLIMATE CHANGE POLICY DIMENSIONS

The UNFCCC and national governments have focused responses to climate change on two broad policy areas: mitigation and adaptation. Mitigation relates to efforts to reduce or stabilize anthropogenic greenhouse gas emissions, in order to abate, moderate, or alleviate changes in climate (Smit et al., 2000). Mitigation figures prominently in the Kyoto Protocol and is a focus of the UNFCCC. In a policy context, adaptation to climate change refers to consciously planned adjustments in a system to reduce or moderate expected negative effects of climate change. The UNFCCC recognizes the role of adaptation by committing parties to develop and implement adaptation strategies (see Article 4.1b and 4.1e).

Both adaptation and mitigation are included as policy responses in the UNFCCC. Academic and political attention, however, has largely focused on mitigation (Burton, 2003), although the needs for adaptation are increasingly being recognized (Smit and Pilifosova, 2001; Burton et al., 2002; Huq et al., 2003; Smith et al., 2003). The UNFCCC has established several programs to support adaptation, including the National Adaptation Policies of Action (NAPA), the Special Climate Change Fund, the Kyoto Protocol Adaptation Fund, and the Least Developed Countries (LDC) Fund (Huq and Burton, 2003). The United Nations Development Program has prepared an Adaptation Policy Framework, intended to provide guidance on practical initiatives to implement adaptation. Canada recognizes adaptation in its Climate Change Plan (Environment Canada, 2002), and the federal government has supported research activities on adaptation, including the creation of the Canadian Climate Impacts and Adaptation Research Network (C-CIARN). The Government of Nunavut has also indicated its intention to promote adaptation to climate change with the release of the Nunavut Climate Change Strategy, in which the development and promotion of adaptation strategies are key goals (Government of Nunavut, 2003).

The importance of adaptation relates, in part, to the recognition that mitigation is unlikely to slow down anthropogenic climate change, let alone stop it. Climate models indicate that current greenhouse gas emissions commit the earth to ongoing increases in mean global temperature (McBean et al., 2001; Hansen et al., 2002). Even the most aggressive emission control measures will allow for a further rise in greenhouse gas concentrations and further changes in climate (Metz et al., 2001). Adverse impacts will inevitably occur. It is not surprising, therefore, that many countries vulnerable to changing climate are now seeking support for adaptation from the international community (Burton, 2003; Huq et al., 2003). In some areas climatic conditions are already changing, posing risks to ecosystems, economies, and societies. This is especially noticeable in the Canadian Arctic, where regional organizations, governments, and communities are stressing the need and importance of developing adaptation options (DSD, 2003; Government of Nunavut, 2003).

A common approach to the analysis of adaptation to climate change starts with scenarios of future average climate, models the impacts of the changed climate variables included in scenarios, and then assumes various adaptation options to see how well, if they were implemented, they would moderate the estimated adverse impacts (Carter et al., 1994; Parry and Carter, 1998). This approach, known as the "standard approach" (Burton et al., 2002), has been widely employed (Smith and Lazo, 2001; Parson et al., 2003). It has been very useful in bringing climate change to the attention of the public, but

TABLE 2. Climate-related changes affecting Arctic communities and adaptations (after Berkes and Jolly, 2001; Fox, 2002; Jolly et al., 2002; Nickels et al., 2002).

Effect	Change in climate	Adaptation/Coping strategy
Safety	Increased weather unpredictability and variability, past no longer a guide to the present, changing wind directions, unpredictable ice conditions	Monitoring conditions more closely; hunting closer to the community; some residents choosing to fly to hunt locations; some hunters using GPS; some using satellite images
Stress/depression	Increased unpredictability means skilled hunters and elders can no longer predict the weather as they did in the past: the result is a change in their relationship with the environment.	None
Access to hunting compromised	Increased unpredictability; changes in the spatial distribution of sea ice; permafrost thaw; changes in species distribution and availability	Changing the time of the hunt; spending less time on the land; hunting for polar bear earlier in the year; waiting
Erosion of shoreline	Sea level increase; temperature change melting the permafrost; increased open water; increasing fetch	Shoreline protection measures; abandoning susceptible land; relocation of homes a possibility; fishing elsewhere
Species availability	Changing water temperatures and distribution of ice affecting the distribution of fish and seal populations	Changing the species hunted and timing of hunt
Cultural loss due to aforementioned effects	All of the above	Lobbying for action on climate change; programs to strengthen community ties

it treats adaptation hypothetically, usually in relation to a limited set of average climatic conditions 40 to 80 years into the future. The standard approach is rarely connected to current experience of communities and usually does not relate to the actual adaptive decision-making process in communities. For countries and communities already at risk, there is interest in identifying adaptation options that address climate-related hazards relevant today as well as in the future: options that are feasible in light of the many conditions other than climate that influence decisions about resource use and livelihoods.

An approach that focuses on community-relevant vulnerabilities and adaptation options, dubbed the "vulnerability approach," has evolved in the climate change field in recent years. This approach starts with an assessment of the vulnerability of the community or region, in terms of who and what are vulnerable, to what stresses, in what way, and what capacity exists to adapt to changing risks (Burton et al., 2002; Polsky et al., 2003; Smit and Pilifosova, 2003). Communities' sensitivity and vulnerability to climate change are related both to their exposure to climatic hazards and to their ability to deal with or adapt to the effects.

THE CONCEPT OF VULNERABILITY

While vulnerability has many definitions in the literature (see Cutter, 1996; Brooks, 2003), there is broad agreement that it refers to the susceptibility to harm in a system relative to a stimulus or stimuli. The concept has evolved in various research fields, including risk assessment and natural hazards (Hewitt, 1983), food security (Sen, 1981; Dreze and Sen, 1990), national security (Homer-Dixon and Blitt, 1998), and environmental change (Liverman, 1990; Kasperson et al., 1995). The literature on vulnerability has two major perspectives: biophysical and social.

In the biophysical approach, vulnerability is viewed as being determined by the nature of the physical event to which the human system is exposed, the likelihood or frequency of occurrence of the event, the extent of human exposure, and the system's sensitivity to the impacts of a particular event (Brooks, 2003). The role of the human system in modifying the event is largely downplayed; the focus is predominantly on the event itself, in terms of magnitude, frequency, rapidity of onset, and spatial distribution. People are treated as being vulnerable owing to their presence in hazardous locations. Cutter (1996:530) terms this the "vulnerability as a pre-existing condition" approach, and it has formed the basis of a significant amount of research in the natural hazards and environmental change literature (Alexander, 1993; Zeidler, 1997; Nicholls et al., 1999).

The social perspective focuses primarily on the human determinants or drivers of vulnerability, namely, the social, political, and economic conditions that make exposure unsafe or challenging. This perspective emerged out of recognition that hazards and disasters were not a result of physical events alone, but were also greatly influenced by the social, economic, and cultural conditions that contributed to hazardous exposures and the ability to plan for and manage them. This work has focused on such factors as marginalization, inequality, the presence and strength of social networks, poverty, and food entitlements (Adger, 2000; O'Brien and Leichenko, 2000; Adger et al., 2002; Pelling, 2002). Blaikie et al. (1994) define vulnerability in terms of the capacity of individuals or groups to anticipate,

cope with, resist, and recover from the impact of a natural hazard. They describe a process by which social, economic, and political pressures operating at varying scales combine to create unsafe conditions that reflect specific forms of vulnerability in space and time. With regard to vulnerability to climate change, a growing number of researchers have focused on the socioeconomic processes that constrain the ability to cope with climatic hazards. Building on Sen's work (1990), Bohle et al. (1994) and Adger and Kelly (1999) emphasize the social construction of vulnerability, focusing on the ways in which the availability of resources and the entitlement of individuals to call on these resources influence their capacity to adapt to climate changes. The importance of this adaptive capacity was highlighted in the IPCC's most recent assessment (Smit and Pilifosova, 2001).

Several recent approaches to vulnerability have explicitly incorporated both the social and the biophysical dimensions (Cutter, 2003; Fraser et al., 2003; Turner et al., 2003). These studies characterize the differential vulnerability of communities, regions, and countries to climate-related hazards in terms of the physical events and the socio-economic and institutional capacities to deal with and adapt to these events.

MODEL OF VULNERABILITY

The conceptual model of community vulnerability to climate change outlined here builds on the literature, conceptualizing vulnerability as a function of exposure of the community to climate-change effects and its adaptive capacity to deal with that exposure. This model can be expressed formally (after Smit and Pilifosova, 2003):

$$V_{ist} = f\,(E_{ist}, A_{ist}) \qquad (1)$$

where V_{ist} = vulnerability of community i to stimulus s in time t; E_{ist}, = exposure of i to s in t; and A_{ist} = adaptive capacity of i to deal with s in time t.

The functional relationship between the two elements is not specified, as it will vary by location, context, sector, and time. However, it is understood that vulnerability is a positive function of a community's exposure and a negative or inverse function of a community's adaptive capacity (Smit and Pilifosova, 2003).

Exposure is a property of a community relative to climatic conditions. It reflects both the nature of the climatic conditions and nature of the community itself. Some communities may be exposed to a particular climate event whereas the same event may not affect another community. Climatic characteristics include magnitude, frequency, spatial dispersion, duration, speed of onset, and temporal spacing of climatic risks, relating to temperatures, precipitation, and wind. The nature of the community concerns its location relative to the climatic risks. One of the main reasons for an increase in vulnerability to natural hazards in Canada is an increase in exposure, related mainly to population growth and economic development. Since 1951, population has increased from 14 million to over 30 million, with much development occurring in marginal or hazard-prone areas (White and Etkin, 1997). In the Northwest Territories, resource development and associated infrastructure development and urban expansion have increased the exposure to permafrost thaw, flooding, and coastal erosion. Changes in exposure can also be attributed to changes in the physical environment, including climate. For many Arctic communities, increased exposure has been due to changes in environmental conditions, such as increasing unpredictability of the weather and ice conditions and melting permafrost.

Adaptive capacity refers to a community's potential or ability to address, plan for, or adapt to exposure (Smit and Pilifosova, 2003). Most communities can cope with normal climatic conditions and a range of deviations around norms. People have learned to modify their behaviour and their environment to manage and take advantage of their local climatic conditions (Jones and Boer, 2003). This ability to cope is referred to in the literature as the "coping range"; it reflects resource use options and risk management strategies to prepare for, avoid or moderate, and recover from exposure effects (Hewitt and Burton, 1971; Smit et al., 1999; Jones, 2001; Smit and Pilifosova, 2003). Adaptive capacity relates to communities' resilience, resistance, flexibility, and robustness (Smithers and Smit, 1997). It is influenced by economic wealth, social networks, infrastructure, social institutions, social capital, experience with previous risk, the range of technological adaptation available, and equity of access to resources within the community, as well as by other stresses that contribute to the environment in which decisions are made (Adger and Kelly, 1999; Smit and Pilifosova, 2001; Smith et al., 2003).

Figure 1 illustrates the relationships between the variation in a community-relevant climatic attribute (in this case, peak annual flood stage) and the capacity of a hypothetical community in the Mackenzie Basin, Northwest Territories, to deal with flooding over time. The variation in flood stage reflects the fact that the magnitude of flooding is influenced by snow pack depth and the rate at which the accumulated snow melts, rainfall during snowmelt, ice deterioration before breakup, and the timing and location of ice jams (Environment Canada, 2003). In some years, a combination of high spring flows and major ice jams can cause significant flood events, while in other years only minor flooding occurs. The shaded area (from $X_1 - X_2$) indicates the coping range: the community has adapted to cope with flood events of a certain magnitude. This adaptability is related to the social, economic, and other resources that the community can draw on to prepare for, cope with, and recover from hazardous conditions. Newton (1995), who studied Aklavik, for example, found that the combination of a strong level of awareness among community members to the signs and conditions preceding a flood event, preparation in anticipation of flooding by

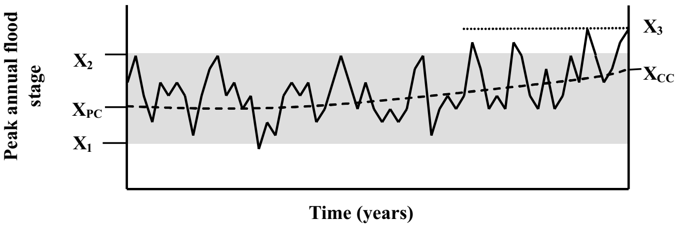

FIG. 1. A diagrammatic representation of variability in the annual peak flood stage over time (solid line), showing an upward trend of the peak with climate warming (dotted line). Shaded area represents the coping range of the system. (Based on Smit and Pilifosova, 2003.)

ensuring an adequate supply of food and by broadcasting information within the community, the existence of evacuation procedures, and strong social networks to help with recovery after the event, underlies the community's adaptability (Newton, 1995). It is flood events of great magnitude to which the community is vulnerable.

Figure 1 also shows how climate change is not just a change in average temperature, but also relates to variability, extreme events, adaptive capacity, and vulnerability. The time series shows an upward trend in peak flood magnitude related to climate change (from X_{PC} to X_{CC}). This trend reflects the likelihood of increased snowmelt and precipitation during the maximum melt season from May to July (Kerr, 1997; Brooks, 2000). The community can still cope with the changed mean (X_{CC}), which is within its historical coping range or adaptive capacity. But with a shift in the mean, and without a shift in variability, the frequency and magnitude of unusually high flooding events increase, and the community becomes more vulnerable.

In response to increasing vulnerability to flooding, the community may adapt by measures such as infrastructure design, relocations, development siting, early warning systems, and protection works (reflected in Fig. 1 by an increase in upper limit of the coping range, from X_2 to X_3). This capacity to cope can vary over time in response to social, economic, political, and future environmental changes. Economic growth and improvements in technology, for example, may lead to a gradual increase in adaptive capacity. But such changes may be equally likely to reduce adaptive capacity by undermining social networks. Newton (1995), in his work among isolated northern communities, found increasing dependency on outside sources of assistance to be undermining individual and communal responsibility and thus undermining traditional means of coping. The ability to cope will also vary between regions and communities. Those communities where political, economic, and social networks and traditional knowledge are strong generally are expected to have more resources for adapting.

VULNERABILITY OF ARCTIC COMMUNITIES

Much of the information on the implications of climate change for communities in the Arctic is in the form of studies conducted by the government, the IPCC, and interest groups (Duerden, 2001). These include the section on Polar Regions in the Third Assessment Report of the IPCC, *Climate Change 2001: Impacts, Adaptation, and Vulnerability* (Anisimov and Fitzharris, 2001), the Arctic section of the Canada Country Study (Maxwell, 1997), and the Mackenzie Basin Impact Study (Cohen, 1997). These studies review the latest developments in impact prediction and discuss the implications of climate change scenarios for key socioeconomic sectors, including oil and gas, transportation, fisheries, and human communities. They have largely been preoccupied with predicting how exposure will change in 50 to 100 years, using climate and biophysical system models. Adaptive capacity of communities is rarely addressed explicitly. The broad conclusions from these studies are that major biophysical, sociological, and economic impacts are anticipated and that indigenous communities are particularly vulnerable to these effects, especially if climate change is rapid.

Other studies have analyzed how communities have adapted to change as a basis for identifying their vulnerability or resilience (Newton, 1995, 1997; Berkes and Jolly, 2001). These studies have shown that indigenous groups of the Arctic have demonstrated significant adaptability and resilience in the face of changing conditions. Survival in the harsh Arctic environment has depended on their adaptability. This adaptability has been facilitated by extensive experience and knowledge about the environment in which they live (Brody, 2000), mobility and flexibility of group size (Langdon, 1995; Cruikshank, 2001), a dynamic and flexible use of the environment (Krupnik, 1993), and strong social networks (Magdanz et al., 2002). Berkes and Jolly (2001), for example, identify several strategies by which the community of Sachs Harbour has coped with recently experienced climatic and environmental changes. These include hunters' staying closer to the community while out on the ice because of increasingly unpredictable sea-ice conditions, closer monitoring of weather and environmental conditions due to increased unpredictability, the use of all-terrain vehicles instead of snowmobiles to reach spring camps when there is not enough snow, and fishing at new lakes when erosion and slumping have limited fishing elsewhere. Newton (1995, 1997) identifies an inherent resilience in the mechanisms used by two isolated communities, Aklavik and Fort Liard in the Northwest Territories, to cope with flooding. This resilience is based on a combination of strong awareness of the signs and conditions preceding a flood event, preparation in anticipation of flooding by the moving of belongings, broadcasting of information within the community, and the existence of evacuation procedures. In Nunavut, examples of adaptations to environmental changes have included being more vigilant when engaged in day-to-day activities, observing water levels and ice conditions, using other navigation methods, such as GPS, and caching meat later in the year (DSD, 2001; Fox, 2002).

While indigenous communities have historically demonstrated adaptability to a variety of stresses, their coping

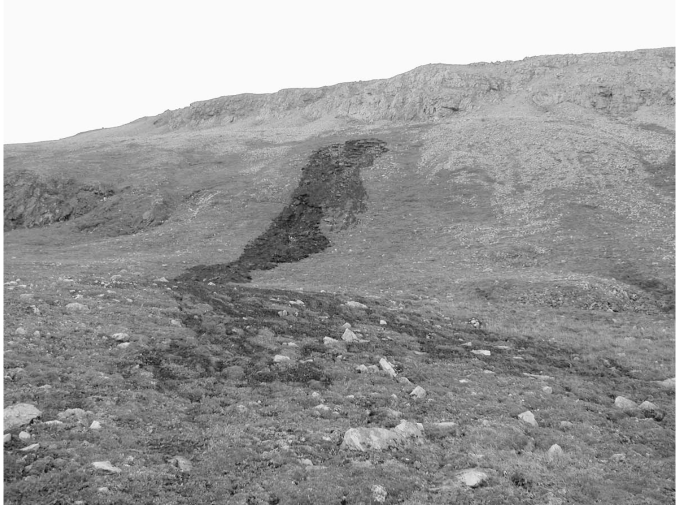

FIG. 2. Mudslide at Arctic Bay. (Photo by John MacDonald.)

abilities have been put under considerable strain by recent climatic and environmental changes (Fast and Berkes, 1998; Fox, 2002). Increasing unpredictability and changing seasonal norms have been particularly problematic. Anomalous warm spells in midwinter and spring have damaged winter transportation routes and interrupted hunter travel on the ice. During the annual hunt for bowhead whales in April and June in Barrow, Alaska, unusual events, including a shift in the timing of ice breakup, caused adaptation problems because of deviations from traditional knowledge of environmental conditions (Norton, 2002).

Subtle changes in climatic conditions, such as changing wind characteristics, have also been problematic. At Baker Lake, Nunavut, changes in the direction, strength, and frequency of wind have caused navigational problems and had implications for safety while out on the ice (Fox, 2002). Reduced snow accumulation, combined with stronger winds that have compacted the snow, has made it more difficult to construct igloos, used as temporary and emergency shelters (DSD, 2001). At other locations, the acceleration of geophysical processes and frequency of geophysical events have caused problems to communities exposed to them. In the Nunavut community of Arctic Bay, unusually high summer rainfall triggered a mudslide (Fig. 2), the first ever in living memory at this location, which has caused concerns regarding future development in the hamlet (J. MacDonald, pers. comm. 2004). Shoreline erosion is a problem along the Beaufort Sea coast of the Northwest Territories, Yukon, and Alaska, where a combination of rising sea level, reduced sea-ice extent during the months of greatest storm activity, and melting permafrost may ultimately make it impossible to stop further erosion, even with the appropriate engineering structures (Shaw et al., 1998; Craver, 2001; Johnson et al., 2003).

The ability of communities to cope has also been put under strain by changing social, economic, and political conditions that have modified the strategies through which communities deal with stress. Arctic communities have experienced, and continue to experience, significant economic, political, and social change (Fenge, 2001; Usher, 2002). Profound changes in Inuit society occurred with the centralization of Inuit communities in fixed settlements in the 1950s and 1960s (Damas, 2002). This change reduced both the locational flexibility and the flexibility of group size with which communities traditionally responded to environmental stresses. Cultural and social changes, including the adoption of nontraditional lifestyles, the growth of full-time waged labor, the erosion of knowledge about the local environment and traditional skill sets among younger generations, and an increased dependence on outside assistance, have also modified community coping capacity. According to Newton (1995), the weakening of land-related skills and knowledge has contributed to increased levels of vulnerability to flooding among northern Canadian communities. For Berkes and Jolly (2001), however, the loss of old skill sets has been compensated to an extent by the development of new ones (the use of GPS as a navigational aid, for example), and they maintain that many of the traditional skills underpinning the ability to adapt still remain strong. Increased political autonomy and comprehensive land-claim agreements may further strengthen the adaptability of communities.

Notwithstanding the research over the last decade on ways in which communities have dealt with climatic hazards, little has been done to assess the ability of communities to cope with future climatic risks. There is now strong evidence that global climate change will alter the Arctic environment, and is doing so already, with significant risks for Arctic communities. To assist in managing those risks, there is a need to understand the ways in which communities are vulnerable, the climate-related conditions that are problematic, and the degree to which existing adaptive capacity can (or cannot) cope with changing conditions.

A RESEARCH FRAMEWORK FOR VULNERABILITY ANALYSIS

A research framework for empirically applying the model of vulnerability proposed above to Arctic communities is illustrated in Figure 3. The first stage assesses current vulnerability by documenting current exposures and current adaptive strategies. The second stage assesses future vulnerability by estimating directional changes in exposure and predicting future adaptive capacity on the basis of past behavior.

Current Vulnerability

The assessment of current vulnerability requires analyzing and documenting communities' experiences with climatic risks (current exposure) and the adaptive options and resource management strategies employed to address these risks (current adaptive capacity). Observations,

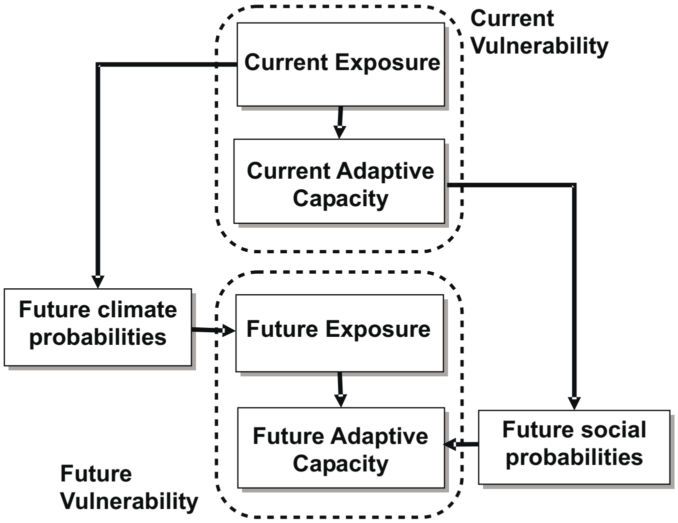

FIG. 3. Analytical framework for vulnerability assessment.

experience, and the traditional and local knowledge of community members (Inuit *Qaujimajatuqangit*) are central to assessing current vulnerability. Indigenous populations possess detailed knowledge of their environment built up through personal observation and experience and from shared experience of members of the community (Duerden and Kuhn, 1998; Huntington, 1998; Usher, 2000). Knowledge about the environment and its use can be employed to identify and reconstruct events and conditions that represent climatic risks to the community and to provide insights into the resource-use options and risk-management strategies employed to prepare for, avoid or moderate, and recover from the effects of exposure.

Such knowledge can be gained through several established ethnographic techniques, including focus groups, interviews, and participant observation. These techniques have been successfully used in research documenting indigenous observations on climate and environmental change throughout Arctic North America (Ferguson et al., 1998; Huntington, 1998; Krupnik and Jolly, 2002; DSD, 2003). Inuit *Qaujimajatuqangit* has also been documented to show how communities are adapting to changes and to identify adaptation needs (Fox, 2002; Nickels et al., 2002; DSD, 2003; Government of Nunavut, 2003).

Information on risks and adaptation strategies can also be derived from content analysis of government reports, newspaper articles, Hudson Bay Company postal records, Distant Early Warning Site reports, and the insights of experienced land and resource use managers (Duerden, 2001). Solomon and Hart (1999) used Hudson Bay Company postal records and ships' logbooks to examine storm frequency and severity in the Beaufort Sea. Fienup-Riordan (1999) used Catholic mission records and letters between government officials to assess the nature and impacts of a storm surge in 1931 in southwestern Alaska.

The analysis of current vulnerability requires a timeframe to establish how far back in time the study should go when analyzing risks and community response. The timeframe depends in part on the extent to which past conditions that determined adaptability are relevant today, as well as on the availability of information. In setting the timeline, one must weigh the value of analyzing how previous generations coped with hazards against the recent social, economic, political, and technological changes, which also determine adaptive capacity. Lim et al. (in press) suggest limiting historical analysis to one or two decades, although many of the traditional coping mechanisms, such as flexibility, detailed local knowledge, social networks, and intercommunity trade, have a much longer history and remain strong among Arctic communities (Berkes and Jolly, 2001).

Future Vulnerability

Future vulnerability is assessed by analyzing how climate change will alter the nature of the climate-related risks and whether the communities' coping strategies will have the capacity to deal with these risks. Assessing future exposure involves collaboration with the climate science community to estimate the likelihood of changes in climatic attributes identified by the community. For example, will extreme events or climatic variability continue to increase? Will the unexpected winds that have caused problems to hunters in many Nunavut communities become even stronger and less predictable? Will the storm surges that have damaged infrastructure and sea defenses increase in magnitude or frequency? Which areas will experience most exposure to erosion? Future exposure also includes estimating the future state of the socioeconomic conditions, given that exposure is a property of the system relative to risk. Future adaptive capacity concerns the degree to which the community can deal with the estimated future exposures. By examining past responses to climate variability and extremes and having the community identify its future adaptation options and constraints, researchers can characterize a community's ability to cope with future changes and collaborate to identify adaptive strategies that will reduce risk.

CONCLUSION

A system's vulnerability to climate change is a function of both its exposure to climatic risks and its adaptive capacity to deal with these risks. Arctic communities have demonstrated significant adaptability in the past, but there is concern that future changes in conditions may exceed conventional coping capacities. Analysis of vulnerability to climate change requires an understanding of the processes by which adaptation occurs, the factors determining adaptive capacity, and the way climatic risks will change in the future. We have presented an analytical framework, based on recent research, for assessing the vulnerability of communities empirically. The first stage is to assess current vulnerability by documenting exposures and current

adaptive strategies. The second stage is to estimate directional changes in those current risk factors and characterize the community's future adaptive capacity. The approach employs information on how communities have experienced and addressed climatic hazards in the past, what conditions are likely to change, and what constraints and opportunities there are for future adaptation.

ACKNOWLEDGEMENTS

Feedback on earlier versions of this approach was provided by attendees at the ARCUS conference on Vulnerability and Resilience in Washington D.C. (April 2003), the Canadian Association of Geographers annual meeting in Victoria (May 2003), and the HARC Workshop in Seattle (October 2003). Thanks to John MacDonald for Figure 2. Lea Berrang and Stefanie Neumann helped with editing. The research was supported by ArcticNet, a Seed Grant from the Integrated Management Node of the Ocean Management Research Network, the Social Sciences and Humanities Research Council of Canada, and the Canadian Rhodes Scholars Foundation. The useful insights and generous hospitality provided by the communities of Igloolik and Arctic Bay are gratefully acknowledged. Anonymous reviewers provided detailed and constructive suggestions.

REFERENCES

ADGER, W.N. 2000. Institutional adaptation to environmental risk under the transition in Vietnam. Annals of the Association of American Geographers 90(4):738–758.

ADGER, W.N., and KELLY, P.M. 1999. Social vulnerability to climate change and the architecture of entitlements. Mitigation and Adaptation Strategies for Global Change 4:253–266.

ADGER, W.N., BROOKS, N., BROWN, K., CONWAY, D., HAZELTINE, A., and HULME, M. 2002. Memorandum of evidence on climate change and sustainable development by the Tyndall Centre for Climate Change Research. House of Commons, International Development Committee, Global Climate Change and Sustainable Development. HC Paper 519-II. Session 2001–02. London, England: The Stationery Office. 59–62.

ALEXANDER, D. 1993. Natural disasters. New York: Chapman and Hall. 650 p.

ANISIMOV, O., and FITZHARRIS, B. 2001. Polar regions (Arctic and Antarctic). In: McCarthy, J., Canziani, O.F., Leary, N.A., Dokken, D.J., and White, K.S., eds. Climate change 2001: Impacts, adaptation and vulnerability. Contribution of Working Group II to the Third Assessment Report of the Intergovernmental Panel on Climate Change. Cambridge, UK: Cambridge University Press. 801–841.

AYLES, G.B., BELL, R., and FAST, H. 2002. The Beaufort Sea Conference 2000 on the renewable marine resources of the Canadian Beaufort Sea. Arctic 55(Supp. 1):iii–v.

AYLSWORTH, J.M., DUK-RODIN, A., ROBERTSON, T., and TRAYNOR, J.A. 2000. Landslides of the Mackenzie valley and adjacent mountainous and coastal regions. In: Dyke, L.D., and Brooks, G.R., eds. The physical environment of the Mackenzie Valley, Northwest Territories: A base line for the assessment of environmental change: Geological Survey of Canada Bulletin 547. 167–176.

BABALUK, J.A., REIST, J.D., JOHNSON, J.D., and JOHNSON, L. 2000. First records of sockeye (*Oncorhynchus nerka*) and pink salmon (*O. gorbuscha*) from Banks Island and other records of Pacific salmon in Northwest Territories. Arctic 53(2): 161–164.

BEILMAN, D.W., VITT, D.H., and HALSEY, L.A. 2001. Localized permafrost peatlands in Western Canada: Definition, distributions, and degradation. Arctic, Antarctic, and Alpine Research 33(1):70–77.

BERKES, F., and JOLLY, D. 2001. Adapting to climate change: Social-ecological resilience in a Canadian Western Arctic community. Conservation Ecology 5(2):18. [online] URL: http://www.consecol.org/vol5/iss2/art18.

BLAIKIE, P., CANNON, T., DAVIS, I., and WISNER, B. 1994. At risk: Natural hazards, people's vulnerability and disasters. New York: Routledge. 284 p.

BOHLE, H.C., DOWNING, T.E., and WATTS, M.J. 1994. Climate change and social vulnerability. Global Environmental Change 4(1):37–48.

BRODY, H. 2000. The other side of Eden. London: Faber and Faber. 374 p.

BROOKS, G.R. 2000. Streamflow in the Mackenzie valley. In: Dyke, L.D., and Brooks, G.R., eds. The physical environment of the Mackenzie valley, Northwest Territories: A baseline for the assessment of environmental change. Geological Survey of Canada Bulletin 547. 153–158.

BROOKS, N. 2003. Vulnerability, risk and adaptation: A conceptual framework. Working Paper 38. Norwich: Tyndall Centre for Climate Change Research. 20 p.

BURTON, I. 2003. Do we have the adaptive capacity to develop and use the adaptive capacity to adapt? In: Smith, J., Klein, R.J.T., and Huq, S., eds. Climate change, adaptive capacity, and development. London: Imperial College Press. 137–161.

BURTON, I., HUQ, S., LIM, B., PILIFOSOVA, O., and SCHIPPER, E.L. 2002. From impacts assessment to adaptation priorities: The shaping of adaptation policy. Climate Policy 2:145–159.

CARTER, T.R., PARRY, M., HARASAWA, H., and NISHIOKA, S. 1994. IPCC technical guidelines for assessing climate change impacts and adaptations with a summary for policy makers and a technical summary. London: Department of Geography, University College and Japan: Centre for Global Environmental Research, National Institute for Environmental Studies. 59 p.

CARTER, T.R., HULME, M., CROSSLEY, S., MALYSHEV, M.G., NEW, M.G., SCHLESINGER, M.E., and TOUMEN-VIRTA, H. 2000. Climate change in the 21st century: Interim characterizations based on the new IPCC emissions scenarios. The Finnish Environment 433. Helsinki: Finnish Environment Institute. 148 p.

COHEN, S.J. 1997. Mackenzie basin impact study (MBIS): Final report. Downsview, Ontario: Environment Canada. 372 p.

CRAVER, A. 2001. Alaska subsistence lifestyles could disappear in the next decade. Northwest Public Health Fall/Winter:8–9.

CRUIKSHANK, J. 2001. Glaciers and climate change: Perspectives from oral tradition. Arctic 54(4):377–393.

CUTTER, S.L. 1996. Vulnerability to environmental hazards. Progress in Human Geography 20(4):529–539.

———. 2003. The vulnerability of science and the science of vulnerability. Annals of the Association of American Geographers 93(1):1–12.

DAMAS, D. 2002. Arctic migrants/Arctic villagers: Montreal: McGill-Queens University Press. 277 p.

DREZE, J., and SEN, A. 1990. The political economy of hunger. Oxford: Clarendon Press.

DSD (DEPARTMENT OF SUSTAINABLE DEVELOPMENT). 2001. Inuit knowledge of climate change: A sample of Inuit experiences of climate change in Nunavut. Baker Lake and Arviat, Nunavut. Iqaluit: Government of Nunavut, Department of Sustainable Development, Environmental Protection Services. 161 p.

———. 2003. Inuit *Qaujimajatuqangit* of climate change in Nunavut: Summary report of activities January 2001 to March 2003. Iqaluit: Government of Nunavut, Department of Sustainable Development, Environmental Protection Services. 15 p.

DUERDEN, F. 2001. Climate change and human activity in northern Canada: What we know, what we don't know, and what we need to know. The Northern Review 24(Winter 2001):150–159.

DUERDEN, F., and KUHN, R.G. 1998. Scale, context, application of traditional knowledge of the Canadian North. Polar Record 34(188):31–38.

ENVIRONMENT CANADA. 2002. Climate change plan for Canada. Ottawa: Environment Canada. 67 p.

———. 2003. Flooding events in Canada – Northwest Territories. Environment Canada. http://www.ec.gc.ca/water/en/manage/floodgen/e_nwt.htm. Accessed March 2004.

FAST, H., and BERKES, F. 1998. Climate change, northern subsistence, and land based economies. In: Mayer, N., and Avis, W., eds. Canada country study: Climate impacts and adaptation. National cross-cutting issues, Vol. 8. Ottawa: Environment Canada. 206–226.

FENGE, T. 2001. The Inuit and climate change. Isuma 2(4):79–85.

FERGUSON, M.A.D., WILLIAMSON, R.G., and MESSIER, F. 1998. Inuit knowledge of long-term changes in a population of Arctic tundra caribou. Arctic 51(3):201–219.

FIENUP-RIORDAN, A. 1999. *Yaqulget qaillun pilartat* (what the birds do): Yup'ik Eskimo understanding of geese and those who study them. Arctic 52(1):1–22.

FOX, S. 2002. These are things that are really happening: Inuit perspectives on the evidence and impacts of climate change in Nunavut. In: Krupnik, I., and Jolly, D., eds. The earth is faster now: Indigenous observations of Arctic environmental change. Fairbanks, Alaska: Arctic Research Consortium of the United States. 12–53.

FRASER, E.D.G., MABEE, W., and SLAYMAKER, O. 2003. Mutual vulnerability, mutual dependence: The reflexive relation between human society and the environment. Global Environmental Change 13(2):137–144.

FURGAL, C., MARTIN, D., and GOSSELIN, P. 2002. Climate change and health in Nunavik and Labrador. In: Krupnik, I., and Jolly, D., eds. The earth is faster now: Indigenous observations of Arctic environmental change. Fairbanks, Alaska: Arctic Research Consortium of the United States. 266–299.

GEORGE, J. 2000. Helicopters rescue 52 Arctic Bay residents. *Nunatsiaq News*, 7 July 2000.

GOVERNMENT OF NUNAVUT. 2003. Nunavut climate change strategy. Iqaluit: Government of Nunavut. 26 p.

HANSEN, J., SATO, M., NAZARENKO, L., RUEDY, R., LACIS, A., KOCH, D., TEGEN, I., HALL, T., SHINDELL, D., SANTER, B., STONE, P., NOVAKOV, T., THOMASON, L., WANG, R., WANG, Y., JACOB, D., HOLLANDSWORTH, S., BISHOP, L., LOGAN, J., THOMPSON, A., STOLARSKI, R., LEAN, J., WILLSON, R., LEVITUS, S., ANTONOV, J., RAYNER, N., PARKER, D., and CHRISTY, J. 2002. Climate forcings in GISS SI2000 simulations. Journal of Geophysical Research – Atmospheres 107:4347–4384.

HEWITT, K. 1983. The idea of calamity in a technocratic age. In: Hewitt K., ed. Interpretations of calamity from the viewpoint of human ecology. Boston: Allen and Unwin. 3–32.

HEWITT, K., and BURTON, I. 1971. The hazardousness of place: A regional ecology of damaging events. Toronto: University of Toronto Press. 154 p.

HOLLAND, M.M., and BITZ, C.M. 2003. Polar amplification of climate change in coupled models. Climate Dynamics 21 (3–4):221–232.

HOMER-DIXON, T., and BLITT, J. 1998. Ecoviolence: Links among environment, population, and security. Lanham, Maryland: Rowman & Littlefield Publishers, Inc. 256 p.

HOUGHTON, J.T., DING, Y., GRIGGS, D.J., NOGUER, M., VAN DER LINDEN, P.J., DAI, X., MASKELL, K., and JOHNSON, C.A. 2001. Climate change 2001: The scientific basis. Contribution of Working Group I to the Third Assessment Report of the Intergovernmental Panel on Climate Change. Cambridge: Cambridge University Press. 881 p.

HUNTINGTON, H.P. 1998. Observations on the utility of the semi-directive interview for documenting traditional ecological knowledge. Arctic 51(3):237–242.

HUQ, S., and BURTON, I. 2003. Funding adaptation to climate change: What, who and how to fund? London: International Institute for Environment and Development, London. 2 p.

HUQ, S., RAHMAN, A., KONATE, M., SOKONA, Y., and REID, H. 2003. Mainstreaming adaptation to climate change in least developed countries. London: International Institute for Environment and Development Climate Change Programme. 38 p.

JOHANNESSEN, O.M., SHALINA, E.V., and MILES, M.W. 1999. Satellite evidence for an Arctic sea ice cover in transformation. Science 286:1937–1939.

JOHNSON, K., SOLOMON, S., BERRY, D., and GRAHAM, P. 2003. Erosion progression and adaptation strategy in a northern coastal community. 8th International Conference on Permafrost, Zurich. http://www.members.shaw.ca/ken.planner/planning+%20erosion.pdf. Accessed February 2004.

JOLLY, D., BERKES, F., CASTLEDEN, J., NICHOLS, T., and THE COMMUNITY OF SACHS HARBOUR. 2002. We can't predict the weather like we used to: Inuvialuit observations of climate change, Sachs Harbour, western Canadian Arctic. In: Krupnik, I., and Jolly, D., eds. The earth is faster now: Indigenous

observations of Arctic environmental change. Fairbanks, Alaska: Arctic Research Consortium of the United States. 92–125.

JONES, R.N. 2001. An environmental risk assessment/management framework for climate change impacts assessment. Natural Hazards 23:197–230.

JONES, R., and BOER, R. 2003. Assessing current climate risks. Chapter 4 of the Working Draft of the Adaptation Policy Framework. New York: United Nations Development Programme. http://www.undp.org/cc/pdf/APF/TP%20final/Tech.Paper_4.qxp_30Aug04.pdf. Accessed September 2004.

KASPERSON, R.E., KASPERSON, J.E.X., and TURNER, B.L. 1995. Regions at risk: Comparisons of threatened environments. Tokyo: United Nations Press. 600 p.

KATTSOV, V.M. 2004. Arctic climate in the 21st century: Modelling and scenarios. International Conference on High-Impact Weather and Climate: Understanding, prediction, and socio-economic consequences, 22–24 March, Seoul, Korea. http://ichwc2004.metri.re.kr/ICHWC2004/absfiles/CL5_06.pdf. Accessed 1 October 2004.

KERR, J.A. 1997. Future water levels and flows for Great Slave and Great Bear Lakes, Mackenzie River and Mackenzie Delta. In: Cohen, S.J., ed. Mackenzie Basin Impact Study (MBIS): Final report. Downsview, Ontario: Environment Canada. 73–91.

KERR, R.A. 2002. Whither Arctic ice? Less of it, for sure. Science 297(5586):1491.

KRUPNIK, I. 1993. Arctic adaptations: Native whalers and reindeer herders of northern Eurasia. Hanover, New Hampshire: University Press of New England. 355 p.

KRUPNIK, I., and JOLLY, D. 2002. The earth is faster now: Indigenous observations of Arctic environmental change. Fairbanks, Alaska: Arctic Research Consortium of the United States. 384 p.

LANGDON, S. 1995. Increments, ranges, and thresholds: Human population responses to climate change in northern Alaska. In: Peterson, D.L., and Johnson, D.R., eds. Human ecology and climate change. Washington: Taylor & Francis. 139–154.

LIM, B., BURTON, I., and HUQ, S. In press. The adaptation policy framework. Cambridge: Cambridge University Press. (First order draft from May 2001 of "An adaptation policy framework: Capacity building for Stage II adaptation. A UNDP-GEF Project" available at www.undp.org/cc/pdf/APF/web/UNDP-GEF%20Framework%20May_v11.pdf. Accessed October 2004.)

LIVERMAN, D. 1990. Vulnerability to global environmental change. In: Kasperson, R.E., ed. Understanding global environmental change. Worcester, Massachusetts: Center for Technology, Environment, and Development, Clark University. 27–44.

MAGDANZ, J., UTERMOHLE, C.J., and WOLFE, R.J. 2002. The production and distribution of wild food in Wales and Deering, Alaska. Juneau: Alaska Department of Fisheries and Game, Division of Subsistence. 136 p.

MAXWELL, B. 1997. Responding to global climate change in Canada's Arctic. Vol. II of the Canada country study: Climate impacts and adaptation. Downsview, Ontario: Environment Canada. 82 p.

McBEAN, G., WEAVER, A., and ROULET, N. 2001. The science of climate change: What do we know? Isuma 2(4):16–25.

McDONALD, M., ARRAGUTAINAQ, L., and NOVALINGA, Z. 1997. Voices from the Bay: Traditional ecological knowledge of Inuit and Cree in the Hudson Bay bioregion. Ottawa: Canadian Arctic Resources Committee and Sanikiluaq: Municipality of Sanikiluaq. 98 p.

METZ, B., DAVIDSON, O., SWART, R., and PAN, J. 2001. Climate change 2001: Mitigation. Cambridge: Cambridge University Press. 702 p.

NELSON, F.E., ANISIMOV, O.A., and SHIKLOMANOV, N.I. 2001. Subsidence risk from thawing permafrost. Nature 410: 889–890.

NEWTON, J. 1995. An assessment of coping with environmental hazards in northern aboriginal communities. The Canadian Geographer 39(2):112–120.

———. 1997. Coping with floods: An analogue for dealing with the transition to a modified climate in the northern sector of the Mackenzie Basin. In: Cohen, S.J., ed. Mackenzie basin impact study (MBIS): Final report. Downsview, Ontario: Environment Canada. 219–224.

NICHOLLS, R.J., HOOZEMANS, F.M.J., and MARCHAND, M. 1999. Increasing flood risk and wetland losses due to global sea-level rise: Regional and global analyses. Global Environmental Change 9:69–87.

NICKELS, S., FURGAL, C., CASTLEDEN, J., MOSS-DAVIES, P., BUELL, M., ARMSTRONG, B., DILLON, D., and FONGER, R. 2002. Putting the human face on climate change through community workshops: Inuit knowledge, partnerships, and research. In: Krupnik, I., and Jolly, D., eds. The earth is faster now: Indigenous observations of Arctic environmental change. Fairbanks, Alaska: Arctic Research Consortium of the United States. 300–333.

NORTON, D. 2002. Coastal sea ice watch: Private confessions of a convert to indigenous knowledge. In: Krupnik, I., and Jolly, D., eds. The earth is faster now: Indigenous observations of Arctic environmental change. Fairbanks, Alaska: Arctic Research Consortium of the United States. 126–155.

NTI (NUNAVUT TUNNGAVIK INCORPORATED). 2001. Elder's conference on climate change. Iqaluit: NTI.

———. 2003. Inuit environment workshop. Iqaluit: NTI.

O'BRIEN, K.L., and LEICHENKO, R.M. 2000. Double exposure: Assessing the impacts of climate change within the context of economic globalization. Global Environmental Change 10(3):221–232.

PARRY, M., and CARTER, T.R. 1998. Climate impact assessment and adaptation assessment. London: Earthscan Publications.

PARSON, E.A., CORELL, R., BARRON, E.J., BURKETT, V., JANETOS, A., JOYCE, L., KARL, T.R., MacCRACKEN, M.C., MELILLO, J., MORGAN, M.G., SCHIMEL, D.S., and WILBANKS, T. 2003. Understanding climatic impacts, vulnerabilities, and adaptation in the United States: Building a capacity for assessment. Climatic Change 57(1):9–42.

PELLING, M. 2002. Assessing urban vulnerability and social adaptation to risk: Evidence from Santo Domingo. International Development Planning Review 24(1):59–76.

POLSKY, C., SCHRÖTER, D., PATT, A., GAFFIN, S., MARTELLO, M.L., NEFF, R., PULSIPHER, A., and SELIN, H. 2003. Assessing vulnerabilities to the effects of global

change: An eight-step approach. Belfer Center for Science and International Affairs, Cambridge, Massachusetts: Harvard University. 31 p.

SEN, A. 1981. Poverty and famines: An essay on entitlement and deprivation. Oxford: Clarendon Press. 416 p.

———. 1990. Food economics and entitlements. In: Dreze, J., and Sen, A., eds. The political economy of hunger. Vol. 1. Oxford: Clarendon Press. 34–50.

SERREZE, M.C., WALSH, J.E., CHAPIN, F.S., III, OSTERKAMP, T., DYURGEROV, M., ROMANOVSKY, V., OECHEL, W.C., MORISON, J., ZHANG, T., and BARRY, R.G. 2000. Observational evidence of recent change in the northern high-latitude environment. Climatic Change 46(1-2):159–207.

SHAW, J., TAYLOR, R.B., SOLOMON, S., CHRISTIAN, H.A., and FORBES, D.L. 1998. Potential impacts of sea level rise of Canadian coasts. The Canadian Geographer 42(4):365–379.

SMIT, B., and PILIFOSOVA, O. 2001. Adaptation to climate change in the context of sustainable development and equity. In: McCarthy, J., Canziani, O.F., Leary, N.A., Dokken, D.J., and White, K.S., eds. Climate change 2001: Impacts, adaptation, vulnerability. Contribution of Working Group II to the Third Assessment Report of the Intergovernmental Panel on Climate Change. Cambridge: Cambridge University Press. 876–912.

———. 2003. From adaptation to adaptive capacity and vulnerability reduction. In: Smith, J.B., Klein, R.J.T., and Huq, S., eds. Climate change, adaptive capacity, and development: London: Imperial College Press. 9–28.

SMIT, B., BURTON, I., KLEIN, R.J.T., and STREET, R. 1999. The science of adaptation: A framework for assessment. Mitigation and Adaptation Strategies for Global Change 4: 199–213.

SMIT, B., BURTON, I., KLEIN, R.J.T., and WANDEL, J. 2000. An anatomy of adaptation to climate change and variability. Climatic Change 45(1):223–251.

SMITH, J.B., and LAZO, J.K. 2001. A summary of climate change impact assessments from the U.S. Country Studies Program. Climatic Change 50(1-2):1–29.

SMITH, J.B., KLEIN, R.J.T., and HUQ, S. 2003. Climate change, adaptive capacity, and development. London: Imperial College Press. 356 p.

SMITH, S.L., and BURGESS, M. 1999. Mapping the sensitivity of Canadian permafrost to climate warming. Interactions between the cryosphere, climate and greenhouse gases. Proceedings of International Union of Geodesy and Geophysics (IUGG) 99 Symposium HS2, July 1999, Birmingham. IAHS Publication No. 256. 71–80.

SMITHERS, J., and SMIT, B. 1997. Human adaptation to climatic variability and change. Global Environmental Change 7(2): 129–146.

SOLOMON, S., and HART, E. 1999. Storminess in the western Arctic: Extending the historical record using Hudson's Bay company archives. Environmental Monitoring and Assessment Network Annual Science Meeting, Victoria. http://www.eman-rese.ca/eman/reports/publications/99_national/hudsonbay.html. Accessed November 2003.

TURNER, B., KASPERSON, R.E., MATSON, P.A., McCARTHY, J., CORELL, R., CHRISTENSEN, L., ECKLEY, N., KASPERSON, J.X., LUERS, A., MARTELLO, M.L., POLSKY, C., PULSIPHER, A., and SCHILLER, A. 2003. A framework for vulnerability analysis in sustainability science. Proceedings of the National Academy of Sciences 100(14):8074–8079.

TYNAN, C.T., and DeMASTER, D.P. 1997. Observations and predictions of Arctic climate change: Potential effects on marine mammals. Arctic 50(4):308–322.

USHER, P.J. 2000. Traditional ecological knowledge in environmental assessment and management. Arctic 53(2): 183–193.

———. 2002. Inuvialuit use of the Beaufort Sea and its resources, 1960–2000. Arctic 55(Supp. 1):18–28.

WEAVER, A.J. 2003. The science of climate change. Geoscience Canada 30:169–187.

WELLER, G., and LANGE, M. 1999. Impacts of global climate change in the Arctic regions. Fairbanks: International Arctic Science Committee, Center for Global Change and Arctic System Research, University of Alaska. 1–59.

WHITE, R., and ETKIN, D. 1997. Climate change, extreme events and the Canadian insurance industry. Natural Hazards 16(2):135–163.

ZEIDLER, R.B. 1997. Climate change vulnerability and response strategies for the coastal zone of Poland. Climatic Change 36 (1–2):151–173.
