# Supplementary material for: Systematic Review of Methods in Low-Consensus Fields: Supporting Commensuration through `Construct-Centered Methods Aggregation’ in the Case of Climate Change Vulnerability Research
Source: PLoS One. 2016 Feb 22;11(2):e0149071. doi: 10.1371/journal.pone.0149071 (PMC4762661; doi:10.1371/journal.pone.0149071)
Supplement: S2 File — Source articles and bibliography. (ZIP) [file pone.0149071.s002.zip › data requirements/Khan&Salman.pdf]

Int. J. Disaster Risk Sci. 2012, 3 (3): 163–176
doi:10.1007/s13753-012-0017-z

ARTICLE

A Simple Human Vulnerability Index to Climate Change Hazards for Pakistan

Fazal Ali Khan[1,2,*] and Ali Salman[1,3]

[1]Social Science and Economics Department, Shaheed Zulfikar Ali Bhutto Institute of Science and Technology (SZABIST), Islamabad 44000, Pakistan
[2]Rural Support Programmes Network (RSPN), Islamabad 44000, Pakistan
[3]Development Pool, Islamabad 44000, Pakistan

Abstract This article explores the spatial pattern of vulnerability to climate change hazards in Pakistan by developing a Human Vulnerability Index (HVI). For this purpose, we use Population Census 1998 and Agriculture Census 2000 data. The HVI places the 103 districts of Pakistan in rank order and looks at whether there is a correlation between human vulnerability and exposure to disaster of the districts with respect to climate change hazards such as floods. The HVI is further validated using an independent flood recovery data set. The study found that the HVI is a useful tool for identifying vulnerable regions and districts for resource allocation. But the HVI is a poor tool for vulnerability assessment at community and household levels. For this purpose we used logistic regression analysis, which indicates that the adult literacy rate, ownership of livestock, and access to electricity are the three (out of six) key variables that play a critical positive role in recovery after the 2010 floods. The primary data collected from households also reveal that the 2010 Pakistan floods have equally affected standing crops, livestock, and house structures. More than two-thirds of sample households had rebuilt their house structures, whereas livestock recovery was negligible since the floods. We also found that the 2010 floods affected some of the poverty regions of the country, but that there is a very weak systematic correlation between human vulnerability and disaster exposure.

Keywords climate change, Pakistan, index validation, flood vulnerability, Human Vulnerability Index (HVI)

1 Introduction

The 2010 floods in Pakistan had a devastating effect on the Pakistani population. From livelihoods of rural populations to food security in urban areas, the core gateway transport, communication, energy, health, water control, and institutional systems upon which populations depended failed during the floods. The flood had immediate consequences for people across all levels of society in Pakistan but the impact on poor and vulnerable populations was direct and severe. According to the joint damage recovery needs assessment undertaken by the Asian Development Bank and the World Bank for the Government of Pakistan, the cost of recovery was estimated at USD 8.74 to 10.85 billion (ADB, WB, and GOP 2010). The 2010 flood disproportionately affected the already deprived and poorest regions, such as rural Sindh and southern Punjab. The majority of the population in these regions has limited income diversification and is mostly dependent on agriculture. The flood not only damaged standing crops, but also deprived rural dwellers of their assets and livelihoods, thereby pushing them deeper into poverty (Arif, Iqbal, and Farooq 2010).

These problems are likely to increase as climate change impacts become more pronounced and floods emerge as a regular phenomenon in Pakistan. According to the Climate Change Vulnerability Index created by Maple Croft, an organization that maps over 100 global risks, Pakistan's ranking has been downgraded to 16 in 2010–2011 from its previous position of 29 a year earlier in the list of countries most vulnerable to disasters due to climate change (Maple Croft 2011). In 2011, floods hit Sindh province and 500 people were killed, 5.2 million people were affected as 797,000 houses were damaged and 328,555 were destroyed. The 2011 floods also impacted 2.28 million acres of standing crops. People lost their primary means of livelihood and agricultural production was severely damaged (UNOCHA 2011).

This research develops an easy to use, objective Human Vulnerability Index (HVI) to help flood response programs in Pakistan target services, track changes in vulnerability over time, and report on the status of community vulnerability at different administrative levels from village to province.[i] The research focuses primarily on flooding, because of the immediate challenges posed by the events in 2010, but the index can also be used for other disasters. The organization of the article is as follows: it first presents a short review of relevant literature, describes the methodology used to construct the HVI, and presents the results generated by the HVI. The final section briefly provides the main conclusions of the HVI project and makes some recommendations for future research.

* Corresponding author. E-mail: fazalean.eco@gmail.com

© The Author(s) 2012. This article is published with open access at Springerlink.com

2 Literature Review

Vulnerability is an important part of hazard and risk research. Vulnerability refers to the susceptibility of people, communities, and regions to natural, human made, or technological hazards (Kumpulainen 2006). There is considerable debate about the conceptualization and definition of vulnerability in the academic community. Physical scientists have typically focused on physical exposure to extreme events and its outcome. Social scientists have stressed social structures and differential access to resources, and the particularly salient exposure of certain social groups to disasters (Adger 2006). Some scholars have also tried to integrate both the physical and social aspects of hazard in an attempt to create a comprehensive understanding of the "vulnerability of place" (Cutter 1996; Cutter, Mitchell, and Scott 2000). In this article, we do not revisit the definition debate but instead focus on regional vulnerability and develop a simple quantitative vulnerability index specific to the regions and districts of Pakistan. We understand vulnerability to be more of a chronic state of being rather than the outcome of an extreme climate change event. Therefore we define vulnerability as damage potential and coping capacity, that is, damage potential + coping capacity = regional vulnerability (McCarthy et al. 2001; Mustafa 1998). We have also attempted to validate the HVI with field survey data gathered on the recovery status of households in the wake of the 2010 Pakistan floods. Households that showed an inability to reconstruct their houses after the 2010 floods are considered as the vulnerable group.

Developing vulnerability indices at the subnational level is a common approach that is being increasingly applied in countries like the United States of America (Clark et al. 1998; Cutter, Mitchell, and Scott 2000; Wu, Yarnal, and Fisher 2002; Rygel, O'Sullivan, and Yarnal 2006; Yarnal 2007), the United Kingdom (Tapsell et al. 2002), Spain (Weichselgartner 2002), Latin America (Cardona 2005), Australia (Dwyer et al. 2004), the Philippines (Acosta-Michlik 2005), Germany (Fekete 2009; Kropp et al. 2006), or generally for regions worldwide (Mustafa et al. 2011). In Pakistan, there has been a general lack of such efforts except for a few attempts that have been made to develop deprivation indices to carry out poverty ranking and poverty mapping at the provincial and district levels. Jamal et al. (2003) developed a Multiple Deprivation Index (MDI) for each district based on the combined education, health, housing quality, housing services, and employment sectoral indices. Said, Musaddiq, and Mahmud (2011) developed a basic need index and asset index using the Pakistan Social and Living Standards Measurement Survey (2007–2008) data set. There is still no comprehensive profile of human vulnerability or subnational index map at the district and lower administrative levels in the context of climate change hazards. Apart from filling this gap in this article, we also validate the HVI with data gathered recently from local communities on the status of recovery and nonrecovery, which is rarely done while developing indices.

3 Data Sources and Methodology

The variables in the HVI are derived from the population, housing, and agriculture censuses conducted by the Government of Pakistan (1998, 2000). The indicator selection criteria include requirements of being objectively verifiable and measureable, and being easily available for all districts of Pakistan. The HVI is developed so it can be easily and consistently applied to all four provinces, 143 districts, and to any subset within the districts (tehsil, union councils, and villages) that may be of interest to particular donor organizations, national, provincial, and local governments, and development organizations working in that area. The results will be shared with development organizations, policy-makers, and donor agencies for wider adoption.

3.1 Data Sources

The data used for most of the variables to construct the HVI is derived from the Government of Pakistan's Population and Housing Census 1998. This 1998 census is the only survey that provides data on some of the important social and economic variables at the village/*muza* level and each administrative tier of the government such as those at the union council, tehsil, district, province, and national levels.[ii] These indicators are related to demography (population density), literacy, and housing structures and housing facilities (sanitation and electricity). Although the data set available is more than a decade old, the HVI indicators selected are not highly time sensitive and therefore these data are still relevant to use for developing the HVI. The Government of Pakistan's Agriculture Census 2000 data have been used to obtain the information on household status such as farm households, livestock households, or non-agricultural households. This Agriculture Census classifies rural households under three broad categories: agricultural households that operate land as owner-cultivators or tenants, livestock owners, and non-agricultural households. This information leads to an understanding of the sources of income across the households and their vulnerability in case of disasters such as floods. The HVI study also examined the districts by their extent of damage in the 2010 floods. For this purpose, we have used the damage classification developed by Arif, Iqbal, and Farooq (2010), who used the assessment of flood damages made by various national and international institutes as primary sources for classifying the districts into three categories: severely, moderately, and not affected districts.

To test the validity of the HVI and its indicators, we have used primary data gathered in an ongoing research project entitled Building Research Capacity to Understand and Adapt to Climate Change in the Indus Basin, Pakistan, commonly known as the Indus Flood Research Project (IFRP) (ISET and RSPN 2011–2012). This research project was conducted jointly by the Institute of Social and Environmental Transition (ISET) and the Rural Support Programmes Network (RSPN) in Pakistan.[iii] These data include a total sample of 235 households in 11 villages and four districts of Pakistan.

3.2 Dimensions and Variables for Developing the Human Vulnerability Index (HVI)

The HVI is a summary measure of human vulnerability. It measures the deprivation (vulnerability) in five key basic dimensions of human development or resilience that help to cope with climate change effects such as floods. The five dimensions and the variables are described below.

(1) Population density: Vulnerability to the effects of climate change consists of vulnerability to death, displacement, trauma, and loss of assets and livelihoods. This is measured by population density. A higher population density indicates higher vulnerability in times of disaster, such as floods and earthquake, since more human causalities, injures, and displacement occur where more people reside (Birkmann 2006). Cutter, Mitchell, and Scott (2000) also used total population as a variable of vulnerability. But Cross (2001) argues that small cities and rural communities—which by definition have a lower population density—are more vulnerable to disasters, because small and scattered populations have fewer resources to deal with hazards and disasters than large cities and megacities.

(2) Lack of knowledge: Exclusion from the world of reading and communications, as measured by the adult illiteracy rate, is an additional factor affecting increased vulnerability. The ability to read and write and language skills improve access to information. Access to information is particularly important in times of disasters. Also, a literate population is better able to lobby for political and civil rights, which in turn allows people to demand a more accountable and effective government. Where such rights exist, governments are more likely to become accountable for reducing the impact of successive high mortality disasters, and are therefore more likely to address vulnerability (Wisner et al. 2002).

(3) Lack of decent housing: Lack of access to a proper housing facility, as measured by the weighted average of two variables, percentage of population having *kacha* (weighted 3/6) and semi-*pacca* (weighted 1/6) houses, is linked closely to vulnerability.[iv] The respective weights calculated for *kacha* and semi-*pacca* houses are based on estimates of the Damage and Needs Assessment (ADB, WB, and GOP 2010). This study determined that the proportion of *kacha* house damage was six times higher than that of *pacca* structures. The housing variable is also a proxy for wealth and assets. The housing sector was directly affected by the 2010 flood, which in addition to crops and infrastructure, badly damaged dwellings, particularly in rural areas (Arif, Iqbal, and Farooq 2010).

(4) Lack of decent standard of living: Lack of access to overall socioeconomic provisions is measured by the average of two variables: the percentage of the population without access to piped water and the percentage of population without access to electricity. Lack of access to safe drinking water impacts people and likely affects the poor more and makes them more vulnerable. Climate change causes floods that result in contamination of drinking water and exacerbate the spread of disease and adverse impact on the health of the population. Women and children particularly face difficulties in times of disasters as they are the ones who fetch water and give up their time and energy. Lack of access to piped water makes them more vulnerable. Similarly lack of access to electricity hampers the ability of communities to diversify their livelihoods and increases their ecosystem-based sources of production, hence increases their vulnerability.

(5) Livestock households and farm households: Approximately 65 percent of Pakistan's population lives in rural areas, and there is no industrial base in the rural areas. A recent study conducted by Arif, Iqbal, and Farooq has shown that households situated in the severely flood affected areas heavily depend on agriculture, livestock, and casual labor with negligible flows of foreign remittances and lack of industrial base (Arif, Iqbal, and Farooq 2010). Therefore, households depending on agriculture and livestock are the most direct victims of floods and are highly vulnerable. Arif, Iqbal, and Farooq (2010), using the 2000 Agriculture Census, classify rural households into three broad categories: farm households that operate land as owner-cultivator or tenants; livestock households that have at least one cow or buffalo, 5 sheep and/or goats, and operate no farm area; and non-agriculture households that do not fall into farm and livestock household categories. In making the human vulnerability index we used two variables: percent of households classified as farm households and percent of households classified as livestock households in each district.

3.3 Computation of Human Vulnerability Index (HVI) Scores and Ranking

For scoring, we borrowed the relative scoring method used in the Economic Freedom in Pakistan Index (Salman and Khalil 2009), because this study has several distinct and useful features. First, it is a standard normalization technique, which is unit free. Even when the raw data are not available in percentage form, this formula brings out a score that is not sensitive to the unit of raw data. Second, it automatically awards 10 marks (maximum) to the highest scoring district and 0 to least scoring district. This normalization technique ensures that each district receives a score, which is relative to the lowest and the highest scores in that particular variable.

All the variables mentioned in the previous section are given in percentages, except for population density. All the variables are arranged in such a way that the higher the percentage, the higher the vulnerability of those districts. For example, the higher the percentage of illiteracy in a district, the higher is the vulnerability of that district. So, lower illiteracy will be the desirable outcome. According to the Economic Freedom Index (Salman and Khalil 2009), the formula is given as follows:

$$(V_i - V_{\min}) / (V_{\max} - V_{\min}), \text{ multiplied by 10.}$$

Where:
V_i = Value of a variable (absolute value, ratio, or percentage) of the district i;
$V_{\min}$ = Lowest value of the variable in all districts;
$V_{\max}$ = Highest value of the variable in all districts.

Multiplying by 10 gives a score between 0 to 10, where 0 stands for minimum vulnerability (highest resilience) and 10 stands for maximum vulnerability (least resilient). To calculate the HVI value for each district, we take a simple arithmetic mean of all six index variables for each district. The HVI value thus again gives a score between the lowest possible value of 0 (highest resilience) and 10. Finally we rank the districts according to the HVI values in descending order and divide the list into five quintiles with 20 percent of the districts in each quintile. The last step is to calculate the HVI incidence or average of HVI values in each quintile.

4 Results

This section presents the HVI results by means of vulnerability mapping of the districts, followed by a comparison of human vulnerability with exposure to floods. The section also presents descriptive statistics about the 2010 flood damage and recovery status and the results of the HVI validity test using logistic regression analysis.

4.1 Vulnerability Mapping

Based on the proposed vulnerability index, we mapped the vulnerability of the 103 districts of the country.[v] Figure 1 presents the vulnerability map of all the districts. The spatial representation of the vulnerability of the districts provides a powerful tool to identify clusters, trends, and patterns (Davis 2002).

As shown in Table 1, the majority of the districts in Balochistan are highly vulnerable, with 46 percent of the two top quintile districts belonging to Balochistan, followed

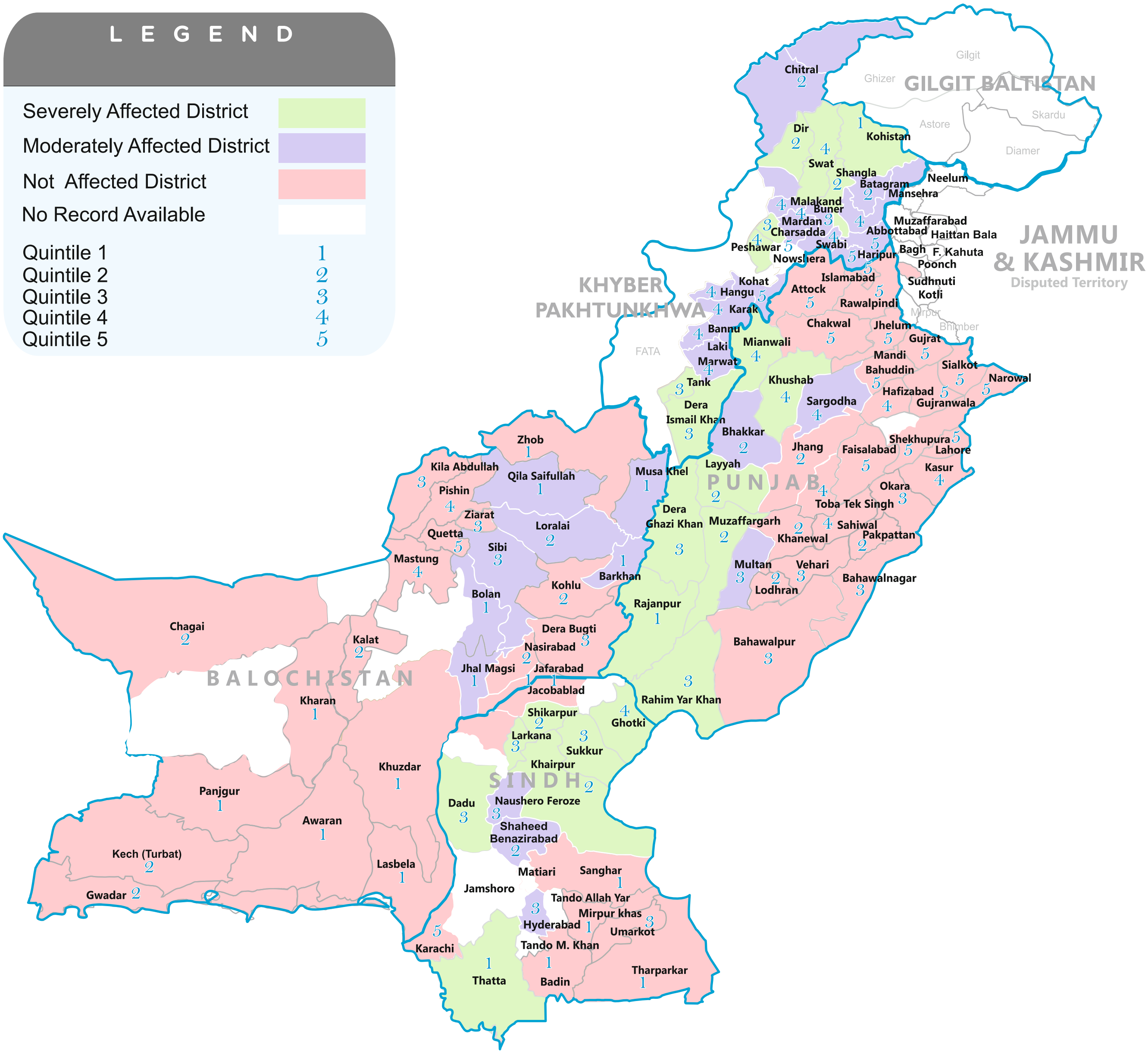

Figure 1. Spatial mapping of the Human Vulnerability Index (HVI) quintiles and the 2010 flood effects

Table 1. Quintile distribution of number of districts on the Human Vulnerability Index (HVI)

Province	Quintile 1 (Top HVI)	Quintile 2	Quintile 3	Quintile 4	Quintile 5 (Bottom HVI)	Total
Balochistan	12	7	4	2	1	26
KPK	1	4	4	11	4	24
Punjab	1	7	7	7	13	35
Sindh	6	3	6	1	2	18
**Total**	**20**	**21**	**21**	**21**	**20**	**103**

by Sindh, which has a share of 22 percent in the top two quintiles. Among the least vulnerable districts, Punjab has the highest share with 65 percent of lowest quintile, followed by the Khyber Pakhtunkhwa (KPK) districts that constitute one-fifth of the lowest quintile. In Balochistan, only Quetta district and in Sindh, only Karachi West and Malir districts have a place in the least vulnerable category. Overall, the top 20 districts score an average of 5.38 in HVI and the lowest 20 districts score an average of 2.49.

To further narrow down, we looked at the top ten and bottom ten districts ranked on the HVI. The results are presented in Table 2. Musakhel and Awaran districts of Balochistan are the top two districts on the HVI, and Rawlpindi and Gujrat of Punjab and the federal capital of Islamabad are the three least vulnerable districts on the HVI. As shown in Table 2, seven out of the top 10 most vulnerable districts in the HVI are from Balochistan, two from Sindh, and one from KPK. Among the least vulnerable districts, five are from Punjab, and only Haripur from KPK and Quetta (the provincial capital) from Balochistan appear on the 10 least vulnerable districts list. For Sindh, only two districts in the provincial capital of Karachi fall in the ten least vulnerable districts. Islamabad is the least vulnerable among all the districts. Surprisingly, the capital district of Punjab, Lahore, ranks 84 and the capital of KPK, Peshawar, ranks 77. In the case of Lahore, the main reason for this is its high population density and in the case of Peshawar, its high illiteracy rate has primarily contributed to its poor ranking.

4.2 Spatial Vulnerability Mapping at Provincial Level

Spatial mapping of human vulnerability indices at the provincial level would help in providing an overview of the results at this level and would identify areas of concern for respective provincial governments (Said, Musaddiq, and Mahmud 2011). This is particularly important given the recently passed 18th Amendment, the 7th National Finance Commission (NFC) Award and the process of devolution under which disaster management has become the responsibility of the provinces, which was earlier a federal subject. Under the 18th Amendment the concurrent list was abolished and a number of ministries including disaster management and environment were transferred from the federation to provinces. The NFC Award is about the distribution of revenues between the federation and provinces. Under the 7th NFC Award the provinces and the federation will expend their tax net to increase their revenue collection and transfer a large share of the federal revenue to the provinces.

Table 3 presents the top three and bottom three districts in each of the provinces in terms of the HVI. The federal capital territory of Islamabad ranks as the least vulnerable district among all the districts at the national level. Among the Punjab districts, the Rawalpindi, Gujrat, and Jhelum districts are least vulnerable. As expected, in Balochistan and Sindh, the provincial capitals are among the top three least vulnerable districts within the provinces. However, for KPK and Punjab, the provincial capitals did not feature as the least vulnerable districts. Among the most vulnerable districts, the top three vulnerable districts in Balochistan, one in KPK, and two in Sindh also appeared in the top districts in the national ranking. All of the three most vulnerable districts within Punjab province and two within the KPK province are ranked far away from the top ten at national level.

4.3 The Human Vulnerability Index (HVI) Key Variables

Figure 2 shows how much each of the selected variables contributes to the HVI scores in all the districts and Figure 3

Table 2. Top 10 and bottom 10 districts on the Human Vulnerability Index (HVI)

Top 10 Vulnerable Districts				Bottom 10 Vulnerable Districts			
Rank	Province	District	Index Value	Rank	Province	District	Index Value
1	Balochistan	Musakhel	6.14	94	Punjab	Sialkot	2.35
2	Balochistan	Awaran	6.09	95	KPK	Haripur	2.33
3	KPK	Kohistan	5.86	96	Sindh	Karachi West	2.30
4	Sindh	Badin	5.75	97	Sindh	Malir	2.29
5	Balochistan	Lasbela	5.68	98	Punjab	Gujranwala	2.20
6	Balochistan	Panjgur	5.66	99	Punjab	Jhelum	2.20
7	Balochistan	Kharan	5.54	100	Balochistan	Quetta	2.17
8	Balochistan	Jhal magsi	5.50	101	Punjab	Gujrat	2.14
9	Sindh	Tharparkar	5.41	102	Punjab	Rawalpindi	1.97
10	Balochistan	Khuzdar	5.39	103	Federal capital	Islamabad	1.05

Table 3. Province-wise top three and bottom three districts on the Human Vulnerability Index (HVI)

	Balochistan		KPK		Punjab		Sindh	
	District	Rank	District	Rank	District	Rank	District	Rank
Bottom 3	Quetta	100	Haripur	95	Rawalpindi	102	Malir	97
	Pishin	70	Nowshera	93	Gujrat	101	Karachi West	96
	Mastung	67	Kohat	90	Jhelum	99	Ghotki	83
Top 3	Lasbela	5	Chitral	36	Bhakkar	31	Thatta	11
	Awaran	2	Shangla	24	Muzaffargarh	27	Tharparkar	9
	Musakhel	1	Kohistan	3	Rajanpur	16	Badin	4

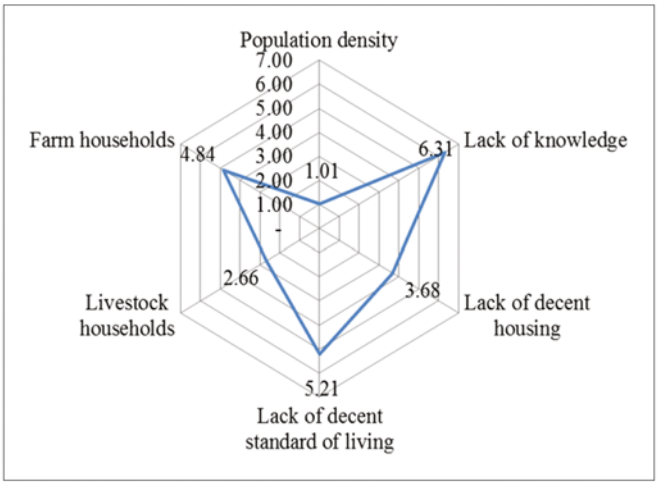

Figure 2. Average score of the key variables of the Human Vulnerability Index (HVI) in all districts

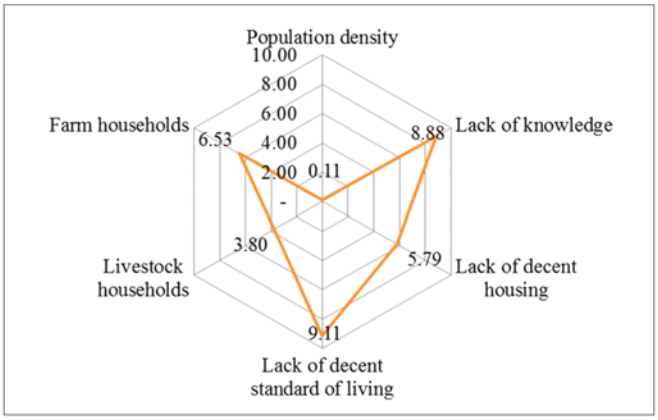

Figure 3. Average score of the key variables of the Human Vulnerability Index (HVI) in the top 10 most vulnerable districts

presents the contribution of each variable in the ten most vulnerable districts.

Comparing the two figures, we found that the lack of decent standard of living, exclusion from the world of knowledge, dependence on farming alone, dependence on livestock, and the lack of decent housing are the main contributors to households' vulnerability to disasters, such as floods. Population density seems to be less important, contributing least to the HVI score of the ten most vulnerable districts. All other variables contribute significantly to the HVI scores of the ten most vulnerable districts.

4.4 Comparison of Human Vulnerability with Exposure to Floods

The 2010 floods in Pakistan affected more than half (55) of the total 103 districts. Among the affected districts, half were severely affected and half were moderately affected (Figure 1). All of the KPK districts, around two-thirds of the districts of Sindh, and one-third of the districts in Punjab and Balochistan were affected. Among the affected districts, a high proportion were in Sindh (73%), followed by Punjab (64%), KPK (42%), and Balochistan (22%).

The districts affected in central and northern Punjab are in the command area of the Jhelum and Chenab Rivers, whereas in southern Punjab the areas are affected by the river Indus. In Sindh, the most affected districts are in upper Sindh, due to the flood of river Indus. In KPK, all the districts were affected largely in the south rather than in the north as a result of floods of the Indus River system. In Balochistan, the area affected by flood is largely in the eastern side of the province, at the right bank of the Indus. This implies that flood exposure (near river) is one of the main contributing factors to severe flooding.

Generally, the floods have severely affected some of the poorest regions of the country, including southern Punjab, northern Sindh, and eastern Balochistan. We have therefore hypothesized that there is a strong positive relationship between flood exposure and human vulnerability among these districts. For this purpose, we performed correlation analysis in SPSS (Statistical Package for the Social Sciences) using the HVI scores and taking the 2010 flood damages as a proxy for flood exposure. We used flood damages classified by the National Disaster Management Authority (NDMA) and UNHCR (United Nations High Commissioner for Refugees), which were also used in Arif, Iqbal, and Farooq (2010). They classified all the districts into three categories: (1) districts severally affected by the 2010 flood; (2) districts affected moderately by the flood; and (3) districts not affected by the flood. The districts were scored according to the damages of the 2010 flood: severely affected as 2, moderately affected as

1, and not affected as 0. Correlation analysis between the flood damage categories and HVI scores in SPSS was then performed. The analysis shows that the relationship between exposure to floods (measured by flood damages of the 2010 floods) and human vulnerability (measured by the score of the HVI) is positive but very weak, with $r = 0.14$, $n = 103$, $p < 0.151$, and r^2 showing only a two percent shared variance. The flood exposure helps to explain only two percent of the variance in the districts' HVI. This is further explained in Figure 4, where we have compared the HVI score quintile distribution of districts according to the level of 2010 flood damage. Only one-third of the not affected districts are least vulnerable (quintile 5) on the HVI. In the rest of the four quintiles, the number of not affected districts is actually dropping when vulnerability decreases, while the number of the severely affected districts is increasing in the first three quintiles. This is probably because the districts less vulnerable on the HVI are actually close to the river systems and have relatively fertile lands and better social infrastructure. On the other hand the districts away from the river systems are poor in land fertility and socioeconomic provisions thus vulnerable on the HVI, but they have less exposure to the floods.

4.5 Validation of Human Vulnerability by Flood Damages

Data collected by the Indus Flood Research Project (ISET and RSPN 2011–2012) was used to test our HVI. The IFRP selected 11 villages from four main districts of Pakistan to test the resilience of communities of varying composition under the stress of extreme disaster events. The survey included 11 core (such as ecosystem, water, air, land) and gateway (such as communication, financial, social services) systems and their links to resilience of communities in times of disasters. However, we only used the data corresponding to most of the HVI variables, that is, adult literacy rate, access to electricity, access to piped water, ownership of land and ownership of livestock (as proxy for farm households and livestock households respectively as given in the HVI), number of *kacha* structure of rooms, and damage and recovery status of households in the 2010 floods.

The IFRP survey includes a total of 235 households. To have maximum geographical variation along the Indus transacts, the IFRP sample includes the following areas (Figure 5):

- Upper reaches (Chitral district);
- Indus-Kabul confluence and piedmont (Charsada district);
- Plains (Dadu district);
- Desert (Tharparkar district).

These areas represent the major physical features of the Indus River basin. They include the upper reaches where glacial melt feeds the river, the piedmont, the plains, and the desert. These areas also cover a wide social and political spectrum in Pakistan. All the selected sites were severely affected by the 2010 floods. In each selected district, at least two most affected villages were selected and within each of the selected villages, a minimum of 15 households was randomly selected for the household survey.

A direct logistic regression model was used to validate the HVI. This model includes the recovery status of houses as the dependent variable and most of the HVI variables (adult literacy rate, access to electricity, access to piped water, ownership of land, ownership of livestock, and number of *kacha* rooms) as independent variables. The main purpose of the logistic regression is to determine the contribution of each independent variable on the likelihood that the household recovered (in terms of house damage recovery degree) from the flood shocks. The primary findings are presented in the following sections.

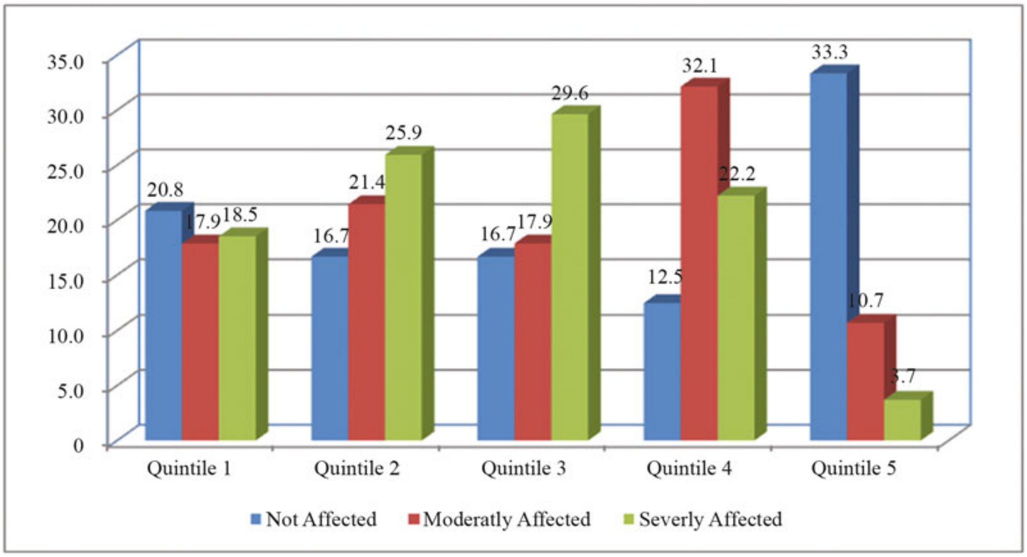

Figure 4. Human Vulnerability Index (HVI) quintile versus flood damage

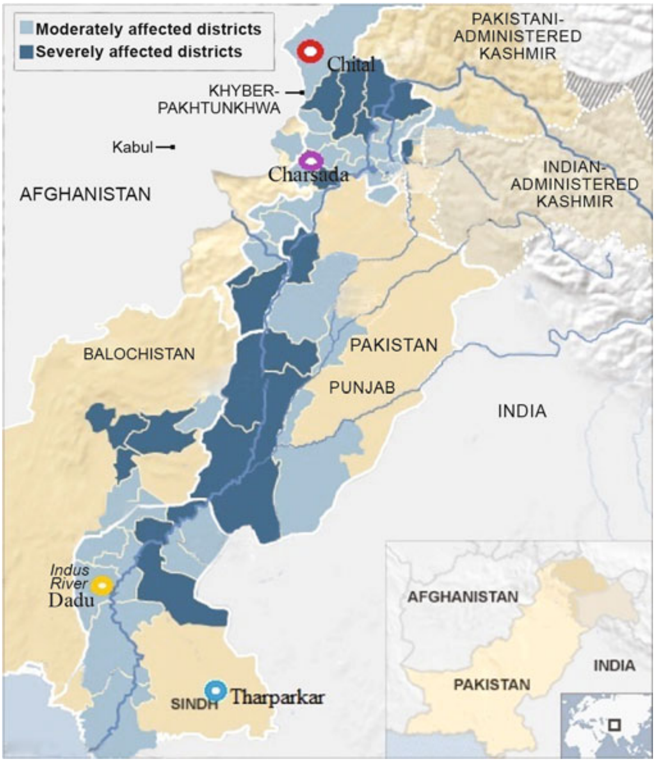

Figure 5. Location of the sample districts of the Indus Flood Research Project (IFRP)
Source: Adapted from UNOCHA 2011.

4.5.1 Descriptive Statistics

In this section we describe the main demographic characteristics of the sample households, including household size, male to female ratio, adults per household, adult literacy rate, and loss and recovery status of households after the 2010 floods. The data presented in this section highlight the difference between geographic locations selected in the sample and can help us to understand the demographic structures of the sample households and damage and recovery status of the households in different sample locations.

As shown in Table 4, the sample households represent a total population of 1651, with an overall male to female ratio of 114:100. The male to female ratio is unexpectedly high, largely because of the very high ratio in the subsamples of Saeed Khan Shhahani (141:100) and Bhakuo (138:100) of Dadu and Tharparkar districts. The difference in this ratio among the districts is partially explained by the adult literacy rate. For Example, Chitral, having the highest literacy rate, has the lowest male to female ratio, followed by Charsada. The overall adult literacy rate is 57 percent, with the highest in Chitral district (71%) and the lowest in Tharparkar district (44%). The IFRP finding of high literacy rate in Chitral is also confirmed by the Pakistan Social and Living Standard Measurement (PSLM) survey 2010–2011, which indicates that Chitral's literacy rate (10+ years) is higher than the national average (62% versus 58%) and in KPK, Chitral is ranked the third, only after Haripur and Abbottabad, in literacy rate. Although Chitral is one of the remotest and mountainous districts of Pakistan, it has at least three unique socioeconomic features that may have contributed to the high literacy rate as compared to the rest of the sample districts: first, due to its difficult terrain, limited land holdings, and lack of industrial base, the local population mainly engage in employment in the service sector. Therefore they place greater emphasis on their children's education in an effort to secure their future economic positions; second, development organizations, particularly the Aga Khan Development Network (AKDN), have long presence of around three decades in the area and have strong networks of schools and other development activities that have helped improve access to and

Table 4. Summary profile of the sample households

District/Village	Number of Sampled Households	Total Population	Household Size	Male/Female (%)	Adults/HHD	Adult Literacy Rate (%)
Charsadda	56	344	6.1	112.3	4.4	63.9
Agra	26	176	6.8	104.7	4.8	64.7
Kharkai	30	168	5.6	121.1	4.1	62.5
Chitral	60	415	6.9	105.4	4.9	70.8
Gouch	15	108	7.2	111.8	4.6	48.8
Madaklasht	15	85	5.7	80.9	4.4	84.6
Rambur	15	120	8.0	126.4	6.0	81.8
Sheikhandeh	15	102	6.8	100.0	4.7	68.6
Dadu	69	506	7.3	121.9	5.1	55.1
Luqman Shahani	28	206	7.4	114.6	5.3	55.2
Saeed Khan Shhahani	17	118	6.9	140.8	4.3	46.8
Seelaro	24	182	7.6	119.3	5.5	60.5
Tharparkar	50	386	7.7	115.6	4.8	44.2
Bhakuo	30	214	7.1	137.8	4.8	50.8
Haryar	20	172	8.6	93.3	4.8	35.2
Total	235	1651	7.0	114.1	4.8	57.1

Data Source: ISET and RSPN 2011–2012.

awareness of education; and third, unlike in many other parts of the country, the government runs fully functional schools across the district. Moreover, there is an emerging private sector network of schools run by the locals in different parts of the district, providing affordable education to both boys and girls.

Table 5 presents the status of animal loss by the sample households during the 2010 floods. In all sample households, 86 percent had animals prior to the floods. Around two-thirds of those who had animals lost almost one-fifth of their livestock. In all sample households, a total of 322 animals, 201 of them being small and 121 being large animals, were lost. The proportion of households that lost animals is highest in Tharparkar, followed by Charsada and Dadu. In Chitral, only seven percent of the households lost animals. Tharpakar is a desert area and had rare experience of floods, but Chitral, being in the mountainous region, is prone to natural disasters especially flash floods, and seems to have developed local adaptation strategies (such as moving animals to safe place and storing fodder, immunizing for diseases) in times of disasters. In Tharparkar the animals could not resist the cold weather, lack of fodder, and disease attacks after the 2010 floods.

The surveyed households were asked about the recovery status of their livestock loss, and the status of the animal loss recovery by September 2011 is as below:

- 100 percent recovery – small animals: Only one household out of 94, and the household is in Agra, Charsada.
- 100 percent recovery – large animals: Three households out of 60, and all these recovered households are in Agra, Charsada.
- Up to 50 percent recovery – large animals: Three households in Chitral and two in Charsada.
- Overall: Only 1.5 percent of those households who lost animals during the floods have shown 100 percent recovery and 4.5 percent households have recovery rates of less than 51 percent.

In addition to this, two of the households in Charsada showed a negative recovery, which means that the animals were either sold or slaughtered post flood.

As shown in Table 6, 57 percent of the sample households had standing crops before the floods. Among them, 90 percent lost their standing crops during the 2010 floods. The crops included cotton and wheat in Dadu, sugarcane in Charsada, wheat and *bajra* in Chitral, and *bajra* in Tharparkar[vi]. The total area of crops lost in the sample households is 506 acres, with an average of 4.2 acres per household. The proportion of households that lost standing crops is the highest in Dadu, followed by Charsada and Chitral. In Tharparkar, all three households with standing crops lost their *bajra* crop.

In 83 percent of the sample households, the houses were either fully damaged or partially damaged. The details of house damage are presented in Table 7. In 69 percent of the sample households, on average 1.6 rooms were fully damaged. The proportion of fully damaged households varies between the sample districts, with the highest in Tharparkar (90%), followed by Dadu (83%) and Charsada (80%). More than one quarter of the houses were also damaged in the Chitral district. In Tharparkar and Dadu, most of the house structures are *kacha* and therefore more vulnerable to flood damage. In 31 percent of the households that experienced

Table 5. Animals lost during the 2010 floods in the sample households

District/Village	Animals Lost During the 2010 Floods						
	Number of Sampled Households	Number of Households that Had Animals Before the Floods	% of Households Had Animals Before the Flood that Lost Animals	Average Animals/ HHD	Total Animals	Small Animals	Large Animals
Charsadda	56	43	83.7	2.0	71	24	47
Agra Payan	26	26	96.2	1.8	44	18	26
Kharakay	30	17	64.7	2.5	27	6	21
Chitral	60	42	7.1	4.7	14	3	11
Gouch	15	14	21.4	4.7	14	3	11
Madaklasht	15	12	–	–	–	–	–
Rambur	15	14	–	–	–	–	–
Sheikhandeh	15	2	–	–	–	–	–
Dadu	69	67	70.1	2.7	125	62	63
Luqman Shahani	28	28	67.9	2.5	48	20	28
Saeed Khan Shhahani	17	15	100.0	2.9	43	19	24
Seelaro	24	24	54.2	2.6	34	23	11
Tharparkar	50	50	90.0	2.5	112	112	–
Bhakuo	30	30	90.0	2.6	71	71	–
Haryar	20	20	90.0	2.3	41	41	–
Total	235	202	64.9	2.5	322	201	121

Data Source: ISET and RSPN 2011–2012.

Table 6. Agricultural crops lost during the 2010 floods in the sample households

District/Village	Number of Sampled Households	Number of Sampled Households that Had Standing Crops Before the Flood	Number of Households Lost Standing Crops	% of Households Lost Standing Crops that Had Standing Crops Before the Flood	Area of Standing Crops Lost (Acres)	Average Area of Standing Crops Lost (Acres)
Charsadda	56	17	14	82.4	42.4	3.0
Agra Payan	26	12	12	100.0	42.0	3.5
Kharakay	30	5	2	40.0	0.4	0.2
Chitral	60	56	45	80.4	156.0	3.5
Gouch	15	15	14	93.3	56.0	4.0
Madaklasht	15	14	9	64.3	34.0	3.8
Rambur	15	15	15	100.0	48.0	3.2
Sheikhandeh	15	12	7	58.3	18.0	2.6
Dadu	69	58	58	100.0	283.0	4.9
Luqman Shahani	28	27	27	100.0	165.0	6.1
Saeed Khan Shhahani	17	13	13	100.0	52.0	4.0
Seelaro	24	18	18	100.0	66.0	3.7
Tharparkar	50	3	3	100.0	25.0	8.3
Bhakuo	30	–	–		–	–
Haryar	20	3	3	100.0	25.0	8.3
Total	235	134	120	89.6	506.4	4.2

Data Source: ISET and RSPN 2011–2012.

Table 7. Houses damaged by the 2010 floods in the sample households

District/Village	Number of Sampled Households	Fully Damaged		Partially Damaged	
		% of Households in Those with Houses Damaged	Average Number of Rooms	% of Households in Those with Houses Damaged	Average Number of Rooms
Charsadda	56	80.4	1.7	10.7	1.2
Agra Payan	26	65.4	1.4	15.4	1.3
Kharakay	30	93.3	1.9	6.7	1.0
Chitral	60	26.7	2.1	18.3	1.2
Gouch	15	40.0	2.7	13.3	2.0
Madaklasht	15	53.3	2.0	46.7	1.0
Rambur	15	13.3	1.0	13.3	1.0
Sheikhandeh	15	–	–	–	–
Dadu	69	82.6	1.7	46.4	1.5
Luqman Shahani	28	85.7	1.3	39.3	1.5
Saeed Khan Shhahani	17	64.7	1.1	35.3	1.2
Seelaro	24	91.7	2.5	62.5	1.5
Tharparkar	50	90.0	1.3	46.0	1.8
Bhakuo	30	96.7	1.2	26.7	1.3
Haryar	20	80.0	1.4	75.0	2.1
Total	235	69.4	1.6	30.6	1.5

Data Source: ISET and RSPN 2011–2012.

damage to house structures, the rooms were partially damaged. The average number of rooms partially damaged is 1.5 per household.

Our survey classifies house structures into three categories: *kacha* rooms, *pacca* rooms, and mixed *kacha-pacca* rooms. Two types of damage occur: fully damaged and partially damaged. Recovery refers to the reconstruction of the fully damaged structures and the repair of partially damaged structures. Given the different types of structure and levels of damage and recovery, a house recovery degree variable is developed (Table 8). The house recovery variable is designed to give high weight to damaged *pacca* structure's reconstruction and repair, and lower weight to damaged *kacha* structure's reconstruction and repair. Finally, we calculated the loss recovery rate. The degree of recovery varies from 0 to 100+, where 0 stands for no recovery, 100 stands for complete recovery (same number of rooms and structure as before the floods), and more than 100 stands for not only a complete recovery but an improvement of preflood structures. The results are presented in Table 8.

As shown in Table 8, of all sampled households with their houses damaged, 12 percent did not recovery from this

Table 8. House structure damage recovery status in the sample households by September 2011

District/Village	Number of Sampled Households	Number of Households with Houses Damaged	House Structure Damage Recovery Rate %				
			No Recovery	Recovery Degree Up to 50	Recovery Degree 50–75	Recovery Degree 75–100	Recovery Degree >100
Charsadda	56	51	27.5	21.6	–	49.0	2.0
Agra Payan	26	21	33.3	9.5	–	57.1	–
Kharakay	30	30	23.3	30.0	–	43.3	3.3
Chitral	60	27	33.3	7.4	3.7	55.6	–
Gouch	15	8	50.0	12.5	–	37.5	–
Madaklasht	15	15	26.7	6.7	6.7	60.0	–
Rambur	15	4	25.0	–	–	75.0	–
Sheikhandeh	15	–	–	–	–	–	–
Dadu	69	68	1.5	17.6	8.8	67.6	4.4
Luqman Shahani	28	28	3.6	14.3	7.1	64.3	10.7
Saeed Khan Shhahani	17	16	–	6.3	–	93.8	–
Seelaro	24	24	–	29.2	16.7	54.2	–
Tharparkar	50	50	–	–	–	100.0	–
Bhakuo	30	30	–	–	–	100.0	–
Haryar	20	20	–	–	–	100.0	–
Total	235	196	12.2	12.8	3.6	69.4	2.0

Data Source: ISET and RSPN 2011–2012.

structural damage, 69 percent of households had a damage recovery degree of 75–100, 13 percent of households had a degree of recovery of up to 50, and another four percent had a damage recovery degree of 50–75. Four households (2%) had a recovery degree of higher than 100. Of these four households, one household lost two of its *kacha* rooms but rebuilt two *pacca* rooms, and one household had one *kacha* room that was fully damaged and one mixed structure room that was partially damaged. This latter household has rebuilt an additional mixed structure room in addition to the repair of the partially damaged room. The remaining two households had two mixed structure rooms each and, in both cases, were partially damaged by flooding. In addition to repairing the damage structures, one of the households has built two additional *kacha* rooms while the other has built an additional *pacca* room. Therefore, these four households have better house structures and a higher numbers of rooms as compared to before the floods.

The recovery rate among the sample districts varies. A 100 percent house recovery rate occurred in Tharparkar. Dadu, Chitral, and Charsada followed in descending scale of recovery. One of the major reasons for the higher rate of recovery in the Sindh districts is the low cost of house construction in these districts.

4.5.2 Regression Analysis

Given that measuring vulnerability to disasters is a complex process, the final and most important research question is whether the HVI, based on variables selected theoretically, is congruent with the actual flood recovery situation of households. For this purpose, we have used physical recovery from extreme flood shocks as a proxy for resilience, and nonrecovery as a proxy for vulnerability. This is possible because we have data available on the recovery status of households for three indicators: livestock loss and recovery, agriculture crop loss, and house structure damage and recovery. As shown in Section 4.5.1, a very small proportion of the households has recovered its lost livestock, but a significant proportion of the households has shown recovery of their damaged house structures. It is common after disasters that people attend to building their houses first given that shelter is their primary need. A possibly longer period of time is needed for the recovery of other assets. For the purpose of testing the validity of HVI based on theory, we used the house recovery degree variable (Table 8), considering the households with a house recovery degree of more than 75 as resilient ($N = 129$) and 75 or less as vulnerable ($N = 96$).

Our direct logistic regression model includes the recovery status of houses as the dependent variable with binary coded recovered = 1 and not recovered = 0. The independent variables consist of most of the HVI variables, including adult literacy rate (ARL), access to electricity (Elect), access to piped water (Water), ownership of land (Land), ownership of livestock (Liv), and number of *kacha* rooms. Four of these six variables (Elect, Water, Land, and Liv) are again binary coded as 1 for yes and 0 for no, but the number of *kacha* rooms and percent of literate adults are actual values.

The model contains all the predicators and is statistically significant, χ^2 (6, $N = 235$) = 20.2, $\rho < 0.005$, indicating that the model is able to distinguish between households that recovered and households that did not recover. The model as a whole explained between 8.2 percent (Cox and Snell R square) and 11 percent (Nagelkerke R square) of the variance in recovery status and correctly classified 63.4 percent of the cases. As shown in Table 9, only three of the independent

Table 9. Logistic regression predicting likelihood of house damage recovery from the 2010 floods

		B	S.E.	Wald	Df	P	Odds Ratio	95.0% C.I. for EXP (B)	
								Lower	Upper
Step 1	ARL**	0.011	0.005	4.687	1	0.03	1.011	1.001	1.021
	Elect***	0.661	0.379	3.039	1	0.08	1.937	0.921	4.073
	Water	–0.131	0.333	0.154	1	0.69	0.878	0.457	1.685
	Liv*	1.257	0.455	7.652	1	0	3.517	1.443	8.571
	Land	0.181	0.286	0.401	1	0.52	1.199	0.684	2.101
	Kacha rooms	–0.156	0.134	1.361	1	0.24	0.855	0.658	1.112
	Constant	–1.590	0.563	7.987	1	0	0.204		

Variable(s) entered on step 1: ARL, Elect, Water, Liv, Land, Kacha rooms.
Note: * Significant at 1%, ** Significant at 5%, *** Significant at 10%; ARL = Adult Literacy Rate, Elect = Access to Electricity, Water = Access to Piped Water, Liv = Livestock Ownership, Land = Land Ownership, *Kacha* Rooms = Number of *Kacha* Rooms.
Data Source: ISET and RSPN 2011–2012.

variables made statistically significant contributions to the model (adult literacy rate, access to electricity, and ownership of livestock). The strongest contributor in recovery (or resilience of households to flood shocks) was ownership of livestock, which recorded the highest odds ratio of 3.5. This indicates that the households who own livestock were over three times more likely to recover than those that did not own livestock, keeping other factors constant. The second contributor was access to electricity, with an odds ratio of 1.9, followed by adult literacy rate with an odds ratio of 1.01. Except the access to water variable, the rest of the variables have the correct relationship (see β signs) as hypothesized in the HVI index but none of these are statistically significant. To further check the regional variability we also performed regression analysis on single district subsamples. The result shows that some of the variables that are insignificant in the overall sample are significant at the district level. For instance Water is insignificant in the overall sample, but is significant in the subsample of Charsada. Land is not significant in the overall sample but significant in the case of the Dadu district subsample. Similarly, Elect was more robust in the case of Chitral than the other districts. In Tharparkar none of the variables were significant.

5 Conclusion and Recommendations

This study set out to develop a Human Vulnerability Index (HVI) at the district level for the 103 districts of Pakistan. The HVI was developed based on variables identified on theoretical grounds and the availability of data. We used the latest available data of the Housing and Population Census of 1998 and the Agriculture Census of 2000. The study first developed the HVI and ranked all the Pakistan districts according to their vulnerability scores, with the purpose of facilitating resource allocations to regions and districts by the government, donors, and development organizations. After reviewing the 2010 flood-affected districts and comparing them using the HVI, we then used household survey data to assess the damages and recovery status of households that were affected by the floods of 2010. Finally, the article validated the HVI variables with real data gathered from the flood-affected households to assess the contribution of each of the variables in building resilience or contributing to the recovery of households from the 2010 flood shocks. Based on that analysis, the following key points have emerged.

- Most of the resource poor and poverty stricken regions and districts also topped the HVI listing.
- Climate change hazards, particularly flood hazards, are neutral to the human vulnerability ranking of the districts. Districts falling in each quintile of the HVI are equally exposed or not exposed to the flood hazards and correlation analysis shows that there is a very weak positive correlation between exposure to floods (as measured by flood damages) and human vulnerability.
- The floods have equally affected the people, livelihood sources such as agriculture crops and livestock, and house structures. But recovery is faster for house structures than the livelihood sources of agriculture and livestock.
- A national-level index may be useful as a resource allocation tool but can be very poor in doing vulnerability assessments, especially in recovery from disasters such as floods. In the overall sample, only three out of the six selected variables are statistically significant. Similarly, some of the variables in the overall sample are insignificant, but when regression is performed for subsamples for single districts, some of the variables become significant—for instance, the Water variable is insignificant in the overall sample but significant in the Charsada district subsample.

The regression results further suggest that the sources of resilience in communities may come from the provision of certain critical services and that it varies in different geographic locations. For instance, in the mountainous region (Chitral district), the advent of electricity has had a critical role in communities' ability to diversify livelihoods and decrease their dependence on ecosystem-based sources of production, thereby improving community resilience. In the

plain areas (Charsada district), access to piped water seems to play a differential role between the recovered and not recovered households, probably because access to improved water reduces waterborne diseases and hence resilience. In the delta (Dadu district), ownership of land is one of the key differences between the recovered and not recovered households. In the desert (Tharparkar district), it seems that some other variables (other than the selected variables) played a key role in differentiating the well recovered and less recovered households as none of the selected variables are significant.

But the question of why some of the factors play a key role in some areas but not in others is beyond the scope of this article. Future research should focus on this emerging question. Also, apart from the HVI variables used in this article, social cohesion and diversification of livelihood options may be important for recovery and building resilience of communities across the districts and worth exploring in future research. Nonetheless the HVI still can be used for the purpose of targeting vulnerable districts for resource allocation prior to disasters and for disaster response. Donors, development and humanitarian organizations, and local governments can also identify vulnerable priority areas at subdistrict level by using population and housing census data of Pakistan and the methodology for constructing the HVI adopted in this article.

Notes

i Pakistan is composed of a core federation of four provinces: Balochistan, Khyber Pakhtunkhwa, Punjab, and Sindh plus Islamabad Capital Territory. Azad Juma Kashmir, Federally Administered Tribal Areas/Frontier Regions, and Gilgit Baltistan are additional administrative districts with varying status and relationship to national core institutions. The country has three-tiered local government system of district, tehsil, and union council. The lowest tier union council consists of a number of villages.

ii Although the data set available is more than a decade old, the selected indicators are not very time sensitive and seem to be relevant for developing the HVI. In addition, we are expecting that the HVI could be easily updated with the availability of new census data.

iii The Indus Flood Research Project (ISET and RSPN 2011–2012) is a project funded by IDRC-Canada and UKAID-DFID and implemented by the Institute for Social and Environmental Transition (ISET) and Rural Support Programme Network (RSPN). The general objective of this research project is to generate knowledge on climate related hazards in the Indus Basin in Pakistan and their impact on marginalized communities so that the specific causes for their vulnerability can be identified and strategies to build resilience may be developed.

iv *Kacha* is mud-based construction, while *pacca* is a brick- or concrete-based structure. A semi *pacca* structure is part *katcha* and part *pacca*.

v We have excluded the Federally Administered Tribal Area (FATA), Gilgit Baltistan (GB), and Azad Jammu and Kashmir (AJK) districts from the analysis due to a lack of available complete data for these districts.

vi *Bajra* is the local name for pearl millet, which is commonly grown in the desert area of Sindh and the mountainous region of northern Pakistan because of its tolerance to difficult growing conditions. It can be grown in areas where other cereal crops such as wheat and maize would not survive. It plays a key role in the local economy of Tharparkar where due to drought other crops could not be grown.

References

Acosta-Michlik, L. 2005. *Intervulnerability Assessment, Shifting Foci From Generic Indices to Adaptive Agents in Assessing Vulnerability to Global Environmental Chnage* (A pilot Project in the Philippines). Louvain-la-Nauve, Belgium: Catholic University of Louvain (UCL), Department of Geography.

ADB (Asian Develoment Bank), WB (World Bank), and GOP (Goverment of Pakistan). 2010. *Pakistan Floods Damage and Need Assessment*. Islamabad, Pakistan.

Adger, W. N. 2006. Vulnerability. *Science Direct* 16 (3): 268–81.

Arif, G. M., N. Iqbal, and S. Farooq. 2010. The 2010 Flood and Poverty in Pakistan: A Preliminary District-Level Analysis. Background paper prepared for the Conference on the Environments of the Poor, 24–26 November 2010, New Delhi. http://www.scribd.com/doc/42890203/Flood-and-Poverty-in-Pakistan-A-Preliminary-Analysis-at-District-level-of-Systemic-and-Disaster-Induced-Causes-of-Poverty-paper 33.

Birkmann, J. 2006. Measuring Vulnerability to Promote Disaster-Resilient Societies: Conceptual Frameworks and Definitions. In *Measuring Vulnerability to Natural Hazards: Towards Disaster Resilient Societies*, edited by J. Birkmann, 9–54. Tokyo: United Nations Univeristy Press.

Cardona, O. D. 2005. *System of Indicators for Disaster Risk Management, Program for Latin America and the Carribean: Summary Report.* Washington, DC: Inter-American Development Bank.

Clark, G. E., S. C. Moser, S. J Ratik, K. Dow, W. B. Meyer, S. Emani, W. Jin, J. X. Kasperson, R. E. Kasperson, and H. E Schwarz. 1998. Assessing the Vulnerablity of Coastal Communities to Extreme Storms: The case of Revere, MA, USA. *Mitigation and Adaptation Stretegies for Global Change* 3 (1): 59–82.

Cross, J. A. 2001. Megacities and Samll Towns: Differnt Perspectives on Hazard Vulnerability. *Environmental Hazard* 3 (2): 63–80.

Cutter, S. L. 1996. Vulnerablity to Environmental Hazards. *Progress in Human Geography* 20 (4): 529–39.

Cutter, S. L., J. K. Mitchell, and M. S. Scott. 2000. Revealing the Vulnerability of People and Places: A Case Study of Georgetown County, South Carolina. *Annals of the Association of American Geographers* 90 (4): 713–37.

Davis, B. 2002. *Is It Possible to Avoid a Lemon? Reflections on Choosing a Poverty Mapping Method.* Agriculture in Economic Development Service, Food and Agricultural Organization of the United Nations.

Dwyer, A., C. Zoppou, O. Nielsen, S. Day, and S. Roberts. 2004. *Quantifying Social Vulnerability: A Methodology for Identifying those at Risk to Natural Hazards.* Canberra City: Geoscience Australia Record.

Fekete, A. 2009. Validation of a Social Vulnerability Index in Context to River-Floods in Germany. *Natural Hazards and Earth System Sciences* 9 (2): 393–403.

Government of Pakistan. 1998. *Population and Housing Census*. Statistics Division, Population Census Organization.

———. 2000. *Agriculture Census*. Statistics Division, Agricultural Census Organization.

ISET (Institute for Social Science and Environmental Transition) and RSPN (Rural Support Programmes Network. 2011–2012. Indus Flood Research Project (IFRP). Unpublished.

Jamal, H., A. J. Khan, I. A.Toor, and N. Amir. 2003. Mapping the Spatial Deprivation of Pakistan. *The Pakistan Development Review* 42 (2): 91–111.

Kropp, J. P., A. Block, F. Reusswig, K. Zickfeld, and H. J. Schellnhuber. 2006. Semiquantitative Assessment of Regional Climate Vulnerability: The North-Rhine Westphalia Study. *Climate Change* 76 (3–4): 265–90.

Kumpulainen, S. 2006. Vulnerablility Concepts in Hazard and Risk Assessment. In *Natural and Technological Haszards and Risks*

Affecting the Spatial Development of European Regions, edited by P. Schmidt-Thomé, 65–74. Geological Survey of Finland Special Paper 42. Helsinki: Centre for Urban and Regional Studies/Helsinki University of Technology.

Maple Croft. 2010. *Big Economies of the Future—Bangladesh, India, Philippines, Vietnam and Pakistan—Most at Risk from Climate Change*. http://maplecroft.com/about/news/ccvi.html.

McCarthy, J. J., O. F. Canziani, N. A. Leary, D. J. Dokken, and K. S. White. 2001. *Climate Change: Impacts, Adaptation and Vulnerablity.* Cambridge: Cambridge University Press.

Mustafa, D. 1998. Structural Causes of Vulnerability to Flood Hazard in Pakistan. *Economic Geography* 74 (3): 289–305.

Mustafa, D., S. Ahmed, E. Saroch, and H. Bell. 2011. Pinning Down Vulnerability: From Narratives to Numbers. *Disasters* 35 (1): 62–86.

Rygel, L., D. O'Sullivan, and B. Yarnal. 2006. A Method for Constructing a Social Vulnerability Index: An Application to Hurricane Storm Surges in a Developed Country. *Mitigation and Adaptation Strategies for Global Change* 11 (3): 741–64.

Salman, A., and A. Khalil. 2009. *Economic Freedom in Pakistan—Sub National Index*. Islamabad: Development Pool Pakistan.

Said, F., T. Musaddiq, and M. Mahmud. 2011. *Macro Level Determinants of Poverty: Investigation through Poverty Mapping of Districts of Pakistan*. Paper presented at the 27th Annual Meeting and Conference of Pakistan Society of Development Economists, 13–15 December 2011, Islamabad. http://www.pide.org.pk/psde/25/index.php?option=com_content&view=article&id=99&Itemid=78.

Tapsell. S. M., E. C. Penning-Rowsell, S. M. Tunstall, and T. L. Wilson. 2002. Vulnerability to Flooding: Health and Social Dimensions. *Philosophical Transactions of the Royal Society A: Mathimatical, Physical and Engineering Sciences* 360 (1796): 1511–25.

UNOCHA (United Nations Office for the Coordination of Humanitarian Affairs). 2011. *Pakistan Floods 2011*. http://reliefweb.int/sites/reliefweb.int/files/resources/Full%20Report_737.pdf.

Wu, S. Y., B. Yarnal, and A. Fisher. 2002. Vulnerability of Coastal Communities to Sea-Level Rise: A Case Study of Cape May County, New Jersey, USA. *Climate Research* 22: 255–70.

Weichselgartner, J. 2002. About the Capacity to Be Wounded: The Need to Link Disaster Mitigation and Sustainable Development. In *Extreme Naturereignisse–Folgen, Vorsorge, Werkzeuge*, edited by G. Tetzlaff, T. Trautmann, and K. L. Radtke, 150–58. Zweites Forum Katastrophevorsorge, 24–26 September, Leipzig. Bonn: German Committee for Disaster Reduction (DKKV).

Wisner, B., P. Blaikie, T. Cannon, and I. Davis. 2002. *At Risk: Natural Hazards, People's Vulnerablity and Disasters*. 2nd ed. London: Routledge.

Yarnal, B. 2007. Vulnerability and All that Jazz: Addressing Vulnerability in New Orleans After Hurricane Katrina. *Technology in Society* 29 (2): 249–55.

Open Access This article is distributed under the terms of the Creative Commons Attribution License which permits any use, distribution, and reproduction in any medium, provided the original author(s) and source are credited.
