# Supplementary material for: Systematic Review of Methods in Low-Consensus Fields: Supporting Commensuration through `Construct-Centered Methods Aggregation’ in the Case of Climate Change Vulnerability Research
Source: PLoS One. 2016 Feb 22;11(2):e0149071. doi: 10.1371/journal.pone.0149071 (PMC4762661; doi:10.1371/journal.pone.0149071)
Supplement: S2 File — Source articles and bibliography. (ZIP) [file pone.0149071.s002.zip › data requirements/MutsvangwaEP.pdf]

Climate Change and Vulnerability to Food Insecurity among Smallholder Farmers: A Case Study of Gweru and Lupane Districts in Zimbabwe

Eness P. Mutsvangwa

Submitted in partial fulfillment of the requirement for the degree of Master of Science in Agriculture (Agriculture Economics)

In the faculty of Natural and Agriculture Sciences

Department of Agricultural Economics

University of Free State
Bloemfontein

South Africa

Abstract

This thesis assesses the vulnerability of smallholder farmers to food insecurity in Gweru and Lupane districts of Zimbabwe and links this to climate change. Current changes in climate for most parts of Zimbabwe have resulted in increased frequency of droughts, dry spells and erratic rainfall. This has resulted in loss of food production and smallholder farmers are most vulnerable to these climatic catastrophes as they affect the food security status of the household. Few studies have been done at local and household levels, most climatic studies have been done at global and national levels. This study seeks to contribute to this knowledge gap.

Poverty and food security studies have proved that poor and food insecure households are more vulnerable to climate change, considering that they have limited options to curb against climate change. Using data obtained from a survey carried out in Gweru and Lupane districts in Zimbabwe, descriptive statistics analysis was undertaken to characterize the households, in terms of gender, education of the household head, cropping patterns of the household, perceptions to climate change and also organizations working within the communities and how they help reduce vulnerability to climate change. Results show that cereal crop production is common and important in these two districts, considering that the largest pieces of land are allocated to cereals. Thus cereals constitute a large proportion to the household's food security. Chuadhuri's model of measuring vulnerability to poverty was used to measure vulnerability to food insecurity for households in Gweru and Lupane districts. Results show about 88% of the households in both districts are vulnerable to food insecurity thus, have more chances of being negatively impacted by climate change.

Dedication

'I dedicate this thesis to the Lord Almighty, my husband Tofara W Sammie, my lovely daughter Ruvarashe Yolanda Sammie and the rest of my family. Love you all so much!'

Acknowledgements

I would like to thank the entire staff of the department of Agriculture Economics, Faculty of Natural Resources and Agriculture Sciences for all the support and encouragement especially Mrs. Annely Minnar and Mrs. Louise Hoffman. Special thanks to my academic advisor and thesis supervisors, Dr G Kundhlande and Professor M F Viljoen, for the mentoring, guidance and assistance throughout my studies.

Special thanks also go to the ICRISAT-Bulawayo team for accommodating me and helping me with my field work and availing working space for my studies. I am really indebted to you for understanding and being there to offer advice and technical help all the time, not forgetting the time I was given to concentrate on my master's work at the expense of ICRISAT work. You are simply the best. I would like to give my sincere gratitude to my immediate boss at ICRISAT, Dr Kizito Mazvimavi. I owe you so much for the support, constant supervision, mentoring and patience. Special thank also goes to the farmers in Gweru and Lupane districts for their cooperation and AGRITEX for the help with the field work. In addition, I would like to thank Professor Mugabe and his team's role in offering me the scholarship and IDRC for funding the scholarship to study for the Masters degree.

Glory to the Lord Almighty, thanks to Christ who was there for me when I thought I would not go on. Dearest husband, Tofara W Sammie, thanks for being there for me, believing in me and giving me the strength to go on. I would also like to thank my family and friends for the support. *Love you all so much and may the good Lord prevail in your lives always.*

Table of Contents

Abstract ..... **i**
Dedication ..... **ii**
Acknowledgements ..... **iii**
Table of Contents ..... **iv**
List of Tables ..... **vi**
List of Figures ..... **vii**
List of Acronyms and Abbreviations ..... **viii**
CHAPTER 1 ..... **1**
INTRODUCTION **1**
1.1. Background 1
1.2. Problem Statement 4
1.3. Objectives 5
1.4. Research Questions 5
1.5. Justification of study 6
1.6. Outline of thesis 7
CHAPTER 2 ..... **8**
LITERATURE REVIEW **8**
2.1. Introduction 8
2.2. Global climate change 8
2.3. Zimbabwe and global climate change 11
2.4. Vulnerability to climate change 15
2.5. Measuring smallholder vulnerability to climate change 18
2.5.1. Vulnerability as Uninsured Exposure to Risk 19
2.5.2. Vulnerability as a Low Expected Utility 19
2.5.3. Vulnerability as expected poverty 19
2.6. Analytical framework 21
2.7. Summary 27
CHAPTER 3 ..... **28**
DESCRIPTION OF STUDY SITES AND RESEARCH METHODOLOGY **28**
3.1. Introduction 28
3.2. Background information 28
3.3. Selection of study sites 29
3.3.1. Administrative set up and agro-ecological regions of Zimbabwe 30
3.3.2. Sampling procedure and description of study sites 32
3.4. Data needs and sample size 39
3.4.1. Quantitative data 40
3.4.2. Qualitative data 41
3.4.3. Secondary data 42
3.5. Data Analysis 43
3.6. Summary 44
CHAPTER 4 ..... **45**
SMALLHOLDER FARMERS LIVELIHOODS, AGRICULTURE AND CLIMATE CHANGE IN GWERU AND LUPANE DISTRICTS **45**
4.1. Introduction 45
4.2. Historical climate trends for the areas of study 45
4.3. Socioeconomic characteristics of the households 48
4.3.1. Asset ownership 52
4.3.2. Crop production in Gweru and Lupane district 57

4.3.3. Other income sources ... 58
4.4. Household consumption and food requirements ... 60
4.5. Farmers perception of climate change ... 63
4.6. Household level adaptation and coping strategies ... 65
4.7. Zimbabwe's policies for address climate change ... 68
4.8. Institutional arrangements in Gweru and Lupane districts ... 69
4.8.1. Agricultural Technical and Extension Services (AGRITEX) ... 70
4.8.2. Zimbabwe Meteorology Services Department ... 71
4.8.3. Traditional Leadership ... 73
4.8.4. Non Governmental Organizations (NGOs) ... 74
4.8.5. Education Sector ... 75
4.8.6. Health Sector ... 76
4.9. Farmers perception on organizations operating in their communities ... 77
4.10. Summary of findings ... 80
CHAPTER 5 ... 81
Measuring Household Vulnerability ... 81
5.1. Introduction ... 81
5.2. Vulnerability of households to food insecurity ... 83
5.3. Summary ... 92
CHAPTER 6 ... 94
CONCLUSIONS AND RECOMMENDATIONS ... 94
6.1. Summary ... 94
6.2. Conclusion ... 95
6.3. Recommendations ... 99
REFERENCES ... 101

List of Tables

Table 1: Interpretations of vulnerability in climate change research....................................17
Table 2: Agro-ecological zones of Zimbabwe and the recommended farming systems in each zone....................................31
Table 3: Description of study sites....................................34
Table 4: Data from the household questionnaires....................................40
Table 5: Tools and information collected from the FGDs....................................41
Table 6: Key informant guide questions....................................42
Table 7: Household characteristics for 358 households interviewed in Lupane and Gweru districts (2009 Survey)....................................49
Table 8: Domestic assets ownership among households (%) in Gweru and Lupane districts (2009 Survey)....................................53
Table 9: Farming implements ownership (% among households) in Gweru and Lupane districts (2009 Survey)....................................54
Table 10: Crops grown and land allocation for the 2008/09 season in Gweru and Lupane districts (2009 Survey)....................................57
Table 11: Income sources for households (%) in Gweru and Lupane Districts (2009 Survey)....................................58
Table 12: Maize yields (kg/ha) obtained by farmers in Gweru and Lupane districts for seasons 2004/05 - 2007/08 (2009 Survey)....................................61
Table 13: Sorghum yields (kg/ha) obtained by farmers in Gweru and Lupane districts for seasons 2004/05 – 2007/08 (2009 Survey)....................................61
Table 14: Farmers' perceptions of trends in weather patterns (2009 Survey)....................................64
Table 15: Mdubiwa ward men matrix ranking of constraints....................................65
Table 16: Mdubiwa ward women matrix ranking of constraints....................................65
Table 17: Adaptive strategies adopted by farmers in Gweru and Lupane districts (2009 Survey)....................................66
Table 18: Coping strategies adopted by farmers in Gweru and Lupane (2009 Survey)..........67
Table 19: Organizations working with farmers in Gweru and their role in addressing climate change impacts (2009 Survey)....................................79
Table 20: Determinants of cereal production for households in Gweru and Lupane (2009 Survey)....................................86
Table 21: Classification of households in Gweru and Lupane districts based on vulnerability status, 2009....................................92

List of Figures

Figure 1: Deviation of global annual atmospheric temperature from the global average temperature. 9
Figure 2: Bulawayo's annual average air temperature between 1951-2001 12
Figure 3: Bulawayo's annual average rainfall for the years 1951-2001 12
Figure 4: Map of Zimbabwe showing Natural Regions and locations of the study sites 32
Figure 5: Annual rainfall for Lupane district in the period 1970-2008 46
Figure 6: Deviation From Annual Average Maximum Temperature for Lupane distrcit in the period 1970-1997 48
Figure 7: Use of scotch carts to transport people and goods in Lupane (2009 Survey) 54
Figure 8: Livestock ownership among households in Gweru and Lupane districts (2009 Survey) 55
Figure 9: Communal grazing and quality of rangelands in Gweru and Lupane district (2009 Survey) 56
Figure 10: Shagari dam wall destroyed by excessive rains (2009 Survey) 59
Figure 11: Proportion of farmers having adequate food in each month (2009 Survey) 62
Figure 12: Rainfall forecast for January-March 2008: as received by Meteorology Lupane Officer 72
Figure 13: World Vision funded community nutrition garden in Lupane, Daluka ward along the Bubi Valley (2009 Survey) 75
Figure 14: A poster at Lower Gweru clinic aimed at raising awareness about child abuse (2009 Survey) 77
Figure 15: Distribution of household and their vulnerability to food insecurity for Gweru and Lupane (2009) 91

List of Acronyms and Abbreviations

AGRITEX	Agriculture Extension Services
BACCC	Building Adaptive Capacity to Climate Change
CCAA	Climate Change Adaptation in Africa
ENSO	El Nino Southern Oscillation
FAO	Food and Agriculture Organization of the United Nations
FGD	Focus Group discussions
GHGs	Green House Gases
GIS	Geographic Information System
GSDRC	Governance and Social Development Resource Centre
ICRISAT	International Crop Research Institute for the Semi Arid Tropics
IDRC	International Development Research Centre
IFAD	International Fund for Agricultural Development
IFPRI	International Food Policy Research Institute
IPCC	Intergovernmental Panel on Climate Change
IUCC	Information Unit for Climate Change
KII	Key Informant Interviews
MSU	Midlands State University
NGO	Non-Governmental Organization
NOAA	National Oceanic and Atmospheric Administration
NR	Natural Region
SARPN	Southern Africa Regional Poverty Network
SSA	Sub Saharan Africa
SPSS	Statistical Package for Social Sciences
UNDP	United Nations Development Project
UNEP	United Nations Environment Program
UNFCCC	United Nations Framework Convention on Climate Change
WFP	World Food Program
WMO	World Meteorology Organization

CHAPTER 1

INTRODUCTION

1.1. Background

Over the past two decades, evidence has mounted showing that the global climate is changing and that anthropogenic greenhouse gases are largely to blame (Maarten *et al.*, 2007). The unimpeded growth of greenhouse gas emissions leading to the raising in the earth's temperature, combined with growth in the world's population, threatens food and livelihood security for large numbers of people especially in developing countries.

Africa is considered very vulnerable to climate change because of widespread poverty (Eriksen *et al.,* 2008). Factors such as; large numbers of populations situated and trying to make a living in marginal areas and lack of technology to facilitate coping and adaptation, have also contributed to high levels of vulnerability, to climate change. South East Asia and Latin America also have large numbers of poor people, who are vulnerable to the impacts of climate change. Over a billion people around the world, two thirds of them women live in extreme poverty on less than 1US$/day (Eriksen *et al.,* 2008). Climate change will compound the existing poverty through reduced food availability, increased water scarcity, financial insecurity and incidence of illness. Projections of climate change suggest that developing nations will be affected the most because of their geographical and climatic conditions, their high dependence on agriculture and natural resources driven activities, and limited capacity to adapt to the changing climate. The capacity to adapt is undermined by the limited availability of social, economic, political and technical resources available to these countries and communities. Within countries the poorest, have the least resources, the least capacity to mitigate the negative impacts of climate change and are thus the most vulnerable (Eriksen *et*

al., 2008). Climate change is thus a serious threat to poverty eradication and the attainment of the Millennium Development Goals, including the goal of halving extreme poverty by 2015, and sustaining progress beyond 2015.

Most countries in Sub-Saharan Africa (SSA) rely heavily on agriculture for employment and food security for their economies. The sector also has large numbers of smallholder farmers, most of who produce under unfavorable conditions characterized by low and erratic rainfall and poor soils. There is need to better understand the nature and magnitude of the impacts of climate change on agriculture in general, and the smallholder sub-sector in particular, in order to help in the identification and development of practical means for enabling communities to reduce vulnerability and to mitigate negative impacts of climate change.

While much research on the impacts of climate change has tended to focus on impacts on a given region or country, less effort has been directed at individual households in developing countries. The IPCC (2001) raises this concern and points out that climate change research has largely focused on predicting impacts on agriculture and other economic activities, with less effort being focused on understanding vulnerability to adverse effects of climate change of individuals and households. The same report also indicates that there is now increased confidence in predictions of climate change at global level, but there is still great uncertainty at regional and local levels, where information is required by farmers to minimize vulnerability to climate change. There is thus need to ascertain how vulnerable farmers are, at the individual household level, and also to understand factors influencing vulnerability to climate change. As such, this study seeks to contribute to the body of research on climate change by investigating the vulnerability of smallholder farmers in a developing country to climatic changes, focusing on the case of Zimbabwe.

For most parts of Zimbabwe especially the low lying areas, global climate change is projected to lead to an even drier local climate, with high incidence of droughts and erratic rainfall distribution, which will affect agriculture yields and thus creating conditions that undermine economic development (Information Unit for Climate Change (IUCC), 1994). The adverse changes in climatic conditions are likely to influence the country's economy which is largely agriculture-dependent. With about 70% of Zimbabweans living and deriving the bulk of their food requirements and income from farming in the rural areas, a fall in agriculture production will have serious consequences for many people (Levina *et al*, 2006).

This study assesses the vulnerability of smallholder farmers in two districts of Zimbabwe, namely Gweru (Midlands Province) and Lupane (Matabeleland North Province) by assessing the likelihood of individual households being food insecure. The two districts are located in the country's agro-ecological Natural Region IV[1] where agriculture is severely limited by low and highly variable rainfall and poor soils. Results from climate studies suggests that negative impacts of climate change will be severe in areas that are already arid, thus the basis for selection of these two study sites. Although conditions are marginal for crop production, smallholder farmers in these areas still engage in rain fed agriculture as their main source of food and income. Climatic changes are expected to result in reduced yields and increased variability in production for these smallholder farmers.

The study examines the vulnerability of the households to climate change by examining the pre-existing food security status of households, and how climate change will exacerbate the food insecurity situation of these households by reducing agricultural production levels of

[1] Zimbabwe is divided into five agro-ecological regions (I to V) on the basis of rainfall and vegetation. Natural region I is the wettest and V is the driest. A detailed discussion of Zimbabwe's natural regions will be presented in chapter 3. Source: Ministry of Agriculture Annual report 2000 and Vincent and Thomas, 1960.

households and the ability to meet their food requirements. Cereals, namely maize, sorghum and millet constitute an important part of the household's diet in Gweru and Lupane districts and are widely grown in these areas. The likelihood of a household's own cereal production to fall short of the household's requirements is calculated to determine vulnerability to food insecurity. It is argued that pre-existing conditions (for example a household's current food security status) influence the magnitude of and the ability of communities to cope with climate change impacts.

There are two notions of vulnerability in climate change research and these include vulnerability as an end point (level of damage after the event has unfolded) and vulnerability as a starting point which focuses on the susceptibility of the household[2] (Füssel., 2007). This study takes on the starting point interpretation, which takes the root problem as social vulnerability and examines the current vulnerability of the households as a measure of vulnerability to climate change. Households that are currently vulnerable to food insecurity will find it difficult to cope with adverse impacts of changes in climatic conditions. Thus measuring the likelihood of being food insecure provides a way to examine vulnerability to climate change.

1.2. Problem Statement

Zimbabwe is located in the semi-arid tropic that is characterized by low and highly variable or erratic rainfall, limiting potential crop yields (Graef and Haigis, 2001). Projections of climate change indicate that the semi-arid tropics will become drier, with increased temperatures and increased frequency of droughts. The current climate situation in most parts of Zimbabwe already shows these projected climate impacts which can be best described by

[2] The two interpretations of vulnerability will be discussed in detail in chapter two.

erratic, unreliable and insufficient rainfall with only 3 percent of the country receiving adequate rainfall for agriculture. Long term data show that a majority of Zimbabwe's wet seasons are often punctuated by mid season droughts which affect crops resulting in poor harvests. Zimbabwe's economy is agro-based mainly depending on rain-fed agriculture and any development is pinned to a successful rainfall season. In addition, Zimbabwe has limited capacity to adapt and cope with negative impacts of climate change due to low income, lack of technologies, poor infrastructure and weak institutions. This also includes unclear policies, lack of adaptive strategies, weak legislation and service providers and to some extent political interference in the country. The study seeks to assess vulnerability to climate change by linking climate change and vulnerability to food insecurity for farmers in Gweru and Lupane districts in Zimbabwe.

1.3. Objectives

The primary objective of this study is to examine vulnerability to climate change of smallholder farmers in Gweru and Lupane districts of Zimbabwe. Specifically the study

a) Characterization of the exposures facing households in the study area
b) Measuring the cereal availability and requirements of the household
c) Assessing the likelihood of households experiencing cereal deficit or food insecurity

1.4. Research Questions

The study is guided by the main research question: 'To what extend or level are smallholder farmers vulnerable to climate change?' This question is further guided by the following sub questions:

a) How will climate change affect smallholder farmers in Gweru and Lupane districts of Zimbabwe?

b) How vulnerable are smallholder farmers to food insecurity?

1.5. Justification of study

Agriculture has historically been the mainstay of Zimbabwe's economy, with about 70% of Zimbabwe's largely rural population deriving its livelihood from agriculture (Levina *et al*, 2006). The majority of the farming communities are smallholder farmers depending on rain fed agriculture, residing in marginal agro-ecological areas, where rainfall is low and unpredictable with lack of technology such as irrigation to curb against these climatic effects. Drought is predominant in Southern Africa with Zimbabwe farmers experiencing drought once every two or three years. Conditions are likely to worsen as a result of changes on local climate. Climate prediction models suggest that Zimbabwe will experience a reduction in rainfall, increased frequency of droughts and an increase in the atmospheric temperatures (Gumbo, 2006). The rainfall season has uncharacteristically started late and farmers are increasingly wary of establishing new optimal times for planting their crops, negatively affecting farm production (Chigwada, 2005). This has negative implications on smallholder farmers' livelihoods thus increasing their vulnerability to climate change and this study seeks to review whether this is the case.

Information generated by this study will also contribute to improve understanding of impacts of climate change in smallholder agriculture and on the welfare of agriculture dependent communities, factors influencing the households' vulnerability and ability to cope with negative impacts of climate change. Information can provide a basis for formulation of

effective and specific strategies to help households curb effects of climate change, cope and adapt to climate change.

1.6. Outline of thesis

The thesis consists of six chapters, including this introductory chapter. The first chapter provides background information on climate change and the significance of the phenomenon for socio-economic development and wellbeing. The chapter also provides a statement of the research problem, the main objective and sub objectives. A review of literature in chapter 2 gives an overview of what is known to date, the causes and impacts of climate change, vulnerability applied to climate change, empirical literature on impacts of, and vulnerability to climate change. The chapter also provides the analytical framework used to guide the assessment of vulnerability to climate change among smallholder farmers. Chapter 3 describes the procedure for selecting study sites, the methodology for data gathering and analysis. Chapter 4 provides a description of climatic conditions in the study sites, characterization of the households and communities, production patterns and other livelihood activities. The chapter also describes farmers' experiences with climatic changes, characterizes exposures facing communities, activities of organizations working in the communities and their effect on vulnerability of households. This is followed by chapter 5, which presents results of analyses of vulnerability to climate change. The final chapter provides a summary, conclusion, limitations of the study and recommendations for further research.

CHAPTER 2

LITERATURE REVIEW

2.1. Introduction

Climate change is now generally accepted to be a major global problem. With reference to smallholder farmers, questions that arise are; what do smallholder farmers understand about climate change, how will climate change affect their livelihoods and how vulnerable are they to the negative impacts of climate change?

To answer these questions this chapter reviews literature on climate change and vulnerability. It begins by looking at global climate change in general and its impacts on the ecosystem and socio-economic system. Literature on climate change impacts on smallholder farmers in developing countries including Zimbabwe is also reviewed. Lastly the chapter looks at vulnerability, how it is conceptualized in this study and how it is measured for smallholder farmers in Gweru and Lupane districts of Zimbabwe.

2.2. Global climate change

Historically the earth's climate has always had cyclical trends and variations through the centuries, although with constant averages (IPCC 2001). Current climatic trends show a deviation from historic trends. The rate of change and the cause of change have been of concern to scientists all over the world. Temperature records collected for over a period of 100 years shows that, the Earth's surface temperature has risen by more than 0.7 degrees Celsius since the 1800s (IPCC 2007). Historical temperature data from Figure 1 shows

deviation of global atmospheric temperature measures from the global average temperature, from 1850 to 2008 and the gradual increase in the temperature. The temperature anomaly refers to the difference from an average and this measure gives a more accurate picture of temperature change. The graph also shows that over the past three decades, the global air temperatures anomaly has since increased, thus showing a general warming of the atmosphere.

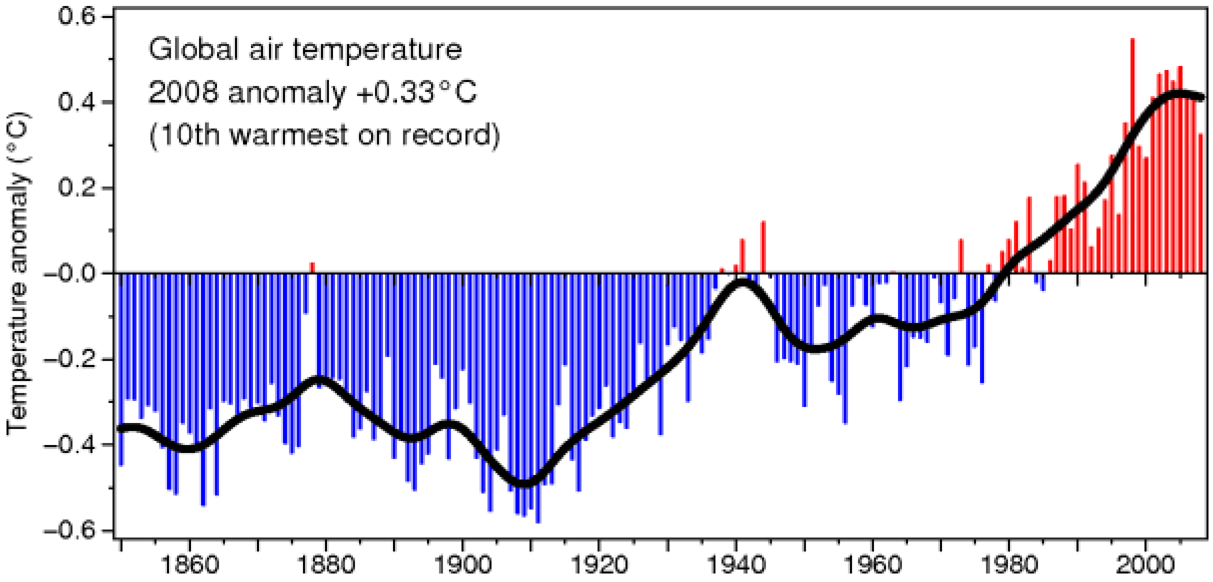

Figure 1: Deviation of global annual atmospheric temperature from the global average temperature.
Source: Brohan *et al,* 2006

The warming of the atmosphere is also supported by the IPCC report (2001a) which reports that, globally, the 1990s were likely the warmest decades over the past millennium and the graph shows that the warming has gone beyond the 1990s to 2000s. Records from WMO (2004) report that; nine out of ten warmest years on record occurred between 1995 and 2004, with 1998, 2002, 2003 and 2004 being the warmest.

Accumulated evidence suggests that anthropogenic activities[3] are to blame for the increasing concentrations of green house gases (GHGs) in the earth's atmosphere and the consequent warming of the planet. The GHGs trap the heat in the atmosphere by preventing some of the radiation from escaping into space. While the greenhouse effect is important for life on earth, as they help trap some of the sun's radiation from reflecting back into space to keep the earth habitable for living creatures, the increasing quantities of greenhouse gases are now of concern because they cause increased global warming and dramatic climate change (IPCC 2001).

The main greenhouse gas, carbon dioxide is emitted when fossil fuels, like coal and oil are burned. Since the industrial revolution in the 1800s, use of fossil fuels has increased at a rapid rate leading to increased emissions and buildup of GHGs in the atmosphere (IPCC 2007). GHGs are also released when ecosystems are altered and vegetation is either burned or removed, the carbon stored in them is released into the atmosphere as carbon dioxide. The clearing of land through cutting down trees and woodland burning often leads to deforestation. Reasons for deforestation include urban growth, where land is cleared to build houses and factories; harvesting timber for fuel, construction and paper making; and agriculture activities. Agriculture involves land tilling which also often releases gases into the atmosphere. Moreover with the growing population more land is cleared for agriculture thus there is expansion of cultivated land to meet the high food requirements. Currently up to a quarter of the carbon dioxide emissions can be attributed to land use activities (National Oceanic and Atmospheric Administration: NOAA, 2005).

[3] Some scientists argue that the increase in Green House Gases (GHGs) is due to natural processes and not anthropogenic activities (IPCC 2001).

Effects of global warming include the melting of the polar ice caps leading to a rise in sea levels and flooding in some regions especially areas near the coast. In regions where high temperatures have been the generally norm, like the semi arid tropics, occurrence of droughts and dry spells will increase (Eriksen *et al*., 2008).

2.3. Zimbabwe and global climate change

Climatic records show that the country is warmer at the end of the twentieth century, compared to historical recoded years and according to Hulmen *et al* (1999) the warming has been the greatest during the dry season, with the 1990s decade being one of the warmest in the century. The 1990s witnessed probably one of the driest periods, a drought certainly related to the prolonged El Nino Southern Oscillation (ENSO) conditions that prevailed during these years in the Pacific Ocean (Hulmen *et al.*, 1999). The ENSO also known as the El Nino in short, is one of the main causes of climate variability for many tropical regions including Zimbabwe. The El Nino phenomenon is a recurring pattern of inter-annual oscillations in both sea surface temperature and sea level atmospheric pressure in the tropical Pacific which strongly correlates with climate patterns around the globe. For Southern Africa, Zimbabwe included, rainfall is strongly influenced by ENSO and scientists use its occurrence to predict rainfall to be received in the country (Hulmen *et al.*, 1999).

Figure 2 and 3 shows temperature and rainfall records for the past 50 years for the city of Bulawayo in Zimbabwe. In figure 2, it appears there is an increase in the temperature experienced in the city over the years.

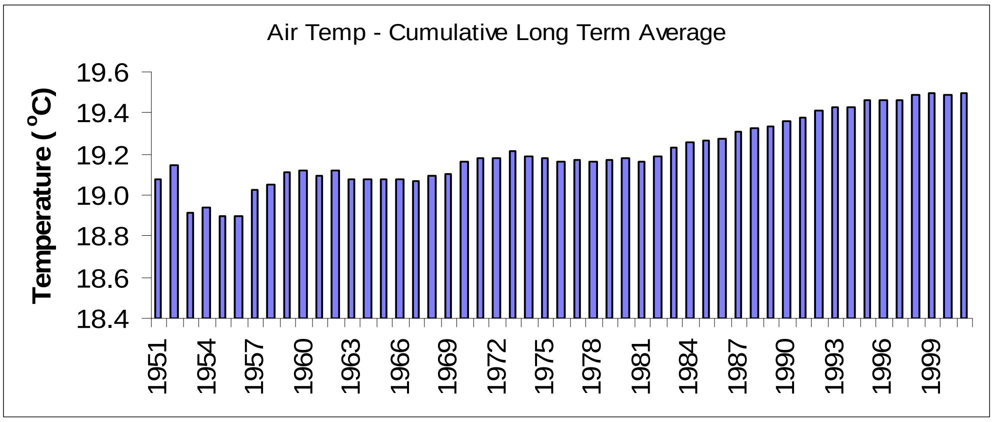

Figure 2: Bulawayo's annual average air temperature between 1951-2001
Source: Dimes *et al.*, 2008

In Figure 3, the annual average rainfall received over a period of about 50 years is displayed. There is no noticeable trend in the rainfall pattern for the city in this case.

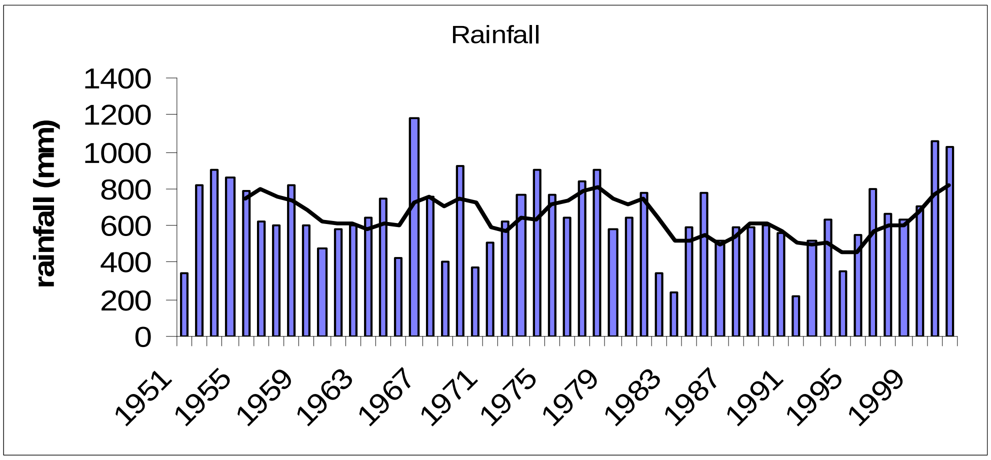

Figure 3: Bulawayo's annual average rainfall for the years 1951-2001
Source: Dimes *et al.,* 2008

In Zimbabwe, research on climate change and its impacts is in its preliminary stages. However the subject is of great importance considering that the majority of the people are dependent on agriculture for their livelihood and that agriculture is heavily dependent on the climate experienced. Rainfall is a critical natural resource for crop and livestock production, much so in the semi-arid areas where annual rainfall is less than 600mm. Droughts in most parts of the country especially the low lying areas have become recurrent over the last two

decades and farmers have been experiencing droughts every two to three years (Mazvimavi *et al*., 2007), with crop failure occurrences in 3 out of every 5 years in the semi-arid areas of the country (Mugabe *et al.*, 2003). Poor rainfall distribution within the growing season is often a cause for crop failure even for years with close to average rainfall, due to dry spells at critical stages of crop growth. In some areas there is insufficient surface or groundwater to irrigate dry land crops even at critical periods (Lovell, 2000).

Studies that have been carried out in Zimbabwe on climate change have documented examples that have been linked to the impacts of climate change. These likely impacts of climate change on Zimbabwe are exemplified in by the 1991/92, 2000/01 and 2007/08 droughts. The catastrophic drought of 1991/92 offers valuable insights into Zimbabwe's vulnerabilities to climatic variability**.** During the drought, which was linked to an El Nino Southern Oscillation (ENSO) event, Zimbabwe's temperatures reached maximums of about 47 degrees Celsius, recorded along the South Africa and Zimbabwe boarder (IPCC, 2001). Rainfall levels fell to just 40% of the long term average, the water table dropped by 100-200m, ground water (including traditional shallow wells and boreholes) dried up and the water table for rivers and lakes was lowered (IUCC, 1994). Crop production levels fell, and a food deficit of 1.5 million metric tons of maize resulted from the insufficient rainfall and poor growing season. In addition to highlighting the vulnerabilities of Zimbabwe's various economic sectors, the drought created widespread awareness among policy-makers and the general public of the need to address the country's dependence on climatic conditions (IUCC, 1994). The other drought was experienced during the 2001/02 season; and during this period crops failed across most parts of the country. The third recorded drought was in 2007 and the government declared 2007 as a drought year and invited experts from World Food Program

(WFP) and Food and Agriculture Organization of the United Nations (FAO) to assess the country's food situation (Cottem, 2007).

Studies have also been carried to investigate how climate change will affect human health. In Zimbabwe, investigations into possible implications of climate change on human health have been limited. Reviews conducted reveal the complex nature of the problem, where demographic changes, increase of malaria incidences, water related issues as well as changes in heat stress associated with temperature increases have been observed. Incidences of malaria usually reach a peak during the rainy season when temperatures are high and bodies of stagnant water are abundant. It is estimated that about one in every three people in Zimbabwe live in malaria risk areas (Hartman *et al*, 2002), but this will likely change under a climate change scenario with more areas becoming prone to the disease.

Land use changes such as deforestation and bush fires in Zimbabwe contribute to the increase in green house gas emissions. The main reasons for deforestation and bush fires in the country are land clearing for agriculture purposes (IUCC, 1994). The major sources of emissions for Zimbabwe includes burning of fossil fuels, namely coal to generate electricity at Hwange power station located in the western part of the country (UNEP, 1997), and vehicle emissions. While Zimbabwe's contribution to global emissions of greenhouse gases is very small, the potential impacts of climate change on the country are likely to be high. It is rather ironic to note that developing countries like Zimbabwe that have contributed the least to rising green house gases will suffer the greatest and are considered the most vulnerable to climate change.

2.4. Vulnerability to climate change

Vulnerability refers to the manner and degree to which a system is susceptible to conditions that negatively affect the well-being of the system. In the climate change field, the IPCC Third Assessment Report defines vulnerability as "the degree to which a system is susceptible to, or unable to cope with, adverse effects of climate change, including climate variability and extremes" (McCarthy *et al*., 2001).

Vulnerability to climate change varies greatly among regions, sectors and social groups and communities. Vulnerability is influenced by a variety of social factors such as provision of services and access to alternative livelihoods. Climate change research shows that the poorest are more likely to be the most vulnerable and negatively affected the most. This is supported by Eriksen *et al*., (2008), whose study finds that some of the factors that generate vulnerability to climate change are closely associated to poverty. Poor people are often the ones to suffer injury, loss, death or harm from droughts, floods and other extreme events. They have less capacity to recover after such events due to lack of assets to engage in alternative economic activities or help arrest decline in the availability of resources. Downing (2003) also finds that more than a decade of research on vulnerability to climate change shows that inevitably it is the marginalized who suffer the impacts of changing environment conditions.

Furthermore, in the literature on rural livelihoods, it is widely accepted that seasonal climate variations (including periodicity and amount of rainfall) is one of the major sources of vulnerability faced by farming households (Ellis, 2000). Economic assets, capital resources, financial means, wealth, or poverty; the economic condition of nations and technological advancement of groups clearly is a determinant of reducing vulnerability to climate change

(Kates, 2000). Better resourced nations are better prepared to bear the costs of adaptation to climate change impacts and risks than poorer nations (Burton, 1996). It is also recognized that poverty is directly related to vulnerability (Fankhauser *et al*., 1997). Adger *et al*, (1999) also mentions that attributes that can increase or decrease a system's vulnerability include marginalization, inequity, presence and strength of institutions, food and resource entitlements, economics and politics.

The majority of Southern Africa's smallholder farmers is engaged in low input farming and has lack of technology to adapt. Zimbabwe is not spared from this, with smallholder farmers in the country being poorly resource endowed. Availability and access of technologies, institutional capacity and wealth are important factors needed to reduce a household's vulnerability to the changing climate. Possible responses for smallholder farmers to mitigate climate change include use of improved seed varieties, use of drought tolerant crops and diversifying out of agriculture (Deressa *et al*., 2008). In Zimbabwe, the political and economic instability over the past decade worsened the situation for the smallholder farmers. Inputs such as improved seed varieties and fertilizers were not found on the local markets. Agriculture extension services and local service providers became less effective due to the high turnover rate, as skilled labor left the country due to low remuneration. This affected the rural smallholder farmers because agriculture extension services were not effective.

Reviews of the interpretation of vulnerability in climate change research have generally identified two different vulnerability concepts. O'Brien *et al* (2004a) distinguishes between an 'end point' and 'starting point' interpretation of vulnerability. The two roles of vulnerability research underlying these interpretations of vulnerability largely correspond with the two types of adaptation research distinguished by Smith *et al* (1996) and by Burton

et al (2002). The differences between these two interpretations of vulnerability are summarized in Table 1.

Vulnerability according to the end point interpretation represent the expected net impacts of a given level of global climate change, taking into account feasible adaptations. Vulnerability according to the starting point interpretation focuses on reducing internal socioeconomic vulnerability to any climatic hazard. This study takes on the starting point interpretation. This approach focuses on identifying and addressing characteristics that make a system vulnerable, with the aim of reducing vulnerability to climate change (Füssel and Klein, 2006). This approach also considers external climate conditions while assessing vulnerability within a particular social unit in order to determine who is vulnerable and why (O'Brien *et al* 2004a).

Table 1: Interpretations of vulnerability in climate change research

**Attributes of vulnerability investigated**	**End point interpretation**	**Starting point interpretation**
Root problem	Climate change	Social vulnerability
Policy context	Climate change mitigation, comprehension, technical adaptation	Social adaption, sustainable development
Illustrative policy question	What are the benefits of climate change mitigation	How can vulnerability of societies to climatic hazards be reduced?
Illustrative research question	What are the expected net impacts of climate change in different regions?	Why are some groups more affected by climatic hazards more than others?
Vulnerability and adaptive capacity	Adaptive capacity determines vulnerability	Vulnerability determines adaptive capacity
Reference for adaptive capacity	Adaptation for future climate change	Adaptation to current climate change
Starting point analysis	Scenarios of future climate hazards	Current vulnerability to climatic stimuli
Analytical function	Descriptive, positivist	Explanatory, normative
Main discipline	Natural sciences	Social sciences
Meaning of vulnerability	Expected net damage for a given level of global climate change	Susceptibility to climate change and variability as determined by socioeconomic factors
Qualification of terminology	Dynamic cross scale integrated vulnerability (for a particular system) to global climate change	Current internal socioeconomic vulnerability (of a particular social unit) to all climatic stressors
Reference	McCarthy *et al* (2001)	Adger (1999)

Source: Based on O'Brien *et al*., (2004); Smit *et al*., (1999); Burton *et al*., (2002); Füssel and Klein, (2006).

2.5. Measuring smallholder vulnerability to climate change

Vulnerability can be thought of in terms of an outcome of a process of household response to risks (Jamal, 2009). The risk-response-outcome framework may be applied to food security (probability of not meeting food needs), environment (survival loss), disaster management (welfare loss) and impact of climate change on welfare of smallholder farmers. Vulnerability is thus the welfare loss from the realization of an undesirable state of nature, thus if climate change occurs and it leads to floods, droughts, dry spells; how much would be the reduction in welfare (below a socially acceptable level) for particular households. The general welfare can be consumption level, utility, poverty; the study will look at the production levels as the indicator of households' welfare.

Scholars from different disciplines conceptualize vulnerability differently based on objectives and methodologies employed. Literature on the conceptual and methodological approaches to vulnerability analysis is summarized in Adger (1999), Füssel and Klein (2006), and Füssel (2007). Thus this section reviews literature on econometric methodologies used to assess vulnerability.

The econometric approach to measuring vulnerability has most of its roots in poverty and development literature. The methodology uses household-level socioeconomic survey as data to analyze the level of vulnerability of different social groups. There are three different methodologies used to assess vulnerability, these include vulnerability as uninsured exposure to risk (VER), vulnerability as low expected utility (VEU) and vulnerability as expected poverty (VEP) (Hoddinot and Quisumbing, 2003). All three methods construct a measure of welfare loss attributed to shocks.

2.5.1. Vulnerability as Uninsured Exposure to Risk

This method is based on *ex post facto* assessment of the extent to which a negative shock causes welfare loss (Hoddinot and Quisumbing, 2003) the impact of shocks is assessed using panel data to quantify the change in induced consumption. Skoufias (2003) employed this approach to analyze the impact of shocks on Russia. In the absence of risk management tools, shocks impose welfare loss that is materialized through reduction in consumption. The amount of loss incurred due to shocks equals the amount paid as insurance to keep a household as well off before any shocks occurred. The limitation of this method is that in the absence of panel data, estimates of impacts, especially from cross sectional data are often biased and thus inconclusive (Skoufias, 2003).

2.5.2. Vulnerability as a Low Expected Utility

Ligon and Schechter (2003) defined vulnerability as the difference between utility derived from some level of certainty-equivalent consumption at and above, which the household would not be considered vulnerable, and the expected utility of consumption. The method was applied to a panel data set from Bulgaria in 1994. The results showed that poverty and risk play roughly equal roles in reducing welfare. The limitation of this method is that it is difficult to account for an individual's risk preference given that individuals are often ill informed about their preference, especially those in uncertain events (Kanbur, 1987).

2.5.3. Vulnerability as expected poverty

In this framework, a person's vulnerability is conceived as the prospect of that person becoming poor in the future if currently not poor or the prospect of that person continuing to be poor if currently poor (Christiaensen and Subbarao, 2004). It is argued that pre-existing

conditions and forces influences the magnitude and the ability of communities to reduce vulnerability to climate change impacts. Thus vulnerability is seen as expected poverty, with consumption or income being used as the welfare indicator. In this conception, the vulnerability is measured by estimating the probability that a given shock, or set of shocks, moves consumption of an individual/household below a given minimum level (for example a consumption poverty line) or forces the consumption level to stay below the given minimum requirement if it is already below that level (Chaudhuri, Jalan and Suryahadi, 2002). In this case vulnerability can be measured using the cross sectional data unlike the other methods (section 2.5.1. and 2.5.2) that require panel data.

Ninno *et al* (2006) used data from the Household Income and Expenditure Survey (HIES) in Pakistan to measure vulnerability of individual households using this conception of vulnerability. The authors found out that a third of the population was vulnerable due to low level of resources. They also discovered that 24 to 34 percent of population's vulnerability comes from high volatility of consumption. In another study by Chaudhuri, Jalan and Suryahadi (2002), results showed that although only 22 percent of the population in Indonesia was poor, as much as 45 percent of that population was vulnerable to poverty. The limitation is that if estimates are made using single cross sectional data, one must make a strong assumption that cross sectional data captures temporal variability.

Although this study measures vulnerability to poverty, it was adopted for this study to measure vulnerability to food insecurity. For the case of smallholder farmers in Zimbabwe, the food security status of a household defines the welfare status of that household. Smallholder farmers depend mainly on rain fed agriculture for production and other resource based activities, therefore adverse climatic changes will affect productivity/income earning

potential, health, social disruptions and in turn affect the overall wellbeing of the households. Among other things, the vulnerability status of smallholder farmers in different locations will be influenced by the household's ability to produce enough to ensure the household's food security. The study seeks to investigate how vulnerable smallholder farmers are to climate change, looking specifically at the food security status of the household. Food insecurity increases the chances of being negatively affected by climate change and a household that is food insecure has greater chances of being negatively impacted by climate change.

2.6. Analytical framework [4]

In Chaudhuri's study, vulnerability is thought of as the prospect of a household becoming poor in the future, if currently poor. This study adopts this model, by taking poverty as the household being food insecure. Thus food insecurity is used as the measure of welfare for this particular study. In addition the other methods of measuring vulnerability use longitudinal data (section 2.5.1. and 2.5.2) and this model uses cross sectional data, thus was suitable for the purposes of this particular study. This approach is divided into three basic steps, i.e. identifying the welfare indicator; identifying the vulnerability threshold; and measuring vulnerability.

Chaudhuri uses consumption measures a welfare indicator because he argues that it provides a more adequate picture of wellbeing especially in low or medium income countries. The other advantage is that consumption is more accurately measured. This study uses the household's cereal production levels as a measure of welfare. Farmers in both Gweru and Lupane mainly depend on what they produce for household food security, thus what the households produce is equated to consumptions levels for the household, in this study.

[4] This section draws extensively on the work of Chaudhuri (2002).

Smallholder farmers in Zimbabwe commonly produce cereals such as maize, millet and sorghum; with maize being the staple food and most commonly grown cereal. The energy content of the three cereals is almost the same, with maize, millet and sorghum producing 358, 329 and 336 kilocalories per 100g of grain respectively (Leder, 2010). In this study maize, sorghum and millet produced by the household is added so as to determine how much per capita cereal is produced by the household. However smallholder farmers historically have cultivated the largest area of maize and a study done by Eicher *et al* (1997) shows that from 1965 to 1994, the area planted to maize by smallholder farmers accounted to 70% of the national maize area, even in the current years maize still accounts for the bulk of area planted to cereal crops.

A household with adequate cereal stock to meet the household's energy needs is generally considered less likely to experience food insecurity or fall into poverty. According to the FAO (2007) annual Zimbabwe reports, a family of six people needs about 165kg per capita of cereal per annum. In addition the Southern Africa Regional Poverty Network's (2003) report on the regional overview of the southern African food security crisis suggests that an average family of 6 people requires about 800 -1000kg annually of cereal to be food secure, which also suggests a per capita cereal requirement of approximately 165kg.

The choice of the vulnerability threshold involves generating a sample that is classified into two groups, that is those that are vulnerable and those that are not vulnerable to food insecurity. It entails establishing a vulnerability threshold, such that a household is said to be vulnerable if its vulnerability probability is greater or equal to v, i.e. $v_h \geq v$. Chaudhuri *et al.* (2002) says the choice of vulnerability threshold is quite arbitrary. A common choice in literature is a threshold vulnerability probability of 0.5.

Thus a household is considered vulnerable food insecurity if the probability is equal or greater than 0.5 and less likely to be vulnerable to food insecurity if the probability is less than 0.5.

The approach developed by Chaudhuri *et al* (2002) being used for this study identified the vulnerability level at a given time as the probability that a household will find itself poor at the next time period, and estimates this probability. Their study measured the likelihood of falling into poverty and this idea was adopted for this study, taking poverty as food insecurity.

The vulnerability level of a household h at time t is defined as the probability that a household will find itself consumption poor, that is the cereal production levels will not be adequate to meet the households' requirements at time $t+1$, this is a basic formulation of vulnerability as the risk of poverty is expressed as:

$$V_{ht} = \Pr\left(c_{h,t+1} \leq z\right) = \int_{-\infty}^{z} f\left(c_{h,t+1}\right)\partial c \qquad \textbf{(1)}$$

Where $c_{h,t+1}$, is the household's consumption at time $t+1$ and z is the appropriate consumption for the household. One of the limitations of this definition is that it is sensitive to the choice of z. Vulnerability is thus the *ex-ante* risk that a household will not be able to cope or adapt to an external pressure (in this case being climate change). To assess a household's vulnerability to climate change, we need to make inferences about its future consumption levels. In order to do that we need a framework for thinking explicitly about both the inter-temporal aspects and cross-sectional determinants of the consumption pattern at the household level (Chaudhuri *et al* 2002).

As discussed earlier, the food security status and consumption is dependent on the household's own cereal production levels. Thus production is influenced by a number of factors. Among them is labor availability, access to extension services, the education status of the household head, availability of production assets, among others. This suggests the following reduced form expression for production:

$$c_{ht} = C(X_{h},) \qquad \textbf{(2)}$$

Where X_h represent a bundle of observable household characteristics that have been mentioned in the paragraph above. The observable household characteristics include; labor availability, access to extension, the education status of the household head, the age of the household head etc. Substituting (2) into (1) we can rewrite the expression for vulnerability level as:

$$V_{ht} = \Pr\left(c(X_h) \leq z \middle| X_{h,}\right) \qquad \textbf{(3)}$$

The expression in equation (3) suggests that a household's vulnerability level is derived from the household observable characteristics and this is compared to the standard consumption requirements (z) given the same household observable characteristics (Chaudhuri *et al* 2002).

Based on limitations imposed by the use of cross-sectional data in capturing temporal variability, and the consequent need to make some assumptions regarding the stochastic process generating the consumption levels of a household h, we specify equation (3) as:

$$\ln c_h = X_h\beta + \varepsilon_h \qquad \textbf{(4)}$$

Where:

c_h is per capita consumption of the household,

X_h represents a bundle of observable household characteristics, including assets and other risk management instruments,

β is a vector of parameters to be estimated,

and ε_h is a mean-zero disturbance term that captures idiosyncratic factors (shocks) that contribute to different per-capita consumption levels for households that are otherwise observationally equivalent.

It should be noted from equation (4) that variance of the regression depends on the household characteristics. Thus:

$$\sigma^2_{\varepsilon,h} = X_h\theta \qquad \textbf{(5)}$$

β and θ (being vectors of parameters to be estimated) can be determined using a three-step feasible generalized least square (FGLS) procedure suggested by Amemiya (1977). Firstly equation (4) is estimated using an ordinary least square (OLS) procedure. The estimated residuals from equation (4) are used to estimate

$$\hat{\varepsilon}^2_{OLS,h} = X_h\theta + \eta_h \qquad \textbf{(6)}$$

Where η_h is an error term that captures shocks that contribute to the estimated residuals from equation 4.

The predictions from equation 6 are used to transform equation 4 as follows

$$\frac{\hat{\varepsilon}^2_{OLS,h}}{X_h\hat{\theta}_{OLS}} = \left(\frac{X_h}{X_h\hat{\theta}_{OLS}}\right)\theta + \frac{\eta_h}{X_h\hat{\theta}_{OLS}} \qquad \textbf{(7)}$$

This transformed equation is estimated using OLS to obtain an asymptotically efficient FGLS estimate, $\hat{\theta}_{FGLS}$. Note that $X_h\hat{\theta}_{FGLS}$ is a consistent estimate of $\sigma^2_{\varepsilon,h}$, the variance of the idiosyncratic component of household consumption. The estimates:

$$\hat{\sigma}_{\varepsilon,h} = \sqrt{X_h\hat{\theta}_{FGLS}} \qquad \textbf{(8)}$$

are then used to transform equation (4) as follows:

$$\frac{\ln c_h}{\hat{\sigma}_{\varepsilon,h}} = \left(\frac{X_h}{\hat{\sigma}_{\varepsilon,h}}\right)\beta + \frac{\varepsilon_h}{\hat{\sigma}_{\varepsilon,h}} \qquad \textbf{(9)}$$

OLS estimates of the equation (4) yields a consistent and asymptotically efficient estimate of β. The standard error of the estimated coefficient, $\hat{\beta}_{FGLS}$, can be obtained by dividing the standard error by the standard error of the regression.

Using the estimated $\hat{\beta}$ and $\hat{\theta}$ the estimate expected log cereal level is measured

$$\hat{E}[\ln c_h | X_h] = X_h\hat{\beta} \qquad \textbf{(10)}$$

and the variance of log cereal level

$$\hat{V}[\ln c_h | X_h] = \hat{\sigma}_{\varepsilon,h} = X_h\hat{\theta} \qquad \textbf{(11)}$$

for each household. By assuming that per capita consumption is log-normally distributed, these estimates are used to form an estimate of the probability that a household with characteristics X_h will be poor (Chaudhuri *et al.,* 2002) and in this case food insecure. Letting $\Phi(\bullet)$ denote the cumulative density of standard normal, this estimated probability will be given by

$$\hat{v}_h = \hat{\Pr}(\ln c_h < \ln z | X_h) = \Phi\left(\frac{\ln z - X_h\hat{\beta}}{\sqrt{X_h\hat{\theta}}}\right) \qquad \textbf{(12)}$$

This is an ex ante vulnerability measure that can be estimated by cross-sectional data. Equation (12) will provide the probability of a household at time *t* becoming food insecure at *t+1* given the distribution of production levels at *t*.

However, the measure correctly reflects a household's vulnerability only if the distribution of consumption across households, given the household characteristics at one time, represents the time-series variation of consumption of the household (Chaudhuri *et al.*, 2002).

These model estimates the probability of a household being vulnerable or not vulnerable to food insecurity. Food insecurity increases the chances of a household being negatively affected by climate change. Thus a household that has a probability of being vulnerable to food insecurity will have greater chances of being negatively impacted by climate change.

2.7. Summary

Climate change is a global issue that is affecting most economies around the globe. The poor and developing countries are said to be more vulnerable to climate change considering the limited technologies and resources at their disposal to mitigate the effects. Zimbabwe is a poor country and this study seeks to assess the degree of vulnerability for smallholder farmers to food insecurity as a result of climatic changes and variability. Zimbabwe's rural population mainly depends on agriculture for their livelihoods and this is a climate sensitive enterprise. Chaudhuri's (2002) approach to measuring vulnerability to poverty was adopted for this study to assess the vulnerability of households to food insecurity. Climate change is going to have a negative impact on food production, causing many houses to become food insecure. Households that already experience food shortages or those that are marginally food secure will likely be the first and worst affected by decreases in food production due to climate change.

CHAPTER 3

DESCRIPTION OF STUDY SITES AND RESEARCH METHODOLOGY

3.1. Introduction

This chapter provides background information for the study, highlighting the origins of this study and its contribution to understanding the effects of climate change on Zimbabwe's agriculture sector and particularly for smallholder farmers. A description of the procedures that were followed during the selection of the areas of study, sampling, questionnaire design and administration are also contained in this chapter. The chapter concludes by outlining how the data was captured and analyzed.

3.2. Background information

The study reported in this thesis is part of a project funded by the International Development Research Centre (IDRC) of Canada, titled: Building Adaptive Capacity to Climate Change, (BACCC) implemented by the Midlands State University (MSU, in Zimbabwe), ICRISAT-Bulawayo (International Crop Research Institute for the Semi-Arid Tropics in Bulawayo, Zimbabwe), the Zimbabwe and Zambia Agriculture Extension Services and Meteorology Services Department. The project was implemented in four districts, two districts in Zimbabwe and two in Zambia. The aim of the project was to understand the effects and impacts of climate change on communities and to improve incentives and opportunities for households in Southern Zambia and South-Western Zimbabwe to cope with climate change. The specific aims of the BACCC project include; investing in improved production technologies of practical value to small-scale farmers and encouraging their adoption by

linking their dissemination with complementary investment in weather forecasting and other projects such as; humanitarian relief, input provision and product marketing. The idea of the overall project is to make the capabilities rather than the vulnerabilities of the poor the starting point for moderating the negative effects of climate change on agricultural production. Key interventions to achieving the objectives of BACCC will include strengthening local institutions, building demand-led rural services, designing decision-support tools for managing smallholder assets including livestock, and developing new technologies for natural resource use under variable rainfall. Once the project objectives are identified and evaluated, dissemination of weather forecast information and encouraging uptake of adaptation strategies will be used among communities to prevent or mitigate the effects of climate change.

The project involves researchers from different disciplines such as sociology, economics, agronomy, soil science and climate sciences; in order to address the diverse issues involved in the project.

In addition to the objectives listed above, the project also aimed at human capacity building through training of graduate students. Students appointed to the project helped with research work and were expected to produce a thesis that addresses part of the objectives of the project. The task for this present study was to provide baseline information for the overall project, concentrating on assessing vulnerabilities of the smallholder farmers

3.3. Selection of study sites

The selection of sites was done at the BACCC project level. The aim was to select areas that are marginal in terms of the climate experienced so as to assess how inhabitants of such communities are being affected or are going to be affected by climate change. Climate

change predictions show that the semi arid to arid areas will be affected the most considering that the current climate conditions are not favorable for smallholder farmers' livelihoods. That was the basis for selecting the two districts which both are in Natural Region (NR) IV.

While the project focused on Zambia and Zimbabwe, this thesis focuses on Zimbabwe. For clarity of the description of the sampling procedure, the administration setup in Zimbabwe and natural agro ecological regions will be discussed.

3.3.1. Administrative set up and agro-ecological regions of Zimbabwe

Zimbabwe is divided into ten provinces, inclusive of the country's two major cities which also have provincial status (Harare and Bulawayo). Each province is itself divided into six or more districts. The districts are in turn divided into wards and a district can have about 20 to 35 wards. Within each ward, are villages that can have more than 30 households each, depending on the size of the village.

Zimbabwe is divided into 5 agro-ecological regions (I to V), primarily defined according to rainfall characteristics as shown in Table 3. The total amount of rainfall and the intensity of specialization in agriculture decreases as one move from Natural Region (NR) I to NR V.

Table 2: Agro-ecological zones of Zimbabwe and the recommended farming systems in each zone

NR	Area (per square km)	Annual rainfall (mm/year)	Rainfall Characteristics	Recommended farming
I	7 000	Greater than 1000	Well distributed throughout the year	Specialized and diversified farming: forestry, fruit, tea, coffee, macadamia nuts and intensive animal husbandry
II	58 600	750 – 1000	Confined to summer	Intensive farming: flue-cured tobacco, cotton, soybeans, coffee, groundnuts, horticultural crops, winter wheat, beef, dairy, poultry and ostrich
III	72 900	650 – 800	Infrequently or heavy; seasonal drought	Semi-intensive farming: extensive beef ranching and marginal production of maize, tobacco and cotton *Important role of irrigation (periodic seasonal droughts, prolonged mid season droughts, rain starts date unreliable)*
IV	147 800	450 – 650	Erratic: frequent seasonal drought	Semi-intensive farming : livestock breeding and production of drought resistant crops (e.g. millet)
V	104 000	Less than 450	Very erratic: drought prone	Extensive farming: extensive cattle farming or game ranching

Source: Vincent and Thomas (1960)

The NR in Zimbabwe and the study sites are also shown in the map in Figure 4. The area covered by the different NR increases as you move from NR I to NR IV (Table 3 and Figure 4) and decreases from NR IV to V. However, most of the land in Zimbabwe is in the semi arid area (III to V) which is vulnerable to climate change since climate change predictions state that the semi arid to arid areas will be affected the most by climate change.

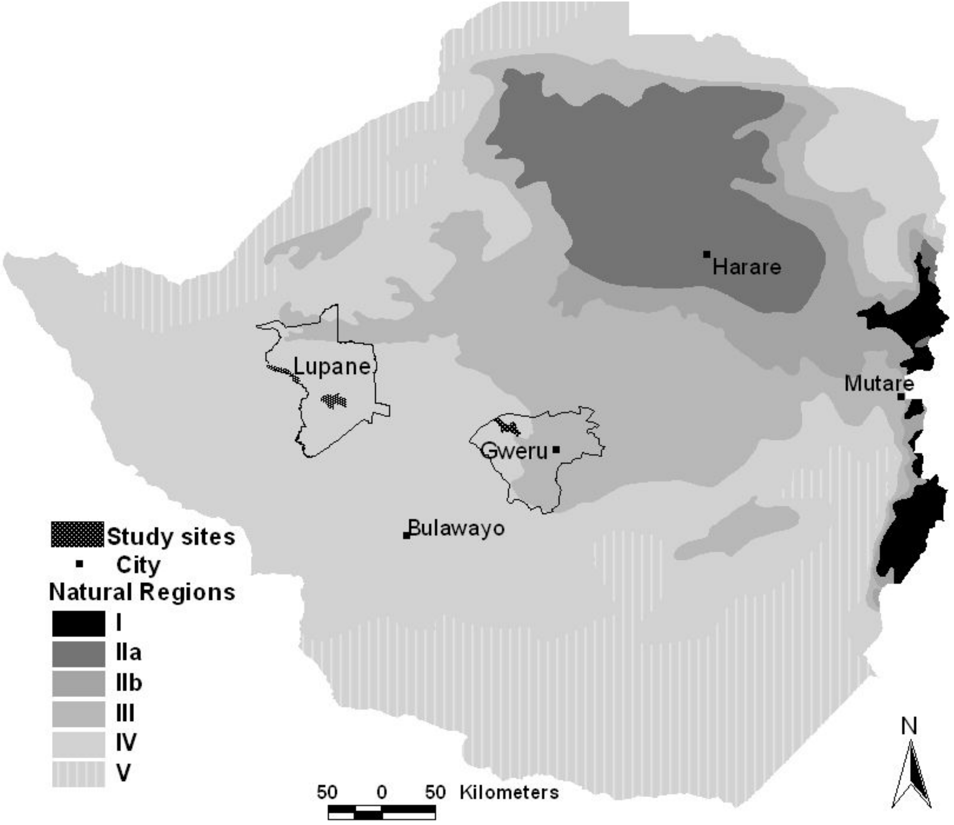

Figure 4: Map of Zimbabwe showing Natural Regions and locations of the study sites
(Constructed by the Geographical Information Systems Department, ICRISAT-Bulawayo)

3.3.2. Sampling procedure and description of study sites

The selection for the study sites was done strategically to meet the objectives of the project and one of the main objectives was to look at smallholder farmers in marginal areas. NR IV was suitable as it is a semi arid area and as stated before, climate change predictions state that the semi arid to arid areas will be affected the most by climate change. Thus Matebeleland North and Midlands provinces were selected. The districts Gweru and Lupane were also selected on the same basis, that is, marginal areas in terms of climate experienced. The two districts have areas that are in NR III and IV as shown in figure 3.

Gweru is in the Midlands province and the district was chosen for its proximity to the Midlands State University, about 80km away, where most of the project members were stationed. The area is also accessible and this would help minimize transport costs. Lupane district (Matabeleland province) was chosen for its proximity to the Zambia BACCC project sites. One of the Zambia wards selected for the study is along the Zambezi valley which is the boundary for the two countries and is on the western side of Zimbabwe and is close to Lupane district. Lupane district has most of its wards in NR IV and is almost 250km away from Gweru. Most people in the districts selected, are smallholder farmers.

At ward level, areas in NR IV and with smallholder farmers were considered; that was the first criteria of selecting the sample. The other criterion used was to list all wards that were accessible and had good road networks. Thus areas which complied with these attributes were included in the sample in which random systematic sampling was used to select two wards for each district. This involved listing all the wards within each district, falling into NR IV, having smallholder farmers as inhabitants and accessible; and then using the random systematic formula to choose two wards from each district. The following formula was used to identify the two wards, where every k^{th} element in the frame is selected, where k is the sampling interval and is calculated as:

$$k = \frac{N}{n}$$

Where n is the sample size, and N is the number of wards within the district which are in NR IV and have smallholder farmers.

The wards selected in Gweru share the same boundary whereas the wards selected for Lupane (Matabeleland province) are about 30km apart with one of the wards being on the district's

boundary (Figure 3). Both areas are also characterized by smallholder farmers. Since the two sites are in the same agro-ecological region, the study aims to identify differences in vulnerability, impacts and responses due to the geographical location and specific socio-economic activities.

At village and household level systematic random sampling was used since all the villages and households were in NR IV. All the households from these villages were also accessible. Table 4 shows the districts, wards and villages selected for this study and a description of these wards.

The sampling procedure involved selecting from each district, two wards; three villages from each ward; and 15 households from each village. A total of 180 households were selected from the two districts, 90 per district, 45 per ward and 15 households in each village. The description of the study sites is also summarized in Table 4.

Table 3: Description of study sites

District	Ward	Villages	Water sources	Soils	Terrain	Framing practices
Gweru	Nyama	Mathonsi<br>Guduza<br>Msingondo	Wetlands, wells and boreholes	Vary from clayey to sandy soils	Flat	Field crops and horticulture production
	Mdubiwa	Madinga<br>Mxotshwa<br>Nsukunengi	Boreholes and wells	Mostly sandy soils	Undulating	Field crops and gardening
Lupane	Daluka	Daluka<br>Gandangula<br>Mafinyela	Boreholes, river, wells	Soils along the river are clayey and sandy away from the river.	Influenced by the river thus is sloppy	Production of drought resistant fodder crops and gardening along the river
	Menyezwa	Banda<br>Masinyane<br>Menyezwa	Boreholes, river and wells	Mostly sandy soils	Flat in two villages and Predominantly undulating in the other village.	Drought resistant fodder crop production. Little gardening done

In Gweru district, the wards selected were Nyama and Mdubiwa wards. Nyama ward has some parts of the land area that consists of wetlands and some of the households have access to these wetlands. The area under wetlands in Nyama is currently decreasing and this has been attributed to the increased frequency of droughts and dry spells experienced in the past 3 decades. These increased frequencies of droughts, dry spells and decrease in wetlands have been given as evidence of climate change impacts in the semi arid tropics (Eriksen *et al*, 2008). Land use patterns such as cultivation of these wetlands can also contribute to the drying up of these wetlands. With increase in population, land pressure has also increased and the demand of the wetlands has also increased with people cultivating on them to meet household food security. The wetlands have dark clayey soils that are very fertile thus boost productivity. Thus farmers prefer them over the dry fields which are characterized by sandy soils that are prone to leaching, thus compromise yields.

Since Mdubiwa shares a boundary with Nyama, some farmers from Mdubiwa have access to some of these wetlands and this has also increased the pressure of land use on these wetlands. Mdubiwa mainly consist of dry land, most farmers used to depend on an irrigation scheme known as Shagari Irrigation Scheme, whose dam wall was destroyed by the December 2008 excessive rains. With current and future climate change, this compromises further, the productivity of these farmers and thus increases their vulnerability to food insecurity and also increases the chances of being negatively impacted by climate change.

According a Gweru AGRITEX officer, farmers from these two wards in Gweru were dependent on horticulture production in the past. This was made possible by the availability of due to the availability of wetlands and irrigation scheme. They were able to produce for home consumption and also have surplus to sell to local and distant markets. Since the past

decade, production has decreased and farmers now concentrate on producing for family consumption and rarely have extra to sell. The type of crops grown has also changed, with farmers focusing on planting more of cereals, such as maize, millet and sorghum for household food security compared to vegetables that were mostly grown in the past. Thus there has been reduction in diversity of crops grown since conditions have deteriorated for crops that require to be irrigated constantly. The farmers from these wards also engage in livestock production although it is not much compared to Lupane. Livestock are mostly kept for draft power, emergencies like meeting medical bills and covering funeral expenses.

The two wards are about 80km away from the city of Gweru, a provincial capital for Midlands. The roads are tarred and make it easy to reach the areas. With the availability of vehicles, the wards are easily accessible. Public transport also frequents the two wards with buses and trucks being prevalent in the area throughout the day. It makes it easy for people to move from one area to another thus farmers can easily get inputs from markets.

There are local organizations that offer different services to the communities and these include agriculture extension (AGRITEX), health, education, traditional leadership among others. Organisation found within communities and the role they in helping to mitigate the impacts of climate change will be discussed further in Chapter 4. The agriculture extension officers are stationed within the wards; the challenge is that due to the high turnover rate with personnel leaving the country, there is limited access and interaction of farmers with officers. The extension officers working in the areas are young ladies and males, who are inexperienced and lack skills and often cannot relate to the local farmers. Compounding the situation with current and future climate change, this exacerbates the vulnerability of these households to food insecurity and climate change.

Lupane is the provincial district for Matabeleland North Province and the wards selected for the study are Daluka and Menyezwa ward. The villages selected for Daluka ward in Lupane district are found along the Bubi River, a tributary of the Gwayi River. The extension officer for Daluka mentioned that; in the past, the Bubi River would have enough water for both humans and livestock throughout the year. He also indicated that due to increased frequency of droughts and dry spells, farmers have resorted to stream bank cultivation resulting in soil erosion and siltation of the river. This has decreased the river flow and the river only flows during the rainy season and dries up soon after the rainy season. Most of the farmers in Daluka ward, have since abandoned their fields, in favour working on their gardens located along the banks of the Bubi River. The fields a located on dry sandy soils, with low water retention capacity thus do not retain adequate water to support plant growth. Stream bank cultivation is illegal in Zimbabwe, but traditional leaders cannot control the farmers considering the situation of droughts and dry spells which have be-fallen the communities. In the past, the plots along the river were smaller and were located 30 meters away from the river as advised by agriculture extension; and environment and resource management institutions. Farmers would plant mostly vegetables on these small plots which need constant irrigation and were able to sell their vegetables to the St Luke Hospital found within the ward. In the past decade, farmers have increased the sizes of the plots along the river and have moved more towards the river than before, with some farmers practical planting within the river during the dry season. The plots are now cultivated for maize production during the cropping season for food security reasons.

Large fields for dry land crop production that are not located along the river but upland where it is dry are often allocated to drought resistant crops such as sorghum and millet. Farmers in

this ward raise livestock that are very rarely slaughtered for meat or sold to buy food for the household. Most households reported that livestock are sold to raise money for paying for school fees for the children or to cover medical expenses. The food supplies of the household mostly depends on own production of maize, sorghum and millet and this is common in all the project sites.

Menyezwa also has some villages along the Shangani River which is not a perennial river, that is, it does not flow throughout the year. This compromises the availability of water for these households, as this is their major source of water for domestic and livestock use. Two thirds of the households selected for this study are not located near the river and have problems with access to water, they depend on boreholes and most of the boreholes are not functioning and some have since dried up. The Ward is generally dry and experiences water shortages during the dry season. With occurrence of climate change, this further compromises their livelihoods and increases vulnerability.

Farmers in the area of Menyezwa are mostly into cereal crop production, that is maize, sorghum and millet and not much gardening is done because of water shortages. Less than 40% of the households reported that they produce vegetables, the majority of sampled household do not produce vegetables because of water shortages. Farmers from the ward mentioned that drought tolerant crops such as pearl millet and sorghum are favorable in their area and are mostly grown. Livestock is also kept by the farmers and like the other wards, are only sold to pay school fees, meet medical and burial expenses.

Both wards (Daluka and Menyezwa) are located along the main road which connects the Bulawayo city and Hwange town. The area is adjacent to the Hwange National Park and the

park is one of the major tourist attractions in the country. The roads are tarred and are in good condition. Public transport is available and the community has access to transport. The wards like in Gweru have organizations such as agriculture extension (AGRITEX), health, education and traditional leadership, forestry, offering services to the communities[5]. The AGRITEX officers reside in the area and unlike in Gweru, the officers did not leave the country so have been in the area for more than 10 years. The officer for Daluka resides at the Lupane district centre which is more than 20km from the ward due to the unavailability of accommodation within the ward of operation. The officer for Menyezwa resides within the community. The role of the AGRITEX officer is to train and advise farmers on agriculture related issues. Considering that these households obtain their livelihood from agriculture, AGRITEX has a big role to play in compounding the effects of climate change on agriculture so as to help farmers mitigate against climate change impacts and reduce vulnerability to climate change.

3.4. Data needs and sample size

The primary data used in this study was obtained from a survey carried out in September 2009. The survey gathered qualitative and quantitative data pertaining to social, demographic and economic aspects of the households, agriculture activities, farmers' perceptions of climate change and the role of local organizations in helping smallholder farmers develop strategies to mitigate against the negative climate change. The tools used to gather data and the data collected are described in the proceeding sections.

[5] AGRITEX will be discussed in this section and the other organisations will be discussed in chapter 4

3.4.1. Quantitative data

Data on production/acquisition of cereals, household size and asset ownership was gathered, as summarized in Table 5. This data was gathered using the household questionnaire.

Table 4: Data from the household questionnaires

Type of data	Specific data collected
Demographic household data	Age, sex, marital status, education level of the household head; number of household members; whether the household head is a member of any social group and has received any agriculture training
Agriculture production	Arable land owned; crops grown and areas allocated to the crops; yields obtained; farming implements available; availability of draft power; livestock owned; crop management practices
Income sources	Sale of crops, livestock and vegetables; informal and formal employment; remittances
Capital and domestic assets	Ox, drawn plough; ox-carts; harrows; cultivator; hoes; radios, bicycles, mobile phones
Farmer perceptions to climate change	Whether they have noticed any changes in climate; what changes they have noticed; access to weather forecast information; sources of information;
Adaptive strategies to climate change	Growing drought resistant crops and early maturing varieties; practicing conservation farming in the form of digging basins, pot holing and ripping

Data collection was carried out with the technical assistance of ICRISAT-Bulawayo. Four enumerators assisted with data collection. During data collection the data collected by the enumerators was checked and thus all the information that needed verification and clarification was dealt with during the survey at the study sites.

3.4.2. Qualitative data

Qualitative data was obtained from Focus Group Discussions (FGD) with farmers and key informant interviews (KII) with local leadership. The objectives of the FGD was to discuss community livelihood strategies, constraints and opportunities; gain in-depth knowledge of the farming systems; get preliminary information/insight into farmer perceptions of climate change and variability; and document common adaptive and coping strategies.

One FGD was conducted per ward and each group had 15 participants. In participatory research it is advised to have a group of about 10 to 15 people in a FGD (Nachamais *et al.*, 2008). A group of more than 15 participants can be difficult to facilitate and less than 10 participants may not be a reliable representation of the community. The FGD participants consisted of males, females and different age groups to capture information from different categories. The participants for the FGD were selected from the villages where the household questionnaires were administered. Information collected from the FGD and the gathering tools used is shown in Table 6.

Table 5: Tools and information collected from the FGDs

Tool	Information
Resource mapping	Natural resources found in the communities
Opportunity and constraints analysis	Livelihoods of the households and cropping patterns
Trend lines	Major events/ occurrences that are related to community resources, rainfall and the general climate
Institutional mapping	Local institutions and the services provided

KII involved carrying out interviews with stakeholders such as local leaders, government and non government service providers in the areas of study. KII were also carried out to get in-depth information on local institutions, that is, their role in the communities; their perceptions

on climate change and how they were addressing impacts of climate change. The list of organizations interviewed included, traditional leadership, AGRITEX, schools, clinics, churches and NGOs. A checklist of the issues discussed with key informant is shown in Table 6.

Table 6: Key informant guide questions

	**Questions**
1	Name of organization
2	Type of services the organization provides to the community
3	The organization's coverage
4	A description of the local environment
5	Have they noticed any changes in the environment and climate
6	A description of the livelihoods of the local households
7	Are they changes in the livelihoods patterns of the households
8	How the organization contributes to the welfare of the households
9	Does the organization have access to weather forecast information
10	If they have access, what is their major source of information
11	Is the organization aware of climate change
12	Are they addressing climate change within the community
13	If they are addressing climate change, how are they doing it
14	If not addressing climate change, do they have plans to do so

3.4.3. Secondary data

Secondary data collected, was information obtained from organizations such as the Zimbabwe Meteorology Services and this included temperature and rainfall data that was used to describe the climate of the two sites. The other sources included information obtained from AGRITEX, on the cropping patterns of the areas and also data from the Climate Change office located in Harare for studies that had been done in Zimbabwe on climate change. Data from the Central Statistics Office located in Bulawayo and FAO reports on Zimbabwe were also useful in informing the background information of the study.

3.5. Data Analysis

After completion of the field work in September 2009, the data from the household questionnaires was post coded. Coding was done to provide a means for structuring and viewing large amounts of data and it also aids analysis. Data was captured using the SPSS spread sheet and cleaned.

The cleaned data was then analyzed by running descriptive statistics; mainly frequencies, descriptive and crosstabs. This was useful in characterizing the two sites, Gweru and Lupane so as to find out issues like gender, education levels of the household heads, assets owned, livestock owned, production patterns and whether they have access to extension and weather forecast information. Data from SPSS was also exported to Excel in order to draw graphs and tables.

The other analyses carried out involved running the 2 stage least squares regression model using SPSS to find estimates for the vulnerability model. This involved a double regression of the per capita cereal production levels against household observable characteristics such as age, gender, education status of the household head, assess to extension services and other factors that were considered pertinent in influencing cereal production. The estimates obtained from the 2 stage least regression was used to measure the degree of each household's vulnerability to food insecurity. The estimated probability was given by:

$$\hat{v}_h = \hat{\Pr}\left(\ln c_h < \ln z \middle| X_h\right) = \Phi\left(\frac{\ln z - X_h\hat{\beta}}{\sqrt{X_h\hat{\theta}}}\right)$$, see equation 12 in chapter 2

The outcome of the above model measures the degree of vulnerability to food insecurity for each household. The probability of a household being vulnerable to food insecurity is ≥ 0.5

and the probability a household not being vulnerable to food insecurity is < 0.5, thus a threshold of 0.5 was used. Food insecurity increases the chances of being negatively impacted by climate change. Thus a household with a probability of < 0.5 has less chances of being negatively impacted by climate change and a household with a probability ≥ 0.5 has greater chances of being impacted by climate change.

3.6. Summary

The chapter has highlighted the origins of this study. It has also described the study sites and methods used in selecting sites, data gathering and data analysis employed. Two districts were selected for the study, namely Gweru and Lupane districts in Zimbabwe. These districts are located within the same agro-ecological region and a total of 4 wards, 12 villages and 180 households were sampled for the study. The data collected from the survey conducted was analyzed using SPSS 14 windows.

The cropping systems are generally similar and cereal production is important in both districts as farmers allocate large areas for cereal production. Thus the level of cereal produced was used to measure the household's degree of vulnerability to food insecurity. The food insecurity status of the household increases the chances of being negatively impacted by climate change. Thus a household that is vulnerable to food insecurity has greater chances of being negatively impacted by climate change.

CHAPTER 4

SMALLHOLDER FARMERS LIVELIHOODS, AGRICULTURE AND CLIMATE CHANGE IN GWERU AND LUPANE DISTRICTS

4.1. Introduction

This chapter provides a description of the households, the agriculture and other economic activities, climatic trends, perceptions of climatic changes in the area and the current and projected impacts of climatic change. The chapter also looks at responses at local level to help cope and adapt to climate change, government policies in this regard and help from organizations to reduce vulnerability to climate change. The first part discusses climate trends in the study areas. The second part describes the households, characterizing smallholder farmers in Gweru and Lupane, in detail. This will be followed by the discussion on agriculture and other livelihood activities carried out by the households. The following section looks at how climate change influences or interacts with household characteristics and livelihood activities and how farmers are coping and adapting to climate change. The final section describes national institutions and the role they can play to help reduce vulnerability to climate change.

4.2. Historical climate trends for the areas of study

The data used for this section was obtained from the Zimbabwe Meteorology Services Department and is data for Lupane district only. The data given for a particular district is derived from averaging climate data taken from different weather or meteorology stations

within that district, thus is average data for the district as a whole and is not specific to the areas of study, although it helps in showing the general climate trends experienced in the areas. Lupane district has more than 80% of its area covered by NR IV and less than 20% covered by NR III[6]. Gweru has more than 50% of its area in NR III and the rest of the area covered by NR IV. Although this is the case, the wards selected for this study are all in NR IV. Therefore for this section, historical climate data for Lupane will be used to explain the climate trends for the areas of study since the data is a better representation for NR IV, in which the study sites are located.

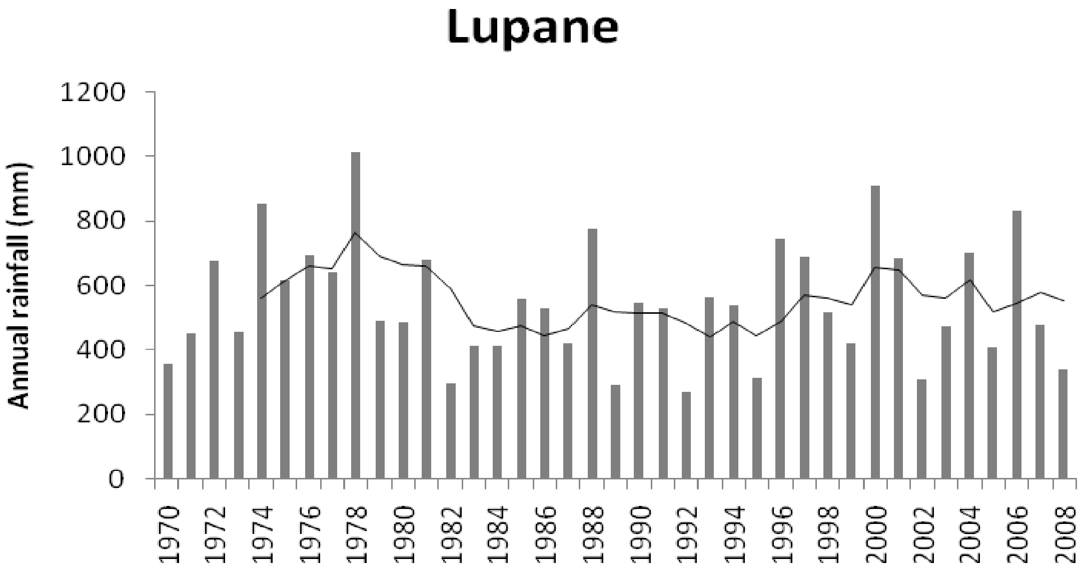

Figure 5: Annual rainfall for Lupane district in the period 1970-2008
Source: Zimbabwe Meteorology Services, Government of Zimbabwe, Harare, Zimbabwe

The graph in figure 5 shows annual rainfall from 1970-2008 and a trend line for 5 year moving averages. The moving averages are used with time series data to smooth out short term fluctuations and highlight longer term trends or cycles. The annual rainfall graph shows that over the past 30 years there is no visible change in annual average rainfall. Although this is the case, during the FGD farmers mentioned that in their areas, they were experiencing changes in the onset of the rainfall season and increased frequency of dry spells. The farmers

[6] This is shown by map of Zimbabwe showing natural regions and locations of study sites in chapter 3

mentioned that the rainfall season is now starting late with some seasons starting end of November or early December compared to previous years when it started in September or early October. The start of the rainfall season is when rainfall accumulated over 1 or 2 days is at least 20mm (Stern, 2006). The increased frequency of dry spells within the rainfall season was also mentioned by farmers and a dry spell is characterized by a period of 10 days or more with less than 0.85mm daily rainfall (Stern, 2006). A case study from the semi arid Zimbabwe done by Mugabe *et al.,* (2003) on opportunities for increasing productive water use from dam water, mentioned that poor rainfall distribution within the growing season was the main cause for crop failure, due to dry spells at critical stages of crop growth. Famers also mentioned that these changes in climate were making it difficult for them to plan for their cropping activities. Households in Gweru and Lupane mainly depend on agriculture for their livelihood and a change in climate is likely to compromise their productivity and increase vulnerability to food insecurity among the households.

The deviation from the average long term annual maximum temperature increased from 1985 to 1997. The IPCC (2007) reports increase in temperature for the semi arid tropics, as evidence of climate change. Increase in temperature compounded with erractic rainfall being experianced in Gweru and Lupane has negatively affected agriculture productivity, thus compromising the food security status of the household.

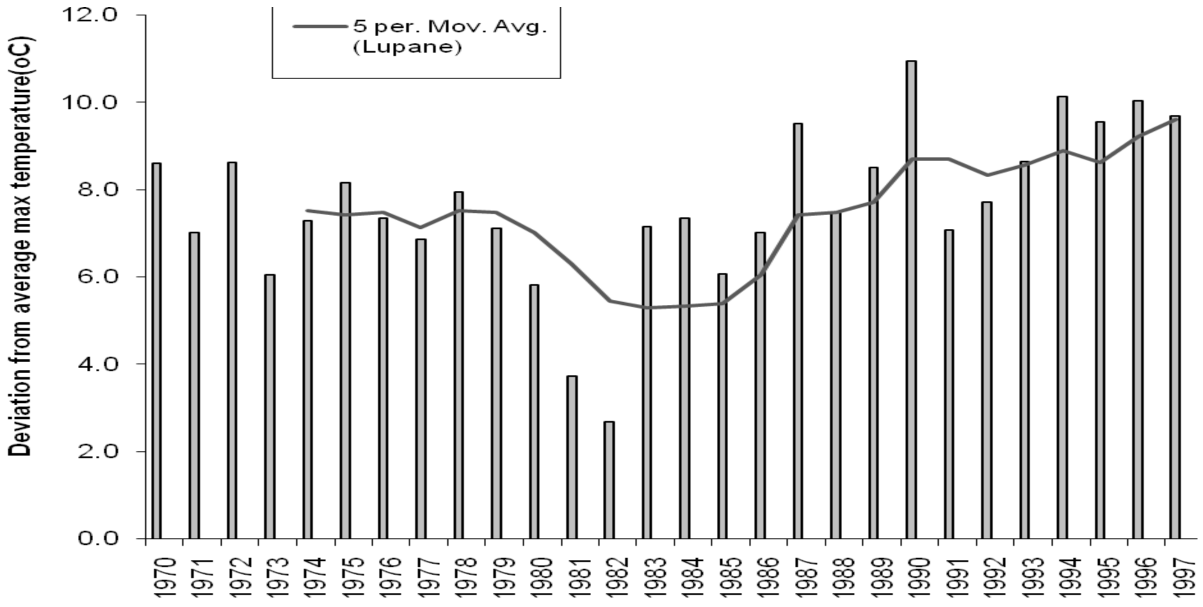

Figure 6: Deviation From Annual Average Maximum Temperature for Lupane distrcit in the period 1970-1997
Source: Zimbabwe Meteorology Services, Government of Zimbabwe, Harare, Zimbabwe

The health departments from Gweru and Lupane also reported higher incidences of malaria and these have been associated with the increased temperature that are lately being experienced. Acoording to the sister in charge at a hospital in Lupane, Lupane district is ranked the third, in terms of malaria prevalence in Zimbabwe because of the hot weather experienced. This is also likely to increase vulnerability among households to current and future climate change.

4.3. Socioeconomic characteristics of the households

The section describes household characteristics of the households in Gweru and Lupane (Table 7) by looking at household attributes such gender, age of the household head, among other factors, that are likely to influence vulnerability of the households.

Table 7: Household characteristics for 358 households interviewed in Lupane and Gweru districts (2009 Survey)

Variables	Description	Gweru	Lupane	Total
Number of respondents		178.	180	358
Gender of household head	Female (%)	48.3	28.7	38.4
Age of household head	Years (mean)	53.7	51.8	53.7
Education level of household head	None (%)	9.4	19.1	15.4
	Primary	55.5	52.2	52.7
	Secondary	32.2	26.4	29.4
	Tertiary	2.8	2.2	2.5
Farming experience of household head	Years	21.9	23.1	22.5
Livestock owned	Cattle (%)	39.4	62.9	51.1
Land owned	Area (ha)	4.1	6.8	5.4
Member of social group	Yes (%)	72.9	46.0	59.3

In the two districts the majority of household heads are male headed (62.6%). From the female headed households, 12.9% are *de facto* female heads, that is, the females are married but not living with their husbands and 87.1% are *de jure* female heads: these are either widowed, divorced or have never been married. Female headship, specifically looking at *de jure* female headship is typically expected to increase the likelihood of the household being found amongst the vulnerable (Horell *et al,* 2006). Female *de jure* headed households are often constrained by their lack of access to resources such as labor, farming implements and income. Most of the female *de jure* headed households also depend mostly on agriculture for their livelihoods and with the presence of climate change; they are more likely to be vulnerable to food insecurity and climate change.

The average age of the household heads across the two districts is about 53 years (Table 8). The retirement or economically inactive age is 65 years, which is higher the than average age found amongst household heads in Gweru and Lupane district. About 69.5% of the

households heads fall within the 25 – 64 years age group and 30.5% fall within the group that is above 65 years old. Thus the majority of household heads are among the economical active age group. The age group above 65 years often becomes a liability to the family as most people often become weaker and have failing health with increase in age thus need to be taken care of, dividing labor that could be used to boost productivity for the household. This could further increase the likelihood of the vulnerability status of the household.

The literacy levels of the farmers in both Gweru and Lupane districts were high with only 15% of the household heads lacking any form of education. Thus about 85% of the household heads had at least 7 years of schooling, suggesting that most farmers in Gweru and Lupane districts are functionally literate. An educated household head is able to read fertilizer and seed instructions found on the packages of the products, they are also able to read educational pamphlets that are often given to farmers to educate them on new innovations and technologies by agriculture extension officers and NGOs. Thus this can help improve the productivity of households and help reducing vulnerability to food insecurity. This can be supported by Weir (1999) who highlighted that education may enhance farm productivity directly by improving the quality of labor. Shultz (1975) can also support the theory about education and he states that education is important to farm production, especially in a rapidly changing technological or economic environment. In addition, traditionally, research has also shown that there is positive correlation between level of education of farmers and the speed with which they pick an innovation (Weir, 1999). This means that farmers who are learned, that is, those who can read, understand and analyze issues and are more capable of adopting a technology, thus are able to compound existing and future climate change by adapting strategies that are relevant to the changing environment.

On average, household heads have been engaged in farming for 22.5 years. The farming experience of the household head is likely to influence productivity of a household. A farmer with more years of farming experience can also notice changes between when they started farming up to the current seasons. In addition such farmers are more likely to adapt better to the changing environment compared to farmers with less farming experience.

Farmers in Gweru have on average 4.1ha of agriculture arable land whereas farmers in Lupane have 6.1ha. There is a 1% level statistical significant difference between the two districts. This can be attributed to the population density in the two districts. Households in Gweru are more clustered compared to Lupane where they are dispersed and the population is lower.

Whether the household head is a member of a social group was also considered for this study. A social group is defined by a group of people who meet for a specific common cause (Julia, 1999). In Gweru and Lupane this included groups specializing in nutritional gardens, dressmaking, baking, money lending and building. Such groups serve as networks where information is exchanged, members can also learn new things and this is a platform for discussing new innovations and addressing constraints being faced by the group members. Such groups can also be used as platforms to disseminate climate change information and this can help educate farmers on the subject and reduce vulnerability to climate change. In Gweru 72.9% of the sampled households heads were members of at least one social group, whereas in Lupane they were 46% (Table 8). The reason could be that, the wards in Gweru are near the Gweru city, so communities are more likely to be in touch with new projects and organizations that fund the projects can visit more often frequently compared to Lupane. In

addition, from the group that mentioned that they were members of a least one social group, 86.7% mentioned that the group they were part of, was agriculture orientated.

4.3.1. Asset ownership

Assets owned by households include domestic assets such as TVs and radios; farming implements such as scotch carts and hoes; and livestock. These assets are often used to determine the welfare status of the households, that is, a household with more domestic assets, farming implements and livestock is considered better off compared to a household with less. The assets can also be sold to mitigate a crisis that befalls a household thus are often a source of income. With current and future climate change, a household can also sell some assets that will enable them to adopt a technology such as a water pump to enable households to irrigate their vegetables which can be sold to improve the household's livelihood and reduce vulnerability to climate change.

Results show that less than half of the farmers from both districts own radios or TVs, bicycles, mobile phone and clocks (Table 8). These assets can be important means of communication and information transfer and are only useful if there are service providers that are using these modes to disseminate information such as agriculture related TV and radio programs. In Zimbabwe there are few agriculture programs aired on TVs and radios and there are no agriculture promotions programs that are sent using mobile phones. Through the TVs and radios there is the daily weather report, which the farmers highlighted to be less informative and one agriculture program is aired once a week which address agriculture activities. In developed countries, farmers have access to internet, through their phones and computers; and also receive relevant information for their areas through TVs and radios to tackle different climate challenges that they often experience.

Table 8: Domestic assets ownership among households (%) in Gweru and Lupane districts (2009 Survey)

Domestic assets	Gweru (% owning)	Lupane (% owning)
Radio/TV	32.1	34.8
Bicycle	27.6	26.4
Watch/clock	4.2	5.3
Mobile phone	45.5	39.1

Ownership of farming implements are important inputs in agriculture and also influence the household's productivity. Households lacking adequate farming implements often wait for those with farming implements to finish their farming operations so that they can borrow. This often results in such households delaying to prepare their land for planting thus compromising productivity since timing especially for planting is critical in influencing yields obtained. Climate change further compromises households without implements because it is important for land preparation to be done early so as to coincide planting with onset of rains.

More households in Gweru have harrows and cultivators compared to households in Lupane (Table 9). There is no significant difference in ownership of implements such as the ox drawn ploughs, oxcarts and ridging ploughs between the two districts. Although there is more than 60% ownership of ox drawn ploughs, hoes and axes, less than 4% of the households have planters, rippers and ridging ploughs. Farmers can use other implements in the place of these implements, thus this shows that they are replaceable.

Table 9: Farming implements ownership (% among households) in Gweru and Lupane districts (2009 Survey)

Farming implements	Districts	
	**Gweru (%)**	**Lupane (%)**
Ox drawn plough	63.3	66.7
Scotch cart	39.1	47.2
Harrow	30.7	10.4
Ridging plough	3.3	1.3
Cultivator	24.2	11.7
Sprayer	8.9	10.2
Hoes	97.4	97.1
Ripper	1.2	0.8
Axe	93.8	96.2
Planter	2.7	1.3

The scotch cart is an important means of transport for the smallholder farmers in Gweru and Lupane districts. It is used to transport products from the fields to the homesteads (Figure 7). The scotch cart is also used in most rural areas for transporting people, for example from the business centers to the homesteads. Scotch carts can also be used to generate income by hiring out the scotch carts to other farmers. Between the two districts, Gweru has 39.1% and Lupane has 47.2% of household in possession of a scotch cart.

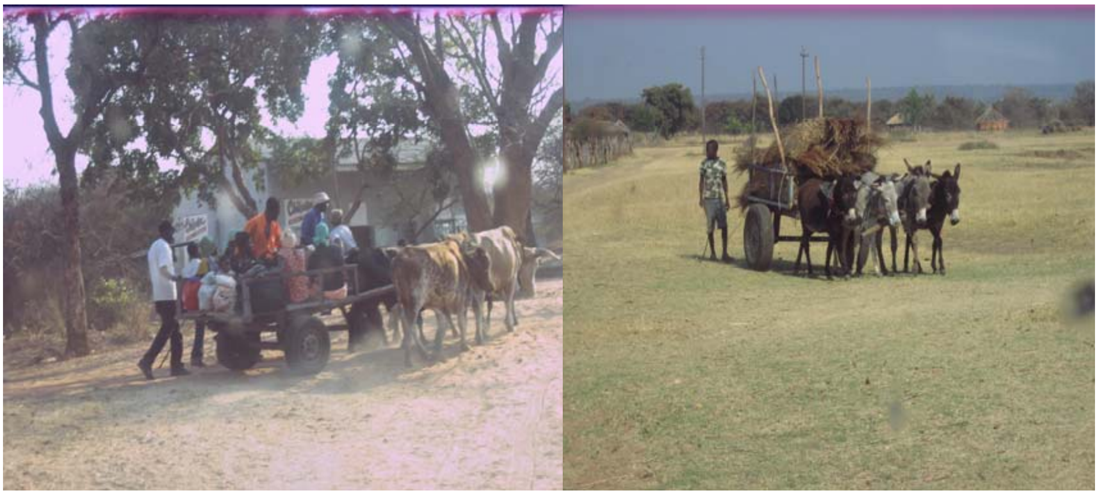

Figure 7: Use of scotch carts to transport people and goods in Lupane (2009 Survey)

Livestock are an important potential asset for rural households (Cousins, 1996). They are often kept for emergencies such as meeting medical bills and funeral expenses. Some households often sell small livestock such as goats, sheep and poultry to raise school fees for their children. More than 80% of the households have poultry (Figure 8). Poultry also contributes to food in the form of the meat and the eggs. This contributes to the food security status of the household since poultry provides food and income for the household.

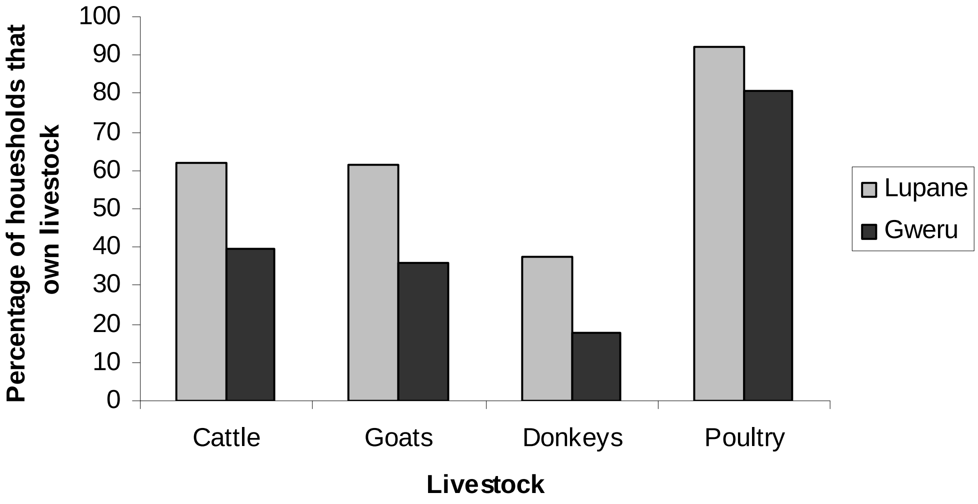

Figure 8: Livestock ownership among households in Gweru and Lupane districts (2009 Survey)

In Lupane, 62% of the households have cattle compared to Gweru where 39% of the households have cattle. On average farmers in Lupane have 5 head of cattle and 2 in Gweru. Smallholder farmers use cattle and donkeys for draft power in farming operations and also to transport goods and people, for example to markets and clinics.

The economic rationale for cattle ownership is firstly to provide income for the household, then draught power for tillage and manure, lastly to provide milk and meat for local

consumption. While they have social and cultural functions which are important these are generally secondary to economic functions (Barrett, 1996). Respectively, about 56% and 33% of the households in Lupane and Gweru sell livestock to obtain income. There is a 1% significance difference between the two districts (Table 11)[7]. Farmers in Lupane are more into livestock rearing and consider it as an important source of income compared to other sources. This is supported in a study done by FAO (2008); the results show that in Matabeleland (includes Lupane district) livestock is an important source of income compared to other areas which have mixed production. Gweru district is an area with mixed production of cereal crops, horticulture and livestock production.

a) Lupane b) Gweru

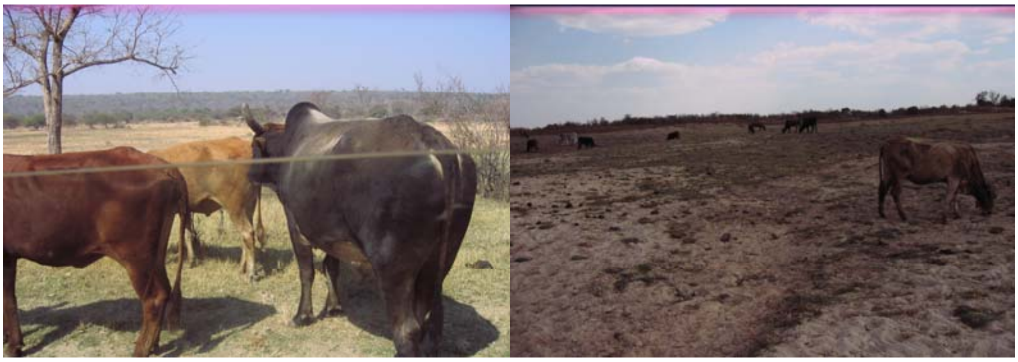

Figure 9: Communal grazing and quality of rangelands in Gweru and Lupane district (2009 Survey)

Communal grazing is practiced in Gweru and Lupane and the major constraint for livestock production in both districts is the poor quality of rangelands (see figure 9). In addition most of the land that was used for grazing has been allocated to people due to the growing population, for their fields and also to build homesteads. This compounded with climate change will reduce livestock productivity since the current quality of graze land is poor and climate change is bound to further reduce the quality and quantity of grazing land for the livestock.

[7] The table that shows household income sources is in section 4.3.3

4.3.2. Crop production in Gweru and Lupane district

For rural livelihoods, crop production constitutes one of the major sources of livelihood. Farmers in both Gweru and Lupane grow a diverse range of crops and these are shown in Table 10. Maize has the largest proportion of area planted in the two districts, occupying almost 1ha of farmers' fields (Table 10).

Table 10: Crops grown and land allocation for the 2008/09 season in Gweru and Lupane districts (2009 Survey)

Crop	Gweru		Lupane	
	Households growing crop (%)	Average area (ha)	Households growing crop (%)	Average area (ha)
Maize	100.0	0.95	100.0	0.92
White Sorghum	27.9	0.10	62.5	0.38
Red Sorghum	12.8	0.11	11.4	0.28
Pearl Millet	0.0	0.00	58.0	0.4
Finger millet	27.9	0.17	1.1	0.05
Groundnut	61.6	0.11	17.1	0.15
Cowpea	23.3	0.14	35.2	0.24
Bambaranut	23.3	0.24	10.2	0.1
Vegetables	29.1	0.19	39.8	0.02
Sunflower	8.1	0.08	0.0	0
Sweet potatoes	11.6	0.11	1.1	0.4
Sugar beans	9.3	0.24	5.7	0.16

Other crops grown include sorghum, millet, groundnuts and cowpeas. About 63% of farmers in Lupane grow and 58% grow pearl millet compared to 28% of Gweru farmers growing white sorghum.

In Gweru 26% of the farmers obtain income from crop sales and 21% in Lupane[8]. There is no significant difference between the two districts. Cereal crops contribute highly to the food

[8] A table of sources of income for the households in Gweru and Lupane is shown in section 4.3.3 (Table 11)

security status of the household. Households keep crops for home consumption as crops are major sources of the household diet and rarely sell to get income.

4.3.3. Other income sources

Other major income sources excluding crop and livestock sales that are likely to contribute significantly to household livelihoods will be discussed in this section. Household income from the rural communities often comes from agriculture activities and other off farm activities. The income is needed to meet some household expenses, for example health services from the clinic and education from schools. The income obtained is also often used as capital for agriculture purposes, in buying inputs. Farmers were asked to list their income sources and Table 12 shows the percentage of farmers who obtain income from the listed sources.

Table 11: Income sources for households (%) in Gweru and Lupane Districts (2009 Survey)

Source of income	Districts		Chi square test	
	Gweru (%)	Lupane (%)	chi Value	p value
Sale of crops	26.1	23.4	0.644	0.422
Sale of livestock	32.8	56.1	18.936***	0.004
Informal work	49.3	35.7	6.128**	0.013
Formal employment	10.2	12.4	0.298	0.585
Remittances	12.1	26.3	12.634***	0.009
Old age pension	2.8	2.3	0.103	0.748
Gifts received in kind	24.3	16.8	2.729	0.099
No income at all	13	8	1.789	0.181
Gardening	67	51	8.934***	0.003
Brick making	4	12	6.501**	0.011

Significant at:
*** 1% level of significance
** 5% level of significance
* 10% level of significance

In Gweru district 67% of the farmers engage in gardening compared with 51% of farmers in Lupane district. Gweru farmers grow their vegetables on small plots located on wetlands. In the past decade farmers were able to produce vegetables throughout the year, but due to the increased frequency of dry spells, droughts and erratic rainfall the wetlands often dry up during the dry season. With current and future climate change this is bound to worsen the situation, making farmers vulnerable to climate change. In the past decade Gweru smallholder farmers in the area of study were known for vegetable production especially tomatoes and they supplied vendors from Gweru city. In addition the Shagari Irrigation Scheme in Mdubiwa ward was a source of livelihood for the communal farmers. The 2008 December rains destroyed the dam wall and farmers can no longer make use of the irrigation facility (Figure 10). In situation of increased drought frequencies, Irrigation can be an important strategy to assure food production.

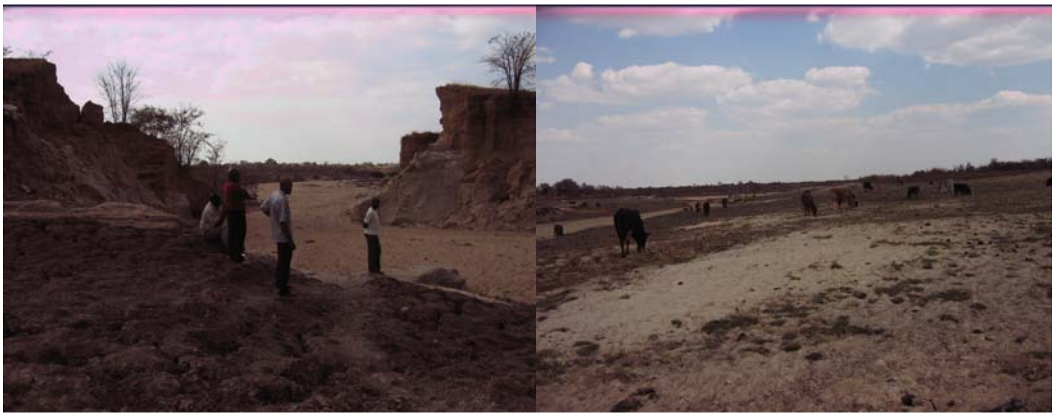

Figure 10: Shagari dam wall destroyed by excessive rains (2009 Survey)

In Lupane, most of the gardening is done along the river banks. This is illegal in Zimbabwe and has increased erosion and siltation of the rivers in Lupane. The rivers are a major source of water for the households but due to the erosion and siltation caused by stream bank cultivation, the amount of water for domestic, livestock and gardening use has been reduced. This compounded with climate change, is likely to induce vulnerability to climate change.

Informal work constitutes off farm activities and is also another source of income for rural households. This includes gold panning, crafts making and firewood trading. More households in Gweru (49%) are involved in informal work compared to the 36% in Lupane. Some of the informal work practiced by the households; such as gold panning and selling of firewood have contributed in destroying the natural ecosystem. Most of the gold panning is done along river banks and this has destroyed rivers, compromising water availability for the communities. Firewood trading involves cutting down trees which leads to deforestation. In Zimbabwe deforestation is one of the major causes of micro climate change (IUCC, 1994). However, farming remains important but rural households are looking for diverse opportunities to increase and stabilize their livelihoods. Although this is the case, farmers cannot exclusively rely on these activities to ensure household food security.

4.4. Household consumption and food requirements

For most rural households in Zimbabwe, cereals contribute highly to the main diet of the household, especially maize which is the staple food for the country (Chigwada, 2005).

Maize yields were generally low between the 2004/05 to 2006/07 seasons (Table 12). The 2007/08 season had the highest yields with farmers in Lupane having an average of 904kg/ha and 705kg/ha for Gweru farmers. The 2007/08 season was characterized by excessive rains throughout the country. This was an advantage for farmers in the drier parts of the country who normally receive below average rainfall, thus they were able to produce more compared to the previous seasons. According to Piha (1993), who did a study in Zimbabwe on optimizing fertilizer use and practical rainfall capture in semi-arid environment with variable rainfall; for NR IV, the expected yield for maize is 2 tons per ha. Over the past 4 seasons recorded, farmers in both Gweru and Lupane have produced far below the expected yield.

Table 12: Maize yields (kg/ha) obtained by farmers in Gweru and Lupane districts for seasons 2004/05 - 2007/08 (2009 Survey)

Season	Gweru	Lupane	t value	p value
2007/08	705	904	1.689	0.093*
2006/07	143	140	-0.115	0.909
2005/06	227	283	1.237	0.217
2004/05	276	204	-1.946	0.053**

Significant at:

*** 1% level of significance

** 5% level of significance

* 10% level of significance

Sorghum yields obtained by farmers in Gweru and Lupane are also shown in Table 13. The 2007/08 was also a good year for Gweru and Lupane farmers and the highest yields were obtained during that season. The previous seasons had low yields compared to the 2007/08 season. The expected yield for sorghum in NR IV is 0.8 tons per ha (Piha, 1993). Lupane in the 2007/08 was able to produce above the expected yield. For the other seasons, farmers in Gweru and Lupane produced below the expected sorghum yield.

Table 13: Sorghum yields (kg/ha) obtained by farmers in Gweru and Lupane districts for seasons 2004/05 – 2007/08 (2009 Survey)

Season	Lupane	Gweru	t value	p value
2007/08	920	714	-1.748	0.098*
2006/07	107	113	-0.235	0.814
2005/06	344	209	0.922	0.358
2004/05	169	226	-0.849	0.397

Significant at:

*** 1% level of significance

** 5% level of significance * 10% level of significance

Farmers were asked about the food security status of the household for each month in the year and the results are shown in Figure 10. Less than 50% of the households have adequate grain supplies in any month during the year. The situation is a little more dire for Lupane compared to Gweru district. The food security situation in the districts starts deteriorating a month or two after the harvest time April/May. November is the month when the largest number of household face food insecurity. This also coincides with the onset of the new growing season. By December those households who planted their maize crop start harvesting some of the maize for consumption as green mealies (corn-on-the-cob); this helps improve food security.

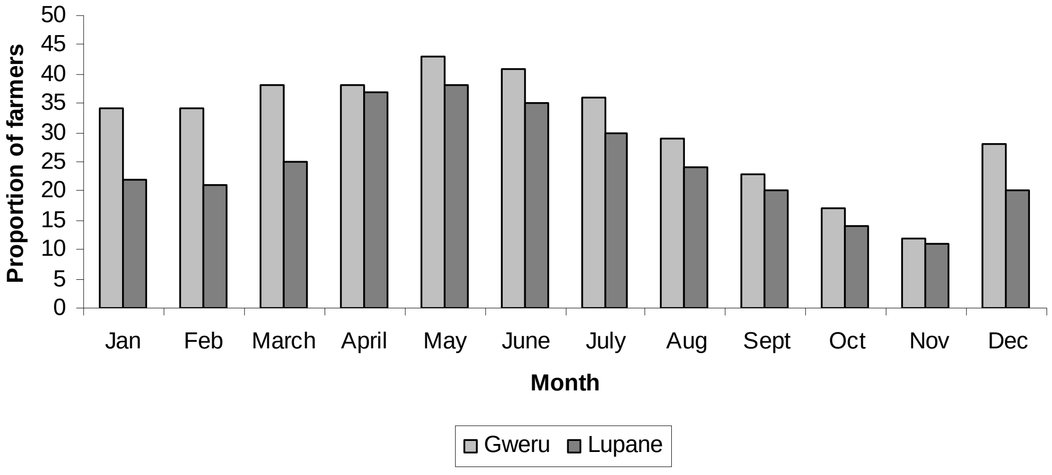

Figure 11: Proportion of farmers having adequate food in each month (2009 Survey)

The chi-squared test was used to test whether there is a difference in the number of food secure households between Gweru and Lupane. From January to December there was a significance difference at 1% level of significance. Thus more households in Gweru were food secure compared to households in Lupane.

4.5. Farmers perception of climate change

In this study, farmers were asked if they had noticed any significant climate changes. Results showed that, from the sampled households, almost all the farmers had noticed significant changes in weather patterns and they ascribed reduction in farm production to changes in the rainfall pattern (reduction in rainfall received, increased frequency of dry spells and droughts). In Gweru all the households said they had noticed changes in climate and 96.6% from Lupane said the same. Thus farmers form both Gweru and Lupane indicate that there has been change in the local climate.

The farmers were also asked to list all weather changes that they had noticed over the past ten years, and the frequency these weather changes had occurred over the past ten years. A farmer would mention more than one weather change and the frequency of occurrence. The frequency of concurrence was averaged for all famers and the percentage of farmers who mentioned the weather change was captured as shown on table 14. Some of the farmers in Gweru (27.5%) and Lupane (24.9%) mentioned that there was increased occurrence of rainfall starting late and ending early. Farmers in Lupane (31.2%) also mentioned increased frequency of drought as a change that was now frequent in their locality and over the past decade, they had experienced about 5 droughts. Rainfall starting early and ending late was the least mentioned by farmers from both districts. In Gweru only 8.1% and 7.3% in Lupane of households had noticed excessive rains.

Table 14: Farmers' perceptions of trends in weather patterns (2009 Survey)

Weather pattern changes	Proportion of farmers who have noticed changes (%)		Frequency of this changes over the past 10 years		Farmers attributing impact of weather changes, to be crop reduction (%)	
	Gweru	Lupane	Gweru	Lupane	Gweru	Lupane
Increased frequency of drought	23.2	31.2	5.0	4.7	77.6	76.6
Rainfall starts late and ends early	27.5	24.9	4.8	4.1	84.6	78.4
Extreme temperatures	11.8	8.8	5.4	4.2	84.0	72.2
Long dry season during cropping season	25.1	24.9	4.1	3.5	86.8	78.4
Excessive rains	8.1	7.3	2.4	1.7	70.6	40
Rainfall starts early and ends late	0.9	1.0	2.5	3.5	100	50

Farmers were also asked to highlight the impact of climate change on their livelihoods. Results of farmers who mentioned crop reduction as an impact of climate change are also shown in Table 15 and it was the most commonly mentioned impact of climate change. 77.6% of the households in Gweru and 76.6% in Lupane mentioned that increased frequency of droughts was resulting in crop reduction. Other households mentioned increased livestock and human diseases as the impacts of weather changes.

During the FGDs farmers were asked to list constraints faced in their community and create criteria for ranking the constraints by importance for a number of outcomes (food security, income generation and general improvement in livelihood). Table 15 and 16 shows results from the FGD carried out in Lupane, Mdubiwa ward. The importance of each constraint was scored on a scale of 1 – 20. Higher scores were assigned to the factors deemed to be of high importance. The total score for each factor was calculated and this was used to arrive at a

ranking for the identified constraining factors on a scale of 1-5, with a rank of 5 assigned to the least important factor.

Table 15: Mdubiwa ward men matrix ranking of constraints

Constraints	Improved livelihoods	Food security	Income generation	Total	Rank
Lack of draught power	12	14	12	38	5
Erratic rains, drought	20	20	20	60	1
Late supply of inputs	16	16	14	46	3
Shortage of drugs in clinics	18	18	18	54	2
Bad roads	14	14	16	44	4

Men were more concerned that crop farming being mostly rain fed, erratic rains and drought were a major hindering factor in production. Women identified the same factors as a major issue.

Table 16: Mdubiwa ward women matrix ranking of constraints

Constraints	Improved livelihoods	Food security	Income generation	Total	Rank
Lack of draught power	12	10	18	40	2
Erratic rains, drought	16	20	20	56	1
Late supply of inputs	10	12	16	38	3
Shortage of drugs in clinics	6	2	2	10	5
Bad roads	14	6	14	34	4

4.6. Household level adaptation and coping strategies

Smallholder systems are often characterized by livelihoods that have evolved to reduce overall vulnerability to external shocks. Changes in climate have prompted farmers to reexamine land use and management practices. This section reviews coping and adaptation strategies for farmers in Gweru and Lupane districts in response to changes in climate. The first part will outline adaptive strategies found in Gweru and Lupane and then the coping strategies.

Conservation farming has been linked with addressing climate change since it improves water availability for crops by conserving soil water and also conserves the soil structure thus reduces erosion (Dumanski *et al*, 2006). However few farmers have adopted the technology in both Gweru and Lupane districts with 16% and 17% respectively of the households practicing conservation farming.

Growing drought tolerant crops is another adaptive strategy practiced by farmers in Gweru and Lupane district. These include crops such as millet, sorghum and cowpeas. The farmers mentioned that due to increased frequency of droughts and dry spells, planting drought tolerant crops is common among the households (Table 17). More than 60% of the households plant drought tolerant crops, with 79.5% in Gweru and 60.5% in Lupane. In addition, planting drought tolerant crops is the most common adaptive strategy among the two districts. However, women from Gweru and Lupane said that small grain crops such as millet and sorghum are hard to process compared to maize. Millet and sorghum are processed manually whereas maize can be taken to the mechanical grinding mills for processing. Women prefer maize to the other cereals and smaller pieces of land are allocated for millet and sorghum compared to larger pieces of land allocated for maize. Thus the preference of individual farmers can also determine the extent of adopting a technology.

Table 17: Adaptive strategies adopted by farmers in Gweru and Lupane districts (2009 Survey)

**Adaptive strategies**	**Districts**		**Chi square test**	
	**Gweru (%)**	**Lupane (%)**	**chi value**	**p value**
Conservation farming	16.1	15.8	0.018	0.892
Ripping	4.3	5.1	0.820	0.775
Use of crop residues	21.4	28.2	2.003	0.157
Using drought tolerant crops	79.5	60.5	7.557***	0.006
Crop Rotation	30.9	26.4	0.757	0.384
Intercropping	11.3	35.1	28.516***	0.000

Significant at:
*** 1% level of significance
** 5% level of significance
* 10% level of significance

Unlike adaptive strategies, coping strategies are short term measures implemented after the shock has already occurred, whereas adaptive measures are more of preventive measures done before and are long term. A coping strategy employed by most households include reducing the number of meals per day, this means that is if a household normally has three meals a day, they reduce to two or less meals a day. The other popular strategy found among households in Gweru and Lupane was reducing the amount eaten and this entails reducing the quantities consumed per meal. More than 80% of the farmers mentioned that they reduced amount eaten, that is reducing and ate fewer meals a day (Table 18).

Farmers in Lupane obtained relief from NGOs and 77% of the households reported using this coping mechanism compared to the 44% in Gweru. Lupane also has 51% of farmers who sell livestock as a coping strategy compared to the 32% in Gweru. The difference is significant at 1% level of significance.

Table 18: Coping strategies adopted by farmers in Gweru and Lupane (2009 Survey)

**Coping Strategy**	**Districts**		**Chi square test**	
	**Gweru (%)**	**Lupane (%)**	**chi value**	**p value**
Sold livestock	32.1	51.4	13.902***	0.001
Sold household assets	12.8	8.2	2.219	0.136
Consumed seed stock	17.9	35.4	12.496***	0.000
Ate food that we normally don’t eat	63.2	58.1	1.619	0.445
Reduced amount of food eaten	83.3	87.7	1.737	0.188
Ate fewer meals per day	84.6	89.2	1.112	0.292
Sought daily work for cash outside the farm	36.1	38.4	0.269	0.604
Migrated	26.3	15.2	5.269	0.022
Borrowed cash	42.1	50.4	2.504	0.114
Borrowed food	45.6	66.7	18.983***	0.000
Worked in other people’s farms for food	37.2	43.4	1.356	0.244
Sold firewood	14.1	7.3	4.702**	0.030
Leasing out land	2.2	2.9	0.535	0.465
Withdrew children from school	25.1	25.9	0.920	0.761
Obtained relief	41.1	77.2	47.542***	0.000

Significant at:
*** 1% level of significance** 5% level of significance* 10% level of significance

Leasing out land was the least adopted coping mechanism in Gweru and Lupane district. This can be attributed to the laws governing land use in the rural areas. The chiefs allocate the land to specific households and they are not allowed to sell or lease out the land.

4.7. Zimbabwe's policies for address climate change

Zimbabwean policy makers appreciate the existence of climate change and its potential impacts on the general welfare of the country. As mentioned before, the country was one of the first to sign and ratify the UN Conversion Framework on Climate Change in 1992 (Zhakata *et al*, 2004). A climate change office was then set up in Harare to coordinate climate change issues. However the country has no specific harmonized climate change policies that address climate change issues. Climate change as an issue is imbedded in various other policies such as the Water Act established in 1997 and Agriculture Act and Environment Management Act (EMA) established in 2003 (Zhakata *et al*, 2004). Not having climate change specific policies in the country has resulted in little progress on addressing climate issues within the country. Gumbo (2006) highlighted that the lack of progress in addressing climate change within the country has been exacerbated by unclear policies and strategies, weak legislation and institutional arrangements, and to some extent political interference.

Research on understanding climate change in the region is hampered by lack of synthesized data on critical variables such as temperature and rainfall records. Schulze *et al* (2001) suggests that Southern Africa has so much data collected over time by different institutions that needs to be synthesized and stored for future use. Thus a data base for climate change information can be created where information can be synthesized and stored for future use. Climate change research is at its preliminary stages globally thus a lot is still to be done and Zimbabwe is no exception. Policy makers and other stakeholders involved have to be well

informed so that they can develop effective policies that address climate change and its projected impacts.

4.8. Institutional arrangements in Gweru and Lupane districts

Institutions influence the ability to mitigate, cope and adapt to climate change within communities. According to Agrawal *et al* (2008), success of past adaption practices among the rural poor depends crucially on the nature of the formal and informal local institutions.

Representatives of organizations located within the communities of Gweru and Lupane were interviewed. This included organizations such as Zimbabwe Meteorology Services, Agricultural Technical and Extension Service (AGRITEX), health sector, education sector, NGOs and traditional leadership. The idea was to find out the core business of the organization within the community and how its activities are relevant in helping households deal with climate change.

Local organizations have a role to play in reducing the community's vulnerability to climate change. The representatives from these organizations make up the elite group of the community which includes teachers, priests, nurses and the police. Thus understanding how these organizations operate is likely to help find ways to make the organizations more effective in facilitating adaption and reduce vulnerability of farmers to climate change.

In general, all the representatives of organizations interviewed had noticed changes in the climate. They indicated that there was increased frequency of extreme weather conditions such as droughts and prolonged dry spells. The following section reviews information provided by different organizations in Gweru and Lupane.

4.8.1. Agricultural Technical and Extension Services (AGRITEX)

AGRITEX is a department in the Ministry of Agriculture which provided extension advice to farmers and has extension officers stationed within the communities. The extension officers train farmers on agriculture related courses, facilitate seed fairs and field days; such platforms are used to empower farmers on information that is relevant for agriculture, specific to their areas. This includes use of improved varieties, improved technologies such as conservation farming and also changes that are happening to their environment and how best they can be addressed. Farmers can also use the opportunity to address individual management challenges in production with the help of the extension officer. Although this is the case, the extension officers' coverage is inhibited by inadequate resources, for example, transport to visit farmers and the operational structure of AGRITEX is such that one extension officer is assigned one ward. Results from the survey show that from the 40% of farmers reported to have access to weather information, 27% of those with access to information mentioned AGRITEX as their source of information.

The other role that extension officers have within the community is to link the farmers with the suitable organizations that work with farmers such as seed and fertilizer companies; researchers and buyers of products. In both Gweru and Lupane districts, AGRITEX collaborates NGO agriculture activities by offering extension services; this encompasses projects such as establishment and management of nutritional gardens and use of improved farming technologies such as conservation farming.

During the interviews with the local extension officers, they highlighted that they did not have information about climate change to enable them to help farmers address climate change

impacts. The extension officers mentioned that they advice farmers to use improved technologies such as staggered planting, drought resistant crops, early maturing varieties and dry planting.

4.8.2. Zimbabwe Meteorology Services Department

This is a government funded organization that deals with weather recording, forecasting and provision of information for aviation. The organization has a department that addresses agriculture activities: Agricultural Meteorology (Agro-Met). The Agro-Met department provides seasonal and weekly forecasts that help farmers plan for their season and also short term activities using the weekly forecast. Agro-Met works with the AGRITEX who offer extension services to farmers in different areas. The Zimbabwe Meteorology Services Department is also a centre for information for veld fires and construction of fire guards.

Agro-met disseminates its information through the media using farmer programs and the daily news. The programs are aired on the radios, television, newspapers and farmer magazines, thus only farmers with access to these different forms of media have access to the information. The rural farmers are at a disadvantage considering that on average only 34% of farmers had radios and TVs. In addition coverage of the local transmission is relatively poor in the rural areas of Gweru and Lupane. An average farmer is unable to afford to buy a newspaper even once a week.

During FGDs farmers reported that the information obtained from the Zimbabwe Meteorology Services Department was not informative and farmers were not confident in using the forecasts to inform their planning.

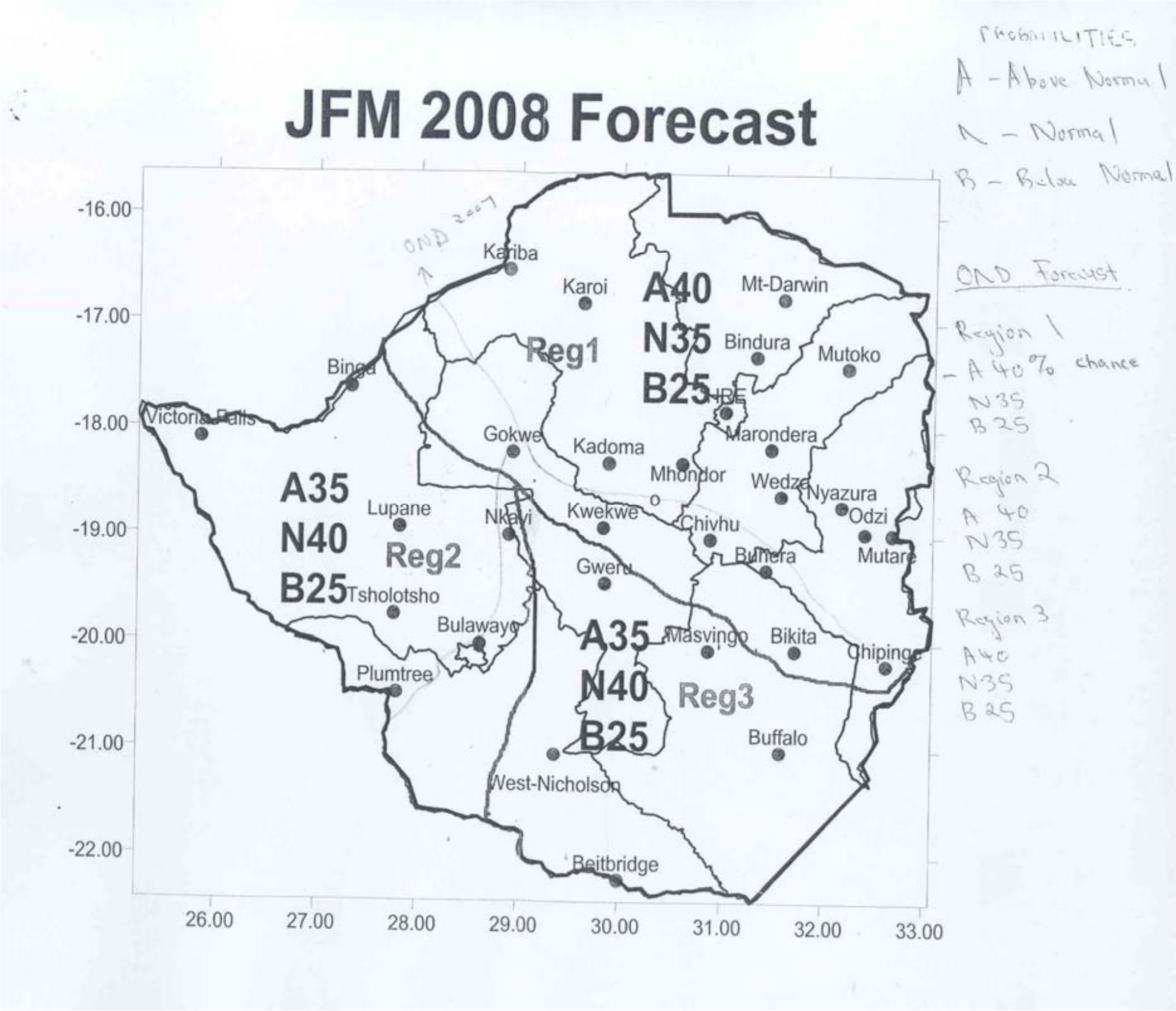

Figure 12: Rainfall forecast for January-March 2008: as received by Meteorology Lupane Officer
Source: Zimbabwe Meteorology Services Department, Government of Zimbabwe, Harare, Zimbabwe

Agro-Met asserts that farmers do not understand fully the terminology used in explaining the weather forecast, that is, the information is probabilistic. The information in figure 12 was received by a lower level officer in Lupane, it shows forecasts for January, February and March, and the information is aggregated. The Zimbabwe Meteorology Services Department divides Zimbabwe into three regions, Region 1 to 3. The information shows that in region one which covers a third of the country, for the three months (January –March), there will be 40% probability that rainfall will be above normal, 35% chance that it will be normal and a 25% chance that it will be below normal. In conclusion the forecast shows that the chances are 75% that rainfall for January to March will be above normal to normal. This information

is too generalized and does not tell the farmer much. The farmers need to know when the rains will start so that they can plan on when and what to plant. The farmer also needs to know if there will be dry spells within the season, when the rainfall season is likely to end and the quantity of rainfall to be expected.

The representative interviewed mentioned that farmers would ideally make use of the weekly forecasts to help them plan for their farming activities but the Zimbabwe Meteorology Services Department does not have the capacity to disseminate information to the rural farmers.

4.8.3. Traditional Leadership

The traditional leadership constitutes of chiefs and headmen. The traditional leaders are responsible for allocating land and enforcing customary laws and norms related to the use and conservation of natural resources. This includes not cutting down certain trees (mostly protected trees like the Teak), stream bank cultivation, gold panning and certain water sources such as wells. The traditional leaders believe that the abuse of natural resources by people is causing climate change.

Traditional leaders indicated that there has been change in the climate. Changes that were mentioned by the traditional leaders from Gweru and Lupane include; increased occurrence of dry spells, droughts and late onset of rainy season that has made it difficult for farmers to plan. Farmers used to rely on traditional forecasting, which included observing how nature behaves such as local trees, insects and movement of winds. For example if the mango tree produces many fruits, it means that there is going to be drought that same year. According to

the traditional leaders, traditional forecasting has not been reliable because results obtained haven't been consistent with what they know.

Traditional leaders also attributed climate change to the diversity of cultural beliefs currently present among smallholder farmers in Gweru and Lupane. The diversity of cultural beliefs has brewed social unrest among the communities and according to traditional leaders, making it difficult for unified climate change strategies to be taken up by farmers. About 31% of the interviewed households attributed changes in climate to the diversity of cultural beliefs.

Traditional leaders suggested that the community should set aside differences that were causing social unrest and work towards achieving adaptive strategies that would mitigate climate change. They also mentioned that farmers should plant more of drought resistant crops such as sorghum, millet and cowpeas.

4.8.4. Non Governmental Organizations (NGOs)

There are several NGOs operating in both Gweru and Lupane districts. Their activities range from food relief to development of the communities. Some the projects include nutritional gardens for farmers (Figure 13), building projects at schools and supplying materials for hospitals.

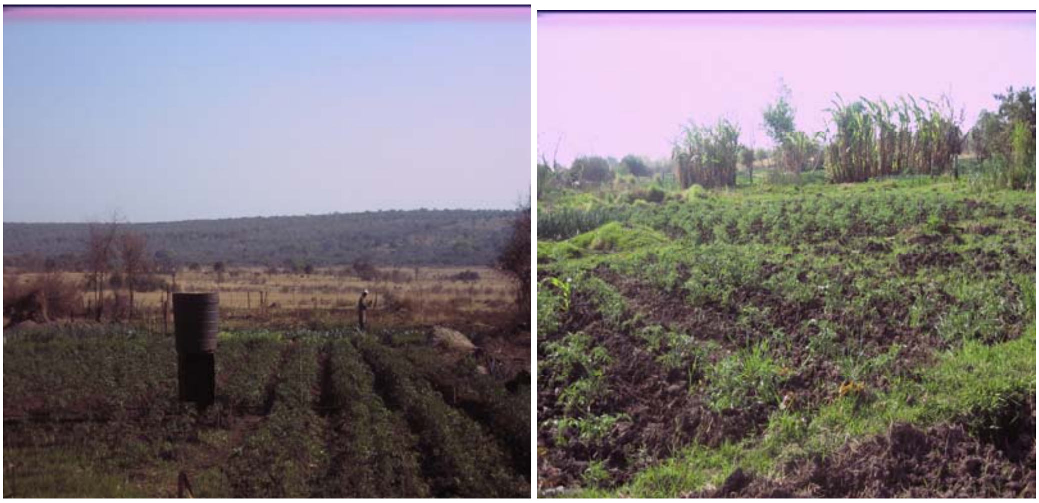

Figure 13: World Vision funded community nutrition garden in Lupane, Daluka ward along the Bubi Valley (2009 Survey)

The NGOs also offer training to the farmers in agriculture related activities, craft making, baking, candle making and dressmaking. The survey shows that an average of 47% farmers had received training. NGOs work with government departments such as AGRITEX, Veterinary department, Health and Education among others. They use expertise from these departments to run the various projects, for example the NGOs work with AGRITEX for agriculture related projects.

In addressing changing climate for dry areas like Gweru and Lupane, the NGOs representatives mentioned that they encourage farmers to use technologies such as conservation farming; use of drought resistant crops such as cowpeas, pearl millet and sorghum; nutritional gardens. They also mentioned that diversifying out of agriculture through other nonfarm projects, would help farmers mitigate against climate change.

4.8.5. Education Sector

In both Gweru and Lupane villages selected for the study, there were primary schools and secondary schools. The schools are responsible for educating children on various subjects

including environment studies such as natural resource conservation. The schools are also a centre of communication for the community. They raise awareness of disasters that might be fall the community such as outbreak of diseases and droughts by telling children to pass the message to the parents.

The local school heads highlighted that they had limited information on the climate change subject to enable them to educate children and the community so that they reduce vulnerability to climate change. Educating children on the subject would empower communities with knowledge about the phenomenon thus reducing vulnerability to climate change.

4.8.6. Health Sector

The health sector is responsible for prevention, cure, control of diseases and raising awareness among the communities.

The sister in charge at St Luke hospital in Lupane reported that, 'malnutrition cases especially for young children were on the rise due to poor crop yields', thus the households were unable to meet the nutrition requirements of the family especially children who are often sensitive to lack of a balanced diet. In addition due to deteriorated livelihoods because of increased frequency of drought, abuse cases especially of children were also been reported (Figure 14).

Gweru and Lupane residents have also experienced increased cases of malaria (Mubaya *et al*, 2010). The hospitals in the rural areas distribute treated mosquito nets, help with larviciding (i.e. destroying mosquito breeding places) the area, spraying and encouraging local retail

shops to sell mosquito coils. They also work with NGOs such as World Vision, Christian Care, Red Cross, UNICEF and Alliance Church of Zimbabwe.

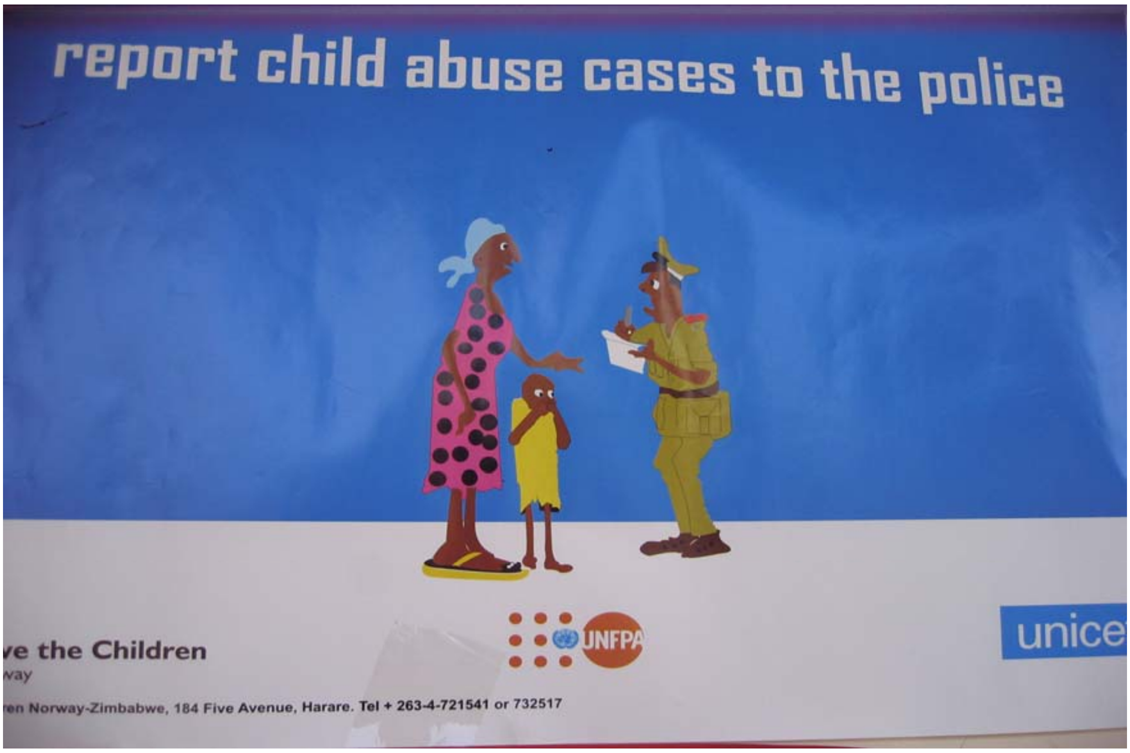

Figure 14: A poster at Lower Gweru clinic aimed at raising awareness about child abuse (2009 Survey)

4.9. Farmers perception on organizations operating in their communities

Local organizations have a role in aiding or reducing vulnerability of farmers to climate change. Robust institutional arrangements are more likely to reduce vulnerability of farmers to climate change. Some organizations found in Gweru and Lupane district may not directly address climate change, but their existence and services may indirectly alleviate vulnerability to climate change for the community. An example can be the veterinary services; with occurrence of climate change in Gweru and Lupane which is predicted to experience frequency of droughts, this might cause increase in livestock diseases. The veterinary services can help farmers combat the livestock diseases, thus reducing vulnerability to climate change.

The roads department can also reconstruct bridges that may be destroyed by floods thus addressing impacts of climate change.

During the FGDs farmers were asked to list all the organizations working within their community and whether they thought that these organizations addressed climate change through their services (Table 20). The idea was to find out farmers perceptions on how the existing organizations would help address climate change.

The participants mentioned only three organisations that they felt addressed the issue of climate change. These include AGRITEX, traditional leadership and seed houses (Pannar and SeedCo). AGRITEX helps them with extension services that address climate change issues like planting dates and types of crop suitable for the climate. Planting dates suitable for the areas are given to the farmers and they are encouraged to plant early to coincide with the first rains. Extension officers often encourage farmers to plant drought resistant crops such as millet, sorghum and cowpeas (small grain crops). Traditional leaders helped the community by enforcing laws that help conserve natural resources. Farmers also felt that seed houses addresses climate change by breeding varieties suitable for the different areas and climates.

Table 19: Organizations working with farmers in Gweru and their role in addressing climate change impacts (2009 Survey)

Institutions	Major activities	Farmer perceptions about whether they thought organisations helped them address impacts of climate change
Red Cross	Assist orphans and HIV and AIDS patients with food, fees, clothes, agric inputs, tablets, fruit trees for planting, herbs. Create awareness on HIV and AIDS	×
AGRITEX	Training-demonstrations, field days and shows, ploughing contests	•
Doctors without Borders	ARVs, Counselling and Testing, Health education, food for children	×
Traditional Leaders	Lead in traditional ceremonies such as rain making ceremonies, maintaining traditional norms, counselling and land allocation	•
BEAM	School fees for disadvantaged children	×
German Aid	Livestock dipping, CF, sweet potato seed, plantations (gum), fencing	×
ORAP	Agric inputs, treadle pumps, drip kits, CF, school stationery	×
Christian Care	Treadle pumps, garden seed, training	×
Church of God	Toilets, borehole repairs	×
Maguta	Seed through AGRITEX, tillage (tractors), fertilisers	×
Veterinary services	Supervise and diagnose livestock diseases, dipping through German Aid	×
Schools	Imparting knowledge	×
Department of Gender	Coordinate/media between government and the community	×
Health	Treatment, water treatment, well construction, awareness on general health, supervise toilet construction,	×
Seed Houses (PANNAR, Seed Coop)	Sell seed through AGRITEX, deliver seed	•
Local authority (Councillors)	Relief food from government	×
DDF	Road maintenance	×

Key

- × No
- • Yes

4.10. Summary of findings

The main focus of the chapter was to have a clear understanding of households in Gweru and Lupane. From the results obtained, in terms of household characteristics, there was not much difference. In addition, livelihood activities were also reviewed and households in both Gweru and Lupane depend on agriculture for their livelihoods. However, changes in climate experienced affected the main source of livelihoods for smallholder farmers in these districts. The cereal yields obtained by the households are generally low. Although this is the case, farmers have strategies in place to minimize the effects of climate change. These involve coping and adaptive strategies. From the results obtained; farmers are practicing more coping than adapting strategies. This can be a result of lack of adequate knowledge about climate change. There is need for farmers to make use of adaptive strategies to minimize loss and improve household food security.

Information from representatives of organizations within the communities was also discussed in this chapter. The representatives interviewed had noticed changes in climate but there is little information about the subject for them to act on. At global and national level, information is available and circulates at global level; there is need for the information to trickle down to the local organizations that then can raise awareness among the smallholder farmers. The Zimbabwe Meteorology Services Department is a key player on climate change but currently they do not have the capacity to work efficiently.

CHAPTER 5

Measuring Household Vulnerability

5.1. Introduction

The assessment of vulnerability to climate change is defined by the exposure and sensitivity of a system to hazardous conditions and the ability or capacity or resilience of the system to cope, adapt or recover from the effects of these conditions (Smit and Wandel, 2006). Zimbabwe and larger regions of the SSA (Sub Saharan Africa) is characterized by the low adaptive capacity in addition to a high degree of exposure and sensitivity to climate change.

Zimbabwe's agriculture sector is sensitive to climate change, variability and extreme events, particularly increased frequency of droughts and mid season dry spells. In many areas, poor soil fertility and inadequate resources have exacerbated low productivity thus affecting the food security status of the households. According to Muamba and Kraybill (2010), some of the most profound and direct impacts of climate change will be on agriculture and food security.

The welfare and livelihoods of smallholder farmers in Gweru and Lupane are agriculture-based with cereal production being predominant within the two districts. Cereals production contributes to household food security status and the cereals are maize, sorghum and millet. In addition, households largely rely on own production and have limited capabilities for purchasing food. If production is low, this often leads to the household becoming food insecure.

Climate change will pose particular challenges for food production among households. Production conditions are already less ideal considering the marginal location (NR IV) of the study sites; characterized by erratic rainfall and poor soils. Climate change will worsen the situation with climate predictions stating that the areas will receive lower rainfall, increased frequency of droughts, dry spells, erratic distribution of rainfall and poorer soils. This will have negative implications on crop production considering that the majority of the households practice rain-fed agriculture, making them susceptible and vulnerable to negative changes in climate. In addition to farmers in Gweru and Lupane having their livelihoods dependent on rain-fed agriculture, the communities also have some of the households being found among the poor. Poor households are more likely to be vulnerable to climate change impacts. They often have low production levels and are often food insecure, with the existence of climate change, the poor households' situation is likely to worsen rather than improve.

The study adopts Chaudhuri *et al* (2002) model that measures vulnerability to poverty, to measure vulnerability to food insecurity. Climate change in Gweru and Lupane will negatively affect the production levels of the households thus reducing the food security status of the households. Households that are found to be vulnerable to food insecurity are more likely to be vulnerable to climate change.

5.2. Vulnerability of households to food insecurity[9]

Vulnerability is a function of hazards or risks people are facing, their ability to cope or manage and if the hazard unfolds; there is need to estimate the level of the hazard's impact on the household's livelihood. The study measures the vulnerability to food insecurity, looking at how households' own production levels interact with household characteristics. Thus the magnitude of vulnerability to food insecurity for each household is determined and how each household characteristic influences the household's cereal produced by the household. Chaudhuri's model was used and the model goes beyond cataloging who is currently food insecure and who is not, to an assessment of household's vulnerability to food insecurity. The study broadens the food insecurity assessment taking into account vulnerability to food insecurity and links this to vulnerability to climate change.

As mentioned earlier, a household requires about 165kg of cereal per capita, thus a household that has equal to or above the 165kg per capita cereal available for the household has adequate quantities to ensure the food security status of the household. According to Chaudhuri *et al* (2002), a household's vulnerability (in this case to food insecurity) can be expressed as a probability statement reflecting the household's inability to attain a certain minimum level of cereal requirements (165kg of cereal per capita) in the future. Consider a household h, the probability that such a household is vulnerable to food insecurity is expressed as:

$V_{ht} = \Pr\left(c_{h,t+1} \leq z\right)$, see equation 1 Chapter 2

[9] This section draws extensively on the work of Chaudhuri *et al* (2002) and the full model is shown in chapter 2 section 2.6

Where V_{ht} is the probability value associated with the household's vulnerability at time *t*, $C_{h,(h+1)}$ measures the household's per capita cereal production at time $t+1$ (future) and *z* is an appropriate household cereal requirement or benchmark (threshold level).

The per capita cereal produced by a household at any time period will generally depend on a number of factors. These factors include income levels, demographic characteristics of the household, weather and extension (knowledge of farmer). Following Chaudhuri *et al* (2002), the stochastic process generating the cereal production of a household *h* is expressed as:

$\ln c_h = X_h\beta + \varepsilon_h$, see equation 4 Chapter 2

Where:

c_h = the household per capita cereal produced

X_h = household characteristics, assets and other risk management instruments,

β = vector of parameters to be estimated,

ε_h = is a mean-zero disturbance term that captures idiosyncratic factors (shocks) that contribute to different per-capita cereal production levels for the households that are otherwise observationally equivalent.

The variance of the regression depends on the explanatory variables in the regression. Thus:

$\sigma^2_{\varepsilon,h} = X_h\theta$, see equation 5 Chapter 2

β and θ are estimated using a three-step feasible generalized least square (FGLS) procedure suggested by Amemiya (1977). A statistical package known as SPSS was used for the analysis and the Two Stage Least Square Regression was used to calculate estimates of $\hat{\beta}$

and $\hat{\theta}$. Results from two stage least square regression model are presented in Table 20. These variables are determinants influencing a household's production level.

The first regression results show a production function where productivity is regressed against household characteristics. The coefficient of β shows how each variable is related to cereal production levels of the household (equation 4).

The gender of the household head influences the level of cereal produced by the household and is significant at 10% level of significance. This means male headship improves the cereals produced by the household and female headship reduces the quantity of cereal produced by the household. The female headed household mainly constitutes of widows and single parents, who have to take responsibility for the household on their own. Female headship thus increases vulnerability of the household to food insecurity. This is supported by Horrel and Krishnam (2006), on a study done in Zimbabwe, on Poverty and Productivity in Female-headed households in Zimbabwe. The study concludes that female headed households in rural Zimbabwe are found among the poor and lack income and resources that constrain their productivity. The study finds that the women often lack assets, particularly assets needed for agriculture production, constraining their ability to diversify the crops grown and area allocated for the crops is often small compared to man headed households.

Table 20: Determinants of cereal production for households in Gweru and Lupane (2009 Survey)

Variables	$\hat{\beta}$ coefficient	T statistic value	Level of significance	$\hat{\theta}$ coefficient	T statistic value	Level of significance
Constant	3.429	2.581	0.011***	-0.820	-0.522	0.602
gender of household head	0.290	1.935	0.055*	0.106	0.597	0.551
Age of household head	-0.291	-0.782	0.435	0.454	1.033	0.303
Education of household head	0.149	1.358	0.176	0.053	0.407	0.685
Farming experience of household head	0.325	2.479	0.014***	-0.033	-0.215	0.830
Presence of chronically ill people	-0.435	-2.39	0.018**	-0.459	-2.139	0.034**
Member of a local social group	0.337	2.18	0.031**	-0.204	-1.118	0.265
Annual household income	0.081	1.76	0.080*	0.096	1.775	0.078*
Livestock	0.084	2.014	0.046**	-0.026	-0.532	0.596
Physical assets	-0.001	-0.005	0.996	-0.107	-1.132	0.259
Extension frequency	-0.136	-1.309	0.193	-0.108	-0.881	0.380
Access to weather information	0.310	1.512	0.132	0.014	0.057	0.955
Hired labor	0.391	2.579***	0.011***	-0.160	-0.895	0.372
Total land owned	0.182	1.646	0.102	0.249	1.911*	0.058*
Family labor	-0.456	-2.830***	0.005***	-0.087	-0.458	0.647

Significance at:
* 10% level of significance
** 5% level of significance
*** 1% level of significance

Results from the model show that increase in age of the household head decreases the cereal productivity of the household (Table 20). However, the relationship is not significant. This is contrary to the results found by Bogale and Shimelis (2009). Their study was looking at

Household level determinants of food insecurity in rural areas of Dire Dawa, Eastern Ethiopia. The study concluded that with increase in age of the household head, there is decrease in the likelihood of the household becoming food insecure. In addition the study concluded that older household heads for rural households acquire more experience in farming operations, accumulate wealth, use better planning and have better chances of becoming food secure. Since the results from the model are not significant, the results from the model are not conclusive, showing that the age of the household does not contribute significantly to the level of cereal produced by the household.

The education level of the household is positively correlated to the cereal produced by the household. This means that increase in the number of years spent in school by the household head increase the quantity of cereal produced by the household. However, the relationship is not significant, showing that in this particular model the level of education for the household head does not contribute significantly to cereal productivity. This can be attributed to the fact that most household heads from Gweru and Lupane were literate as shown in chapter 4. Results from the survey, show that farmers in Gweru and Lupane district are literate with only 15% of the household heads lacking any form of education.

The farming experience was also included in the analysis and results show that increase in farming experience increases the cereal produced by the household. The relationship between farming experience and cereal productivity is significant at 1% level of significance. Farming experience of the household head is likely to improve efficiency as the farmers learns from his mistakes and improves over the years, increasing productivity.

The presence of a chronically ill person also influences cereal production. The results show that the presence of a chronically ill person reduces the cereal production level of the household and this is significant at 5% level of significance. A chronically ill person is likely to consume the household financial reserves and other resources to meet health expenses. In addition, when a household has an ill person, productivity is also compromised; labor has to be divided among taking care of patient and working to boost productivity of the household.

Whether the household head is a member of a social group was also incorporated in the model. If a household head is a member of a social group, the cereal production level of the household is also increased and this is significant at 5% level of significance. Social groups are a source of information within communities and being part of such groups helps farmers learn specific skills, share experiences and inform each other, thus improving efficiency in their productivity.

Household income influences cereal productivity and the relationship is significant at 10% level of significance. Increase in the household income increases the cereal production level of the household. Income from the household is often used to buy inputs such as improved seed varieties and fertilizer that increase production levels of the household. In addition, households that have access to better income opportunities are less likely to become food insecure than households who have less or little access (Bogale and Shimelis, (2009).

The livestock owned by the household increases the cereal production levels and this is significant at 5% level of significance. Livestock is a vital input into crop production and influences production positively. Livestock such as cattle and donkeys are used as draft power. A household with draft power is more likely to perform farming operations effectively

than a household without draft power. Livestock also contributes manure which most smallholder farmers use to increase fertility of their soils. In addition, ownership of livestock acts as a hedge against food insecurity and serves to accumulate wealth that can be disposed during times of need (Bogale and Shimelis, 2009).

Access to extension services and weather forecast information were also analyzed against cereal produced by the household. The results show that the two variables are not significant in explaining the household's cereal production levels. This can be attributed to the quality of services and information available for farmers to improve productivity, especially looking at the conditions that farmers in Gweru and Lupane are experiencing. This includes erratic rainfall, increased frequency of droughts and dry spells. With future climate change this situation will worsen and farmers need services that will help them minimize climate change impacts and ensure food security of the household.

The ability of a household to hire labor increases the level of cereal produced by the household and this is significant at 1% level of significance. A household that affords to hire additional labor is more likely to be resource endowed thus has extra resources to hire labor and increase household productivity.

Results also show that increase in household labor is negatively correlated to the quantity of cereals produced by the household and this is significant at 1% level of significance. This means that increase in household labor reduces the quantity of cereal the household produces. This is contrary to what is expected because in a production function, increase in an input (in this case labor) increases productivity. This might mean there are other factors in play influencing productivity, which is not labor or the quality of household labor could be an

issue. Labor for this study was calculated as a total of the household members contributing full time labor and half of members contributing part time labor. The household would identify the members that contributed full time, part time and those that did not contribute to household labor. The age group that contributed most of the full time labor was the age group between 18-64 years and part time labor was found in the 6 -17 years and above 65 years age group. The age group below 5 years did not contribute to household labor. A study done by Zezza *et al* (2007) on Rural Household Access to Assets and Agrarian Institutions (cross country comparison: 15 countries) defines the working age group as 15-60 years of age and calculates the rural dependency shares; defined as the number of dependents divided by total household size. For this study; the working group that contributes full time labor is 18 – 64 years age group. The dependency share was also calculated for Lupane and Gweru and was found to be 62.42%. This means that 62.42% rural laborers in Gweru and Lupane have more dependent household members. This compromises the cereal produced by the household considering that there are less people in the household that contribute to full time labor in relation to the dependent members. This compounded with future climate change, will worsen the livelihood for smallholder farmers in Gweru and Lupane considering that they rely on own production to ensure household food security.

After the estimates $\hat{\beta}$ and $\hat{\theta}$ are established using the two stage least squares regression model, following Chaudhuri *et al* (2002), the estimates are then each multiplied by X_h (household characteristics), to obtain $X_h\hat{\beta}$ and $X_h\hat{\theta}$.

$$X_h\hat{\beta} = \hat{\beta}(X_h) \qquad (4)$$

$$X_h\hat{\theta} = \hat{\theta}(X_h) \qquad (5)$$

According to Chaudhuri *et al* (2002) the probability of a household not being able to produce adequate quantities of cereal is given by the formula:

$$\hat{v}_h = \hat{\Pr}\left(\ln c_h < \ln z \middle| X_h\right) = \Phi\left(\frac{\ln z - X_h\hat{\beta}}{\sqrt{X_h\hat{\theta}}}\right) \tag{6}$$

A household is vulnerable to food insecurity if the estimated probability is equal or greater to 0.5 and if the estimated probability is less than 0.5, the household is less likely to experience food insecurity. Estimates for the likelihood of being food insecure for every household were derived from equation 6. A frequency distribution was constructed (Figure 15) and the distribution of vulnerability among households in Gweru and Lupane districts follows a skewed distribution with mean 0.76, thus the majority of households in both Gweru and Lupane are vulnerable to food insecurity, with most household having a probability of more than 0.5.

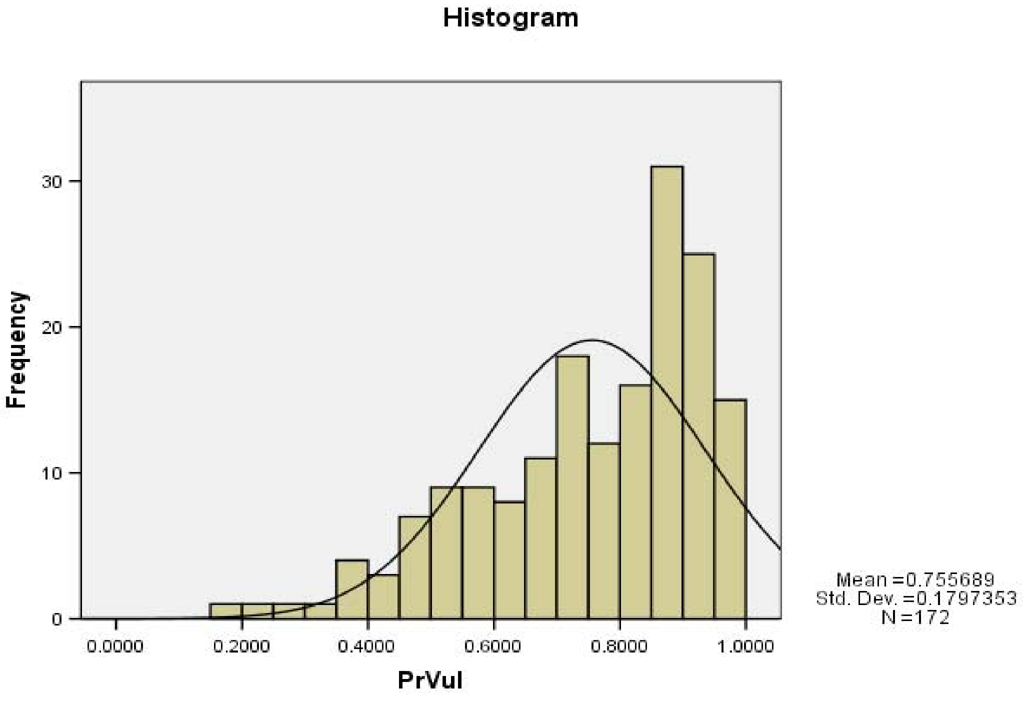

Figure 15: Distribution of household and their vulnerability to food insecurity for Gweru and Lupane (2009)

Based on results obtained from calculating the probability of households being vulnerable to food insecurity, the households were divided into two groups (Table 21), that is those vulnerable to food insecurity and those that are not.

Table 21: Classification of households in Gweru and Lupane districts based on vulnerability status, 2009

Group	Probability	Gweru (%)	Lupane (%)	Proportion of sample (%)
Not vulnerable	0.00 – 0.49	11.8	9.2	10.5
Vulnerable	0.50 – 1.00	88.2	90.8	89.5

From the households sampled in Gweru and Lupane districts, less than 11% of the household were not vulnerable to food insecurity (Table 21). There is not much difference in the vulnerability status of the households between the two districts. As highlighted before, this method goes beyond cataloging who is currently food insecure and who is not, to an assessment of household's vulnerability to food insecurity. This shows that these households are likely to be vulnerable to food security in the future and with future climate change, this will negatively affect the households, considering that they depend on own production to ensure food security. In addition, their production is rain-fed, thus relies on climate and a negative change in climate, for example droughts and reduced rainfall will reduce the productivity of households in Gweru and Lupane districts.

5.3. Summary

This study used a model used to assess vulnerability to poverty to assess vulnerability to food insecurity and links this to climate change vulnerability. Climate change is an external risk that can be conceptualized as a component that contributes to household vulnerability and poverty because it affects the welfare of the household. Households in Gweru and Lupane

depend on own production to secure household food security and the general welfare of the household. More than 80% of the sampled households in Gweru and Lupane districts were found to be vulnerable to food insecurity.

CHAPTER 6

CONCLUSIONS AND RECOMMENDATIONS

6.1. Summary

The overall aim of this research was to assess vulnerability to food insecurity and this was used to infer vulnerability to climate change among smallholder farmers in Gweru and Lupane districts of Zimbabwe. Three research objectives were developed for addressing this aim. These objectives include characterization of the exposures facing households in Gweru and Lupane districts; measuring the cereal availability and requirements of the household; and assessing the likelihood of households experiencing cereal deficit or food insecurity.

The study made use of household questionnaires, FGDs and key informant interviews (KII) to collect data that was used in the study. The exposures facing households in Gweru and Lupane were identified by looking at the study locations in terms of weather patterns experienced, natural resources available and how these contribute to the livelihoods of the smallholder farmers. A descriptive analysis was conducted characterizing households according to demographic household characteristics such as gender, age, education level of the household head; household assets; income sources; adaptive and coping strategies. Perceptions of both the organizations working with the smallholder farmers and smallholder farmers, on climate change also helped understand how climate change will affect smallholder farmers' livelihoods. Policies in Zimbabwe addressing climate change were also identified and discussed.

The household cereal availability and requirements of the households were assessed by looking at sources of livelihoods for the farmers and also activities contributing the most to the food security status of the household. The cereal available for the household was measured by adding the total quantity of maize, sorghum and millet produced by the household. The yields obtained from each cereal were also calculated and compared to the expected cereal yields in NR IV. Literature was used to determine the cereal requirements of the household to ensure food security.

The last objective was to assess the likelihood of households experiencing cereal deficit or food insecurity. The household's cereal production levels were captured and these were regressed against individual household characteristics. The relationship of each variable in contributing to the quantity of cereal produced was also established and discussed. A model used by Chaudhuri *et al* (2002) to measure vulnerability to poverty was used to measure the probability of a household being vulnerable to food insecurity.

6.2. Conclusion

In Zimbabwe, climate change studies are underway so as to ascertain its impacts on people's livelihoods. This study contributes to understanding impacts of climate change on smallholder farmers by studying communal land farmers from Gweru and Lupane districts. Most vulnerability studies have been done at national and global level. Few studies examine impacts at household level and this study tries to fill the gap. This section presents conclusions derived from the study and this will be done by looking at each objective.

Objective one: *Characterization of exposures facing households in Gweru and Lupane districts*

The study sites are located in the semi arid tropics of Zimbabwe, in NR IV characterized by erratic rainfall, increased frequency of droughts, mid season dry spells and poor soils. With future climate change the situation is likely to worsen, with climate predictions indicating that for the semi-arid tropics, there will be increase in temperatures and reduction in rainfall received (IPCC, 2007). In addition the households residing in these areas derive their livelihood from agriculture, which is climate dependent. The community also has large numbers of poor households characterized by low annual income and lack of resources to improve the welfare of the households. Literature suggests that the poor are more vulnerable to climate change, since they have fewer options to mitigate against the impacts of climate change (Eriksen *et al,* 2008). The communities are also characterized by limited adaptive strategies to the current unfavorable climate being experience by the farmers. Results showed that farmers are more likely to employ coping mechanism than adaption strategies, with more than 80% of the households reducing the amount of food eaten and also the number of meals a day. The most common adaptive strategy mentioned by farmers was use of drought tolerant crops and varieties. Limited availability of adaptive strategies within these communities is of concern because the situation is likely to worsen and they need to have strategies in place to reduce vulnerability to climate change and food insecurity among the households.

The weak institutional arrangements in the country are also of concern when it comes to mitigating climate change impacts. Most organizations in the country have been incapacitated by the economic situation of the country and also skilled personnel have left the country as mentioned by the AGRITEX representatives, leaving young staff without experience. The Zimbabwe Meteorology Services Department does not have the capacity to capture and

disseminate information required by farmers due to lack of equipment. In addition, Zimbabwe does not have clear policies that address climate change, but the subject is embedded in other Polices and Acts like the Water Act. Lack of clear policies on the subject has also hindered progress in addressing climate change issues in the country.

Objective 2: *Measuring cereal availability and requirements of the households*

The households depend on rain-fed agriculture for their livelihoods, with cereal production being common among the households. Any improvement on the food security status of the household depends on a successful rainfall season. With current and future current climate change, this will increase vulnerability to food insecurity.

The cereal yields obtained from the households over a period of four seasons from 2004/05 to 2007/08 showed that the farmers were producing below the yields expected for NR IV. The 2007/08 season had farmers producing the highest yields and the season had been characterized by above average rainfall. This showed how much the production of the farmers was pinned on the amount of rainfall received. The other three seasons 2004/05 to 2006/07, were characterized by below average rainfall, droughts and dry spells.

The cereal requirements of the household were derived from reports on Zimbabwe studies done to assess smallholder farmers' livelihoods. The FAO (2007) report shows that a household requires about 165kg of cereal per capita to be food insecure.

Objective three: *Assessing the likelihood of households experiencing cereal deficit or food insecurity*

The results showed that the quantity of cereal produced is influenced by household characteristics such as gender, age, farming experience of the household head; presence of chronically ill person within the household; whether the household head is a member of a social group; household income; livestock; hired labor and household labor. The relationship of these variables to the quantity of cereal produced was consistent with what was expected and also what other studies have obtained. The only exception was for household labor. Household labor is an input in the production function and an increase in labor is supposed to increase the quantity of output. For this study, an increase in labor results in a decrease in cereal produced by the household. This may be attributed to the fact that there are other variables affecting productivity or the quality of household labor could be an issue. The dependency share (Zezza *et al.*, 2007) was calculated for the households. The results show that about 62% of the household members were dependent members and 38% were economically active, providing full time labor to meet the food requirements for every household member.

Chaudhuri *et al* (2002) model of measuring vulnerability to poverty was adopted for this study. Most econometric vulnerability models need longitudinal data collected over time so as to get the overall trend. This particular model uses cross sectional data which was suitable for this study because only cross sectional data was collected. The model goes beyond cataloging who is currently food insecure and who is not, to an assessment of household's vulnerability to food insecurity and this helps understand how climate change will affect smallholder farmers. Results show that about 88% of the population in Gweru and Lupane is likely to experience food insecurity. Climate change will likely worsen the situation since the livelihoods of most households depend on rain-fed agriculture.

6.3. Recommendations

One of the key findings of this study was that a large proportion of smallholder farmers are vulnerable to food insecurity. This means that these communities will need food aid to minimize vulnerability to food insecurity as a short term strategy and this need to be developed soon. There is also need to develop long term solutions to ensure food security among the households because currently own food production is not sustainable. The communities need to be capacitated through training that can help alleviate vulnerability to food insecurity. Other options could be training on off farm activities thus helping farmers have diverse activities that can help them improve their livelihoods and minimize risk of having only one livelihood source.

The other key finding was that although communities have noticed changes in local climatic conditions, there is not much information to help them mitigate against it. Households predominantly employ coping than adaptive strategies. There is need for information to be availed to the farmers and options regarding technologies that they can use to reduce vulnerability to climate change and improve their livelihoods. Organizations like AGRITEX who work with the farmers should receive capacity building training on climate change so that they in turn can also train the farmers. The Zimbabwe Meteorology Services Department, through Agro-met also has a role to play in disseminating weather forecast information to the farmers. The government and other funding organization need to help the Zimbabwe Meteorology Services Department with funds to carry out their services efficiently to ensure that they can provide farmers with reliable and timely information on weather patterns so that farmers can better plan their activities.

The study focused on the vulnerability of farmers to food insecurity, what the study failed to address was looking at the capabilities of these farmers that help them improve their livelihoods. Assessing the capabilities of the farmers by doing an in-depth assessment of how they are currently adapting to current climate change impacts, would help also inform how best they can deal with future climate change. This information can be used by organizations that work with farmers, thus developing strategies that are suitable and acceptable by the communities.

The weakness of the study was that it was unable to directly incorporate climate in the model for measuring vulnerability to food insecurity. This could have been achieved if the areas of study would be in different agro-ecological regions thus experiencing different weather patterns. The study should have had five different sites to represent each agro-ecological region in Zimbabwe, and then assess vulnerability to food insecurity, directly measuring how climate change affects the food security status of the households.

REFERENCES

ADB *et al.,* (2003): *Poverty and climate change: reducing the vulnerability of the poor through adaptation*", VARG multi development agency paper, United Nations Development Project (UNDP) United Nations, New York. *www.undp.org/energy/povcc.htm.*

Adger, W.N., (1999): *Social vulnerability to climate change and extremes in coastal Vietnam*. World Development 27:249–269.

Agrawal A., McSweeney C., and Perrin N., (2008): *Local institutions and climate change adaptation*. Social Development Notes, Community Driven Development: The social Dimensions of Climate Change No 113. World Bank

Amemiya, T., (1977): *The maximum likelihood estimator and the non-linear three stage least squares estimator in the general nonlinear simultaneous equation model.* Econometrica

Barrett, C B., (1996): *Urban bias in price risk: The geography of food price distributions in low-income economies*. Journal of Development Studies. 32(6):830-49.

Bogale, A, and Shimelis A., (2009): *Household LEVEL determinants of food insecurity in rural areas of Dire Dawa, Eastern Ethiopia*. African Journal of Food, Agriculture, Nutrition and Development. 6ETHI

Brohan, P., Kennedy J.J., Harris I., Tett S.F.B., and Jones P.D., (2006): *Uncertainty estimates in regional and global observed temperature changes*: *a new dataset from 1850*. J. Geophysical Research **111**, D12106, doi:10.1029/2005JD006548

Burton, I., (1996): *The growth of adaptation capacity: practice and policy. In: Adapting to Climate Change: An International Perspective*. Springer-Verlag, New York, NY, USA, pp. 55-67.

Burton, I., Huq, S., Lim, B., Pilifosova, O., Schipper, E.L., (2002): *From impact assessment to adaptation priorities: the shaping of adaptation policy*. Climate Policy 2:145–149.

Chaudhuri, S., Jalan J., and Suryahadi A., (2002): *Assessing household vulnerability to poverty: A methodology and estimates for Indonesia*. Department of Economics Discussion Paper 0102-52. New York: Columbia University

Chigwada, J., (2005): *Climate proofing infrastructure and diversifying livelihoods in Zimbabwe*. IDS Bull, **36**, S103-S116.

Christiaensen, J., and Subbarao K., (2004): *Toward an understanding of household vulnerability in rural Kenya*. World Bank Policy Research Working Paper 3326. Washington, DC: World Bank.

Cottem W., (2007): *Drought and food crisis in Zimbabwe (afrol)*. Blog at Word press.com

Cousins B., (1996): *Livestock production and common property struggles in South Africa's agrarian reform*. Journal of Peasant Studies 23 (1996), pp. 166–208.

Deressa T., Hassan R.M., Alemu T., Yesuf M., Ringler C., (2008): *Analyzing the determinants of farmers' choice of adaptation methods and perceptions of climate change in the Nile Basin of Ethiopia*. IFPRI Discussion Paper 00798, International Food Policy Research Institute, Washington, D.C

Dimes J., Cooper P., and Rao K.P.C., (2008): *Impact of climate change on crop productivity in Zimbabwe SAT at the end of 21st century*. SADC-EU Symposium on Climate Change and Soil and Water Management, Lusaka, February 27-30, 2008.

Downing, T.E. (2003): Linking sustainable livelihoods and global climate change in vulnerable food systems. *Die Erde,* 133:363-378.

Dumanski, J., R. Peiretti, J. Benetis, D. McGarry, and C. Pieri. (2006): *The paradigm of conservation tillage*. Proc. World Assoc. Soil and Water Conserv., P1: 58-64.

Eicher C. K., and Kupfuma B., (1997): *Zimbabwe's Emerging Maize Revolution* in Byrelee D., and Eicher C. K., *Africa's emerging maize revolution*. Lynne Rienne Publishers, Inc. USA

Ellis, F., (2000): *The determinants of rural livelihood diversification in developing countries*. Journal of Agricultural Economics 51:289–302.

Eriksen S., O'Brien K., and Rosentrater L., (2008): *Climate change in Eastern and Southern Africa: impacts, vulnerability and adaptation*. Department of Sociology and Human Geography, University of Olso

Fankhauser S., Tol R S J., and Pearce D W., (1997): *The aggregation of climate change damages: a welfare theoretic approach*. Environmental and Resource Economics, 10:249-266.

FAO., (1997): *"Seawater intrusion in coastal aquifers. Guidelines for study, monitoring and control"*. FAO Water Reports N. 11, Rome. 152 pp.

Füssel, H.-M., Klein, R.J.T., (2006): *Climate change vulnerability assessments: an evolution of conceptual thinking*. Climatic Change 75 (3):301–329.

Füssel H., (2007): Vulnerability: *A generally applicable conceptual framework for climate change research*. Global Environmental Change 17(2): 155–167.

Graef F. and Haigis J., (2001): *Spatial and temporal rainfall variability in the Sahel and its effects on farmers' management strategies*. Journal of Arid Environments (2001) 48:221–231

Gumbo D., (2006): *Zimbabwe: Country Case Study on Domestic Policy Frameworks for Adaptation in the Water Sector*. WWF-Southern Africa

Handmer, J W., Dovers S. and Downing T E., (1999): *Societal vulnerability to climate change and variability*. Mitigation and Adaptation Strategies for Global Change, 4:267–281.

Hartman J., Ebi K., McConnell K.J., Chan N., and Weyant J., (2002): *Climate Suitability for stable malaria transmission in Zimbabwe under different Climate Change Scenarios*. Global Change and Human Health, Volume 3, Number 1, 42-54, DOI: 10.1023/A:1011962123196

Hoddinott, J., and Quisumbing A., (2003): *Methods for micro-econometric risk and vulnerability assessments. Social Protection Discussion Paper* Series 0324. Washington, DC: The World Bank, Human Development Network.

Horrel S., and Krishnan P., (2006): *Poverty and Productivity in Female-Headed Households in Zimbabwe*. Faculty of Economics, University of Cambridge, Cambridge CB3 9DD

Hulmen M, and Sheard N., (1999): *Climate Change Scenarios for Zimbabwe*. Climatic Research Unit, Norwich, UK, 6pp

Information Unit on Climate Change (IUCC), (1994): *Zimbabwe's vulnerability to climate change*. UNEP, Switzerland. iucc@unep.ch

IPCC (2001). *Climate change 2001: impacts, adaptation, and vulnerability*. Cambridge, Cambridge University Press.

IPCC (2007). *Summary for Policymakers*: *The Physical Science Basis. Contribution of Working Group I to the Fourth Assessment Report of the Intergovernmental Panel on Climate Change*. Cambridge University Press, Cambridge, United Kingdom and New York, NY, USA.

Jamal H., (2009): Assessing vulnerability to poverty: Evidence from Pakistan. SPDC Research Report 80, Social Policy and Development Centre, Karachi

Julia M., (1999): *One NGO's contribution to women economic empowerment and social development in Zimbabwe*. Journal of Social Development in Africa, 14, 1, 19-30

Kanbur R., (1987): *The standard of living: Uncertainty, inequality and opportunity.* In The Standard of Living, Ed. G. Hawtorn. New York: Cambridge University Press.

Kates RW., (2000): *Cautionary tales: adaptation and the global poor*. Climate Change 45(1):5–17

Leder L., (2010): *Sorghum and millets*. Department of technology, Central Food Research Institute, Hungary

Levine, E., (2006): *Domestic Policy Frameworks for Adaptation to Climate Change in the Water Sector Part II: Non-Annex I Countries. Lessons Learned from Mexico, India, Argentina and Zimbabwe*. OECD, Paris http://www.oecd.org/dataoecd/46/15/37671630.pdf

Lovell, C., (2000): *Productive Water Points: Guidelines on Integrated Planning for Rural Water Supply*. ITDG Publishing.

Ligon, E., and Schechter L., (2003): *Measuring vulnerability*. Economic Journal 113(March): C95–C102.

Maarten K. van Aalst (2007): *The Impacts of Climate Change on the Risk of Natural Disasters* .Red Cross/Red Crescent Centre on Climate Change and Disaster Preparedness. Netherlands.

Mazvimavi K., Twomlow S., Murendo C., and Musitini T., (2007): *Science in Agricultural Relief and Development Programs: The Case of conservation farming in Zimbabwe*. AAAE Conference Proceedings (2007) 321-325.

Mbaya S., (2003): *The Southern African food security crisis, causes and responses: a regional overview*. Southern Regional Poverty Network, Human Research Council, Pretoria

McCarthy J J., Canziani O F., Leary N A., Dokken D J., and White K S., (2001): *In Climate Change 2001: Impacts, Adaptation and Vulnerability*- Contribution of Working Group II to the Third Assessment Report of the Intergovernmental Panel on Climate Change. Cambridge: Cambridge University Press.

Muamba F and Kraybill D., (2010): *Weather vulnerability, climate change and food security in Mt Kilimanjaro*. Selected paper 11887. Department of Agricultural, Environment and Development Economics. The Ohio State University, Columbia, OH 43210

Mubaya C.P., Njuki J., Liwenga E., Mutsvangwa E.P., and Mugabe FT., (2010): *Perceived impacts of climate related parameters on smallholder farmers in Zambia and Zimbabwe*. Journal of Sustainable Development in Africa (Volume 12, No.5, 2010) ISSN: 1520-5509, Clarion University of Pennsylvania, Clarion, Pennsylvania

Mugabe FT., Hodnett MD, Senzanje A., (2003): *Opportunities for increasing productive water use from dam water: a case study from the semi arid Zimbabwe*. Agriculture Water Management 1827 (2003):1-15

Nachmais, CF.,and Nachmais, D., (2008): *Research methods in the Social Sciences: Seventh Edition.* New York, NY: Worth Publishers

Ninno C., Vecchi G., and Hussain N., (2006): *Poverty, Risk and Vulnerability in Pakistan*. Centre for Research in Poverty and Income Distribution (CRPRID). Planning Commission, Government of Pakistan http://www.crprid.org/Publications/PAKVulnerabilitytoPoverty

NOAA (National Oceanic and Atmospheric Administration), (2005): What causes global climate change? Project under grant # NA16GP2576. The trustees of Columbia University

O'Brien, K., Eriksen, S., Schjolen, A., Nygaard, L., (2004a): *What's in a word? Conflicting interpretations of vulnerability in climate change research*. CICERO Working Paper 2004:04, CICERO, Oslo University, Oslo, Norway.

UNEP (United Nations Environment Programme), (1997): *Implementation strategy to reduce environmental impact on energy related activities in Zimbabwe*. Working Paper No 5, UNEP Collaborating Centre on Energy and Environment. Riso Notion Laboratory, Denmark

Piha, M.I., (1993): *Optimizing fertilizer use and practical rainfall capture in a semi-arid environment with variable rainfall*. Experimental Agriculture 29:405–415.

Schulze R., Meigh J., and Horan M., (2001): *Present and future vulnerability of Eastern and Southern Africa's hydrology and water resources*. S. Afr. J. Sci. 97:150-160

Shultz, T., (1975): *The value of ability to deal with disequilibria*. Journal of Economic Literature, 13:827-96.

Skoufias, E., (2003): *Consumption smoothing in Russia. Economics of Transition* 11(1): 67–91.

Smith, J., and S. S. Lenhart., (1996): *Climate change adaptation policy options*. *Climate Research* 6(2):193–201.

Smit, B., Burton, I., Klein, R.J.T., Street, R., (1999): *The science of adaptation: a framework for assessment*. Mitigation and Adaptation Strategies for Global Change 4:199–213.

Smit, B., and Wandel, J., (2006): *Adaptation, adaptive capacity and vulnerability*. Global Environmental Change, 16:282-292.

Stern N., (2006): *Stern Review Report on the Economics of Climate Change*. Pre-publication edition available at www.hm-treasury.gov.uk. Published edition

Thomas D S G and Twyman C., (2005): *Equity and justice in climate change adaptation amongst natural-resource-dependent societies*. Global Environmental Change 15:115-124.

Vincent, V., Thomas, R.G., (1960): *Agricultural survey of Southern Rhodesia*. Part 1. Agro-Ecological Survey. Government Printers, Salisbury.

Weir S., (1999): *The effects of education on farmer productivity in Rural Ethiopia*: WPS99-7. Center for the Study of African Economies, Department of Economics, University of Oxford, UK

WMO (World Meteorological Organization)., (2004): '*WMO statement on the global climate in 2004*'. WMO Press Release. No. 718. 15 December. WMO

Zezza A., Winters P., and Carletto G., (2007): Rural household access to assets and Agrarian Institutions: a cross country comparison. ESA Working Paper No. 07-17. Agricultural Development Economics Division. The Food and Agriculture Organization of the United Nations

Zhakata W., Chidzambwa S., and Tsiga A., (2004): *Technology transfer needs in Zimbabwe: National overview*. Climate change office, Ministry of Environment and Tourism, Zimbabwe
