# Supplementary material for: Systematic Review of Methods in Low-Consensus Fields: Supporting Commensuration through `Construct-Centered Methods Aggregation’ in the Case of Climate Change Vulnerability Research
Source: PLoS One. 2016 Feb 22;11(2):e0149071. doi: 10.1371/journal.pone.0149071 (PMC4762661; doi:10.1371/journal.pone.0149071)
Supplement: S2 File — Source articles and bibliography. (ZIP) [file pone.0149071.s002.zip › data requirements/Nkodze et al.pdf]

Journal of Agricultural Science; Vol. 5, No. 10; 2013
ISSN 1916-9752 E-ISSN 1916-9760
Published by Canadian Center of Science and Education

Factors Affecting Households Vulnerability to Climate Change in Swaziland: A Case of Mpolonjeni Area Development Programme (ADP)

Majahodvwa S. Nkondze[1], Micah B. Masuku[2] & Absalom Manyatsi[3]

[1] P.O. Box 3, Kwaluseni, Swaziland

[2] Department of Agricultural Economics and Management, P. O. Luyengo, Luyengo. M205. University of Swaziland, Swaziland

[3] Department of Agricultural and Biosystems Engineering, P. O. Luyengo, Luyengo. M205. University of Swaziland, Swaziland

Correspondence: Micah B. Masuku, Department of Agricultural Economics and Management, P. O. Luyengo, Luyengo. M205. University of Swaziland, Swaziland. Tel: 268-7602-6557. E-mail: mbmasuku@uniswa.sz

Received: June 9, 2013 Accepted: July 8, 2013 Online Published: September 15, 2013

doi:10.5539/jas.v5n10p108 URL: http://dx.doi.org/10.5539/jas.v5n10p108

Abstract

This study investigated household vulnerability to climate change and the factors affecting vulnerability of the households at Mpolonjeni Area Development Programme in Swaziland. Primary data were collected through personal interviews from 323 randomly sampled households. The household vulnerability index was used to establish household vulnerability and the multinomial logistic regression model was used to identify the factors affecting household vulnerability. The results show that 39.6% of the households were lowly vulnerable, 58.2% were moderately vulnerable and 2.2% were highly vulnerable. Parameter estimates of the multinomial regression model show that the number of sick members, number of employed members, number of dependants, household size and the livestock index influence households to move from low vulnerability to moderate vulnerability or high vulnerability. Households are vulnerable to external shocks thus appropriate policy interventions should be put in place. A health policy would help reduce vulnerability of households and a rural development policy would create employment opportunities leading to improved livelihoods.

Keywords: households vulnerability, climate change, multinomial logistic regression

1. Introduction

According to Thompson (2013) about 77% of the Swazi population reside in the rural areas and rely on agriculture for their livelihood. With the poverty level at 69% these people are vulnerable to all sorts of external shocks such as disease and climate change. HIV and AIDS is one of the serious threats to livelihood with more than 40% of the population infected with the pandemic. Masuku and Sithole (2009) reported that there was a positive relationship between HIV and AIDS and food insecurity in the country, and that people were selling livestock as a means of sustenance and to pay medical bills and post death expenses. Crop production has gone down due to a decline in land utilisation, as inputs become unaffordable when the sick or dead member was the one providing finance for inputs. Household labour is being diverted to caring for the sick, and skilled people die or fall sick living behind people with little or no skill on production. This has resulted in more households falling below the poverty line (Masuku & Sithole, 2009). Climate change is another threat faced by households in the country. According to Manyatsi (2010) climate change will have a significant effect on agriculture in Swaziland as temperatures are predicted to increase by as much as 2.5^{o}C by 2050 and precipitation is likely to decrease by as much as 100mm. This will result to low maize yields making the poor even more vulnerable. According to the Food Agriculture and Natural Resources Policy Analysis Network (FANRPAN) (2011) the majority of rural households lack the necessary capacity to adapt to the negative impacts of the external shocks and that policy response is limited, institutional arrangements are weak, whilst interventions are not carefully matched to needs. The social and ecological conditions under which people live have an influence on the way they are affected by climate change. As a result, the causes of people’s vulnerability to climate change are to a large extent societal and resulting from political and economic processes. According to Kirsten and Eriksen (2007) other challenges

faced by people other than climate change affects the way in which they can cope and adapt to climate related problems. Some of the social and ecological conditions that influence people's lives and can lead to their vulnerability to climate change constitute lack of access to basic social services, loss of employment, violence, environmental degradation and loss of access to natural resources. Small scale farmers are vulnerable to drought, floods and changing seasonality. This is as a result of degraded ecosystems, biodiversity loss, loss of natural resources, land rights and valuable trees, lack of fodder for animals and drinking water shortage. Orphans and old people caring for orphans are also vulnerable to drought, floods and cyclones. This is due to lack of income, HIV and AIDS, little household labour, lack of education, illness and hunger (Kirsten & Eriksen, 2007). As a result, there is a need to measure household vulnerability to inform policy decisions and programme interventions aimed at adaptation to external hazards. With climate change being a threat to households livelihoods, and its impact on households have not yet been studied in Swaziland, therefore, the study was thus carried out to measure households' vulnerability to climate change and to identify the factors affecting their vulnerability to climate change.

2. The Concept of Vulnerability

According to Kasperson, Turner, Kasperson and Hsich (2003) the term "vulnerability" has no universally accepted definition, largely because different disciplines use the term differently to explain their areas of concern. Studies on epidemiology define vulnerability as the degree to which an exposed unit is susceptible to being harmed by exposure to a perturbation or stress, in conjunction with its ability (or lack thereof) to cope, recover or adapt (become a new system or go extinct). In contrast, poverty and development literature, which focuses on social, economic and political conditions, defines vulnerability as an aggregate measure of human welfare that integrates environmental, social, economic, and political exposure to a range of harmful perturbations. FANRPAN (2011) defines vulnerability as the inability to withstand the adverse impact of exposure to stresses or shocks associated with environmental and social change, and the absence of the capacity to adapt to the impact. Adger and Kelly (1999) define social vulnerability as the exposure of groups or individuals to stress as a result of social and environmental change, where "stress" refers to unexpected changes and disruptions to livelihoods.

2.1 Climate Change and Household Vulnerability

The Intergovernmental Panel on Climate Change (IPCC) (2007) defines vulnerability to climate change as the degree to which a system is susceptible, or unable to cope with adverse effects of climate change, including climate variability and extremes, and vulnerability is a function of the character, magnitude and rate of climate variation to which a system is exposed, its sensitivity, and its adaptive capacity. Literature shows that households are vulnerable to climate change. Thornton and Herrero (2008) reports that in Sub-Saharan Africa, many vulnerable regions are likely to be adversely affected by climate change. These include the mixed arid-semiarid systems in Sahel, arid- semiarid rangeland systems in parts of Eastern Africa, the systems in the Great Lakes region of Eastern Africa, the Coastal regions of Eastern Africa and many of the drier zones in Southern Africa. According to Showmake (2008), households that do not own livestock as well as large households, and those that rely on rain-fed agriculture are more vulnerable to climate change. Poor and landless households, children, women and large sized families are mostly affected by climatic shocks. This is as a result of among other things little house labour, rising input costs, water shortage, lack of income and inadequate veterinary services.

Social and ecological conditions that influence poor people's lives and make people vulnerable to climate change include lack of access to basic social services, loss of employment opportunities, lack of empowerment to participate in political processes, violence and insecurity as well as environmental degradation and lack of access to important natural resources (Kirsten & Eriksen, 2007; Abate, 2009).

2.2 Factors Contributing to Households Vulnerability

According to Damas and Israt (2004) many factors contribute to vulnerability, and these factors act to undermine capacity for self-protection, blocks or diminish access to social protection, delays recovery or expose some groups to greater or more frequent hazards than other groups. The factors that contribute to vulnerability include rapid population growth, poverty and hunger, poor health, low levels of education, gender inequality, fragile and hazardous location, and lack of access to resources and services, including knowledge and technological means and disintegration of social patterns (social vulnerability). Damas and Israt (2004) further report that other causes of vulnerability include lack of access to information and knowledge, lack of public awareness, limited access to political power and representation (political vulnerability). When people are socially disadvantaged or lack political voice, their vulnerability is exacerbated further. Economic vulnerability is related to a number of interacting elements, including its importance in the overall national economy, trade and foreign exchange

earnings, aid and investments, international prices of commodities and inputs, and production and consumption patterns. Environmental vulnerability concerns land degradation, earthquake, floods, hurricane, drought, storms, water scarcity, deforestation, and other threats to biodiversity (Damas & Israt, 2004). Makoka and Kaplan (2005) argue that vulnerability is caused by a broad range of political, institutional, economic, environmental and socio-cultural factors such as insufficient knowledge, organisational gaps, lack of personal and financial resources and inadequate legislation. As a result, vulnerability must not be restricted to a simple cause-effect relationship. According to Dirway (2010), income from selling livestock, remittances, household size, education level of the head of the household, skills acquired from training and age of the household head influence participation of farmers in massive food production. Inayatullah, Munir, Khan, Shakeel and Tariq (2012) report that age of the household head, education of the household head, job experience of the head of the household, number of working members in the household, index of livestock holding and per capita income of household affect rural livelihood and hence vulnerability status of households. An educated and older household head is likely to be employed and thus having consistent income. The more employed members in a household the better in the sense that it would have a steady income inflow. Livestock ownership benefits households in that they may sell and generate income, get food from the animals, manure and use the animals for power. Large households are likely to have more dependants hence likely to be vulnerable to external shocks.

2.3 Measures of Vulnerability

Vulnerability may be measured using economic approach, indicator approach and the household Vulnerability Index (HVI). The econometric approach method uses household level socio-economic survey data to analyse the vulnerability levels of different social groups. It includes three assessments namely vulnerability as expected poverty (VEP), vulnerability as low expected utility (VEU) and vulnerability as uninsured exposure to risk (VER). These approaches measure welfare loss as a result of shocks, but differ in that VEP and VEU measure the ex-ante probability of a household's consumption of falling below a given minimum level in the future due to current or past shocks, while VER measures ex post welfare loss due to shocks. The most commonly cited shocks are climatic, economic, political, social, legal, crime and health conditions (Hoddinott & Quisumbing, 2003). The disadvantages of the VEP method is that estimates across a single cross-section requires an assumption that the cross-sectional variability captures temporal variability, whereas the weakness of the VEU method is that it is difficult to account for an individual's risk preference, given that individuals are ill-informed about their preferences especially those related to uncertain events (Kanbur, 1987). The other limitation of the VER method is the absence of panel data, it is typically impossible to measure impact of shocks in the absence of panel data as ex-ante and ex-post consumption and income data are generally not included in cross-sectional data sets.

The indicator approach is another method that could be used to measure vulnerability. This is based on developing a range of indicators and selecting some of them through expert judgement, principal component analysis, or correlation with past disaster events. Each of these selection procedures is used to choose the indicators that account for the highest proportion of vulnerability. The selected indicators may be used at the local, national, regional or global scales (IFPRI, 2009). The indicator approaches are important in monitoring trends and exploring conceptual frameworks. However, this approach is constrained by being subjective in the selection of variables and their relative weights, the availability of data at various scales, and the difficulty of testing or validating the different metrics (Luers, 2005).

FANRPAN developed the household Vulnerability Index (HVI), which is a statistical tool for measuring household vulnerability. The HVI measures the vulnerability of households and communities in relation to the impact of diseases and shocks such as HIV and AIDS, erratic weather patterns and poverty (Sibanda, Chipfupa & Kureya, 2008). According to Sibanda et al. (2008), the HVI categorizes a household by assessing external vulnerability that is induced by shocks and internal vulnerability of such a household to withstand shocks, then, classifies the household as coping, acute, or in an emergency situation depending on the household's ability to cope. The tool achieves this by assessing a household's access to five livelihood capital assets: natural assets such as land, soil and water; physical assets such as livestock and equipment; financial assets such as savings, salaries, remittances or pensions; human capital assets such as farm labour, gender composition and dependants; and social assets such as information, community support, extended families and formal or informal social welfare support. To compute the index, 15 variable classes (called dimensions) are assessed together, and a statistical score is calculated for each household. External vulnerability introduced by a shock is assessed separately and used to introduce weights on the household's access to the five capital assets. The output is the classification of households into three categories according to the level of vulnerability. The first category is low vulnerability, which means that the household is in a vulnerable situation but is still able to cope without external

assistance. The second category is moderate vulnerability, which classifies households that need urgent, but temporary external assistance for them to recover when hit hard by a shock. Lastly, there is the high vulnerability category, which is a class of households that are in a situation of almost a point of no return, but could be resuscitated only with the best possible expertise (Sibanda et al., 2008).

3. Methodology

3.1 Study Design

This was a descriptive quantitative study that sought to assess vulnerability of households to climate change. The study also investigated the factors that influence vulnerability of the households.

3.2 Study Area

The study was carried out at Mpolonjeni Area Development Programme (ADP). The ADP is located in the central east of Swaziland in the Lubombo region and it is about 105 kilometres from Mbabane. Five chiefdoms (communities) are covered by the programme, namely: Ngcina, Mpolonjeni, Shoba, Ndzangu, and Langa. According to FANRPAN (2011), the Mpolonjeni Area Development Programme has 3212 households with an estimated population of 24 000. The Mpolonjeni Area Development Programme falls under the Lowveld climatic zone and it has high summer temperatures with mean temperature ranging between 17-27°C. On average 600-750 mm of rainfall are received annually. Its altitude is 200-500 metres above sea level. Other characteristics of the Lowveld include winter frosts and fertile soils. The area is prone to drought and climate variability (Kureya, Chipfupa, & Nxumalo, 2009). Households rely on raising crops such as maize, beans, groundnuts, cotton, sweet potato, jugo beans, potatoes, sorghum, cowpeas, and pumpkins for livelihood. However, due to high temperatures and less rainfall, yields, in particular for maize are poor in most years. As a result, the Lubombo region has consistently received food aid over the years. They also rear livestock such as cattle, goats, poultry, pigs and sheep (Kureya et al., 2009). Figure 1 is the map of Swaziland with the shaded area showing the location of Mpolonjeni Area Development Programme.

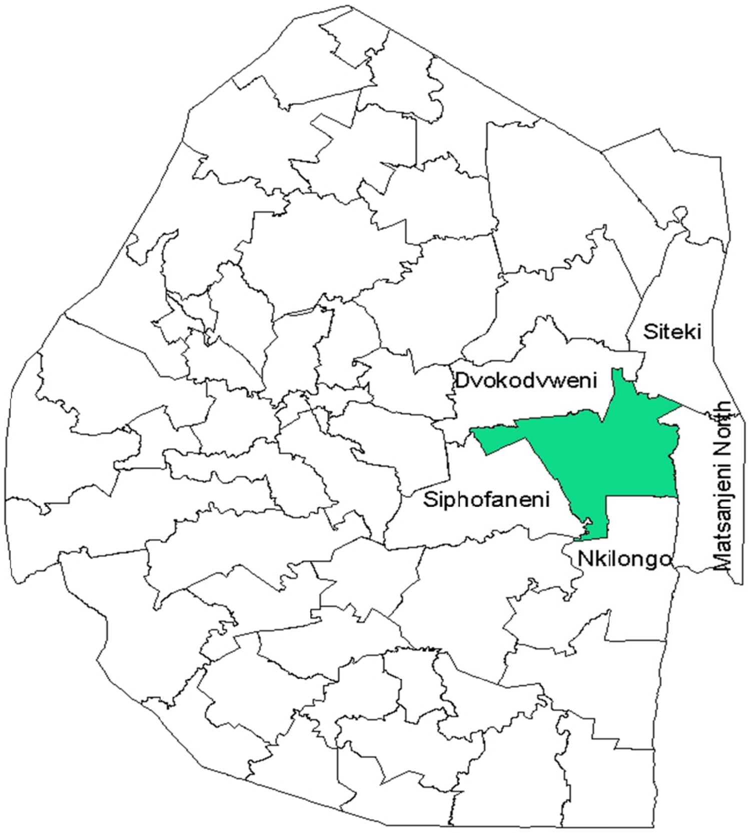

Figure 1. Map of Swaziland showing location of Mpolonjeni ADP

3.3 Sampling Procedure and Sample Size

A stratified random sampling procedure was used to come up with the required sample. The master table from the census data collected by FANRPAN in 2009 at Mpolonjeni Area Development Programme was used as the sampling frame. The sample frame consisted of 3212 households, which were divided according to the five chiefdoms (communities) under Mpolonjeni ADP: Ngcina, Mpolonjeni, Shoba, Ndzangu and Langa. Thereafter, ten percent of households from each of the chiefdoms were randomly sampled to come up with the study sample of 350 households: 179 households were sampled from Kalanga, 36 were sampled from Kandzangu, 49 households from Kashoba, 69 from Mpolonjeni and 17 from Ngcina. However, due in ability to trace some of the households, only 323 households were studied. Table 1 shows the sampled households from each of the five chiefdoms.

Table 1. Sampled households from each chiefdom

**Chiefdom**	**Number of households**	**Sampled households**	**%**
Mpolonjeni	618	69	11.1
Ngcina	157	17	10.8
Kashoba	441	49	11.8
Kandzangu	351	36	10.2
Kalanga	1645	179	10.8
Total	3212	350	10.9

3.4 Data Collection

Primary data were collected through personal interviews from 323 sampled households using an interview schedule. The data were collected between January 2013 and March, 2013.

3.5 Data Analysis

Descriptive statistics such as percentages and frequencies were used to analyse the data. The Statistical Package for Social Sciences (SPSS) version 20 and Microsoft Excel were used to analyse the data. The factors affecting vulnerability was analysed using multinomial logistic regression.

3.6 Analytical Framework

To establish the vulnerability of the households, the household vulnerability index was calculated. The fuzzy set approach was used to calculate the HVI as follows:

- One can state that for the population N made up of n households i.e. (N = $\{hh_1, hh_2, hh_3 \ldots hh_n\}$, V is a subset of *v* households that have some degree of vulnerability, hence having internal vulnerability. Thus $v \leq n$ and $v=0$ implies that there are no vulnerable households, and $v=n$ implies that all households are vulnerable.
- One can also break down the vulnerability X into *m* specific dimensions of impact, and give a corresponding weight (w_i, $i=1,\ldots,m$) to each dimension. The weights can be predetermined, or developed using an appropriate function. For the generalized HVI model, the weights correspond to the external component of vulnerability.
- The vulnerability of any given household hh_i $i=1\ldots n$ to the *jth* $j=1,\ldots m$ dimension of impact can be expressed as X_{ij}, and set to take values between 0 and 1 such that 0 = no impact and 1 full impact. Thus each X_{ij} denotes the degree of vulnerability of household *i* to the *j*th dimension of impact, and $X_{ij}w_i$ will be the corresponding weighted vulnerability.
- The sum of the weighted vulnerabilities across all dimensions will give the particular household's total vulnerability Vhh_i to HIV and AIDS, that is:

$$\sum_{j=1}^{m} X_{wj} / \sum_{j=1}^{m} w_j \quad = \quad Vhh_i$$

- It is also possible to sum down the dimensions and calculate the particular dimension's contribution to vulnerability.

For the HVI, within the homogenous community context, the sum of the weights are set to

$$\sum_{j=1}^{m} w_j = 100$$

The multinomial logistic regression model is an extension of the binary logit model, with more than two values for the dependent variable (Dragos & Veres, 2007). In this study, the dependant variable has three categories: low vulnerability, moderate vulnerability and high vulnerability. The low vulnerability category was used as the reference category. The model was expressed as:

Thus,

$$P(y_i = j) = P_{ij} = \frac{\exp(x_i \beta_j)}{\sum \exp(x_i \beta_k)} \quad \text{where } 0 < P_{ij} < 1 \qquad (1)$$

$$P(y_i = j) = P_{ij} (\beta_0 + \beta_1 x_1 + \ldots \beta_k x_k) = P_{ij}(\beta_0 + x\beta) \qquad (2)$$

Where: p_{ij} = the probability of a household *i* to be moderate or highly vulnerable with respect to:

x_i = low vulnerability vector of the independent variables associated with the household *i*

β_j = the vector of parameters associated to the alternative j

Table 2 is a description of the dependant variable used in the multinomial logistic regression model. Lowly vulnerable households have an HVI ranging from 0 to 47, moderately vulnerable households have an HVI of 47.1 to 63.7 and highly vulnerable households have an HVI of 63.71 to 100. Table 3 presents the summary of the explanatory variables for the multinomial logistic regression model.

Table 2. Description of the dependent variable for multinomial logistic regression model

Household Vulnerability Category	Description
Lowly vulnerable	Household with an HVI of 0-47
Moderately vulnerable	Household with an HVI of 47.1-63.7
Highly vulnerable	Households with an HVI of 63.71-100

Table 3. Summary of the explanatory variables for multinomial logistic regression model

**Explanatory Variable**	**Values**	**Expected sign**	**Transformation**
Livestock index(x_1)	index	(-)	Ca+Gb+Sc+Dd (where C=cattle, G=goats, S=sheep, D=donkeys; Livestock weights: a=0.5, a = 0.1, c=0.1 and d=0.3)
Household size (x_2)	number	(+)	N/A
Number of dependants (x_3)	number	(+)	Children aged 0-18; scholars aged 18-63; adults>64 and bedridden or disabled members
Number of sick members ($x_{4)}$	number	(+)	N/A
Number of employed members (x_5)	number	(-)	N/A
Educational level of the head of the household (x_6)	Years	(-)	N/A

4. Results and Discussion

4.1 Household Vulnerability at Mpolonjeni Area Development Programme

To address the question of vulnerability to external shocks at Mpolonjeni ADP, the Household Vulnerability Index was used to establish the vulnerability status of the studied households. According to FANRPAN (2011), through the HVI, the households are classified into three categories: lowly vulnerable, which are households that are in a vulnerable situation, but can still cope; moderately vulnerable households are those that need urgent but temporary assistance in case of a shock and the highly vulnerable households are those that are almost at a point of no return. As shown by Table 4, about 39.6% of the households were lowly vulnerable, 58.2% were moderately vulnerable and 2.2% were highly vulnerable.

Table 4. Household vulnerability status of the households (n=323)

Vulnerability category	HVI range	Situation of household	F	%
Lowly Vulnerable	0-47	Coping household-household in a vulnerable situation but still able to cope.	128	39.6
Moderately Vulnerable	47.1-63.7	When hit hard by a shock, the household needs urgent but temporary external assistance for it to recover.	188	58.2
Highly Vulnerable	63.71-100	Emergency level household-the equivalent of an intensive care situation-could be resuscitated only with the best possible expertise.	7	2.2
Total			323	100

4.2 Factors Affecting the Vulnerability of Households at Mpolonjeni Area Development Programme

According to available literature, a number of factors can influence household vulnerability to external shocks. Abate (2009) reports that poor and landless households, children, women and large sized families are mostly affected by climatic shocks. The education level of the head of the household, age of the household head, job experience of the head of the household, number of employed members of the household, index of livestock holding and per capita income of the household affect rural livelihood (Inayatullah et al., 2012). The results in Table 5 show that for a household to shift from the low vulnerable category to the other moderate or high vulnerable category was significantly ($p<0.01$) influenced by the livestock index, number of employed members and number of sick members. The odds ratio (0.851) for livestock index suggests that a unit increase in livestock index decreases the probability of a household shifting from low vulnerability category to moderate category by 14.9% {(0.851-1)x100}. A unit increase in the number of employed members would decrease the probability of a household shifting from low vulnerability category to moderate category by 30.3% {(0.697-1) x100} whilst a unit increase in the number of sick members would increase the probability of a household shifting from low vulnerability to high vulnerability by 440% {(1-45.073)x100}. Household size, number of dependents and number of sick members were statistically significant at 5% level ($p<0.05$). A unit increase in the number of sick members would increase the probability of a household shifting from low vulnerability to moderate vulnerability by 75.4% {(1.754-1)x100} and a unit increase in the number of dependants would increase the probability of a household shifting from low vulnerability to highly vulnerable category by 15811% {(159.11-1)x100}. A unit increase in household size would decrease the probability of a household shifting from low vulnerability to high vulnerability by 99.5% {(0.005-1)x100}. The results show that a household with sick members is likely to be vulnerable and this is as a result of the household diverting resources that they would otherwise use for producing food to caring for the sick members. Sick members of a household also deprive that particular household of valuable production labour. A household with employed members is likely to be lowly vulnerable. This is because employed members would generate income for the household hence in case of a shock such a household may cope. The more dependants a household has the more likely for it to be vulnerable since a larger proportion of its resources are directed to the dependants without the dependants making any reasonable contribution towards the welfare of the household.

The negative sign for household size was not expected. This is because one would expect a larger household to be highly vulnerable in the sense that it is likely to have more dependants. The educational level of the head of

the household was the only factor that was not statistically significant for a household to shift from low vulnerability to either of the other two categories.

Table 5. Estimates for multinomial logistic regression model

Variables	Moderately Vulnerable			Highly Vulnerable		
	B	Exp β	p-value	B	Exp β	p-value
Intercept	0.0981* (0.443)		0.027	5.316 (3.428)		0.102
Livestock Index	-0.162** (0.030)	0.851	0.000	-1.323 (2.295)	0.266	0.5645
Number of Employed members	0.361** (0.124)	0.697	0.004	-0.220 (1.286)	1.286	0.864
Number of Sick members	0.562* (0.273)	1.754	0.040	3.808** (1.368)	45.073	0.005
Number of dependants	0.093 (0.084)	1.097	0.271	5.070* (2.453)	159.11	0.039
Educational level of the head of the household	-0.011 (0.026)	0.989	0.677	-0.067 (0.183)	0.93	0.712
Household size	-0.004 (0.068)	0.996	0.958	-5.308* (2.573)	0.005	0.039

**denotes significance at 0.01 level; *denotes significance at 0.05 level. Low vulnerability was the reference category. Figures in brackets are standard errors.

4.3 Conclusion

The results show that 2.2% of the households were highly vulnerable, suggesting they are in a state of emergency indicating that they need immediate attention and special expertise if they are to pull out of the situation they are in. A majority of the households (58.2%) were moderately vulnerable, implying that in case of an external shock they would need some assistance for them to recover. Only 39.6% of the households were able to cope even though they were also vulnerable. The shift of a household from the state of low vulnerability to the state of moderate or high vulnerability was influenced by the number of sick members, the number of employed members, number of dependants, household size and the livestock index in the household.

4.4 Implications

The majority of households in Mpolonjeni Area Development Programme would need external assistance in case of an external shock and even though not many in number, some households were in an emergency situation. As a result government should come up with appropriate intervention policies in order to help these households. Such policies should include a health policy as it has been shown by the study that the number of sick members in a household affects the vulnerability status of the households. This health policy would also help bring in check the issue of dependants in a household. Due to pandemics such as HIV and AIDS more households were losing skilled members and more members were falling ill, increasing the number of dependants in a household and making more vulnerable to climate change. Such a policy would assist households to cope with the shock. Employment affects vulnerability, thus a rural development policy that would create job opportunities in the rural areas would help improve the livelihoods of the households since it will provide them with an alternative source of income. In order to capture the impact of climate change on households, there is need to conduct studies that will involve panel data. This will assist in in reflecting the main impact due to climate change.

Acknowledgements

The authors would like to acknowledge Food, Agriculture, and Natural Resources Policy Analysis Network (FANRPAN) for providing financial support for the research.

References

Abate, F. S. (2009). Climate Change Impact on Livelihood, Vulnerability and Coping Mechanisms in West-Arsi Zone, Ethiopia.

Adger, N., & Kelly, M. (1999). Social Vulnerability to Climate Change and the Architecture of Entitlements. *Miti. Adap strat. Gl. Change, 4*, 253-266. http://dx.doi.org/10.1023/A:1009601904210

Damas, P., & Israt, R. (2004). Vulnerability and poverty: What are the causes and how are they related.

Deressa, T. T., Hassan, R. M., & Ringler, C. (2009). Assessing Household Vulnerability to Climate Change: The Case of Farmers in The Nile Basin of Ethiopia. *International Food Policy Research Institute (IFPRI) Discussion Paper 00935*.

Dirway, T. P. (2010). Application of the sustainable livelihood framework to the analysis of the provincial growth and development plan of the Eastern Cape – a case study of mass food production programme in Nkonkobe municipality and Baffalo City municipality.

Digambar, D. S. (2011). Impact of Climate Change on Livelihood and Biodiversity in Rural Communities: A case study of Siddhi Ganesh and Nepane Community Forestry User Groups of Sindhupalchwok District of Nepal.

Dracos, C., & Veres, V. (2007). Romanian Farmers' market. A multinomial Logit Model Approach. *Zb. Rad. Ekon. Fak. Rij., 25*(2), 291-308.

Food Agriculture and Natural Resources Policy Analysis Network (FANRPAN). (2011). Measuring Vulnerability-Challenges and opportunities. *FANRPAN., 2*(11).

Hoddinott, J., & Quisumbing, A. (2003). Methods for micro econometric risk and vulnerability assessments. Social Protection Discussion Paper Series No. 0324. Social Protection Unit, Human Development Network. Washington D.C. World Bank.

International Food Policy Research Institute (IFPRI). (2009). *Climate Change: Impact of Agriculture and Cost of Adaptation*. Washington, D.C.

Inayatullah, J., Munir, K. K., Khan, M. A., Shakeel, H., & Tariq, R. (2012). Factors affecting rural livelihood choices in Northwest Pakistan. *Sarhad J. Agric., 28*(4), 681-688.

Intergovernmental Panel on Climate Change (IPCC). (2007). *Climate Change 2007: Impacts, Adaptation and Vulnerability*. Summary for policy makers. Retrieved 5 May, 2012 from http://www.ipcc.cg/SPMpdf

Kanbur Ravi, S. M. (1987). Measurement and Alleviation of Poverty. *International Monetary Fund, 34*(1), 60-85. http://dx.doi.org/10.2307/3867024

Kasperson, J. X., Kasperson, R. E., Tunner II, B. L., & Hsich, A. (2003). *Vulnerability to Global Environmental Change*. MIT, Cambridge, MA.

Kirsten, U., & Eriksen, S. (2007). Vulnerability and adaptation to climate change: New challenges for poverty eradication.

Kureya, T., Chipfupa, U., & Nxumalo, D. (2009). *Household Vulnerability Index Pilot Project in Swaziland: Baseline Results.*

Luers, A. L. (2005). The surface of vulnerability: An analytical framework for examining environmental change, *Global Environmental Change, 15*, 214-223. http://dx.doi.org/10.1016/j.gloenvcha.2005.04.003

Makoka, D., & Kaplan, M. (2005). *Poverty and Vulnerability*. University of Bonn, Centre for Development Research.

Manyatsi, A. M. (2010). *Assessing the Vulnerability of Agriculture to Climate Change in Swaziland*. A paper presented at side event of Agriculture and Rural Development day 2010 on the 4th December 2010, Cancun, Mexico

Masuku, M. B., & Sithole, M. M. (2009). The impact of HVI/AIDS on food security and household vulnerability in Swaziland. *Agrekon, 48*(2), 1-23. http://dx.doi.org/10.1080/03031853.2009.9523824

Naqvi, S. M. K., & Sejian, V. (2011). Global Climate Change: Role of Livestock. *Asian Journal for Agricultural Science, 3*(1), 19-25.

Shewmake, S. (2008). Vulnerability and the Impact of Climate Change in South Africa's Limpopo River Basin. *International Food Policy Research Institute discussion paper 00804*.

Sibanda, L. M., Kureya, T., & Chipfupa, U. (2008). The Household Vulnerability Index Framework.

Thompson, C. F. (2007). *Swaziland Business Yearbook*. Retrieved 20 April, 2012 from http://www.swazi.com.sz

Thornton, P., & Herrero, M. (2008). *Climate Change, Vulnerability, and Livestock Keepers: Challenges for Poverty Alleviation*.

Appendix

Variables used in calculating the HVI

Dimension	Hypothesis tested	Variables for testing hypothesis	Transformation
Natural Capital	Soil fertility declines for vulnerable households as application of natural fertilizers declines.	Proportion of field fertilized by natural means. What proportion X of the fields is fertilized by natural means?	2X; CLH:50-100%=0, ALH:0-50%=1,ELH:none=2
	Barriers to access to land for agriculture increase vulnerability	Barred from use of land that you used to cultivate	No=0 Yes =1
	Households revert to the environment for "free" products such as wood when vulnerable. HIV and AIDS affected households rely more on the forest for their livelihoods.	Tree cutting or wood selling as a means of survival, wild fruits collection, environmental management in the presence of sickness or death, quality of water used by household, participation in water or environmental management	2X/5; CLH:answer yes to at most 1 question=0; ALH:answer yes to 1-3 environment questions=1; ELH:answer yes to at least 4 environment questions
	Affected households have difficulties in fully utilizing their land due to limited labour and draft power availability. Vulnerable households do not fully utilize their existing land	% of land not utilized due to sickness (X) What is the total land under cultivation (A)? What land is available but not cultivated due to illness or death in the last season (B) ?	X=B/(A+B) 0% =0 CLH:0-20%=1 ALH:20-50%=3 ELH:>50%=5
Human Capital	Affected households are vulnerable when they have sick members, and the more the number of sick members, the more the vulnerability. Also worse if the sick member is the	Proportion of sick members (X). What is the total Household size (Y)? How many members are sick regularly (have been bedridden for at	X=Z/Y

	head of the household.	least three different times in the last year, with each bout extending to up to a week? Or have been diagnosed with any of TB, Meningitis, Caporsi Sarcoma, Hepatitis, Pneumonia (Z)?)	
	Households that have productive sick members are more vulnerable.	Who is regularly sick None=0 dependent =1 productive adult = 2 Spouse=3 HH head = 4	Highest possible score
	Affected households have a greater number of dependents due to the increasing number of orphans in such households	Dependency ratio (economic burden)X :Number of dependants ({0-15}+{>65}+{bedridden or disabled})/Number of economically active.	Modified dependency ratio: X=dependants/total HH size. CLH: X<0.4; ALH: 0.4<X<0.75 ; ELH: X>0.75
	Female headed and/or child headed households are less able to cope with shocks, compared to male headed households	Age and gender of household head	CLH=0 ALH=3 ELH=6
	HIV and AIDS has caused disintegration in affected households	Household members who have moved away due to sickness or death	CLH: 0; ALH:2 CLH: 2
Physical Capital	Vulnerability especially to food insecurity increases with less use of fertilizers	Nitrogen fertilizer use for staple crop(X). What is your land size Y in ha? What is the weight Z of top dressing fertilizer used in the last season in Kg?	X=Z/400Y CLH:X>0.5; ALH:0.25<X<0.5; ELH: X<0.25
	Affected households have reduced harvests due to limited labour and draft power	Staple cereal output per capita (X). What is the total household size (Y)? How kgs of Maize were harvested (Z)? X=Z/Y	X=Z/150Y CLH:X>0.5; ALH:0.25<X<0.5; ELH: X<0.25
	Households that do not own an ox drawn plough or cart are likely to	Ownership of a plough or ox	Owns a plough and cart = 0, plough only = 1 cart only =2

	face difficulties in cultivation, planting and other farming operations.	drawn cart	none =3
	Households that do not own or own fewer cattle and other livestock are more vulnerable due to limited access to draft power and alternative sources of income and nutritious food.	Productive livestock index X = 3c+ G+S+2D. How many Cattle do you own (C)? Goats (G)? Sheep (S)? Donkeys (D)?	CLH: X>6; ALH: 6>X.>3; ELH: 3>X
	Affected households adopt unsustainable short term coping strategies which might include the selling of assets such as livestock and farm	Livestock sales index X = (3c+g+s+2d)/(3C+ G+S+2D) How many Cattle do you own (C)? Goats (G)? Sheep (S)? Donkeys (D)? How many Z (=3c+g+s+2d) of each were sold in the last year?	CLH: X<0.2; ALH: 0.2<X<0.5; ELH: X >0.5
	Affected households have limited access to extension services due to ill health and inadequate time to devote to such activities.	Access to extension services	Used both = 0; used crop only = 1Used livestock only = 2; do not even know = 3
Financial	Households with little or no savings are more vulnerable	Reliance on bank savings	Every month = 0 In crises only = 1 Do not have many in the bank anymore=2 Do not own a bank account =3
	Affected households have fewer sources of regular income due to unavailability or limited number of formally employed members in a household	Regular sources of financial resources Salary (S), Crop Sales (Cs); Livestock Sales (Ls); Remittance from HH member (Rm), No regular source (Ns)	S=0; Rm=1 Cs=2; Ls=1; Ns=3
	Affected households have limited access to credit loans due to increased risks and lack of collateral associated with such households	Access to credit loans	Household is part of a community or formal credit scheme= 0 borrow from extended family/neighbour = 1 no access to credit loans

		at all = 3,
Households with unpaid debts are most vulnerable.	Presence of unpaid debts	No=0, Yes= 3
Affected households experience increased expenditure on health care due to the presence of more ill members in the household	Expenditure patterns. Food (F), Non-food basic goods (nF), Health (H), Savings (S), Transport to Clinics (Tc), Transport to Work (Tw), Farming inputs/implements (FI), Do not prioritize/plan (Nm) Other (o) , Beer and recreation (B), School Fees (SF),	FI/S/o=0, Tw/B/SF/F/nF=1, H/Tc/=2, Nm=1
Use of additional resources indicate choices under vulnerability	Expenditure of additional financial resources Food (F), Non-food basic goods (nF), Health (H), Savings (S), Transport to Clinics (Tc), Transport to Work Tw), Farming inputs/implements (FI), Other (o) , Beer and recreation (B), School Fees (SF), Income generating projects (Pr)	FI/Tw/o/B/S/Pr=0, Tw/nF=1, Tc/SF=1, F/H=2
Purpose for selling harvests indicates levels of vulnerability.	Use of revenue from crop sales Food (F), Non-food basic goods (nF), Health (H), Savings (S), Transport to Clinics (Tc), Transport to Work (Tw), Farming inputs including Veterinary (FI), Do not get enough to sell (Nm) Other (o) , Beer and recreation (B), School Fees (SF), Income generating	FI/S/o=0, Tw/B/SF/F/nF=1, Tc/=1, H/Nm=2

	Affected households eat less per day due to inadequate food availability	Meals per day	Breakfast, Lunch, Dinner, give 0 for each taken ie 1 meal= 3, 2 meals=2; 3 meals=0;
	Affected households eat less variety per day due to inadequate food availability	Describe the typical food stuffs in meals taken in your household? Maize (porridge/sadza/samp) (St), tea St, sorgum brew St, green vegetable V, wild fruit F, bananas/oranges/apple F, sugar cane St, pumkins V, groundnuts Pr sweet potatoes St, meat Pr, fish Pr,	Give 1 for each category taken, CLH:X>3 ALH: 2<x<3; ELH: X<2
Social Capital	The lesser the number and quality of support channels from external sources, the greater the vulnerability	What support was obtained from Government, NGOs, community and other external support networks in the last 3 months? Give the commonest 2. Food (F), Non-food basic goods (nF), Health (H), Savings (S), Transport to Clinics (Tc), Transport to Work (Tw), Farming inputs including Veterinary (FI), Do not get support(Ns) Other (o) , Beer and recreation (B), School Fees	Tc/H=0; Tw/B/SF/F/nF=1, FI/S/o=2, Ns=4
	The lesser the volume of support from external sources the greater the vulnerability	In which areas did support from Government, NGOs, community and other external support networks completely meet households' requirements? Food (F), Health (H), Transport to Clinics (Tc), Farming inputs including Veterinary (FI), Do not get support(Ns) School Fees	none=2 ; F/H=1; else =2

		(SF),	
	The more informed a household is, the less vulnerable the household	1. Do you have adequate knowledge to cope with AIDS related illnesses for family members?, 2. Do you have adequate knowledge on type of crops to grow, and when to. 3. In any given season, do you know- in advance- the weather forecasts and use this forecasts	count of "No" answers

Copyrights

Copyright for this article is retained by the author(s), with first publication rights granted to the journal.

This is an open-access article distributed under the terms and conditions of the Creative Commons Attribution license (http://creativecommons.org/licenses/by/3.0/).
