# Supplementary material for: Systematic Review of Methods in Low-Consensus Fields: Supporting Commensuration through `Construct-Centered Methods Aggregation’ in the Case of Climate Change Vulnerability Research
Source: PLoS One. 2016 Feb 22;11(2):e0149071. doi: 10.1371/journal.pone.0149071 (PMC4762661; doi:10.1371/journal.pone.0149071)
Supplement: S2 File — Source articles and bibliography. (ZIP) [file pone.0149071.s002.zip › data requirements/Refs for non-open access.docx]

Non-open access articles analysed in the systematic review of research methods for local vulnerability to climate change:

1. Antwi-Agyei P, Dougill AJ, Fraser EDG, Stringer LC. Characterising the nature of household vulnerability to climate variability: empirical evidence from two regions of Ghana. Environ Dev Sustain. 2013;15: 903–926. doi:10.1007/s10668-012-9418-9

2. Bogale A, Taeb M, Endo M. Land ownership and conflicts over the use of resources: Implication for household vulnerability in eastern Ethiopia. Ecol Econ. 2006;58: 134– 145.

3. Calvo C, Dercon S. Vulnerability to individual and aggregate poverty. Soc Choice Welf. 2013;41: 721–740. doi:10.1007/s00355-012-0706-y

4. Chhihn N, Poch B. Climate Change Impacts on Agriculture and Vulnerability as Expected Poverty of Kampong Speu Province, Cambodia. IJERD – Int J Environ Rural Dev. 2012;3: 28–37.

5. Dasgupta A, Baschieri A. Vulnerability to Climate Change in rural Ghana: Mainstreaming climate change in poverty-reduction strategies. J Int Dev. 2010;22: 803–820.

6. Eakin H, Winkels A, Sendzimir J. Nested vulnerability: exploring cross-scale linkages and vulnerability teleconnections in Mexican and Vietnamese coffee systems. Environ Sci Policy. 2009;12: 398–412. doi:10.1016/j.envsci.2008.09.003

7. Eakin H, Benessaiah K, Baretta JF, Cruz-Bello GM, Morales H. Livelihoods and landscapes at the threshold of change: disaster and resilience in a Chiapas coffee community. Reg Environ Change. 2012;12: 475–488. doi:DOI 10.1007/s10113-011-0263-4

8. Füssel H-M, Klein RJT. Climate change vulnerability Assessments: An evolution of conceptual thinking. Clim Change. 2006;75: 301–329. doi:DOI: 10.1007/s10584-006-0329-3

9. Gandure S, Walker S, Botha JJ. Farmers’ perceptions of adaptation to climate change and water stress in a South African rural community. Environ Dev. 2013;5: 39–53.

10. Günther I, Harttgen K. Estimating Households Vulnerability to Idiosyncratic and Covariate Shocks: A Novel Method Applied in Madagascar. World Dev. 2009;37: 1222–1234. doi:doi:10.1016/j.worlddev.2008.11.006

11. Hahn MB, Riederer A, Foster S. The Livelihood Vulnerability Index: A pragmatic approach to assessing risks from climate variability and change—A case study in Mozambique. Glob Environ Change. 2009;19: 74–88. doi:10.1016/j.gloenvcha.2008.11.002

12. Ionesco C, Klein RJT, Hinkel J, Kumar KSK, Klein R. Towards a Formal Framework of Vulnerability to Climate Change. Environ Model Assess. 2009;14: 1–16.

13. Jamir C, Sharma N, Sengupta A, Ravindranath NH. Farmers’ vulnerability to climate variability in Dimapur district of Nagaland, India. Reg Environ Change. 2013;13: 153–164. doi:10.1007/s10113-012-0324-3

14. Luers A, Lobell DB, Sklar LS, Lee Addams C, Matson PA. A method for quantifying vulnerability, applied to the agricultural system of the Yaqui Valley, Mexico. Glob Environ Change. 2003;13: 255–267.

15. Marshall NA. Understanding social resilience to climate variability in primary enterprises and industries. Glob Environ Change. 2010;20: 36–43. doi:doi:10.1016/j.gloenvcha.2009.10.003

16. Mengistu D. Farmers’ perception and knowledge of climate change and their coping strategies to the related hazards: Case study from Adiha, central Tigray, Ethiopia. Agric Sci. 2011;2: 138–145. doi:doi:10.4236/as.2011.22020

17. Misselhorn AA. What drives food insecurityin southern Africa? a meta-analysis of household economy studies. Glob Environ Change. 2005;15: 33–43.

18. Mubaya CP, Njuki J, Mutsvangwa EP, Mugabe FT, Nanja D. Climate variability and change or multiple stressors? Farmer perceptions regarding threats to livelihoods in Zimbabwe and Zambia. J Environ Manage. 2012;102: 9–17.

19. Notenbaert A, Karanja SN, Herrero M, Felisberto M, Moyo S. Derivation of a household-level vulnerability index for empirically testing measures of adaptive capacity and vulnerability. Reg Environ Change. 2013;13: 459–470. doi:10.1007/s10113-012-0368-4

20. Sarris A, Karfakis P. Vulnerability to Covariate and Idiosyncratic Shocks and Safety Net Targeting of Rural Households with an Application to Rural Tanzania. Paris; 2010. Available: http://erd.eui.eu/media/2010/Sarris.pdf.

21. Sietz D, Choque SEM, Lüdeke MKB. Typical patterns of smallholder vulnerability to weather extremes with regard to food security in the Peruvian Altiplano. Reg Environ Change. 2012;12: 489–505. doi:10.1007/s10113-011-0246-5

22. Westerhoff L, Smit B. The rains are disappointing us: dynamic vulnerability and adaptation to multiple stressors in the Afram Plains, Ghana. Mitig Adapt Strateg Glob Change. 2009;14: 317–337. doi:10.1007/s11027-008-9166-1
