# Supplementary material for: Systematic Review of Methods in Low-Consensus Fields: Supporting Commensuration through `Construct-Centered Methods Aggregation’ in the Case of Climate Change Vulnerability Research
Source: PLoS One. 2016 Feb 22;11(2):e0149071. doi: 10.1371/journal.pone.0149071 (PMC4762661; doi:10.1371/journal.pone.0149071)
Supplement: S2 File — Source articles and bibliography. (ZIP) [file pone.0149071.s002.zip › data requirements/Salluetal.pdf]

Copyright © 2010 by the author(s). Published here under license by the Resilience Alliance.
Sallu, S. M., C. Twyman, and L. C. Stringer. 2010. Resilient or vulnerable livelihoods? Assessing livelihood dynamics and trajectories in rural Botswana. *Ecology and Society* **15**(4): 3. [online] URL: http://www.ecologyandsociety.org/vol15/iss4/art3/

Research, part of a Special Feature on Resilience and Vulnerability of Arid and Semi-Arid Social Ecological Systems

Resilient or Vulnerable Livelihoods? Assessing Livelihood Dynamics and Trajectories in Rural Botswana

Susannah M. Sallu [1], *Chasca Twyman* [2], *and Lindsay C. Stringer* [1]

ABSTRACT. In this paper, we explore the resilience and vulnerability of livelihoods within two different social-ecological dryland contexts of Botswana over the last 30 years. We drew on primary field data sources, including oral histories, livelihood surveys, ecological surveys, as well as documented evidence of environmental, socioeconomic, and institutional dynamics to identify a broad range of activities that combine to create a range of different household livelihood outcomes. We used this information as a starting point to assess the ways in which livelihoods have changed over time, and evaluated whether they have become more resilient or more vulnerable, and considered the factors that have contributed to these outcomes. In the context of dynamic dryland social-ecological systems, we applied a livelihood trajectory approach to explore the shocks and stresses that affect livelihoods and to elucidate the characteristics of livelihood strategies that contribute to increased resilience or vulnerability. We used a vulnerability framework as a means of framing discussion about vulnerability and resilience and as a means of identifying broader insights. The research identified "accumulator", "diversifier", and "dependent" households and the ways in which they move between these categories. More resilient livelihood trajectories can be achieved if the important role of formal and informal institutions is recognized.

Key Words: *Botswana; livelihood strategies; livelihood trajectories; resilience; vulnerability*

INTRODUCTION

Pastoralism is an important component of many rural livelihood strategies within Botswana (Dougill et al. 2010). However, livelihoods also comprise a number of other nonpastoral activities, many of which depend on a variety of different components of the natural resource base (Scoones 1996, Twyman 2001, Sporton and Thomas 2002, Sallu et al. 2009). As such, many of the shocks and stresses that can destroy or damage the natural resource base can also adversely affect livelihood prospects over both the long and short term. In this paper, we investigate the resilience and vulnerability of rural livelihoods, and consider their relation to the dynamic natural resource base in two different social-ecological contexts of Botswana. In doing so, we first outline some of the key concepts related to livelihoods in terms of livelihood strategies, trajectories, resilience, and vulnerability. We next outline the research process and develop a background narrative of the environmental and livelihood systems in our study area, qualitatively determining those factors (environmental and nonenvironmental) that contribute to increased vulnerability and/or resilience. We apply Fraser's (2006, 2010) vulnerability framework to help us understand these processes and to inform the direction of future interventions.

Livelihood approaches, resilience, and vulnerability

Chambers and Conway (1992) define a livelihood system as comprising the capabilities, assets (including both material and social resources), and activities required for a means of living. The chosen combination of assets and activities, undertaken usually at the household level, is often referred to as the household's "livelihood strategy". A livelihood strategy encompasses not only activities that generate income but many other kinds of elements, including cultural and social choices

[1]Sustainability Research Institute, School of Earth and Environment, University of Leeds, [2]Department of Geography, University of Sheffield

(Ellis 2000). Livelihoods approaches illustrate how, in different contexts, sustainable livelihoods can be achieved through access to a range of livelihood assets (e.g., natural, social, financial, physical, and human capital) which, within the context of personal, institutional, and environmental provisions and constraints, are combined in the pursuit of different livelihood strategies. Within the sustainable livelihoods framework (Chambers and Conway 1992, Scoones 1998), context is framed within the “vulnerability context”, which includes issues of “seasonality”, “trends”, and “shocks”.

Carney (1998) explains that “a livelihood is sustainable when it can cope with and recover from stresses and shocks and maintain or enhance its capabilities and assets both now and in the future, while not undermining the natural resource base”. This interpretation of sustainability relates strongly to definitions that consider the “resilience” of social-ecological systems. Walker et al. (2006) define resilience as “the capacity of a system to experience shocks while retaining essentially the same function, structure, feedbacks, and therefore identity”. As Marschke and Berkes (2006) suggest, “resilience offers a lens with which to explore stresses and shocks and to understand livelihood dynamics”, and is “future oriented, and is used to characterize a system’s ability to deal with change”. By stresses, we mean “enduring shifts” (such as seasonality and trends) and shocks “transient disruption” (Leach et al. 2007). Incorporation of ideas surrounding resilience alongside understandings of vulnerability can contribute an essential temporal dimension to analysis, allowing the combinations of strategies and circumstances that move households towards more resilient outcomes over time to be identified, ultimately enabling them to embrace change as a result of shocks and/or stresses as opportunities for innovation and accumulation (Folke et al. 2002). In this paper, a focus on resilience can help us learn from the past to inform future planning.

Fraser et al.’s (2010) vulnerability framework draws on several elements of the livelihoods approach, and in the context of this research stimulates the following questions:

- Does the agroecosystem have the resilience to remain productive in a changing vulnerability context?
- Do people have access to livelihood strategies that allow them to survive changes to the vulnerability context?
- Do the institutions have the ability, capacity, and willingness to respond to a changing vulnerability context, especially in crisis situations?

In this paper, we use this framework to inform discussion of the direction and dynamics of livelihoods over a 30-year period. Through comparative research we provide a rich contextual narrative and use it to explore those factors that in isolation and combination push livelihoods along particular “trajectories” towards vulnerability or resilience.

Bagchi et al. (1998) use the term “livelihood trajectories” to describe and explain the direction and pattern of livelihoods of individuals or groups of people (e.g., households). A livelihood trajectory approach allows the examination of an individual household’s “strategic behavior that is embedded in a historical repertoire, in social differentiation” (de Haan and Zoomers 2005), and in perceptions of risk. Such an approach is sensitive to life histories (an individual’s own “story” of their changing livelihoods). A focus on livelihood trajectories allows a deeper penetration into the beliefs, needs, aspirations, and limitations of people’s lives, but one that is also contextualized in relation to power and institutions (de Haan and Zoomers 2005). An increasingly important application of the livelihood trajectory approach is in exploring the shocks and stresses that can affect livelihoods, as well as in elucidating the characteristics of the overall livelihood strategy that contribute to increased resilience or vulnerability.

METHODS

Data were collected in 2004 and 2005 when fieldwork was carried out as part of a larger research project that considered environmental, socioeconomic, and institutional dynamics in two of Botswana’s remote rural settlements, Khawa and Kedia settlements in Central and Kgalagadi Districts, respectively (Fig. 1). These settlements were chosen for comparison because they were of similarly low economic status and were classified by the

Fig. 1. Location of study settlements Khawa and Kedia, Botswana.

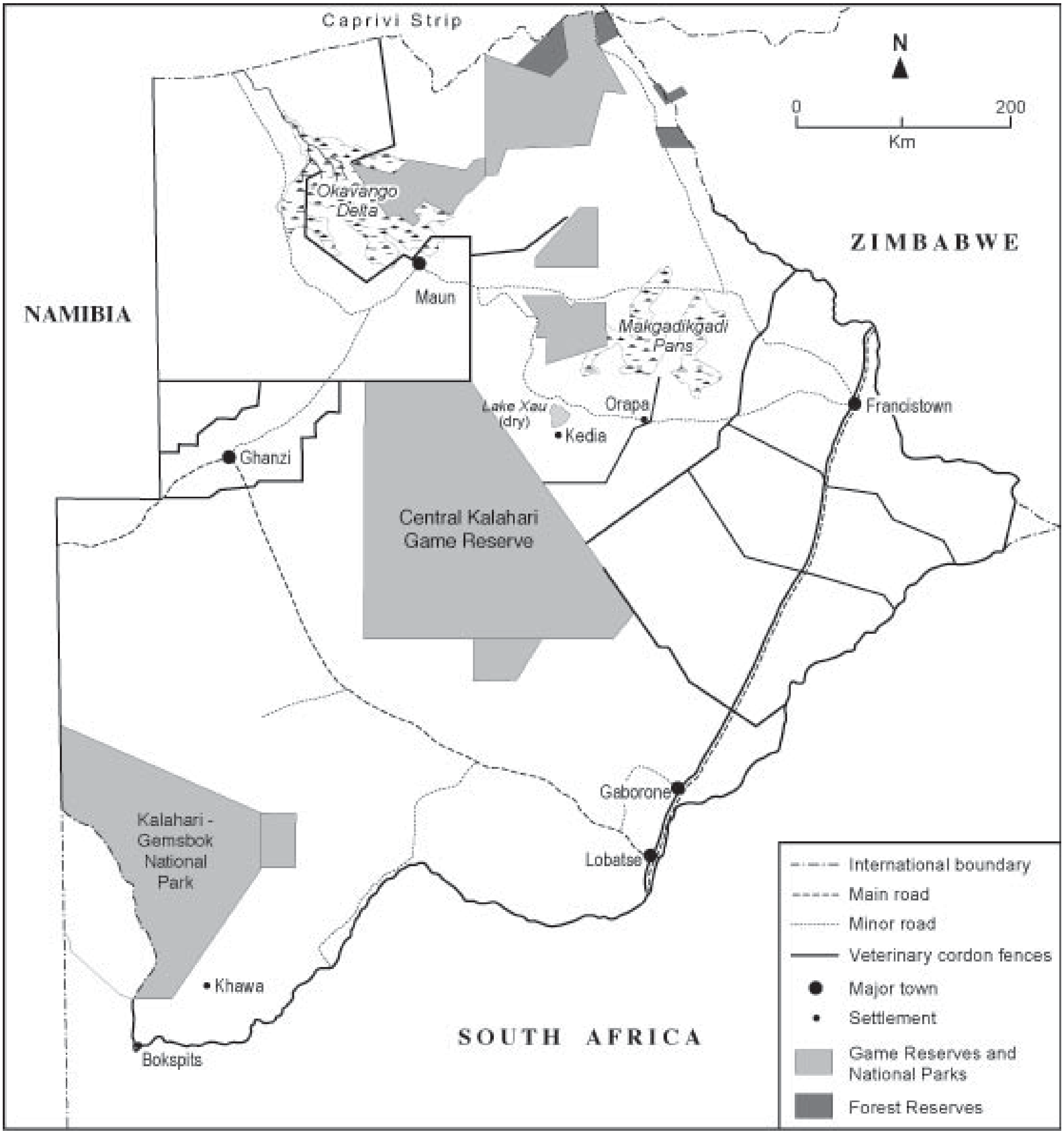

Table 1. Study site descriptions.

Study site	Khawa settlement	Kedia settlement
**Geographical location**	21°21’87.6” S 24°43’80.7” E	26°17’01.3” S 21°22’03.7” E
**Vegetation** (Olson and Dinterstein 2001)	Desert and xeric shrubland	Subtropical grasslands, savannas, and shrublands
**Diversity of soil types**	Low	High
**Hydrology** (Thomas and Shaw 1991)	-	Lake Xau, Boteti River (Makgadikgadi-Okavango-Zambezi rift depression wetlands complex)
**Average annual rainfall between 1995 and 1999**	129.8 mm	386.5 mm
**History of human settlement**	Short – first settled in 1974 when a borehole was dug and equipped; previously, ancestral hunting and gathering grounds	Long – archaeological evidence of settlement dating back to the Middle and Late Stone Age (Cooke and Paterson 1960, Helgren 1984)
**Population** (Census 2001)	510	805
**Population estimated from sampling (2004/5)**	683	1033
**Average number of people per household (2004/5)**	9	6
**Number of people interviewed in survey (number of oral history and trajectory mapping exercises)**	58 (9)	40 (8)
**Ethnic composition (2004/5)** (Sporton and Thomas 2002)	Dominated by Batlharo (74%)	Dominated by mix of Bakalanga (43%), Basarwa (28%), and Bateti (13%) groups
**Primary livelihood options (2004/5)**	Pastoralism (goats, cattle, sheep, donkey); hunting (legal and illegal); veld product collection; employment (mostly temporary); small business (alcohol sales, leatherworks)	Pastoralism (cattle, goats); arable cultivation; employment; small business (alcohol sales, chicken/egg sales, baking, crafts); veld product collection, vegetable gardening; illegal hunting
**Primary food sources (2004/5)**	Wild meat, wild vegetables, destitute rations, livestock, shop-bought foods	Cultivated crops (e.g., maize, sorghum, beans, pumpkin, melon), shop-bought foods, livestock, wild vegetables, destitute rations
**Water availability (2004/5)**	Drinking water rationed and supplied by bowser;<br>livestock water available from open-access community borehole and/or private syndicate borehole	Drinking water available from taps in settlement centre;<br>livestock water available from private wells and open-access seasonal supplies of surface water

government as “remote area dweller” settlements, yet were representative of distinct social-ecological systems with different environmental contexts, social compositions, and histories. Social and environmental characteristics of each settlement are summarized in Table 1. Residents in both settlements had access to surrounding communal lands in order to pursue their livelihoods.

A mixed-method approach was taken in collecting the data. Methods used included oral histories and in-depth livelihood trajectory mapping exercises (n = 17), as well as household-level livelihood and resource use surveys (n = 98). These sought to identify the ways in which households use their environment, how environmental changes (drought, land degradation, etc.) affect livelihood decisions,

and how environmental factors interact with broader socioeconomic and political processes to determine resource use outcomes and impacts on livelihood systems. Repeated vegetation and wild animal surveys were conducted before and after rains, and time-series sets of Landsat images and wild animal aerial count data records were collected from the Department of Surveys and Mapping and the Department of Wildlife and National Parks. Soil and climate data were collected from the Department of Surveys and Mapping and the Department of Meteorological Services, respectively (see Sallu [2007] for a more detailed outline of the methodology and data). Environmental change data were then analyzed in conjunction with livelihood trajectory results in order to elucidate the key dynamics of relationships between livelihoods and the natural resource base. The average time span covered by the investigation was limited to the 30 years between 1974 and 2005, primarily due to restrictions on the availability of climate, soil, vegetation, and large wild animal data. Consideration of this 30-year temporal frame nevertheless permitted incorporation of the periods of formal settlement establishment, which proved important in setting the boundaries of the livelihood context.

Data analysis was conducted throughout the period of information gathering. Initially, this was at a descriptive level in order to note any trends in the data, but it progressed to a more detailed level as both qualitative and quantitative social and environmental information was drawn together. Qualitative data were coded through processes of indexing the data under emerging themes. This permitted the identification of the factors that played an important role in the construction of livelihood strategies. Consistent triangulation of the results highlighted any contradictions and similarities in the different data sources. Where contradictions were found, further iterative reflection took place in the form of focus groups in order to ascertain why and how the conflicts in information may have occurred. This became a circular process that led to inductive interpretation and explanation as the ecological information was gradually juxtaposed within the emergent socioeconomic context. Quantitative data sets were analyzed using multivariate statistics. Livelihood and environmental data were classified using cluster analysis, and correlations were tested using principal components analysis. Landsat images were classified using ERDAS Imagine V.9 software and landscape-level changes were detected from raster attribute comparison (see Sallu [2007] for a more detailed outline of data analysis procedures). Based on this analysis, we aimed to identify contemporary strategies and the nature of trajectories to which they led. In doing this, we also identified the key changes to the vulnerability context and the combination of factors that have led to more resilient or vulnerable livelihood outcomes.

RESULTS - TOWARDS A NARRATIVE OF RESILIENCE

Social-ecological dynamics

The productivity of the natural resource base in dryland Botswana is exceptionally dynamic, with the provision of ecosystem goods and services largely determined by the extreme environmental conditions that affect water, soil, and landscape form. These include large diurnal and seasonal temperature variations, low average annual rainfall (ranging from a minimum of less than 200 mm in the southwest to a maximum of more than 800 mm in the northeast of the country), frequent and extended periods of drought (caused by cyclical [multidecadal] climate factors (Figs. 2 and 3; Thomas and Shaw 1991, Batisani and Yarnal 2010), sporadic heavy rainfall events (Bhalotra 1987, Goomes and Petrassi 1996), edaphic (soil related) variation (Ministry of Agriculture 1990), and hydrologic flux (Fig. 4; du Plessis and Rowntree 2003, McCarthy et al. 2003). In particular, both natural and human-influenced riverine and lacustrine dynamics affect the ecology of landscapes associated with Okavango and Makgadikgadi systems in northern Botswana. These therefore have important impacts on our Kedia study area (Fig. 1). Soils in Khawa and in much of Kedia exhibit low fertility with limited key nutrient contents (nitrogen, phosphorous, potassium). Indeed, much of the country's northern, central, and southern regions are unsuitable for cultivation (Buckley et al. 1987, Tolsma et al. 1987, Ministry of Agriculture 1990). Despite this, soil heterogeneity can be diverse, leading to vegetative diversity equal to that of other savanna areas in Africa (Thomas and Shaw 1991). The resultant human responses to dynamism are manifest in the flexibility of livelihood activities. These are structured in such a way that people are able to take advantage of changing availabilities of and access to natural assets.

Fig. 2. Rainfall variability in Khawa and Kedia between 1975 and 2004 as illustrated by deviations from mean annual rainfall. The 1980s drought period is evident from the recurrent and prolonged period of below average rainfall. Data from nearest weather stations – Bokspits (100 km southwest of Khawa) and Orapa (55 km east of Kedia) – are presented. (Data source: Department of Meteorological Services, Gaborone)

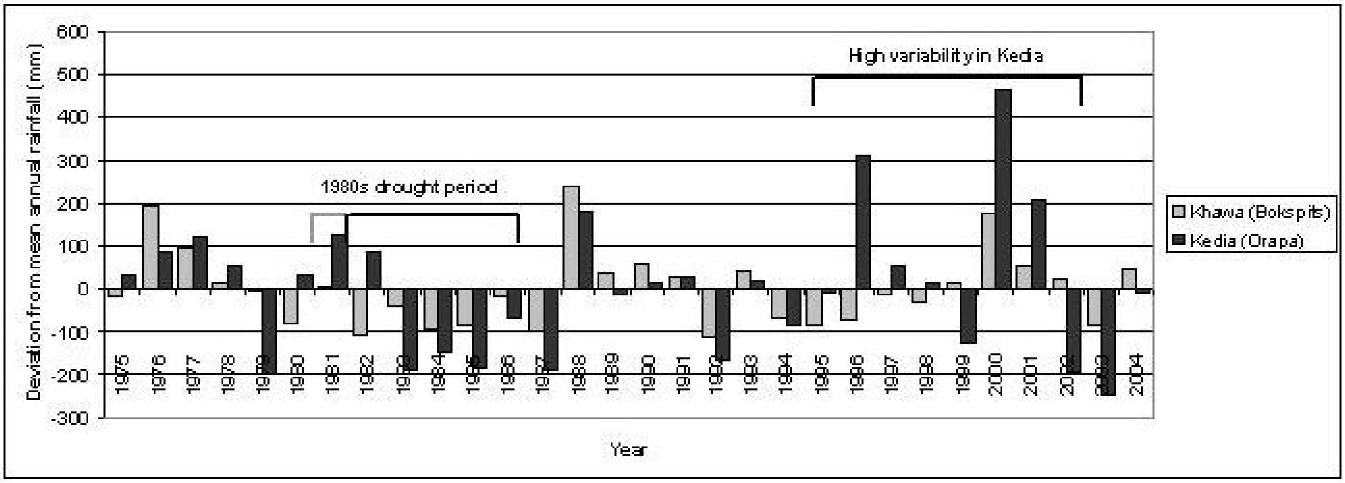

In the rural settlements in our study areas, livelihoods are highly dependent on biodiversity (Sallu et al. 2009), and the environmental dynamics outlined above create both opportunities for and threats to the livelihood strategies that are followed at different times. For example, soil heterogeneity plays a significant role in determining landscape and species diversity. In Kedia, these dynamics have led to a diverse ecological landscape that is in stark contrast to Khawa where the landscape is edaphically homogeneous and less species diverse. The situation in Kedia thus offers more natural resource-based livelihood opportunities than are found in Khawa, across a similar sized landscape.

In both settlements, livelihood activities are strongly influenced by the spatial and temporal variability of rainfall. In Khawa and in far southwestern parts of Kedia, this results in patches, hot- or cold-spots of abundance, which punctuate the landscape and create spatially and temporally distributed opportunities for gathering and hunting. For example, after heavy rain in 2004, hotspots of water-rich veld fruits such as wild watermelon (*Citrullus lanatus*) and cucumbers (e.g., *Cucumis africanus* and *Acanthosicyos naudinianus*) were witnessed in patches of duneveld beyond 13 km from Khawa (Fig. 5). These particular hotspots provide water and nutrient-rich resources for wild animals, livestock, and humans during the dry season. If accessible, these hotspots provide valuable opportunities in times of inner-settlement food and water scarcity, thus making an important contribution to wider livelihood systems (Fig. 6).

Environmental change

The inherent dynamics described above create diversity in otherwise homogeneous and species-poor semi-arid landscapes, and provide spatially and temporally limited opportunities for different livelihood activities. However, longer term and larger scale environmental changes have altered the vulnerability context over the past 30 years and have influenced the livelihood trajectories that households have followed. This section considers five significant environmental changes that have occurred in either one or both of our study settlements over this time period.

1. Mid-1980s drought. Both settlements experienced prolonged drought in the 1980s, beyond the inherent rainfall variability that characterizes dryland environments (Fig. 2). This led to a significant

Fig. 3. Monthly rainfall patterns for consecutive five-year periods in Bokspits (100 km southwest of Khawa), 1975–2004. (Data source: Department of Meteorological Services, Gaborone)

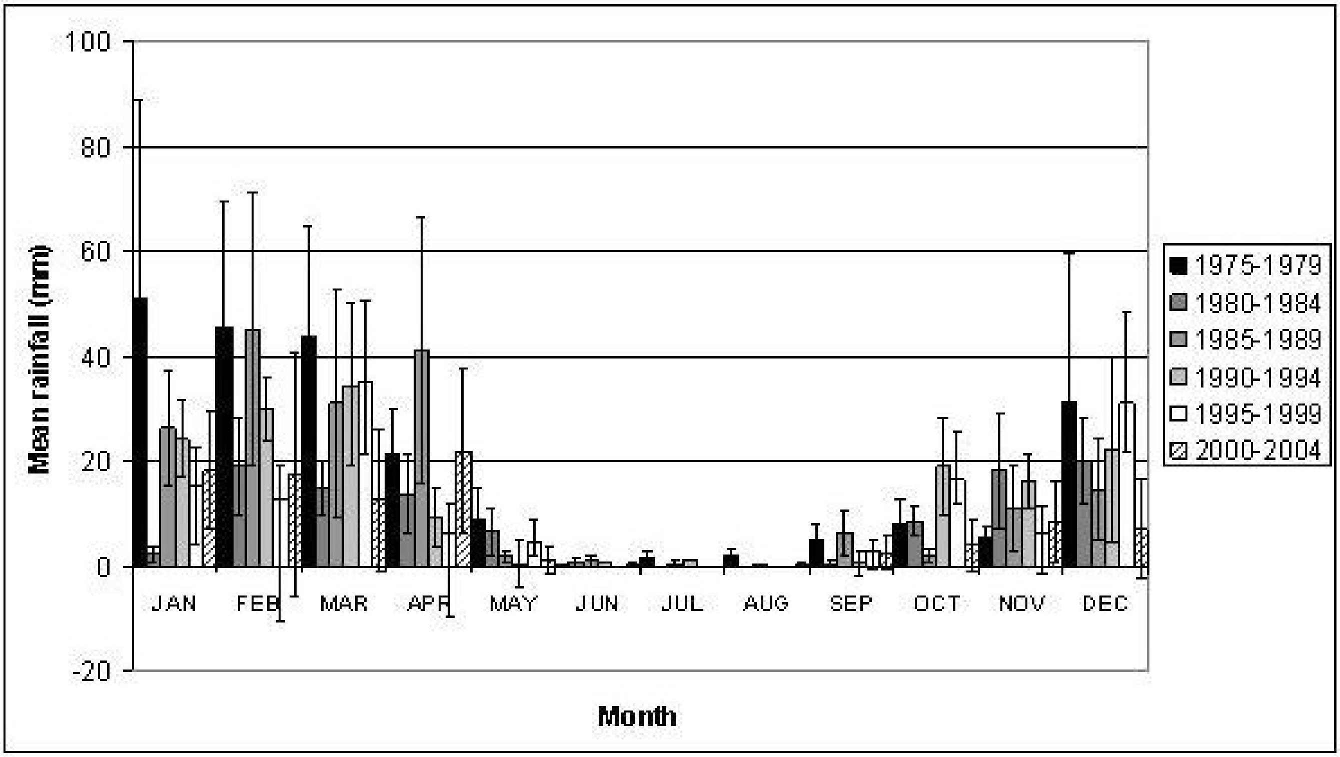

reduction in the diversity and productivity of vegetation (Sallu 2007), and rapid declines in wild animal populations (Table 2; Williamson and Mbano 1988), which limited both the potential for hunting and the availability of grazing and fodder resources for pastoralism. While the ecology in these dryland systems has evolved to withstand drought, the prolonged nature of this event resulted in changes that exceeded the magnitude and extent of that associated with normal rainfall variability for the area. This drought created a shock within the dynamic system that affected both of our study settlements.

2. Late onset of rainfall. While no significant long-term change in average annual rainfall occurred between 1974 and 2004 in either settlement, data obtained from the Department of Meteorological Services show that peak monthly rainfall in Khawa started to fall an average of one month later than usual after 1984. Peaks in mean monthly rainfall between 1985 and 2004 fell in February, March, and April compared to January, February, and March in 1975 to 1984 (Fig. 3). Above average levels of rain fell in 2000–2004 (Fig. 2) with the largest amounts of rainfall occurring in April. This has important implications for the productivity of vegetation growth (Tadross et al. 2005). If peak rainfall periods occur late in the season, low winter temperatures and frosts (usually in June, July, and August) may kill plants before they are fully mature. This has detrimental knock-on effects on the production of human foods, such as wild herbs and fruits, wild medicines, and plant-based materials used as building products and for crafts, and on rain-fed cultivation and the availability of grazing and browse resources for cattle and wild animals. This was an issue of particular concern in Khawa settlement, where diurnal temperatures were greatest. While it is difficult to conclusively link this stress to global climate change due to the inherent dryland rainfall dynamics described above and

Fig. 4. Drying of the River Boteti as illustrated by the deviation from the mean annual volume flow (271 m^3 x 10^6) at Maun (1951–2000). (Source: Sharing Water Okavango/Kubango database http://www.sharing-water.net/)

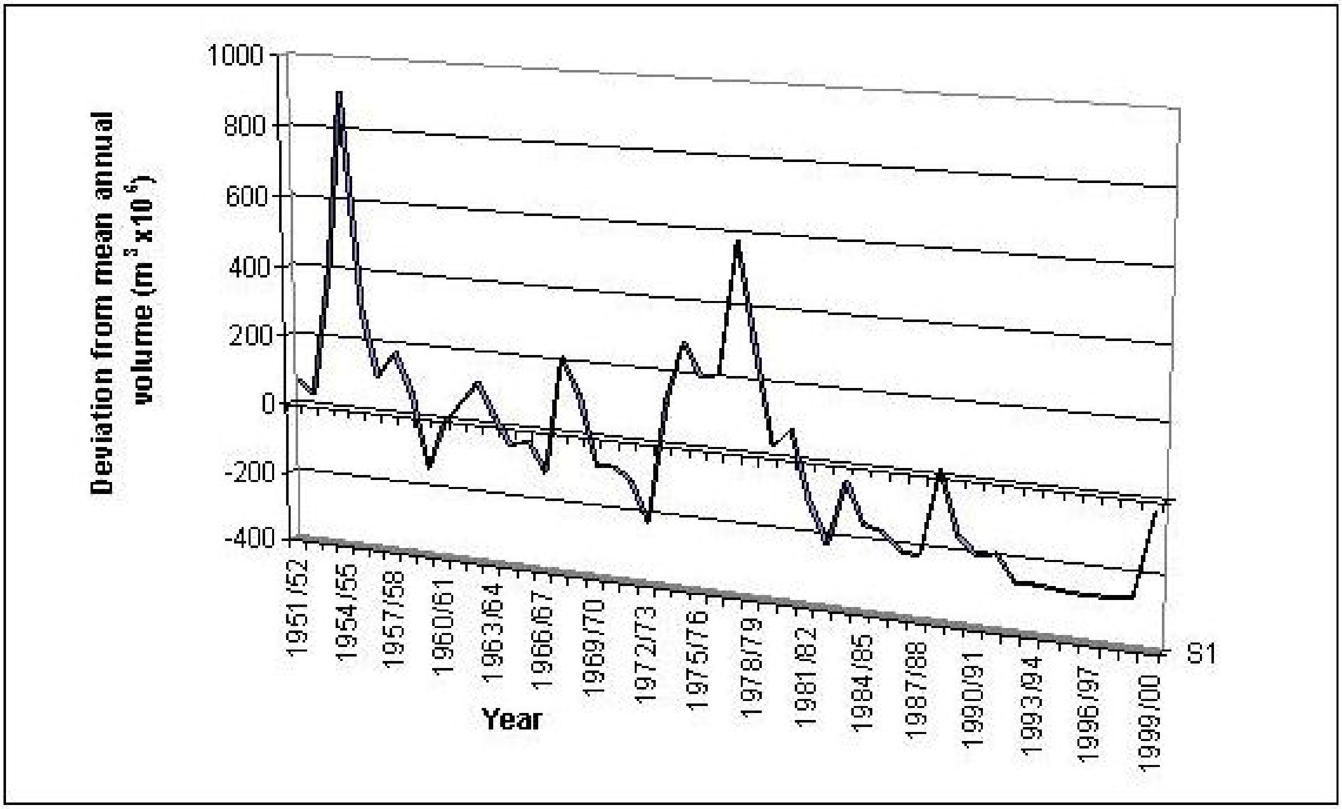

limited data with sparse spatial coverage, Tadross et al. (2007) show increasing evidence that there may be links between climate change and the reduced length of rainfall seasons in southern Africa.

3. Increased unpredictability of rainfall. In Kedia, there was a clear trend towards increased annual rainfall variability after 1996, with greatest volatility noted during the most recent time period, 1996–2004 (Fig. 2). Increased variability has resulted in increased unpredictability of rainfall-dependent natural capital, which has resulted in years of either boom or bust with little in between. In particular, this stress on the system has increased the risks associated with rain-fed cultivation, which has resulted in significant impacts on the provision of livelihood opportunities. No such trend was seen in Khawa.

4. Drying of Lake Xau. During the mid-1980s, in the context of changes to rainfall patterns and prolonged drought, Kedia experienced significantly reduced water flows in the Boteti River (Fig. 4). Coupled with infrastructural developments in the river channel upstream (Zufferey 1983), this contributed to the complete drying of Lake Xau by 1984 (Cashdan 1985). Although in some years the lake has been known to dry up, to date the waters have not returned. This has resulted in the eradication of a seasonal surface water resource and the extinction of fish and water-dependent species such as hippopotamus (*Hippopotamus amphibus*), crocodile (*Crocodylus niloticus*), reeds (“lethaka”), and water lily (*Nymphaea nouchali*) in the area. It has also caused significant changes in vegetation composition, structure, and functioning, and has resulted in the cessation of flood recession cultivation (Sallu 2007). In turn, this added stress

Fig. 5. Dry season hotspot of water-rich veld fruits (*Citrullus lanatus* dominant), 40 km from Khawa settlement, August 2004. Such hotspots are typically positioned in patches that have received repeated localized rainfall events.

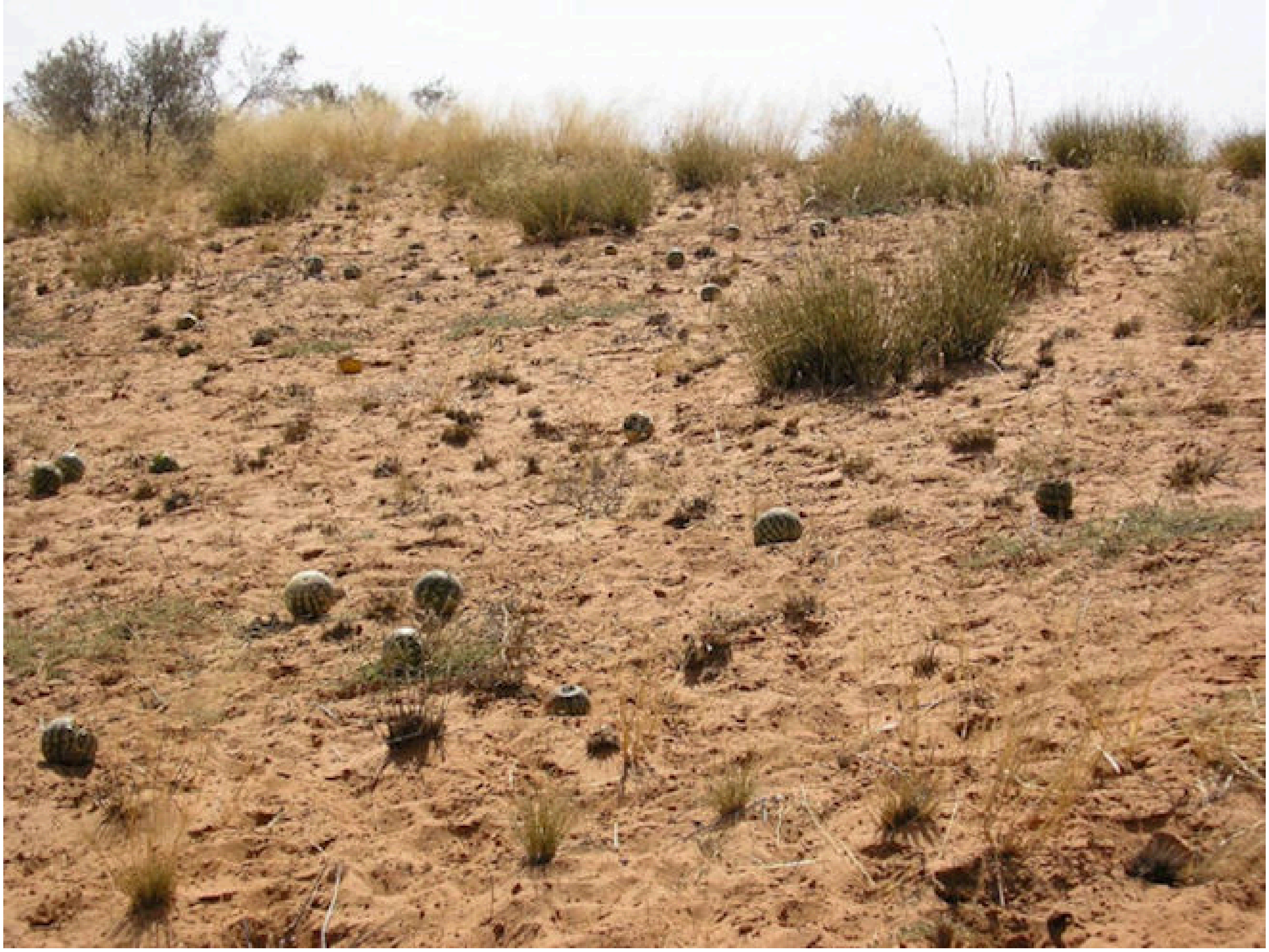

has had an important impact on the provision of livelihood opportunities.

5. Land degradation. In both settlements, land degradation (manifest as soil erosion and compaction, salination, and vegetation changes at a variety of scales) has also become an emerging environmental constraint through the associated decline in productivity it represents. Data from vegetation surveys illustrated that degradation was most typically recorded near settlements, cattle posts, watering holes (boreholes or wells), and transport routes (roads and tracks) (Sallu 2007). As was seen in Kedia during the mid-1980s, temporary deterioration of the land was also commonplace, particularly in areas seasonally frequented by large numbers of wild herbivores (Sallu 2007). This additional system stress meant that herders had to travel further afield to access water and suitable grasses for their cattle, while opportunities for hunting and arable cultivation as livelihood activities also decreased.

Fig. 6. Villagers separating *Citrullus lanatus* flesh and seeds at a temporary camp, 40 km from Khawa settlement, August 2004. Flesh is heated to form water, and the seeds are pounded into flour for porridge.

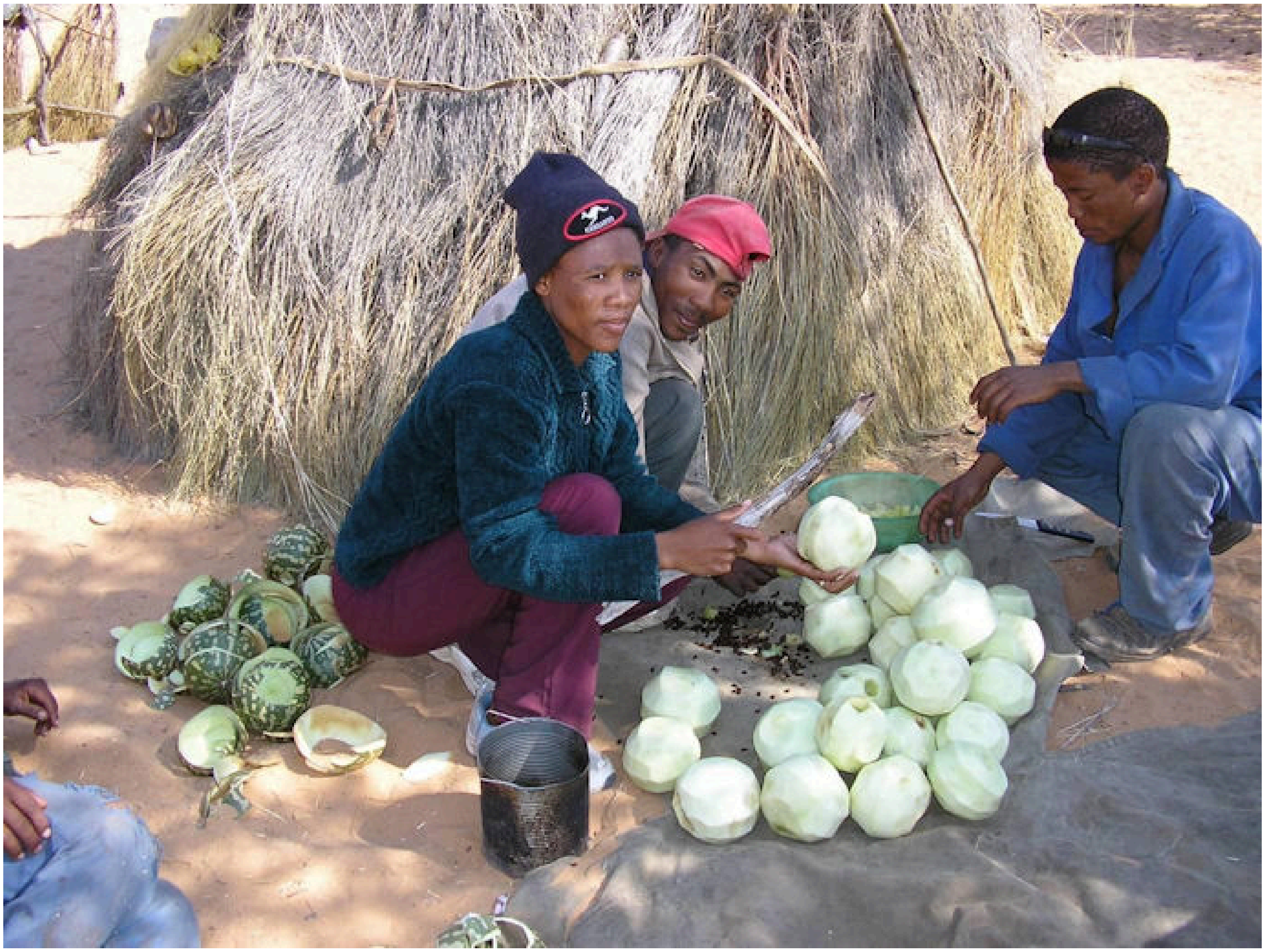

Contemporary livelihood strategies

In the context of these environmental dynamics and livelihood struggles, and despite significant socioeconomic and cultural differences within settlements (Table 1), three major groups of households, conducting similar or related livelihood activities, were identified in 2004/5 (Fig. 7). These strategy groups were classified using cluster analysis of household livelihood survey data. A description of the key characteristics of the households and livelihoods of each cluster group is presented in Table 3. Principal activities that determined strategy differentiation based on principal components analysis can be linked to Fraser et al.'s (2010) vulnerability framework, and included (1) ownership of livestock (Fraser et al.'s "access to assets"), (2) cultivation of arable crops (Fraser et al.'s "capacity of ecosystem to remain productive"), (3) reliance on government-provided social security benefits[1] (Fraser et al.'s "strength of formal and informal institutions"), and (4) permanent and temporary employment (Fraser et al. 's "access to assets") (Table 3).

Table 2. Population change of selected wild animal indicator species between the 1970s and 1994 for the southern Kalahari system associated with Khawa (data sources: DHV [1980] and DWNP [1994*a*, *b*]; modified from Boggs [2000]) and the northern Kalahari system associated with Kedia (data sources: Van Der Maas [1995] and Bonifica [1992]). CKGR = Central Kalahari Game Reserve; MPNP = Makgadikgadi Pans National Park. "-" refers to no data available.

Species	Population change Southern Kalahari (1978–1994) (%)	Population change CKGR (1979–1994) (%)	Population change MPNP (1973–1994) (%)
Zebra (*Equus burchelli*)	-79.1	-	-58.5
Red hartebeest (*Alcelaphus buselaphus*)	-84.8	-95.8	-
Blue wildebeest (*Connonchaetes taurinus*)	-94.3	-87.1	-93.1
Springbok (*Antidorcas marsupialis*)	-33.2	+375.1	-99.5
Kudu (*Tragelaphus strepsiceros*)	+22.1	-	-65.1
Common ostrich (*Struthio camelus*)	-70.0	-66.6	-64.2
Eland (*Taurotragus oryx*)	-	-8.2	-
Gemsbok (*Oryx gazelle*)	-	-0.8	-24.3
Giraffe (*Giraffa camelopardalis*)	-	-59.8	-

A small number of households (13% in Khawa and1 7% in Kedia) with a tendency to specialize and thus accumulate large numbers of livestock, described here as "accumulators", existed in each settlement (Fig. 7). In many cases, accumulator households accrued income mainly through permanent and/or temporary employment and livestock (e.g., Case 1, Table 4). In Kedia, investment was also directed to arable cultivation, with money being spent on inputs such as land, seeds, and/or labor (e.g., Case 4, Table 4). As well as occupying financially superior positions within communities, accumulators were often politically powerful: most either currently or in the recent past had assumed a leadership role (e. g.,village chief, Village Development Committee chairman, Remote Area Dweller Programme assistant, councillor) in the settlement (e.g., Cases 1 and 4, Table 4). This sector of society therefore represented a politically as well as economically influential sector of the community, similar to Peters' (1984) "rural elite". Ethnic bias was noted too. Elite accumulators were generally composed of dominant Batlharo and Bakalanga descent in Khawa and Kedia, respectively (Table 3).

A more varied strategy, undertaken by what we have called "diversifiers", was followed by 26% of households in Khawa and 25% of households in

Fig. 7. Proportion of households in livelihood-determined cluster groups (strategy groups) and respective mean livestock unit (LSU) values for each group in Khawa and Kedia in 2004 and 2005. Refer to Table 3 for full description of cluster group livelihoods. Livestock units: 1 cow = 1 LSU; 5 goats/sheep = 1 LSU (Sallu 2007).

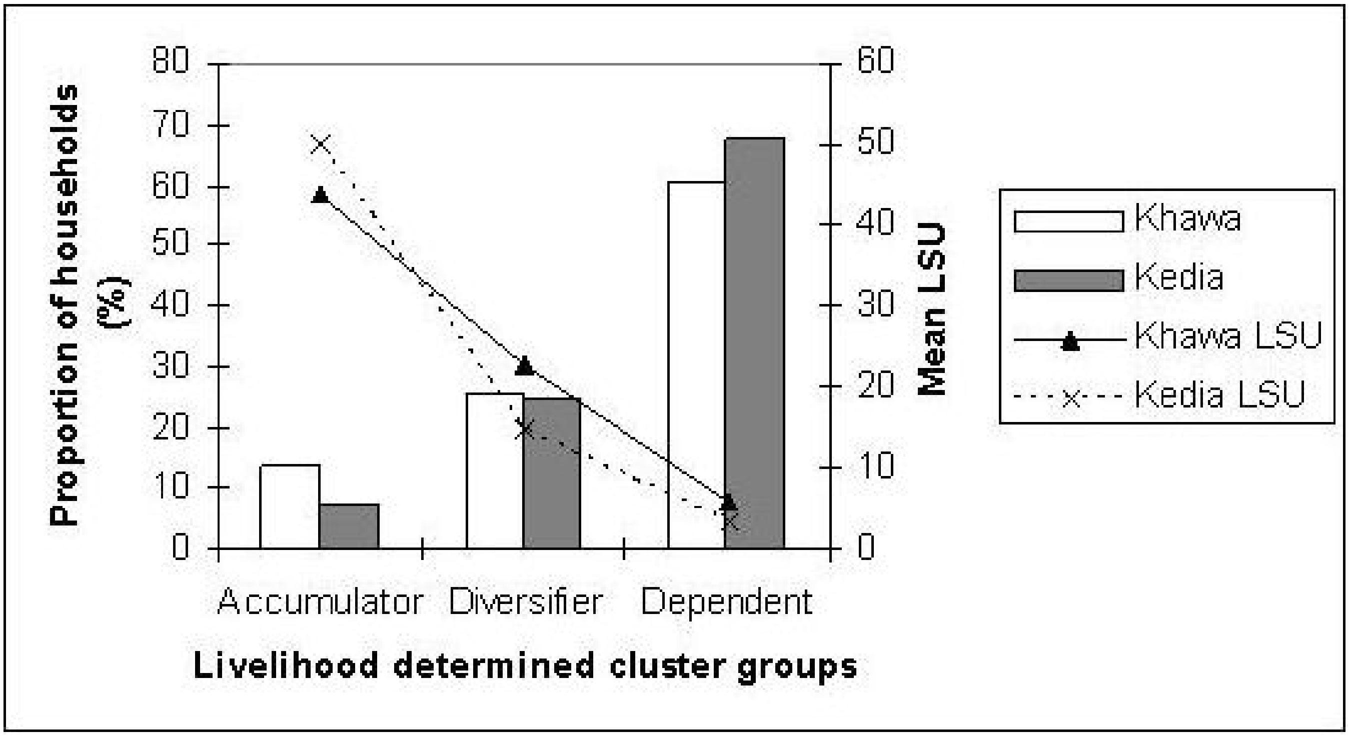

Kedia (Table 3, Fig. 7). These households tended to distribute effort across multiple livelihood activities, with lesser tendency towards specialization (reflected by moderate livestock units, Fig. 7). While these households were typically composed of a range of ethnic groups, minority groups, such as Bakgalagadi in Khawa and Banajwa and Bakwena in Kedia, were absent.

Finally, a “dependency strategy”, undertaken by “dependents”, was followed by the remaining households. Households within this group were characterized as smaller than average in size, with low (e.g., 10) to zero livestock units, and were highly dependent on social security benefits. In Kedia, household members were also frequently employed as herders or laborers for other members of the community, often based outside the settlement (Table 3, Fig. 7). This category comprised the majority of households in both settlements (Fig. 7), and households were typically composed of a range of ethnic groups. In both settlements, this included minority groups, and in Kedia, involved a high proportion of Basarwa (Table 3).

Livelihood trajectory analyses

As a means of teasing out the relative importance of each of the multiple interacting factors that resulted in the contemporary livelihood strategies presented above, detailed investigation of livelihood trajectory data for 17 households across the two settlements was conducted for the 1974–2005 period. Some of the trajectories encountered are illustrated through the narrative cases presented in Table 4. While Table 4 clearly illustrates that trajectories were unique to households, some generic trends were evident. These trends are described below.

Table 3. Livelihood strategies associated with household cluster groups, and the principal activities determining each strategy for Khawa and Kedia in 2004 and 2005. Principal activities were identified using principal components analysis. Differentiation of livelihood activities between cluster groups was more significant (clear cut) in Kedia than in Khawa, with a greater proportion of the cumulative percentage of total variance explained in Kedia than in Khawa at each level of analysis.

Settlement	Khawa			Kedia		
**Household cluster group**	Accumulator	Diversifier	Dependent	Accumulator	Diversifier	Dependent
**Livelihood strategy**	Diversified strategy with tendency to specialize	Diversified strategy with tendency for dependency	Dependency strategy	Specialized strategy	Diversified strategy	Dependency strategy
**Principal livelihood activities differentiating clusters (in order of significance)**	Livestock specialization often accompanied with permanent employment and/ or commercial business	Involved in broad range of activities; greatest dependence of all clusters on child benefits	Dependent on destitute relief, orphan relief, pensions, and drought relief	Livestock and arable cultivation specialization	Employment, commercial business, livestock, and/or cultivation	Dependent on destitute relief, orphan relief, pensions, and drought relief, as well as household level income strategies (e.g., alcohol brewing, baking) and laborer activities (e.g., herding/ digging wells)
**Social Identity**	Pastoralist<br>Hunter<br>Employee (mainly working for government)<br>Syndicate members<br>Settlement leader	Pastoralist<br>Hunter-gatherer<br>Herder<br>Destitute	Destitute<br>Hunter-gatherer	Agro-pastoralist	Employee<br>Agro-pastoralist<br>Hunter	Destitute<br>Labourer
**Ethnic Identity**	Mixed (Bakgalagadi absent)<br>50% Batlharo<br>38% Bakgatla<br>12% Other	Mixed (Bakgalagadi absent)<br>73% Batlharo<br>7% Bakgatla<br>20% Other	Mixed (Bakgalagadi present)<br>77% Batlharo<br>9% Bakgatla<br>9% Bakgalagadi<br>5% Mix	Bakgalanga<br>100% Bakalanga	Mixed (minority groups absent)<br>40% Bakalanga<br>20% Bakurutse<br>20% Basarwa<br>20% Bateti and Bakgalagadi	Mixed (includes a high proportion of Basarwa and all minority groups)<br>37% Bakalanga<br>33% Basarwa<br>15% Bateti<br>7% minority groups (Banajwa and Bakwena)
**Average household size (average number of adults absent from settlement in 2004/5)**	10.5 (1.4)	10.5 (1.4)	9.1 (1.5)	6.3 (1.7)	6.7(1.6)	6.2 (0.9)

Table 4. Livelihood dynamics and trajectories of example case study households.

Case study household	Trajectory (through time, T) and factors leading to resilience (R) and vulnerability (V)
Case 1 – Mr. Thau, Khawa	*Accumulative trajectory – building resilience*
Batlharo, aged 54, living with his wife, seven youth, and 10 additional children	T1, 1985 – Limited assets, low capacity of agroecosystem to remain productive, some collective capacity to cope through social security benefits
Thau moved to Khawa in 1985 with his wife and young children. They moved to benefit from the government support available. This included food and schooling for the children, in particular. At this time, Thau had 25 goats, two donkeys, and a donkey cart. Once settled in Khawa, Thau’s family also started collecting veld products, making leatherworks, and brewing and selling traditional beer. In 1989, Thau’s family stopped their leatherwork activities because an expensive licence was imposed on the sale of products. This did not significantly affect the household because that year Thau became Councillor for five years. He earned 700 Pula per month. He invested much of this money in livestock. He bought two cows in 1990, and then every month he bought a goat. He also invested money in a car and a syndicate-run borehole. His wife cared for the livestock when he worked, and by 1994, he had 150 goats and 10 cows, despite some losses to wild animals (four cows killed by lions in 1992 but compensation from the Wildlife Department was granted). While Thau was working, his wife and children collected veld products to supplement their diet. In 1994 and 1995, rains fell late so no veld products were available. Thau also depended on piece jobs and drought relief work during the 1990s – e.g., between 1994 and 2002, Thau was a Member, and later Chairman of the Village Development Committee. In these roles, he received cash sitting allowances. He continued to invest his income in livestock and began paying a monthly fee to have a private water tap in his compound. In 1999, Thau’s wife died, but in 2001 he remarried the Health Clinic cleaner. In 2004, additional household income was achieved when Thau’s daughter started working as the Manager of the Co-operative Shop. In 2004, due to low rainfall and degradation around the settlement, grazing and water resources were limited. Due to his access to transport in the form of a donkey cart and car (which facilitated the transport of water and food for those caring for the livestock), Thau sent his livestock 40 km west to access an area of rangeland that had benefited from rain earlier in the season (Fig. 5). This area provided sufficient wild watermelons (*Citrullus* sp.) and grazing to sustain his and other household herds for up to three months during the dry season. The extra nutrients gained from these resources enabled his goats to reproduce twice in that year, leading to rapid growth in numbers.	**R1. Diversification of livelihood activities**<br><br>T2, 1990s – rapid accumulation of financial, physical, and human assets, moderate capacity of agroecosystem to remain productive (rainfall higher but degradation increasing), increasing individual and collective capacity to respond to crises as he became Councillor and his children got older<br><br>**R2. Salaried employment**<br>**R3. Investment in livestock accumulation**<br>**R4. Investment in transport**<br>**R5. Investment in access to water**<br><br>T3, 2000s – continued accumulation of financial, physical, and human assets (children gained jobs), decreasing capacity of agroecosystem to remain productive, capacity to respond to agroecosystem decline high due to access to transport and private water source
Case 2 – Mr. Mpoelang, Khawa	*Fluctuating trajectory*
Batlharo, aged 74, living with his wife, an adult son, and two children (one of whom has Down’s syndrome)<br><br>Mpoelang was one of the first settlers in Khawa in the late 1970s. At that time, the rains were so plentiful and appropriately timed that he was able to cultivate melons and beans near the settlement. He also hunted for wild meat and made leatherworks, and the household sold the leathers and dried meat (biltong) across the border into South Africa.	T1, late 1970s – high capacity for agroecosystem to remain productive (high and appropriately timed rainfall, low numbers of people and livestock, and no degradation), high levels of access to natural assets (horses), moderate to high accumulation of financial or physical assets (car, livestock), moderate capacity to respond (social networks, no trade or resource-associated restrictions, no formal institutions, transport)

(con'd)

At that time, Mpoelang had 30 cows, 18 horses, and a car. In the mid-1980s, trade was no longer allowed across the border, and he ceased cultivation due to the drought and inappropriate timing of rainfall. In 1984, he also lost 15 cows and 18 horses to drought. In 1987, his household was not allocated a Special Game Licence, and as a consequence they were no longer able to hunt. Despite this, Mpoelang was still able to make leatherworks by purchasing skins from those who were hunting, and when the licence for selling leatherwork products was introduced, he purchased one. In the early 1990s, Mpoelang's livestock numbers slowly began to recover. There was, however, a setback in 1993 when eight of his cows were struck by lightening. Between 2000 and 2003, Mpoelang lost an additional 11 cows to lion predation. Financial compensation from the Wildlife Department, access to pensions, and the sale of horses for cows allowed some recovery of cattle stocks. In 2000, Mr. Mpoelang's household started to benefit from the quota system, with a small share of meat from the community-allocated hunt available for his household. In 2003, the household successfully cultivated rain-fed melons on a small scale near the house. In 2004, the household retained three horses and seven cows. The child with Down's Syndrome did not receive support from the government.	**R1. Accumulation of livestock in high rainfall years**<br>**R2. Cultivation of crops in years of appropriately timed rainfall**<br><br>T2, mid-1980s – low capacity of agroecosystem to remain productive (prolonged drought, inappropriate timing of rainfall), limited natural assets (drought), some physical and financial asset stores (leatherworks), moderate capacity to respond (reduced from high by permits restricting hunting and by border restrictions to trade)<br><br>**V1. Loss of livestock**<br>**V2. Loss of livelihood activity**<br><br>T3, 1990s – moderate capacity of agroecosystem to remain productive, fluctuating financial, physical, and natural assets, changing institutional support to assist household's capacity to respond<br><br>T4, 2000s – some capacity of agroecosystem to remain productive (ability to cultivate), some financial and physical assets (horses), moderate capacity to cope (trade horses for cows), high capacity to cope (access to pensions, compensation, quota meat, and pension)<br><br>**R4. Diversity of livestock types (spread risks)**<br>**R5. Access to government support mechanisms to supplement livelihood activities in elder years**
Case 3 – Mr. Bakghotu, Khawa	*Degenerative trajectory – vulnerable*
Bakgalagadi, aged 66, living with his wife and no children<br><br>Bakghotu first came to Khawa in 1974 with his wife. At that time he was hunting and he and his wife were making leatherworks from the skins of bat-eared fox (*Otocyon megalotis*), black-backed jackal (*Canis mesomelas*), and springhare (*Pedetes capensis*). Bakghotu and his wife were making cushions and rugs and selling them to people for money. In addition, they were collecting veld products for food and wild medicines for medicine and sale. Bakghotu was also engaged in woodcrafts. Between 1980 and 1992, as a destitute, he and his wife also received a Special Game Licence, which allowed them to hunt. Also, the remote area dweller (RAD) program provided his household with food rations, clothing, and blankets. Between 1987 and 1988, Bakghotu was also employed for a short time on piece jobs. These combined activities allowed him and his wife to purchase a few livestock – goats, donkeys, and horses. Bakghotu described himself as making "a very nice living up until the changes in licences". From 1989, Bakghotu was no longer able to sell his leatherworks without a licence, and from 1992 when the Quota Hunting Licenses were introduced, he was no longer able to hunt for himself. Even in more recent years, a licence has been introduced to prevent the sale of devil's claw (*Sengaparile* – a medicinal plant), and the other veld products Bakghotu used to collect for food, medicine, and crafts are now located far from the settlement due to increased degradation. These combined restrictions have severely limited the range of activities he is able to practice for his livelihood, and with the loss of his livestock due to drought and predation, he is now dependent on the government. He relies heavily on the old-age pension and destitute rations but complained of shortages in the destitute rations and reductions in the money provided to those in need.	T1, 1974 – high capacity for agroecosystem to remain productive, high levels of access to natural assets, moderate accumulation of financial and physical assets (livestock), moderate capacity to respond (asset stock)<br><br>**R1. Engagement in diverse livelihood activities**<br><br>T2, 1980s – low capacity of agroecosystem to remain productive, retained access to some natural assets (hunting permit), limited access to other natural assets (drought), some physical and financial asset stores, moderate capacity to respond (RAD program support)<br><br>**V1. Loss of livestock**<br><br>T3, 1990s – moderate capacity of agroecosystem to remain productive, loss of financial and physical asset stores, loss of access to natural assets (permit changes), increasing reliance on government support<br><br>**V2. Loss of livelihood activity**<br><br>T4, 2000s – some capacity of agroecosystem to remain productive, no asset stocks, total reliance on government support<br><br>**V3. Sole reliance on government support**

(con'd)

Case 4 – Mr. Mathoa, Kedia

Bakalanga, aged 51, widowed, living with three youth (two present and one absent at time of survey), and three children

Between 1976 and 1979, Mathoa worked in Orapa. At this time, he had very few livestock. In 1979, when he finished working, he invested the money he had saved into livestock, mainly cattle and goats, the digging of a well, and some cultivation. Between 1979 and 1984, the number of livestock units he owned increased from 19 to 57 due to the availability of good grazing, browse, and access to water. He also managed to accumulate land. However, between 1984 and 1994, he lost 50% of his livestock to drought, and no cultivation was possible. During this time, Mathoa gained employment with the Land Board and married a woman who was employed as a nurse at the clinic. The financial capital accumulated from both forms of employment allowed re-investment in livestock and land after 1994. By 2005, he had 15 hectares of land (12 hectares on one side of the settlement, three on the other side). The number of livestock units Mathoa accumulated peaked at 72 in 2000 after a particularly good rainfall year. Cultivation of sweet reed, maize, sorghum, watermelon, and beans was also possible that year. Food was generated for both subsistence and sale. During this period, Mathoa finished working with the Land Board and was retrained as a welder. He also received small amounts of money at this time in his role as Village Development Committee (VDC) chairman. The income generated through self-employment as a welder and from the VDC enabled him to maintain his livelihood status despite the death of his wife in 2004 and a drop in livestock units to 53 in 2005 after two successive years of below average rainfall. Some years (e.g., 2001), he was unable to harvest due to pest attack on crops.

Stable trajectory - retained resilience

T1, late 1970s–early 1980s – high capacity of agroecosystem to remain productive, access to and accumulation of financial and physical assets, moderate capacity to respond to change

R1. Salaried employment
R2. Accumulation of livestock
R3. Improved access to water
R4. Accumulation of agricultural land

T2, 1984–1994 – low capacity of agroecosystem to remain productive (prolonged drought and lakeside degradation), loss of some physical assets (livestock), high capacity to respond (employment, powerful social network)

V1. Loss of livestock

T3, 1994–2005 – moderate capacity of agroecosystem to remain productive (some rainfall but increasing unpredictability of rainfall, degradation), accumulation of financial and physical assets, high capacity to respond (self-employment, retained and expanding social networks)

R5. Salaried employment
R6. Income generation from self-employment
R7. Re-accumulation of livestock
R8. Accumulation of agricultural land
R9. Cultivation of a range of food crops
R10. Sale of some of the harvested crop

Case 5 – Mr. Mmegwa, Kedia

Bakalanga, aged 76, married and living with one wife, four youth, and five children

Mr. Mmegwa worked for six years in South African mines. Between 1951 and 1975, he lived in Xhumo and Beetsao. He was a healthy man who owned land and small shops. His household used to cultivate enough to sell and managed to dig a well from the money this generated. Since their move to Kedia in 1975, they have not experienced a good harvest. Instead, livelihood activities at this time specialized in livestock farming and small amounts of hunting around the lake. Livestock units in this household peaked at 163 in 1991. Mr. Mmegwa stopped hunting around this time because he considered himself to be rich. In 1993, however, Mr. Mmegwa’s well was stolen and sold by someone else without his knowledge. This resulted in the death and/or loss of all of his livestock and the reliance on the only other livelihood activity that contributed significantly to the household at that time – his wife’s brewing and sale of alcohol. This activity protected the household livelihood from collapse, and over time, in combination with the collection and sale of medicinal plants and Mr. Mmegwa’s monthly receipt of an old-age pension, generated enough money to purchase livestock and access water once more. By 2005, the numbers of livestock in the household had reached 11 cows, 16 goats, six donkeys, two horses, and 10 chickens. Despite several of Mmegwa’s children being away from the settlement, either working or studying, none of them send remittances.

Fluctuating trajectory with shift from accumulator-diversifier strategy

T1, 1975–1991 – fluctuating capacity of agroecosystem to remain productive, access to and accumulation of natural assets, high capacity to respond to change (financial and physical capital stores, social network outside settlement)

R1. Accumulation of livestock

T2, 1993 – moderate capacity of agroecosystem to remain productive (some rainfall but increasingly unpredictable, degradation, lake dry), loss of access to water, moderate capacity to respond (skills and knowledge to diversify activities)

V1. Loss of livestock
R2. Diversification of livelihood activities

T3, 2005 – moderate capacity of agroecosystem to remain productive (some rainfall but increasingly unpredictable, degradation, lake dry), moderate access to assets, moderate capacity to respond (diverse skills, access to government support)

R3. Reaccumulation of livestock

(con'd)

Case 6 – Mr. Baitsile, Kedia	*Fluctuating trajectory*
Basarwa, aged 57, married and living with one wife, one other adult, three youth, and eight children<br><br>During the 1970s, Baitsile’s household livelihood was composed of a range of livelihood activities including fishing, hunting, cultivation, livestock farming, and veld product collection. Of greatest significance at that time was cultivation and veld product collection. In a good season, he was able to cultivate up to ten 50-kg bags of maize, ten 50-kg bags of sorghum, and eight 50-kg bags of beans, as well as plentiful supplies of pumpkin (maputse), green melon (marotse), and watermelon (moghapo). Veld products collected after rain included the fruits of *Grewia* species (mogwana, moretlwa/ moseme, motsotsojane) and moretologa (*Ximenia americana*), as well as the Mophane worm. Baitsile was able to sell some of these natural products. However, he described how in more recent time periods (1990 onwards) veld products had become increasingly scarce near the settlement due to the numbers of people collecting such resources. During the mid-1980s, Baitsile was unable to cultivate due to poor rainfall, he was no longer able to fish due to the lake drying, veld products were significantly reduced due to low rainfall, and he was no longer able to hunt due to permit changes. His livestock unit declined from 13 in 1981 to none in 1991. In 1991, the only activity that kept the household going was government-provided employment in the form of piece jobs laying water pipes. Between 1997 and 1999, Baitsile was employed by the Community Hunting Project. In 2000, after this project had ceased, the government gave Baitsile 15 cows. The number of livestock has remained similar since. In 2005, Baitsile gained employment as a night watchman at the settlement shop.	T1, 1970s–early 1980s – high capacity of agroecosystem to remain productive, access to and accumulation of natural and financial assets, moderate capacity to respond to change<br><br>**R1. Accumulation of a variety of natural asset stocks**<br>**R2. Cultivation of range of crops**<br>**R3. Supplementary collection of wild food**<br><br>T2, mid-1980s – low capacity of agroecosystem to remain productive (drought, degradation, dry lake), reduced access to natural assets (permit changes, drought, dry lake), no ability to respond to change (low to no asset stocks)<br><br>**V1. Loss of livelihood activity(ies)**<br><br>T3, 1990s–2005 – moderate capacity of agroecosystem to remain productive (some rainfall but increasingly unpredictable, degradation, lake dry), some access to natural assets, moderate capacity to respond (government support and employment)<br><br>**R1. Salaried employment**<br>**R2. Re-accumulation of livestock**
Case 7 – Mr. Charlie, Kedia	*Diversified trajectory – increasing resilience*
Basarwa, aged 34, married and living with one wife and two young children; lives adjacent to his sick mother and elderly stepfather<br><br>When Charlie was growing up (late 1970s–early 1980s), the family's food came from the lake. They used to eat reeds and fish. When the lake dried, life changed, and he can remember being hungry as a child. Charlie was given one cow and one goat by relatives when he started his own household, and by 2005 there were five cows and four goats. Some died that year due to a lack of water. Charlie currently lives approximately 10 km outside the center of the settlement in an area of Mopane woodland. He spends much of his time now (2005) helping his sick mother care for her livestock, collecting and preparing medicines and woodcrafts, and illegally hunting for small game. Cultivation has not been possible since 2000 due to the irregular rainfall, and even when it is possible, his household has only a small amount of land to cultivate. Charlie’s household has access to a well, which is a shared resource with other Basarwa families. His children are cared for by the government during school term time. They come home during holidays. The elder parents both receive a pension.	T1, late 1970s–early 1980s – high capacity of agroecosystem to remain productive, access to and accumulation of natural assets, low capacity to respond to change<br><br>**V1. Use of wild foods (lake products)**<br><br>T2, mid-1980s – low capacity of agroecosystem to remain productive (drought, dry lake), reduced access to natural assets (drought, dry lake), some ability to respond to change (family support, livestock)<br><br>**V2. Loss of livestock**<br>**V3. Loss of wild lake foods**<br><br>T3, late 1980s–2005 – moderate capacity of agroecosystem to remain productive (some rainfall but increasingly unpredictable, degradation, lake dry), good access to natural assets (living outside settlement), high capacity to respond (diverse skills, family support, access to government support)<br><br>**R1. Diversification of livelihood activities**<br>**R2. Accumulation of livestock**

Khawa settlement

Between 1974 and 2005, trajectories of elite accumulator households in Khawa were primarily aimed at building up asset bases, with periodic peaks and troughs due mainly to a gain or loss of employment, livestock disease, and/or drought (e. g., Case 1, Table 4). Asset accumulation in Khawa focused on the conversion of employment income into physical assets, primarily the expansion of livestock herds, and investment in improved access to water and transport. It is well known that in some arid and semi-arid environments (in particular those where stock mortality is density independent) the maximization of stocking densities helps ensure long-term survival after drought stock loss (Campbell et al. 2006, Barrow et al. 2007). With only a small number of elite accumulators and an expansive communal rangeland landscape, which is largely functionally intact beyond 4 km of the settlement, cattleposts, or boreholes, this accumulative strategy led these household livelihoods to become increasingly resilient to environmental changes over time. In addition, investment in improved access to water increased household resilience to drought-induced water stress. Access to transport (in particular a vehicle) improved resilience to degradation as well as drought. The availability of transport improved access to areas outside the settlement that were rain-fed and/or most ecologically intact. Transport also facilitated access to external institutions and distant markets for the direct sale or purchase of goods. As socially and politically elite members of the community, members of accumulator households were best placed to predict, monitor, and adapt to economic and institutional changes, and therefore were most likely able to position themselves to achieve the most resilient trajectories in the face of environmental change.

Over the same time period, many households in Khawa that followed a dependency strategy had not demonstrated such resilience in their livelihoods (e. g., Case 3, Table 4). These households were especially vulnerable to the combined impacts of drought and degradation, particularly over the past 15 to 20 years, with many failing to recover from the prolonged mid-1980s drought. A lack of financial income and limited access to water and transport coupled with a range of institutional and policy-related changes (one of the factors that Fraser et al.'s vulnerability framework identifies as a key for the successful management of environmental shocks) meant that these households had not been able to overcome drought shock or degradation stress. The most significant institutional and policy changes that have affected these households in Khawa have been (1) changes to the hunting licensing system on hunting-associated livelihoods, (2) settlement-specific trends towards elite capture of productivity hotspots and water resources in the settlement, (3) failure in the effective provision of government support, and (4) breakdown of social capital within the community. Both (1) and (2) have reduced nonaccumulator households' access to water and ecological diversity, which has limited their options with regard to the livelihood activities they can pursue and has increased their vulnerability to drought and degradation. Shifts to a new quota licensing system in Khawa have provided more equitable community-wide access to wild animal (hunting) resources but have preferentially benefited elite accumulator households, who were previously expected to purchase licences, and negatively affected the less successful households, who now experience more limited access to this resource and more limited opportunities to generate income and/or obtain food from it (e.g., Cases 1, 2, and 3, Table 4). While government support prevented many dependent households from experiencing a total loss of resilience (e.g., a shift from a dependent strategy to a strategy that might be labeled as a "leaver" – a household that is forced to leave the area and settle elsewhere to gain a new livelihood), the failure to effectively provide destitute (Case 3, Table 4) and disabled community members (Case 2, Table 4) with government support in this community (the reasons for which are unclear) failed to create opportunities for improved livelihoods or trajectory shifts among many households. In the context of fluctuating agroecosystem conditions, ineffective distribution of support from the government, elite capture of natural assets, and the general breakdown of social capital within the community (e.g., breakdown in inter-generational and intra-family support [e.g., Case 5, Table 4]), several diversifier households also experienced a downward trajectory shift from diversifier to dependent over the study period.

Kedia settlement

Household livelihood trajectory trends in Kedia differed from those of Khawa due to both social and environmental differences between sites. Since the 1970s in Kedia, trajectories of accumulator households, composed entirely of the Bakalanga

ethnic group (Table 3), have consistently retained a tendency to specialize in livelihoods built upon pastoralism and arable cultivation. Similar to accumulator households in Khawa, these households have been subject to peaks and troughs in their livelihood activities over time. They have invested income from salaried employment in accumulating livestock and gaining access to water and land as a means to build up asset stocks (e.g., Case 4, Table 4). The trajectories of the accumulators in Kedia, however, demonstrated greater vulnerability to climate shocks over the same time period. With less opportunity for elite capture, only one accumulator household studied in Kedia (Case 4, Table 4) was able to re-accumulate livestock to pre-1980s drought levels by 2000, and even though re-accumulation had been possible, it had occurred at a much slower rate than in Khawa. Livestock assets of all accumulator households declined due to drought-induced starvation and/or thirst, which was compounded by the drying lake and increased degradation. Over time, restrictions to the area available for grazing due to the introduction of veterinary fencing dictated by government policy (1988 and 1996) and increased competition from nearby settlements has led to increased grazing pressure, which has reduced the ability of the agroecosystem to remain productive. This has created or increased the likelihood of density dependent livestock mortality. Coupled with the amplified risks associated with cultivation under less predictable rainfall regimes and dry lake conditions, it is not surprising that accumulator household livelihoods have become increasingly vulnerable to climate-induced shocks. For example, since the mid-1980s drought, successful large-scale cultivation in Kedia has been achieved only once, in 2000 (Cases 4 and 7, Table 4).

The impacts of this vulnerability on livelihood trajectories in Kedia has caused some accumulator households to shift to a more diversified strategy (e. g., Case 5, Table 4). In some cases, however, reduced human capital (e.g., as a result of within-household sickness, ageing, death, or outmigration of household members) and/or reduced social capital (e.g., reduced family support, trust, and/or reciprocity), and policy changes have compounded the extent to which a strategy can remain resilient. As such, factors beyond the environment clearly also exacerbate the stress and cause shifts in livelihood trajectories. It is therefore apparent that Fraser et al.'s (2010) "capacity of agro-ecosystems to remain productive", "capacity for individuals to adapt based on access to assets", and the "strength of formal and informal institutions" have all proved relevant in determining the direction of these households' trajectories.

Households in Kedia that were characterized as following diversified and dependency strategies also followed post-1980s downward trajectories but showed greatest resilience to climate-induced shock and/or stress. These less accumulative and less specialized livelihood strategies and/or those that were reliant on the effective functioning of social security mechanisms (either or both government and traditional) had fewer accumulated assets to lose and had access to a wider range of livelihood activity or substitution options, which facilitated the absorption of shock effects and prevented strategy shifts. Indeed, over the historical period covered by our study, the livelihood strategies of Basarwa in Botswana experienced a long-term shift towards diversification. Since formal settlement establishment in 1978, many Basarwa in Kedia have become increasingly involved in pastoralism and cultivation as well as in maintaining their more traditional focus on hunting, gathering, and fishing (e.g., Case 7, Table 4). As opportunities for fishing ceased when the lake dried in the mid-1980s, and when restrictive hunting permits were introduced, skills in crafts, gathering, and inconspicuous (illegal) forms of hunting, which are typically common among the Basarwa, provided opportunities for greater livelihood resiliency. In contrast, livelihoods that were solely dependent on more climate-sensitive activities – e.g., strategies that specialized in cultivation and/or pastoralism, which are common among the Bakalanga – were typically more vulnerable. The practice of a diverse range of activities therefore helped buffer the stresses and shocks of the 1980s and limited the overall impact on livelihood trajectories.

The government's effective provisioning of financial, nutritional, and educational support to children, destitutes, orphans, and the elderly in Kedia, coupled with strong traditional social security mechanisms (which were of particular importance to the livelihoods of Basarwa and Bateti groups – e.g., Cases 6 and 7, Table 4) led none of the diversifier or dependent households to experience such a radical loss of resilience that they were pushed to leave the settlement. Family and friendship sharing networks (Silberbauer 1981, Kent 1995) outside the settlement, and in many cases beyond the veterinary fences surrounding the

settlement (e.g., Case 7, Table 4), buffered the livelihood impacts of agroecosystem stress caused by degradation and drought. Such mechanisms allowed family members or friends to provide support through the sharing of each other's advantage or disadvantage (e.g., a gain or loss of physical, financial, or natural assets). This opportunity to access and utilize social networks and thus benefit from high social capital maintained the resiliency of such livelihoods because it allowed risks to be spread over a wider geographical area. It is clear, therefore, that in Kedia, formal and informal institutions (Fraser et al.'s [2010] vulnerability framework) have played a key role in reducing vulnerability among dependents during the study period.

DISCUSSION - TOWARDS FUTURE LIVELIHOOD RESILIENCE

In both Kedia and Khawa settlements there were some generic choices that, if taken, increased the likelihood of a livelihood trajectory increasing in resilience between 1974 and 2005:

1. accumulation of financial assets (through waged- or self-employment, business, and/or the sale of natural and/or physical assets);

2. investment in and accumulation of physical assets (e.g., land and diverse herds of livestock);

3. opportunistic and strategic diversification of livelihood activities;

4. diversity within livelihood activities (e.g., investment in a range of stock types and planting of a mixture of crop types);

5. investment in improved access to water;

6. investment in transport; and

7. access to government support for elders, destitutes, and the disabled.

The following factors typically precipitated a shift towards increased vulnerability: (1) loss of livestock, (2) reduced access to natural assets (environmentally or policy determined), and (3) reduced diversity of livelihood activities conducted/loss of livelihood activity/option.

In light of the continued heavy reliance of remote rural households on natural assets, the impacts of current and past dynamics, and the potential future impacts of climate change in the Kalahari (Thomas et al. 2005) on agroecosystems, there is likely to be an increasing role for formal and informal institutions in reducing vulnerability in Botswana. Clearly, in the context of these two settlements, ensuring access to a diversity of assets is vital. In the face of increasing climatic uncertainty, the key challenges to maintaining the effective functioning of this social-ecological system include (1) maintaining agroecosystem health to ensure adequate future supplies of natural resources (most essentially water, plant, and wildlife resources), (2) preventing elite capture and accumulation of opportunities, and (3) ensuring opportunities for diversification and generation of financial capital.

Many of these challenges could be addressed through improved functioning of formal and informal institutions (Twyman et al. 2004). Developments that facilitate (1) improved efficiency and accuracy of the distribution of government support, (2) adaptive management of dryland agroecosystems to ensure accommodation of dynamics rather than the imposition of stability and control, (3) more equitable access to natural resources, and (4) more equitable access to diversification opportunities and accumulation of financial and physical assets, will assist. As was illustrated in both settlements, collective action within communities is required along with government incentives and programs. If such developments cannot be achieved, and the predicted impacts of climate change continue, households are likely to increasingly face the need to move in search of better functioning and more resilient social-ecological systems.

CONCLUSION

This paper has drawn on the concepts of livelihood trajectories and resilience to assist in the exploration of vulnerability in the drylands of Botswana. We used a combination of primary and secondary data to examine the inherent social-ecological dynamics in the study area and to categorize households into three different groups according to the ways in which their livelihood strategies exploited these inherent dynamics. Based on this information, we qualitatively assessed those factors that contributed to the emergence of vulnerability and/or resilience,

and elucidated five environmental changes operating independently of the inherent environmental dynamics that in many cases altered the vulnerability context and the overall direction of livelihood trajectories. In returning to the questions outlined earlier in relation to Fraser et al.'s (2010) framework, we illustrated the combined influence of environmental change and formal and informal institutions in determining a household's access to and use of assets and therefore its ability to create more resilient livelihood outcomes. In some cases, the agroecosystem remained productive in a changing vulnerability context, and for some people, their survival was supported by the combination of the livelihood strategies they pursue and the institutional capacity and willingness related to their particular context. Our paper has nevertheless indicated that the everyday details in each narrative have a profound influence on households' livelihood trajectories and resilience. In view of projected climate changes in this part of southern Africa and their potential impacts, these findings have highlighted the importance of formal and informal institutions in building resilience and the need for increased effort to ensure the most vulnerable households have access to a diversity of assets.

Responses to this article can be read online at:
http://www.ecologyandsociety.org/vol15/iss4/art3/responses/

Acknowledgments:

This research was conducted as part of the first author's DPhil thesis at the School of Geography and Environment, University of Oxford under the supervision of Professor David Thomas, Professor Sarah Whatmore, and Dr. Chasca Twyman (Sheffield University). The research was funded by an ESRC/NERC interdisciplinary studentship, Hertford College Oxford, Slawson Award (RGS-IBG), Dudley Stamp Memorial Trust (Royal Society), Victor Ford Scholarship (Sir Richard Stapley Educational Trust), and Sheffield University. Research was conducted with the permission of The Ministry of Agriculture in Botswana (Permit A34/6 XVII(82)PS) and in collaboration with the UNDP-GEF Indigenous Vegetation Project. This research would not have been possible without the support and cooperation of Khawa and Kedia communities.

LITERATURE CITED

Bagchi, D. K., P. Blaikie, J. Cameron, M. Chattopadhyay, N. Gyawali, and D. Seddon. 1998. Conceptual and methodological challenges in the study of livelihood trajectories: case-studies in eastern India and western Nepal. *Journal of International Development* **10**:453–468.

Barrow, E., J. Davies, S. Berhe, V. Matiru, N. Mohamed, W. Olenasha, and M. Rugadya. 2007. Pastoralists as shrewd managers of risk and resilience in the Horn of Africa. IUCN Eastern Africa Regional Office Policy Brief No. 1, Nairobi, Kenya. [online] URL: http://cmsdata.iucn.org/downloads/pastoralists_as_shrewd_managers_of_risk_and_resilience.pdf.

Batisani, N., and B. Yarnal. 2010. Rainfall variability and trends in semi-arid Botswana: implications for climate change adaptation policy. *Applied Geography*. **30**(4):483–489.

Bhalotra, Y. P. R. 1987. *Climate of Botswana Part II: Elements of Climate I. Rainfall.* Department of Meteorological Services, Ministry of Works and Communication, Gaborone, Botswana.

Boggs, L. 2000. Community power, participation, conflict and development choice: community wildlife conservation in the Okavango region of northern Botswana. Evaluating Eden Series Discussion Paper No. 17. International Institute for Environment and Development, London, UK.

Bonifica, M. 1992. Aerial surveys of Botswana 1989–1991. Final Report. Technical Assistance to the Department of Wildlife and National Parks, Gaborone, Botswana.

Buckley, R. C., R. J. Wasson, and A. Gubb. 1987. Phosphorus and potassium status of arid dunefield soils in central Australia and southern Africa and biogeographic implications. *Journal of Arid Environments* **13**:211–216.

Campbell, B. M., I. J. Gordon, M. K. Luckert, L. Petheram, and S. Vetter. 2006. In search of optimal stocking regimes in semi-arid grazing lands: One size does not fit all. *Ecological Economics* **60**(1):75–85.

Carney, D. 1998. Implementing the sustainable rural livelihoods approach. Chapter 1 *in* D. Carney, editor. *Sustainable rural livelihoods: What contribution can we make?* Department for International Development, London, UK.

Cashdan, E. A. 1985. Coping with risk: reciprocity among the Basarwa of Northern Botswana. *Man (N. S.)* **20**(3):454–474.

Chambers, R., and G. Conway. 1992. Sustainable rural livelihoods: practical concepts for the 21st century. IDS Discussion Paper 296. Institute of Development Studies, Brighton, UK.

de Haan, L. J., and A. Zoomers. 2005. Exploring the frontier of livelihoods research. *Development and Change* **36**(1):27–47.

Department of Wildlife and National Parks (DWNP). 1994. *Aerial census of animals in Botswana: dry season 1994*. Technical assistance to the Department of Wildlife and National Parks, Gaborone, Botswana. No. 6100.37.14.046. ULG Consultants Ltd., Warwick, UK.

Department of Wildlife and National Parks (DWNP). 1994. *Aerial census of animals in Botswana: wet season 1994*. Technical assistance to the Department of Wildlife and National Parks, Gaborone, Botswana. No. 6100.37.14.040. ULG Consultants Ltd., Warwick, UK.

DHV Consulting Engineers (DHV). 1980. Special subject report: animal populations. Countrywide animal and range assessment project. Volume 4. DHV Consulting Engineers, Amersfoort, The Netherlands.

Dougill, A. J., E. D. G. Fraser, and M. S. Reed. 2010. Anticipating vulnerability to climate change in dryland pastoral systems: using dynamic systems models for the Kalahari. *Ecology and Society* **15** (2):17. [online] URL: http://www.ecologyandsociety.org/vol15/iss2/art17/.

du Plessis, A. J. E., and K. M. Rowntree. 2003. Water resources in Botswana with particular reference to savanna regions. *South African Geographical Journal* **85**(1):42–49.

Ellis, F. 2000. *Rural livelihoods and diversity in developing countries*. Oxford University Press, Oxford, UK.

Folke, C., S. Carpenter, T. Elmqvist, L. Gunderson, C. S. Holling, and B. Walker. 2002. Resilience and sustainable development: building adaptive capacity in a world of transformations. *AMBIO: A Journal of the Human Environment* **31** (5):437–440.

Fraser, E. D. G. 2006. Agro-ecosystem vulnerability. Using past famines to help understand adaptation to future problems in todays global agri-food system. *Ecological Complexity* **3**:328–335.

Fraser, E.D.G, A. Dougill, K. Hubacek, C. Quinn, J. Sendzimir, and M. Termansen. 2010. Assessing vulnerability to climate change in dryland livelihood systems: conceptual challenges and interdisciplinary solutions. *Ecology and Society*. [online] URL:

Goomes, R., and F. Petrassi. 1996. Rainfall variability and drought in sub-Saharan Africa since 1960. FAO Agrometeorology Series Working Paper No. 9. Food and Agriculture Organization of the United Nations, Rome, Italy.

Kent, S. 1995. Unstable households in a stable Kalahari community in Botswana. *American Anthropologist* **97**(2):297–312.

Leach, M., I. Scoones, and A. Stirling. 2007. Pathways to sustainability: an overview of the STEPS Centre approach, STEPS Approach Paper, Brighton: STEPS Centre. ISBN – 13: 978 185864 656 1. [online] URL: http://www.steps-centre.org/PDFs/final_steps_overview.pdf.

Marschke, M. J., and F. Berkes. 2006. Exploring strategies that build livelihood resilience: a case from Cambodia. *Ecology and Society* **11**(2):42. [online] URL: http://www.ecologyandsociety.org/vol11/iss1/art42/.

McCarthy, J. M., T. Gumbricht, T. McCarthy, P. Frost, K. Wessels, and F. Seidel. 2003. Flooding patterns of the Okavango Wetland in Botswana

between 1972 and 2000. *Ambio* **32**(7):453–457.

Ministry of Agriculture. 1990. Soil map of the Republic of Botswana. Soil mapping and advisory service FAO/BOT/85/011. Scale 1:1,000,000. Ministry of Agriculture, Gaborone, Botswana.

Peters, P.E. 1984. Struggles over water, struggles over meaning: cattle, water and the state in Botswana. *Africa* **54**(3):29–49.

Sallu, S. M. 2007. *Biodiversity dynamics, livelihoods and knowledges in Kalahari dryland biomes*. Dissertation. University of Oxford, Oxford, UK.

Sallu, S. M., C. Twyman, and D. S. G. Thomas. 2009. The multidimensional nature of biodiversity and social dynamics and implications for contemporary rural livelihoods in remote Kalahari settlements, Botswana. *African Journal of Ecology* **47**(Suppl. 1):1–9.

Scoones, I. 1998. Sustainable rural livelihoods: a framework for analysis. IDS Working Paper 72. Institute of Development Studies, Brighton, UK.

Scoones, I. 1996. *Hazards and opportunities. Farming livelihoods in dryland Africa: lessons from Zimbabwe.* Zed Books, London, UK.

Silberbauer, G. B. 1981. *Hunter and habitat in the central Kalahari desert.* Cambridge University Press, Cambridge, UK.

Sporton, D., and D. S. G. Thomas. 2002. *Sustainable livelihoods in Kalahari environments – a contribution to global debates.* Oxford University Press, Oxford, UK.

Tadross, M. A., B. C. Hewitson, and M. T. Usman. 2005. The interannual variability of the onset of the maize growing season over South Africa and Zimbabwe. *Journal of Climatology* **18**:3356–3372.

Tadross, M. A., P. Suarez, A. Lotsch, S. Hachigonta, M. Mdoka, L. Unganai, F. Lucio, D. Kamdonyo, and M. Muchinda. 2007. Pages 193–204 *in* Changes in growing-season rainfall characteristics and downscaled scenarios of change over southern Africa: implications for growing maize. *IPCC Regional Expert Meeting on Regional Impacts, Adaptation, Vulnerability, and Mitigation*, June 20, Nadi, Fiji. [online] URL: http://www.wikiadapt.org/filestore/wikiADAPT/Growing_Season_Changes_Southern_Africa.pdf.

Thomas, D. S. G., M. Knight, and G. F. S. Wiggs. 2005. Remobilization of southern African desert dune systems by twenty-first century global warming. *Nature* **435**:1218–1221.

Thomas, D. S. G., and P. A. Shaw. 1991. *The Kalahari environment.* Cambridge University Press, Cambridge, UK.

Thomas, D. S. G., and D. Sporton. 2002. Sustainable livelihoods in Kalahari environments: a contribution to global debates. Pages 1–19 *in* D. Sporton and D.S.G. Thomas, editors. *Sustainable livelihoods in Kalahari environments – a contribution to global debates.* Oxford University Press, Oxford, UK.

Tolsma, D. J., W. H. O. Ernst, and R. A. Verwey. 1987. Nutrients in soil and vegetation around two artificial waterpoints in Eastern Botswana. *Journal of Applied Ecology* **24**:991–1000.

Twyman, C. 2001. Natural resource use and livelihoods in Botswana's Wildlife Management Areas. *Applied Geography* **21**:45–68.

Twyman, C., D. Sporton, and D. S. G. Thomas. 2004. 'Where is the life in farming?': the viability of smallholder farming on the margins of the Kalahari, southern Africa. *Geoforum* **35**(1):69–85

Van der Maas, H.A., H.Th. Riezebos, W. Hoffmans, W. Rozenga, and P. Slaa. 1995. Kedia baseline survey. Final report of the socio-economic and biophysical baseline survey of Kedia RAD-settlement and surrounding areas in the Boteti sub-District. Division of Planning, Statistics and Research, Ministry of Local Government, Lands and Housing, Republic of Botswana and Faculty of Geographical Sciences, Utrecht University, The Netherlands. ISBN 9912-1-188-8.

Walker, B., L. Gunderson, A. Kinzig, C. Folke, S. Carpenter, and L. Schultz. 2006. A handful of heuristics and some propositions for understanding resilience in social-ecological systems. *Ecology and Society* **11**(1):13. [online] URL: http://www.ecologyandsociety.org/vol11/iss1/art13/.

Williamson, D. T., and B. Mbano. 1988. Wildebeest mortality during 1983 at Lake Xau, Botswana. *African Journal of Ecology* **26**:341–344.

Zufferey, F. S. 1983. A study of local institutions in Mopipi Communal First Development Area, Central District. Applied Research Unit/MLGL, Gaborone, Botswana.

[1] For detailed information about social security benefit provision in Botswana for the period 2004/2005, refer to http://www.ssa.gov/policy/docs/progdesc/ssptw/2004-2005/africa/botswana.html.
