# Supplementary material for: Systematic Review of Methods in Low-Consensus Fields: Supporting Commensuration through `Construct-Centered Methods Aggregation’ in the Case of Climate Change Vulnerability Research
Source: PLoS One. 2016 Feb 22;11(2):e0149071. doi: 10.1371/journal.pone.0149071 (PMC4762661; doi:10.1371/journal.pone.0149071)
Supplement: S2 File — Source articles and bibliography. (ZIP) [file pone.0149071.s002.zip › data requirements/Tesso et al.pdf]

Vol.3, No.6, 871-888 (2012)
http://dx.doi.org/10.4236/as.2012.36106

Agricultural Sciences

Analysis of vulnerability and resilience to climate change induced shocks in North Shewa, Ethiopia

Gutu Tesso[1*], Bezabih Emana[2], Mengistu Ketema[1]

[1]College of Agriculture, School of Agricultural Economics and Agricultural Business, Haramaya University, Dire Dawa, Ethiopia;
[*]Corresponding Author: gutessoo@yahoo.com
[2]HEZBEZ Business and Consultancy PLC, Addis Ababa, Ethiopia

Received 4 August 2012; revised 30 August 2012; accepted 10 September 2012

ABSTRACT

This article analyzes the vulnerability and resilience levels of farm households in North Shewa, Ethiopia, using a survey of 452 households. Agro ecological based classification was done to analyze vulnerability to climate change induced shocks. Integrated vulnerability analysis approach was employed to develop indexes for socioeconomic and biophysical indicators. The indicators have been classified into adaptive capacity, exposure and sensitivity to climate change impact. Then Principal Component Analysis was used to compute vulnerability index of each agro ecological zone. The result shows that farmers living in the highland areas were very much vulnerable to natural shocks compared to those living in the lowland area. In order to identify and analyse the determinants of resilience to climate change impacts, ordered probit model was used. Households were classified into three categories based on the time they take to bounce back after natural shocks. The model outputs indicate that farmers with better investment on natural resource management, access to market, better social network, access to credit, preparedness, saving liquid assets, access to irrigation and better level of education exhibited greater level of resilience during and after climate change induced shocks.

Keywords: Climate Change; Vulnerability; Resilience; Principal Component Analysis; Ordered Probit; Ethiopia

1. INTRODUCTION

Climate Change and its impact on the developed as well as developing countries are becoming the greatest worries of life and livelihoods. The impacts of climate change are heterogeneous across a diverse range of geo-political scales. For instance, the risk is generally believed to be more acute in developing countries because they rely heavily on climate-sensitive sectors, such as agriculture and fisheries, and have low gross domestic products, high level of poverty, low level of education, and limited human, institutional, economic, technical, and financial capacities as cited in [1-3]. Vulnerability of countries and societies to the effects of climate change depends not only on the magnitude of climatic stress but also on the sensitivity and capacity of affected societies to adapt to or cope with such stress. Therefore, vulnerability is the degree to which a system is susceptible or unable to cope with the adverse effects of climate change, including climate variability and extremes. In this regard, vulnerability is a function of the character, magnitude, and rate of climate variation to which a system is exposed, its sensitivity, and its adaptive capacity [4].

A number of climate change impact studies have been conducted in many countries on specific sectors such as water resources, agriculture, health, coastal zones, and forestry by using impact models and to a lesser extent socioeconomic analyses [5-7]. Global recommendation for Africa calls for an integrated assessment approach for vulnerability study, at national scale and local level [8]. From the perspective of rural farm households, an analysis of vulnerability to climate change is needed at the level that would specifically address specific geographic location so that the smallholders will get a lesson to tackle climate change problems with the precision that is necessary [9].

On the other hand, the resilience of households to climate change impact is another important issue in maintaining sustainable livelihood. According to DFID, resilience at community level is explained as the ability of countries, communities and households to manage change, by maintaining or transforming living standards in the face of shocks or stresses—such as earthquakes, drought or violent conflict—without compromising their long-term prospects [10]. Similarly, resilience is the ability of

Copyright © 2012 SciRes.

a social or ecological system to absorb disturbances while retaining the same basic structure and ways of functioning, the capacity for self-organization, and the capacity to adapt to stress and change. This is a measurement of community's capacity to absorb external shocks. In the aftermath of occurrence of climate change induced shocks, how do farmer bounce back to normal livelihood is about the resilience level of farming community. A resilient community is able to respond to changes or stress in a positive way, and is able to maintain its core functions as a community despite those stresses [11]. An important issue would be what enables a particular community to easily or hardly bounce back.

It is against this background that this research sets out to determine quantitatively the magnitudes and patterns of rural households' vulnerability to climate change and then identifies the important determinants for resilience at household level in North Shewa zone of Ethiopia. The findings of the research can assist in identifying specific areas that are most vulnerable to climate change and guide policymakers and development actors in determining where investments in reducing vulnerability and building household's resilience may be most effective against adverse effects of climate change.

2. METHODOLOGY

2.1. The Study Area

The study area is North Shewa Zone of Oromia national regional state. North Shewa Zone is found in north-west direction of Addis Ababa. Fiche town which is located at 147 km away from Addis Ababa is the capital of the zone. The zone has 13 rural districts with a total land area of 10,323 km^2. It is situated between 9˚30"N and 38˚40"E. The zone is boarded by Amahara region in the north and the east, West Shewa zone in the west and Addis Ababa in the south. The topography of the area is mountainous in the highland and midland, while it is plain in the lowland areas. The altitude of the area ranges between 1300 - 2500 meters above sea level. It is divided into three agro-ecologies, namely, 15% highland (>2500 meter above sea level), 40% midland (1500 - 2500 meter above sea level) and 45% lowland (500 - 1500 meter above sea level) [12]. The area gets rainfall during both *Belg* (February to April) and *Meher* (June to September) seasons. The average annual rainfall of the area ranges from less than 800 mm to 1600 mm while the mean annual temperature varies between 15˚C and 19˚C.

The population of the zone is estimated to be 1,431,305 with population density of 138.7 persons per km^2 and average of 4.6 persons per household. The community practices mixed farming of cereal crops, pulses and oil crops. Livestock production also constitutes an important part of agricultural activities of the zone. The average land holding is 1.1 hectare per household. Due to the continuous reduction of farmland to degradation by frequent flooding and drought, farming intruded into steep sloping areas, forest lands and expanded to marginal lands and communal lands covering 81% of the total area of the zone. Only 3% of the total land is put under grazing, 3.7% forest land, 11.33% degraded and bare land and 0.65% is other form of land. The crops, livestock and other livelihoods of the community are subjected to damage to climate change induced hazards. This coupled with the continually decreasing farm size have serious impact threatening farmers adaptive capacity and livelihood improvements [12].

2.2. Data and Analytical Tools

The data for the research was obtained from a survey of 452 farm households in three districts of the Zone in 2011/2012. The districts include Yaya Gullele, Hidha Abote and Derra. The specific study sites within the districts were selected based on a multi stage random sampling procedure. Consequently, 19 Kebeles were selected from which the sample households were selected randomly proportional to population size. A structured questionnaire was used to interview the farmers. Data collected from the farmers include household characteristics, landholding, crops and livestock production, disaster occurrence, perception level (on precipitation, temperature, soil moisture, air moisture and wind direction), adaptation strategies pursued, different coping strategies pursued, level of resilience, and other relevant information.

In addition, secondary data relevant for this analysis was obtained from the National Meteorological Service Agency (NMSA), Central Statistical Authority (CSA), and Zonal and district agricultural offices. In order to understand the research questions at community level, qualitative data were collected through focused group discussion using checklist prepared for the purpose.

2.3. Method of Analysis

2.3.1. Conceptual Framework of Vulnerability Analysis

Vulnerability analysis involves various approaches; the first one is called the socioeconomic vulnerability assessment approach which focuses on the socioeconomic and political status of individuals or social groups. Individuals in a community often vary in terms of education, gender, wealth, health status, access to credit, access to information and technology, formal and informal (social) capital, political power, and so on, which are responsible for the variations in vulnerability levels [5, 13]. Consequently, vulnerability is considered to be a starting point or a state that exists within a system before

it encounters a hazard event [14]. In this regard, vulnerability is constructed by society as a result of institutional and economic changes. This explains why the socioeconomic approach focuses on identifying the adaptive capacity of individuals or communities based on their internal characteristics. One major limitation of the socioeconomic approach is that it focuses only on variations within society, but in reality, societies vary not only due to sociopolitical factors but also due to environmental factors. It does not also account for the availability of natural resource bases to potentially counteract the negative impacts of these environmental shocks. For example, areas with easily accessible underground water can better cope with drought by utilizing this resource [5].

The second approach is called the biophysical approach that attempts to assess the level of damage that a given environmental stress causes on both social and biological systems. It is sometimes known as an impact assessment. The emphasis is on the vulnerability or degradation of biophysical conditions [15]. It is a dominant approach employed in studies of vulnerability to natural hazards and climate change [16]. Füssel identified this approach as a risk-hazard approach. The biophysical approach, although very informative, has its limitations [13]. A major limitation is that the assessment of biophysical factors is not a sufficient condition for understanding the complex dynamics of vulnerability. It also neglects structural factors and human agency both in producing vulnerability and in coping or adapting to it. The approach overemphasizes extreme events while neglecting root causes and everyday social processes that influence differential vulnerability [15,17].

The third approach is called integrated assessment approach which combines both socioeconomic and biophysical approaches to determine vulnerability. The IPCC definition—which conceptualizes vulnerability to climate as a function of adaptive capacity, sensitivity, and exposure—accommodates the integrated approach to vulnerability analysis [4,13,18]. According to Füssel and Klein, the risk-hazard framework (biophysical approach) corresponds most closely to sensitivity in the IPCC terminology while the adaptive capacity (broader social development) is largely consistent with the socioeconomic approach [18]. Furthermore, in the IPCC framework, exposure has an external dimension, whereas both sensitivity and adaptive capacity have an internal dimension, which is implicitly assumed in the integrated vulnerability assessment framework [13].

Although the integrated assessment approach corrects the weaknesses of the other approaches, it also has some limitations. The main limitation is that there is no standard method for combining the biophysical and socioeconomic indicators. This approach uses different datasets, ranging from socioeconomic datasets to biophysical factors. These datasets certainly have different yet unknown weights [19]. The other weakness of this approach is that it does not account for the dynamism in vulnerability. Despite its weaknesses, the approach has much to offer in terms of policy decisions [5]. Vulnerability in this context is a physical risk and a social response within a defined geographic territory [19].

In order to solve the challenges of standards for combining the different variables different methods have been suggested. The first is assuming that all indicators of vulnerability have equal importance and thus giving them equal weights [19]. The second method is assigning different weights to avoid the uncertainty of equal weighting given the diversity of indicators used. In line with the second method, many methodological approaches have been suggested to make up for the weight differences of indicators. Some of these approaches include use of expert judgment [5,20,21], principal component analysis [22,23], correlation with past disaster events [24], and use of fuzzy logic [25]. Even though there are attempts in giving weights, their appropriateness is still dubious; because there is no standard weighting method against which each method is tested for precision [26]. **Annex 1** show different indicators and the scales at which they could be used and indicators added based on the context of the study area.

For the analysis of vulnerability in the study area both physical and social vulnerability perspectives have been integrated. Fusel and Klein have summarized the framework for vulnerability analysis to include the three components; adaptive capacity, sensitivity and exposure. In the framework, exposure to climate change and variability will lead to vulnerability based on the sensitivity level of the communities' lives and livelihood. Moreover, when the capacity to withstand the negative conesquences of exposure and sensitivity become very low, the vulnerability of climate change impact will be very much higher. In the framework, capacity is generated from the implementation of adaptation and mitigation intervenetions [18].

With this background, the first stage of analysis of vulnerability in the study area involved descriptive analysis of the socioeconomic and environmental characteristics particularly adaptive capacity, sensitivity, and exposure to CC marked by red and green color in the above framework. Second, the vulnerability indices were obtained by applying Principal Component Analysis on the adaptive, sensitivity, and exposure variables following the works of Deressa, Hassan, and Ringler, Fussel and Ignatius [1,5,13]. Principal component analysis is frequently used in research that constructs indices for which there are no well-defined weights, such as assetbased indices used for the measurements of wealth across different social groups. The argument here is that, as with

Copyright © 2012 SciRes.

the asset-based indices for wealth comparison, there are no well-defined weights assigned to the vulnerability indices. Therefore, principal component analysis generated the weights, based on the assumption that there is a common factor that explains the variance in the vulnerability. Instinctively, the first principal component of a set of variables is the linear index of all the variables that captures the largest amount of information common to all the variables. Accordingly, the first component scores from the principal component analysis measured the weighted sum of score of all variables.

The model specification is given as

$$\text{Vulnerability} = (\text{adaptive capacity}) - (\text{sensitivity} + \text{exposure}) \quad (1)$$

In this case vulnerability is the difference between adaptive capacity of a household and its sensitivity and exposure to climate change induced hazards. When adaptive capacity of the area exceeds that of sensitivity and exposure, the area becomes less vulnerable to climate change impacts. As explained above, each set (adaptive capacity, sensitivity and exposure) are composed of different variables. The model specification is as follows:

$$V_1 = \left(A_1X_{1j} + A_2X_{2j} + \cdots + A_nX_{nj}\right) - \left(A_{n+1}Y_{1j} + A_{n+2}Y_{2j} + \ldots + A_{n+n}Y_{nj}\right) \quad (2)$$

where V_1 is vulnerability index, while *Xs*, are elements of adaptive capacity, and *Ys* are exposure and sensitivity. The values of X and Y is obtained by normalization using their mean and standard errors. For instance; $X_{1j} = \left(x_{1j} - x_1^*\right)/s_1^*$, where x_1^* is the mean of x_{1j} across the different agro ecological zones, s_1^* is its standard deviation. In this regard, the first principal component of a set of variables is the linear index of all the variables that captures the largest amount of information common to all the variables. The whole matrix of X_{1j} appears as follows:

$$X_{ij}/Y_{ij} = \begin{cases} \left(X_{11} + X_{12} + \cdots + X_{2n}\right) - \left(Y_{11} + Y_{12} + \cdots + Y_{1n}\right) \\ \vdots \\ \left(X_{m1} + X_{m2} + \cdots + X_{mn}\right) - \left(Y_{m1} + Y_{m2} + \cdots + Y_{mn}\right) \end{cases} \quad (3)$$

The *i* and *j* in the above notation implies the number of rows (in this case agro ecological zone) and the number of columns (in this case variables of adaptive capacity, exposure and sensitivity) respectively. In **Eq.4**, the *As*, are the first component score of each variable computed using Principal Component Analysis in STATA. Finally, the vulnerability index of each location is obtained using **Eq.4**:

$$V_i = \begin{pmatrix} A_1 \\ A_2 \\ \vdots \\ A_{n+n} \end{pmatrix} \times \begin{pmatrix} \left(X_{11} + \cdots + X_{1n}\right) - \left(Y_{11} + \cdots + Y_{1n}\right) \\ \vdots \\ \left(X_{m1} + \cdots + X_{mn}\right) - \left(Y_{m1} + \cdots + Y_{mn}\right) \end{pmatrix} \quad (4)$$

In calculating the direction of relationship in vulnerability indicators (*i.e.*, their sign), a negative value was assigned to both exposure and sensitivity. The justification is that areas that are highly exposed to climate shocks are more sensitive to damage, assumeing constant adaptive capacity. The implication is that a higher net value indicates lesser vulnerability and vice versa. However, in creating the indices, the scale of analysis is important. As coated Deressa, Hassan, and Ringler. [5], vulnerability analysis ranges from local or household [11] level to the global level [24]. The choice of scale is dictated by the objectives, methodologies, and data availability. For this study, the scale of analysis was local level. This is because, all the earlier studies using aggregated regional and national levels data has overlooked local variations which is important for household level analysis.

2.3.2. Determinants of Resilience

Ordered *probit* regression model was used to identify and analyze the determinants of households' resilience to climate change induced shocks. In this analysis, the level of resilience was classified into three categories: 1) households that were fast in bouncing back; which means households that have gone back to their normal agricultural operation in the following production season; 2) moderate in bouncing back; which means households which took one to two agricultural seasons to get back to normal operation as before the event; and 3) slow in bouncing back; which means households which were unable to bounce back within one to two agricultural seasons to their normal livelihood activities. In this research, a farmer is said to have fully bounced back, when it begins its livelyhood operation as time before the shock. The speed of bouncing back was measured by number of agricultural seasons taken to bounce back to their livelihood without external intervention by government or non-governmental organization. And then comparison was done based on certain defined characteristics. Thus, resilience in this measurment involved ordered outcome. This is with the basic hypothesis that a given natural shock will have differencial impact on households' resilience.

$$Y_j^* = X_j^1\beta + U_{1j} \quad (5)$$

$$Y = 0 \text{ if } Y^* \le 0$$

Copyright © 2012 SciRes.

$$Y = 1 \text{ if } 0 < Y^* \leq 1$$

$$Y = 2 \text{ if } 1 < Y^* \leq 2$$

Y^* is level of resilience and involves ordered outcome, that is $Y = 0$ was given to households taking more than two years to bounce back, $Y = 1$ was given households taking greater than one year and less than or equals to two years; and $Y = 2$, was given to households taking less than or equals to one year. The X_{ij} are the explanatory variables determining the time taken to bounce back. The independent variables included in the model were availability of food stock (dummy), income diversification (number of enterprises), number of plots, number of dependent family members, age of household head (years), access to credit (dummy), social capital (number of institutional involvement), area under perennial crops (ha), preparedness (dummy), propensity to invest on natural resources (percentage of area under conservation), propensity to save (percentage of saving), access to irrigation (ha), geographic locations (dummy), etc. βs are parameters estimated and U_{ij} is the disturbance term.

3. RESULT AND DISCUSSION

3.1. Vulnerability Analysis

3.1.1. Vulnerability to Climate Change Induced Shocks

In Ethiopia in general and the study area in particular, small-scale farmers bear largely the brunt of the negative impacts of climate change, which include increased poverty, water scarcity, and food insecurity. People who are already poor and marginalized are struggling to cope with the added burden of increasingly unpredictable weather, which is triggered by climate change. Families and communities are getting harder and harder to bounce back from ever-changing, inconsistent weather affecting their livelihoods, and many have been forced to sell livestock or remove children from school—coping mechanisms that only increase the cycle of vulnerability.

Women headed households, families with high dependency ratio, farmers operating on less fertile and steeply sloping farms and less diversified enterprises, in particular, are disproportionately affected by climate variability. In times of crisis, this categories of community tend to move away to look for alternative means of survival. These households have fewer options to find other ways of making a living, especially since literacy rate is very low engaging in alternative coping mechanisms through wage employment. Women are also not empowered to make household decisions and are frequently without cash savings or assets to sell or to buy food and other basic items. This vulnerability can be further classified into social, economic and environmental in the context of agriculture based community.

3.1.1.1. Social Vulnerability

Social vulnerability can be loosely defined as the predisposition of people, organizations, and societies to impacts from natural and man-made disasters. Quantitative description of the overall social vulnerability of an area or a region to shocks is measured based on such variables as proportion of elderly and children, rural housing density, gender, marital status, age, health status, educational level of household heads, etc. in the context of rural household's social vulnerability to climate change: it is vulnerability due to the low social profile. Farmers with high institutional participation, many relatives in a community, family size with working potential, and participation in different social meetings usually have high social power to withstand adverse effects. **Table 1** presents farmers position in terms of their social status in the community based on the data from the household survey.

From **Table 1**, it is clearly observed that literacy rate of the community is extreemly low, dependancy ratio of household members with more than four dependents is very high, which implies the proportion of dependent household member with less than 18 and greater than 60 is significant, participation in different institutions is also low. Thus, it is easier to observe that vulnerability level of community members to the frequently occuring natural shocks from their social capital endowment perspective is high.

3.1.1.2. Economic Vulnerability

The economic vulnerability assessment approach mainly focuses on the economic status of individuals or social groups. Individuals in a community often vary in terms of wealth, health status, access to credit, access to information and technology and so on. These variations are responsible for the variations in vulnerability levels. In this case, vulnerability is considered to be a *starting point* or a *state* (*i.e.*, a variable describing the internal state of a system) that exists within a system before it encounters a hazard event [14,27]. Thus, vulnerability is considered to be constructed by the society as a result of economic changes. In general, the economic approach focuses on identifying the adaptive capacity of individuals or communities based on their internal characteristics.

In the study area, climate vulnerability weakens the different economic capacities. It is historically known for the socio-economic setbacks and agricultural failures caused by dry spells and droughts associated with deficits in political-institutional capacities. Economic variables were selected to be applied for the study area based on the concept of vulnerability, which is primarily a function of adaptive capacity. In this context of adaptive

Copyright © 2012 SciRes.

Table 1. Social, economic and environmental vulnerability indicators for the study area.

**I. Social Vulnerability Variables**	**Percentage**
Sex: Female headed	15.9
Education: illiterate and less than grade 2	86.1
Marital status: Single (including divorce and widow)	14.2
No. of relatives: relative to less than 5 households	38.3
No. institutions: Participation in less than 2.35 institutions	57.1
Dependency: High dependency of 4 person and more	86.3
Farm to farm ext: No access to farmer to farmer extension	31.6
Year Ag. Experience: Lack of farm experience if ≤3 years	7.3
Access to indigenous early warning information: Having no access	43.8
**II. Economic Vulnerability Variables**	**Percentage**
Livestock ownership: Own less than 2 tropical livestock unit	35.6
Access to information: Having no access to	73.9
Ownership of perennial crops: no area under perennial crops	87.2
Land size: own less than 0.5 ha of land	36.1
Land fragmentation: own only one plots	74.6
Non-farm income: Have no non-farm income	82.7
Soil and water conservation structures: More than 50% is not conserved	32.3
Income level: Having less than minimum requirement	74.2
Consumption expenditure: Spending less than minimum requirement	62.4
Crop diversity: less than 50% of the 8 major crops grown in the area	70.7
Land under irrigation: no access to irrigation at all	64.2
Land under improved seed: area not covered with improved seed (average of high yielding, drought tolerant, early maturing)	64
Land under commercial fertilizer: Having no access to fertilizer at all	38.5
Cash reserve: Having no cash saving at all	92
Food reserve: Having no food reserve for next year	71
Credit: Having no access to credit at all	44.5
**III. Environmental Vulnerability Variables (Measures of Sensitivity and Exposure)**	**Amount (%)**
Land topography: Slope greater than 15% and 0% slope	49.1
Fertility: Poor fertility and cannot produce without heavy fertilizer use	31.6
Vegetation cover: Bare land	96.3
Frequency of hazards: People facing more than two natural hazards in a year	84.3
Rainfall: Receiving below average	46.2
Temperature: Experiencing above average	95.4
Change in wind direction: Encountering change in wind direction than usual	91.4

Sources: Computed from HH survey 2011/12 and Districts report.

Copyright © 2012 SciRes. OPEN ACCESS

capacity, farmers' economic condition palys significant role in reducing vulnerability. In this connection, the economic charcterstics of farmers in the study area shows that, large majority of the hosueholds operate on less diversified livelihoods, low non-farm engagement, low access to credit and market, small landholding, low holding of perenial crops, small or no area under irrigation, etc. This indicates a high level of economic vulnerability of farmers to shocks. **Table 1** displays the economic characteristics of farmers as related to vulnerability to climate change impact.

Clearly the economic status of the households in the zone to withstand climate change induced hazards can be judged from the above statistics to be very low. Apparently, large majority of the farmers are economically vulnerable to the impact of climate change. This can be evidenced by the fact that 38.5% of the population (or 5 out of 13 rural districts of the zone) are recurrent beneficiaries of safety net program from year to year.

3.1.1.3. Environmental and Physical Vulnerability

There are many environmental challenges that derive from being a smallholder. The disadvantages include: a narrow range of resources, which leads to high level of economic specialization, high population densities, which can lead to degradation and depletion of limited natural resources; small watersheds and vulnerable water supplies; and thus easy susceptibility of the farms to climate change impacts. In this connection, increasingly different indicators have been used to assess vulnerability, both at the national and local scales. At different levels, indicators have been embraced for empirically assessing biophysical vulnerability. These exist on a location or geographic specific basis for vulnerability [21,22, 28-30], as well as specifically for climate change [24, 31-33].

However, the range and extent of indicators varies from study to study. Complex analyses incorporating multiple stressors have been carried out at the local level in various locations [34,35]. The relationship between natural capital and vulnerability to climate change is arguably one of the least contested. The greater the level of reliance of a household on natural resources, such as farming, fishing, or forestry, the greater will be their vulnerability to climate change. This is because the availability of such natural resources is dependent on climatic variables such as rainfall, which are projected to change under climate change. It is likely that the level of dependence on natural resources will vary from household to household: for some households farming constitutes the main base of their livelihood; for others it is an equal or lesser contributor alongside other economic activities; and several households do not participate in farming at all. In this study area, however, almost all households directly depend on farming activities. Thus the variable measuring environmental vulnerability considers most vulnerable households (with total dependence on agriculture) as compared to the medium vulnerability (partial dependence) and low vulnerability (no dependence on agriculture).

According to Deressa, Hassan, and Ringler [5], Fussel [36] and Nhemachena, Benhin, and Glwadys [37], indicators for environmental vulnerability includes but not limited to slope of the land, soil fertility, rainfall, temperature, frequency of hazards (drought, flooding, forest fire, disease outbreaks, etc.), vegetation cover, and others. In the overall vulnerability analysis model, these are variables for the measurement of sensitivity and exposure. From **Table 1**, the environmental vulnerability of the community to climate change can be easily observed. The undulating and steeply sloping farmlands, low fertility level due to frequent degradation to soil erosion, extremely low vegetation cover, frequently occurring climate change induced shocks (at least 5 in a year), below average rain and mounting temperature have significantly contributing to the vulnerability level of smallholder farmers.

3.1.2. Measuring Vulnerablity Level by Agro-Ecology

The above method of measuring vulnerability using certain social, economic and environmental variables is usually called the indicator method. The indicator method of quantifying vulnerability is based on selecting some indicators from the whole set of potential indicators and then systematically combining the selected indicators to indicate the levels of vulnerability as indicated under the above section; farmer's vulnerability to climate change. These levels of vulnerability may be analyzed at local [11,38,39], national [40], regional [28,41], and global [37] scales. For the purpose of this study, however, a local level analysis is proposed based on the recommendations given by various researchers, who have done at macro level.

For the analysis of vulnerability condition in the study area, a household level varaibles were used to make comparisons between communities residing in different agro-ecological zones using the principal component analysis. The variables used in the analysis are listed under the social, economic and environmental vulnerabilities in **Table 1** above. The variables under I and II in **Table 1** measure adaptive capacity while the variables in under section III measure the sensitivity and exposure to climate change impacts. Based on STATA output, the findings of the study for the agro ecology based classification on vulnerability indicators revealed two components with eigenvalues greater than 1. These two components explain 99 percent of the total variation in the

Copyright © 2012 SciRes.

dataset. The first principal component explained most of the variation (57.2 percent), and the second principal component explained 42.8 percent. Based on the level of variation explained in constructing indices, the first principal component was taken, which explained majority of the variation in the dataset. **Table 2** presents the principal component analysis result for factor scores.

From **Table 2**, it is observed that the result of the principal component analysis for factor score was positively associated with majority of the indicators identified under adaptive capacity and negatively associated with all the indicators categorized under exposure and sensitivity. Therefore, in order to construct vulnerability indices, indicators of adaptive capacity, which are positively associated with the first principal component analysis, and indicators of sensitivity and exposure; which are negatively associated with the principal component analysis were taken. In total 22 indices were considered. Higher values of the vulnerability index show less vulnerability and vice versa. This is because, adaptive capacity is considered as positively contributing to the reduction of vulnerability, while exposure and sensitivity are negatively contributing in vulnerability reduction.

Based on the formula under **Eq.4**, the vulner- ability index of each agro ecological zone is calculated. In the calculation of vulnerability index for each agro ecology, normalized value of each variable (using its mean and standard deviation) as shown in **Annex 2** is used. Accordingly the vulnerability index of the three agro ecology is shown in **Figure 1**.

The different social, economic and environmental variables used to generate the 22 indices were tabulated for each agro-ecology. **Annex 2** shows how much values of each variable deviate from the mean of the total observation for each agro-ecology. Biggest positive deviance for a given variable indicates that the agro-ecology has better measurement in that specific factor. On the other hand, biggest negative deviance implies that the agro-ecology has lower level of measurement in the specific factor. For instance, the values for educational level of household head indicate –0.094 and 0.075 for highland and lowland respectively. This implies that the average education level of farmers in the lowland is 7.5% higher than the mean of educational level of the total respondents and while it was 9.4% lower for the highland.

The **Figure 1** shows the net effect of adaptation, exposure, and sensitivity computed from principal component analysis results. It is apparent from the figure that the net value is only positive for community living in the lowland areas; while it is negative for those living in midland and highland agro ecologies. The most vulnerable agro-ecology is the highland; this is due to small

Table 2. Factor score for the first principal component analysis.

S/N	Variables	Factor Score
**A**	**Social Vulnerability Variables**	
	Gender (sex of the household head)	−0.9997
	Age of the household head above 60 and less than 18	−0.8051
	Educational level	0.9119
	Marital status	0.8974
	Number of relatives	0.6145
	Institutional participation	−0.5787
	HH size	0.6673
	Farmer to farmer extension	0.8263
	Agric. extension	0.9109
	Year of agr. experience	0.6531
	Indigenous early warning system	0.5761
**B**	**Economic Vulnerability Variables**	
	Livestock ownership	0.962
	Access to information	−0.3305
	Ownership of perennial crops	0.6115
	Size of land cultivable	0.5004
	Number of farm plots	0.9801
	Non-farm income	0.9805
	level of land conservation	−0.2864
	Crop diversity	0.6352
	Irrigation usage	0.9805
	Improved seed usage	−0.3983
	Commercial fertilizer usage	0.7527
	Credit Access	−0.9206
**C**	**Environmental Vulnerability Variables**	
	Slope of farmland	−0.2386
	Fertility of farmland	−0.5564
	Vegetation cover	0.8129
	Natural hazards	−0.8459
	Rainfall	−0.8264
	Temperature	−0.9578
	Wind direction change	−0.7006

Sources: STATA output of principal component analysis from data of 2011/2012 household survey.

Copyright © 2012 SciRes.

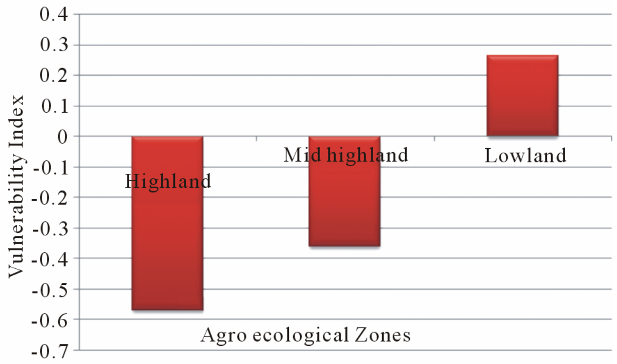

Figure 1. Household's vulnerability to climate change impacts in North Shewa. Sources: Own computaiton (lowland 45%, midland 40% and highland 15%)

land size, highly fragmented farm, low productivity of land due to fertility lose, high degradation of farmlands due to steep sloping, lower level of asset building like livestock and perennial crops, and generally lower level of experience to adapt to climate change impacts.

In the context of the study are, the midland was less vulnerable as compared to the highland areas, This less vulnerability is attributed to lower level of prevalence of pest and diseases, potential to grow diversity of crops, relatively gentle sloping of farmlands, moderate rainfall and low frequency of natural hazards.

Contrary to the expectations the lowland area was not vulnerable when compared with the midland and highland. From the above variables considered in vulnerability analysis, the lowland was not vulnerable because of better experience of operating agricultural activities under stressful conditions, relatively larger farm size with optimal number of farm plots, moderate slope of farm lands, better fertility level of farmlands, better size of land under irrigation, better adaptation to changing climatic conditions and access to early warning information.

3.2. Household Resilience and Its Indicators

3.2.1. Indicators of Resilience to Climate Change Impacts

Due to the frequency of shocks, traditional coping mechanisms adopted by vulnerable communities are eroding. During previous drought, floods, disease outbreaks, landslides and shocks episodes, households have been able to draw on kinship support network, barter animals or other assets for food, and/or migrate to areas with more plentiful natural resources. However, due to a variety of factors-including continual population growth, environmental degradations, and the increasing severity and frequency of climate change induced shocks, communities are less able to provide informal social safety nets for the neediest households. Similarly, responses formulated to cope with the periodic occurrence of shocks have been further challenged by dramatic increase in food price, and the growing prevalence of conflict on communal resources along districts and zonal boarders. As a result, many households affected by the growing frequencies of shocks are forced to adopt adverse coping mechanisms such as charcoal production, overgrazing of reserve and dispute over water and grasses.

Furthermore, combination of severe shortfalls and disruption of rainfall pattern, depletion of natural resources, ongoing conflicts and the lack of viable livelihood alternatives are increasing which in turn challenge the resilience of vulnerable households. However, some household still exhibit characteristics of resilience and are always been able to overcome extreme shocks and sustain their livelihood and lives. And, what is important under this section of the study is what characteristics determine to be resilient to changing climatic conditions.

Resilience is more than an "adaptive capacity"—that is, society's capability to draw upon its individual, collective and institutional resources & competencies to cope with, adapt to & develop from the demands, challenges and changes encountered before, during and after a disaster. Much of the literature on resilience from the perspective of hazards and disasters falls within the domain of hazard mitigation planning.

Households that take adverse coping mechanisms often fail to bounce back after the shock. For instance households that engage in the sales of liquid assets and sales of productive assets lacks the capacity to continue their livelihood operation after disaster shock is over, this is because they have already deteriorated their operational capacity. The most damaging form of household coping strategy (prior to total destitution) is the liquidation of household productive assets such as seeds, tools, large animals, and land. This category could also include taking on significant levels of formal or informal debt from financial institutions or village/neighborhood money lenders. Such households continue to suffer even after the stressful seasons are over and consequently fail to be resilient. Therefore, it is important to consider the level of such asset maintenance during climate shocks to measure the level of household's resilience.

Thus it is important to measure these indicators and link this information to how resilient a community is currently. That is which of the personal, community and institutional factors are strongly affecting resilience in that community and see resilience as function of the interdependencies between these factors. This also means that intervention to improve resilience to climate change must be directed at all factors. They cannot be treated separately. However, intervention may not be required for a given predictor if its assessment reveals that it is

Copyright © 2012 SciRes.

present at high levels. **Table 3** presents the statistical measure of the different variables of resilience in the study area.

From the statistical analysis result, the time taken to bounce back after climate change induced shocks ranges from 1 agricultural year to more than 5 years. That is if the climate change induced shocks seriously affects the crop and livestock production system during the current year, only 13.9% can get back to normal operation during the next production year, where as large majority of the households as 56.9% needs more than 3 years to bounce back if no other shock hits them again. The average number of seasons required for normal resilience is estimated to be 3 years. This is in agreement with the above analysis of vulnerability, where households residing in the highland and midland are very much vulnerable to climate change induced shocks. When disaggregated, household's residing in the highland areas take 3.7 years to bounce back, while those residing in the midland take 3 years to fully bounce back. The households in the lowland take less than 1.5 years on average to bounce back.

Even though, the topography of farm lands in the study area is characterized by steep sloping and ragged terrain, the investment made by households on conservation of their natural resources endowment is very low. A large majority of the households (58%) have worked conservation structure on less than 50% of their farmland and the annual loss of fertile soil to erosion was high; which in turn exacerbates the vulnerability of the households and reduces easy bounce back after natural hazard. In this area, the dependent family members is high, the family size of the sample households ranges from 1 to 10; while only one or two members of household work and cover livelihood needs from agricultural activities. In this case, improving the resilience of a family by being dependent only on the head of household would be very difficult.

Access to financial services in time of crises is an important factor to recover from the impact of natural hazard in the area. Even though 55.5% the households have indicated that they do have access to credit, so far only 41.4% was able to access credit for agricultural operation to recover from the impact of natural hazards. In spite of the significant importance of financial and food saving, the culture of financial saving and keeping food stock

Table 3. Statistical values of factors of resilience to climate change induced shocks.

Variables	Mean	Maximum	Minimum	St. Deviation
Time taken to bounce back (Agr. seasons)	3	5	1	1.3898
Diversity of income sources<br>(Type of crops + type of Livestock + Types non-farm)	7.193	20	1	3.1666
Investment on land conservation<br>(4 is 100%, 3 is 75%, 2 is 50%, 1 is 25% and 0 is 0%)	1.998	4	0	1.342
Saving (% annual earning)	−0.83	0.1	−2.39	0.171
Agr. Extension visit (frequency per year)	2.659	7	0	1.6896
Food reserve (% of food total harvest)	0.044	0.1	−0.86	0.0709
Preparedness (1 yes, 0 otherwise)	0.946	1	0	0.2244
Educational level of HH head (Year of Schooling)	0.9735	15	0	2.149
Dependency (number of dependents)	3.12	7	1	2.45
Farm size (Ha)	1.13	6.87	0	7.349
Credit access (1 is yes, 0 otherwise)	0.555	1	0	0.497
Distance between plots (hours)	0.59	4	0	0.617
Irrigation (Area irrigated in Ha)	0.053	2.25	0	1.121
Adaptation level (Likert scale ranging between 0 and 1)	0.347	1	0	0.2334
Last year production (1 good, 0 bad)	0.389	1	0	0.488
Asset not liquidated during disaster time (1 yes, 0 otherwise)	0.365	1	0	0.48198
Experience of natural shock (Number/year)	1.694	5	1	0.9625
Years of farming Experience	26.65	80	1	15.4

Source: Own computation from household survey of 2011/2012.

Copyright © 2012 SciRes. OPEN ACCESS

over a period is very low. Even during normal years, the balance between earning and expenditure shows negative for significant proportion of households (28.9%). That is large proportion of people usually seek loan from friends and relatives to sustain their lives until the next production season. Similarly, 10.8% needs external food support in addition to own production. These are all tide to preparedness for the coming season's possible natural shock, which plays significant role to bounce back. In general, large majority of the households (86.3%) do not have preparedness plan either at household or community level. This makes households to encounter natural shocks as a surprise. Using chi square test between the prepared and unprepared there was significant difference in terms of the time taken to bounce back after a natural shock.

The level of involvement in local institutions in the area shows that on average, a household participate in 2.35 institutions with a maximum of 6 and minimum of 0. Participation in existing local institutions and having relatives in the area were used as a measure of household's social capital. Moreover, diversification of income sources is an important strategy to minimize risk. From the statistical analysis result, the average number of enterprises taken up by farmers is 7.2. Some households have engaged in the production of only one enterprise (say production of single crop), while others have engaged in the production of even more than 20 different crops and livestock enterprises.

3.2.2. Econometric Results: Determinants of Household Resilience

3.2.2.1. Dependent Variable: Resilience; Time Taken to Bounce Back to Normal

The frequency distribution of time taken to bounce back indicates that 57.1% of the respondents were given value of 0, as it takes them greater than 2 years to bounce back (years > 2), 29% was given 1, as it takes them more than one year and less than or equals to 2 years ($1 <$ Years ≥ 2) and 13.9% was given value 2, as it takes them less than or equals to one year (year ≤ 1) to bounce back to their normal farm oparation as a time before the shocks. Test of significance using a t-test was done to make sure that there is a statistically significance difference between the categories falling above and below the cut points for the independent variables. **Table 4** presents the regression coefficient and marginal effect of each factor on the time taken to bounce back.

3.2.2.2. The Propensity to Invest on Natural Resources, Maintain Soil Fertility and Access to Irrigation

Local community have a variety of techniques at their disposal to enhance the sustainability of the natural resources, which will have significant impact on community's resilience during climate change induced disturbances. Some of the practices in the study area include construction of soil and water conservation structures, undertaking agro forestry practices, planting of trees around their farmlands, crop rotation, fallow years and use of natural fertilizer. In fact, the practice of such natural resource conservation in turn depends up on households' farm size, farm locations, alternative income and others. The hypothesis here was that households that have higher proportion of their land conserved as an investment on their natural resources management will have better level of resilience. From the t-test result in terms of resilience level between those having better investment on their land and those not having, the test shows significant difference at 1% level of significance. Similarly, from the econometric result (**Table 4**), the regression coefficient for the marginal effect shows 0.062, which is significant at 1% indicate that increment of farmland conserved by 25% (out of their total landholding), will increase the probability to move to the next category for bouncing back faster by 6.2%.

In addition to investment made to protect the natural environment, the natural fertility level of farm plots is important determinant for speed bounce back and produce from the land in the following season. Farmers have already exhausted their farmland and even expanded agriculture to steep sloping areas, marginal lands and forest areas. This is due to the continual decrease in the productivity of their farmland that has come to hardly sustain household's food need. Still households with better fertile soil have better production level and hence better capacity to bounce back after the natural shock is over. The above result indicates that the a coefficient of 0.030 for the marginal effect, which implies, households having better fertile land have a 3% likelihood to bounce back faster as compared to those with unfertile farmlands. Moreover, households having area under irrigation experience better level of resilience. The marginal effect for area under irrigation is 0.115 indicating a 1 ha increase in area under irrigation would lead to an 11.5% probability to move from lower category to higher one for bounce back faster.

3.2.2.3. Preparedness

In building resilience level of households', preparedness for the next season's possible natural shock plays vital role. Community members that are well prepared were found to have better level of resilience as compared to those unprepared. Preparedness in the econometric model was measured using dummy, where those that have preparedness were assigned a value of 1 and 0 otherwise. The coefficient of the marginal effect equals 0.196 implies that households with preparedness have

Table 4. Ordered probit model output for time taken to bounce back after natural shock.

Variables	Regression		Marginal Effect	
	Coefficient	St. Error	Coefficient	St. Error
Propensity to invest on land	0.156***	0.051	0.062***	0.020
Propensity to save	0.245	0.463	0.097	0.183
Agr. extension	0.060	0.039	0.024	0.015
Availability of Food reserve	0.087	1.062	0.034	0.419
Preparedness	0.499**	0.274	0.196**	0.104
Educational level HH head	0.073**	0.030	0.029**	0.012
Number of HH's dependant	−0.013	0.055	−0.005	0.022
Farm size	−0.009	0.010	0.004	0.004
Access to credit	0.422***	0.131	0.166***	0.051
Farm plot distance	0.070	0.114	0.028	0.045
Irrigation	0.291***	0.083	0.115***	0.033
Saving of productive asset from liquidation	0.451***	0.136	0.178***	0.053
Experience of natural shock	0.043	0.069	−0.017	0.027
Age HH head	0.003	0.005	0.001	0.002
Agro-ecology: highland	0.054	0.111	−0.021	0.044
Midland	0.047	0.012	−0.010	0.0078
Lowland	0.0103*	0.166	0.0045	0.007
Sex of HH head	0.060	0.176	0.024	0.070
Social capital: Nº insti. participated in	0.106*	0.058	0.042*	0.042
Perennial crops ownership	0.118	0.123	0.046	0.049
Access to input/output Market	−0.218**	0.094	−0.086**	0.037
Diversity income sources (livelihood diversification)	0.035*	0.023	0.014*	0.009
Adaptation level	0.035	0.101	−0.014	0.040
Nº. relatives	0.001	0.001	0.000	0.000
Nº. farm plots	−0.014	0.038	0.005	0.015
Level of soil fertility	0.076***	0.027	0.030***	0.011
Log likelihood	−345.74			
Number of observation	397			
LR chi2 (24)	79.54			
Prob > chi2	0.000			
Pseudo R2	0.4032			

***, ** and * indicates significance at 1%, 5% and 10% probability levels respectively.

Copyright © 2012 SciRes. OPEN ACCESS

19.6% probability to move to the next category for bounce back faster as compared to those who do not have preparedness for next year.

3.2.2.4. Educational Level

From range of households' characteristics, the educational level of household head was found to be significant determinants of resilience to climate change induced shocks. Heads with higher level of education have better level of planning, access and understanding of early warning information, better decision making skills during natural shocks, alter agricultural operation, adopt extension packages and more. Thus education is one of the key factors in building the resilience level of households to climate change impacts. The analytical result shows that this variable is significant at 5% level. An increase in a year of schooling by one increases the probability to move to next better category for bouncing back faster by 2.9%.

3.2.2.5. Access to Credit

One of the most challenging factors in the study area for smallholder farmers to be resilient to climate change impact is access to cash needs in times of crises. The available micro finances institutions in the area are not as such willing to advance loan during crises. Consequently, farmers resort to borrowing from local lenders at exorbitantly high interest rates. And cash constraints during period of natural shocks lead farmers to fall in short of access to early maturing varieties, drought tolerant varieties and fertilizer. In the model result, access to credit was significant determinant of resilience at 5% probability level. The marginal effect of access to credit shows that farmers who have access to credit have a 16.6% probability to move to the next category for bounce back faster as compared to those who do not have access.

3.2.2.6. Saving of Productive Assets from Liquidation

Subsequently, and often concurrently with household short-term strategies, asset divestment (sales) strategies are employed. Of these, less damaging are divestments of "liquid" assets such as small animals and household possessions. Strategies where resources from relatives or extended family are tapped (e.g., informal loans of food or money from relatives) also are included in this category. The most damaging form of household coping strategy (prior to total destitution) is the liquidation of household productive assets such as seeds, tools, large animals, and land. This category could also include taking on significant levels of formal or informal debt from financial institutions or village/neighborhood moneylenders. Significant percentage of those who sold out their liquid asset to survive natural shocks has hardly bounced back. That was because they have already lost their predictive capacity. And saving of productive asset during time of shocks was a good determinant of resilience as evidenced by the regression coefficient which was significant at all conventional probability levels. The marginal effect of 0.178 indicates that those who have not liquidated their productive asset has a 17.8% likelyhood to move to the next better category over those that have liquidate their assets to bounce back faster after the climate change induced shock.

3.2.2.7. Social Capital: Involvement in Local Institutions

Social networks build a sense of community that contributes to the resilience of individuals and groups. In the study area types of networks that are important include families, friends and community organizations. These groups provide strong bonds within a social group; a sense of belonging, identity and social support; and strong linkages to other outside groups that can bring in additional social, financial or political resources. Successful and enduring local institutions create relationships with a common purpose and promote shared interests, but can also have adaptable and flexible functions. They can provide emotional and practical support, information and resource sharing. They stay open, inclusive and diverse, and build community members capital to mitigate and respond to any natural and manmade hazards. These local institutions include, *Idir*, *Mahiber*, *Iqub*, *Senebte*, *Debo*, etc. The participation in local institutions is a strong determinant of household's resilience to climate change impact. The marginal effect of 0.042 indicates that involvement in one additional local institution fosters the likelihood to move to the next category for bounce back faster by 4.2%.

3.2.2.8. Access to Input/Output Market

Households' getting easy access to market have a chance of getting access to input, sale their product, exchange information, and diversify their livelihood by even engaging in small scale trade. The availability of market in the area benefits households by enabling them to immediately sale their perishable agricultural commodities like vegetables, fruits, and livestock products in a market to survive from lose that may come due to change in weather conditions. Moreover, access to market or being proximity to market is an important measurement in climate change to bounce back or even to adapt to the changing condition, presumably because market serves as a means of exchanging information with other farmers. In this connection, this study hypothesizes that there is positive relationship between access to output and input markets and households' resilience to climate change induced shocks. From the

Copyright © 2012 SciRes. OPEN ACCESS

econometric result of **Table 4**, the regression coefficient for the marginal effect was −0.086, which implies an increase in one hour travel away from the market will decrease the probability to move to lower category for bounce back by 8.6%.

3.2.2.9. Diversity of Income Sources (Livelihood Diversification)

The diversity of livelihood sources plays vital role in that in an event one of the livelihood means is damaged by climate change induced shocks, households would survive on the other alternatives. In various climate change impact literatures, diversifying income sources stands as the primary measure of household vulnerability and resilience. The more the household rely on multiple source of income, the less it is affected by shocks. In this analysis, livelihood diversity was measured by counting the different types of crops, livestock and non-farm a family produce during a year. As a determinant of resilience to climate change, the coefficient marginal effect for income diversity, 0.014 imply if the household increases its enterprises by 1, the probability to bounce back faster than normal will increase by 1.4%.

4. CONCLUSIONS

The vulnerability of rural farm households is largely determined by variety of factors that include social, economic, and natural factors. Households living in different agro ecological location exhibit vulnerability to different types of hazards. The effect of location in terms of agro ecology also determines households' susceptibility to the risks; where people living in the highland areas are relatively much vulnerable to risks of climate change as compared to lowlanders, in the context of the study area. This basically emanates from the topography of farmlands, frequency of natural shocks, low experience of people to adopt to climate change impacts, degradation of farmlands to erosion and more. Social factors like low level of literacy or lack of awareness on hazard related issues have been another exacerbating factor in the districts for vulnerability. On the other hand, households living in the lowland areas were vulnerable to drought, disease outbreaks and alien weeds. However, when comparison is made between three agro-ecological zones in the study area, lowland was not vulnerable because of better experience of operating agricultural activities under stressful conditions, relatively bigger size of farm land with optimal number of farm plots, better access to credit, moderate slope of farm lands, better fertility level of farmlands, better adaptation to changing climatic conditions and relatively access to early warning information.

The resilience levels of farm households living in the same area differ based on certain socio-economic and natural factors attributable to lives and livelihood of the farmers. The capacity to bounce back during and after climate change induced shocks depends on a number of households' characteristics, institutional arrangements, social networks, economic capacity and natural setting. Maintaining productive assets from deterioration during shocks, accessing to irrigation, investing on farmland, improving the fertility level of farms through usage of organic processes, having preparedness, diversifying income sources and participating in local institutions are some of the households' action that can build their resilience to climate change impacts. Organizational responses from government and development actors through creation of access to market, access to farm loans, improving educational level, and increased access to early warning information can be considered as an intervention to build the resilience of community in the study area.

REFERENCES

[1] Ignatius, A. (2012) Spatial vulnerability of rural households to CC in Nigeria: Implications for internal security. Lagos.

[2] United Nations Framework Convention on CC (2007) CC: Impacts, vulnerabilities and adaptation in developing countries. CC Secretariat, Bonn.

[3] Preston, B.L., Smith, T., Brooke, C., Gorddard, R., Measham, T. and Withycombe, G. (2008). Mapping CC vulnerability in the Sydney Coastal Councils Group. Sydney Coastal Councils Group by the CSIRO Climate Adaptation Flagship Canberra, Sydney.

[4] Intergovernmental Panel on CC (2001) CC 2001: Impacts, adaptation and vulnerability. 3*rd Assessment Report of the Intergovernmental Panel on CC*, *Adaptation to CC in the Context of Sustainable Development and Equity*, Chapter 18, Cambridge University Press, Cambridge, 877-912.

[5] Deressa, T., Hassan, R.M. and Ringler, C. (2008) Measuring Ethiopian farmers' vulnerability to Climate Change across regional states. IFPRI discussion paper No. 806, Washington. http://www.ifpri.org/pubs/dp/ifpridp00806.asp

[6] Pearson, L. and Langridge, J. (2008). CC vulnerability assessment: Review of agricultural productivity. *CSIRO Climate Adaptation Flagship Working Paper* No. 1.

[7] Odjugo, P.A. (2010) General overview of CC impacts in Nigeria. *Journal of Human Ecology*, **29**, 47-55.

[8] United Nations Framework Convention on CC (2006) Background paper on impacts, vulnerability and adaptation to CC in Africa. *African Workshop on Adaptation Implementation of Decision* 1/*CP*.10 *of the UNFCCC Convention*, Accra, 21-23 September 2006.

[9] Klein, R.J.T. (2004) Approaches, methods and tools for cc impact, vulnerability and adaptation assessment. *In-Session Workshop on Impacts of, and Vulnerability and*

Adaptation to CC, 21*st Session of the UNFCCC Subsidiary Body for Scientific and Technical Advice*, Buenos Aires, 8 December 2004

[10] DFID (2011) Defining disaster resilience. *A DFID Approach Paper.*

[11] Adger, W.N. and Kelly, P.M. (1999) Social vulnerability and the architecture of entitlements. *Mitigation and Adaptation Strategies for Global Change*, **4**, 253-266. doi:10.1023/A:1009601904210

[12] Central Statistical Authority (2007) Population census. Addis Ababa.

[13] Fussel, H. (2007). Vulnerability: A generally applicable conceptual framework for CC research. *Global Environmental Change*, **17**, 155-167. doi:10.1016/j.gloenvcha.2006.05.002

[14] Kelly, P.M. and Adger, W.N. (1999) Theory and practice in assessing vulnerability to CC and facilitation adaptation. *Climatic Change*, **47**, 925-1352.

[15] Liverman, D.M. (1990) Vulnerability to Global environmental change. In: Kasperson, R.E., *et al.*, *Understanding Global Environmental Change*, 27-44, Center for Technology, Environment, and Development, Clark University, Worcester.

[16] Hewitt, K. (1995) Excluded perspectives in the social construction of disaster. *International Journal of Mass Emergencies and Disasters*, **13**, 317-319.

[17] Pulwarty, R.S. and Riebsame, W.E. (1997) The political ecology of vulnerability to hurricane related hazards. *Hurricanes*: *Climate and Socio-Economic Impacts*.

[18] Füssel, H.-M. and Klein, R.J.T. (2006) CC vulnerability assessments: An evolution of conceptual lthinking. *Climatic Change*, **75**, 301-329. doi:10.1007/s10584-006-0329-3

[19] Cutter, S.L., Mitchell, J.T. and Scott, M.S. (2000) Revealing the vulnerability of people and places: A case study of Georgetown county, South Carolina. *Annals of the Association of American Geographers*, **90**, 713-737. doi:10.1111/0004-5608.00219

[20] Kaly, U. and Pratt, C. (2000) Environmental vulnerability index: Development and provisional indices and profiles for Fiji, Samoa, Tuvalu and Vanuatu. *Phase II Report for NZODA*. *SOPAC Technical Report* 306.

[21] Kaly, U., Briguglio, L., McLeod, H., Schmall, S., Pratt, C. and Pal, R. (1999) Environmental Vulnerability Index (EVI) to summarize national environmental vulnerability profiles. *SOPAC Technical Report* 275, 66 p.

[22] Easter, C. (1999) Small states development: A Commonwealth Vulnerability Index. *The Round Table*, **351**, 403-422. doi:10.1080/003585399107947

[23] Cutter, S.L., Boruff, B.J. and Shirley, W.L. (2003) Social vulnerability to environmental hazards. *Social Sciences Quarterly*, **84**, 243-261. doi:10.1111/1540-6237.8402002

[24] Brooks, N., Adger, W.N. and Kelly, P.M. (2005) The determinants of vulnerability and adaptive capacity at the national level and the implications for adaptation. *Global Environmental Change*, **15**, 151-163. doi:10.1016/j.gloenvcha.2004.12.006

[25] Eakin, H. and Tapia, B. (2008) Insights into the composition of household vulnerability from multi criteria decision analysis. *Global Environmental Change*, **18**, 112-127. doi:10.1016/j.gloenvcha.2007.09.001

[26] Luers, A.L., Lobell, D.B., Skar, L.S., Addams, C.L. and Matson, P.A. (2003) A method for quantifying vulnerability, applied to the agricultural system of the Yaqui Valley, Mexico. *Global Environmental Change*, **13**, 255-267. doi:10.1016/S0959-3780(03)00054-2

[27] Allen, K. (2003) Vulnerability reduction and the community-based approach: A Philippines study. In: Pelling, M., Ed., *Natural Disasters and Development in a Globalizing World*, Routledge, New York, 170-184.

[28] Vincent, K. (2004) Creating an index of social vulnerability for Africa. *Working Paper* 56, Tyndall Centre for CC Research, University of East Anglia, Norwich.

[29] Crowards, T. (1999) An Economic Vulnerability Index for developing countries, with special reference to the Caribbean: Alternative methodologies and provisional results (draft). Caribbean Development Bank, Barbados.

[30] Briguglio, L. (1995) Small island developing states and their economic vulnerabilities. *World Development*, **23**, 1615-1632. doi:10.1016/0305-750X(95)00065-K

[31] Yohe, G. and Tol, R.S.J. (2002) Indicators for social and economic coping capacity: Moving toward a working definition of adaptive capacity. *Global Environmental Change*, **12**, 25-40. doi:10.1016/S0959-3780(01)00026-7

[32] Downing, T.E., Butterfield, R., Cohen, S., Huq, S., Moss, R., Rahman, A., Sokona Y. and Stephen, L. (2001) Vulnerability indices: CC impacts and adaptation. *UNEP Policy Series*, UNEP, Nairobi.

[33] Moss, R., Brenkert, A. and Malone, E. (2001) Vulnerability to CC, A quantitative approach. http://www.ntis.gov.html

[34] Bankoff, G., Frerks, G. and Hilhorst, D. (2004) Mapping vulnerability: Disasters, development and people. Earthscan, London.

[35] Cutter, S.L. (1996) Societal responses to environmental hazards. *International Social Science Journal*, **48**, 525-536. doi:10.1111/1468-2451.00053

[36] Fussel, H. (2009) Review and quantitative analysis of indices of CC exposure, adaptive capacity, sensitivity, and impacts. Potsdam Institute for Climate Impact Research (PIK), Potsdam.

[37] Nhemachena, C., Benhin, J. and Glwadys, G. (2006) Vulnerability to CC and adaptive capacity in South African agriculture. *Food and Water Security Under Global Change*: *Developing Adaptive Capacity with a Focus on Rural Africa*, IFPRI Workshop, Washington.

[38] Leon-Vasquez, M., West, T. and Finan, J. (2003) A comparative assessment of climate vulnerability: Agriculture and ranching on both sides of the US-Mexico border. *Global Environmental Change*, **13**, 159-173. doi:10.1016/S0959-3780(03)00034-7

[39] Morrow, B.H. (1999) Identifying and mapping community vulnerability. *Disasters*, **23**, 1-8. doi:10.1111/1467-7717.00102

Copyright © 2012 SciRes. OPEN ACCESS

[40] O'Brien, K., Eriksen, S., Schjolden, A. and Nygaard, L.P. (2004) What's in a word? Conflicting interpretations of vulnerability in CC research. www.cicero.uio.no

[41] Leichenko, R.M. and O'Brien, K.L. (2001) The dynamics of rural vulnerability to global change: The case of Southern Africa. *Mitigation and Adaptation Strategies for Global Change*, **7**, 1-18. doi:10.1023/A:1015860421954

[42] Nyong, A.O., Adepetu, A.A., Ihemegbulem, V.C. and Dabi, D.D. (2003) Vulnerability and Adaptive capacity of Rural Populations in the Sahel to CC and climate variability: A case study of semi-arid Northern Nigeria. *EMF Snowmass Workshop "CC Impacts and Integrated Assessment*", 28 July-6 August 2003, Snowmass.

[43] Paavola, J. (2004) Livelihoods, vulnerability and adaptation to CC in the Morogoro region, Tanzania. *Working Paper EDM* 04-12, Centre for Social and Economic Research on the Global Environment, University of East Anglia, Norwich.

[44] Næss, L.O., Norland, I.T., Lafferty, W.M. and Aall, C. (2006) Data and processes linking vulnerability assessment to adaptation decision-making on climate change in Norway. *Global Environmental*. doi:10.1016/j.gloenvcha.2006.01.007

[45] Kuhl, J.J. (2004) Disaggregating household vulnerability: Analysing fluctuations in consumption using a simulation approach. *Growth*, *Poverty Reduction*, *and Human Development in Africa*, Centre for the Study of African Economies and Global Poverty Research Group, Oxford.

[46] Eakin, H. (2002) Rural responses to climatic variability and institutional change in central Mexico. *Working Paper* 10/2002, Center for US-Mexican Studies, University of California, San Diego.

[47] Ford, D.J, Barry, S. and Wandel, J. (2006) Vulnerability to CC in the Arctic: A case study from Arctic Bay, Canada. *Global Environmental Change*, **16**, 145-160. doi:10.1016/j.gloenvcha.2005.11.007

[48] Haan, N., Farmer, G. and Wheeler, R. (2001) Chronic vulnerability to food insecurity in Kenya—2001. *A World Food Program Pilot Study for Improving Vulnerability Analysis*. http://www.wfp.org/operations/vam/country_experience/kenya_vam/index.asp.

[49] Aandahi, G. and O'Brien, K. (2001) Vulnerability to CCs and economic changes in India agriculture. *Waters of Hope*: *The Role of Water in South Asian Development*, *Biannual Conference of the Nordic Association for South Asian Studies*, Voss.

[50] Bantilan, C.S. and Anupama, K.V. (2002) Vulnerability and adaptation in dry land agriculture in India's SAT: Experiences from ICRISAT village level studies. *Adaptation to CC for Agricultural Productivity*, South Asia Expert Workshop, New Delhi, 1-3 May.

[51] Canadian International Development Agency (2003) Methodology used in the CIDA study: An example of India. *AIACC Asia-Pacific Region Open Meeting*. http://www.aiaccproject.org/meetings/Bangkok_03/Workshop%20presetations/C1_S_Bhadwal.ppt

Copyright © 2012 SciRes. OPEN ACCESS

APPENDIX

Annex 1. Indicators and proxy variables for vulnerability analysis.

Categories of Indicator	Indicator	Level of Analysis	Authors
1) HH characteristics	Household size	HH	[11,42]
	Female headed households	HH, District	[40,42,43]
	Labour unit	HH	
	Age of HH head	HH	[44]
	Educational level of HH head	HH, District and National	[24,42,45]
	Asset ownership (land, houses, farm equipments and other assets)	HH, District	[24]
	Non-farm engagement	HH, District	[11,42,46-48]
	Food stock	HH, District and National	[42]
	Drinking water	HH	[43,49]
Additional variables included for study area			
	Marital status	HH	
	Access to EWS	HH	
	Experience of Agr. Activity	HH	
2) Economic characteristics	Farm income level	HH	[48]
	% of HH below poverty line	District	[43]
	Expenditure on food	HH	[40,48]
	Infrastructure	HH, District and National	[40,48]
Additional variables included for the study area			
	Ownership of radio	HH	
	Ownership of perennial crops	HH	
	Number of farm plots	HH	
	Food reserve	HH	
	Cash reserve	HH	
3) Institutional characteristics	Social network	HH	[42,47]
	Institutional arrangements	District and National	[40,47]
Additional Variables for the study area			
	Access to credit	HH	
	Access to Agr. extension	HH	
4) Farm charters tics	livestock ownership	HH	[43]
	Cropping system	HH	[40,50]
	Fertilizer applications	HH	[49,50]
	Irrigation usage (rate or sources)	HH, District	[40,49]
Variables added for the study area			
	Area under improved technology	HH	
5) Environmental (biophysical) characteristics	Soil conditions	HH, District and National	[40]
	Climatic conditions	HH, District and National	[40]
	Vegetation	District and National	[48]
	CC induced shocks(drought and flood)	District and National	[40,51]
Variables added for the study area			
	Soil and water conservation	HH	
	Topography of the farmlands	HH	

Source: Adopted with modification from Nhemachena, Benhin, and Glwadys [37] also coated by Deressa, Hassan, and Ringler [5].

Copyright © 2012 SciRes. OPEN ACCESS

Annex 2. Vulnerability index for each agro ecology, normalized value of each variable (using its mean and standard deviation).

Geographic locations	Gender	Age	Educational level	Marital status	No relatives	Institutional Participation	HH size	Farmer to farmer Extension	Agric. Extension	Year of Agr. Experience	Indigenous EWS	TLU	Radio Ownership	Ownership of perennial Crops	Size of Land cultivable	No Plots	Non-farm income
Highland	0.052	0.02	−0.094	−0.027	−0.124	0.061	−0.01	−0.039	−0.038	−0.037	−0.103	−0.032	0.017	-0.011	-0.015	-0.006	-0.072
Mid highland	−0.032	−0.02	0.020	0.028	−0.051	−0.124	−0.003	0.047	0.038	0.064	−0.052	0.011	-0.069	0.021	0.035	0.002	0.055
Lowland	−0.021	0.01	0.075	−0.001	0.175	0.062	0.015	−0.009	0.000	−0.027	0.154	0.021	0.052	-0.010	-0.021	0.003	0.017

level of Land conservation	Crop Diversity	Irrigation usage	Improved seed usage	Commercial fertilizer usage	Credit Access	Slope of farmland	Fertility of farmland	Vegetation cover	Natural Hazards	Rainfall	Temperature	Wind Direction Change
0.019	−0.093	−0.060	0.012	−0.174	0.068	−0.020	0.125	−0.017	0.062	−0.050	0.122	0.039
−0.096	−0.031	0.025	−0.038	−0.021	−0.016	0.128	0.069	0.000	−0.072	0.001	−0.105	0.009
0.076	0.124	0.035	0.026	0.195	−0.053	−0.109	−0.195	0.017	0.011	0.048	−0.018	−0.047

Copyright © 2012 SciRes.
