# Supplementary material for: Systematic Review of Methods in Low-Consensus Fields: Supporting Commensuration through `Construct-Centered Methods Aggregation’ in the Case of Climate Change Vulnerability Research
Source: PLoS One. 2016 Feb 22;11(2):e0149071. doi: 10.1371/journal.pone.0149071 (PMC4762661; doi:10.1371/journal.pone.0149071)
Supplement: S3 File — Research questions posed in systematic review. (PDF) [file pone.0149071.s003.pdf]

Research questions posed in systematic reviews of theory and methods in the domain of climate change studies

Describing use of results

- What are the expected uses of findings arising from a given method? [47]
- How is the use of findings arising from a given method addressed by the literature? [47]
- What research applications are addressed using data arising from study of a given construct? [37]

Describing methods used

- What methods are used for study? [37]
- What are the indicators used to study a given topic? [18]
- What tools are used to research a given complex construct? [2]
- What general approaches are used in research on a given topic? [46]
- What specific methods are used in research on a given topic? [46]

Assessing methods used

- Are the tools used to research a given complex construct socially adequate? [2]
- Are the tools used to research a given complex construct ecologically adequate? [2]
- How do indicators used to study a given topic fit into existing frameworks for the study of that topic? [18]
- To what extent are the tools used to research a given complex construct consistent with the principles of a specified theory? [2]

Describing links between theory and practice

- How could the set of principles found in the literature relevant to a desirable practice be improved? [42]
- How useful are definitions of a given construct to a given application? [17]
- What insights does a given literature have for problems related to a specific complex construct? [43]

Describing theory

- What are the key features of the literature on principles for a specific desirable practice? [42]
- How is a given construct defined in the literature? [17]
- What is the most suitable term for denoting a specific complex construct? [45]

- How does a given literature consider a specific complex construct? [49]

Describing coverage

- What is the coverage of research on a given topic? [18]
- What are the gaps in research on a given topic? [18]
- Which principles relevant to a desirable practice are not sufficiently covered in the literature? [48]

References

1. Espeland WN, Stevens ML. Commensuration as a social process. Annu Rev Sociol. 1998;24: 313–343. doi:10.1146/annurev.soc.24.1.313.
2. Plummer R, de Loe R, Armitage D. A Systematic Review of Water Vulnerability Assessment Tools. Water Resour Manag. 2012;26: 4327–4346. doi:10.1007/s11269-012-0147-5.
3. Delaney A, Chesterman S, Crane TA, Tamás PA, Ericksen P. A systematic review of local vulnerability to climate change: In search of transparency, coherence and compatability. CCAFS Work Pap. 2014;97. Available: http://hdl.handle.net/10568/56692.
4. Dixon-Woods M, Cavers D, Agarwal S, Annandale E, Arthur A, Harvey J, et al. Conducting a critical interpretive synthesis of the literature on access to healthcare by vulnerable groups. BMC Med Res Methodol. 2006;6: 35. doi:10.1186/1471-2288-6-35.
5. Lakerveld RP, Lele S, Crane TA, Fortuin KPJ, Springate-Baginski O. The social distribution of provisioning forest ecosystem services: Evidence and insights from Odisha, India. Ecosyst Serv. 2015;14: 56–66. doi:doi:10.1016/j.ecoser.2015.04.001.
6. Pronk M, Maat H, Crane TA. Vulnerability Assessments as a Political Creation: Tsunami Management in Portugal. Disasters. Submitted.
7. Weiler V, Udo HMJ, Viets T, Crane TA, De Boer IJM. Handling multi-functionality of livestock in a life cycle assessment: the case of smallholder dairying in Kenya. Curr Opin Environ Sustain. 2014;8: 29–38. doi:10.1016/j.cosust.2014.07.009.
8. Beauchamp A, Backholer K, Magliano D, Peeters A. The effect of obesity prevention interventions according to socioeconomic position: a systematic review. Obes Rev. 2014;15: 541–554. doi:10.1111/obr.12161.
9. Edwards PN. “A vast machine”: Standards as social technology. Science. 2004;304: 827–828. doi:10.1126/science.1099290.
10. Lepori B, Bonaccorsi A. The socio-political construction of a European census of higher education institutions: design, methodology and comparability issues. Minerva. 2013;51: 271–293. doi:10.1007/s11024-013-9235-9.
11. Lovell H. Climate change, markets and standards: the case of financial accounting. Econ Soc. 2014;43: 260–284. doi:10.1080/03085147.2013.812830.
12. Ascui F, Lovell H. As frames collide: making sense of carbon accounting. Account Audit Account J. 2011;24: 978–999. doi:10.1108/09513571111184724.

13. Timmermans S, Epstein S. A World of Standards but not a Standard World: Toward a Sociology of Standards and Standardization*. Annu Rev Sociol. 2010;36: 69–89. doi:10.1146/annurev.soc.012809.102629.

14. Merry SE, Coutin SB. Technologies of truth in the anthropology of conflict: AES/APLA Presidential Address, 2013. Am Ethnol. 2014;41: 1–16. doi:10.1111/amet.12055.

15. Alastalo M, Pösö T. Number of Children Placed Outside the Home as an Indicator - Social and Moral Implications of Commensuration. Soc Policy Adm. 2014;48: 721–738. doi:10.1111/spol.12073.

16. Brunsson N, Rasche A, Seidl D. The Dynamics of Standardization: Three Perspectives on Standards in Organization Studies. Organ Stud. 2012;33: 613–632. doi:10.1177/0170840612450120.

17. Castleden M, McKee M, Murray V, Leonardi G. Resilience thinking in health protection. J Public Health. 2011; 369–377. doi:10.1093/pubmed/fdr027.

18. Liquete C, Piroddi C, Drakou EG, Gurney L, Katsanevakis S, Charef A, et al. Current Status and Future Prospects for the Assessment of Marine and Coastal Ecosystem Services: A Systematic Review. PLoS ONE. 2013;8: e67737. doi:10.1371/journal.pone.0067737.

19. Magarey JM. Elements of a systematic review. Int J Nurs Pract. 2001;7: 376–382. doi:10.1046/j.1440-172X.2001.00295.x.

20. Thomas, James, and Angela Harden. Methods for the Thematic Synthesis of Qualitative Research in Systematic Reviews. BMC Medical Research Methodology. 2008;8 (1): 45. doi:10.1186/1471-2288-8-45.

21. Dixon-Woods, Mary, Sheila Bonas, Andrew Booth, David R. Jones, Tina Miller, Alex J. Sutton, Rachel L. Shaw, Jonathan A. Smith, and Bridget Young. How Can Systematic Reviews Incorporate Qualitative Research? A Critical Perspective. Qualitative Research. 2006;6 (1): 27–44. doi:10.1177/1468794106058867.

22. Bing-Jonsson, Pia Cecilie, Ida Torunn Bjørk, Dag Hofoss, Marit Kirkevold, and Christina Foss. Instruments Measuring Nursing Staff Competence in Community Health Care: A Systematic Literature Review. Home Health Care Management and Practice. 2013;25 (6): 282–94. doi:10.1177/1084822313494784.

23. Dubois, Carl-Ardy, Danielle D'Amour, Marie-Pascale Pomey, Francine Girard, and Isabelle Brault. Conceptualizing Performance of Nursing Care as a Prerequisite for Better Measurement: A Systematic and Interpretive Review. BMC Nursing. 2013;12: 7. doi:10.1186/1472-6955-12-7.

24. Le Reste, Jean Yves, Patrice Nabbe, Benedicte Manceau, Charilaos Lygidakis, Christa Doerr, Heidrun Lingner, Slawomir Czachowski, et al. The European General Practice Research Network Presents a Comprehensive Definition of Multimorbidity in Family Medicine and Long Term Care, Following a Systematic Review of Relevant Literature. Journal of American Medical Directors Association. 2013;14: 319–25. doi:10.1016/j.jamda.2013.01.001.

25. van der Lee, Johanna, Lidwine Mokkink, Martha Grootenhuis, Hugo Heymans, and Martin Offringa. Definitions and Measurement of Chronic Health Conditions in Childhood: A Systematic Review. JAMA?: The Journal of the American Medical Association. 2007;297 (24): 2741–51.

26. Wells, Kathleen, and Julia H. Littell. Study Quality Assessment in Systematic Reviews of Research on Intervention Effects. Research on Social Work Practice. 2009;19 (1): 52–62. doi:10.1177/1049731508317278.

27. Carroll C, Booth A, Leaviss J, Rick J. Best fit? framework synthesis: refining the method. BMC Med Res Methodol. 2013;13: 37. doi:10.1186/1471-2288-13-37.

28. Morse JM. Constructing Qualitatively Derived Theory: Concept Construction and Concept Typologies. Qual Health Res. 2004;14: 1387–1395. doi:10.1177/1049732304269676

29. Borgatti SP. Cultural Domain Analysis. J Quant Anthropol. 1994;4: 261–278.

30. Glaser BG. The constant comparative method of qualitative analysis. Soc Probl. 1965;12: 436–445.

31. Noblit GW, Hare RD. Meta-ethnography: Synthesizing qualitative studies. Newbury Park, California: Sage; 1988.

32. Kampen JK, Tamás PA. Should I take this seriously? A simple checklist for calling bullshit on policy supporting research. Qual Quant. 2014;48: 1213–1223. doi:DOI 10.1007/s11135-013-9830-8.

33. da Silva S, Tamás PA, Kampen JK. Is research on Latin American social movements interpretable? Using systematic review to test for transparency and structure prior to quality assessment. Lat Am Res Rev. Forthcoming;

34. Jensen, Mads Dagnis, and Peter Marcus Kristensen. ?The Elephant in the Room: Mapping the Latent Communication Pattern in European Union Studies. Journal of European Public Policy. 2013;20 (1): 1–20. doi:10.1080/13501763.2012.699656.

35. Baca M, L derach P, Haggar J, Schroth G, Ovalle O. An Integrated Framework for Assessing Vulnerability to Climate Change and Developing Adaptation Strategies for Coffee Growing Families in Mesoamerica. PLoS ONE. 2014;9: e88463. doi:10.1371/journal.pone.0088463.

36. Hahn MB, Riederer A, Foster S. The Livelihood Vulnerability Index: A pragmatic approach to assessing risks from climate variability and change? A case study in Mozambique. Glob Environ Change. 2009;19: 74–88. doi:10.1016/j.gloenvcha.2008.11.002.

37. Driscoll DA, Banks SC, Barton PS, Ikin K, Lentini P, Lindenmayer DB, et al. The Trajectory of Dispersal Research in Conservation Biology. Systematic Review. PLoS ONE. 2014;9: e95053. doi:10.1371/journal.pone.0095053.

38. Ford J, Smit B. A Framework for Assessing the Vulnerability of Communities in the Canadian Arctic to Risks Associated with Climate Change. Arctic. 2004;57: 389– 400. doi:10.14430/arctic516.

39. Dubois C-A, Amour D D', Pomey M-P, Girard F, Brault I. Conceptualizing performance of nursing care as a prerequisite for better measurement: a systematic and interpretive review. BMC Nurs. 2013;12: 7. doi:10.1186/1472-6955-12-7.

40. Delaney A, Tamás PA, Tobi H. Which standards from which disciplines? A test of Systematic Review for designing quality interdisciplinary evaluations. J Dev Eff. Forthcoming;

41. Campbell R, Pound P, Pope C, Britten N, Pill R, Morgan M, et al. Evaluating meta-ethnography: a synthesis of qualitative research on lay experiences of diabetes and diabetes care. Soc Sci Med. 2003;56: 671–684. doi:10.1016/S0277-9536(02)00064-3.

42. Carroll C, Booth A, Lloyd-Jones M. Should We Exclude Inadequately Reported Studies From Qualitative Systematic Reviews? An Evaluation of Sensitivity Analyses in Two Case Study Reviews. Qual Health Res. 2012;22: 1425–1434. doi:10.1177/1049732312452937.

43. Edwards AG, Russell IT, Stott NC. Signal versus noise in the evidence base for medicine: an alternative to hierarchies of evidence? Fam Pract. 1998;15: 319–322.

44. Lemmer B, Grellier R, Steven J. Systematic Review of Nonrandom and Qualitative Research Literature: Exploring and Uncovering an Evidence Base for Health Visiting and Decision Making. Qual Health Res. 1999;9: 315–328. doi:10.1177/104973299129121884.

45. Hallfors MH, Vaara EM, Hyvarinen M, Oksanen M, Schulman LE, Siipi H, et al. Coming to Terms with the Concept of Moving Species Threatened by Climate Change - A Systematic Review of the Terminology and Definitions. PLoS ONE. 2014;9: e102979. doi:10.1371/journal.pone.0102979.

46. Schultz CA. The U.S. Forest Service's analysis of cumulative effects to wildlife: A study of legal standards, current practice, and ongoing challenges on a National Forest. Environ Impact Assess Rev. 2012;32: 74–81. doi:10.1016/J.EIAR.2011.03.003.

47. Laurans Y, Rankovic A, Bille R, Pirard R, Mermet L. Use of ecosystem services economic valuation for decision making: Questioning a literature blindspot. J Environ Manage. 2013;119: 208–219. doi:10.1016/j.jenvman.2013.01.008.

48. Luederitz C, Lang DJ, Wehrden H Von. A systematic review of guiding principles for sustainable urban neighborhood development. Landsc Urban Plan. 2013;118: 40–52. doi:10.1016/j.landurbplan.2013.06.002.

49. Plummer R, Armitage DR, de Loe RC. Adaptive Comanagement and Its Relationship to Environmental Governance. Ecol Soc. 2013;18: 21. doi:10.5751/ES-05383-180121.
