# Supplementary material for: Systematic Review of Methods in Low-Consensus Fields: Supporting Commensuration through `Construct-Centered Methods Aggregation’ in the Case of Climate Change Vulnerability Research
Source: PLoS One. 2016 Feb 22;11(2):e0149071. doi: 10.1371/journal.pone.0149071 (PMC4762661; doi:10.1371/journal.pone.0149071)
Supplement: S1 Table — (PDF) [file pone.0149071.s004.pdf]

S4 File. Example of data extraction form, as used for Hahn

Construct	Defined?	Definition or further info	Directly Op?	Indirectly op via:	Operational text
2 week illness	Yes	Percentage of households that report at least 1 family member who had to miss school of work due to illness in the last 2 weeks. (Hahn, Riederer, and Foster 2009, 77)	Yes	[name of construct]	Has anyone in your family been so sick in the past 2 weeks that they had to miss work or schoo (Hahn, Riederer, and Foster 2009, 77)
Adaptive capacity	Yes	adaptive capacity is the system's ability to withstand or recover from the exposure (Ebi et al., 2006). (Hahn, Riederer, and Foster 2009, 75)	No	Socio-demographic profile; livelihood strategies; social network	
agriculture dependend households	Yes	Percentage of households that report only agriculture as a source of income. (Hahn, Riederer, and Foster 2009, 77)	Yes		Do you or someone else in your household raise animals? Do you or someone else in your household grow crops? Do you or someone else in your household collect something from the bush, the forest, or lakes and rivers to sell? (Hahn, Riederer, and Foster 2009, 77)
average precipitation	Yes	Standard deviation of the average monthly precipitation between 1998 and 2003 was averaged for each province (Hahn, Riederer, and Foster 2009, 79)	Yes		1998-2003: provincial data; weather station based in the provincial capital (Hahn, Riederer, and Foster 2009, 79)
borrow-lend ratio	Yes	Ratio of a household borrowing money in the past month to a household lending money in the past month, e.g., If a household borrowed money but did not lend money, the ratio = 2:1 or 2 and if they lent money but did not borrow any, the ratio = 1:2 or 0.5. (Hahn, Riederer, and Foster 2009) (Hahn, Riederer, and Foster 2009, 78)	Yes		Did you borrow any money from relatives or friends in the past month? Did you lend any money to relatives or friends in the past month? (Hahn, Riederer, and Foster 2009) (Hahn, Riederer, and Foster 2009, 78)
