# Supplementary material for: Systematic Review of Methods in Low-Consensus Fields: Supporting Commensuration through `Construct-Centered Methods Aggregation’ in the Case of Climate Change Vulnerability Research
Source: PLoS One. 2016 Feb 22;11(2):e0149071. doi: 10.1371/journal.pone.0149071 (PMC4762661; doi:10.1371/journal.pone.0149071)
Supplement: S2 Table — Framework for coding of author reported frameworks. (PDF) [file pone.0149071.s005.pdf]

Coding framework for coding of author-reported frameworks

Code	Refers to	Possible indicators
IPCC	The framework used by the Intergovernmental Panel on Climate Change (IPCC), which views 'vulnerability' as composed of three elements: 'exposure to climate change-induced stress'; 'sensitivity to climate change-induced stress'; and 'adaptive capacity'	The theoretical framework contains the three elements of 'exposure', 'sensitivity', and 'adaptive capacity'.<br>The authors report that they build their framework with reference to any publications from the IPCC
VEP	This framework, called 'Vulnerability as Expected Poverty" conceives of 'vulnerability' as the probability that research units will be below a given poverty threshold given certain risk factors associated with climate change.	The theoretical framework contains the construct 'poverty', 'probability', and 'risk'.<br>The authors report that they use a framework based on the "Vulnerability as Expected Poverty" approach. The authors report that they build their framework with reference to any of the following publications:<br>http://info.worldbank.org/etools/docs/library/97185/ Keny_0304/Ke_0304/vulnerabilityassessment.pdf.<br>Chaudhuri S., Jalan, J. and Suryahadi, A. (2002) Assessing household vulnerability to poverty from cross-sectional data: a methodology and estimates from Indonesia. Discussion Paper 0102-02, Department of Economics, Columbia University<br>Christiaensen, L., and Subbarao, K. (2005) Towards an understanding of vulnerability in rural Kenya. Journal of African Economies, 14(4), 520-558.
Food Insecurity	This code refers to frameworks where vulnerability is conceived in terms of food security/insecurity. There are usually four subconstructs under food security: 'availability of food'; 'access to food'; 'stability of access'; 'utilization of accessible food'.	The theoretical framework contains a conception of 'food security' or 'food insecurity', which may be subdivided into four subconstructs similar to: 'availability'; 'access'; 'stability'; 'utilization'.<br>The authors report that they use a framework based on a "Food security" or "food insecurity" approach.<br>The authors report that they use a framework which is built on any of the following references: Løvendal C.R and M. Knowles, 2005. "Tomorrow's hunger: a framework for analyzing vulnerability to food insecurity". FAO- ESA Working Paper No. 05-07. FAO, Agricultural and Development Economics Division, Rome.<br>FAO (2000) Guidelines for national FIVIMS. Background and principles. www.fao.org/docrep/003/X8346E/X8346E00.HTM
Livelihoods Approach	This code refers to a series of similar frameworks which contain conceptions of 'livelihood capabilities', 'livelihood strategies', and 'livelihood assets'. The later is usually composed of natural, social, financial, physical, and human capital. The theoretical framework contains a combination of some of the following constructs: 'livelihood capabilities', 'livelihood strategies', 'livelihood assets', 'natural capital', 'social capital', 'financial capital', 'physical capital', or 'human capital'.	The authors report that they use a framework based on a "Livelihoods" or "Sustainable livelihoods" approach.<br>The authors report that they use a framework which is built on any of the following references:<br>Fraser, E.D.G, A. Dougill, K. Hubacek, C. Quinn, J. Sendzimir, and M. Termansen. 2010. Assessing vulnerability to climate change in dryland livelihood systems: conceptual challenges and interdisciplinary solutions. Ecology and Society.<br>Chambers, R., and G. Conway. 1992. Sustainable rural livelihoods: practical concepts for the 21st century. IDS Discussion Paper 296. Institute of Development Studies, Brighton, UK.<br>Scoones, I. 1998. Sustainable rural livelihoods: a framework for analysis. IDS Working Paper 72. Institute of Development Studies, Brighton, UK<br>DFID. (1999). Sustainable Livelihoods Guidance Sheets. London, UK: Department for International Development.
Other framework	This denotes that a framework is used which doesn't not fall into any of the other frameworks specified.	
